# Time-series transcriptome analysis identified differentially expressed genes in broiler chicken infected with mixed *Eimeria* species

**DOI:** 10.3389/fgene.2022.886781

**Published:** 2022-08-08

**Authors:** Minjun Kim, Yoonji Chung, Prabuddha Manjula, Dongwon Seo, Sunghyun Cho, Eunjin Cho, Thisarani Kalhari Ediriweera, Myunghwan Yu, Sunju Nam, Jun Heon Lee

**Affiliations:** ^1^ Division of Animal and Dairy Science, Chungnam National University, Daejeon, Korea; ^2^ Department of Animal Science, Uva Wellassa University, Badulla, Sri Lanka; ^3^ Research Institute TNT Research Company, Jeonju, Korea; ^4^ Department of Bio AI Convergence, Chungnam National University, Daejeon, Korea

**Keywords:** chicken, host-pathogen interaction, innate immunity, anti-inflammation, transcriptome analysis, gene co-expression network, Eimeria

## Abstract

Coccidiosis caused by the *Eimeria* species is a highly problematic disease in the chicken industry. Here, we used RNA sequencing to observe the time-dependent host responses of *Eimeria*-infected chickens to examine the genes and biological functions associated with immunity to the parasite. Transcriptome analysis was performed at three time points: 4, 7, and 21 days post-infection (dpi). Based on the changes in gene expression patterns, we defined three groups of genes that showed differential expression. This enabled us to capture evidence of endoplasmic reticulum stress at the initial stage of *Eimeria* infection. Furthermore, we found that innate immune responses against the parasite were activated at the first exposure; they then showed gradual normalization. Although the cytokine-cytokine receptor interaction pathway was significantly operative at 4 dpi, its downregulation led to an anti-inflammatory effect. Additionally, the construction of gene co-expression networks enabled identification of immunoregulation hub genes and critical pattern recognition receptors after *Eimeria* infection. Our results provide a detailed understanding of the host-pathogen interaction between chicken and *Eimeria*. The clusters of genes defined in this study can be utilized to improve chickens for coccidiosis control.

## Introduction

Chicken coccidiosis is a disease caused by protozoan parasites of the genus *Eimeria* (Blake and Tomley, 2014). These parasites invade the small or large intestine of chickens and cause tissue damage, leading to malnutrition, diarrhea, bloody stools, and (in severe cases) death (Lillehoj et al., 2007). Chicken coccidiosis is thus an important livestock disease that causes great economic loss to the poultry industry; anticoccidial drugs and vaccines are continuously used for prevention (Chapman et al., 2010). The establishment of a coccidiosis-resistant chicken variety would be an effective strategy for overcoming coccidiosis because it is consistent with the recent trend of reducing antibiotics; it would also reduce the cost of disease prevention (Jeffers, 1981). To achieve this goal, there is a need to identify physiological functions in chickens that are important under coccidial conditions. *Eimeria* infection promotes cell-mediated immunity in chickens; several immune cells (e.g., T lymphocytes and macrophages) have major roles in such immunity (Lillehoj and Trout, 1996). Previous studies have used a quantitative reverse transcription polymerase chain reaction (qRT-PCR) to identify differentially expressed genes (DEGs) during *Eimeria* infection by observing the immune response or using RNA sequencing (RNA-Seq) technology (Laurent et al., 2001; Wang et al., 2019).

The host response to coccidiosis parasites appears to differ over time (Bremner et al., 2021; Choi et al., 2021; Yu et al., 2021). For a more detailed understanding of the host response, there is a need to understand the biological functions performed in the body at different stages of infection. In this study, chronological DEGs in the cecum of chickens infected with multiple *Eimeria* species were identified via RNA-Seq. Clustering analysis was performed and gene co-expression networks were constructed to observe the main functions using enrichment analyses.

## Materials and methods

### Sample preparation and RNA-Seq

**FIGURE 1:**
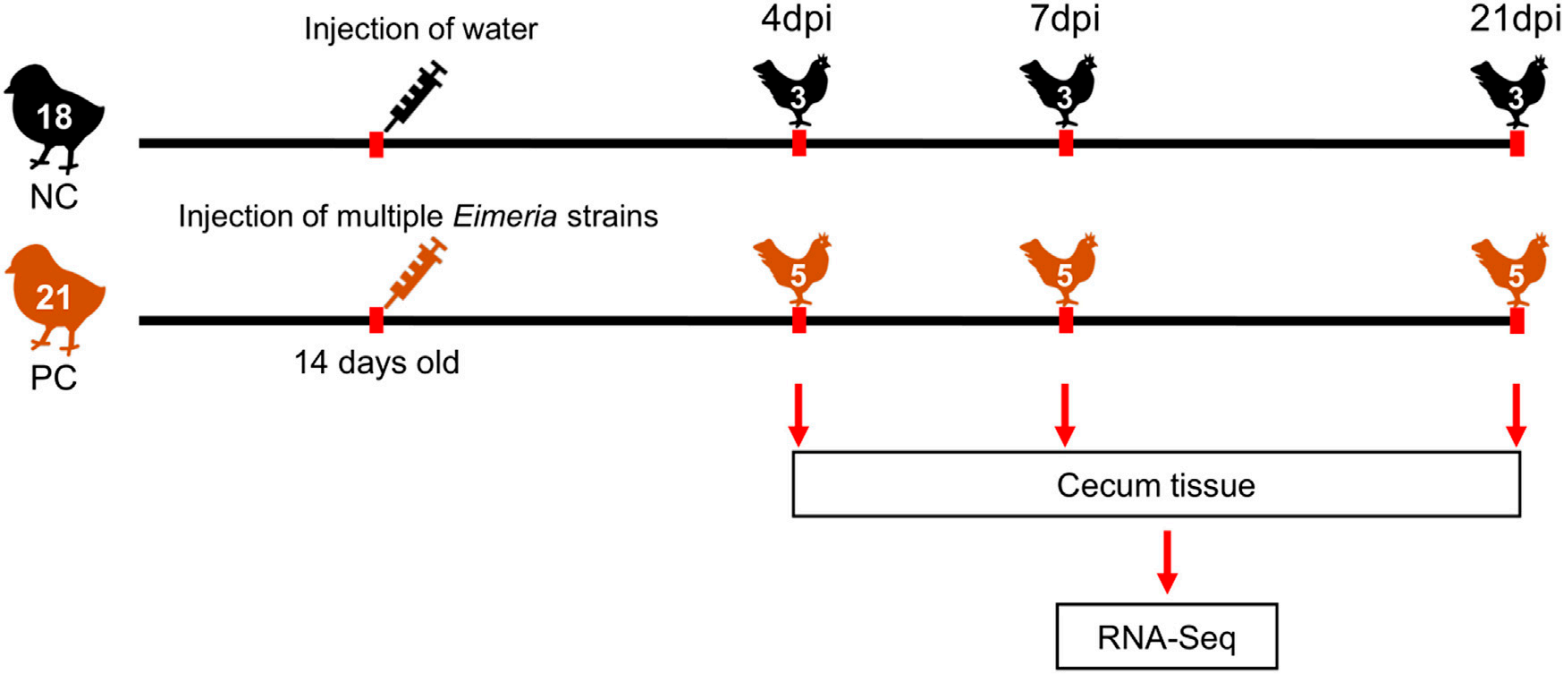
Overview of the experimental design. NC, Negative control; PC, Positive control; dpi, days post-infection.

Thirty-nine 1-day-old (Indian River; Aviagen™) male broiler chickens were randomly allocated to 13 oocyst-free cages. After 14 days, 21 chickens (PC) were orally administrated with 1 ml of Livacox® T™ (Biopharm Co., Prague, Czech Republic) as positive control chickens (PC). A 10-fold dosage containing 3–5 × 10^3^ active oocysts of each of *Eimeria acervulina*, *Eimeria tenella*, and *Eimeria maxima* were used for infection. Eighteen non-challenged negative control (NC) birds were equally inoculated with distilled water to simulate inoculation stress. All PC and NC chickens were humanely euthanized at 4, 7, and 21 days post-infection (dpi), respectively. Cecum tissue samples of all chickens were collected in tubes with RNA-later and stored at −80°C for RNA extraction. Finally, 3 samples of NC and 5 samples of PC were randomly selected for sequencing at each time point. The overall experimental design is shown in Figure 1.

Total RNA was isolated from tissue samples using TRIzol reagent (Invitrogen, Carlsbad, CA, United States) and processed with an RNeasy MinElute Cleanup Kit (Qiagen, Hilden, Germany). Total RNA integrity was determined using a 2,100 Bioanalyzer (Agilent Technologies, Palo Alto, CA, United States). All samples with RNA integrity numbers ≥ 7 were used for cDNA library construction, which was performed using a TruSeq stranded mRNA sample preparation kit (Illumina, San Diego, CA, United States) in accordance with the manufacturer’s guidelines. After library construction, all samples were sequenced using the Illumina NovaSeq 6,000 platform and 101 bp paired-end reads were generated.

### Sequencing data processing and quantification of differentially expressed genes

Raw sequence read quality control was performed using the FastQC software v0.11.9. Sequencing adaptors and low-quality reads were trimmed using Cutadapt software v1.15 and Trimmomatic software v0.39. Before analysis, pre-processed reads were re-checked with FastQC, then mapped to the chicken reference genome (GRCg6a, GCA_000002315.5) using STAR software v2.7.7a. The reference genome was downloaded from the Ensemble genome browser FTP site (ftp.ensembl.org/pub/release-104/fasta/gallus_gallus/dna/); the index file of the reference genome was built using bowtie2 software v2.4.2 and samtools software v1.11. STAR software was used to count reads matching the genes, based on the exons in *Gallus gallus* GRCg6a v102 GTF (Ensembl) as a genomic annotation reference file. The EdgeR package v3.30.3 in Bioconductor software was used to quantify the reads mapped to each gene. Genes with a total read count ≤ 8 for all genes were excluded to avoid statistical bias in the identification of DEGs. Read count normalization was conducted with the trimmed mean of M-value (TMM) method. Differential expression analysis of two treatments (PC versus NC) at three time points (4, 7, and 21 dpi) was performed; the resulting *p*-values were corrected using the Benjamini-Hochberg procedure. The DEGs were determined with a level of absolute log_2_ fold-change (FC) ≥ 1 on an adjusted false discovery rate (FDR) corrected *p*-value of < 0.05.

### Gene clustering analysis and gene co-expression network construction

After DEGs had been identified, their expression changes over time were analyzed via gene clustering. Genes were classified with the k-means clustering algorithm using each normalized TMM count. The k value that best distinguished the features of the gene expression pattern was used for subsequent analysis. MeV v4.9.0 software was used for the analysis and visualization of the results.

GCNs were established to enable analysis of the functional associations of genes. DEGs identified at least once at each time point were collected; instances of significant co-expression between genes were determined using the partial correlation coefficient with information theory (PCIT) algorithm. Associations with an absolute co-expression correlation ≥0.90 between DEGs were used to construct GCNs. After construction, GCNs containing at least 10 genes were considered meaningful. Cytoscape v3.8.2 software was used for network visualization.

### Functional enrichment analysis

Annotations of gene ontology (GO) terms and a Kyoto Encyclopedia of Genes and Genomes (KEGG) pathway were performed for the enrichment analysis of genes clustered in gene groups. The biological process (BP) and molecular function (MF) databases were used for GO analysis. ClusterProfiler (Yu et al., 2012) R packages in Bioconductor were used for GO and KEGG analysis, and with a significant cut-off of *p* < 0.05. All annotation procedures were performed in *Gallus gallus*.

To identify significantly different enrichment terms between the PC and NC treatments at each time point, gene set enrichment analysis (GSEA) was performed via GSEA v4.1.0 software (Subramanian et al., 2005; Luo and Brouwer, 2013) using log_2_-normalized TMM counts of genes at each time point. Gene sets in the BP, cellular component, MF, and KEGG databases were used for the analysis; significant pathways (FDR-corrected *p* < 0.05) in the gene sets were listed as results.

### Validation of RNA-Seq analysis via qRT-PCR

RNA isolation and cDNA synthesis were performed using cecum tissue samples from each individual of the treatment at each of the three time points. A Qiagen RNeasy kit was used for isolation; mRNA was converted into cDNA in the presence of Oligo d(T) primer (SuperScript IV, Invitrogen). Nine DEGs from each expression type were selected for validation; primers for qRT-PCR were designed using Primer-BLAST (https://www.ncbi.nlm.nih.gov/-tools/primer-blast/) and are listed (Supplementary Table S1 of Supplementary Material S1). qRT-PCR was carried out on a CFX Connect Real-Time System (Bio-Rad, Hercules, CA, United States) using SYBR green master mix (GeNetBio, Daejeon, Korea), in accordance with the manufacturer’s instructions. Relative gene expression was calculated using the delta-delta-Ct method. Finally, correlations of gene expression between the qRT-PCR and RNA-Seq analyses were estimated using R^2^ value.

## Results

### Gene expression analysis

**FIGURE 2:**
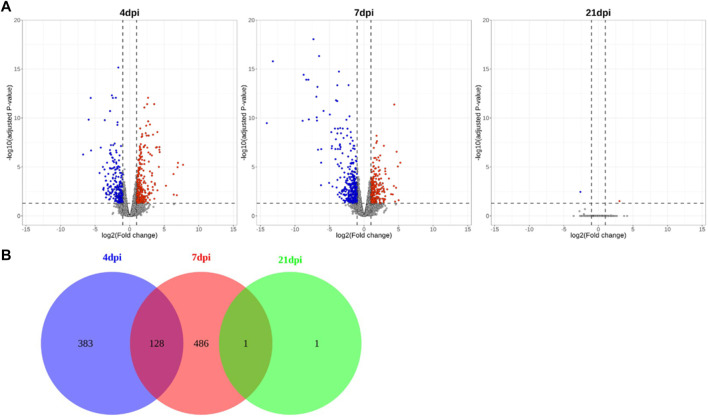
Volcano plots and Venn diagram showing significant DEGs obtained via time-series comparison between PC and NC treatments. **(A)** Volcano plots showing different time points, **(B)** Venn diagram indicating numbers of DEGs.

In total, 983 million paired-end reads were generated using cecum tissue samples from 24 chickens. Read data were processed (processing overview is shown in Supplementary Materials S2) and mapped to the chicken reference genome (GRCg6a). Based on the calculated FC, 511 and 615 significant DEGs were detected at 4 and 7 dpi, respectively, whereas only 2 DEGs were found at 21 dpi; these results indicated a stark contrast, as demonstrated in volcano plots and a Venn diagram (Figure 2). Over time, the trends in gene regulation changed. At 4 dpi, 53% of the DEGs were upregulated, while this value was only 41% at 7 dpi. The number of DEGs indicates that the difference in gene expression between the NC and PC treatments was small at 21 dpi. Analysis of genes from all time points together revealed 999 unique DEGs in *Eimeria*-infected chickens.

### Clustering genes and gene co-expression network analysis

**FIGURE 3:**
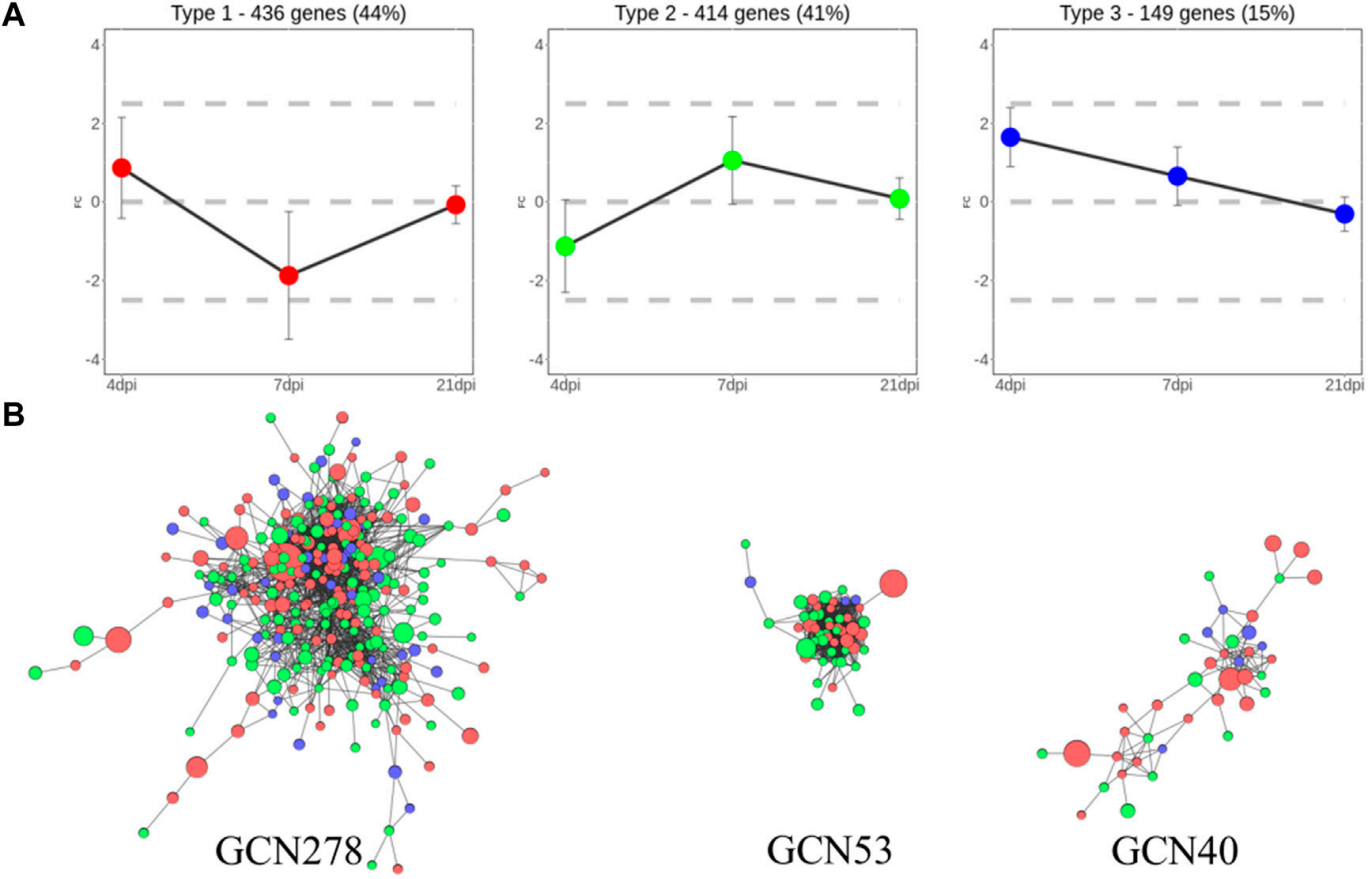
Clustered DEGs using two different grouping methods **(A)** Three types of clusters separated based on their gene expression patterns. In total, 436, 414, and 149 genes were assigned to Types 1, 2, and 3, respectively. **(B)** GCNs clustered using gene co-expression values. Each node is a gene colored with its expression type. Node size reflects absolute log_2_FC. Edges between nodes represent significant co-expression of genes. Network names reflect the number of genes they contain.

To analyze the biological regulation of the host response to *Eimeria* infection, DEGs were classified based on their expression patterns at each of the three time points. Overall, 999 genes were clustered into three groups, which comprised 436, 414, and 149 genes, respectively. The groups were referred to as Types 1 to 3 because of their distinct expression patterns, as shown in Figure 3A. The mean log_2_FC values of Type I genes over time were 0.87, −1.87, and −0.07, respectively; thus, expression level tended to sharply decrease after an initially high level in the initial stage of infection. In contrast, Type 2 genes were not active in the early stage of infection, then rebounded at 7 dpi and gradually normalized; the mean log_2_FC values over time were -1.13, 1.06, and 0.08, respectively. The mean log_2_FC values of Type 3 genes were 1.65, 0.65, and -0.31, respectively; these values tended to be high at 4 dpi, then decreased over time. Notably, 106 Type 1 genes were significantly upregulated at 4 dpi, then significantly downregulated at 7 dpi. Among Type 2 genes, 66 exhibited inverted regulation from 4 to 7 dpi.

For additional clustering, GCNs were constructed using 371 genes with 2,656 connections after data had been passed through the PCIT algorithm. Three meaningful networks were formed; they were named GCN278, GCN53, and GCN40, based on the number of genes included in each network. Co-expression findings did not match the expression pattern clusters, as shown in Figure 3B. GCN278 comprised 111 Type 1 genes, 126 Type 2 genes, and 41 Type 3 genes. GCN53 comprised 22 Type 1 genes, 28 Type 2 genes, and three Type 3 genes (i.e., only a small number of Type 3 genes). GCN40 consisted of 21 Type 1 genes, 13 Type 2 genes, and six Type 3 genes; thus, it was characterized by a high proportion of Type 1 genes. All expression values, expression type group, and GCNs assigned to each DEGs are summarized in Supplementary Materials S3.

### Functional enrichment analyses

**FIGURE 4:**
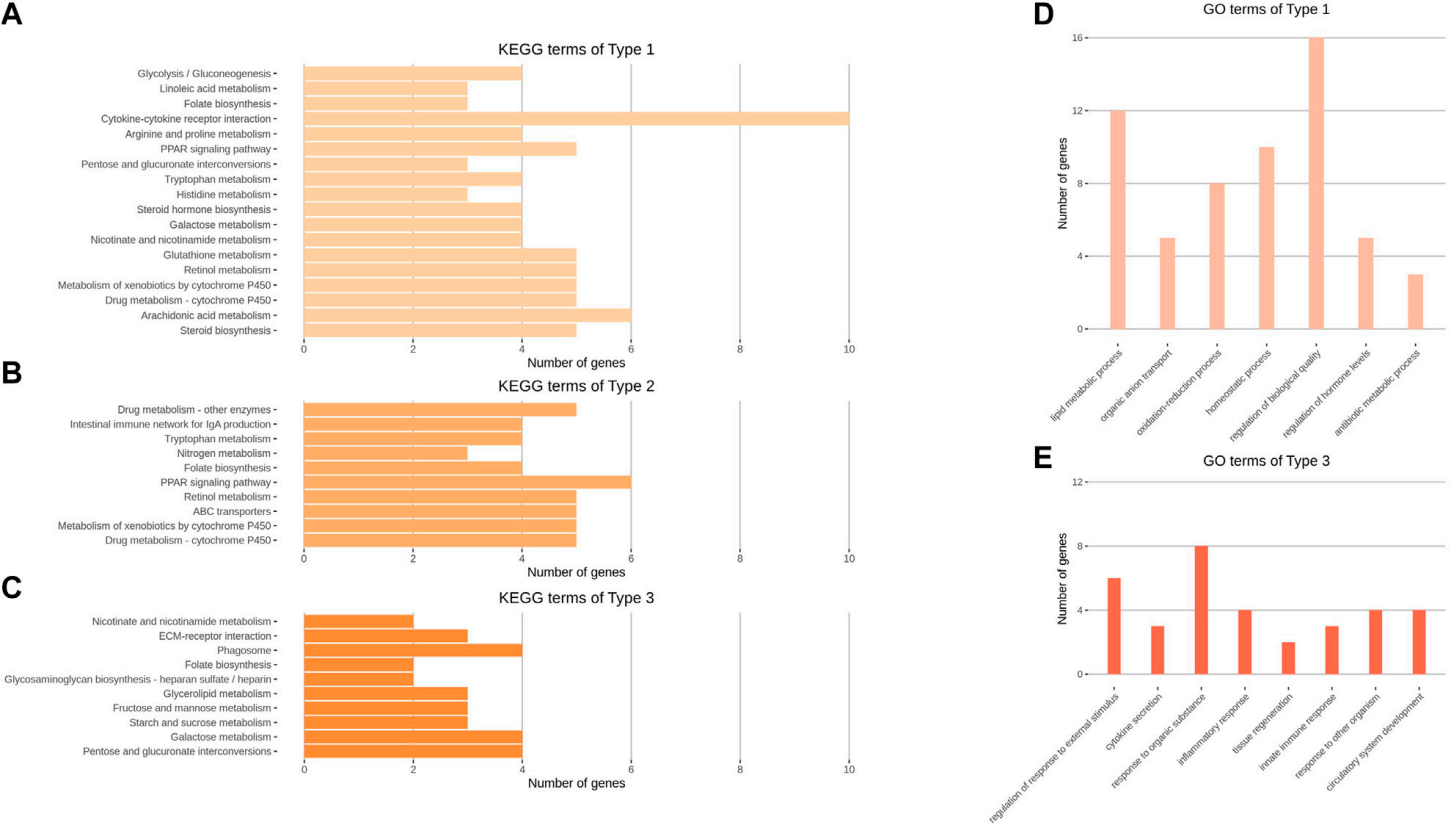
KEGG and GO analysis results of Type 1, 2, and 3 gene clusters. **(A–C)** Enriched KEGG pathways of Type 1, 2, and 3 genes clustered using the k-means clustering method. **(D–E)** Enriched GO terms of the biological process category of Types 1 and 3 gene clusters. Representative GO terms were selected from 53 to 188 significant terms of Types 1 and 3, respectively. GO analysis of Type 2 genes did not show any significant terms. **(A–E)** All enrichment terms passed a significance cut-off (*p* < 0.05) and were sorted in the order of largest significance levels.

**FIGURE 5:**
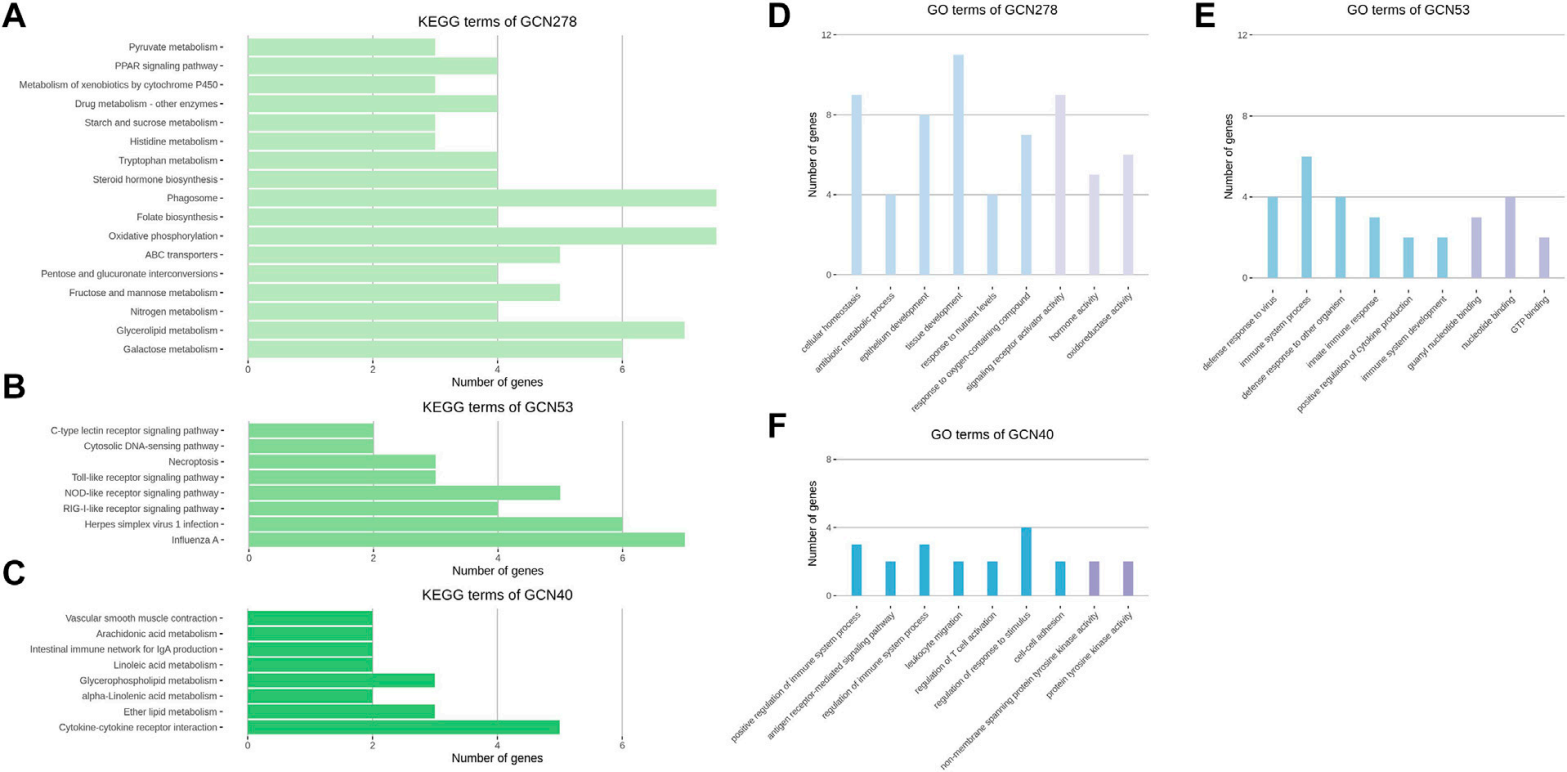
KEGG and GO analysis results of three GCNs. **(A–C)** Enriched KEGG pathways of the GCN278, GCN53, and GCN40 networks. **(D–F)** Enriched GO terms of the GCN278, GCN53, and GCN40 networks. GO terms were categorized as a biological process and molecular functions (colored blue and purple, respectively). Representative GO terms were selected from 26, 42, and 70 significant biological process terms and 12, 15, and 6 significant molecular functions terms for GCN278, GCN53, and GCN40, respectively. **(A–F)** All enrichment terms passed a significance cut-off (*p* < 0.05) and were sorted in the order of largest significance levels.

GO and KEGG enrichment analyses were performed on the three types of expression pattern clusters and three GCNs. The results of the analyses are presented in Figures 4, 5, and Supplementary Materials S4. The top eight BP terms for the GO analysis of Type 1 genes involved lipid production and utilization processes; other functions were also observed (e.g., organic anion transport, homeostatic process, regulation of biological quality, and antibiotic metabolic process). Among Type 3 genes, BP terms related to the innate immune response (e.g., regulation of defense response, cytokine secretion, and inflammatory response) exhibited high significance; tissue regeneration and circulatory system development terms were also observed. No significant (*p* < 0.05) terms were found among Type 2 genes (Figure 4D,ED).

Characteristic results of KEGG analysis were identified in expression pattern clusters. Type 1 terms included steroid biosynthesis and cytokine-cytokine receptor interaction, while Type 2 terms included ABC transporters, nitrogen metabolism, and intestinal immune network for IgA production. Type 3 terms included nutrient metabolism, such as starch and sucrose metabolism, and phagosome and extracellular matrix–receptor interaction functions (Figure 4A–C).

The results of GCN functional analysis were as follows. The BP terms for GCN278 were cellular homeostasis, antibiotic metabolic process, and epithelium development; the MF terms were signaling receptor activator activity, hormone activity, and oxidoreductase activity. The top BP terms for GCN53 were innate immune response-related functions; nucleotide binding-related terms were identified in the MF database. In the GO analysis of GCN40, positive regulation of immune system process was the most significant BP term, together with leukocyte migration and cell-cell adhesion. Among the MF terms, functions related to protein tyrosine kinase activity were identified (Figure 5D–FD).

KEGG analysis of GCNs revealed distinct biological functions. In GCN278, there were oxidative phosphorylation and PPAR signaling pathway terms; in GCN53, influenza A and several types of receptor signaling pathways were enriched. Finally, in GCN40, the cytokine-cytokine receptor interaction, lipid metabolism, and intestinal immune network for IgA production terms were identified (Figure 5A–C).

### Gene set enrichment analysis results associated with the time-series host response to *Eimeria* infection

GSEA was performed on the GO and KEGG databases to focus on the functions performed over time during *Eimeria* infection (Supplementary Material S5). At 21 dpi, genes with a small differential expression level were excluded from the analysis. Among the KEGG gene sets upregulated at 4 dpi after *Eimeria* infection, the most enriched term was steroid biosynthesis, consistent with the KEGG analysis results for Type 1 DEGs. There were 12 core enrichment genes in steroid biosynthesis; seven were DEGs. In addition, inflammatory response-related signaling pathways were upregulated terms. In terms of the normalized enrichment score, six of the top 10 GO terms were related to lipid or sterol synthesis and metabolic functions. In addition, the keratinization function had a high significance, such that 19 core enriched genes (including four DEGs) constituted the gene set.

Nutrient metabolic function terms such as glutathione, tyrosine, nitrogen, and phenylalanine were found in the KEGG terms upregulated at 7 dpi, consistent with the KEGG analysis results for Type 2 and Type 3 clusters. In addition, epithelial cell signaling in the *Helicobacter pylori* infection KEGG term was upregulated at 7 dpi, with 12 core enriched genes containing nine DEGs. GO analysis revealed many oxidative phosphorylation-related terms with a high normalized enrichment score; among the terms belonging to BP, the phagosome acidification term contained 10 core enrichment genes (including six DEGs) in the gene set. GSEA confirmed the functions downregulated during *Eimeria* infection. KEGG analysis at 7 dpi showed that the natural killer cell-mediated cytotoxicity pathway had 40 core genes, including three DEGs. GO analysis identified the phosphotyrosine residue binding term in the MF database, with 22 core-enriched genes including two DEGs.

### qRT-PCR

To validate the differential expression indicated in RNA-Seq analysis, qRT-PCR was conducted using nine genes that were selected according to their gene expression types. The target genes were *FABP2* (fatty acid binding protein 2), *IFI6* (interferon alpha inducible protein 6), *INSIG1* (insulin induced gene 1), *KRT40* (keratin 40), *IL13RA2* (interleukin 13 receptor subunit alpha 2), *ART7B* (GPI-anchored ADP-ribosyltransferase), *IRF9* (interferon regulatory factor 9), *CCKAR* (cholecystokinin A receptor), and *XKR9* (XK related 9). Relative quantification of gene expression was performed with *ACTB* (actin, beta) and *YWHAZ* (tyrosine 3-monooxygenase-/tryptophan 5-monooxygenase activation protein zeta) as control genes. Experimental samples were selected among the same treatment accordant with those used in RNA-Seq analysis. Throughout the three time points, expression values were calculated (Supplementary Table S2 in Supplementary Material S1) and the mean correlation of the expression patterns of the genes between RNA-Seq and qRT-PCR reached 0.811 (R^2^ = 0.658). Therefore, statistical analysis showed good correspondence between the qRT-PCR and RNA-Seq results.

## Discussion

Coccidiosis is a critical parasitic disease in the chicken industry. To overcome the situation, recent studies indicated that chages from traditional methods to develop more effective anticoccidial vaccine (Venkatas and Adeleke, 2019). As another approach to the promising strategies to prevent the disease, this study aimed to characterize the host immune response that occurs in chickens under coccidiosis infection.

### Blended effects on gene expression of cecum by multiple *Eimeria* species infection

*Eimeria* species is known as habitat-specific (Li et al., 2020). Accordingly, *Eimeria acervulina*, *Eimeria tenella*, and *Eimeria maxima* inhabit basically in duodenum, cecum, and jejunum respectively (Raman et al., 2011; Quiroz-Castañeda and Dantán-González, 2015). As the mRNA derived from the cecum tissue has been studied here, most of the effects on the identified responses are attributed to *E. tenella*. However, Li et al. (2019) observed that *E. maxima*-infection also affected the differential gene expression in the cecum tissue of chicken. Nonetheless, DEGs and the corresponding functions tended to be similar to those of chickens infected with *E. tenella*. Further, in the cecum, the transcriptome levels of genes responsive to *E. tenella* is known to be higher (Li et al., 2019). Hence, it manifests that the major effects described in this study are due to *E. tenella* and while some minor effects are shared with the other two strains.

### Changes in number of differentially expressed genes over time

Whole serial transcriptomes for three time points (4, 7, and 21 dpi) after mixed strains of *Eimeria* infection were compared and integrated. The number of DEGs increased from 4 to 7 dpi, but DEGs almost disappeared at 21 dpi. According to Morris et al. (2007) and Long et al. (1981), coccidiosis-damaged cecal mucosa expresses inflammatory symptoms before 8 to 10 dpi; the mucosal surface then becomes normal. After 7 dpi, the degree of damage is alleviated and the host response decreases; thus, fewer significant differences were observed at 21 dpi.

An increase in the number of downregulated genes was observed at 7 dpi, consistent with the previous finding that strong downregulation occurred in chicken immune cells after infection (Sandholt et al., 2021); the effect of self-regulation was evident at 7 dpi. Self-regulation may be an immune mechanism to alleviate inflammation; alternatively, *Eimeria* may induce downregulation of host pathways as an immune evasion mechanism (Sandholt et al., 2021).

### Functional analysis of endoplasmic reticulum stress response under *Eimeria* infection

GO, KEGG, and GSEA assessments of the Type 1 expression pattern cluster in this study suggested that lipid and sterol synthesis and metabolism are highly active at 4 dpi during *Eimeria* infection. Furthermore, some studies have shown that host cells can undergo ER stress because of intestinal parasite infection (e.g., coccidiosis), leading to an unfolded protein response (Galluzzi et al., 2017). A previous microarray analysis of intraepithelial lymphocytes from *Eimeria*-infected chickens revealed activation of the ER stress response mechanism (Liu et al., 2021). The ER is an organelle that synthesizes lipids; when homeostasis is disrupted because of continuous ER stress and excessive unfolded protein response, lipid synthesis regulation fails, eventually leading to metabolic disorders and apoptosis (Han and Kaufman, 2016). Therefore, the rapid increases in lipid synthesis and metabolism in the chickens at 4 dpi in this study were caused by *Eimeria* infection-related ER stress. To overcome the risk of ER homeostasis disruption, cells reduce the ribosome and protein synthesis pathways by downregulating the transcription of rRNA; this reduces the overall burden on the ER (Hotamisligil, 2010; Han and Kaufman, 2016). Thus, the GSEA results in this study indicated that downregulation of the ribosome term at 4 dpi occurred in response to ER stress. Furthermore, GSEA of the data from 7 dpi indicated upregulation of intracellular ribosome function, suggesting that ER homeostasis had been restored by that time. Key Type 1 genes for lipid biosynthesis (e.g., *FDPS*, *BC O 1*, and *EXFABP*) showed significant downregulation at 7 dpi; this presumably aided in ER homeostasis.

### Innate immune response after *Eimeria* infection

The findings in GO analysis of Type 3 genes could be explained by the innate immune response to *Eimeria* infection, including both the direct defense mechanisms and cytokines released *via* leukocyte activity and inflammatory responses. Innate immune responses were initially active and relieved afterward, following the chronological expression of Type 3 genes. In addition, functional enrichment analysis indicated that genes in the GCN53 network were mainly responsible for the innate immune mechanism. In the KEGG analysis of GCN53, DEGs included in influenza A and herpes simplex virus 1 infection-related terms showed high significance; these genes also have a protective function during coccidiosis. The activation of necroptosis and pattern recognition receptor (RIG-I-like receptor, NOD-like receptor, and Toll-like receptor) signaling pathways leads to the release of pathogen-associated molecular patterns or danger-associated molecular patterns through the death of infected cells. This release is likely to promote innate immune system activation and the upregulation of inflammatory responses (Franchi et al., 2009; Haunshi et al., 2017). In addition, among the terms upregulated at 4 dpi in the GSEA results, the virus defense response, MDA5 signaling, interferon I, response to cytokine stimulus, and adipocytokine signaling pathway terms supported the results of the analysis described above. Because the keratinization, cornification, epidermis development, and cornified envelope terms exhibited concomitant upregulation at 4 dpi, the development and differentiation of intestinal epithelial cells were presumed to defend against parasitic stimuli. Furthermore, the formation of a physical barrier through keratinization, such as the response of gizzard epithelial cells to parasitic infection in waterfowl (Padilla-Aguilar et al., 2020) provided a potential defense against *Eimeria* invasion. For the same purpose, bicellular tight junction assembly, a GO term upregulated at 7 dpi according to GSEA, forms a gut barrier through cell proliferation, differentiation, and organization (von Buchholz et al., 2021).

### Cytokine-cytokine receptor interaction and the anti-inflammatory process in response to *Eimeria* infection

The cytokine-cytokine receptor interaction term had the largest number of genes (10 genes) among the KEGG enrichment analysis results of the Type 1 cluster. Genes related to the cytokine-cytokine receptor interaction term belonged to the Type 1 cluster; these included *IL13RA2* (interleukin 13 receptor subunit alpha 2), *CXCL13* (CXC motif chemokine ligand 13), *CXCR5* (CXC motif chemokine receptor 5), *GDF15* (growth differentiation factor 15), *IL7R* (interleukin 7 receptor), *IL12RB2* (interleukin 12 receptor subunit beta 2), *BMP7* (bone morphogenetic protein 7), *TNFRSF11B* (TNF receptor superfamily member 11b), *IL22* (interleukin 22), and *IFNGR1* (interferon-gamma receptor 1). These genes were involved in the inflammatory pathway; they were also responsible for activating the immune response through the migration of T and B lymphocytes. Importantly, growth differentiation factor 15 levels have been shown to increase in injured tissues; this gene has a role in inflammatory processes (Wischhusen et al., 2020). Moreover, interleukin 12 receptor subunit beta 2 contributes to the inflammatory response and host defense (Zou et al., 1997). All genes except *IFNGR1* were upregulated at 4 dpi during *Eimeria* infection, although they were significantly downregulated at 7 dpi. The *IFNGR1* gene codes interferon-gamma receptor 1; it interacts with interferon-gamma molecules. Interferon-gamma, a cytokine produced by macrophages, has an important role in cell-mediated acquired immunity; it functions in a synergistic manner with major histocompatibility complex (MHC) class II molecules (Yun et al., 2000). There have also been reports of the protective immune functions of interferon-gamma against *Eimeria* (Shah et al., 2010). However, in this study, significant downregulation of the *IFNGR1* gene continued at 4 and 7 dpi. The interferon-gamma-related function may have been activated in chickens immediately after infection (i.e., before the first sampling), and the downregulation began prior to 4 dpi. Similar to the immune systems described by Sandholt et al. (2021), the cytokine-cytokine receptor interaction pathway exhibits continuous downregulation to suppress the *Eimeria*-induced inflammatory response and autoimmunity after the initial stage of innate immune activity.

KEGG analyses of GCN40 revealed that the cytokine-cytokine receptor interaction pathway exhibited the highest enrichment score; BP terms related to the regulation of the immune system process were identified in the GO analysis of GCN40. The *BTK*, *SYK*, and *FAM65B* genes were the enriched core genes for the BP terms. *BTK* and *SYK* together have critical roles in the immune response that involve the activation of PLC-gamma, which is necessary for the activity and migration of B and T lymphocytes (Kurosaki and Tsukada, 2000). In the present study, *BYK* and *SYK* genes were significantly downregulated at 4 and 7 dpi, respectively. In contrast, the *FAM65B* gene was significantly upregulated at 7 dpi, in association with negative regulation of the leukocyte activation GO term. Therefore, while an active innate immune response was underway against *Eimeria* invasion, opposing mechanisms to inhibit lymphocyte activity were activated for anti-inflammatory effects, as described above.

## Conclusion

In this study, time-series host response pathways in *Eimeria*-infected broiler chicken were observed via RNA-Seq. From 4dpi to 21dpi, *Eimeria-*infected chickens experienced dynamic changes; a total of three types of gene expression patterns, which are characterized by fluctuant up and down regulation and the different number of DEGs. These transitions reflected the real-time state of immune and homeostasis mechanisms against parasitic invasion. Upon closer inspection, organelle malfunction and activated innate immune response occurred at 4 dpi, and the next, recovery from the impairment and inflammation control were dominant at 7 dpi. Eventually, there were few significant DEGs between infected and uninfected birds at 21dpi. In other words, we summarized that the main host responses were related to ER stress-induced functional changes and the signaling systems responsible for the innate immune response. Additionally, gene clustering analysis and GCN analysis revealed networks of genes that play important roles during *Eimeria* infection. For example, the Type 3 cluster and GCN53 genes are mostly involved with innate immune response functions, and the part of Type 1 cluster and GCN40 genes mainly work for inflammation and anti-inflammation respectively. The identification of significant DEGs and the results of the gene grouping analysis in this study will help to improve disease control by aiding in the selection of chickens.

## Data Availability

The datasets presented in this study can be found in online repositories. The names of the repository/repositories and accession number(s) can be found below: NCBI SRA BioProject, accession no: PRJNA844579.
