# Supplementary material for: Time-series transcriptome analysis identified differentially expressed genes in broiler chicken infected with mixed Eimeria species
Source: Front Genet. 2022 Aug 8;13:886781. doi: 10.3389/fgene.2022.886781 (PMC9393255; doi:10.3389/fgene.2022.886781)
Supplement: Supplementary file 2 [file DataSheet1.ZIP › 4dpi_GO.Gsea.1625071243202/GOBP_ACYLGLYCEROL_HOMEOSTASIS.html]

Details for gene set GOBP\_ACYLGLYCEROL\_HOMEOSTASIS[GSEA]

|  || Dataset | TMM\_4dpi\_gct\_format\_4dpi\_gct\_format.Class\_4dpi.cls #PC\_versus\_NC.Class\_4dpi.cls #PC\_versus\_NC\_repos |
| Phenotype | Class\_4dpi.cls#PC\_versus\_NC\_repos |
| Upregulated in class | 1 |
| GeneSet | GOBP\_ACYLGLYCEROL\_HOMEOSTASIS |
| Enrichment Score (ES) | 0.772853 |
| Normalized Enrichment Score (NES) | 2.1484587 |
| Nominal p-value | 0.0 |
| FDR q-value | 0.0015179211 |
| FWER p-Value | 0.0184 |
Table: GSEA Results Summary

  

Fig 1: Enrichment plot: GOBP\_ACYLGLYCEROL\_HOMEOSTASIS      
 Profile of the Running ES Score & Positions of GeneSet Members on the Rank Ordered List

  

| SYMBOL | TITLE | RANK IN GENE LIST | RANK METRIC SCORE | RUNNING ES | CORE ENRICHMENT || 1 | ABCG8 | na | 2 | 3.384 | 0.2120 | Yes |
| 2 | ABCG5 | na | 41 | 2.059 | 0.3380 | Yes |
| 3 | PLA2G12B | na | 115 | 1.564 | 0.4300 | Yes |
| 4 | PNPLA8 | na | 119 | 1.559 | 0.5275 | Yes |
| 5 | APOA1 | na | 589 | 0.889 | 0.5441 | Yes |
| 6 | DGAT2 | na | 643 | 0.854 | 0.5933 | Yes |
| 7 | APOA4 | na | 733 | 0.797 | 0.6359 | Yes |
| 8 | LPL | na | 954 | 0.691 | 0.6609 | Yes |
| 9 | IL18 | na | 1198 | 0.604 | 0.6785 | Yes |
| 10 | MLXIPL | na | 1199 | 0.604 | 0.7163 | Yes |
| 11 | ANGPTL4 | na | 1324 | 0.568 | 0.7416 | Yes |
| 12 | HNF4A | na | 1369 | 0.556 | 0.7729 | Yes |
| 13 | RORA | na | 2627 | 0.332 | 0.6889 | No |
| 14 | NR1H3 | na | 3213 | 0.255 | 0.6561 | No |
| 15 | SCARB1 | na | 5664 | 0.020 | 0.4530 | No |
| 16 | XBP1 | na | 5735 | 0.014 | 0.4481 | No |
| 17 | NR1H4 | na | 7657 | -0.148 | 0.2972 | No |
| 18 | SIRT1 | na | 9868 | -0.394 | 0.1376 | No |
| 19 | SESN2 | na | 11162 | -0.653 | 0.0707 | No |
Table: GSEA details [plain text format]

  

Fig 2: GOBP\_ACYLGLYCEROL\_HOMEOSTASIS      
 Blue-Pink O' Gram in the Space of the Analyzed GeneSet

  

Fig 3: GOBP\_ACYLGLYCEROL\_HOMEOSTASIS: Random ES distribution      
 Gene set null distribution of ES for **GOBP\_ACYLGLYCEROL\_HOMEOSTASIS**

  
