# Supplementary material for: Time-series transcriptome analysis identified differentially expressed genes in broiler chicken infected with mixed Eimeria species
Source: Front Genet. 2022 Aug 8;13:886781. doi: 10.3389/fgene.2022.886781 (PMC9393255; doi:10.3389/fgene.2022.886781)
Supplement: Supplementary file 2 [file DataSheet1.ZIP › 4dpi_GO.Gsea.1625071243202/GOBP_CYTOPLASMIC_TRANSLATION.html]

Details for gene set GOBP\_CYTOPLASMIC\_TRANSLATION[GSEA]

|  || Dataset | TMM\_4dpi\_gct\_format\_4dpi\_gct\_format.Class\_4dpi.cls #PC\_versus\_NC.Class\_4dpi.cls #PC\_versus\_NC\_repos |
| Phenotype | Class\_4dpi.cls#PC\_versus\_NC\_repos |
| Upregulated in class | 0 |
| GeneSet | GOBP\_CYTOPLASMIC\_TRANSLATION |
| Enrichment Score (ES) | -0.627917 |
| Normalized Enrichment Score (NES) | -2.5295384 |
| Nominal p-value | 0.0 |
| FDR q-value | 0.0 |
| FWER p-Value | 0.0 |
Table: GSEA Results Summary

  

Fig 1: Enrichment plot: GOBP\_CYTOPLASMIC\_TRANSLATION      
 Profile of the Running ES Score & Positions of GeneSet Members on the Rank Ordered List

  

| SYMBOL | TITLE | RANK IN GENE LIST | RANK METRIC SCORE | RUNNING ES | CORE ENRICHMENT || 1 | FMR1 | na | 647 | 0.851 | -0.0325 | No |
| 2 | CPEB3 | na | 738 | 0.795 | -0.0197 | No |
| 3 | UNK | na | 1916 | 0.437 | -0.1071 | No |
| 4 | CPEB1 | na | 2205 | 0.388 | -0.1213 | No |
| 5 | DHX29 | na | 2779 | 0.311 | -0.1614 | No |
| 6 | EIF4H | na | 3405 | 0.232 | -0.2079 | No |
| 7 | EIF2S2 | na | 4294 | 0.142 | -0.2786 | No |
| 8 | MTOR | na | 4329 | 0.140 | -0.2779 | No |
| 9 | CPEB4 | na | 4588 | 0.117 | -0.2966 | No |
| 10 | DENR | na | 4591 | 0.117 | -0.2937 | No |
| 11 | EIF5 | na | 4786 | 0.099 | -0.3075 | No |
| 12 | NCK1 | na | 4852 | 0.093 | -0.3105 | No |
| 13 | CNBP | na | 5374 | 0.048 | -0.3530 | No |
| 14 | ZC3H15 | na | 5450 | 0.041 | -0.3582 | No |
| 15 | EIF4G1 | na | 5833 | 0.007 | -0.3901 | No |
| 16 | GSPT2 | na | 6351 | -0.034 | -0.4325 | No |
| 17 | DNAJC24 | na | 6842 | -0.074 | -0.4717 | No |
| 18 | DRG2 | na | 6891 | -0.078 | -0.4737 | No |
| 19 | EIF2B3 | na | 7252 | -0.113 | -0.5010 | No |
| 20 | ETF1 | na | 7386 | -0.125 | -0.5089 | No |
| 21 | DPH5 | na | 7544 | -0.138 | -0.5186 | No |
| 22 | YBX3 | na | 7689 | -0.152 | -0.5267 | No |
| 23 | DHX36 | na | 7816 | -0.163 | -0.5331 | No |
| 24 | EIF3J | na | 7832 | -0.164 | -0.5302 | No |
| 25 | RPL22L1 | na | 8070 | -0.186 | -0.5453 | No |
| 26 | RPS23 | na | 8269 | -0.206 | -0.5566 | No |
| 27 | CPEB2 | na | 8397 | -0.218 | -0.5617 | No |
| 28 | RPL17 | na | 8583 | -0.239 | -0.5711 | No |
| 29 | MCTS1 | na | 8783 | -0.260 | -0.5811 | No |
| 30 | DPH1 | na | 8850 | -0.268 | -0.5798 | No |
| 31 | DRG1 | na | 8943 | -0.278 | -0.5804 | No |
| 32 | DPH7 | na | 9193 | -0.307 | -0.5934 | No |
| 33 | RPL36 | na | 9363 | -0.330 | -0.5991 | No |
| 34 | EIF2D | na | 9431 | -0.340 | -0.5960 | No |
| 35 | METTL3 | na | 9660 | -0.369 | -0.6057 | No |
| 36 | RPL38 | na | 9661 | -0.369 | -0.5962 | No |
| 37 | DPH3P1 | na | 10040 | -0.420 | -0.6172 | Yes |
| 38 | RPLP2 | na | 10048 | -0.421 | -0.6070 | Yes |
| 39 | EIF3I | na | 10227 | -0.450 | -0.6104 | Yes |
| 40 | DPH6 | na | 10267 | -0.455 | -0.6020 | Yes |
| 41 | RWDD1 | na | 10402 | -0.479 | -0.6009 | Yes |
| 42 | EIF4A2 | na | 10458 | -0.490 | -0.5930 | Yes |
| 43 | RPS28 | na | 10524 | -0.504 | -0.5856 | Yes |
| 44 | RPL30 | na | 10537 | -0.506 | -0.5736 | Yes |
| 45 | YTHDF2 | na | 10549 | -0.507 | -0.5616 | Yes |
| 46 | RPL22 | na | 10554 | -0.508 | -0.5489 | Yes |
| 47 | EIF3A | na | 10585 | -0.517 | -0.5382 | Yes |
| 48 | RPL36A | na | 10605 | -0.520 | -0.5265 | Yes |
| 49 | RPL29 | na | 10742 | -0.548 | -0.5238 | Yes |
| 50 | RBM24 | na | 10874 | -0.576 | -0.5201 | Yes |
| 51 | RPL24 | na | 10877 | -0.576 | -0.5055 | Yes |
| 52 | RPL35A | na | 10982 | -0.602 | -0.4988 | Yes |
| 53 | EIF4B | na | 11083 | -0.631 | -0.4910 | Yes |
| 54 | DPH2 | na | 11087 | -0.632 | -0.4751 | Yes |
| 55 | RPL6 | na | 11150 | -0.650 | -0.4637 | Yes |
| 56 | RPL11 | na | 11195 | -0.662 | -0.4504 | Yes |
| 57 | EIF3G | na | 11205 | -0.665 | -0.4341 | Yes |
| 58 | RPLP1 | na | 11249 | -0.682 | -0.4203 | Yes |
| 59 | RPS26 | na | 11254 | -0.686 | -0.4030 | Yes |
| 60 | EIF3B | na | 11276 | -0.694 | -0.3870 | Yes |
| 61 | RPL26L1 | na | 11283 | -0.698 | -0.3697 | Yes |
| 62 | RPL31 | na | 11306 | -0.706 | -0.3535 | Yes |
| 63 | RPS21 | na | 11313 | -0.709 | -0.3358 | Yes |
| 64 | RPL32 | na | 11355 | -0.725 | -0.3207 | Yes |
| 65 | EIF2S3 | na | 11414 | -0.759 | -0.3061 | Yes |
| 66 | RPL15 | na | 11440 | -0.769 | -0.2886 | Yes |
| 67 | RPS29 | na | 11444 | -0.771 | -0.2691 | Yes |
| 68 | RPL18A | na | 11474 | -0.789 | -0.2513 | Yes |
| 69 | RPLP0 | na | 11488 | -0.800 | -0.2319 | Yes |
| 70 | RPL9 | na | 11521 | -0.819 | -0.2137 | Yes |
| 71 | EIF3D | na | 11578 | -0.857 | -0.1964 | Yes |
| 72 | RPL19 | na | 11611 | -0.882 | -0.1765 | Yes |
| 73 | EEF2 | na | 11615 | -0.885 | -0.1541 | Yes |
| 74 | EIF3H | na | 11627 | -0.893 | -0.1322 | Yes |
| 75 | RPL10A | na | 11658 | -0.921 | -0.1111 | Yes |
| 76 | EIF3M | na | 11707 | -0.974 | -0.0902 | Yes |
| 77 | RPL8 | na | 11772 | -1.039 | -0.0690 | Yes |
| 78 | EIF3L | na | 11808 | -1.126 | -0.0431 | Yes |
| 79 | EIF3F | na | 11816 | -1.139 | -0.0145 | Yes |
| 80 | EIF3E | na | 11859 | -1.199 | 0.0127 | Yes |
Table: GSEA details [plain text format]

  

Fig 2: GOBP\_CYTOPLASMIC\_TRANSLATION      
 Blue-Pink O' Gram in the Space of the Analyzed GeneSet

  

Fig 3: GOBP\_CYTOPLASMIC\_TRANSLATION: Random ES distribution      
 Gene set null distribution of ES for **GOBP\_CYTOPLASMIC\_TRANSLATION**

  
