# Supplementary material for: Time-series transcriptome analysis identified differentially expressed genes in broiler chicken infected with mixed Eimeria species
Source: Front Genet. 2022 Aug 8;13:886781. doi: 10.3389/fgene.2022.886781 (PMC9393255; doi:10.3389/fgene.2022.886781)
Supplement: Supplementary file 2 [file DataSheet1.ZIP › 4dpi_GO.Gsea.1625071243202/GOBP_DEFENSE_RESPONSE_TO_VIRUS.html]

Details for gene set GOBP\_DEFENSE\_RESPONSE\_TO\_VIRUS[GSEA]

|  || Dataset | TMM\_4dpi\_gct\_format\_4dpi\_gct\_format.Class\_4dpi.cls #PC\_versus\_NC.Class\_4dpi.cls #PC\_versus\_NC\_repos |
| Phenotype | Class\_4dpi.cls#PC\_versus\_NC\_repos |
| Upregulated in class | 1 |
| GeneSet | GOBP\_DEFENSE\_RESPONSE\_TO\_VIRUS |
| Enrichment Score (ES) | 0.5437291 |
| Normalized Enrichment Score (NES) | 2.216894 |
| Nominal p-value | 0.0 |
| FDR q-value | 3.9133584E-4 |
| FWER p-Value | 0.0026 |
Table: GSEA Results Summary

  

Fig 1: Enrichment plot: GOBP\_DEFENSE\_RESPONSE\_TO\_VIRUS      
 Profile of the Running ES Score & Positions of GeneSet Members on the Rank Ordered List

  

| SYMBOL | TITLE | RANK IN GENE LIST | RANK METRIC SCORE | RUNNING ES | CORE ENRICHMENT || 1 | DDX60 | na | 18 | 2.282 | 0.0296 | Yes |
| 2 | ZC3HAV1 | na | 21 | 2.247 | 0.0600 | Yes |
| 3 | IFIH1 | na | 23 | 2.222 | 0.0901 | Yes |
| 4 | MOV10 | na | 27 | 2.190 | 0.1197 | Yes |
| 5 | GBP1 | na | 34 | 2.116 | 0.1480 | Yes |
| 6 | MX1 | na | 36 | 2.093 | 0.1764 | Yes |
| 7 | TLR3 | na | 40 | 2.065 | 0.2043 | Yes |
| 8 | OASL | na | 52 | 2.001 | 0.2306 | Yes |
| 9 | PARP9 | na | 53 | 1.996 | 0.2578 | Yes |
| 10 | STAT1 | na | 78 | 1.743 | 0.2795 | Yes |
| 11 | IRF7 | na | 85 | 1.706 | 0.3022 | Yes |
| 12 | IRF9 | na | 103 | 1.617 | 0.3228 | Yes |
| 13 | IRF1 | na | 114 | 1.568 | 0.3433 | Yes |
| 14 | DHX58 | na | 116 | 1.564 | 0.3645 | Yes |
| 15 | EIF2AK2 | na | 139 | 1.499 | 0.3831 | Yes |
| 16 | DTX3L | na | 143 | 1.489 | 0.4031 | Yes |
| 17 | IFI6 | na | 145 | 1.476 | 0.4231 | Yes |
| 18 | NLRC5 | na | 162 | 1.431 | 0.4412 | Yes |
| 19 | SAMHD1 | na | 170 | 1.414 | 0.4599 | Yes |
| 20 | RSAD2 | na | 220 | 1.319 | 0.4737 | Yes |
| 21 | IFIT5 | na | 253 | 1.247 | 0.4880 | Yes |
| 22 | TRAF3IP2 | na | 293 | 1.183 | 0.5008 | Yes |
| 23 | RIPK3 | na | 409 | 1.055 | 0.5055 | Yes |
| 24 | ADAR | na | 412 | 1.051 | 0.5196 | Yes |
| 25 | IL10RB | na | 437 | 1.026 | 0.5316 | Yes |
| 26 | BIRC3 | na | 482 | 0.983 | 0.5413 | Yes |
| 27 | RIOK3 | na | 724 | 0.802 | 0.5319 | Yes |
| 28 | STAT2 | na | 751 | 0.788 | 0.5404 | Yes |
| 29 | PMAIP1 | na | 980 | 0.678 | 0.5305 | Yes |
| 30 | EXOC1 | na | 1020 | 0.665 | 0.5362 | Yes |
| 31 | IFNAR2 | na | 1101 | 0.637 | 0.5382 | Yes |
| 32 | TSPAN6 | na | 1137 | 0.625 | 0.5437 | Yes |
| 33 | KCNJ8 | na | 1854 | 0.447 | 0.4895 | No |
| 34 | EIF2AK4 | na | 2101 | 0.406 | 0.4743 | No |
| 35 | RNF216 | na | 2108 | 0.405 | 0.4793 | No |
| 36 | TRAF3IP1 | na | 2234 | 0.383 | 0.4740 | No |
| 37 | POLR3A | na | 2318 | 0.373 | 0.4721 | No |
| 38 | GPAM | na | 2506 | 0.348 | 0.4611 | No |
| 39 | TOMM70 | na | 2562 | 0.341 | 0.4611 | No |
| 40 | BECN1 | na | 2572 | 0.338 | 0.4650 | No |
| 41 | AGBL4 | na | 2634 | 0.331 | 0.4643 | No |
| 42 | IL15 | na | 2691 | 0.325 | 0.4640 | No |
| 43 | F2RL1 | na | 2701 | 0.323 | 0.4677 | No |
| 44 | RNASEL | na | 2765 | 0.313 | 0.4666 | No |
| 45 | FADD | na | 2797 | 0.309 | 0.4682 | No |
| 46 | IL12RB1 | na | 2978 | 0.285 | 0.4569 | No |
| 47 | SELENOK | na | 3100 | 0.269 | 0.4504 | No |
| 48 | NLRX1 | na | 3156 | 0.262 | 0.4493 | No |
| 49 | DDX1 | na | 3186 | 0.258 | 0.4504 | No |
| 50 | ANKRD17 | na | 3379 | 0.235 | 0.4374 | No |
| 51 | RNASE6 | na | 3620 | 0.205 | 0.4200 | No |
| 52 | ABCC9 | na | 3637 | 0.204 | 0.4215 | No |
| 53 | TKFC | na | 3694 | 0.199 | 0.4194 | No |
| 54 | POLR3D | na | 3731 | 0.195 | 0.4191 | No |
| 55 | CNOT7 | na | 3796 | 0.188 | 0.4162 | No |
| 56 | ZMYND11 | na | 4125 | 0.157 | 0.3908 | No |
| 57 | FLNA | na | 4149 | 0.155 | 0.3909 | No |
| 58 | PTPN22 | na | 4177 | 0.153 | 0.3907 | No |
| 59 | SKP2 | na | 4210 | 0.150 | 0.3901 | No |
| 60 | ITCH | na | 4477 | 0.126 | 0.3694 | No |
| 61 | PCBP2 | na | 4714 | 0.105 | 0.3510 | No |
| 62 | TBK1 | na | 4986 | 0.079 | 0.3292 | No |
| 63 | LSM14A | na | 5048 | 0.074 | 0.3251 | No |
| 64 | PPM1B | na | 5075 | 0.072 | 0.3239 | No |
| 65 | BNIP3L | na | 5255 | 0.058 | 0.3096 | No |
| 66 | TICAM1 | na | 5280 | 0.056 | 0.3083 | No |
| 67 | TRAF3 | na | 5463 | 0.040 | 0.2936 | No |
| 68 | SERINC3 | na | 5528 | 0.034 | 0.2886 | No |
| 69 | BCL2L1 | na | 5617 | 0.024 | 0.2816 | No |
| 70 | ATG7 | na | 5762 | 0.012 | 0.2696 | No |
| 71 | ILF3 | na | 5791 | 0.010 | 0.2674 | No |
| 72 | SEC14L1 | na | 5880 | 0.003 | 0.2600 | No |
| 73 | PUM1 | na | 5905 | 0.001 | 0.2580 | No |
| 74 | RELA | na | 5979 | -0.005 | 0.2519 | No |
| 75 | DNAJC3 | na | 5982 | -0.005 | 0.2518 | No |
| 76 | POLR3B | na | 5986 | -0.005 | 0.2516 | No |
| 77 | UNC93B1 | na | 6108 | -0.015 | 0.2417 | No |
| 78 | TRIM44 | na | 6161 | -0.019 | 0.2375 | No |
| 79 | ADARB1 | na | 6206 | -0.023 | 0.2342 | No |
| 80 | SIN3A | na | 6385 | -0.037 | 0.2197 | No |
| 81 | MAVS | na | 6400 | -0.038 | 0.2190 | No |
| 82 | HTRA1 | na | 6418 | -0.039 | 0.2181 | No |
| 83 | NCBP3 | na | 6543 | -0.050 | 0.2084 | No |
| 84 | ABCE1 | na | 6592 | -0.054 | 0.2050 | No |
| 85 | IL2RA | na | 6593 | -0.054 | 0.2058 | No |
| 86 | MICA | na | 6922 | -0.081 | 0.1793 | No |
| 87 | PUM2 | na | 6942 | -0.084 | 0.1788 | No |
| 88 | HSP90AA1 | na | 7065 | -0.095 | 0.1698 | No |
| 89 | IRF2 | na | 7126 | -0.100 | 0.1662 | No |
| 90 | USP15 | na | 7256 | -0.113 | 0.1568 | No |
| 91 | DMBT1 | na | 7422 | -0.129 | 0.1447 | No |
| 92 | POLR3H | na | 7428 | -0.129 | 0.1460 | No |
| 93 | TNFAIP3 | na | 7642 | -0.147 | 0.1301 | No |
| 94 | ZC3H12A | na | 7694 | -0.152 | 0.1279 | No |
| 95 | ZMPSTE24 | na | 7701 | -0.153 | 0.1295 | No |
| 96 | DHX36 | na | 7816 | -0.163 | 0.1221 | No |
| 97 | SETD2 | na | 7829 | -0.164 | 0.1233 | No |
| 98 | FGL2 | na | 7929 | -0.172 | 0.1173 | No |
| 99 | PLSCR1 | na | 7952 | -0.174 | 0.1178 | No |
| 100 | C1QBP | na | 8129 | -0.191 | 0.1056 | No |
| 101 | GPATCH3 | na | 8233 | -0.202 | 0.0997 | No |
| 102 | UNC13D | na | 8298 | -0.208 | 0.0971 | No |
| 103 | EXOSC5 | na | 8301 | -0.208 | 0.0998 | No |
| 104 | PHB | na | 8376 | -0.216 | 0.0965 | No |
| 105 | DDX17 | na | 8526 | -0.232 | 0.0871 | No |
| 106 | PTPRC | na | 8879 | -0.271 | 0.0612 | No |
| 107 | AIMP1 | na | 9043 | -0.288 | 0.0514 | No |
| 108 | POLR3F | na | 9085 | -0.294 | 0.0519 | No |
| 109 | RNF26 | na | 9150 | -0.302 | 0.0506 | No |
| 110 | TTC4 | na | 9255 | -0.316 | 0.0462 | No |
| 111 | G3BP1 | na | 9360 | -0.330 | 0.0419 | No |
| 112 | ABCF3 | na | 9461 | -0.343 | 0.0382 | No |
| 113 | AKAP1 | na | 9508 | -0.350 | 0.0390 | No |
| 114 | MAP3K14 | na | 9607 | -0.363 | 0.0357 | No |
| 115 | ELMOD2 | na | 9626 | -0.366 | 0.0392 | No |
| 116 | AGBL5 | na | 10353 | -0.470 | -0.0155 | No |
| 117 | CARD9 | na | 10374 | -0.475 | -0.0108 | No |
| 118 | SERINC5 | na | 10417 | -0.482 | -0.0077 | No |
| 119 | PHB2 | na | 10512 | -0.499 | -0.0088 | No |
| 120 | HYAL2 | na | 10514 | -0.500 | -0.0021 | No |
| 121 | NPLOC4 | na | 10561 | -0.510 | 0.0009 | No |
| 122 | ACOD1 | na | 10577 | -0.514 | 0.0067 | No |
| 123 | BNIP3 | na | 10582 | -0.516 | 0.0134 | No |
| 124 | POLR3E | na | 10590 | -0.517 | 0.0198 | No |
| 125 | BCL2 | na | 10609 | -0.520 | 0.0254 | No |
| 126 | TLR7 | na | 10620 | -0.523 | 0.0317 | No |
| 127 | CRCP | na | 10701 | -0.539 | 0.0323 | No |
| 128 | CD40 | na | 10769 | -0.552 | 0.0341 | No |
| 129 | PDE12 | na | 10911 | -0.584 | 0.0302 | No |
| 130 | DDX21 | na | 10923 | -0.586 | 0.0373 | No |
| 131 | MUL1 | na | 10974 | -0.600 | 0.0412 | No |
| 132 | AICDA | na | 11146 | -0.648 | 0.0357 | No |
| 133 | NT5C3A | na | 11149 | -0.650 | 0.0443 | No |
| 134 | IRF5 | na | 11267 | -0.688 | 0.0439 | No |
| 135 | MLKL | na | 11918 | -1.368 | 0.0077 | No |
Table: GSEA details [plain text format]

  

Fig 2: GOBP\_DEFENSE\_RESPONSE\_TO\_VIRUS      
 Blue-Pink O' Gram in the Space of the Analyzed GeneSet

  

Fig 3: GOBP\_DEFENSE\_RESPONSE\_TO\_VIRUS: Random ES distribution      
 Gene set null distribution of ES for **GOBP\_DEFENSE\_RESPONSE\_TO\_VIRUS**

  
