# Supplementary material for: Time-series transcriptome analysis identified differentially expressed genes in broiler chicken infected with mixed Eimeria species
Source: Front Genet. 2022 Aug 8;13:886781. doi: 10.3389/fgene.2022.886781 (PMC9393255; doi:10.3389/fgene.2022.886781)
Supplement: Supplementary file 2 [file DataSheet1.ZIP › 4dpi_GO.Gsea.1625071243202/GOBP_DIGESTION.html]

Details for gene set GOBP\_DIGESTION[GSEA]

|  || Dataset | TMM\_4dpi\_gct\_format\_4dpi\_gct\_format.Class\_4dpi.cls #PC\_versus\_NC.Class\_4dpi.cls #PC\_versus\_NC\_repos |
| Phenotype | Class\_4dpi.cls#PC\_versus\_NC\_repos |
| Upregulated in class | 1 |
| GeneSet | GOBP\_DIGESTION |
| Enrichment Score (ES) | 0.57493246 |
| Normalized Enrichment Score (NES) | 2.1167653 |
| Nominal p-value | 0.0 |
| FDR q-value | 0.0020887316 |
| FWER p-Value | 0.037 |
Table: GSEA Results Summary

  

Fig 1: Enrichment plot: GOBP\_DIGESTION      
 Profile of the Running ES Score & Positions of GeneSet Members on the Rank Ordered List

  

| SYMBOL | TITLE | RANK IN GENE LIST | RANK METRIC SCORE | RUNNING ES | CORE ENRICHMENT || 1 | ABCG8 | na | 2 | 3.384 | 0.0711 | Yes |
| 2 | ABCG5 | na | 41 | 2.059 | 0.1113 | Yes |
| 3 | SNX10 | na | 87 | 1.706 | 0.1435 | Yes |
| 4 | SST | na | 88 | 1.694 | 0.1792 | Yes |
| 5 | VIL1 | na | 96 | 1.665 | 0.2137 | Yes |
| 6 | SCT | na | 132 | 1.515 | 0.2427 | Yes |
| 7 | CCK | na | 155 | 1.450 | 0.2714 | Yes |
| 8 | LDLR | na | 159 | 1.437 | 0.3014 | Yes |
| 9 | PLS1 | na | 173 | 1.404 | 0.3299 | Yes |
| 10 | UCN3 | na | 210 | 1.337 | 0.3550 | Yes |
| 11 | SLC26A6 | na | 312 | 1.158 | 0.3710 | Yes |
| 12 | CAPN8 | na | 356 | 1.118 | 0.3909 | Yes |
| 13 | SGK1 | na | 485 | 0.979 | 0.4008 | Yes |
| 14 | CAPN9 | na | 560 | 0.906 | 0.4137 | Yes |
| 15 | APOA1 | na | 589 | 0.889 | 0.4301 | Yes |
| 16 | TJP2 | na | 629 | 0.860 | 0.4450 | Yes |
| 17 | FABP2 | na | 653 | 0.848 | 0.4609 | Yes |
| 18 | APOA4 | na | 733 | 0.797 | 0.4711 | Yes |
| 19 | EZR | na | 800 | 0.761 | 0.4816 | Yes |
| 20 | MOGAT2 | na | 861 | 0.731 | 0.4920 | Yes |
| 21 | NPR3 | na | 960 | 0.686 | 0.4983 | Yes |
| 22 | SLC2A5 | na | 1056 | 0.653 | 0.5041 | Yes |
| 23 | LIMA1 | na | 1076 | 0.647 | 0.5161 | Yes |
| 24 | UGCG | na | 1236 | 0.593 | 0.5153 | Yes |
| 25 | MGAM | na | 1261 | 0.586 | 0.5256 | Yes |
| 26 | SI | na | 1279 | 0.582 | 0.5365 | Yes |
| 27 | CD36 | na | 1326 | 0.568 | 0.5446 | Yes |
| 28 | SLC5A1 | na | 1360 | 0.558 | 0.5536 | Yes |
| 29 | GUCY2C | na | 1413 | 0.544 | 0.5607 | Yes |
| 30 | STK39 | na | 1416 | 0.543 | 0.5720 | Yes |
| 31 | HIP1R | na | 1604 | 0.494 | 0.5667 | Yes |
| 32 | NMU | na | 1771 | 0.462 | 0.5626 | Yes |
| 33 | SLC46A1 | na | 1786 | 0.458 | 0.5710 | Yes |
| 34 | LPCAT3 | na | 1853 | 0.447 | 0.5749 | Yes |
| 35 | FGF10 | na | 2274 | 0.378 | 0.5477 | No |
| 36 | COPA | na | 2349 | 0.369 | 0.5493 | No |
| 37 | FABP1 | na | 2389 | 0.363 | 0.5537 | No |
| 38 | HRH2 | na | 3135 | 0.264 | 0.4969 | No |
| 39 | NR1H3 | na | 3213 | 0.255 | 0.4958 | No |
| 40 | ADRA2A | na | 4508 | 0.123 | 0.3901 | No |
| 41 | AQP1 | na | 5079 | 0.072 | 0.3439 | No |
| 42 | PBLD | na | 5379 | 0.047 | 0.3198 | No |
| 43 | MUC4 | na | 5527 | 0.034 | 0.3082 | No |
| 44 | SCARB1 | na | 5664 | 0.020 | 0.2973 | No |
| 45 | STRAP | na | 5665 | 0.020 | 0.2977 | No |
| 46 | ACO1 | na | 5837 | 0.006 | 0.2835 | No |
| 47 | SOX9 | na | 5991 | -0.005 | 0.2708 | No |
| 48 | TLR4 | na | 6033 | -0.009 | 0.2676 | No |
| 49 | SLC22A5 | na | 6333 | -0.032 | 0.2432 | No |
| 50 | CHIA | na | 7002 | -0.090 | 0.1892 | No |
| 51 | MUC13 | na | 7127 | -0.101 | 0.1809 | No |
| 52 | TFF3 | na | 7318 | -0.119 | 0.1675 | No |
| 53 | AQP5 | na | 7690 | -0.152 | 0.1396 | No |
| 54 | CHRM3 | na | 8085 | -0.187 | 0.1106 | No |
| 55 | LCT | na | 8218 | -0.200 | 0.1038 | No |
| 56 | ZNF830 | na | 8247 | -0.203 | 0.1057 | No |
| 57 | IREB2 | na | 8275 | -0.206 | 0.1078 | No |
| 58 | EPB41 | na | 8952 | -0.279 | 0.0571 | No |
| 59 | CYP8B1 | na | 9820 | -0.389 | -0.0073 | No |
| 60 | NEUROD1 | na | 9870 | -0.394 | -0.0031 | No |
| 61 | OXTR | na | 10498 | -0.498 | -0.0451 | No |
| 62 | CYP39A1 | na | 10542 | -0.507 | -0.0381 | No |
| 63 | PPARGC1A | na | 11134 | -0.645 | -0.0740 | No |
| 64 | AKR1D1 | na | 11209 | -0.667 | -0.0661 | No |
| 65 | GUCA2B | na | 11353 | -0.724 | -0.0628 | No |
| 66 | PTGER3 | na | 11476 | -0.791 | -0.0564 | No |
| 67 | KCNQ1 | na | 11999 | -2.392 | -0.0497 | No |
| 68 | TAC1 | na | 12000 | -2.398 | 0.0008 | No |
Table: GSEA details [plain text format]

  

Fig 2: GOBP\_DIGESTION      
 Blue-Pink O' Gram in the Space of the Analyzed GeneSet

  

Fig 3: GOBP\_DIGESTION: Random ES distribution      
 Gene set null distribution of ES for **GOBP\_DIGESTION**

  
