# Supplementary material for: Time-series transcriptome analysis identified differentially expressed genes in broiler chicken infected with mixed Eimeria species
Source: Front Genet. 2022 Aug 8;13:886781. doi: 10.3389/fgene.2022.886781 (PMC9393255; doi:10.3389/fgene.2022.886781)
Supplement: Supplementary file 2 [file DataSheet1.ZIP › 4dpi_GO.Gsea.1625071243202/GOBP_ESTABLISHMENT_OF_PROTEIN_LOCALIZATION_TO_ENDOPLASMIC_RETICULUM.html]

Details for gene set GOBP\_ESTABLISHMENT\_OF\_PROTEIN\_LOCALIZATION\_TO\_ENDOPLASMIC\_RETICULUM[GSEA]

|  || Dataset | TMM\_4dpi\_gct\_format\_4dpi\_gct\_format.Class\_4dpi.cls #PC\_versus\_NC.Class\_4dpi.cls #PC\_versus\_NC\_repos |
| Phenotype | Class\_4dpi.cls#PC\_versus\_NC\_repos |
| Upregulated in class | 0 |
| GeneSet | GOBP\_ESTABLISHMENT\_OF\_PROTEIN\_LOCALIZATION\_TO\_ENDOPLASMIC\_RETICULUM |
| Enrichment Score (ES) | -0.63892967 |
| Normalized Enrichment Score (NES) | -2.6901176 |
| Nominal p-value | 0.0 |
| FDR q-value | 0.0 |
| FWER p-Value | 0.0 |
Table: GSEA Results Summary

  

Fig 1: Enrichment plot: GOBP\_ESTABLISHMENT\_OF\_PROTEIN\_LOCALIZATION\_TO\_ENDOPLASMIC\_RETICULUM      
 Profile of the Running ES Score & Positions of GeneSet Members on the Rank Ordered List

  

| SYMBOL | TITLE | RANK IN GENE LIST | RANK METRIC SCORE | RUNNING ES | CORE ENRICHMENT || 1 | MAN1A1 | na | 62 | 1.954 | 0.0295 | No |
| 2 | RN7SL1 | na | 179 | 1.390 | 0.0445 | No |
| 3 | SEC61B | na | 866 | 0.729 | -0.0002 | No |
| 4 | SEC61G | na | 1475 | 0.525 | -0.0419 | No |
| 5 | RAB3GAP2 | na | 1753 | 0.465 | -0.0569 | No |
| 6 | HSPA5 | na | 2167 | 0.394 | -0.0846 | No |
| 7 | SEC63 | na | 2308 | 0.375 | -0.0897 | No |
| 8 | RAB10 | na | 2578 | 0.338 | -0.1062 | No |
| 9 | CHMP4B | na | 2783 | 0.311 | -0.1179 | No |
| 10 | SGTA | na | 2968 | 0.286 | -0.1282 | No |
| 11 | SGTB | na | 3260 | 0.248 | -0.1482 | No |
| 12 | SPCS1 | na | 3477 | 0.224 | -0.1624 | No |
| 13 | SEC61A1 | na | 3573 | 0.210 | -0.1666 | No |
| 14 | SRP19 | na | 3621 | 0.205 | -0.1669 | No |
| 15 | SRP54 | na | 3757 | 0.192 | -0.1749 | No |
| 16 | TRAM1L1 | na | 3856 | 0.182 | -0.1799 | No |
| 17 | SRPRB | na | 3949 | 0.172 | -0.1845 | No |
| 18 | RAB3GAP1 | na | 4775 | 0.099 | -0.2520 | No |
| 19 | SRPRA | na | 4934 | 0.085 | -0.2638 | No |
| 20 | SRP68 | na | 5003 | 0.078 | -0.2681 | No |
| 21 | SEC61A2 | na | 5014 | 0.077 | -0.2675 | No |
| 22 | TRAM2 | na | 5068 | 0.073 | -0.2707 | No |
| 23 | SSR3 | na | 5621 | 0.024 | -0.3166 | No |
| 24 | SRP14 | na | 6579 | -0.052 | -0.3960 | No |
| 25 | SRP72 | na | 6615 | -0.056 | -0.3980 | No |
| 26 | SEC62 | na | 7190 | -0.107 | -0.4442 | No |
| 27 | SPCS3 | na | 8059 | -0.185 | -0.5138 | No |
| 28 | RPS23 | na | 8269 | -0.206 | -0.5277 | No |
| 29 | RPL17 | na | 8583 | -0.239 | -0.5497 | No |
| 30 | EDEM1 | na | 8721 | -0.253 | -0.5567 | No |
| 31 | RPS6 | na | 8904 | -0.274 | -0.5672 | No |
| 32 | RPL36 | na | 9363 | -0.330 | -0.5997 | No |
| 33 | UBA52 | na | 9532 | -0.353 | -0.6076 | No |
| 34 | RPL38 | na | 9661 | -0.369 | -0.6118 | No |
| 35 | RPS24 | na | 9689 | -0.372 | -0.6074 | No |
| 36 | SRP9 | na | 9772 | -0.382 | -0.6075 | No |
| 37 | RPLP2 | na | 10048 | -0.421 | -0.6231 | No |
| 38 | RPL37 | na | 10057 | -0.423 | -0.6163 | No |
| 39 | RPS8 | na | 10245 | -0.452 | -0.6240 | No |
| 40 | RPL27 | na | 10333 | -0.467 | -0.6230 | No |
| 41 | RPS28 | na | 10524 | -0.504 | -0.6300 | Yes |
| 42 | RPL30 | na | 10537 | -0.506 | -0.6220 | Yes |
| 43 | RPL22 | na | 10554 | -0.508 | -0.6143 | Yes |
| 44 | RPL36A | na | 10605 | -0.520 | -0.6093 | Yes |
| 45 | RPL14 | na | 10662 | -0.531 | -0.6046 | Yes |
| 46 | RPL29 | na | 10742 | -0.548 | -0.6015 | Yes |
| 47 | RPL37A | na | 10838 | -0.569 | -0.5993 | Yes |
| 48 | RPS19 | na | 10840 | -0.569 | -0.5893 | Yes |
| 49 | RPS12 | na | 10875 | -0.576 | -0.5819 | Yes |
| 50 | RPL24 | na | 10877 | -0.576 | -0.5718 | Yes |
| 51 | RPL34 | na | 10933 | -0.588 | -0.5660 | Yes |
| 52 | RPL23 | na | 10981 | -0.602 | -0.5592 | Yes |
| 53 | RPL35A | na | 10982 | -0.602 | -0.5485 | Yes |
| 54 | RPS25 | na | 11017 | -0.613 | -0.5405 | Yes |
| 55 | RPS7 | na | 11068 | -0.627 | -0.5335 | Yes |
| 56 | RPL23A | na | 11076 | -0.629 | -0.5230 | Yes |
| 57 | RPL5 | na | 11121 | -0.643 | -0.5153 | Yes |
| 58 | RPS16 | na | 11138 | -0.645 | -0.5051 | Yes |
| 59 | RPL6 | na | 11150 | -0.650 | -0.4945 | Yes |
| 60 | RPL11 | na | 11195 | -0.662 | -0.4865 | Yes |
| 61 | RPS15A | na | 11196 | -0.662 | -0.4747 | Yes |
| 62 | RPLP1 | na | 11249 | -0.682 | -0.4669 | Yes |
| 63 | RPS26 | na | 11254 | -0.686 | -0.4551 | Yes |
| 64 | RPL35 | na | 11280 | -0.696 | -0.4448 | Yes |
| 65 | RPL31 | na | 11306 | -0.706 | -0.4344 | Yes |
| 66 | RPS21 | na | 11313 | -0.709 | -0.4223 | Yes |
| 67 | RPS3A | na | 11328 | -0.713 | -0.4108 | Yes |
| 68 | RPL21 | na | 11330 | -0.714 | -0.3982 | Yes |
| 69 | RPS10 | na | 11344 | -0.720 | -0.3865 | Yes |
| 70 | RPL32 | na | 11355 | -0.725 | -0.3745 | Yes |
| 71 | RPS11 | na | 11371 | -0.735 | -0.3627 | Yes |
| 72 | RPL12 | na | 11398 | -0.748 | -0.3516 | Yes |
| 73 | RPL7A | na | 11426 | -0.763 | -0.3403 | Yes |
| 74 | RPS15 | na | 11434 | -0.767 | -0.3273 | Yes |
| 75 | RPS27A | na | 11435 | -0.767 | -0.3137 | Yes |
| 76 | RPL15 | na | 11440 | -0.769 | -0.3003 | Yes |
| 77 | RPS29 | na | 11444 | -0.771 | -0.2869 | Yes |
| 78 | RPL18A | na | 11474 | -0.789 | -0.2753 | Yes |
| 79 | RPS14 | na | 11478 | -0.792 | -0.2615 | Yes |
| 80 | RPL7 | na | 11484 | -0.796 | -0.2478 | Yes |
| 81 | RPLP0 | na | 11488 | -0.800 | -0.2339 | Yes |
| 82 | RPL27A | na | 11507 | -0.812 | -0.2209 | Yes |
| 83 | RPL9 | na | 11521 | -0.819 | -0.2075 | Yes |
| 84 | HERPUD1 | na | 11531 | -0.824 | -0.1936 | Yes |
| 85 | RPS2 | na | 11562 | -0.846 | -0.1811 | Yes |
| 86 | RPL13 | na | 11563 | -0.847 | -0.1661 | Yes |
| 87 | RPS20 | na | 11594 | -0.868 | -0.1532 | Yes |
| 88 | RPS27 | na | 11600 | -0.875 | -0.1380 | Yes |
| 89 | RPL19 | na | 11611 | -0.882 | -0.1232 | Yes |
| 90 | RPS13 | na | 11633 | -0.897 | -0.1091 | Yes |
| 91 | RPS3 | na | 11641 | -0.903 | -0.0936 | Yes |
| 92 | RPS17 | na | 11652 | -0.916 | -0.0782 | Yes |
| 93 | RPL10A | na | 11658 | -0.921 | -0.0623 | Yes |
| 94 | RYR2 | na | 11674 | -0.941 | -0.0468 | Yes |
| 95 | RPL4 | na | 11715 | -0.983 | -0.0327 | Yes |
| 96 | RPL8 | na | 11772 | -1.039 | -0.0189 | Yes |
| 97 | RPS4Y1 | na | 11774 | -1.043 | -0.0005 | Yes |
| 98 | RPL3 | na | 11817 | -1.140 | 0.0162 | Yes |
Table: GSEA details [plain text format]

  

Fig 2: GOBP\_ESTABLISHMENT\_OF\_PROTEIN\_LOCALIZATION\_TO\_ENDOPLASMIC\_RETICULUM      
 Blue-Pink O' Gram in the Space of the Analyzed GeneSet

  

Fig 3: GOBP\_ESTABLISHMENT\_OF\_PROTEIN\_LOCALIZATION\_TO\_ENDOPLASMIC\_RETICULUM: Random ES distribution      
 Gene set null distribution of ES for **GOBP\_ESTABLISHMENT\_OF\_PROTEIN\_LOCALIZATION\_TO\_ENDOPLASMIC\_RETICULUM**

  
