# Supplementary material for: Time-series transcriptome analysis identified differentially expressed genes in broiler chicken infected with mixed Eimeria species
Source: Front Genet. 2022 Aug 8;13:886781. doi: 10.3389/fgene.2022.886781 (PMC9393255; doi:10.3389/fgene.2022.886781)
Supplement: Supplementary file 2 [file DataSheet1.ZIP › 4dpi_GO.Gsea.1625071243202/GOBP_INTERFERON_GAMMA_MEDIATED_SIGNALING_PATHWAY.html]

Details for gene set GOBP\_INTERFERON\_GAMMA\_MEDIATED\_SIGNALING\_PATHWAY[GSEA]

|  || Dataset | TMM\_4dpi\_gct\_format\_4dpi\_gct\_format.Class\_4dpi.cls #PC\_versus\_NC.Class\_4dpi.cls #PC\_versus\_NC\_repos |
| Phenotype | Class\_4dpi.cls#PC\_versus\_NC\_repos |
| Upregulated in class | 1 |
| GeneSet | GOBP\_INTERFERON\_GAMMA\_MEDIATED\_SIGNALING\_PATHWAY |
| Enrichment Score (ES) | 0.64356333 |
| Normalized Enrichment Score (NES) | 2.1776764 |
| Nominal p-value | 0.0 |
| FDR q-value | 6.352922E-4 |
| FWER p-Value | 0.0072 |
Table: GSEA Results Summary

  

Fig 1: Enrichment plot: GOBP\_INTERFERON\_GAMMA\_MEDIATED\_SIGNALING\_PATHWAY      
 Profile of the Running ES Score & Positions of GeneSet Members on the Rank Ordered List

  

| SYMBOL | TITLE | RANK IN GENE LIST | RANK METRIC SCORE | RUNNING ES | CORE ENRICHMENT || 1 | GBP1 | na | 34 | 2.116 | 0.0605 | Yes |
| 2 | OASL | na | 52 | 2.001 | 0.1189 | Yes |
| 3 | PARP9 | na | 53 | 1.996 | 0.1787 | Yes |
| 4 | NMI | na | 66 | 1.838 | 0.2327 | Yes |
| 5 | STAT1 | na | 78 | 1.743 | 0.2840 | Yes |
| 6 | IRF7 | na | 85 | 1.706 | 0.3345 | Yes |
| 7 | IRF9 | na | 103 | 1.617 | 0.3815 | Yes |
| 8 | IRF1 | na | 114 | 1.568 | 0.4276 | Yes |
| 9 | NLRC5 | na | 162 | 1.431 | 0.4665 | Yes |
| 10 | PARP14 | na | 191 | 1.367 | 0.5051 | Yes |
| 11 | TRIM25 | na | 408 | 1.056 | 0.5187 | Yes |
| 12 | B2M | na | 509 | 0.954 | 0.5389 | Yes |
| 13 | PIAS1 | na | 583 | 0.893 | 0.5595 | Yes |
| 14 | PRKCD | na | 605 | 0.875 | 0.5839 | Yes |
| 15 | SOCS1 | na | 672 | 0.839 | 0.6035 | Yes |
| 16 | SOCS3 | na | 686 | 0.829 | 0.6272 | Yes |
| 17 | IFNGR2 | na | 965 | 0.685 | 0.6245 | Yes |
| 18 | IRF8 | na | 981 | 0.678 | 0.6436 | Yes |
| 19 | JAK2 | na | 1856 | 0.446 | 0.5839 | No |
| 20 | CAMK2G | na | 2166 | 0.394 | 0.5698 | No |
| 21 | CIITA | na | 2348 | 0.369 | 0.5658 | No |
| 22 | TRIM8 | na | 2825 | 0.305 | 0.5351 | No |
| 23 | NR1H3 | na | 3213 | 0.255 | 0.5104 | No |
| 24 | MID1 | na | 3753 | 0.193 | 0.4711 | No |
| 25 | CAMK2B | na | 3829 | 0.184 | 0.4704 | No |
| 26 | CDC37 | na | 5605 | 0.025 | 0.3228 | No |
| 27 | IFNGR1 | na | 5858 | 0.005 | 0.3019 | No |
| 28 | VCAM1 | na | 6006 | -0.007 | 0.2898 | No |
| 29 | IRF2 | na | 7126 | -0.100 | 0.1993 | No |
| 30 | JAK1 | na | 7146 | -0.103 | 0.2008 | No |
| 31 | PPARG | na | 7261 | -0.114 | 0.1947 | No |
| 32 | CD44 | na | 7341 | -0.122 | 0.1917 | No |
| 33 | SUMO1 | na | 7638 | -0.147 | 0.1714 | No |
| 34 | PTAFR | na | 8527 | -0.232 | 0.1041 | No |
| 35 | HSP90AB1 | na | 8645 | -0.244 | 0.1016 | No |
| 36 | PTPN2 | na | 8813 | -0.263 | 0.0956 | No |
| 37 | CAMK2D | na | 8964 | -0.280 | 0.0914 | No |
| 38 | MED1 | na | 9560 | -0.358 | 0.0524 | No |
| 39 | IRF6 | na | 10011 | -0.416 | 0.0272 | No |
| 40 | NCAM1 | na | 10023 | -0.418 | 0.0388 | No |
| 41 | IRF4 | na | 10673 | -0.534 | 0.0006 | No |
| 42 | HCK | na | 11136 | -0.645 | -0.0188 | No |
| 43 | IRF5 | na | 11267 | -0.688 | -0.0090 | No |
| 44 | TRIM62 | na | 11497 | -0.806 | -0.0040 | No |
| 45 | IFI30 | na | 11950 | -1.564 | 0.0050 | No |
Table: GSEA details [plain text format]

  

Fig 2: GOBP\_INTERFERON\_GAMMA\_MEDIATED\_SIGNALING\_PATHWAY      
 Blue-Pink O' Gram in the Space of the Analyzed GeneSet

  

Fig 3: GOBP\_INTERFERON\_GAMMA\_MEDIATED\_SIGNALING\_PATHWAY: Random ES distribution      
 Gene set null distribution of ES for **GOBP\_INTERFERON\_GAMMA\_MEDIATED\_SIGNALING\_PATHWAY**

  
