# Supplementary material for: Time-series transcriptome analysis identified differentially expressed genes in broiler chicken infected with mixed Eimeria species
Source: Front Genet. 2022 Aug 8;13:886781. doi: 10.3389/fgene.2022.886781 (PMC9393255; doi:10.3389/fgene.2022.886781)
Supplement: Supplementary file 2 [file DataSheet1.ZIP › 4dpi_GO.Gsea.1625071243202/GOBP_KERATINIZATION.html]

Details for gene set GOBP\_KERATINIZATION[GSEA]

|  || Dataset | TMM\_4dpi\_gct\_format\_4dpi\_gct\_format.Class\_4dpi.cls #PC\_versus\_NC.Class\_4dpi.cls #PC\_versus\_NC\_repos |
| Phenotype | Class\_4dpi.cls#PC\_versus\_NC\_repos |
| Upregulated in class | 1 |
| GeneSet | GOBP\_KERATINIZATION |
| Enrichment Score (ES) | 0.74121624 |
| Normalized Enrichment Score (NES) | 2.2670255 |
| Nominal p-value | 0.0 |
| FDR q-value | 2.1119595E-4 |
| FWER p-Value | 0.001 |
Table: GSEA Results Summary

  

Fig 1: Enrichment plot: GOBP\_KERATINIZATION      
 Profile of the Running ES Score & Positions of GeneSet Members on the Rank Ordered List

  

| SYMBOL | TITLE | RANK IN GENE LIST | RANK METRIC SCORE | RUNNING ES | CORE ENRICHMENT || 1 | PKP2 | na | 7 | 2.810 | 0.1194 | Yes |
| 2 | DSC1 | na | 8 | 2.652 | 0.2326 | Yes |
| 3 | KRT7 | na | 24 | 2.205 | 0.3255 | Yes |
| 4 | KRT40 | na | 38 | 2.072 | 0.4129 | Yes |
| 5 | KRT80 | na | 292 | 1.183 | 0.4423 | Yes |
| 6 | DSP | na | 401 | 1.061 | 0.4786 | Yes |
| 7 | KRT18 | na | 477 | 0.988 | 0.5145 | Yes |
| 8 | KAZN | na | 484 | 0.980 | 0.5559 | Yes |
| 9 | CASP14 | na | 642 | 0.854 | 0.5792 | Yes |
| 10 | EVPL | na | 743 | 0.791 | 0.6046 | Yes |
| 11 | CSTA | na | 779 | 0.769 | 0.6345 | Yes |
| 12 | KRT8 | na | 820 | 0.752 | 0.6633 | Yes |
| 13 | KRT10 | na | 930 | 0.698 | 0.6840 | Yes |
| 14 | DSG2 | na | 985 | 0.678 | 0.7085 | Yes |
| 15 | KRT23 | na | 1173 | 0.612 | 0.7190 | Yes |
| 16 | PPL | na | 1389 | 0.551 | 0.7245 | Yes |
| 17 | PKP4 | na | 1583 | 0.500 | 0.7298 | Yes |
| 18 | CAPN1 | na | 1747 | 0.466 | 0.7360 | Yes |
| 19 | ST14 | na | 1910 | 0.438 | 0.7412 | Yes |
| 20 | KRT24 | na | 2352 | 0.368 | 0.7201 | No |
| 21 | PCSK6 | na | 2412 | 0.360 | 0.7306 | No |
| 22 | CERS3 | na | 3332 | 0.239 | 0.6641 | No |
| 23 | JUP | na | 3549 | 0.213 | 0.6552 | No |
| 24 | PPHLN1 | na | 5846 | 0.006 | 0.4638 | No |
| 25 | TMEM79 | na | 7320 | -0.120 | 0.3460 | No |
| 26 | SPINK5 | na | 9063 | -0.291 | 0.2130 | No |
| 27 | FURIN | na | 9299 | -0.321 | 0.2071 | No |
| 28 | CDH3 | na | 10206 | -0.445 | 0.1505 | No |
Table: GSEA details [plain text format]

  

Fig 2: GOBP\_KERATINIZATION      
 Blue-Pink O' Gram in the Space of the Analyzed GeneSet

  

Fig 3: GOBP\_KERATINIZATION: Random ES distribution      
 Gene set null distribution of ES for **GOBP\_KERATINIZATION**

  
