# Supplementary material for: Time-series transcriptome analysis identified differentially expressed genes in broiler chicken infected with mixed Eimeria species
Source: Front Genet. 2022 Aug 8;13:886781. doi: 10.3389/fgene.2022.886781 (PMC9393255; doi:10.3389/fgene.2022.886781)
Supplement: Supplementary file 2 [file DataSheet1.ZIP › 4dpi_GO.Gsea.1625071243202/GOBP_KERATINOCYTE_DIFFERENTIATION.html]

Details for gene set GOBP\_KERATINOCYTE\_DIFFERENTIATION[GSEA]

|  || Dataset | TMM\_4dpi\_gct\_format\_4dpi\_gct\_format.Class\_4dpi.cls #PC\_versus\_NC.Class\_4dpi.cls #PC\_versus\_NC\_repos |
| Phenotype | Class\_4dpi.cls#PC\_versus\_NC\_repos |
| Upregulated in class | 1 |
| GeneSet | GOBP\_KERATINOCYTE\_DIFFERENTIATION |
| Enrichment Score (ES) | 0.5697976 |
| Normalized Enrichment Score (NES) | 2.1323853 |
| Nominal p-value | 0.0 |
| FDR q-value | 0.0016221766 |
| FWER p-Value | 0.0258 |
Table: GSEA Results Summary

  

Fig 1: Enrichment plot: GOBP\_KERATINOCYTE\_DIFFERENTIATION      
 Profile of the Running ES Score & Positions of GeneSet Members on the Rank Ordered List

  

| SYMBOL | TITLE | RANK IN GENE LIST | RANK METRIC SCORE | RUNNING ES | CORE ENRICHMENT || 1 | PKP2 | na | 7 | 2.810 | 0.0697 | Yes |
| 2 | DSC1 | na | 8 | 2.652 | 0.1360 | Yes |
| 3 | KRT7 | na | 24 | 2.205 | 0.1898 | Yes |
| 4 | KRT40 | na | 38 | 2.072 | 0.2406 | Yes |
| 5 | FOSL2 | na | 100 | 1.634 | 0.2763 | Yes |
| 6 | KRT80 | na | 292 | 1.183 | 0.2899 | Yes |
| 7 | DSP | na | 401 | 1.061 | 0.3074 | Yes |
| 8 | KRT18 | na | 477 | 0.988 | 0.3258 | Yes |
| 9 | KAZN | na | 484 | 0.980 | 0.3498 | Yes |
| 10 | BCL11B | na | 604 | 0.876 | 0.3617 | Yes |
| 11 | CASP14 | na | 642 | 0.854 | 0.3799 | Yes |
| 12 | EVPL | na | 743 | 0.791 | 0.3913 | Yes |
| 13 | CSTA | na | 779 | 0.769 | 0.4076 | Yes |
| 14 | KRT8 | na | 820 | 0.752 | 0.4231 | Yes |
| 15 | KRT10 | na | 930 | 0.698 | 0.4314 | Yes |
| 16 | DSG2 | na | 985 | 0.678 | 0.4438 | Yes |
| 17 | ANXA1 | na | 1060 | 0.651 | 0.4539 | Yes |
| 18 | GRHL1 | na | 1069 | 0.649 | 0.4694 | Yes |
| 19 | CLIC4 | na | 1121 | 0.631 | 0.4810 | Yes |
| 20 | KRT23 | na | 1173 | 0.612 | 0.4920 | Yes |
| 21 | UGCG | na | 1236 | 0.593 | 0.5016 | Yes |
| 22 | AQP3 | na | 1320 | 0.570 | 0.5089 | Yes |
| 23 | CASP3 | na | 1333 | 0.565 | 0.5220 | Yes |
| 24 | PPL | na | 1389 | 0.551 | 0.5312 | Yes |
| 25 | RBPJ | na | 1394 | 0.549 | 0.5446 | Yes |
| 26 | PKP4 | na | 1583 | 0.500 | 0.5413 | Yes |
| 27 | NCOA3 | na | 1705 | 0.473 | 0.5430 | Yes |
| 28 | CAPN1 | na | 1747 | 0.466 | 0.5512 | Yes |
| 29 | ACER1 | na | 1866 | 0.445 | 0.5525 | Yes |
| 30 | ST14 | na | 1910 | 0.438 | 0.5598 | Yes |
| 31 | ROCK2 | na | 1922 | 0.436 | 0.5698 | Yes |
| 32 | CD109 | na | 2317 | 0.373 | 0.5461 | No |
| 33 | KRT24 | na | 2352 | 0.368 | 0.5525 | No |
| 34 | PCSK6 | na | 2412 | 0.360 | 0.5565 | No |
| 35 | EPHA2 | na | 2548 | 0.341 | 0.5538 | No |
| 36 | VDR | na | 2998 | 0.282 | 0.5232 | No |
| 37 | WNT5A | na | 3037 | 0.277 | 0.5269 | No |
| 38 | IFT74 | na | 3162 | 0.261 | 0.5231 | No |
| 39 | CERS3 | na | 3332 | 0.239 | 0.5149 | No |
| 40 | ROCK1 | na | 3380 | 0.235 | 0.5168 | No |
| 41 | ZFP36L1 | na | 3496 | 0.221 | 0.5127 | No |
| 42 | JUP | na | 3549 | 0.213 | 0.5137 | No |
| 43 | ETV4 | na | 3743 | 0.194 | 0.5024 | No |
| 44 | NUMA1 | na | 3803 | 0.187 | 0.5021 | No |
| 45 | YAP1 | na | 3813 | 0.187 | 0.5060 | No |
| 46 | EREG | na | 3839 | 0.183 | 0.5085 | No |
| 47 | CTSK | na | 4067 | 0.162 | 0.4935 | No |
| 48 | LATS1 | na | 4302 | 0.142 | 0.4775 | No |
| 49 | ADAM9 | na | 4515 | 0.123 | 0.4628 | No |
| 50 | MAP2K1 | na | 4692 | 0.107 | 0.4507 | No |
| 51 | PALLD | na | 4949 | 0.084 | 0.4313 | No |
| 52 | KDF1 | na | 5010 | 0.078 | 0.4283 | No |
| 53 | JAG1 | na | 5698 | 0.017 | 0.3711 | No |
| 54 | PPHLN1 | na | 5846 | 0.006 | 0.3590 | No |
| 55 | TSG101 | na | 6359 | -0.035 | 0.3169 | No |
| 56 | PRKCH | na | 6364 | -0.035 | 0.3175 | No |
| 57 | CBFB | na | 6768 | -0.068 | 0.2854 | No |
| 58 | STK4 | na | 6949 | -0.085 | 0.2725 | No |
| 59 | TMEM79 | na | 7320 | -0.120 | 0.2445 | No |
| 60 | SRSF6 | na | 7573 | -0.141 | 0.2269 | No |
| 61 | ASAH1 | na | 7648 | -0.148 | 0.2244 | No |
| 62 | ERRFI1 | na | 7805 | -0.162 | 0.2153 | No |
| 63 | SAV1 | na | 8152 | -0.193 | 0.1912 | No |
| 64 | NOTCH1 | na | 8388 | -0.217 | 0.1769 | No |
| 65 | SPINK5 | na | 9063 | -0.291 | 0.1277 | No |
| 66 | RUNX1 | na | 9252 | -0.316 | 0.1199 | No |
| 67 | FURIN | na | 9299 | -0.321 | 0.1240 | No |
| 68 | MED1 | na | 9560 | -0.358 | 0.1112 | No |
| 69 | BCR | na | 9627 | -0.366 | 0.1148 | No |
| 70 | IRF6 | na | 10011 | -0.416 | 0.0931 | No |
| 71 | CDH3 | na | 10206 | -0.445 | 0.0880 | No |
| 72 | CTSV | na | 10774 | -0.554 | 0.0543 | No |
| 73 | PIP5K1A | na | 11015 | -0.613 | 0.0496 | No |
| 74 | NME2 | na | 11220 | -0.671 | 0.0492 | No |
| 75 | HOXA7 | na | 11232 | -0.675 | 0.0652 | No |
Table: GSEA details [plain text format]

  

Fig 2: GOBP\_KERATINOCYTE\_DIFFERENTIATION      
 Blue-Pink O' Gram in the Space of the Analyzed GeneSet

  

Fig 3: GOBP\_KERATINOCYTE\_DIFFERENTIATION: Random ES distribution      
 Gene set null distribution of ES for **GOBP\_KERATINOCYTE\_DIFFERENTIATION**

  
