# Supplementary material for: Time-series transcriptome analysis identified differentially expressed genes in broiler chicken infected with mixed Eimeria species
Source: Front Genet. 2022 Aug 8;13:886781. doi: 10.3389/fgene.2022.886781 (PMC9393255; doi:10.3389/fgene.2022.886781)
Supplement: Supplementary file 2 [file DataSheet1.ZIP › 4dpi_GO.Gsea.1625071243202/GOBP_LIPID_HOMEOSTASIS.html]

Details for gene set GOBP\_LIPID\_HOMEOSTASIS[GSEA]

|  || Dataset | TMM\_4dpi\_gct\_format\_4dpi\_gct\_format.Class\_4dpi.cls #PC\_versus\_NC.Class\_4dpi.cls #PC\_versus\_NC\_repos |
| Phenotype | Class\_4dpi.cls#PC\_versus\_NC\_repos |
| Upregulated in class | 1 |
| GeneSet | GOBP\_LIPID\_HOMEOSTASIS |
| Enrichment Score (ES) | 0.5784745 |
| Normalized Enrichment Score (NES) | 2.2257268 |
| Nominal p-value | 0.0 |
| FDR q-value | 3.509825E-4 |
| FWER p-Value | 0.002 |
Table: GSEA Results Summary

  

Fig 1: Enrichment plot: GOBP\_LIPID\_HOMEOSTASIS      
 Profile of the Running ES Score & Positions of GeneSet Members on the Rank Ordered List

  

| SYMBOL | TITLE | RANK IN GENE LIST | RANK METRIC SCORE | RUNNING ES | CORE ENRICHMENT || 1 | ABCG8 | na | 2 | 3.384 | 0.0677 | Yes |
| 2 | ABCG5 | na | 41 | 2.059 | 0.1059 | Yes |
| 3 | PLA2G12B | na | 115 | 1.564 | 0.1311 | Yes |
| 4 | PNPLA8 | na | 119 | 1.559 | 0.1622 | Yes |
| 5 | PNPLA2 | na | 141 | 1.495 | 0.1904 | Yes |
| 6 | INSIG1 | na | 148 | 1.471 | 0.2194 | Yes |
| 7 | LDLR | na | 159 | 1.437 | 0.2474 | Yes |
| 8 | APOB | na | 248 | 1.255 | 0.2652 | Yes |
| 9 | RBP1 | na | 314 | 1.157 | 0.2830 | Yes |
| 10 | SREBF2 | na | 333 | 1.135 | 0.3042 | Yes |
| 11 | MALRD1 | na | 336 | 1.133 | 0.3268 | Yes |
| 12 | SOAT1 | na | 350 | 1.121 | 0.3482 | Yes |
| 13 | ACACA | na | 361 | 1.109 | 0.3696 | Yes |
| 14 | MALL | na | 364 | 1.107 | 0.3917 | Yes |
| 15 | MTTP | na | 492 | 0.971 | 0.4005 | Yes |
| 16 | APOA1 | na | 589 | 0.889 | 0.4103 | Yes |
| 17 | DGAT2 | na | 643 | 0.854 | 0.4230 | Yes |
| 18 | APOA4 | na | 733 | 0.797 | 0.4315 | Yes |
| 19 | IRS2 | na | 755 | 0.787 | 0.4455 | Yes |
| 20 | PNPLA3 | na | 817 | 0.753 | 0.4555 | Yes |
| 21 | LPL | na | 954 | 0.691 | 0.4580 | Yes |
| 22 | NPC1 | na | 1035 | 0.659 | 0.4645 | Yes |
| 23 | LIMA1 | na | 1076 | 0.647 | 0.4741 | Yes |
| 24 | FABP3 | na | 1105 | 0.636 | 0.4846 | Yes |
| 25 | IL18 | na | 1198 | 0.604 | 0.4890 | Yes |
| 26 | MLXIPL | na | 1199 | 0.604 | 0.5011 | Yes |
| 27 | ABHD4 | na | 1312 | 0.572 | 0.5032 | Yes |
| 28 | ANGPTL4 | na | 1324 | 0.568 | 0.5137 | Yes |
| 29 | HNF4A | na | 1369 | 0.556 | 0.5211 | Yes |
| 30 | PRKAA1 | na | 1419 | 0.542 | 0.5279 | Yes |
| 31 | FABP4 | na | 1519 | 0.515 | 0.5299 | Yes |
| 32 | RALY | na | 1524 | 0.514 | 0.5399 | Yes |
| 33 | TTC39B | na | 1575 | 0.502 | 0.5458 | Yes |
| 34 | ATP13A2 | na | 1579 | 0.500 | 0.5556 | Yes |
| 35 | TSKU | na | 1738 | 0.468 | 0.5517 | Yes |
| 36 | THADA | na | 1746 | 0.466 | 0.5605 | Yes |
| 37 | DISP3 | na | 1775 | 0.461 | 0.5674 | Yes |
| 38 | TLCD1 | na | 1813 | 0.453 | 0.5733 | Yes |
| 39 | NUS1 | na | 1926 | 0.436 | 0.5727 | Yes |
| 40 | CAV1 | na | 1967 | 0.429 | 0.5779 | Yes |
| 41 | ORMDL1 | na | 2060 | 0.411 | 0.5785 | Yes |
| 42 | GPAM | na | 2506 | 0.348 | 0.5481 | No |
| 43 | RORA | na | 2627 | 0.332 | 0.5447 | No |
| 44 | CLN8 | na | 2628 | 0.332 | 0.5514 | No |
| 45 | ZBTB20 | na | 3022 | 0.279 | 0.5240 | No |
| 46 | PNPLA4 | na | 3086 | 0.270 | 0.5241 | No |
| 47 | ABCB11 | na | 3136 | 0.264 | 0.5253 | No |
| 48 | NR1H3 | na | 3213 | 0.255 | 0.5241 | No |
| 49 | ACOX3 | na | 3827 | 0.184 | 0.4763 | No |
| 50 | GOT1 | na | 3928 | 0.174 | 0.4714 | No |
| 51 | PCSK9 | na | 3963 | 0.171 | 0.4720 | No |
| 52 | USF1 | na | 4497 | 0.124 | 0.4298 | No |
| 53 | LIPG | na | 4514 | 0.123 | 0.4309 | No |
| 54 | TLCD2 | na | 4674 | 0.108 | 0.4198 | No |
| 55 | NR1D2 | na | 4759 | 0.101 | 0.4147 | No |
| 56 | ACOX1 | na | 4782 | 0.099 | 0.4149 | No |
| 57 | PRKAA2 | na | 4804 | 0.097 | 0.4151 | No |
| 58 | TMEM97 | na | 4835 | 0.095 | 0.4144 | No |
| 59 | RTN4 | na | 5155 | 0.064 | 0.3890 | No |
| 60 | CNBP | na | 5374 | 0.048 | 0.3716 | No |
| 61 | MYLIP | na | 5422 | 0.044 | 0.3686 | No |
| 62 | EPHX2 | na | 5461 | 0.040 | 0.3662 | No |
| 63 | SCARB1 | na | 5664 | 0.020 | 0.3497 | No |
| 64 | XBP1 | na | 5735 | 0.014 | 0.3441 | No |
| 65 | LDLRAP1 | na | 5933 | -0.002 | 0.3276 | No |
| 66 | DDX3X | na | 6973 | -0.087 | 0.2421 | No |
| 67 | GRAMD1B | na | 7049 | -0.094 | 0.2377 | No |
| 68 | ADCK1 | na | 7230 | -0.111 | 0.2249 | No |
| 69 | PPARG | na | 7261 | -0.114 | 0.2246 | No |
| 70 | ORMDL2 | na | 7384 | -0.125 | 0.2169 | No |
| 71 | NR1H4 | na | 7657 | -0.148 | 0.1971 | No |
| 72 | LAMTOR1 | na | 7770 | -0.159 | 0.1909 | No |
| 73 | HDAC9 | na | 7771 | -0.159 | 0.1940 | No |
| 74 | LRP5 | na | 7897 | -0.170 | 0.1870 | No |
| 75 | ITGB6 | na | 7961 | -0.175 | 0.1852 | No |
| 76 | ACOX2 | na | 7988 | -0.178 | 0.1866 | No |
| 77 | NPC2 | na | 8394 | -0.218 | 0.1570 | No |
| 78 | ABCA2 | na | 8565 | -0.237 | 0.1475 | No |
| 79 | FBXW7 | na | 8851 | -0.268 | 0.1289 | No |
| 80 | ABCA3 | na | 9099 | -0.295 | 0.1141 | No |
| 81 | FGFR4 | na | 9361 | -0.330 | 0.0989 | No |
| 82 | NR5A2 | na | 9423 | -0.339 | 0.1005 | No |
| 83 | ABCA1 | na | 9675 | -0.371 | 0.0869 | No |
| 84 | SIRT1 | na | 9868 | -0.394 | 0.0787 | No |
| 85 | ORMDL3 | na | 10175 | -0.441 | 0.0619 | No |
| 86 | NR1D1 | na | 10243 | -0.451 | 0.0653 | No |
| 87 | CYP39A1 | na | 10542 | -0.507 | 0.0505 | No |
| 88 | COMMD9 | na | 10592 | -0.518 | 0.0568 | No |
| 89 | ABCG1 | na | 11046 | -0.620 | 0.0312 | No |
| 90 | CD24 | na | 11079 | -0.630 | 0.0412 | No |
| 91 | SESN2 | na | 11162 | -0.653 | 0.0474 | No |
| 92 | LCAT | na | 11849 | -1.179 | 0.0135 | No |
Table: GSEA details [plain text format]

  

Fig 2: GOBP\_LIPID\_HOMEOSTASIS      
 Blue-Pink O' Gram in the Space of the Analyzed GeneSet

  

Fig 3: GOBP\_LIPID\_HOMEOSTASIS: Random ES distribution      
 Gene set null distribution of ES for **GOBP\_LIPID\_HOMEOSTASIS**

  
