# Supplementary material for: Time-series transcriptome analysis identified differentially expressed genes in broiler chicken infected with mixed Eimeria species
Source: Front Genet. 2022 Aug 8;13:886781. doi: 10.3389/fgene.2022.886781 (PMC9393255; doi:10.3389/fgene.2022.886781)
Supplement: Supplementary file 2 [file DataSheet1.ZIP › 4dpi_GO.Gsea.1625071243202/GOBP_NUCLEAR_TRANSCRIBED_MRNA_CATABOLIC_PROCESS.html]

Details for gene set GOBP\_NUCLEAR\_TRANSCRIBED\_MRNA\_CATABOLIC\_PROCESS[GSEA]

|  || Dataset | TMM\_4dpi\_gct\_format\_4dpi\_gct\_format.Class\_4dpi.cls #PC\_versus\_NC.Class\_4dpi.cls #PC\_versus\_NC\_repos |
| Phenotype | Class\_4dpi.cls#PC\_versus\_NC\_repos |
| Upregulated in class | 0 |
| GeneSet | GOBP\_NUCLEAR\_TRANSCRIBED\_MRNA\_CATABOLIC\_PROCESS |
| Enrichment Score (ES) | -0.4903439 |
| Normalized Enrichment Score (NES) | -2.2269535 |
| Nominal p-value | 0.0 |
| FDR q-value | 3.6971708E-4 |
| FWER p-Value | 0.008 |
Table: GSEA Results Summary

  

Fig 1: Enrichment plot: GOBP\_NUCLEAR\_TRANSCRIBED\_MRNA\_CATABOLIC\_PROCESS      
 Profile of the Running ES Score & Positions of GeneSet Members on the Rank Ordered List

  

| SYMBOL | TITLE | RANK IN GENE LIST | RANK METRIC SCORE | RUNNING ES | CORE ENRICHMENT || 1 | HELZ2 | na | 64 | 1.875 | 0.0199 | No |
| 2 | PATL2 | na | 316 | 1.156 | 0.0143 | No |
| 3 | BTG2 | na | 502 | 0.961 | 0.0116 | No |
| 4 | XRN1 | na | 666 | 0.844 | 0.0093 | No |
| 5 | CPEB3 | na | 738 | 0.795 | 0.0140 | No |
| 6 | UPF2 | na | 1044 | 0.656 | -0.0029 | No |
| 7 | MLH1 | na | 1305 | 0.575 | -0.0171 | No |
| 8 | TNRC6B | na | 1666 | 0.481 | -0.0410 | No |
| 9 | CTIF | na | 1868 | 0.444 | -0.0520 | No |
| 10 | XRN2 | na | 2035 | 0.416 | -0.0604 | No |
| 11 | LSM4 | na | 2061 | 0.411 | -0.0570 | No |
| 12 | RC3H2 | na | 2068 | 0.410 | -0.0520 | No |
| 13 | LSM6 | na | 2124 | 0.401 | -0.0512 | No |
| 14 | NT5C3B | na | 2172 | 0.393 | -0.0499 | No |
| 15 | THRAP3 | na | 2176 | 0.392 | -0.0448 | No |
| 16 | RC3H1 | na | 2457 | 0.353 | -0.0637 | No |
| 17 | LSM7 | na | 2580 | 0.337 | -0.0695 | No |
| 18 | ATM | na | 2597 | 0.336 | -0.0663 | No |
| 19 | SECISBP2 | na | 2598 | 0.336 | -0.0618 | No |
| 20 | TNRC6A | na | 2648 | 0.330 | -0.0615 | No |
| 21 | DCP1A | na | 2726 | 0.319 | -0.0637 | No |
| 22 | DCP2 | na | 2829 | 0.305 | -0.0682 | No |
| 23 | SMG1 | na | 2854 | 0.302 | -0.0661 | No |
| 24 | NBAS | na | 2866 | 0.301 | -0.0630 | No |
| 25 | LSM3 | na | 2913 | 0.294 | -0.0629 | No |
| 26 | ZFP36L2 | na | 2962 | 0.287 | -0.0631 | No |
| 27 | LSM5 | na | 3035 | 0.277 | -0.0654 | No |
| 28 | CNOT2 | na | 3174 | 0.260 | -0.0736 | No |
| 29 | TTC37 | na | 3201 | 0.256 | -0.0723 | No |
| 30 | DDX6 | na | 3238 | 0.252 | -0.0720 | No |
| 31 | PELO | na | 3406 | 0.232 | -0.0829 | No |
| 32 | CNOT8 | na | 3439 | 0.228 | -0.0826 | No |
| 33 | HBS1L | na | 3442 | 0.227 | -0.0797 | No |
| 34 | ZFP36L1 | na | 3496 | 0.221 | -0.0812 | No |
| 35 | PPP2R2A | na | 3550 | 0.213 | -0.0828 | No |
| 36 | EXOSC8 | na | 3571 | 0.210 | -0.0816 | No |
| 37 | UPF3B | na | 3628 | 0.205 | -0.0836 | No |
| 38 | CNOT7 | na | 3796 | 0.188 | -0.0952 | No |
| 39 | MRTO4 | na | 3990 | 0.169 | -0.1092 | No |
| 40 | EXOSC1 | na | 4012 | 0.166 | -0.1087 | No |
| 41 | PPP2CA | na | 4110 | 0.159 | -0.1148 | No |
| 42 | WDR61 | na | 4191 | 0.152 | -0.1195 | No |
| 43 | PARN | na | 4246 | 0.147 | -0.1221 | No |
| 44 | PNRC2 | na | 4384 | 0.135 | -0.1318 | No |
| 45 | TNKS1BP1 | na | 4795 | 0.098 | -0.1651 | No |
| 46 | NANOS1 | na | 4862 | 0.092 | -0.1694 | No |
| 47 | SKIV2L | na | 4939 | 0.085 | -0.1747 | No |
| 48 | CASC3 | na | 5177 | 0.063 | -0.1939 | No |
| 49 | CNOT6 | na | 5320 | 0.052 | -0.2052 | No |
| 50 | EIF4A3 | na | 5359 | 0.049 | -0.2077 | No |
| 51 | DIS3L2 | na | 5592 | 0.027 | -0.2270 | No |
| 52 | EXOSC10 | na | 5622 | 0.024 | -0.2291 | No |
| 53 | DCP1B | na | 5707 | 0.016 | -0.2360 | No |
| 54 | NCBP1 | na | 5782 | 0.010 | -0.2421 | No |
| 55 | LSM1 | na | 5786 | 0.010 | -0.2422 | No |
| 56 | EIF4G1 | na | 5833 | 0.007 | -0.2460 | No |
| 57 | MAGOH | na | 6115 | -0.016 | -0.2695 | No |
| 58 | PCID2 | na | 6124 | -0.017 | -0.2700 | No |
| 59 | EIF4ENIF1 | na | 6154 | -0.019 | -0.2722 | No |
| 60 | NOCT | na | 6241 | -0.026 | -0.2791 | No |
| 61 | SAMD4A | na | 6322 | -0.032 | -0.2854 | No |
| 62 | MTPAP | na | 6334 | -0.032 | -0.2859 | No |
| 63 | CSDE1 | na | 6343 | -0.033 | -0.2861 | No |
| 64 | GSPT2 | na | 6351 | -0.034 | -0.2863 | No |
| 65 | PAN3 | na | 6353 | -0.034 | -0.2859 | No |
| 66 | SSB | na | 6362 | -0.035 | -0.2861 | No |
| 67 | PATL1 | na | 6368 | -0.035 | -0.2860 | No |
| 68 | DCPS | na | 6369 | -0.035 | -0.2856 | No |
| 69 | EXOSC7 | na | 6423 | -0.040 | -0.2895 | No |
| 70 | PAN2 | na | 6434 | -0.041 | -0.2898 | No |
| 71 | RBM8A | na | 6463 | -0.044 | -0.2916 | No |
| 72 | PRPF18 | na | 6741 | -0.066 | -0.3141 | No |
| 73 | CNOT6L | na | 6893 | -0.078 | -0.3258 | No |
| 74 | UPF1 | na | 6934 | -0.083 | -0.3280 | No |
| 75 | ETF1 | na | 7386 | -0.125 | -0.3644 | No |
| 76 | RNPS1 | na | 7453 | -0.131 | -0.3682 | No |
| 77 | SMG7 | na | 7459 | -0.132 | -0.3669 | No |
| 78 | SMG5 | na | 7562 | -0.140 | -0.3736 | No |
| 79 | EDC4 | na | 7659 | -0.148 | -0.3797 | No |
| 80 | ZC3H12A | na | 7694 | -0.152 | -0.3805 | No |
| 81 | DHX36 | na | 7816 | -0.163 | -0.3886 | No |
| 82 | CNOT4 | na | 8028 | -0.182 | -0.4039 | No |
| 83 | DDX5 | na | 8057 | -0.185 | -0.4038 | No |
| 84 | CNOT10 | na | 8209 | -0.199 | -0.4139 | No |
| 85 | RPS23 | na | 8269 | -0.206 | -0.4161 | No |
| 86 | EXOSC5 | na | 8301 | -0.208 | -0.4159 | No |
| 87 | CNOT11 | na | 8483 | -0.227 | -0.4281 | No |
| 88 | RPL17 | na | 8583 | -0.239 | -0.4332 | No |
| 89 | AGO1 | na | 8653 | -0.245 | -0.4358 | No |
| 90 | EXOSC6 | na | 8803 | -0.262 | -0.4448 | No |
| 91 | CNOT1 | na | 8856 | -0.269 | -0.4456 | No |
| 92 | RPS6 | na | 8904 | -0.274 | -0.4459 | No |
| 93 | EXOSC2 | na | 8930 | -0.277 | -0.4442 | No |
| 94 | DIS3 | na | 8937 | -0.277 | -0.4410 | No |
| 95 | EDC3 | na | 9023 | -0.285 | -0.4443 | No |
| 96 | SMG6 | na | 9097 | -0.295 | -0.4465 | No |
| 97 | RPL36 | na | 9363 | -0.330 | -0.4645 | No |
| 98 | UBA52 | na | 9532 | -0.353 | -0.4739 | No |
| 99 | RPL38 | na | 9661 | -0.369 | -0.4797 | No |
| 100 | RPS24 | na | 9689 | -0.372 | -0.4770 | No |
| 101 | NCBP2 | na | 9714 | -0.376 | -0.4739 | No |
| 102 | TNRC6C | na | 9727 | -0.377 | -0.4699 | No |
| 103 | CNOT9 | na | 9734 | -0.378 | -0.4653 | No |
| 104 | POLR2D | na | 9821 | -0.389 | -0.4673 | No |
| 105 | SAMD4B | na | 9914 | -0.400 | -0.4697 | No |
| 106 | RPLP2 | na | 10048 | -0.421 | -0.4752 | No |
| 107 | RPL37 | na | 10057 | -0.423 | -0.4702 | No |
| 108 | RPS8 | na | 10245 | -0.452 | -0.4799 | No |
| 109 | RPL27 | na | 10333 | -0.467 | -0.4809 | No |
| 110 | PYM1 | na | 10431 | -0.485 | -0.4826 | No |
| 111 | RPS28 | na | 10524 | -0.504 | -0.4835 | Yes |
| 112 | RPL30 | na | 10537 | -0.506 | -0.4777 | Yes |
| 113 | RPL22 | na | 10554 | -0.508 | -0.4722 | Yes |
| 114 | RPL36A | na | 10605 | -0.520 | -0.4694 | Yes |
| 115 | EXOSC9 | na | 10652 | -0.530 | -0.4662 | Yes |
| 116 | RPL14 | na | 10662 | -0.531 | -0.4598 | Yes |
| 117 | RPL29 | na | 10742 | -0.548 | -0.4591 | Yes |
| 118 | SMG8 | na | 10782 | -0.555 | -0.4549 | Yes |
| 119 | RPL37A | na | 10838 | -0.569 | -0.4518 | Yes |
| 120 | RPS19 | na | 10840 | -0.569 | -0.4442 | Yes |
| 121 | RPS12 | na | 10875 | -0.576 | -0.4393 | Yes |
| 122 | RPL24 | na | 10877 | -0.576 | -0.4316 | Yes |
| 123 | EXOSC3 | na | 10892 | -0.581 | -0.4250 | Yes |
| 124 | PDE12 | na | 10911 | -0.584 | -0.4186 | Yes |
| 125 | RPL34 | na | 10933 | -0.588 | -0.4124 | Yes |
| 126 | RPL23 | na | 10981 | -0.602 | -0.4083 | Yes |
| 127 | RPL35A | na | 10982 | -0.602 | -0.4002 | Yes |
| 128 | RPS25 | na | 11017 | -0.613 | -0.3948 | Yes |
| 129 | RPS7 | na | 11068 | -0.627 | -0.3905 | Yes |
| 130 | RPL23A | na | 11076 | -0.629 | -0.3826 | Yes |
| 131 | RPL5 | na | 11121 | -0.643 | -0.3777 | Yes |
| 132 | RPS16 | na | 11138 | -0.645 | -0.3703 | Yes |
| 133 | RPL6 | na | 11150 | -0.650 | -0.3625 | Yes |
| 134 | RPL11 | na | 11195 | -0.662 | -0.3573 | Yes |
| 135 | RPS15A | na | 11196 | -0.662 | -0.3483 | Yes |
| 136 | RPLP1 | na | 11249 | -0.682 | -0.3435 | Yes |
| 137 | RPS26 | na | 11254 | -0.686 | -0.3346 | Yes |
| 138 | RPL35 | na | 11280 | -0.696 | -0.3273 | Yes |
| 139 | RPL31 | na | 11306 | -0.706 | -0.3199 | Yes |
| 140 | RPS21 | na | 11313 | -0.709 | -0.3108 | Yes |
| 141 | RPS3A | na | 11328 | -0.713 | -0.3024 | Yes |
| 142 | RPL21 | na | 11330 | -0.714 | -0.2928 | Yes |
| 143 | RPS10 | na | 11344 | -0.720 | -0.2842 | Yes |
| 144 | RPL32 | na | 11355 | -0.725 | -0.2753 | Yes |
| 145 | RPS11 | na | 11371 | -0.735 | -0.2666 | Yes |
| 146 | RPL12 | na | 11398 | -0.748 | -0.2587 | Yes |
| 147 | RPL7A | na | 11426 | -0.763 | -0.2507 | Yes |
| 148 | RPS15 | na | 11434 | -0.767 | -0.2410 | Yes |
| 149 | RPS27A | na | 11435 | -0.767 | -0.2306 | Yes |
| 150 | RPL15 | na | 11440 | -0.769 | -0.2206 | Yes |
| 151 | RPS29 | na | 11444 | -0.771 | -0.2104 | Yes |
| 152 | RPL18A | na | 11474 | -0.789 | -0.2022 | Yes |
| 153 | RPS14 | na | 11478 | -0.792 | -0.1918 | Yes |
| 154 | RPL7 | na | 11484 | -0.796 | -0.1815 | Yes |
| 155 | RPLP0 | na | 11488 | -0.800 | -0.1709 | Yes |
| 156 | RPL27A | na | 11507 | -0.812 | -0.1615 | Yes |
| 157 | RPL9 | na | 11521 | -0.819 | -0.1515 | Yes |
| 158 | PABPC1 | na | 11551 | -0.839 | -0.1427 | Yes |
| 159 | RPS2 | na | 11562 | -0.846 | -0.1321 | Yes |
| 160 | RPL13 | na | 11563 | -0.847 | -0.1207 | Yes |
| 161 | RPS20 | na | 11594 | -0.868 | -0.1115 | Yes |
| 162 | RPS27 | na | 11600 | -0.875 | -0.1001 | Yes |
| 163 | RPL19 | na | 11611 | -0.882 | -0.0890 | Yes |
| 164 | RPS13 | na | 11633 | -0.897 | -0.0787 | Yes |
| 165 | RPS3 | na | 11641 | -0.903 | -0.0671 | Yes |
| 166 | RPS17 | na | 11652 | -0.916 | -0.0556 | Yes |
| 167 | RPL10A | na | 11658 | -0.921 | -0.0436 | Yes |
| 168 | RPL4 | na | 11715 | -0.983 | -0.0351 | Yes |
| 169 | RPL8 | na | 11772 | -1.039 | -0.0258 | Yes |
| 170 | RPS4Y1 | na | 11774 | -1.043 | -0.0118 | Yes |
| 171 | RPL3 | na | 11817 | -1.140 | 0.0000 | Yes |
| 172 | EIF3E | na | 11859 | -1.199 | 0.0128 | Yes |
Table: GSEA details [plain text format]

  

Fig 2: GOBP\_NUCLEAR\_TRANSCRIBED\_MRNA\_CATABOLIC\_PROCESS      
 Blue-Pink O' Gram in the Space of the Analyzed GeneSet

  

Fig 3: GOBP\_NUCLEAR\_TRANSCRIBED\_MRNA\_CATABOLIC\_PROCESS: Random ES distribution      
 Gene set null distribution of ES for **GOBP\_NUCLEAR\_TRANSCRIBED\_MRNA\_CATABOLIC\_PROCESS**

  
