# Supplementary material for: Time-series transcriptome analysis identified differentially expressed genes in broiler chicken infected with mixed Eimeria species
Source: Front Genet. 2022 Aug 8;13:886781. doi: 10.3389/fgene.2022.886781 (PMC9393255; doi:10.3389/fgene.2022.886781)
Supplement: Supplementary file 2 [file DataSheet1.ZIP › 4dpi_GO.Gsea.1625071243202/GOBP_NUCLEAR_TRANSCRIBED_MRNA_CATABOLIC_PROCESS_NONSENSE_MEDIATED_DECAY.html]

Details for gene set GOBP\_NUCLEAR\_TRANSCRIBED\_MRNA\_CATABOLIC\_PROCESS\_NONSENSE\_MEDIATED\_DECAY[GSEA]

|  || Dataset | TMM\_4dpi\_gct\_format\_4dpi\_gct\_format.Class\_4dpi.cls #PC\_versus\_NC.Class\_4dpi.cls #PC\_versus\_NC\_repos |
| Phenotype | Class\_4dpi.cls#PC\_versus\_NC\_repos |
| Upregulated in class | 0 |
| GeneSet | GOBP\_NUCLEAR\_TRANSCRIBED\_MRNA\_CATABOLIC\_PROCESS\_NONSENSE\_MEDIATED\_DECAY |
| Enrichment Score (ES) | -0.6748537 |
| Normalized Enrichment Score (NES) | -2.833605 |
| Nominal p-value | 0.0 |
| FDR q-value | 0.0 |
| FWER p-Value | 0.0 |
Table: GSEA Results Summary

  

Fig 1: Enrichment plot: GOBP\_NUCLEAR\_TRANSCRIBED\_MRNA\_CATABOLIC\_PROCESS\_NONSENSE\_MEDIATED\_DECAY      
 Profile of the Running ES Score & Positions of GeneSet Members on the Rank Ordered List

  

| SYMBOL | TITLE | RANK IN GENE LIST | RANK METRIC SCORE | RUNNING ES | CORE ENRICHMENT || 1 | HELZ2 | na | 64 | 1.875 | 0.0287 | No |
| 2 | UPF2 | na | 1044 | 0.656 | -0.0415 | No |
| 3 | CTIF | na | 1868 | 0.444 | -0.1025 | No |
| 4 | SECISBP2 | na | 2598 | 0.336 | -0.1576 | No |
| 5 | DCP1A | na | 2726 | 0.319 | -0.1625 | No |
| 6 | DCP2 | na | 2829 | 0.305 | -0.1655 | No |
| 7 | SMG1 | na | 2854 | 0.302 | -0.1621 | No |
| 8 | NBAS | na | 2866 | 0.301 | -0.1575 | No |
| 9 | PPP2R2A | na | 3550 | 0.213 | -0.2110 | No |
| 10 | UPF3B | na | 3628 | 0.205 | -0.2137 | No |
| 11 | PPP2CA | na | 4110 | 0.159 | -0.2512 | No |
| 12 | PARN | na | 4246 | 0.147 | -0.2599 | No |
| 13 | PNRC2 | na | 4384 | 0.135 | -0.2689 | No |
| 14 | SKIV2L | na | 4939 | 0.085 | -0.3139 | No |
| 15 | CASC3 | na | 5177 | 0.063 | -0.3327 | No |
| 16 | EIF4A3 | na | 5359 | 0.049 | -0.3470 | No |
| 17 | EXOSC10 | na | 5622 | 0.024 | -0.3686 | No |
| 18 | DCP1B | na | 5707 | 0.016 | -0.3753 | No |
| 19 | NCBP1 | na | 5782 | 0.010 | -0.3813 | No |
| 20 | EIF4G1 | na | 5833 | 0.007 | -0.3854 | No |
| 21 | MAGOH | na | 6115 | -0.016 | -0.4087 | No |
| 22 | GSPT2 | na | 6351 | -0.034 | -0.4278 | No |
| 23 | RBM8A | na | 6463 | -0.044 | -0.4364 | No |
| 24 | UPF1 | na | 6934 | -0.083 | -0.4743 | No |
| 25 | ETF1 | na | 7386 | -0.125 | -0.5099 | No |
| 26 | RNPS1 | na | 7453 | -0.131 | -0.5131 | No |
| 27 | SMG7 | na | 7459 | -0.132 | -0.5111 | No |
| 28 | SMG5 | na | 7562 | -0.140 | -0.5171 | No |
| 29 | RPS23 | na | 8269 | -0.206 | -0.5726 | No |
| 30 | RPL17 | na | 8583 | -0.239 | -0.5946 | No |
| 31 | RPS6 | na | 8904 | -0.274 | -0.6165 | No |
| 32 | SMG6 | na | 9097 | -0.295 | -0.6272 | No |
| 33 | RPL36 | na | 9363 | -0.330 | -0.6435 | No |
| 34 | UBA52 | na | 9532 | -0.353 | -0.6512 | No |
| 35 | RPL38 | na | 9661 | -0.369 | -0.6552 | No |
| 36 | RPS24 | na | 9689 | -0.372 | -0.6507 | No |
| 37 | NCBP2 | na | 9714 | -0.376 | -0.6459 | No |
| 38 | RPLP2 | na | 10048 | -0.421 | -0.6662 | No |
| 39 | RPL37 | na | 10057 | -0.423 | -0.6592 | No |
| 40 | RPS8 | na | 10245 | -0.452 | -0.6666 | Yes |
| 41 | RPL27 | na | 10333 | -0.467 | -0.6654 | Yes |
| 42 | PYM1 | na | 10431 | -0.485 | -0.6648 | Yes |
| 43 | RPS28 | na | 10524 | -0.504 | -0.6633 | Yes |
| 44 | RPL30 | na | 10537 | -0.506 | -0.6551 | Yes |
| 45 | RPL22 | na | 10554 | -0.508 | -0.6472 | Yes |
| 46 | RPL36A | na | 10605 | -0.520 | -0.6420 | Yes |
| 47 | RPL14 | na | 10662 | -0.531 | -0.6370 | Yes |
| 48 | RPL29 | na | 10742 | -0.548 | -0.6337 | Yes |
| 49 | SMG8 | na | 10782 | -0.555 | -0.6269 | Yes |
| 50 | RPL37A | na | 10838 | -0.569 | -0.6211 | Yes |
| 51 | RPS19 | na | 10840 | -0.569 | -0.6109 | Yes |
| 52 | RPS12 | na | 10875 | -0.576 | -0.6032 | Yes |
| 53 | RPL24 | na | 10877 | -0.576 | -0.5928 | Yes |
| 54 | RPL34 | na | 10933 | -0.588 | -0.5868 | Yes |
| 55 | RPL23 | na | 10981 | -0.602 | -0.5798 | Yes |
| 56 | RPL35A | na | 10982 | -0.602 | -0.5688 | Yes |
| 57 | RPS25 | na | 11017 | -0.613 | -0.5605 | Yes |
| 58 | RPS7 | na | 11068 | -0.627 | -0.5533 | Yes |
| 59 | RPL23A | na | 11076 | -0.629 | -0.5425 | Yes |
| 60 | RPL5 | na | 11121 | -0.643 | -0.5345 | Yes |
| 61 | RPS16 | na | 11138 | -0.645 | -0.5241 | Yes |
| 62 | RPL6 | na | 11150 | -0.650 | -0.5132 | Yes |
| 63 | RPL11 | na | 11195 | -0.662 | -0.5048 | Yes |
| 64 | RPS15A | na | 11196 | -0.662 | -0.4928 | Yes |
| 65 | RPLP1 | na | 11249 | -0.682 | -0.4847 | Yes |
| 66 | RPS26 | na | 11254 | -0.686 | -0.4726 | Yes |
| 67 | RPL35 | na | 11280 | -0.696 | -0.4620 | Yes |
| 68 | RPL31 | na | 11306 | -0.706 | -0.4513 | Yes |
| 69 | RPS21 | na | 11313 | -0.709 | -0.4389 | Yes |
| 70 | RPS3A | na | 11328 | -0.713 | -0.4271 | Yes |
| 71 | RPL21 | na | 11330 | -0.714 | -0.4142 | Yes |
| 72 | RPS10 | na | 11344 | -0.720 | -0.4022 | Yes |
| 73 | RPL32 | na | 11355 | -0.725 | -0.3898 | Yes |
| 74 | RPS11 | na | 11371 | -0.735 | -0.3777 | Yes |
| 75 | RPL12 | na | 11398 | -0.748 | -0.3663 | Yes |
| 76 | RPL7A | na | 11426 | -0.763 | -0.3547 | Yes |
| 77 | RPS15 | na | 11434 | -0.767 | -0.3413 | Yes |
| 78 | RPS27A | na | 11435 | -0.767 | -0.3274 | Yes |
| 79 | RPL15 | na | 11440 | -0.769 | -0.3137 | Yes |
| 80 | RPS29 | na | 11444 | -0.771 | -0.2999 | Yes |
| 81 | RPL18A | na | 11474 | -0.789 | -0.2880 | Yes |
| 82 | RPS14 | na | 11478 | -0.792 | -0.2739 | Yes |
| 83 | RPL7 | na | 11484 | -0.796 | -0.2598 | Yes |
| 84 | RPLP0 | na | 11488 | -0.800 | -0.2455 | Yes |
| 85 | RPL27A | na | 11507 | -0.812 | -0.2323 | Yes |
| 86 | RPL9 | na | 11521 | -0.819 | -0.2185 | Yes |
| 87 | PABPC1 | na | 11551 | -0.839 | -0.2056 | Yes |
| 88 | RPS2 | na | 11562 | -0.846 | -0.1911 | Yes |
| 89 | RPL13 | na | 11563 | -0.847 | -0.1757 | Yes |
| 90 | RPS20 | na | 11594 | -0.868 | -0.1624 | Yes |
| 91 | RPS27 | na | 11600 | -0.875 | -0.1469 | Yes |
| 92 | RPL19 | na | 11611 | -0.882 | -0.1317 | Yes |
| 93 | RPS13 | na | 11633 | -0.897 | -0.1171 | Yes |
| 94 | RPS3 | na | 11641 | -0.903 | -0.1013 | Yes |
| 95 | RPS17 | na | 11652 | -0.916 | -0.0855 | Yes |
| 96 | RPL10A | na | 11658 | -0.921 | -0.0692 | Yes |
| 97 | RPL4 | na | 11715 | -0.983 | -0.0560 | Yes |
| 98 | RPL8 | na | 11772 | -1.039 | -0.0418 | Yes |
| 99 | RPS4Y1 | na | 11774 | -1.043 | -0.0229 | Yes |
| 100 | RPL3 | na | 11817 | -1.140 | -0.0057 | Yes |
| 101 | EIF3E | na | 11859 | -1.199 | 0.0127 | Yes |
Table: GSEA details [plain text format]

  

Fig 2: GOBP\_NUCLEAR\_TRANSCRIBED\_MRNA\_CATABOLIC\_PROCESS\_NONSENSE\_MEDIATED\_DECAY      
 Blue-Pink O' Gram in the Space of the Analyzed GeneSet

  

Fig 3: GOBP\_NUCLEAR\_TRANSCRIBED\_MRNA\_CATABOLIC\_PROCESS\_NONSENSE\_MEDIATED\_DECAY: Random ES distribution      
 Gene set null distribution of ES for **GOBP\_NUCLEAR\_TRANSCRIBED\_MRNA\_CATABOLIC\_PROCESS\_NONSENSE\_MEDIATED\_DECAY**

  
