# Supplementary material for: Time-series transcriptome analysis identified differentially expressed genes in broiler chicken infected with mixed Eimeria species
Source: Front Genet. 2022 Aug 8;13:886781. doi: 10.3389/fgene.2022.886781 (PMC9393255; doi:10.3389/fgene.2022.886781)
Supplement: Supplementary file 2 [file DataSheet1.ZIP › 4dpi_GO.Gsea.1625071243202/GOBP_PROTEIN_TARGETING_TO_MEMBRANE.html]

Details for gene set GOBP\_PROTEIN\_TARGETING\_TO\_MEMBRANE[GSEA]

|  || Dataset | TMM\_4dpi\_gct\_format\_4dpi\_gct\_format.Class\_4dpi.cls #PC\_versus\_NC.Class\_4dpi.cls #PC\_versus\_NC\_repos |
| Phenotype | Class\_4dpi.cls#PC\_versus\_NC\_repos |
| Upregulated in class | 0 |
| GeneSet | GOBP\_PROTEIN\_TARGETING\_TO\_MEMBRANE |
| Enrichment Score (ES) | -0.51833755 |
| Normalized Enrichment Score (NES) | -2.3079531 |
| Nominal p-value | 0.0 |
| FDR q-value | 8.265404E-5 |
| FWER p-Value | 0.0016 |
Table: GSEA Results Summary

  

Fig 1: Enrichment plot: GOBP\_PROTEIN\_TARGETING\_TO\_MEMBRANE      
 Profile of the Running ES Score & Positions of GeneSet Members on the Rank Ordered List

  

| SYMBOL | TITLE | RANK IN GENE LIST | RANK METRIC SCORE | RUNNING ES | CORE ENRICHMENT || 1 | SLC51B | na | 111 | 1.591 | 0.0118 | No |
| 2 | RN7SL1 | na | 179 | 1.390 | 0.0247 | No |
| 3 | ZDHHC4 | na | 457 | 1.010 | 0.0148 | No |
| 4 | ZDHHC20 | na | 573 | 0.901 | 0.0172 | No |
| 5 | VPS37B | na | 638 | 0.856 | 0.0232 | No |
| 6 | PAK1 | na | 716 | 0.806 | 0.0274 | No |
| 7 | SLC1A1 | na | 831 | 0.747 | 0.0278 | No |
| 8 | SEC61B | na | 866 | 0.729 | 0.0346 | No |
| 9 | PRKCI | na | 1053 | 0.654 | 0.0277 | No |
| 10 | RAB8B | na | 1274 | 0.583 | 0.0169 | No |
| 11 | SEC61G | na | 1475 | 0.525 | 0.0070 | No |
| 12 | C2CD5 | na | 1626 | 0.489 | 0.0009 | No |
| 13 | ZDHHC3 | na | 1943 | 0.433 | -0.0200 | No |
| 14 | MYO1C | na | 1956 | 0.431 | -0.0153 | No |
| 15 | INPP5K | na | 1977 | 0.427 | -0.0113 | No |
| 16 | HSPA5 | na | 2167 | 0.394 | -0.0219 | No |
| 17 | SEC63 | na | 2308 | 0.375 | -0.0288 | No |
| 18 | AQP11 | na | 2673 | 0.327 | -0.0551 | No |
| 19 | KCNB1 | na | 2778 | 0.311 | -0.0597 | No |
| 20 | CHMP4B | na | 2783 | 0.311 | -0.0559 | No |
| 21 | SGTA | na | 2968 | 0.286 | -0.0676 | No |
| 22 | SGTB | na | 3260 | 0.248 | -0.0888 | No |
| 23 | SEC61A1 | na | 3573 | 0.210 | -0.1123 | No |
| 24 | SRP19 | na | 3621 | 0.205 | -0.1136 | No |
| 25 | PARD3 | na | 3629 | 0.205 | -0.1114 | No |
| 26 | SRP54 | na | 3757 | 0.192 | -0.1196 | No |
| 27 | TRAM1L1 | na | 3856 | 0.182 | -0.1254 | No |
| 28 | GDI1 | na | 3862 | 0.181 | -0.1234 | No |
| 29 | ZDHHC23 | na | 4128 | 0.157 | -0.1437 | No |
| 30 | CEMIP | na | 4197 | 0.151 | -0.1474 | No |
| 31 | ATG3 | na | 4204 | 0.150 | -0.1459 | No |
| 32 | ZDHHC12 | na | 4766 | 0.100 | -0.1919 | No |
| 33 | CHM | na | 4816 | 0.096 | -0.1947 | No |
| 34 | SRPRA | na | 4934 | 0.085 | -0.2034 | No |
| 35 | MFF | na | 4974 | 0.081 | -0.2057 | No |
| 36 | PIKFYVE | na | 4994 | 0.079 | -0.2062 | No |
| 37 | SRP68 | na | 5003 | 0.078 | -0.2058 | No |
| 38 | SEC61A2 | na | 5014 | 0.077 | -0.2057 | No |
| 39 | TRAM2 | na | 5068 | 0.073 | -0.2092 | No |
| 40 | SSR1 | na | 5188 | 0.062 | -0.2184 | No |
| 41 | SDCBP | na | 5294 | 0.054 | -0.2265 | No |
| 42 | ANK3 | na | 5304 | 0.054 | -0.2265 | No |
| 43 | SSR3 | na | 5621 | 0.024 | -0.2529 | No |
| 44 | PEX26 | na | 5894 | 0.002 | -0.2758 | No |
| 45 | ZDHHC15 | na | 5994 | -0.006 | -0.2840 | No |
| 46 | VPS37A | na | 6119 | -0.016 | -0.2943 | No |
| 47 | PEX16 | na | 6477 | -0.045 | -0.3238 | No |
| 48 | ICMT | na | 6486 | -0.045 | -0.3239 | No |
| 49 | SRP14 | na | 6579 | -0.052 | -0.3309 | No |
| 50 | SRP72 | na | 6615 | -0.056 | -0.3331 | No |
| 51 | CHP1 | na | 6932 | -0.083 | -0.3587 | No |
| 52 | ERBB2 | na | 7090 | -0.097 | -0.3706 | No |
| 53 | SEC62 | na | 7190 | -0.107 | -0.3775 | No |
| 54 | FYN | na | 7681 | -0.151 | -0.4168 | No |
| 55 | CDK5R1 | na | 7736 | -0.155 | -0.4193 | No |
| 56 | DMTN | na | 7787 | -0.160 | -0.4214 | No |
| 57 | ITGB2 | na | 7941 | -0.173 | -0.4320 | No |
| 58 | MICALL1 | na | 8011 | -0.180 | -0.4354 | No |
| 59 | ITGB1BP1 | na | 8228 | -0.201 | -0.4510 | No |
| 60 | RPS23 | na | 8269 | -0.206 | -0.4516 | No |
| 61 | ZDHHC2 | na | 8535 | -0.233 | -0.4708 | No |
| 62 | RPL17 | na | 8583 | -0.239 | -0.4716 | No |
| 63 | CDK5 | na | 8689 | -0.250 | -0.4771 | No |
| 64 | MIEF1 | na | 8691 | -0.250 | -0.4739 | No |
| 65 | ZDHHC7 | na | 8775 | -0.259 | -0.4774 | No |
| 66 | PEX3 | na | 8825 | -0.265 | -0.4780 | No |
| 67 | RPS6 | na | 8904 | -0.274 | -0.4810 | No |
| 68 | EXOC4 | na | 8948 | -0.279 | -0.4809 | No |
| 69 | PRNP | na | 9074 | -0.292 | -0.4875 | No |
| 70 | VPS37C | na | 9136 | -0.300 | -0.4887 | No |
| 71 | SSR2 | na | 9215 | -0.311 | -0.4911 | No |
| 72 | HRAS | na | 9244 | -0.315 | -0.4893 | No |
| 73 | GOLGA7 | na | 9288 | -0.320 | -0.4886 | No |
| 74 | RPL36 | na | 9363 | -0.330 | -0.4905 | No |
| 75 | ZDHHC6 | na | 9481 | -0.345 | -0.4957 | No |
| 76 | UBA52 | na | 9532 | -0.353 | -0.4952 | No |
| 77 | ZDHHC9 | na | 9561 | -0.358 | -0.4928 | No |
| 78 | RPL38 | na | 9661 | -0.369 | -0.4963 | No |
| 79 | RPS24 | na | 9689 | -0.372 | -0.4936 | No |
| 80 | SRP9 | na | 9772 | -0.382 | -0.4954 | No |
| 81 | ARL6IP1 | na | 9852 | -0.393 | -0.4968 | No |
| 82 | VPS37D | na | 9897 | -0.397 | -0.4952 | No |
| 83 | RPLP2 | na | 10048 | -0.421 | -0.5023 | No |
| 84 | RPL37 | na | 10057 | -0.423 | -0.4973 | No |
| 85 | PEX5 | na | 10116 | -0.432 | -0.4964 | No |
| 86 | RPS8 | na | 10245 | -0.452 | -0.5012 | No |
| 87 | RPL27 | na | 10333 | -0.467 | -0.5023 | No |
| 88 | RPS28 | na | 10524 | -0.504 | -0.5116 | Yes |
| 89 | RPL30 | na | 10537 | -0.506 | -0.5059 | Yes |
| 90 | RPL22 | na | 10554 | -0.508 | -0.5005 | Yes |
| 91 | RPL36A | na | 10605 | -0.520 | -0.4977 | Yes |
| 92 | RPL14 | na | 10662 | -0.531 | -0.4954 | Yes |
| 93 | RPL29 | na | 10742 | -0.548 | -0.4948 | Yes |
| 94 | RPL37A | na | 10838 | -0.569 | -0.4952 | Yes |
| 95 | RPS19 | na | 10840 | -0.569 | -0.4877 | Yes |
| 96 | NACAD | na | 10865 | -0.575 | -0.4820 | Yes |
| 97 | RPS12 | na | 10875 | -0.576 | -0.4751 | Yes |
| 98 | RPL24 | na | 10877 | -0.576 | -0.4675 | Yes |
| 99 | ZDHHC18 | na | 10890 | -0.580 | -0.4608 | Yes |
| 100 | RPL34 | na | 10933 | -0.588 | -0.4565 | Yes |
| 101 | RPL23 | na | 10981 | -0.602 | -0.4524 | Yes |
| 102 | RPL35A | na | 10982 | -0.602 | -0.4444 | Yes |
| 103 | ARL6 | na | 10998 | -0.607 | -0.4376 | Yes |
| 104 | RPS25 | na | 11017 | -0.613 | -0.4309 | Yes |
| 105 | RPS7 | na | 11068 | -0.627 | -0.4268 | Yes |
| 106 | RPL23A | na | 11076 | -0.629 | -0.4190 | Yes |
| 107 | RPL5 | na | 11121 | -0.643 | -0.4141 | Yes |
| 108 | RPS16 | na | 11138 | -0.645 | -0.4069 | Yes |
| 109 | NACA | na | 11140 | -0.646 | -0.3983 | Yes |
| 110 | RPL6 | na | 11150 | -0.650 | -0.3904 | Yes |
| 111 | SIL1 | na | 11172 | -0.655 | -0.3835 | Yes |
| 112 | RPL11 | na | 11195 | -0.662 | -0.3765 | Yes |
| 113 | RPS15A | na | 11196 | -0.662 | -0.3677 | Yes |
| 114 | RPLP1 | na | 11249 | -0.682 | -0.3630 | Yes |
| 115 | RPS26 | na | 11254 | -0.686 | -0.3541 | Yes |
| 116 | MIEF2 | na | 11278 | -0.695 | -0.3468 | Yes |
| 117 | RPL35 | na | 11280 | -0.696 | -0.3376 | Yes |
| 118 | ZDHHC21 | na | 11296 | -0.702 | -0.3295 | Yes |
| 119 | RPL31 | na | 11306 | -0.706 | -0.3209 | Yes |
| 120 | RPS21 | na | 11313 | -0.709 | -0.3119 | Yes |
| 121 | RPS3A | na | 11328 | -0.713 | -0.3036 | Yes |
| 122 | RPL21 | na | 11330 | -0.714 | -0.2941 | Yes |
| 123 | RPS10 | na | 11344 | -0.720 | -0.2856 | Yes |
| 124 | RPL32 | na | 11355 | -0.725 | -0.2768 | Yes |
| 125 | RPS11 | na | 11371 | -0.735 | -0.2683 | Yes |
| 126 | RPL12 | na | 11398 | -0.748 | -0.2605 | Yes |
| 127 | RPL7A | na | 11426 | -0.763 | -0.2526 | Yes |
| 128 | RPS15 | na | 11434 | -0.767 | -0.2430 | Yes |
| 129 | RPS27A | na | 11435 | -0.767 | -0.2327 | Yes |
| 130 | RPL15 | na | 11440 | -0.769 | -0.2228 | Yes |
| 131 | RPS29 | na | 11444 | -0.771 | -0.2128 | Yes |
| 132 | RPL18A | na | 11474 | -0.789 | -0.2047 | Yes |
| 133 | RPS14 | na | 11478 | -0.792 | -0.1944 | Yes |
| 134 | RPL7 | na | 11484 | -0.796 | -0.1842 | Yes |
| 135 | RPLP0 | na | 11488 | -0.800 | -0.1738 | Yes |
| 136 | RPL27A | na | 11507 | -0.812 | -0.1645 | Yes |
| 137 | RPL9 | na | 11521 | -0.819 | -0.1547 | Yes |
| 138 | RPS2 | na | 11562 | -0.846 | -0.1468 | Yes |
| 139 | RPL13 | na | 11563 | -0.847 | -0.1355 | Yes |
| 140 | RPS20 | na | 11594 | -0.868 | -0.1264 | Yes |
| 141 | RPS27 | na | 11600 | -0.875 | -0.1152 | Yes |
| 142 | RPL19 | na | 11611 | -0.882 | -0.1043 | Yes |
| 143 | RPS13 | na | 11633 | -0.897 | -0.0941 | Yes |
| 144 | RPS3 | na | 11641 | -0.903 | -0.0827 | Yes |
| 145 | RPS17 | na | 11652 | -0.916 | -0.0713 | Yes |
| 146 | RPL10A | na | 11658 | -0.921 | -0.0594 | Yes |
| 147 | RPL4 | na | 11715 | -0.983 | -0.0510 | Yes |
| 148 | RPL8 | na | 11772 | -1.039 | -0.0419 | Yes |
| 149 | RPS4Y1 | na | 11774 | -1.043 | -0.0281 | Yes |
| 150 | RPL3 | na | 11817 | -1.140 | -0.0164 | Yes |
| 151 | RAB3IP | na | 11854 | -1.191 | -0.0036 | Yes |
| 152 | STOM | na | 11882 | -1.249 | 0.0108 | Yes |
Table: GSEA details [plain text format]

  

Fig 2: GOBP\_PROTEIN\_TARGETING\_TO\_MEMBRANE      
 Blue-Pink O' Gram in the Space of the Analyzed GeneSet

  

Fig 3: GOBP\_PROTEIN\_TARGETING\_TO\_MEMBRANE: Random ES distribution      
 Gene set null distribution of ES for **GOBP\_PROTEIN\_TARGETING\_TO\_MEMBRANE**

  
