# Supplementary material for: Time-series transcriptome analysis identified differentially expressed genes in broiler chicken infected with mixed Eimeria species
Source: Front Genet. 2022 Aug 8;13:886781. doi: 10.3389/fgene.2022.886781 (PMC9393255; doi:10.3389/fgene.2022.886781)
Supplement: Supplementary file 2 [file DataSheet1.ZIP › 4dpi_GO.Gsea.1625071243202/GOBP_REGULATION_OF_ALCOHOL_BIOSYNTHETIC_PROCESS.html]

Details for gene set GOBP\_REGULATION\_OF\_ALCOHOL\_BIOSYNTHETIC\_PROCESS[GSEA]

|  || Dataset | TMM\_4dpi\_gct\_format\_4dpi\_gct\_format.Class\_4dpi.cls #PC\_versus\_NC.Class\_4dpi.cls #PC\_versus\_NC\_repos |
| Phenotype | Class\_4dpi.cls#PC\_versus\_NC\_repos |
| Upregulated in class | 1 |
| GeneSet | GOBP\_REGULATION\_OF\_ALCOHOL\_BIOSYNTHETIC\_PROCESS |
| Enrichment Score (ES) | 0.60460204 |
| Normalized Enrichment Score (NES) | 2.134454 |
| Nominal p-value | 0.0 |
| FDR q-value | 0.0017955774 |
| FWER p-Value | 0.0252 |
Table: GSEA Results Summary

  

Fig 1: Enrichment plot: GOBP\_REGULATION\_OF\_ALCOHOL\_BIOSYNTHETIC\_PROCESS      
 Profile of the Running ES Score & Positions of GeneSet Members on the Rank Ordered List

  

| SYMBOL | TITLE | RANK IN GENE LIST | RANK METRIC SCORE | RUNNING ES | CORE ENRICHMENT || 1 | DHCR7 | na | 17 | 2.286 | 0.0640 | Yes |
| 2 | CYP51A1 | na | 28 | 2.188 | 0.1257 | Yes |
| 3 | HMGCS1 | na | 30 | 2.183 | 0.1880 | Yes |
| 4 | FDFT1 | na | 56 | 1.986 | 0.2427 | Yes |
| 5 | FDPS | na | 71 | 1.792 | 0.2928 | Yes |
| 6 | SQLE | na | 92 | 1.678 | 0.3391 | Yes |
| 7 | LSS | na | 138 | 1.500 | 0.3782 | Yes |
| 8 | APOB | na | 248 | 1.255 | 0.4050 | Yes |
| 9 | HMGCR | na | 299 | 1.179 | 0.4345 | Yes |
| 10 | SREBF2 | na | 333 | 1.135 | 0.4642 | Yes |
| 11 | ACACA | na | 361 | 1.109 | 0.4937 | Yes |
| 12 | SC5D | na | 418 | 1.048 | 0.5189 | Yes |
| 13 | SREBF1 | na | 691 | 0.823 | 0.5197 | Yes |
| 14 | PTK2B | na | 713 | 0.810 | 0.5411 | Yes |
| 15 | FGF1 | na | 971 | 0.684 | 0.5392 | Yes |
| 16 | SCD | na | 1084 | 0.644 | 0.5482 | Yes |
| 17 | MBTPS2 | na | 1196 | 0.605 | 0.5562 | Yes |
| 18 | DGKQ | na | 1245 | 0.590 | 0.5691 | Yes |
| 19 | BMP2 | na | 1379 | 0.553 | 0.5738 | Yes |
| 20 | PRKAA1 | na | 1419 | 0.542 | 0.5860 | Yes |
| 21 | DKK3 | na | 1533 | 0.512 | 0.5912 | Yes |
| 22 | SP1 | na | 1630 | 0.488 | 0.5971 | Yes |
| 23 | MVD | na | 1777 | 0.461 | 0.5981 | Yes |
| 24 | LPCAT3 | na | 1853 | 0.447 | 0.6046 | Yes |
| 25 | GPAM | na | 2506 | 0.348 | 0.5600 | No |
| 26 | ELOVL6 | na | 2559 | 0.341 | 0.5654 | No |
| 27 | P2RY1 | na | 2802 | 0.309 | 0.5540 | No |
| 28 | H6PD | na | 3109 | 0.268 | 0.5360 | No |
| 29 | NFYA | na | 3482 | 0.223 | 0.5113 | No |
| 30 | KPNB1 | na | 3483 | 0.223 | 0.5177 | No |
| 31 | ERLIN1 | na | 3789 | 0.189 | 0.4975 | No |
| 32 | BMP6 | na | 3816 | 0.186 | 0.5007 | No |
| 33 | GFI1 | na | 4541 | 0.121 | 0.4436 | No |
| 34 | CD244 | na | 5031 | 0.076 | 0.4049 | No |
| 35 | RAN | na | 5091 | 0.071 | 0.4020 | No |
| 36 | FASN | na | 5288 | 0.055 | 0.3871 | No |
| 37 | P2RY6 | na | 6669 | -0.060 | 0.2734 | No |
| 38 | NFKB1 | na | 6765 | -0.068 | 0.2674 | No |
| 39 | MVK | na | 6801 | -0.071 | 0.2665 | No |
| 40 | MBTPS1 | na | 6903 | -0.080 | 0.2603 | No |
| 41 | BMP5 | na | 7077 | -0.096 | 0.2486 | No |
| 42 | PMVK | na | 7240 | -0.112 | 0.2383 | No |
| 43 | GGPS1 | na | 7444 | -0.131 | 0.2250 | No |
| 44 | NFYC | na | 7623 | -0.146 | 0.2143 | No |
| 45 | ERLIN2 | na | 7983 | -0.178 | 0.1893 | No |
| 46 | SEC14L2 | na | 8060 | -0.186 | 0.1883 | No |
| 47 | PTAFR | na | 8527 | -0.232 | 0.1560 | No |
| 48 | WNT4 | na | 8711 | -0.252 | 0.1478 | No |
| 49 | CLCN2 | na | 9370 | -0.331 | 0.1023 | No |
| 50 | PLEK | na | 10092 | -0.428 | 0.0542 | No |
| 51 | SOD1 | na | 10118 | -0.432 | 0.0645 | No |
| 52 | HRH1 | na | 10858 | -0.575 | 0.0191 | No |
| 53 | ABCG1 | na | 11046 | -0.620 | 0.0212 | No |
| 54 | SCAP | na | 11086 | -0.632 | 0.0360 | No |
| 55 | SNCA | na | 11241 | -0.679 | 0.0425 | No |
| 56 | NTSR1 | na | 11415 | -0.759 | 0.0498 | No |
Table: GSEA details [plain text format]

  

Fig 2: GOBP\_REGULATION\_OF\_ALCOHOL\_BIOSYNTHETIC\_PROCESS      
 Blue-Pink O' Gram in the Space of the Analyzed GeneSet

  

Fig 3: GOBP\_REGULATION\_OF\_ALCOHOL\_BIOSYNTHETIC\_PROCESS: Random ES distribution      
 Gene set null distribution of ES for **GOBP\_REGULATION\_OF\_ALCOHOL\_BIOSYNTHETIC\_PROCESS**

  
