# Supplementary material for: Time-series transcriptome analysis identified differentially expressed genes in broiler chicken infected with mixed Eimeria species
Source: Front Genet. 2022 Aug 8;13:886781. doi: 10.3389/fgene.2022.886781 (PMC9393255; doi:10.3389/fgene.2022.886781)
Supplement: Supplementary file 2 [file DataSheet1.ZIP › 4dpi_GO.Gsea.1625071243202/GOBP_REGULATION_OF_CHOLESTEROL_BIOSYNTHETIC_PROCESS.html]

Details for gene set GOBP\_REGULATION\_OF\_CHOLESTEROL\_BIOSYNTHETIC\_PROCESS[GSEA]

|  || Dataset | TMM\_4dpi\_gct\_format\_4dpi\_gct\_format.Class\_4dpi.cls #PC\_versus\_NC.Class\_4dpi.cls #PC\_versus\_NC\_repos |
| Phenotype | Class\_4dpi.cls#PC\_versus\_NC\_repos |
| Upregulated in class | 1 |
| GeneSet | GOBP\_REGULATION\_OF\_CHOLESTEROL\_BIOSYNTHETIC\_PROCESS |
| Enrichment Score (ES) | 0.70309556 |
| Normalized Enrichment Score (NES) | 2.3124368 |
| Nominal p-value | 0.0 |
| FDR q-value | 1.0636127E-4 |
| FWER p-Value | 2.0E-4 |
Table: GSEA Results Summary

  

Fig 1: Enrichment plot: GOBP\_REGULATION\_OF\_CHOLESTEROL\_BIOSYNTHETIC\_PROCESS      
 Profile of the Running ES Score & Positions of GeneSet Members on the Rank Ordered List

  

| SYMBOL | TITLE | RANK IN GENE LIST | RANK METRIC SCORE | RUNNING ES | CORE ENRICHMENT || 1 | DHCR7 | na | 17 | 2.286 | 0.0800 | Yes |
| 2 | CYP51A1 | na | 28 | 2.188 | 0.1572 | Yes |
| 3 | HMGCS1 | na | 30 | 2.183 | 0.2349 | Yes |
| 4 | FDFT1 | na | 56 | 1.986 | 0.3035 | Yes |
| 5 | FDPS | na | 71 | 1.792 | 0.3662 | Yes |
| 6 | SQLE | na | 92 | 1.678 | 0.4243 | Yes |
| 7 | LSS | na | 138 | 1.500 | 0.4740 | Yes |
| 8 | APOB | na | 248 | 1.255 | 0.5096 | Yes |
| 9 | HMGCR | na | 299 | 1.179 | 0.5474 | Yes |
| 10 | SREBF2 | na | 333 | 1.135 | 0.5851 | Yes |
| 11 | ACACA | na | 361 | 1.109 | 0.6224 | Yes |
| 12 | SC5D | na | 418 | 1.048 | 0.6550 | Yes |
| 13 | SREBF1 | na | 691 | 0.823 | 0.6616 | Yes |
| 14 | FGF1 | na | 971 | 0.684 | 0.6627 | Yes |
| 15 | SCD | na | 1084 | 0.644 | 0.6763 | Yes |
| 16 | MBTPS2 | na | 1196 | 0.605 | 0.6886 | Yes |
| 17 | PRKAA1 | na | 1419 | 0.542 | 0.6893 | Yes |
| 18 | SP1 | na | 1630 | 0.488 | 0.6892 | Yes |
| 19 | MVD | na | 1777 | 0.461 | 0.6934 | Yes |
| 20 | LPCAT3 | na | 1853 | 0.447 | 0.7031 | Yes |
| 21 | GPAM | na | 2506 | 0.348 | 0.6610 | No |
| 22 | ELOVL6 | na | 2559 | 0.341 | 0.6688 | No |
| 23 | NFYA | na | 3482 | 0.223 | 0.5998 | No |
| 24 | KPNB1 | na | 3483 | 0.223 | 0.6077 | No |
| 25 | ERLIN1 | na | 3789 | 0.189 | 0.5889 | No |
| 26 | RAN | na | 5091 | 0.071 | 0.4828 | No |
| 27 | FASN | na | 5288 | 0.055 | 0.4684 | No |
| 28 | MVK | na | 6801 | -0.071 | 0.3446 | No |
| 29 | MBTPS1 | na | 6903 | -0.080 | 0.3390 | No |
| 30 | PMVK | na | 7240 | -0.112 | 0.3150 | No |
| 31 | GGPS1 | na | 7444 | -0.131 | 0.3027 | No |
| 32 | NFYC | na | 7623 | -0.146 | 0.2930 | No |
| 33 | ERLIN2 | na | 7983 | -0.178 | 0.2694 | No |
| 34 | SEC14L2 | na | 8060 | -0.186 | 0.2696 | No |
| 35 | SOD1 | na | 10118 | -0.432 | 0.1132 | No |
| 36 | ABCG1 | na | 11046 | -0.620 | 0.0579 | No |
| 37 | SCAP | na | 11086 | -0.632 | 0.0772 | No |
Table: GSEA details [plain text format]

  

Fig 2: GOBP\_REGULATION\_OF\_CHOLESTEROL\_BIOSYNTHETIC\_PROCESS      
 Blue-Pink O' Gram in the Space of the Analyzed GeneSet

  

Fig 3: GOBP\_REGULATION\_OF\_CHOLESTEROL\_BIOSYNTHETIC\_PROCESS: Random ES distribution      
 Gene set null distribution of ES for **GOBP\_REGULATION\_OF\_CHOLESTEROL\_BIOSYNTHETIC\_PROCESS**

  
