# Supplementary material for: Time-series transcriptome analysis identified differentially expressed genes in broiler chicken infected with mixed Eimeria species
Source: Front Genet. 2022 Aug 8;13:886781. doi: 10.3389/fgene.2022.886781 (PMC9393255; doi:10.3389/fgene.2022.886781)
Supplement: Supplementary file 2 [file DataSheet1.ZIP › 4dpi_GO.Gsea.1625071243202/GOBP_REGULATION_OF_CHOLESTEROL_METABOLIC_PROCESS.html]

Details for gene set GOBP\_REGULATION\_OF\_CHOLESTEROL\_METABOLIC\_PROCESS[GSEA]

|  || Dataset | TMM\_4dpi\_gct\_format\_4dpi\_gct\_format.Class\_4dpi.cls #PC\_versus\_NC.Class\_4dpi.cls #PC\_versus\_NC\_repos |
| Phenotype | Class\_4dpi.cls#PC\_versus\_NC\_repos |
| Upregulated in class | 1 |
| GeneSet | GOBP\_REGULATION\_OF\_CHOLESTEROL\_METABOLIC\_PROCESS |
| Enrichment Score (ES) | 0.68178964 |
| Normalized Enrichment Score (NES) | 2.310491 |
| Nominal p-value | 0.0 |
| FDR q-value | 7.090751E-5 |
| FWER p-Value | 2.0E-4 |
Table: GSEA Results Summary

  

Fig 1: Enrichment plot: GOBP\_REGULATION\_OF\_CHOLESTEROL\_METABOLIC\_PROCESS      
 Profile of the Running ES Score & Positions of GeneSet Members on the Rank Ordered List

  

| SYMBOL | TITLE | RANK IN GENE LIST | RANK METRIC SCORE | RUNNING ES | CORE ENRICHMENT || 1 | DHCR7 | na | 17 | 2.286 | 0.0721 | Yes |
| 2 | CYP51A1 | na | 28 | 2.188 | 0.1415 | Yes |
| 3 | HMGCS1 | na | 30 | 2.183 | 0.2116 | Yes |
| 4 | FDFT1 | na | 56 | 1.986 | 0.2733 | Yes |
| 5 | FDPS | na | 71 | 1.792 | 0.3298 | Yes |
| 6 | SQLE | na | 92 | 1.678 | 0.3820 | Yes |
| 7 | LSS | na | 138 | 1.500 | 0.4264 | Yes |
| 8 | APOB | na | 248 | 1.255 | 0.4577 | Yes |
| 9 | HMGCR | na | 299 | 1.179 | 0.4914 | Yes |
| 10 | SREBF2 | na | 333 | 1.135 | 0.5251 | Yes |
| 11 | ACACA | na | 361 | 1.109 | 0.5585 | Yes |
| 12 | SC5D | na | 418 | 1.048 | 0.5875 | Yes |
| 13 | DGAT2 | na | 643 | 0.854 | 0.5962 | Yes |
| 14 | SREBF1 | na | 691 | 0.823 | 0.6187 | Yes |
| 15 | FGF1 | na | 971 | 0.684 | 0.6174 | Yes |
| 16 | SCD | na | 1084 | 0.644 | 0.6287 | Yes |
| 17 | MBTPS2 | na | 1196 | 0.605 | 0.6389 | Yes |
| 18 | DGKQ | na | 1245 | 0.590 | 0.6538 | Yes |
| 19 | PRKAA1 | na | 1419 | 0.542 | 0.6567 | Yes |
| 20 | TTC39B | na | 1575 | 0.502 | 0.6599 | Yes |
| 21 | SP1 | na | 1630 | 0.488 | 0.6711 | Yes |
| 22 | MVD | na | 1777 | 0.461 | 0.6737 | Yes |
| 23 | LPCAT3 | na | 1853 | 0.447 | 0.6818 | Yes |
| 24 | GPAM | na | 2506 | 0.348 | 0.6385 | No |
| 25 | ELOVL6 | na | 2559 | 0.341 | 0.6451 | No |
| 26 | ARV1 | na | 2818 | 0.307 | 0.6334 | No |
| 27 | NFYA | na | 3482 | 0.223 | 0.5851 | No |
| 28 | KPNB1 | na | 3483 | 0.223 | 0.5923 | No |
| 29 | ERLIN1 | na | 3789 | 0.189 | 0.5729 | No |
| 30 | RAN | na | 5091 | 0.071 | 0.4664 | No |
| 31 | FASN | na | 5288 | 0.055 | 0.4518 | No |
| 32 | EPHX2 | na | 5461 | 0.040 | 0.4387 | No |
| 33 | LDLRAP1 | na | 5933 | -0.002 | 0.3994 | No |
| 34 | MVK | na | 6801 | -0.071 | 0.3292 | No |
| 35 | MBTPS1 | na | 6903 | -0.080 | 0.3233 | No |
| 36 | PMVK | na | 7240 | -0.112 | 0.2989 | No |
| 37 | GGPS1 | na | 7444 | -0.131 | 0.2861 | No |
| 38 | NFYC | na | 7623 | -0.146 | 0.2759 | No |
| 39 | NR1H4 | na | 7657 | -0.148 | 0.2779 | No |
| 40 | ERLIN2 | na | 7983 | -0.178 | 0.2565 | No |
| 41 | SEC14L2 | na | 8060 | -0.186 | 0.2561 | No |
| 42 | SOD1 | na | 10118 | -0.432 | 0.0981 | No |
| 43 | ACADL | na | 11004 | -0.609 | 0.0437 | No |
| 44 | ABCG1 | na | 11046 | -0.620 | 0.0602 | No |
| 45 | SCAP | na | 11086 | -0.632 | 0.0772 | No |
Table: GSEA details [plain text format]

  

Fig 2: GOBP\_REGULATION\_OF\_CHOLESTEROL\_METABOLIC\_PROCESS      
 Blue-Pink O' Gram in the Space of the Analyzed GeneSet

  

Fig 3: GOBP\_REGULATION\_OF\_CHOLESTEROL\_METABOLIC\_PROCESS: Random ES distribution      
 Gene set null distribution of ES for **GOBP\_REGULATION\_OF\_CHOLESTEROL\_METABOLIC\_PROCESS**

  
