# Supplementary material for: Time-series transcriptome analysis identified differentially expressed genes in broiler chicken infected with mixed Eimeria species
Source: Front Genet. 2022 Aug 8;13:886781. doi: 10.3389/fgene.2022.886781 (PMC9393255; doi:10.3389/fgene.2022.886781)
Supplement: Supplementary file 2 [file DataSheet1.ZIP › 4dpi_GO.Gsea.1625071243202/GOBP_REGULATION_OF_LIPID_BIOSYNTHETIC_PROCESS.html]

Details for gene set GOBP\_REGULATION\_OF\_LIPID\_BIOSYNTHETIC\_PROCESS[GSEA]

|  || Dataset | TMM\_4dpi\_gct\_format\_4dpi\_gct\_format.Class\_4dpi.cls #PC\_versus\_NC.Class\_4dpi.cls #PC\_versus\_NC\_repos |
| Phenotype | Class\_4dpi.cls#PC\_versus\_NC\_repos |
| Upregulated in class | 1 |
| GeneSet | GOBP\_REGULATION\_OF\_LIPID\_BIOSYNTHETIC\_PROCESS |
| Enrichment Score (ES) | 0.51398516 |
| Normalized Enrichment Score (NES) | 2.1189609 |
| Nominal p-value | 0.0 |
| FDR q-value | 0.0021217335 |
| FWER p-Value | 0.0356 |
Table: GSEA Results Summary

  

Fig 1: Enrichment plot: GOBP\_REGULATION\_OF\_LIPID\_BIOSYNTHETIC\_PROCESS      
 Profile of the Running ES Score & Positions of GeneSet Members on the Rank Ordered List

  

| SYMBOL | TITLE | RANK IN GENE LIST | RANK METRIC SCORE | RUNNING ES | CORE ENRICHMENT || 1 | DHCR7 | na | 17 | 2.286 | 0.0291 | Yes |
| 2 | CYP51A1 | na | 28 | 2.188 | 0.0575 | Yes |
| 3 | HMGCS1 | na | 30 | 2.183 | 0.0866 | Yes |
| 4 | FDFT1 | na | 56 | 1.986 | 0.1110 | Yes |
| 5 | PDK4 | na | 58 | 1.978 | 0.1374 | Yes |
| 6 | FDPS | na | 71 | 1.792 | 0.1603 | Yes |
| 7 | SQLE | na | 92 | 1.678 | 0.1810 | Yes |
| 8 | LSS | na | 138 | 1.500 | 0.1973 | Yes |
| 9 | INSIG1 | na | 148 | 1.471 | 0.2162 | Yes |
| 10 | LDLR | na | 159 | 1.437 | 0.2345 | Yes |
| 11 | APOB | na | 248 | 1.255 | 0.2439 | Yes |
| 12 | HMGCR | na | 299 | 1.179 | 0.2554 | Yes |
| 13 | SREBF2 | na | 333 | 1.135 | 0.2678 | Yes |
| 14 | MALRD1 | na | 336 | 1.133 | 0.2828 | Yes |
| 15 | ACACA | na | 361 | 1.109 | 0.2956 | Yes |
| 16 | MFSD2A | na | 373 | 1.098 | 0.3093 | Yes |
| 17 | SC5D | na | 418 | 1.048 | 0.3196 | Yes |
| 18 | PCK1 | na | 446 | 1.020 | 0.3310 | Yes |
| 19 | HTR2A | na | 452 | 1.014 | 0.3441 | Yes |
| 20 | LPGAT1 | na | 479 | 0.986 | 0.3551 | Yes |
| 21 | FABP5 | na | 504 | 0.960 | 0.3659 | Yes |
| 22 | APOA1 | na | 589 | 0.889 | 0.3707 | Yes |
| 23 | SAMD8 | na | 591 | 0.888 | 0.3825 | Yes |
| 24 | PRKCD | na | 605 | 0.875 | 0.3931 | Yes |
| 25 | SMPD3 | na | 626 | 0.863 | 0.4030 | Yes |
| 26 | DGAT2 | na | 643 | 0.854 | 0.4130 | Yes |
| 27 | SREBF1 | na | 691 | 0.823 | 0.4201 | Yes |
| 28 | STARD4 | na | 710 | 0.810 | 0.4294 | Yes |
| 29 | APOA4 | na | 733 | 0.797 | 0.4382 | Yes |
| 30 | FGF1 | na | 971 | 0.684 | 0.4273 | Yes |
| 31 | ADIPOR2 | na | 989 | 0.676 | 0.4349 | Yes |
| 32 | ANXA1 | na | 1060 | 0.651 | 0.4377 | Yes |
| 33 | SCD | na | 1084 | 0.644 | 0.4444 | Yes |
| 34 | ABHD6 | na | 1089 | 0.642 | 0.4526 | Yes |
| 35 | FABP3 | na | 1105 | 0.636 | 0.4599 | Yes |
| 36 | MBTPS2 | na | 1196 | 0.605 | 0.4604 | Yes |
| 37 | MLXIPL | na | 1199 | 0.604 | 0.4683 | Yes |
| 38 | PPARA | na | 1223 | 0.596 | 0.4743 | Yes |
| 39 | DGKQ | na | 1245 | 0.590 | 0.4804 | Yes |
| 40 | FGF19 | na | 1264 | 0.585 | 0.4867 | Yes |
| 41 | SNAI2 | na | 1276 | 0.583 | 0.4936 | Yes |
| 42 | BMP2 | na | 1379 | 0.553 | 0.4924 | Yes |
| 43 | ACSL3 | na | 1396 | 0.548 | 0.4984 | Yes |
| 44 | SORBS1 | na | 1415 | 0.543 | 0.5041 | Yes |
| 45 | PRKAA1 | na | 1419 | 0.542 | 0.5111 | Yes |
| 46 | DKK3 | na | 1533 | 0.512 | 0.5084 | Yes |
| 47 | BRCA1 | na | 1559 | 0.505 | 0.5130 | Yes |
| 48 | SP1 | na | 1630 | 0.488 | 0.5137 | Yes |
| 49 | THRSP | na | 1759 | 0.464 | 0.5091 | Yes |
| 50 | MVD | na | 1777 | 0.461 | 0.5138 | Yes |
| 51 | LPCAT3 | na | 1853 | 0.447 | 0.5135 | Yes |
| 52 | CAPN2 | na | 1917 | 0.437 | 0.5140 | Yes |
| 53 | ORMDL1 | na | 2060 | 0.411 | 0.5075 | No |
| 54 | GPAM | na | 2506 | 0.348 | 0.4747 | No |
| 55 | ELOVL6 | na | 2559 | 0.341 | 0.4748 | No |
| 56 | ADIPOQ | na | 2680 | 0.326 | 0.4691 | No |
| 57 | ATP1A1 | na | 3009 | 0.281 | 0.4452 | No |
| 58 | ZBTB20 | na | 3022 | 0.279 | 0.4479 | No |
| 59 | H6PD | na | 3109 | 0.268 | 0.4442 | No |
| 60 | NR1H3 | na | 3213 | 0.255 | 0.4390 | No |
| 61 | SIRT4 | na | 3355 | 0.237 | 0.4302 | No |
| 62 | MID1IP1 | na | 3422 | 0.230 | 0.4278 | No |
| 63 | NFYA | na | 3482 | 0.223 | 0.4258 | No |
| 64 | KPNB1 | na | 3483 | 0.223 | 0.4287 | No |
| 65 | PLIN5 | na | 3601 | 0.207 | 0.4216 | No |
| 66 | ERLIN1 | na | 3789 | 0.189 | 0.4084 | No |
| 67 | BMP6 | na | 3816 | 0.186 | 0.4087 | No |
| 68 | SIRT3 | na | 4105 | 0.159 | 0.3866 | No |
| 69 | CNEP1R1 | na | 4163 | 0.154 | 0.3838 | No |
| 70 | WDTC1 | na | 4181 | 0.152 | 0.3844 | No |
| 71 | ASXL3 | na | 4213 | 0.150 | 0.3838 | No |
| 72 | MTOR | na | 4329 | 0.140 | 0.3760 | No |
| 73 | DHH | na | 4419 | 0.132 | 0.3702 | No |
| 74 | CREBL2 | na | 4444 | 0.129 | 0.3699 | No |
| 75 | GFI1 | na | 4541 | 0.121 | 0.3635 | No |
| 76 | ABCD2 | na | 4562 | 0.119 | 0.3634 | No |
| 77 | ENHO | na | 4668 | 0.109 | 0.3560 | No |
| 78 | IGFBP7 | na | 4706 | 0.106 | 0.3543 | No |
| 79 | PRKAA2 | na | 4804 | 0.097 | 0.3474 | No |
| 80 | ADGRF5 | na | 4842 | 0.094 | 0.3455 | No |
| 81 | PDGFB | na | 5083 | 0.072 | 0.3263 | No |
| 82 | RAN | na | 5091 | 0.071 | 0.3266 | No |
| 83 | PRKAG2 | na | 5102 | 0.070 | 0.3267 | No |
| 84 | PDGFA | na | 5143 | 0.066 | 0.3242 | No |
| 85 | FASN | na | 5288 | 0.055 | 0.3128 | No |
| 86 | AKT1 | na | 5330 | 0.052 | 0.3101 | No |
| 87 | ELOVL5 | na | 5559 | 0.030 | 0.2912 | No |
| 88 | IDH1 | na | 5624 | 0.024 | 0.2862 | No |
| 89 | SCARB1 | na | 5664 | 0.020 | 0.2832 | No |
| 90 | PIBF1 | na | 6178 | -0.020 | 0.2402 | No |
| 91 | SMPD2 | na | 6421 | -0.040 | 0.2203 | No |
| 92 | CREB1 | na | 6598 | -0.054 | 0.2062 | No |
| 93 | NFKB1 | na | 6765 | -0.068 | 0.1931 | No |
| 94 | MVK | na | 6801 | -0.071 | 0.1911 | No |
| 95 | LPCAT1 | na | 6902 | -0.079 | 0.1838 | No |
| 96 | MBTPS1 | na | 6903 | -0.080 | 0.1848 | No |
| 97 | CHP1 | na | 6932 | -0.083 | 0.1836 | No |
| 98 | BMP5 | na | 7077 | -0.096 | 0.1727 | No |
| 99 | PROX1 | na | 7123 | -0.100 | 0.1703 | No |
| 100 | PMVK | na | 7240 | -0.112 | 0.1620 | No |
| 101 | SNAI1 | na | 7275 | -0.116 | 0.1607 | No |
| 102 | ORMDL2 | na | 7384 | -0.125 | 0.1533 | No |
| 103 | GGPS1 | na | 7444 | -0.131 | 0.1500 | No |
| 104 | AVPR1A | na | 7446 | -0.131 | 0.1517 | No |
| 105 | EIF6 | na | 7476 | -0.133 | 0.1510 | No |
| 106 | SPHK1 | na | 7479 | -0.134 | 0.1527 | No |
| 107 | EGR1 | na | 7575 | -0.141 | 0.1465 | No |
| 108 | NFYC | na | 7623 | -0.146 | 0.1445 | No |
| 109 | TNFRSF1A | na | 7639 | -0.147 | 0.1452 | No |
| 110 | ASAH1 | na | 7648 | -0.148 | 0.1465 | No |
| 111 | NR1H4 | na | 7657 | -0.148 | 0.1478 | No |
| 112 | ERLIN2 | na | 7983 | -0.178 | 0.1228 | No |
| 113 | SEC14L2 | na | 8060 | -0.186 | 0.1189 | No |
| 114 | ABCA2 | na | 8565 | -0.237 | 0.0796 | No |
| 115 | NR0B1 | na | 8635 | -0.243 | 0.0770 | No |
| 116 | WNT4 | na | 8711 | -0.252 | 0.0741 | No |
| 117 | PTGS2 | na | 8720 | -0.253 | 0.0768 | No |
| 118 | FBXW7 | na | 8851 | -0.268 | 0.0694 | No |
| 119 | TSPO | na | 8903 | -0.274 | 0.0688 | No |
| 120 | C3 | na | 8977 | -0.281 | 0.0664 | No |
| 121 | CD74 | na | 9026 | -0.286 | 0.0661 | No |
| 122 | ABCA3 | na | 9099 | -0.295 | 0.0640 | No |
| 123 | PRKAB2 | na | 9322 | -0.325 | 0.0496 | No |
| 124 | FGFR4 | na | 9361 | -0.330 | 0.0508 | No |
| 125 | CLCN2 | na | 9370 | -0.331 | 0.0546 | No |
| 126 | NSMAF | na | 9457 | -0.342 | 0.0519 | No |
| 127 | ADIPOR1 | na | 9523 | -0.352 | 0.0511 | No |
| 128 | SIRT1 | na | 9868 | -0.394 | 0.0274 | No |
| 129 | SOD1 | na | 10118 | -0.432 | 0.0122 | No |
| 130 | ORMDL3 | na | 10175 | -0.441 | 0.0134 | No |
| 131 | NR1D1 | na | 10243 | -0.451 | 0.0138 | No |
| 132 | SIK1 | na | 10602 | -0.519 | -0.0095 | No |
| 133 | SLC45A3 | na | 10760 | -0.550 | -0.0153 | No |
| 134 | ACADL | na | 11004 | -0.609 | -0.0277 | No |
| 135 | IL1B | na | 11033 | -0.617 | -0.0218 | No |
| 136 | ABCG1 | na | 11046 | -0.620 | -0.0145 | No |
| 137 | SCAP | na | 11086 | -0.632 | -0.0093 | No |
| 138 | PPARGC1A | na | 11134 | -0.645 | -0.0047 | No |
| 139 | RDH10 | na | 11297 | -0.703 | -0.0090 | No |
| 140 | RACK1 | na | 11554 | -0.842 | -0.0193 | No |
| 141 | RGN | na | 11564 | -0.848 | -0.0087 | No |
| 142 | SLC27A1 | na | 11586 | -0.861 | 0.0010 | No |
| 143 | ADM | na | 11649 | -0.915 | 0.0080 | No |
| 144 | HTR2B | na | 11963 | -1.669 | 0.0040 | No |
Table: GSEA details [plain text format]

  

Fig 2: GOBP\_REGULATION\_OF\_LIPID\_BIOSYNTHETIC\_PROCESS      
 Blue-Pink O' Gram in the Space of the Analyzed GeneSet

  

Fig 3: GOBP\_REGULATION\_OF\_LIPID\_BIOSYNTHETIC\_PROCESS: Random ES distribution      
 Gene set null distribution of ES for **GOBP\_REGULATION\_OF\_LIPID\_BIOSYNTHETIC\_PROCESS**

  
