# Supplementary material for: Time-series transcriptome analysis identified differentially expressed genes in broiler chicken infected with mixed Eimeria species
Source: Front Genet. 2022 Aug 8;13:886781. doi: 10.3389/fgene.2022.886781 (PMC9393255; doi:10.3389/fgene.2022.886781)
Supplement: Supplementary file 2 [file DataSheet1.ZIP › 4dpi_GO.Gsea.1625071243202/GOBP_REGULATION_OF_STEROID_BIOSYNTHETIC_PROCESS.html]

Details for gene set GOBP\_REGULATION\_OF\_STEROID\_BIOSYNTHETIC\_PROCESS[GSEA]

|  || Dataset | TMM\_4dpi\_gct\_format\_4dpi\_gct\_format.Class\_4dpi.cls #PC\_versus\_NC.Class\_4dpi.cls #PC\_versus\_NC\_repos |
| Phenotype | Class\_4dpi.cls#PC\_versus\_NC\_repos |
| Upregulated in class | 1 |
| GeneSet | GOBP\_REGULATION\_OF\_STEROID\_BIOSYNTHETIC\_PROCESS |
| Enrichment Score (ES) | 0.6029234 |
| Normalized Enrichment Score (NES) | 2.204267 |
| Nominal p-value | 0.0 |
| FDR q-value | 3.745738E-4 |
| FWER p-Value | 0.0032 |
Table: GSEA Results Summary

  

Fig 1: Enrichment plot: GOBP\_REGULATION\_OF\_STEROID\_BIOSYNTHETIC\_PROCESS      
 Profile of the Running ES Score & Positions of GeneSet Members on the Rank Ordered List

  

| SYMBOL | TITLE | RANK IN GENE LIST | RANK METRIC SCORE | RUNNING ES | CORE ENRICHMENT || 1 | DHCR7 | na | 17 | 2.286 | 0.0557 | Yes |
| 2 | CYP51A1 | na | 28 | 2.188 | 0.1095 | Yes |
| 3 | HMGCS1 | na | 30 | 2.183 | 0.1639 | Yes |
| 4 | FDFT1 | na | 56 | 1.986 | 0.2114 | Yes |
| 5 | FDPS | na | 71 | 1.792 | 0.2550 | Yes |
| 6 | SQLE | na | 92 | 1.678 | 0.2952 | Yes |
| 7 | LSS | na | 138 | 1.500 | 0.3289 | Yes |
| 8 | INSIG1 | na | 148 | 1.471 | 0.3648 | Yes |
| 9 | APOB | na | 248 | 1.255 | 0.3879 | Yes |
| 10 | HMGCR | na | 299 | 1.179 | 0.4131 | Yes |
| 11 | SREBF2 | na | 333 | 1.135 | 0.4387 | Yes |
| 12 | MALRD1 | na | 336 | 1.133 | 0.4668 | Yes |
| 13 | ACACA | na | 361 | 1.109 | 0.4925 | Yes |
| 14 | SC5D | na | 418 | 1.048 | 0.5140 | Yes |
| 15 | SREBF1 | na | 691 | 0.823 | 0.5118 | Yes |
| 16 | STARD4 | na | 710 | 0.810 | 0.5305 | Yes |
| 17 | FGF1 | na | 971 | 0.684 | 0.5258 | Yes |
| 18 | SCD | na | 1084 | 0.644 | 0.5325 | Yes |
| 19 | MBTPS2 | na | 1196 | 0.605 | 0.5383 | Yes |
| 20 | DGKQ | na | 1245 | 0.590 | 0.5490 | Yes |
| 21 | FGF19 | na | 1264 | 0.585 | 0.5621 | Yes |
| 22 | SNAI2 | na | 1276 | 0.583 | 0.5757 | Yes |
| 23 | BMP2 | na | 1379 | 0.553 | 0.5810 | Yes |
| 24 | PRKAA1 | na | 1419 | 0.542 | 0.5913 | Yes |
| 25 | DKK3 | na | 1533 | 0.512 | 0.5946 | Yes |
| 26 | SP1 | na | 1630 | 0.488 | 0.5988 | Yes |
| 27 | MVD | na | 1777 | 0.461 | 0.5980 | Yes |
| 28 | LPCAT3 | na | 1853 | 0.447 | 0.6029 | Yes |
| 29 | GPAM | na | 2506 | 0.348 | 0.5570 | No |
| 30 | ELOVL6 | na | 2559 | 0.341 | 0.5612 | No |
| 31 | ATP1A1 | na | 3009 | 0.281 | 0.5306 | No |
| 32 | H6PD | na | 3109 | 0.268 | 0.5290 | No |
| 33 | NFYA | na | 3482 | 0.223 | 0.5034 | No |
| 34 | KPNB1 | na | 3483 | 0.223 | 0.5090 | No |
| 35 | ERLIN1 | na | 3789 | 0.189 | 0.4881 | No |
| 36 | BMP6 | na | 3816 | 0.186 | 0.4906 | No |
| 37 | DHH | na | 4419 | 0.132 | 0.4435 | No |
| 38 | GFI1 | na | 4541 | 0.121 | 0.4364 | No |
| 39 | IGFBP7 | na | 4706 | 0.106 | 0.4253 | No |
| 40 | RAN | na | 5091 | 0.071 | 0.3949 | No |
| 41 | FASN | na | 5288 | 0.055 | 0.3799 | No |
| 42 | NFKB1 | na | 6765 | -0.068 | 0.2580 | No |
| 43 | MVK | na | 6801 | -0.071 | 0.2568 | No |
| 44 | MBTPS1 | na | 6903 | -0.080 | 0.2503 | No |
| 45 | BMP5 | na | 7077 | -0.096 | 0.2383 | No |
| 46 | PROX1 | na | 7123 | -0.100 | 0.2370 | No |
| 47 | PMVK | na | 7240 | -0.112 | 0.2301 | No |
| 48 | SNAI1 | na | 7275 | -0.116 | 0.2301 | No |
| 49 | GGPS1 | na | 7444 | -0.131 | 0.2193 | No |
| 50 | EGR1 | na | 7575 | -0.141 | 0.2120 | No |
| 51 | NFYC | na | 7623 | -0.146 | 0.2117 | No |
| 52 | ASAH1 | na | 7648 | -0.148 | 0.2134 | No |
| 53 | NR1H4 | na | 7657 | -0.148 | 0.2164 | No |
| 54 | ERLIN2 | na | 7983 | -0.178 | 0.1936 | No |
| 55 | SEC14L2 | na | 8060 | -0.186 | 0.1919 | No |
| 56 | NR0B1 | na | 8635 | -0.243 | 0.1499 | No |
| 57 | WNT4 | na | 8711 | -0.252 | 0.1499 | No |
| 58 | TSPO | na | 8903 | -0.274 | 0.1408 | No |
| 59 | FGFR4 | na | 9361 | -0.330 | 0.1107 | No |
| 60 | CLCN2 | na | 9370 | -0.331 | 0.1183 | No |
| 61 | SIRT1 | na | 9868 | -0.394 | 0.0866 | No |
| 62 | SOD1 | na | 10118 | -0.432 | 0.0765 | No |
| 63 | NR1D1 | na | 10243 | -0.451 | 0.0774 | No |
| 64 | ABCG1 | na | 11046 | -0.620 | 0.0258 | No |
| 65 | SCAP | na | 11086 | -0.632 | 0.0383 | No |
| 66 | PPARGC1A | na | 11134 | -0.645 | 0.0504 | No |
| 67 | ADM | na | 11649 | -0.915 | 0.0302 | No |
Table: GSEA details [plain text format]

  

Fig 2: GOBP\_REGULATION\_OF\_STEROID\_BIOSYNTHETIC\_PROCESS      
 Blue-Pink O' Gram in the Space of the Analyzed GeneSet

  

Fig 3: GOBP\_REGULATION\_OF\_STEROID\_BIOSYNTHETIC\_PROCESS: Random ES distribution      
 Gene set null distribution of ES for **GOBP\_REGULATION\_OF\_STEROID\_BIOSYNTHETIC\_PROCESS**

  
