# Supplementary material for: Time-series transcriptome analysis identified differentially expressed genes in broiler chicken infected with mixed Eimeria species
Source: Front Genet. 2022 Aug 8;13:886781. doi: 10.3389/fgene.2022.886781 (PMC9393255; doi:10.3389/fgene.2022.886781)
Supplement: Supplementary file 2 [file DataSheet1.ZIP › 4dpi_GO.Gsea.1625071243202/GOBP_REGULATION_OF_STEROID_METABOLIC_PROCESS.html]

Details for gene set GOBP\_REGULATION\_OF\_STEROID\_METABOLIC\_PROCESS[GSEA]

|  || Dataset | TMM\_4dpi\_gct\_format\_4dpi\_gct\_format.Class\_4dpi.cls #PC\_versus\_NC.Class\_4dpi.cls #PC\_versus\_NC\_repos |
| Phenotype | Class\_4dpi.cls#PC\_versus\_NC\_repos |
| Upregulated in class | 1 |
| GeneSet | GOBP\_REGULATION\_OF\_STEROID\_METABOLIC\_PROCESS |
| Enrichment Score (ES) | 0.55837387 |
| Normalized Enrichment Score (NES) | 2.1337948 |
| Nominal p-value | 0.0 |
| FDR q-value | 0.0016967909 |
| FWER p-Value | 0.0254 |
Table: GSEA Results Summary

  

Fig 1: Enrichment plot: GOBP\_REGULATION\_OF\_STEROID\_METABOLIC\_PROCESS      
 Profile of the Running ES Score & Positions of GeneSet Members on the Rank Ordered List

  

| SYMBOL | TITLE | RANK IN GENE LIST | RANK METRIC SCORE | RUNNING ES | CORE ENRICHMENT || 1 | DHCR7 | na | 17 | 2.286 | 0.0458 | Yes |
| 2 | CYP51A1 | na | 28 | 2.188 | 0.0901 | Yes |
| 3 | HMGCS1 | na | 30 | 2.183 | 0.1352 | Yes |
| 4 | FDFT1 | na | 56 | 1.986 | 0.1741 | Yes |
| 5 | FDPS | na | 71 | 1.792 | 0.2099 | Yes |
| 6 | SQLE | na | 92 | 1.678 | 0.2429 | Yes |
| 7 | LSS | na | 138 | 1.500 | 0.2701 | Yes |
| 8 | INSIG1 | na | 148 | 1.471 | 0.2997 | Yes |
| 9 | APOB | na | 248 | 1.255 | 0.3173 | Yes |
| 10 | HMGCR | na | 299 | 1.179 | 0.3375 | Yes |
| 11 | SREBF2 | na | 333 | 1.135 | 0.3581 | Yes |
| 12 | MALRD1 | na | 336 | 1.133 | 0.3814 | Yes |
| 13 | GAL | na | 353 | 1.119 | 0.4031 | Yes |
| 14 | ACACA | na | 361 | 1.109 | 0.4255 | Yes |
| 15 | SC5D | na | 418 | 1.048 | 0.4424 | Yes |
| 16 | APOA1 | na | 589 | 0.889 | 0.4465 | Yes |
| 17 | DGAT2 | na | 643 | 0.854 | 0.4597 | Yes |
| 18 | SREBF1 | na | 691 | 0.823 | 0.4728 | Yes |
| 19 | STARD4 | na | 710 | 0.810 | 0.4880 | Yes |
| 20 | APOA4 | na | 733 | 0.797 | 0.5026 | Yes |
| 21 | FGF1 | na | 971 | 0.684 | 0.4969 | Yes |
| 22 | SCD | na | 1084 | 0.644 | 0.5008 | Yes |
| 23 | MBTPS2 | na | 1196 | 0.605 | 0.5040 | Yes |
| 24 | DGKQ | na | 1245 | 0.590 | 0.5121 | Yes |
| 25 | FGF19 | na | 1264 | 0.585 | 0.5227 | Yes |
| 26 | SNAI2 | na | 1276 | 0.583 | 0.5338 | Yes |
| 27 | BMP2 | na | 1379 | 0.553 | 0.5367 | Yes |
| 28 | PRKAA1 | na | 1419 | 0.542 | 0.5446 | Yes |
| 29 | DKK3 | na | 1533 | 0.512 | 0.5457 | Yes |
| 30 | TTC39B | na | 1575 | 0.502 | 0.5526 | Yes |
| 31 | SP1 | na | 1630 | 0.488 | 0.5582 | Yes |
| 32 | MVD | na | 1777 | 0.461 | 0.5554 | Yes |
| 33 | LPCAT3 | na | 1853 | 0.447 | 0.5584 | Yes |
| 34 | GPAM | na | 2506 | 0.348 | 0.5109 | No |
| 35 | ELOVL6 | na | 2559 | 0.341 | 0.5136 | No |
| 36 | RORA | na | 2627 | 0.332 | 0.5148 | No |
| 37 | ARV1 | na | 2818 | 0.307 | 0.5052 | No |
| 38 | ATP1A1 | na | 3009 | 0.281 | 0.4951 | No |
| 39 | H6PD | na | 3109 | 0.268 | 0.4923 | No |
| 40 | ABCB11 | na | 3136 | 0.264 | 0.4956 | No |
| 41 | NFYA | na | 3482 | 0.223 | 0.4712 | No |
| 42 | KPNB1 | na | 3483 | 0.223 | 0.4758 | No |
| 43 | AGTR1 | na | 3755 | 0.192 | 0.4571 | No |
| 44 | ERLIN1 | na | 3789 | 0.189 | 0.4582 | No |
| 45 | BMP6 | na | 3816 | 0.186 | 0.4599 | No |
| 46 | RORC | na | 4015 | 0.166 | 0.4467 | No |
| 47 | DHH | na | 4419 | 0.132 | 0.4156 | No |
| 48 | GFI1 | na | 4541 | 0.121 | 0.4080 | No |
| 49 | IGFBP7 | na | 4706 | 0.106 | 0.3964 | No |
| 50 | RAN | na | 5091 | 0.071 | 0.3657 | No |
| 51 | KIT | na | 5279 | 0.056 | 0.3512 | No |
| 52 | FASN | na | 5288 | 0.055 | 0.3516 | No |
| 53 | EPHX2 | na | 5461 | 0.040 | 0.3380 | No |
| 54 | LDLRAP1 | na | 5933 | -0.002 | 0.2986 | No |
| 55 | NFKB1 | na | 6765 | -0.068 | 0.2303 | No |
| 56 | MVK | na | 6801 | -0.071 | 0.2288 | No |
| 57 | MBTPS1 | na | 6903 | -0.080 | 0.2220 | No |
| 58 | BMP5 | na | 7077 | -0.096 | 0.2094 | No |
| 59 | PROX1 | na | 7123 | -0.100 | 0.2077 | No |
| 60 | PMVK | na | 7240 | -0.112 | 0.2003 | No |
| 61 | SNAI1 | na | 7275 | -0.116 | 0.1999 | No |
| 62 | GGPS1 | na | 7444 | -0.131 | 0.1885 | No |
| 63 | EGR1 | na | 7575 | -0.141 | 0.1805 | No |
| 64 | NFYC | na | 7623 | -0.146 | 0.1796 | No |
| 65 | ASAH1 | na | 7648 | -0.148 | 0.1806 | No |
| 66 | NR1H4 | na | 7657 | -0.148 | 0.1830 | No |
| 67 | PANK2 | na | 7754 | -0.158 | 0.1782 | No |
| 68 | LAMTOR1 | na | 7770 | -0.159 | 0.1802 | No |
| 69 | ERLIN2 | na | 7983 | -0.178 | 0.1661 | No |
| 70 | SEC14L2 | na | 8060 | -0.186 | 0.1636 | No |
| 71 | ABCA2 | na | 8565 | -0.237 | 0.1262 | No |
| 72 | NR0B1 | na | 8635 | -0.243 | 0.1254 | No |
| 73 | WNT4 | na | 8711 | -0.252 | 0.1243 | No |
| 74 | TSPO | na | 8903 | -0.274 | 0.1140 | No |
| 75 | FGFR4 | na | 9361 | -0.330 | 0.0825 | No |
| 76 | CLCN2 | na | 9370 | -0.331 | 0.0887 | No |
| 77 | STUB1 | na | 9707 | -0.375 | 0.0682 | No |
| 78 | SIRT1 | na | 9868 | -0.394 | 0.0629 | No |
| 79 | SOD1 | na | 10118 | -0.432 | 0.0510 | No |
| 80 | NR1D1 | na | 10243 | -0.451 | 0.0499 | No |
| 81 | ACADL | na | 11004 | -0.609 | -0.0012 | No |
| 82 | ABCG1 | na | 11046 | -0.620 | 0.0081 | No |
| 83 | SCAP | na | 11086 | -0.632 | 0.0179 | No |
| 84 | PPARGC1A | na | 11134 | -0.645 | 0.0273 | No |
| 85 | ADM | na | 11649 | -0.915 | 0.0031 | No |
| 86 | UGT1A1 | na | 11907 | -1.313 | 0.0086 | No |
Table: GSEA details [plain text format]

  

Fig 2: GOBP\_REGULATION\_OF\_STEROID\_METABOLIC\_PROCESS      
 Blue-Pink O' Gram in the Space of the Analyzed GeneSet

  

Fig 3: GOBP\_REGULATION\_OF\_STEROID\_METABOLIC\_PROCESS: Random ES distribution      
 Gene set null distribution of ES for **GOBP\_REGULATION\_OF\_STEROID\_METABOLIC\_PROCESS**

  
