# Supplementary material for: Time-series transcriptome analysis identified differentially expressed genes in broiler chicken infected with mixed Eimeria species
Source: Front Genet. 2022 Aug 8;13:886781. doi: 10.3389/fgene.2022.886781 (PMC9393255; doi:10.3389/fgene.2022.886781)
Supplement: Supplementary file 2 [file DataSheet1.ZIP › 4dpi_GO.Gsea.1625071243202/GOBP_RESPONSE_TO_GLUCAGON.html]

Details for gene set GOBP\_RESPONSE\_TO\_GLUCAGON[GSEA]

|  || Dataset | TMM\_4dpi\_gct\_format\_4dpi\_gct\_format.Class\_4dpi.cls #PC\_versus\_NC.Class\_4dpi.cls #PC\_versus\_NC\_repos |
| Phenotype | Class\_4dpi.cls#PC\_versus\_NC\_repos |
| Upregulated in class | 1 |
| GeneSet | GOBP\_RESPONSE\_TO\_GLUCAGON |
| Enrichment Score (ES) | 0.7159511 |
| Normalized Enrichment Score (NES) | 2.1796584 |
| Nominal p-value | 0.0 |
| FDR q-value | 6.93046E-4 |
| FWER p-Value | 0.0072 |
Table: GSEA Results Summary

  

Fig 1: Enrichment plot: GOBP\_RESPONSE\_TO\_GLUCAGON      
 Profile of the Running ES Score & Positions of GeneSet Members on the Rank Ordered List

  

| SYMBOL | TITLE | RANK IN GENE LIST | RANK METRIC SCORE | RUNNING ES | CORE ENRICHMENT || 1 | GCG | na | 65 | 1.843 | 0.1392 | Yes |
| 2 | CDO1 | na | 205 | 1.345 | 0.2332 | Yes |
| 3 | PCK1 | na | 446 | 1.020 | 0.2933 | Yes |
| 4 | SREBF1 | na | 691 | 0.823 | 0.3375 | Yes |
| 5 | ADCY8 | na | 750 | 0.789 | 0.3946 | Yes |
| 6 | PRKAR2B | na | 791 | 0.764 | 0.4512 | Yes |
| 7 | ABCC2 | na | 792 | 0.764 | 0.5112 | Yes |
| 8 | CRY1 | na | 876 | 0.723 | 0.5610 | Yes |
| 9 | PRKAR2A | na | 925 | 0.700 | 0.6120 | Yes |
| 10 | GLP2R | na | 939 | 0.697 | 0.6656 | Yes |
| 11 | ADCY7 | na | 1147 | 0.620 | 0.6969 | Yes |
| 12 | GCGR | na | 1427 | 0.539 | 0.7160 | Yes |
| 13 | GLP1R | na | 3401 | 0.232 | 0.5695 | No |
| 14 | ADCY2 | na | 3608 | 0.206 | 0.5685 | No |
| 15 | PRKAR1B | na | 3650 | 0.202 | 0.5810 | No |
| 16 | CCNA2 | na | 4342 | 0.139 | 0.5342 | No |
| 17 | RPS6KB1 | na | 4509 | 0.123 | 0.5300 | No |
| 18 | PRKAR1A | na | 5407 | 0.045 | 0.4587 | No |
| 19 | CREB1 | na | 6598 | -0.054 | 0.3637 | No |
| 20 | ADCY3 | na | 6636 | -0.057 | 0.3651 | No |
| 21 | GJB2 | na | 6834 | -0.073 | 0.3544 | No |
| 22 | PRKACB | na | 7013 | -0.090 | 0.3466 | No |
| 23 | CPS1 | na | 7092 | -0.097 | 0.3478 | No |
| 24 | QDPR | na | 7495 | -0.135 | 0.3248 | No |
| 25 | ASS1 | na | 8042 | -0.184 | 0.2936 | No |
| 26 | ADCY6 | na | 8259 | -0.205 | 0.2917 | No |
| 27 | ADCY9 | na | 8880 | -0.271 | 0.2612 | No |
Table: GSEA details [plain text format]

  

Fig 2: GOBP\_RESPONSE\_TO\_GLUCAGON      
 Blue-Pink O' Gram in the Space of the Analyzed GeneSet

  

Fig 3: GOBP\_RESPONSE\_TO\_GLUCAGON: Random ES distribution      
 Gene set null distribution of ES for **GOBP\_RESPONSE\_TO\_GLUCAGON**

  
