# Supplementary material for: Time-series transcriptome analysis identified differentially expressed genes in broiler chicken infected with mixed Eimeria species
Source: Front Genet. 2022 Aug 8;13:886781. doi: 10.3389/fgene.2022.886781 (PMC9393255; doi:10.3389/fgene.2022.886781)
Supplement: Supplementary file 2 [file DataSheet1.ZIP › 4dpi_GO.Gsea.1625071243202/GOBP_RESPONSE_TO_TYPE_I_INTERFERON.html]

Details for gene set GOBP\_RESPONSE\_TO\_TYPE\_I\_INTERFERON[GSEA]

|  || Dataset | TMM\_4dpi\_gct\_format\_4dpi\_gct\_format.Class\_4dpi.cls #PC\_versus\_NC.Class\_4dpi.cls #PC\_versus\_NC\_repos |
| Phenotype | Class\_4dpi.cls#PC\_versus\_NC\_repos |
| Upregulated in class | 1 |
| GeneSet | GOBP\_RESPONSE\_TO\_TYPE\_I\_INTERFERON |
| Enrichment Score (ES) | 0.64081025 |
| Normalized Enrichment Score (NES) | 2.2091966 |
| Nominal p-value | 0.0 |
| FDR q-value | 3.94736E-4 |
| FWER p-Value | 0.003 |
Table: GSEA Results Summary

  

Fig 1: Enrichment plot: GOBP\_RESPONSE\_TO\_TYPE\_I\_INTERFERON      
 Profile of the Running ES Score & Positions of GeneSet Members on the Rank Ordered List

  

| SYMBOL | TITLE | RANK IN GENE LIST | RANK METRIC SCORE | RUNNING ES | CORE ENRICHMENT || 1 | MX1 | na | 36 | 2.093 | 0.0615 | Yes |
| 2 | OASL | na | 52 | 2.001 | 0.1219 | Yes |
| 3 | STAT1 | na | 78 | 1.743 | 0.1735 | Yes |
| 4 | IRF7 | na | 85 | 1.706 | 0.2255 | Yes |
| 5 | IRF9 | na | 103 | 1.617 | 0.2739 | Yes |
| 6 | IRF1 | na | 114 | 1.568 | 0.3214 | Yes |
| 7 | IFI6 | na | 145 | 1.476 | 0.3644 | Yes |
| 8 | NLRC5 | na | 162 | 1.431 | 0.4071 | Yes |
| 9 | SAMHD1 | na | 170 | 1.414 | 0.4501 | Yes |
| 10 | RSAD2 | na | 220 | 1.319 | 0.4867 | Yes |
| 11 | IFI35 | na | 262 | 1.229 | 0.5211 | Yes |
| 12 | MYD88 | na | 304 | 1.174 | 0.5538 | Yes |
| 13 | ADAR | na | 412 | 1.051 | 0.5773 | Yes |
| 14 | IKBKE | na | 505 | 0.959 | 0.5992 | Yes |
| 15 | IFNAR1 | na | 640 | 0.855 | 0.6143 | Yes |
| 16 | STAT2 | na | 751 | 0.788 | 0.6294 | Yes |
| 17 | IRF8 | na | 981 | 0.678 | 0.6311 | Yes |
| 18 | IFNAR2 | na | 1101 | 0.637 | 0.6408 | Yes |
| 19 | PTPN11 | na | 2311 | 0.374 | 0.5513 | No |
| 20 | TYK2 | na | 2527 | 0.344 | 0.5439 | No |
| 21 | RNASEL | na | 2765 | 0.313 | 0.5337 | No |
| 22 | FADD | na | 2797 | 0.309 | 0.5406 | No |
| 23 | WNT5A | na | 3037 | 0.277 | 0.5292 | No |
| 24 | CNOT7 | na | 3796 | 0.188 | 0.4716 | No |
| 25 | PTPN1 | na | 4467 | 0.127 | 0.4195 | No |
| 26 | TBK1 | na | 4986 | 0.079 | 0.3787 | No |
| 27 | LSM14A | na | 5048 | 0.074 | 0.3758 | No |
| 28 | UBE2K | na | 5277 | 0.056 | 0.3585 | No |
| 29 | CDC37 | na | 5605 | 0.025 | 0.3319 | No |
| 30 | YTHDF3 | na | 6099 | -0.014 | 0.2912 | No |
| 31 | MAVS | na | 6400 | -0.038 | 0.2673 | No |
| 32 | ABCE1 | na | 6592 | -0.054 | 0.2530 | No |
| 33 | IRF2 | na | 7126 | -0.100 | 0.2115 | No |
| 34 | JAK1 | na | 7146 | -0.103 | 0.2131 | No |
| 35 | EGR1 | na | 7575 | -0.141 | 0.1816 | No |
| 36 | SETD2 | na | 7829 | -0.164 | 0.1656 | No |
| 37 | HSP90AB1 | na | 8645 | -0.244 | 0.1049 | No |
| 38 | PTPN2 | na | 8813 | -0.263 | 0.0991 | No |
| 39 | TTLL12 | na | 9189 | -0.307 | 0.0772 | No |
| 40 | IP6K2 | na | 9376 | -0.332 | 0.0719 | No |
| 41 | PTPN6 | na | 9540 | -0.354 | 0.0692 | No |
| 42 | METTL3 | na | 9660 | -0.369 | 0.0706 | No |
| 43 | IRF6 | na | 10011 | -0.416 | 0.0541 | No |
| 44 | YTHDF2 | na | 10549 | -0.507 | 0.0249 | No |
| 45 | IRF4 | na | 10673 | -0.534 | 0.0310 | No |
| 46 | MUL1 | na | 10974 | -0.600 | 0.0244 | No |
| 47 | CACTIN | na | 11024 | -0.616 | 0.0393 | No |
| 48 | IRF5 | na | 11267 | -0.688 | 0.0403 | No |
| 49 | SMPD1 | na | 11304 | -0.706 | 0.0590 | No |
Table: GSEA details [plain text format]

  

Fig 2: GOBP\_RESPONSE\_TO\_TYPE\_I\_INTERFERON      
 Blue-Pink O' Gram in the Space of the Analyzed GeneSet

  

Fig 3: GOBP\_RESPONSE\_TO\_TYPE\_I\_INTERFERON: Random ES distribution      
 Gene set null distribution of ES for **GOBP\_RESPONSE\_TO\_TYPE\_I\_INTERFERON**

  
