# Supplementary material for: Time-series transcriptome analysis identified differentially expressed genes in broiler chicken infected with mixed Eimeria species
Source: Front Genet. 2022 Aug 8;13:886781. doi: 10.3389/fgene.2022.886781 (PMC9393255; doi:10.3389/fgene.2022.886781)
Supplement: Supplementary file 2 [file DataSheet1.ZIP › 4dpi_GO.Gsea.1625071243202/GOBP_RIBOSOMAL_LARGE_SUBUNIT_BIOGENESIS.html]

Details for gene set GOBP\_RIBOSOMAL\_LARGE\_SUBUNIT\_BIOGENESIS[GSEA]

|  || Dataset | TMM\_4dpi\_gct\_format\_4dpi\_gct\_format.Class\_4dpi.cls #PC\_versus\_NC.Class\_4dpi.cls #PC\_versus\_NC\_repos |
| Phenotype | Class\_4dpi.cls#PC\_versus\_NC\_repos |
| Upregulated in class | 0 |
| GeneSet | GOBP\_RIBOSOMAL\_LARGE\_SUBUNIT\_BIOGENESIS |
| Enrichment Score (ES) | -0.625106 |
| Normalized Enrichment Score (NES) | -2.4136186 |
| Nominal p-value | 0.0 |
| FDR q-value | 1.1631902E-5 |
| FWER p-Value | 2.0E-4 |
Table: GSEA Results Summary

  

Fig 1: Enrichment plot: GOBP\_RIBOSOMAL\_LARGE\_SUBUNIT\_BIOGENESIS      
 Profile of the Running ES Score & Positions of GeneSet Members on the Rank Ordered List

  

| SYMBOL | TITLE | RANK IN GENE LIST | RANK METRIC SCORE | RUNNING ES | CORE ENRICHMENT || 1 | RPF2 | na | 2744 | 0.316 | -0.2152 | No |
| 2 | MRTO4 | na | 3990 | 0.169 | -0.3116 | No |
| 3 | NHP2 | na | 4041 | 0.164 | -0.3082 | No |
| 4 | ZNHIT6 | na | 4168 | 0.154 | -0.3117 | No |
| 5 | TRAF7 | na | 4427 | 0.131 | -0.3273 | No |
| 6 | WDR74 | na | 4430 | 0.131 | -0.3214 | No |
| 7 | ZNHIT3 | na | 4620 | 0.114 | -0.3320 | No |
| 8 | NVL | na | 5165 | 0.064 | -0.3746 | No |
| 9 | MDN1 | na | 5326 | 0.052 | -0.3856 | No |
| 10 | BRIX1 | na | 5688 | 0.018 | -0.4150 | No |
| 11 | SDAD1 | na | 5900 | 0.002 | -0.4326 | No |
| 12 | GTF3A | na | 5937 | -0.002 | -0.4355 | No |
| 13 | RRP15 | na | 5998 | -0.006 | -0.4403 | No |
| 14 | SURF6 | na | 6022 | -0.008 | -0.4418 | No |
| 15 | WDR12 | na | 6181 | -0.021 | -0.4541 | No |
| 16 | RSL24D1 | na | 6280 | -0.029 | -0.4610 | No |
| 17 | LAS1L | na | 6358 | -0.035 | -0.4658 | No |
| 18 | DDX18 | na | 6475 | -0.045 | -0.4735 | No |
| 19 | BOP1 | na | 6502 | -0.046 | -0.4735 | No |
| 20 | MALSU1 | na | 6548 | -0.050 | -0.4750 | No |
| 21 | RPF1 | na | 6697 | -0.063 | -0.4845 | No |
| 22 | HEATR3 | na | 7100 | -0.098 | -0.5136 | No |
| 23 | NIP7 | na | 7188 | -0.107 | -0.5160 | No |
| 24 | EBNA1BP2 | na | 7464 | -0.132 | -0.5329 | No |
| 25 | EIF6 | na | 7476 | -0.133 | -0.5277 | No |
| 26 | MRPL1 | na | 7631 | -0.147 | -0.5339 | No |
| 27 | PAK1IP1 | na | 7881 | -0.169 | -0.5469 | No |
| 28 | NOL9 | na | 8161 | -0.194 | -0.5614 | No |
| 29 | NIFK | na | 8383 | -0.217 | -0.5699 | No |
| 30 | MRPL20 | na | 8418 | -0.220 | -0.5626 | No |
| 31 | RPL7L1 | na | 8437 | -0.222 | -0.5539 | No |
| 32 | ZNF622 | na | 8452 | -0.224 | -0.5448 | No |
| 33 | MAK16 | na | 9062 | -0.291 | -0.5824 | No |
| 34 | GTPBP4 | na | 9573 | -0.359 | -0.6086 | Yes |
| 35 | NOP16 | na | 9650 | -0.368 | -0.5981 | Yes |
| 36 | RPL38 | na | 9661 | -0.369 | -0.5819 | Yes |
| 37 | RRS1 | na | 9732 | -0.378 | -0.5704 | Yes |
| 38 | FASTKD2 | na | 9983 | -0.411 | -0.5725 | Yes |
| 39 | NSA2 | na | 9996 | -0.414 | -0.5544 | Yes |
| 40 | DHX30 | na | 10240 | -0.451 | -0.5540 | Yes |
| 41 | NOC2L | na | 10327 | -0.466 | -0.5398 | Yes |
| 42 | DDX28 | na | 10412 | -0.481 | -0.5247 | Yes |
| 43 | RSL1D1 | na | 10488 | -0.496 | -0.5082 | Yes |
| 44 | NOP2 | na | 10578 | -0.514 | -0.4920 | Yes |
| 45 | RPL14 | na | 10662 | -0.531 | -0.4745 | Yes |
| 46 | URB1 | na | 10751 | -0.549 | -0.4567 | Yes |
| 47 | RPL24 | na | 10877 | -0.576 | -0.4406 | Yes |
| 48 | FTSJ3 | na | 10878 | -0.577 | -0.4141 | Yes |
| 49 | NLE1 | na | 10907 | -0.583 | -0.3897 | Yes |
| 50 | RPL35A | na | 10982 | -0.602 | -0.3682 | Yes |
| 51 | RPL23A | na | 11076 | -0.629 | -0.3471 | Yes |
| 52 | RPL5 | na | 11121 | -0.643 | -0.3212 | Yes |
| 53 | RPL6 | na | 11150 | -0.650 | -0.2937 | Yes |
| 54 | RPL11 | na | 11195 | -0.662 | -0.2669 | Yes |
| 55 | NPM1 | na | 11221 | -0.672 | -0.2382 | Yes |
| 56 | RPL35 | na | 11280 | -0.696 | -0.2110 | Yes |
| 57 | RPL26L1 | na | 11283 | -0.698 | -0.1791 | Yes |
| 58 | RPL7A | na | 11426 | -0.763 | -0.1559 | Yes |
| 59 | PES1 | na | 11473 | -0.789 | -0.1235 | Yes |
| 60 | RPL7 | na | 11484 | -0.796 | -0.0877 | Yes |
| 61 | RPLP0 | na | 11488 | -0.800 | -0.0512 | Yes |
| 62 | RPL10A | na | 11658 | -0.921 | -0.0230 | Yes |
| 63 | RPL3 | na | 11817 | -1.140 | 0.0162 | Yes |
Table: GSEA details [plain text format]

  

Fig 2: GOBP\_RIBOSOMAL\_LARGE\_SUBUNIT\_BIOGENESIS      
 Blue-Pink O' Gram in the Space of the Analyzed GeneSet

  

Fig 3: GOBP\_RIBOSOMAL\_LARGE\_SUBUNIT\_BIOGENESIS: Random ES distribution      
 Gene set null distribution of ES for **GOBP\_RIBOSOMAL\_LARGE\_SUBUNIT\_BIOGENESIS**

  
