# Supplementary material for: Time-series transcriptome analysis identified differentially expressed genes in broiler chicken infected with mixed Eimeria species
Source: Front Genet. 2022 Aug 8;13:886781. doi: 10.3389/fgene.2022.886781 (PMC9393255; doi:10.3389/fgene.2022.886781)
Supplement: Supplementary file 2 [file DataSheet1.ZIP › 4dpi_GO.Gsea.1625071243202/GOBP_STEROL_BIOSYNTHETIC_PROCESS.html]

Details for gene set GOBP\_STEROL\_BIOSYNTHETIC\_PROCESS[GSEA]

|  || Dataset | TMM\_4dpi\_gct\_format\_4dpi\_gct\_format.Class\_4dpi.cls #PC\_versus\_NC.Class\_4dpi.cls #PC\_versus\_NC\_repos |
| Phenotype | Class\_4dpi.cls#PC\_versus\_NC\_repos |
| Upregulated in class | 1 |
| GeneSet | GOBP\_STEROL\_BIOSYNTHETIC\_PROCESS |
| Enrichment Score (ES) | 0.65238994 |
| Normalized Enrichment Score (NES) | 2.3211606 |
| Nominal p-value | 0.0 |
| FDR q-value | 0.0 |
| FWER p-Value | 0.0 |
Table: GSEA Results Summary

  

Fig 1: Enrichment plot: GOBP\_STEROL\_BIOSYNTHETIC\_PROCESS      
 Profile of the Running ES Score & Positions of GeneSet Members on the Rank Ordered List

  

| SYMBOL | TITLE | RANK IN GENE LIST | RANK METRIC SCORE | RUNNING ES | CORE ENRICHMENT || 1 | DHCR7 | na | 17 | 2.286 | 0.0510 | Yes |
| 2 | MSMO1 | na | 25 | 2.193 | 0.1007 | Yes |
| 3 | CYP51A1 | na | 28 | 2.188 | 0.1507 | Yes |
| 4 | HMGCS1 | na | 30 | 2.183 | 0.2006 | Yes |
| 5 | FDFT1 | na | 56 | 1.986 | 0.2441 | Yes |
| 6 | IDI2 | na | 67 | 1.826 | 0.2851 | Yes |
| 7 | FDPS | na | 71 | 1.792 | 0.3259 | Yes |
| 8 | SQLE | na | 92 | 1.678 | 0.3627 | Yes |
| 9 | LSS | na | 138 | 1.500 | 0.3933 | Yes |
| 10 | INSIG1 | na | 148 | 1.471 | 0.4263 | Yes |
| 11 | DHCR24 | na | 221 | 1.313 | 0.4503 | Yes |
| 12 | APOB | na | 248 | 1.255 | 0.4769 | Yes |
| 13 | HMGCR | na | 299 | 1.179 | 0.4998 | Yes |
| 14 | SREBF2 | na | 333 | 1.135 | 0.5230 | Yes |
| 15 | ACACA | na | 361 | 1.109 | 0.5462 | Yes |
| 16 | NSDHL | na | 369 | 1.104 | 0.5709 | Yes |
| 17 | HSD17B7 | na | 410 | 1.054 | 0.5917 | Yes |
| 18 | SC5D | na | 418 | 1.048 | 0.6151 | Yes |
| 19 | APOA1 | na | 589 | 0.889 | 0.6213 | Yes |
| 20 | SREBF1 | na | 691 | 0.823 | 0.6317 | Yes |
| 21 | APOA4 | na | 733 | 0.797 | 0.6466 | Yes |
| 22 | FGF1 | na | 971 | 0.684 | 0.6424 | Yes |
| 23 | SCD | na | 1084 | 0.644 | 0.6478 | Yes |
| 24 | MBTPS2 | na | 1196 | 0.605 | 0.6524 | Yes |
| 25 | PRKAA1 | na | 1419 | 0.542 | 0.6462 | No |
| 26 | SP1 | na | 1630 | 0.488 | 0.6399 | No |
| 27 | MVD | na | 1777 | 0.461 | 0.6382 | No |
| 28 | LPCAT3 | na | 1853 | 0.447 | 0.6422 | No |
| 29 | ACAT2 | na | 2179 | 0.392 | 0.6240 | No |
| 30 | GPAM | na | 2506 | 0.348 | 0.6047 | No |
| 31 | ELOVL6 | na | 2559 | 0.341 | 0.6081 | No |
| 32 | ARV1 | na | 2818 | 0.307 | 0.5936 | No |
| 33 | LBR | na | 2914 | 0.294 | 0.5924 | No |
| 34 | CH25H | na | 2984 | 0.284 | 0.5931 | No |
| 35 | ACLY | na | 3252 | 0.250 | 0.5765 | No |
| 36 | NFYA | na | 3482 | 0.223 | 0.5624 | No |
| 37 | KPNB1 | na | 3483 | 0.223 | 0.5676 | No |
| 38 | ERLIN1 | na | 3789 | 0.189 | 0.5464 | No |
| 39 | PRKAA2 | na | 4804 | 0.097 | 0.4638 | No |
| 40 | RAN | na | 5091 | 0.071 | 0.4414 | No |
| 41 | PRKAG2 | na | 5102 | 0.070 | 0.4422 | No |
| 42 | FASN | na | 5288 | 0.055 | 0.4280 | No |
| 43 | ACAA2 | na | 5307 | 0.053 | 0.4277 | No |
| 44 | MVK | na | 6801 | -0.071 | 0.3044 | No |
| 45 | MBTPS1 | na | 6903 | -0.080 | 0.2978 | No |
| 46 | FAXDC2 | na | 7179 | -0.106 | 0.2772 | No |
| 47 | PMVK | na | 7240 | -0.112 | 0.2748 | No |
| 48 | GGPS1 | na | 7444 | -0.131 | 0.2608 | No |
| 49 | NFYC | na | 7623 | -0.146 | 0.2492 | No |
| 50 | ERLIN2 | na | 7983 | -0.178 | 0.2233 | No |
| 51 | SEC14L2 | na | 8060 | -0.186 | 0.2212 | No |
| 52 | CYB5R1 | na | 9292 | -0.320 | 0.1255 | No |
| 53 | SOD1 | na | 10118 | -0.432 | 0.0664 | No |
| 54 | CYB5R2 | na | 10695 | -0.537 | 0.0306 | No |
| 55 | ABCG1 | na | 11046 | -0.620 | 0.0155 | No |
| 56 | SCAP | na | 11086 | -0.632 | 0.0267 | No |
| 57 | CFTR | na | 11758 | -1.030 | -0.0058 | No |
| 58 | INSIG2 | na | 11843 | -1.170 | 0.0140 | No |
Table: GSEA details [plain text format]

  

Fig 2: GOBP\_STEROL\_BIOSYNTHETIC\_PROCESS      
 Blue-Pink O' Gram in the Space of the Analyzed GeneSet

  

Fig 3: GOBP\_STEROL\_BIOSYNTHETIC\_PROCESS: Random ES distribution      
 Gene set null distribution of ES for **GOBP\_STEROL\_BIOSYNTHETIC\_PROCESS**

  
