# Supplementary material for: Time-series transcriptome analysis identified differentially expressed genes in broiler chicken infected with mixed Eimeria species
Source: Front Genet. 2022 Aug 8;13:886781. doi: 10.3389/fgene.2022.886781 (PMC9393255; doi:10.3389/fgene.2022.886781)
Supplement: Supplementary file 2 [file DataSheet1.ZIP › 4dpi_GO.Gsea.1625071243202/GOBP_STEROL_HOMEOSTASIS.html]

Details for gene set GOBP\_STEROL\_HOMEOSTASIS[GSEA]

|  || Dataset | TMM\_4dpi\_gct\_format\_4dpi\_gct\_format.Class\_4dpi.cls #PC\_versus\_NC.Class\_4dpi.cls #PC\_versus\_NC\_repos |
| Phenotype | Class\_4dpi.cls#PC\_versus\_NC\_repos |
| Upregulated in class | 1 |
| GeneSet | GOBP\_STEROL\_HOMEOSTASIS |
| Enrichment Score (ES) | 0.60591054 |
| Normalized Enrichment Score (NES) | 2.1411195 |
| Nominal p-value | 0.0 |
| FDR q-value | 0.0017421079 |
| FWER p-Value | 0.0228 |
Table: GSEA Results Summary

  

Fig 1: Enrichment plot: GOBP\_STEROL\_HOMEOSTASIS      
 Profile of the Running ES Score & Positions of GeneSet Members on the Rank Ordered List

  

| SYMBOL | TITLE | RANK IN GENE LIST | RANK METRIC SCORE | RUNNING ES | CORE ENRICHMENT || 1 | ABCG8 | na | 2 | 3.384 | 0.0987 | Yes |
| 2 | ABCG5 | na | 41 | 2.059 | 0.1557 | Yes |
| 3 | PLA2G12B | na | 115 | 1.564 | 0.1953 | Yes |
| 4 | INSIG1 | na | 148 | 1.471 | 0.2356 | Yes |
| 5 | LDLR | na | 159 | 1.437 | 0.2767 | Yes |
| 6 | APOB | na | 248 | 1.255 | 0.3060 | Yes |
| 7 | SREBF2 | na | 333 | 1.135 | 0.3321 | Yes |
| 8 | MALRD1 | na | 336 | 1.133 | 0.3651 | Yes |
| 9 | SOAT1 | na | 350 | 1.121 | 0.3967 | Yes |
| 10 | MALL | na | 364 | 1.107 | 0.4280 | Yes |
| 11 | MTTP | na | 492 | 0.971 | 0.4457 | Yes |
| 12 | APOA1 | na | 589 | 0.889 | 0.4637 | Yes |
| 13 | DGAT2 | na | 643 | 0.854 | 0.4842 | Yes |
| 14 | APOA4 | na | 733 | 0.797 | 0.5001 | Yes |
| 15 | LPL | na | 954 | 0.691 | 0.5019 | Yes |
| 16 | NPC1 | na | 1035 | 0.659 | 0.5144 | Yes |
| 17 | LIMA1 | na | 1076 | 0.647 | 0.5300 | Yes |
| 18 | FABP3 | na | 1105 | 0.636 | 0.5462 | Yes |
| 19 | IL18 | na | 1198 | 0.604 | 0.5562 | Yes |
| 20 | HNF4A | na | 1369 | 0.556 | 0.5582 | Yes |
| 21 | FABP4 | na | 1519 | 0.515 | 0.5608 | Yes |
| 22 | RALY | na | 1524 | 0.514 | 0.5755 | Yes |
| 23 | TTC39B | na | 1575 | 0.502 | 0.5860 | Yes |
| 24 | TSKU | na | 1738 | 0.468 | 0.5861 | Yes |
| 25 | DISP3 | na | 1775 | 0.461 | 0.5965 | Yes |
| 26 | NUS1 | na | 1926 | 0.436 | 0.5967 | Yes |
| 27 | CAV1 | na | 1967 | 0.429 | 0.6059 | Yes |
| 28 | RORA | na | 2627 | 0.332 | 0.5605 | No |
| 29 | ABCB11 | na | 3136 | 0.264 | 0.5257 | No |
| 30 | NR1H3 | na | 3213 | 0.255 | 0.5268 | No |
| 31 | PCSK9 | na | 3963 | 0.171 | 0.4692 | No |
| 32 | LIPG | na | 4514 | 0.123 | 0.4267 | No |
| 33 | TMEM97 | na | 4835 | 0.095 | 0.4027 | No |
| 34 | CNBP | na | 5374 | 0.048 | 0.3591 | No |
| 35 | MYLIP | na | 5422 | 0.044 | 0.3565 | No |
| 36 | EPHX2 | na | 5461 | 0.040 | 0.3545 | No |
| 37 | SCARB1 | na | 5664 | 0.020 | 0.3382 | No |
| 38 | XBP1 | na | 5735 | 0.014 | 0.3327 | No |
| 39 | LDLRAP1 | na | 5933 | -0.002 | 0.3163 | No |
| 40 | GRAMD1B | na | 7049 | -0.094 | 0.2258 | No |
| 41 | NR1H4 | na | 7657 | -0.148 | 0.1793 | No |
| 42 | LAMTOR1 | na | 7770 | -0.159 | 0.1746 | No |
| 43 | HDAC9 | na | 7771 | -0.159 | 0.1792 | No |
| 44 | LRP5 | na | 7897 | -0.170 | 0.1738 | No |
| 45 | NPC2 | na | 8394 | -0.218 | 0.1386 | No |
| 46 | ABCA2 | na | 8565 | -0.237 | 0.1313 | No |
| 47 | FGFR4 | na | 9361 | -0.330 | 0.0745 | No |
| 48 | NR5A2 | na | 9423 | -0.339 | 0.0793 | No |
| 49 | ABCA1 | na | 9675 | -0.371 | 0.0691 | No |
| 50 | SIRT1 | na | 9868 | -0.394 | 0.0646 | No |
| 51 | NR1D1 | na | 10243 | -0.451 | 0.0465 | No |
| 52 | CYP39A1 | na | 10542 | -0.507 | 0.0364 | No |
| 53 | COMMD9 | na | 10592 | -0.518 | 0.0474 | No |
| 54 | ABCG1 | na | 11046 | -0.620 | 0.0276 | No |
| 55 | CD24 | na | 11079 | -0.630 | 0.0433 | No |
| 56 | LCAT | na | 11849 | -1.179 | 0.0135 | No |
Table: GSEA details [plain text format]

  

Fig 2: GOBP\_STEROL\_HOMEOSTASIS      
 Blue-Pink O' Gram in the Space of the Analyzed GeneSet

  

Fig 3: GOBP\_STEROL\_HOMEOSTASIS: Random ES distribution      
 Gene set null distribution of ES for **GOBP\_STEROL\_HOMEOSTASIS**

  
