# Supplementary material for: Time-series transcriptome analysis identified differentially expressed genes in broiler chicken infected with mixed Eimeria species
Source: Front Genet. 2022 Aug 8;13:886781. doi: 10.3389/fgene.2022.886781 (PMC9393255; doi:10.3389/fgene.2022.886781)
Supplement: Supplementary file 2 [file DataSheet1.ZIP › 4dpi_GO.Gsea.1625071243202/GOBP_STEROL_METABOLIC_PROCESS.html]

Details for gene set GOBP\_STEROL\_METABOLIC\_PROCESS[GSEA]

|  || Dataset | TMM\_4dpi\_gct\_format\_4dpi\_gct\_format.Class\_4dpi.cls #PC\_versus\_NC.Class\_4dpi.cls #PC\_versus\_NC\_repos |
| Phenotype | Class\_4dpi.cls#PC\_versus\_NC\_repos |
| Upregulated in class | 1 |
| GeneSet | GOBP\_STEROL\_METABOLIC\_PROCESS |
| Enrichment Score (ES) | 0.55160594 |
| Normalized Enrichment Score (NES) | 2.18601 |
| Nominal p-value | 0.0 |
| FDR q-value | 5.4935477E-4 |
| FWER p-Value | 0.0052 |
Table: GSEA Results Summary

  

Fig 1: Enrichment plot: GOBP\_STEROL\_METABOLIC\_PROCESS      
 Profile of the Running ES Score & Positions of GeneSet Members on the Rank Ordered List

  

| SYMBOL | TITLE | RANK IN GENE LIST | RANK METRIC SCORE | RUNNING ES | CORE ENRICHMENT || 1 | DHCR7 | na | 17 | 2.286 | 0.0307 | Yes |
| 2 | MSMO1 | na | 25 | 2.193 | 0.0609 | Yes |
| 3 | CYP51A1 | na | 28 | 2.188 | 0.0915 | Yes |
| 4 | HMGCS1 | na | 30 | 2.183 | 0.1221 | Yes |
| 5 | CYP2D6 | na | 49 | 2.025 | 0.1490 | Yes |
| 6 | FDFT1 | na | 56 | 1.986 | 0.1764 | Yes |
| 7 | IDI2 | na | 67 | 1.826 | 0.2012 | Yes |
| 8 | FDPS | na | 71 | 1.792 | 0.2262 | Yes |
| 9 | SQLE | na | 92 | 1.678 | 0.2481 | Yes |
| 10 | LSS | na | 138 | 1.500 | 0.2654 | Yes |
| 11 | CYP1B1 | na | 146 | 1.473 | 0.2855 | Yes |
| 12 | INSIG1 | na | 148 | 1.471 | 0.3060 | Yes |
| 13 | LDLR | na | 159 | 1.437 | 0.3254 | Yes |
| 14 | OSBPL1A | na | 203 | 1.347 | 0.3407 | Yes |
| 15 | DHCR24 | na | 221 | 1.313 | 0.3577 | Yes |
| 16 | APOB | na | 248 | 1.255 | 0.3732 | Yes |
| 17 | LIPA | na | 288 | 1.188 | 0.3866 | Yes |
| 18 | HMGCR | na | 299 | 1.179 | 0.4023 | Yes |
| 19 | SREBF2 | na | 333 | 1.135 | 0.4155 | Yes |
| 20 | SOAT1 | na | 350 | 1.121 | 0.4299 | Yes |
| 21 | ACACA | na | 361 | 1.109 | 0.4446 | Yes |
| 22 | NSDHL | na | 369 | 1.104 | 0.4595 | Yes |
| 23 | HSD17B7 | na | 410 | 1.054 | 0.4710 | Yes |
| 24 | SC5D | na | 418 | 1.048 | 0.4851 | Yes |
| 25 | LIPE | na | 458 | 1.009 | 0.4960 | Yes |
| 26 | APOA1 | na | 589 | 0.889 | 0.4976 | Yes |
| 27 | DGAT2 | na | 643 | 0.854 | 0.5051 | Yes |
| 28 | SREBF1 | na | 691 | 0.823 | 0.5128 | Yes |
| 29 | APOA4 | na | 733 | 0.797 | 0.5205 | Yes |
| 30 | FGF1 | na | 971 | 0.684 | 0.5102 | Yes |
| 31 | NPC1 | na | 1035 | 0.659 | 0.5142 | Yes |
| 32 | OSBPL5 | na | 1045 | 0.656 | 0.5226 | Yes |
| 33 | LIMA1 | na | 1076 | 0.647 | 0.5292 | Yes |
| 34 | SCD | na | 1084 | 0.644 | 0.5377 | Yes |
| 35 | MBTPS2 | na | 1196 | 0.605 | 0.5368 | Yes |
| 36 | DGKQ | na | 1245 | 0.590 | 0.5411 | Yes |
| 37 | PRKAA1 | na | 1419 | 0.542 | 0.5342 | Yes |
| 38 | TTC39B | na | 1575 | 0.502 | 0.5282 | Yes |
| 39 | SCARF1 | na | 1589 | 0.498 | 0.5341 | Yes |
| 40 | SP1 | na | 1630 | 0.488 | 0.5376 | Yes |
| 41 | TSKU | na | 1738 | 0.468 | 0.5352 | Yes |
| 42 | CUBN | na | 1763 | 0.463 | 0.5397 | Yes |
| 43 | DISP3 | na | 1775 | 0.461 | 0.5452 | Yes |
| 44 | MVD | na | 1777 | 0.461 | 0.5516 | Yes |
| 45 | LPCAT3 | na | 1853 | 0.447 | 0.5516 | No |
| 46 | CYP4V2 | na | 1980 | 0.426 | 0.5470 | No |
| 47 | ACAT2 | na | 2179 | 0.392 | 0.5358 | No |
| 48 | RXRA | na | 2286 | 0.377 | 0.5322 | No |
| 49 | GPAM | na | 2506 | 0.348 | 0.5187 | No |
| 50 | ELOVL6 | na | 2559 | 0.341 | 0.5191 | No |
| 51 | CLN8 | na | 2628 | 0.332 | 0.5181 | No |
| 52 | ARV1 | na | 2818 | 0.307 | 0.5065 | No |
| 53 | CYP11A1 | na | 2856 | 0.302 | 0.5076 | No |
| 54 | LBR | na | 2914 | 0.294 | 0.5070 | No |
| 55 | CH25H | na | 2984 | 0.284 | 0.5052 | No |
| 56 | ACLY | na | 3252 | 0.250 | 0.4862 | No |
| 57 | NFYA | na | 3482 | 0.223 | 0.4701 | No |
| 58 | KPNB1 | na | 3483 | 0.223 | 0.4733 | No |
| 59 | ERLIN1 | na | 3789 | 0.189 | 0.4503 | No |
| 60 | PCSK9 | na | 3963 | 0.171 | 0.4381 | No |
| 61 | LEPR | na | 4060 | 0.162 | 0.4324 | No |
| 62 | GBA2 | na | 4202 | 0.150 | 0.4226 | No |
| 63 | NFE2L1 | na | 4275 | 0.144 | 0.4186 | No |
| 64 | CLN6 | na | 4365 | 0.137 | 0.4130 | No |
| 65 | PRKAA2 | na | 4804 | 0.097 | 0.3776 | No |
| 66 | CYP46A1 | na | 4856 | 0.092 | 0.3746 | No |
| 67 | RAN | na | 5091 | 0.071 | 0.3559 | No |
| 68 | PRKAG2 | na | 5102 | 0.070 | 0.3561 | No |
| 69 | FASN | na | 5288 | 0.055 | 0.3413 | No |
| 70 | ACAA2 | na | 5307 | 0.053 | 0.3405 | No |
| 71 | APP | na | 5347 | 0.050 | 0.3379 | No |
| 72 | EPHX2 | na | 5461 | 0.040 | 0.3290 | No |
| 73 | SCARB1 | na | 5664 | 0.020 | 0.3123 | No |
| 74 | LDLRAP1 | na | 5933 | -0.002 | 0.2898 | No |
| 75 | NR0B2 | na | 6011 | -0.007 | 0.2834 | No |
| 76 | CAT | na | 6426 | -0.040 | 0.2492 | No |
| 77 | HDLBP | na | 6581 | -0.052 | 0.2370 | No |
| 78 | MVK | na | 6801 | -0.071 | 0.2196 | No |
| 79 | MBTPS1 | na | 6903 | -0.080 | 0.2122 | No |
| 80 | PON1 | na | 7018 | -0.090 | 0.2039 | No |
| 81 | FAXDC2 | na | 7179 | -0.106 | 0.1920 | No |
| 82 | PMVK | na | 7240 | -0.112 | 0.1885 | No |
| 83 | GGPS1 | na | 7444 | -0.131 | 0.1733 | No |
| 84 | SNX17 | na | 7542 | -0.138 | 0.1671 | No |
| 85 | NFYC | na | 7623 | -0.146 | 0.1624 | No |
| 86 | NR1H4 | na | 7657 | -0.148 | 0.1617 | No |
| 87 | LRP5 | na | 7897 | -0.170 | 0.1440 | No |
| 88 | ERLIN2 | na | 7983 | -0.178 | 0.1394 | No |
| 89 | FDX1 | na | 8034 | -0.183 | 0.1377 | No |
| 90 | SEC14L2 | na | 8060 | -0.186 | 0.1382 | No |
| 91 | NPC2 | na | 8394 | -0.218 | 0.1133 | No |
| 92 | FDXR | na | 9126 | -0.299 | 0.0561 | No |
| 93 | CYB5R1 | na | 9292 | -0.320 | 0.0467 | No |
| 94 | CYP3A4 | na | 9602 | -0.362 | 0.0258 | No |
| 95 | ABCA1 | na | 9675 | -0.371 | 0.0250 | No |
| 96 | CYP8B1 | na | 9820 | -0.389 | 0.0184 | No |
| 97 | STARD3 | na | 9823 | -0.389 | 0.0237 | No |
| 98 | VLDLR | na | 9840 | -0.391 | 0.0278 | No |
| 99 | SOD1 | na | 10118 | -0.432 | 0.0106 | No |
| 100 | CYP39A1 | na | 10542 | -0.507 | -0.0178 | No |
| 101 | CYB5R2 | na | 10695 | -0.537 | -0.0230 | No |
| 102 | ACADL | na | 11004 | -0.609 | -0.0404 | No |
| 103 | PPARD | na | 11032 | -0.617 | -0.0340 | No |
| 104 | ABCG1 | na | 11046 | -0.620 | -0.0263 | No |
| 105 | SCAP | na | 11086 | -0.632 | -0.0208 | No |
| 106 | AKR1D1 | na | 11209 | -0.667 | -0.0216 | No |
| 107 | SMPD1 | na | 11304 | -0.706 | -0.0196 | No |
| 108 | CYP27A1 | na | 11384 | -0.740 | -0.0159 | No |
| 109 | CFTR | na | 11758 | -1.030 | -0.0327 | No |
| 110 | INSIG2 | na | 11843 | -1.170 | -0.0234 | No |
| 111 | LCAT | na | 11849 | -1.179 | -0.0072 | No |
| 112 | MT3 | na | 11935 | -1.470 | 0.0063 | No |
Table: GSEA details [plain text format]

  

Fig 2: GOBP\_STEROL\_METABOLIC\_PROCESS      
 Blue-Pink O' Gram in the Space of the Analyzed GeneSet

  

Fig 3: GOBP\_STEROL\_METABOLIC\_PROCESS: Random ES distribution      
 Gene set null distribution of ES for **GOBP\_STEROL\_METABOLIC\_PROCESS**

  
