# Supplementary material for: Time-series transcriptome analysis identified differentially expressed genes in broiler chicken infected with mixed Eimeria species
Source: Front Genet. 2022 Aug 8;13:886781. doi: 10.3389/fgene.2022.886781 (PMC9393255; doi:10.3389/fgene.2022.886781)
Supplement: Supplementary file 2 [file DataSheet1.ZIP › 4dpi_GO.Gsea.1625071243202/GOBP_TRANSLATIONAL_INITIATION.html]

Details for gene set GOBP\_TRANSLATIONAL\_INITIATION[GSEA]

|  || Dataset | TMM\_4dpi\_gct\_format\_4dpi\_gct\_format.Class\_4dpi.cls #PC\_versus\_NC.Class\_4dpi.cls #PC\_versus\_NC\_repos |
| Phenotype | Class\_4dpi.cls#PC\_versus\_NC\_repos |
| Upregulated in class | 0 |
| GeneSet | GOBP\_TRANSLATIONAL\_INITIATION |
| Enrichment Score (ES) | -0.60085094 |
| Normalized Enrichment Score (NES) | -2.7032804 |
| Nominal p-value | 0.0 |
| FDR q-value | 0.0 |
| FWER p-Value | 0.0 |
Table: GSEA Results Summary

  

Fig 1: Enrichment plot: GOBP\_TRANSLATIONAL\_INITIATION      
 Profile of the Running ES Score & Positions of GeneSet Members on the Rank Ordered List

  

| SYMBOL | TITLE | RANK IN GENE LIST | RANK METRIC SCORE | RUNNING ES | CORE ENRICHMENT || 1 | EIF2AK2 | na | 139 | 1.499 | 0.0076 | No |
| 2 | EIF2AK3 | na | 277 | 1.204 | 0.0115 | No |
| 3 | CCL5 | na | 438 | 1.026 | 0.0112 | No |
| 4 | FMR1 | na | 647 | 0.851 | 0.0046 | No |
| 5 | EIF2AK1 | na | 997 | 0.672 | -0.0162 | No |
| 6 | CTIF | na | 1868 | 0.444 | -0.0839 | No |
| 7 | EIF2AK4 | na | 2101 | 0.406 | -0.0982 | No |
| 8 | PAIP1 | na | 2147 | 0.397 | -0.0969 | No |
| 9 | MTIF3 | na | 2222 | 0.385 | -0.0982 | No |
| 10 | HSPB1 | na | 2376 | 0.366 | -0.1064 | No |
| 11 | DHX29 | na | 2779 | 0.311 | -0.1363 | No |
| 12 | EIF4E3 | na | 2844 | 0.303 | -0.1378 | No |
| 13 | IMPACT | na | 2863 | 0.301 | -0.1354 | No |
| 14 | MIF4GD | na | 2940 | 0.290 | -0.1381 | No |
| 15 | DDX1 | na | 3186 | 0.258 | -0.1555 | No |
| 16 | EIF1AX | na | 3333 | 0.239 | -0.1647 | No |
| 17 | EIF4H | na | 3405 | 0.232 | -0.1677 | No |
| 18 | EIF2S1 | na | 4048 | 0.164 | -0.2198 | No |
| 19 | EIF2S2 | na | 4294 | 0.142 | -0.2386 | No |
| 20 | MTOR | na | 4329 | 0.140 | -0.2397 | No |
| 21 | RPS6KB1 | na | 4509 | 0.123 | -0.2532 | No |
| 22 | CDC123 | na | 4585 | 0.117 | -0.2580 | No |
| 23 | DENR | na | 4591 | 0.117 | -0.2569 | No |
| 24 | EIF5 | na | 4786 | 0.099 | -0.2720 | No |
| 25 | NCK1 | na | 4852 | 0.093 | -0.2763 | No |
| 26 | TMED2 | na | 5016 | 0.077 | -0.2890 | No |
| 27 | EIF5B | na | 5180 | 0.062 | -0.3020 | No |
| 28 | TPR | na | 5209 | 0.060 | -0.3036 | No |
| 29 | NCBP1 | na | 5782 | 0.010 | -0.3517 | No |
| 30 | EIF4G1 | na | 5833 | 0.007 | -0.3558 | No |
| 31 | GLE1 | na | 5863 | 0.005 | -0.3582 | No |
| 32 | DNAJC3 | na | 5982 | -0.005 | -0.3681 | No |
| 33 | MTFMT | na | 6096 | -0.014 | -0.3775 | No |
| 34 | YTHDF3 | na | 6099 | -0.014 | -0.3775 | No |
| 35 | CSDE1 | na | 6343 | -0.033 | -0.3975 | No |
| 36 | EIF2A | na | 6388 | -0.037 | -0.4008 | No |
| 37 | BANK1 | na | 6438 | -0.042 | -0.4044 | No |
| 38 | MTIF2 | na | 6558 | -0.051 | -0.4137 | No |
| 39 | ABCE1 | na | 6592 | -0.054 | -0.4158 | No |
| 40 | EIF2B4 | na | 6614 | -0.056 | -0.4169 | No |
| 41 | EIF4G3 | na | 6699 | -0.063 | -0.4232 | No |
| 42 | DDX3X | na | 6973 | -0.087 | -0.4451 | No |
| 43 | LARP1 | na | 7036 | -0.093 | -0.4491 | No |
| 44 | EIF4E | na | 7081 | -0.096 | -0.4516 | No |
| 45 | RPS6KB2 | na | 7141 | -0.103 | -0.4552 | No |
| 46 | EIF2B3 | na | 7252 | -0.113 | -0.4631 | No |
| 47 | EIF6 | na | 7476 | -0.133 | -0.4802 | No |
| 48 | PAIP2B | na | 7566 | -0.140 | -0.4859 | No |
| 49 | UHMK1 | na | 7695 | -0.152 | -0.4947 | No |
| 50 | EIF3J | na | 7832 | -0.164 | -0.5041 | No |
| 51 | PPP1CA | na | 7964 | -0.175 | -0.5128 | No |
| 52 | KHDRBS1 | na | 8009 | -0.180 | -0.5142 | No |
| 53 | RPS23 | na | 8269 | -0.206 | -0.5334 | No |
| 54 | RPL17 | na | 8583 | -0.239 | -0.5568 | No |
| 55 | EIF4EBP1 | na | 8639 | -0.244 | -0.5583 | No |
| 56 | MCTS1 | na | 8783 | -0.260 | -0.5670 | No |
| 57 | EIF2B2 | na | 8786 | -0.260 | -0.5638 | No |
| 58 | RPS6 | na | 8904 | -0.274 | -0.5702 | No |
| 59 | EIF4G2 | na | 8993 | -0.282 | -0.5740 | No |
| 60 | KLHL25 | na | 9308 | -0.323 | -0.5963 | No |
| 61 | RPL36 | na | 9363 | -0.330 | -0.5966 | Yes |
| 62 | EIF4E2 | na | 9414 | -0.338 | -0.5965 | Yes |
| 63 | PPP1R15B | na | 9416 | -0.338 | -0.5922 | Yes |
| 64 | EIF2D | na | 9431 | -0.340 | -0.5890 | Yes |
| 65 | EIF2B5 | na | 9500 | -0.348 | -0.5903 | Yes |
| 66 | UBA52 | na | 9532 | -0.353 | -0.5883 | Yes |
| 67 | ATF4 | na | 9591 | -0.361 | -0.5886 | Yes |
| 68 | METTL3 | na | 9660 | -0.369 | -0.5896 | Yes |
| 69 | RPL38 | na | 9661 | -0.369 | -0.5848 | Yes |
| 70 | RPS24 | na | 9689 | -0.372 | -0.5823 | Yes |
| 71 | EIF4EBP2 | na | 9692 | -0.373 | -0.5777 | Yes |
| 72 | NCBP2 | na | 9714 | -0.376 | -0.5746 | Yes |
| 73 | POLR2D | na | 9821 | -0.389 | -0.5785 | Yes |
| 74 | EIF1 | na | 9882 | -0.395 | -0.5785 | Yes |
| 75 | HABP4 | na | 9973 | -0.410 | -0.5808 | Yes |
| 76 | RPLP2 | na | 10048 | -0.421 | -0.5817 | Yes |
| 77 | RPL37 | na | 10057 | -0.423 | -0.5769 | Yes |
| 78 | COPS5 | na | 10078 | -0.426 | -0.5731 | Yes |
| 79 | EIF2B1 | na | 10172 | -0.440 | -0.5753 | Yes |
| 80 | EIF3I | na | 10227 | -0.450 | -0.5740 | Yes |
| 81 | RPS8 | na | 10245 | -0.452 | -0.5697 | Yes |
| 82 | RPL27 | na | 10333 | -0.467 | -0.5710 | Yes |
| 83 | ALKBH1 | na | 10396 | -0.479 | -0.5701 | Yes |
| 84 | EIF1B | na | 10404 | -0.479 | -0.5645 | Yes |
| 85 | YTHDF1 | na | 10436 | -0.486 | -0.5608 | Yes |
| 86 | EIF4A2 | na | 10458 | -0.490 | -0.5563 | Yes |
| 87 | DHX33 | na | 10480 | -0.493 | -0.5517 | Yes |
| 88 | RPS28 | na | 10524 | -0.504 | -0.5489 | Yes |
| 89 | RPL30 | na | 10537 | -0.506 | -0.5434 | Yes |
| 90 | YTHDF2 | na | 10549 | -0.507 | -0.5378 | Yes |
| 91 | RPL22 | na | 10554 | -0.508 | -0.5316 | Yes |
| 92 | EIF3A | na | 10585 | -0.517 | -0.5275 | Yes |
| 93 | RPL36A | na | 10605 | -0.520 | -0.5224 | Yes |
| 94 | RPL14 | na | 10662 | -0.531 | -0.5203 | Yes |
| 95 | RPL29 | na | 10742 | -0.548 | -0.5199 | Yes |
| 96 | RPL37A | na | 10838 | -0.569 | -0.5206 | Yes |
| 97 | RPS19 | na | 10840 | -0.569 | -0.5133 | Yes |
| 98 | RPS12 | na | 10875 | -0.576 | -0.5088 | Yes |
| 99 | RPL24 | na | 10877 | -0.576 | -0.5014 | Yes |
| 100 | RPL34 | na | 10933 | -0.588 | -0.4985 | Yes |
| 101 | RPL23 | na | 10981 | -0.602 | -0.4947 | Yes |
| 102 | RPL35A | na | 10982 | -0.602 | -0.4870 | Yes |
| 103 | RPS25 | na | 11017 | -0.613 | -0.4820 | Yes |
| 104 | RPS7 | na | 11068 | -0.627 | -0.4781 | Yes |
| 105 | RPL23A | na | 11076 | -0.629 | -0.4706 | Yes |
| 106 | EIF4B | na | 11083 | -0.631 | -0.4630 | Yes |
| 107 | RPL5 | na | 11121 | -0.643 | -0.4578 | Yes |
| 108 | RPS16 | na | 11138 | -0.645 | -0.4509 | Yes |
| 109 | RPL6 | na | 11150 | -0.650 | -0.4435 | Yes |
| 110 | RPL11 | na | 11195 | -0.662 | -0.4386 | Yes |
| 111 | RPS15A | na | 11196 | -0.662 | -0.4301 | Yes |
| 112 | EIF3G | na | 11205 | -0.665 | -0.4222 | Yes |
| 113 | NPM1 | na | 11221 | -0.672 | -0.4149 | Yes |
| 114 | RPLP1 | na | 11249 | -0.682 | -0.4084 | Yes |
| 115 | RPS26 | na | 11254 | -0.686 | -0.3999 | Yes |
| 116 | EIF3B | na | 11276 | -0.694 | -0.3927 | Yes |
| 117 | RPL35 | na | 11280 | -0.696 | -0.3840 | Yes |
| 118 | RPL31 | na | 11306 | -0.706 | -0.3770 | Yes |
| 119 | RPS21 | na | 11313 | -0.709 | -0.3684 | Yes |
| 120 | RPS3A | na | 11328 | -0.713 | -0.3604 | Yes |
| 121 | RPL21 | na | 11330 | -0.714 | -0.3513 | Yes |
| 122 | RPS10 | na | 11344 | -0.720 | -0.3431 | Yes |
| 123 | RPL32 | na | 11355 | -0.725 | -0.3346 | Yes |
| 124 | RPS11 | na | 11371 | -0.735 | -0.3265 | Yes |
| 125 | RPL12 | na | 11398 | -0.748 | -0.3190 | Yes |
| 126 | EIF2S3 | na | 11414 | -0.759 | -0.3105 | Yes |
| 127 | RPL7A | na | 11426 | -0.763 | -0.3016 | Yes |
| 128 | RPS15 | na | 11434 | -0.767 | -0.2923 | Yes |
| 129 | RPS27A | na | 11435 | -0.767 | -0.2825 | Yes |
| 130 | RPL15 | na | 11440 | -0.769 | -0.2729 | Yes |
| 131 | RPS29 | na | 11444 | -0.771 | -0.2633 | Yes |
| 132 | RPL18A | na | 11474 | -0.789 | -0.2555 | Yes |
| 133 | RPS14 | na | 11478 | -0.792 | -0.2456 | Yes |
| 134 | RPL7 | na | 11484 | -0.796 | -0.2358 | Yes |
| 135 | RPLP0 | na | 11488 | -0.800 | -0.2257 | Yes |
| 136 | RPL27A | na | 11507 | -0.812 | -0.2168 | Yes |
| 137 | RPL9 | na | 11521 | -0.819 | -0.2074 | Yes |
| 138 | PABPC1 | na | 11551 | -0.839 | -0.1990 | Yes |
| 139 | RPS2 | na | 11562 | -0.846 | -0.1890 | Yes |
| 140 | RPL13 | na | 11563 | -0.847 | -0.1781 | Yes |
| 141 | EIF3D | na | 11578 | -0.857 | -0.1682 | Yes |
| 142 | RPS20 | na | 11594 | -0.868 | -0.1583 | Yes |
| 143 | RPS27 | na | 11600 | -0.875 | -0.1475 | Yes |
| 144 | RPL19 | na | 11611 | -0.882 | -0.1370 | Yes |
| 145 | EIF3H | na | 11627 | -0.893 | -0.1267 | Yes |
| 146 | RPS13 | na | 11633 | -0.897 | -0.1156 | Yes |
| 147 | RPS3 | na | 11641 | -0.903 | -0.1046 | Yes |
| 148 | RPS17 | na | 11652 | -0.916 | -0.0936 | Yes |
| 149 | RPL10A | na | 11658 | -0.921 | -0.0822 | Yes |
| 150 | EIF3M | na | 11707 | -0.974 | -0.0737 | Yes |
| 151 | RPL4 | na | 11715 | -0.983 | -0.0617 | Yes |
| 152 | RPL8 | na | 11772 | -1.039 | -0.0530 | Yes |
| 153 | RPS4Y1 | na | 11774 | -1.043 | -0.0397 | Yes |
| 154 | EIF3L | na | 11808 | -1.126 | -0.0280 | Yes |
| 155 | EIF3F | na | 11816 | -1.139 | -0.0139 | Yes |
| 156 | RPL3 | na | 11817 | -1.140 | 0.0008 | Yes |
| 157 | EIF3E | na | 11859 | -1.199 | 0.0127 | Yes |
Table: GSEA details [plain text format]

  

Fig 2: GOBP\_TRANSLATIONAL\_INITIATION      
 Blue-Pink O' Gram in the Space of the Analyzed GeneSet

  

Fig 3: GOBP\_TRANSLATIONAL\_INITIATION: Random ES distribution      
 Gene set null distribution of ES for **GOBP\_TRANSLATIONAL\_INITIATION**

  
