# Supplementary material for: Time-series transcriptome analysis identified differentially expressed genes in broiler chicken infected with mixed Eimeria species
Source: Front Genet. 2022 Aug 8;13:886781. doi: 10.3389/fgene.2022.886781 (PMC9393255; doi:10.3389/fgene.2022.886781)
Supplement: Supplementary file 2 [file DataSheet1.ZIP › 4dpi_GO.Gsea.1625071243202/GOBP_VIRAL_GENE_EXPRESSION.html]

Details for gene set GOBP\_VIRAL\_GENE\_EXPRESSION[GSEA]

|  || Dataset | TMM\_4dpi\_gct\_format\_4dpi\_gct\_format.Class\_4dpi.cls #PC\_versus\_NC.Class\_4dpi.cls #PC\_versus\_NC\_repos |
| Phenotype | Class\_4dpi.cls#PC\_versus\_NC\_repos |
| Upregulated in class | 0 |
| GeneSet | GOBP\_VIRAL\_GENE\_EXPRESSION |
| Enrichment Score (ES) | -0.58150893 |
| Normalized Enrichment Score (NES) | -2.621212 |
| Nominal p-value | 0.0 |
| FDR q-value | 0.0 |
| FWER p-Value | 0.0 |
Table: GSEA Results Summary

  

Fig 1: Enrichment plot: GOBP\_VIRAL\_GENE\_EXPRESSION      
 Profile of the Running ES Score & Positions of GeneSet Members on the Rank Ordered List

  

| SYMBOL | TITLE | RANK IN GENE LIST | RANK METRIC SCORE | RUNNING ES | CORE ENRICHMENT || 1 | CCL5 | na | 438 | 1.026 | -0.0227 | No |
| 2 | JUN | na | 912 | 0.705 | -0.0528 | No |
| 3 | NUP37 | na | 1504 | 0.520 | -0.0955 | No |
| 4 | SP1 | na | 1630 | 0.488 | -0.0992 | No |
| 5 | NDC1 | na | 1670 | 0.480 | -0.0959 | No |
| 6 | CCL4 | na | 1945 | 0.433 | -0.1130 | No |
| 7 | INPP5K | na | 1977 | 0.427 | -0.1096 | No |
| 8 | EIF2AK4 | na | 2101 | 0.406 | -0.1144 | No |
| 9 | PTBP1 | na | 2406 | 0.360 | -0.1350 | No |
| 10 | POLR2J | na | 2459 | 0.353 | -0.1345 | No |
| 11 | NUP98 | na | 2694 | 0.324 | -0.1498 | No |
| 12 | TARDBP | na | 2717 | 0.320 | -0.1472 | No |
| 13 | TRIM8 | na | 2825 | 0.305 | -0.1519 | No |
| 14 | POLR2K | na | 2849 | 0.303 | -0.1497 | No |
| 15 | SEC13 | na | 3316 | 0.241 | -0.1857 | No |
| 16 | SPCS1 | na | 3477 | 0.224 | -0.1960 | No |
| 17 | NUP133 | na | 3648 | 0.202 | -0.2076 | No |
| 18 | NUP107 | na | 3837 | 0.183 | -0.2209 | No |
| 19 | RSF1 | na | 3875 | 0.180 | -0.2215 | No |
| 20 | NUCKS1 | na | 4295 | 0.142 | -0.2549 | No |
| 21 | CCNT2 | na | 4438 | 0.130 | -0.2651 | No |
| 22 | NUP155 | na | 4450 | 0.129 | -0.2642 | No |
| 23 | USF1 | na | 4497 | 0.124 | -0.2664 | No |
| 24 | DENR | na | 4591 | 0.117 | -0.2726 | No |
| 25 | PCBP2 | na | 4714 | 0.105 | -0.2814 | No |
| 26 | NELFE | na | 5000 | 0.078 | -0.3044 | No |
| 27 | NUP85 | na | 5017 | 0.077 | -0.3047 | No |
| 28 | TPR | na | 5209 | 0.060 | -0.3200 | No |
| 29 | LARP7 | na | 5321 | 0.052 | -0.3286 | No |
| 30 | POLR2F | na | 5669 | 0.020 | -0.3576 | No |
| 31 | MDFIC | na | 5711 | 0.016 | -0.3608 | No |
| 32 | TFAP4 | na | 5766 | 0.012 | -0.3652 | No |
| 33 | ZNF639 | na | 5774 | 0.011 | -0.3657 | No |
| 34 | UBP1 | na | 6023 | -0.008 | -0.3865 | No |
| 35 | EP300 | na | 6138 | -0.018 | -0.3958 | No |
| 36 | NUP50 | na | 6200 | -0.023 | -0.4007 | No |
| 37 | AAAS | na | 6302 | -0.030 | -0.4088 | No |
| 38 | CSDE1 | na | 6343 | -0.033 | -0.4117 | No |
| 39 | NUP58 | na | 6350 | -0.034 | -0.4117 | No |
| 40 | SSB | na | 6362 | -0.035 | -0.4122 | No |
| 41 | GTF2F2 | na | 6420 | -0.040 | -0.4164 | No |
| 42 | NUP205 | na | 6527 | -0.049 | -0.4247 | No |
| 43 | PSMC3 | na | 6671 | -0.060 | -0.4359 | No |
| 44 | POLR2I | na | 6763 | -0.068 | -0.4427 | No |
| 45 | POLR2L | na | 6820 | -0.072 | -0.4464 | No |
| 46 | GTF2B | na | 6845 | -0.074 | -0.4474 | No |
| 47 | NUP62 | na | 6862 | -0.075 | -0.4477 | No |
| 48 | NUP160 | na | 6915 | -0.081 | -0.4510 | No |
| 49 | POLR2H | na | 6975 | -0.087 | -0.4547 | No |
| 50 | SUPT5H | na | 7111 | -0.099 | -0.4647 | No |
| 51 | SMARCA4 | na | 7296 | -0.117 | -0.4786 | No |
| 52 | NELFB | na | 7330 | -0.120 | -0.4798 | No |
| 53 | NELFA | na | 7343 | -0.122 | -0.4791 | No |
| 54 | SNW1 | na | 7382 | -0.125 | -0.4805 | No |
| 55 | NUP88 | na | 7451 | -0.131 | -0.4845 | No |
| 56 | NUP93 | na | 8014 | -0.180 | -0.5294 | No |
| 57 | SPCS3 | na | 8059 | -0.185 | -0.5305 | No |
| 58 | NUP54 | na | 8176 | -0.196 | -0.5376 | No |
| 59 | RPS23 | na | 8269 | -0.206 | -0.5425 | No |
| 60 | CCNT1 | na | 8271 | -0.206 | -0.5397 | No |
| 61 | NELFCD | na | 8369 | -0.216 | -0.5449 | No |
| 62 | RAE1 | na | 8423 | -0.220 | -0.5463 | No |
| 63 | RPL17 | na | 8583 | -0.239 | -0.5564 | No |
| 64 | TRIM14 | na | 8702 | -0.251 | -0.5629 | No |
| 65 | POLR2B | na | 8782 | -0.260 | -0.5659 | No |
| 66 | MCTS1 | na | 8783 | -0.260 | -0.5623 | No |
| 67 | CTDP1 | na | 8837 | -0.267 | -0.5631 | No |
| 68 | NUP153 | na | 8890 | -0.272 | -0.5637 | No |
| 69 | RPS6 | na | 8904 | -0.274 | -0.5610 | No |
| 70 | MID2 | na | 8947 | -0.278 | -0.5607 | No |
| 71 | NUP42 | na | 9195 | -0.308 | -0.5772 | Yes |
| 72 | NUP214 | na | 9212 | -0.310 | -0.5743 | Yes |
| 73 | HMGA2 | na | 9234 | -0.313 | -0.5717 | Yes |
| 74 | SEH1L | na | 9274 | -0.318 | -0.5706 | Yes |
| 75 | FURIN | na | 9299 | -0.321 | -0.5681 | Yes |
| 76 | RPL36 | na | 9363 | -0.330 | -0.5688 | Yes |
| 77 | NUP210 | na | 9373 | -0.332 | -0.5650 | Yes |
| 78 | EIF2D | na | 9431 | -0.340 | -0.5651 | Yes |
| 79 | NUP188 | na | 9441 | -0.341 | -0.5611 | Yes |
| 80 | POLR2E | na | 9459 | -0.342 | -0.5578 | Yes |
| 81 | SMARCB1 | na | 9471 | -0.344 | -0.5539 | Yes |
| 82 | UBA52 | na | 9532 | -0.353 | -0.5541 | Yes |
| 83 | TAF11 | na | 9553 | -0.357 | -0.5508 | Yes |
| 84 | RPL38 | na | 9661 | -0.369 | -0.5547 | Yes |
| 85 | POM121C | na | 9676 | -0.371 | -0.5508 | Yes |
| 86 | RPS24 | na | 9689 | -0.372 | -0.5466 | Yes |
| 87 | POLR2D | na | 9821 | -0.389 | -0.5522 | Yes |
| 88 | RRP1B | na | 9921 | -0.401 | -0.5550 | Yes |
| 89 | RPLP2 | na | 10048 | -0.421 | -0.5598 | Yes |
| 90 | RPL37 | na | 10057 | -0.423 | -0.5546 | Yes |
| 91 | RPS8 | na | 10245 | -0.452 | -0.5641 | Yes |
| 92 | RPL27 | na | 10333 | -0.467 | -0.5650 | Yes |
| 93 | HDAC1 | na | 10365 | -0.472 | -0.5610 | Yes |
| 94 | CDK9 | na | 10367 | -0.472 | -0.5545 | Yes |
| 95 | RPS28 | na | 10524 | -0.504 | -0.5607 | Yes |
| 96 | RPL30 | na | 10537 | -0.506 | -0.5547 | Yes |
| 97 | RPL22 | na | 10554 | -0.508 | -0.5490 | Yes |
| 98 | EIF3A | na | 10585 | -0.517 | -0.5443 | Yes |
| 99 | RPL36A | na | 10605 | -0.520 | -0.5387 | Yes |
| 100 | LEF1 | na | 10632 | -0.526 | -0.5336 | Yes |
| 101 | RPL14 | na | 10662 | -0.531 | -0.5286 | Yes |
| 102 | RPL29 | na | 10742 | -0.548 | -0.5277 | Yes |
| 103 | RPL37A | na | 10838 | -0.569 | -0.5278 | Yes |
| 104 | RPS19 | na | 10840 | -0.569 | -0.5200 | Yes |
| 105 | POLR2C | na | 10864 | -0.575 | -0.5139 | Yes |
| 106 | RPS12 | na | 10875 | -0.576 | -0.5068 | Yes |
| 107 | RPL24 | na | 10877 | -0.576 | -0.4988 | Yes |
| 108 | RPL34 | na | 10933 | -0.588 | -0.4953 | Yes |
| 109 | RPL23 | na | 10981 | -0.602 | -0.4909 | Yes |
| 110 | RPL35A | na | 10982 | -0.602 | -0.4825 | Yes |
| 111 | RPS25 | na | 11017 | -0.613 | -0.4769 | Yes |
| 112 | RPS7 | na | 11068 | -0.627 | -0.4724 | Yes |
| 113 | RPL23A | na | 11076 | -0.629 | -0.4642 | Yes |
| 114 | RPL5 | na | 11121 | -0.643 | -0.4590 | Yes |
| 115 | RPS16 | na | 11138 | -0.645 | -0.4514 | Yes |
| 116 | RPL6 | na | 11150 | -0.650 | -0.4433 | Yes |
| 117 | SUPT4H1 | na | 11188 | -0.660 | -0.4373 | Yes |
| 118 | RPL11 | na | 11195 | -0.662 | -0.4286 | Yes |
| 119 | RPS15A | na | 11196 | -0.662 | -0.4194 | Yes |
| 120 | EIF3G | na | 11205 | -0.665 | -0.4108 | Yes |
| 121 | RPLP1 | na | 11249 | -0.682 | -0.4049 | Yes |
| 122 | RPS26 | na | 11254 | -0.686 | -0.3957 | Yes |
| 123 | EIF3B | na | 11276 | -0.694 | -0.3879 | Yes |
| 124 | RPL35 | na | 11280 | -0.696 | -0.3785 | Yes |
| 125 | RPL31 | na | 11306 | -0.706 | -0.3708 | Yes |
| 126 | RPS21 | na | 11313 | -0.709 | -0.3614 | Yes |
| 127 | RPS3A | na | 11328 | -0.713 | -0.3527 | Yes |
| 128 | RPL21 | na | 11330 | -0.714 | -0.3428 | Yes |
| 129 | RPS10 | na | 11344 | -0.720 | -0.3339 | Yes |
| 130 | RPL32 | na | 11355 | -0.725 | -0.3247 | Yes |
| 131 | RPS11 | na | 11371 | -0.735 | -0.3157 | Yes |
| 132 | RPL12 | na | 11398 | -0.748 | -0.3075 | Yes |
| 133 | RPL7A | na | 11426 | -0.763 | -0.2992 | Yes |
| 134 | RPS15 | na | 11434 | -0.767 | -0.2891 | Yes |
| 135 | RPS27A | na | 11435 | -0.767 | -0.2785 | Yes |
| 136 | RPL15 | na | 11440 | -0.769 | -0.2681 | Yes |
| 137 | RPS29 | na | 11444 | -0.771 | -0.2577 | Yes |
| 138 | RPL18A | na | 11474 | -0.789 | -0.2492 | Yes |
| 139 | RPS14 | na | 11478 | -0.792 | -0.2384 | Yes |
| 140 | RPL7 | na | 11484 | -0.796 | -0.2278 | Yes |
| 141 | RPLP0 | na | 11488 | -0.800 | -0.2169 | Yes |
| 142 | TRIM62 | na | 11497 | -0.806 | -0.2064 | Yes |
| 143 | RPL27A | na | 11507 | -0.812 | -0.1959 | Yes |
| 144 | RPL9 | na | 11521 | -0.819 | -0.1856 | Yes |
| 145 | RPS2 | na | 11562 | -0.846 | -0.1772 | Yes |
| 146 | RPL13 | na | 11563 | -0.847 | -0.1654 | Yes |
| 147 | EIF3D | na | 11578 | -0.857 | -0.1547 | Yes |
| 148 | RPS20 | na | 11594 | -0.868 | -0.1439 | Yes |
| 149 | RPS27 | na | 11600 | -0.875 | -0.1321 | Yes |
| 150 | RPL19 | na | 11611 | -0.882 | -0.1207 | Yes |
| 151 | RPS13 | na | 11633 | -0.897 | -0.1100 | Yes |
| 152 | RPS3 | na | 11641 | -0.903 | -0.0981 | Yes |
| 153 | RPS17 | na | 11652 | -0.916 | -0.0862 | Yes |
| 154 | RPL10A | na | 11658 | -0.921 | -0.0738 | Yes |
| 155 | TRIM13 | na | 11669 | -0.936 | -0.0616 | Yes |
| 156 | RPL4 | na | 11715 | -0.983 | -0.0518 | Yes |
| 157 | RPL8 | na | 11772 | -1.039 | -0.0421 | Yes |
| 158 | RPS4Y1 | na | 11774 | -1.043 | -0.0277 | Yes |
| 159 | EIF3L | na | 11808 | -1.126 | -0.0148 | Yes |
| 160 | EIF3F | na | 11816 | -1.139 | 0.0004 | Yes |
| 161 | RPL3 | na | 11817 | -1.140 | 0.0163 | Yes |
Table: GSEA details [plain text format]

  

Fig 2: GOBP\_VIRAL\_GENE\_EXPRESSION      
 Blue-Pink O' Gram in the Space of the Analyzed GeneSet

  

Fig 3: GOBP\_VIRAL\_GENE\_EXPRESSION: Random ES distribution      
 Gene set null distribution of ES for **GOBP\_VIRAL\_GENE\_EXPRESSION**

  
