# Supplementary material for: Time-series transcriptome analysis identified differentially expressed genes in broiler chicken infected with mixed Eimeria species
Source: Front Genet. 2022 Aug 8;13:886781. doi: 10.3389/fgene.2022.886781 (PMC9393255; doi:10.3389/fgene.2022.886781)
Supplement: Supplementary file 2 [file DataSheet1.ZIP › 4dpi_GO.Gsea.1625071243202/GOCC_CORNIFIED_ENVELOPE.html]

Details for gene set GOCC\_CORNIFIED\_ENVELOPE[GSEA]

|  || Dataset | TMM\_4dpi\_gct\_format\_4dpi\_gct\_format.Class\_4dpi.cls #PC\_versus\_NC.Class\_4dpi.cls #PC\_versus\_NC\_repos |
| Phenotype | Class\_4dpi.cls#PC\_versus\_NC\_repos |
| Upregulated in class | 1 |
| GeneSet | GOCC\_CORNIFIED\_ENVELOPE |
| Enrichment Score (ES) | 0.8516696 |
| Normalized Enrichment Score (NES) | 2.1047106 |
| Nominal p-value | 0.0 |
| FDR q-value | 0.0025563755 |
| FWER p-Value | 0.0474 |
Table: GSEA Results Summary

  

Fig 1: Enrichment plot: GOCC\_CORNIFIED\_ENVELOPE      
 Profile of the Running ES Score & Positions of GeneSet Members on the Rank Ordered List

  

| SYMBOL | TITLE | RANK IN GENE LIST | RANK METRIC SCORE | RUNNING ES | CORE ENRICHMENT || 1 | PKP2 | na | 7 | 2.810 | 0.2269 | Yes |
| 2 | DSC1 | na | 8 | 2.652 | 0.4416 | Yes |
| 3 | DSP | na | 401 | 1.061 | 0.4948 | Yes |
| 4 | KAZN | na | 484 | 0.980 | 0.5673 | Yes |
| 5 | EVPL | na | 743 | 0.791 | 0.6098 | Yes |
| 6 | CSTA | na | 779 | 0.769 | 0.6691 | Yes |
| 7 | KRT10 | na | 930 | 0.698 | 0.7131 | Yes |
| 8 | DSG2 | na | 985 | 0.678 | 0.7635 | Yes |
| 9 | ANXA1 | na | 1060 | 0.651 | 0.8101 | Yes |
| 10 | PPL | na | 1389 | 0.551 | 0.8273 | Yes |
| 11 | PKP4 | na | 1583 | 0.500 | 0.8517 | Yes |
| 12 | JUP | na | 3549 | 0.213 | 0.7051 | No |
Table: GSEA details [plain text format]

  

Fig 2: GOCC\_CORNIFIED\_ENVELOPE      
 Blue-Pink O' Gram in the Space of the Analyzed GeneSet

  

Fig 3: GOCC\_CORNIFIED\_ENVELOPE: Random ES distribution      
 Gene set null distribution of ES for **GOCC\_CORNIFIED\_ENVELOPE**

  
