# Supplementary material for: Time-series transcriptome analysis identified differentially expressed genes in broiler chicken infected with mixed Eimeria species
Source: Front Genet. 2022 Aug 8;13:886781. doi: 10.3389/fgene.2022.886781 (PMC9393255; doi:10.3389/fgene.2022.886781)
Supplement: Supplementary file 2 [file DataSheet1.ZIP › 4dpi_GO.Gsea.1625071243202/GOCC_CYTOSOLIC_LARGE_RIBOSOMAL_SUBUNIT.html]

Details for gene set GOCC\_CYTOSOLIC\_LARGE\_RIBOSOMAL\_SUBUNIT[GSEA]

|  || Dataset | TMM\_4dpi\_gct\_format\_4dpi\_gct\_format.Class\_4dpi.cls #PC\_versus\_NC.Class\_4dpi.cls #PC\_versus\_NC\_repos |
| Phenotype | Class\_4dpi.cls#PC\_versus\_NC\_repos |
| Upregulated in class | 0 |
| GeneSet | GOCC\_CYTOSOLIC\_LARGE\_RIBOSOMAL\_SUBUNIT |
| Enrichment Score (ES) | -0.7908055 |
| Normalized Enrichment Score (NES) | -2.8141828 |
| Nominal p-value | 0.0 |
| FDR q-value | 0.0 |
| FWER p-Value | 0.0 |
Table: GSEA Results Summary

  

Fig 1: Enrichment plot: GOCC\_CYTOSOLIC\_LARGE\_RIBOSOMAL\_SUBUNIT      
 Profile of the Running ES Score & Positions of GeneSet Members on the Rank Ordered List

  

| SYMBOL | TITLE | RANK IN GENE LIST | RANK METRIC SCORE | RUNNING ES | CORE ENRICHMENT || 1 | RSL24D1 | na | 6280 | -0.029 | -0.5237 | No |
| 2 | MRPL1 | na | 7631 | -0.147 | -0.6312 | No |
| 3 | RPL7L1 | na | 8437 | -0.222 | -0.6905 | No |
| 4 | RPL17 | na | 8583 | -0.239 | -0.6939 | No |
| 5 | RPL36 | na | 9363 | -0.330 | -0.7471 | No |
| 6 | RPL38 | na | 9661 | -0.369 | -0.7586 | No |
| 7 | RPLP2 | na | 10048 | -0.421 | -0.7756 | Yes |
| 8 | RPL37 | na | 10057 | -0.423 | -0.7609 | Yes |
| 9 | RPL27 | na | 10333 | -0.467 | -0.7670 | Yes |
| 10 | RPL30 | na | 10537 | -0.506 | -0.7657 | Yes |
| 11 | RPL22 | na | 10554 | -0.508 | -0.7486 | Yes |
| 12 | RPL36A | na | 10605 | -0.520 | -0.7340 | Yes |
| 13 | RPL39L | na | 10613 | -0.521 | -0.7157 | Yes |
| 14 | RPL14 | na | 10662 | -0.531 | -0.7005 | Yes |
| 15 | RPL29 | na | 10742 | -0.548 | -0.6873 | Yes |
| 16 | RPL37A | na | 10838 | -0.569 | -0.6746 | Yes |
| 17 | RPL24 | na | 10877 | -0.576 | -0.6569 | Yes |
| 18 | RPL34 | na | 10933 | -0.588 | -0.6402 | Yes |
| 19 | RPL23 | na | 10981 | -0.602 | -0.6224 | Yes |
| 20 | RPL35A | na | 10982 | -0.602 | -0.6006 | Yes |
| 21 | RPL23A | na | 11076 | -0.629 | -0.5856 | Yes |
| 22 | RPL5 | na | 11121 | -0.643 | -0.5660 | Yes |
| 23 | RPL6 | na | 11150 | -0.650 | -0.5449 | Yes |
| 24 | RPL11 | na | 11195 | -0.662 | -0.5246 | Yes |
| 25 | RPLP1 | na | 11249 | -0.682 | -0.5043 | Yes |
| 26 | RPL35 | na | 11280 | -0.696 | -0.4816 | Yes |
| 27 | RPL26L1 | na | 11283 | -0.698 | -0.4565 | Yes |
| 28 | RPL31 | na | 11306 | -0.706 | -0.4328 | Yes |
| 29 | RPL21 | na | 11330 | -0.714 | -0.4089 | Yes |
| 30 | RPL32 | na | 11355 | -0.725 | -0.3847 | Yes |
| 31 | RPL12 | na | 11398 | -0.748 | -0.3611 | Yes |
| 32 | RPL7A | na | 11426 | -0.763 | -0.3357 | Yes |
| 33 | RPL15 | na | 11440 | -0.769 | -0.3090 | Yes |
| 34 | RPL18A | na | 11474 | -0.789 | -0.2832 | Yes |
| 35 | RPL7 | na | 11484 | -0.796 | -0.2551 | Yes |
| 36 | RPLP0 | na | 11488 | -0.800 | -0.2264 | Yes |
| 37 | RPL27A | na | 11507 | -0.812 | -0.1986 | Yes |
| 38 | RPL9 | na | 11521 | -0.819 | -0.1700 | Yes |
| 39 | RPL13 | na | 11563 | -0.847 | -0.1428 | Yes |
| 40 | RPL19 | na | 11611 | -0.882 | -0.1148 | Yes |
| 41 | RPL10A | na | 11658 | -0.921 | -0.0853 | Yes |
| 42 | RPL4 | na | 11715 | -0.983 | -0.0544 | Yes |
| 43 | RPL8 | na | 11772 | -1.039 | -0.0215 | Yes |
| 44 | RPL3 | na | 11817 | -1.140 | 0.0161 | Yes |
Table: GSEA details [plain text format]

  

Fig 2: GOCC\_CYTOSOLIC\_LARGE\_RIBOSOMAL\_SUBUNIT      
 Blue-Pink O' Gram in the Space of the Analyzed GeneSet

  

Fig 3: GOCC\_CYTOSOLIC\_LARGE\_RIBOSOMAL\_SUBUNIT: Random ES distribution      
 Gene set null distribution of ES for **GOCC\_CYTOSOLIC\_LARGE\_RIBOSOMAL\_SUBUNIT**

  
