# Supplementary material for: Time-series transcriptome analysis identified differentially expressed genes in broiler chicken infected with mixed Eimeria species
Source: Front Genet. 2022 Aug 8;13:886781. doi: 10.3389/fgene.2022.886781 (PMC9393255; doi:10.3389/fgene.2022.886781)
Supplement: Supplementary file 2 [file DataSheet1.ZIP › 4dpi_GO.Gsea.1625071243202/GOCC_CYTOSOLIC_RIBOSOME.html]

Details for gene set GOCC\_CYTOSOLIC\_RIBOSOME[GSEA]

|  || Dataset | TMM\_4dpi\_gct\_format\_4dpi\_gct\_format.Class\_4dpi.cls #PC\_versus\_NC.Class\_4dpi.cls #PC\_versus\_NC\_repos |
| Phenotype | Class\_4dpi.cls#PC\_versus\_NC\_repos |
| Upregulated in class | 0 |
| GeneSet | GOCC\_CYTOSOLIC\_RIBOSOME |
| Enrichment Score (ES) | -0.73408496 |
| Normalized Enrichment Score (NES) | -2.999865 |
| Nominal p-value | 0.0 |
| FDR q-value | 0.0 |
| FWER p-Value | 0.0 |
Table: GSEA Results Summary

  

Fig 1: Enrichment plot: GOCC\_CYTOSOLIC\_RIBOSOME      
 Profile of the Running ES Score & Positions of GeneSet Members on the Rank Ordered List

  

| SYMBOL | TITLE | RANK IN GENE LIST | RANK METRIC SCORE | RUNNING ES | CORE ENRICHMENT || 1 | HBA1 | na | 1865 | 0.445 | -0.1476 | No |
| 2 | APOD | na | 1919 | 0.437 | -0.1433 | No |
| 3 | EIF2AK4 | na | 2101 | 0.406 | -0.1505 | No |
| 4 | DHX29 | na | 2779 | 0.311 | -0.2011 | No |
| 5 | RPS27L | na | 5614 | 0.024 | -0.4382 | No |
| 6 | RSL24D1 | na | 6280 | -0.029 | -0.4934 | No |
| 7 | EIF2A | na | 6388 | -0.037 | -0.5016 | No |
| 8 | DDX3X | na | 6973 | -0.087 | -0.5489 | No |
| 9 | MRPS11 | na | 7235 | -0.112 | -0.5685 | No |
| 10 | MRPL1 | na | 7631 | -0.147 | -0.5987 | No |
| 11 | RPS23 | na | 8269 | -0.206 | -0.6481 | No |
| 12 | RPL7L1 | na | 8437 | -0.222 | -0.6577 | No |
| 13 | RPL17 | na | 8583 | -0.239 | -0.6651 | No |
| 14 | MCTS1 | na | 8783 | -0.260 | -0.6766 | No |
| 15 | RPS6 | na | 8904 | -0.274 | -0.6813 | No |
| 16 | RPL36 | na | 9363 | -0.330 | -0.7131 | No |
| 17 | EIF2D | na | 9431 | -0.340 | -0.7120 | No |
| 18 | UBA52 | na | 9532 | -0.353 | -0.7134 | No |
| 19 | RPL38 | na | 9661 | -0.369 | -0.7168 | No |
| 20 | RPS24 | na | 9689 | -0.372 | -0.7117 | No |
| 21 | NUFIP1 | na | 9750 | -0.379 | -0.7092 | No |
| 22 | RPLP2 | na | 10048 | -0.421 | -0.7257 | Yes |
| 23 | RPL37 | na | 10057 | -0.423 | -0.7180 | Yes |
| 24 | LARP4 | na | 10149 | -0.437 | -0.7170 | Yes |
| 25 | RPS8 | na | 10245 | -0.452 | -0.7160 | Yes |
| 26 | RPL27 | na | 10333 | -0.467 | -0.7140 | Yes |
| 27 | RPS28 | na | 10524 | -0.504 | -0.7199 | Yes |
| 28 | RPL30 | na | 10537 | -0.506 | -0.7109 | Yes |
| 29 | RPL22 | na | 10554 | -0.508 | -0.7022 | Yes |
| 30 | RPL36A | na | 10605 | -0.520 | -0.6961 | Yes |
| 31 | RPL39L | na | 10613 | -0.521 | -0.6863 | Yes |
| 32 | RPL14 | na | 10662 | -0.531 | -0.6798 | Yes |
| 33 | RPL29 | na | 10742 | -0.548 | -0.6756 | Yes |
| 34 | RPL37A | na | 10838 | -0.569 | -0.6722 | Yes |
| 35 | RPS19 | na | 10840 | -0.569 | -0.6610 | Yes |
| 36 | RPS12 | na | 10875 | -0.576 | -0.6524 | Yes |
| 37 | RPL24 | na | 10877 | -0.576 | -0.6411 | Yes |
| 38 | RPL34 | na | 10933 | -0.588 | -0.6340 | Yes |
| 39 | RPL23 | na | 10981 | -0.602 | -0.6260 | Yes |
| 40 | RPL35A | na | 10982 | -0.602 | -0.6141 | Yes |
| 41 | RPS25 | na | 11017 | -0.613 | -0.6048 | Yes |
| 42 | RPS7 | na | 11068 | -0.627 | -0.5965 | Yes |
| 43 | RPL23A | na | 11076 | -0.629 | -0.5847 | Yes |
| 44 | RPL5 | na | 11121 | -0.643 | -0.5756 | Yes |
| 45 | PPARGC1A | na | 11134 | -0.645 | -0.5638 | Yes |
| 46 | RPS16 | na | 11138 | -0.645 | -0.5513 | Yes |
| 47 | RPL6 | na | 11150 | -0.650 | -0.5393 | Yes |
| 48 | RPL11 | na | 11195 | -0.662 | -0.5299 | Yes |
| 49 | RPS15A | na | 11196 | -0.662 | -0.5167 | Yes |
| 50 | RPLP1 | na | 11249 | -0.682 | -0.5075 | Yes |
| 51 | RPS26 | na | 11254 | -0.686 | -0.4943 | Yes |
| 52 | RPL35 | na | 11280 | -0.696 | -0.4826 | Yes |
| 53 | RPL26L1 | na | 11283 | -0.698 | -0.4689 | Yes |
| 54 | RPL31 | na | 11306 | -0.706 | -0.4567 | Yes |
| 55 | RPS21 | na | 11313 | -0.709 | -0.4432 | Yes |
| 56 | RPS3A | na | 11328 | -0.713 | -0.4302 | Yes |
| 57 | RPL21 | na | 11330 | -0.714 | -0.4161 | Yes |
| 58 | RPS10 | na | 11344 | -0.720 | -0.4029 | Yes |
| 59 | RPL32 | na | 11355 | -0.725 | -0.3894 | Yes |
| 60 | RPS11 | na | 11371 | -0.735 | -0.3761 | Yes |
| 61 | RPL12 | na | 11398 | -0.748 | -0.3634 | Yes |
| 62 | RPL7A | na | 11426 | -0.763 | -0.3505 | Yes |
| 63 | RPS15 | na | 11434 | -0.767 | -0.3359 | Yes |
| 64 | RPS27A | na | 11435 | -0.767 | -0.3207 | Yes |
| 65 | RPL15 | na | 11440 | -0.769 | -0.3058 | Yes |
| 66 | RPS29 | na | 11444 | -0.771 | -0.2907 | Yes |
| 67 | RPL18A | na | 11474 | -0.789 | -0.2775 | Yes |
| 68 | RPS14 | na | 11478 | -0.792 | -0.2620 | Yes |
| 69 | RPL7 | na | 11484 | -0.796 | -0.2467 | Yes |
| 70 | RPLP0 | na | 11488 | -0.800 | -0.2311 | Yes |
| 71 | RPL27A | na | 11507 | -0.812 | -0.2165 | Yes |
| 72 | RPL9 | na | 11521 | -0.819 | -0.2013 | Yes |
| 73 | RPS2 | na | 11562 | -0.846 | -0.1879 | Yes |
| 74 | RPL13 | na | 11563 | -0.847 | -0.1711 | Yes |
| 75 | RPS20 | na | 11594 | -0.868 | -0.1564 | Yes |
| 76 | RPS27 | na | 11600 | -0.875 | -0.1394 | Yes |
| 77 | RPL19 | na | 11611 | -0.882 | -0.1228 | Yes |
| 78 | RPS13 | na | 11633 | -0.897 | -0.1067 | Yes |
| 79 | RPS3 | na | 11641 | -0.903 | -0.0894 | Yes |
| 80 | RPS17 | na | 11652 | -0.916 | -0.0721 | Yes |
| 81 | RPL10A | na | 11658 | -0.921 | -0.0542 | Yes |
| 82 | RPL4 | na | 11715 | -0.983 | -0.0394 | Yes |
| 83 | RPL8 | na | 11772 | -1.039 | -0.0235 | Yes |
| 84 | RPS4Y1 | na | 11774 | -1.043 | -0.0029 | Yes |
| 85 | RPL3 | na | 11817 | -1.140 | 0.0162 | Yes |
Table: GSEA details [plain text format]

  

Fig 2: GOCC\_CYTOSOLIC\_RIBOSOME      
 Blue-Pink O' Gram in the Space of the Analyzed GeneSet

  

Fig 3: GOCC\_CYTOSOLIC\_RIBOSOME: Random ES distribution      
 Gene set null distribution of ES for **GOCC\_CYTOSOLIC\_RIBOSOME**

  
