# Supplementary material for: Time-series transcriptome analysis identified differentially expressed genes in broiler chicken infected with mixed Eimeria species
Source: Front Genet. 2022 Aug 8;13:886781. doi: 10.3389/fgene.2022.886781 (PMC9393255; doi:10.3389/fgene.2022.886781)
Supplement: Supplementary file 2 [file DataSheet1.ZIP › 4dpi_GO.Gsea.1625071243202/GOCC_CYTOSOLIC_SMALL_RIBOSOMAL_SUBUNIT.html]

Details for gene set GOCC\_CYTOSOLIC\_SMALL\_RIBOSOMAL\_SUBUNIT[GSEA]

|  || Dataset | TMM\_4dpi\_gct\_format\_4dpi\_gct\_format.Class\_4dpi.cls #PC\_versus\_NC.Class\_4dpi.cls #PC\_versus\_NC\_repos |
| Phenotype | Class\_4dpi.cls#PC\_versus\_NC\_repos |
| Upregulated in class | 0 |
| GeneSet | GOCC\_CYTOSOLIC\_SMALL\_RIBOSOMAL\_SUBUNIT |
| Enrichment Score (ES) | -0.7117235 |
| Normalized Enrichment Score (NES) | -2.4583545 |
| Nominal p-value | 0.0 |
| FDR q-value | 0.0 |
| FWER p-Value | 0.0 |
Table: GSEA Results Summary

  

Fig 1: Enrichment plot: GOCC\_CYTOSOLIC\_SMALL\_RIBOSOMAL\_SUBUNIT      
 Profile of the Running ES Score & Positions of GeneSet Members on the Rank Ordered List

  

| SYMBOL | TITLE | RANK IN GENE LIST | RANK METRIC SCORE | RUNNING ES | CORE ENRICHMENT || 1 | HBA1 | na | 1865 | 0.445 | -0.1345 | No |
| 2 | DHX29 | na | 2779 | 0.311 | -0.1959 | No |
| 3 | RPS27L | na | 5614 | 0.024 | -0.4314 | No |
| 4 | EIF2A | na | 6388 | -0.037 | -0.4941 | No |
| 5 | DDX3X | na | 6973 | -0.087 | -0.5388 | No |
| 6 | MRPS11 | na | 7235 | -0.112 | -0.5552 | No |
| 7 | RPS23 | na | 8269 | -0.206 | -0.6316 | No |
| 8 | MCTS1 | na | 8783 | -0.260 | -0.6621 | No |
| 9 | RPS6 | na | 8904 | -0.274 | -0.6590 | No |
| 10 | EIF2D | na | 9431 | -0.340 | -0.6867 | No |
| 11 | UBA52 | na | 9532 | -0.353 | -0.6782 | No |
| 12 | RPS24 | na | 9689 | -0.372 | -0.6734 | No |
| 13 | LARP4 | na | 10149 | -0.437 | -0.6908 | Yes |
| 14 | RPS8 | na | 10245 | -0.452 | -0.6771 | Yes |
| 15 | RPS28 | na | 10524 | -0.504 | -0.6763 | Yes |
| 16 | RPS19 | na | 10840 | -0.569 | -0.6754 | Yes |
| 17 | RPS12 | na | 10875 | -0.576 | -0.6506 | Yes |
| 18 | RPS25 | na | 11017 | -0.613 | -0.6331 | Yes |
| 19 | RPS7 | na | 11068 | -0.627 | -0.6073 | Yes |
| 20 | RPS16 | na | 11138 | -0.645 | -0.5822 | Yes |
| 21 | RPS15A | na | 11196 | -0.662 | -0.5553 | Yes |
| 22 | RPS26 | na | 11254 | -0.686 | -0.5273 | Yes |
| 23 | RPS21 | na | 11313 | -0.709 | -0.4982 | Yes |
| 24 | RPS3A | na | 11328 | -0.713 | -0.4653 | Yes |
| 25 | RPS10 | na | 11344 | -0.720 | -0.4321 | Yes |
| 26 | RPS11 | na | 11371 | -0.735 | -0.3991 | Yes |
| 27 | RPS15 | na | 11434 | -0.767 | -0.3676 | Yes |
| 28 | RPS27A | na | 11435 | -0.767 | -0.3309 | Yes |
| 29 | RPS29 | na | 11444 | -0.771 | -0.2948 | Yes |
| 30 | RPS14 | na | 11478 | -0.792 | -0.2597 | Yes |
| 31 | RPS2 | na | 11562 | -0.846 | -0.2261 | Yes |
| 32 | RPS20 | na | 11594 | -0.868 | -0.1872 | Yes |
| 33 | RPS27 | na | 11600 | -0.875 | -0.1458 | Yes |
| 34 | RPS13 | na | 11633 | -0.897 | -0.1056 | Yes |
| 35 | RPS3 | na | 11641 | -0.903 | -0.0630 | Yes |
| 36 | RPS17 | na | 11652 | -0.916 | -0.0200 | Yes |
| 37 | RPS4Y1 | na | 11774 | -1.043 | 0.0197 | Yes |
Table: GSEA details [plain text format]

  

Fig 2: GOCC\_CYTOSOLIC\_SMALL\_RIBOSOMAL\_SUBUNIT      
 Blue-Pink O' Gram in the Space of the Analyzed GeneSet

  

Fig 3: GOCC\_CYTOSOLIC\_SMALL\_RIBOSOMAL\_SUBUNIT: Random ES distribution      
 Gene set null distribution of ES for **GOCC\_CYTOSOLIC\_SMALL\_RIBOSOMAL\_SUBUNIT**

  
