# Supplementary material for: Time-series transcriptome analysis identified differentially expressed genes in broiler chicken infected with mixed Eimeria species
Source: Front Genet. 2022 Aug 8;13:886781. doi: 10.3389/fgene.2022.886781 (PMC9393255; doi:10.3389/fgene.2022.886781)
Supplement: Supplementary file 2 [file DataSheet1.ZIP › 4dpi_GO.Gsea.1625071243202/GOCC_LARGE_RIBOSOMAL_SUBUNIT.html]

Details for gene set GOCC\_LARGE\_RIBOSOMAL\_SUBUNIT[GSEA]

|  || Dataset | TMM\_4dpi\_gct\_format\_4dpi\_gct\_format.Class\_4dpi.cls #PC\_versus\_NC.Class\_4dpi.cls #PC\_versus\_NC\_repos |
| Phenotype | Class\_4dpi.cls#PC\_versus\_NC\_repos |
| Upregulated in class | 0 |
| GeneSet | GOCC\_LARGE\_RIBOSOMAL\_SUBUNIT |
| Enrichment Score (ES) | -0.59072876 |
| Normalized Enrichment Score (NES) | -2.4466383 |
| Nominal p-value | 0.0 |
| FDR q-value | 0.0 |
| FWER p-Value | 0.0 |
Table: GSEA Results Summary

  

Fig 1: Enrichment plot: GOCC\_LARGE\_RIBOSOMAL\_SUBUNIT      
 Profile of the Running ES Score & Positions of GeneSet Members on the Rank Ordered List

  

| SYMBOL | TITLE | RANK IN GENE LIST | RANK METRIC SCORE | RUNNING ES | CORE ENRICHMENT || 1 | MRPL30 | na | 1846 | 0.447 | -0.1434 | No |
| 2 | MRPL27 | na | 2096 | 0.406 | -0.1539 | No |
| 3 | NSUN3 | na | 2518 | 0.345 | -0.1804 | No |
| 4 | MRPL54 | na | 2852 | 0.302 | -0.2006 | No |
| 5 | MRPL18 | na | 3417 | 0.231 | -0.2420 | No |
| 6 | MRPL58 | na | 3930 | 0.174 | -0.2805 | No |
| 7 | RBM3 | na | 4037 | 0.164 | -0.2851 | No |
| 8 | MRPL33 | na | 4174 | 0.153 | -0.2926 | No |
| 9 | MRPL24 | na | 4317 | 0.140 | -0.3009 | No |
| 10 | MRPL42 | na | 4338 | 0.139 | -0.2991 | No |
| 11 | MRPL19 | na | 4506 | 0.123 | -0.3099 | No |
| 12 | MRPL48 | na | 4543 | 0.121 | -0.3098 | No |
| 13 | MRPL12 | na | 4639 | 0.111 | -0.3149 | No |
| 14 | MRPS18A | na | 4841 | 0.094 | -0.3294 | No |
| 15 | MRPL28 | na | 5550 | 0.031 | -0.3880 | No |
| 16 | MRPL53 | na | 5600 | 0.026 | -0.3915 | No |
| 17 | MRPL39 | na | 5658 | 0.020 | -0.3957 | No |
| 18 | MPV17L2 | na | 5725 | 0.015 | -0.4009 | No |
| 19 | MRPL21 | na | 6237 | -0.026 | -0.4431 | No |
| 20 | RSL24D1 | na | 6280 | -0.029 | -0.4459 | No |
| 21 | MRPL35 | na | 6348 | -0.034 | -0.4507 | No |
| 22 | MRPL50 | na | 6380 | -0.037 | -0.4523 | No |
| 23 | MRPL3 | na | 6450 | -0.043 | -0.4570 | No |
| 24 | MALSU1 | na | 6548 | -0.050 | -0.4639 | No |
| 25 | MRPL47 | na | 6623 | -0.056 | -0.4686 | No |
| 26 | MRPL40 | na | 7136 | -0.102 | -0.5090 | No |
| 27 | MRPL41 | na | 7359 | -0.123 | -0.5244 | No |
| 28 | MRPL14 | na | 7372 | -0.124 | -0.5223 | No |
| 29 | MRPL15 | na | 7420 | -0.129 | -0.5229 | No |
| 30 | MRPL1 | na | 7631 | -0.147 | -0.5368 | No |
| 31 | MRPL51 | na | 7664 | -0.149 | -0.5356 | No |
| 32 | MRPL32 | na | 7673 | -0.150 | -0.5325 | No |
| 33 | MRPS30 | na | 7688 | -0.152 | -0.5297 | No |
| 34 | MRPL10 | na | 8032 | -0.183 | -0.5538 | No |
| 35 | MRPL44 | na | 8072 | -0.186 | -0.5523 | No |
| 36 | MRPL20 | na | 8418 | -0.220 | -0.5756 | No |
| 37 | RPL7L1 | na | 8437 | -0.222 | -0.5715 | No |
| 38 | MRPL43 | na | 8470 | -0.226 | -0.5683 | No |
| 39 | MRPL34 | na | 8555 | -0.236 | -0.5693 | No |
| 40 | RPL17 | na | 8583 | -0.239 | -0.5655 | No |
| 41 | MRPL23 | na | 8616 | -0.242 | -0.5620 | No |
| 42 | MRPL46 | na | 8643 | -0.244 | -0.5579 | No |
| 43 | MRPL17 | na | 8744 | -0.255 | -0.5597 | No |
| 44 | MRPL9 | na | 9025 | -0.286 | -0.5759 | No |
| 45 | MRPL22 | na | 9179 | -0.306 | -0.5809 | Yes |
| 46 | NDUFAB1 | na | 9185 | -0.306 | -0.5734 | Yes |
| 47 | MRPL16 | na | 9210 | -0.310 | -0.5675 | Yes |
| 48 | RPL36 | na | 9363 | -0.330 | -0.5718 | Yes |
| 49 | NSUN4 | na | 9550 | -0.356 | -0.5782 | Yes |
| 50 | RPL38 | na | 9661 | -0.369 | -0.5780 | Yes |
| 51 | MRPL2 | na | 9691 | -0.372 | -0.5709 | Yes |
| 52 | MRPL45 | na | 9808 | -0.387 | -0.5707 | Yes |
| 53 | RPLP2 | na | 10048 | -0.421 | -0.5799 | Yes |
| 54 | RPL37 | na | 10057 | -0.423 | -0.5697 | Yes |
| 55 | MRPL57 | na | 10202 | -0.445 | -0.5704 | Yes |
| 56 | RPL27 | na | 10333 | -0.467 | -0.5693 | Yes |
| 57 | MRPL37 | na | 10453 | -0.489 | -0.5668 | Yes |
| 58 | RPL30 | na | 10537 | -0.506 | -0.5607 | Yes |
| 59 | RPL22 | na | 10554 | -0.508 | -0.5490 | Yes |
| 60 | RPL36A | na | 10605 | -0.520 | -0.5399 | Yes |
| 61 | RPL39L | na | 10613 | -0.521 | -0.5271 | Yes |
| 62 | RPL14 | na | 10662 | -0.531 | -0.5175 | Yes |
| 63 | RPL29 | na | 10742 | -0.548 | -0.5101 | Yes |
| 64 | RPL37A | na | 10838 | -0.569 | -0.5034 | Yes |
| 65 | RPL24 | na | 10877 | -0.576 | -0.4918 | Yes |
| 66 | MRPL55 | na | 10925 | -0.587 | -0.4807 | Yes |
| 67 | RPL34 | na | 10933 | -0.588 | -0.4662 | Yes |
| 68 | RPL23 | na | 10981 | -0.602 | -0.4547 | Yes |
| 69 | RPL35A | na | 10982 | -0.602 | -0.4392 | Yes |
| 70 | RPL23A | na | 11076 | -0.629 | -0.4309 | Yes |
| 71 | RPL5 | na | 11121 | -0.643 | -0.4181 | Yes |
| 72 | RPL6 | na | 11150 | -0.650 | -0.4038 | Yes |
| 73 | RPL11 | na | 11195 | -0.662 | -0.3905 | Yes |
| 74 | RPLP1 | na | 11249 | -0.682 | -0.3774 | Yes |
| 75 | RPL35 | na | 11280 | -0.696 | -0.3620 | Yes |
| 76 | RPL26L1 | na | 11283 | -0.698 | -0.3443 | Yes |
| 77 | RPL31 | na | 11306 | -0.706 | -0.3280 | Yes |
| 78 | RPL21 | na | 11330 | -0.714 | -0.3116 | Yes |
| 79 | RPL32 | na | 11355 | -0.725 | -0.2950 | Yes |
| 80 | MRPL13 | na | 11364 | -0.731 | -0.2769 | Yes |
| 81 | MRPL38 | na | 11387 | -0.742 | -0.2597 | Yes |
| 82 | RPL12 | na | 11398 | -0.748 | -0.2414 | Yes |
| 83 | RPL7A | na | 11426 | -0.763 | -0.2240 | Yes |
| 84 | RPL15 | na | 11440 | -0.769 | -0.2054 | Yes |
| 85 | RPL18A | na | 11474 | -0.789 | -0.1879 | Yes |
| 86 | RPL7 | na | 11484 | -0.796 | -0.1682 | Yes |
| 87 | RPLP0 | na | 11488 | -0.800 | -0.1479 | Yes |
| 88 | RPL27A | na | 11507 | -0.812 | -0.1286 | Yes |
| 89 | RPL9 | na | 11521 | -0.819 | -0.1087 | Yes |
| 90 | RPL13 | na | 11563 | -0.847 | -0.0904 | Yes |
| 91 | RPL19 | na | 11611 | -0.882 | -0.0717 | Yes |
| 92 | RPL10A | na | 11658 | -0.921 | -0.0519 | Yes |
| 93 | RPL4 | na | 11715 | -0.983 | -0.0314 | Yes |
| 94 | RPL8 | na | 11772 | -1.039 | -0.0094 | Yes |
| 95 | RPL3 | na | 11817 | -1.140 | 0.0162 | Yes |
Table: GSEA details [plain text format]

  

Fig 2: GOCC\_LARGE\_RIBOSOMAL\_SUBUNIT      
 Blue-Pink O' Gram in the Space of the Analyzed GeneSet

  

Fig 3: GOCC\_LARGE\_RIBOSOMAL\_SUBUNIT: Random ES distribution      
 Gene set null distribution of ES for **GOCC\_LARGE\_RIBOSOMAL\_SUBUNIT**

  
