# Supplementary material for: Time-series transcriptome analysis identified differentially expressed genes in broiler chicken infected with mixed Eimeria species
Source: Front Genet. 2022 Aug 8;13:886781. doi: 10.3389/fgene.2022.886781 (PMC9393255; doi:10.3389/fgene.2022.886781)
Supplement: Supplementary file 2 [file DataSheet1.ZIP › 4dpi_GO.Gsea.1625071243202/GOCC_POLYSOMAL_RIBOSOME.html]

Details for gene set GOCC\_POLYSOMAL\_RIBOSOME[GSEA]

|  || Dataset | TMM\_4dpi\_gct\_format\_4dpi\_gct\_format.Class\_4dpi.cls #PC\_versus\_NC.Class\_4dpi.cls #PC\_versus\_NC\_repos |
| Phenotype | Class\_4dpi.cls#PC\_versus\_NC\_repos |
| Upregulated in class | 0 |
| GeneSet | GOCC\_POLYSOMAL\_RIBOSOME |
| Enrichment Score (ES) | -0.6957709 |
| Normalized Enrichment Score (NES) | -2.2508335 |
| Nominal p-value | 0.0 |
| FDR q-value | 2.0505262E-4 |
| FWER p-Value | 0.0042 |
Table: GSEA Results Summary

  

Fig 1: Enrichment plot: GOCC\_POLYSOMAL\_RIBOSOME      
 Profile of the Running ES Score & Positions of GeneSet Members on the Rank Ordered List

  

| SYMBOL | TITLE | RANK IN GENE LIST | RANK METRIC SCORE | RUNNING ES | CORE ENRICHMENT || 1 | FMR1 | na | 647 | 0.851 | -0.0012 | No |
| 2 | LARP4B | na | 3339 | 0.238 | -0.2110 | No |
| 3 | NUFIP2 | na | 4340 | 0.139 | -0.2858 | No |
| 4 | PNPT1 | na | 5062 | 0.073 | -0.3414 | No |
| 5 | LARP1 | na | 7036 | -0.093 | -0.5004 | No |
| 6 | RPS23 | na | 8269 | -0.206 | -0.5904 | No |
| 7 | NR0B1 | na | 8635 | -0.243 | -0.6058 | No |
| 8 | RPL36 | na | 9363 | -0.330 | -0.6460 | No |
| 9 | RPL38 | na | 9661 | -0.369 | -0.6478 | No |
| 10 | BTF3 | na | 9796 | -0.385 | -0.6351 | No |
| 11 | RPS28 | na | 10524 | -0.504 | -0.6645 | Yes |
| 12 | RPL30 | na | 10537 | -0.506 | -0.6341 | Yes |
| 13 | RPL36A | na | 10605 | -0.520 | -0.6075 | Yes |
| 14 | RPL24 | na | 10877 | -0.576 | -0.5943 | Yes |
| 15 | RPL6 | na | 11150 | -0.650 | -0.5767 | Yes |
| 16 | RPL11 | na | 11195 | -0.662 | -0.5392 | Yes |
| 17 | RPS26 | na | 11254 | -0.686 | -0.5015 | Yes |
| 18 | RPL31 | na | 11306 | -0.706 | -0.4620 | Yes |
| 19 | RPS21 | na | 11313 | -0.709 | -0.4184 | Yes |
| 20 | RPL32 | na | 11355 | -0.725 | -0.3769 | Yes |
| 21 | RPL7A | na | 11426 | -0.763 | -0.3354 | Yes |
| 22 | RPS29 | na | 11444 | -0.771 | -0.2890 | Yes |
| 23 | RPL18A | na | 11474 | -0.789 | -0.2424 | Yes |
| 24 | RPL19 | na | 11611 | -0.882 | -0.1990 | Yes |
| 25 | EEF2 | na | 11615 | -0.885 | -0.1444 | Yes |
| 26 | EIF3H | na | 11627 | -0.893 | -0.0898 | Yes |
| 27 | RPL10A | na | 11658 | -0.921 | -0.0352 | Yes |
| 28 | RPL8 | na | 11772 | -1.039 | 0.0199 | Yes |
Table: GSEA details [plain text format]

  

Fig 2: GOCC\_POLYSOMAL\_RIBOSOME      
 Blue-Pink O' Gram in the Space of the Analyzed GeneSet

  

Fig 3: GOCC\_POLYSOMAL\_RIBOSOME: Random ES distribution      
 Gene set null distribution of ES for **GOCC\_POLYSOMAL\_RIBOSOME**

  
