# Supplementary material for: Time-series transcriptome analysis identified differentially expressed genes in broiler chicken infected with mixed Eimeria species
Source: Front Genet. 2022 Aug 8;13:886781. doi: 10.3389/fgene.2022.886781 (PMC9393255; doi:10.3389/fgene.2022.886781)
Supplement: Supplementary file 2 [file DataSheet1.ZIP › 4dpi_GO.Gsea.1625071243202/GOCC_RIBOSOMAL_SUBUNIT.html]

Details for gene set GOCC\_RIBOSOMAL\_SUBUNIT[GSEA]

|  || Dataset | TMM\_4dpi\_gct\_format\_4dpi\_gct\_format.Class\_4dpi.cls #PC\_versus\_NC.Class\_4dpi.cls #PC\_versus\_NC\_repos |
| Phenotype | Class\_4dpi.cls#PC\_versus\_NC\_repos |
| Upregulated in class | 0 |
| GeneSet | GOCC\_RIBOSOMAL\_SUBUNIT |
| Enrichment Score (ES) | -0.61665124 |
| Normalized Enrichment Score (NES) | -2.7711208 |
| Nominal p-value | 0.0 |
| FDR q-value | 0.0 |
| FWER p-Value | 0.0 |
Table: GSEA Results Summary

  

Fig 1: Enrichment plot: GOCC\_RIBOSOMAL\_SUBUNIT      
 Profile of the Running ES Score & Positions of GeneSet Members on the Rank Ordered List

  

| SYMBOL | TITLE | RANK IN GENE LIST | RANK METRIC SCORE | RUNNING ES | CORE ENRICHMENT || 1 | MRPL30 | na | 1846 | 0.447 | -0.1489 | No |
| 2 | HBA1 | na | 1865 | 0.445 | -0.1437 | No |
| 3 | MRPL27 | na | 2096 | 0.406 | -0.1570 | No |
| 4 | NSUN3 | na | 2518 | 0.345 | -0.1873 | No |
| 5 | DHX29 | na | 2779 | 0.311 | -0.2045 | No |
| 6 | MRPL54 | na | 2852 | 0.302 | -0.2060 | No |
| 7 | MRPL18 | na | 3417 | 0.231 | -0.2501 | No |
| 8 | MRPS34 | na | 3713 | 0.198 | -0.2720 | No |
| 9 | MRPL58 | na | 3930 | 0.174 | -0.2876 | No |
| 10 | RBM3 | na | 4037 | 0.164 | -0.2940 | No |
| 11 | MRPL33 | na | 4174 | 0.153 | -0.3032 | No |
| 12 | MRPL24 | na | 4317 | 0.140 | -0.3130 | No |
| 13 | MRPL42 | na | 4338 | 0.139 | -0.3126 | No |
| 14 | MRPL19 | na | 4506 | 0.123 | -0.3248 | No |
| 15 | MRPL48 | na | 4543 | 0.121 | -0.3260 | No |
| 16 | MRPL12 | na | 4639 | 0.111 | -0.3324 | No |
| 17 | MRPS17 | na | 4827 | 0.095 | -0.3467 | No |
| 18 | MRPS18A | na | 4841 | 0.094 | -0.3464 | No |
| 19 | MRPS33 | na | 5356 | 0.049 | -0.3890 | No |
| 20 | MRPS14 | na | 5490 | 0.037 | -0.3996 | No |
| 21 | MRPL28 | na | 5550 | 0.031 | -0.4042 | No |
| 22 | MRPL53 | na | 5600 | 0.026 | -0.4079 | No |
| 23 | RPS27L | na | 5614 | 0.024 | -0.4086 | No |
| 24 | MRPL39 | na | 5658 | 0.020 | -0.4119 | No |
| 25 | MRPS5 | na | 5706 | 0.016 | -0.4157 | No |
| 26 | MPV17L2 | na | 5725 | 0.015 | -0.4169 | No |
| 27 | MRPS9 | na | 5780 | 0.011 | -0.4213 | No |
| 28 | MRPL21 | na | 6237 | -0.026 | -0.4594 | No |
| 29 | RSL24D1 | na | 6280 | -0.029 | -0.4625 | No |
| 30 | MRPL35 | na | 6348 | -0.034 | -0.4677 | No |
| 31 | MRPL50 | na | 6380 | -0.037 | -0.4697 | No |
| 32 | EIF2A | na | 6388 | -0.037 | -0.4698 | No |
| 33 | MRPL3 | na | 6450 | -0.043 | -0.4743 | No |
| 34 | MRPS18C | na | 6501 | -0.046 | -0.4778 | No |
| 35 | MALSU1 | na | 6548 | -0.050 | -0.4809 | No |
| 36 | MRPL47 | na | 6623 | -0.056 | -0.4863 | No |
| 37 | MRPS35 | na | 6894 | -0.078 | -0.5079 | No |
| 38 | DAP3 | na | 6904 | -0.080 | -0.5074 | No |
| 39 | DDX3X | na | 6973 | -0.087 | -0.5118 | No |
| 40 | MRPS26 | na | 7030 | -0.092 | -0.5152 | No |
| 41 | MRPL40 | na | 7136 | -0.102 | -0.5225 | No |
| 42 | MRPS11 | na | 7235 | -0.112 | -0.5291 | No |
| 43 | MRPL41 | na | 7359 | -0.123 | -0.5376 | No |
| 44 | MRPL14 | na | 7372 | -0.124 | -0.5367 | No |
| 45 | MRPL15 | na | 7420 | -0.129 | -0.5387 | No |
| 46 | MRPS27 | na | 7421 | -0.129 | -0.5368 | No |
| 47 | MRPS16 | na | 7477 | -0.134 | -0.5394 | No |
| 48 | MRPL1 | na | 7631 | -0.147 | -0.5501 | No |
| 49 | MRPL51 | na | 7664 | -0.149 | -0.5505 | No |
| 50 | MRPL32 | na | 7673 | -0.150 | -0.5489 | No |
| 51 | MRPS30 | na | 7688 | -0.152 | -0.5478 | No |
| 52 | MRPL10 | na | 8032 | -0.183 | -0.5740 | No |
| 53 | MRPL44 | na | 8072 | -0.186 | -0.5745 | No |
| 54 | RPS23 | na | 8269 | -0.206 | -0.5879 | No |
| 55 | MRPL20 | na | 8418 | -0.220 | -0.5970 | No |
| 56 | MRPS22 | na | 8420 | -0.220 | -0.5938 | No |
| 57 | MRPS31 | na | 8432 | -0.222 | -0.5914 | No |
| 58 | RPL7L1 | na | 8437 | -0.222 | -0.5884 | No |
| 59 | MRPL43 | na | 8470 | -0.226 | -0.5876 | No |
| 60 | MRPS36 | na | 8541 | -0.233 | -0.5900 | No |
| 61 | MRPL34 | na | 8555 | -0.236 | -0.5875 | No |
| 62 | RPL17 | na | 8583 | -0.239 | -0.5862 | No |
| 63 | MRPL23 | na | 8616 | -0.242 | -0.5853 | No |
| 64 | MRPL46 | na | 8643 | -0.244 | -0.5838 | No |
| 65 | MRPL17 | na | 8744 | -0.255 | -0.5883 | No |
| 66 | MCTS1 | na | 8783 | -0.260 | -0.5876 | No |
| 67 | RPS6 | na | 8904 | -0.274 | -0.5936 | No |
| 68 | MRPS7 | na | 8923 | -0.275 | -0.5909 | No |
| 69 | MRPL9 | na | 9025 | -0.286 | -0.5951 | No |
| 70 | MRPL22 | na | 9179 | -0.306 | -0.6034 | No |
| 71 | NDUFAB1 | na | 9185 | -0.306 | -0.5992 | No |
| 72 | MRPL16 | na | 9210 | -0.310 | -0.5966 | No |
| 73 | RPL36 | na | 9363 | -0.330 | -0.6044 | No |
| 74 | EIF2D | na | 9431 | -0.340 | -0.6049 | No |
| 75 | UBA52 | na | 9532 | -0.353 | -0.6080 | No |
| 76 | NSUN4 | na | 9550 | -0.356 | -0.6040 | No |
| 77 | RPL38 | na | 9661 | -0.369 | -0.6077 | No |
| 78 | RPS24 | na | 9689 | -0.372 | -0.6044 | No |
| 79 | MRPL2 | na | 9691 | -0.372 | -0.5988 | No |
| 80 | MRPL45 | na | 9808 | -0.387 | -0.6028 | No |
| 81 | MRPS28 | na | 9971 | -0.409 | -0.6102 | No |
| 82 | RPLP2 | na | 10048 | -0.421 | -0.6103 | Yes |
| 83 | MRPS12 | na | 10055 | -0.422 | -0.6044 | Yes |
| 84 | RPL37 | na | 10057 | -0.423 | -0.5981 | Yes |
| 85 | MRPS21 | na | 10061 | -0.423 | -0.5919 | Yes |
| 86 | MRPS10 | na | 10111 | -0.431 | -0.5896 | Yes |
| 87 | LARP4 | na | 10149 | -0.437 | -0.5861 | Yes |
| 88 | MRPL57 | na | 10202 | -0.445 | -0.5837 | Yes |
| 89 | RPS8 | na | 10245 | -0.452 | -0.5804 | Yes |
| 90 | RPL27 | na | 10333 | -0.467 | -0.5807 | Yes |
| 91 | MRPL37 | na | 10453 | -0.489 | -0.5833 | Yes |
| 92 | RPS28 | na | 10524 | -0.504 | -0.5816 | Yes |
| 93 | RPL30 | na | 10537 | -0.506 | -0.5750 | Yes |
| 94 | RPL22 | na | 10554 | -0.508 | -0.5687 | Yes |
| 95 | RPL36A | na | 10605 | -0.520 | -0.5650 | Yes |
| 96 | RPL39L | na | 10613 | -0.521 | -0.5577 | Yes |
| 97 | RPL14 | na | 10662 | -0.531 | -0.5537 | Yes |
| 98 | RPL29 | na | 10742 | -0.548 | -0.5521 | Yes |
| 99 | RPL37A | na | 10838 | -0.569 | -0.5515 | Yes |
| 100 | RPS19 | na | 10840 | -0.569 | -0.5430 | Yes |
| 101 | RPS12 | na | 10875 | -0.576 | -0.5371 | Yes |
| 102 | RPL24 | na | 10877 | -0.576 | -0.5285 | Yes |
| 103 | MRPL55 | na | 10925 | -0.587 | -0.5236 | Yes |
| 104 | RPL34 | na | 10933 | -0.588 | -0.5153 | Yes |
| 105 | RPL23 | na | 10981 | -0.602 | -0.5102 | Yes |
| 106 | RPL35A | na | 10982 | -0.602 | -0.5010 | Yes |
| 107 | RPS25 | na | 11017 | -0.613 | -0.4946 | Yes |
| 108 | RPS7 | na | 11068 | -0.627 | -0.4894 | Yes |
| 109 | RPL23A | na | 11076 | -0.629 | -0.4805 | Yes |
| 110 | RPL5 | na | 11121 | -0.643 | -0.4745 | Yes |
| 111 | RPS16 | na | 11138 | -0.645 | -0.4660 | Yes |
| 112 | RPL6 | na | 11150 | -0.650 | -0.4571 | Yes |
| 113 | RPL11 | na | 11195 | -0.662 | -0.4508 | Yes |
| 114 | RPS15A | na | 11196 | -0.662 | -0.4408 | Yes |
| 115 | RPLP1 | na | 11249 | -0.682 | -0.4349 | Yes |
| 116 | RPS26 | na | 11254 | -0.686 | -0.4248 | Yes |
| 117 | RPL35 | na | 11280 | -0.696 | -0.4164 | Yes |
| 118 | RPL26L1 | na | 11283 | -0.698 | -0.4060 | Yes |
| 119 | RPL31 | na | 11306 | -0.706 | -0.3972 | Yes |
| 120 | RPS21 | na | 11313 | -0.709 | -0.3870 | Yes |
| 121 | RPS3A | na | 11328 | -0.713 | -0.3774 | Yes |
| 122 | RPL21 | na | 11330 | -0.714 | -0.3667 | Yes |
| 123 | RPS10 | na | 11344 | -0.720 | -0.3569 | Yes |
| 124 | RPL32 | na | 11355 | -0.725 | -0.3468 | Yes |
| 125 | MRPL13 | na | 11364 | -0.731 | -0.3364 | Yes |
| 126 | RPS11 | na | 11371 | -0.735 | -0.3258 | Yes |
| 127 | MRPL38 | na | 11387 | -0.742 | -0.3158 | Yes |
| 128 | RPL12 | na | 11398 | -0.748 | -0.3053 | Yes |
| 129 | RPL7A | na | 11426 | -0.763 | -0.2961 | Yes |
| 130 | RPS15 | na | 11434 | -0.767 | -0.2851 | Yes |
| 131 | RPS27A | na | 11435 | -0.767 | -0.2735 | Yes |
| 132 | RPL15 | na | 11440 | -0.769 | -0.2622 | Yes |
| 133 | RPS29 | na | 11444 | -0.771 | -0.2508 | Yes |
| 134 | MRPS6 | na | 11465 | -0.783 | -0.2406 | Yes |
| 135 | RPL18A | na | 11474 | -0.789 | -0.2294 | Yes |
| 136 | RPS14 | na | 11478 | -0.792 | -0.2176 | Yes |
| 137 | RPL7 | na | 11484 | -0.796 | -0.2060 | Yes |
| 138 | RPLP0 | na | 11488 | -0.800 | -0.1942 | Yes |
| 139 | RPL27A | na | 11507 | -0.812 | -0.1834 | Yes |
| 140 | RPL9 | na | 11521 | -0.819 | -0.1721 | Yes |
| 141 | RACK1 | na | 11554 | -0.842 | -0.1621 | Yes |
| 142 | RPS2 | na | 11562 | -0.846 | -0.1499 | Yes |
| 143 | RPL13 | na | 11563 | -0.847 | -0.1371 | Yes |
| 144 | RPS20 | na | 11594 | -0.868 | -0.1265 | Yes |
| 145 | RPS27 | na | 11600 | -0.875 | -0.1136 | Yes |
| 146 | RPL19 | na | 11611 | -0.882 | -0.1011 | Yes |
| 147 | RPS13 | na | 11633 | -0.897 | -0.0894 | Yes |
| 148 | RPS3 | na | 11641 | -0.903 | -0.0763 | Yes |
| 149 | RPS17 | na | 11652 | -0.916 | -0.0633 | Yes |
| 150 | RPL10A | na | 11658 | -0.921 | -0.0498 | Yes |
| 151 | RPL4 | na | 11715 | -0.983 | -0.0396 | Yes |
| 152 | MRPS2 | na | 11754 | -1.021 | -0.0274 | Yes |
| 153 | RPL8 | na | 11772 | -1.039 | -0.0131 | Yes |
| 154 | RPS4Y1 | na | 11774 | -1.043 | 0.0026 | Yes |
| 155 | RPL3 | na | 11817 | -1.140 | 0.0163 | Yes |
Table: GSEA details [plain text format]

  

Fig 2: GOCC\_RIBOSOMAL\_SUBUNIT      
 Blue-Pink O' Gram in the Space of the Analyzed GeneSet

  

Fig 3: GOCC\_RIBOSOMAL\_SUBUNIT: Random ES distribution      
 Gene set null distribution of ES for **GOCC\_RIBOSOMAL\_SUBUNIT**

  
