# Supplementary material for: Time-series transcriptome analysis identified differentially expressed genes in broiler chicken infected with mixed Eimeria species
Source: Front Genet. 2022 Aug 8;13:886781. doi: 10.3389/fgene.2022.886781 (PMC9393255; doi:10.3389/fgene.2022.886781)
Supplement: Supplementary file 2 [file DataSheet1.ZIP › 4dpi_GO.Gsea.1625071243202/GOCC_RIBOSOME.html]

Details for gene set GOCC\_RIBOSOME[GSEA]

|  || Dataset | TMM\_4dpi\_gct\_format\_4dpi\_gct\_format.Class\_4dpi.cls #PC\_versus\_NC.Class\_4dpi.cls #PC\_versus\_NC\_repos |
| Phenotype | Class\_4dpi.cls#PC\_versus\_NC\_repos |
| Upregulated in class | 0 |
| GeneSet | GOCC\_RIBOSOME |
| Enrichment Score (ES) | -0.5585936 |
| Normalized Enrichment Score (NES) | -2.5656047 |
| Nominal p-value | 0.0 |
| FDR q-value | 0.0 |
| FWER p-Value | 0.0 |
Table: GSEA Results Summary

  

Fig 1: Enrichment plot: GOCC\_RIBOSOME      
 Profile of the Running ES Score & Positions of GeneSet Members on the Rank Ordered List

  

| SYMBOL | TITLE | RANK IN GENE LIST | RANK METRIC SCORE | RUNNING ES | CORE ENRICHMENT || 1 | EIF2AK2 | na | 139 | 1.499 | 0.0079 | No |
| 2 | FMR1 | na | 647 | 0.851 | -0.0239 | No |
| 3 | MRPL30 | na | 1846 | 0.447 | -0.1193 | No |
| 4 | HBA1 | na | 1865 | 0.445 | -0.1150 | No |
| 5 | APOD | na | 1919 | 0.437 | -0.1138 | No |
| 6 | MRPL27 | na | 2096 | 0.406 | -0.1233 | No |
| 7 | EIF2AK4 | na | 2101 | 0.406 | -0.1184 | No |
| 8 | RRBP1 | na | 2197 | 0.389 | -0.1213 | No |
| 9 | NSUN3 | na | 2518 | 0.345 | -0.1438 | No |
| 10 | DHX29 | na | 2779 | 0.311 | -0.1618 | No |
| 11 | MRPS25 | na | 2828 | 0.305 | -0.1618 | No |
| 12 | MRPL54 | na | 2852 | 0.302 | -0.1598 | No |
| 13 | LARP4B | na | 3339 | 0.238 | -0.1978 | No |
| 14 | MRPL18 | na | 3417 | 0.231 | -0.2013 | No |
| 15 | MTG2 | na | 3616 | 0.206 | -0.2153 | No |
| 16 | MRPS34 | na | 3713 | 0.198 | -0.2209 | No |
| 17 | MRPL58 | na | 3930 | 0.174 | -0.2368 | No |
| 18 | RBM3 | na | 4037 | 0.164 | -0.2437 | No |
| 19 | MRPL33 | na | 4174 | 0.153 | -0.2532 | No |
| 20 | MRPL24 | na | 4317 | 0.140 | -0.2633 | No |
| 21 | MRPL42 | na | 4338 | 0.139 | -0.2632 | No |
| 22 | NUFIP2 | na | 4340 | 0.139 | -0.2615 | No |
| 23 | CHCHD1 | na | 4475 | 0.126 | -0.2711 | No |
| 24 | MRPL19 | na | 4506 | 0.123 | -0.2721 | No |
| 25 | MRPL48 | na | 4543 | 0.121 | -0.2735 | No |
| 26 | MRPL12 | na | 4639 | 0.111 | -0.2801 | No |
| 27 | MRPS17 | na | 4827 | 0.095 | -0.2947 | No |
| 28 | MRPS18A | na | 4841 | 0.094 | -0.2945 | No |
| 29 | NCK1 | na | 4852 | 0.093 | -0.2942 | No |
| 30 | SRP68 | na | 5003 | 0.078 | -0.3058 | No |
| 31 | PNPT1 | na | 5062 | 0.073 | -0.3098 | No |
| 32 | SERP1 | na | 5121 | 0.068 | -0.3138 | No |
| 33 | MRPS33 | na | 5356 | 0.049 | -0.3329 | No |
| 34 | GCN1 | na | 5390 | 0.046 | -0.3351 | No |
| 35 | MRPS14 | na | 5490 | 0.037 | -0.3430 | No |
| 36 | MRPL28 | na | 5550 | 0.031 | -0.3476 | No |
| 37 | MRPL53 | na | 5600 | 0.026 | -0.3514 | No |
| 38 | RPS27L | na | 5614 | 0.024 | -0.3522 | No |
| 39 | MRPL39 | na | 5658 | 0.020 | -0.3555 | No |
| 40 | MRPS5 | na | 5706 | 0.016 | -0.3593 | No |
| 41 | MPV17L2 | na | 5725 | 0.015 | -0.3606 | No |
| 42 | MRPS9 | na | 5780 | 0.011 | -0.3650 | No |
| 43 | PTCD3 | na | 6126 | -0.017 | -0.3940 | No |
| 44 | MRPL21 | na | 6237 | -0.026 | -0.4030 | No |
| 45 | RSL24D1 | na | 6280 | -0.029 | -0.4061 | No |
| 46 | MRPL35 | na | 6348 | -0.034 | -0.4114 | No |
| 47 | MRPL50 | na | 6380 | -0.037 | -0.4135 | No |
| 48 | EIF2A | na | 6388 | -0.037 | -0.4136 | No |
| 49 | MRPL3 | na | 6450 | -0.043 | -0.4182 | No |
| 50 | MRPS18C | na | 6501 | -0.046 | -0.4218 | No |
| 51 | MALSU1 | na | 6548 | -0.050 | -0.4251 | No |
| 52 | MRPL47 | na | 6623 | -0.056 | -0.4306 | No |
| 53 | MRPS35 | na | 6894 | -0.078 | -0.4524 | No |
| 54 | DAP3 | na | 6904 | -0.080 | -0.4521 | No |
| 55 | DDX3X | na | 6973 | -0.087 | -0.4567 | No |
| 56 | MRPS26 | na | 7030 | -0.092 | -0.4603 | No |
| 57 | LARP1 | na | 7036 | -0.093 | -0.4595 | No |
| 58 | MRPL40 | na | 7136 | -0.102 | -0.4665 | No |
| 59 | MRPS11 | na | 7235 | -0.112 | -0.4733 | No |
| 60 | MRPL41 | na | 7359 | -0.123 | -0.4821 | No |
| 61 | MRPL14 | na | 7372 | -0.124 | -0.4815 | No |
| 62 | MRPL15 | na | 7420 | -0.129 | -0.4838 | No |
| 63 | MRPS27 | na | 7421 | -0.129 | -0.4821 | No |
| 64 | MRPS16 | na | 7477 | -0.134 | -0.4850 | No |
| 65 | NDUFA7 | na | 7501 | -0.135 | -0.4852 | No |
| 66 | HSPA14 | na | 7595 | -0.143 | -0.4912 | No |
| 67 | MRPL1 | na | 7631 | -0.147 | -0.4922 | No |
| 68 | MRPL51 | na | 7664 | -0.149 | -0.4930 | No |
| 69 | MRPL32 | na | 7673 | -0.150 | -0.4917 | No |
| 70 | AURKAIP1 | na | 7686 | -0.151 | -0.4907 | No |
| 71 | MRPS30 | na | 7688 | -0.152 | -0.4888 | No |
| 72 | MRPL10 | na | 8032 | -0.183 | -0.5154 | No |
| 73 | RPL22L1 | na | 8070 | -0.186 | -0.5161 | No |
| 74 | MRPL44 | na | 8072 | -0.186 | -0.5138 | No |
| 75 | RPS23 | na | 8269 | -0.206 | -0.5277 | No |
| 76 | MRPL20 | na | 8418 | -0.220 | -0.5373 | No |
| 77 | MRPS22 | na | 8420 | -0.220 | -0.5345 | No |
| 78 | MRPS31 | na | 8432 | -0.222 | -0.5325 | No |
| 79 | RPL7L1 | na | 8437 | -0.222 | -0.5299 | No |
| 80 | MRPL43 | na | 8470 | -0.226 | -0.5297 | No |
| 81 | MRPS36 | na | 8541 | -0.233 | -0.5326 | No |
| 82 | MRPL34 | na | 8555 | -0.236 | -0.5306 | No |
| 83 | RPL17 | na | 8583 | -0.239 | -0.5297 | No |
| 84 | MRPL23 | na | 8616 | -0.242 | -0.5293 | No |
| 85 | NR0B1 | na | 8635 | -0.243 | -0.5276 | No |
| 86 | MRPL46 | na | 8643 | -0.244 | -0.5250 | No |
| 87 | MRPL17 | na | 8744 | -0.255 | -0.5301 | No |
| 88 | MCTS1 | na | 8783 | -0.260 | -0.5299 | No |
| 89 | MRPS23 | na | 8869 | -0.270 | -0.5336 | No |
| 90 | RPS6 | na | 8904 | -0.274 | -0.5329 | No |
| 91 | MRPS7 | na | 8923 | -0.275 | -0.5308 | No |
| 92 | MRPL9 | na | 9025 | -0.286 | -0.5356 | No |
| 93 | MRPL22 | na | 9179 | -0.306 | -0.5445 | No |
| 94 | NDUFAB1 | na | 9185 | -0.306 | -0.5410 | No |
| 95 | MRPL16 | na | 9210 | -0.310 | -0.5389 | No |
| 96 | RPL36 | na | 9363 | -0.330 | -0.5475 | No |
| 97 | EIF2D | na | 9431 | -0.340 | -0.5487 | No |
| 98 | UBA52 | na | 9532 | -0.353 | -0.5525 | No |
| 99 | NSUN4 | na | 9550 | -0.356 | -0.5493 | No |
| 100 | RPL38 | na | 9661 | -0.369 | -0.5538 | Yes |
| 101 | RPS24 | na | 9689 | -0.372 | -0.5512 | Yes |
| 102 | MRPL2 | na | 9691 | -0.372 | -0.5464 | Yes |
| 103 | NUFIP1 | na | 9750 | -0.379 | -0.5463 | Yes |
| 104 | BTF3 | na | 9796 | -0.385 | -0.5451 | Yes |
| 105 | MRPL45 | na | 9808 | -0.387 | -0.5409 | Yes |
| 106 | MRPS28 | na | 9971 | -0.409 | -0.5493 | Yes |
| 107 | RPLP2 | na | 10048 | -0.421 | -0.5502 | Yes |
| 108 | MRPS12 | na | 10055 | -0.422 | -0.5452 | Yes |
| 109 | RPL37 | na | 10057 | -0.423 | -0.5397 | Yes |
| 110 | MRPS21 | na | 10061 | -0.423 | -0.5344 | Yes |
| 111 | MTG1 | na | 10081 | -0.426 | -0.5305 | Yes |
| 112 | MRPS10 | na | 10111 | -0.431 | -0.5273 | Yes |
| 113 | LARP4 | na | 10149 | -0.437 | -0.5247 | Yes |
| 114 | MRPL57 | na | 10202 | -0.445 | -0.5232 | Yes |
| 115 | RPS8 | na | 10245 | -0.452 | -0.5209 | Yes |
| 116 | RPS6KL1 | na | 10309 | -0.461 | -0.5202 | Yes |
| 117 | RPL27 | na | 10333 | -0.467 | -0.5160 | Yes |
| 118 | MRPL37 | na | 10453 | -0.489 | -0.5197 | Yes |
| 119 | RPS28 | na | 10524 | -0.504 | -0.5190 | Yes |
| 120 | RPL30 | na | 10537 | -0.506 | -0.5134 | Yes |
| 121 | RPL22 | na | 10554 | -0.508 | -0.5081 | Yes |
| 122 | RPL36A | na | 10605 | -0.520 | -0.5055 | Yes |
| 123 | RPL39L | na | 10613 | -0.521 | -0.4993 | Yes |
| 124 | RPL14 | na | 10662 | -0.531 | -0.4964 | Yes |
| 125 | RPL29 | na | 10742 | -0.548 | -0.4959 | Yes |
| 126 | RPL37A | na | 10838 | -0.569 | -0.4965 | Yes |
| 127 | RPS19 | na | 10840 | -0.569 | -0.4891 | Yes |
| 128 | RPS12 | na | 10875 | -0.576 | -0.4844 | Yes |
| 129 | RPL24 | na | 10877 | -0.576 | -0.4770 | Yes |
| 130 | MRPL55 | na | 10925 | -0.587 | -0.4733 | Yes |
| 131 | RPL34 | na | 10933 | -0.588 | -0.4661 | Yes |
| 132 | RPL23 | na | 10981 | -0.602 | -0.4622 | Yes |
| 133 | RPL35A | na | 10982 | -0.602 | -0.4544 | Yes |
| 134 | RPS25 | na | 11017 | -0.613 | -0.4492 | Yes |
| 135 | RPS7 | na | 11068 | -0.627 | -0.4452 | Yes |
| 136 | RPL23A | na | 11076 | -0.629 | -0.4376 | Yes |
| 137 | RPL5 | na | 11121 | -0.643 | -0.4329 | Yes |
| 138 | PPARGC1A | na | 11134 | -0.645 | -0.4255 | Yes |
| 139 | RPS16 | na | 11138 | -0.645 | -0.4173 | Yes |
| 140 | RPL6 | na | 11150 | -0.650 | -0.4097 | Yes |
| 141 | RPL11 | na | 11195 | -0.662 | -0.4048 | Yes |
| 142 | RPS15A | na | 11196 | -0.662 | -0.3961 | Yes |
| 143 | RPLP1 | na | 11249 | -0.682 | -0.3916 | Yes |
| 144 | RPS26 | na | 11254 | -0.686 | -0.3829 | Yes |
| 145 | RPL35 | na | 11280 | -0.696 | -0.3759 | Yes |
| 146 | RPL26L1 | na | 11283 | -0.698 | -0.3669 | Yes |
| 147 | RPL31 | na | 11306 | -0.706 | -0.3596 | Yes |
| 148 | RPS21 | na | 11313 | -0.709 | -0.3508 | Yes |
| 149 | RPS3A | na | 11328 | -0.713 | -0.3426 | Yes |
| 150 | RPL21 | na | 11330 | -0.714 | -0.3334 | Yes |
| 151 | RPS10 | na | 11344 | -0.720 | -0.3250 | Yes |
| 152 | RPL32 | na | 11355 | -0.725 | -0.3164 | Yes |
| 153 | MRPL13 | na | 11364 | -0.731 | -0.3075 | Yes |
| 154 | RPS11 | na | 11371 | -0.735 | -0.2984 | Yes |
| 155 | MRPL38 | na | 11387 | -0.742 | -0.2899 | Yes |
| 156 | RPL12 | na | 11398 | -0.748 | -0.2810 | Yes |
| 157 | RPL7A | na | 11426 | -0.763 | -0.2733 | Yes |
| 158 | RPS15 | na | 11434 | -0.767 | -0.2638 | Yes |
| 159 | RPS27A | na | 11435 | -0.767 | -0.2538 | Yes |
| 160 | RPL15 | na | 11440 | -0.769 | -0.2441 | Yes |
| 161 | RPS29 | na | 11444 | -0.771 | -0.2342 | Yes |
| 162 | MRPS6 | na | 11465 | -0.783 | -0.2257 | Yes |
| 163 | RPL18A | na | 11474 | -0.789 | -0.2160 | Yes |
| 164 | RPS14 | na | 11478 | -0.792 | -0.2059 | Yes |
| 165 | RPL7 | na | 11484 | -0.796 | -0.1959 | Yes |
| 166 | RPLP0 | na | 11488 | -0.800 | -0.1857 | Yes |
| 167 | RPL27A | na | 11507 | -0.812 | -0.1766 | Yes |
| 168 | RPL9 | na | 11521 | -0.819 | -0.1670 | Yes |
| 169 | RACK1 | na | 11554 | -0.842 | -0.1586 | Yes |
| 170 | RPS2 | na | 11562 | -0.846 | -0.1482 | Yes |
| 171 | RPL13 | na | 11563 | -0.847 | -0.1371 | Yes |
| 172 | RPS20 | na | 11594 | -0.868 | -0.1282 | Yes |
| 173 | RPS27 | na | 11600 | -0.875 | -0.1172 | Yes |
| 174 | RPL19 | na | 11611 | -0.882 | -0.1065 | Yes |
| 175 | EEF2 | na | 11615 | -0.885 | -0.0952 | Yes |
| 176 | EIF3H | na | 11627 | -0.893 | -0.0844 | Yes |
| 177 | RPS13 | na | 11633 | -0.897 | -0.0731 | Yes |
| 178 | RPS3 | na | 11641 | -0.903 | -0.0619 | Yes |
| 179 | RPS17 | na | 11652 | -0.916 | -0.0507 | Yes |
| 180 | RPL10A | na | 11658 | -0.921 | -0.0391 | Yes |
| 181 | RPL4 | na | 11715 | -0.983 | -0.0309 | Yes |
| 182 | MRPS2 | na | 11754 | -1.021 | -0.0208 | Yes |
| 183 | RPL8 | na | 11772 | -1.039 | -0.0086 | Yes |
| 184 | RPS4Y1 | na | 11774 | -1.043 | 0.0049 | Yes |
| 185 | RPL3 | na | 11817 | -1.140 | 0.0163 | Yes |
Table: GSEA details [plain text format]

  

Fig 2: GOCC\_RIBOSOME      
 Blue-Pink O' Gram in the Space of the Analyzed GeneSet

  

Fig 3: GOCC\_RIBOSOME: Random ES distribution      
 Gene set null distribution of ES for **GOCC\_RIBOSOME**

  
