# Supplementary material for: Time-series transcriptome analysis identified differentially expressed genes in broiler chicken infected with mixed Eimeria species
Source: Front Genet. 2022 Aug 8;13:886781. doi: 10.3389/fgene.2022.886781 (PMC9393255; doi:10.3389/fgene.2022.886781)
Supplement: Supplementary file 2 [file DataSheet1.ZIP › 4dpi_GO.Gsea.1625071243202/GOCC_SMALL_RIBOSOMAL_SUBUNIT.html]

Details for gene set GOCC\_SMALL\_RIBOSOMAL\_SUBUNIT[GSEA]

|  || Dataset | TMM\_4dpi\_gct\_format\_4dpi\_gct\_format.Class\_4dpi.cls #PC\_versus\_NC.Class\_4dpi.cls #PC\_versus\_NC\_repos |
| Phenotype | Class\_4dpi.cls#PC\_versus\_NC\_repos |
| Upregulated in class | 0 |
| GeneSet | GOCC\_SMALL\_RIBOSOMAL\_SUBUNIT |
| Enrichment Score (ES) | -0.6505819 |
| Normalized Enrichment Score (NES) | -2.4873261 |
| Nominal p-value | 0.0 |
| FDR q-value | 0.0 |
| FWER p-Value | 0.0 |
Table: GSEA Results Summary

  

Fig 1: Enrichment plot: GOCC\_SMALL\_RIBOSOMAL\_SUBUNIT      
 Profile of the Running ES Score & Positions of GeneSet Members on the Rank Ordered List

  

| SYMBOL | TITLE | RANK IN GENE LIST | RANK METRIC SCORE | RUNNING ES | CORE ENRICHMENT || 1 | HBA1 | na | 1865 | 0.445 | -0.1398 | No |
| 2 | DHX29 | na | 2779 | 0.311 | -0.2049 | No |
| 3 | MRPS34 | na | 3713 | 0.198 | -0.2758 | No |
| 4 | MRPL42 | na | 4338 | 0.139 | -0.3229 | No |
| 5 | MRPS17 | na | 4827 | 0.095 | -0.3603 | No |
| 6 | MRPS18A | na | 4841 | 0.094 | -0.3579 | No |
| 7 | MRPS33 | na | 5356 | 0.049 | -0.3992 | No |
| 8 | MRPS14 | na | 5490 | 0.037 | -0.4089 | No |
| 9 | RPS27L | na | 5614 | 0.024 | -0.4183 | No |
| 10 | MRPS5 | na | 5706 | 0.016 | -0.4254 | No |
| 11 | MRPS9 | na | 5780 | 0.011 | -0.4311 | No |
| 12 | EIF2A | na | 6388 | -0.037 | -0.4805 | No |
| 13 | MRPS18C | na | 6501 | -0.046 | -0.4882 | No |
| 14 | MRPS35 | na | 6894 | -0.078 | -0.5181 | No |
| 15 | DAP3 | na | 6904 | -0.080 | -0.5160 | No |
| 16 | DDX3X | na | 6973 | -0.087 | -0.5185 | No |
| 17 | MRPS26 | na | 7030 | -0.092 | -0.5198 | No |
| 18 | MRPS11 | na | 7235 | -0.112 | -0.5328 | No |
| 19 | MRPS27 | na | 7421 | -0.129 | -0.5436 | No |
| 20 | MRPS16 | na | 7477 | -0.134 | -0.5433 | No |
| 21 | RPS23 | na | 8269 | -0.206 | -0.6020 | No |
| 22 | MRPS22 | na | 8420 | -0.220 | -0.6065 | No |
| 23 | MRPS31 | na | 8432 | -0.222 | -0.5994 | No |
| 24 | MRPS36 | na | 8541 | -0.233 | -0.5999 | No |
| 25 | MCTS1 | na | 8783 | -0.260 | -0.6106 | No |
| 26 | RPS6 | na | 8904 | -0.274 | -0.6106 | No |
| 27 | MRPS7 | na | 8923 | -0.275 | -0.6021 | No |
| 28 | EIF2D | na | 9431 | -0.340 | -0.6321 | No |
| 29 | UBA52 | na | 9532 | -0.353 | -0.6276 | No |
| 30 | RPS24 | na | 9689 | -0.372 | -0.6271 | No |
| 31 | MRPS28 | na | 9971 | -0.409 | -0.6356 | Yes |
| 32 | MRPS12 | na | 10055 | -0.422 | -0.6272 | Yes |
| 33 | MRPS21 | na | 10061 | -0.423 | -0.6121 | Yes |
| 34 | MRPS10 | na | 10111 | -0.431 | -0.6005 | Yes |
| 35 | LARP4 | na | 10149 | -0.437 | -0.5876 | Yes |
| 36 | RPS8 | na | 10245 | -0.452 | -0.5791 | Yes |
| 37 | RPS28 | na | 10524 | -0.504 | -0.5840 | Yes |
| 38 | RPS19 | na | 10840 | -0.569 | -0.5895 | Yes |
| 39 | RPS12 | na | 10875 | -0.576 | -0.5714 | Yes |
| 40 | RPS25 | na | 11017 | -0.613 | -0.5608 | Yes |
| 41 | RPS7 | na | 11068 | -0.627 | -0.5421 | Yes |
| 42 | RPS16 | na | 11138 | -0.645 | -0.5243 | Yes |
| 43 | RPS15A | na | 11196 | -0.662 | -0.5049 | Yes |
| 44 | RPS26 | na | 11254 | -0.686 | -0.4846 | Yes |
| 45 | RPS21 | na | 11313 | -0.709 | -0.4636 | Yes |
| 46 | RPS3A | na | 11328 | -0.713 | -0.4387 | Yes |
| 47 | RPS10 | na | 11344 | -0.720 | -0.4137 | Yes |
| 48 | RPS11 | na | 11371 | -0.735 | -0.3890 | Yes |
| 49 | RPS15 | na | 11434 | -0.767 | -0.3662 | Yes |
| 50 | RPS27A | na | 11435 | -0.767 | -0.3382 | Yes |
| 51 | RPS29 | na | 11444 | -0.771 | -0.3108 | Yes |
| 52 | MRPS6 | na | 11465 | -0.783 | -0.2839 | Yes |
| 53 | RPS14 | na | 11478 | -0.792 | -0.2560 | Yes |
| 54 | RACK1 | na | 11554 | -0.842 | -0.2315 | Yes |
| 55 | RPS2 | na | 11562 | -0.846 | -0.2012 | Yes |
| 56 | RPS20 | na | 11594 | -0.868 | -0.1721 | Yes |
| 57 | RPS27 | na | 11600 | -0.875 | -0.1406 | Yes |
| 58 | RPS13 | na | 11633 | -0.897 | -0.1105 | Yes |
| 59 | RPS3 | na | 11641 | -0.903 | -0.0781 | Yes |
| 60 | RPS17 | na | 11652 | -0.916 | -0.0456 | Yes |
| 61 | MRPS2 | na | 11754 | -1.021 | -0.0167 | Yes |
| 62 | RPS4Y1 | na | 11774 | -1.043 | 0.0198 | Yes |
Table: GSEA details [plain text format]

  

Fig 2: GOCC\_SMALL\_RIBOSOMAL\_SUBUNIT      
 Blue-Pink O' Gram in the Space of the Analyzed GeneSet

  

Fig 3: GOCC\_SMALL\_RIBOSOMAL\_SUBUNIT: Random ES distribution      
 Gene set null distribution of ES for **GOCC\_SMALL\_RIBOSOMAL\_SUBUNIT**

  
