# Supplementary material for: Time-series transcriptome analysis identified differentially expressed genes in broiler chicken infected with mixed Eimeria species
Source: Front Genet. 2022 Aug 8;13:886781. doi: 10.3389/fgene.2022.886781 (PMC9393255; doi:10.3389/fgene.2022.886781)
Supplement: Supplementary file 2 [file DataSheet1.ZIP › 4dpi_GO.Gsea.1625071243202/GOMF_RRNA_BINDING.html]

Details for gene set GOMF\_RRNA\_BINDING[GSEA]

|  || Dataset | TMM\_4dpi\_gct\_format\_4dpi\_gct\_format.Class\_4dpi.cls #PC\_versus\_NC.Class\_4dpi.cls #PC\_versus\_NC\_repos |
| Phenotype | Class\_4dpi.cls#PC\_versus\_NC\_repos |
| Upregulated in class | 0 |
| GeneSet | GOMF\_RRNA\_BINDING |
| Enrichment Score (ES) | -0.6415597 |
| Normalized Enrichment Score (NES) | -2.317253 |
| Nominal p-value | 0.0 |
| FDR q-value | 5.4584376E-5 |
| FWER p-Value | 0.001 |
Table: GSEA Results Summary

  

Fig 1: Enrichment plot: GOMF\_RRNA\_BINDING      
 Profile of the Running ES Score & Positions of GeneSet Members on the Rank Ordered List

  

| SYMBOL | TITLE | RANK IN GENE LIST | RANK METRIC SCORE | RUNNING ES | CORE ENRICHMENT || 1 | MDM2 | na | 2264 | 0.380 | -0.1717 | No |
| 2 | RPF2 | na | 2744 | 0.316 | -0.1972 | No |
| 3 | RNASEL | na | 2765 | 0.313 | -0.1844 | No |
| 4 | MRPL18 | na | 3417 | 0.231 | -0.2282 | No |
| 5 | SBDS | na | 3563 | 0.212 | -0.2306 | No |
| 6 | MRPS17 | na | 4827 | 0.095 | -0.3317 | No |
| 7 | MRPS18A | na | 4841 | 0.094 | -0.3285 | No |
| 8 | GTF3A | na | 5937 | -0.002 | -0.4199 | No |
| 9 | PTCD3 | na | 6126 | -0.017 | -0.4348 | No |
| 10 | MRPS18C | na | 6501 | -0.046 | -0.4640 | No |
| 11 | RPF1 | na | 6697 | -0.063 | -0.4774 | No |
| 12 | IMP3 | na | 6880 | -0.077 | -0.4890 | No |
| 13 | MRPS11 | na | 7235 | -0.112 | -0.5135 | No |
| 14 | ERAL1 | na | 7352 | -0.123 | -0.5175 | No |
| 15 | MRPS27 | na | 7421 | -0.129 | -0.5172 | No |
| 16 | ERI1 | na | 7552 | -0.139 | -0.5217 | No |
| 17 | UTP23 | na | 8279 | -0.207 | -0.5728 | No |
| 18 | MRPL20 | na | 8418 | -0.220 | -0.5742 | No |
| 19 | MRPS7 | na | 8923 | -0.275 | -0.6037 | No |
| 20 | RCC1L | na | 9107 | -0.296 | -0.6053 | No |
| 21 | MRPL16 | na | 9210 | -0.310 | -0.5995 | No |
| 22 | NSUN4 | na | 9550 | -0.356 | -0.6115 | No |
| 23 | RRS1 | na | 9732 | -0.378 | -0.6092 | No |
| 24 | FASTKD2 | na | 9983 | -0.411 | -0.6111 | No |
| 25 | RPL37 | na | 10057 | -0.423 | -0.5977 | No |
| 26 | DDX28 | na | 10412 | -0.481 | -0.6051 | No |
| 27 | EMG1 | na | 10849 | -0.573 | -0.6151 | Yes |
| 28 | DDX21 | na | 10923 | -0.586 | -0.5942 | Yes |
| 29 | RPL23 | na | 10981 | -0.602 | -0.5712 | Yes |
| 30 | NOL12 | na | 10991 | -0.605 | -0.5441 | Yes |
| 31 | RPL23A | na | 11076 | -0.629 | -0.5221 | Yes |
| 32 | RPL5 | na | 11121 | -0.643 | -0.4961 | Yes |
| 33 | RPL11 | na | 11195 | -0.662 | -0.4717 | Yes |
| 34 | RPS11 | na | 11371 | -0.735 | -0.4525 | Yes |
| 35 | RPL12 | na | 11398 | -0.748 | -0.4201 | Yes |
| 36 | MRPS6 | na | 11465 | -0.783 | -0.3895 | Yes |
| 37 | RPS14 | na | 11478 | -0.792 | -0.3540 | Yes |
| 38 | RPLP0 | na | 11488 | -0.800 | -0.3179 | Yes |
| 39 | RPL9 | na | 11521 | -0.819 | -0.2828 | Yes |
| 40 | EEF2 | na | 11615 | -0.885 | -0.2498 | Yes |
| 41 | RPS13 | na | 11633 | -0.897 | -0.2098 | Yes |
| 42 | RPS3 | na | 11641 | -0.903 | -0.1688 | Yes |
| 43 | RPL8 | na | 11772 | -1.039 | -0.1317 | Yes |
| 44 | RPS4Y1 | na | 11774 | -1.043 | -0.0837 | Yes |
| 45 | TST | na | 11798 | -1.099 | -0.0349 | Yes |
| 46 | RPL3 | na | 11817 | -1.140 | 0.0161 | Yes |
Table: GSEA details [plain text format]

  

Fig 2: GOMF\_RRNA\_BINDING      
 Blue-Pink O' Gram in the Space of the Analyzed GeneSet

  

Fig 3: GOMF\_RRNA\_BINDING: Random ES distribution      
 Gene set null distribution of ES for **GOMF\_RRNA\_BINDING**

  
