# Supplementary material for: Time-series transcriptome analysis identified differentially expressed genes in broiler chicken infected with mixed Eimeria species
Source: Front Genet. 2022 Aug 8;13:886781. doi: 10.3389/fgene.2022.886781 (PMC9393255; doi:10.3389/fgene.2022.886781)
Supplement: Supplementary file 2 [file DataSheet1.ZIP › 4dpi_GO.Gsea.1625071243202/gsea_report_for_0_1625071243202.html]

Report for 0 1625071243202 [GSEA]

| GS  follow link to MSigDB | GS DETAILS | SIZE | ES | NES | NOM p-val | FDR q-val | FWER p-val | RANK AT MAX | LEADING EDGE || 1 | GOCC\_CYTOSOLIC\_RIBOSOME | Details ... | 85 | -0.73 | -3.00 | 0.000 | 0.000 | 0.000 | 1964 | tags=75%, list=16%, signal=89% |
| 2 | GOBP\_COTRANSLATIONAL\_PROTEIN\_TARGETING\_TO\_MEMBRANE | Details ... | 87 | -0.70 | -2.89 | 0.000 | 0.000 | 0.000 | 1488 | tags=66%, list=12%, signal=74% |
| 3 | GOMF\_STRUCTURAL\_CONSTITUENT\_OF\_RIBOSOME | Details ... | 133 | -0.65 | -2.85 | 0.000 | 0.000 | 0.000 | 1964 | tags=53%, list=16%, signal=62% |
| 4 | GOBP\_NUCLEAR\_TRANSCRIBED\_MRNA\_CATABOLIC\_PROCESS\_NONSENSE\_MEDIATED\_DECAY | Details ... | 101 | -0.67 | -2.83 | 0.000 | 0.000 | 0.000 | 1767 | tags=61%, list=15%, signal=71% |
| 5 | GOCC\_CYTOSOLIC\_LARGE\_RIBOSOMAL\_SUBUNIT | Details ... | 44 | -0.79 | -2.81 | 0.000 | 0.000 | 0.000 | 1964 | tags=86%, list=16%, signal=103% |
| 6 | GOCC\_RIBOSOMAL\_SUBUNIT | Details ... | 155 | -0.62 | -2.77 | 0.000 | 0.000 | 0.000 | 1964 | tags=48%, list=16%, signal=56% |
| 7 | GOBP\_TRANSLATIONAL\_INITIATION | Details ... | 157 | -0.60 | -2.70 | 0.000 | 0.000 | 0.000 | 2649 | tags=62%, list=22%, signal=78% |
| 8 | GOBP\_ESTABLISHMENT\_OF\_PROTEIN\_LOCALIZATION\_TO\_ENDOPLASMIC\_RETICULUM | Details ... | 98 | -0.64 | -2.69 | 0.000 | 0.000 | 0.000 | 1488 | tags=59%, list=12%, signal=67% |
| 9 | GOBP\_VIRAL\_GENE\_EXPRESSION | Details ... | 161 | -0.58 | -2.62 | 0.000 | 0.000 | 0.000 | 2817 | tags=57%, list=23%, signal=73% |
| 10 | GOCC\_RIBOSOME | Details ... | 185 | -0.56 | -2.57 | 0.000 | 0.000 | 0.000 | 2351 | tags=46%, list=20%, signal=57% |
| 11 | GOBP\_CYTOPLASMIC\_TRANSLATION | Details ... | 80 | -0.63 | -2.53 | 0.000 | 0.000 | 0.000 | 1972 | tags=55%, list=16%, signal=65% |
| 12 | GOCC\_SMALL\_RIBOSOMAL\_SUBUNIT | Details ... | 62 | -0.65 | -2.49 | 0.000 | 0.000 | 0.000 | 2041 | tags=52%, list=17%, signal=62% |
| 13 | GOBP\_PROTEIN\_LOCALIZATION\_TO\_ENDOPLASMIC\_RETICULUM | Details ... | 118 | -0.57 | -2.48 | 0.000 | 0.000 | 0.000 | 1488 | tags=52%, list=12%, signal=58% |
| 14 | GOCC\_CYTOSOLIC\_SMALL\_RIBOSOMAL\_SUBUNIT | Details ... | 37 | -0.71 | -2.46 | 0.000 | 0.000 | 0.000 | 1863 | tags=68%, list=16%, signal=80% |
| 15 | GOCC\_LARGE\_RIBOSOMAL\_SUBUNIT | Details ... | 95 | -0.59 | -2.45 | 0.000 | 0.000 | 0.000 | 1964 | tags=45%, list=16%, signal=54% |
| 16 | GOBP\_RIBOSOMAL\_LARGE\_SUBUNIT\_BIOGENESIS | Details ... | 63 | -0.63 | -2.41 | 0.000 | 0.000 | 0.000 | 2439 | tags=48%, list=20%, signal=59% |
| 17 | GOMF\_RRNA\_BINDING | Details ... | 46 | -0.64 | -2.32 | 0.000 | 0.000 | 0.001 | 1163 | tags=43%, list=10%, signal=48% |
| 18 | GOBP\_PROTEIN\_TARGETING\_TO\_MEMBRANE | Details ... | 152 | -0.52 | -2.31 | 0.000 | 0.000 | 0.002 | 1488 | tags=43%, list=12%, signal=48% |
| 19 | GOCC\_POLYSOMAL\_RIBOSOME | Details ... | 28 | -0.70 | -2.25 | 0.000 | 0.000 | 0.004 | 1488 | tags=64%, list=12%, signal=73% |
| 20 | GOBP\_NUCLEAR\_TRANSCRIBED\_MRNA\_CATABOLIC\_PROCESS | Details ... | 172 | -0.49 | -2.23 | 0.000 | 0.000 | 0.008 | 1488 | tags=36%, list=12%, signal=41% |
| 21 | GOCC\_EUKARYOTIC\_TRANSLATION\_INITIATION\_FACTOR\_3\_COMPLEX |  | 14 | -0.81 | -2.17 | 0.000 | 0.001 | 0.027 | 1934 | tags=79%, list=16%, signal=94% |
| 22 | GOBP\_CYTOPLASMIC\_TRANSLATIONAL\_INITIATION |  | 27 | -0.67 | -2.16 | 0.000 | 0.001 | 0.035 | 1785 | tags=52%, list=15%, signal=61% |
| 23 | GOBP\_FORMATION\_OF\_CYTOPLASMIC\_TRANSLATION\_INITIATION\_COMPLEX |  | 13 | -0.81 | -2.14 | 0.000 | 0.002 | 0.045 | 1785 | tags=77%, list=15%, signal=90% |
| 24 | GOBP\_RIBOSOMAL\_LARGE\_SUBUNIT\_ASSEMBLY |  | 22 | -0.70 | -2.12 | 0.000 | 0.003 | 0.073 | 2351 | tags=64%, list=20%, signal=79% |
| 25 | GOCC\_EUKARYOTIC\_48S\_PREINITIATION\_COMPLEX |  | 12 | -0.82 | -2.11 | 0.000 | 0.003 | 0.083 | 1785 | tags=83%, list=15%, signal=98% |
| 26 | GOCC\_TRANSLATION\_PREINITIATION\_COMPLEX |  | 15 | -0.76 | -2.10 | 0.000 | 0.004 | 0.102 | 2130 | tags=80%, list=18%, signal=97% |
| 27 | GOBP\_STEROID\_CATABOLIC\_PROCESS |  | 16 | -0.74 | -2.06 | 0.000 | 0.007 | 0.197 | 803 | tags=50%, list=7%, signal=54% |
| 28 | GOMF\_TRANSLATION\_INITIATION\_FACTOR\_ACTIVITY |  | 42 | -0.57 | -2.05 | 0.000 | 0.009 | 0.228 | 2598 | tags=48%, list=22%, signal=61% |
| 29 | GOCC\_POLYSOME |  | 57 | -0.53 | -1.99 | 0.000 | 0.020 | 0.473 | 1610 | tags=39%, list=13%, signal=44% |
| 30 | GOMF\_ALDO\_KETO\_REDUCTASE\_NADP\_ACTIVITY |  | 16 | -0.71 | -1.99 | 0.001 | 0.022 | 0.511 | 1385 | tags=50%, list=12%, signal=56% |
| 31 | GOMF\_ALCOHOL\_DEHYDROGENASE\_NADPPLUS\_ACTIVITY |  | 12 | -0.77 | -1.98 | 0.000 | 0.023 | 0.536 | 967 | tags=50%, list=8%, signal=54% |
| 32 | GOBP\_SULFUR\_COMPOUND\_TRANSPORT |  | 38 | -0.56 | -1.96 | 0.000 | 0.031 | 0.659 | 1603 | tags=32%, list=13%, signal=36% |
| 33 | GOBP\_RIBOSOME\_BIOGENESIS |  | 243 | -0.41 | -1.95 | 0.000 | 0.034 | 0.701 | 3935 | tags=50%, list=33%, signal=73% |
| 34 | GOBP\_COLLATERAL\_SPROUTING |  | 15 | -0.70 | -1.94 | 0.001 | 0.037 | 0.737 | 82 | tags=27%, list=1%, signal=27% |
| 35 | GOBP\_RIBOSOME\_ASSEMBLY |  | 50 | -0.52 | -1.93 | 0.000 | 0.043 | 0.797 | 2351 | tags=44%, list=20%, signal=54% |
| 36 | GOBP\_3\_PHOSPHOADENOSINE\_5\_PHOSPHOSULFATE\_METABOLIC\_PROCESS |  | 12 | -0.75 | -1.93 | 0.001 | 0.044 | 0.817 | 2014 | tags=67%, list=17%, signal=80% |
| 37 | GOMF\_ENOYL\_COA\_HYDRATASE\_ACTIVITY |  | 9 | -0.81 | -1.92 | 0.000 | 0.048 | 0.850 | 1313 | tags=56%, list=11%, signal=62% |
| 38 | GOMF\_SUPEROXIDE\_GENERATING\_NADPH\_OXIDASE\_ACTIVATOR\_ACTIVITY |  | 8 | -0.84 | -1.91 | 0.000 | 0.055 | 0.889 | 1292 | tags=50%, list=11%, signal=56% |
| 39 | GOMF\_SMALL\_RIBOSOMAL\_SUBUNIT\_RRNA\_BINDING |  | 8 | -0.84 | -1.90 | 0.001 | 0.057 | 0.907 | 547 | tags=50%, list=5%, signal=52% |
| 40 | GOBP\_REGULATION\_OF\_COLLATERAL\_SPROUTING |  | 10 | -0.78 | -1.90 | 0.002 | 0.057 | 0.913 | 82 | tags=40%, list=1%, signal=40% |
| 41 | GOBP\_PROTEIN\_TARGETING |  | 342 | -0.38 | -1.89 | 0.000 | 0.069 | 0.954 | 3323 | tags=46%, list=28%, signal=62% |
| 42 | GOMF\_TRANSLATION\_FACTOR\_ACTIVITY\_RNA\_BINDING |  | 67 | -0.48 | -1.89 | 0.001 | 0.069 | 0.959 | 2598 | tags=39%, list=22%, signal=49% |
| 43 | GOBP\_MITOCHONDRIAL\_TRNA\_PROCESSING |  | 12 | -0.72 | -1.87 | 0.005 | 0.084 | 0.980 | 1978 | tags=67%, list=16%, signal=80% |
| 44 | GOBP\_REGULATION\_OF\_MAMMARY\_GLAND\_EPITHELIAL\_CELL\_PROLIFERATION |  | 15 | -0.68 | -1.86 | 0.003 | 0.088 | 0.985 | 1956 | tags=53%, list=16%, signal=64% |
| 45 | GOMF\_SULFATE\_TRANSMEMBRANE\_TRANSPORTER\_ACTIVITY |  | 12 | -0.72 | -1.85 | 0.003 | 0.098 | 0.993 | 78 | tags=33%, list=1%, signal=34% |
| 46 | GOBP\_SULFATE\_TRANSPORT |  | 13 | -0.70 | -1.85 | 0.003 | 0.102 | 0.994 | 78 | tags=31%, list=1%, signal=31% |
| 47 | GOMF\_SULFUR\_COMPOUND\_TRANSMEMBRANE\_TRANSPORTER\_ACTIVITY |  | 36 | -0.54 | -1.85 | 0.004 | 0.101 | 0.995 | 1603 | tags=33%, list=13%, signal=38% |
| 48 | GOBP\_RESPIRATORY\_BURST |  | 26 | -0.58 | -1.85 | 0.002 | 0.101 | 0.995 | 2209 | tags=58%, list=18%, signal=71% |
| 49 | GOMF\_SECONDARY\_ACTIVE\_SULFATE\_TRANSMEMBRANE\_TRANSPORTER\_ACTIVITY |  | 8 | -0.81 | -1.85 | 0.003 | 0.100 | 0.996 | 78 | tags=50%, list=1%, signal=50% |
| 50 | GOBP\_PEPTIDE\_BIOSYNTHETIC\_PROCESS |  | 500 | -0.36 | -1.84 | 0.000 | 0.100 | 0.996 | 2352 | tags=30%, list=20%, signal=36% |
| 51 | GOMF\_AXON\_GUIDANCE\_RECEPTOR\_ACTIVITY |  | 6 | -0.88 | -1.84 | 0.001 | 0.099 | 0.996 | 1489 | tags=67%, list=12%, signal=76% |
| 52 | GOCC\_90S\_PRERIBOSOME |  | 24 | -0.60 | -1.84 | 0.003 | 0.101 | 0.997 | 3796 | tags=63%, list=32%, signal=91% |
| 53 | GOMF\_HYDRO\_LYASE\_ACTIVITY |  | 45 | -0.51 | -1.84 | 0.002 | 0.103 | 0.998 | 2645 | tags=44%, list=22%, signal=57% |
| 54 | GOMF\_BICARBONATE\_TRANSMEMBRANE\_TRANSPORTER\_ACTIVITY |  | 17 | -0.66 | -1.84 | 0.004 | 0.101 | 0.998 | 489 | tags=29%, list=4%, signal=31% |
| 55 | GOBP\_PURINE\_NUCLEOSIDE\_BIOSYNTHETIC\_PROCESS |  | 16 | -0.66 | -1.84 | 0.003 | 0.100 | 0.998 | 2219 | tags=50%, list=18%, signal=61% |
| 56 | GOBP\_BETA\_CATENIN\_TCF\_COMPLEX\_ASSEMBLY |  | 29 | -0.57 | -1.83 | 0.001 | 0.109 | 0.999 | 1797 | tags=38%, list=15%, signal=44% |
| 57 | GOBP\_INORGANIC\_ANION\_TRANSMEMBRANE\_TRANSPORT |  | 65 | -0.47 | -1.83 | 0.000 | 0.108 | 0.999 | 644 | tags=20%, list=5%, signal=21% |
| 58 | GOMF\_CARBON\_SULFUR\_LYASE\_ACTIVITY |  | 9 | -0.77 | -1.82 | 0.003 | 0.112 | 0.999 | 1940 | tags=56%, list=16%, signal=66% |
| 59 | GOMF\_ALDITOL\_NADPPLUS\_1\_OXIDOREDUCTASE\_ACTIVITY |  | 7 | -0.82 | -1.81 | 0.002 | 0.128 | 1.000 | 803 | tags=57%, list=7%, signal=61% |
| 60 | GOBP\_CELL\_REDOX\_HOMEOSTASIS |  | 34 | -0.53 | -1.80 | 0.002 | 0.141 | 1.000 | 1300 | tags=38%, list=11%, signal=43% |
| 61 | GOBP\_NEUROMUSCULAR\_SYNAPTIC\_TRANSMISSION |  | 14 | -0.67 | -1.80 | 0.004 | 0.148 | 1.000 | 2170 | tags=64%, list=18%, signal=78% |
| 62 | GOMF\_TRANSLATION\_REGULATOR\_ACTIVITY\_NUCLEIC\_ACID\_BINDING |  | 80 | -0.44 | -1.79 | 0.000 | 0.155 | 1.000 | 1934 | tags=29%, list=16%, signal=34% |
| 63 | GOBP\_TRNA\_THREONYLCARBAMOYLADENOSINE\_METABOLIC\_PROCESS |  | 11 | -0.72 | -1.79 | 0.003 | 0.153 | 1.000 | 1978 | tags=64%, list=16%, signal=76% |
| 64 | GOBP\_HORMONE\_CATABOLIC\_PROCESS |  | 8 | -0.78 | -1.79 | 0.003 | 0.159 | 1.000 | 814 | tags=63%, list=7%, signal=67% |
| 65 | GOBP\_EMBRYONIC\_SKELETAL\_SYSTEM\_MORPHOGENESIS |  | 50 | -0.48 | -1.78 | 0.001 | 0.165 | 1.000 | 1121 | tags=36%, list=9%, signal=40% |
| 66 | GOBP\_GUANOSINE\_CONTAINING\_COMPOUND\_BIOSYNTHETIC\_PROCESS |  | 8 | -0.78 | -1.78 | 0.004 | 0.163 | 1.000 | 2219 | tags=75%, list=18%, signal=92% |
| 67 | GOMF\_INORGANIC\_ANION\_TRANSMEMBRANE\_TRANSPORTER\_ACTIVITY |  | 81 | -0.44 | -1.78 | 0.001 | 0.163 | 1.000 | 644 | tags=20%, list=5%, signal=21% |
| 68 | GOMF\_ACYL\_COA\_DEHYDROGENASE\_ACTIVITY |  | 8 | -0.78 | -1.78 | 0.003 | 0.161 | 1.000 | 1697 | tags=75%, list=14%, signal=87% |
| 69 | GOBP\_ESTABLISHMENT\_OF\_PROTEIN\_LOCALIZATION\_TO\_MEMBRANE |  | 259 | -0.37 | -1.78 | 0.000 | 0.164 | 1.000 | 1488 | tags=28%, list=12%, signal=31% |
| 70 | GOCC\_EUKARYOTIC\_TRANSLATION\_INITIATION\_FACTOR\_3\_COMPLEX\_EIF3M |  | 6 | -0.85 | -1.78 | 0.003 | 0.165 | 1.000 | 1785 | tags=100%, list=15%, signal=117% |
| 71 | GOMF\_OXIDOREDUCTASE\_ACTIVITY\_ACTING\_ON\_METAL\_IONS\_NAD\_OR\_NADP\_AS\_ACCEPTOR |  | 7 | -0.81 | -1.78 | 0.006 | 0.165 | 1.000 | 292 | tags=43%, list=2%, signal=44% |
| 72 | GOBP\_MATURATION\_OF\_LSU\_RRNA |  | 27 | -0.56 | -1.77 | 0.006 | 0.165 | 1.000 | 2439 | tags=41%, list=20%, signal=51% |
| 73 | GOBP\_MAMMARY\_GLAND\_EPITHELIAL\_CELL\_DIFFERENTIATION |  | 11 | -0.70 | -1.76 | 0.005 | 0.182 | 1.000 | 2001 | tags=45%, list=17%, signal=54% |
| 74 | GOMF\_CARBON\_OXYGEN\_LYASE\_ACTIVITY |  | 58 | -0.47 | -1.76 | 0.002 | 0.181 | 1.000 | 2778 | tags=45%, list=23%, signal=58% |
| 75 | GOBP\_ORGANIC\_ACID\_CATABOLIC\_PROCESS |  | 192 | -0.38 | -1.75 | 0.000 | 0.201 | 1.000 | 1792 | tags=31%, list=15%, signal=36% |
| 76 | GOBP\_VIRAL\_TRANSLATIONAL\_TERMINATION\_REINITIATION |  | 5 | -0.88 | -1.75 | 0.002 | 0.210 | 1.000 | 1427 | tags=100%, list=12%, signal=113% |
| 77 | GOBP\_PROTEIN\_TRANSMEMBRANE\_IMPORT\_INTO\_INTRACELLULAR\_ORGANELLE |  | 30 | -0.53 | -1.75 | 0.005 | 0.215 | 1.000 | 3284 | tags=50%, list=27%, signal=69% |
| 78 | GOBP\_PEPTIDE\_METABOLIC\_PROCESS |  | 614 | -0.34 | -1.75 | 0.000 | 0.213 | 1.000 | 3268 | tags=39%, list=27%, signal=51% |
| 79 | GOBP\_NCRNA\_PROCESSING |  | 297 | -0.36 | -1.74 | 0.000 | 0.212 | 1.000 | 3940 | tags=48%, list=33%, signal=69% |
| 80 | GOCC\_MITOCHONDRIAL\_PROTEIN\_CONTAINING\_COMPLEX |  | 196 | -0.38 | -1.74 | 0.000 | 0.211 | 1.000 | 4653 | tags=57%, list=39%, signal=91% |
| 81 | GOBP\_MITOCHONDRIAL\_RNA\_PROCESSING |  | 15 | -0.64 | -1.74 | 0.012 | 0.216 | 1.000 | 1978 | tags=53%, list=16%, signal=64% |
| 82 | GOBP\_PYRIMIDINE\_RIBONUCLEOSIDE\_TRIPHOSPHATE\_BIOSYNTHETIC\_PROCESS |  | 11 | -0.69 | -1.74 | 0.011 | 0.220 | 1.000 | 1626 | tags=55%, list=14%, signal=63% |
| 83 | GOBP\_BICARBONATE\_TRANSPORT |  | 31 | -0.52 | -1.73 | 0.006 | 0.230 | 1.000 | 489 | tags=19%, list=4%, signal=20% |
| 84 | GOMF\_SOLUTE\_ANION\_ANTIPORTER\_ACTIVITY |  | 23 | -0.56 | -1.73 | 0.007 | 0.229 | 1.000 | 78 | tags=17%, list=1%, signal=17% |
| 85 | GOMF\_ANNEALING\_HELICASE\_ACTIVITY |  | 5 | -0.86 | -1.73 | 0.004 | 0.230 | 1.000 | 1175 | tags=60%, list=10%, signal=66% |
| 86 | GOBP\_SYNAPTIC\_TRANSMISSION\_CHOLINERGIC |  | 11 | -0.69 | -1.73 | 0.012 | 0.236 | 1.000 | 1245 | tags=45%, list=10%, signal=51% |
| 87 | GOBP\_RHYTHMIC\_BEHAVIOR |  | 26 | -0.54 | -1.72 | 0.010 | 0.243 | 1.000 | 1952 | tags=46%, list=16%, signal=55% |
| 88 | GOBP\_DNA\_STRAND\_RENATURATION |  | 5 | -0.86 | -1.72 | 0.004 | 0.242 | 1.000 | 1175 | tags=60%, list=10%, signal=66% |
| 89 | GOBP\_HOMOSERINE\_METABOLIC\_PROCESS |  | 4 | -0.94 | -1.72 | 0.001 | 0.242 | 1.000 | 779 | tags=100%, list=6%, signal=107% |
| 90 | GOBP\_MATURATION\_OF\_5\_8S\_RRNA\_FROM\_TRICISTRONIC\_RRNA\_TRANSCRIPT\_SSU\_RRNA\_5\_8S\_RRNA\_LSU\_RRNA |  | 21 | -0.57 | -1.72 | 0.007 | 0.243 | 1.000 | 2309 | tags=43%, list=19%, signal=53% |
| 91 | GOBP\_PIGMENT\_CATABOLIC\_PROCESS |  | 4 | -0.94 | -1.72 | 0.000 | 0.253 | 1.000 | 777 | tags=75%, list=6%, signal=80% |
| 92 | GOMF\_5S\_RRNA\_BINDING |  | 10 | -0.70 | -1.72 | 0.011 | 0.251 | 1.000 | 891 | tags=50%, list=7%, signal=54% |
| 93 | GOBP\_RRNA\_METABOLIC\_PROCESS |  | 187 | -0.37 | -1.71 | 0.000 | 0.260 | 1.000 | 3935 | tags=50%, list=33%, signal=73% |
| 94 | GOMF\_GLUTATHIONE\_TRANSFERASE\_ACTIVITY |  | 14 | -0.64 | -1.71 | 0.014 | 0.258 | 1.000 | 1267 | tags=43%, list=11%, signal=48% |
| 95 | GOBP\_GTP\_BIOSYNTHETIC\_PROCESS |  | 6 | -0.82 | -1.71 | 0.009 | 0.260 | 1.000 | 1626 | tags=67%, list=14%, signal=77% |
| 96 | GOMF\_NEUROTROPHIN\_BINDING |  | 8 | -0.74 | -1.71 | 0.008 | 0.267 | 1.000 | 156 | tags=25%, list=1%, signal=25% |
| 97 | GOBP\_PROXIMAL\_DISTAL\_PATTERN\_FORMATION |  | 18 | -0.59 | -1.71 | 0.004 | 0.264 | 1.000 | 1168 | tags=39%, list=10%, signal=43% |
| 98 | GOBP\_RIBONUCLEOPROTEIN\_COMPLEX\_BIOGENESIS |  | 342 | -0.35 | -1.70 | 0.000 | 0.266 | 1.000 | 3733 | tags=42%, list=31%, signal=59% |
| 99 | GOCC\_ISWI\_TYPE\_COMPLEX |  | 7 | -0.79 | -1.70 | 0.010 | 0.265 | 1.000 | 447 | tags=29%, list=4%, signal=30% |
| 100 | GOBP\_RIBOSOMAL\_SMALL\_SUBUNIT\_BIOGENESIS |  | 59 | -0.44 | -1.70 | 0.002 | 0.263 | 1.000 | 3219 | tags=54%, list=27%, signal=74% |
| 101 | GOBP\_NEGATIVE\_REGULATION\_OF\_COLLATERAL\_SPROUTING |  | 5 | -0.87 | -1.70 | 0.004 | 0.265 | 1.000 | 82 | tags=60%, list=1%, signal=60% |
| 102 | GOCC\_SECONDARY\_LYSOSOME |  | 13 | -0.65 | -1.70 | 0.016 | 0.264 | 1.000 | 3240 | tags=77%, list=27%, signal=105% |
| 103 | GOBP\_VIRAL\_TRANSLATION |  | 14 | -0.63 | -1.70 | 0.011 | 0.270 | 1.000 | 1427 | tags=43%, list=12%, signal=49% |
| 104 | GOBP\_HYPOTHALAMUS\_CELL\_DIFFERENTIATION |  | 5 | -0.86 | -1.70 | 0.007 | 0.273 | 1.000 | 640 | tags=40%, list=5%, signal=42% |
| 105 | GOBP\_MONOCARBOXYLIC\_ACID\_CATABOLIC\_PROCESS |  | 100 | -0.41 | -1.69 | 0.001 | 0.274 | 1.000 | 1786 | tags=35%, list=15%, signal=41% |
| 106 | GOMF\_GALACTOSIDASE\_ACTIVITY |  | 6 | -0.81 | -1.69 | 0.007 | 0.279 | 1.000 | 1366 | tags=83%, list=11%, signal=94% |
| 107 | GOCC\_RIBONUCLEOPROTEIN\_COMPLEX |  | 536 | -0.33 | -1.69 | 0.000 | 0.278 | 1.000 | 2480 | tags=26%, list=21%, signal=32% |
| 108 | GOMF\_CHLORIDE\_TRANSMEMBRANE\_TRANSPORTER\_ACTIVITY |  | 50 | -0.46 | -1.68 | 0.004 | 0.294 | 1.000 | 591 | tags=22%, list=5%, signal=23% |
| 109 | GOBP\_PIGMENT\_METABOLIC\_PROCESS |  | 47 | -0.47 | -1.68 | 0.005 | 0.291 | 1.000 | 3311 | tags=45%, list=28%, signal=61% |
| 110 | GOCC\_INTRINSIC\_COMPONENT\_OF\_EXTERNAL\_SIDE\_OF\_PLASMA\_MEMBRANE |  | 11 | -0.67 | -1.68 | 0.014 | 0.303 | 1.000 | 1975 | tags=55%, list=16%, signal=65% |
| 111 | GOBP\_TRNA\_MODIFICATION |  | 69 | -0.43 | -1.68 | 0.001 | 0.311 | 1.000 | 3120 | tags=46%, list=26%, signal=62% |
| 112 | GOBP\_WATER\_SOLUBLE\_VITAMIN\_METABOLIC\_PROCESS |  | 58 | -0.44 | -1.68 | 0.003 | 0.312 | 1.000 | 1580 | tags=29%, list=13%, signal=34% |
| 113 | GOMF\_OXIDOREDUCTASE\_ACTIVITY\_ACTING\_ON\_OTHER\_NITROGENOUS\_COMPOUNDS\_AS\_DONORS |  | 4 | -0.91 | -1.67 | 0.002 | 0.314 | 1.000 | 923 | tags=50%, list=8%, signal=54% |
| 114 | GOBP\_ESTABLISHMENT\_OF\_PROTEIN\_LOCALIZATION\_TO\_ORGANELLE |  | 441 | -0.33 | -1.67 | 0.000 | 0.319 | 1.000 | 3122 | tags=37%, list=26%, signal=48% |
| 115 | GOBP\_RESPIRATORY\_ELECTRON\_TRANSPORT\_CHAIN |  | 85 | -0.41 | -1.67 | 0.001 | 0.318 | 1.000 | 4262 | tags=54%, list=35%, signal=83% |
| 116 | GOMF\_OXIDOREDUCTASE\_ACTIVITY\_ACTING\_ON\_PAIRED\_DONORS\_WITH\_INCORPORATION\_OR\_REDUCTION\_OF\_MOLECULAR\_OXYGEN\_REDUCED\_PTERIDINE\_AS\_ONE\_DONOR\_AND\_INCORPORATION\_OF\_ONE\_ATOM\_OF\_OXYGEN |  | 4 | -0.92 | -1.67 | 0.001 | 0.317 | 1.000 | 319 | tags=50%, list=3%, signal=51% |
| 117 | GOBP\_CYSTEINE\_METABOLIC\_PROCESS |  | 8 | -0.73 | -1.67 | 0.020 | 0.315 | 1.000 | 779 | tags=63%, list=6%, signal=67% |
| 118 | GOMF\_MANNOSE\_BINDING |  | 10 | -0.68 | -1.67 | 0.018 | 0.315 | 1.000 | 2027 | tags=60%, list=17%, signal=72% |
| 119 | GOBP\_ATP\_SYNTHESIS\_COUPLED\_ELECTRON\_TRANSPORT |  | 71 | -0.42 | -1.67 | 0.002 | 0.314 | 1.000 | 4262 | tags=52%, list=35%, signal=80% |
| 120 | GOBP\_SULFATION |  | 12 | -0.65 | -1.67 | 0.013 | 0.315 | 1.000 | 2650 | tags=67%, list=22%, signal=85% |
| 121 | GOMF\_UBIQUITIN\_LIGASE\_INHIBITOR\_ACTIVITY |  | 5 | -0.85 | -1.67 | 0.008 | 0.317 | 1.000 | 1031 | tags=80%, list=9%, signal=87% |
| 122 | GOBP\_REGULATION\_OF\_RESPIRATORY\_BURST |  | 12 | -0.66 | -1.67 | 0.017 | 0.316 | 1.000 | 2209 | tags=58%, list=18%, signal=71% |
| 123 | GOBP\_HEME\_METABOLIC\_PROCESS |  | 26 | -0.52 | -1.66 | 0.014 | 0.314 | 1.000 | 3121 | tags=50%, list=26%, signal=67% |
| 124 | GOMF\_UBIQUITIN\_PROTEIN\_TRANSFERASE\_INHIBITOR\_ACTIVITY |  | 6 | -0.80 | -1.66 | 0.011 | 0.314 | 1.000 | 1031 | tags=67%, list=9%, signal=73% |
| 125 | GOBP\_NEGATIVE\_REGULATION\_OF\_MITOCHONDRIAL\_OUTER\_MEMBRANE\_PERMEABILIZATION\_INVOLVED\_IN\_APOPTOTIC\_SIGNALING\_PATHWAY |  | 5 | -0.85 | -1.66 | 0.008 | 0.313 | 1.000 | 428 | tags=40%, list=4%, signal=41% |
| 126 | GOBP\_LYSOSOMAL\_MEMBRANE\_ORGANIZATION |  | 5 | -0.83 | -1.66 | 0.009 | 0.322 | 1.000 | 918 | tags=40%, list=8%, signal=43% |
| 127 | GOMF\_FRIZZLED\_BINDING |  | 20 | -0.56 | -1.66 | 0.021 | 0.319 | 1.000 | 2226 | tags=40%, list=19%, signal=49% |
| 128 | GOBP\_SPERMINE\_METABOLIC\_PROCESS |  | 4 | -0.89 | -1.66 | 0.008 | 0.326 | 1.000 | 552 | tags=50%, list=5%, signal=52% |
| 129 | GOBP\_MICTURITION |  | 4 | -0.90 | -1.66 | 0.004 | 0.327 | 1.000 | 1245 | tags=100%, list=10%, signal=112% |
| 130 | GOBP\_LOCOMOTORY\_BEHAVIOR |  | 97 | -0.40 | -1.65 | 0.001 | 0.337 | 1.000 | 1289 | tags=25%, list=11%, signal=27% |
| 131 | GOBP\_OUTER\_DYNEIN\_ARM\_ASSEMBLY |  | 8 | -0.72 | -1.65 | 0.013 | 0.336 | 1.000 | 279 | tags=25%, list=2%, signal=26% |
| 132 | GOCC\_MITOCHONDRIAL\_MATRIX |  | 364 | -0.33 | -1.65 | 0.000 | 0.337 | 1.000 | 3613 | tags=40%, list=30%, signal=56% |
| 133 | GOMF\_OXIDOREDUCTASE\_ACTIVITY |  | 493 | -0.32 | -1.65 | 0.000 | 0.335 | 1.000 | 2005 | tags=27%, list=17%, signal=31% |
| 134 | GOBP\_GLYCOSIDE\_METABOLIC\_PROCESS |  | 12 | -0.64 | -1.65 | 0.020 | 0.335 | 1.000 | 2344 | tags=50%, list=20%, signal=62% |
| 135 | GOBP\_REGULATION\_OF\_CELLULAR\_PH\_REDUCTION |  | 6 | -0.78 | -1.65 | 0.014 | 0.344 | 1.000 | 1403 | tags=67%, list=12%, signal=75% |
| 136 | GOCC\_SIN3\_COMPLEX |  | 10 | -0.68 | -1.65 | 0.026 | 0.342 | 1.000 | 2923 | tags=50%, list=24%, signal=66% |
| 137 | GOBP\_NCRNA\_3\_END\_PROCESSING |  | 31 | -0.50 | -1.65 | 0.011 | 0.341 | 1.000 | 3825 | tags=48%, list=32%, signal=71% |
| 138 | GOBP\_OXALATE\_TRANSPORT |  | 6 | -0.79 | -1.64 | 0.015 | 0.342 | 1.000 | 78 | tags=50%, list=1%, signal=50% |
| 139 | GOCC\_NEURONAL\_DENSE\_CORE\_VESICLE |  | 11 | -0.65 | -1.64 | 0.019 | 0.347 | 1.000 | 2628 | tags=55%, list=22%, signal=70% |
| 140 | GOMF\_OXALATE\_TRANSMEMBRANE\_TRANSPORTER\_ACTIVITY |  | 6 | -0.79 | -1.64 | 0.015 | 0.355 | 1.000 | 78 | tags=50%, list=1%, signal=50% |
| 141 | GOBP\_BRANCHED\_CHAIN\_AMINO\_ACID\_METABOLIC\_PROCESS |  | 20 | -0.56 | -1.64 | 0.022 | 0.361 | 1.000 | 3225 | tags=60%, list=27%, signal=82% |
| 142 | GOBP\_NEGATIVE\_REGULATION\_OF\_OXIDATIVE\_STRESS\_INDUCED\_INTRINSIC\_APOPTOTIC\_SIGNALING\_PATHWAY |  | 15 | -0.60 | -1.64 | 0.023 | 0.359 | 1.000 | 2505 | tags=47%, list=21%, signal=59% |
| 143 | GOMF\_HEME\_COPPER\_TERMINAL\_OXIDASE\_ACTIVITY |  | 13 | -0.63 | -1.64 | 0.022 | 0.358 | 1.000 | 3121 | tags=62%, list=26%, signal=83% |
| 144 | GOBP\_SYNAPTIC\_TRANSMISSION\_GABAERGIC |  | 21 | -0.54 | -1.64 | 0.021 | 0.358 | 1.000 | 1514 | tags=33%, list=13%, signal=38% |
| 145 | GOBP\_NEGATIVE\_REGULATION\_OF\_UBIQUITIN\_DEPENDENT\_PROTEIN\_CATABOLIC\_PROCESS |  | 37 | -0.48 | -1.63 | 0.010 | 0.357 | 1.000 | 2656 | tags=38%, list=22%, signal=48% |
| 146 | GOBP\_APOPTOTIC\_PROCESS\_INVOLVED\_IN\_MORPHOGENESIS |  | 13 | -0.62 | -1.63 | 0.024 | 0.357 | 1.000 | 1918 | tags=46%, list=16%, signal=55% |
| 147 | GOBP\_NEGATIVE\_REGULATION\_OF\_CYCLIC\_NUCLEOTIDE\_PHOSPHODIESTERASE\_ACTIVITY |  | 4 | -0.88 | -1.63 | 0.008 | 0.354 | 1.000 | 1303 | tags=75%, list=11%, signal=84% |
| 148 | GOBP\_RIBONUCLEOSIDE\_TRIPHOSPHATE\_BIOSYNTHETIC\_PROCESS |  | 34 | -0.48 | -1.63 | 0.009 | 0.352 | 1.000 | 2669 | tags=44%, list=22%, signal=57% |
| 149 | GOBP\_NCRNA\_METABOLIC\_PROCESS |  | 353 | -0.33 | -1.63 | 0.000 | 0.354 | 1.000 | 3940 | tags=46%, list=33%, signal=66% |
| 150 | GOCC\_AXONEMAL\_DYNEIN\_COMPLEX |  | 5 | -0.82 | -1.63 | 0.014 | 0.352 | 1.000 | 21 | tags=20%, list=0%, signal=20% |
| 151 | GOMF\_NAD\_BINDING |  | 40 | -0.47 | -1.63 | 0.010 | 0.351 | 1.000 | 2909 | tags=45%, list=24%, signal=59% |
| 152 | GOBP\_ATRIAL\_CARDIAC\_MUSCLE\_CELL\_TO\_AV\_NODE\_CELL\_SIGNALING |  | 13 | -0.62 | -1.63 | 0.024 | 0.366 | 1.000 | 1424 | tags=31%, list=12%, signal=35% |
| 153 | GOCC\_CLATHRIN\_SCULPTED\_VESICLE |  | 7 | -0.75 | -1.63 | 0.023 | 0.365 | 1.000 | 1257 | tags=71%, list=10%, signal=80% |
| 154 | GOBP\_ETHANOL\_METABOLIC\_PROCESS |  | 8 | -0.71 | -1.63 | 0.018 | 0.365 | 1.000 | 244 | tags=50%, list=2%, signal=51% |
| 155 | GOCC\_TRICARBOXYLIC\_ACID\_CYCLE\_ENZYME\_COMPLEX |  | 13 | -0.62 | -1.63 | 0.026 | 0.364 | 1.000 | 3613 | tags=77%, list=30%, signal=110% |
| 156 | GOBP\_ANTERIOR\_POSTERIOR\_PATTERN\_SPECIFICATION |  | 121 | -0.38 | -1.62 | 0.001 | 0.364 | 1.000 | 1121 | tags=26%, list=9%, signal=28% |
| 157 | GOBP\_NEGATIVE\_REGULATION\_OF\_TISSUE\_REMODELING |  | 13 | -0.61 | -1.62 | 0.026 | 0.363 | 1.000 | 2046 | tags=69%, list=17%, signal=83% |
| 158 | GOMF\_TRANSLATION\_ELONGATION\_FACTOR\_ACTIVITY |  | 14 | -0.60 | -1.62 | 0.024 | 0.363 | 1.000 | 1552 | tags=43%, list=13%, signal=49% |
| 159 | GOMF\_OXIDOREDUCTASE\_ACTIVITY\_ACTING\_ON\_CH\_OH\_GROUP\_OF\_DONORS |  | 82 | -0.40 | -1.62 | 0.004 | 0.366 | 1.000 | 1902 | tags=33%, list=16%, signal=39% |
| 160 | GOBP\_POSITIVE\_REGULATION\_OF\_OLIGODENDROCYTE\_DIFFERENTIATION |  | 18 | -0.56 | -1.62 | 0.021 | 0.364 | 1.000 | 2881 | tags=61%, list=24%, signal=80% |
| 161 | GOBP\_PROTEIN\_IMPORT\_INTO\_MITOCHONDRIAL\_MATRIX |  | 15 | -0.60 | -1.62 | 0.023 | 0.363 | 1.000 | 3284 | tags=67%, list=27%, signal=92% |
| 162 | GOBP\_PANTOTHENATE\_METABOLIC\_PROCESS |  | 5 | -0.82 | -1.62 | 0.010 | 0.362 | 1.000 | 479 | tags=40%, list=4%, signal=42% |
| 163 | GOMF\_C\_X\_C\_CHEMOKINE\_RECEPTOR\_ACTIVITY |  | 4 | -0.88 | -1.62 | 0.013 | 0.377 | 1.000 | 1472 | tags=100%, list=12%, signal=114% |
| 164 | GOBP\_ANDROGEN\_CATABOLIC\_PROCESS |  | 3 | -0.96 | -1.62 | 0.003 | 0.380 | 1.000 | 471 | tags=100%, list=4%, signal=104% |
| 165 | GOBP\_UTP\_BIOSYNTHETIC\_PROCESS |  | 6 | -0.77 | -1.61 | 0.020 | 0.378 | 1.000 | 1626 | tags=67%, list=14%, signal=77% |
| 166 | GOBP\_DECIDUALIZATION |  | 11 | -0.64 | -1.61 | 0.026 | 0.380 | 1.000 | 1424 | tags=45%, list=12%, signal=52% |
| 167 | GOBP\_EMBRYONIC\_SKELETAL\_SYSTEM\_DEVELOPMENT |  | 69 | -0.41 | -1.61 | 0.003 | 0.383 | 1.000 | 780 | tags=26%, list=6%, signal=28% |
| 168 | GOCC\_TIM23\_MITOCHONDRIAL\_IMPORT\_INNER\_MEMBRANE\_TRANSLOCASE\_COMPLEX |  | 11 | -0.64 | -1.61 | 0.030 | 0.381 | 1.000 | 3891 | tags=82%, list=32%, signal=121% |
| 169 | GOBP\_QUINONE\_METABOLIC\_PROCESS |  | 25 | -0.51 | -1.61 | 0.021 | 0.386 | 1.000 | 3068 | tags=48%, list=26%, signal=64% |
| 170 | GOMF\_CATALYTIC\_ACTIVITY\_ACTING\_ON\_A\_TRNA |  | 85 | -0.40 | -1.61 | 0.002 | 0.390 | 1.000 | 3334 | tags=44%, list=28%, signal=60% |
| 171 | GOBP\_GLUTATHIONE\_DERIVATIVE\_METABOLIC\_PROCESS |  | 14 | -0.59 | -1.61 | 0.030 | 0.397 | 1.000 | 2887 | tags=64%, list=24%, signal=85% |
| 172 | GOBP\_CHEMOKINE\_C\_C\_MOTIF\_LIGAND\_5\_PRODUCTION |  | 6 | -0.77 | -1.60 | 0.024 | 0.405 | 1.000 | 1038 | tags=50%, list=9%, signal=55% |
| 173 | GOBP\_REGULATION\_OF\_WNT\_SIGNALING\_PATHWAY\_PLANAR\_CELL\_POLARITY\_PATHWAY |  | 11 | -0.64 | -1.60 | 0.028 | 0.411 | 1.000 | 2230 | tags=55%, list=19%, signal=67% |
| 174 | GOCC\_OUTER\_DYNEIN\_ARM |  | 4 | -0.85 | -1.60 | 0.010 | 0.414 | 1.000 | 21 | tags=25%, list=0%, signal=25% |
| 175 | GOBP\_RNA\_CATABOLIC\_PROCESS |  | 319 | -0.33 | -1.60 | 0.000 | 0.412 | 1.000 | 2352 | tags=29%, list=20%, signal=35% |
| 176 | GOMF\_NADP\_RETINOL\_DEHYDROGENASE\_ACTIVITY |  | 7 | -0.73 | -1.60 | 0.033 | 0.419 | 1.000 | 967 | tags=43%, list=8%, signal=47% |
| 177 | GOBP\_3\_PHOSPHOADENOSINE\_5\_PHOSPHOSULFATE\_BIOSYNTHETIC\_PROCESS |  | 5 | -0.81 | -1.60 | 0.021 | 0.420 | 1.000 | 2 | tags=20%, list=0%, signal=20% |
| 178 | GOBP\_VITAMIN\_CATABOLIC\_PROCESS |  | 6 | -0.77 | -1.60 | 0.021 | 0.418 | 1.000 | 349 | tags=50%, list=3%, signal=51% |
| 179 | GOBP\_PLANAR\_CELL\_POLARITY\_PATHWAY\_INVOLVED\_IN\_NEURAL\_TUBE\_CLOSURE |  | 8 | -0.70 | -1.60 | 0.024 | 0.416 | 1.000 | 677 | tags=38%, list=6%, signal=40% |
| 180 | GOBP\_SMALL\_MOLECULE\_CATABOLIC\_PROCESS |  | 312 | -0.33 | -1.59 | 0.000 | 0.420 | 1.000 | 2025 | tags=29%, list=17%, signal=34% |
| 181 | GOBP\_SULFUR\_AMINO\_ACID\_METABOLIC\_PROCESS |  | 28 | -0.50 | -1.59 | 0.027 | 0.419 | 1.000 | 821 | tags=25%, list=7%, signal=27% |
| 182 | GOBP\_NOREPINEPHRINE\_METABOLIC\_PROCESS |  | 6 | -0.77 | -1.59 | 0.026 | 0.417 | 1.000 | 1044 | tags=67%, list=9%, signal=73% |
| 183 | GOMF\_CHLORIDE\_CHANNEL\_INHIBITOR\_ACTIVITY |  | 7 | -0.73 | -1.59 | 0.031 | 0.416 | 1.000 | 411 | tags=29%, list=3%, signal=30% |
| 184 | GOMF\_STEROID\_DEHYDROGENASE\_ACTIVITY |  | 16 | -0.57 | -1.59 | 0.028 | 0.415 | 1.000 | 1455 | tags=56%, list=12%, signal=64% |
| 185 | GOBP\_MAMMARY\_GLAND\_EPITHELIAL\_CELL\_PROLIFERATION |  | 23 | -0.52 | -1.59 | 0.021 | 0.414 | 1.000 | 2611 | tags=43%, list=22%, signal=55% |
| 186 | GOBP\_PHAGOCYTOSIS\_RECOGNITION |  | 17 | -0.56 | -1.59 | 0.028 | 0.419 | 1.000 | 2123 | tags=47%, list=18%, signal=57% |
| 187 | GOBP\_GLYCOSIDE\_CATABOLIC\_PROCESS |  | 7 | -0.73 | -1.59 | 0.026 | 0.420 | 1.000 | 1865 | tags=57%, list=16%, signal=68% |
| 188 | GOMF\_O\_ACETYLTRANSFERASE\_ACTIVITY |  | 6 | -0.77 | -1.59 | 0.024 | 0.418 | 1.000 | 1566 | tags=50%, list=13%, signal=57% |
| 189 | GOMF\_CARBONYL\_REDUCTASE\_NADPH\_ACTIVITY |  | 3 | -0.94 | -1.59 | 0.006 | 0.419 | 1.000 | 409 | tags=67%, list=3%, signal=69% |
| 190 | GOCC\_SIN3\_TYPE\_COMPLEX |  | 12 | -0.62 | -1.59 | 0.028 | 0.416 | 1.000 | 3423 | tags=50%, list=28%, signal=70% |
| 191 | GOBP\_OXIDATIVE\_PHOSPHORYLATION |  | 85 | -0.39 | -1.59 | 0.004 | 0.416 | 1.000 | 4262 | tags=51%, list=35%, signal=78% |
| 192 | GOBP\_PEROXISOME\_ORGANIZATION |  | 71 | -0.40 | -1.59 | 0.011 | 0.415 | 1.000 | 1896 | tags=30%, list=16%, signal=35% |
| 193 | GOBP\_ORGANIC\_HYDROXY\_COMPOUND\_CATABOLIC\_PROCESS |  | 49 | -0.43 | -1.59 | 0.008 | 0.414 | 1.000 | 2359 | tags=37%, list=20%, signal=46% |
| 194 | GOBP\_REGULATION\_OF\_SHORT\_TERM\_NEURONAL\_SYNAPTIC\_PLASTICITY |  | 5 | -0.80 | -1.59 | 0.026 | 0.416 | 1.000 | 1257 | tags=60%, list=10%, signal=67% |
| 195 | GOBP\_NEGATIVE\_REGULATION\_OF\_MESENCHYMAL\_CELL\_APOPTOTIC\_PROCESS |  | 4 | -0.86 | -1.59 | 0.014 | 0.417 | 1.000 | 1656 | tags=75%, list=14%, signal=87% |
| 196 | GOBP\_AMIDE\_BIOSYNTHETIC\_PROCESS |  | 603 | -0.30 | -1.58 | 0.000 | 0.421 | 1.000 | 2421 | tags=29%, list=20%, signal=35% |
| 197 | GOMF\_IRON\_ION\_TRANSMEMBRANE\_TRANSPORTER\_ACTIVITY |  | 10 | -0.65 | -1.58 | 0.034 | 0.422 | 1.000 | 2527 | tags=60%, list=21%, signal=76% |
| 198 | GOBP\_OPSONIZATION |  | 8 | -0.70 | -1.58 | 0.032 | 0.420 | 1.000 | 2812 | tags=75%, list=23%, signal=98% |
| 199 | GOBP\_HYPOTHALAMUS\_DEVELOPMENT |  | 11 | -0.63 | -1.58 | 0.037 | 0.419 | 1.000 | 640 | tags=27%, list=5%, signal=29% |
| 200 | GOBP\_RNA\_MODIFICATION |  | 120 | -0.37 | -1.58 | 0.001 | 0.417 | 1.000 | 3180 | tags=43%, list=26%, signal=57% |
| 201 | GOBP\_ESTABLISHMENT\_OF\_PLANAR\_POLARITY\_INVOLVED\_IN\_NEURAL\_TUBE\_CLOSURE |  | 9 | -0.66 | -1.58 | 0.036 | 0.417 | 1.000 | 677 | tags=33%, list=6%, signal=35% |
| 202 | GOBP\_NEGATIVE\_REGULATION\_OF\_EXCITATORY\_POSTSYNAPTIC\_POTENTIAL |  | 7 | -0.72 | -1.58 | 0.032 | 0.420 | 1.000 | 1338 | tags=43%, list=11%, signal=48% |
| 203 | GOMF\_INOSITOL\_1\_3\_4\_5\_TETRAKISPHOSPHATE\_BINDING |  | 3 | -0.94 | -1.58 | 0.006 | 0.422 | 1.000 | 4 | tags=33%, list=0%, signal=33% |
| 204 | GOBP\_CHOLESTEROL\_CATABOLIC\_PROCESS |  | 8 | -0.70 | -1.58 | 0.034 | 0.423 | 1.000 | 1470 | tags=50%, list=12%, signal=57% |
| 205 | GOBP\_POSITIVE\_REGULATION\_OF\_INSULIN\_LIKE\_GROWTH\_FACTOR\_RECEPTOR\_SIGNALING\_PATHWAY |  | 9 | -0.66 | -1.58 | 0.035 | 0.424 | 1.000 | 3701 | tags=78%, list=31%, signal=112% |
| 206 | GOMF\_PHOSPHATE\_ION\_BINDING |  | 4 | -0.86 | -1.58 | 0.014 | 0.428 | 1.000 | 1643 | tags=75%, list=14%, signal=87% |
| 207 | GOBP\_RESPONSE\_TO\_EPINEPHRINE |  | 13 | -0.60 | -1.58 | 0.038 | 0.427 | 1.000 | 878 | tags=46%, list=7%, signal=50% |
| 208 | GOMF\_ATP\_DEPENDENT\_PROTEIN\_BINDING |  | 5 | -0.80 | -1.58 | 0.023 | 0.427 | 1.000 | 23 | tags=20%, list=0%, signal=20% |
| 209 | GOBP\_EXTRACELLULAR\_TRANSPORT |  | 20 | -0.53 | -1.57 | 0.027 | 0.433 | 1.000 | 664 | tags=25%, list=6%, signal=26% |
| 210 | GOBP\_TRNA\_METABOLIC\_PROCESS |  | 125 | -0.36 | -1.57 | 0.001 | 0.439 | 1.000 | 3120 | tags=41%, list=26%, signal=55% |
| 211 | GOBP\_ENDONUCLEOLYTIC\_CLEAVAGE\_INVOLVED\_IN\_RRNA\_PROCESSING |  | 12 | -0.61 | -1.57 | 0.031 | 0.437 | 1.000 | 4068 | tags=75%, list=34%, signal=113% |
| 212 | GOBP\_TRNA\_PROCESSING |  | 95 | -0.38 | -1.57 | 0.004 | 0.437 | 1.000 | 3120 | tags=42%, list=26%, signal=56% |
| 213 | GOMF\_INTRONIC\_TRANSCRIPTION\_REGULATORY\_REGION\_SEQUENCE\_SPECIFIC\_DNA\_BINDING |  | 4 | -0.85 | -1.57 | 0.022 | 0.444 | 1.000 | 954 | tags=75%, list=8%, signal=81% |
| 214 | GOBP\_REGULATION\_OF\_SOMATIC\_STEM\_CELL\_POPULATION\_MAINTENANCE |  | 4 | -0.85 | -1.57 | 0.018 | 0.450 | 1.000 | 502 | tags=50%, list=4%, signal=52% |
| 215 | GOCC\_PRERIBOSOME |  | 64 | -0.41 | -1.57 | 0.011 | 0.450 | 1.000 | 3900 | tags=53%, list=32%, signal=78% |
| 216 | GOBP\_REGULATION\_OF\_DENDRITE\_EXTENSION |  | 18 | -0.54 | -1.56 | 0.030 | 0.452 | 1.000 | 2662 | tags=56%, list=22%, signal=71% |
| 217 | GOCC\_ACETYLCHOLINE\_GATED\_CHANNEL\_COMPLEX |  | 6 | -0.74 | -1.56 | 0.033 | 0.451 | 1.000 | 2156 | tags=67%, list=18%, signal=81% |
| 218 | GOBP\_POSITIVE\_REGULATION\_OF\_VASCULAR\_ASSOCIATED\_SMOOTH\_MUSCLE\_CELL\_DIFFERENTIATION |  | 5 | -0.79 | -1.56 | 0.031 | 0.455 | 1.000 | 133 | tags=20%, list=1%, signal=20% |
| 219 | GOBP\_AXONEME\_ASSEMBLY |  | 29 | -0.48 | -1.56 | 0.020 | 0.457 | 1.000 | 2863 | tags=48%, list=24%, signal=63% |
| 220 | GOBP\_REGULATION\_OF\_CYCLIC\_NUCLEOTIDE\_PHOSPHODIESTERASE\_ACTIVITY |  | 7 | -0.71 | -1.56 | 0.035 | 0.456 | 1.000 | 1303 | tags=57%, list=11%, signal=64% |
| 221 | GOBP\_RESPONSE\_TO\_CATECHOLAMINE |  | 59 | -0.41 | -1.56 | 0.016 | 0.457 | 1.000 | 2421 | tags=37%, list=20%, signal=46% |
| 222 | GOCC\_PRERIBOSOME\_LARGE\_SUBUNIT\_PRECURSOR |  | 21 | -0.52 | -1.56 | 0.037 | 0.459 | 1.000 | 2950 | tags=38%, list=25%, signal=50% |
| 223 | GOMF\_ALCOHOL\_DEHYDROGENASE\_NAD\_P\_PLUS\_ACTIVITY |  | 5 | -0.79 | -1.56 | 0.030 | 0.457 | 1.000 | 2370 | tags=80%, list=20%, signal=100% |
| 224 | GOBP\_NEGATIVE\_REGULATION\_OF\_MONOCYTE\_CHEMOTAXIS |  | 6 | -0.74 | -1.56 | 0.034 | 0.456 | 1.000 | 2042 | tags=67%, list=17%, signal=80% |
| 225 | GOBP\_HOMOCYSTEINE\_METABOLIC\_PROCESS |  | 10 | -0.64 | -1.56 | 0.039 | 0.455 | 1.000 | 779 | tags=40%, list=6%, signal=43% |
| 226 | GOBP\_INORGANIC\_ANION\_TRANSPORT |  | 99 | -0.37 | -1.56 | 0.005 | 0.455 | 1.000 | 644 | tags=15%, list=5%, signal=16% |
| 227 | GOBP\_HISTONE\_H3\_ACETYLATION |  | 48 | -0.43 | -1.56 | 0.014 | 0.457 | 1.000 | 2214 | tags=35%, list=18%, signal=43% |
| 228 | GOCC\_AZUROPHIL\_GRANULE\_MEMBRANE |  | 44 | -0.43 | -1.56 | 0.017 | 0.458 | 1.000 | 2656 | tags=36%, list=22%, signal=47% |
| 229 | GOBP\_REGULATION\_OF\_SYNAPTIC\_VESICLE\_FUSION\_TO\_PRESYNAPTIC\_ACTIVE\_ZONE\_MEMBRANE |  | 6 | -0.74 | -1.55 | 0.038 | 0.460 | 1.000 | 1353 | tags=67%, list=11%, signal=75% |
| 230 | GOMF\_PHOSPHATE\_ION\_TRANSMEMBRANE\_TRANSPORTER\_ACTIVITY |  | 6 | -0.75 | -1.55 | 0.035 | 0.463 | 1.000 | 644 | tags=50%, list=5%, signal=53% |
| 231 | GOBP\_MITOCHONDRIAL\_RNA\_MODIFICATION |  | 8 | -0.68 | -1.55 | 0.039 | 0.462 | 1.000 | 1978 | tags=63%, list=16%, signal=75% |
| 232 | GOBP\_POSITIVE\_REGULATION\_OF\_FAT\_CELL\_DIFFERENTIATION |  | 46 | -0.43 | -1.55 | 0.015 | 0.464 | 1.000 | 1209 | tags=22%, list=10%, signal=24% |
| 233 | GOMF\_RNA\_POLYMERASE\_II\_INTRONIC\_TRANSCRIPTION\_REGULATORY\_REGION\_SEQUENCE\_SPECIFIC\_DNA\_BINDING |  | 3 | -0.92 | -1.55 | 0.010 | 0.471 | 1.000 | 954 | tags=100%, list=8%, signal=109% |
| 234 | GOCC\_TRANSCRIPTION\_EXPORT\_COMPLEX\_2 |  | 4 | -0.84 | -1.55 | 0.023 | 0.470 | 1.000 | 88 | tags=25%, list=1%, signal=25% |
| 235 | GOBP\_REGULATION\_OF\_SYSTEMIC\_ARTERIAL\_BLOOD\_PRESSURE |  | 55 | -0.41 | -1.55 | 0.014 | 0.469 | 1.000 | 2170 | tags=36%, list=18%, signal=44% |
| 236 | GOBP\_REGULATION\_OF\_TRANSCRIPTION\_FROM\_RNA\_POLYMERASE\_II\_PROMOTER\_IN\_RESPONSE\_TO\_OXIDATIVE\_STRESS |  | 9 | -0.66 | -1.55 | 0.047 | 0.477 | 1.000 | 1418 | tags=44%, list=12%, signal=50% |
| 237 | GOBP\_PYRIMIDINE\_RIBONUCLEOSIDE\_TRIPHOSPHATE\_METABOLIC\_PROCESS |  | 14 | -0.57 | -1.55 | 0.046 | 0.475 | 1.000 | 2669 | tags=57%, list=22%, signal=73% |
| 238 | GOCC\_MICROBODY\_LUMEN |  | 44 | -0.43 | -1.55 | 0.017 | 0.473 | 1.000 | 1719 | tags=34%, list=14%, signal=40% |
| 239 | GOBP\_NEGATIVE\_REGULATION\_OF\_CARDIAC\_MUSCLE\_CELL\_PROLIFERATION |  | 9 | -0.66 | -1.55 | 0.042 | 0.476 | 1.000 | 2722 | tags=56%, list=23%, signal=72% |
| 240 | GOMF\_POSTSYNAPTIC\_NEUROTRANSMITTER\_RECEPTOR\_ACTIVITY |  | 17 | -0.54 | -1.55 | 0.041 | 0.474 | 1.000 | 2156 | tags=47%, list=18%, signal=57% |
| 241 | GOBP\_ANDROGEN\_METABOLIC\_PROCESS |  | 20 | -0.52 | -1.54 | 0.041 | 0.475 | 1.000 | 1656 | tags=50%, list=14%, signal=58% |
| 242 | GOBP\_MATURATION\_OF\_LSU\_RRNA\_FROM\_TRICISTRONIC\_RRNA\_TRANSCRIPT\_SSU\_RRNA\_5\_8S\_RRNA\_LSU\_RRNA |  | 15 | -0.56 | -1.54 | 0.041 | 0.475 | 1.000 | 1261 | tags=33%, list=10%, signal=37% |
| 243 | GOBP\_NEURON\_RECOGNITION |  | 33 | -0.46 | -1.54 | 0.024 | 0.476 | 1.000 | 970 | tags=27%, list=8%, signal=30% |
| 244 | GOMF\_MORPHOGEN\_ACTIVITY |  | 4 | -0.83 | -1.54 | 0.025 | 0.475 | 1.000 | 2042 | tags=100%, list=17%, signal=120% |
| 245 | GOBP\_REGULATION\_OF\_GAMMA\_DELTA\_T\_CELL\_ACTIVATION |  | 8 | -0.68 | -1.54 | 0.047 | 0.474 | 1.000 | 1903 | tags=63%, list=16%, signal=74% |
| 246 | GOMF\_LARGE\_RIBOSOMAL\_SUBUNIT\_RRNA\_BINDING |  | 3 | -0.91 | -1.54 | 0.010 | 0.473 | 1.000 | 1031 | tags=100%, list=9%, signal=109% |
| 247 | GOBP\_REGULATION\_OF\_SYSTEMIC\_ARTERIAL\_BLOOD\_PRESSURE\_BY\_VASOPRESSIN |  | 4 | -0.83 | -1.54 | 0.027 | 0.478 | 1.000 | 1514 | tags=75%, list=13%, signal=86% |
| 248 | GOBP\_MAGNESIUM\_ION\_TRANSPORT |  | 13 | -0.59 | -1.54 | 0.043 | 0.476 | 1.000 | 3009 | tags=62%, list=25%, signal=82% |
| 249 | GOMF\_CATALYTIC\_ACTIVITY\_ACTING\_ON\_A\_RRNA |  | 16 | -0.55 | -1.54 | 0.044 | 0.475 | 1.000 | 2754 | tags=44%, list=23%, signal=57% |
| 250 | GOBP\_BRAIN\_DERIVED\_NEUROTROPHIC\_FACTOR\_RECEPTOR\_SIGNALING\_PATHWAY |  | 3 | -0.91 | -1.54 | 0.015 | 0.474 | 1.000 | 15 | tags=33%, list=0%, signal=33% |
| 251 | GOBP\_REGULATION\_OF\_GAMMA\_DELTA\_T\_CELL\_DIFFERENTIATION |  | 8 | -0.68 | -1.54 | 0.041 | 0.474 | 1.000 | 1903 | tags=63%, list=16%, signal=74% |
| 252 | GOBP\_NEGATIVE\_REGULATION\_OF\_OXIDATIVE\_STRESS\_INDUCED\_NEURON\_DEATH |  | 9 | -0.65 | -1.54 | 0.046 | 0.473 | 1.000 | 2505 | tags=56%, list=21%, signal=70% |
| 253 | GOBP\_PROTEIN\_SULFATION |  | 5 | -0.78 | -1.54 | 0.031 | 0.473 | 1.000 | 2650 | tags=100%, list=22%, signal=128% |
| 254 | GOBP\_TRANSLATIONAL\_TERMINATION |  | 88 | -0.37 | -1.54 | 0.010 | 0.473 | 1.000 | 4660 | tags=52%, list=39%, signal=85% |
| 255 | GOBP\_SERTOLI\_CELL\_DEVELOPMENT |  | 7 | -0.71 | -1.54 | 0.043 | 0.476 | 1.000 | 1818 | tags=43%, list=15%, signal=50% |
| 256 | GOBP\_PEROXISOMAL\_TRANSPORT |  | 63 | -0.40 | -1.54 | 0.012 | 0.476 | 1.000 | 1566 | tags=27%, list=13%, signal=31% |
| 257 | GOMF\_RETINOIC\_ACID\_4\_HYDROXYLASE\_ACTIVITY |  | 4 | -0.83 | -1.54 | 0.028 | 0.474 | 1.000 | 112 | tags=50%, list=1%, signal=50% |
| 258 | GOBP\_ALDEHYDE\_CATABOLIC\_PROCESS |  | 8 | -0.68 | -1.53 | 0.043 | 0.484 | 1.000 | 2885 | tags=75%, list=24%, signal=99% |
| 259 | GOBP\_ELECTRON\_TRANSPORT\_CHAIN |  | 121 | -0.35 | -1.53 | 0.004 | 0.484 | 1.000 | 4262 | tags=54%, list=35%, signal=82% |
| 260 | GOMF\_PROTON\_TRANSMEMBRANE\_TRANSPORTER\_ACTIVITY |  | 72 | -0.39 | -1.53 | 0.009 | 0.493 | 1.000 | 3121 | tags=42%, list=26%, signal=56% |
| 261 | GOBP\_PERIPHERAL\_NERVOUS\_SYSTEM\_NEURON\_DIFFERENTIATION |  | 10 | -0.62 | -1.53 | 0.047 | 0.491 | 1.000 | 563 | tags=30%, list=5%, signal=31% |
| 262 | GOBP\_MITOCHONDRIAL\_RIBOSOME\_ASSEMBLY |  | 6 | -0.73 | -1.53 | 0.052 | 0.492 | 1.000 | 2029 | tags=67%, list=17%, signal=80% |
| 263 | GOBP\_NOREPINEPHRINE\_BIOSYNTHETIC\_PROCESS |  | 4 | -0.83 | -1.53 | 0.028 | 0.490 | 1.000 | 1044 | tags=75%, list=9%, signal=82% |
| 264 | GOBP\_POSITIVE\_REGULATION\_OF\_VASCULAR\_ENDOTHELIAL\_GROWTH\_FACTOR\_RECEPTOR\_SIGNALING\_PATHWAY |  | 9 | -0.65 | -1.53 | 0.052 | 0.488 | 1.000 | 1418 | tags=44%, list=12%, signal=50% |
| 265 | GOBP\_POSITIVE\_REGULATION\_OF\_RESPIRATORY\_BURST |  | 5 | -0.78 | -1.53 | 0.039 | 0.487 | 1.000 | 2209 | tags=80%, list=18%, signal=98% |
| 266 | GOBP\_CHLORIDE\_TRANSPORT |  | 57 | -0.40 | -1.53 | 0.010 | 0.485 | 1.000 | 254 | tags=16%, list=2%, signal=16% |
| 267 | GOMF\_1\_ACYLGLYCEROPHOSPHOCHOLINE\_O\_ACYLTRANSFERASE\_ACTIVITY |  | 7 | -0.69 | -1.53 | 0.046 | 0.488 | 1.000 | 108 | tags=29%, list=1%, signal=29% |
| 268 | GOBP\_SKELETAL\_MUSCLE\_THIN\_FILAMENT\_ASSEMBLY |  | 3 | -0.90 | -1.53 | 0.019 | 0.489 | 1.000 | 555 | tags=67%, list=5%, signal=70% |
| 269 | GOBP\_GLUTAMATE\_METABOLIC\_PROCESS |  | 22 | -0.50 | -1.53 | 0.041 | 0.489 | 1.000 | 2025 | tags=36%, list=17%, signal=44% |
| 270 | GOMF\_ACETYLCHOLINE\_GATED\_CATION\_SELECTIVE\_CHANNEL\_ACTIVITY |  | 5 | -0.77 | -1.53 | 0.046 | 0.489 | 1.000 | 2156 | tags=80%, list=18%, signal=97% |
| 271 | GOBP\_HISTONE\_H4\_ACETYLATION |  | 53 | -0.41 | -1.53 | 0.015 | 0.489 | 1.000 | 4508 | tags=57%, list=38%, signal=90% |
| 272 | GOCC\_VOLTAGE\_GATED\_SODIUM\_CHANNEL\_COMPLEX |  | 8 | -0.67 | -1.53 | 0.048 | 0.489 | 1.000 | 1316 | tags=50%, list=11%, signal=56% |
| 273 | GOBP\_INTESTINE\_SMOOTH\_MUSCLE\_CONTRACTION |  | 5 | -0.77 | -1.53 | 0.037 | 0.489 | 1.000 | 536 | tags=40%, list=4%, signal=42% |
| 274 | GOCC\_INNER\_MITOCHONDRIAL\_MEMBRANE\_PROTEIN\_COMPLEX |  | 93 | -0.37 | -1.52 | 0.007 | 0.489 | 1.000 | 4262 | tags=52%, list=35%, signal=79% |
| 275 | GOCC\_JUXTAPARANODE\_REGION\_OF\_AXON |  | 6 | -0.73 | -1.52 | 0.047 | 0.489 | 1.000 | 1385 | tags=67%, list=12%, signal=75% |
| 276 | GOBP\_PROTEIN\_LOCALIZATION\_TO\_CILIARY\_TRANSITION\_ZONE |  | 6 | -0.73 | -1.52 | 0.040 | 0.489 | 1.000 | 3257 | tags=100%, list=27%, signal=137% |
| 277 | GOBP\_MATURATION\_OF\_5\_8S\_RRNA |  | 30 | -0.47 | -1.52 | 0.030 | 0.488 | 1.000 | 2309 | tags=37%, list=19%, signal=45% |
| 278 | GOBP\_IRES\_DEPENDENT\_VIRAL\_TRANSLATIONAL\_INITIATION |  | 11 | -0.60 | -1.52 | 0.046 | 0.487 | 1.000 | 1427 | tags=36%, list=12%, signal=41% |
| 279 | GOBP\_NEGATIVE\_REGULATION\_OF\_MULTICELLULAR\_ORGANISM\_GROWTH |  | 7 | -0.70 | -1.52 | 0.045 | 0.487 | 1.000 | 3142 | tags=71%, list=26%, signal=97% |
| 280 | GOBP\_TRANSLATIONAL\_ELONGATION |  | 115 | -0.35 | -1.52 | 0.003 | 0.486 | 1.000 | 3615 | tags=39%, list=30%, signal=55% |
| 281 | GOBP\_AV\_NODE\_CELL\_TO\_BUNDLE\_OF\_HIS\_CELL\_COMMUNICATION |  | 7 | -0.70 | -1.52 | 0.053 | 0.484 | 1.000 | 2248 | tags=71%, list=19%, signal=88% |
| 282 | GOBP\_ETHANOL\_OXIDATION |  | 5 | -0.77 | -1.52 | 0.043 | 0.487 | 1.000 | 244 | tags=60%, list=2%, signal=61% |
| 283 | GOBP\_PH\_REDUCTION |  | 45 | -0.42 | -1.52 | 0.021 | 0.488 | 1.000 | 1852 | tags=31%, list=15%, signal=37% |
| 284 | GOBP\_NEURON\_FATE\_DETERMINATION |  | 3 | -0.91 | -1.52 | 0.015 | 0.487 | 1.000 | 669 | tags=67%, list=6%, signal=71% |
| 285 | GOBP\_NEGATIVE\_REGULATION\_OF\_UBIQUITIN\_PROTEIN\_LIGASE\_ACTIVITY |  | 9 | -0.64 | -1.52 | 0.059 | 0.487 | 1.000 | 1031 | tags=44%, list=9%, signal=49% |
| 286 | GOBP\_NEGATIVE\_REGULATION\_OF\_OXIDATIVE\_STRESS\_INDUCED\_CELL\_DEATH |  | 29 | -0.47 | -1.52 | 0.033 | 0.490 | 1.000 | 2505 | tags=41%, list=21%, signal=52% |
| 287 | GOMF\_OXIDOREDUCTASE\_ACTIVITY\_ACTING\_ON\_THE\_ALDEHYDE\_OR\_OXO\_GROUP\_OF\_DONORS |  | 31 | -0.46 | -1.52 | 0.030 | 0.490 | 1.000 | 1850 | tags=29%, list=15%, signal=34% |
| 288 | GOBP\_CELL\_RECOGNITION |  | 86 | -0.37 | -1.52 | 0.009 | 0.492 | 1.000 | 2132 | tags=31%, list=18%, signal=38% |
| 289 | GOBP\_SERTOLI\_CELL\_DIFFERENTIATION |  | 10 | -0.62 | -1.52 | 0.053 | 0.492 | 1.000 | 1935 | tags=40%, list=16%, signal=48% |
| 290 | GOMF\_LYASE\_ACTIVITY |  | 139 | -0.34 | -1.52 | 0.002 | 0.491 | 1.000 | 2083 | tags=28%, list=17%, signal=34% |
| 291 | GOBP\_PHENOL\_CONTAINING\_COMPOUND\_METABOLIC\_PROCESS |  | 56 | -0.40 | -1.52 | 0.018 | 0.494 | 1.000 | 2505 | tags=41%, list=21%, signal=52% |
| 292 | GOBP\_TRNA\_PSEUDOURIDINE\_SYNTHESIS |  | 5 | -0.76 | -1.52 | 0.044 | 0.493 | 1.000 | 2466 | tags=80%, list=21%, signal=101% |
| 293 | GOBP\_PROTON\_TRANSMEMBRANE\_TRANSPORT |  | 85 | -0.37 | -1.52 | 0.009 | 0.492 | 1.000 | 3121 | tags=39%, list=26%, signal=52% |
| 294 | GOBP\_NEGATIVE\_REGULATION\_OF\_STRIATED\_MUSCLE\_CELL\_APOPTOTIC\_PROCESS |  | 13 | -0.57 | -1.51 | 0.057 | 0.492 | 1.000 | 629 | tags=31%, list=5%, signal=32% |
| 295 | GOBP\_POSITIVE\_REGULATION\_OF\_TRANSCRIPTION\_FROM\_RNA\_POLYMERASE\_II\_PROMOTER\_IN\_RESPONSE\_TO\_OXIDATIVE\_STRESS |  | 4 | -0.82 | -1.51 | 0.035 | 0.492 | 1.000 | 850 | tags=75%, list=7%, signal=81% |
| 296 | GOBP\_ACUTE\_INFLAMMATORY\_RESPONSE |  | 53 | -0.41 | -1.51 | 0.021 | 0.492 | 1.000 | 1124 | tags=23%, list=9%, signal=25% |
| 297 | GOCC\_IMMUNOGLOBULIN\_COMPLEX |  | 4 | -0.82 | -1.51 | 0.037 | 0.505 | 1.000 | 2209 | tags=100%, list=18%, signal=122% |
| 298 | GOMF\_ANTIOXIDANT\_ACTIVITY |  | 52 | -0.41 | -1.51 | 0.024 | 0.505 | 1.000 | 1940 | tags=29%, list=16%, signal=34% |
| 299 | GOBP\_MEMBRANE\_REPOLARIZATION\_DURING\_ATRIAL\_CARDIAC\_MUSCLE\_CELL\_ACTION\_POTENTIAL |  | 4 | -0.82 | -1.51 | 0.036 | 0.504 | 1.000 | 13 | tags=25%, list=0%, signal=25% |
| 300 | GOBP\_TETRAPYRROLE\_METABOLIC\_PROCESS |  | 43 | -0.42 | -1.51 | 0.026 | 0.503 | 1.000 | 3294 | tags=49%, list=27%, signal=67% |
| 301 | GOBP\_POSITIVE\_REGULATION\_OF\_HORMONE\_METABOLIC\_PROCESS |  | 9 | -0.65 | -1.51 | 0.064 | 0.502 | 1.000 | 1418 | tags=44%, list=12%, signal=50% |
| 302 | GOBP\_ADENYLATE\_CYCLASE\_INHIBITING\_SEROTONIN\_RECEPTOR\_SIGNALING\_PATHWAY |  | 3 | -0.89 | -1.51 | 0.019 | 0.504 | 1.000 | 947 | tags=67%, list=8%, signal=72% |
| 303 | GOBP\_APOPTOTIC\_PROCESS\_INVOLVED\_IN\_DEVELOPMENT |  | 21 | -0.51 | -1.51 | 0.048 | 0.513 | 1.000 | 1918 | tags=38%, list=16%, signal=45% |
| 304 | GOMF\_3\_HYDROXYACYL\_COA\_DEHYDROGENASE\_ACTIVITY |  | 6 | -0.72 | -1.51 | 0.055 | 0.512 | 1.000 | 2475 | tags=67%, list=21%, signal=84% |
| 305 | GOBP\_NEGATIVE\_REGULATION\_OF\_OSSIFICATION |  | 21 | -0.50 | -1.50 | 0.048 | 0.512 | 1.000 | 2277 | tags=43%, list=19%, signal=53% |
| 306 | GOCC\_NURF\_COMPLEX |  | 3 | -0.89 | -1.50 | 0.027 | 0.510 | 1.000 | 314 | tags=33%, list=3%, signal=34% |
| 307 | GOBP\_RESPIRATORY\_BURST\_INVOLVED\_IN\_DEFENSE\_RESPONSE |  | 9 | -0.64 | -1.50 | 0.054 | 0.512 | 1.000 | 2123 | tags=56%, list=18%, signal=67% |
| 308 | GOCC\_CYTOCHROME\_COMPLEX |  | 18 | -0.52 | -1.50 | 0.051 | 0.511 | 1.000 | 4233 | tags=56%, list=35%, signal=86% |
| 309 | GOCC\_CYTOPLASMIC\_SIDE\_OF\_ROUGH\_ENDOPLASMIC\_RETICULUM\_MEMBRANE |  | 5 | -0.76 | -1.50 | 0.047 | 0.512 | 1.000 | 1679 | tags=80%, list=14%, signal=93% |
| 310 | GOBP\_URATE\_METABOLIC\_PROCESS |  | 4 | -0.82 | -1.50 | 0.040 | 0.516 | 1.000 | 1781 | tags=75%, list=15%, signal=88% |
| 311 | GOBP\_HYDROGEN\_SULFIDE\_METABOLIC\_PROCESS |  | 4 | -0.81 | -1.50 | 0.038 | 0.518 | 1.000 | 779 | tags=75%, list=6%, signal=80% |
| 312 | GOBP\_REGULATION\_OF\_GRANULOCYTE\_DIFFERENTIATION |  | 12 | -0.58 | -1.50 | 0.061 | 0.518 | 1.000 | 2760 | tags=58%, list=23%, signal=76% |
| 313 | GOBP\_SKELETAL\_SYSTEM\_MORPHOGENESIS |  | 132 | -0.34 | -1.50 | 0.006 | 0.516 | 1.000 | 1201 | tags=21%, list=10%, signal=23% |
| 314 | GOBP\_ENDONUCLEOLYTIC\_CLEAVAGE\_IN\_ITS1\_TO\_SEPARATE\_SSU\_RRNA\_FROM\_5\_8S\_RRNA\_AND\_LSU\_RRNA\_FROM\_TRICISTRONIC\_RRNA\_TRANSCRIPT\_SSU\_RRNA\_5\_8S\_RRNA\_LSU\_RRNA |  | 6 | -0.72 | -1.50 | 0.055 | 0.515 | 1.000 | 2309 | tags=67%, list=19%, signal=82% |
| 315 | GOBP\_ANDROGEN\_BIOSYNTHETIC\_PROCESS |  | 6 | -0.72 | -1.50 | 0.051 | 0.518 | 1.000 | 3301 | tags=83%, list=27%, signal=115% |
| 316 | GOBP\_NEGATIVE\_REGULATION\_OF\_PHOTORECEPTOR\_CELL\_DIFFERENTIATION |  | 4 | -0.81 | -1.50 | 0.041 | 0.517 | 1.000 | 141 | tags=25%, list=1%, signal=25% |
| 317 | GOMF\_TRANSFERASE\_ACTIVITY\_TRANSFERRING\_SULFUR\_CONTAINING\_GROUPS |  | 45 | -0.42 | -1.50 | 0.022 | 0.518 | 1.000 | 2014 | tags=40%, list=17%, signal=48% |
| 318 | GOMF\_CADMIUM\_ION\_BINDING |  | 3 | -0.89 | -1.50 | 0.022 | 0.519 | 1.000 | 77 | tags=33%, list=1%, signal=34% |
| 319 | GOMF\_HYDROLASE\_ACTIVITY\_ACTING\_ON\_CARBON\_NITROGEN\_BUT\_NOT\_PEPTIDE\_BONDS\_IN\_LINEAR\_AMIDES |  | 49 | -0.41 | -1.50 | 0.025 | 0.518 | 1.000 | 2783 | tags=41%, list=23%, signal=53% |
| 320 | GOBP\_ORGANIC\_CYCLIC\_COMPOUND\_CATABOLIC\_PROCESS |  | 461 | -0.29 | -1.50 | 0.000 | 0.518 | 1.000 | 2359 | tags=28%, list=20%, signal=34% |
| 321 | GOBP\_REGULATION\_OF\_ATPASE\_COUPLED\_CALCIUM\_TRANSMEMBRANE\_TRANSPORTER\_ACTIVITY |  | 5 | -0.76 | -1.50 | 0.044 | 0.518 | 1.000 | 448 | tags=60%, list=4%, signal=62% |
| 322 | GOBP\_REGULATION\_OF\_PHOTORECEPTOR\_CELL\_DIFFERENTIATION |  | 4 | -0.81 | -1.50 | 0.036 | 0.518 | 1.000 | 141 | tags=25%, list=1%, signal=25% |
| 323 | GOCC\_AZUROPHIL\_GRANULE |  | 103 | -0.35 | -1.50 | 0.010 | 0.516 | 1.000 | 3238 | tags=39%, list=27%, signal=53% |
| 324 | GOBP\_TACHYKININ\_RECEPTOR\_SIGNALING\_PATHWAY |  | 5 | -0.76 | -1.50 | 0.047 | 0.517 | 1.000 | 12 | tags=20%, list=0%, signal=20% |
| 325 | GOBP\_POSITIVE\_REGULATION\_OF\_SYNAPTIC\_TRANSMISSION\_GABAERGIC |  | 8 | -0.66 | -1.50 | 0.057 | 0.515 | 1.000 | 48 | tags=25%, list=0%, signal=25% |
| 326 | GOBP\_NEGATIVE\_T\_CELL\_SELECTION |  | 11 | -0.60 | -1.50 | 0.067 | 0.514 | 1.000 | 3133 | tags=64%, list=26%, signal=86% |
| 327 | GOBP\_PHOTOTRANSDUCTION |  | 22 | -0.49 | -1.49 | 0.047 | 0.516 | 1.000 | 1438 | tags=23%, list=12%, signal=26% |
| 328 | GOCC\_INTRINSIC\_COMPONENT\_OF\_POSTSYNAPTIC\_SPECIALIZATION\_MEMBRANE |  | 25 | -0.48 | -1.49 | 0.047 | 0.518 | 1.000 | 2479 | tags=44%, list=21%, signal=55% |
| 329 | GOBP\_REGULATION\_OF\_PROTEIN\_AUTOUBIQUITINATION |  | 4 | -0.81 | -1.49 | 0.045 | 0.518 | 1.000 | 1952 | tags=75%, list=16%, signal=90% |
| 330 | GOBP\_OXIDATIVE\_DNA\_DEMETHYLATION |  | 6 | -0.71 | -1.49 | 0.065 | 0.517 | 1.000 | 1616 | tags=67%, list=13%, signal=77% |
| 331 | GOBP\_ELASTIN\_METABOLIC\_PROCESS |  | 3 | -0.87 | -1.49 | 0.028 | 0.518 | 1.000 | 1418 | tags=67%, list=12%, signal=76% |
| 332 | GOBP\_LEUKOCYTE\_MIGRATION\_INVOLVED\_IN\_INFLAMMATORY\_RESPONSE |  | 13 | -0.57 | -1.49 | 0.052 | 0.518 | 1.000 | 3240 | tags=62%, list=27%, signal=84% |
| 333 | GOBP\_GAMMA\_DELTA\_T\_CELL\_DIFFERENTIATION |  | 9 | -0.63 | -1.49 | 0.066 | 0.520 | 1.000 | 1903 | tags=56%, list=16%, signal=66% |
| 334 | GOBP\_SALIVA\_SECRETION |  | 5 | -0.75 | -1.49 | 0.044 | 0.519 | 1.000 | 12 | tags=20%, list=0%, signal=20% |
| 335 | GOBP\_PHOSPHATE\_ION\_TRANSPORT |  | 19 | -0.51 | -1.49 | 0.052 | 0.520 | 1.000 | 3494 | tags=63%, list=29%, signal=89% |
| 336 | GOBP\_VITAMIN\_METABOLIC\_PROCESS |  | 87 | -0.36 | -1.49 | 0.009 | 0.518 | 1.000 | 1114 | tags=23%, list=9%, signal=25% |
| 337 | GOBP\_FATTY\_ACID\_CATABOLIC\_PROCESS |  | 86 | -0.37 | -1.49 | 0.015 | 0.519 | 1.000 | 1786 | tags=33%, list=15%, signal=38% |
| 338 | GOBP\_MESENCHYMAL\_CELL\_APOPTOTIC\_PROCESS |  | 6 | -0.72 | -1.49 | 0.049 | 0.517 | 1.000 | 1656 | tags=50%, list=14%, signal=58% |
| 339 | GOBP\_CONVERGENT\_EXTENSION |  | 11 | -0.60 | -1.49 | 0.062 | 0.517 | 1.000 | 1391 | tags=36%, list=12%, signal=41% |
| 340 | GOCC\_ANCHORED\_COMPONENT\_OF\_SYNAPTIC\_MEMBRANE |  | 9 | -0.63 | -1.49 | 0.066 | 0.517 | 1.000 | 2158 | tags=44%, list=18%, signal=54% |
| 341 | GOBP\_REGULATION\_OF\_ARACHIDONIC\_ACID\_SECRETION |  | 3 | -0.88 | -1.49 | 0.029 | 0.517 | 1.000 | 1481 | tags=100%, list=12%, signal=114% |
| 342 | GOMF\_ANDROSTAN\_3\_ALPHA\_17\_BETA\_DIOL\_DEHYDROGENASE\_ACTIVITY |  | 3 | -0.88 | -1.49 | 0.029 | 0.523 | 1.000 | 1455 | tags=100%, list=12%, signal=114% |
| 343 | GOBP\_REGULATION\_OF\_SKELETAL\_MUSCLE\_TISSUE\_REGENERATION |  | 3 | -0.88 | -1.49 | 0.035 | 0.522 | 1.000 | 1269 | tags=67%, list=11%, signal=75% |
| 344 | GOBP\_PATTERN\_SPECIFICATION\_PROCESS |  | 252 | -0.31 | -1.49 | 0.001 | 0.522 | 1.000 | 1512 | tags=24%, list=13%, signal=27% |
| 345 | GOMF\_TESTOSTERONE\_DEHYDROGENASE\_NAD\_P\_ACTIVITY |  | 3 | -0.88 | -1.49 | 0.035 | 0.521 | 1.000 | 1455 | tags=100%, list=12%, signal=114% |
| 346 | GOMF\_CUPROUS\_ION\_BINDING |  | 6 | -0.71 | -1.49 | 0.057 | 0.520 | 1.000 | 3495 | tags=100%, list=29%, signal=141% |
| 347 | GOBP\_REGULATION\_OF\_I\_KAPPAB\_PHOSPHORYLATION |  | 6 | -0.71 | -1.49 | 0.060 | 0.519 | 1.000 | 378 | tags=33%, list=3%, signal=34% |
| 348 | GOMF\_OXIDOREDUCTASE\_ACTIVITY\_ACTING\_ON\_PEROXIDE\_AS\_ACCEPTOR |  | 33 | -0.44 | -1.48 | 0.040 | 0.520 | 1.000 | 1940 | tags=30%, list=16%, signal=36% |
| 349 | GOBP\_REGULATION\_OF\_LYMPHOID\_PROGENITOR\_CELL\_DIFFERENTIATION |  | 8 | -0.65 | -1.48 | 0.060 | 0.520 | 1.000 | 3624 | tags=88%, list=30%, signal=125% |
| 350 | GOBP\_UREA\_CYCLE |  | 5 | -0.76 | -1.48 | 0.061 | 0.523 | 1.000 | 1587 | tags=60%, list=13%, signal=69% |
| 351 | GOBP\_HYDROGEN\_SULFIDE\_BIOSYNTHETIC\_PROCESS |  | 4 | -0.81 | -1.48 | 0.042 | 0.525 | 1.000 | 779 | tags=75%, list=6%, signal=80% |
| 352 | GOBP\_SNRNA\_PROCESSING |  | 22 | -0.48 | -1.48 | 0.048 | 0.525 | 1.000 | 3711 | tags=41%, list=31%, signal=59% |
| 353 | GOMF\_NEUROTRANSMITTER\_RECEPTOR\_ACTIVITY\_INVOLVED\_IN\_REGULATION\_OF\_POSTSYNAPTIC\_MEMBRANE\_POTENTIAL |  | 11 | -0.59 | -1.48 | 0.064 | 0.525 | 1.000 | 2156 | tags=45%, list=18%, signal=55% |
| 354 | GOBP\_POSITIVE\_REGULATION\_OF\_SMOOTHENED\_SIGNALING\_PATHWAY |  | 26 | -0.47 | -1.48 | 0.044 | 0.524 | 1.000 | 3365 | tags=46%, list=28%, signal=64% |
| 355 | GOCC\_MITOCHONDRIAL\_SMALL\_RIBOSOMAL\_SUBUNIT |  | 25 | -0.47 | -1.48 | 0.053 | 0.524 | 1.000 | 3592 | tags=40%, list=30%, signal=57% |
| 356 | GOBP\_SINGLE\_FERTILIZATION |  | 61 | -0.38 | -1.48 | 0.018 | 0.524 | 1.000 | 1353 | tags=21%, list=11%, signal=24% |
| 357 | GOBP\_CLEAVAGE\_INVOLVED\_IN\_RRNA\_PROCESSING |  | 24 | -0.48 | -1.48 | 0.049 | 0.526 | 1.000 | 4068 | tags=58%, list=34%, signal=88% |
| 358 | GOBP\_NEGATIVE\_REGULATION\_OF\_SYNAPSE\_ASSEMBLY |  | 5 | -0.76 | -1.48 | 0.059 | 0.529 | 1.000 | 2052 | tags=80%, list=17%, signal=96% |
| 359 | GOBP\_NEGATIVE\_REGULATION\_OF\_RESPIRATORY\_BURST\_INVOLVED\_IN\_INFLAMMATORY\_RESPONSE |  | 4 | -0.80 | -1.48 | 0.051 | 0.528 | 1.000 | 1172 | tags=50%, list=10%, signal=55% |
| 360 | GOBP\_OTOLITH\_DEVELOPMENT |  | 4 | -0.80 | -1.48 | 0.055 | 0.527 | 1.000 | 1700 | tags=50%, list=14%, signal=58% |
| 361 | GOBP\_MULTI\_ORGANISM\_MEMBRANE\_FUSION |  | 6 | -0.71 | -1.48 | 0.065 | 0.526 | 1.000 | 1498 | tags=50%, list=12%, signal=57% |
| 362 | GOBP\_RESPIRATORY\_BURST\_INVOLVED\_IN\_INFLAMMATORY\_RESPONSE |  | 5 | -0.75 | -1.48 | 0.057 | 0.534 | 1.000 | 2123 | tags=60%, list=18%, signal=73% |
| 363 | GOMF\_GLYCERALDEHYDE\_3\_PHOSPHATE\_DEHYDROGENASE\_NADPLUS\_NON\_PHOSPHORYLATING\_ACTIVITY |  | 4 | -0.80 | -1.48 | 0.052 | 0.533 | 1.000 | 1850 | tags=75%, list=15%, signal=89% |
| 364 | GOCC\_NURD\_COMPLEX |  | 13 | -0.57 | -1.47 | 0.061 | 0.533 | 1.000 | 2317 | tags=54%, list=19%, signal=67% |
| 365 | GOBP\_RESPONSE\_TO\_GONADOTROPIN\_RELEASING\_HORMONE |  | 3 | -0.87 | -1.47 | 0.038 | 0.533 | 1.000 | 1091 | tags=67%, list=9%, signal=73% |
| 366 | GOBP\_POSITIVE\_REGULATION\_OF\_INTRINSIC\_APOPTOTIC\_SIGNALING\_PATHWAY\_BY\_P53\_CLASS\_MEDIATOR |  | 3 | -0.87 | -1.47 | 0.038 | 0.531 | 1.000 | 944 | tags=67%, list=8%, signal=72% |
| 367 | GOBP\_NEGATIVE\_REGULATION\_OF\_TOR\_SIGNALING |  | 33 | -0.44 | -1.47 | 0.035 | 0.533 | 1.000 | 2607 | tags=42%, list=22%, signal=54% |
| 368 | GOBP\_POSITIVE\_REGULATION\_OF\_SKELETAL\_MUSCLE\_TISSUE\_REGENERATION |  | 3 | -0.88 | -1.47 | 0.030 | 0.532 | 1.000 | 1269 | tags=67%, list=11%, signal=75% |
| 369 | GOBP\_POSITIVE\_REGULATION\_OF\_IMMATURE\_T\_CELL\_PROLIFERATION |  | 3 | -0.86 | -1.47 | 0.038 | 0.534 | 1.000 | 1656 | tags=100%, list=14%, signal=116% |
| 370 | GOBP\_GAMMA\_DELTA\_T\_CELL\_ACTIVATION |  | 11 | -0.58 | -1.47 | 0.076 | 0.535 | 1.000 | 3376 | tags=64%, list=28%, signal=88% |
| 371 | GOMF\_ACETYLCHOLINE\_BINDING |  | 6 | -0.71 | -1.47 | 0.071 | 0.534 | 1.000 | 2156 | tags=67%, list=18%, signal=81% |
| 372 | GOBP\_POSITIVE\_REGULATION\_OF\_LEUKOCYTE\_TETHERING\_OR\_ROLLING |  | 5 | -0.75 | -1.47 | 0.059 | 0.534 | 1.000 | 1133 | tags=60%, list=9%, signal=66% |
| 373 | GOBP\_MITOCHONDRIAL\_ELECTRON\_TRANSPORT\_NADH\_TO\_UBIQUINONE |  | 42 | -0.42 | -1.47 | 0.037 | 0.533 | 1.000 | 4520 | tags=57%, list=38%, signal=91% |
| 374 | GOBP\_TRACHEA\_DEVELOPMENT |  | 13 | -0.56 | -1.47 | 0.067 | 0.533 | 1.000 | 3066 | tags=54%, list=26%, signal=72% |
| 375 | GOBP\_CELLULAR\_RESPIRATION |  | 138 | -0.33 | -1.47 | 0.006 | 0.532 | 1.000 | 4286 | tags=51%, list=36%, signal=78% |
| 376 | GOMF\_MAGNESIUM\_ION\_TRANSMEMBRANE\_TRANSPORTER\_ACTIVITY |  | 12 | -0.58 | -1.47 | 0.059 | 0.532 | 1.000 | 3009 | tags=58%, list=25%, signal=78% |
| 377 | GOBP\_CELLULAR\_HORMONE\_METABOLIC\_PROCESS |  | 76 | -0.37 | -1.47 | 0.014 | 0.531 | 1.000 | 1255 | tags=30%, list=10%, signal=34% |
| 378 | GOMF\_ALDEHYDE\_DEHYDROGENASE\_NAD\_P\_PLUS\_ACTIVITY |  | 10 | -0.60 | -1.47 | 0.078 | 0.534 | 1.000 | 1850 | tags=50%, list=15%, signal=59% |
| 379 | GOBP\_UREA\_METABOLIC\_PROCESS |  | 6 | -0.71 | -1.47 | 0.062 | 0.533 | 1.000 | 1587 | tags=50%, list=13%, signal=58% |
| 380 | GOBP\_OPTIC\_NERVE\_DEVELOPMENT |  | 9 | -0.63 | -1.47 | 0.075 | 0.533 | 1.000 | 1245 | tags=33%, list=10%, signal=37% |
| 381 | GOMF\_ACETYLCHOLINE\_RECEPTOR\_ACTIVITY |  | 8 | -0.65 | -1.47 | 0.069 | 0.532 | 1.000 | 2156 | tags=63%, list=18%, signal=76% |
| 382 | GOBP\_CELLULAR\_RESPONSE\_TO\_AMYLOID\_BETA |  | 24 | -0.48 | -1.47 | 0.049 | 0.531 | 1.000 | 3323 | tags=54%, list=28%, signal=75% |
| 383 | GOBP\_AMP\_BIOSYNTHETIC\_PROCESS |  | 5 | -0.74 | -1.47 | 0.073 | 0.532 | 1.000 | 464 | tags=40%, list=4%, signal=42% |
| 384 | GOBP\_RRNA\_BASE\_METHYLATION |  | 5 | -0.75 | -1.47 | 0.067 | 0.534 | 1.000 | 2754 | tags=80%, list=23%, signal=104% |
| 385 | GOBP\_MACROPINOCYTOSIS |  | 7 | -0.67 | -1.47 | 0.075 | 0.537 | 1.000 | 2694 | tags=71%, list=22%, signal=92% |
| 386 | GOBP\_NEGATIVE\_REGULATION\_OF\_NEUTROPHIL\_MIGRATION |  | 3 | -0.87 | -1.47 | 0.039 | 0.536 | 1.000 | 579 | tags=67%, list=5%, signal=70% |
| 387 | GOMF\_RDNA\_BINDING |  | 5 | -0.74 | -1.47 | 0.068 | 0.535 | 1.000 | 2417 | tags=80%, list=20%, signal=100% |
| 388 | GOBP\_NEGATIVE\_REGULATION\_OF\_SYNAPSE\_ORGANIZATION |  | 6 | -0.70 | -1.47 | 0.074 | 0.535 | 1.000 | 2789 | tags=83%, list=23%, signal=108% |
| 389 | GOBP\_MITOCHONDRIAL\_ELECTRON\_TRANSPORT\_CYTOCHROME\_C\_TO\_OXYGEN |  | 11 | -0.59 | -1.46 | 0.077 | 0.535 | 1.000 | 3121 | tags=55%, list=26%, signal=74% |
| 390 | GOCC\_BBSOME |  | 7 | -0.67 | -1.46 | 0.080 | 0.534 | 1.000 | 2587 | tags=71%, list=22%, signal=91% |
| 391 | GOBP\_RETINOIC\_ACID\_METABOLIC\_PROCESS |  | 15 | -0.53 | -1.46 | 0.064 | 0.534 | 1.000 | 715 | tags=33%, list=6%, signal=35% |
| 392 | GOBP\_CADMIUM\_ION\_TRANSMEMBRANE\_TRANSPORT |  | 4 | -0.79 | -1.46 | 0.051 | 0.533 | 1.000 | 1852 | tags=75%, list=15%, signal=89% |
| 393 | GOBP\_5\_PHOSPHORIBOSE\_1\_DIPHOSPHATE\_METABOLIC\_PROCESS |  | 5 | -0.74 | -1.46 | 0.062 | 0.534 | 1.000 | 2468 | tags=80%, list=21%, signal=101% |
| 394 | GOBP\_CARDIOLIPIN\_METABOLIC\_PROCESS |  | 14 | -0.55 | -1.46 | 0.079 | 0.536 | 1.000 | 1872 | tags=50%, list=16%, signal=59% |
| 395 | GOMF\_VOLTAGE\_GATED\_CATION\_CHANNEL\_ACTIVITY |  | 66 | -0.37 | -1.46 | 0.024 | 0.535 | 1.000 | 1538 | tags=30%, list=13%, signal=35% |
| 396 | GOBP\_RIBONUCLEOPROTEIN\_COMPLEX\_SUBUNIT\_ORGANIZATION |  | 150 | -0.33 | -1.46 | 0.007 | 0.537 | 1.000 | 2351 | tags=27%, list=20%, signal=34% |
| 397 | GOCC\_ORGANELLAR\_RIBOSOME |  | 77 | -0.36 | -1.46 | 0.017 | 0.538 | 1.000 | 4653 | tags=52%, list=39%, signal=84% |
| 398 | GOMF\_GATED\_CHANNEL\_ACTIVITY |  | 163 | -0.32 | -1.46 | 0.005 | 0.537 | 1.000 | 1704 | tags=28%, list=14%, signal=32% |
| 399 | GOCC\_EKC\_KEOPS\_COMPLEX |  | 3 | -0.86 | -1.46 | 0.040 | 0.537 | 1.000 | 1672 | tags=67%, list=14%, signal=77% |
| 400 | GOCC\_NEURONAL\_DENSE\_CORE\_VESICLE\_MEMBRANE |  | 4 | -0.79 | -1.46 | 0.054 | 0.536 | 1.000 | 1665 | tags=50%, list=14%, signal=58% |
| 401 | GOBP\_LEUKOTRIENE\_METABOLIC\_PROCESS |  | 17 | -0.52 | -1.46 | 0.067 | 0.535 | 1.000 | 2005 | tags=41%, list=17%, signal=49% |
| 402 | GOBP\_RRNA\_TRANSPORT |  | 4 | -0.79 | -1.46 | 0.066 | 0.537 | 1.000 | 791 | tags=50%, list=7%, signal=54% |
| 403 | GOMF\_ANION\_SODIUM\_SYMPORTER\_ACTIVITY |  | 8 | -0.64 | -1.46 | 0.076 | 0.536 | 1.000 | 1096 | tags=50%, list=9%, signal=55% |
| 404 | GOBP\_NEGATIVE\_REGULATION\_OF\_INTERLEUKIN\_17\_PRODUCTION |  | 6 | -0.70 | -1.46 | 0.076 | 0.537 | 1.000 | 1557 | tags=50%, list=13%, signal=57% |
| 405 | GOBP\_NEGATIVE\_REGULATION\_OF\_MAST\_CELL\_DEGRANULATION |  | 3 | -0.86 | -1.46 | 0.039 | 0.536 | 1.000 | 1641 | tags=67%, list=14%, signal=77% |
| 406 | GOBP\_MITOCHONDRIAL\_TRANSLATIONAL\_TERMINATION |  | 78 | -0.37 | -1.46 | 0.021 | 0.535 | 1.000 | 4660 | tags=53%, list=39%, signal=85% |
| 407 | GOMF\_OXIDOREDUCTASE\_ACTIVITY\_ACTING\_ON\_A\_SULFUR\_GROUP\_OF\_DONORS |  | 47 | -0.40 | -1.46 | 0.033 | 0.536 | 1.000 | 3553 | tags=51%, list=30%, signal=72% |
| 408 | GOMF\_MHC\_CLASS\_II\_PROTEIN\_BINDING |  | 5 | -0.74 | -1.46 | 0.073 | 0.536 | 1.000 | 2986 | tags=80%, list=25%, signal=106% |
| 409 | GOMF\_ENONE\_REDUCTASE\_ACTIVITY |  | 4 | -0.79 | -1.46 | 0.066 | 0.537 | 1.000 | 1171 | tags=75%, list=10%, signal=83% |
| 410 | GOBP\_DETECTION\_OF\_LIGHT\_STIMULUS |  | 30 | -0.44 | -1.46 | 0.050 | 0.540 | 1.000 | 1646 | tags=27%, list=14%, signal=31% |
| 411 | GOBP\_FATTY\_ACID\_BETA\_OXIDATION\_USING\_ACYL\_COA\_OXIDASE |  | 13 | -0.56 | -1.46 | 0.076 | 0.539 | 1.000 | 1486 | tags=38%, list=12%, signal=44% |
| 412 | GOMF\_PASSIVE\_TRANSMEMBRANE\_TRANSPORTER\_ACTIVITY |  | 231 | -0.31 | -1.46 | 0.001 | 0.539 | 1.000 | 1849 | tags=26%, list=15%, signal=31% |
| 413 | GOBP\_MITOCHONDRIAL\_GENE\_EXPRESSION |  | 137 | -0.33 | -1.45 | 0.011 | 0.541 | 1.000 | 4660 | tags=55%, list=39%, signal=90% |
| 414 | GOMF\_RETINAL\_DEHYDROGENASE\_ACTIVITY |  | 6 | -0.70 | -1.45 | 0.073 | 0.540 | 1.000 | 57 | tags=33%, list=0%, signal=33% |
| 415 | GOBP\_POSITIVE\_REGULATION\_OF\_ACUTE\_INFLAMMATORY\_RESPONSE |  | 17 | -0.52 | -1.45 | 0.065 | 0.544 | 1.000 | 1124 | tags=35%, list=9%, signal=39% |
| 416 | GOBP\_CELLULAR\_AMIDE\_METABOLIC\_PROCESS |  | 816 | -0.27 | -1.45 | 0.000 | 0.543 | 1.000 | 2352 | tags=27%, list=20%, signal=32% |
| 417 | GOBP\_GAS\_TRANSPORT |  | 9 | -0.61 | -1.45 | 0.082 | 0.545 | 1.000 | 1061 | tags=33%, list=9%, signal=37% |
| 418 | GOBP\_GROOMING\_BEHAVIOR |  | 7 | -0.67 | -1.45 | 0.085 | 0.549 | 1.000 | 521 | tags=29%, list=4%, signal=30% |
| 419 | GOBP\_DNA\_DEALKYLATION\_INVOLVED\_IN\_DNA\_REPAIR |  | 10 | -0.60 | -1.45 | 0.077 | 0.549 | 1.000 | 2402 | tags=60%, list=20%, signal=75% |
| 420 | GOBP\_HEPARAN\_SULFATE\_PROTEOGLYCAN\_BIOSYNTHETIC\_PROCESS |  | 20 | -0.49 | -1.45 | 0.068 | 0.548 | 1.000 | 1162 | tags=35%, list=10%, signal=39% |
| 421 | GOMF\_INTRACELLULAR\_LIGAND\_GATED\_ION\_CHANNEL\_ACTIVITY |  | 18 | -0.51 | -1.45 | 0.073 | 0.547 | 1.000 | 679 | tags=22%, list=6%, signal=24% |
| 422 | GOBP\_EMBRYONIC\_APPENDAGE\_MORPHOGENESIS |  | 70 | -0.37 | -1.45 | 0.024 | 0.546 | 1.000 | 2452 | tags=37%, list=20%, signal=46% |
| 423 | GOMF\_TRNA\_GUANINE\_METHYLTRANSFERASE\_ACTIVITY |  | 10 | -0.60 | -1.45 | 0.081 | 0.547 | 1.000 | 3825 | tags=60%, list=32%, signal=88% |
| 424 | GOBP\_TORC1\_SIGNALING |  | 36 | -0.42 | -1.45 | 0.043 | 0.546 | 1.000 | 2974 | tags=44%, list=25%, signal=59% |
| 425 | GOBP\_MYOTUBE\_DIFFERENTIATION |  | 58 | -0.38 | -1.45 | 0.033 | 0.548 | 1.000 | 2313 | tags=36%, list=19%, signal=45% |
| 426 | GOBP\_REGULATION\_OF\_ACUTE\_INFLAMMATORY\_RESPONSE |  | 26 | -0.46 | -1.45 | 0.059 | 0.551 | 1.000 | 1124 | tags=27%, list=9%, signal=30% |
| 427 | GOBP\_REGULATION\_OF\_HYPERSENSITIVITY |  | 4 | -0.79 | -1.45 | 0.071 | 0.550 | 1.000 | 1063 | tags=75%, list=9%, signal=82% |
| 428 | GOMF\_SUCCINATE\_DEHYDROGENASE\_ACTIVITY |  | 5 | -0.73 | -1.45 | 0.076 | 0.549 | 1.000 | 220 | tags=20%, list=2%, signal=20% |
| 429 | GOBP\_FATTY\_ACID\_BETA\_OXIDATION |  | 62 | -0.38 | -1.45 | 0.030 | 0.549 | 1.000 | 1786 | tags=32%, list=15%, signal=38% |
| 430 | GOBP\_CONVERGENT\_EXTENSION\_INVOLVED\_IN\_AXIS\_ELONGATION |  | 4 | -0.78 | -1.45 | 0.067 | 0.548 | 1.000 | 1391 | tags=75%, list=12%, signal=85% |
| 431 | GOBP\_MITOCHONDRIAL\_TRANSLATION |  | 114 | -0.34 | -1.45 | 0.010 | 0.549 | 1.000 | 4660 | tags=54%, list=39%, signal=87% |
| 432 | GOBP\_AXONEMAL\_DYNEIN\_COMPLEX\_ASSEMBLY |  | 12 | -0.57 | -1.45 | 0.074 | 0.548 | 1.000 | 279 | tags=17%, list=2%, signal=17% |
| 433 | GOBP\_REACTIVE\_OXYGEN\_SPECIES\_METABOLIC\_PROCESS |  | 178 | -0.31 | -1.44 | 0.007 | 0.553 | 1.000 | 1481 | tags=25%, list=12%, signal=28% |
| 434 | GOMF\_7SK\_SNRNA\_BINDING |  | 6 | -0.69 | -1.44 | 0.078 | 0.552 | 1.000 | 1645 | tags=50%, list=14%, signal=58% |
| 435 | GOBP\_REGULATION\_OF\_OLIGODENDROCYTE\_DIFFERENTIATION |  | 27 | -0.45 | -1.44 | 0.054 | 0.554 | 1.000 | 2881 | tags=44%, list=24%, signal=58% |
| 436 | GOBP\_POSITIVE\_REGULATION\_OF\_T\_CELL\_RECEPTOR\_SIGNALING\_PATHWAY |  | 10 | -0.58 | -1.44 | 0.083 | 0.553 | 1.000 | 2063 | tags=50%, list=17%, signal=60% |
| 437 | GOBP\_LYSINE\_METABOLIC\_PROCESS |  | 13 | -0.55 | -1.44 | 0.081 | 0.552 | 1.000 | 1792 | tags=38%, list=15%, signal=45% |
| 438 | GOBP\_SNRNA\_METABOLIC\_PROCESS |  | 31 | -0.44 | -1.44 | 0.056 | 0.552 | 1.000 | 3711 | tags=42%, list=31%, signal=61% |
| 439 | GOBP\_BIOTIN\_METABOLIC\_PROCESS |  | 9 | -0.61 | -1.44 | 0.090 | 0.551 | 1.000 | 1580 | tags=44%, list=13%, signal=51% |
| 440 | GOMF\_ELECTRON\_TRANSFER\_ACTIVITY |  | 91 | -0.35 | -1.44 | 0.021 | 0.550 | 1.000 | 4173 | tags=51%, list=35%, signal=77% |
| 441 | GOMF\_HYDROLASE\_ACTIVITY\_ACTING\_ON\_CARBON\_NITROGEN\_BUT\_NOT\_PEPTIDE\_BONDS |  | 78 | -0.36 | -1.44 | 0.016 | 0.550 | 1.000 | 2317 | tags=37%, list=19%, signal=46% |
| 442 | GOMF\_PROTEIN\_ANTIGEN\_BINDING |  | 3 | -0.85 | -1.44 | 0.045 | 0.550 | 1.000 | 1133 | tags=67%, list=9%, signal=74% |
| 443 | GOBP\_NEGATIVE\_REGULATION\_OF\_SMOOTH\_MUSCLE\_CELL\_MIGRATION |  | 16 | -0.52 | -1.44 | 0.081 | 0.550 | 1.000 | 1003 | tags=31%, list=8%, signal=34% |
| 444 | GOBP\_CELLULAR\_RESPONSE\_TO\_EPINEPHRINE\_STIMULUS |  | 10 | -0.59 | -1.44 | 0.084 | 0.551 | 1.000 | 771 | tags=40%, list=6%, signal=43% |
| 445 | GOBP\_REGULATION\_OF\_MOTOR\_NEURON\_APOPTOTIC\_PROCESS |  | 4 | -0.79 | -1.44 | 0.060 | 0.551 | 1.000 | 61 | tags=25%, list=1%, signal=25% |
| 446 | GOMF\_INOSITOL\_HEXAKISPHOSPHATE\_5\_KINASE\_ACTIVITY |  | 4 | -0.78 | -1.44 | 0.065 | 0.558 | 1.000 | 2636 | tags=75%, list=22%, signal=96% |
| 447 | GOBP\_SULFUR\_COMPOUND\_METABOLIC\_PROCESS |  | 271 | -0.30 | -1.44 | 0.001 | 0.557 | 1.000 | 2087 | tags=26%, list=17%, signal=31% |
| 448 | GOBP\_GLYCOLIPID\_TRANSPORT |  | 6 | -0.70 | -1.44 | 0.082 | 0.557 | 1.000 | 3666 | tags=100%, list=31%, signal=144% |
| 449 | GOBP\_RESPONSE\_TO\_XENOBIOTIC\_STIMULUS |  | 60 | -0.38 | -1.44 | 0.033 | 0.559 | 1.000 | 1303 | tags=27%, list=11%, signal=30% |
| 450 | GOMF\_FERROUS\_IRON\_BINDING |  | 20 | -0.48 | -1.44 | 0.068 | 0.559 | 1.000 | 2316 | tags=55%, list=19%, signal=68% |
| 451 | GOBP\_IRON\_ION\_TRANSMEMBRANE\_TRANSPORT |  | 14 | -0.54 | -1.44 | 0.072 | 0.558 | 1.000 | 3520 | tags=64%, list=29%, signal=91% |
| 452 | GOMF\_GLUTATHIONE\_PEROXIDASE\_ACTIVITY |  | 16 | -0.52 | -1.44 | 0.080 | 0.558 | 1.000 | 1940 | tags=38%, list=16%, signal=45% |
| 453 | GOBP\_POSITIVE\_REGULATION\_OF\_BONE\_RESORPTION |  | 15 | -0.52 | -1.44 | 0.075 | 0.557 | 1.000 | 2611 | tags=53%, list=22%, signal=68% |
| 454 | GOMF\_LIGASE\_ACTIVITY\_FORMING\_CARBON\_NITROGEN\_BONDS |  | 38 | -0.41 | -1.44 | 0.044 | 0.556 | 1.000 | 4105 | tags=58%, list=34%, signal=88% |
| 455 | GOCC\_SAGA\_COMPLEX |  | 11 | -0.58 | -1.44 | 0.092 | 0.559 | 1.000 | 1054 | tags=27%, list=9%, signal=30% |
| 456 | GOBP\_REGULATION\_OF\_TRANSLATIONAL\_FIDELITY |  | 12 | -0.56 | -1.43 | 0.077 | 0.559 | 1.000 | 3334 | tags=75%, list=28%, signal=104% |
| 457 | GOMF\_RETINOL\_DEHYDROGENASE\_ACTIVITY |  | 10 | -0.59 | -1.43 | 0.089 | 0.558 | 1.000 | 1068 | tags=50%, list=9%, signal=55% |
| 458 | GOBP\_REGULATION\_OF\_SYNAPTIC\_TRANSMISSION\_GABAERGIC |  | 17 | -0.51 | -1.43 | 0.073 | 0.557 | 1.000 | 1514 | tags=29%, list=13%, signal=34% |
| 459 | GOMF\_UDP\_XYLOSYLTRANSFERASE\_ACTIVITY |  | 8 | -0.63 | -1.43 | 0.087 | 0.558 | 1.000 | 518 | tags=25%, list=4%, signal=26% |
| 460 | GOBP\_OXIDATIVE\_RNA\_DEMETHYLATION |  | 5 | -0.73 | -1.43 | 0.081 | 0.558 | 1.000 | 2402 | tags=80%, list=20%, signal=100% |
| 461 | GOBP\_MATERNAL\_PLACENTA\_DEVELOPMENT |  | 18 | -0.50 | -1.43 | 0.069 | 0.558 | 1.000 | 1424 | tags=28%, list=12%, signal=31% |
| 462 | GOCC\_DENSE\_CORE\_GRANULE\_MEMBRANE |  | 6 | -0.69 | -1.43 | 0.082 | 0.560 | 1.000 | 3240 | tags=67%, list=27%, signal=91% |
| 463 | GOBP\_CELLULAR\_TRIVALENT\_INORGANIC\_ANION\_HOMEOSTASIS |  | 3 | -0.84 | -1.43 | 0.062 | 0.559 | 1.000 | 1250 | tags=67%, list=10%, signal=74% |
| 464 | GOCC\_AUTOLYSOSOME |  | 9 | -0.61 | -1.43 | 0.093 | 0.560 | 1.000 | 3197 | tags=67%, list=27%, signal=91% |
| 465 | GOBP\_NEGATIVE\_REGULATION\_OF\_PROTEOLYSIS\_INVOLVED\_IN\_CELLULAR\_PROTEIN\_CATABOLIC\_PROCESS |  | 47 | -0.39 | -1.43 | 0.042 | 0.564 | 1.000 | 2678 | tags=36%, list=22%, signal=46% |
| 466 | GOMF\_LIPOPEPTIDE\_BINDING |  | 4 | -0.78 | -1.43 | 0.075 | 0.564 | 1.000 | 2123 | tags=75%, list=18%, signal=91% |
| 467 | GOBP\_AXON\_MIDLINE\_CHOICE\_POINT\_RECOGNITION |  | 3 | -0.85 | -1.43 | 0.064 | 0.567 | 1.000 | 1489 | tags=67%, list=12%, signal=76% |
| 468 | GOBP\_TANGENTIAL\_MIGRATION\_FROM\_THE\_SUBVENTRICULAR\_ZONE\_TO\_THE\_OLFACTORY\_BULB |  | 4 | -0.77 | -1.43 | 0.075 | 0.567 | 1.000 | 969 | tags=50%, list=8%, signal=54% |
| 469 | GOBP\_REGULATION\_OF\_SALIVA\_SECRETION |  | 3 | -0.84 | -1.43 | 0.054 | 0.568 | 1.000 | 12 | tags=33%, list=0%, signal=33% |
| 470 | GOBP\_POSITIVE\_REGULATION\_OF\_ENDOTHELIAL\_CELL\_DEVELOPMENT |  | 5 | -0.72 | -1.43 | 0.091 | 0.568 | 1.000 | 1338 | tags=40%, list=11%, signal=45% |
| 471 | GOMF\_SOLUTE\_PROTON\_SYMPORTER\_ACTIVITY |  | 13 | -0.54 | -1.43 | 0.086 | 0.567 | 1.000 | 2888 | tags=62%, list=24%, signal=81% |
| 472 | GOMF\_HYDROLASE\_ACTIVITY\_ACTING\_ON\_CARBON\_NITROGEN\_BUT\_NOT\_PEPTIDE\_BONDS\_IN\_LINEAR\_AMIDINES |  | 4 | -0.77 | -1.42 | 0.068 | 0.577 | 1.000 | 1402 | tags=75%, list=12%, signal=85% |
| 473 | GOBP\_MATURATION\_OF\_SSU\_RRNA\_FROM\_TRICISTRONIC\_RRNA\_TRANSCRIPT\_SSU\_RRNA\_5\_8S\_RRNA\_LSU\_RRNA |  | 27 | -0.45 | -1.42 | 0.070 | 0.577 | 1.000 | 4068 | tags=67%, list=34%, signal=101% |
| 474 | GOBP\_URONIC\_ACID\_METABOLIC\_PROCESS |  | 9 | -0.60 | -1.42 | 0.095 | 0.576 | 1.000 | 2606 | tags=67%, list=22%, signal=85% |
| 475 | GOBP\_NEGATIVE\_REGULATION\_OF\_TORC1\_SIGNALING |  | 12 | -0.56 | -1.42 | 0.092 | 0.575 | 1.000 | 2607 | tags=50%, list=22%, signal=64% |
| 476 | GOMF\_ALPHA\_2A\_ADRENERGIC\_RECEPTOR\_BINDING |  | 3 | -0.85 | -1.42 | 0.053 | 0.576 | 1.000 | 768 | tags=33%, list=6%, signal=36% |
| 477 | GOBP\_LEUKOTRIENE\_BIOSYNTHETIC\_PROCESS |  | 11 | -0.57 | -1.42 | 0.100 | 0.576 | 1.000 | 1940 | tags=36%, list=16%, signal=43% |
| 478 | GOBP\_RECOGNITION\_OF\_APOPTOTIC\_CELL |  | 5 | -0.72 | -1.42 | 0.091 | 0.577 | 1.000 | 1661 | tags=40%, list=14%, signal=46% |
| 479 | GOCC\_RESPIRATORY\_CHAIN\_COMPLEX\_II |  | 5 | -0.73 | -1.42 | 0.086 | 0.578 | 1.000 | 220 | tags=20%, list=2%, signal=20% |
| 480 | GOBP\_REGULATION\_OF\_TORC1\_SIGNALING |  | 28 | -0.44 | -1.42 | 0.067 | 0.580 | 1.000 | 2974 | tags=46%, list=25%, signal=62% |
| 481 | GOBP\_SKELETAL\_MUSCLE\_SATELLITE\_CELL\_ACTIVATION |  | 5 | -0.72 | -1.42 | 0.086 | 0.580 | 1.000 | 3020 | tags=80%, list=25%, signal=107% |
| 482 | GOBP\_ESTABLISHMENT\_OF\_PLANAR\_POLARITY\_OF\_EMBRYONIC\_EPITHELIUM |  | 11 | -0.57 | -1.42 | 0.095 | 0.581 | 1.000 | 677 | tags=27%, list=6%, signal=29% |
| 483 | GOBP\_REGULATION\_OF\_HUMORAL\_IMMUNE\_RESPONSE\_MEDIATED\_BY\_CIRCULATING\_IMMUNOGLOBULIN |  | 6 | -0.68 | -1.42 | 0.081 | 0.580 | 1.000 | 3133 | tags=67%, list=26%, signal=90% |
| 484 | GOBP\_EMBRYO\_IMPLANTATION |  | 34 | -0.42 | -1.42 | 0.044 | 0.582 | 1.000 | 2065 | tags=44%, list=17%, signal=53% |
| 485 | GOCC\_LEWY\_BODY |  | 6 | -0.68 | -1.42 | 0.099 | 0.581 | 1.000 | 3197 | tags=83%, list=27%, signal=114% |
| 486 | GOMF\_TRANSLATION\_REGULATOR\_ACTIVITY |  | 103 | -0.34 | -1.42 | 0.020 | 0.580 | 1.000 | 2130 | tags=27%, list=18%, signal=33% |
| 487 | GOCC\_AZUROPHIL\_GRANULE\_LUMEN |  | 55 | -0.38 | -1.42 | 0.045 | 0.582 | 1.000 | 3238 | tags=44%, list=27%, signal=59% |
| 488 | GOMF\_DIPHOSPHOTRANSFERASE\_ACTIVITY |  | 5 | -0.71 | -1.42 | 0.089 | 0.582 | 1.000 | 2468 | tags=60%, list=21%, signal=75% |
| 489 | GOBP\_MAMMARY\_GLAND\_ALVEOLUS\_DEVELOPMENT |  | 14 | -0.53 | -1.42 | 0.096 | 0.581 | 1.000 | 2611 | tags=57%, list=22%, signal=73% |
| 490 | GOBP\_CELLULAR\_RESPONSE\_TO\_ZINC\_ION |  | 10 | -0.58 | -1.42 | 0.091 | 0.580 | 1.000 | 651 | tags=30%, list=5%, signal=32% |
| 491 | GOBP\_CELLULAR\_OXIDANT\_DETOXIFICATION |  | 61 | -0.37 | -1.42 | 0.039 | 0.582 | 1.000 | 1940 | tags=30%, list=16%, signal=35% |
| 492 | GOMF\_VOLTAGE\_GATED\_SODIUM\_CHANNEL\_ACTIVITY\_INVOLVED\_IN\_CARDIAC\_MUSCLE\_CELL\_ACTION\_POTENTIAL |  | 3 | -0.83 | -1.42 | 0.066 | 0.581 | 1.000 | 728 | tags=67%, list=6%, signal=71% |
| 493 | GOMF\_OXIDATIVE\_RNA\_DEMETHYLASE\_ACTIVITY |  | 5 | -0.73 | -1.42 | 0.090 | 0.579 | 1.000 | 2402 | tags=80%, list=20%, signal=100% |
| 494 | GOBP\_SUPEROXIDE\_METABOLIC\_PROCESS |  | 44 | -0.40 | -1.42 | 0.044 | 0.579 | 1.000 | 1292 | tags=30%, list=11%, signal=33% |
| 495 | GOBP\_PROGESTERONE\_METABOLIC\_PROCESS |  | 6 | -0.67 | -1.42 | 0.100 | 0.580 | 1.000 | 878 | tags=50%, list=7%, signal=54% |
| 496 | GOBP\_THALAMUS\_DEVELOPMENT |  | 9 | -0.60 | -1.41 | 0.089 | 0.585 | 1.000 | 3066 | tags=67%, list=26%, signal=89% |
| 497 | GOMF\_PROTEIN\_TYROSINE\_KINASE\_INHIBITOR\_ACTIVITY |  | 3 | -0.84 | -1.41 | 0.066 | 0.585 | 1.000 | 1498 | tags=67%, list=12%, signal=76% |
| 498 | GOBP\_UTERUS\_DEVELOPMENT |  | 11 | -0.57 | -1.41 | 0.086 | 0.584 | 1.000 | 1589 | tags=36%, list=13%, signal=42% |
| 499 | GOCC\_SODIUM\_CHANNEL\_COMPLEX |  | 12 | -0.55 | -1.41 | 0.097 | 0.583 | 1.000 | 1316 | tags=33%, list=11%, signal=37% |
| 500 | GOBP\_REGULATION\_OF\_DENDRITIC\_CELL\_CHEMOTAXIS |  | 6 | -0.68 | -1.41 | 0.102 | 0.582 | 1.000 | 1063 | tags=33%, list=9%, signal=37% |
| 501 | GOMF\_ACID\_AMINO\_ACID\_LIGASE\_ACTIVITY |  | 16 | -0.51 | -1.41 | 0.079 | 0.584 | 1.000 | 3427 | tags=50%, list=29%, signal=70% |
| 502 | GOMF\_C\_METHYLTRANSFERASE\_ACTIVITY |  | 5 | -0.72 | -1.41 | 0.085 | 0.583 | 1.000 | 2754 | tags=60%, list=23%, signal=78% |
| 503 | GOMF\_FERRIC\_IRON\_BINDING |  | 6 | -0.68 | -1.41 | 0.091 | 0.582 | 1.000 | 819 | tags=50%, list=7%, signal=54% |
| 504 | GOBP\_NEGATIVE\_REGULATION\_OF\_MONOCYTE\_DIFFERENTIATION |  | 5 | -0.72 | -1.41 | 0.097 | 0.582 | 1.000 | 2046 | tags=80%, list=17%, signal=96% |
| 505 | GOBP\_REGIONALIZATION |  | 190 | -0.31 | -1.41 | 0.006 | 0.581 | 1.000 | 2375 | tags=35%, list=20%, signal=43% |
| 506 | GOBP\_LOCOMOTOR\_RHYTHM |  | 11 | -0.56 | -1.41 | 0.098 | 0.579 | 1.000 | 1952 | tags=45%, list=16%, signal=54% |
| 507 | GOBP\_NEGATIVE\_REGULATION\_OF\_INTERLEUKIN\_13\_PRODUCTION |  | 3 | -0.84 | -1.41 | 0.061 | 0.579 | 1.000 | 1380 | tags=67%, list=11%, signal=75% |
| 508 | GOBP\_MESENCHYME\_MIGRATION |  | 5 | -0.72 | -1.41 | 0.095 | 0.579 | 1.000 | 1641 | tags=60%, list=14%, signal=69% |
| 509 | GOCC\_DENSE\_CORE\_GRANULE |  | 20 | -0.48 | -1.41 | 0.085 | 0.579 | 1.000 | 2628 | tags=55%, list=22%, signal=70% |
| 510 | GOCC\_DYNACTIN\_COMPLEX |  | 10 | -0.58 | -1.41 | 0.097 | 0.579 | 1.000 | 555 | tags=20%, list=5%, signal=21% |
| 511 | GOBP\_REGULATION\_OF\_VIRAL\_TRANSCRIPTION |  | 29 | -0.44 | -1.41 | 0.073 | 0.578 | 1.000 | 3741 | tags=45%, list=31%, signal=65% |
| 512 | GOBP\_HISTONE\_H3\_K9\_TRIMETHYLATION |  | 7 | -0.65 | -1.41 | 0.102 | 0.577 | 1.000 | 2541 | tags=57%, list=21%, signal=72% |
| 513 | GOBP\_POSITIVE\_REGULATION\_OF\_GAMMA\_DELTA\_T\_CELL\_DIFFERENTIATION |  | 6 | -0.68 | -1.41 | 0.102 | 0.578 | 1.000 | 3133 | tags=83%, list=26%, signal=113% |
| 514 | GOMF\_DIPEPTIDYL\_PEPTIDASE\_ACTIVITY |  | 8 | -0.62 | -1.41 | 0.093 | 0.577 | 1.000 | 638 | tags=38%, list=5%, signal=40% |
| 515 | GOBP\_FATTY\_ACID\_OMEGA\_OXIDATION |  | 3 | -0.83 | -1.41 | 0.061 | 0.576 | 1.000 | 244 | tags=67%, list=2%, signal=68% |
| 516 | GOBP\_MEMBRANE\_DEPOLARIZATION\_DURING\_AV\_NODE\_CELL\_ACTION\_POTENTIAL |  | 4 | -0.77 | -1.41 | 0.085 | 0.575 | 1.000 | 2248 | tags=75%, list=19%, signal=92% |
| 517 | GOBP\_NEGATIVE\_REGULATION\_OF\_MITOCHONDRION\_ORGANIZATION |  | 34 | -0.42 | -1.41 | 0.057 | 0.575 | 1.000 | 3109 | tags=44%, list=26%, signal=59% |
| 518 | GOBP\_POSITIVE\_REGULATION\_OF\_INTERLEUKIN\_4\_PRODUCTION |  | 14 | -0.52 | -1.41 | 0.093 | 0.574 | 1.000 | 3790 | tags=64%, list=32%, signal=94% |
| 519 | GOBP\_RELAXATION\_OF\_VASCULAR\_ASSOCIATED\_SMOOTH\_MUSCLE |  | 4 | -0.77 | -1.41 | 0.081 | 0.577 | 1.000 | 2737 | tags=75%, list=23%, signal=97% |
| 520 | GOBP\_PEPTIDYL\_DIPHTHAMIDE\_BIOSYNTHETIC\_PROCESS\_FROM\_PEPTIDYL\_HISTIDINE |  | 7 | -0.64 | -1.41 | 0.108 | 0.578 | 1.000 | 3162 | tags=71%, list=26%, signal=97% |
| 521 | GOBP\_AGGRESSIVE\_BEHAVIOR |  | 4 | -0.76 | -1.41 | 0.094 | 0.577 | 1.000 | 2271 | tags=75%, list=19%, signal=92% |
| 522 | GOMF\_VOLTAGE\_GATED\_POTASSIUM\_CHANNEL\_ACTIVITY |  | 39 | -0.41 | -1.41 | 0.059 | 0.578 | 1.000 | 1385 | tags=31%, list=12%, signal=35% |
| 523 | GOBP\_VENTRICULAR\_SYSTEM\_DEVELOPMENT |  | 19 | -0.49 | -1.41 | 0.089 | 0.579 | 1.000 | 1371 | tags=32%, list=11%, signal=36% |
| 524 | GOBP\_REGULATION\_OF\_DOPAMINERGIC\_NEURON\_DIFFERENTIATION |  | 6 | -0.68 | -1.41 | 0.102 | 0.578 | 1.000 | 1656 | tags=50%, list=14%, signal=58% |
| 525 | GOBP\_ETHANOL\_CATABOLIC\_PROCESS |  | 3 | -0.83 | -1.41 | 0.063 | 0.579 | 1.000 | 2014 | tags=100%, list=17%, signal=120% |
| 526 | GOCC\_VACUOLE |  | 570 | -0.27 | -1.41 | 0.000 | 0.580 | 1.000 | 3273 | tags=37%, list=27%, signal=48% |
| 527 | GOMF\_ION\_TRANSMEMBRANE\_TRANSPORTER\_ACTIVITY |  | 535 | -0.27 | -1.41 | 0.000 | 0.579 | 1.000 | 1961 | tags=25%, list=16%, signal=29% |
| 528 | GOBP\_MACROPHAGE\_INFLAMMATORY\_PROTEIN\_1\_ALPHA\_PRODUCTION |  | 4 | -0.76 | -1.41 | 0.087 | 0.580 | 1.000 | 378 | tags=50%, list=3%, signal=52% |
| 529 | GOBP\_RRNA\_METHYLATION |  | 17 | -0.50 | -1.41 | 0.090 | 0.579 | 1.000 | 2754 | tags=41%, list=23%, signal=53% |
| 530 | GOBP\_MULTI\_ORGANISM\_MEMBRANE\_ORGANIZATION |  | 8 | -0.62 | -1.41 | 0.107 | 0.579 | 1.000 | 1498 | tags=38%, list=12%, signal=43% |
| 531 | GOCC\_MITOCHONDRIAL\_TRICARBOXYLIC\_ACID\_CYCLE\_ENZYME\_COMPLEX |  | 5 | -0.71 | -1.41 | 0.105 | 0.578 | 1.000 | 3471 | tags=100%, list=29%, signal=141% |
| 532 | GOMF\_ALCOHOL\_DEHYDROGENASE\_ACTIVITY\_ZINC\_DEPENDENT |  | 3 | -0.83 | -1.41 | 0.069 | 0.578 | 1.000 | 244 | tags=67%, list=2%, signal=68% |
| 533 | GOMF\_GLYCINE\_TRANSMEMBRANE\_TRANSPORTER\_ACTIVITY |  | 3 | -0.83 | -1.40 | 0.062 | 0.577 | 1.000 | 637 | tags=67%, list=5%, signal=70% |
| 534 | GOBP\_MYOTUBE\_CELL\_DEVELOPMENT |  | 18 | -0.49 | -1.40 | 0.089 | 0.576 | 1.000 | 2255 | tags=39%, list=19%, signal=48% |
| 535 | GOMF\_MOLECULAR\_CARRIER\_ACTIVITY |  | 48 | -0.38 | -1.40 | 0.046 | 0.576 | 1.000 | 3508 | tags=38%, list=29%, signal=53% |
| 536 | GOBP\_SULFUR\_AMINO\_ACID\_CATABOLIC\_PROCESS |  | 10 | -0.57 | -1.40 | 0.098 | 0.578 | 1.000 | 779 | tags=40%, list=6%, signal=43% |
| 537 | GOCC\_RESPIRATORY\_CHAIN\_COMPLEX |  | 56 | -0.37 | -1.40 | 0.043 | 0.578 | 1.000 | 4883 | tags=54%, list=41%, signal=90% |
| 538 | GOMF\_VOLTAGE\_GATED\_ION\_CHANNEL\_ACTIVITY |  | 102 | -0.33 | -1.40 | 0.019 | 0.579 | 1.000 | 1538 | tags=28%, list=13%, signal=32% |
| 539 | GOBP\_POSITIVE\_REGULATION\_OF\_GAMMA\_DELTA\_T\_CELL\_ACTIVATION |  | 6 | -0.68 | -1.40 | 0.102 | 0.579 | 1.000 | 3133 | tags=83%, list=26%, signal=113% |
| 540 | GOBP\_POSITIVE\_REGULATION\_OF\_HORMONE\_BIOSYNTHETIC\_PROCESS |  | 7 | -0.65 | -1.40 | 0.107 | 0.581 | 1.000 | 1418 | tags=43%, list=12%, signal=49% |
| 541 | GOMF\_LEUKOTRIENE\_C4\_SYNTHASE\_ACTIVITY |  | 3 | -0.84 | -1.40 | 0.060 | 0.582 | 1.000 | 1940 | tags=67%, list=16%, signal=79% |
| 542 | GOBP\_INTERLEUKIN\_4\_PRODUCTION |  | 19 | -0.48 | -1.40 | 0.080 | 0.581 | 1.000 | 2711 | tags=53%, list=23%, signal=68% |
| 543 | GOMF\_ARYL\_SULFOTRANSFERASE\_ACTIVITY |  | 3 | -0.83 | -1.40 | 0.074 | 0.583 | 1.000 | 2014 | tags=100%, list=17%, signal=120% |
| 544 | GOBP\_AMINE\_BIOSYNTHETIC\_PROCESS |  | 26 | -0.44 | -1.40 | 0.069 | 0.583 | 1.000 | 1044 | tags=31%, list=9%, signal=34% |
| 545 | GOBP\_POLYAMINE\_BIOSYNTHETIC\_PROCESS |  | 12 | -0.55 | -1.40 | 0.096 | 0.583 | 1.000 | 584 | tags=25%, list=5%, signal=26% |
| 546 | GOBP\_CELLULAR\_RESPONSE\_TO\_TOXIC\_SUBSTANCE |  | 70 | -0.36 | -1.40 | 0.033 | 0.583 | 1.000 | 1940 | tags=29%, list=16%, signal=34% |
| 547 | GOBP\_NEGATIVE\_REGULATION\_OF\_MITOCHONDRIAL\_MEMBRANE\_PERMEABILITY |  | 4 | -0.76 | -1.40 | 0.087 | 0.585 | 1.000 | 2461 | tags=75%, list=20%, signal=94% |
| 548 | GOBP\_HEXOSE\_PHOSPHATE\_TRANSPORT |  | 4 | -0.76 | -1.40 | 0.095 | 0.585 | 1.000 | 956 | tags=75%, list=8%, signal=81% |
| 549 | GOMF\_BETA\_GALACTOSIDASE\_ACTIVITY |  | 4 | -0.76 | -1.40 | 0.081 | 0.584 | 1.000 | 1366 | tags=75%, list=11%, signal=85% |
| 550 | GOBP\_VESICLE\_FUSION\_TO\_PLASMA\_MEMBRANE |  | 13 | -0.54 | -1.40 | 0.104 | 0.584 | 1.000 | 1711 | tags=46%, list=14%, signal=54% |
| 551 | GOMF\_PEROXIREDOXIN\_ACTIVITY |  | 5 | -0.71 | -1.40 | 0.100 | 0.585 | 1.000 | 2505 | tags=60%, list=21%, signal=76% |
| 552 | GOMF\_OXIDOREDUCTASE\_ACTIVITY\_ACTING\_ON\_METAL\_IONS |  | 14 | -0.52 | -1.40 | 0.101 | 0.585 | 1.000 | 819 | tags=36%, list=7%, signal=38% |
| 553 | GOBP\_FLAVONOID\_METABOLIC\_PROCESS |  | 3 | -0.83 | -1.40 | 0.068 | 0.584 | 1.000 | 2014 | tags=100%, list=17%, signal=120% |
| 554 | GOBP\_NADH\_DEHYDROGENASE\_COMPLEX\_ASSEMBLY |  | 51 | -0.38 | -1.40 | 0.049 | 0.587 | 1.000 | 4520 | tags=49%, list=38%, signal=78% |
| 555 | GOBP\_FRUCTOSE\_METABOLIC\_PROCESS |  | 12 | -0.55 | -1.40 | 0.106 | 0.587 | 1.000 | 57 | tags=17%, list=0%, signal=17% |
| 556 | GOMF\_FOUR\_WAY\_JUNCTION\_HELICASE\_ACTIVITY |  | 5 | -0.70 | -1.40 | 0.108 | 0.587 | 1.000 | 2331 | tags=40%, list=19%, signal=50% |
| 557 | GOBP\_REGULATION\_OF\_TISSUE\_REMODELING |  | 51 | -0.38 | -1.39 | 0.057 | 0.588 | 1.000 | 2194 | tags=37%, list=18%, signal=45% |
| 558 | GOMF\_RETROMER\_COMPLEX\_BINDING |  | 5 | -0.70 | -1.39 | 0.111 | 0.587 | 1.000 | 2851 | tags=60%, list=24%, signal=79% |
| 559 | GOBP\_LEUKOCYTE\_CHEMOTAXIS\_INVOLVED\_IN\_INFLAMMATORY\_RESPONSE |  | 5 | -0.71 | -1.39 | 0.113 | 0.587 | 1.000 | 2789 | tags=60%, list=23%, signal=78% |
| 560 | GOBP\_BRAINSTEM\_DEVELOPMENT |  | 4 | -0.75 | -1.39 | 0.093 | 0.588 | 1.000 | 713 | tags=50%, list=6%, signal=53% |
| 561 | GOMF\_ORGANIC\_ANION\_TRANSMEMBRANE\_TRANSPORTER\_ACTIVITY |  | 117 | -0.32 | -1.39 | 0.021 | 0.588 | 1.000 | 1561 | tags=24%, list=13%, signal=27% |
| 562 | GOCC\_GEMINI\_OF\_COILED\_BODIES |  | 8 | -0.61 | -1.39 | 0.107 | 0.591 | 1.000 | 3349 | tags=50%, list=28%, signal=69% |
| 563 | GOBP\_PLANAR\_CELL\_POLARITY\_PATHWAY\_INVOLVED\_IN\_AXIS\_ELONGATION |  | 4 | -0.76 | -1.39 | 0.093 | 0.592 | 1.000 | 629 | tags=50%, list=5%, signal=53% |
| 564 | GOBP\_SPINAL\_CORD\_MOTOR\_NEURON\_DIFFERENTIATION |  | 18 | -0.49 | -1.39 | 0.090 | 0.591 | 1.000 | 2558 | tags=44%, list=21%, signal=56% |
| 565 | GOBP\_SENSORY\_PERCEPTION\_OF\_MECHANICAL\_STIMULUS |  | 85 | -0.34 | -1.39 | 0.033 | 0.591 | 1.000 | 2582 | tags=33%, list=21%, signal=42% |
| 566 | GOMF\_DICARBOXYLIC\_ACID\_TRANSMEMBRANE\_TRANSPORTER\_ACTIVITY |  | 22 | -0.46 | -1.39 | 0.084 | 0.591 | 1.000 | 353 | tags=18%, list=3%, signal=19% |
| 567 | GOBP\_NEGATIVE\_REGULATION\_OF\_GTPASE\_ACTIVITY |  | 31 | -0.42 | -1.39 | 0.067 | 0.591 | 1.000 | 2091 | tags=32%, list=17%, signal=39% |
| 568 | GOBP\_UTP\_METABOLIC\_PROCESS |  | 8 | -0.61 | -1.39 | 0.116 | 0.590 | 1.000 | 1626 | tags=50%, list=14%, signal=58% |
| 569 | GOBP\_REGULATION\_OF\_BONE\_REMODELING |  | 38 | -0.40 | -1.39 | 0.070 | 0.589 | 1.000 | 2611 | tags=47%, list=22%, signal=60% |
| 570 | GOBP\_METANEPHRIC\_TUBULE\_FORMATION |  | 3 | -0.81 | -1.39 | 0.082 | 0.589 | 1.000 | 141 | tags=33%, list=1%, signal=34% |
| 571 | GOBP\_NEGATIVE\_REGULATION\_OF\_REACTIVE\_OXYGEN\_SPECIES\_METABOLIC\_PROCESS |  | 35 | -0.41 | -1.39 | 0.072 | 0.589 | 1.000 | 1596 | tags=31%, list=13%, signal=36% |
| 572 | GOBP\_PROTEIN\_LOCALIZATION\_TO\_NUCLEOPLASM |  | 11 | -0.55 | -1.39 | 0.111 | 0.590 | 1.000 | 3344 | tags=45%, list=28%, signal=63% |
| 573 | GOBP\_GALACTOSE\_METABOLIC\_PROCESS |  | 12 | -0.54 | -1.39 | 0.111 | 0.592 | 1.000 | 3139 | tags=58%, list=26%, signal=79% |
| 574 | GOBP\_HYPOXIA\_INDUCIBLE\_FACTOR\_1ALPHA\_SIGNALING\_PATHWAY |  | 5 | -0.70 | -1.39 | 0.113 | 0.591 | 1.000 | 1418 | tags=60%, list=12%, signal=68% |
| 575 | GOBP\_TRNA\_3\_END\_PROCESSING |  | 6 | -0.66 | -1.39 | 0.115 | 0.593 | 1.000 | 3825 | tags=83%, list=32%, signal=122% |
| 576 | GOBP\_CELLULAR\_AMINO\_ACID\_METABOLIC\_PROCESS |  | 229 | -0.30 | -1.39 | 0.005 | 0.593 | 1.000 | 2421 | tags=27%, list=20%, signal=33% |
| 577 | GOBP\_POSITIVE\_REGULATION\_OF\_RECEPTOR\_CLUSTERING |  | 5 | -0.70 | -1.39 | 0.114 | 0.594 | 1.000 | 3477 | tags=80%, list=29%, signal=113% |
| 578 | GOBP\_NEGATIVE\_REGULATION\_OF\_OXIDOREDUCTASE\_ACTIVITY |  | 13 | -0.53 | -1.39 | 0.108 | 0.597 | 1.000 | 844 | tags=31%, list=7%, signal=33% |
| 579 | GOBP\_POSITIVE\_REGULATION\_OF\_CELL\_GROWTH |  | 108 | -0.33 | -1.39 | 0.027 | 0.597 | 1.000 | 2662 | tags=32%, list=22%, signal=41% |
| 580 | GOMF\_OXIDOREDUCTASE\_ACTIVITY\_ACTING\_ON\_THE\_ALDEHYDE\_OR\_OXO\_GROUP\_OF\_DONORS\_NAD\_OR\_NADP\_AS\_ACCEPTOR |  | 25 | -0.44 | -1.38 | 0.086 | 0.600 | 1.000 | 1850 | tags=28%, list=15%, signal=33% |
| 581 | GOBP\_POSITIVE\_REGULATION\_OF\_CELLULAR\_PH\_REDUCTION |  | 4 | -0.75 | -1.38 | 0.101 | 0.602 | 1.000 | 1061 | tags=50%, list=9%, signal=55% |
| 582 | GOBP\_RESPIRATORY\_CHAIN\_COMPLEX\_IV\_ASSEMBLY |  | 17 | -0.49 | -1.38 | 0.097 | 0.603 | 1.000 | 3562 | tags=59%, list=30%, signal=84% |
| 583 | GOBP\_PROTEIN\_LOCALIZATION\_TO\_NUCLEAR\_BODY |  | 11 | -0.55 | -1.38 | 0.118 | 0.603 | 1.000 | 3344 | tags=45%, list=28%, signal=63% |
| 584 | GOBP\_N\_TERMINAL\_PROTEIN\_LIPIDATION |  | 5 | -0.70 | -1.38 | 0.114 | 0.603 | 1.000 | 707 | tags=40%, list=6%, signal=42% |
| 585 | GOBP\_CANONICAL\_WNT\_SIGNALING\_PATHWAY\_INVOLVED\_IN\_REGULATION\_OF\_CELL\_PROLIFERATION |  | 4 | -0.75 | -1.38 | 0.102 | 0.605 | 1.000 | 677 | tags=50%, list=6%, signal=53% |
| 586 | GOBP\_GLYOXYLATE\_METABOLIC\_PROCESS |  | 7 | -0.63 | -1.38 | 0.120 | 0.605 | 1.000 | 2591 | tags=43%, list=22%, signal=55% |
| 587 | GOBP\_SENSORY\_PERCEPTION\_OF\_SMELL |  | 10 | -0.57 | -1.38 | 0.111 | 0.604 | 1.000 | 2586 | tags=50%, list=22%, signal=64% |
| 588 | GOMF\_OXIDOREDUCTASE\_ACTIVITY\_ACTING\_ON\_NAD\_P\_H\_QUINONE\_OR\_SIMILAR\_COMPOUND\_AS\_ACCEPTOR |  | 45 | -0.38 | -1.38 | 0.062 | 0.604 | 1.000 | 4520 | tags=51%, list=38%, signal=82% |
| 589 | GOBP\_LIPOXYGENASE\_PATHWAY |  | 7 | -0.63 | -1.38 | 0.115 | 0.603 | 1.000 | 1558 | tags=43%, list=13%, signal=49% |
| 590 | GOBP\_ALPHA\_AMINO\_ACID\_METABOLIC\_PROCESS |  | 130 | -0.32 | -1.38 | 0.018 | 0.604 | 1.000 | 2099 | tags=27%, list=17%, signal=32% |
| 591 | GOCC\_BETA\_CATENIN\_TCF\_COMPLEX |  | 12 | -0.54 | -1.38 | 0.107 | 0.605 | 1.000 | 1797 | tags=50%, list=15%, signal=59% |
| 592 | GOBP\_PHOSPHOLIPASE\_C\_ACTIVATING\_DOPAMINE\_RECEPTOR\_SIGNALING\_PATHWAY |  | 4 | -0.75 | -1.38 | 0.103 | 0.605 | 1.000 | 1289 | tags=50%, list=11%, signal=56% |
| 593 | GOBP\_POSITIVE\_REGULATION\_OF\_NEURON\_MIGRATION |  | 11 | -0.56 | -1.38 | 0.113 | 0.606 | 1.000 | 1259 | tags=18%, list=10%, signal=20% |
| 594 | GOBP\_LATE\_ENDOSOME\_TO\_LYSOSOME\_TRANSPORT |  | 6 | -0.67 | -1.38 | 0.119 | 0.606 | 1.000 | 1711 | tags=50%, list=14%, signal=58% |
| 595 | GOBP\_POSITIVE\_REGULATION\_OF\_VASCULAR\_WOUND\_HEALING |  | 3 | -0.81 | -1.38 | 0.091 | 0.607 | 1.000 | 970 | tags=33%, list=8%, signal=36% |
| 596 | GOMF\_ANION\_CATION\_SYMPORTER\_ACTIVITY |  | 15 | -0.50 | -1.38 | 0.108 | 0.610 | 1.000 | 1941 | tags=47%, list=16%, signal=56% |
| 597 | GOBP\_HISTONE\_H4\_K5\_ACETYLATION |  | 13 | -0.52 | -1.38 | 0.098 | 0.610 | 1.000 | 2484 | tags=46%, list=21%, signal=58% |
| 598 | GOBP\_ADULT\_LOCOMOTORY\_BEHAVIOR |  | 37 | -0.40 | -1.38 | 0.066 | 0.611 | 1.000 | 661 | tags=16%, list=6%, signal=17% |
| 599 | GOMF\_UBIQUITIN\_PROTEIN\_TRANSFERASE\_REGULATOR\_ACTIVITY |  | 15 | -0.51 | -1.38 | 0.113 | 0.611 | 1.000 | 3161 | tags=53%, list=26%, signal=72% |
| 600 | GOBP\_DICARBOXYLIC\_ACID\_METABOLIC\_PROCESS |  | 68 | -0.35 | -1.38 | 0.044 | 0.612 | 1.000 | 1783 | tags=26%, list=15%, signal=31% |
| 601 | GOCC\_RESPIRASOME |  | 65 | -0.35 | -1.37 | 0.048 | 0.613 | 1.000 | 4883 | tags=54%, list=41%, signal=90% |
| 602 | GOBP\_ARGININE\_CATABOLIC\_PROCESS |  | 7 | -0.62 | -1.37 | 0.127 | 0.612 | 1.000 | 2936 | tags=57%, list=24%, signal=76% |
| 603 | GOCC\_NASCENT\_POLYPEPTIDE\_ASSOCIATED\_COMPLEX |  | 3 | -0.82 | -1.37 | 0.086 | 0.611 | 1.000 | 2216 | tags=100%, list=18%, signal=123% |
| 604 | GOBP\_DETECTION\_OF\_ABIOTIC\_STIMULUS |  | 65 | -0.36 | -1.37 | 0.046 | 0.619 | 1.000 | 1712 | tags=23%, list=14%, signal=27% |
| 605 | GOBP\_DETOXIFICATION |  | 75 | -0.35 | -1.37 | 0.045 | 0.620 | 1.000 | 1940 | tags=28%, list=16%, signal=33% |
| 606 | GOBP\_XYLULOSE\_5\_PHOSPHATE\_METABOLIC\_PROCESS |  | 5 | -0.69 | -1.37 | 0.114 | 0.623 | 1.000 | 2606 | tags=80%, list=22%, signal=102% |
| 607 | GOMF\_CALCIUM\_RELEASE\_CHANNEL\_ACTIVITY |  | 14 | -0.51 | -1.37 | 0.113 | 0.622 | 1.000 | 2831 | tags=43%, list=24%, signal=56% |
| 608 | GOMF\_PROTEIN\_TAG |  | 9 | -0.58 | -1.37 | 0.125 | 0.621 | 1.000 | 2605 | tags=44%, list=22%, signal=57% |
| 609 | GOBP\_PIGMENT\_BIOSYNTHETIC\_PROCESS |  | 40 | -0.39 | -1.37 | 0.065 | 0.620 | 1.000 | 3520 | tags=43%, list=29%, signal=60% |
| 610 | GOMF\_1\_PHOSPHATIDYLINOSITOL\_4\_PHOSPHATE\_5\_KINASE\_ACTIVITY |  | 6 | -0.67 | -1.37 | 0.133 | 0.620 | 1.000 | 2813 | tags=67%, list=23%, signal=87% |
| 611 | GOBP\_PML\_BODY\_ORGANIZATION |  | 4 | -0.74 | -1.37 | 0.112 | 0.620 | 1.000 | 3081 | tags=100%, list=26%, signal=134% |
| 612 | GOBP\_PRIMARY\_ALCOHOL\_CATABOLIC\_PROCESS |  | 4 | -0.74 | -1.37 | 0.102 | 0.621 | 1.000 | 3068 | tags=100%, list=26%, signal=134% |
| 613 | GOBP\_METANEPHRIC\_COLLECTING\_DUCT\_DEVELOPMENT |  | 7 | -0.62 | -1.37 | 0.128 | 0.623 | 1.000 | 1656 | tags=29%, list=14%, signal=33% |
| 614 | GOMF\_C\_X3\_C\_CHEMOKINE\_BINDING |  | 5 | -0.69 | -1.37 | 0.134 | 0.624 | 1.000 | 1133 | tags=40%, list=9%, signal=44% |
| 615 | GOMF\_SERINE\_TRANSMEMBRANE\_TRANSPORTER\_ACTIVITY |  | 6 | -0.66 | -1.37 | 0.119 | 0.627 | 1.000 | 1140 | tags=50%, list=9%, signal=55% |
| 616 | GOMF\_ICOSANOID\_RECEPTOR\_ACTIVITY |  | 9 | -0.58 | -1.37 | 0.128 | 0.627 | 1.000 | 3314 | tags=67%, list=28%, signal=92% |
| 617 | GOMF\_VOLTAGE\_GATED\_SODIUM\_CHANNEL\_ACTIVITY |  | 10 | -0.56 | -1.37 | 0.118 | 0.627 | 1.000 | 1316 | tags=40%, list=11%, signal=45% |
| 618 | GOBP\_B\_CELL\_RECEPTOR\_SIGNALING\_PATHWAY |  | 40 | -0.39 | -1.37 | 0.073 | 0.628 | 1.000 | 3454 | tags=55%, list=29%, signal=77% |
| 619 | GOBP\_REGULATION\_OF\_OXIDATIVE\_STRESS\_INDUCED\_NEURON\_DEATH |  | 14 | -0.51 | -1.36 | 0.123 | 0.630 | 1.000 | 3161 | tags=50%, list=26%, signal=68% |
| 620 | GOMF\_ORGANIC\_ACID\_TRANSMEMBRANE\_TRANSPORTER\_ACTIVITY |  | 100 | -0.32 | -1.36 | 0.033 | 0.629 | 1.000 | 1561 | tags=27%, list=13%, signal=31% |
| 621 | GOBP\_LATERAL\_VENTRICLE\_DEVELOPMENT |  | 9 | -0.57 | -1.36 | 0.133 | 0.629 | 1.000 | 920 | tags=33%, list=8%, signal=36% |
| 622 | GOCC\_CLATHRIN\_SCULPTED\_MONOAMINE\_TRANSPORT\_VESICLE |  | 5 | -0.69 | -1.36 | 0.125 | 0.629 | 1.000 | 1257 | tags=80%, list=10%, signal=89% |
| 623 | GOCC\_CLATHRIN\_SCULPTED\_GAMMA\_AMINOBUTYRIC\_ACID\_TRANSPORT\_VESICLE |  | 5 | -0.69 | -1.36 | 0.122 | 0.630 | 1.000 | 1257 | tags=60%, list=10%, signal=67% |
| 624 | GOBP\_SARCOPLASMIC\_RETICULUM\_CALCIUM\_ION\_TRANSPORT |  | 22 | -0.45 | -1.36 | 0.100 | 0.630 | 1.000 | 338 | tags=14%, list=3%, signal=14% |
| 625 | GOBP\_DICARBOXYLIC\_ACID\_TRANSPORT |  | 62 | -0.36 | -1.36 | 0.058 | 0.629 | 1.000 | 1561 | tags=24%, list=13%, signal=28% |
| 626 | GOBP\_PHOSPHATE\_ION\_TRANSMEMBRANE\_TRANSPORT |  | 13 | -0.52 | -1.36 | 0.118 | 0.629 | 1.000 | 1247 | tags=31%, list=10%, signal=34% |
| 627 | GOCC\_GUANYL\_NUCLEOTIDE\_EXCHANGE\_FACTOR\_COMPLEX |  | 12 | -0.53 | -1.36 | 0.121 | 0.632 | 1.000 | 1534 | tags=33%, list=13%, signal=38% |
| 628 | GOBP\_NEGATIVE\_REGULATION\_OF\_MEMBRANE\_PERMEABILITY |  | 5 | -0.69 | -1.36 | 0.133 | 0.631 | 1.000 | 2461 | tags=60%, list=20%, signal=75% |
| 629 | GOMF\_TETRAPYRROLE\_BINDING |  | 72 | -0.34 | -1.36 | 0.046 | 0.631 | 1.000 | 945 | tags=21%, list=8%, signal=22% |
| 630 | GOBP\_REGULATION\_OF\_SYNAPTIC\_TRANSMISSION\_CHOLINERGIC |  | 5 | -0.69 | -1.36 | 0.130 | 0.630 | 1.000 | 973 | tags=40%, list=8%, signal=44% |
| 631 | GOBP\_ORGANIC\_ACID\_METABOLIC\_PROCESS |  | 715 | -0.26 | -1.36 | 0.000 | 0.630 | 1.000 | 2710 | tags=30%, list=23%, signal=36% |
| 632 | GOBP\_TRIVALENT\_INORGANIC\_ANION\_HOMEOSTASIS |  | 4 | -0.73 | -1.36 | 0.129 | 0.632 | 1.000 | 2651 | tags=75%, list=22%, signal=96% |
| 633 | GOBP\_MITOCHONDRIAL\_TRANSMEMBRANE\_TRANSPORT |  | 69 | -0.35 | -1.36 | 0.051 | 0.632 | 1.000 | 3005 | tags=45%, list=25%, signal=60% |
| 634 | GOCC\_DIHYDROLIPOYL\_DEHYDROGENASE\_COMPLEX |  | 9 | -0.57 | -1.36 | 0.127 | 0.631 | 1.000 | 3613 | tags=78%, list=30%, signal=111% |
| 635 | GOMF\_RIBOSE\_PHOSPHATE\_DIPHOSPHOKINASE\_ACTIVITY |  | 4 | -0.73 | -1.36 | 0.128 | 0.632 | 1.000 | 2468 | tags=75%, list=21%, signal=94% |
| 636 | GOBP\_SKELETAL\_MYOFIBRIL\_ASSEMBLY |  | 5 | -0.68 | -1.36 | 0.131 | 0.631 | 1.000 | 2044 | tags=60%, list=17%, signal=72% |
| 637 | GOBP\_RESPONSE\_TO\_SYMBIONT |  | 5 | -0.69 | -1.36 | 0.130 | 0.631 | 1.000 | 3485 | tags=80%, list=29%, signal=113% |
| 638 | GOBP\_GLUTAMINE\_FAMILY\_AMINO\_ACID\_METABOLIC\_PROCESS |  | 47 | -0.37 | -1.36 | 0.059 | 0.630 | 1.000 | 3016 | tags=38%, list=25%, signal=51% |
| 639 | GOBP\_NEGATIVE\_REGULATION\_OF\_HEXOKINASE\_ACTIVITY |  | 3 | -0.80 | -1.36 | 0.108 | 0.632 | 1.000 | 1100 | tags=33%, list=9%, signal=37% |
| 640 | GOBP\_POSITIVE\_REGULATION\_OF\_ISOTYPE\_SWITCHING |  | 17 | -0.48 | -1.36 | 0.105 | 0.632 | 1.000 | 3557 | tags=65%, list=30%, signal=92% |
| 641 | GOBP\_SNO\_S\_RNA\_3\_END\_PROCESSING |  | 8 | -0.59 | -1.36 | 0.132 | 0.632 | 1.000 | 3711 | tags=63%, list=31%, signal=90% |
| 642 | GOBP\_NEGATIVE\_REGULATION\_OF\_JUN\_KINASE\_ACTIVITY |  | 10 | -0.56 | -1.36 | 0.140 | 0.634 | 1.000 | 629 | tags=20%, list=5%, signal=21% |
| 643 | GOBP\_EXTRACELLULAR\_MATRIX\_CELL\_SIGNALING |  | 5 | -0.69 | -1.36 | 0.141 | 0.635 | 1.000 | 56 | tags=20%, list=0%, signal=20% |
| 644 | GOMF\_RRNA\_CYTOSINE\_METHYLTRANSFERASE\_ACTIVITY |  | 4 | -0.74 | -1.36 | 0.120 | 0.635 | 1.000 | 2754 | tags=75%, list=23%, signal=97% |
| 645 | GOBP\_NEGATIVE\_REGULATION\_OF\_EPITHELIAL\_TO\_MESENCHYMAL\_TRANSITION |  | 25 | -0.43 | -1.36 | 0.098 | 0.635 | 1.000 | 629 | tags=16%, list=5%, signal=17% |
| 646 | GOBP\_COBALAMIN\_METABOLIC\_PROCESS |  | 13 | -0.51 | -1.36 | 0.119 | 0.634 | 1.000 | 2377 | tags=54%, list=20%, signal=67% |
| 647 | GOBP\_INORGANIC\_ION\_TRANSMEMBRANE\_TRANSPORT |  | 495 | -0.27 | -1.36 | 0.000 | 0.633 | 1.000 | 1852 | tags=21%, list=15%, signal=24% |
| 648 | GOMF\_VOLTAGE\_GATED\_CALCIUM\_CHANNEL\_ACTIVITY |  | 22 | -0.44 | -1.36 | 0.096 | 0.634 | 1.000 | 1538 | tags=32%, list=13%, signal=36% |
| 649 | GOBP\_CHONDROITIN\_SULFATE\_PROTEOGLYCAN\_METABOLIC\_PROCESS |  | 33 | -0.40 | -1.36 | 0.088 | 0.634 | 1.000 | 1824 | tags=27%, list=15%, signal=32% |
| 650 | GOCC\_ORGANELLE\_ENVELOPE\_LUMEN |  | 67 | -0.35 | -1.36 | 0.054 | 0.633 | 1.000 | 3822 | tags=46%, list=32%, signal=67% |
| 651 | GOBP\_NEURON\_CELL\_CELL\_ADHESION |  | 10 | -0.56 | -1.35 | 0.134 | 0.633 | 1.000 | 4 | tags=10%, list=0%, signal=10% |
| 652 | GOBP\_NEGATIVE\_REGULATION\_OF\_HUMORAL\_IMMUNE\_RESPONSE\_MEDIATED\_BY\_CIRCULATING\_IMMUNOGLOBULIN |  | 4 | -0.74 | -1.35 | 0.127 | 0.635 | 1.000 | 2812 | tags=75%, list=23%, signal=98% |
| 653 | GOMF\_SECONDARY\_ACTIVE\_TRANSMEMBRANE\_TRANSPORTER\_ACTIVITY |  | 149 | -0.30 | -1.35 | 0.019 | 0.634 | 1.000 | 644 | tags=15%, list=5%, signal=16% |
| 654 | GOBP\_PORPHYRIN\_CONTAINING\_COMPOUND\_METABOLIC\_PROCESS |  | 30 | -0.42 | -1.35 | 0.095 | 0.637 | 1.000 | 3737 | tags=53%, list=31%, signal=77% |
| 655 | GOBP\_REGULATION\_OF\_BLOOD\_VESSEL\_REMODELING |  | 4 | -0.73 | -1.35 | 0.118 | 0.637 | 1.000 | 2040 | tags=50%, list=17%, signal=60% |
| 656 | GOBP\_PEPTIDYL\_TYROSINE\_DEPHOSPHORYLATION\_INVOLVED\_IN\_INACTIVATION\_OF\_PROTEIN\_KINASE\_ACTIVITY |  | 6 | -0.65 | -1.35 | 0.138 | 0.638 | 1.000 | 1062 | tags=33%, list=9%, signal=37% |
| 657 | GOBP\_NEGATIVE\_REGULATION\_OF\_NATURAL\_KILLER\_CELL\_ACTIVATION |  | 4 | -0.74 | -1.35 | 0.121 | 0.637 | 1.000 | 1142 | tags=25%, list=10%, signal=28% |
| 658 | GOBP\_RIBONUCLEOSIDE\_TRIPHOSPHATE\_METABOLIC\_PROCESS |  | 47 | -0.37 | -1.35 | 0.075 | 0.637 | 1.000 | 2974 | tags=40%, list=25%, signal=54% |
| 659 | GOMF\_ANION\_TRANSMEMBRANE\_TRANSPORTER\_ACTIVITY |  | 276 | -0.28 | -1.35 | 0.003 | 0.638 | 1.000 | 1994 | tags=26%, list=17%, signal=31% |
| 660 | GOBP\_REGULATION\_OF\_ENDOCYTIC\_RECYCLING |  | 6 | -0.65 | -1.35 | 0.131 | 0.640 | 1.000 | 123 | tags=33%, list=1%, signal=34% |
| 661 | GOBP\_GLUTAMINE\_FAMILY\_AMINO\_ACID\_CATABOLIC\_PROCESS |  | 18 | -0.47 | -1.35 | 0.101 | 0.640 | 1.000 | 3016 | tags=44%, list=25%, signal=59% |
| 662 | GOBP\_POSITIVE\_REGULATION\_OF\_HEMATOPOIETIC\_STEM\_CELL\_PROLIFERATION |  | 4 | -0.74 | -1.35 | 0.135 | 0.639 | 1.000 | 1567 | tags=50%, list=13%, signal=57% |
| 663 | GOMF\_EUKARYOTIC\_INITIATION\_FACTOR\_4E\_BINDING |  | 7 | -0.62 | -1.35 | 0.135 | 0.639 | 1.000 | 3373 | tags=57%, list=28%, signal=79% |
| 664 | GOBP\_NUCLEOSIDE\_TRIPHOSPHATE\_BIOSYNTHETIC\_PROCESS |  | 42 | -0.38 | -1.35 | 0.071 | 0.638 | 1.000 | 2040 | tags=33%, list=17%, signal=40% |
| 665 | GOBP\_CALCIUM\_ION\_TRANSMEMBRANE\_TRANSPORT\_VIA\_HIGH\_VOLTAGE\_GATED\_CALCIUM\_CHANNEL |  | 10 | -0.55 | -1.35 | 0.138 | 0.638 | 1.000 | 3048 | tags=40%, list=25%, signal=54% |
| 666 | GOMF\_N\_ACETYLGLUCOSAMINE\_6\_O\_SULFOTRANSFERASE\_ACTIVITY |  | 5 | -0.68 | -1.35 | 0.140 | 0.637 | 1.000 | 951 | tags=40%, list=8%, signal=43% |
| 667 | GOCC\_PROTON\_TRANSPORTING\_TWO\_SECTOR\_ATPASE\_COMPLEX\_PROTON\_TRANSPORTING\_DOMAIN |  | 11 | -0.54 | -1.35 | 0.138 | 0.638 | 1.000 | 1849 | tags=27%, list=15%, signal=32% |
| 668 | GOBP\_REGULATION\_OF\_T\_CELL\_DIFFERENTIATION\_IN\_THYMUS |  | 18 | -0.47 | -1.35 | 0.119 | 0.638 | 1.000 | 3240 | tags=50%, list=27%, signal=68% |
| 669 | GOCC\_ORGANELLE\_INNER\_MEMBRANE |  | 390 | -0.27 | -1.35 | 0.002 | 0.639 | 1.000 | 3599 | tags=36%, list=30%, signal=50% |
| 670 | GOBP\_RESPONSE\_TO\_AMINE |  | 29 | -0.42 | -1.35 | 0.103 | 0.638 | 1.000 | 2737 | tags=48%, list=23%, signal=62% |
| 671 | GOBP\_MITOCHONDRIAL\_RESPIRATORY\_CHAIN\_COMPLEX\_ASSEMBLY |  | 79 | -0.33 | -1.35 | 0.048 | 0.638 | 1.000 | 4262 | tags=43%, list=35%, signal=66% |
| 672 | GOBP\_RESPONSE\_TO\_NERVE\_GROWTH\_FACTOR |  | 39 | -0.39 | -1.35 | 0.084 | 0.639 | 1.000 | 523 | tags=10%, list=4%, signal=11% |
| 673 | GOMF\_NAADP\_SENSITIVE\_CALCIUM\_RELEASE\_CHANNEL\_ACTIVITY |  | 4 | -0.73 | -1.35 | 0.133 | 0.639 | 1.000 | 2831 | tags=75%, list=24%, signal=98% |
| 674 | GOCC\_INTRINSIC\_COMPONENT\_OF\_THE\_CYTOPLASMIC\_SIDE\_OF\_THE\_PLASMA\_MEMBRANE |  | 4 | -0.73 | -1.35 | 0.125 | 0.638 | 1.000 | 910 | tags=50%, list=8%, signal=54% |
| 675 | GOBP\_BASE\_EXCISION\_REPAIR\_AP\_SITE\_FORMATION |  | 8 | -0.59 | -1.35 | 0.148 | 0.637 | 1.000 | 4012 | tags=75%, list=33%, signal=113% |
| 676 | GOBP\_REGULATION\_OF\_MITOCHONDRIAL\_ELECTRON\_TRANSPORT\_NADH\_TO\_UBIQUINONE |  | 4 | -0.73 | -1.35 | 0.127 | 0.637 | 1.000 | 3284 | tags=100%, list=27%, signal=138% |
| 677 | GOBP\_GLUTAMATE\_BIOSYNTHETIC\_PROCESS |  | 3 | -0.79 | -1.35 | 0.113 | 0.636 | 1.000 | 1561 | tags=67%, list=13%, signal=77% |
| 678 | GOBP\_MITOCHONDRIAL\_RNA\_METABOLIC\_PROCESS |  | 37 | -0.39 | -1.35 | 0.088 | 0.642 | 1.000 | 1978 | tags=38%, list=16%, signal=45% |
| 679 | GOBP\_NEGATIVE\_REGULATION\_OF\_VIRAL\_TRANSCRIPTION |  | 7 | -0.62 | -1.34 | 0.144 | 0.642 | 1.000 | 515 | tags=29%, list=4%, signal=30% |
| 680 | GOBP\_UV\_DAMAGE\_EXCISION\_REPAIR |  | 9 | -0.57 | -1.34 | 0.128 | 0.642 | 1.000 | 2167 | tags=33%, list=18%, signal=41% |
| 681 | GOBP\_NEGATIVE\_REGULATION\_OF\_CELLULAR\_PROTEIN\_CATABOLIC\_PROCESS |  | 56 | -0.36 | -1.34 | 0.062 | 0.642 | 1.000 | 2713 | tags=34%, list=23%, signal=44% |
| 682 | GOMF\_VOLTAGE\_GATED\_POTASSIUM\_CHANNEL\_ACTIVITY\_INVOLVED\_IN\_CARDIAC\_MUSCLE\_CELL\_ACTION\_POTENTIAL\_REPOLARIZATION |  | 8 | -0.59 | -1.34 | 0.143 | 0.647 | 1.000 | 611 | tags=25%, list=5%, signal=26% |
| 683 | GOCC\_PAM\_COMPLEX\_TIM23\_ASSOCIATED\_IMPORT\_MOTOR |  | 4 | -0.73 | -1.34 | 0.132 | 0.647 | 1.000 | 3284 | tags=100%, list=27%, signal=138% |
| 684 | GOBP\_MIDBRAIN\_DOPAMINERGIC\_NEURON\_DIFFERENTIATION |  | 12 | -0.52 | -1.34 | 0.142 | 0.647 | 1.000 | 669 | tags=25%, list=6%, signal=26% |
| 685 | GOBP\_SULFUR\_COMPOUND\_BIOSYNTHETIC\_PROCESS |  | 148 | -0.30 | -1.34 | 0.020 | 0.646 | 1.000 | 2087 | tags=24%, list=17%, signal=29% |
| 686 | GOBP\_REGULATION\_OF\_SYSTEMIC\_ARTERIAL\_BLOOD\_PRESSURE\_BY\_HORMONE |  | 25 | -0.43 | -1.34 | 0.113 | 0.645 | 1.000 | 1514 | tags=32%, list=13%, signal=37% |
| 687 | GOBP\_NEGATIVE\_REGULATION\_OF\_AXONOGENESIS |  | 49 | -0.36 | -1.34 | 0.076 | 0.646 | 1.000 | 674 | tags=16%, list=6%, signal=17% |
| 688 | GOBP\_NEGATIVE\_REGULATION\_OF\_INTRINSIC\_APOPTOTIC\_SIGNALING\_PATHWAY\_IN\_RESPONSE\_TO\_DNA\_DAMAGE |  | 19 | -0.46 | -1.34 | 0.123 | 0.645 | 1.000 | 3011 | tags=47%, list=25%, signal=63% |
| 689 | GOBP\_CELLULAR\_MODIFIED\_AMINO\_ACID\_METABOLIC\_PROCESS |  | 126 | -0.31 | -1.34 | 0.025 | 0.645 | 1.000 | 1267 | tags=20%, list=11%, signal=22% |
| 690 | GOBP\_NEGATIVE\_REGULATION\_OF\_DEVELOPMENTAL\_GROWTH |  | 72 | -0.34 | -1.34 | 0.058 | 0.644 | 1.000 | 394 | tags=13%, list=3%, signal=13% |
| 691 | GOBP\_RESPONSE\_TO\_AMYLOID\_BETA |  | 30 | -0.41 | -1.34 | 0.101 | 0.644 | 1.000 | 3323 | tags=50%, list=28%, signal=69% |
| 692 | GOBP\_RNA\_IMPORT\_INTO\_MITOCHONDRION |  | 4 | -0.73 | -1.34 | 0.136 | 0.648 | 1.000 | 1321 | tags=50%, list=11%, signal=56% |
| 693 | GOCC\_CHROMAFFIN\_GRANULE |  | 8 | -0.59 | -1.34 | 0.146 | 0.648 | 1.000 | 1005 | tags=38%, list=8%, signal=41% |
| 694 | GOBP\_ALCOHOL\_CATABOLIC\_PROCESS |  | 38 | -0.39 | -1.34 | 0.089 | 0.647 | 1.000 | 2014 | tags=29%, list=17%, signal=35% |
| 695 | GOBP\_RESPONSE\_TO\_LEUCINE |  | 11 | -0.53 | -1.34 | 0.137 | 0.652 | 1.000 | 2417 | tags=45%, list=20%, signal=57% |
| 696 | GOMF\_NEUROTRANSMITTER\_RECEPTOR\_ACTIVITY |  | 30 | -0.41 | -1.34 | 0.098 | 0.653 | 1.000 | 1289 | tags=33%, list=11%, signal=37% |
| 697 | GOBP\_WOUND\_HEALING\_INVOLVED\_IN\_INFLAMMATORY\_RESPONSE |  | 4 | -0.72 | -1.34 | 0.148 | 0.654 | 1.000 | 2040 | tags=50%, list=17%, signal=60% |
| 698 | GOMF\_CYTOCHROME\_B5\_REDUCTASE\_ACTIVITY\_ACTING\_ON\_NAD\_P\_H |  | 4 | -0.72 | -1.34 | 0.135 | 0.654 | 1.000 | 2720 | tags=75%, list=23%, signal=97% |
| 699 | GOBP\_MITOCHONDRIAL\_DNA\_METABOLIC\_PROCESS |  | 11 | -0.53 | -1.34 | 0.140 | 0.653 | 1.000 | 878 | tags=27%, list=7%, signal=29% |
| 700 | GOCC\_CRD\_MEDIATED\_MRNA\_STABILITY\_COMPLEX |  | 5 | -0.68 | -1.34 | 0.152 | 0.654 | 1.000 | 3030 | tags=60%, list=25%, signal=80% |
| 701 | GOBP\_MATURATION\_OF\_SSU\_RRNA |  | 39 | -0.38 | -1.34 | 0.081 | 0.653 | 1.000 | 4068 | tags=59%, list=34%, signal=89% |
| 702 | GOCC\_PERIKARYON |  | 88 | -0.33 | -1.33 | 0.049 | 0.658 | 1.000 | 1286 | tags=18%, list=11%, signal=20% |
| 703 | GOMF\_CATION\_CHANNEL\_ACTIVITY |  | 168 | -0.29 | -1.33 | 0.024 | 0.658 | 1.000 | 1716 | tags=25%, list=14%, signal=29% |
| 704 | GOBP\_DRUG\_METABOLIC\_PROCESS |  | 19 | -0.46 | -1.33 | 0.116 | 0.657 | 1.000 | 1347 | tags=32%, list=11%, signal=36% |
| 705 | GOBP\_DOPAMINE\_SECRETION |  | 21 | -0.45 | -1.33 | 0.122 | 0.656 | 1.000 | 2135 | tags=57%, list=18%, signal=69% |
| 706 | GOBP\_RNA\_5\_END\_PROCESSING |  | 15 | -0.49 | -1.33 | 0.129 | 0.656 | 1.000 | 3935 | tags=47%, list=33%, signal=69% |
| 707 | GOMF\_OXIDOREDUCTASE\_ACTIVITY\_ACTING\_ON\_NAD\_P\_H\_HEME\_PROTEIN\_AS\_ACCEPTOR |  | 7 | -0.61 | -1.33 | 0.150 | 0.655 | 1.000 | 3873 | tags=71%, list=32%, signal=105% |
| 708 | GOMF\_OXIDOREDUCTASE\_ACTIVITY\_ACTING\_ON\_THE\_CH\_NH\_GROUP\_OF\_DONORS |  | 19 | -0.45 | -1.33 | 0.121 | 0.655 | 1.000 | 1109 | tags=21%, list=9%, signal=23% |
| 709 | GOBP\_CELLULAR\_MODIFIED\_AMINO\_ACID\_BIOSYNTHETIC\_PROCESS |  | 35 | -0.39 | -1.33 | 0.088 | 0.654 | 1.000 | 581 | tags=20%, list=5%, signal=21% |
| 710 | GOCC\_VACUOLAR\_LUMEN |  | 117 | -0.31 | -1.33 | 0.033 | 0.653 | 1.000 | 3273 | tags=42%, list=27%, signal=57% |
| 711 | GOBP\_DENDRITE\_EXTENSION |  | 25 | -0.43 | -1.33 | 0.112 | 0.653 | 1.000 | 2839 | tags=48%, list=24%, signal=63% |
| 712 | GOMF\_CALCIUM\_DEPENDENT\_PROTEIN\_SERINE\_THREONINE\_PHOSPHATASE\_REGULATOR\_ACTIVITY |  | 4 | -0.72 | -1.33 | 0.144 | 0.659 | 1.000 | 3266 | tags=75%, list=27%, signal=103% |
| 713 | GOBP\_POSITIVE\_REGULATION\_OF\_DOPAMINE\_SECRETION |  | 4 | -0.72 | -1.33 | 0.147 | 0.658 | 1.000 | 2560 | tags=75%, list=21%, signal=95% |
| 714 | GOBP\_APPENDAGE\_MORPHOGENESIS |  | 83 | -0.33 | -1.33 | 0.056 | 0.660 | 1.000 | 2452 | tags=34%, list=20%, signal=42% |
| 715 | GOCC\_RIBOSE\_PHOSPHATE\_DIPHOSPHOKINASE\_COMPLEX |  | 4 | -0.73 | -1.33 | 0.151 | 0.660 | 1.000 | 2468 | tags=75%, list=21%, signal=94% |
| 716 | GOBP\_ADULT\_BEHAVIOR |  | 65 | -0.34 | -1.33 | 0.071 | 0.660 | 1.000 | 1289 | tags=22%, list=11%, signal=24% |
| 717 | GOBP\_RESPONSE\_TO\_AMPHETAMINE |  | 21 | -0.44 | -1.33 | 0.121 | 0.659 | 1.000 | 2737 | tags=52%, list=23%, signal=68% |
| 718 | GOBP\_LOCOMOTORY\_EXPLORATION\_BEHAVIOR |  | 6 | -0.64 | -1.33 | 0.155 | 0.659 | 1.000 | 969 | tags=50%, list=8%, signal=54% |
| 719 | GOBP\_TRANSFERRIN\_TRANSPORT |  | 27 | -0.41 | -1.33 | 0.107 | 0.659 | 1.000 | 3769 | tags=44%, list=31%, signal=65% |
| 720 | GOBP\_LIPOXIN\_METABOLIC\_PROCESS |  | 4 | -0.72 | -1.33 | 0.143 | 0.658 | 1.000 | 1558 | tags=50%, list=13%, signal=57% |
| 721 | GOMF\_G\_PROTEIN\_ALPHA\_SUBUNIT\_BINDING |  | 18 | -0.46 | -1.33 | 0.126 | 0.659 | 1.000 | 1655 | tags=33%, list=14%, signal=39% |
| 722 | GOMF\_VOLUME\_SENSITIVE\_ANION\_CHANNEL\_ACTIVITY |  | 6 | -0.64 | -1.33 | 0.153 | 0.659 | 1.000 | 1649 | tags=33%, list=14%, signal=39% |
| 723 | GOBP\_NEURAL\_PLATE\_DEVELOPMENT |  | 4 | -0.73 | -1.33 | 0.143 | 0.664 | 1.000 | 677 | tags=25%, list=6%, signal=26% |
| 724 | GOBP\_TRNA\_5\_END\_PROCESSING |  | 11 | -0.52 | -1.33 | 0.142 | 0.664 | 1.000 | 4530 | tags=64%, list=38%, signal=102% |
| 725 | GOBP\_SERINE\_FAMILY\_AMINO\_ACID\_BIOSYNTHETIC\_PROCESS |  | 11 | -0.53 | -1.33 | 0.147 | 0.666 | 1.000 | 1595 | tags=36%, list=13%, signal=42% |
| 726 | GOBP\_POSITIVE\_REGULATION\_OF\_CD8\_POSITIVE\_ALPHA\_BETA\_T\_CELL\_DIFFERENTIATION |  | 4 | -0.71 | -1.32 | 0.155 | 0.671 | 1.000 | 2760 | tags=75%, list=23%, signal=97% |
| 727 | GOBP\_REGULATION\_OF\_SKELETAL\_MUSCLE\_ACETYLCHOLINE\_GATED\_CHANNEL\_CLUSTERING |  | 4 | -0.72 | -1.32 | 0.140 | 0.670 | 1.000 | 2789 | tags=75%, list=23%, signal=98% |
| 728 | GOBP\_REGULATION\_OF\_INTRINSIC\_APOPTOTIC\_SIGNALING\_PATHWAY\_IN\_RESPONSE\_TO\_DNA\_DAMAGE\_BY\_P53\_CLASS\_MEDIATOR |  | 10 | -0.54 | -1.32 | 0.152 | 0.670 | 1.000 | 4671 | tags=90%, list=39%, signal=147% |
| 729 | GOBP\_OXYGEN\_TRANSPORT |  | 5 | -0.67 | -1.32 | 0.158 | 0.671 | 1.000 | 502 | tags=40%, list=4%, signal=42% |
| 730 | GOMF\_METALLOCHAPERONE\_ACTIVITY |  | 7 | -0.60 | -1.32 | 0.156 | 0.674 | 1.000 | 3508 | tags=71%, list=29%, signal=101% |
| 731 | GOCC\_ANCHORED\_COMPONENT\_OF\_POSTSYNAPTIC\_MEMBRANE |  | 5 | -0.67 | -1.32 | 0.152 | 0.675 | 1.000 | 2158 | tags=60%, list=18%, signal=73% |
| 732 | GOBP\_NERVOUS\_SYSTEM\_PROCESS\_INVOLVED\_IN\_REGULATION\_OF\_SYSTEMIC\_ARTERIAL\_BLOOD\_PRESSURE |  | 5 | -0.67 | -1.32 | 0.162 | 0.675 | 1.000 | 523 | tags=40%, list=4%, signal=42% |
| 733 | GOCC\_PHOSPHORYLASE\_KINASE\_COMPLEX |  | 4 | -0.72 | -1.32 | 0.153 | 0.675 | 1.000 | 1029 | tags=25%, list=9%, signal=27% |
| 734 | GOBP\_GLUTATHIONE\_METABOLIC\_PROCESS |  | 40 | -0.37 | -1.32 | 0.094 | 0.675 | 1.000 | 1267 | tags=23%, list=11%, signal=25% |
| 735 | GOBP\_REGULATION\_OF\_VENTRICULAR\_CARDIAC\_MUSCLE\_CELL\_MEMBRANE\_REPOLARIZATION |  | 14 | -0.49 | -1.32 | 0.136 | 0.674 | 1.000 | 728 | tags=21%, list=6%, signal=23% |
| 736 | GOBP\_GLYCOSYL\_COMPOUND\_METABOLIC\_PROCESS |  | 92 | -0.32 | -1.32 | 0.051 | 0.675 | 1.000 | 2825 | tags=40%, list=24%, signal=52% |
| 737 | GOMF\_PROSTAGLANDIN\_E\_RECEPTOR\_ACTIVITY |  | 4 | -0.72 | -1.32 | 0.154 | 0.675 | 1.000 | 3297 | tags=75%, list=27%, signal=103% |
| 738 | GOBP\_FATTY\_ACID\_BETA\_OXIDATION\_USING\_ACYL\_COA\_DEHYDROGENASE |  | 8 | -0.57 | -1.32 | 0.163 | 0.677 | 1.000 | 1697 | tags=50%, list=14%, signal=58% |
| 739 | GOBP\_RELAXATION\_OF\_SMOOTH\_MUSCLE |  | 6 | -0.63 | -1.32 | 0.169 | 0.682 | 1.000 | 2737 | tags=50%, list=23%, signal=65% |
| 740 | GOMF\_PROTEIN\_TYROSINE\_THREONINE\_PHOSPHATASE\_ACTIVITY |  | 8 | -0.58 | -1.32 | 0.161 | 0.682 | 1.000 | 826 | tags=25%, list=7%, signal=27% |
| 741 | GOBP\_DUCTUS\_ARTERIOSUS\_CLOSURE |  | 3 | -0.77 | -1.32 | 0.142 | 0.684 | 1.000 | 1641 | tags=67%, list=14%, signal=77% |
| 742 | GOMF\_INSULIN\_LIKE\_GROWTH\_FACTOR\_II\_BINDING |  | 7 | -0.60 | -1.32 | 0.164 | 0.684 | 1.000 | 3491 | tags=71%, list=29%, signal=101% |
| 743 | GOMF\_ESTRADIOL\_17\_BETA\_DEHYDROGENASE\_ACTIVITY |  | 7 | -0.60 | -1.32 | 0.159 | 0.683 | 1.000 | 1455 | tags=57%, list=12%, signal=65% |
| 744 | GOBP\_PROTEIN\_DEMETHYLATION |  | 20 | -0.44 | -1.32 | 0.131 | 0.685 | 1.000 | 3231 | tags=40%, list=27%, signal=55% |
| 745 | GOCC\_OXIDOREDUCTASE\_COMPLEX |  | 82 | -0.33 | -1.32 | 0.061 | 0.684 | 1.000 | 4262 | tags=50%, list=35%, signal=77% |
| 746 | GOCC\_VOLTAGE\_GATED\_CALCIUM\_CHANNEL\_COMPLEX |  | 19 | -0.45 | -1.32 | 0.139 | 0.684 | 1.000 | 2248 | tags=37%, list=19%, signal=45% |
| 747 | GOCC\_ADA2\_GCN5\_ADA3\_TRANSCRIPTION\_ACTIVATOR\_COMPLEX |  | 13 | -0.50 | -1.32 | 0.143 | 0.683 | 1.000 | 1428 | tags=31%, list=12%, signal=35% |
| 748 | GOBP\_ETHER\_BIOSYNTHETIC\_PROCESS |  | 9 | -0.56 | -1.31 | 0.163 | 0.683 | 1.000 | 1719 | tags=33%, list=14%, signal=39% |
| 749 | GOBP\_ODONTOGENESIS\_OF\_DENTIN\_CONTAINING\_TOOTH |  | 56 | -0.35 | -1.31 | 0.083 | 0.683 | 1.000 | 1842 | tags=34%, list=15%, signal=40% |
| 750 | GOBP\_CRD\_MEDIATED\_MRNA\_STABILIZATION |  | 4 | -0.71 | -1.31 | 0.161 | 0.683 | 1.000 | 3030 | tags=75%, list=25%, signal=100% |
| 751 | GOBP\_SNRNA\_MODIFICATION |  | 6 | -0.63 | -1.31 | 0.159 | 0.683 | 1.000 | 1678 | tags=50%, list=14%, signal=58% |
| 752 | GOBP\_EXTRACELLULAR\_MATRIX\_DISASSEMBLY |  | 48 | -0.36 | -1.31 | 0.088 | 0.682 | 1.000 | 3240 | tags=40%, list=27%, signal=54% |
| 753 | GOBP\_MULTICELLULAR\_ORGANISMAL\_IRON\_ION\_HOMEOSTASIS |  | 6 | -0.63 | -1.31 | 0.161 | 0.683 | 1.000 | 1852 | tags=50%, list=15%, signal=59% |
| 754 | GOBP\_SYNAPTIC\_VESICLE\_MATURATION |  | 11 | -0.52 | -1.31 | 0.156 | 0.684 | 1.000 | 1711 | tags=45%, list=14%, signal=53% |
| 755 | GOBP\_REGULATION\_OF\_BRANCHING\_INVOLVED\_IN\_PROSTATE\_GLAND\_MORPHOGENESIS |  | 4 | -0.72 | -1.31 | 0.167 | 0.685 | 1.000 | 350 | tags=50%, list=3%, signal=51% |
| 756 | GOMF\_LIGASE\_ACTIVITY\_FORMING\_CARBON\_OXYGEN\_BONDS |  | 20 | -0.44 | -1.31 | 0.129 | 0.684 | 1.000 | 3334 | tags=60%, list=28%, signal=83% |
| 757 | GOBP\_REGULATION\_OF\_DELAYED\_RECTIFIER\_POTASSIUM\_CHANNEL\_ACTIVITY |  | 11 | -0.52 | -1.31 | 0.155 | 0.686 | 1.000 | 1002 | tags=27%, list=8%, signal=30% |
| 758 | GOMF\_MYOSIN\_LIGHT\_CHAIN\_BINDING |  | 5 | -0.66 | -1.31 | 0.184 | 0.687 | 1.000 | 1389 | tags=40%, list=12%, signal=45% |
| 759 | GOCC\_MITOCHONDRIAL\_ALPHA\_KETOGLUTARATE\_DEHYDROGENASE\_COMPLEX |  | 4 | -0.71 | -1.31 | 0.158 | 0.687 | 1.000 | 3471 | tags=100%, list=29%, signal=141% |
| 760 | GOMF\_SELENIUM\_BINDING |  | 5 | -0.67 | -1.31 | 0.170 | 0.688 | 1.000 | 636 | tags=40%, list=5%, signal=42% |
| 761 | GOBP\_UBIQUINONE\_METABOLIC\_PROCESS |  | 16 | -0.47 | -1.31 | 0.152 | 0.688 | 1.000 | 2886 | tags=38%, list=24%, signal=49% |
| 762 | GOCC\_PROTEIN\_ACETYLTRANSFERASE\_COMPLEX |  | 80 | -0.32 | -1.31 | 0.060 | 0.690 | 1.000 | 3869 | tags=36%, list=32%, signal=53% |
| 763 | GOBP\_L\_SERINE\_TRANSPORT |  | 4 | -0.70 | -1.31 | 0.161 | 0.691 | 1.000 | 909 | tags=50%, list=8%, signal=54% |
| 764 | GOBP\_REGULATION\_OF\_RETROGRADE\_TRANSPORT\_ENDOSOME\_TO\_GOLGI |  | 3 | -0.77 | -1.31 | 0.146 | 0.691 | 1.000 | 1945 | tags=67%, list=16%, signal=80% |
| 765 | GOBP\_POSITIVE\_REGULATION\_OF\_OSTEOCLAST\_DEVELOPMENT |  | 3 | -0.78 | -1.31 | 0.146 | 0.691 | 1.000 | 2611 | tags=100%, list=22%, signal=128% |
| 766 | GOBP\_SEMAPHORIN\_PLEXIN\_SIGNALING\_PATHWAY\_INVOLVED\_IN\_AXON\_GUIDANCE |  | 8 | -0.58 | -1.31 | 0.161 | 0.693 | 1.000 | 2290 | tags=38%, list=19%, signal=46% |
| 767 | GOBP\_IRON\_IMPORT\_INTO\_CELL |  | 5 | -0.66 | -1.31 | 0.179 | 0.693 | 1.000 | 3520 | tags=80%, list=29%, signal=113% |
| 768 | GOMF\_HEXOSAMINIDASE\_ACTIVITY |  | 9 | -0.56 | -1.31 | 0.172 | 0.696 | 1.000 | 1699 | tags=44%, list=14%, signal=52% |
| 769 | GOBP\_REGULATION\_OF\_ER\_ASSOCIATED\_UBIQUITIN\_DEPENDENT\_PROTEIN\_CATABOLIC\_PROCESS |  | 8 | -0.57 | -1.30 | 0.182 | 0.700 | 1.000 | 1747 | tags=38%, list=15%, signal=44% |
| 770 | GOBP\_NEUROTRANSMITTER\_METABOLIC\_PROCESS |  | 16 | -0.47 | -1.30 | 0.155 | 0.699 | 1.000 | 2570 | tags=44%, list=21%, signal=56% |
| 771 | GOMF\_L\_SERINE\_TRANSMEMBRANE\_TRANSPORTER\_ACTIVITY |  | 4 | -0.70 | -1.30 | 0.165 | 0.699 | 1.000 | 909 | tags=50%, list=8%, signal=54% |
| 772 | GOBP\_MEMBRANE\_REPOLARIZATION\_DURING\_VENTRICULAR\_CARDIAC\_MUSCLE\_CELL\_ACTION\_POTENTIAL |  | 8 | -0.58 | -1.30 | 0.171 | 0.701 | 1.000 | 13 | tags=13%, list=0%, signal=13% |
| 773 | GOBP\_SODIUM\_DEPENDENT\_PHOSPHATE\_TRANSPORT |  | 4 | -0.71 | -1.30 | 0.169 | 0.704 | 1.000 | 3407 | tags=75%, list=28%, signal=105% |
| 774 | GOMF\_NUCLEOBASE\_BINDING |  | 4 | -0.70 | -1.30 | 0.174 | 0.704 | 1.000 | 1892 | tags=50%, list=16%, signal=59% |
| 775 | GOBP\_DEPYRIMIDINATION |  | 6 | -0.62 | -1.30 | 0.174 | 0.708 | 1.000 | 4012 | tags=83%, list=33%, signal=125% |
| 776 | GOBP\_CHORIO\_ALLANTOIC\_FUSION |  | 4 | -0.71 | -1.30 | 0.174 | 0.709 | 1.000 | 1380 | tags=50%, list=11%, signal=56% |
| 777 | GOBP\_CELLULAR\_MONOVALENT\_INORGANIC\_CATION\_HOMEOSTASIS |  | 86 | -0.32 | -1.30 | 0.063 | 0.708 | 1.000 | 1403 | tags=22%, list=12%, signal=25% |
| 778 | GOBP\_REGULATION\_OF\_NAD\_P\_H\_OXIDASE\_ACTIVITY |  | 7 | -0.60 | -1.30 | 0.175 | 0.707 | 1.000 | 3691 | tags=71%, list=31%, signal=103% |
| 779 | GOBP\_RESPONSE\_TO\_WATER\_DEPRIVATION |  | 3 | -0.77 | -1.30 | 0.150 | 0.707 | 1.000 | 44 | tags=33%, list=0%, signal=33% |
| 780 | GOBP\_GAMMA\_AMINOBUTYRIC\_ACID\_SIGNALING\_PATHWAY |  | 6 | -0.62 | -1.30 | 0.185 | 0.708 | 1.000 | 3912 | tags=67%, list=33%, signal=99% |
| 781 | GOCC\_NUCLEAR\_EXOSOME\_RNASE\_COMPLEX |  | 14 | -0.48 | -1.30 | 0.157 | 0.708 | 1.000 | 3711 | tags=50%, list=31%, signal=72% |
| 782 | GOMF\_CARBOHYDRATE\_PROTON\_SYMPORTER\_ACTIVITY |  | 5 | -0.66 | -1.30 | 0.180 | 0.707 | 1.000 | 1252 | tags=60%, list=10%, signal=67% |
| 783 | GOBP\_AEROBIC\_RESPIRATION |  | 64 | -0.34 | -1.30 | 0.082 | 0.708 | 1.000 | 4286 | tags=48%, list=36%, signal=75% |
| 784 | GOMF\_CATION\_TRANSMEMBRANE\_TRANSPORTER\_ACTIVITY |  | 356 | -0.26 | -1.30 | 0.009 | 0.708 | 1.000 | 1948 | tags=26%, list=16%, signal=30% |
| 785 | GOCC\_PRERIBOSOME\_SMALL\_SUBUNIT\_PRECURSOR |  | 13 | -0.50 | -1.30 | 0.158 | 0.710 | 1.000 | 2945 | tags=62%, list=25%, signal=81% |
| 786 | GOBP\_POSITIVE\_REGULATION\_OF\_CELL\_PROLIFERATION\_INVOLVED\_IN\_KIDNEY\_DEVELOPMENT |  | 6 | -0.62 | -1.30 | 0.181 | 0.715 | 1.000 | 1051 | tags=33%, list=9%, signal=37% |
| 787 | GOBP\_NEGATIVE\_REGULATION\_OF\_EXTRACELLULAR\_MATRIX\_DISASSEMBLY |  | 4 | -0.71 | -1.30 | 0.174 | 0.714 | 1.000 | 2040 | tags=75%, list=17%, signal=90% |
| 788 | GOMF\_MRNA\_5\_UTR\_BINDING |  | 22 | -0.43 | -1.30 | 0.145 | 0.715 | 1.000 | 2059 | tags=36%, list=17%, signal=44% |
| 789 | GOBP\_INHIBITORY\_SYNAPSE\_ASSEMBLY |  | 7 | -0.59 | -1.30 | 0.183 | 0.716 | 1.000 | 527 | tags=43%, list=4%, signal=45% |
| 790 | GOBP\_NEGATIVE\_REGULATION\_OF\_SYSTEMIC\_ARTERIAL\_BLOOD\_PRESSURE |  | 8 | -0.57 | -1.29 | 0.173 | 0.716 | 1.000 | 597 | tags=38%, list=5%, signal=39% |
| 791 | GOBP\_CIRCADIAN\_SLEEP\_WAKE\_CYCLE\_REM\_SLEEP |  | 3 | -0.76 | -1.29 | 0.158 | 0.715 | 1.000 | 1245 | tags=67%, list=10%, signal=74% |
| 792 | GOBP\_TRANSCRIPTION\_ELONGATION\_FROM\_RNA\_POLYMERASE\_II\_PROMOTER |  | 72 | -0.33 | -1.29 | 0.082 | 0.716 | 1.000 | 3741 | tags=32%, list=31%, signal=46% |
| 793 | GOMF\_ANTIPORTER\_ACTIVITY |  | 58 | -0.34 | -1.29 | 0.082 | 0.715 | 1.000 | 644 | tags=14%, list=5%, signal=15% |
| 794 | GOMF\_VOLTAGE\_GATED\_POTASSIUM\_CHANNEL\_ACTIVITY\_INVOLVED\_IN\_VENTRICULAR\_CARDIAC\_MUSCLE\_CELL\_ACTION\_POTENTIAL\_REPOLARIZATION |  | 6 | -0.63 | -1.29 | 0.182 | 0.714 | 1.000 | 13 | tags=17%, list=0%, signal=17% |
| 795 | GOMF\_STRUCTURAL\_MOLECULE\_ACTIVITY |  | 423 | -0.26 | -1.29 | 0.004 | 0.714 | 1.000 | 1488 | tags=20%, list=12%, signal=22% |
| 796 | GOMF\_CARBONATE\_DEHYDRATASE\_ACTIVITY |  | 9 | -0.55 | -1.29 | 0.160 | 0.714 | 1.000 | 1061 | tags=33%, list=9%, signal=37% |
| 797 | GOBP\_NUCLEOTIDE\_BINDING\_OLIGOMERIZATION\_DOMAIN\_CONTAINING\_1\_SIGNALING\_PATHWAY |  | 6 | -0.61 | -1.29 | 0.187 | 0.714 | 1.000 | 4370 | tags=67%, list=36%, signal=105% |
| 798 | GOBP\_PEPTIDYL\_HISTIDINE\_MODIFICATION |  | 11 | -0.52 | -1.29 | 0.174 | 0.715 | 1.000 | 3707 | tags=64%, list=31%, signal=92% |
| 799 | GOBP\_MITOCHONDRIAL\_ACETYL\_COA\_BIOSYNTHETIC\_PROCESS\_FROM\_PYRUVATE |  | 4 | -0.70 | -1.29 | 0.172 | 0.716 | 1.000 | 3386 | tags=75%, list=28%, signal=104% |
| 800 | GOBP\_AMINOGLYCOSIDE\_ANTIBIOTIC\_METABOLIC\_PROCESS |  | 5 | -0.66 | -1.29 | 0.181 | 0.715 | 1.000 | 3725 | tags=80%, list=31%, signal=116% |
| 801 | GOBP\_NETRIN\_ACTIVATED\_SIGNALING\_PATHWAY |  | 9 | -0.55 | -1.29 | 0.183 | 0.714 | 1.000 | 1104 | tags=44%, list=9%, signal=49% |
| 802 | GOBP\_ESTABLISHMENT\_OF\_TISSUE\_POLARITY |  | 89 | -0.32 | -1.29 | 0.074 | 0.714 | 1.000 | 2288 | tags=19%, list=19%, signal=23% |
| 803 | GOBP\_PROTEIN\_LIPOYLATION |  | 5 | -0.65 | -1.29 | 0.187 | 0.713 | 1.000 | 3382 | tags=60%, list=28%, signal=83% |
| 804 | GOMF\_ACTIVE\_ION\_TRANSMEMBRANE\_TRANSPORTER\_ACTIVITY |  | 127 | -0.30 | -1.29 | 0.049 | 0.713 | 1.000 | 1321 | tags=22%, list=11%, signal=25% |
| 805 | GOBP\_CELLULAR\_ION\_HOMEOSTASIS |  | 420 | -0.26 | -1.29 | 0.008 | 0.713 | 1.000 | 1571 | tags=21%, list=13%, signal=23% |
| 806 | GOBP\_REGULATION\_OF\_STORE\_OPERATED\_CALCIUM\_CHANNEL\_ACTIVITY |  | 4 | -0.70 | -1.29 | 0.180 | 0.713 | 1.000 | 1378 | tags=50%, list=11%, signal=56% |
| 807 | GOBP\_PROTEIN\_O\_LINKED\_FUCOSYLATION |  | 4 | -0.70 | -1.29 | 0.174 | 0.713 | 1.000 | 3379 | tags=50%, list=28%, signal=70% |
| 808 | GOBP\_DETOXIFICATION\_OF\_INORGANIC\_COMPOUND |  | 4 | -0.70 | -1.29 | 0.180 | 0.712 | 1.000 | 109 | tags=50%, list=1%, signal=50% |
| 809 | GOMF\_POTASSIUM\_CHANNEL\_ACTIVITY |  | 59 | -0.34 | -1.29 | 0.099 | 0.713 | 1.000 | 1689 | tags=29%, list=14%, signal=33% |
| 810 | GOBP\_POSITIVE\_REGULATION\_OF\_MAST\_CELL\_ACTIVATION\_INVOLVED\_IN\_IMMUNE\_RESPONSE |  | 8 | -0.57 | -1.29 | 0.185 | 0.714 | 1.000 | 4293 | tags=75%, list=36%, signal=117% |
| 811 | GOBP\_ACTIVATION\_OF\_ADENYLATE\_CYCLASE\_ACTIVITY |  | 23 | -0.42 | -1.29 | 0.137 | 0.715 | 1.000 | 1289 | tags=26%, list=11%, signal=29% |
| 812 | GOBP\_REGULATION\_OF\_SOMITOGENESIS |  | 6 | -0.61 | -1.29 | 0.190 | 0.715 | 1.000 | 2176 | tags=50%, list=18%, signal=61% |
| 813 | GOMF\_CULLIN\_FAMILY\_PROTEIN\_BINDING |  | 19 | -0.45 | -1.29 | 0.150 | 0.716 | 1.000 | 1959 | tags=32%, list=16%, signal=38% |
| 814 | GOCC\_ANCHORED\_COMPONENT\_OF\_PLASMA\_MEMBRANE |  | 29 | -0.40 | -1.29 | 0.133 | 0.716 | 1.000 | 2366 | tags=38%, list=20%, signal=47% |
| 815 | GOBP\_POSITIVE\_REGULATION\_OF\_AXONOGENESIS |  | 58 | -0.34 | -1.29 | 0.099 | 0.716 | 1.000 | 2560 | tags=33%, list=21%, signal=41% |
| 816 | GOBP\_REGULATION\_OF\_PH |  | 79 | -0.32 | -1.29 | 0.074 | 0.717 | 1.000 | 1403 | tags=23%, list=12%, signal=26% |
| 817 | GOBP\_REDUCTION\_OF\_FOOD\_INTAKE\_IN\_RESPONSE\_TO\_DIETARY\_EXCESS |  | 3 | -0.76 | -1.29 | 0.170 | 0.718 | 1.000 | 2928 | tags=100%, list=24%, signal=132% |
| 818 | GOCC\_TRNA\_METHYLTRANSFERASE\_COMPLEX |  | 4 | -0.70 | -1.29 | 0.189 | 0.718 | 1.000 | 590 | tags=25%, list=5%, signal=26% |
| 819 | GOCC\_SYNAPTIC\_VESICLE\_MEMBRANE |  | 62 | -0.34 | -1.29 | 0.086 | 0.718 | 1.000 | 1447 | tags=26%, list=12%, signal=29% |
| 820 | GOBP\_PURKINJE\_MYOCYTE\_TO\_VENTRICULAR\_CARDIAC\_MUSCLE\_CELL\_SIGNALING |  | 3 | -0.76 | -1.29 | 0.162 | 0.719 | 1.000 | 713 | tags=67%, list=6%, signal=71% |
| 821 | GOBP\_NEGATIVE\_REGULATION\_OF\_PHAGOCYTOSIS |  | 14 | -0.48 | -1.29 | 0.160 | 0.720 | 1.000 | 2055 | tags=29%, list=17%, signal=34% |
| 822 | GOBP\_RRNA\_MODIFICATION |  | 25 | -0.41 | -1.29 | 0.143 | 0.719 | 1.000 | 3180 | tags=36%, list=26%, signal=49% |
| 823 | GOCC\_NADPH\_OXIDASE\_COMPLEX |  | 8 | -0.56 | -1.28 | 0.188 | 0.722 | 1.000 | 1292 | tags=50%, list=11%, signal=56% |
| 824 | GOCC\_LYSOSOMAL\_LUMEN |  | 73 | -0.32 | -1.28 | 0.082 | 0.722 | 1.000 | 3273 | tags=44%, list=27%, signal=60% |
| 825 | GOCC\_ANCHORED\_COMPONENT\_OF\_MEMBRANE |  | 78 | -0.32 | -1.28 | 0.079 | 0.724 | 1.000 | 1622 | tags=26%, list=14%, signal=29% |
| 826 | GOBP\_FRUCTOSE\_CATABOLIC\_PROCESS\_TO\_HYDROXYACETONE\_PHOSPHATE\_AND\_GLYCERALDEHYDE\_3\_PHOSPHATE |  | 5 | -0.66 | -1.28 | 0.184 | 0.723 | 1.000 | 57 | tags=20%, list=0%, signal=20% |
| 827 | GOBP\_HORMONE\_METABOLIC\_PROCESS |  | 126 | -0.30 | -1.28 | 0.055 | 0.724 | 1.000 | 1510 | tags=26%, list=13%, signal=30% |
| 828 | GOBP\_SEMAPHORIN\_PLEXIN\_SIGNALING\_PATHWAY\_INVOLVED\_IN\_NEURON\_PROJECTION\_GUIDANCE |  | 10 | -0.52 | -1.28 | 0.186 | 0.724 | 1.000 | 2290 | tags=40%, list=19%, signal=49% |
| 829 | GOMF\_MAP\_KINASE\_PHOSPHATASE\_ACTIVITY |  | 10 | -0.52 | -1.28 | 0.173 | 0.724 | 1.000 | 826 | tags=20%, list=7%, signal=21% |
| 830 | GOBP\_HISTONE\_H3\_K14\_ACETYLATION |  | 12 | -0.50 | -1.28 | 0.166 | 0.724 | 1.000 | 2144 | tags=50%, list=18%, signal=61% |
| 831 | GOCC\_NSL\_COMPLEX |  | 7 | -0.58 | -1.28 | 0.198 | 0.724 | 1.000 | 4923 | tags=86%, list=41%, signal=145% |
| 832 | GOMF\_RNA\_POLYMERASE\_ACTIVITY |  | 30 | -0.39 | -1.28 | 0.139 | 0.725 | 1.000 | 3230 | tags=40%, list=27%, signal=55% |
| 833 | GOBP\_ENDOPLASMIC\_RETICULUM\_CALCIUM\_ION\_HOMEOSTASIS |  | 20 | -0.43 | -1.28 | 0.163 | 0.725 | 1.000 | 3422 | tags=50%, list=28%, signal=70% |
| 834 | GOBP\_EMBRYONIC\_VISCEROCRANIUM\_MORPHOGENESIS |  | 5 | -0.65 | -1.28 | 0.195 | 0.725 | 1.000 | 1999 | tags=60%, list=17%, signal=72% |
| 835 | GOCC\_AP\_3\_ADAPTOR\_COMPLEX |  | 9 | -0.54 | -1.28 | 0.185 | 0.725 | 1.000 | 2899 | tags=67%, list=24%, signal=88% |
| 836 | GOMF\_SULFOTRANSFERASE\_ACTIVITY |  | 29 | -0.39 | -1.28 | 0.135 | 0.724 | 1.000 | 2014 | tags=41%, list=17%, signal=50% |
| 837 | GOBP\_REGULATION\_OF\_B\_CELL\_MEDIATED\_IMMUNITY |  | 32 | -0.38 | -1.28 | 0.138 | 0.727 | 1.000 | 3209 | tags=53%, list=27%, signal=72% |
| 838 | GOBP\_NEURAL\_PLATE\_MORPHOGENESIS |  | 3 | -0.76 | -1.28 | 0.172 | 0.726 | 1.000 | 677 | tags=33%, list=6%, signal=35% |
| 839 | GOBP\_RENAL\_VESICLE\_FORMATION |  | 7 | -0.58 | -1.28 | 0.205 | 0.728 | 1.000 | 3475 | tags=57%, list=29%, signal=80% |
| 840 | GOCC\_PYRUVATE\_DEHYDROGENASE\_COMPLEX |  | 6 | -0.61 | -1.28 | 0.204 | 0.733 | 1.000 | 4007 | tags=67%, list=33%, signal=100% |
| 841 | GOMF\_INORGANIC\_PHOSPHATE\_TRANSMEMBRANE\_TRANSPORTER\_ACTIVITY |  | 4 | -0.70 | -1.28 | 0.190 | 0.732 | 1.000 | 2888 | tags=50%, list=24%, signal=66% |
| 842 | GOBP\_MICROGLIA\_DIFFERENTIATION |  | 4 | -0.69 | -1.28 | 0.202 | 0.735 | 1.000 | 2211 | tags=50%, list=18%, signal=61% |
| 843 | GOBP\_REGULATION\_OF\_OXIDATIVE\_STRESS\_INDUCED\_INTRINSIC\_APOPTOTIC\_SIGNALING\_PATHWAY |  | 23 | -0.42 | -1.28 | 0.149 | 0.735 | 1.000 | 2625 | tags=39%, list=22%, signal=50% |
| 844 | GOCC\_BOX\_C\_D\_RNP\_COMPLEX |  | 4 | -0.69 | -1.28 | 0.205 | 0.735 | 1.000 | 2464 | tags=75%, list=21%, signal=94% |
| 845 | GOCC\_PHAGOLYSOSOME |  | 5 | -0.64 | -1.28 | 0.189 | 0.734 | 1.000 | 1292 | tags=60%, list=11%, signal=67% |
| 846 | GOBP\_POSITIVE\_REGULATION\_OF\_GLIAL\_CELL\_DIFFERENTIATION |  | 30 | -0.39 | -1.28 | 0.144 | 0.734 | 1.000 | 2881 | tags=43%, list=24%, signal=57% |
| 847 | GOBP\_NEGATIVE\_REGULATION\_OF\_BONE\_RESORPTION |  | 10 | -0.52 | -1.27 | 0.197 | 0.737 | 1.000 | 2046 | tags=60%, list=17%, signal=72% |
| 848 | GOBP\_REGULATION\_OF\_INFLAMMATORY\_RESPONSE\_TO\_ANTIGENIC\_STIMULUS |  | 12 | -0.49 | -1.27 | 0.175 | 0.740 | 1.000 | 3035 | tags=67%, list=25%, signal=89% |
| 849 | GOBP\_FATTY\_ACID\_DERIVATIVE\_CATABOLIC\_PROCESS |  | 7 | -0.58 | -1.27 | 0.196 | 0.740 | 1.000 | 4736 | tags=86%, list=39%, signal=141% |
| 850 | GOBP\_RIBOSOMAL\_SMALL\_SUBUNIT\_ASSEMBLY |  | 15 | -0.47 | -1.27 | 0.164 | 0.739 | 1.000 | 3089 | tags=60%, list=26%, signal=81% |
| 851 | GOBP\_NEGATIVE\_REGULATION\_OF\_DENDRITIC\_SPINE\_DEVELOPMENT |  | 8 | -0.56 | -1.27 | 0.196 | 0.739 | 1.000 | 2842 | tags=50%, list=24%, signal=65% |
| 852 | GOBP\_SENSORY\_PERCEPTION |  | 234 | -0.27 | -1.27 | 0.028 | 0.738 | 1.000 | 2587 | tags=30%, list=22%, signal=38% |
| 853 | GOBP\_MALATE\_METABOLIC\_PROCESS |  | 6 | -0.61 | -1.27 | 0.207 | 0.741 | 1.000 | 928 | tags=33%, list=8%, signal=36% |
| 854 | GOBP\_ARGININE\_METABOLIC\_PROCESS |  | 11 | -0.51 | -1.27 | 0.185 | 0.742 | 1.000 | 2936 | tags=45%, list=24%, signal=60% |
| 855 | GOMF\_INTRACILIARY\_TRANSPORT\_PARTICLE\_B\_BINDING |  | 4 | -0.69 | -1.27 | 0.202 | 0.741 | 1.000 | 3536 | tags=75%, list=29%, signal=106% |
| 856 | GOBP\_DETECTION\_OF\_STIMULUS\_INVOLVED\_IN\_SENSORY\_PERCEPTION |  | 36 | -0.37 | -1.27 | 0.132 | 0.741 | 1.000 | 1712 | tags=31%, list=14%, signal=36% |
| 857 | GOBP\_ATRIAL\_CARDIAC\_MUSCLE\_CELL\_MEMBRANE\_REPOLARIZATION |  | 6 | -0.61 | -1.27 | 0.199 | 0.740 | 1.000 | 713 | tags=33%, list=6%, signal=35% |
| 858 | GOBP\_REGULATION\_OF\_ODONTOGENESIS\_OF\_DENTIN\_CONTAINING\_TOOTH |  | 5 | -0.65 | -1.27 | 0.209 | 0.740 | 1.000 | 156 | tags=40%, list=1%, signal=41% |
| 859 | GOMF\_C2H2\_ZINC\_FINGER\_DOMAIN\_BINDING |  | 8 | -0.55 | -1.27 | 0.197 | 0.739 | 1.000 | 3674 | tags=63%, list=31%, signal=90% |
| 860 | GOBP\_NEPHRIC\_DUCT\_DEVELOPMENT |  | 9 | -0.54 | -1.27 | 0.188 | 0.740 | 1.000 | 27 | tags=11%, list=0%, signal=11% |
| 861 | GOMF\_TRANSCRIPTION\_REGULATOR\_INHIBITOR\_ACTIVITY |  | 14 | -0.48 | -1.27 | 0.183 | 0.741 | 1.000 | 1070 | tags=21%, list=9%, signal=23% |
| 862 | GOBP\_CELL\_MIGRATION\_INVOLVED\_IN\_HEART\_DEVELOPMENT |  | 14 | -0.47 | -1.27 | 0.181 | 0.740 | 1.000 | 563 | tags=29%, list=5%, signal=30% |
| 863 | GOBP\_OXYGEN\_METABOLIC\_PROCESS |  | 5 | -0.64 | -1.27 | 0.203 | 0.740 | 1.000 | 77 | tags=20%, list=1%, signal=20% |
| 864 | GOBP\_LIPID\_OXIDATION |  | 88 | -0.31 | -1.27 | 0.085 | 0.740 | 1.000 | 1786 | tags=27%, list=15%, signal=32% |
| 865 | GOBP\_POSITIVE\_REGULATION\_OF\_T\_CELL\_DIFFERENTIATION\_IN\_THYMUS |  | 9 | -0.54 | -1.27 | 0.197 | 0.740 | 1.000 | 3240 | tags=44%, list=27%, signal=61% |
| 866 | GOBP\_REGULATION\_OF\_CELL\_PROLIFERATION\_INVOLVED\_IN\_KIDNEY\_DEVELOPMENT |  | 10 | -0.52 | -1.27 | 0.190 | 0.740 | 1.000 | 1051 | tags=30%, list=9%, signal=33% |
| 867 | GOBP\_C\_TERMINAL\_PROTEIN\_LIPIDATION |  | 4 | -0.69 | -1.27 | 0.204 | 0.741 | 1.000 | 1642 | tags=25%, list=14%, signal=29% |
| 868 | GOBP\_HYPERSENSITIVITY |  | 6 | -0.60 | -1.27 | 0.213 | 0.741 | 1.000 | 1063 | tags=50%, list=9%, signal=55% |
| 869 | GOCC\_POTASSIUM\_CHANNEL\_COMPLEX |  | 50 | -0.35 | -1.27 | 0.112 | 0.741 | 1.000 | 2114 | tags=32%, list=18%, signal=39% |
| 870 | GOBP\_OSTEOBLAST\_DIFFERENTIATION |  | 149 | -0.29 | -1.27 | 0.060 | 0.740 | 1.000 | 1529 | tags=19%, list=13%, signal=22% |
| 871 | GOBP\_LEUKOCYTE\_AGGREGATION |  | 10 | -0.52 | -1.27 | 0.200 | 0.740 | 1.000 | 1650 | tags=40%, list=14%, signal=46% |
| 872 | GOBP\_SUCCINATE\_METABOLIC\_PROCESS |  | 5 | -0.64 | -1.27 | 0.208 | 0.739 | 1.000 | 2570 | tags=60%, list=21%, signal=76% |
| 873 | GOBP\_POSITIVE\_REGULATION\_OF\_VOLTAGE\_GATED\_POTASSIUM\_CHANNEL\_ACTIVITY |  | 7 | -0.58 | -1.27 | 0.202 | 0.739 | 1.000 | 894 | tags=29%, list=7%, signal=31% |
| 874 | GOMF\_TRIPEPTIDE\_TRANSMEMBRANE\_TRANSPORTER\_ACTIVITY |  | 4 | -0.69 | -1.27 | 0.201 | 0.739 | 1.000 | 3785 | tags=100%, list=32%, signal=146% |
| 875 | GOBP\_REGULATION\_OF\_RELAXATION\_OF\_MUSCLE |  | 7 | -0.58 | -1.27 | 0.207 | 0.739 | 1.000 | 3048 | tags=57%, list=25%, signal=77% |
| 876 | GOMF\_TRNA\_BINDING |  | 49 | -0.35 | -1.27 | 0.114 | 0.739 | 1.000 | 3270 | tags=45%, list=27%, signal=61% |
| 877 | GOCC\_MRNA\_EDITING\_COMPLEX |  | 7 | -0.58 | -1.27 | 0.205 | 0.739 | 1.000 | 4272 | tags=57%, list=36%, signal=89% |
| 878 | GOMF\_PROTEIN\_DEMETHYLASE\_ACTIVITY |  | 17 | -0.45 | -1.27 | 0.179 | 0.739 | 1.000 | 3813 | tags=41%, list=32%, signal=60% |
| 879 | GOCC\_ANCHORED\_COMPONENT\_OF\_EXTERNAL\_SIDE\_OF\_PLASMA\_MEMBRANE |  | 8 | -0.55 | -1.27 | 0.199 | 0.738 | 1.000 | 2938 | tags=63%, list=24%, signal=83% |
| 880 | GOBP\_POSITIVE\_REGULATION\_OF\_HYPERSENSITIVITY |  | 3 | -0.75 | -1.27 | 0.194 | 0.739 | 1.000 | 3035 | tags=100%, list=25%, signal=134% |
| 881 | GOBP\_MYELOID\_PROGENITOR\_CELL\_DIFFERENTIATION |  | 4 | -0.69 | -1.27 | 0.204 | 0.739 | 1.000 | 3160 | tags=75%, list=26%, signal=102% |
| 882 | GOCC\_EXTERNAL\_SIDE\_OF\_APICAL\_PLASMA\_MEMBRANE |  | 5 | -0.64 | -1.27 | 0.211 | 0.739 | 1.000 | 1902 | tags=40%, list=16%, signal=48% |
| 883 | GOMF\_IMMUNE\_RECEPTOR\_ACTIVITY |  | 77 | -0.32 | -1.27 | 0.091 | 0.739 | 1.000 | 1546 | tags=23%, list=13%, signal=27% |
| 884 | GOBP\_MEMBRANE\_HYPERPOLARIZATION |  | 10 | -0.52 | -1.26 | 0.196 | 0.740 | 1.000 | 604 | tags=30%, list=5%, signal=32% |
| 885 | GOBP\_AMINO\_ACID\_ACTIVATION |  | 27 | -0.40 | -1.26 | 0.158 | 0.740 | 1.000 | 3334 | tags=52%, list=28%, signal=72% |
| 886 | GOBP\_POSITIVE\_REGULATION\_OF\_RENAL\_SODIUM\_EXCRETION |  | 8 | -0.55 | -1.26 | 0.203 | 0.741 | 1.000 | 12 | tags=13%, list=0%, signal=13% |
| 887 | GOBP\_CONVERGENT\_EXTENSION\_INVOLVED\_IN\_GASTRULATION |  | 6 | -0.60 | -1.26 | 0.205 | 0.741 | 1.000 | 677 | tags=33%, list=6%, signal=35% |
| 888 | GOBP\_TRIPEPTIDE\_TRANSMEMBRANE\_TRANSPORT |  | 4 | -0.69 | -1.26 | 0.207 | 0.741 | 1.000 | 3785 | tags=100%, list=32%, signal=146% |
| 889 | GOMF\_NAD\_P\_H\_DEHYDROGENASE\_QUINONE\_ACTIVITY |  | 38 | -0.37 | -1.26 | 0.135 | 0.742 | 1.000 | 4520 | tags=50%, list=38%, signal=80% |
| 890 | GOBP\_POSITIVE\_REGULATION\_OF\_INFLAMMATORY\_RESPONSE\_TO\_ANTIGENIC\_STIMULUS |  | 7 | -0.58 | -1.26 | 0.204 | 0.743 | 1.000 | 3035 | tags=86%, list=25%, signal=115% |
| 891 | GOBP\_NEGATIVE\_REGULATION\_OF\_FIBROBLAST\_PROLIFERATION |  | 18 | -0.44 | -1.26 | 0.177 | 0.742 | 1.000 | 819 | tags=22%, list=7%, signal=24% |
| 892 | GOMF\_C\_C\_CHEMOKINE\_BINDING |  | 12 | -0.49 | -1.26 | 0.187 | 0.742 | 1.000 | 1063 | tags=42%, list=9%, signal=46% |
| 893 | GOBP\_MITOCHONDRIAL\_RESPIRASOME\_ASSEMBLY |  | 4 | -0.69 | -1.26 | 0.212 | 0.742 | 1.000 | 2749 | tags=75%, list=23%, signal=97% |
| 894 | GOBP\_VALINE\_METABOLIC\_PROCESS |  | 6 | -0.60 | -1.26 | 0.217 | 0.741 | 1.000 | 2270 | tags=50%, list=19%, signal=62% |
| 895 | GOCC\_STAGA\_COMPLEX |  | 12 | -0.49 | -1.26 | 0.184 | 0.741 | 1.000 | 4607 | tags=67%, list=38%, signal=108% |
| 896 | GOBP\_VENTRICULAR\_CARDIAC\_MUSCLE\_CELL\_DEVELOPMENT |  | 6 | -0.61 | -1.26 | 0.217 | 0.741 | 1.000 | 2773 | tags=50%, list=23%, signal=65% |
| 897 | GOMF\_OUTWARD\_RECTIFIER\_POTASSIUM\_CHANNEL\_ACTIVITY |  | 7 | -0.57 | -1.26 | 0.209 | 0.741 | 1.000 | 1943 | tags=57%, list=16%, signal=68% |
| 898 | GOBP\_G\_PROTEIN\_COUPLED\_RECEPTOR\_INTERNALIZATION |  | 8 | -0.56 | -1.26 | 0.204 | 0.740 | 1.000 | 947 | tags=50%, list=8%, signal=54% |
| 899 | GOMF\_CHEMOKINE\_BINDING |  | 18 | -0.43 | -1.26 | 0.171 | 0.742 | 1.000 | 1133 | tags=33%, list=9%, signal=37% |
| 900 | GOBP\_NEGATIVE\_REGULATION\_OF\_COLLAGEN\_METABOLIC\_PROCESS |  | 7 | -0.57 | -1.26 | 0.210 | 0.742 | 1.000 | 980 | tags=29%, list=8%, signal=31% |
| 901 | GOMF\_DEMETHYLASE\_ACTIVITY |  | 25 | -0.40 | -1.26 | 0.159 | 0.743 | 1.000 | 2402 | tags=40%, list=20%, signal=50% |
| 902 | GOBP\_AXIS\_ELONGATION\_INVOLVED\_IN\_SOMITOGENESIS |  | 4 | -0.68 | -1.26 | 0.211 | 0.743 | 1.000 | 1391 | tags=50%, list=12%, signal=57% |
| 903 | GOBP\_MONOCYTE\_AGGREGATION |  | 5 | -0.64 | -1.26 | 0.219 | 0.743 | 1.000 | 979 | tags=40%, list=8%, signal=44% |
| 904 | GOBP\_POSITIVE\_REGULATION\_OF\_PLATELET\_DERIVED\_GROWTH\_FACTOR\_RECEPTOR\_SIGNALING\_PATHWAY |  | 5 | -0.64 | -1.26 | 0.206 | 0.742 | 1.000 | 18 | tags=20%, list=0%, signal=20% |
| 905 | GOBP\_RESPONSE\_TO\_FUNGICIDE |  | 4 | -0.68 | -1.26 | 0.213 | 0.742 | 1.000 | 3813 | tags=100%, list=32%, signal=146% |
| 906 | GOBP\_PHAGOSOME\_ACIDIFICATION |  | 21 | -0.42 | -1.26 | 0.170 | 0.742 | 1.000 | 3769 | tags=38%, list=31%, signal=55% |
| 907 | GOBP\_NEGATIVE\_REGULATION\_OF\_CAMP\_MEDIATED\_SIGNALING |  | 11 | -0.50 | -1.26 | 0.198 | 0.742 | 1.000 | 2737 | tags=45%, list=23%, signal=59% |
| 908 | GOCC\_NUCLEAR\_PORE\_NUCLEAR\_BASKET |  | 9 | -0.53 | -1.26 | 0.199 | 0.742 | 1.000 | 88 | tags=11%, list=1%, signal=11% |
| 909 | GOBP\_NEGATIVE\_REGULATION\_OF\_NEURON\_DEATH |  | 117 | -0.29 | -1.26 | 0.077 | 0.742 | 1.000 | 2881 | tags=34%, list=24%, signal=45% |
| 910 | GOBP\_RETINAL\_ROD\_CELL\_DIFFERENTIATION |  | 8 | -0.55 | -1.26 | 0.212 | 0.742 | 1.000 | 2789 | tags=50%, list=23%, signal=65% |
| 911 | GOBP\_NEGATIVE\_REGULATION\_OF\_TRANSCRIPTION\_BY\_COMPETITIVE\_PROMOTER\_BINDING |  | 8 | -0.56 | -1.26 | 0.208 | 0.743 | 1.000 | 1116 | tags=25%, list=9%, signal=28% |
| 912 | GOMF\_PROTEIN\_DISULFIDE\_OXIDOREDUCTASE\_ACTIVITY |  | 19 | -0.43 | -1.26 | 0.174 | 0.744 | 1.000 | 3540 | tags=53%, list=29%, signal=75% |
| 913 | GOBP\_NEGATIVE\_REGULATION\_OF\_GROWTH |  | 163 | -0.28 | -1.26 | 0.049 | 0.744 | 1.000 | 2056 | tags=24%, list=17%, signal=28% |
| 914 | GOMF\_PHOSPHOLIPASE\_A2\_ACTIVITY\_CONSUMING\_1\_2\_DIPALMITOYLPHOSPHATIDYLCHOLINE |  | 9 | -0.53 | -1.26 | 0.209 | 0.746 | 1.000 | 808 | tags=44%, list=7%, signal=48% |
| 915 | GOBP\_INTESTINAL\_EPITHELIAL\_CELL\_DEVELOPMENT |  | 12 | -0.49 | -1.26 | 0.192 | 0.746 | 1.000 | 1418 | tags=33%, list=12%, signal=38% |
| 916 | GOMF\_NUCLEOTIDASE\_ACTIVITY |  | 11 | -0.50 | -1.26 | 0.200 | 0.746 | 1.000 | 863 | tags=36%, list=7%, signal=39% |
| 917 | GOMF\_CLASS\_I\_DNA\_APURINIC\_OR\_APYRIMIDINIC\_SITE\_ENDONUCLEASE\_ACTIVITY |  | 5 | -0.63 | -1.26 | 0.217 | 0.746 | 1.000 | 1616 | tags=60%, list=13%, signal=69% |
| 918 | GOBP\_REGULATION\_OF\_EXCRETION |  | 10 | -0.52 | -1.26 | 0.201 | 0.745 | 1.000 | 12 | tags=10%, list=0%, signal=10% |
| 919 | GOBP\_REGULATION\_OF\_SKELETAL\_MUSCLE\_CONTRACTION |  | 4 | -0.68 | -1.25 | 0.213 | 0.747 | 1.000 | 26 | tags=25%, list=0%, signal=25% |
| 920 | GOBP\_HYDROGEN\_PEROXIDE\_CATABOLIC\_PROCESS |  | 13 | -0.48 | -1.25 | 0.201 | 0.746 | 1.000 | 1188 | tags=31%, list=10%, signal=34% |
| 921 | GOBP\_TUBULIN\_COMPLEX\_ASSEMBLY |  | 7 | -0.58 | -1.25 | 0.219 | 0.747 | 1.000 | 3344 | tags=57%, list=28%, signal=79% |
| 922 | GOBP\_DETECTION\_OF\_STIMULUS |  | 139 | -0.28 | -1.25 | 0.062 | 0.747 | 1.000 | 2625 | tags=28%, list=22%, signal=35% |
| 923 | GOBP\_REGULATION\_OF\_GENE\_EXPRESSION\_BY\_GENETIC\_IMPRINTING |  | 11 | -0.50 | -1.25 | 0.195 | 0.746 | 1.000 | 13 | tags=9%, list=0%, signal=9% |
| 924 | GOBP\_PURINE\_DEOXYRIBONUCLEOTIDE\_BIOSYNTHETIC\_PROCESS |  | 4 | -0.68 | -1.25 | 0.213 | 0.745 | 1.000 | 1302 | tags=50%, list=11%, signal=56% |
| 925 | GOCC\_PHAGOCYTIC\_CUP |  | 19 | -0.43 | -1.25 | 0.180 | 0.745 | 1.000 | 2527 | tags=37%, list=21%, signal=47% |
| 926 | GOBP\_NUCLEOTIDE\_BINDING\_OLIGOMERIZATION\_DOMAIN\_CONTAINING\_2\_SIGNALING\_PATHWAY |  | 9 | -0.54 | -1.25 | 0.209 | 0.744 | 1.000 | 2403 | tags=33%, list=20%, signal=42% |
| 927 | GOMF\_TRANSLATION\_INITIATION\_FACTOR\_BINDING |  | 24 | -0.40 | -1.25 | 0.163 | 0.744 | 1.000 | 2512 | tags=42%, list=21%, signal=53% |
| 928 | GOBP\_NEGATIVE\_REGULATION\_OF\_HUMORAL\_IMMUNE\_RESPONSE |  | 7 | -0.57 | -1.25 | 0.205 | 0.743 | 1.000 | 2949 | tags=71%, list=25%, signal=95% |
| 929 | GOBP\_REGULATION\_OF\_RIBONUCLEOPROTEIN\_COMPLEX\_LOCALIZATION |  | 6 | -0.60 | -1.25 | 0.221 | 0.744 | 1.000 | 4798 | tags=100%, list=40%, signal=166% |
| 930 | GOBP\_POSITIVE\_REGULATION\_BY\_HOST\_OF\_VIRAL\_PROCESS |  | 11 | -0.50 | -1.25 | 0.199 | 0.746 | 1.000 | 1531 | tags=36%, list=13%, signal=42% |
| 931 | GOMF\_VASOPRESSIN\_RECEPTOR\_ACTIVITY |  | 4 | -0.69 | -1.25 | 0.217 | 0.745 | 1.000 | 1514 | tags=50%, list=13%, signal=57% |
| 932 | GOMF\_CYTOKINE\_RECEPTOR\_ACTIVITY |  | 71 | -0.32 | -1.25 | 0.106 | 0.745 | 1.000 | 1546 | tags=23%, list=13%, signal=26% |
| 933 | GOBP\_TERPENOID\_CATABOLIC\_PROCESS |  | 3 | -0.73 | -1.25 | 0.202 | 0.746 | 1.000 | 42 | tags=33%, list=0%, signal=33% |
| 934 | GOBP\_CLATHRIN\_COAT\_DISASSEMBLY |  | 4 | -0.68 | -1.25 | 0.218 | 0.746 | 1.000 | 160 | tags=25%, list=1%, signal=25% |
| 935 | GOBP\_SYNAPSE\_PRUNING |  | 9 | -0.53 | -1.25 | 0.218 | 0.746 | 1.000 | 3937 | tags=78%, list=33%, signal=116% |
| 936 | GOCC\_CYTOPLASMIC\_EXOSOME\_RNASE\_COMPLEX |  | 13 | -0.48 | -1.25 | 0.187 | 0.746 | 1.000 | 3711 | tags=62%, list=31%, signal=89% |
| 937 | GOBP\_HISTONE\_H4\_K16\_ACETYLATION |  | 16 | -0.45 | -1.25 | 0.188 | 0.746 | 1.000 | 4465 | tags=63%, list=37%, signal=99% |
| 938 | GOBP\_CELL\_CELL\_ADHESION\_MEDIATED\_BY\_INTEGRIN |  | 15 | -0.46 | -1.25 | 0.200 | 0.746 | 1.000 | 1133 | tags=33%, list=9%, signal=37% |
| 939 | GOMF\_APOLIPOPROTEIN\_A\_I\_BINDING |  | 5 | -0.64 | -1.25 | 0.224 | 0.746 | 1.000 | 2337 | tags=40%, list=19%, signal=50% |
| 940 | GOBP\_HISTONE\_H4\_K12\_ACETYLATION |  | 7 | -0.57 | -1.25 | 0.229 | 0.746 | 1.000 | 1584 | tags=57%, list=13%, signal=66% |
| 941 | GOBP\_RIBOSOMAL\_SMALL\_SUBUNIT\_EXPORT\_FROM\_NUCLEUS |  | 7 | -0.58 | -1.25 | 0.217 | 0.746 | 1.000 | 791 | tags=29%, list=7%, signal=31% |
| 942 | GOBP\_MITOCHONDRIAL\_CYTOCHROME\_C\_OXIDASE\_ASSEMBLY |  | 15 | -0.46 | -1.25 | 0.188 | 0.746 | 1.000 | 3562 | tags=53%, list=30%, signal=76% |
| 943 | GOBP\_RESPONSE\_TO\_COPPER\_ION |  | 18 | -0.44 | -1.25 | 0.186 | 0.745 | 1.000 | 2938 | tags=61%, list=24%, signal=81% |
| 944 | GOBP\_PROTEIN\_NEDDYLATION |  | 17 | -0.44 | -1.25 | 0.186 | 0.745 | 1.000 | 2605 | tags=41%, list=22%, signal=53% |
| 945 | GOBP\_CELLULAR\_RESPONSE\_TO\_COPPER\_ION |  | 11 | -0.49 | -1.25 | 0.199 | 0.745 | 1.000 | 771 | tags=36%, list=6%, signal=39% |
| 946 | GOBP\_EXPLORATION\_BEHAVIOR |  | 11 | -0.50 | -1.25 | 0.204 | 0.745 | 1.000 | 969 | tags=27%, list=8%, signal=30% |
| 947 | GOBP\_CYTOCHROME\_COMPLEX\_ASSEMBLY |  | 25 | -0.40 | -1.25 | 0.159 | 0.746 | 1.000 | 3562 | tags=44%, list=30%, signal=62% |
| 948 | GOBP\_U4\_SNRNA\_3\_END\_PROCESSING |  | 7 | -0.57 | -1.25 | 0.219 | 0.745 | 1.000 | 3711 | tags=71%, list=31%, signal=103% |
| 949 | GOMF\_AMINE\_BINDING |  | 6 | -0.60 | -1.25 | 0.207 | 0.745 | 1.000 | 947 | tags=50%, list=8%, signal=54% |
| 950 | GOCC\_PROTON\_TRANSPORTING\_V\_TYPE\_ATPASE\_V0\_DOMAIN |  | 10 | -0.52 | -1.25 | 0.201 | 0.745 | 1.000 | 1264 | tags=20%, list=11%, signal=22% |
| 951 | GOBP\_VENTRICULAR\_CARDIAC\_MUSCLE\_CELL\_MEMBRANE\_REPOLARIZATION |  | 16 | -0.45 | -1.25 | 0.196 | 0.744 | 1.000 | 728 | tags=19%, list=6%, signal=20% |
| 952 | GOBP\_REGULATION\_OF\_PROTEIN\_NEDDYLATION |  | 10 | -0.51 | -1.25 | 0.201 | 0.744 | 1.000 | 1950 | tags=50%, list=16%, signal=60% |
| 953 | GOBP\_ANTIMICROBIAL\_HUMORAL\_IMMUNE\_RESPONSE\_MEDIATED\_BY\_ANTIMICROBIAL\_PEPTIDE |  | 11 | -0.50 | -1.25 | 0.204 | 0.743 | 1.000 | 2949 | tags=55%, list=25%, signal=72% |
| 954 | GOBP\_POSITIVE\_REGULATION\_OF\_CYTOPLASMIC\_TRANSLATION |  | 8 | -0.55 | -1.25 | 0.217 | 0.744 | 1.000 | 2352 | tags=38%, list=20%, signal=47% |
| 955 | GOBP\_PEPTIDYL\_LYSINE\_ACETYLATION |  | 131 | -0.29 | -1.25 | 0.069 | 0.744 | 1.000 | 2541 | tags=27%, list=21%, signal=34% |
| 956 | GOBP\_CIRCADIAN\_SLEEP\_WAKE\_CYCLE |  | 12 | -0.49 | -1.25 | 0.213 | 0.745 | 1.000 | 1769 | tags=42%, list=15%, signal=49% |
| 957 | GOBP\_PURINE\_NUCLEOTIDE\_TRANSPORT |  | 18 | -0.44 | -1.25 | 0.169 | 0.744 | 1.000 | 2911 | tags=50%, list=24%, signal=66% |
| 958 | GOMF\_PHOSPHATIDYLINOSITOL\_3\_4\_5\_TRISPHOSPHATE\_3\_PHOSPHATASE\_ACTIVITY |  | 4 | -0.68 | -1.25 | 0.221 | 0.745 | 1.000 | 2046 | tags=50%, list=17%, signal=60% |
| 959 | GOBP\_POSITIVE\_REGULATION\_OF\_NERVOUS\_SYSTEM\_DEVELOPMENT |  | 183 | -0.27 | -1.25 | 0.059 | 0.744 | 1.000 | 2881 | tags=31%, list=24%, signal=40% |
| 960 | GOBP\_NUCLEAR\_RETENTION\_OF\_PRE\_MRNA\_AT\_THE\_SITE\_OF\_TRANSCRIPTION |  | 5 | -0.63 | -1.25 | 0.222 | 0.744 | 1.000 | 3082 | tags=40%, list=26%, signal=54% |
| 961 | GOBP\_NEPHRIC\_DUCT\_MORPHOGENESIS |  | 7 | -0.57 | -1.25 | 0.223 | 0.744 | 1.000 | 27 | tags=14%, list=0%, signal=14% |
| 962 | GOBP\_ADAPTATION\_OF\_SIGNALING\_PATHWAY |  | 11 | -0.49 | -1.25 | 0.208 | 0.743 | 1.000 | 947 | tags=36%, list=8%, signal=39% |
| 963 | GOBP\_PROTEIN\_TRIMERIZATION |  | 12 | -0.48 | -1.25 | 0.211 | 0.743 | 1.000 | 2203 | tags=33%, list=18%, signal=41% |
| 964 | GOBP\_POLY\_A\_PLUS\_MRNA\_EXPORT\_FROM\_NUCLEUS |  | 14 | -0.47 | -1.25 | 0.208 | 0.743 | 1.000 | 827 | tags=21%, list=7%, signal=23% |
| 965 | GOBP\_RESPONSE\_TO\_THYROID\_HORMONE |  | 16 | -0.44 | -1.25 | 0.196 | 0.742 | 1.000 | 3654 | tags=44%, list=30%, signal=63% |
| 966 | GOBP\_RRNA\_3\_END\_PROCESSING |  | 10 | -0.51 | -1.24 | 0.208 | 0.743 | 1.000 | 1360 | tags=30%, list=11%, signal=34% |
| 967 | GOMF\_ANNEALING\_ACTIVITY |  | 11 | -0.50 | -1.24 | 0.207 | 0.748 | 1.000 | 1175 | tags=36%, list=10%, signal=40% |
| 968 | GOBP\_DE\_NOVO\_IMP\_BIOSYNTHETIC\_PROCESS |  | 5 | -0.62 | -1.24 | 0.227 | 0.748 | 1.000 | 255 | tags=20%, list=2%, signal=20% |
| 969 | GOMF\_G\_PROTEIN\_COUPLED\_CHEMOATTRACTANT\_RECEPTOR\_ACTIVITY |  | 14 | -0.47 | -1.24 | 0.196 | 0.748 | 1.000 | 1472 | tags=43%, list=12%, signal=49% |
| 970 | GOBP\_DELAMINATION |  | 3 | -0.74 | -1.24 | 0.209 | 0.747 | 1.000 | 3169 | tags=100%, list=26%, signal=136% |
| 971 | GOMF\_SODIUM\_CHANNEL\_ACTIVITY |  | 22 | -0.41 | -1.24 | 0.175 | 0.747 | 1.000 | 1691 | tags=27%, list=14%, signal=32% |
| 972 | GOBP\_REGULATION\_OF\_GROWTH\_RATE |  | 4 | -0.67 | -1.24 | 0.237 | 0.747 | 1.000 | 3624 | tags=75%, list=30%, signal=107% |
| 973 | GOMF\_OXIDOREDUCTASE\_ACTIVITY\_ACTING\_ON\_A\_SULFUR\_GROUP\_OF\_DONORS\_NAD\_P\_AS\_ACCEPTOR |  | 9 | -0.52 | -1.24 | 0.217 | 0.746 | 1.000 | 3324 | tags=56%, list=28%, signal=77% |
| 974 | GOMF\_PEPTIDE\_ALPHA\_N\_ACETYLTRANSFERASE\_ACTIVITY |  | 7 | -0.57 | -1.24 | 0.218 | 0.746 | 1.000 | 3420 | tags=57%, list=28%, signal=80% |
| 975 | GOBP\_INNER\_MITOCHONDRIAL\_MEMBRANE\_ORGANIZATION |  | 31 | -0.38 | -1.24 | 0.159 | 0.745 | 1.000 | 3110 | tags=42%, list=26%, signal=56% |
| 976 | GOBP\_OLFACTORY\_BULB\_INTERNEURON\_DEVELOPMENT |  | 4 | -0.67 | -1.24 | 0.229 | 0.744 | 1.000 | 1489 | tags=50%, list=12%, signal=57% |
| 977 | GOBP\_REGULATION\_OF\_ENDOSOME\_TO\_PLASMA\_MEMBRANE\_PROTEIN\_TRANSPORT |  | 3 | -0.73 | -1.24 | 0.214 | 0.744 | 1.000 | 194 | tags=33%, list=2%, signal=34% |
| 978 | GOMF\_OXIDOREDUCTASE\_ACTIVITY\_ACTING\_ON\_A\_SULFUR\_GROUP\_OF\_DONORS\_OXYGEN\_AS\_ACCEPTOR |  | 10 | -0.51 | -1.24 | 0.206 | 0.744 | 1.000 | 582 | tags=30%, list=5%, signal=32% |
| 979 | GOBP\_MAST\_CELL\_ACTIVATION |  | 40 | -0.35 | -1.24 | 0.147 | 0.745 | 1.000 | 4293 | tags=65%, list=36%, signal=101% |
| 980 | GOBP\_REGULATION\_OF\_ISOTYPE\_SWITCHING |  | 23 | -0.41 | -1.24 | 0.175 | 0.744 | 1.000 | 3557 | tags=57%, list=30%, signal=80% |
| 981 | GOBP\_REGULATION\_OF\_EXTENT\_OF\_CELL\_GROWTH |  | 73 | -0.31 | -1.24 | 0.106 | 0.744 | 1.000 | 261 | tags=10%, list=2%, signal=10% |
| 982 | GOBP\_PERIPHERAL\_NERVOUS\_SYSTEM\_DEVELOPMENT |  | 55 | -0.33 | -1.24 | 0.128 | 0.747 | 1.000 | 563 | tags=11%, list=5%, signal=11% |
| 983 | GOBP\_REGULATION\_OF\_RNA\_BINDING |  | 9 | -0.52 | -1.24 | 0.215 | 0.746 | 1.000 | 1645 | tags=33%, list=14%, signal=39% |
| 984 | GOBP\_REGULATION\_OF\_HISTONE\_H4\_ACETYLATION |  | 11 | -0.50 | -1.24 | 0.208 | 0.746 | 1.000 | 3394 | tags=64%, list=28%, signal=89% |
| 985 | GOMF\_STRUCTURAL\_CONSTITUENT\_OF\_EYE\_LENS |  | 3 | -0.73 | -1.24 | 0.232 | 0.748 | 1.000 | 1373 | tags=67%, list=11%, signal=75% |
| 986 | GOBP\_REGULATION\_OF\_TRANSLATIONAL\_ELONGATION |  | 15 | -0.45 | -1.24 | 0.194 | 0.749 | 1.000 | 3615 | tags=67%, list=30%, signal=95% |
| 987 | GOBP\_BRANCHING\_MORPHOGENESIS\_OF\_A\_NERVE |  | 7 | -0.56 | -1.24 | 0.225 | 0.749 | 1.000 | 969 | tags=29%, list=8%, signal=31% |
| 988 | GOCC\_G\_PROTEIN\_BETA\_GAMMA\_SUBUNIT\_COMPLEX |  | 6 | -0.59 | -1.24 | 0.227 | 0.749 | 1.000 | 4130 | tags=83%, list=34%, signal=127% |
| 989 | GOBP\_NEGATIVE\_REGULATION\_OF\_HAIR\_FOLLICLE\_DEVELOPMENT |  | 4 | -0.67 | -1.24 | 0.229 | 0.748 | 1.000 | 1806 | tags=50%, list=15%, signal=59% |
| 990 | GOCC\_N\_TERMINAL\_PROTEIN\_ACETYLTRANSFERASE\_COMPLEX |  | 8 | -0.55 | -1.24 | 0.214 | 0.748 | 1.000 | 4897 | tags=75%, list=41%, signal=127% |
| 991 | GOBP\_LACTATE\_METABOLIC\_PROCESS |  | 11 | -0.50 | -1.24 | 0.213 | 0.747 | 1.000 | 2505 | tags=55%, list=21%, signal=69% |
| 992 | GOBP\_EPITHELIAL\_CELL\_MATURATION |  | 13 | -0.47 | -1.24 | 0.215 | 0.747 | 1.000 | 1418 | tags=23%, list=12%, signal=26% |
| 993 | GOBP\_OLFACTORY\_LOBE\_DEVELOPMENT |  | 16 | -0.45 | -1.24 | 0.201 | 0.747 | 1.000 | 1489 | tags=31%, list=12%, signal=36% |
| 994 | GOCC\_VACUOLAR\_MEMBRANE |  | 322 | -0.25 | -1.24 | 0.026 | 0.749 | 1.000 | 3116 | tags=34%, list=26%, signal=44% |
| 995 | GOBP\_MRNA\_PSEUDOURIDINE\_SYNTHESIS |  | 5 | -0.62 | -1.24 | 0.234 | 0.751 | 1.000 | 2466 | tags=80%, list=21%, signal=101% |
| 996 | GOBP\_BONE\_REMODELING |  | 65 | -0.32 | -1.24 | 0.115 | 0.750 | 1.000 | 2194 | tags=38%, list=18%, signal=47% |
| 997 | GOMF\_2\_IRON\_2\_SULFUR\_CLUSTER\_BINDING |  | 18 | -0.43 | -1.24 | 0.187 | 0.752 | 1.000 | 460 | tags=17%, list=4%, signal=17% |
| 998 | GOMF\_ARACHIDONIC\_ACID\_MONOOXYGENASE\_ACTIVITY |  | 3 | -0.73 | -1.23 | 0.220 | 0.753 | 1.000 | 3236 | tags=100%, list=27%, signal=137% |
| 999 | GOBP\_GLYCERALDEHYDE\_3\_PHOSPHATE\_METABOLIC\_PROCESS |  | 7 | -0.57 | -1.23 | 0.227 | 0.754 | 1.000 | 57 | tags=14%, list=0%, signal=14% |
| 1000 | GOBP\_POSITIVE\_REGULATION\_OF\_ESTABLISHMENT\_OF\_PROTEIN\_LOCALIZATION\_TO\_TELOMERE |  | 9 | -0.52 | -1.23 | 0.218 | 0.754 | 1.000 | 5172 | tags=78%, list=43%, signal=136% |
| 1001 | GOBP\_PEPTIDE\_CROSS\_LINKING\_VIA\_CHONDROITIN\_4\_SULFATE\_GLYCOSAMINOGLYCAN |  | 6 | -0.59 | -1.23 | 0.240 | 0.754 | 1.000 | 1202 | tags=33%, list=10%, signal=37% |
| 1002 | GOCC\_HISTONE\_DEACETYLASE\_COMPLEX |  | 56 | -0.33 | -1.23 | 0.131 | 0.755 | 1.000 | 2317 | tags=27%, list=19%, signal=33% |
| 1003 | GOBP\_SUCKLING\_BEHAVIOR |  | 8 | -0.54 | -1.23 | 0.210 | 0.755 | 1.000 | 1514 | tags=25%, list=13%, signal=29% |
| 1004 | GOBP\_REGULATION\_OF\_AXONOGENESIS |  | 112 | -0.29 | -1.23 | 0.092 | 0.755 | 1.000 | 2656 | tags=30%, list=22%, signal=39% |
| 1005 | GOBP\_CARBOHYDRATE\_DERIVATIVE\_TRANSPORT |  | 56 | -0.33 | -1.23 | 0.124 | 0.754 | 1.000 | 2985 | tags=46%, list=25%, signal=61% |
| 1006 | GOBP\_PARATHYROID\_GLAND\_DEVELOPMENT |  | 4 | -0.67 | -1.23 | 0.243 | 0.754 | 1.000 | 2530 | tags=75%, list=21%, signal=95% |
| 1007 | GOMF\_RNA\_POLYMERASE\_II\_CTD\_HEPTAPEPTIDE\_REPEAT\_KINASE\_ACTIVITY |  | 10 | -0.51 | -1.23 | 0.224 | 0.754 | 1.000 | 1645 | tags=20%, list=14%, signal=23% |
| 1008 | GOBP\_NEGATIVE\_REGULATION\_OF\_LIPOPROTEIN\_METABOLIC\_PROCESS |  | 5 | -0.62 | -1.23 | 0.243 | 0.754 | 1.000 | 162 | tags=20%, list=1%, signal=20% |
| 1009 | GOBP\_TETRAPYRROLE\_BIOSYNTHETIC\_PROCESS |  | 24 | -0.40 | -1.23 | 0.182 | 0.755 | 1.000 | 3737 | tags=54%, list=31%, signal=78% |
| 1010 | GOBP\_RRNA\_CONTAINING\_RIBONUCLEOPROTEIN\_COMPLEX\_EXPORT\_FROM\_NUCLEUS |  | 13 | -0.47 | -1.23 | 0.207 | 0.755 | 1.000 | 4582 | tags=62%, list=38%, signal=99% |
| 1011 | GOBP\_REGULATION\_OF\_ATRIAL\_CARDIAC\_MUSCLE\_CELL\_MEMBRANE\_REPOLARIZATION |  | 4 | -0.67 | -1.23 | 0.231 | 0.755 | 1.000 | 713 | tags=50%, list=6%, signal=53% |
| 1012 | GOBP\_POSITIVE\_REGULATION\_OF\_MITOCHONDRIAL\_TRANSLATION |  | 13 | -0.47 | -1.23 | 0.206 | 0.754 | 1.000 | 3883 | tags=46%, list=32%, signal=68% |
| 1013 | GOBP\_SEROTONIN\_RECEPTOR\_SIGNALING\_PATHWAY |  | 13 | -0.46 | -1.23 | 0.216 | 0.755 | 1.000 | 1701 | tags=38%, list=14%, signal=45% |
| 1014 | GOBP\_POSITIVE\_REGULATION\_OF\_IMMUNOGLOBULIN\_PRODUCTION |  | 23 | -0.40 | -1.23 | 0.196 | 0.755 | 1.000 | 3209 | tags=57%, list=27%, signal=77% |
| 1015 | GOBP\_GTP\_METABOLIC\_PROCESS |  | 18 | -0.42 | -1.23 | 0.209 | 0.754 | 1.000 | 1626 | tags=33%, list=14%, signal=38% |
| 1016 | GOMF\_OXIDOREDUCTASE\_ACTIVITY\_ACTING\_ON\_A\_SULFUR\_GROUP\_OF\_DONORS\_DISULFIDE\_AS\_ACCEPTOR |  | 8 | -0.54 | -1.23 | 0.230 | 0.754 | 1.000 | 913 | tags=25%, list=8%, signal=27% |
| 1017 | GOBP\_POSITIVE\_REGULATION\_OF\_GRANULOCYTE\_DIFFERENTIATION |  | 6 | -0.59 | -1.23 | 0.239 | 0.753 | 1.000 | 2760 | tags=50%, list=23%, signal=65% |
| 1018 | GOMF\_ATPASE\_COUPLED\_LIPID\_TRANSMEMBRANE\_TRANSPORTER\_ACTIVITY |  | 4 | -0.66 | -1.23 | 0.248 | 0.753 | 1.000 | 2256 | tags=75%, list=19%, signal=92% |
| 1019 | GOBP\_REGULATION\_OF\_MACROPHAGE\_PROLIFERATION |  | 4 | -0.68 | -1.23 | 0.240 | 0.752 | 1.000 | 964 | tags=50%, list=8%, signal=54% |
| 1020 | GOBP\_GLUCOSE\_IMPORT\_IN\_RESPONSE\_TO\_INSULIN\_STIMULUS |  | 3 | -0.73 | -1.23 | 0.226 | 0.752 | 1.000 | 3237 | tags=100%, list=27%, signal=137% |
| 1021 | GOBP\_REGULATION\_OF\_HISTONE\_H3\_K9\_TRIMETHYLATION |  | 3 | -0.72 | -1.23 | 0.231 | 0.753 | 1.000 | 2541 | tags=67%, list=21%, signal=85% |
| 1022 | GOBP\_SYMPATHETIC\_NERVOUS\_SYSTEM\_DEVELOPMENT |  | 13 | -0.47 | -1.23 | 0.219 | 0.753 | 1.000 | 1903 | tags=38%, list=16%, signal=46% |
| 1023 | GOCC\_SPINDLE\_POLE\_CENTROSOME |  | 11 | -0.49 | -1.23 | 0.219 | 0.752 | 1.000 | 791 | tags=27%, list=7%, signal=29% |
| 1024 | GOBP\_MODULATION\_BY\_HOST\_OF\_VIRAL\_PROCESS |  | 21 | -0.41 | -1.23 | 0.191 | 0.751 | 1.000 | 2516 | tags=43%, list=21%, signal=54% |
| 1025 | GOBP\_HEMATOPOIETIC\_PROGENITOR\_CELL\_DIFFERENTIATION |  | 125 | -0.28 | -1.23 | 0.096 | 0.752 | 1.000 | 3160 | tags=34%, list=26%, signal=45% |
| 1026 | GOBP\_POSITIVE\_REGULATION\_OF\_ER\_ASSOCIATED\_UBIQUITIN\_DEPENDENT\_PROTEIN\_CATABOLIC\_PROCESS |  | 6 | -0.59 | -1.23 | 0.236 | 0.752 | 1.000 | 1747 | tags=50%, list=15%, signal=58% |
| 1027 | GOBP\_REGULATION\_OF\_3\_UTR\_MEDIATED\_MRNA\_STABILIZATION |  | 4 | -0.66 | -1.23 | 0.241 | 0.754 | 1.000 | 1678 | tags=50%, list=14%, signal=58% |
| 1028 | GOMF\_TRANSPORTER\_ACTIVITY |  | 669 | -0.23 | -1.23 | 0.003 | 0.756 | 1.000 | 1961 | tags=23%, list=16%, signal=26% |
| 1029 | GOBP\_MUSCLE\_FILAMENT\_SLIDING |  | 14 | -0.45 | -1.23 | 0.225 | 0.755 | 1.000 | 1439 | tags=29%, list=12%, signal=32% |
| 1030 | GOBP\_STEM\_CELL\_DIVISION |  | 18 | -0.43 | -1.23 | 0.203 | 0.754 | 1.000 | 1139 | tags=28%, list=9%, signal=31% |
| 1031 | GOBP\_ASSOCIATIVE\_LEARNING |  | 44 | -0.34 | -1.23 | 0.162 | 0.755 | 1.000 | 1691 | tags=25%, list=14%, signal=29% |
| 1032 | GOBP\_REGULATION\_OF\_MITOCHONDRIAL\_MRNA\_STABILITY |  | 6 | -0.59 | -1.23 | 0.239 | 0.755 | 1.000 | 4199 | tags=83%, list=35%, signal=128% |
| 1033 | GOBP\_RESPONSE\_TO\_IRON\_ION |  | 17 | -0.43 | -1.23 | 0.203 | 0.755 | 1.000 | 2569 | tags=41%, list=21%, signal=52% |
| 1034 | GOBP\_TRIPEPTIDE\_TRANSPORT |  | 5 | -0.62 | -1.23 | 0.256 | 0.757 | 1.000 | 3785 | tags=80%, list=32%, signal=117% |
| 1035 | GOMF\_PROTEIN\_KINASE\_A\_CATALYTIC\_SUBUNIT\_BINDING |  | 12 | -0.47 | -1.23 | 0.223 | 0.757 | 1.000 | 935 | tags=25%, list=8%, signal=27% |
| 1036 | GOBP\_VESICLE\_UNCOATING |  | 5 | -0.62 | -1.23 | 0.250 | 0.757 | 1.000 | 160 | tags=20%, list=1%, signal=20% |
| 1037 | GOBP\_MATERNAL\_PROCESS\_INVOLVED\_IN\_FEMALE\_PREGNANCY |  | 38 | -0.35 | -1.22 | 0.166 | 0.757 | 1.000 | 3086 | tags=39%, list=26%, signal=53% |
| 1038 | GOBP\_ONE\_CARBON\_METABOLIC\_PROCESS |  | 29 | -0.38 | -1.22 | 0.179 | 0.757 | 1.000 | 1061 | tags=21%, list=9%, signal=23% |
| 1039 | GOMF\_PHOSPHATIDIC\_ACID\_TRANSFER\_ACTIVITY |  | 5 | -0.62 | -1.22 | 0.254 | 0.757 | 1.000 | 3698 | tags=80%, list=31%, signal=116% |
| 1040 | GOCC\_GROWTH\_FACTOR\_COMPLEX |  | 3 | -0.72 | -1.22 | 0.225 | 0.757 | 1.000 | 3384 | tags=100%, list=28%, signal=139% |
| 1041 | GOBP\_REGULATION\_OF\_INSULIN\_LIKE\_GROWTH\_FACTOR\_RECEPTOR\_SIGNALING\_PATHWAY |  | 14 | -0.45 | -1.22 | 0.211 | 0.756 | 1.000 | 3701 | tags=57%, list=31%, signal=82% |
| 1042 | GOBP\_REGULATION\_OF\_NEUTROPHIL\_DEGRANULATION |  | 4 | -0.66 | -1.22 | 0.249 | 0.756 | 1.000 | 4071 | tags=100%, list=34%, signal=151% |
| 1043 | GOCC\_SEMAPHORIN\_RECEPTOR\_COMPLEX |  | 8 | -0.54 | -1.22 | 0.233 | 0.756 | 1.000 | 2290 | tags=50%, list=19%, signal=62% |
| 1044 | GOMF\_PEPTIDOGLYCAN\_BINDING |  | 6 | -0.58 | -1.22 | 0.250 | 0.757 | 1.000 | 3937 | tags=83%, list=33%, signal=124% |
| 1045 | GOMF\_PROTON\_CHANNEL\_ACTIVITY |  | 7 | -0.57 | -1.22 | 0.239 | 0.756 | 1.000 | 651 | tags=29%, list=5%, signal=30% |
| 1046 | GOBP\_LYMPHOCYTE\_AGGREGATION |  | 4 | -0.67 | -1.22 | 0.253 | 0.756 | 1.000 | 3972 | tags=100%, list=33%, signal=149% |
| 1047 | GOMF\_PHOSPHATIDYLCHOLINE\_FLOPPASE\_ACTIVITY |  | 3 | -0.72 | -1.22 | 0.235 | 0.756 | 1.000 | 2337 | tags=67%, list=19%, signal=83% |
| 1048 | GOBP\_EMBRYONIC\_ORGAN\_MORPHOGENESIS |  | 162 | -0.27 | -1.22 | 0.084 | 0.756 | 1.000 | 836 | tags=16%, list=7%, signal=17% |
| 1049 | GOBP\_HISTONE\_H3\_K4\_DEMETHYLATION |  | 5 | -0.61 | -1.22 | 0.254 | 0.759 | 1.000 | 4652 | tags=100%, list=39%, signal=163% |
| 1050 | GOBP\_NEGATIVE\_REGULATION\_OF\_JNK\_CASCADE |  | 23 | -0.40 | -1.22 | 0.196 | 0.761 | 1.000 | 1359 | tags=17%, list=11%, signal=20% |
| 1051 | GOBP\_THYMIC\_T\_CELL\_SELECTION |  | 18 | -0.43 | -1.22 | 0.211 | 0.760 | 1.000 | 3199 | tags=50%, list=27%, signal=68% |
| 1052 | GOBP\_TYROSINE\_METABOLIC\_PROCESS |  | 4 | -0.66 | -1.22 | 0.242 | 0.760 | 1.000 | 2357 | tags=50%, list=20%, signal=62% |
| 1053 | GOMF\_COA\_TRANSFERASE\_ACTIVITY |  | 4 | -0.66 | -1.22 | 0.256 | 0.760 | 1.000 | 4046 | tags=100%, list=34%, signal=151% |
| 1054 | GOBP\_RESPONSE\_TO\_DOPAMINE |  | 44 | -0.34 | -1.22 | 0.156 | 0.759 | 1.000 | 2774 | tags=36%, list=23%, signal=47% |
| 1055 | GOMF\_LIPID\_KINASE\_ACTIVITY |  | 4 | -0.67 | -1.22 | 0.251 | 0.759 | 1.000 | 2853 | tags=75%, list=24%, signal=98% |
| 1056 | GOCC\_MITOCHONDRIAL\_INTERMEMBRANE\_SPACE\_PROTEIN\_TRANSPORTER\_COMPLEX |  | 4 | -0.66 | -1.22 | 0.252 | 0.759 | 1.000 | 2998 | tags=75%, list=25%, signal=100% |
| 1057 | GOMF\_LEUCINE\_BINDING |  | 6 | -0.58 | -1.22 | 0.251 | 0.758 | 1.000 | 1344 | tags=33%, list=11%, signal=38% |
| 1058 | GOBP\_POSITIVE\_REGULATION\_OF\_RNA\_BINDING |  | 7 | -0.56 | -1.22 | 0.240 | 0.759 | 1.000 | 1645 | tags=43%, list=14%, signal=50% |
| 1059 | GOBP\_POSITIVE\_REGULATION\_OF\_INTERLEUKIN\_2\_PRODUCTION |  | 24 | -0.40 | -1.22 | 0.191 | 0.759 | 1.000 | 2760 | tags=46%, list=23%, signal=59% |
| 1060 | GOMF\_MOLECULAR\_TRANSDUCER\_ACTIVITY |  | 551 | -0.24 | -1.22 | 0.011 | 0.758 | 1.000 | 2200 | tags=25%, list=18%, signal=29% |
| 1061 | GOBP\_ANION\_TRANSMEMBRANE\_TRANSPORT |  | 355 | -0.25 | -1.22 | 0.038 | 0.759 | 1.000 | 1941 | tags=23%, list=16%, signal=26% |
| 1062 | GOBP\_REGULATION\_OF\_MATRIX\_METALLOPEPTIDASE\_SECRETION |  | 4 | -0.67 | -1.22 | 0.240 | 0.761 | 1.000 | 2591 | tags=50%, list=22%, signal=64% |
| 1063 | GOCC\_PWP2P\_CONTAINING\_SUBCOMPLEX\_OF\_90S\_PRERIBOSOME |  | 5 | -0.62 | -1.22 | 0.253 | 0.760 | 1.000 | 3449 | tags=60%, list=29%, signal=84% |
| 1064 | GOCC\_CELL\_POLE |  | 5 | -0.61 | -1.22 | 0.256 | 0.759 | 1.000 | 1200 | tags=40%, list=10%, signal=44% |
| 1065 | GOBP\_LEUKOTRIENE\_SIGNALING\_PATHWAY |  | 3 | -0.72 | -1.22 | 0.239 | 0.759 | 1.000 | 3314 | tags=100%, list=28%, signal=138% |
| 1066 | GOBP\_THYROID\_HORMONE\_METABOLIC\_PROCESS |  | 13 | -0.47 | -1.22 | 0.222 | 0.759 | 1.000 | 636 | tags=23%, list=5%, signal=24% |
| 1067 | GOBP\_NEGATIVE\_REGULATION\_OF\_HEMATOPOIETIC\_STEM\_CELL\_DIFFERENTIATION |  | 3 | -0.72 | -1.22 | 0.259 | 0.758 | 1.000 | 581 | tags=33%, list=5%, signal=35% |
| 1068 | GOBP\_NEGATIVE\_REGULATION\_OF\_PHOSPHOLIPID\_METABOLIC\_PROCESS |  | 5 | -0.62 | -1.22 | 0.251 | 0.758 | 1.000 | 426 | tags=20%, list=4%, signal=21% |
| 1069 | GOBP\_REGULATION\_OF\_NEUTROPHIL\_ACTIVATION |  | 5 | -0.62 | -1.22 | 0.255 | 0.758 | 1.000 | 4071 | tags=80%, list=34%, signal=121% |
| 1070 | GOBP\_PLACENTA\_DEVELOPMENT |  | 91 | -0.30 | -1.22 | 0.112 | 0.762 | 1.000 | 2018 | tags=24%, list=17%, signal=29% |
| 1071 | GOBP\_PRESYNAPTIC\_MEMBRANE\_ORGANIZATION |  | 6 | -0.58 | -1.22 | 0.251 | 0.762 | 1.000 | 1168 | tags=33%, list=10%, signal=37% |
| 1072 | GOMF\_SEROTONIN\_RECEPTOR\_ACTIVITY |  | 13 | -0.46 | -1.22 | 0.227 | 0.761 | 1.000 | 1701 | tags=38%, list=14%, signal=45% |
| 1073 | GOBP\_PROTEIN\_INSERTION\_INTO\_MITOCHONDRIAL\_INNER\_MEMBRANE |  | 9 | -0.51 | -1.22 | 0.238 | 0.761 | 1.000 | 2998 | tags=56%, list=25%, signal=74% |
| 1074 | GOCC\_CHROMAFFIN\_GRANULE\_MEMBRANE |  | 6 | -0.59 | -1.22 | 0.245 | 0.760 | 1.000 | 1005 | tags=33%, list=8%, signal=36% |
| 1075 | GOBP\_POSITIVE\_REGULATION\_OF\_LEUKOCYTE\_ADHESION\_TO\_VASCULAR\_ENDOTHELIAL\_CELL |  | 13 | -0.46 | -1.22 | 0.224 | 0.761 | 1.000 | 4329 | tags=62%, list=36%, signal=96% |
| 1076 | GOCC\_NATA\_COMPLEX |  | 3 | -0.72 | -1.22 | 0.242 | 0.760 | 1.000 | 3420 | tags=100%, list=28%, signal=140% |
| 1077 | GOBP\_FERTILIZATION |  | 75 | -0.31 | -1.21 | 0.139 | 0.761 | 1.000 | 651 | tags=13%, list=5%, signal=14% |
| 1078 | GOBP\_RNA\_METHYLATION |  | 62 | -0.32 | -1.21 | 0.142 | 0.761 | 1.000 | 3120 | tags=37%, list=26%, signal=50% |
| 1079 | GOBP\_UROGENITAL\_SYSTEM\_DEVELOPMENT |  | 243 | -0.26 | -1.21 | 0.058 | 0.761 | 1.000 | 1168 | tags=16%, list=10%, signal=17% |
| 1080 | GOBP\_DORSAL\_VENTRAL\_PATTERN\_FORMATION |  | 43 | -0.34 | -1.21 | 0.164 | 0.762 | 1.000 | 2656 | tags=40%, list=22%, signal=51% |
| 1081 | GOBP\_NEGATIVE\_REGULATION\_OF\_HISTONE\_H4\_ACETYLATION |  | 5 | -0.61 | -1.21 | 0.259 | 0.761 | 1.000 | 2909 | tags=60%, list=24%, signal=79% |
| 1082 | GOBP\_VENTRICULAR\_CARDIAC\_MUSCLE\_CELL\_DIFFERENTIATION |  | 9 | -0.51 | -1.21 | 0.243 | 0.761 | 1.000 | 3144 | tags=44%, list=26%, signal=60% |
| 1083 | GOBP\_NUCLEOTIDE\_TRANSPORT |  | 22 | -0.40 | -1.21 | 0.216 | 0.761 | 1.000 | 2911 | tags=45%, list=24%, signal=60% |
| 1084 | GOBP\_NEGATIVE\_REGULATION\_OF\_HEART\_RATE |  | 5 | -0.62 | -1.21 | 0.255 | 0.760 | 1.000 | 12 | tags=20%, list=0%, signal=20% |
| 1085 | GOBP\_MIDBRAIN\_HINDBRAIN\_BOUNDARY\_DEVELOPMENT |  | 3 | -0.72 | -1.21 | 0.257 | 0.760 | 1.000 | 3362 | tags=100%, list=28%, signal=139% |
| 1086 | GOBP\_POSITIVE\_REGULATION\_OF\_COMPLEMENT\_ACTIVATION |  | 4 | -0.66 | -1.21 | 0.266 | 0.760 | 1.000 | 3636 | tags=75%, list=30%, signal=108% |
| 1087 | GOBP\_REGULATION\_OF\_PROTEIN\_LIPIDATION |  | 6 | -0.58 | -1.21 | 0.260 | 0.760 | 1.000 | 162 | tags=17%, list=1%, signal=17% |
| 1088 | GOBP\_PTERIDINE\_CONTAINING\_COMPOUND\_BIOSYNTHETIC\_PROCESS |  | 13 | -0.46 | -1.21 | 0.226 | 0.760 | 1.000 | 349 | tags=23%, list=3%, signal=24% |
| 1089 | GOBP\_POLYADENYLATION\_DEPENDENT\_SNORNA\_3\_END\_PROCESSING |  | 6 | -0.58 | -1.21 | 0.257 | 0.761 | 1.000 | 3711 | tags=67%, list=31%, signal=96% |
| 1090 | GOBP\_CILIUM\_MOVEMENT |  | 64 | -0.31 | -1.21 | 0.155 | 0.760 | 1.000 | 1371 | tags=19%, list=11%, signal=21% |
| 1091 | GOBP\_NONRIBOSOMAL\_PEPTIDE\_BIOSYNTHETIC\_PROCESS |  | 13 | -0.46 | -1.21 | 0.224 | 0.760 | 1.000 | 581 | tags=15%, list=5%, signal=16% |
| 1092 | GOBP\_RENAL\_SYSTEM\_PROCESS |  | 78 | -0.30 | -1.21 | 0.137 | 0.759 | 1.000 | 1759 | tags=21%, list=15%, signal=24% |
| 1093 | GOCC\_SEH1\_ASSOCIATED\_COMPLEX |  | 14 | -0.45 | -1.21 | 0.223 | 0.760 | 1.000 | 1586 | tags=29%, list=13%, signal=33% |
| 1094 | GOBP\_NUCLEAR\_NCRNA\_SURVEILLANCE |  | 6 | -0.58 | -1.21 | 0.258 | 0.760 | 1.000 | 3082 | tags=50%, list=26%, signal=67% |
| 1095 | GOBP\_INTESTINAL\_EPITHELIAL\_STRUCTURE\_MAINTENANCE |  | 5 | -0.61 | -1.21 | 0.265 | 0.760 | 1.000 | 3765 | tags=40%, list=31%, signal=58% |
| 1096 | GOBP\_LYMPHOID\_PROGENITOR\_CELL\_DIFFERENTIATION |  | 19 | -0.42 | -1.21 | 0.210 | 0.759 | 1.000 | 3624 | tags=68%, list=30%, signal=98% |
| 1097 | GOBP\_ACTIVATION\_OF\_STORE\_OPERATED\_CALCIUM\_CHANNEL\_ACTIVITY |  | 3 | -0.72 | -1.21 | 0.260 | 0.759 | 1.000 | 1378 | tags=67%, list=11%, signal=75% |
| 1098 | GOBP\_SUPEROXIDE\_ANION\_GENERATION |  | 20 | -0.41 | -1.21 | 0.206 | 0.758 | 1.000 | 2040 | tags=40%, list=17%, signal=48% |
| 1099 | GOBP\_ISOLEUCINE\_METABOLIC\_PROCESS |  | 5 | -0.61 | -1.21 | 0.256 | 0.760 | 1.000 | 3167 | tags=60%, list=26%, signal=81% |
| 1100 | GOBP\_REGULATION\_OF\_PSEUDOPODIUM\_ASSEMBLY |  | 9 | -0.51 | -1.21 | 0.229 | 0.760 | 1.000 | 1805 | tags=33%, list=15%, signal=39% |
| 1101 | GOMF\_COMPLEMENT\_BINDING |  | 12 | -0.47 | -1.21 | 0.231 | 0.760 | 1.000 | 1958 | tags=42%, list=16%, signal=50% |
| 1102 | GOBP\_COLLECTING\_DUCT\_DEVELOPMENT |  | 9 | -0.52 | -1.21 | 0.248 | 0.760 | 1.000 | 1656 | tags=22%, list=14%, signal=26% |
| 1103 | GOBP\_REGULATION\_OF\_INTRINSIC\_APOPTOTIC\_SIGNALING\_PATHWAY\_IN\_RESPONSE\_TO\_DNA\_DAMAGE |  | 24 | -0.39 | -1.21 | 0.204 | 0.761 | 1.000 | 3011 | tags=42%, list=25%, signal=55% |
| 1104 | GOMF\_SOLUTE\_CATION\_SYMPORTER\_ACTIVITY |  | 61 | -0.31 | -1.21 | 0.162 | 0.761 | 1.000 | 1275 | tags=28%, list=11%, signal=31% |
| 1105 | GOBP\_REGULATION\_OF\_MRNA\_BINDING |  | 7 | -0.55 | -1.21 | 0.253 | 0.763 | 1.000 | 1645 | tags=43%, list=14%, signal=50% |
| 1106 | GOBP\_POSITIVE\_REGULATION\_OF\_MAST\_CELL\_ACTIVATION |  | 12 | -0.47 | -1.21 | 0.233 | 0.762 | 1.000 | 4618 | tags=75%, list=38%, signal=122% |
| 1107 | GOBP\_RESPONSE\_TO\_LEPTIN |  | 19 | -0.41 | -1.21 | 0.215 | 0.762 | 1.000 | 2586 | tags=37%, list=22%, signal=47% |
| 1108 | GOBP\_RESPONSE\_TO\_PAIN |  | 20 | -0.41 | -1.21 | 0.206 | 0.762 | 1.000 | 523 | tags=15%, list=4%, signal=16% |
| 1109 | GOMF\_METAL\_ION\_TRANSMEMBRANE\_TRANSPORTER\_ACTIVITY |  | 244 | -0.25 | -1.21 | 0.056 | 0.762 | 1.000 | 1948 | tags=25%, list=16%, signal=30% |
| 1110 | GOBP\_KIDNEY\_MORPHOGENESIS |  | 59 | -0.32 | -1.21 | 0.154 | 0.762 | 1.000 | 2042 | tags=27%, list=17%, signal=33% |
| 1111 | GOCC\_MITOCHONDRIAL\_RESPIRATORY\_CHAIN\_COMPLEX\_IV |  | 7 | -0.56 | -1.21 | 0.266 | 0.763 | 1.000 | 4233 | tags=57%, list=35%, signal=88% |
| 1112 | GOBP\_GLUTATHIONE\_TRANSMEMBRANE\_TRANSPORT |  | 3 | -0.71 | -1.21 | 0.265 | 0.764 | 1.000 | 1476 | tags=67%, list=12%, signal=76% |
| 1113 | GOBP\_DOPAMINE\_TRANSPORT |  | 31 | -0.37 | -1.21 | 0.203 | 0.764 | 1.000 | 2560 | tags=55%, list=21%, signal=70% |
| 1114 | GOBP\_REGULATION\_OF\_JUN\_KINASE\_ACTIVITY |  | 56 | -0.32 | -1.21 | 0.149 | 0.764 | 1.000 | 1574 | tags=20%, list=13%, signal=22% |
| 1115 | GOBP\_CHAPERONE\_MEDIATED\_PROTEIN\_TRANSPORT |  | 7 | -0.55 | -1.21 | 0.263 | 0.766 | 1.000 | 2998 | tags=57%, list=25%, signal=76% |
| 1116 | GOBP\_PYRIMIDINE\_NUCLEOSIDE\_TRIPHOSPHATE\_BIOSYNTHETIC\_PROCESS |  | 15 | -0.44 | -1.21 | 0.230 | 0.765 | 1.000 | 834 | tags=33%, list=7%, signal=36% |
| 1117 | GOBP\_SKELETAL\_MUSCLE\_ORGAN\_DEVELOPMENT |  | 101 | -0.28 | -1.20 | 0.134 | 0.766 | 1.000 | 1209 | tags=15%, list=10%, signal=16% |
| 1118 | GOBP\_SMOOTH\_MUSCLE\_ADAPTATION |  | 3 | -0.71 | -1.20 | 0.258 | 0.765 | 1.000 | 3499 | tags=67%, list=29%, signal=94% |
| 1119 | GOBP\_PURINE\_NUCLEOSIDE\_MONOPHOSPHATE\_BIOSYNTHETIC\_PROCESS |  | 18 | -0.42 | -1.20 | 0.227 | 0.765 | 1.000 | 2219 | tags=39%, list=18%, signal=48% |
| 1120 | GOBP\_SENSORY\_PERCEPTION\_OF\_CHEMICAL\_STIMULUS |  | 30 | -0.37 | -1.20 | 0.194 | 0.767 | 1.000 | 2201 | tags=37%, list=18%, signal=45% |
| 1121 | GOBP\_NEGATIVE\_REGULATION\_OF\_NITRIC\_OXIDE\_METABOLIC\_PROCESS |  | 10 | -0.50 | -1.20 | 0.248 | 0.767 | 1.000 | 448 | tags=30%, list=4%, signal=31% |
| 1122 | GOCC\_KINOCILIUM |  | 5 | -0.61 | -1.20 | 0.258 | 0.767 | 1.000 | 95 | tags=20%, list=1%, signal=20% |
| 1123 | GOBP\_POSITIVE\_REGULATION\_OF\_NON\_CANONICAL\_WNT\_SIGNALING\_PATHWAY |  | 12 | -0.47 | -1.20 | 0.244 | 0.767 | 1.000 | 166 | tags=17%, list=1%, signal=17% |
| 1124 | GOBP\_NEGATIVE\_REGULATION\_OF\_TROPHOBLAST\_CELL\_MIGRATION |  | 3 | -0.71 | -1.20 | 0.259 | 0.767 | 1.000 | 1670 | tags=67%, list=14%, signal=77% |
| 1125 | GOBP\_EPITHELIAL\_CELL\_DIFFERENTIATION\_INVOLVED\_IN\_PROSTATE\_GLAND\_DEVELOPMENT |  | 8 | -0.52 | -1.20 | 0.254 | 0.767 | 1.000 | 3624 | tags=75%, list=30%, signal=107% |
| 1126 | GOBP\_POSITIVE\_REGULATION\_OF\_NUCLEOTIDE\_BINDING\_OLIGOMERIZATION\_DOMAIN\_CONTAINING\_SIGNALING\_PATHWAY |  | 3 | -0.71 | -1.20 | 0.262 | 0.768 | 1.000 | 3353 | tags=67%, list=28%, signal=92% |
| 1127 | GOBP\_MAINTENANCE\_OF\_PROTEIN\_LOCATION\_IN\_MITOCHONDRION |  | 4 | -0.65 | -1.20 | 0.266 | 0.768 | 1.000 | 3621 | tags=75%, list=30%, signal=107% |
| 1128 | GOBP\_LYSOSOME\_LOCALIZATION |  | 55 | -0.32 | -1.20 | 0.165 | 0.768 | 1.000 | 3716 | tags=53%, list=31%, signal=76% |
| 1129 | GOMF\_IMMUNOGLOBULIN\_RECEPTOR\_BINDING |  | 4 | -0.65 | -1.20 | 0.272 | 0.768 | 1.000 | 2209 | tags=50%, list=18%, signal=61% |
| 1130 | GOCC\_SWI\_SNF\_SUPERFAMILY\_TYPE\_COMPLEX |  | 55 | -0.32 | -1.20 | 0.160 | 0.768 | 1.000 | 2541 | tags=24%, list=21%, signal=30% |
| 1131 | GOBP\_MONOVALENT\_INORGANIC\_CATION\_HOMEOSTASIS |  | 108 | -0.28 | -1.20 | 0.126 | 0.767 | 1.000 | 1941 | tags=23%, list=16%, signal=27% |
| 1132 | GOCC\_RDNA\_HETEROCHROMATIN |  | 3 | -0.71 | -1.20 | 0.263 | 0.767 | 1.000 | 3510 | tags=100%, list=29%, signal=141% |
| 1133 | GOBP\_ION\_HOMEOSTASIS |  | 493 | -0.24 | -1.20 | 0.028 | 0.767 | 1.000 | 1774 | tags=22%, list=15%, signal=24% |
| 1134 | GOMF\_ATP\_DEPENDENT\_MICROTUBULE\_MOTOR\_ACTIVITY\_MINUS\_END\_DIRECTED |  | 7 | -0.55 | -1.20 | 0.264 | 0.767 | 1.000 | 21 | tags=14%, list=0%, signal=14% |
| 1135 | GOBP\_INFLAMMATORY\_RESPONSE\_TO\_ANTIGENIC\_STIMULUS |  | 24 | -0.38 | -1.20 | 0.205 | 0.767 | 1.000 | 2505 | tags=42%, list=21%, signal=53% |
| 1136 | GOBP\_POLYADENYLATION\_DEPENDENT\_RNA\_CATABOLIC\_PROCESS |  | 7 | -0.54 | -1.20 | 0.259 | 0.766 | 1.000 | 3082 | tags=43%, list=26%, signal=58% |
| 1137 | GOBP\_APOPTOTIC\_PROCESS\_INVOLVED\_IN\_BLOOD\_VESSEL\_MORPHOGENESIS |  | 4 | -0.65 | -1.20 | 0.273 | 0.766 | 1.000 | 1380 | tags=50%, list=11%, signal=56% |
| 1138 | GOBP\_HEPARAN\_SULFATE\_PROTEOGLYCAN\_METABOLIC\_PROCESS |  | 25 | -0.39 | -1.20 | 0.211 | 0.765 | 1.000 | 1162 | tags=28%, list=10%, signal=31% |
| 1139 | GOBP\_IRON\_ION\_IMPORT\_ACROSS\_PLASMA\_MEMBRANE |  | 3 | -0.71 | -1.20 | 0.262 | 0.765 | 1.000 | 3520 | tags=100%, list=29%, signal=141% |
| 1140 | GOBP\_MAST\_CELL\_ACTIVATION\_INVOLVED\_IN\_IMMUNE\_RESPONSE |  | 31 | -0.37 | -1.20 | 0.210 | 0.765 | 1.000 | 3714 | tags=55%, list=31%, signal=79% |
| 1141 | GOBP\_OSTEOCLAST\_DEVELOPMENT |  | 8 | -0.53 | -1.20 | 0.265 | 0.765 | 1.000 | 3161 | tags=63%, list=26%, signal=85% |
| 1142 | GOBP\_HISTONE\_H3\_K4\_DEMETHYLATION\_TRIMETHYL\_H3\_K4\_SPECIFIC |  | 3 | -0.71 | -1.20 | 0.265 | 0.766 | 1.000 | 2056 | tags=67%, list=17%, signal=80% |
| 1143 | GOCC\_RIBBON\_SYNAPSE |  | 6 | -0.57 | -1.20 | 0.267 | 0.766 | 1.000 | 3422 | tags=67%, list=28%, signal=93% |
| 1144 | GOBP\_POSITIVE\_REGULATION\_OF\_ANTIGEN\_RECEPTOR\_MEDIATED\_SIGNALING\_PATHWAY |  | 17 | -0.43 | -1.20 | 0.222 | 0.766 | 1.000 | 2063 | tags=41%, list=17%, signal=50% |
| 1145 | GOBP\_AXON\_CHOICE\_POINT\_RECOGNITION |  | 4 | -0.65 | -1.20 | 0.268 | 0.766 | 1.000 | 1489 | tags=50%, list=12%, signal=57% |
| 1146 | GOBP\_T\_CELL\_SELECTION |  | 33 | -0.36 | -1.20 | 0.187 | 0.766 | 1.000 | 3199 | tags=52%, list=27%, signal=70% |
| 1147 | GOBP\_REGULATION\_OF\_REACTIVE\_OXYGEN\_SPECIES\_METABOLIC\_PROCESS |  | 129 | -0.27 | -1.20 | 0.108 | 0.765 | 1.000 | 1445 | tags=22%, list=12%, signal=25% |
| 1148 | GOBP\_CELLULAR\_RESPONSE\_TO\_LEUCINE |  | 9 | -0.50 | -1.20 | 0.243 | 0.766 | 1.000 | 2417 | tags=44%, list=20%, signal=56% |
| 1149 | GOMF\_OXO\_ACID\_LYASE\_ACTIVITY |  | 5 | -0.61 | -1.20 | 0.273 | 0.767 | 1.000 | 2203 | tags=60%, list=18%, signal=73% |
| 1150 | GOBP\_COMPLEMENT\_RECEPTOR\_MEDIATED\_SIGNALING\_PATHWAY |  | 3 | -0.70 | -1.20 | 0.270 | 0.766 | 1.000 | 2995 | tags=67%, list=25%, signal=89% |
| 1151 | GOBP\_REGULATION\_OF\_RESPONSE\_TO\_OXIDATIVE\_STRESS |  | 60 | -0.31 | -1.20 | 0.174 | 0.766 | 1.000 | 1418 | tags=23%, list=12%, signal=26% |
| 1152 | GOBP\_HISTONE\_H3\_K9\_DIMETHYLATION |  | 4 | -0.65 | -1.20 | 0.273 | 0.768 | 1.000 | 3147 | tags=75%, list=26%, signal=102% |
| 1153 | GOBP\_ENERGY\_DERIVATION\_BY\_OXIDATION\_OF\_ORGANIC\_COMPOUNDS |  | 198 | -0.26 | -1.20 | 0.081 | 0.769 | 1.000 | 4286 | tags=46%, list=36%, signal=70% |
| 1154 | GOCC\_LAMELLAR\_BODY |  | 7 | -0.55 | -1.20 | 0.257 | 0.770 | 1.000 | 2949 | tags=71%, list=25%, signal=95% |
| 1155 | GOBP\_NEGATIVE\_REGULATION\_OF\_RELEASE\_OF\_CYTOCHROME\_C\_FROM\_MITOCHONDRIA |  | 12 | -0.46 | -1.20 | 0.233 | 0.769 | 1.000 | 3602 | tags=50%, list=30%, signal=71% |
| 1156 | GOBP\_ALPHA\_AMINO\_ACID\_CATABOLIC\_PROCESS |  | 62 | -0.31 | -1.20 | 0.161 | 0.769 | 1.000 | 2099 | tags=27%, list=17%, signal=33% |
| 1157 | GOBP\_NEGATIVE\_REGULATION\_OF\_RESPONSE\_TO\_OXIDATIVE\_STRESS |  | 16 | -0.42 | -1.20 | 0.227 | 0.769 | 1.000 | 1290 | tags=31%, list=11%, signal=35% |
| 1158 | GOBP\_POSITIVE\_REGULATION\_OF\_CYTOSOLIC\_CALCIUM\_ION\_CONCENTRATION\_INVOLVED\_IN\_PHOSPHOLIPASE\_C\_ACTIVATING\_G\_PROTEIN\_COUPLED\_SIGNALING\_PATHWAY |  | 25 | -0.38 | -1.20 | 0.212 | 0.768 | 1.000 | 1545 | tags=32%, list=13%, signal=37% |
| 1159 | GOBP\_LIPID\_HYDROXYLATION |  | 3 | -0.71 | -1.20 | 0.261 | 0.769 | 1.000 | 3521 | tags=100%, list=29%, signal=141% |
| 1160 | GOBP\_REGULATION\_OF\_PLATELET\_DERIVED\_GROWTH\_FACTOR\_RECEPTOR\_SIGNALING\_PATHWAY |  | 21 | -0.40 | -1.20 | 0.224 | 0.769 | 1.000 | 771 | tags=14%, list=6%, signal=15% |
| 1161 | GOBP\_CHAPERONE\_MEDIATED\_AUTOPHAGY |  | 13 | -0.45 | -1.19 | 0.237 | 0.770 | 1.000 | 2305 | tags=38%, list=19%, signal=48% |
| 1162 | GOMF\_BH\_DOMAIN\_BINDING |  | 8 | -0.52 | -1.19 | 0.259 | 0.770 | 1.000 | 1403 | tags=38%, list=12%, signal=42% |
| 1163 | GOBP\_REGULATION\_OF\_OSTEOCLAST\_DEVELOPMENT |  | 6 | -0.57 | -1.19 | 0.282 | 0.769 | 1.000 | 3161 | tags=83%, list=26%, signal=113% |
| 1164 | GOBP\_ESTROGEN\_BIOSYNTHETIC\_PROCESS |  | 5 | -0.61 | -1.19 | 0.280 | 0.769 | 1.000 | 322 | tags=40%, list=3%, signal=41% |
| 1165 | GOBP\_REGULATION\_OF\_ANIMAL\_ORGAN\_MORPHOGENESIS |  | 121 | -0.28 | -1.19 | 0.128 | 0.769 | 1.000 | 2284 | tags=21%, list=19%, signal=26% |
| 1166 | GOBP\_NEGATIVE\_REGULATION\_OF\_INTRINSIC\_APOPTOTIC\_SIGNALING\_PATHWAY |  | 66 | -0.31 | -1.19 | 0.165 | 0.768 | 1.000 | 3011 | tags=30%, list=25%, signal=40% |
| 1167 | GOCC\_INTRINSIC\_COMPONENT\_OF\_PLASMA\_MEMBRANE |  | 918 | -0.22 | -1.19 | 0.008 | 0.769 | 1.000 | 1725 | tags=19%, list=14%, signal=21% |
| 1168 | GOCC\_MRNA\_CLEAVAGE\_FACTOR\_COMPLEX |  | 17 | -0.42 | -1.19 | 0.231 | 0.769 | 1.000 | 3103 | tags=35%, list=26%, signal=48% |
| 1169 | GOCC\_INTRINSIC\_COMPONENT\_OF\_SYNAPTIC\_VESICLE\_MEMBRANE |  | 28 | -0.37 | -1.19 | 0.209 | 0.769 | 1.000 | 1257 | tags=21%, list=10%, signal=24% |
| 1170 | GOBP\_GLOMERULAR\_MESANGIAL\_CELL\_PROLIFERATION |  | 8 | -0.52 | -1.19 | 0.259 | 0.768 | 1.000 | 1051 | tags=25%, list=9%, signal=27% |
| 1171 | GOMF\_COPPER\_CHAPERONE\_ACTIVITY |  | 5 | -0.60 | -1.19 | 0.275 | 0.768 | 1.000 | 3508 | tags=80%, list=29%, signal=113% |
| 1172 | GOBP\_AROMATIC\_AMINO\_ACID\_FAMILY\_METABOLIC\_PROCESS |  | 17 | -0.42 | -1.19 | 0.232 | 0.767 | 1.000 | 3078 | tags=47%, list=26%, signal=63% |
| 1173 | GOMF\_UBIQUITINATION\_LIKE\_MODIFICATION\_DEPENDENT\_PROTEIN\_BINDING |  | 10 | -0.49 | -1.19 | 0.246 | 0.769 | 1.000 | 3639 | tags=50%, list=30%, signal=72% |
| 1174 | GOBP\_TRACHEA\_CARTILAGE\_DEVELOPMENT |  | 4 | -0.65 | -1.19 | 0.279 | 0.768 | 1.000 | 4191 | tags=75%, list=35%, signal=115% |
| 1175 | GOCC\_MKS\_COMPLEX |  | 12 | -0.47 | -1.19 | 0.249 | 0.769 | 1.000 | 3710 | tags=58%, list=31%, signal=84% |
| 1176 | GOBP\_NEGATIVE\_REGULATION\_OF\_PROTEIN\_DEPHOSPHORYLATION |  | 39 | -0.34 | -1.19 | 0.200 | 0.772 | 1.000 | 3754 | tags=46%, list=31%, signal=67% |
| 1177 | GOMF\_FLOPPASE\_ACTIVITY |  | 4 | -0.65 | -1.19 | 0.279 | 0.773 | 1.000 | 3447 | tags=75%, list=29%, signal=105% |
| 1178 | GOMF\_CARBOHYDRATE\_DERIVATIVE\_TRANSMEMBRANE\_TRANSPORTER\_ACTIVITY |  | 33 | -0.36 | -1.19 | 0.204 | 0.773 | 1.000 | 2985 | tags=52%, list=25%, signal=68% |
| 1179 | GOMF\_UBIQUITIN\_PROTEIN\_TRANSFERASE\_ACTIVATOR\_ACTIVITY |  | 7 | -0.55 | -1.19 | 0.275 | 0.773 | 1.000 | 3161 | tags=57%, list=26%, signal=78% |
| 1180 | GOBP\_REGULATION\_OF\_MITOCHONDRIAL\_FISSION |  | 21 | -0.40 | -1.19 | 0.230 | 0.772 | 1.000 | 1430 | tags=33%, list=12%, signal=38% |
| 1181 | GOBP\_CELLULAR\_AMINO\_ACID\_CATABOLIC\_PROCESS |  | 76 | -0.30 | -1.19 | 0.156 | 0.772 | 1.000 | 2025 | tags=26%, list=17%, signal=31% |
| 1182 | GOCC\_SMALL\_SUBUNIT\_PROCESSOME |  | 30 | -0.36 | -1.19 | 0.212 | 0.773 | 1.000 | 3796 | tags=53%, list=32%, signal=78% |
| 1183 | GOBP\_ANTIVIRAL\_INNATE\_IMMUNE\_RESPONSE |  | 3 | -0.70 | -1.19 | 0.272 | 0.773 | 1.000 | 3636 | tags=100%, list=30%, signal=143% |
| 1184 | GOCC\_PRESYNAPTIC\_CYTOSOL |  | 10 | -0.48 | -1.19 | 0.259 | 0.772 | 1.000 | 2699 | tags=40%, list=22%, signal=52% |
| 1185 | GOBP\_PROTEIN\_TARGETING\_TO\_MITOCHONDRION |  | 77 | -0.30 | -1.19 | 0.160 | 0.773 | 1.000 | 3411 | tags=43%, list=28%, signal=59% |
| 1186 | GOCC\_MITOCHONDRIAL\_OUTER\_MEMBRANE\_TRANSLOCASE\_COMPLEX |  | 5 | -0.60 | -1.19 | 0.273 | 0.772 | 1.000 | 1820 | tags=60%, list=15%, signal=71% |
| 1187 | GOBP\_ZINC\_ION\_IMPORT\_ACROSS\_PLASMA\_MEMBRANE |  | 4 | -0.64 | -1.19 | 0.286 | 0.772 | 1.000 | 1774 | tags=50%, list=15%, signal=59% |
| 1188 | GOBP\_REGULATION\_OF\_MRNA\_EXPORT\_FROM\_NUCLEUS |  | 5 | -0.60 | -1.19 | 0.288 | 0.773 | 1.000 | 4798 | tags=100%, list=40%, signal=166% |
| 1189 | GOBP\_CELLULAR\_RESPONSE\_TO\_CADMIUM\_ION |  | 17 | -0.42 | -1.19 | 0.243 | 0.773 | 1.000 | 660 | tags=18%, list=5%, signal=19% |
| 1190 | GOBP\_PROTEIN\_LOCALIZATION\_TO\_ORGANELLE |  | 763 | -0.23 | -1.19 | 0.014 | 0.773 | 1.000 | 3128 | tags=31%, list=26%, signal=39% |
| 1191 | GOBP\_TONGUE\_MORPHOGENESIS |  | 3 | -0.71 | -1.19 | 0.275 | 0.772 | 1.000 | 2317 | tags=67%, list=19%, signal=83% |
| 1192 | GOBP\_REGULATION\_OF\_PEPTIDYL\_LYSINE\_ACETYLATION |  | 49 | -0.33 | -1.19 | 0.189 | 0.773 | 1.000 | 2974 | tags=41%, list=25%, signal=54% |
| 1193 | GOBP\_REGULATION\_OF\_ANDROGEN\_RECEPTOR\_SIGNALING\_PATHWAY |  | 18 | -0.41 | -1.19 | 0.245 | 0.773 | 1.000 | 2505 | tags=33%, list=21%, signal=42% |
| 1194 | GOCC\_MITOCHONDRIAL\_LARGE\_RIBOSOMAL\_SUBUNIT |  | 50 | -0.32 | -1.19 | 0.186 | 0.774 | 1.000 | 4653 | tags=52%, list=39%, signal=85% |
| 1195 | GOMF\_RNA\_METHYLTRANSFERASE\_ACTIVITY |  | 52 | -0.32 | -1.19 | 0.200 | 0.773 | 1.000 | 3120 | tags=38%, list=26%, signal=52% |
| 1196 | GOBP\_SYNAPSE\_MATURATION |  | 16 | -0.42 | -1.19 | 0.245 | 0.775 | 1.000 | 2822 | tags=38%, list=23%, signal=49% |
| 1197 | GOBP\_POSTSYNAPSE\_TO\_NUCLEUS\_SIGNALING\_PATHWAY |  | 6 | -0.57 | -1.19 | 0.267 | 0.775 | 1.000 | 188 | tags=17%, list=2%, signal=17% |
| 1198 | GOBP\_CELLULAR\_HOMEOSTASIS |  | 606 | -0.23 | -1.19 | 0.018 | 0.775 | 1.000 | 1903 | tags=22%, list=16%, signal=24% |
| 1199 | GOBP\_NEUROTRANSMITTER\_SECRETION |  | 104 | -0.28 | -1.19 | 0.147 | 0.775 | 1.000 | 1711 | tags=25%, list=14%, signal=29% |
| 1200 | GOMF\_UBIQUITIN\_DEPENDENT\_PROTEIN\_BINDING |  | 10 | -0.49 | -1.19 | 0.265 | 0.775 | 1.000 | 3639 | tags=50%, list=30%, signal=72% |
| 1201 | GOMF\_COMPLEMENT\_RECEPTOR\_ACTIVITY |  | 3 | -0.70 | -1.18 | 0.276 | 0.775 | 1.000 | 2995 | tags=67%, list=25%, signal=89% |
| 1202 | GOMF\_G\_PROTEIN\_COUPLED\_RECEPTOR\_KINASE\_ACTIVITY |  | 5 | -0.59 | -1.18 | 0.287 | 0.775 | 1.000 | 4271 | tags=80%, list=36%, signal=124% |
| 1203 | GOBP\_REGULATION\_OF\_ODONTOGENESIS |  | 8 | -0.52 | -1.18 | 0.268 | 0.775 | 1.000 | 156 | tags=25%, list=1%, signal=25% |
| 1204 | GOBP\_HOMOPHILIC\_CELL\_ADHESION\_VIA\_PLASMA\_MEMBRANE\_ADHESION\_MOLECULES |  | 76 | -0.30 | -1.18 | 0.160 | 0.775 | 1.000 | 2334 | tags=32%, list=19%, signal=39% |
| 1205 | GOCC\_INCLUSION\_BODY |  | 57 | -0.32 | -1.18 | 0.175 | 0.775 | 1.000 | 3388 | tags=46%, list=28%, signal=63% |
| 1206 | GOBP\_NEGATIVE\_REGULATION\_OF\_MUSCLE\_CELL\_APOPTOTIC\_PROCESS |  | 21 | -0.40 | -1.18 | 0.222 | 0.775 | 1.000 | 629 | tags=19%, list=5%, signal=20% |
| 1207 | GOBP\_EXCRETION |  | 35 | -0.35 | -1.18 | 0.215 | 0.776 | 1.000 | 1396 | tags=26%, list=12%, signal=29% |
| 1208 | GOMF\_UBIQUINONE\_BINDING |  | 5 | -0.60 | -1.18 | 0.289 | 0.779 | 1.000 | 2036 | tags=40%, list=17%, signal=48% |
| 1209 | GOBP\_REGULATION\_OF\_INTRINSIC\_APOPTOTIC\_SIGNALING\_PATHWAY\_BY\_P53\_CLASS\_MEDIATOR |  | 17 | -0.42 | -1.18 | 0.242 | 0.780 | 1.000 | 2986 | tags=41%, list=25%, signal=55% |
| 1210 | GOBP\_VITAMIN\_B6\_METABOLIC\_PROCESS |  | 5 | -0.60 | -1.18 | 0.285 | 0.781 | 1.000 | 3261 | tags=60%, list=27%, signal=82% |
| 1211 | GOBP\_REGULATION\_OF\_VENTRICULAR\_CARDIAC\_MUSCLE\_CELL\_MEMBRANE\_DEPOLARIZATION |  | 4 | -0.64 | -1.18 | 0.297 | 0.782 | 1.000 | 713 | tags=25%, list=6%, signal=27% |
| 1212 | GOBP\_CELLULAR\_RESPONSE\_TO\_CAMP |  | 36 | -0.35 | -1.18 | 0.209 | 0.781 | 1.000 | 1003 | tags=17%, list=8%, signal=18% |
| 1213 | GOBP\_NEGATIVE\_REGULATION\_OF\_CELL\_KILLING |  | 6 | -0.57 | -1.18 | 0.280 | 0.781 | 1.000 | 5090 | tags=67%, list=42%, signal=116% |
| 1214 | GOBP\_KETONE\_CATABOLIC\_PROCESS |  | 8 | -0.52 | -1.18 | 0.273 | 0.782 | 1.000 | 2505 | tags=50%, list=21%, signal=63% |
| 1215 | GOMF\_ISOCITRATE\_DEHYDROGENASE\_ACTIVITY |  | 4 | -0.64 | -1.18 | 0.295 | 0.783 | 1.000 | 2591 | tags=50%, list=22%, signal=64% |
| 1216 | GOBP\_REGULATION\_OF\_SODIUM\_DEPENDENT\_PHOSPHATE\_TRANSPORT |  | 3 | -0.70 | -1.18 | 0.288 | 0.782 | 1.000 | 3407 | tags=67%, list=28%, signal=93% |
| 1217 | GOBP\_REGULATION\_OF\_VASCULAR\_ENDOTHELIAL\_GROWTH\_FACTOR\_RECEPTOR\_SIGNALING\_PATHWAY |  | 21 | -0.39 | -1.18 | 0.228 | 0.782 | 1.000 | 1418 | tags=24%, list=12%, signal=27% |
| 1218 | GOBP\_ROUNDABOUT\_SIGNALING\_PATHWAY |  | 5 | -0.59 | -1.18 | 0.283 | 0.781 | 1.000 | 197 | tags=20%, list=2%, signal=20% |
| 1219 | GOBP\_BEHAVIOR |  | 325 | -0.24 | -1.18 | 0.065 | 0.781 | 1.000 | 2320 | tags=25%, list=19%, signal=30% |
| 1220 | GOBP\_ISOCITRATE\_METABOLIC\_PROCESS |  | 4 | -0.64 | -1.18 | 0.296 | 0.783 | 1.000 | 2591 | tags=50%, list=22%, signal=64% |
| 1221 | GOBP\_NEURON\_DEATH\_IN\_RESPONSE\_TO\_HYDROGEN\_PEROXIDE |  | 3 | -0.70 | -1.18 | 0.284 | 0.783 | 1.000 | 2505 | tags=67%, list=21%, signal=84% |
| 1222 | GOMF\_HYDROLASE\_ACTIVITY\_ACTING\_ON\_CARBON\_NITROGEN\_BUT\_NOT\_PEPTIDE\_BONDS\_IN\_CYCLIC\_AMIDES |  | 4 | -0.64 | -1.18 | 0.301 | 0.783 | 1.000 | 4334 | tags=100%, list=36%, signal=156% |
| 1223 | GOCC\_EUKARYOTIC\_TRANSLATION\_INITIATION\_FACTOR\_2\_COMPLEX |  | 4 | -0.64 | -1.18 | 0.297 | 0.784 | 1.000 | 598 | tags=25%, list=5%, signal=26% |
| 1224 | GOBP\_METHYLGLYOXAL\_METABOLIC\_PROCESS |  | 5 | -0.60 | -1.18 | 0.279 | 0.784 | 1.000 | 2505 | tags=60%, list=21%, signal=76% |
| 1225 | GOBP\_DETECTION\_OF\_MOLECULE\_OF\_BACTERIAL\_ORIGIN |  | 6 | -0.56 | -1.18 | 0.289 | 0.784 | 1.000 | 4929 | tags=67%, list=41%, signal=113% |
| 1226 | GOBP\_POSITIVE\_REGULATION\_OF\_MAP\_KINASE\_ACTIVITY |  | 166 | -0.26 | -1.18 | 0.116 | 0.784 | 1.000 | 3022 | tags=30%, list=25%, signal=39% |
| 1227 | GOMF\_MONOCARBOXYLIC\_ACID\_BINDING |  | 40 | -0.33 | -1.18 | 0.210 | 0.784 | 1.000 | 1531 | tags=30%, list=13%, signal=34% |
| 1228 | GOBP\_NEGATIVE\_REGULATION\_OF\_NEURON\_APOPTOTIC\_PROCESS |  | 79 | -0.29 | -1.18 | 0.174 | 0.785 | 1.000 | 2881 | tags=34%, list=24%, signal=45% |
| 1229 | GOBP\_POSITIVE\_REGULATION\_OF\_STRIATED\_MUSCLE\_CONTRACTION |  | 5 | -0.60 | -1.18 | 0.301 | 0.784 | 1.000 | 13 | tags=20%, list=0%, signal=20% |
| 1230 | GOCC\_EXORIBONUCLEASE\_COMPLEX |  | 22 | -0.39 | -1.18 | 0.235 | 0.784 | 1.000 | 3209 | tags=36%, list=27%, signal=50% |
| 1231 | GOBP\_L\_ASCORBIC\_ACID\_METABOLIC\_PROCESS |  | 7 | -0.54 | -1.18 | 0.293 | 0.783 | 1.000 | 2785 | tags=71%, list=23%, signal=93% |
| 1232 | GOCC\_RESPIRATORY\_CHAIN\_COMPLEX\_IV |  | 8 | -0.51 | -1.18 | 0.279 | 0.783 | 1.000 | 4801 | tags=63%, list=40%, signal=104% |
| 1233 | GOBP\_TAURINE\_TRANSPORT |  | 5 | -0.59 | -1.18 | 0.292 | 0.783 | 1.000 | 228 | tags=20%, list=2%, signal=20% |
| 1234 | GOBP\_POSITIVE\_REGULATION\_OF\_ACTION\_POTENTIAL |  | 3 | -0.70 | -1.18 | 0.284 | 0.783 | 1.000 | 713 | tags=67%, list=6%, signal=71% |
| 1235 | GOCC\_PREFOLDIN\_COMPLEX |  | 5 | -0.60 | -1.18 | 0.289 | 0.783 | 1.000 | 3614 | tags=60%, list=30%, signal=86% |
| 1236 | GOBP\_POSITIVE\_REGULATION\_OF\_CARDIAC\_MUSCLE\_CONTRACTION |  | 4 | -0.64 | -1.18 | 0.295 | 0.782 | 1.000 | 13 | tags=25%, list=0%, signal=25% |
| 1237 | GOBP\_GLYCOSYL\_COMPOUND\_CATABOLIC\_PROCESS |  | 28 | -0.37 | -1.18 | 0.230 | 0.783 | 1.000 | 2825 | tags=50%, list=24%, signal=65% |
| 1238 | GOMF\_G\_PROTEIN\_COUPLED\_RECEPTOR\_ACTIVITY |  | 196 | -0.25 | -1.17 | 0.102 | 0.786 | 1.000 | 1627 | tags=23%, list=14%, signal=27% |
| 1239 | GOBP\_METANEPHRIC\_NEPHRON\_TUBULE\_MORPHOGENESIS |  | 5 | -0.60 | -1.17 | 0.295 | 0.786 | 1.000 | 141 | tags=20%, list=1%, signal=20% |
| 1240 | GOCC\_BOX\_H\_ACA\_TELOMERASE\_RNP\_COMPLEX |  | 3 | -0.69 | -1.17 | 0.294 | 0.788 | 1.000 | 621 | tags=33%, list=5%, signal=35% |
| 1241 | GOBP\_REGULATION\_OF\_NEUROTRANSMITTER\_LEVELS |  | 139 | -0.26 | -1.17 | 0.133 | 0.789 | 1.000 | 2656 | tags=33%, list=22%, signal=42% |
| 1242 | GOBP\_PHOSPHATIDYLGLYCEROL\_METABOLIC\_PROCESS |  | 24 | -0.38 | -1.17 | 0.231 | 0.791 | 1.000 | 1872 | tags=38%, list=16%, signal=44% |
| 1243 | GOCC\_CHLORIDE\_CHANNEL\_COMPLEX |  | 16 | -0.42 | -1.17 | 0.253 | 0.793 | 1.000 | 254 | tags=25%, list=2%, signal=26% |
| 1244 | GOBP\_PERIPHERAL\_NERVOUS\_SYSTEM\_MYELIN\_MAINTENANCE |  | 5 | -0.59 | -1.17 | 0.305 | 0.792 | 1.000 | 3727 | tags=60%, list=31%, signal=87% |
| 1245 | GOBP\_RENAL\_SODIUM\_EXCRETION |  | 10 | -0.48 | -1.17 | 0.279 | 0.792 | 1.000 | 12 | tags=10%, list=0%, signal=10% |
| 1246 | GOBP\_C21\_STEROID\_HORMONE\_METABOLIC\_PROCESS |  | 22 | -0.39 | -1.17 | 0.235 | 0.792 | 1.000 | 1455 | tags=27%, list=12%, signal=31% |
| 1247 | GOBP\_REGULATION\_OF\_SYSTEMIC\_ARTERIAL\_BLOOD\_PRESSURE\_MEDIATED\_BY\_A\_CHEMICAL\_SIGNAL |  | 31 | -0.36 | -1.17 | 0.227 | 0.792 | 1.000 | 1514 | tags=26%, list=13%, signal=29% |
| 1248 | GOMF\_RRNA\_ADENINE\_METHYLTRANSFERASE\_ACTIVITY |  | 9 | -0.50 | -1.17 | 0.278 | 0.793 | 1.000 | 5088 | tags=78%, list=42%, signal=135% |
| 1249 | GOBP\_POSITIVE\_REGULATION\_OF\_PEPTIDYL\_LYSINE\_ACETYLATION |  | 23 | -0.38 | -1.17 | 0.246 | 0.793 | 1.000 | 2908 | tags=48%, list=24%, signal=63% |
| 1250 | GOBP\_BRANCHING\_INVOLVED\_IN\_SALIVARY\_GLAND\_MORPHOGENESIS |  | 18 | -0.41 | -1.17 | 0.260 | 0.793 | 1.000 | 605 | tags=17%, list=5%, signal=18% |
| 1251 | GOCC\_H4\_HISTONE\_ACETYLTRANSFERASE\_COMPLEX |  | 39 | -0.34 | -1.17 | 0.218 | 0.792 | 1.000 | 3869 | tags=38%, list=32%, signal=57% |
| 1252 | GOCC\_MICROBODY |  | 109 | -0.28 | -1.17 | 0.164 | 0.793 | 1.000 | 1915 | tags=27%, list=16%, signal=31% |
| 1253 | GOBP\_EMBRYONIC\_MORPHOGENESIS |  | 366 | -0.23 | -1.17 | 0.069 | 0.793 | 1.000 | 2363 | tags=25%, list=20%, signal=30% |
| 1254 | GOBP\_FOREBRAIN\_RADIAL\_GLIAL\_CELL\_DIFFERENTIATION |  | 5 | -0.59 | -1.17 | 0.294 | 0.794 | 1.000 | 3362 | tags=60%, list=28%, signal=83% |
| 1255 | GOCC\_POSTSYNAPTIC\_SPECIALIZATION\_MEMBRANE |  | 46 | -0.32 | -1.17 | 0.209 | 0.793 | 1.000 | 2634 | tags=33%, list=22%, signal=42% |
| 1256 | GOMF\_MITOCHONDRION\_TARGETING\_SEQUENCE\_BINDING |  | 4 | -0.63 | -1.17 | 0.306 | 0.794 | 1.000 | 2134 | tags=75%, list=18%, signal=91% |
| 1257 | GOBP\_MRNA\_METABOLIC\_PROCESS |  | 647 | -0.22 | -1.17 | 0.027 | 0.796 | 1.000 | 2480 | tags=21%, list=21%, signal=25% |
| 1258 | GOBP\_SYNAPTIC\_SIGNALING |  | 398 | -0.23 | -1.17 | 0.055 | 0.795 | 1.000 | 1717 | tags=20%, list=14%, signal=22% |
| 1259 | GOMF\_XENOBIOTIC\_TRANSMEMBRANE\_TRANSPORTER\_ACTIVITY |  | 22 | -0.38 | -1.17 | 0.237 | 0.795 | 1.000 | 2985 | tags=55%, list=25%, signal=72% |
| 1260 | GOBP\_REGULATION\_OF\_VESICLE\_FUSION |  | 15 | -0.42 | -1.17 | 0.263 | 0.796 | 1.000 | 1353 | tags=33%, list=11%, signal=38% |
| 1261 | GOBP\_RESPONSE\_TO\_FUNGUS |  | 18 | -0.40 | -1.17 | 0.263 | 0.795 | 1.000 | 2874 | tags=44%, list=24%, signal=58% |
| 1262 | GOBP\_POSITIVE\_REGULATION\_OF\_HUMORAL\_IMMUNE\_RESPONSE |  | 9 | -0.49 | -1.17 | 0.287 | 0.796 | 1.000 | 3636 | tags=67%, list=30%, signal=96% |
| 1263 | GOBP\_PYRIMIDINE\_RIBONUCLEOTIDE\_BIOSYNTHETIC\_PROCESS |  | 17 | -0.41 | -1.17 | 0.250 | 0.795 | 1.000 | 834 | tags=35%, list=7%, signal=38% |
| 1264 | GOCC\_INTEGRAL\_COMPONENT\_OF\_SYNAPTIC\_VESICLE\_MEMBRANE |  | 18 | -0.41 | -1.17 | 0.259 | 0.796 | 1.000 | 1169 | tags=22%, list=10%, signal=25% |
| 1265 | GOBP\_IRON\_ION\_TRANSPORT |  | 55 | -0.31 | -1.17 | 0.207 | 0.796 | 1.000 | 2527 | tags=33%, list=21%, signal=41% |
| 1266 | GOMF\_POTASSIUM\_ION\_TRANSMEMBRANE\_TRANSPORTER\_ACTIVITY |  | 78 | -0.29 | -1.17 | 0.190 | 0.796 | 1.000 | 1943 | tags=27%, list=16%, signal=32% |
| 1267 | GOMF\_MACROLIDE\_BINDING |  | 8 | -0.51 | -1.17 | 0.296 | 0.796 | 1.000 | 5344 | tags=75%, list=44%, signal=135% |
| 1268 | GOMF\_FK506\_BINDING |  | 7 | -0.53 | -1.17 | 0.293 | 0.795 | 1.000 | 1924 | tags=29%, list=16%, signal=34% |
| 1269 | GOBP\_DNA\_DEALKYLATION |  | 20 | -0.39 | -1.16 | 0.249 | 0.796 | 1.000 | 2402 | tags=50%, list=20%, signal=62% |
| 1270 | GOBP\_REGULATION\_OF\_GLOMERULAR\_FILTRATION |  | 9 | -0.49 | -1.16 | 0.292 | 0.796 | 1.000 | 1630 | tags=33%, list=14%, signal=39% |
| 1271 | GOBP\_POSITIVE\_REGULATION\_OF\_G1\_S\_TRANSITION\_OF\_MITOTIC\_CELL\_CYCLE |  | 28 | -0.36 | -1.16 | 0.250 | 0.798 | 1.000 | 2417 | tags=32%, list=20%, signal=40% |
| 1272 | GOCC\_SUPER\_ELONGATION\_COMPLEX |  | 5 | -0.59 | -1.16 | 0.298 | 0.799 | 1.000 | 1254 | tags=40%, list=10%, signal=45% |
| 1273 | GOBP\_SEQUESTERING\_OF\_EXTRACELLULAR\_LIGAND\_FROM\_RECEPTOR |  | 7 | -0.53 | -1.16 | 0.298 | 0.798 | 1.000 | 2211 | tags=57%, list=18%, signal=70% |
| 1274 | GOMF\_GPI\_LINKED\_EPHRIN\_RECEPTOR\_ACTIVITY |  | 4 | -0.63 | -1.16 | 0.312 | 0.800 | 1.000 | 27 | tags=25%, list=0%, signal=25% |
| 1275 | GOBP\_RDNA\_HETEROCHROMATIN\_ASSEMBLY |  | 7 | -0.53 | -1.16 | 0.287 | 0.799 | 1.000 | 4125 | tags=71%, list=34%, signal=109% |
| 1276 | GOMF\_OXIDOREDUCTASE\_ACTIVITY\_ACTING\_ON\_THE\_CH\_CH\_GROUP\_OF\_DONORS |  | 48 | -0.32 | -1.16 | 0.211 | 0.798 | 1.000 | 1831 | tags=29%, list=15%, signal=34% |
| 1277 | GOBP\_OLFACTORY\_BULB\_INTERNEURON\_DIFFERENTIATION |  | 5 | -0.59 | -1.16 | 0.309 | 0.799 | 1.000 | 1489 | tags=40%, list=12%, signal=46% |
| 1278 | GOBP\_RENAL\_SYSTEM\_PROCESS\_INVOLVED\_IN\_REGULATION\_OF\_BLOOD\_VOLUME |  | 9 | -0.49 | -1.16 | 0.281 | 0.800 | 1.000 | 1630 | tags=33%, list=14%, signal=39% |
| 1279 | GOBP\_MYOFIBRIL\_ASSEMBLY |  | 33 | -0.35 | -1.16 | 0.236 | 0.801 | 1.000 | 1269 | tags=18%, list=11%, signal=20% |
| 1280 | GOMF\_S\_METHYLTRANSFERASE\_ACTIVITY |  | 4 | -0.63 | -1.16 | 0.315 | 0.801 | 1.000 | 2377 | tags=75%, list=20%, signal=93% |
| 1281 | GOBP\_RESPONSE\_TO\_OXYGEN\_RADICAL |  | 17 | -0.41 | -1.16 | 0.268 | 0.800 | 1.000 | 1188 | tags=29%, list=10%, signal=33% |
| 1282 | GOBP\_REGULATION\_OF\_MELANOCYTE\_DIFFERENTIATION |  | 5 | -0.59 | -1.16 | 0.305 | 0.801 | 1.000 | 1449 | tags=40%, list=12%, signal=45% |
| 1283 | GOBP\_BRADYKININ\_CATABOLIC\_PROCESS |  | 5 | -0.59 | -1.16 | 0.309 | 0.801 | 1.000 | 3216 | tags=60%, list=27%, signal=82% |
| 1284 | GOBP\_VASOCONSTRICTION |  | 62 | -0.30 | -1.16 | 0.198 | 0.800 | 1.000 | 1514 | tags=24%, list=13%, signal=28% |
| 1285 | GOCC\_MAST\_CELL\_GRANULE |  | 3 | -0.68 | -1.16 | 0.318 | 0.801 | 1.000 | 44 | tags=33%, list=0%, signal=33% |
| 1286 | GOMF\_PROTEIN\_CARBOXYL\_O\_METHYLTRANSFERASE\_ACTIVITY |  | 6 | -0.56 | -1.16 | 0.303 | 0.802 | 1.000 | 1761 | tags=33%, list=15%, signal=39% |
| 1287 | GOMF\_PSEUDOURIDINE\_SYNTHASE\_ACTIVITY |  | 11 | -0.46 | -1.16 | 0.283 | 0.803 | 1.000 | 3180 | tags=55%, list=26%, signal=74% |
| 1288 | GOBP\_SYNAPSE\_ASSEMBLY |  | 94 | -0.28 | -1.16 | 0.171 | 0.802 | 1.000 | 2822 | tags=33%, list=23%, signal=43% |
| 1289 | GOBP\_NUCLEOLUS\_ORGANIZATION |  | 5 | -0.59 | -1.16 | 0.309 | 0.803 | 1.000 | 2925 | tags=60%, list=24%, signal=79% |
| 1290 | GOCC\_GATOR2\_COMPLEX |  | 9 | -0.49 | -1.16 | 0.286 | 0.803 | 1.000 | 2738 | tags=44%, list=23%, signal=58% |
| 1291 | GOMF\_TRNA\_METHYLTRANSFERASE\_ACTIVITY |  | 27 | -0.36 | -1.16 | 0.248 | 0.804 | 1.000 | 3120 | tags=37%, list=26%, signal=50% |
| 1292 | GOCC\_INTRINSIC\_COMPONENT\_OF\_POSTSYNAPTIC\_DENSITY\_MEMBRANE |  | 21 | -0.39 | -1.16 | 0.263 | 0.803 | 1.000 | 2479 | tags=33%, list=21%, signal=42% |
| 1293 | GOBP\_POSITIVE\_REGULATION\_OF\_NEUROGENESIS |  | 157 | -0.26 | -1.16 | 0.141 | 0.805 | 1.000 | 2881 | tags=29%, list=24%, signal=38% |
| 1294 | GOBP\_RESPONSE\_TO\_VITAMIN\_D |  | 23 | -0.38 | -1.16 | 0.252 | 0.804 | 1.000 | 1012 | tags=30%, list=8%, signal=33% |
| 1295 | GOMF\_PEROXISOME\_TARGETING\_SEQUENCE\_BINDING |  | 3 | -0.69 | -1.16 | 0.307 | 0.805 | 1.000 | 1896 | tags=33%, list=16%, signal=40% |
| 1296 | GOBP\_MITOCHONDRIAL\_RNA\_3\_END\_PROCESSING |  | 5 | -0.59 | -1.16 | 0.311 | 0.805 | 1.000 | 4309 | tags=80%, list=36%, signal=125% |
| 1297 | GOBP\_REGULATION\_OF\_INORGANIC\_ANION\_TRANSMEMBRANE\_TRANSPORT |  | 4 | -0.63 | -1.16 | 0.316 | 0.805 | 1.000 | 3485 | tags=75%, list=29%, signal=106% |
| 1298 | GOCC\_ESC\_E\_Z\_COMPLEX |  | 12 | -0.45 | -1.16 | 0.275 | 0.804 | 1.000 | 2317 | tags=42%, list=19%, signal=52% |
| 1299 | GOBP\_SPLICEOSOMAL\_TRI\_SNRNP\_COMPLEX\_ASSEMBLY |  | 9 | -0.49 | -1.16 | 0.285 | 0.804 | 1.000 | 848 | tags=22%, list=7%, signal=24% |
| 1300 | GOCC\_CYTOPLASMIC\_SIDE\_OF\_ENDOPLASMIC\_RETICULUM\_MEMBRANE |  | 13 | -0.44 | -1.16 | 0.281 | 0.804 | 1.000 | 1679 | tags=31%, list=14%, signal=36% |
| 1301 | GOMF\_PYRUVATE\_DEHYDROGENASE\_ACTIVITY |  | 5 | -0.58 | -1.16 | 0.320 | 0.804 | 1.000 | 4007 | tags=60%, list=33%, signal=90% |
| 1302 | GOBP\_SERINE\_FAMILY\_AMINO\_ACID\_METABOLIC\_PROCESS |  | 29 | -0.35 | -1.16 | 0.240 | 0.805 | 1.000 | 2099 | tags=31%, list=17%, signal=38% |
| 1303 | GOBP\_PERICARDIUM\_MORPHOGENESIS |  | 8 | -0.51 | -1.16 | 0.296 | 0.805 | 1.000 | 350 | tags=25%, list=3%, signal=26% |
| 1304 | GOMF\_CARDIOLIPIN\_BINDING |  | 7 | -0.53 | -1.16 | 0.306 | 0.805 | 1.000 | 700 | tags=29%, list=6%, signal=30% |
| 1305 | GOBP\_DNA\_METHYLATION\_DEPENDENT\_HETEROCHROMATIN\_ASSEMBLY |  | 12 | -0.45 | -1.15 | 0.274 | 0.805 | 1.000 | 2579 | tags=42%, list=21%, signal=53% |
| 1306 | GOBP\_POSITIVE\_REGULATION\_OF\_MUSCLE\_ADAPTATION |  | 4 | -0.62 | -1.15 | 0.324 | 0.805 | 1.000 | 437 | tags=25%, list=4%, signal=26% |
| 1307 | GOMF\_3\_HYDROXYACYL\_COA\_DEHYDRATASE\_ACTIVITY |  | 5 | -0.58 | -1.15 | 0.316 | 0.806 | 1.000 | 2083 | tags=40%, list=17%, signal=48% |
| 1308 | GOMF\_OXIDOREDUCTASE\_ACTIVITY\_ACTING\_ON\_NAD\_P\_H |  | 73 | -0.29 | -1.15 | 0.202 | 0.807 | 1.000 | 4173 | tags=47%, list=35%, signal=71% |
| 1309 | GOBP\_TOR\_SIGNALING |  | 93 | -0.28 | -1.15 | 0.195 | 0.807 | 1.000 | 3108 | tags=34%, list=26%, signal=46% |
| 1310 | GOBP\_SPERM\_EGG\_RECOGNITION |  | 21 | -0.38 | -1.15 | 0.251 | 0.808 | 1.000 | 651 | tags=14%, list=5%, signal=15% |
| 1311 | GOCC\_SPERM\_PRINCIPAL\_PIECE |  | 9 | -0.49 | -1.15 | 0.288 | 0.810 | 1.000 | 2719 | tags=44%, list=23%, signal=57% |
| 1312 | GOBP\_RRNA\_5\_END\_PROCESSING |  | 3 | -0.69 | -1.15 | 0.324 | 0.810 | 1.000 | 1456 | tags=33%, list=12%, signal=38% |
| 1313 | GOMF\_N\_ACYLSPHINGOSINE\_AMIDOHYDROLASE\_ACTIVITY |  | 5 | -0.59 | -1.15 | 0.313 | 0.809 | 1.000 | 762 | tags=40%, list=6%, signal=43% |
| 1314 | GOBP\_REGULATION\_OF\_MEMBRANE\_POTENTIAL |  | 231 | -0.24 | -1.15 | 0.114 | 0.809 | 1.000 | 1472 | tags=19%, list=12%, signal=21% |
| 1315 | GOBP\_LYTIC\_VACUOLE\_ORGANIZATION |  | 55 | -0.31 | -1.15 | 0.222 | 0.811 | 1.000 | 3037 | tags=38%, list=25%, signal=51% |
| 1316 | GOMF\_PHOSPHOTRANSFERASE\_ACTIVITY\_NITROGENOUS\_GROUP\_AS\_ACCEPTOR |  | 4 | -0.63 | -1.15 | 0.323 | 0.811 | 1.000 | 792 | tags=25%, list=7%, signal=27% |
| 1317 | GOBP\_SPERMIDINE\_METABOLIC\_PROCESS |  | 5 | -0.59 | -1.15 | 0.314 | 0.811 | 1.000 | 1791 | tags=40%, list=15%, signal=47% |
| 1318 | GOBP\_NEGATIVE\_REGULATION\_OF\_PROTEASOMAL\_UBIQUITIN\_DEPENDENT\_PROTEIN\_CATABOLIC\_PROCESS |  | 27 | -0.36 | -1.15 | 0.257 | 0.811 | 1.000 | 2505 | tags=26%, list=21%, signal=33% |
| 1319 | GOBP\_POSITIVE\_REGULATION\_OF\_VASCULATURE\_DEVELOPMENT |  | 109 | -0.27 | -1.15 | 0.172 | 0.810 | 1.000 | 2798 | tags=37%, list=23%, signal=47% |
| 1320 | GOBP\_POSITIVE\_REGULATION\_OF\_GUANYLATE\_CYCLASE\_ACTIVITY |  | 3 | -0.68 | -1.15 | 0.322 | 0.810 | 1.000 | 2936 | tags=67%, list=24%, signal=88% |
| 1321 | GOCC\_CHAPERONIN\_CONTAINING\_T\_COMPLEX |  | 8 | -0.51 | -1.15 | 0.302 | 0.810 | 1.000 | 5474 | tags=88%, list=46%, signal=161% |
| 1322 | GOBP\_REGULATION\_OF\_CARDIAC\_MUSCLE\_CELL\_MEMBRANE\_REPOLARIZATION |  | 16 | -0.41 | -1.15 | 0.280 | 0.809 | 1.000 | 728 | tags=19%, list=6%, signal=20% |
| 1323 | GOBP\_POSITIVE\_REGULATION\_OF\_ANOIKIS |  | 4 | -0.63 | -1.15 | 0.333 | 0.809 | 1.000 | 1735 | tags=75%, list=14%, signal=88% |
| 1324 | GOBP\_DETECTION\_OF\_TEMPERATURE\_STIMULUS |  | 11 | -0.46 | -1.15 | 0.288 | 0.809 | 1.000 | 970 | tags=36%, list=8%, signal=40% |
| 1325 | GOBP\_NEGATIVE\_REGULATION\_OF\_RECEPTOR\_INTERNALIZATION |  | 9 | -0.49 | -1.15 | 0.310 | 0.809 | 1.000 | 3639 | tags=44%, list=30%, signal=64% |
| 1326 | GOBP\_NEGATIVE\_REGULATION\_OF\_EXTRACELLULAR\_MATRIX\_ORGANIZATION |  | 9 | -0.49 | -1.15 | 0.298 | 0.809 | 1.000 | 2040 | tags=44%, list=17%, signal=53% |
| 1327 | GOCC\_RIBONUCLEASE\_P\_COMPLEX |  | 8 | -0.50 | -1.15 | 0.300 | 0.809 | 1.000 | 4530 | tags=63%, list=38%, signal=100% |
| 1328 | GOBP\_INTERLEUKIN\_13\_PRODUCTION |  | 9 | -0.49 | -1.15 | 0.296 | 0.808 | 1.000 | 1380 | tags=33%, list=11%, signal=38% |
| 1329 | GOBP\_REGULATION\_OF\_SKELETAL\_MUSCLE\_TISSUE\_GROWTH |  | 6 | -0.55 | -1.15 | 0.307 | 0.809 | 1.000 | 38 | tags=17%, list=0%, signal=17% |
| 1330 | GOBP\_DISTAL\_TUBULE\_DEVELOPMENT |  | 6 | -0.55 | -1.15 | 0.325 | 0.809 | 1.000 | 4056 | tags=50%, list=34%, signal=75% |
| 1331 | GOBP\_CELLULAR\_RESPONSE\_TO\_ETHER |  | 5 | -0.59 | -1.15 | 0.318 | 0.809 | 1.000 | 4976 | tags=100%, list=41%, signal=171% |
| 1332 | GOBP\_REGULATION\_OF\_CYTOSOLIC\_CALCIUM\_ION\_CONCENTRATION |  | 209 | -0.25 | -1.15 | 0.136 | 0.809 | 1.000 | 1545 | tags=21%, list=13%, signal=24% |
| 1333 | GOMF\_IRON\_ION\_BINDING |  | 87 | -0.28 | -1.15 | 0.188 | 0.810 | 1.000 | 1055 | tags=22%, list=9%, signal=24% |
| 1334 | GOMF\_PROSTANOID\_RECEPTOR\_ACTIVITY |  | 7 | -0.53 | -1.15 | 0.304 | 0.811 | 1.000 | 3297 | tags=57%, list=27%, signal=79% |
| 1335 | GOCC\_CLATHRIN\_COATED\_VESICLE |  | 126 | -0.26 | -1.15 | 0.164 | 0.812 | 1.000 | 1747 | tags=19%, list=15%, signal=22% |
| 1336 | GOBP\_ACTIVATION\_OF\_PROTEIN\_KINASE\_C\_ACTIVITY |  | 4 | -0.62 | -1.15 | 0.332 | 0.815 | 1.000 | 3636 | tags=75%, list=30%, signal=108% |
| 1337 | GOMF\_HISTONE\_ACETYLTRANSFERASE\_ACTIVITY\_H3\_K23\_SPECIFIC |  | 5 | -0.58 | -1.15 | 0.330 | 0.815 | 1.000 | 1929 | tags=60%, list=16%, signal=71% |
| 1338 | GOMF\_S\_ADENOSYL\_L\_METHIONINE\_BINDING |  | 13 | -0.43 | -1.15 | 0.292 | 0.815 | 1.000 | 1141 | tags=23%, list=9%, signal=25% |
| 1339 | GOCC\_MICOS\_COMPLEX |  | 7 | -0.52 | -1.14 | 0.312 | 0.816 | 1.000 | 3110 | tags=43%, list=26%, signal=58% |
| 1340 | GOBP\_REGULATION\_OF\_SNRNA\_TRANSCRIPTION\_BY\_RNA\_POLYMERASE\_II |  | 4 | -0.62 | -1.14 | 0.332 | 0.816 | 1.000 | 4086 | tags=75%, list=34%, signal=114% |
| 1341 | GOMF\_COMPLEMENT\_COMPONENT\_C3B\_BINDING |  | 4 | -0.62 | -1.14 | 0.332 | 0.816 | 1.000 | 4071 | tags=75%, list=34%, signal=113% |
| 1342 | GOBP\_POSITIVE\_REGULATION\_OF\_WNT\_SIGNALING\_PATHWAY\_PLANAR\_CELL\_POLARITY\_PATHWAY |  | 7 | -0.53 | -1.14 | 0.311 | 0.816 | 1.000 | 2230 | tags=43%, list=19%, signal=53% |
| 1343 | GOBP\_CEREBELLAR\_PURKINJE\_CELL\_GRANULE\_CELL\_PRECURSOR\_CELL\_SIGNALING\_INVOLVED\_IN\_REGULATION\_OF\_GRANULE\_CELL\_PRECURSOR\_CELL\_PROLIFERATION |  | 5 | -0.58 | -1.14 | 0.330 | 0.815 | 1.000 | 4988 | tags=80%, list=42%, signal=137% |
| 1344 | GOBP\_HISTONE\_H3\_K23\_ACETYLATION |  | 5 | -0.58 | -1.14 | 0.330 | 0.815 | 1.000 | 1929 | tags=60%, list=16%, signal=71% |
| 1345 | GOCC\_CUL5\_RING\_UBIQUITIN\_LIGASE\_COMPLEX |  | 4 | -0.62 | -1.14 | 0.338 | 0.815 | 1.000 | 3792 | tags=75%, list=32%, signal=110% |
| 1346 | GOBP\_POSITIVE\_REGULATION\_OF\_HEART\_RATE |  | 18 | -0.40 | -1.14 | 0.274 | 0.815 | 1.000 | 363 | tags=17%, list=3%, signal=17% |
| 1347 | GOBP\_DICARBOXYLIC\_ACID\_CATABOLIC\_PROCESS |  | 12 | -0.45 | -1.14 | 0.299 | 0.814 | 1.000 | 1710 | tags=33%, list=14%, signal=39% |
| 1348 | GOBP\_REGULATION\_OF\_TUBE\_SIZE |  | 91 | -0.28 | -1.14 | 0.199 | 0.815 | 1.000 | 1514 | tags=22%, list=13%, signal=25% |
| 1349 | GOCC\_SCHMIDT\_LANTERMAN\_INCISURE |  | 8 | -0.50 | -1.14 | 0.301 | 0.815 | 1.000 | 44 | tags=13%, list=0%, signal=13% |
| 1350 | GOBP\_HEPARAN\_SULFATE\_PROTEOGLYCAN\_BIOSYNTHETIC\_PROCESS\_ENZYMATIC\_MODIFICATION |  | 4 | -0.62 | -1.14 | 0.340 | 0.815 | 1.000 | 1162 | tags=75%, list=10%, signal=83% |
| 1351 | GOBP\_PURINE\_RIBONUCLEOSIDE\_SALVAGE |  | 5 | -0.59 | -1.14 | 0.332 | 0.815 | 1.000 | 2219 | tags=40%, list=18%, signal=49% |
| 1352 | GOBP\_TONGUE\_DEVELOPMENT |  | 10 | -0.47 | -1.14 | 0.306 | 0.814 | 1.000 | 2317 | tags=40%, list=19%, signal=50% |
| 1353 | GOBP\_CELLULAR\_RESPONSE\_TO\_OXYGEN\_RADICAL |  | 15 | -0.42 | -1.14 | 0.285 | 0.815 | 1.000 | 1188 | tags=33%, list=10%, signal=37% |
| 1354 | GOMF\_OXYGEN\_BINDING |  | 15 | -0.42 | -1.14 | 0.291 | 0.815 | 1.000 | 484 | tags=20%, list=4%, signal=21% |
| 1355 | GOBP\_GENERATION\_OF\_PRECURSOR\_METABOLITES\_AND\_ENERGY |  | 350 | -0.23 | -1.14 | 0.085 | 0.815 | 1.000 | 2886 | tags=27%, list=24%, signal=35% |
| 1356 | GOBP\_NEGATIVE\_REGULATION\_OF\_INTERLEUKIN\_12\_PRODUCTION |  | 7 | -0.52 | -1.14 | 0.315 | 0.815 | 1.000 | 3883 | tags=57%, list=32%, signal=84% |
| 1357 | GOBP\_POSITIVE\_REGULATION\_OF\_TRANSCRIPTION\_FROM\_RNA\_POLYMERASE\_II\_PROMOTER\_IN\_RESPONSE\_TO\_STRESS |  | 18 | -0.40 | -1.14 | 0.271 | 0.814 | 1.000 | 1418 | tags=33%, list=12%, signal=38% |
| 1358 | GOBP\_SKELETAL\_MUSCLE\_TISSUE\_GROWTH |  | 6 | -0.55 | -1.14 | 0.324 | 0.814 | 1.000 | 38 | tags=17%, list=0%, signal=17% |
| 1359 | GOBP\_POSITIVE\_REGULATION\_OF\_GLIOGENESIS |  | 45 | -0.32 | -1.14 | 0.244 | 0.814 | 1.000 | 2881 | tags=36%, list=24%, signal=47% |
| 1360 | GOMF\_CALCIUM\_POTASSIUM\_SODIUM\_ANTIPORTER\_ACTIVITY |  | 3 | -0.68 | -1.14 | 0.335 | 0.815 | 1.000 | 3865 | tags=100%, list=32%, signal=147% |
| 1361 | GOBP\_POSTSYNAPTIC\_SIGNAL\_TRANSDUCTION |  | 24 | -0.37 | -1.14 | 0.266 | 0.815 | 1.000 | 2530 | tags=29%, list=21%, signal=37% |
| 1362 | GOBP\_POTASSIUM\_ION\_EXPORT\_ACROSS\_PLASMA\_MEMBRANE |  | 9 | -0.49 | -1.14 | 0.309 | 0.815 | 1.000 | 1396 | tags=33%, list=12%, signal=38% |
| 1363 | GOBP\_MITOCHONDRIAL\_RNA\_CATABOLIC\_PROCESS |  | 6 | -0.55 | -1.14 | 0.323 | 0.814 | 1.000 | 4689 | tags=83%, list=39%, signal=137% |
| 1364 | GOBP\_NEGATIVE\_REGULATION\_BY\_HOST\_OF\_VIRAL\_PROCESS |  | 9 | -0.48 | -1.14 | 0.302 | 0.814 | 1.000 | 2516 | tags=44%, list=21%, signal=56% |
| 1365 | GOBP\_DOPAMINE\_CATABOLIC\_PROCESS |  | 3 | -0.67 | -1.14 | 0.340 | 0.814 | 1.000 | 1044 | tags=33%, list=9%, signal=36% |
| 1366 | GOBP\_PROTEIN\_LOCALIZATION\_TO\_AXON |  | 5 | -0.58 | -1.14 | 0.332 | 0.813 | 1.000 | 154 | tags=20%, list=1%, signal=20% |
| 1367 | GOBP\_MESONEPHROS\_DEVELOPMENT |  | 66 | -0.29 | -1.14 | 0.222 | 0.814 | 1.000 | 669 | tags=14%, list=6%, signal=14% |
| 1368 | GOBP\_POSITIVE\_REGULATION\_OF\_POTASSIUM\_ION\_TRANSMEMBRANE\_TRANSPORT |  | 21 | -0.38 | -1.14 | 0.272 | 0.813 | 1.000 | 894 | tags=19%, list=7%, signal=21% |
| 1369 | GOMF\_U4\_SNRNA\_BINDING |  | 3 | -0.67 | -1.14 | 0.349 | 0.814 | 1.000 | 3968 | tags=100%, list=33%, signal=149% |
| 1370 | GOMF\_BH3\_DOMAIN\_BINDING |  | 4 | -0.63 | -1.14 | 0.337 | 0.814 | 1.000 | 1403 | tags=50%, list=12%, signal=57% |
| 1371 | GOBP\_ICOSANOID\_CATABOLIC\_PROCESS |  | 3 | -0.67 | -1.14 | 0.328 | 0.814 | 1.000 | 2005 | tags=67%, list=17%, signal=80% |
| 1372 | GOBP\_CIRCADIAN\_SLEEP\_WAKE\_CYCLE\_SLEEP |  | 10 | -0.47 | -1.14 | 0.305 | 0.814 | 1.000 | 1677 | tags=40%, list=14%, signal=46% |
| 1373 | GOBP\_MAINTENANCE\_OF\_DNA\_METHYLATION |  | 6 | -0.55 | -1.14 | 0.321 | 0.815 | 1.000 | 1945 | tags=33%, list=16%, signal=40% |
| 1374 | GOMF\_OPIOID\_RECEPTOR\_BINDING |  | 5 | -0.57 | -1.14 | 0.336 | 0.815 | 1.000 | 731 | tags=20%, list=6%, signal=21% |
| 1375 | GOBP\_KIDNEY\_EPITHELIUM\_DEVELOPMENT |  | 93 | -0.27 | -1.14 | 0.202 | 0.814 | 1.000 | 2042 | tags=23%, list=17%, signal=27% |
| 1376 | GOBP\_ANTIMICROBIAL\_HUMORAL\_RESPONSE |  | 28 | -0.36 | -1.14 | 0.277 | 0.816 | 1.000 | 3222 | tags=46%, list=27%, signal=63% |
| 1377 | GOBP\_NEGATIVE\_REGULATION\_OF\_BLOOD\_CIRCULATION |  | 13 | -0.43 | -1.14 | 0.297 | 0.816 | 1.000 | 12 | tags=8%, list=0%, signal=8% |
| 1378 | GOMF\_NAD\_DEPENDENT\_PROTEIN\_DEACETYLASE\_ACTIVITY |  | 14 | -0.42 | -1.14 | 0.296 | 0.815 | 1.000 | 2783 | tags=50%, list=23%, signal=65% |
| 1379 | GOCC\_KICSTOR\_COMPLEX |  | 4 | -0.62 | -1.14 | 0.340 | 0.816 | 1.000 | 4532 | tags=100%, list=38%, signal=161% |
| 1380 | GOBP\_NEURAL\_FOLD\_FORMATION |  | 5 | -0.58 | -1.14 | 0.331 | 0.817 | 1.000 | 1418 | tags=60%, list=12%, signal=68% |
| 1381 | GOBP\_SYNAPTIC\_TRANSMISSION\_DOPAMINERGIC |  | 13 | -0.44 | -1.14 | 0.305 | 0.817 | 1.000 | 1644 | tags=46%, list=14%, signal=53% |
| 1382 | GOBP\_REGULATION\_OF\_DENDRITIC\_CELL\_DIFFERENTIATION |  | 3 | -0.67 | -1.14 | 0.345 | 0.817 | 1.000 | 3844 | tags=67%, list=32%, signal=98% |
| 1383 | GOCC\_INTRINSIC\_COMPONENT\_OF\_MITOCHONDRIAL\_INNER\_MEMBRANE |  | 43 | -0.32 | -1.14 | 0.255 | 0.816 | 1.000 | 3110 | tags=37%, list=26%, signal=50% |
| 1384 | GOBP\_REGULATION\_OF\_RESTING\_MEMBRANE\_POTENTIAL |  | 5 | -0.58 | -1.14 | 0.324 | 0.817 | 1.000 | 3937 | tags=80%, list=33%, signal=119% |
| 1385 | GOBP\_MYELOID\_CELL\_DEVELOPMENT |  | 46 | -0.31 | -1.14 | 0.253 | 0.817 | 1.000 | 3161 | tags=39%, list=26%, signal=53% |
| 1386 | GOBP\_B\_1\_B\_CELL\_DIFFERENTIATION |  | 3 | -0.67 | -1.14 | 0.344 | 0.817 | 1.000 | 3912 | tags=100%, list=33%, signal=148% |
| 1387 | GOBP\_HISTONE\_H2A\_ACETYLATION |  | 13 | -0.43 | -1.14 | 0.304 | 0.818 | 1.000 | 5494 | tags=62%, list=46%, signal=113% |
| 1388 | GOBP\_POSITIVE\_REGULATION\_OF\_B\_CELL\_ACTIVATION |  | 52 | -0.31 | -1.14 | 0.231 | 0.818 | 1.000 | 3223 | tags=50%, list=27%, signal=68% |
| 1389 | GOMF\_DNA\_APURINIC\_OR\_APYRIMIDINIC\_SITE\_ENDONUCLEASE\_ACTIVITY |  | 8 | -0.50 | -1.13 | 0.317 | 0.818 | 1.000 | 1616 | tags=38%, list=13%, signal=43% |
| 1390 | GOMF\_ABC\_TYPE\_GLUTATHIONE\_S\_CONJUGATE\_TRANSPORTER\_ACTIVITY |  | 5 | -0.58 | -1.13 | 0.337 | 0.818 | 1.000 | 2256 | tags=80%, list=19%, signal=98% |
| 1391 | GOBP\_CELLULAR\_RESPONSE\_TO\_ESTROGEN\_STIMULUS |  | 11 | -0.45 | -1.13 | 0.312 | 0.818 | 1.000 | 755 | tags=27%, list=6%, signal=29% |
| 1392 | GOBP\_TRANSMEMBRANE\_TRANSPORT |  | 959 | -0.21 | -1.13 | 0.028 | 0.819 | 1.000 | 1501 | tags=17%, list=12%, signal=18% |
| 1393 | GOBP\_SERINE\_TRANSPORT |  | 7 | -0.52 | -1.13 | 0.320 | 0.820 | 1.000 | 1140 | tags=43%, list=9%, signal=47% |
| 1394 | GOCC\_EXOCYTIC\_VESICLE |  | 143 | -0.26 | -1.13 | 0.179 | 0.819 | 1.000 | 2361 | tags=27%, list=20%, signal=33% |
| 1395 | GOBP\_MAGNESIUM\_ION\_HOMEOSTASIS |  | 6 | -0.54 | -1.13 | 0.322 | 0.819 | 1.000 | 2981 | tags=33%, list=25%, signal=44% |
| 1396 | GOBP\_COP9\_SIGNALOSOME\_ASSEMBLY |  | 4 | -0.61 | -1.13 | 0.344 | 0.820 | 1.000 | 4629 | tags=100%, list=39%, signal=163% |
| 1397 | GOCC\_MITOCHONDRIAL\_ENVELOPE |  | 559 | -0.22 | -1.13 | 0.069 | 0.819 | 1.000 | 3154 | tags=30%, list=26%, signal=39% |
| 1398 | GOBP\_CELLULAR\_RESPONSE\_TO\_LEPTIN\_STIMULUS |  | 15 | -0.42 | -1.13 | 0.297 | 0.819 | 1.000 | 2586 | tags=40%, list=22%, signal=51% |
| 1399 | GOBP\_STRESS\_RESPONSE\_TO\_METAL\_ION |  | 5 | -0.57 | -1.13 | 0.344 | 0.819 | 1.000 | 109 | tags=40%, list=1%, signal=40% |
| 1400 | GOBP\_SNO\_S\_RNA\_METABOLIC\_PROCESS |  | 10 | -0.46 | -1.13 | 0.324 | 0.820 | 1.000 | 3711 | tags=50%, list=31%, signal=72% |
| 1401 | GOBP\_PROTEIN\_ACETYLATION |  | 152 | -0.25 | -1.13 | 0.168 | 0.820 | 1.000 | 2541 | tags=24%, list=21%, signal=30% |
| 1402 | GOBP\_PHOSPHOLIPASE\_C\_ACTIVATING\_G\_PROTEIN\_COUPLED\_RECEPTOR\_SIGNALING\_PATHWAY |  | 58 | -0.30 | -1.13 | 0.242 | 0.819 | 1.000 | 1701 | tags=29%, list=14%, signal=34% |
| 1403 | GOMF\_NERVE\_GROWTH\_FACTOR\_BINDING |  | 4 | -0.61 | -1.13 | 0.341 | 0.820 | 1.000 | 156 | tags=25%, list=1%, signal=25% |
| 1404 | GOBP\_POSITIVE\_REGULATION\_OF\_TAU\_PROTEIN\_KINASE\_ACTIVITY |  | 4 | -0.61 | -1.13 | 0.347 | 0.819 | 1.000 | 407 | tags=25%, list=3%, signal=26% |
| 1405 | GOBP\_RESPONSE\_TO\_IRON\_II\_ION |  | 4 | -0.62 | -1.13 | 0.341 | 0.819 | 1.000 | 1808 | tags=50%, list=15%, signal=59% |
| 1406 | GOBP\_POSITIVE\_REGULATION\_OF\_CARDIAC\_MUSCLE\_CELL\_DIFFERENTIATION |  | 7 | -0.51 | -1.13 | 0.317 | 0.819 | 1.000 | 3543 | tags=71%, list=29%, signal=101% |
| 1407 | GOBP\_BONE\_CELL\_DEVELOPMENT |  | 21 | -0.38 | -1.13 | 0.286 | 0.820 | 1.000 | 3161 | tags=48%, list=26%, signal=65% |
| 1408 | GOBP\_POSITIVE\_REGULATION\_OF\_DENDRITIC\_CELL\_CYTOKINE\_PRODUCTION |  | 4 | -0.61 | -1.13 | 0.343 | 0.820 | 1.000 | 1089 | tags=25%, list=9%, signal=27% |
| 1409 | GOBP\_HUMORAL\_IMMUNE\_RESPONSE |  | 92 | -0.27 | -1.13 | 0.199 | 0.820 | 1.000 | 3223 | tags=47%, list=27%, signal=63% |
| 1410 | GOMF\_ACTIVIN\_RECEPTOR\_ACTIVITY\_TYPE\_I |  | 4 | -0.61 | -1.13 | 0.345 | 0.820 | 1.000 | 4513 | tags=75%, list=38%, signal=120% |
| 1411 | GOBP\_SPINAL\_CORD\_MOTOR\_NEURON\_CELL\_FATE\_SPECIFICATION |  | 6 | -0.54 | -1.13 | 0.332 | 0.819 | 1.000 | 9 | tags=17%, list=0%, signal=17% |
| 1412 | GOBP\_NEGATIVE\_REGULATION\_OF\_LOW\_DENSITY\_LIPOPROTEIN\_PARTICLE\_CLEARANCE |  | 3 | -0.67 | -1.13 | 0.354 | 0.820 | 1.000 | 935 | tags=33%, list=8%, signal=36% |
| 1413 | GOBP\_LUNG\_ASSOCIATED\_MESENCHYME\_DEVELOPMENT |  | 4 | -0.61 | -1.13 | 0.341 | 0.819 | 1.000 | 1656 | tags=50%, list=14%, signal=58% |
| 1414 | GOBP\_NEGATIVE\_REGULATION\_OF\_ENDOTHELIAL\_CELL\_APOPTOTIC\_PROCESS |  | 21 | -0.38 | -1.13 | 0.293 | 0.819 | 1.000 | 1130 | tags=19%, list=9%, signal=21% |
| 1415 | GOBP\_2FE\_2S\_CLUSTER\_ASSEMBLY |  | 4 | -0.61 | -1.13 | 0.362 | 0.818 | 1.000 | 4664 | tags=100%, list=39%, signal=163% |
| 1416 | GOMF\_G\_PROTEIN\_COUPLED\_AMINE\_RECEPTOR\_ACTIVITY |  | 18 | -0.39 | -1.13 | 0.291 | 0.818 | 1.000 | 1701 | tags=28%, list=14%, signal=32% |
| 1417 | GOBP\_ANTERIOR\_POSTERIOR\_AXIS\_SPECIFICATION |  | 26 | -0.36 | -1.13 | 0.275 | 0.818 | 1.000 | 2230 | tags=38%, list=19%, signal=47% |
| 1418 | GOBP\_REGULATION\_OF\_UBIQUITIN\_DEPENDENT\_PROTEIN\_CATABOLIC\_PROCESS |  | 120 | -0.26 | -1.13 | 0.203 | 0.818 | 1.000 | 2842 | tags=28%, list=24%, signal=37% |
| 1419 | GOBP\_CILIUM\_OR\_FLAGELLUM\_DEPENDENT\_CELL\_MOTILITY |  | 53 | -0.30 | -1.13 | 0.245 | 0.820 | 1.000 | 2803 | tags=30%, list=23%, signal=39% |
| 1420 | GOBP\_INOSITOL\_PHOSPHATE\_METABOLIC\_PROCESS |  | 55 | -0.30 | -1.13 | 0.249 | 0.821 | 1.000 | 1577 | tags=25%, list=13%, signal=29% |
| 1421 | GOCC\_PARALLEL\_FIBER\_TO\_PURKINJE\_CELL\_SYNAPSE |  | 7 | -0.52 | -1.13 | 0.328 | 0.820 | 1.000 | 1092 | tags=29%, list=9%, signal=31% |
| 1422 | GOBP\_MUSCLE\_CELL\_CELLULAR\_HOMEOSTASIS |  | 14 | -0.42 | -1.13 | 0.300 | 0.820 | 1.000 | 1894 | tags=36%, list=16%, signal=42% |
| 1423 | GOMF\_SODIUM\_ION\_BINDING |  | 6 | -0.54 | -1.13 | 0.338 | 0.820 | 1.000 | 3609 | tags=67%, list=30%, signal=95% |
| 1424 | GOBP\_DIADENOSINE\_POLYPHOSPHATE\_METABOLIC\_PROCESS |  | 3 | -0.66 | -1.13 | 0.351 | 0.819 | 1.000 | 2359 | tags=67%, list=20%, signal=83% |
| 1425 | GOBP\_HORMONE\_BIOSYNTHETIC\_PROCESS |  | 40 | -0.32 | -1.13 | 0.258 | 0.819 | 1.000 | 1418 | tags=25%, list=12%, signal=28% |
| 1426 | GOBP\_DRUG\_CATABOLIC\_PROCESS |  | 9 | -0.47 | -1.13 | 0.324 | 0.819 | 1.000 | 112 | tags=22%, list=1%, signal=22% |
| 1427 | GOBP\_SULFUR\_COMPOUND\_CATABOLIC\_PROCESS |  | 37 | -0.33 | -1.13 | 0.257 | 0.820 | 1.000 | 1744 | tags=22%, list=15%, signal=25% |
| 1428 | GOBP\_ATP\_BIOSYNTHETIC\_PROCESS |  | 22 | -0.37 | -1.13 | 0.285 | 0.820 | 1.000 | 2974 | tags=41%, list=25%, signal=54% |
| 1429 | GOCC\_SWR1\_COMPLEX |  | 9 | -0.48 | -1.13 | 0.312 | 0.819 | 1.000 | 5318 | tags=67%, list=44%, signal=120% |
| 1430 | GOBP\_NEGATIVE\_REGULATION\_OF\_CLATHRIN\_DEPENDENT\_ENDOCYTOSIS |  | 4 | -0.61 | -1.13 | 0.352 | 0.819 | 1.000 | 2055 | tags=50%, list=17%, signal=60% |
| 1431 | GOBP\_MICROTUBULE\_BUNDLE\_FORMATION |  | 52 | -0.30 | -1.13 | 0.253 | 0.819 | 1.000 | 2435 | tags=29%, list=20%, signal=36% |
| 1432 | GOBP\_MYELOID\_DENDRITIC\_CELL\_CYTOKINE\_PRODUCTION |  | 4 | -0.61 | -1.13 | 0.354 | 0.819 | 1.000 | 1089 | tags=25%, list=9%, signal=27% |
| 1433 | GOBP\_POSITIVE\_REGULATION\_OF\_B\_CELL\_MEDIATED\_IMMUNITY |  | 22 | -0.37 | -1.13 | 0.296 | 0.819 | 1.000 | 3209 | tags=55%, list=27%, signal=74% |
| 1434 | GOBP\_PROTEIN\_IMPORT\_INTO\_PEROXISOME\_MATRIX |  | 12 | -0.44 | -1.13 | 0.312 | 0.818 | 1.000 | 1896 | tags=25%, list=16%, signal=30% |
| 1435 | GOCC\_GPI\_ANCHOR\_TRANSAMIDASE\_COMPLEX |  | 4 | -0.61 | -1.13 | 0.357 | 0.819 | 1.000 | 3465 | tags=75%, list=29%, signal=105% |
| 1436 | GOMF\_BOX\_H\_ACA\_SNORNA\_BINDING |  | 4 | -0.61 | -1.13 | 0.357 | 0.819 | 1.000 | 621 | tags=25%, list=5%, signal=26% |
| 1437 | GOBP\_SMOOTHENED\_SIGNALING\_PATHWAY\_INVOLVED\_IN\_REGULATION\_OF\_CEREBELLAR\_GRANULE\_CELL\_PRECURSOR\_CELL\_PROLIFERATION |  | 5 | -0.58 | -1.13 | 0.351 | 0.819 | 1.000 | 4988 | tags=80%, list=42%, signal=137% |
| 1438 | GOBP\_POSITIVE\_REGULATION\_OF\_VIRAL\_TRANSCRIPTION |  | 22 | -0.37 | -1.13 | 0.292 | 0.819 | 1.000 | 3741 | tags=41%, list=31%, signal=59% |
| 1439 | GOBP\_URETER\_MORPHOGENESIS |  | 5 | -0.57 | -1.13 | 0.367 | 0.819 | 1.000 | 141 | tags=20%, list=1%, signal=20% |
| 1440 | GOCC\_PARANODAL\_JUNCTION |  | 7 | -0.52 | -1.13 | 0.337 | 0.818 | 1.000 | 1286 | tags=29%, list=11%, signal=32% |
| 1441 | GOBP\_SENSORY\_PERCEPTION\_OF\_TASTE |  | 17 | -0.40 | -1.13 | 0.302 | 0.819 | 1.000 | 2170 | tags=41%, list=18%, signal=50% |
| 1442 | GOBP\_RESPONSE\_TO\_LEAD\_ION |  | 16 | -0.40 | -1.12 | 0.293 | 0.819 | 1.000 | 4517 | tags=63%, list=38%, signal=100% |
| 1443 | GOBP\_REGULATION\_OF\_B\_CELL\_ACTIVATION |  | 81 | -0.28 | -1.12 | 0.222 | 0.819 | 1.000 | 3223 | tags=43%, list=27%, signal=59% |
| 1444 | GOBP\_NEPHRON\_TUBULE\_FORMATION |  | 5 | -0.57 | -1.12 | 0.347 | 0.821 | 1.000 | 2042 | tags=40%, list=17%, signal=48% |
| 1445 | GOBP\_NEGATIVE\_REGULATION\_OF\_HEMATOPOIETIC\_PROGENITOR\_CELL\_DIFFERENTIATION |  | 6 | -0.54 | -1.12 | 0.338 | 0.821 | 1.000 | 3624 | tags=50%, list=30%, signal=72% |
| 1446 | GOBP\_PROTEASOME\_ASSEMBLY |  | 11 | -0.45 | -1.12 | 0.323 | 0.821 | 1.000 | 4541 | tags=64%, list=38%, signal=102% |
| 1447 | GOBP\_NEGATIVE\_REGULATION\_OF\_TRIGLYCERIDE\_METABOLIC\_PROCESS |  | 5 | -0.57 | -1.12 | 0.349 | 0.821 | 1.000 | 3527 | tags=80%, list=29%, signal=113% |
| 1448 | GOMF\_ANION\_CHANNEL\_ACTIVITY |  | 37 | -0.32 | -1.12 | 0.274 | 0.821 | 1.000 | 591 | tags=16%, list=5%, signal=17% |
| 1449 | GOBP\_APPENDAGE\_DEVELOPMENT |  | 106 | -0.27 | -1.12 | 0.208 | 0.821 | 1.000 | 2355 | tags=28%, list=20%, signal=35% |
| 1450 | GOBP\_ACTIVATION\_OF\_MAPK\_ACTIVITY |  | 107 | -0.26 | -1.12 | 0.218 | 0.821 | 1.000 | 3022 | tags=30%, list=25%, signal=40% |
| 1451 | GOMF\_ORGANOPHOSPHATE\_ESTER\_TRANSMEMBRANE\_TRANSPORTER\_ACTIVITY |  | 22 | -0.37 | -1.12 | 0.286 | 0.820 | 1.000 | 2911 | tags=45%, list=24%, signal=60% |
| 1452 | GOMF\_NUCLEAR\_LOCALIZATION\_SEQUENCE\_BINDING |  | 19 | -0.38 | -1.12 | 0.300 | 0.820 | 1.000 | 3122 | tags=37%, list=26%, signal=50% |
| 1453 | GOBP\_POSITIVE\_REGULATION\_OF\_COLLATERAL\_SPROUTING |  | 5 | -0.57 | -1.12 | 0.344 | 0.820 | 1.000 | 71 | tags=20%, list=1%, signal=20% |
| 1454 | GOBP\_PROTEIN\_LOCALIZATION\_TO\_MEMBRANE |  | 477 | -0.22 | -1.12 | 0.094 | 0.820 | 1.000 | 1488 | tags=18%, list=12%, signal=20% |
| 1455 | GOBP\_POSITIVE\_REGULATION\_OF\_CELL\_CYCLE\_G1\_S\_PHASE\_TRANSITION |  | 36 | -0.33 | -1.12 | 0.278 | 0.820 | 1.000 | 2417 | tags=28%, list=20%, signal=35% |
| 1456 | GOBP\_MITOCHONDRIAL\_TRANSPORT |  | 185 | -0.24 | -1.12 | 0.175 | 0.820 | 1.000 | 3005 | tags=35%, list=25%, signal=45% |
| 1457 | GOBP\_REGULATION\_OF\_CHAPERONE\_MEDIATED\_AUTOPHAGY |  | 6 | -0.54 | -1.12 | 0.342 | 0.820 | 1.000 | 1713 | tags=50%, list=14%, signal=58% |
| 1458 | GOBP\_MORPHOGENESIS\_OF\_A\_POLARIZED\_EPITHELIUM |  | 104 | -0.27 | -1.12 | 0.211 | 0.820 | 1.000 | 2288 | tags=18%, list=19%, signal=22% |
| 1459 | GOBP\_REGULATION\_OF\_NEUROBLAST\_PROLIFERATION |  | 20 | -0.38 | -1.12 | 0.294 | 0.820 | 1.000 | 2789 | tags=40%, list=23%, signal=52% |
| 1460 | GOMF\_CATECHOLAMINE\_BINDING |  | 8 | -0.49 | -1.12 | 0.326 | 0.819 | 1.000 | 1604 | tags=38%, list=13%, signal=43% |
| 1461 | GOBP\_NEGATIVE\_REGULATION\_OF\_LEUKOCYTE\_MEDIATED\_CYTOTOXICITY |  | 5 | -0.57 | -1.12 | 0.350 | 0.820 | 1.000 | 5090 | tags=60%, list=42%, signal=104% |
| 1462 | GOBP\_NEGATIVE\_REGULATION\_OF\_CELL\_JUNCTION\_ASSEMBLY |  | 26 | -0.35 | -1.12 | 0.293 | 0.819 | 1.000 | 2052 | tags=27%, list=17%, signal=32% |
| 1463 | GOBP\_UBIQUITIN\_RECYCLING |  | 5 | -0.57 | -1.12 | 0.345 | 0.819 | 1.000 | 3216 | tags=60%, list=27%, signal=82% |
| 1464 | GOBP\_DIADENOSINE\_POLYPHOSPHATE\_CATABOLIC\_PROCESS |  | 3 | -0.66 | -1.12 | 0.362 | 0.818 | 1.000 | 2359 | tags=67%, list=20%, signal=83% |
| 1465 | GOBP\_NEGATIVE\_REGULATION\_OF\_DEFENSE\_RESPONSE\_TO\_BACTERIUM |  | 3 | -0.66 | -1.12 | 0.365 | 0.819 | 1.000 | 280 | tags=33%, list=2%, signal=34% |
| 1466 | GOMF\_HISTONE\_DEMETHYLASE\_ACTIVITY\_H3\_K4\_SPECIFIC |  | 4 | -0.61 | -1.12 | 0.365 | 0.819 | 1.000 | 4652 | tags=100%, list=39%, signal=163% |
| 1467 | GOBP\_CELLULAR\_RESPONSE\_TO\_ALDEHYDE |  | 10 | -0.46 | -1.12 | 0.323 | 0.819 | 1.000 | 244 | tags=20%, list=2%, signal=20% |
| 1468 | GOBP\_GASTRO\_INTESTINAL\_SYSTEM\_SMOOTH\_MUSCLE\_CONTRACTION |  | 9 | -0.47 | -1.12 | 0.331 | 0.819 | 1.000 | 536 | tags=22%, list=4%, signal=23% |
| 1469 | GOBP\_CELLULAR\_RESPONSE\_TO\_PEPTIDOGLYCAN |  | 3 | -0.67 | -1.12 | 0.366 | 0.819 | 1.000 | 3937 | tags=67%, list=33%, signal=99% |
| 1470 | GOBP\_GLIAL\_CELL\_FATE\_COMMITMENT |  | 5 | -0.57 | -1.12 | 0.346 | 0.819 | 1.000 | 514 | tags=40%, list=4%, signal=42% |
| 1471 | GOBP\_REGULATION\_OF\_OXIDATIVE\_PHOSPHORYLATION |  | 10 | -0.46 | -1.12 | 0.327 | 0.819 | 1.000 | 3284 | tags=70%, list=27%, signal=96% |
| 1472 | GOCC\_MOTILE\_CILIUM |  | 80 | -0.28 | -1.12 | 0.239 | 0.819 | 1.000 | 2803 | tags=30%, list=23%, signal=39% |
| 1473 | GOBP\_DISACCHARIDE\_BIOSYNTHETIC\_PROCESS |  | 3 | -0.66 | -1.12 | 0.366 | 0.818 | 1.000 | 3656 | tags=67%, list=30%, signal=96% |
| 1474 | GOBP\_REGULATION\_OF\_TYPE\_B\_PANCREATIC\_CELL\_PROLIFERATION |  | 8 | -0.49 | -1.12 | 0.335 | 0.818 | 1.000 | 1932 | tags=38%, list=16%, signal=45% |
| 1475 | GOBP\_PROGESTERONE\_BIOSYNTHETIC\_PROCESS |  | 4 | -0.61 | -1.12 | 0.374 | 0.818 | 1.000 | 878 | tags=50%, list=7%, signal=54% |
| 1476 | GOMF\_PEPTIDOGLYCAN\_MURALYTIC\_ACTIVITY |  | 3 | -0.66 | -1.12 | 0.364 | 0.818 | 1.000 | 3222 | tags=67%, list=27%, signal=91% |
| 1477 | GOBP\_ESTABLISHMENT\_OF\_PROTEIN\_LOCALIZATION\_TO\_POSTSYNAPTIC\_MEMBRANE |  | 10 | -0.46 | -1.12 | 0.327 | 0.818 | 1.000 | 617 | tags=20%, list=5%, signal=21% |
| 1478 | GOMF\_CYSTEINE\_TYPE\_EXOPEPTIDASE\_ACTIVITY |  | 3 | -0.66 | -1.12 | 0.368 | 0.818 | 1.000 | 3253 | tags=67%, list=27%, signal=91% |
| 1479 | GOCC\_CATION\_CHANNEL\_COMPLEX |  | 110 | -0.26 | -1.12 | 0.227 | 0.817 | 1.000 | 1385 | tags=19%, list=12%, signal=21% |
| 1480 | GOBP\_PHOSPHATIDYLINOSITOL\_BIOSYNTHETIC\_PROCESS |  | 100 | -0.27 | -1.12 | 0.225 | 0.817 | 1.000 | 2867 | tags=34%, list=24%, signal=44% |
| 1481 | GOBP\_PYRIMIDINE\_DIMER\_REPAIR |  | 7 | -0.51 | -1.12 | 0.336 | 0.817 | 1.000 | 2167 | tags=29%, list=18%, signal=35% |
| 1482 | GOBP\_POSTSYNAPTIC\_MEMBRANE\_ORGANIZATION |  | 21 | -0.37 | -1.12 | 0.294 | 0.819 | 1.000 | 3477 | tags=48%, list=29%, signal=67% |
| 1483 | GOMF\_EXOGENOUS\_PROTEIN\_BINDING |  | 47 | -0.31 | -1.12 | 0.269 | 0.819 | 1.000 | 2159 | tags=34%, list=18%, signal=41% |
| 1484 | GOCC\_BOX\_H\_ACA\_SNORNP\_COMPLEX |  | 4 | -0.61 | -1.12 | 0.360 | 0.818 | 1.000 | 621 | tags=25%, list=5%, signal=26% |
| 1485 | GOBP\_RESPONSE\_TO\_UV\_A |  | 8 | -0.49 | -1.12 | 0.333 | 0.819 | 1.000 | 295 | tags=13%, list=2%, signal=13% |
| 1486 | GOBP\_LEPTIN\_MEDIATED\_SIGNALING\_PATHWAY |  | 9 | -0.47 | -1.12 | 0.340 | 0.818 | 1.000 | 2586 | tags=56%, list=22%, signal=71% |
| 1487 | GOBP\_RIBOSE\_PHOSPHATE\_BIOSYNTHETIC\_PROCESS |  | 125 | -0.26 | -1.12 | 0.207 | 0.818 | 1.000 | 2669 | tags=31%, list=22%, signal=40% |
| 1488 | GOCC\_BOX\_H\_ACA\_RNP\_COMPLEX |  | 4 | -0.61 | -1.12 | 0.362 | 0.818 | 1.000 | 621 | tags=25%, list=5%, signal=26% |
| 1489 | GOBP\_REGULATION\_OF\_STEM\_CELL\_DIVISION |  | 6 | -0.54 | -1.12 | 0.348 | 0.817 | 1.000 | 629 | tags=33%, list=5%, signal=35% |
| 1490 | GOCC\_PRE\_SNORNP\_COMPLEX |  | 6 | -0.53 | -1.12 | 0.357 | 0.818 | 1.000 | 2464 | tags=50%, list=21%, signal=63% |
| 1491 | GOBP\_RNA\_SECONDARY\_STRUCTURE\_UNWINDING |  | 5 | -0.56 | -1.12 | 0.354 | 0.818 | 1.000 | 5255 | tags=100%, list=44%, signal=178% |
| 1492 | GOBP\_NEGATIVE\_REGULATION\_OF\_GRANULOCYTE\_DIFFERENTIATION |  | 7 | -0.51 | -1.12 | 0.345 | 0.820 | 1.000 | 2583 | tags=57%, list=22%, signal=73% |
| 1493 | GOBP\_ATP\_TRANSPORT |  | 12 | -0.44 | -1.12 | 0.319 | 0.820 | 1.000 | 2911 | tags=50%, list=24%, signal=66% |
| 1494 | GOBP\_POSITIVE\_REGULATION\_OF\_I\_KAPPAB\_PHOSPHORYLATION |  | 5 | -0.56 | -1.12 | 0.357 | 0.820 | 1.000 | 351 | tags=20%, list=3%, signal=21% |
| 1495 | GOBP\_NEGATIVE\_REGULATION\_OF\_SMOOTH\_MUSCLE\_CELL\_APOPTOTIC\_PROCESS |  | 5 | -0.56 | -1.11 | 0.362 | 0.820 | 1.000 | 3506 | tags=60%, list=29%, signal=85% |
| 1496 | GOBP\_NUCLEOTIDE\_EXCISION\_REPAIR\_DNA\_DAMAGE\_RECOGNITION |  | 21 | -0.37 | -1.11 | 0.312 | 0.820 | 1.000 | 4882 | tags=57%, list=41%, signal=96% |
| 1497 | GOBP\_POSITIVE\_REGULATION\_OF\_CORTICOSTEROID\_HORMONE\_SECRETION |  | 4 | -0.61 | -1.11 | 0.375 | 0.820 | 1.000 | 12 | tags=25%, list=0%, signal=25% |
| 1498 | GOBP\_REGULATION\_OF\_HEXOKINASE\_ACTIVITY |  | 6 | -0.53 | -1.11 | 0.359 | 0.819 | 1.000 | 5461 | tags=83%, list=45%, signal=153% |
| 1499 | GOBP\_REGULATION\_OF\_DEFENSE\_RESPONSE\_TO\_BACTERIUM |  | 8 | -0.49 | -1.11 | 0.338 | 0.819 | 1.000 | 4125 | tags=63%, list=34%, signal=95% |
| 1500 | GOBP\_CENTRAL\_NERVOUS\_SYSTEM\_MATURATION |  | 4 | -0.60 | -1.11 | 0.369 | 0.818 | 1.000 | 4770 | tags=100%, list=40%, signal=166% |
| 1501 | GOBP\_SPERM\_AXONEME\_ASSEMBLY |  | 15 | -0.41 | -1.11 | 0.322 | 0.819 | 1.000 | 2803 | tags=47%, list=23%, signal=61% |
| 1502 | GOCC\_COMPACT\_MYELIN |  | 9 | -0.47 | -1.11 | 0.343 | 0.820 | 1.000 | 44 | tags=11%, list=0%, signal=11% |
| 1503 | GOBP\_VITAMIN\_TRANSMEMBRANE\_TRANSPORT |  | 12 | -0.44 | -1.11 | 0.324 | 0.820 | 1.000 | 1001 | tags=25%, list=8%, signal=27% |
| 1504 | GOBP\_IRON\_SULFUR\_CLUSTER\_ASSEMBLY |  | 18 | -0.39 | -1.11 | 0.306 | 0.819 | 1.000 | 4664 | tags=61%, list=39%, signal=100% |
| 1505 | GOBP\_AMELOGENESIS |  | 13 | -0.42 | -1.11 | 0.326 | 0.820 | 1.000 | 2508 | tags=46%, list=21%, signal=58% |
| 1506 | GOBP\_HYDROGEN\_PEROXIDE\_METABOLIC\_PROCESS |  | 28 | -0.35 | -1.11 | 0.288 | 0.820 | 1.000 | 1300 | tags=21%, list=11%, signal=24% |
| 1507 | GOBP\_POSITIVE\_REGULATION\_OF\_SYNAPTIC\_TRANSMISSION |  | 83 | -0.27 | -1.11 | 0.248 | 0.820 | 1.000 | 2563 | tags=30%, list=21%, signal=38% |
| 1508 | GOBP\_VERY\_LONG\_CHAIN\_FATTY\_ACID\_CATABOLIC\_PROCESS |  | 5 | -0.56 | -1.11 | 0.365 | 0.820 | 1.000 | 3695 | tags=60%, list=31%, signal=87% |
| 1509 | GOBP\_PROTEIN\_IMPORT |  | 145 | -0.25 | -1.11 | 0.212 | 0.820 | 1.000 | 3484 | tags=33%, list=29%, signal=46% |
| 1510 | GOBP\_CONVERGENT\_EXTENSION\_INVOLVED\_IN\_ORGANOGENESIS |  | 4 | -0.60 | -1.11 | 0.368 | 0.821 | 1.000 | 677 | tags=25%, list=6%, signal=26% |
| 1511 | GOBP\_BIOLOGICAL\_PROCESS\_INVOLVED\_IN\_SYMBIOTIC\_INTERACTION |  | 713 | -0.21 | -1.11 | 0.084 | 0.820 | 1.000 | 2662 | tags=27%, list=22%, signal=33% |
| 1512 | GOCC\_MITOCHONDRIAL\_PYRUVATE\_DEHYDROGENASE\_COMPLEX |  | 4 | -0.60 | -1.11 | 0.368 | 0.822 | 1.000 | 4007 | tags=75%, list=33%, signal=113% |
| 1513 | GOBP\_POSITIVE\_REGULATION\_OF\_TRANSCRIPTION\_BY\_RNA\_POLYMERASE\_I |  | 18 | -0.39 | -1.11 | 0.309 | 0.823 | 1.000 | 2541 | tags=50%, list=21%, signal=63% |
| 1514 | GOBP\_REGULATION\_OF\_INCLUSION\_BODY\_ASSEMBLY |  | 10 | -0.45 | -1.11 | 0.339 | 0.822 | 1.000 | 3317 | tags=40%, list=28%, signal=55% |
| 1515 | GOBP\_POSITIVE\_REGULATION\_OF\_STEROID\_HORMONE\_SECRETION |  | 4 | -0.61 | -1.11 | 0.366 | 0.822 | 1.000 | 12 | tags=25%, list=0%, signal=25% |
| 1516 | GOCC\_INTRINSIC\_COMPONENT\_OF\_POSTSYNAPTIC\_MEMBRANE |  | 47 | -0.31 | -1.11 | 0.262 | 0.822 | 1.000 | 2575 | tags=36%, list=21%, signal=46% |
| 1517 | GOBP\_DEVELOPMENTAL\_GROWTH\_INVOLVED\_IN\_MORPHOGENESIS |  | 162 | -0.25 | -1.11 | 0.206 | 0.821 | 1.000 | 1133 | tags=14%, list=9%, signal=15% |
| 1518 | GOBP\_NEGATIVE\_REGULATION\_OF\_OXIDATIVE\_STRESS\_INDUCED\_NEURON\_INTRINSIC\_APOPTOTIC\_SIGNALING\_PATHWAY |  | 3 | -0.66 | -1.11 | 0.364 | 0.821 | 1.000 | 4103 | tags=100%, list=34%, signal=152% |
| 1519 | GOBP\_POSITIVE\_REGULATION\_OF\_INTRACELLULAR\_STEROID\_HORMONE\_RECEPTOR\_SIGNALING\_PATHWAY |  | 9 | -0.47 | -1.11 | 0.339 | 0.821 | 1.000 | 3561 | tags=78%, list=30%, signal=110% |
| 1520 | GOBP\_MAINTENANCE\_OF\_PROTEIN\_LOCATION\_IN\_NUCLEUS |  | 17 | -0.40 | -1.11 | 0.321 | 0.821 | 1.000 | 3415 | tags=53%, list=28%, signal=74% |
| 1521 | GOBP\_NEGATIVE\_REGULATION\_OF\_CELL\_GROWTH |  | 128 | -0.25 | -1.11 | 0.213 | 0.822 | 1.000 | 2042 | tags=23%, list=17%, signal=27% |
| 1522 | GOBP\_NERVE\_DEVELOPMENT |  | 41 | -0.31 | -1.11 | 0.287 | 0.821 | 1.000 | 1245 | tags=22%, list=10%, signal=24% |
| 1523 | GOCC\_9PLUS2\_MOTILE\_CILIUM |  | 44 | -0.31 | -1.11 | 0.287 | 0.821 | 1.000 | 664 | tags=11%, list=6%, signal=12% |
| 1524 | GOBP\_REGULATION\_OF\_RENAL\_SYSTEM\_PROCESS |  | 20 | -0.38 | -1.11 | 0.322 | 0.820 | 1.000 | 1630 | tags=20%, list=14%, signal=23% |
| 1525 | GOBP\_NEGATIVE\_REGULATION\_OF\_T\_CELL\_MEDIATED\_CYTOTOXICITY |  | 3 | -0.66 | -1.11 | 0.360 | 0.820 | 1.000 | 3969 | tags=67%, list=33%, signal=100% |
| 1526 | GOMF\_ENDORIBONUCLEASE\_ACTIVITY\_PRODUCING\_3\_PHOSPHOMONOESTERS |  | 3 | -0.66 | -1.11 | 0.376 | 0.821 | 1.000 | 3067 | tags=67%, list=26%, signal=90% |
| 1527 | GOBP\_PHAGOSOME\_MATURATION |  | 37 | -0.32 | -1.11 | 0.291 | 0.820 | 1.000 | 2055 | tags=22%, list=17%, signal=26% |
| 1528 | GOBP\_SA\_NODE\_CELL\_TO\_ATRIAL\_CARDIAC\_MUSCLE\_CELL\_SIGNALING |  | 6 | -0.53 | -1.11 | 0.357 | 0.821 | 1.000 | 1316 | tags=50%, list=11%, signal=56% |
| 1529 | GOBP\_GENETIC\_IMPRINTING |  | 14 | -0.41 | -1.11 | 0.320 | 0.822 | 1.000 | 13 | tags=7%, list=0%, signal=7% |
| 1530 | GOBP\_ASPARTATE\_FAMILY\_AMINO\_ACID\_CATABOLIC\_PROCESS |  | 22 | -0.37 | -1.11 | 0.305 | 0.822 | 1.000 | 521 | tags=18%, list=4%, signal=19% |
| 1531 | GOMF\_G\_PROTEIN\_BETA\_SUBUNIT\_BINDING |  | 13 | -0.42 | -1.11 | 0.332 | 0.822 | 1.000 | 4157 | tags=62%, list=35%, signal=94% |
| 1532 | GOCC\_MYELIN\_SHEATH |  | 30 | -0.34 | -1.11 | 0.289 | 0.823 | 1.000 | 1595 | tags=20%, list=13%, signal=23% |
| 1533 | GOBP\_TRNA\_METHYLATION |  | 31 | -0.34 | -1.11 | 0.303 | 0.823 | 1.000 | 3843 | tags=45%, list=32%, signal=66% |
| 1534 | GOMF\_PROTON\_TRANSPORTING\_ATP\_SYNTHASE\_ACTIVITY\_ROTATIONAL\_MECHANISM |  | 3 | -0.66 | -1.11 | 0.392 | 0.823 | 1.000 | 3530 | tags=67%, list=29%, signal=94% |
| 1535 | GOBP\_RECEPTOR\_LOCALIZATION\_TO\_NON\_MOTILE\_CILIUM |  | 4 | -0.60 | -1.11 | 0.368 | 0.822 | 1.000 | 2151 | tags=50%, list=18%, signal=61% |
| 1536 | GOMF\_NUCLEOBASE\_CONTAINING\_COMPOUND\_KINASE\_ACTIVITY |  | 31 | -0.33 | -1.11 | 0.303 | 0.822 | 1.000 | 1626 | tags=32%, list=14%, signal=37% |
| 1537 | GOMF\_SPHINGOSINE\_1\_PHOSPHATE\_RECEPTOR\_ACTIVITY |  | 5 | -0.56 | -1.11 | 0.373 | 0.823 | 1.000 | 1338 | tags=40%, list=11%, signal=45% |
| 1538 | GOCC\_EXTERNAL\_SIDE\_OF\_PLASMA\_MEMBRANE |  | 183 | -0.24 | -1.11 | 0.191 | 0.822 | 1.000 | 1989 | tags=24%, list=17%, signal=28% |
| 1539 | GOBP\_NEGATIVE\_REGULATION\_OF\_MYOTUBE\_DIFFERENTIATION |  | 8 | -0.48 | -1.11 | 0.346 | 0.822 | 1.000 | 1482 | tags=38%, list=12%, signal=43% |
| 1540 | GOMF\_UDP\_N\_ACETYLGLUCOSAMINE\_TRANSMEMBRANE\_TRANSPORTER\_ACTIVITY |  | 3 | -0.65 | -1.11 | 0.376 | 0.822 | 1.000 | 185 | tags=33%, list=2%, signal=34% |
| 1541 | GOBP\_NEGATIVE\_REGULATION\_OF\_STRIATED\_MUSCLE\_CONTRACTION |  | 5 | -0.56 | -1.11 | 0.364 | 0.822 | 1.000 | 5332 | tags=100%, list=44%, signal=180% |
| 1542 | GOCC\_PROTON\_TRANSPORTING\_V\_TYPE\_ATPASE\_V1\_DOMAIN |  | 6 | -0.53 | -1.11 | 0.362 | 0.821 | 1.000 | 3730 | tags=67%, list=31%, signal=97% |
| 1543 | GOMF\_RNA\_POLYMERASE\_CORE\_ENZYME\_BINDING |  | 35 | -0.32 | -1.11 | 0.297 | 0.821 | 1.000 | 1465 | tags=14%, list=12%, signal=16% |
| 1544 | GOBP\_HISTONE\_H2A\_PHOSPHORYLATION |  | 5 | -0.56 | -1.11 | 0.361 | 0.821 | 1.000 | 5298 | tags=100%, list=44%, signal=179% |
| 1545 | GOBP\_CALCIUM\_ACTIVATED\_PHOSPHOLIPID\_SCRAMBLING |  | 3 | -0.65 | -1.11 | 0.386 | 0.821 | 1.000 | 103 | tags=33%, list=1%, signal=34% |
| 1546 | GOBP\_CELLULAR\_DETOXIFICATION\_OF\_ALDEHYDE |  | 6 | -0.54 | -1.11 | 0.364 | 0.820 | 1.000 | 3725 | tags=83%, list=31%, signal=121% |
| 1547 | GOCC\_SCHAFFER\_COLLATERAL\_CA1\_SYNAPSE |  | 40 | -0.32 | -1.11 | 0.299 | 0.820 | 1.000 | 2479 | tags=25%, list=21%, signal=31% |
| 1548 | GOBP\_REGULATION\_OF\_VASCULAR\_WOUND\_HEALING |  | 6 | -0.52 | -1.11 | 0.359 | 0.820 | 1.000 | 970 | tags=17%, list=8%, signal=18% |
| 1549 | GOMF\_ALPHA\_N\_ACETYLNEURAMINATE\_ALPHA\_2\_8\_SIALYLTRANSFERASE\_ACTIVITY |  | 4 | -0.61 | -1.11 | 0.379 | 0.819 | 1.000 | 2870 | tags=50%, list=24%, signal=66% |
| 1550 | GOCC\_MSL\_COMPLEX |  | 3 | -0.66 | -1.11 | 0.375 | 0.819 | 1.000 | 3869 | tags=67%, list=32%, signal=98% |
| 1551 | GOMF\_WNT\_PROTEIN\_BINDING |  | 25 | -0.36 | -1.10 | 0.306 | 0.820 | 1.000 | 2226 | tags=24%, list=19%, signal=29% |
| 1552 | GOMF\_DEACETYLASE\_ACTIVITY |  | 29 | -0.34 | -1.10 | 0.315 | 0.820 | 1.000 | 2783 | tags=41%, list=23%, signal=54% |
| 1553 | GOBP\_NADP\_METABOLIC\_PROCESS |  | 26 | -0.35 | -1.10 | 0.321 | 0.822 | 1.000 | 1720 | tags=23%, list=14%, signal=27% |
| 1554 | GOBP\_REGULATION\_OF\_GTP\_BINDING |  | 11 | -0.44 | -1.10 | 0.346 | 0.823 | 1.000 | 1190 | tags=27%, list=10%, signal=30% |
| 1555 | GOBP\_CELL\_CELL\_SIGNALING\_INVOLVED\_IN\_CARDIAC\_CONDUCTION |  | 19 | -0.38 | -1.10 | 0.331 | 0.824 | 1.000 | 1424 | tags=32%, list=12%, signal=36% |
| 1556 | GOMF\_RNA\_POLYMERASE\_III\_TRANSCRIPTION\_REGULATORY\_REGION\_SEQUENCE\_SPECIFIC\_DNA\_BINDING |  | 6 | -0.53 | -1.10 | 0.367 | 0.824 | 1.000 | 2912 | tags=50%, list=24%, signal=66% |
| 1557 | GOBP\_UDP\_N\_ACETYLGLUCOSAMINE\_TRANSMEMBRANE\_TRANSPORT |  | 3 | -0.65 | -1.10 | 0.401 | 0.825 | 1.000 | 185 | tags=33%, list=2%, signal=34% |
| 1558 | GOBP\_CARDIAC\_MYOFIBRIL\_ASSEMBLY |  | 10 | -0.46 | -1.10 | 0.340 | 0.825 | 1.000 | 1013 | tags=30%, list=8%, signal=33% |
| 1559 | GOMF\_GLUCURONOSYLTRANSFERASE\_ACTIVITY |  | 13 | -0.42 | -1.10 | 0.341 | 0.825 | 1.000 | 1967 | tags=31%, list=16%, signal=37% |
| 1560 | GOBP\_REGULATION\_OF\_HISTONE\_H3\_K36\_METHYLATION |  | 5 | -0.56 | -1.10 | 0.373 | 0.825 | 1.000 | 3394 | tags=60%, list=28%, signal=84% |
| 1561 | GOBP\_SULFUR\_AMINO\_ACID\_BIOSYNTHETIC\_PROCESS |  | 14 | -0.41 | -1.10 | 0.328 | 0.825 | 1.000 | 821 | tags=21%, list=7%, signal=23% |
| 1562 | GOBP\_ASPARTATE\_FAMILY\_AMINO\_ACID\_METABOLIC\_PROCESS |  | 42 | -0.31 | -1.10 | 0.290 | 0.825 | 1.000 | 821 | tags=14%, list=7%, signal=15% |
| 1563 | GOBP\_DIVALENT\_INORGANIC\_CATION\_HOMEOSTASIS |  | 298 | -0.23 | -1.10 | 0.171 | 0.825 | 1.000 | 1571 | tags=20%, list=13%, signal=23% |
| 1564 | GOMF\_PROTEIN\_TYROSINE\_SERINE\_THREONINE\_PHOSPHATASE\_ACTIVITY |  | 32 | -0.33 | -1.10 | 0.312 | 0.824 | 1.000 | 2155 | tags=22%, list=18%, signal=27% |
| 1565 | GOBP\_REGULATION\_OF\_ATRIAL\_CARDIAC\_MUSCLE\_CELL\_MEMBRANE\_DEPOLARIZATION |  | 4 | -0.60 | -1.10 | 0.390 | 0.824 | 1.000 | 1316 | tags=50%, list=11%, signal=56% |
| 1566 | GOBP\_N\_TERMINAL\_PEPTIDYL\_METHIONINE\_ACETYLATION |  | 6 | -0.53 | -1.10 | 0.355 | 0.824 | 1.000 | 5463 | tags=83%, list=45%, signal=153% |
| 1567 | GOBP\_NEURONAL\_ACTION\_POTENTIAL |  | 16 | -0.39 | -1.10 | 0.329 | 0.824 | 1.000 | 1472 | tags=38%, list=12%, signal=43% |
| 1568 | GOCC\_PROTON\_TRANSPORTING\_TWO\_SECTOR\_ATPASE\_COMPLEX\_CATALYTIC\_DOMAIN |  | 7 | -0.51 | -1.10 | 0.350 | 0.824 | 1.000 | 3730 | tags=57%, list=31%, signal=83% |
| 1569 | GOMF\_ISOMERASE\_ACTIVITY |  | 116 | -0.26 | -1.10 | 0.242 | 0.824 | 1.000 | 1924 | tags=27%, list=16%, signal=32% |
| 1570 | GOBP\_TRANSMISSION\_OF\_NERVE\_IMPULSE |  | 29 | -0.34 | -1.10 | 0.315 | 0.824 | 1.000 | 1472 | tags=28%, list=12%, signal=31% |
| 1571 | GOBP\_POSITIVE\_REGULATION\_OF\_COAGULATION |  | 16 | -0.40 | -1.10 | 0.331 | 0.824 | 1.000 | 581 | tags=13%, list=5%, signal=13% |
| 1572 | GOBP\_REGULATION\_OF\_CHLORIDE\_TRANSPORT |  | 7 | -0.50 | -1.10 | 0.358 | 0.825 | 1.000 | 254 | tags=29%, list=2%, signal=29% |
| 1573 | GOCC\_TORC2\_COMPLEX |  | 12 | -0.43 | -1.10 | 0.345 | 0.824 | 1.000 | 1344 | tags=25%, list=11%, signal=28% |
| 1574 | GOBP\_NEURONAL\_ION\_CHANNEL\_CLUSTERING |  | 8 | -0.48 | -1.10 | 0.349 | 0.824 | 1.000 | 611 | tags=25%, list=5%, signal=26% |
| 1575 | GOMF\_RETINAL\_BINDING |  | 8 | -0.48 | -1.10 | 0.356 | 0.824 | 1.000 | 244 | tags=25%, list=2%, signal=26% |
| 1576 | GOBP\_REGULATION\_OF\_CYTOPLASMIC\_TRANSLATIONAL\_INITIATION |  | 4 | -0.59 | -1.10 | 0.381 | 0.824 | 1.000 | 2352 | tags=50%, list=20%, signal=62% |
| 1577 | GOBP\_PYRIMIDINE\_RIBONUCLEOTIDE\_METABOLIC\_PROCESS |  | 23 | -0.35 | -1.10 | 0.323 | 0.825 | 1.000 | 1922 | tags=39%, list=16%, signal=46% |
| 1578 | GOBP\_NEGATIVE\_REGULATION\_OF\_PROTEIN\_LOCALIZATION\_TO\_CELL\_SURFACE |  | 11 | -0.44 | -1.10 | 0.361 | 0.826 | 1.000 | 4 | tags=9%, list=0%, signal=9% |
| 1579 | GOBP\_POSITIVE\_REGULATION\_OF\_ALCOHOL\_BIOSYNTHETIC\_PROCESS |  | 14 | -0.41 | -1.10 | 0.338 | 0.826 | 1.000 | 1154 | tags=36%, list=10%, signal=39% |
| 1580 | GOBP\_L\_PHENYLALANINE\_METABOLIC\_PROCESS |  | 6 | -0.53 | -1.10 | 0.361 | 0.827 | 1.000 | 4517 | tags=83%, list=38%, signal=133% |
| 1581 | GOBP\_REGULATION\_OF\_NEUROTRANSMITTER\_TRANSPORT |  | 73 | -0.28 | -1.10 | 0.260 | 0.826 | 1.000 | 2617 | tags=36%, list=22%, signal=45% |
| 1582 | GOBP\_NEGATIVE\_REGULATION\_OF\_LEUKOCYTE\_CHEMOTAXIS |  | 12 | -0.43 | -1.10 | 0.338 | 0.826 | 1.000 | 2042 | tags=42%, list=17%, signal=50% |
| 1583 | GOBP\_POSITIVE\_REGULATION\_OF\_SEQUESTERING\_OF\_CALCIUM\_ION |  | 9 | -0.47 | -1.10 | 0.353 | 0.826 | 1.000 | 1815 | tags=33%, list=15%, signal=39% |
| 1584 | GOBP\_METANEPHRIC\_TUBULE\_MORPHOGENESIS |  | 6 | -0.53 | -1.10 | 0.376 | 0.826 | 1.000 | 141 | tags=17%, list=1%, signal=17% |
| 1585 | GOBP\_DORSAL\_SPINAL\_CORD\_DEVELOPMENT |  | 8 | -0.48 | -1.10 | 0.347 | 0.826 | 1.000 | 669 | tags=25%, list=6%, signal=26% |
| 1586 | GOBP\_GDP\_MANNOSE\_METABOLIC\_PROCESS |  | 5 | -0.56 | -1.10 | 0.382 | 0.826 | 1.000 | 1676 | tags=40%, list=14%, signal=46% |
| 1587 | GOBP\_REGULATION\_OF\_MAP\_KINASE\_ACTIVITY |  | 220 | -0.23 | -1.10 | 0.201 | 0.826 | 1.000 | 3022 | tags=27%, list=25%, signal=36% |
| 1588 | GOBP\_RESPONSE\_TO\_NITRIC\_OXIDE |  | 15 | -0.40 | -1.10 | 0.347 | 0.826 | 1.000 | 1290 | tags=20%, list=11%, signal=22% |
| 1589 | GOBP\_PURINE\_CONTAINING\_COMPOUND\_BIOSYNTHETIC\_PROCESS |  | 127 | -0.25 | -1.10 | 0.247 | 0.826 | 1.000 | 2625 | tags=28%, list=22%, signal=36% |
| 1590 | GOBP\_EXTRACELLULAR\_REGULATION\_OF\_SIGNAL\_TRANSDUCTION |  | 9 | -0.47 | -1.10 | 0.354 | 0.827 | 1.000 | 2211 | tags=44%, list=18%, signal=54% |
| 1591 | GOBP\_INOSITOL\_PHOSPHATE\_BIOSYNTHETIC\_PROCESS |  | 18 | -0.38 | -1.10 | 0.324 | 0.828 | 1.000 | 1920 | tags=44%, list=16%, signal=53% |
| 1592 | GOBP\_NEUROTRANSMITTER\_TRANSPORT |  | 135 | -0.25 | -1.10 | 0.243 | 0.828 | 1.000 | 1711 | tags=23%, list=14%, signal=26% |
| 1593 | GOCC\_OUTER\_MITOCHONDRIAL\_MEMBRANE\_PROTEIN\_COMPLEX |  | 16 | -0.39 | -1.10 | 0.344 | 0.828 | 1.000 | 3110 | tags=38%, list=26%, signal=51% |
| 1594 | GOBP\_DETECTION\_OF\_CHEMICAL\_STIMULUS |  | 25 | -0.35 | -1.09 | 0.317 | 0.828 | 1.000 | 2170 | tags=36%, list=18%, signal=44% |
| 1595 | GOBP\_B\_CELL\_ACTIVATION |  | 168 | -0.24 | -1.09 | 0.236 | 0.828 | 1.000 | 3223 | tags=39%, list=27%, signal=52% |
| 1596 | GOBP\_CELLULAR\_BIOGENIC\_AMINE\_METABOLIC\_PROCESS |  | 62 | -0.28 | -1.09 | 0.287 | 0.828 | 1.000 | 2300 | tags=34%, list=19%, signal=42% |
| 1597 | GOBP\_REGULATION\_OF\_RECEPTOR\_BINDING |  | 14 | -0.40 | -1.09 | 0.343 | 0.827 | 1.000 | 1927 | tags=43%, list=16%, signal=51% |
| 1598 | GOBP\_POSITIVE\_REGULATION\_OF\_MYELOID\_LEUKOCYTE\_DIFFERENTIATION |  | 35 | -0.32 | -1.09 | 0.317 | 0.827 | 1.000 | 2986 | tags=46%, list=25%, signal=61% |
| 1599 | GOBP\_NERVOUS\_SYSTEM\_PROCESS |  | 479 | -0.22 | -1.09 | 0.141 | 0.827 | 1.000 | 1717 | tags=19%, list=14%, signal=21% |
| 1600 | GOBP\_ACTIVATION\_OF\_JNKK\_ACTIVITY |  | 6 | -0.52 | -1.09 | 0.366 | 0.827 | 1.000 | 1574 | tags=33%, list=13%, signal=38% |
| 1601 | GOBP\_DNA\_TEMPLATED\_TRANSCRIPTION\_ELONGATION |  | 93 | -0.26 | -1.09 | 0.272 | 0.828 | 1.000 | 3505 | tags=27%, list=29%, signal=38% |
| 1602 | GOMF\_ATPASE\_ACTIVITY\_COUPLED\_TO\_TRANSMEMBRANE\_MOVEMENT\_OF\_IONS\_ROTATIONAL\_MECHANISM |  | 19 | -0.37 | -1.09 | 0.338 | 0.827 | 1.000 | 3769 | tags=37%, list=31%, signal=54% |
| 1603 | GOBP\_ACUTE\_PHASE\_RESPONSE |  | 21 | -0.36 | -1.09 | 0.326 | 0.827 | 1.000 | 1051 | tags=24%, list=9%, signal=26% |
| 1604 | GOMF\_PATTERN\_RECOGNITION\_RECEPTOR\_ACTIVITY |  | 12 | -0.42 | -1.09 | 0.341 | 0.826 | 1.000 | 1392 | tags=25%, list=12%, signal=28% |
| 1605 | GOBP\_PHENOL\_CONTAINING\_COMPOUND\_BIOSYNTHETIC\_PROCESS |  | 24 | -0.35 | -1.09 | 0.315 | 0.827 | 1.000 | 1044 | tags=25%, list=9%, signal=27% |
| 1606 | GOBP\_POSITIVE\_REGULATION\_BY\_HOST\_OF\_VIRAL\_GENOME\_REPLICATION |  | 7 | -0.50 | -1.09 | 0.361 | 0.828 | 1.000 | 130 | tags=14%, list=1%, signal=14% |
| 1607 | GOCC\_SECRETORY\_GRANULE\_MEMBRANE |  | 197 | -0.24 | -1.09 | 0.228 | 0.828 | 1.000 | 1975 | tags=23%, list=16%, signal=27% |
| 1608 | GOBP\_CATECHOLAMINE\_SECRETION |  | 34 | -0.32 | -1.09 | 0.308 | 0.828 | 1.000 | 2628 | tags=44%, list=22%, signal=56% |
| 1609 | GOBP\_ENDOTHELIAL\_CELL\_APOPTOTIC\_PROCESS |  | 37 | -0.32 | -1.09 | 0.299 | 0.828 | 1.000 | 1855 | tags=24%, list=15%, signal=29% |
| 1610 | GOBP\_INTRACELLULAR\_PROTEIN\_TRANSPORT |  | 892 | -0.21 | -1.09 | 0.086 | 0.828 | 1.000 | 2946 | tags=27%, list=25%, signal=33% |
| 1611 | GOBP\_MAINTENANCE\_OF\_GASTROINTESTINAL\_EPITHELIUM |  | 10 | -0.45 | -1.09 | 0.357 | 0.830 | 1.000 | 6633 | tags=100%, list=55%, signal=223% |
| 1612 | GOBP\_B\_CELL\_PROLIFERATION\_INVOLVED\_IN\_IMMUNE\_RESPONSE |  | 4 | -0.59 | -1.09 | 0.389 | 0.829 | 1.000 | 1099 | tags=25%, list=9%, signal=28% |
| 1613 | GOBP\_NEURAL\_TUBE\_PATTERNING |  | 25 | -0.35 | -1.09 | 0.324 | 0.829 | 1.000 | 3365 | tags=48%, list=28%, signal=67% |
| 1614 | GOBP\_POLYOL\_BIOSYNTHETIC\_PROCESS |  | 46 | -0.30 | -1.09 | 0.296 | 0.829 | 1.000 | 1920 | tags=30%, list=16%, signal=36% |
| 1615 | GOBP\_NEGATIVE\_REGULATION\_OF\_LIPID\_KINASE\_ACTIVITY |  | 6 | -0.52 | -1.09 | 0.385 | 0.830 | 1.000 | 2732 | tags=50%, list=23%, signal=65% |
| 1616 | GOCC\_CORVET\_COMPLEX |  | 5 | -0.56 | -1.09 | 0.384 | 0.830 | 1.000 | 2790 | tags=60%, list=23%, signal=78% |
| 1617 | GOBP\_SKELETAL\_MUSCLE\_TISSUE\_REGENERATION |  | 21 | -0.37 | -1.09 | 0.340 | 0.830 | 1.000 | 2255 | tags=38%, list=19%, signal=47% |
| 1618 | GOBP\_REGULATION\_OF\_GUANYL\_NUCLEOTIDE\_EXCHANGE\_FACTOR\_ACTIVITY |  | 7 | -0.50 | -1.09 | 0.374 | 0.830 | 1.000 | 1190 | tags=29%, list=10%, signal=32% |
| 1619 | GOBP\_BONE\_RESORPTION |  | 45 | -0.30 | -1.09 | 0.316 | 0.830 | 1.000 | 1300 | tags=29%, list=11%, signal=32% |
| 1620 | GOCC\_SNO\_S\_RNA\_CONTAINING\_RIBONUCLEOPROTEIN\_COMPLEX |  | 16 | -0.39 | -1.09 | 0.334 | 0.830 | 1.000 | 2464 | tags=38%, list=21%, signal=47% |
| 1621 | GOBP\_REGULATION\_OF\_INOSITOL\_PHOSPHATE\_BIOSYNTHETIC\_PROCESS |  | 9 | -0.46 | -1.09 | 0.362 | 0.830 | 1.000 | 1920 | tags=44%, list=16%, signal=53% |
| 1622 | GOBP\_CELLULAR\_RESPONSE\_TO\_ETHANOL |  | 13 | -0.41 | -1.09 | 0.339 | 0.830 | 1.000 | 1213 | tags=31%, list=10%, signal=34% |
| 1623 | GOBP\_CD40\_SIGNALING\_PATHWAY |  | 11 | -0.43 | -1.09 | 0.351 | 0.830 | 1.000 | 4370 | tags=64%, list=36%, signal=100% |
| 1624 | GOMF\_EXCITATORY\_EXTRACELLULAR\_LIGAND\_GATED\_ION\_CHANNEL\_ACTIVITY |  | 11 | -0.43 | -1.09 | 0.348 | 0.831 | 1.000 | 2170 | tags=64%, list=18%, signal=78% |
| 1625 | GOBP\_STEM\_CELL\_FATE\_COMMITMENT |  | 4 | -0.59 | -1.09 | 0.403 | 0.831 | 1.000 | 166 | tags=25%, list=1%, signal=25% |
| 1626 | GOBP\_BRANCHED\_CHAIN\_AMINO\_ACID\_BIOSYNTHETIC\_PROCESS |  | 3 | -0.65 | -1.09 | 0.406 | 0.831 | 1.000 | 3167 | tags=67%, list=26%, signal=91% |
| 1627 | GOBP\_ADENYLATE\_CYCLASE\_ACTIVATING\_DOPAMINE\_RECEPTOR\_SIGNALING\_PATHWAY |  | 7 | -0.50 | -1.09 | 0.372 | 0.832 | 1.000 | 1289 | tags=43%, list=11%, signal=48% |
| 1628 | GOBP\_RETINAL\_GANGLION\_CELL\_AXON\_GUIDANCE |  | 15 | -0.40 | -1.09 | 0.334 | 0.832 | 1.000 | 2052 | tags=40%, list=17%, signal=48% |
| 1629 | GOBP\_NEGATIVE\_REGULATION\_OF\_CHOLESTEROL\_METABOLIC\_PROCESS |  | 4 | -0.59 | -1.09 | 0.400 | 0.832 | 1.000 | 1894 | tags=50%, list=16%, signal=59% |
| 1630 | GOBP\_POSITIVE\_REGULATION\_OF\_ADIPOSE\_TISSUE\_DEVELOPMENT |  | 3 | -0.64 | -1.09 | 0.409 | 0.832 | 1.000 | 4355 | tags=100%, list=36%, signal=157% |
| 1631 | GOCC\_NADH\_DEHYDROGENASE\_COMPLEX |  | 37 | -0.32 | -1.09 | 0.326 | 0.831 | 1.000 | 4520 | tags=49%, list=38%, signal=78% |
| 1632 | GOBP\_CATECHOL\_CONTAINING\_COMPOUND\_METABOLIC\_PROCESS |  | 29 | -0.33 | -1.09 | 0.313 | 0.832 | 1.000 | 2505 | tags=41%, list=21%, signal=52% |
| 1633 | GOBP\_NEGATIVE\_REGULATION\_OF\_DOUBLE\_STRAND\_BREAK\_REPAIR\_VIA\_NONHOMOLOGOUS\_END\_JOINING |  | 4 | -0.59 | -1.09 | 0.393 | 0.832 | 1.000 | 4895 | tags=100%, list=41%, signal=169% |
| 1634 | GOBP\_CELLULAR\_RESPONSE\_TO\_UV\_A |  | 5 | -0.55 | -1.09 | 0.387 | 0.832 | 1.000 | 295 | tags=20%, list=2%, signal=20% |
| 1635 | GOBP\_REGULATION\_OF\_TOR\_SIGNALING |  | 74 | -0.27 | -1.09 | 0.288 | 0.832 | 1.000 | 2974 | tags=34%, list=25%, signal=45% |
| 1636 | GOMF\_EFFLUX\_TRANSMEMBRANE\_TRANSPORTER\_ACTIVITY |  | 12 | -0.42 | -1.09 | 0.363 | 0.832 | 1.000 | 1902 | tags=42%, list=16%, signal=49% |
| 1637 | GOCC\_A\_BAND |  | 25 | -0.35 | -1.09 | 0.336 | 0.832 | 1.000 | 626 | tags=20%, list=5%, signal=21% |
| 1638 | GOBP\_REGULATION\_OF\_PHOSPHATIDYLINOSITOL\_BIOSYNTHETIC\_PROCESS |  | 4 | -0.59 | -1.09 | 0.405 | 0.831 | 1.000 | 49 | tags=25%, list=0%, signal=25% |
| 1639 | GOBP\_NEGATIVE\_REGULATION\_OF\_VASOCONSTRICTION |  | 6 | -0.52 | -1.09 | 0.382 | 0.833 | 1.000 | 363 | tags=33%, list=3%, signal=34% |
| 1640 | GOBP\_BRONCHUS\_DEVELOPMENT |  | 7 | -0.50 | -1.09 | 0.378 | 0.833 | 1.000 | 382 | tags=14%, list=3%, signal=15% |
| 1641 | GOBP\_POSITIVE\_REGULATION\_OF\_ENDOTHELIAL\_CELL\_PROLIFERATION |  | 70 | -0.27 | -1.08 | 0.305 | 0.836 | 1.000 | 2783 | tags=30%, list=23%, signal=39% |
| 1642 | GOBP\_NEUROPEPTIDE\_SIGNALING\_PATHWAY |  | 33 | -0.32 | -1.08 | 0.318 | 0.836 | 1.000 | 1311 | tags=27%, list=11%, signal=31% |
| 1643 | GOBP\_WATER\_SOLUBLE\_VITAMIN\_BIOSYNTHETIC\_PROCESS |  | 6 | -0.51 | -1.08 | 0.389 | 0.836 | 1.000 | 3725 | tags=50%, list=31%, signal=72% |
| 1644 | GOBP\_NEGATIVE\_REGULATION\_OF\_NERVOUS\_SYSTEM\_DEVELOPMENT |  | 90 | -0.26 | -1.08 | 0.289 | 0.836 | 1.000 | 674 | tags=11%, list=6%, signal=12% |
| 1645 | GOBP\_POSITIVE\_REGULATION\_OF\_MAMMARY\_GLAND\_EPITHELIAL\_CELL\_PROLIFERATION |  | 7 | -0.50 | -1.08 | 0.387 | 0.836 | 1.000 | 1589 | tags=29%, list=13%, signal=33% |
| 1646 | GOMF\_DNA\_POLYMERASE\_ACTIVITY |  | 22 | -0.36 | -1.08 | 0.334 | 0.836 | 1.000 | 1130 | tags=23%, list=9%, signal=25% |
| 1647 | GOBP\_CELLULAR\_RESPONSE\_TO\_ESTRADIOL\_STIMULUS |  | 23 | -0.35 | -1.08 | 0.335 | 0.836 | 1.000 | 878 | tags=22%, list=7%, signal=23% |
| 1648 | GOBP\_T\_CELL\_TOLERANCE\_INDUCTION |  | 6 | -0.52 | -1.08 | 0.381 | 0.837 | 1.000 | 3138 | tags=33%, list=26%, signal=45% |
| 1649 | GOBP\_MAMMALIAN\_OOGENESIS\_STAGE |  | 5 | -0.55 | -1.08 | 0.385 | 0.837 | 1.000 | 3765 | tags=80%, list=31%, signal=116% |
| 1650 | GOBP\_NUCLEOSIDE\_METABOLIC\_PROCESS |  | 77 | -0.27 | -1.08 | 0.291 | 0.838 | 1.000 | 2070 | tags=30%, list=17%, signal=36% |
| 1651 | GOBP\_REGULATION\_OF\_HUMORAL\_IMMUNE\_RESPONSE |  | 38 | -0.31 | -1.08 | 0.326 | 0.838 | 1.000 | 3133 | tags=50%, list=26%, signal=67% |
| 1652 | GOMF\_LIGAND\_GATED\_ION\_CHANNEL\_ACTIVITY |  | 59 | -0.28 | -1.08 | 0.304 | 0.838 | 1.000 | 1691 | tags=25%, list=14%, signal=29% |
| 1653 | GOBP\_NEGATIVE\_REGULATION\_OF\_ANDROGEN\_RECEPTOR\_SIGNALING\_PATHWAY |  | 12 | -0.42 | -1.08 | 0.361 | 0.839 | 1.000 | 2144 | tags=33%, list=18%, signal=41% |
| 1654 | GOBP\_LEUCINE\_METABOLIC\_PROCESS |  | 7 | -0.49 | -1.08 | 0.383 | 0.838 | 1.000 | 1543 | tags=43%, list=13%, signal=49% |
| 1655 | GOBP\_NEGATIVE\_REGULATION\_OF\_GLYCOPROTEIN\_METABOLIC\_PROCESS |  | 13 | -0.41 | -1.08 | 0.360 | 0.838 | 1.000 | 1958 | tags=38%, list=16%, signal=46% |
| 1656 | GOBP\_REGULATION\_OF\_MYOTUBE\_DIFFERENTIATION |  | 19 | -0.37 | -1.08 | 0.349 | 0.838 | 1.000 | 1861 | tags=37%, list=15%, signal=44% |
| 1657 | GOMF\_DOPAMINE\_BINDING |  | 3 | -0.64 | -1.08 | 0.420 | 0.838 | 1.000 | 1289 | tags=67%, list=11%, signal=75% |
| 1658 | GOMF\_MRF\_BINDING |  | 5 | -0.55 | -1.08 | 0.397 | 0.839 | 1.000 | 1209 | tags=40%, list=10%, signal=44% |
| 1659 | GOBP\_CELLULAR\_RESPONSE\_TO\_THYROID\_HORMONE\_STIMULUS |  | 12 | -0.42 | -1.08 | 0.366 | 0.839 | 1.000 | 3654 | tags=42%, list=30%, signal=60% |
| 1660 | GOCC\_EUKARYOTIC\_TRANSLATION\_INITIATION\_FACTOR\_4F\_COMPLEX |  | 9 | -0.46 | -1.08 | 0.366 | 0.839 | 1.000 | 3019 | tags=44%, list=25%, signal=59% |
| 1661 | GOBP\_SEQUESTERING\_OF\_IRON\_ION |  | 3 | -0.63 | -1.08 | 0.409 | 0.839 | 1.000 | 4397 | tags=100%, list=37%, signal=158% |
| 1662 | GOBP\_REGULATION\_OF\_THE\_FORCE\_OF\_HEART\_CONTRACTION |  | 17 | -0.38 | -1.08 | 0.351 | 0.839 | 1.000 | 363 | tags=18%, list=3%, signal=18% |
| 1663 | GOBP\_NEGATIVE\_REGULATION\_OF\_MACROPHAGE\_DERIVED\_FOAM\_CELL\_DIFFERENTIATION |  | 11 | -0.43 | -1.08 | 0.362 | 0.839 | 1.000 | 2337 | tags=36%, list=19%, signal=45% |
| 1664 | GOBP\_MESONEPHRIC\_TUBULE\_MORPHOGENESIS |  | 40 | -0.31 | -1.08 | 0.333 | 0.839 | 1.000 | 2230 | tags=28%, list=19%, signal=34% |
| 1665 | GOBP\_REGULATION\_OF\_INNER\_EAR\_AUDITORY\_RECEPTOR\_CELL\_DIFFERENTIATION |  | 6 | -0.52 | -1.08 | 0.382 | 0.839 | 1.000 | 3624 | tags=50%, list=30%, signal=72% |
| 1666 | GOBP\_REGULATION\_OF\_URINE\_VOLUME |  | 14 | -0.40 | -1.08 | 0.359 | 0.839 | 1.000 | 1759 | tags=29%, list=15%, signal=33% |
| 1667 | GOBP\_PROTON\_TRANSPORTING\_ATP\_SYNTHASE\_COMPLEX\_ASSEMBLY |  | 5 | -0.55 | -1.08 | 0.395 | 0.839 | 1.000 | 5425 | tags=100%, list=45%, signal=182% |
| 1668 | GOBP\_CELLULAR\_RESPONSE\_TO\_DRUG |  | 49 | -0.29 | -1.08 | 0.318 | 0.840 | 1.000 | 979 | tags=16%, list=8%, signal=18% |
| 1669 | GOBP\_REGULATION\_OF\_INTRINSIC\_APOPTOTIC\_SIGNALING\_PATHWAY |  | 108 | -0.25 | -1.08 | 0.276 | 0.841 | 1.000 | 2625 | tags=28%, list=22%, signal=35% |
| 1670 | GOBP\_NUCLEAR\_TRANSCRIBED\_MRNA\_CATABOLIC\_PROCESS\_EXONUCLEOLYTIC\_3\_5 |  | 10 | -0.44 | -1.08 | 0.366 | 0.841 | 1.000 | 3711 | tags=50%, list=31%, signal=72% |
| 1671 | GOBP\_SCARNA\_LOCALIZATION\_TO\_CAJAL\_BODY |  | 4 | -0.58 | -1.08 | 0.406 | 0.842 | 1.000 | 621 | tags=25%, list=5%, signal=26% |
| 1672 | GOMF\_HSP70\_PROTEIN\_BINDING |  | 27 | -0.34 | -1.08 | 0.337 | 0.842 | 1.000 | 1684 | tags=26%, list=14%, signal=30% |
| 1673 | GOBP\_INOSITOL\_PHOSPHATE\_CATABOLIC\_PROCESS |  | 17 | -0.38 | -1.08 | 0.356 | 0.842 | 1.000 | 1084 | tags=18%, list=9%, signal=19% |
| 1674 | GOBP\_NEPHRON\_MORPHOGENESIS |  | 46 | -0.30 | -1.08 | 0.333 | 0.842 | 1.000 | 2230 | tags=28%, list=19%, signal=35% |
| 1675 | GOBP\_INNER\_DYNEIN\_ARM\_ASSEMBLY |  | 4 | -0.58 | -1.08 | 0.411 | 0.841 | 1.000 | 279 | tags=25%, list=2%, signal=26% |
| 1676 | GOBP\_PROTEIN\_ACYLATION |  | 182 | -0.23 | -1.08 | 0.263 | 0.842 | 1.000 | 2541 | tags=23%, list=21%, signal=29% |
| 1677 | GOBP\_DOPAMINERGIC\_NEURON\_DIFFERENTIATION |  | 20 | -0.36 | -1.08 | 0.348 | 0.841 | 1.000 | 1656 | tags=25%, list=14%, signal=29% |
| 1678 | GOBP\_BRANCHING\_INVOLVED\_IN\_PROSTATE\_GLAND\_MORPHOGENESIS |  | 10 | -0.44 | -1.08 | 0.362 | 0.841 | 1.000 | 350 | tags=20%, list=3%, signal=21% |
| 1679 | GOBP\_POSITIVE\_REGULATION\_OF\_CELL\_GROWTH\_INVOLVED\_IN\_CARDIAC\_MUSCLE\_CELL\_DEVELOPMENT |  | 5 | -0.54 | -1.08 | 0.407 | 0.841 | 1.000 | 2255 | tags=40%, list=19%, signal=49% |
| 1680 | GOBP\_REGULATION\_OF\_FAT\_CELL\_DIFFERENTIATION |  | 89 | -0.26 | -1.08 | 0.300 | 0.842 | 1.000 | 980 | tags=13%, list=8%, signal=15% |
| 1681 | GOCC\_MANCHETTE |  | 5 | -0.54 | -1.08 | 0.397 | 0.841 | 1.000 | 1711 | tags=40%, list=14%, signal=47% |
| 1682 | GOBP\_PEROXISOMAL\_MEMBRANE\_TRANSPORT |  | 17 | -0.38 | -1.08 | 0.361 | 0.841 | 1.000 | 3187 | tags=35%, list=27%, signal=48% |
| 1683 | GOBP\_PHOSPHATIDYLINOSITOL\_METABOLIC\_PROCESS |  | 143 | -0.24 | -1.08 | 0.273 | 0.841 | 1.000 | 2867 | tags=33%, list=24%, signal=43% |
| 1684 | GOBP\_NEGATIVE\_REGULATION\_OF\_B\_CELL\_DIFFERENTIATION |  | 4 | -0.58 | -1.08 | 0.409 | 0.841 | 1.000 | 166 | tags=25%, list=1%, signal=25% |
| 1685 | GOBP\_REGULATION\_OF\_OXIDATIVE\_STRESS\_INDUCED\_CELL\_DEATH |  | 43 | -0.30 | -1.08 | 0.328 | 0.841 | 1.000 | 2625 | tags=33%, list=22%, signal=42% |
| 1686 | GOBP\_HETEROPHILIC\_CELL\_CELL\_ADHESION\_VIA\_PLASMA\_MEMBRANE\_CELL\_ADHESION\_MOLECULES |  | 26 | -0.34 | -1.08 | 0.352 | 0.840 | 1.000 | 2132 | tags=31%, list=18%, signal=37% |
| 1687 | GOCC\_ACROSOMAL\_MEMBRANE |  | 9 | -0.45 | -1.07 | 0.372 | 0.840 | 1.000 | 422 | tags=22%, list=4%, signal=23% |
| 1688 | GOBP\_NEUROTRANSMITTER\_BIOSYNTHETIC\_PROCESS |  | 8 | -0.47 | -1.07 | 0.373 | 0.841 | 1.000 | 1724 | tags=38%, list=14%, signal=44% |
| 1689 | GOMF\_OLIGOPEPTIDE\_BINDING |  | 9 | -0.46 | -1.07 | 0.372 | 0.840 | 1.000 | 740 | tags=22%, list=6%, signal=24% |
| 1690 | GOMF\_NUCLEAR\_EXPORT\_SIGNAL\_RECEPTOR\_ACTIVITY |  | 10 | -0.44 | -1.07 | 0.380 | 0.840 | 1.000 | 4390 | tags=60%, list=37%, signal=94% |
| 1691 | GOMF\_FLAVIN\_ADENINE\_DINUCLEOTIDE\_BINDING |  | 65 | -0.28 | -1.07 | 0.313 | 0.839 | 1.000 | 1697 | tags=23%, list=14%, signal=27% |
| 1692 | GOCC\_ZONA\_PELLUCIDA\_RECEPTOR\_COMPLEX |  | 5 | -0.54 | -1.07 | 0.409 | 0.839 | 1.000 | 5474 | tags=100%, list=46%, signal=184% |
| 1693 | GOBP\_NEGATIVE\_REGULATION\_OF\_SMOOTH\_MUSCLE\_CONTRACTION |  | 11 | -0.43 | -1.07 | 0.370 | 0.839 | 1.000 | 3142 | tags=55%, list=26%, signal=74% |
| 1694 | GOBP\_POSITIVE\_REGULATION\_OF\_TRANSLATIONAL\_ELONGATION |  | 3 | -0.63 | -1.07 | 0.420 | 0.839 | 1.000 | 1552 | tags=67%, list=13%, signal=77% |
| 1695 | GOBP\_NEGATIVE\_REGULATION\_OF\_CREB\_TRANSCRIPTION\_FACTOR\_ACTIVITY |  | 3 | -0.63 | -1.07 | 0.424 | 0.839 | 1.000 | 1410 | tags=67%, list=12%, signal=76% |
| 1696 | GOBP\_NEGATIVE\_REGULATION\_OF\_NECROTIC\_CELL\_DEATH |  | 10 | -0.43 | -1.07 | 0.370 | 0.838 | 1.000 | 428 | tags=20%, list=4%, signal=21% |
| 1697 | GOBP\_MITOCHONDRIAL\_TRNA\_METHYLATION |  | 4 | -0.58 | -1.07 | 0.417 | 0.841 | 1.000 | 3825 | tags=75%, list=32%, signal=110% |
| 1698 | GOMF\_DOPAMINE\_NEUROTRANSMITTER\_RECEPTOR\_ACTIVITY |  | 3 | -0.64 | -1.07 | 0.429 | 0.841 | 1.000 | 1289 | tags=67%, list=11%, signal=75% |
| 1699 | GOBP\_REGULATION\_OF\_ANGIOTENSIN\_LEVELS\_IN\_BLOOD |  | 8 | -0.47 | -1.07 | 0.381 | 0.841 | 1.000 | 1510 | tags=38%, list=13%, signal=43% |
| 1700 | GOBP\_DENDRITE\_SELF\_AVOIDANCE |  | 12 | -0.42 | -1.07 | 0.383 | 0.842 | 1.000 | 422 | tags=25%, list=4%, signal=26% |
| 1701 | GOBP\_AUTONOMIC\_NERVOUS\_SYSTEM\_DEVELOPMENT |  | 26 | -0.34 | -1.07 | 0.350 | 0.845 | 1.000 | 640 | tags=15%, list=5%, signal=16% |
| 1702 | GOMF\_H3\_HISTONE\_ACETYLTRANSFERASE\_ACTIVITY |  | 7 | -0.49 | -1.07 | 0.394 | 0.845 | 1.000 | 1929 | tags=43%, list=16%, signal=51% |
| 1703 | GOCC\_CONNEXIN\_COMPLEX |  | 8 | -0.47 | -1.07 | 0.388 | 0.845 | 1.000 | 1424 | tags=25%, list=12%, signal=28% |
| 1704 | GOBP\_ENDONUCLEOLYTIC\_CLEAVAGE\_IN\_5\_ETS\_OF\_TRICISTRONIC\_RRNA\_TRANSCRIPT\_SSU\_RRNA\_5\_8S\_RRNA\_LSU\_RRNA |  | 5 | -0.54 | -1.07 | 0.406 | 0.845 | 1.000 | 4068 | tags=60%, list=34%, signal=91% |
| 1705 | GOBP\_NEGATIVE\_REGULATION\_OF\_MONONUCLEAR\_CELL\_MIGRATION |  | 15 | -0.39 | -1.07 | 0.353 | 0.846 | 1.000 | 2057 | tags=40%, list=17%, signal=48% |
| 1706 | GOMF\_MONOSACCHARIDE\_BINDING |  | 47 | -0.30 | -1.07 | 0.321 | 0.848 | 1.000 | 2027 | tags=32%, list=17%, signal=38% |
| 1707 | GOBP\_CELL\_PROLIFERATION\_IN\_MIDBRAIN |  | 5 | -0.54 | -1.07 | 0.400 | 0.849 | 1.000 | 1900 | tags=40%, list=16%, signal=47% |
| 1708 | GOBP\_REGULATION\_OF\_SUPEROXIDE\_ANION\_GENERATION |  | 13 | -0.41 | -1.07 | 0.371 | 0.849 | 1.000 | 4269 | tags=77%, list=36%, signal=119% |
| 1709 | GOBP\_NEUROTRANSMITTER\_GATED\_ION\_CHANNEL\_CLUSTERING |  | 5 | -0.54 | -1.07 | 0.405 | 0.848 | 1.000 | 475 | tags=20%, list=4%, signal=21% |
| 1710 | GOBP\_PULMONARY\_VALVE\_MORPHOGENESIS |  | 14 | -0.40 | -1.07 | 0.377 | 0.849 | 1.000 | 2018 | tags=29%, list=17%, signal=34% |
| 1711 | GOBP\_INSEMINATION |  | 8 | -0.47 | -1.07 | 0.389 | 0.849 | 1.000 | 521 | tags=25%, list=4%, signal=26% |
| 1712 | GOBP\_PYRIMIDINE\_NUCLEOSIDE\_TRIPHOSPHATE\_METABOLIC\_PROCESS |  | 19 | -0.37 | -1.07 | 0.373 | 0.848 | 1.000 | 834 | tags=26%, list=7%, signal=28% |
| 1713 | GOBP\_REGULATION\_OF\_OXIDOREDUCTASE\_ACTIVITY |  | 66 | -0.27 | -1.07 | 0.328 | 0.849 | 1.000 | 1500 | tags=20%, list=12%, signal=22% |
| 1714 | GOBP\_REGULATION\_OF\_KETONE\_BIOSYNTHETIC\_PROCESS |  | 14 | -0.40 | -1.07 | 0.367 | 0.848 | 1.000 | 878 | tags=21%, list=7%, signal=23% |
| 1715 | GOMF\_JUN\_KINASE\_KINASE\_KINASE\_ACTIVITY |  | 5 | -0.54 | -1.07 | 0.413 | 0.849 | 1.000 | 1574 | tags=40%, list=13%, signal=46% |
| 1716 | GOCC\_SYNAPSE |  | 827 | -0.20 | -1.07 | 0.159 | 0.849 | 1.000 | 1955 | tags=20%, list=16%, signal=22% |
| 1717 | GOMF\_POLYSACCHARIDE\_BINDING |  | 11 | -0.43 | -1.07 | 0.376 | 0.849 | 1.000 | 3 | tags=9%, list=0%, signal=9% |
| 1718 | GOMF\_RIBOSOMAL\_SMALL\_SUBUNIT\_BINDING |  | 16 | -0.39 | -1.07 | 0.378 | 0.849 | 1.000 | 2130 | tags=31%, list=18%, signal=38% |
| 1719 | GOCC\_OLIGOSACCHARYLTRANSFERASE\_COMPLEX |  | 11 | -0.43 | -1.07 | 0.376 | 0.850 | 1.000 | 83 | tags=9%, list=1%, signal=9% |
| 1720 | GOCC\_PCG\_PROTEIN\_COMPLEX |  | 37 | -0.31 | -1.07 | 0.342 | 0.851 | 1.000 | 4005 | tags=46%, list=33%, signal=69% |
| 1721 | GOBP\_GLYCOSYL\_COMPOUND\_BIOSYNTHETIC\_PROCESS |  | 33 | -0.32 | -1.07 | 0.358 | 0.851 | 1.000 | 2669 | tags=39%, list=22%, signal=51% |
| 1722 | GOMF\_ENDOPEPTIDASE\_ACTIVATOR\_ACTIVITY |  | 6 | -0.51 | -1.07 | 0.404 | 0.851 | 1.000 | 3350 | tags=67%, list=28%, signal=92% |
| 1723 | GOBP\_TISSUE\_REMODELING |  | 109 | -0.25 | -1.06 | 0.305 | 0.852 | 1.000 | 1061 | tags=19%, list=9%, signal=21% |
| 1724 | GOBP\_AMINOGLYCAN\_BIOSYNTHETIC\_PROCESS |  | 83 | -0.26 | -1.06 | 0.317 | 0.853 | 1.000 | 2087 | tags=27%, list=17%, signal=32% |
| 1725 | GOBP\_DETECTION\_OF\_MECHANICAL\_STIMULUS\_INVOLVED\_IN\_SENSORY\_PERCEPTION |  | 16 | -0.38 | -1.06 | 0.373 | 0.853 | 1.000 | 2582 | tags=44%, list=21%, signal=56% |
| 1726 | GOMF\_GAP\_JUNCTION\_CHANNEL\_ACTIVITY |  | 8 | -0.47 | -1.06 | 0.399 | 0.853 | 1.000 | 1424 | tags=25%, list=12%, signal=28% |
| 1727 | GOBP\_NEGATIVE\_CHEMOTAXIS |  | 35 | -0.31 | -1.06 | 0.350 | 0.852 | 1.000 | 674 | tags=17%, list=6%, signal=18% |
| 1728 | GOBP\_POSITIVE\_REGULATION\_OF\_LYASE\_ACTIVITY |  | 13 | -0.41 | -1.06 | 0.382 | 0.852 | 1.000 | 2936 | tags=46%, list=24%, signal=61% |
| 1729 | GOBP\_CELLULAR\_RESPONSE\_TO\_CAFFEINE |  | 7 | -0.49 | -1.06 | 0.403 | 0.853 | 1.000 | 2804 | tags=57%, list=23%, signal=75% |
| 1730 | GOBP\_REGULATION\_OF\_G\_PROTEIN\_COUPLED\_RECEPTOR\_SIGNALING\_PATHWAY |  | 89 | -0.26 | -1.06 | 0.315 | 0.853 | 1.000 | 947 | tags=16%, list=8%, signal=17% |
| 1731 | GOBP\_STEROID\_HORMONE\_BIOSYNTHETIC\_PROCESS |  | 29 | -0.33 | -1.06 | 0.373 | 0.853 | 1.000 | 1171 | tags=24%, list=10%, signal=27% |
| 1732 | GOBP\_SEROTONIN\_METABOLIC\_PROCESS |  | 5 | -0.54 | -1.06 | 0.408 | 0.853 | 1.000 | 471 | tags=40%, list=4%, signal=42% |
| 1733 | GOBP\_MICROGLIAL\_CELL\_PROLIFERATION |  | 5 | -0.53 | -1.06 | 0.417 | 0.855 | 1.000 | 964 | tags=40%, list=8%, signal=43% |
| 1734 | GOBP\_MULTI\_ORGANISM\_CELLULAR\_PROCESS |  | 13 | -0.40 | -1.06 | 0.376 | 0.855 | 1.000 | 1859 | tags=31%, list=15%, signal=36% |
| 1735 | GOMF\_EPHRIN\_RECEPTOR\_ACTIVITY |  | 10 | -0.44 | -1.06 | 0.380 | 0.855 | 1.000 | 885 | tags=20%, list=7%, signal=22% |
| 1736 | GOBP\_CELL\_ADHESION\_MEDIATED\_BY\_INTEGRIN |  | 56 | -0.28 | -1.06 | 0.353 | 0.857 | 1.000 | 1557 | tags=23%, list=13%, signal=27% |
| 1737 | GOBP\_CARDIAC\_MUSCLE\_CELL\_MEMBRANE\_REPOLARIZATION |  | 24 | -0.34 | -1.06 | 0.364 | 0.857 | 1.000 | 728 | tags=17%, list=6%, signal=18% |
| 1738 | GOMF\_ISOPEPTIDASE\_ACTIVITY |  | 15 | -0.39 | -1.06 | 0.374 | 0.856 | 1.000 | 1934 | tags=27%, list=16%, signal=32% |
| 1739 | GOCC\_CLATHRIN\_COATED\_VESICLE\_MEMBRANE |  | 76 | -0.27 | -1.06 | 0.321 | 0.856 | 1.000 | 2986 | tags=33%, list=25%, signal=44% |
| 1740 | GOBP\_FOREBRAIN\_NEURON\_DEVELOPMENT |  | 17 | -0.38 | -1.06 | 0.383 | 0.856 | 1.000 | 2525 | tags=35%, list=21%, signal=45% |
| 1741 | GOBP\_POSITIVE\_REGULATION\_OF\_APOPTOTIC\_PROCESS\_INVOLVED\_IN\_DEVELOPMENT |  | 3 | -0.64 | -1.06 | 0.446 | 0.856 | 1.000 | 4373 | tags=100%, list=36%, signal=157% |
| 1742 | GOBP\_POSITIVE\_REGULATION\_OF\_EPITHELIAL\_CELL\_DIFFERENTIATION |  | 23 | -0.35 | -1.06 | 0.367 | 0.858 | 1.000 | 2900 | tags=35%, list=24%, signal=46% |
| 1743 | GOBP\_IMMATURE\_T\_CELL\_PROLIFERATION |  | 8 | -0.46 | -1.06 | 0.398 | 0.858 | 1.000 | 1656 | tags=38%, list=14%, signal=43% |
| 1744 | GOBP\_NEGATIVE\_REGULATION\_OF\_NEURON\_PROJECTION\_DEVELOPMENT |  | 93 | -0.26 | -1.06 | 0.317 | 0.858 | 1.000 | 1329 | tags=16%, list=11%, signal=18% |
| 1745 | GOMF\_DYNEIN\_INTERMEDIATE\_CHAIN\_BINDING |  | 13 | -0.41 | -1.06 | 0.376 | 0.858 | 1.000 | 21 | tags=8%, list=0%, signal=8% |
| 1746 | GOBP\_DETECTION\_OF\_CHEMICAL\_STIMULUS\_INVOLVED\_IN\_SENSORY\_PERCEPTION\_OF\_TASTE |  | 3 | -0.63 | -1.06 | 0.438 | 0.859 | 1.000 | 1033 | tags=67%, list=9%, signal=73% |
| 1747 | GOBP\_NEURON\_MIGRATION |  | 97 | -0.25 | -1.06 | 0.321 | 0.859 | 1.000 | 1742 | tags=18%, list=15%, signal=20% |
| 1748 | GOBP\_AMINOGLYCAN\_METABOLIC\_PROCESS |  | 121 | -0.24 | -1.06 | 0.318 | 0.859 | 1.000 | 2087 | tags=25%, list=17%, signal=30% |
| 1749 | GOBP\_POSITIVE\_REGULATION\_OF\_CYCLASE\_ACTIVITY |  | 13 | -0.41 | -1.06 | 0.386 | 0.858 | 1.000 | 902 | tags=23%, list=8%, signal=25% |
| 1750 | GOCC\_CALYX\_OF\_HELD |  | 11 | -0.43 | -1.06 | 0.384 | 0.858 | 1.000 | 1943 | tags=45%, list=16%, signal=54% |
| 1751 | GOBP\_REGULATION\_OF\_RELAXATION\_OF\_CARDIAC\_MUSCLE |  | 4 | -0.58 | -1.06 | 0.425 | 0.858 | 1.000 | 3048 | tags=75%, list=25%, signal=100% |
| 1752 | GOMF\_NUCLEOTIDYLTRANSFERASE\_ACTIVITY |  | 85 | -0.26 | -1.06 | 0.345 | 0.858 | 1.000 | 2953 | tags=32%, list=25%, signal=42% |
| 1753 | GOCC\_NUCLEOID |  | 33 | -0.32 | -1.06 | 0.357 | 0.858 | 1.000 | 4649 | tags=70%, list=39%, signal=113% |
| 1754 | GOBP\_CATION\_TRANSMEMBRANE\_TRANSPORT |  | 516 | -0.21 | -1.06 | 0.236 | 0.858 | 1.000 | 1948 | tags=21%, list=16%, signal=23% |
| 1755 | GOMF\_TRANSFERASE\_ACTIVITY\_TRANSFERRING\_ALKYL\_OR\_ARYL\_OTHER\_THAN\_METHYL\_GROUPS |  | 42 | -0.30 | -1.06 | 0.354 | 0.857 | 1.000 | 1888 | tags=26%, list=16%, signal=31% |
| 1756 | GOBP\_CELLULAR\_RESPONSE\_TO\_REACTIVE\_NITROGEN\_SPECIES |  | 13 | -0.40 | -1.06 | 0.372 | 0.858 | 1.000 | 878 | tags=15%, list=7%, signal=17% |
| 1757 | GOBP\_CYCLOOXYGENASE\_PATHWAY |  | 8 | -0.46 | -1.06 | 0.396 | 0.858 | 1.000 | 3292 | tags=63%, list=27%, signal=86% |
| 1758 | GOBP\_L\_SERINE\_METABOLIC\_PROCESS |  | 7 | -0.48 | -1.06 | 0.411 | 0.859 | 1.000 | 1595 | tags=29%, list=13%, signal=33% |
| 1759 | GOBP\_ASPARAGINE\_METABOLIC\_PROCESS |  | 5 | -0.54 | -1.06 | 0.426 | 0.859 | 1.000 | 3726 | tags=80%, list=31%, signal=116% |
| 1760 | GOBP\_REGULATION\_OF\_CIRCADIAN\_SLEEP\_WAKE\_CYCLE |  | 9 | -0.45 | -1.06 | 0.380 | 0.858 | 1.000 | 1769 | tags=44%, list=15%, signal=52% |
| 1761 | GOBP\_MULTI\_MULTICELLULAR\_ORGANISM\_PROCESS |  | 119 | -0.24 | -1.06 | 0.319 | 0.859 | 1.000 | 1670 | tags=20%, list=14%, signal=23% |
| 1762 | GOBP\_METAL\_ION\_HOMEOSTASIS |  | 392 | -0.21 | -1.06 | 0.244 | 0.859 | 1.000 | 1852 | tags=21%, list=15%, signal=24% |
| 1763 | GOBP\_SKELETAL\_MUSCLE\_ADAPTATION |  | 14 | -0.39 | -1.06 | 0.385 | 0.859 | 1.000 | 1003 | tags=29%, list=8%, signal=31% |
| 1764 | GOMF\_OPSONIN\_BINDING |  | 12 | -0.41 | -1.06 | 0.389 | 0.860 | 1.000 | 4116 | tags=67%, list=34%, signal=101% |
| 1765 | GOBP\_REGULATION\_OF\_SUPEROXIDE\_DISMUTASE\_ACTIVITY |  | 4 | -0.57 | -1.06 | 0.446 | 0.860 | 1.000 | 2505 | tags=50%, list=21%, signal=63% |
| 1766 | GOBP\_COPPER\_ION\_HOMEOSTASIS |  | 11 | -0.42 | -1.06 | 0.392 | 0.860 | 1.000 | 1326 | tags=18%, list=11%, signal=20% |
| 1767 | GOBP\_PURINE\_NUCLEOSIDE\_MONOPHOSPHATE\_METABOLIC\_PROCESS |  | 34 | -0.31 | -1.06 | 0.367 | 0.860 | 1.000 | 1309 | tags=24%, list=11%, signal=26% |
| 1768 | GOBP\_POSITIVE\_REGULATION\_OF\_NEUROBLAST\_PROLIFERATION |  | 16 | -0.38 | -1.06 | 0.378 | 0.859 | 1.000 | 2525 | tags=38%, list=21%, signal=47% |
| 1769 | GOMF\_SEMAPHORIN\_RECEPTOR\_ACTIVITY |  | 9 | -0.44 | -1.05 | 0.388 | 0.860 | 1.000 | 1034 | tags=33%, list=9%, signal=36% |
| 1770 | GOCC\_EXTRINSIC\_COMPONENT\_OF\_ENDOPLASMIC\_RETICULUM\_MEMBRANE |  | 5 | -0.53 | -1.05 | 0.413 | 0.860 | 1.000 | 624 | tags=20%, list=5%, signal=21% |
| 1771 | GOMF\_SUPEROXIDE\_GENERATING\_NAD\_P\_H\_OXIDASE\_ACTIVITY |  | 6 | -0.51 | -1.05 | 0.409 | 0.860 | 1.000 | 1292 | tags=50%, list=11%, signal=56% |
| 1772 | GOMF\_2\_OXOGLUTARATE\_DEPENDENT\_DIOXYGENASE\_ACTIVITY |  | 37 | -0.31 | -1.05 | 0.361 | 0.860 | 1.000 | 2646 | tags=38%, list=22%, signal=48% |
| 1773 | GOMF\_RNA\_POLYMERASE\_BINDING |  | 50 | -0.29 | -1.05 | 0.353 | 0.860 | 1.000 | 1465 | tags=12%, list=12%, signal=14% |
| 1774 | GOBP\_VACUOLE\_FUSION |  | 3 | -0.63 | -1.05 | 0.449 | 0.859 | 1.000 | 3511 | tags=67%, list=29%, signal=94% |
| 1775 | GOBP\_UBIQUITIN\_DEPENDENT\_SMAD\_PROTEIN\_CATABOLIC\_PROCESS |  | 6 | -0.51 | -1.05 | 0.422 | 0.859 | 1.000 | 2305 | tags=33%, list=19%, signal=41% |
| 1776 | GOBP\_NEGATIVE\_REGULATION\_OF\_REACTIVE\_OXYGEN\_SPECIES\_BIOSYNTHETIC\_PROCESS |  | 19 | -0.36 | -1.05 | 0.378 | 0.859 | 1.000 | 507 | tags=21%, list=4%, signal=22% |
| 1777 | GOMF\_CALCIUM\_DEPENDENT\_PHOSPHOLIPASE\_A2\_ACTIVITY |  | 5 | -0.54 | -1.05 | 0.434 | 0.859 | 1.000 | 787 | tags=40%, list=7%, signal=43% |
| 1778 | GOBP\_CELLULAR\_GLUCURONIDATION |  | 4 | -0.57 | -1.05 | 0.438 | 0.859 | 1.000 | 1967 | tags=50%, list=16%, signal=60% |
| 1779 | GOBP\_EPITHELIAL\_CELL\_CELL\_ADHESION |  | 14 | -0.40 | -1.05 | 0.385 | 0.859 | 1.000 | 212 | tags=21%, list=2%, signal=22% |
| 1780 | GOMF\_PHOSPHATASE\_INHIBITOR\_ACTIVITY |  | 24 | -0.34 | -1.05 | 0.381 | 0.860 | 1.000 | 3644 | tags=46%, list=30%, signal=66% |
| 1781 | GOBP\_DETECTION\_OF\_EXTERNAL\_BIOTIC\_STIMULUS |  | 8 | -0.46 | -1.05 | 0.412 | 0.861 | 1.000 | 4929 | tags=63%, list=41%, signal=106% |
| 1782 | GOCC\_CUL3\_RING\_UBIQUITIN\_LIGASE\_COMPLEX |  | 32 | -0.31 | -1.05 | 0.370 | 0.861 | 1.000 | 1959 | tags=19%, list=16%, signal=22% |
| 1783 | GOBP\_B\_CELL\_HOMEOSTASIS |  | 22 | -0.35 | -1.05 | 0.383 | 0.861 | 1.000 | 3213 | tags=50%, list=27%, signal=68% |
| 1784 | GOMF\_GLYCOLIPID\_BINDING |  | 17 | -0.37 | -1.05 | 0.378 | 0.861 | 1.000 | 1875 | tags=35%, list=16%, signal=42% |
| 1785 | GOMF\_AMINOPEPTIDASE\_ACTIVITY |  | 31 | -0.32 | -1.05 | 0.373 | 0.861 | 1.000 | 2190 | tags=32%, list=18%, signal=39% |
| 1786 | GOMF\_SH2\_DOMAIN\_BINDING |  | 28 | -0.33 | -1.05 | 0.375 | 0.862 | 1.000 | 3197 | tags=36%, list=27%, signal=49% |
| 1787 | GOBP\_PH\_ELEVATION |  | 6 | -0.50 | -1.05 | 0.415 | 0.863 | 1.000 | 1403 | tags=50%, list=12%, signal=57% |
| 1788 | GOBP\_CRISTAE\_FORMATION |  | 16 | -0.38 | -1.05 | 0.383 | 0.862 | 1.000 | 3599 | tags=50%, list=30%, signal=71% |
| 1789 | GOBP\_REGULATION\_OF\_ACUTE\_INFLAMMATORY\_RESPONSE\_TO\_ANTIGENIC\_STIMULUS |  | 7 | -0.48 | -1.05 | 0.403 | 0.863 | 1.000 | 3035 | tags=71%, list=25%, signal=96% |
| 1790 | GOBP\_NEGATIVE\_REGULATION\_OF\_DELAYED\_RECTIFIER\_POTASSIUM\_CHANNEL\_ACTIVITY |  | 6 | -0.50 | -1.05 | 0.422 | 0.862 | 1.000 | 1002 | tags=33%, list=8%, signal=36% |
| 1791 | GOCC\_CHROMATIN\_SILENCING\_COMPLEX |  | 7 | -0.49 | -1.05 | 0.417 | 0.862 | 1.000 | 4125 | tags=71%, list=34%, signal=109% |
| 1792 | GOBP\_PROTEIN\_TRANSMEMBRANE\_TRANSPORT |  | 47 | -0.29 | -1.05 | 0.358 | 0.863 | 1.000 | 3284 | tags=38%, list=27%, signal=53% |
| 1793 | GOBP\_CARBON\_DIOXIDE\_TRANSPORT |  | 4 | -0.57 | -1.05 | 0.448 | 0.863 | 1.000 | 1061 | tags=25%, list=9%, signal=27% |
| 1794 | GOBP\_SA\_NODE\_CELL\_TO\_ATRIAL\_CARDIAC\_MUSCLE\_CELL\_COMMUNICATION |  | 7 | -0.47 | -1.05 | 0.417 | 0.862 | 1.000 | 1316 | tags=43%, list=11%, signal=48% |
| 1795 | GOBP\_POSITIVE\_REGULATION\_OF\_EPITHELIAL\_CELL\_PROLIFERATION |  | 132 | -0.24 | -1.05 | 0.341 | 0.864 | 1.000 | 2789 | tags=30%, list=23%, signal=38% |
| 1796 | GOMF\_GLUTATHIONE\_DISULFIDE\_OXIDOREDUCTASE\_ACTIVITY |  | 4 | -0.57 | -1.05 | 0.438 | 0.864 | 1.000 | 3324 | tags=50%, list=28%, signal=69% |
| 1797 | GOBP\_RNA\_PROCESSING |  | 712 | -0.20 | -1.05 | 0.239 | 0.863 | 1.000 | 3230 | tags=26%, list=27%, signal=33% |
| 1798 | GOBP\_MOTOR\_NEURON\_AXON\_GUIDANCE |  | 17 | -0.37 | -1.05 | 0.389 | 0.864 | 1.000 | 2052 | tags=29%, list=17%, signal=35% |
| 1799 | GOCC\_H3\_HISTONE\_ACETYLTRANSFERASE\_COMPLEX |  | 10 | -0.43 | -1.05 | 0.405 | 0.864 | 1.000 | 1929 | tags=40%, list=16%, signal=48% |
| 1800 | GOBP\_POSITIVE\_REGULATION\_OF\_RNA\_SPLICING |  | 26 | -0.33 | -1.05 | 0.389 | 0.865 | 1.000 | 302 | tags=8%, list=3%, signal=8% |
| 1801 | GOBP\_COLUMNAR\_CUBOIDAL\_EPITHELIAL\_CELL\_DEVELOPMENT |  | 34 | -0.31 | -1.05 | 0.381 | 0.867 | 1.000 | 1418 | tags=18%, list=12%, signal=20% |
| 1802 | GOMF\_ADENYLATE\_KINASE\_ACTIVITY |  | 4 | -0.57 | -1.05 | 0.444 | 0.867 | 1.000 | 2588 | tags=50%, list=22%, signal=64% |
| 1803 | GOMF\_NF\_KAPPAB\_BINDING |  | 24 | -0.34 | -1.05 | 0.390 | 0.867 | 1.000 | 3896 | tags=46%, list=32%, signal=68% |
| 1804 | GOMF\_MOLYBDOPTERIN\_COFACTOR\_BINDING |  | 3 | -0.62 | -1.05 | 0.455 | 0.869 | 1.000 | 284 | tags=33%, list=2%, signal=34% |
| 1805 | GOBP\_POSITIVE\_REGULATION\_OF\_INTRINSIC\_APOPTOTIC\_SIGNALING\_PATHWAY |  | 38 | -0.30 | -1.05 | 0.384 | 0.868 | 1.000 | 2194 | tags=32%, list=18%, signal=39% |
| 1806 | GOMF\_THIOREDOXIN\_PEROXIDASE\_ACTIVITY |  | 3 | -0.62 | -1.05 | 0.467 | 0.868 | 1.000 | 1188 | tags=33%, list=10%, signal=37% |
| 1807 | GOMF\_PURINE\_NUCLEOTIDE\_TRANSMEMBRANE\_TRANSPORTER\_ACTIVITY |  | 14 | -0.39 | -1.05 | 0.385 | 0.868 | 1.000 | 2911 | tags=50%, list=24%, signal=66% |
| 1808 | GOBP\_MEMBRANE\_REPOLARIZATION\_DURING\_ACTION\_POTENTIAL |  | 17 | -0.37 | -1.05 | 0.396 | 0.868 | 1.000 | 611 | tags=12%, list=5%, signal=12% |
| 1809 | GOBP\_REGULATION\_OF\_PHOSPHOLIPID\_BIOSYNTHETIC\_PROCESS |  | 14 | -0.39 | -1.05 | 0.394 | 0.868 | 1.000 | 426 | tags=14%, list=4%, signal=15% |
| 1810 | GOBP\_REGULATION\_OF\_UBIQUITIN\_PROTEIN\_TRANSFERASE\_ACTIVITY |  | 45 | -0.29 | -1.05 | 0.376 | 0.868 | 1.000 | 2672 | tags=33%, list=22%, signal=43% |
| 1811 | GOBP\_SPERM\_MOTILITY |  | 43 | -0.29 | -1.04 | 0.379 | 0.869 | 1.000 | 2803 | tags=30%, list=23%, signal=39% |
| 1812 | GOBP\_POSTSYNAPTIC\_MEMBRANE\_ASSEMBLY |  | 6 | -0.50 | -1.04 | 0.424 | 0.870 | 1.000 | 2822 | tags=50%, list=23%, signal=65% |
| 1813 | GOBP\_ALPHA\_AMINO\_ACID\_BIOSYNTHETIC\_PROCESS |  | 43 | -0.29 | -1.04 | 0.380 | 0.871 | 1.000 | 1809 | tags=19%, list=15%, signal=22% |
| 1814 | GOCC\_CELL\_BODY |  | 357 | -0.21 | -1.04 | 0.297 | 0.873 | 1.000 | 1322 | tags=14%, list=11%, signal=15% |
| 1815 | GOBP\_REACTIVE\_NITROGEN\_SPECIES\_METABOLIC\_PROCESS |  | 51 | -0.28 | -1.04 | 0.367 | 0.873 | 1.000 | 1445 | tags=25%, list=12%, signal=29% |
| 1816 | GOBP\_POSITIVE\_REGULATION\_OF\_FIBROBLAST\_APOPTOTIC\_PROCESS |  | 7 | -0.47 | -1.04 | 0.415 | 0.872 | 1.000 | 1947 | tags=43%, list=16%, signal=51% |
| 1817 | GOMF\_DELAYED\_RECTIFIER\_POTASSIUM\_CHANNEL\_ACTIVITY |  | 14 | -0.39 | -1.04 | 0.399 | 0.872 | 1.000 | 13 | tags=7%, list=0%, signal=7% |
| 1818 | GOBP\_PROTEIN\_MATURATION\_BY\_PROTEIN\_FOLDING |  | 8 | -0.46 | -1.04 | 0.422 | 0.872 | 1.000 | 4483 | tags=63%, list=37%, signal=100% |
| 1819 | GOMF\_T\_CELL\_RECEPTOR\_BINDING |  | 4 | -0.56 | -1.04 | 0.443 | 0.872 | 1.000 | 4518 | tags=75%, list=38%, signal=120% |
| 1820 | GOBP\_REGULATION\_OF\_APOPTOTIC\_PROCESS\_INVOLVED\_IN\_DEVELOPMENT |  | 5 | -0.53 | -1.04 | 0.440 | 0.872 | 1.000 | 1918 | tags=40%, list=16%, signal=48% |
| 1821 | GOMF\_NUCLEOCYTOPLASMIC\_CARRIER\_ACTIVITY |  | 27 | -0.33 | -1.04 | 0.398 | 0.872 | 1.000 | 4390 | tags=44%, list=37%, signal=70% |
| 1822 | GOMF\_P53\_BINDING |  | 47 | -0.29 | -1.04 | 0.380 | 0.872 | 1.000 | 3813 | tags=43%, list=32%, signal=62% |
| 1823 | GOBP\_POSITIVE\_THYMIC\_T\_CELL\_SELECTION |  | 11 | -0.42 | -1.04 | 0.406 | 0.872 | 1.000 | 4191 | tags=73%, list=35%, signal=112% |
| 1824 | GOMF\_NUCLEOBASE\_CONTAINING\_COMPOUND\_TRANSMEMBRANE\_TRANSPORTER\_ACTIVITY |  | 32 | -0.32 | -1.04 | 0.383 | 0.872 | 1.000 | 2985 | tags=44%, list=25%, signal=58% |
| 1825 | GOCC\_RNA\_POLYMERASE\_III\_COMPLEX |  | 14 | -0.39 | -1.04 | 0.400 | 0.872 | 1.000 | 2927 | tags=36%, list=24%, signal=47% |
| 1826 | GOCC\_NUCLEAR\_UBIQUITIN\_LIGASE\_COMPLEX |  | 35 | -0.30 | -1.04 | 0.374 | 0.874 | 1.000 | 3504 | tags=43%, list=29%, signal=60% |
| 1827 | GOCC\_T\_CELL\_RECEPTOR\_COMPLEX |  | 14 | -0.39 | -1.04 | 0.412 | 0.874 | 1.000 | 2472 | tags=36%, list=21%, signal=45% |
| 1828 | GOBP\_AUTOCRINE\_SIGNALING |  | 3 | -0.62 | -1.04 | 0.463 | 0.875 | 1.000 | 351 | tags=33%, list=3%, signal=34% |
| 1829 | GOBP\_REGULATION\_OF\_SYNAPSE\_ASSEMBLY |  | 56 | -0.28 | -1.04 | 0.368 | 0.877 | 1.000 | 2052 | tags=25%, list=17%, signal=30% |
| 1830 | GOMF\_PROTEIN\_KINASE\_B\_BINDING |  | 7 | -0.48 | -1.04 | 0.422 | 0.877 | 1.000 | 3090 | tags=57%, list=26%, signal=77% |
| 1831 | GOBP\_REGULATION\_OF\_NERVOUS\_SYSTEM\_DEVELOPMENT |  | 290 | -0.21 | -1.04 | 0.323 | 0.878 | 1.000 | 2881 | tags=28%, list=24%, signal=35% |
| 1832 | GOBP\_KETONE\_BIOSYNTHETIC\_PROCESS |  | 29 | -0.32 | -1.04 | 0.393 | 0.877 | 1.000 | 2886 | tags=34%, list=24%, signal=45% |
| 1833 | GOBP\_SKELETAL\_MUSCLE\_CONTRACTION |  | 15 | -0.38 | -1.04 | 0.397 | 0.878 | 1.000 | 2612 | tags=40%, list=22%, signal=51% |
| 1834 | GOBP\_REGULATION\_OF\_B\_CELL\_DIFFERENTIATION |  | 20 | -0.35 | -1.04 | 0.403 | 0.878 | 1.000 | 2472 | tags=50%, list=21%, signal=63% |
| 1835 | GOBP\_SHORT\_CHAIN\_FATTY\_ACID\_CATABOLIC\_PROCESS |  | 8 | -0.46 | -1.04 | 0.416 | 0.879 | 1.000 | 1783 | tags=50%, list=15%, signal=59% |
| 1836 | GOBP\_N\_TERMINAL\_PROTEIN\_AMINO\_ACID\_MODIFICATION |  | 23 | -0.34 | -1.04 | 0.397 | 0.879 | 1.000 | 2090 | tags=30%, list=17%, signal=37% |
| 1837 | GOMF\_CHAPERONE\_BINDING |  | 74 | -0.26 | -1.04 | 0.366 | 0.879 | 1.000 | 2592 | tags=28%, list=22%, signal=36% |
| 1838 | GOMF\_CATALYTIC\_ACTIVITY\_ACTING\_ON\_RNA |  | 269 | -0.22 | -1.04 | 0.337 | 0.878 | 1.000 | 3082 | tags=32%, list=26%, signal=42% |
| 1839 | GOBP\_ANTIGEN\_PROCESSING\_AND\_PRESENTATION\_OF\_LIPID\_ANTIGEN\_VIA\_MHC\_CLASS\_IB |  | 4 | -0.56 | -1.04 | 0.454 | 0.878 | 1.000 | 2899 | tags=75%, list=24%, signal=99% |
| 1840 | GOBP\_DNA\_PROTECTION |  | 4 | -0.57 | -1.04 | 0.462 | 0.878 | 1.000 | 69 | tags=25%, list=1%, signal=25% |
| 1841 | GOBP\_GLIAL\_CELL\_PROLIFERATION |  | 33 | -0.31 | -1.04 | 0.394 | 0.878 | 1.000 | 979 | tags=18%, list=8%, signal=20% |
| 1842 | GOBP\_REGULATION\_OF\_SKELETAL\_MUSCLE\_ADAPTATION |  | 7 | -0.47 | -1.04 | 0.424 | 0.878 | 1.000 | 1003 | tags=29%, list=8%, signal=31% |
| 1843 | GOBP\_FIBROBLAST\_APOPTOTIC\_PROCESS |  | 17 | -0.36 | -1.04 | 0.407 | 0.878 | 1.000 | 2375 | tags=35%, list=20%, signal=44% |
| 1844 | GOBP\_CYTOPLASMIC\_TRANSLATIONAL\_ELONGATION |  | 10 | -0.43 | -1.04 | 0.418 | 0.877 | 1.000 | 3615 | tags=70%, list=30%, signal=100% |
| 1845 | GOBP\_POSITIVE\_REGULATION\_OF\_HEAT\_GENERATION |  | 9 | -0.44 | -1.04 | 0.416 | 0.877 | 1.000 | 979 | tags=33%, list=8%, signal=36% |
| 1846 | GOBP\_CELL\_MORPHOGENESIS\_INVOLVED\_IN\_NEURON\_DIFFERENTIATION |  | 411 | -0.21 | -1.04 | 0.314 | 0.878 | 1.000 | 2290 | tags=24%, list=19%, signal=28% |
| 1847 | GOCC\_SAGA\_TYPE\_COMPLEX |  | 23 | -0.34 | -1.04 | 0.391 | 0.878 | 1.000 | 2539 | tags=26%, list=21%, signal=33% |
| 1848 | GOBP\_NEGATIVE\_REGULATION\_OF\_MAP\_KINASE\_ACTIVITY |  | 53 | -0.28 | -1.04 | 0.385 | 0.878 | 1.000 | 1655 | tags=21%, list=14%, signal=24% |
| 1849 | GOMF\_NUCLEOSIDE\_DIPHOSPHATE\_KINASE\_ACTIVITY |  | 10 | -0.43 | -1.04 | 0.412 | 0.878 | 1.000 | 1626 | tags=50%, list=14%, signal=58% |
| 1850 | GOMF\_LONG\_CHAIN\_FATTY\_ACID\_BINDING |  | 7 | -0.47 | -1.04 | 0.415 | 0.878 | 1.000 | 980 | tags=43%, list=8%, signal=47% |
| 1851 | GOBP\_NEGATIVE\_REGULATION\_OF\_CARDIAC\_MUSCLE\_CONTRACTION |  | 4 | -0.57 | -1.04 | 0.466 | 0.878 | 1.000 | 5214 | tags=100%, list=43%, signal=177% |
| 1852 | GOMF\_SUPERCOILED\_DNA\_BINDING |  | 5 | -0.52 | -1.04 | 0.432 | 0.878 | 1.000 | 371 | tags=20%, list=3%, signal=21% |
| 1853 | GOBP\_TRACHEA\_MORPHOGENESIS |  | 7 | -0.47 | -1.04 | 0.418 | 0.877 | 1.000 | 3066 | tags=43%, list=26%, signal=58% |
| 1854 | GOBP\_INCLUSION\_BODY\_ASSEMBLY |  | 14 | -0.39 | -1.04 | 0.412 | 0.878 | 1.000 | 3317 | tags=36%, list=28%, signal=49% |
| 1855 | GOCC\_INTEGRATOR\_COMPLEX |  | 14 | -0.38 | -1.04 | 0.405 | 0.878 | 1.000 | 5173 | tags=50%, list=43%, signal=88% |
| 1856 | GOBP\_HISTONE\_H4\_K20\_TRIMETHYLATION |  | 5 | -0.53 | -1.03 | 0.443 | 0.878 | 1.000 | 725 | tags=20%, list=6%, signal=21% |
| 1857 | GOBP\_PEROXISOME\_FISSION |  | 7 | -0.48 | -1.03 | 0.448 | 0.879 | 1.000 | 1766 | tags=43%, list=15%, signal=50% |
| 1858 | GOBP\_ACTIVATION\_OF\_PHOSPHOLIPASE\_C\_ACTIVITY |  | 20 | -0.35 | -1.03 | 0.403 | 0.879 | 1.000 | 846 | tags=20%, list=7%, signal=21% |
| 1859 | GOBP\_SPINAL\_CORD\_DEVELOPMENT |  | 59 | -0.27 | -1.03 | 0.383 | 0.879 | 1.000 | 779 | tags=12%, list=6%, signal=13% |
| 1860 | GOBP\_REGULATION\_OF\_SYNAPTIC\_VESICLE\_CLUSTERING |  | 3 | -0.61 | -1.03 | 0.468 | 0.879 | 1.000 | 4645 | tags=100%, list=39%, signal=163% |
| 1861 | GOBP\_TRNA\_5\_LEADER\_REMOVAL |  | 8 | -0.45 | -1.03 | 0.427 | 0.879 | 1.000 | 5686 | tags=88%, list=47%, signal=166% |
| 1862 | GOBP\_REGULATION\_OF\_AUTOPHAGIC\_CELL\_DEATH |  | 5 | -0.53 | -1.03 | 0.455 | 0.880 | 1.000 | 1500 | tags=40%, list=12%, signal=46% |
| 1863 | GOBP\_MITOCHONDRIAL\_GENOME\_MAINTENANCE |  | 18 | -0.36 | -1.03 | 0.403 | 0.879 | 1.000 | 1246 | tags=22%, list=10%, signal=25% |
| 1864 | GOCC\_L\_TYPE\_VOLTAGE\_GATED\_CALCIUM\_CHANNEL\_COMPLEX |  | 6 | -0.50 | -1.03 | 0.445 | 0.879 | 1.000 | 41 | tags=17%, list=0%, signal=17% |
| 1865 | GOBP\_RESPONSE\_TO\_TOXIC\_SUBSTANCE |  | 140 | -0.23 | -1.03 | 0.365 | 0.878 | 1.000 | 1403 | tags=19%, list=12%, signal=21% |
| 1866 | GOBP\_POLYOL\_METABOLIC\_PROCESS |  | 99 | -0.25 | -1.03 | 0.383 | 0.878 | 1.000 | 1154 | tags=18%, list=10%, signal=20% |
| 1867 | GOBP\_STRIATED\_MUSCLE\_CELL\_DEVELOPMENT |  | 57 | -0.27 | -1.03 | 0.391 | 0.879 | 1.000 | 2170 | tags=23%, list=18%, signal=28% |
| 1868 | GOBP\_NEGATIVE\_REGULATION\_OF\_EPIDERMAL\_GROWTH\_FACTOR\_ACTIVATED\_RECEPTOR\_ACTIVITY |  | 10 | -0.42 | -1.03 | 0.428 | 0.879 | 1.000 | 4563 | tags=50%, list=38%, signal=81% |
| 1869 | GOBP\_PTERIDINE\_CONTAINING\_COMPOUND\_METABOLIC\_PROCESS |  | 27 | -0.32 | -1.03 | 0.402 | 0.878 | 1.000 | 532 | tags=15%, list=4%, signal=15% |
| 1870 | GOCC\_RIBONUCLEASE\_MRP\_COMPLEX |  | 5 | -0.53 | -1.03 | 0.446 | 0.878 | 1.000 | 3935 | tags=60%, list=33%, signal=89% |
| 1871 | GOBP\_CHONDROITIN\_SULFATE\_PROTEOGLYCAN\_BIOSYNTHETIC\_PROCESS |  | 22 | -0.34 | -1.03 | 0.409 | 0.880 | 1.000 | 1824 | tags=23%, list=15%, signal=27% |
| 1872 | GOBP\_POSITIVE\_REGULATION\_OF\_PROTEIN\_SERINE\_THREONINE\_KINASE\_ACTIVITY |  | 228 | -0.22 | -1.03 | 0.365 | 0.879 | 1.000 | 3022 | tags=27%, list=25%, signal=36% |
| 1873 | GOBP\_MUCOPOLYSACCHARIDE\_METABOLIC\_PROCESS |  | 87 | -0.25 | -1.03 | 0.376 | 0.879 | 1.000 | 2087 | tags=23%, list=17%, signal=28% |
| 1874 | GOBP\_POLYAMINE\_METABOLIC\_PROCESS |  | 13 | -0.39 | -1.03 | 0.406 | 0.880 | 1.000 | 584 | tags=23%, list=5%, signal=24% |
| 1875 | GOBP\_REGULATION\_OF\_PROTEIN\_K63\_LINKED\_UBIQUITINATION |  | 7 | -0.47 | -1.03 | 0.436 | 0.882 | 1.000 | 2718 | tags=29%, list=23%, signal=37% |
| 1876 | GOBP\_NEGATIVE\_REGULATION\_OF\_CHEMOTAXIS |  | 40 | -0.29 | -1.03 | 0.393 | 0.882 | 1.000 | 1489 | tags=25%, list=12%, signal=28% |
| 1877 | GOBP\_MORPHOGENESIS\_OF\_A\_BRANCHING\_STRUCTURE |  | 132 | -0.24 | -1.03 | 0.387 | 0.882 | 1.000 | 1656 | tags=17%, list=14%, signal=20% |
| 1878 | GOMF\_ARYLESTERASE\_ACTIVITY |  | 3 | -0.61 | -1.03 | 0.471 | 0.882 | 1.000 | 1061 | tags=33%, list=9%, signal=37% |
| 1879 | GOBP\_PROTEIN\_TARGETING\_TO\_VACUOLE |  | 36 | -0.30 | -1.03 | 0.403 | 0.881 | 1.000 | 3319 | tags=39%, list=28%, signal=54% |
| 1880 | GOBP\_REGULATION\_OF\_CILIUM\_BEAT\_FREQUENCY\_INVOLVED\_IN\_CILIARY\_MOTILITY |  | 5 | -0.52 | -1.03 | 0.451 | 0.882 | 1.000 | 2586 | tags=40%, list=22%, signal=51% |
| 1881 | GOBP\_RESPONSE\_TO\_INTERLEUKIN\_4 |  | 23 | -0.33 | -1.03 | 0.411 | 0.882 | 1.000 | 2569 | tags=43%, list=21%, signal=55% |
| 1882 | GOBP\_GUANOSINE\_CONTAINING\_COMPOUND\_METABOLIC\_PROCESS |  | 31 | -0.31 | -1.03 | 0.405 | 0.882 | 1.000 | 1626 | tags=29%, list=14%, signal=33% |
| 1883 | GOBP\_REGULATION\_OF\_MEMBRANE\_REPOLARIZATION |  | 21 | -0.34 | -1.03 | 0.420 | 0.883 | 1.000 | 728 | tags=14%, list=6%, signal=15% |
| 1884 | GOBP\_FIBROBLAST\_ACTIVATION |  | 8 | -0.45 | -1.03 | 0.428 | 0.882 | 1.000 | 1560 | tags=25%, list=13%, signal=29% |
| 1885 | GOBP\_SOMITE\_DEVELOPMENT |  | 54 | -0.28 | -1.03 | 0.406 | 0.882 | 1.000 | 3624 | tags=44%, list=30%, signal=63% |
| 1886 | GOCC\_NEURON\_TO\_NEURON\_SYNAPSE |  | 223 | -0.22 | -1.03 | 0.362 | 0.882 | 1.000 | 1655 | tags=19%, list=14%, signal=22% |
| 1887 | GOBP\_DETECTION\_OF\_OTHER\_ORGANISM |  | 4 | -0.56 | -1.03 | 0.466 | 0.882 | 1.000 | 1142 | tags=25%, list=10%, signal=28% |
| 1888 | GOBP\_FLAVIN\_CONTAINING\_COMPOUND\_METABOLIC\_PROCESS |  | 5 | -0.52 | -1.03 | 0.456 | 0.882 | 1.000 | 3534 | tags=60%, list=29%, signal=85% |
| 1889 | GOBP\_POSITIVE\_REGULATION\_OF\_DIGESTIVE\_SYSTEM\_PROCESS |  | 6 | -0.49 | -1.03 | 0.447 | 0.882 | 1.000 | 12 | tags=17%, list=0%, signal=17% |
| 1890 | GOBP\_LUNG\_VASCULATURE\_DEVELOPMENT |  | 3 | -0.61 | -1.03 | 0.494 | 0.882 | 1.000 | 4734 | tags=100%, list=39%, signal=165% |
| 1891 | GOCC\_PERICENTRIOLAR\_MATERIAL |  | 15 | -0.38 | -1.03 | 0.413 | 0.883 | 1.000 | 810 | tags=13%, list=7%, signal=14% |
| 1892 | GOBP\_CELLULAR\_ALDEHYDE\_METABOLIC\_PROCESS |  | 47 | -0.29 | -1.03 | 0.400 | 0.883 | 1.000 | 3301 | tags=43%, list=27%, signal=58% |
| 1893 | GOBP\_CIRCADIAN\_REGULATION\_OF\_GENE\_EXPRESSION |  | 52 | -0.28 | -1.03 | 0.410 | 0.882 | 1.000 | 2421 | tags=31%, list=20%, signal=38% |
| 1894 | GOBP\_GERMINAL\_CENTER\_FORMATION |  | 11 | -0.41 | -1.03 | 0.432 | 0.882 | 1.000 | 3811 | tags=64%, list=32%, signal=93% |
| 1895 | GOBP\_COMPLEMENT\_ACTIVATION\_ALTERNATIVE\_PATHWAY |  | 8 | -0.45 | -1.03 | 0.435 | 0.882 | 1.000 | 3035 | tags=50%, list=25%, signal=67% |
| 1896 | GOBP\_ISOTYPE\_SWITCHING\_TO\_IGG\_ISOTYPES |  | 10 | -0.42 | -1.03 | 0.429 | 0.882 | 1.000 | 3133 | tags=60%, list=26%, signal=81% |
| 1897 | GOBP\_DNA\_DEAMINATION |  | 5 | -0.52 | -1.03 | 0.454 | 0.882 | 1.000 | 3711 | tags=80%, list=31%, signal=116% |
| 1898 | GOBP\_NEGATIVE\_REGULATION\_OF\_COMPLEMENT\_ACTIVATION |  | 5 | -0.52 | -1.03 | 0.459 | 0.883 | 1.000 | 2812 | tags=60%, list=23%, signal=78% |
| 1899 | GOBP\_REGULATION\_OF\_GROWTH\_HORMONE\_RECEPTOR\_SIGNALING\_PATHWAY |  | 4 | -0.55 | -1.03 | 0.473 | 0.883 | 1.000 | 199 | tags=25%, list=2%, signal=25% |
| 1900 | GOBP\_HYDROCARBON\_METABOLIC\_PROCESS |  | 4 | -0.56 | -1.03 | 0.469 | 0.883 | 1.000 | 44 | tags=25%, list=0%, signal=25% |
| 1901 | GOMF\_SODIUM\_CHLORIDE\_SYMPORTER\_ACTIVITY |  | 7 | -0.47 | -1.03 | 0.441 | 0.883 | 1.000 | 1096 | tags=43%, list=9%, signal=47% |
| 1902 | GOBP\_CHONDROITIN\_SULFATE\_BIOSYNTHETIC\_PROCESS |  | 20 | -0.35 | -1.03 | 0.417 | 0.884 | 1.000 | 1824 | tags=20%, list=15%, signal=24% |
| 1903 | GOBP\_TRNA\_WOBBLE\_BASE\_MODIFICATION |  | 15 | -0.37 | -1.03 | 0.420 | 0.884 | 1.000 | 1972 | tags=40%, list=16%, signal=48% |
| 1904 | GOBP\_POSITIVE\_REGULATION\_OF\_ERAD\_PATHWAY |  | 9 | -0.44 | -1.03 | 0.429 | 0.884 | 1.000 | 1747 | tags=33%, list=15%, signal=39% |
| 1905 | GOBP\_INTESTINAL\_EPITHELIAL\_CELL\_DIFFERENTIATION |  | 18 | -0.35 | -1.03 | 0.420 | 0.883 | 1.000 | 1418 | tags=28%, list=12%, signal=31% |
| 1906 | GOBP\_POSITIVE\_REGULATION\_OF\_TRANSCRIPTION\_OF\_NUCLEOLAR\_LARGE\_RRNA\_BY\_RNA\_POLYMERASE\_I |  | 8 | -0.45 | -1.03 | 0.435 | 0.883 | 1.000 | 2541 | tags=50%, list=21%, signal=63% |
| 1907 | GOCC\_3M\_COMPLEX |  | 3 | -0.61 | -1.03 | 0.487 | 0.883 | 1.000 | 626 | tags=33%, list=5%, signal=35% |
| 1908 | GOBP\_RENAL\_WATER\_HOMEOSTASIS |  | 24 | -0.33 | -1.03 | 0.425 | 0.883 | 1.000 | 44 | tags=8%, list=0%, signal=8% |
| 1909 | GOCC\_EUKARYOTIC\_TRANSLATION\_INITIATION\_FACTOR\_2B\_COMPLEX |  | 6 | -0.49 | -1.02 | 0.455 | 0.882 | 1.000 | 3226 | tags=50%, list=27%, signal=68% |
| 1910 | GOBP\_MACROPHAGE\_DIFFERENTIATION |  | 35 | -0.30 | -1.02 | 0.418 | 0.882 | 1.000 | 2625 | tags=40%, list=22%, signal=51% |
| 1911 | GOBP\_AXIS\_ELONGATION |  | 21 | -0.34 | -1.02 | 0.417 | 0.882 | 1.000 | 1656 | tags=24%, list=14%, signal=28% |
| 1912 | GOBP\_SKELETAL\_SYSTEM\_DEVELOPMENT |  | 315 | -0.21 | -1.02 | 0.370 | 0.882 | 1.000 | 2472 | tags=25%, list=21%, signal=30% |
| 1913 | GOBP\_MYELOID\_LEUKOCYTE\_MEDIATED\_IMMUNITY |  | 357 | -0.21 | -1.02 | 0.359 | 0.882 | 1.000 | 2766 | tags=27%, list=23%, signal=34% |
| 1914 | GOMF\_HISTONE\_DEACETYLASE\_ACTIVITY\_H3\_K14\_SPECIFIC |  | 8 | -0.45 | -1.02 | 0.442 | 0.885 | 1.000 | 2317 | tags=50%, list=19%, signal=62% |
| 1915 | GOBP\_NEGATIVE\_REGULATION\_OF\_UBIQUITIN\_PROTEIN\_TRANSFERASE\_ACTIVITY |  | 16 | -0.36 | -1.02 | 0.420 | 0.884 | 1.000 | 2656 | tags=44%, list=22%, signal=56% |
| 1916 | GOBP\_EYE\_PIGMENTATION |  | 3 | -0.60 | -1.02 | 0.487 | 0.884 | 1.000 | 3391 | tags=67%, list=28%, signal=93% |
| 1917 | GOCC\_ENVELOPE |  | 885 | -0.19 | -1.02 | 0.329 | 0.884 | 1.000 | 3005 | tags=27%, list=25%, signal=34% |
| 1918 | GOBP\_ACUTE\_INFLAMMATORY\_RESPONSE\_TO\_ANTIGENIC\_STIMULUS |  | 11 | -0.41 | -1.02 | 0.428 | 0.886 | 1.000 | 3035 | tags=55%, list=25%, signal=73% |
| 1919 | GOCC\_PEPTIDASE\_COMPLEX |  | 73 | -0.26 | -1.02 | 0.410 | 0.886 | 1.000 | 4014 | tags=32%, list=33%, signal=47% |
| 1920 | GOCC\_PHOTORECEPTOR\_OUTER\_SEGMENT\_MEMBRANE |  | 6 | -0.49 | -1.02 | 0.444 | 0.885 | 1.000 | 143 | tags=17%, list=1%, signal=17% |
| 1921 | GOMF\_CARBOXYLIC\_ACID\_BINDING |  | 101 | -0.24 | -1.02 | 0.403 | 0.885 | 1.000 | 1531 | tags=23%, list=13%, signal=26% |
| 1922 | GOMF\_MITOGEN\_ACTIVATED\_PROTEIN\_KINASE\_KINASE\_KINASE\_BINDING |  | 15 | -0.37 | -1.02 | 0.406 | 0.885 | 1.000 | 2483 | tags=27%, list=21%, signal=34% |
| 1923 | GOBP\_REGULATION\_OF\_ENDOTHELIAL\_CELL\_DEVELOPMENT |  | 15 | -0.38 | -1.02 | 0.419 | 0.886 | 1.000 | 1338 | tags=27%, list=11%, signal=30% |
| 1924 | GOCC\_EPSILON\_DNA\_POLYMERASE\_COMPLEX |  | 5 | -0.51 | -1.02 | 0.456 | 0.886 | 1.000 | 313 | tags=20%, list=3%, signal=21% |
| 1925 | GOMF\_SIGNAL\_SEQUENCE\_BINDING |  | 37 | -0.30 | -1.02 | 0.395 | 0.889 | 1.000 | 3122 | tags=32%, list=26%, signal=44% |
| 1926 | GOBP\_RESPONSE\_TO\_CORTICOTROPIN\_RELEASING\_HORMONE |  | 4 | -0.56 | -1.02 | 0.469 | 0.888 | 1.000 | 2200 | tags=50%, list=18%, signal=61% |
| 1927 | GOCC\_TOR\_COMPLEX |  | 14 | -0.38 | -1.02 | 0.430 | 0.889 | 1.000 | 1344 | tags=21%, list=11%, signal=24% |
| 1928 | GOMF\_AMINO\_ACID\_TRANSMEMBRANE\_TRANSPORTER\_ACTIVITY |  | 50 | -0.28 | -1.02 | 0.412 | 0.889 | 1.000 | 1561 | tags=26%, list=13%, signal=30% |
| 1929 | GOMF\_POTASSIUM\_ION\_LEAK\_CHANNEL\_ACTIVITY |  | 8 | -0.45 | -1.02 | 0.437 | 0.889 | 1.000 | 1578 | tags=50%, list=13%, signal=58% |
| 1930 | GOBP\_NEGATIVE\_REGULATION\_OF\_RESPONSE\_TO\_REACTIVE\_OXYGEN\_SPECIES |  | 12 | -0.40 | -1.02 | 0.435 | 0.890 | 1.000 | 1290 | tags=33%, list=11%, signal=37% |
| 1931 | GOMF\_LIGAND\_GATED\_CALCIUM\_CHANNEL\_ACTIVITY |  | 18 | -0.35 | -1.02 | 0.419 | 0.890 | 1.000 | 679 | tags=17%, list=6%, signal=18% |
| 1932 | GOBP\_REGULATION\_OF\_IMMUNOGLOBULIN\_PRODUCTION |  | 36 | -0.30 | -1.02 | 0.426 | 0.890 | 1.000 | 3209 | tags=44%, list=27%, signal=60% |
| 1933 | GOBP\_RIBOSOMAL\_LARGE\_SUBUNIT\_EXPORT\_FROM\_NUCLEUS |  | 7 | -0.47 | -1.02 | 0.443 | 0.890 | 1.000 | 2280 | tags=29%, list=19%, signal=35% |
| 1934 | GOBP\_DNA\_DEMETHYLATION |  | 14 | -0.38 | -1.02 | 0.432 | 0.891 | 1.000 | 2022 | tags=50%, list=17%, signal=60% |
| 1935 | GOCC\_AP\_TYPE\_MEMBRANE\_COAT\_ADAPTOR\_COMPLEX |  | 29 | -0.31 | -1.02 | 0.437 | 0.891 | 1.000 | 2899 | tags=34%, list=24%, signal=45% |
| 1936 | GOBP\_DEVELOPMENTAL\_CELL\_GROWTH |  | 151 | -0.23 | -1.02 | 0.398 | 0.890 | 1.000 | 1133 | tags=13%, list=9%, signal=14% |
| 1937 | GOBP\_REGULATION\_OF\_TRANSLATIONAL\_INITIATION |  | 63 | -0.26 | -1.02 | 0.415 | 0.890 | 1.000 | 2704 | tags=33%, list=23%, signal=43% |
| 1938 | GOBP\_SEGMENTATION |  | 59 | -0.27 | -1.02 | 0.411 | 0.890 | 1.000 | 1641 | tags=25%, list=14%, signal=29% |
| 1939 | GOBP\_POSITIVE\_REGULATION\_OF\_MEIOTIC\_CELL\_CYCLE\_PHASE\_TRANSITION |  | 3 | -0.60 | -1.02 | 0.501 | 0.890 | 1.000 | 3257 | tags=67%, list=27%, signal=91% |
| 1940 | GOMF\_ISOPRENOID\_BINDING |  | 18 | -0.35 | -1.02 | 0.436 | 0.890 | 1.000 | 1467 | tags=33%, list=12%, signal=38% |
| 1941 | GOBP\_PURINE\_NUCLEOSIDE\_METABOLIC\_PROCESS |  | 47 | -0.28 | -1.02 | 0.409 | 0.891 | 1.000 | 1309 | tags=23%, list=11%, signal=26% |
| 1942 | GOBP\_NEUROTRANSMITTER\_RECEPTOR\_TRANSPORT\_ENDOSOME\_TO\_PLASMA\_MEMBRANE |  | 8 | -0.45 | -1.02 | 0.443 | 0.891 | 1.000 | 194 | tags=13%, list=2%, signal=13% |
| 1943 | GOBP\_CELL\_MIGRATION\_INVOLVED\_IN\_KIDNEY\_DEVELOPMENT |  | 5 | -0.51 | -1.02 | 0.462 | 0.891 | 1.000 | 823 | tags=20%, list=7%, signal=21% |
| 1944 | GOMF\_NUCLEOTIDE\_TRANSMEMBRANE\_TRANSPORTER\_ACTIVITY |  | 18 | -0.36 | -1.02 | 0.421 | 0.890 | 1.000 | 2911 | tags=44%, list=24%, signal=59% |
| 1945 | GOBP\_NEGATIVE\_REGULATION\_OF\_DNA\_TEMPLATED\_TRANSCRIPTION\_INITIATION |  | 6 | -0.49 | -1.02 | 0.456 | 0.891 | 1.000 | 3844 | tags=67%, list=32%, signal=98% |
| 1946 | GOBP\_REGULATION\_OF\_MORPHOGENESIS\_OF\_AN\_EPITHELIUM |  | 38 | -0.29 | -1.02 | 0.416 | 0.891 | 1.000 | 2089 | tags=29%, list=17%, signal=35% |
| 1947 | GOMF\_TRANSFERASE\_ACTIVITY\_TRANSFERRING\_ONE\_CARBON\_GROUPS |  | 158 | -0.23 | -1.02 | 0.416 | 0.893 | 1.000 | 2653 | tags=26%, list=22%, signal=33% |
| 1948 | GOCC\_PROTON\_TRANSPORTING\_TWO\_SECTOR\_ATPASE\_COMPLEX |  | 24 | -0.33 | -1.02 | 0.438 | 0.893 | 1.000 | 3769 | tags=46%, list=31%, signal=67% |
| 1949 | GOBP\_DETERMINATION\_OF\_LEFT\_RIGHT\_ASYMMETRY\_IN\_LATERAL\_MESODERM |  | 3 | -0.60 | -1.02 | 0.495 | 0.893 | 1.000 | 4793 | tags=100%, list=40%, signal=166% |
| 1950 | GOBP\_RESPONSE\_TO\_OXIDATIVE\_STRESS |  | 312 | -0.21 | -1.02 | 0.380 | 0.893 | 1.000 | 1498 | tags=16%, list=12%, signal=18% |
| 1951 | GOMF\_WNT\_ACTIVATED\_RECEPTOR\_ACTIVITY |  | 13 | -0.39 | -1.01 | 0.441 | 0.893 | 1.000 | 5027 | tags=62%, list=42%, signal=106% |
| 1952 | GOMF\_AMINOACYL\_TRNA\_EDITING\_ACTIVITY |  | 8 | -0.44 | -1.01 | 0.450 | 0.893 | 1.000 | 3334 | tags=63%, list=28%, signal=86% |
| 1953 | GOCC\_TRANSCRIPTION\_ELONGATION\_FACTOR\_COMPLEX |  | 44 | -0.28 | -1.01 | 0.422 | 0.894 | 1.000 | 3741 | tags=36%, list=31%, signal=53% |
| 1954 | GOBP\_POSITIVE\_REGULATION\_OF\_INTERLEUKIN\_13\_PRODUCTION |  | 5 | -0.51 | -1.01 | 0.468 | 0.895 | 1.000 | 2583 | tags=40%, list=22%, signal=51% |
| 1955 | GOBP\_RESPONSE\_TO\_ISCHEMIA |  | 39 | -0.29 | -1.01 | 0.422 | 0.896 | 1.000 | 1604 | tags=28%, list=13%, signal=32% |
| 1956 | GOBP\_SENSORY\_PERCEPTION\_OF\_PAIN |  | 53 | -0.27 | -1.01 | 0.429 | 0.896 | 1.000 | 1482 | tags=23%, list=12%, signal=26% |
| 1957 | GOBP\_NEGATIVE\_REGULATION\_OF\_G\_PROTEIN\_COUPLED\_RECEPTOR\_SIGNALING\_PATHWAY |  | 31 | -0.31 | -1.01 | 0.421 | 0.897 | 1.000 | 947 | tags=23%, list=8%, signal=24% |
| 1958 | GOBP\_NEGATIVE\_REGULATION\_OF\_INTERLEUKIN\_4\_PRODUCTION |  | 5 | -0.51 | -1.01 | 0.471 | 0.897 | 1.000 | 1519 | tags=60%, list=13%, signal=69% |
| 1959 | GOMF\_S\_ADENOSYLMETHIONINE\_DEPENDENT\_METHYLTRANSFERASE\_ACTIVITY |  | 114 | -0.24 | -1.01 | 0.418 | 0.897 | 1.000 | 2919 | tags=29%, list=24%, signal=38% |
| 1960 | GOBP\_POSITIVE\_REGULATION\_OF\_ACUTE\_INFLAMMATORY\_RESPONSE\_TO\_ANTIGENIC\_STIMULUS |  | 5 | -0.52 | -1.01 | 0.463 | 0.897 | 1.000 | 3035 | tags=80%, list=25%, signal=107% |
| 1961 | GOBP\_REGULATION\_OF\_CATECHOLAMINE\_METABOLIC\_PROCESS |  | 8 | -0.45 | -1.01 | 0.439 | 0.897 | 1.000 | 2505 | tags=50%, list=21%, signal=63% |
| 1962 | GOBP\_REGULATION\_OF\_TRANSCRIPTION\_ELONGATION\_FROM\_RNA\_POLYMERASE\_II\_PROMOTER |  | 26 | -0.32 | -1.01 | 0.444 | 0.897 | 1.000 | 3643 | tags=31%, list=30%, signal=44% |
| 1963 | GOBP\_NEGATIVE\_REGULATION\_OF\_CELL\_PROJECTION\_ORGANIZATION |  | 124 | -0.23 | -1.01 | 0.420 | 0.897 | 1.000 | 1370 | tags=15%, list=11%, signal=17% |
| 1964 | GOBP\_SOMITOGENESIS |  | 42 | -0.29 | -1.01 | 0.430 | 0.897 | 1.000 | 3624 | tags=50%, list=30%, signal=71% |
| 1965 | GOBP\_POSITIVE\_REGULATION\_OF\_JUN\_KINASE\_ACTIVITY |  | 45 | -0.28 | -1.01 | 0.433 | 0.897 | 1.000 | 1038 | tags=16%, list=9%, signal=17% |
| 1966 | GOBP\_LEUKOCYTE\_MEDIATED\_IMMUNITY |  | 462 | -0.20 | -1.01 | 0.416 | 0.897 | 1.000 | 2940 | tags=30%, list=24%, signal=38% |
| 1967 | GOBP\_NEGATIVE\_REGULATION\_OF\_PROTEIN\_TARGETING\_TO\_MITOCHONDRION |  | 3 | -0.59 | -1.01 | 0.502 | 0.897 | 1.000 | 3649 | tags=67%, list=30%, signal=96% |
| 1968 | GOBP\_REGULATION\_OF\_UBIQUITIN\_PROTEIN\_LIGASE\_ACTIVITY |  | 20 | -0.34 | -1.01 | 0.431 | 0.897 | 1.000 | 1031 | tags=20%, list=9%, signal=22% |
| 1969 | GOBP\_POSITIVE\_REGULATION\_OF\_ENDOPLASMIC\_RETICULUM\_STRESS\_INDUCED\_INTRINSIC\_APOPTOTIC\_SIGNALING\_PATHWAY |  | 8 | -0.45 | -1.01 | 0.450 | 0.898 | 1.000 | 2144 | tags=38%, list=18%, signal=46% |
| 1970 | GOBP\_ANIMAL\_ORGAN\_MORPHOGENESIS |  | 658 | -0.19 | -1.01 | 0.399 | 0.897 | 1.000 | 2052 | tags=19%, list=17%, signal=22% |
| 1971 | GOBP\_CELLULAR\_COPPER\_ION\_HOMEOSTASIS |  | 9 | -0.43 | -1.01 | 0.452 | 0.898 | 1.000 | 1326 | tags=22%, list=11%, signal=25% |
| 1972 | GOBP\_POSITIVE\_REGULATION\_OF\_BROWN\_FAT\_CELL\_DIFFERENTIATION |  | 11 | -0.40 | -1.01 | 0.450 | 0.898 | 1.000 | 408 | tags=18%, list=3%, signal=19% |
| 1973 | GOBP\_PROTEIN\_DENEDDYLATION |  | 9 | -0.43 | -1.01 | 0.456 | 0.899 | 1.000 | 4634 | tags=78%, list=39%, signal=127% |
| 1974 | GOBP\_COCHLEA\_DEVELOPMENT |  | 28 | -0.31 | -1.01 | 0.439 | 0.899 | 1.000 | 836 | tags=14%, list=7%, signal=15% |
| 1975 | GOCC\_ANCHORED\_COMPONENT\_OF\_PRESYNAPTIC\_MEMBRANE |  | 5 | -0.51 | -1.01 | 0.481 | 0.899 | 1.000 | 2158 | tags=40%, list=18%, signal=49% |
| 1976 | GOCC\_NUCLEAR\_ENVELOPE\_LUMEN |  | 8 | -0.44 | -1.01 | 0.446 | 0.899 | 1.000 | 3531 | tags=50%, list=29%, signal=71% |
| 1977 | GOBP\_CELL\_PART\_MORPHOGENESIS |  | 484 | -0.20 | -1.01 | 0.409 | 0.898 | 1.000 | 2311 | tags=23%, list=19%, signal=27% |
| 1978 | GOMF\_N6\_METHYLADENOSINE\_CONTAINING\_RNA\_BINDING |  | 6 | -0.48 | -1.01 | 0.463 | 0.899 | 1.000 | 1576 | tags=33%, list=13%, signal=38% |
| 1979 | GOBP\_GROWTH\_INVOLVED\_IN\_HEART\_MORPHOGENESIS |  | 3 | -0.59 | -1.01 | 0.506 | 0.899 | 1.000 | 4908 | tags=100%, list=41%, signal=169% |
| 1980 | GOMF\_NADPH\_BINDING |  | 14 | -0.38 | -1.01 | 0.436 | 0.899 | 1.000 | 1224 | tags=29%, list=10%, signal=32% |
| 1981 | GOBP\_POSITIVE\_REGULATION\_OF\_CALCIUM\_MEDIATED\_SIGNALING |  | 21 | -0.34 | -1.01 | 0.445 | 0.900 | 1.000 | 2255 | tags=33%, list=19%, signal=41% |
| 1982 | GOBP\_NEGATIVE\_REGULATION\_OF\_NEURON\_PROJECTION\_REGENERATION |  | 11 | -0.40 | -1.01 | 0.443 | 0.900 | 1.000 | 82 | tags=9%, list=1%, signal=9% |
| 1983 | GOBP\_STRIATED\_MUSCLE\_CELL\_DIFFERENTIATION |  | 154 | -0.22 | -1.01 | 0.439 | 0.900 | 1.000 | 2063 | tags=23%, list=17%, signal=28% |
| 1984 | GOBP\_SPHINGOID\_METABOLIC\_PROCESS |  | 16 | -0.36 | -1.01 | 0.445 | 0.900 | 1.000 | 762 | tags=19%, list=6%, signal=20% |
| 1985 | GOBP\_SECRETION\_OF\_LYSOSOMAL\_ENZYMES |  | 4 | -0.55 | -1.01 | 0.496 | 0.901 | 1.000 | 2396 | tags=50%, list=20%, signal=62% |
| 1986 | GOBP\_AMINOACYL\_TRNA\_METABOLISM\_INVOLVED\_IN\_TRANSLATIONAL\_FIDELITY |  | 8 | -0.44 | -1.01 | 0.463 | 0.901 | 1.000 | 3334 | tags=63%, list=28%, signal=86% |
| 1987 | GOBP\_LIPID\_EXPORT\_FROM\_CELL |  | 57 | -0.27 | -1.01 | 0.441 | 0.903 | 1.000 | 1589 | tags=21%, list=13%, signal=24% |
| 1988 | GOBP\_REGULATION\_OF\_ESTABLISHMENT\_OR\_MAINTENANCE\_OF\_CELL\_POLARITY |  | 22 | -0.33 | -1.01 | 0.443 | 0.903 | 1.000 | 458 | tags=14%, list=4%, signal=14% |
| 1989 | GOBP\_RESPONSE\_TO\_ISOQUINOLINE\_ALKALOID |  | 19 | -0.34 | -1.01 | 0.438 | 0.903 | 1.000 | 970 | tags=16%, list=8%, signal=17% |
| 1990 | GOBP\_LIPID\_MODIFICATION |  | 201 | -0.22 | -1.01 | 0.443 | 0.903 | 1.000 | 1486 | tags=18%, list=12%, signal=21% |
| 1991 | GOMF\_CYTOKINE\_BINDING |  | 91 | -0.24 | -1.01 | 0.436 | 0.903 | 1.000 | 1133 | tags=19%, list=9%, signal=20% |
| 1992 | GOBP\_PANCREATIC\_A\_CELL\_DIFFERENTIATION |  | 7 | -0.46 | -1.00 | 0.472 | 0.903 | 1.000 | 514 | tags=29%, list=4%, signal=30% |
| 1993 | GOBP\_NEGATIVE\_REGULATION\_OF\_MUSCLE\_CONTRACTION |  | 16 | -0.36 | -1.00 | 0.445 | 0.903 | 1.000 | 3527 | tags=44%, list=29%, signal=62% |
| 1994 | GOBP\_EXPORT\_ACROSS\_PLASMA\_MEMBRANE |  | 35 | -0.30 | -1.00 | 0.437 | 0.904 | 1.000 | 1986 | tags=29%, list=17%, signal=34% |
| 1995 | GOBP\_REGULATION\_OF\_MAST\_CELL\_ACTIVATION\_INVOLVED\_IN\_IMMUNE\_RESPONSE |  | 19 | -0.34 | -1.00 | 0.450 | 0.904 | 1.000 | 2758 | tags=42%, list=23%, signal=55% |
| 1996 | GOBP\_RIBONUCLEOSIDE\_METABOLIC\_PROCESS |  | 51 | -0.27 | -1.00 | 0.434 | 0.904 | 1.000 | 1309 | tags=25%, list=11%, signal=28% |
| 1997 | GOBP\_MODULATION\_BY\_SYMBIONT\_OF\_HOST\_CELLULAR\_PROCESS |  | 19 | -0.35 | -1.00 | 0.435 | 0.903 | 1.000 | 1500 | tags=21%, list=12%, signal=24% |
| 1998 | GOBP\_NEUROTRANSMITTER\_LOADING\_INTO\_SYNAPTIC\_VESICLE |  | 3 | -0.59 | -1.00 | 0.514 | 0.903 | 1.000 | 1096 | tags=67%, list=9%, signal=73% |
| 1999 | GOBP\_REGULATION\_OF\_ICOSANOID\_SECRETION |  | 12 | -0.39 | -1.00 | 0.460 | 0.903 | 1.000 | 1481 | tags=33%, list=12%, signal=38% |
| 2000 | GOBP\_REGULATION\_OF\_CALCIUM\_ION\_DEPENDENT\_EXOCYTOSIS |  | 26 | -0.32 | -1.00 | 0.443 | 0.904 | 1.000 | 3691 | tags=50%, list=31%, signal=72% |
| 2001 | GOBP\_POSITIVE\_REGULATION\_OF\_CELL\_ACTIVATION |  | 214 | -0.21 | -1.00 | 0.442 | 0.904 | 1.000 | 2777 | tags=32%, list=23%, signal=41% |
| 2002 | GOCC\_U12\_TYPE\_SPLICEOSOMAL\_COMPLEX |  | 24 | -0.32 | -1.00 | 0.438 | 0.905 | 1.000 | 2465 | tags=29%, list=21%, signal=37% |
| 2003 | GOBP\_REGULATION\_OF\_MITOCHONDRIAL\_ATP\_SYNTHESIS\_COUPLED\_ELECTRON\_TRANSPORT |  | 5 | -0.51 | -1.00 | 0.479 | 0.905 | 1.000 | 3284 | tags=60%, list=27%, signal=83% |
| 2004 | GOBP\_POSITIVE\_REGULATION\_OF\_HISTONE\_H4\_ACETYLATION |  | 4 | -0.54 | -1.00 | 0.492 | 0.904 | 1.000 | 2541 | tags=75%, list=21%, signal=95% |
| 2005 | GOMF\_SULFURTRANSFERASE\_ACTIVITY |  | 10 | -0.41 | -1.00 | 0.455 | 0.904 | 1.000 | 779 | tags=20%, list=6%, signal=21% |
| 2006 | GOBP\_REGULATION\_OF\_MEMBRANE\_DEPOLARIZATION |  | 30 | -0.31 | -1.00 | 0.444 | 0.904 | 1.000 | 1403 | tags=27%, list=12%, signal=30% |
| 2007 | GOBP\_MEMBRANE\_REPOLARIZATION |  | 28 | -0.31 | -1.00 | 0.453 | 0.904 | 1.000 | 728 | tags=14%, list=6%, signal=15% |
| 2008 | GOBP\_MONOAMINE\_TRANSPORT |  | 54 | -0.27 | -1.00 | 0.443 | 0.904 | 1.000 | 2628 | tags=41%, list=22%, signal=52% |
| 2009 | GOMF\_GAMMA\_CATENIN\_BINDING |  | 10 | -0.41 | -1.00 | 0.467 | 0.904 | 1.000 | 1976 | tags=40%, list=16%, signal=48% |
| 2010 | GOBP\_CELLULAR\_TRANSITION\_METAL\_ION\_HOMEOSTASIS |  | 72 | -0.25 | -1.00 | 0.447 | 0.904 | 1.000 | 1894 | tags=24%, list=16%, signal=28% |
| 2011 | GOBP\_PROTEIN\_DNA\_COMPLEX\_DISASSEMBLY |  | 14 | -0.37 | -1.00 | 0.453 | 0.905 | 1.000 | 3963 | tags=50%, list=33%, signal=75% |
| 2012 | GOMF\_COBALAMIN\_BINDING |  | 6 | -0.48 | -1.00 | 0.476 | 0.905 | 1.000 | 945 | tags=33%, list=8%, signal=36% |
| 2013 | GOMF\_THIOL\_OXIDASE\_ACTIVITY |  | 6 | -0.48 | -1.00 | 0.476 | 0.905 | 1.000 | 582 | tags=33%, list=5%, signal=35% |
| 2014 | GOBP\_CHAPERONE\_MEDIATED\_PROTEIN\_FOLDING |  | 46 | -0.28 | -1.00 | 0.461 | 0.907 | 1.000 | 1873 | tags=17%, list=16%, signal=21% |
| 2015 | GOBP\_REGULATION\_OF\_TAU\_PROTEIN\_KINASE\_ACTIVITY |  | 7 | -0.46 | -1.00 | 0.460 | 0.906 | 1.000 | 3367 | tags=43%, list=28%, signal=60% |
| 2016 | GOBP\_ENTERIC\_NERVOUS\_SYSTEM\_DEVELOPMENT |  | 7 | -0.46 | -1.00 | 0.472 | 0.907 | 1.000 | 141 | tags=14%, list=1%, signal=14% |
| 2017 | GOBP\_REGULATION\_OF\_PROTEIN\_ACETYLATION |  | 58 | -0.26 | -1.00 | 0.443 | 0.906 | 1.000 | 2974 | tags=34%, list=25%, signal=46% |
| 2018 | GOBP\_NEGATIVE\_REGULATION\_OF\_INTRINSIC\_APOPTOTIC\_SIGNALING\_PATHWAY\_BY\_P53\_CLASS\_MEDIATOR |  | 13 | -0.38 | -1.00 | 0.467 | 0.906 | 1.000 | 4815 | tags=77%, list=40%, signal=128% |
| 2019 | GOBP\_POSITIVE\_REGULATION\_OF\_CATECHOLAMINE\_SECRETION |  | 9 | -0.43 | -1.00 | 0.459 | 0.906 | 1.000 | 2560 | tags=44%, list=21%, signal=56% |
| 2020 | GOMF\_POLYNUCLEOTIDE\_ADENYLYLTRANSFERASE\_ACTIVITY |  | 3 | -0.59 | -1.00 | 0.510 | 0.906 | 1.000 | 4299 | tags=67%, list=36%, signal=104% |
| 2021 | GOCC\_REGION\_OF\_CYTOSOL |  | 19 | -0.34 | -1.00 | 0.452 | 0.909 | 1.000 | 3373 | tags=32%, list=28%, signal=44% |
| 2022 | GOBP\_REGULATION\_OF\_CLATHRIN\_DEPENDENT\_ENDOCYTOSIS |  | 16 | -0.36 | -1.00 | 0.452 | 0.908 | 1.000 | 726 | tags=19%, list=6%, signal=20% |
| 2023 | GOBP\_PULMONARY\_VALVE\_DEVELOPMENT |  | 18 | -0.34 | -1.00 | 0.448 | 0.908 | 1.000 | 2018 | tags=28%, list=17%, signal=33% |
| 2024 | GOBP\_PROSTATE\_GLANDULAR\_ACINUS\_DEVELOPMENT |  | 8 | -0.44 | -1.00 | 0.467 | 0.908 | 1.000 | 960 | tags=25%, list=8%, signal=27% |
| 2025 | GOBP\_CYTOPLASM\_ORGANIZATION |  | 7 | -0.46 | -1.00 | 0.458 | 0.908 | 1.000 | 960 | tags=29%, list=8%, signal=31% |
| 2026 | GOBP\_ATP\_SYNTHESIS\_COUPLED\_PROTON\_TRANSPORT |  | 4 | -0.55 | -1.00 | 0.508 | 0.907 | 1.000 | 3530 | tags=50%, list=29%, signal=71% |
| 2027 | GOCC\_PRC1\_COMPLEX |  | 12 | -0.39 | -1.00 | 0.463 | 0.908 | 1.000 | 904 | tags=25%, list=8%, signal=27% |
| 2028 | GOBP\_REGULATION\_OF\_NEUROGENESIS |  | 245 | -0.21 | -1.00 | 0.463 | 0.908 | 1.000 | 2127 | tags=19%, list=18%, signal=23% |
| 2029 | GOBP\_NEGATIVE\_REGULATION\_OF\_MONOOXYGENASE\_ACTIVITY |  | 7 | -0.46 | -1.00 | 0.464 | 0.908 | 1.000 | 771 | tags=29%, list=6%, signal=31% |
| 2030 | GOBP\_MAMMARY\_GLAND\_DEVELOPMENT |  | 95 | -0.24 | -1.00 | 0.456 | 0.908 | 1.000 | 2089 | tags=23%, list=17%, signal=28% |
| 2031 | GOBP\_TETRAHYDROFOLATE\_METABOLIC\_PROCESS |  | 15 | -0.36 | -1.00 | 0.468 | 0.908 | 1.000 | 532 | tags=20%, list=4%, signal=21% |
| 2032 | GOBP\_REGULATION\_OF\_HISTONE\_H3\_K14\_ACETYLATION |  | 4 | -0.54 | -1.00 | 0.501 | 0.907 | 1.000 | 2144 | tags=50%, list=18%, signal=61% |
| 2033 | GOMF\_BMP\_RECEPTOR\_BINDING |  | 9 | -0.42 | -1.00 | 0.448 | 0.907 | 1.000 | 350 | tags=22%, list=3%, signal=23% |
| 2034 | GOBP\_CELLULAR\_AMINO\_ACID\_BIOSYNTHETIC\_PROCESS |  | 49 | -0.27 | -1.00 | 0.453 | 0.907 | 1.000 | 1922 | tags=22%, list=16%, signal=27% |
| 2035 | GOBP\_FORMATION\_OF\_TRANSLATION\_PREINITIATION\_COMPLEX |  | 9 | -0.42 | -1.00 | 0.471 | 0.908 | 1.000 | 3229 | tags=44%, list=27%, signal=61% |
| 2036 | GOMF\_TRNA\_SPECIFIC\_RIBONUCLEASE\_ACTIVITY |  | 8 | -0.43 | -1.00 | 0.464 | 0.908 | 1.000 | 4530 | tags=50%, list=38%, signal=80% |
| 2037 | GOBP\_DEVELOPMENT\_OF\_SECONDARY\_SEXUAL\_CHARACTERISTICS |  | 4 | -0.54 | -1.00 | 0.507 | 0.908 | 1.000 | 2452 | tags=50%, list=20%, signal=63% |
| 2038 | GOBP\_TRANSITION\_METAL\_ION\_HOMEOSTASIS |  | 88 | -0.24 | -1.00 | 0.459 | 0.908 | 1.000 | 1894 | tags=23%, list=16%, signal=27% |
| 2039 | GOBP\_REGULATION\_OF\_TRANSCRIPTION\_BY\_RNA\_POLYMERASE\_I |  | 24 | -0.32 | -1.00 | 0.460 | 0.908 | 1.000 | 2541 | tags=42%, list=21%, signal=53% |
| 2040 | GOBP\_PROTEIN\_FOLDING |  | 166 | -0.22 | -1.00 | 0.462 | 0.908 | 1.000 | 3367 | tags=29%, list=28%, signal=40% |
| 2041 | GOCC\_CELL\_BODY\_MEMBRANE |  | 16 | -0.36 | -1.00 | 0.457 | 0.908 | 1.000 | 1444 | tags=31%, list=12%, signal=35% |
| 2042 | GOMF\_DNA\_N\_GLYCOSYLASE\_ACTIVITY |  | 10 | -0.41 | -1.00 | 0.463 | 0.907 | 1.000 | 4012 | tags=60%, list=33%, signal=90% |
| 2043 | GOMF\_CAMP\_RESPONSE\_ELEMENT\_BINDING\_PROTEIN\_BINDING |  | 8 | -0.44 | -1.00 | 0.463 | 0.907 | 1.000 | 3895 | tags=63%, list=32%, signal=92% |
| 2044 | GOBP\_POSITIVE\_REGULATION\_OF\_3\_UTR\_MEDIATED\_MRNA\_STABILIZATION |  | 3 | -0.59 | -0.99 | 0.523 | 0.909 | 1.000 | 1138 | tags=33%, list=9%, signal=37% |
| 2045 | GOMF\_ADENYL\_NUCLEOTIDE\_EXCHANGE\_FACTOR\_ACTIVITY |  | 11 | -0.40 | -0.99 | 0.465 | 0.909 | 1.000 | 3649 | tags=45%, list=30%, signal=65% |
| 2046 | GOBP\_NEGATIVE\_REGULATION\_OF\_TRANSFERASE\_ACTIVITY |  | 214 | -0.21 | -0.99 | 0.455 | 0.909 | 1.000 | 1837 | tags=18%, list=15%, signal=21% |
| 2047 | GOMF\_KINESIN\_BINDING |  | 30 | -0.30 | -0.99 | 0.451 | 0.909 | 1.000 | 1299 | tags=13%, list=11%, signal=15% |
| 2048 | GOBP\_REGULATION\_OF\_MONOCYTE\_DIFFERENTIATION |  | 16 | -0.35 | -0.99 | 0.451 | 0.909 | 1.000 | 2046 | tags=44%, list=17%, signal=53% |
| 2049 | GOBP\_AMINO\_SUGAR\_CATABOLIC\_PROCESS |  | 6 | -0.47 | -0.99 | 0.485 | 0.909 | 1.000 | 124 | tags=17%, list=1%, signal=17% |
| 2050 | GOBP\_PERICARDIUM\_DEVELOPMENT |  | 15 | -0.37 | -0.99 | 0.474 | 0.909 | 1.000 | 563 | tags=20%, list=5%, signal=21% |
| 2051 | GOBP\_REGULATION\_OF\_NEURON\_PROJECTION\_DEVELOPMENT |  | 311 | -0.20 | -0.99 | 0.471 | 0.910 | 1.000 | 1742 | tags=17%, list=15%, signal=19% |
| 2052 | GOCC\_ALVEOLAR\_LAMELLAR\_BODY |  | 4 | -0.53 | -0.99 | 0.489 | 0.911 | 1.000 | 2913 | tags=75%, list=24%, signal=99% |
| 2053 | GOBP\_PSEUDOURIDINE\_SYNTHESIS |  | 15 | -0.36 | -0.99 | 0.468 | 0.911 | 1.000 | 2466 | tags=33%, list=21%, signal=42% |
| 2054 | GOBP\_POSITIVE\_REGULATION\_OF\_SYNAPSE\_ASSEMBLY |  | 31 | -0.30 | -0.99 | 0.468 | 0.911 | 1.000 | 3020 | tags=39%, list=25%, signal=52% |
| 2055 | GOBP\_ESTROGEN\_METABOLIC\_PROCESS |  | 17 | -0.35 | -0.99 | 0.466 | 0.911 | 1.000 | 322 | tags=24%, list=3%, signal=24% |
| 2056 | GOBP\_SKELETAL\_MUSCLE\_ACETYLCHOLINE\_GATED\_CHANNEL\_CLUSTERING |  | 9 | -0.42 | -0.99 | 0.460 | 0.912 | 1.000 | 3295 | tags=56%, list=27%, signal=77% |
| 2057 | GOBP\_LIPID\_GLYCOSYLATION |  | 4 | -0.54 | -0.99 | 0.502 | 0.912 | 1.000 | 3946 | tags=75%, list=33%, signal=112% |
| 2058 | GOBP\_REGULATION\_OF\_GOLGI\_TO\_PLASMA\_MEMBRANE\_PROTEIN\_TRANSPORT |  | 6 | -0.47 | -0.99 | 0.487 | 0.912 | 1.000 | 1748 | tags=50%, list=15%, signal=58% |
| 2059 | GOBP\_POSITIVE\_REGULATION\_OF\_T\_CELL\_PROLIFERATION |  | 57 | -0.26 | -0.99 | 0.466 | 0.913 | 1.000 | 2427 | tags=37%, list=20%, signal=46% |
| 2060 | GOBP\_CELLULAR\_COMPONENT\_MORPHOGENESIS |  | 535 | -0.19 | -0.99 | 0.488 | 0.913 | 1.000 | 2311 | tags=22%, list=19%, signal=26% |
| 2061 | GOBP\_NEURON\_PROJECTION\_GUIDANCE |  | 190 | -0.21 | -0.99 | 0.469 | 0.913 | 1.000 | 2340 | tags=26%, list=19%, signal=32% |
| 2062 | GOBP\_BRONCHIOLE\_DEVELOPMENT |  | 4 | -0.54 | -0.99 | 0.509 | 0.913 | 1.000 | 382 | tags=25%, list=3%, signal=26% |
| 2063 | GOBP\_XENOBIOTIC\_TRANSPORT |  | 30 | -0.30 | -0.99 | 0.459 | 0.913 | 1.000 | 2985 | tags=43%, list=25%, signal=58% |
| 2064 | GOBP\_CELL\_GROWTH |  | 320 | -0.20 | -0.99 | 0.481 | 0.913 | 1.000 | 2846 | tags=27%, list=24%, signal=34% |
| 2065 | GOBP\_REGULATION\_OF\_POSTSYNAPTIC\_MEMBRANE\_POTENTIAL |  | 58 | -0.26 | -0.99 | 0.468 | 0.912 | 1.000 | 1457 | tags=21%, list=12%, signal=23% |
| 2066 | GOMF\_PROTEIN\_DEACETYLASE\_ACTIVITY |  | 19 | -0.34 | -0.99 | 0.469 | 0.913 | 1.000 | 4241 | tags=63%, list=35%, signal=97% |
| 2067 | GOMF\_INTERLEUKIN\_17\_RECEPTOR\_ACTIVITY |  | 4 | -0.54 | -0.99 | 0.513 | 0.913 | 1.000 | 306 | tags=25%, list=3%, signal=26% |
| 2068 | GOBP\_DETECTION\_OF\_CALCIUM\_ION |  | 10 | -0.41 | -0.99 | 0.460 | 0.913 | 1.000 | 1005 | tags=40%, list=8%, signal=44% |
| 2069 | GOBP\_EAR\_DEVELOPMENT |  | 123 | -0.23 | -0.99 | 0.484 | 0.914 | 1.000 | 2018 | tags=22%, list=17%, signal=26% |
| 2070 | GOBP\_SECONDARY\_METABOLIC\_PROCESS |  | 28 | -0.30 | -0.99 | 0.470 | 0.914 | 1.000 | 1881 | tags=29%, list=16%, signal=34% |
| 2071 | GOMF\_SYNDECAN\_BINDING |  | 5 | -0.50 | -0.99 | 0.491 | 0.915 | 1.000 | 261 | tags=20%, list=2%, signal=20% |
| 2072 | GOMF\_LACTATE\_DEHYDROGENASE\_ACTIVITY |  | 3 | -0.59 | -0.99 | 0.533 | 0.915 | 1.000 | 2064 | tags=67%, list=17%, signal=80% |
| 2073 | GOBP\_NEGATIVE\_REGULATION\_OF\_INTERLEUKIN\_6\_PRODUCTION |  | 22 | -0.33 | -0.99 | 0.469 | 0.914 | 1.000 | 2962 | tags=36%, list=25%, signal=48% |
| 2074 | GOBP\_CDC42\_PROTEIN\_SIGNAL\_TRANSDUCTION |  | 9 | -0.42 | -0.99 | 0.474 | 0.914 | 1.000 | 364 | tags=22%, list=3%, signal=23% |
| 2075 | GOBP\_GLUTAMATE\_SECRETION |  | 29 | -0.30 | -0.99 | 0.471 | 0.915 | 1.000 | 1424 | tags=28%, list=12%, signal=31% |
| 2076 | GOBP\_REGULATION\_OF\_POSTSYNAPTIC\_DENSITY\_ORGANIZATION |  | 8 | -0.44 | -0.99 | 0.482 | 0.914 | 1.000 | 3918 | tags=63%, list=33%, signal=93% |
| 2077 | GOBP\_POSITIVE\_REGULATION\_OF\_MRNA\_SPLICING\_VIA\_SPLICEOSOME |  | 15 | -0.36 | -0.99 | 0.476 | 0.915 | 1.000 | 40 | tags=7%, list=0%, signal=7% |
| 2078 | GOBP\_CELLULAR\_RESPONSE\_TO\_NITROGEN\_STARVATION |  | 9 | -0.42 | -0.99 | 0.481 | 0.915 | 1.000 | 2222 | tags=33%, list=18%, signal=41% |
| 2079 | GOBP\_POSITIVE\_REGULATION\_OF\_INTERLEUKIN\_8\_PRODUCTION |  | 33 | -0.30 | -0.99 | 0.465 | 0.916 | 1.000 | 1498 | tags=24%, list=12%, signal=28% |
| 2080 | GOBP\_T\_CELL\_DIFFERENTIATION\_IN\_THYMUS |  | 57 | -0.26 | -0.99 | 0.476 | 0.916 | 1.000 | 3301 | tags=46%, list=27%, signal=63% |
| 2081 | GOBP\_REGULATION\_OF\_CILIUM\_DEPENDENT\_CELL\_MOTILITY |  | 11 | -0.39 | -0.99 | 0.478 | 0.916 | 1.000 | 2586 | tags=36%, list=22%, signal=46% |
| 2082 | GOBP\_REGULATION\_OF\_TRANSLATIONAL\_TERMINATION |  | 7 | -0.45 | -0.99 | 0.479 | 0.916 | 1.000 | 5078 | tags=71%, list=42%, signal=124% |
| 2083 | GOBP\_REGULATION\_OF\_HISTONE\_UBIQUITINATION |  | 10 | -0.40 | -0.99 | 0.470 | 0.916 | 1.000 | 3813 | tags=40%, list=32%, signal=59% |
| 2084 | GOBP\_PROTEIN\_LOCALIZATION\_TO\_MITOCHONDRION |  | 108 | -0.23 | -0.99 | 0.466 | 0.916 | 1.000 | 3649 | tags=39%, list=30%, signal=55% |
| 2085 | GOMF\_SERINE\_HYDROLASE\_ACTIVITY |  | 79 | -0.25 | -0.99 | 0.490 | 0.917 | 1.000 | 864 | tags=14%, list=7%, signal=15% |
| 2086 | GOBP\_POSITIVE\_REGULATION\_OF\_LEUKOCYTE\_PROLIFERATION |  | 91 | -0.24 | -0.99 | 0.475 | 0.916 | 1.000 | 2427 | tags=33%, list=20%, signal=41% |
| 2087 | GOBP\_REGULATION\_OF\_COHESIN\_LOADING |  | 3 | -0.59 | -0.99 | 0.548 | 0.916 | 1.000 | 1684 | tags=33%, list=14%, signal=39% |
| 2088 | GOBP\_REGULATION\_OF\_MORPHOGENESIS\_OF\_A\_BRANCHING\_STRUCTURE |  | 32 | -0.30 | -0.99 | 0.479 | 0.916 | 1.000 | 2089 | tags=28%, list=17%, signal=34% |
| 2089 | GOBP\_GALACTOLIPID\_METABOLIC\_PROCESS |  | 5 | -0.50 | -0.99 | 0.498 | 0.916 | 1.000 | 3244 | tags=60%, list=27%, signal=82% |
| 2090 | GOBP\_REGULATION\_OF\_BLOOD\_PRESSURE |  | 108 | -0.23 | -0.99 | 0.480 | 0.915 | 1.000 | 1630 | tags=19%, list=14%, signal=21% |
| 2091 | GOBP\_EPITHELIAL\_TUBE\_BRANCHING\_INVOLVED\_IN\_LUNG\_MORPHOGENESIS |  | 23 | -0.32 | -0.99 | 0.484 | 0.916 | 1.000 | 2190 | tags=26%, list=18%, signal=32% |
| 2092 | GOBP\_ADP\_BIOSYNTHETIC\_PROCESS |  | 4 | -0.54 | -0.99 | 0.516 | 0.916 | 1.000 | 2588 | tags=50%, list=22%, signal=64% |
| 2093 | GOBP\_REGULATION\_OF\_AEROBIC\_RESPIRATION |  | 5 | -0.50 | -0.99 | 0.495 | 0.916 | 1.000 | 4286 | tags=80%, list=36%, signal=124% |
| 2094 | GOCC\_LATE\_ENDOSOME |  | 202 | -0.21 | -0.99 | 0.489 | 0.915 | 1.000 | 2582 | tags=24%, list=21%, signal=30% |
| 2095 | GOBP\_ATP\_METABOLIC\_PROCESS |  | 201 | -0.21 | -0.98 | 0.499 | 0.916 | 1.000 | 4173 | tags=43%, list=35%, signal=64% |
| 2096 | GOMF\_WD40\_REPEAT\_DOMAIN\_BINDING |  | 4 | -0.53 | -0.98 | 0.510 | 0.917 | 1.000 | 5680 | tags=100%, list=47%, signal=190% |
| 2097 | GOBP\_NEUROTROPHIN\_SIGNALING\_PATHWAY |  | 28 | -0.30 | -0.98 | 0.475 | 0.917 | 1.000 | 2012 | tags=21%, list=17%, signal=26% |
| 2098 | GOBP\_NEURON\_DEVELOPMENT |  | 766 | -0.19 | -0.98 | 0.538 | 0.918 | 1.000 | 1712 | tags=17%, list=14%, signal=18% |
| 2099 | GOCC\_INTRINSIC\_COMPONENT\_OF\_MITOCHONDRIAL\_MEMBRANE |  | 66 | -0.25 | -0.98 | 0.479 | 0.917 | 1.000 | 3722 | tags=39%, list=31%, signal=57% |
| 2100 | GOBP\_PRIMARY\_AMINO\_COMPOUND\_METABOLIC\_PROCESS |  | 9 | -0.42 | -0.98 | 0.487 | 0.918 | 1.000 | 1232 | tags=33%, list=10%, signal=37% |
| 2101 | GOBP\_DEVELOPMENTAL\_INDUCTION |  | 20 | -0.33 | -0.98 | 0.483 | 0.918 | 1.000 | 2195 | tags=35%, list=18%, signal=43% |
| 2102 | GOBP\_CHRONIC\_INFLAMMATORY\_RESPONSE |  | 10 | -0.40 | -0.98 | 0.489 | 0.918 | 1.000 | 1424 | tags=30%, list=12%, signal=34% |
| 2103 | GOMF\_POTASSIUM\_CHANNEL\_INHIBITOR\_ACTIVITY |  | 6 | -0.48 | -0.98 | 0.497 | 0.919 | 1.000 | 2662 | tags=33%, list=22%, signal=43% |
| 2104 | GOBP\_CARNITINE\_BIOSYNTHETIC\_PROCESS |  | 4 | -0.53 | -0.98 | 0.514 | 0.919 | 1.000 | 1724 | tags=50%, list=14%, signal=58% |
| 2105 | GOBP\_REGULATION\_OF\_AUTOPHAGY\_OF\_MITOCHONDRION\_IN\_RESPONSE\_TO\_MITOCHONDRIAL\_DEPOLARIZATION |  | 8 | -0.43 | -0.98 | 0.494 | 0.919 | 1.000 | 1820 | tags=50%, list=15%, signal=59% |
| 2106 | GOMF\_ADP\_RIBOSE\_DIPHOSPHATASE\_ACTIVITY |  | 4 | -0.53 | -0.98 | 0.517 | 0.919 | 1.000 | 5607 | tags=100%, list=47%, signal=187% |
| 2107 | GOMF\_PROTEIN\_SERINE\_PHOSPHATASE\_ACTIVITY |  | 56 | -0.26 | -0.98 | 0.485 | 0.919 | 1.000 | 4132 | tags=38%, list=34%, signal=57% |
| 2108 | GOMF\_OXIDIZED\_DNA\_BINDING |  | 8 | -0.43 | -0.98 | 0.491 | 0.919 | 1.000 | 371 | tags=13%, list=3%, signal=13% |
| 2109 | GOBP\_RESPONSE\_TO\_CADMIUM\_ION |  | 33 | -0.29 | -0.98 | 0.490 | 0.918 | 1.000 | 1130 | tags=15%, list=9%, signal=17% |
| 2110 | GOBP\_POSITIVE\_REGULATION\_OF\_EMBRYONIC\_DEVELOPMENT |  | 11 | -0.39 | -0.98 | 0.488 | 0.918 | 1.000 | 669 | tags=18%, list=6%, signal=19% |
| 2111 | GOMF\_EXOPEPTIDASE\_ACTIVITY |  | 65 | -0.25 | -0.98 | 0.478 | 0.918 | 1.000 | 2190 | tags=31%, list=18%, signal=37% |
| 2112 | GOBP\_POSITIVE\_REGULATION\_OF\_TRANSLATION |  | 90 | -0.24 | -0.98 | 0.480 | 0.919 | 1.000 | 2512 | tags=26%, list=21%, signal=32% |
| 2113 | GOBP\_BONE\_TRABECULA\_FORMATION |  | 6 | -0.47 | -0.98 | 0.493 | 0.919 | 1.000 | 2042 | tags=33%, list=17%, signal=40% |
| 2114 | GOBP\_CENTROSOME\_SEPARATION |  | 13 | -0.37 | -0.98 | 0.483 | 0.918 | 1.000 | 752 | tags=15%, list=6%, signal=16% |
| 2115 | GOBP\_ACTIVATION\_OF\_JUN\_KINASE\_ACTIVITY |  | 27 | -0.31 | -0.98 | 0.481 | 0.918 | 1.000 | 2083 | tags=22%, list=17%, signal=27% |
| 2116 | GOMF\_MONOAMINE\_TRANSMEMBRANE\_TRANSPORTER\_ACTIVITY |  | 6 | -0.47 | -0.98 | 0.490 | 0.918 | 1.000 | 1096 | tags=50%, list=9%, signal=55% |
| 2117 | GOBP\_REGULATION\_OF\_MITOCHONDRIAL\_OUTER\_MEMBRANE\_PERMEABILIZATION\_INVOLVED\_IN\_APOPTOTIC\_SIGNALING\_PATHWAY |  | 28 | -0.30 | -0.98 | 0.481 | 0.918 | 1.000 | 1403 | tags=18%, list=12%, signal=20% |
| 2118 | GOBP\_N\_ACETYLNEURAMINATE\_CATABOLIC\_PROCESS |  | 4 | -0.53 | -0.98 | 0.524 | 0.918 | 1.000 | 124 | tags=25%, list=1%, signal=25% |
| 2119 | GOMF\_RACEMASE\_AND\_EPIMERASE\_ACTIVITY |  | 10 | -0.40 | -0.98 | 0.486 | 0.918 | 1.000 | 1486 | tags=40%, list=12%, signal=46% |
| 2120 | GOBP\_NEGATIVE\_REGULATION\_OF\_ENDOPLASMIC\_RETICULUM\_CALCIUM\_ION\_CONCENTRATION |  | 5 | -0.50 | -0.98 | 0.496 | 0.918 | 1.000 | 1747 | tags=40%, list=15%, signal=47% |
| 2121 | GOBP\_POSITIVE\_REGULATION\_OF\_DENDRITIC\_CELL\_CHEMOTAXIS |  | 5 | -0.50 | -0.98 | 0.513 | 0.917 | 1.000 | 1063 | tags=20%, list=9%, signal=22% |
| 2122 | GOMF\_OXIDOREDUCTASE\_ACTIVITY\_ACTING\_ON\_NAD\_P\_H\_OXYGEN\_AS\_ACCEPTOR |  | 9 | -0.41 | -0.98 | 0.489 | 0.919 | 1.000 | 1292 | tags=33%, list=11%, signal=37% |
| 2123 | GOBP\_REGULATION\_OF\_MITOCHONDRIAL\_RNA\_CATABOLIC\_PROCESS |  | 5 | -0.50 | -0.98 | 0.506 | 0.919 | 1.000 | 4689 | tags=80%, list=39%, signal=131% |
| 2124 | GOBP\_GRANULOCYTE\_DIFFERENTIATION |  | 23 | -0.32 | -0.98 | 0.483 | 0.919 | 1.000 | 2760 | tags=39%, list=23%, signal=51% |
| 2125 | GOBP\_B\_CELL\_ACTIVATION\_INVOLVED\_IN\_IMMUNE\_RESPONSE |  | 57 | -0.26 | -0.98 | 0.498 | 0.919 | 1.000 | 3223 | tags=40%, list=27%, signal=55% |
| 2126 | GOBP\_AMINE\_METABOLIC\_PROCESS |  | 104 | -0.23 | -0.98 | 0.502 | 0.920 | 1.000 | 2300 | tags=23%, list=19%, signal=28% |
| 2127 | GOBP\_POSITIVE\_REGULATION\_OF\_DNA\_BINDING\_TRANSCRIPTION\_FACTOR\_ACTIVITY |  | 171 | -0.21 | -0.98 | 0.507 | 0.920 | 1.000 | 2742 | tags=28%, list=23%, signal=36% |
| 2128 | GOBP\_SALIVARY\_GLAND\_DEVELOPMENT |  | 28 | -0.30 | -0.98 | 0.484 | 0.921 | 1.000 | 2943 | tags=29%, list=25%, signal=38% |
| 2129 | GOBP\_VASCULAR\_ASSOCIATED\_SMOOTH\_MUSCLE\_CELL\_APOPTOTIC\_PROCESS |  | 9 | -0.42 | -0.98 | 0.484 | 0.922 | 1.000 | 2421 | tags=33%, list=20%, signal=42% |
| 2130 | GOMF\_PEROXISOME\_PROLIFERATOR\_ACTIVATED\_RECEPTOR\_BINDING |  | 9 | -0.41 | -0.98 | 0.495 | 0.923 | 1.000 | 4388 | tags=56%, list=37%, signal=87% |
| 2131 | GOCC\_POSTSYNAPSE |  | 397 | -0.19 | -0.98 | 0.550 | 0.923 | 1.000 | 2391 | tags=23%, list=20%, signal=28% |
| 2132 | GOBP\_ENDOSOME\_TO\_PLASMA\_MEMBRANE\_PROTEIN\_TRANSPORT |  | 10 | -0.40 | -0.98 | 0.492 | 0.922 | 1.000 | 194 | tags=10%, list=2%, signal=10% |
| 2133 | GOMF\_AMINO\_ACID\_CATION\_SYMPORTER\_ACTIVITY |  | 11 | -0.39 | -0.98 | 0.482 | 0.922 | 1.000 | 553 | tags=27%, list=5%, signal=29% |
| 2134 | GOBP\_RESPONSE\_TO\_MURAMYL\_DIPEPTIDE |  | 15 | -0.36 | -0.98 | 0.490 | 0.923 | 1.000 | 1638 | tags=20%, list=14%, signal=23% |
| 2135 | GOBP\_POSITIVE\_REGULATION\_OF\_TORC1\_SIGNALING |  | 12 | -0.38 | -0.98 | 0.485 | 0.922 | 1.000 | 3981 | tags=58%, list=33%, signal=87% |
| 2136 | GOBP\_CGMP\_MEDIATED\_SIGNALING |  | 13 | -0.37 | -0.98 | 0.488 | 0.922 | 1.000 | 659 | tags=15%, list=5%, signal=16% |
| 2137 | GOBP\_NUCLEOTIDE\_SUGAR\_BIOSYNTHETIC\_PROCESS |  | 19 | -0.33 | -0.98 | 0.477 | 0.922 | 1.000 | 433 | tags=16%, list=4%, signal=16% |
| 2138 | GOBP\_POSITIVE\_REGULATION\_OF\_MYELOID\_LEUKOCYTE\_CYTOKINE\_PRODUCTION\_INVOLVED\_IN\_IMMUNE\_RESPONSE |  | 13 | -0.37 | -0.98 | 0.478 | 0.922 | 1.000 | 1089 | tags=23%, list=9%, signal=25% |
| 2139 | GOBP\_NEGATIVE\_REGULATION\_OF\_LIPOPROTEIN\_PARTICLE\_CLEARANCE |  | 4 | -0.53 | -0.98 | 0.524 | 0.923 | 1.000 | 3313 | tags=50%, list=28%, signal=69% |
| 2140 | GOMF\_OXIDOREDUCTASE\_ACTIVITY\_ACTING\_ON\_THE\_ALDEHYDE\_OR\_OXO\_GROUP\_OF\_DONORS\_DISULFIDE\_AS\_ACCEPTOR |  | 7 | -0.45 | -0.98 | 0.488 | 0.923 | 1.000 | 3386 | tags=43%, list=28%, signal=60% |
| 2141 | GOMF\_RIBOSOMAL\_PROTEIN\_S6\_KINASE\_ACTIVITY |  | 6 | -0.47 | -0.97 | 0.515 | 0.924 | 1.000 | 4871 | tags=67%, list=41%, signal=112% |
| 2142 | GOBP\_NEGATIVE\_REGULATION\_OF\_CHEMOKINE\_PRODUCTION |  | 12 | -0.38 | -0.97 | 0.486 | 0.925 | 1.000 | 1038 | tags=25%, list=9%, signal=27% |
| 2143 | GOBP\_INTERLEUKIN\_8\_PRODUCTION |  | 45 | -0.27 | -0.97 | 0.481 | 0.925 | 1.000 | 1498 | tags=22%, list=12%, signal=25% |
| 2144 | GOBP\_RESPONSE\_TO\_GROWTH\_HORMONE |  | 25 | -0.31 | -0.97 | 0.502 | 0.926 | 1.000 | 2389 | tags=28%, list=20%, signal=35% |
| 2145 | GOBP\_REGULATION\_OF\_HYDROGEN\_PEROXIDE\_METABOLIC\_PROCESS |  | 10 | -0.39 | -0.97 | 0.491 | 0.928 | 1.000 | 1300 | tags=30%, list=11%, signal=34% |
| 2146 | GOBP\_EXTRAEMBRYONIC\_MEMBRANE\_DEVELOPMENT |  | 6 | -0.47 | -0.97 | 0.500 | 0.927 | 1.000 | 350 | tags=17%, list=3%, signal=17% |
| 2147 | GOBP\_POSITIVE\_REGULATION\_OF\_RECEPTOR\_RECYCLING |  | 10 | -0.40 | -0.97 | 0.490 | 0.927 | 1.000 | 814 | tags=30%, list=7%, signal=32% |
| 2148 | GOBP\_ANTERIOR\_HEAD\_DEVELOPMENT |  | 3 | -0.57 | -0.97 | 0.546 | 0.928 | 1.000 | 3830 | tags=33%, list=32%, signal=49% |
| 2149 | GOBP\_CENTRAL\_NERVOUS\_SYSTEM\_NEURON\_DIFFERENTIATION |  | 116 | -0.23 | -0.97 | 0.527 | 0.927 | 1.000 | 2699 | tags=26%, list=22%, signal=33% |
| 2150 | GOCC\_ENZYME\_ACTIVATOR\_COMPLEX |  | 5 | -0.49 | -0.97 | 0.512 | 0.927 | 1.000 | 1937 | tags=20%, list=16%, signal=24% |
| 2151 | GOMF\_INSULIN\_BINDING |  | 5 | -0.50 | -0.97 | 0.518 | 0.928 | 1.000 | 4069 | tags=60%, list=34%, signal=91% |
| 2152 | GOBP\_TUBE\_FORMATION |  | 100 | -0.23 | -0.97 | 0.523 | 0.928 | 1.000 | 2042 | tags=24%, list=17%, signal=29% |
| 2153 | GOMF\_HYDROXYMETHYL\_FORMYL\_AND\_RELATED\_TRANSFERASE\_ACTIVITY |  | 6 | -0.46 | -0.97 | 0.494 | 0.928 | 1.000 | 255 | tags=17%, list=2%, signal=17% |
| 2154 | GOMF\_COMPLEMENT\_COMPONENT\_C1Q\_COMPLEX\_BINDING |  | 6 | -0.47 | -0.97 | 0.516 | 0.929 | 1.000 | 1958 | tags=50%, list=16%, signal=60% |
| 2155 | GOBP\_POSITIVE\_REGULATION\_OF\_ANIMAL\_ORGAN\_MORPHOGENESIS |  | 21 | -0.33 | -0.97 | 0.487 | 0.929 | 1.000 | 197 | tags=14%, list=2%, signal=14% |
| 2156 | GOBP\_NEUROTRANSMITTER\_CATABOLIC\_PROCESS |  | 8 | -0.43 | -0.97 | 0.495 | 0.929 | 1.000 | 2570 | tags=38%, list=21%, signal=48% |
| 2157 | GOBP\_TRABECULA\_MORPHOGENESIS |  | 33 | -0.29 | -0.97 | 0.497 | 0.928 | 1.000 | 350 | tags=12%, list=3%, signal=12% |
| 2158 | GOBP\_PROTEOGLYCAN\_METABOLIC\_PROCESS |  | 66 | -0.25 | -0.97 | 0.503 | 0.928 | 1.000 | 1999 | tags=23%, list=17%, signal=27% |
| 2159 | GOBP\_VASCULAR\_ASSOCIATED\_SMOOTH\_MUSCLE\_CELL\_DIFFERENTIATION |  | 23 | -0.31 | -0.97 | 0.503 | 0.928 | 1.000 | 363 | tags=9%, list=3%, signal=9% |
| 2160 | GOBP\_NEURON\_FATE\_COMMITMENT |  | 28 | -0.30 | -0.97 | 0.500 | 0.928 | 1.000 | 669 | tags=14%, list=6%, signal=15% |
| 2161 | GOBP\_TRANSDIFFERENTIATION |  | 5 | -0.49 | -0.97 | 0.513 | 0.929 | 1.000 | 1130 | tags=40%, list=9%, signal=44% |
| 2162 | GOBP\_SEROTONIN\_TRANSPORT |  | 14 | -0.36 | -0.97 | 0.495 | 0.928 | 1.000 | 1096 | tags=36%, list=9%, signal=39% |
| 2163 | GOBP\_LEARNING |  | 82 | -0.24 | -0.97 | 0.518 | 0.928 | 1.000 | 1691 | tags=21%, list=14%, signal=24% |
| 2164 | GOBP\_REGULATION\_OF\_LYMPHOCYTE\_ACTIVATION |  | 275 | -0.20 | -0.97 | 0.564 | 0.928 | 1.000 | 3240 | tags=35%, list=27%, signal=47% |
| 2165 | GOBP\_PSEUDOPODIUM\_ORGANIZATION |  | 11 | -0.39 | -0.97 | 0.495 | 0.929 | 1.000 | 1805 | tags=27%, list=15%, signal=32% |
| 2166 | GOMF\_TYPE\_5\_METABOTROPIC\_GLUTAMATE\_RECEPTOR\_BINDING |  | 4 | -0.53 | -0.97 | 0.532 | 0.929 | 1.000 | 362 | tags=25%, list=3%, signal=26% |
| 2167 | GOMF\_CHROMATIN\_INSULATOR\_SEQUENCE\_BINDING |  | 3 | -0.57 | -0.97 | 0.556 | 0.929 | 1.000 | 5092 | tags=67%, list=42%, signal=116% |
| 2168 | GOBP\_RESPONSE\_TO\_MAGNESIUM\_ION |  | 11 | -0.38 | -0.97 | 0.507 | 0.929 | 1.000 | 771 | tags=18%, list=6%, signal=19% |
| 2169 | GOBP\_NATURAL\_KILLER\_CELL\_DIFFERENTIATION |  | 14 | -0.36 | -0.97 | 0.509 | 0.929 | 1.000 | 3133 | tags=50%, list=26%, signal=68% |
| 2170 | GOBP\_TOXIN\_METABOLIC\_PROCESS |  | 8 | -0.42 | -0.97 | 0.492 | 0.929 | 1.000 | 1881 | tags=50%, list=16%, signal=59% |
| 2171 | GOBP\_REGULATION\_OF\_CILIUM\_MOVEMENT |  | 13 | -0.37 | -0.97 | 0.507 | 0.929 | 1.000 | 448 | tags=15%, list=4%, signal=16% |
| 2172 | GOBP\_REGULATION\_OF\_RECEPTOR\_CLUSTERING |  | 8 | -0.42 | -0.97 | 0.487 | 0.929 | 1.000 | 3477 | tags=63%, list=29%, signal=88% |
| 2173 | GOBP\_CELL\_DIFFERENTIATION\_IN\_SPINAL\_CORD |  | 27 | -0.31 | -0.97 | 0.498 | 0.928 | 1.000 | 1903 | tags=26%, list=16%, signal=31% |
| 2174 | GOBP\_NEGATIVE\_REGULATION\_OF\_CHROMATIN\_SILENCING |  | 4 | -0.53 | -0.97 | 0.539 | 0.928 | 1.000 | 5260 | tags=75%, list=44%, signal=133% |
| 2175 | GOMF\_ASPARTIC\_ENDOPEPTIDASE\_ACTIVITY\_INTRAMEMBRANE\_CLEAVING |  | 6 | -0.46 | -0.97 | 0.513 | 0.928 | 1.000 | 4438 | tags=67%, list=37%, signal=106% |
| 2176 | GOMF\_VITAMIN\_TRANSMEMBRANE\_TRANSPORTER\_ACTIVITY |  | 20 | -0.33 | -0.97 | 0.488 | 0.928 | 1.000 | 1476 | tags=25%, list=12%, signal=28% |
| 2177 | GOBP\_BETA\_CATENIN\_DESTRUCTION\_COMPLEX\_DISASSEMBLY |  | 13 | -0.37 | -0.97 | 0.503 | 0.928 | 1.000 | 3066 | tags=38%, list=26%, signal=52% |
| 2178 | GOMF\_CHLORIDE\_CHANNEL\_REGULATOR\_ACTIVITY |  | 12 | -0.38 | -0.97 | 0.493 | 0.928 | 1.000 | 411 | tags=17%, list=3%, signal=17% |
| 2179 | GOBP\_POSITIVE\_REGULATION\_OF\_KIDNEY\_DEVELOPMENT |  | 7 | -0.44 | -0.97 | 0.505 | 0.928 | 1.000 | 502 | tags=29%, list=4%, signal=30% |
| 2180 | GOBP\_NUCLEOBASE\_CONTAINING\_COMPOUND\_TRANSPORT |  | 186 | -0.21 | -0.97 | 0.545 | 0.927 | 1.000 | 3053 | tags=26%, list=25%, signal=34% |
| 2181 | GOMF\_INTRAMOLECULAR\_OXIDOREDUCTASE\_ACTIVITY\_TRANSPOSING\_C\_C\_BONDS |  | 6 | -0.46 | -0.97 | 0.511 | 0.928 | 1.000 | 1313 | tags=67%, list=11%, signal=75% |
| 2182 | GOBP\_SUBSTRATE\_DEPENDENT\_CELL\_MIGRATION |  | 20 | -0.33 | -0.97 | 0.495 | 0.928 | 1.000 | 197 | tags=10%, list=2%, signal=10% |
| 2183 | GOBP\_NEGATIVE\_REGULATION\_OF\_VASCULAR\_ENDOTHELIAL\_CELL\_PROLIFERATION |  | 3 | -0.57 | -0.97 | 0.554 | 0.927 | 1.000 | 5184 | tags=100%, list=43%, signal=176% |
| 2184 | GOBP\_POSITIVE\_REGULATION\_OF\_EXOCYTOSIS |  | 61 | -0.25 | -0.97 | 0.520 | 0.928 | 1.000 | 1353 | tags=21%, list=11%, signal=24% |
| 2185 | GOMF\_DYNEIN\_LIGHT\_INTERMEDIATE\_CHAIN\_BINDING |  | 14 | -0.36 | -0.97 | 0.506 | 0.928 | 1.000 | 1020 | tags=14%, list=8%, signal=16% |
| 2186 | GOMF\_MRNA\_BINDING |  | 200 | -0.21 | -0.97 | 0.557 | 0.928 | 1.000 | 3100 | tags=25%, list=26%, signal=32% |
| 2187 | GOBP\_GLUTAMINE\_METABOLIC\_PROCESS |  | 15 | -0.35 | -0.97 | 0.498 | 0.928 | 1.000 | 1309 | tags=20%, list=11%, signal=22% |
| 2188 | GOBP\_CARDIAC\_MUSCLE\_CELL\_PROLIFERATION |  | 32 | -0.29 | -0.97 | 0.524 | 0.929 | 1.000 | 2810 | tags=31%, list=23%, signal=41% |
| 2189 | GOBP\_CD4\_POSITIVE\_OR\_CD8\_POSITIVE\_ALPHA\_BETA\_T\_CELL\_LINEAGE\_COMMITMENT |  | 12 | -0.38 | -0.97 | 0.493 | 0.929 | 1.000 | 2934 | tags=58%, list=24%, signal=77% |
| 2190 | GOBP\_IMMUNOGLOBULIN\_PRODUCTION |  | 62 | -0.25 | -0.97 | 0.514 | 0.929 | 1.000 | 3209 | tags=42%, list=27%, signal=57% |
| 2191 | GOBP\_NEUTROPHIL\_EXTRAVASATION |  | 10 | -0.39 | -0.97 | 0.499 | 0.929 | 1.000 | 3527 | tags=50%, list=29%, signal=71% |
| 2192 | GOBP\_BRAIN\_MORPHOGENESIS |  | 27 | -0.30 | -0.97 | 0.504 | 0.929 | 1.000 | 2656 | tags=33%, list=22%, signal=43% |
| 2193 | GOBP\_FOLIC\_ACID\_METABOLIC\_PROCESS |  | 14 | -0.36 | -0.96 | 0.503 | 0.930 | 1.000 | 532 | tags=14%, list=4%, signal=15% |
| 2194 | GOCC\_NUCLEOLAR\_RIBONUCLEASE\_P\_COMPLEX |  | 3 | -0.57 | -0.96 | 0.556 | 0.930 | 1.000 | 3935 | tags=67%, list=33%, signal=99% |
| 2195 | GOBP\_METANEPHRIC\_PART\_OF\_URETERIC\_BUD\_DEVELOPMENT |  | 3 | -0.57 | -0.96 | 0.560 | 0.931 | 1.000 | 5210 | tags=100%, list=43%, signal=177% |
| 2196 | GOMF\_NUCLEOSOME\_BINDING |  | 42 | -0.27 | -0.96 | 0.510 | 0.931 | 1.000 | 3963 | tags=40%, list=33%, signal=60% |
| 2197 | GOMF\_ACETYLTRANSFERASE\_ACTIVITY |  | 69 | -0.25 | -0.96 | 0.524 | 0.931 | 1.000 | 2214 | tags=19%, list=18%, signal=23% |
| 2198 | GOBP\_B\_CELL\_DIFFERENTIATION |  | 83 | -0.24 | -0.96 | 0.524 | 0.931 | 1.000 | 2483 | tags=31%, list=21%, signal=39% |
| 2199 | GOBP\_PROSTATE\_GLAND\_GROWTH |  | 9 | -0.41 | -0.96 | 0.509 | 0.930 | 1.000 | 2842 | tags=44%, list=24%, signal=58% |
| 2200 | GOBP\_REGULATION\_OF\_BONE\_DEVELOPMENT |  | 5 | -0.49 | -0.96 | 0.526 | 0.930 | 1.000 | 448 | tags=20%, list=4%, signal=21% |
| 2201 | GOBP\_NORADRENERGIC\_NEURON\_DIFFERENTIATION |  | 4 | -0.52 | -0.96 | 0.534 | 0.931 | 1.000 | 1903 | tags=75%, list=16%, signal=89% |
| 2202 | GOBP\_NEURON\_DIFFERENTIATION |  | 913 | -0.18 | -0.96 | 0.708 | 0.931 | 1.000 | 2597 | tags=23%, list=22%, signal=28% |
| 2203 | GOBP\_HIGH\_DENSITY\_LIPOPROTEIN\_PARTICLE\_REMODELING |  | 9 | -0.40 | -0.96 | 0.508 | 0.932 | 1.000 | 1384 | tags=33%, list=12%, signal=38% |
| 2204 | GOMF\_CONNEXIN\_BINDING |  | 4 | -0.52 | -0.96 | 0.540 | 0.932 | 1.000 | 1748 | tags=50%, list=15%, signal=58% |
| 2205 | GOBP\_ETHANOLAMINE\_CONTAINING\_COMPOUND\_METABOLIC\_PROCESS |  | 6 | -0.46 | -0.96 | 0.512 | 0.932 | 1.000 | 1232 | tags=50%, list=10%, signal=56% |
| 2206 | GOBP\_ANIMAL\_ORGAN\_FORMATION |  | 41 | -0.27 | -0.96 | 0.515 | 0.931 | 1.000 | 1656 | tags=24%, list=14%, signal=28% |
| 2207 | GOBP\_NEGATIVE\_REGULATION\_OF\_STRESS\_ACTIVATED\_PROTEIN\_KINASE\_SIGNALING\_CASCADE |  | 32 | -0.29 | -0.96 | 0.512 | 0.931 | 1.000 | 629 | tags=13%, list=5%, signal=13% |
| 2208 | GOBP\_NUCLEAR\_BODY\_ORGANIZATION |  | 10 | -0.39 | -0.96 | 0.512 | 0.931 | 1.000 | 3863 | tags=60%, list=32%, signal=88% |
| 2209 | GOBP\_GAP\_JUNCTION\_ASSEMBLY |  | 10 | -0.39 | -0.96 | 0.512 | 0.931 | 1.000 | 1424 | tags=30%, list=12%, signal=34% |
| 2210 | GOBP\_ADAPTIVE\_IMMUNE\_RESPONSE |  | 217 | -0.21 | -0.96 | 0.575 | 0.931 | 1.000 | 3223 | tags=39%, list=27%, signal=52% |
| 2211 | GOBP\_MULTICELLULAR\_ORGANISMAL\_RESPONSE\_TO\_STRESS |  | 44 | -0.27 | -0.96 | 0.504 | 0.930 | 1.000 | 1691 | tags=18%, list=14%, signal=21% |
| 2212 | GOBP\_NEURAL\_CREST\_CELL\_MIGRATION |  | 42 | -0.27 | -0.96 | 0.509 | 0.930 | 1.000 | 674 | tags=17%, list=6%, signal=18% |
| 2213 | GOBP\_FOLIC\_ACID\_CONTAINING\_COMPOUND\_BIOSYNTHETIC\_PROCESS |  | 8 | -0.43 | -0.96 | 0.504 | 0.930 | 1.000 | 349 | tags=25%, list=3%, signal=26% |
| 2214 | GOBP\_MEMBRANE\_DEPOLARIZATION\_DURING\_ACTION\_POTENTIAL |  | 20 | -0.33 | -0.96 | 0.519 | 0.932 | 1.000 | 1316 | tags=20%, list=11%, signal=22% |
| 2215 | GOBP\_POSITIVE\_REGULATION\_OF\_MYOBLAST\_FUSION |  | 7 | -0.44 | -0.96 | 0.524 | 0.932 | 1.000 | 199 | tags=14%, list=2%, signal=15% |
| 2216 | GOMF\_DNA\_DIRECTED\_DNA\_POLYMERASE\_ACTIVITY |  | 17 | -0.34 | -0.96 | 0.512 | 0.931 | 1.000 | 931 | tags=18%, list=8%, signal=19% |
| 2217 | GOBP\_PROTEIN\_LOCALIZATION\_TO\_NON\_MOTILE\_CILIUM |  | 6 | -0.46 | -0.96 | 0.518 | 0.931 | 1.000 | 2388 | tags=50%, list=20%, signal=62% |
| 2218 | GOMF\_INTERLEUKIN\_1\_RECEPTOR\_ACTIVITY |  | 7 | -0.44 | -0.96 | 0.526 | 0.931 | 1.000 | 5532 | tags=86%, list=46%, signal=159% |
| 2219 | GOBP\_POSITIVE\_REGULATION\_OF\_CELL\_DEVELOPMENT |  | 208 | -0.21 | -0.96 | 0.576 | 0.930 | 1.000 | 2127 | tags=20%, list=18%, signal=24% |
| 2220 | GOBP\_REGULATION\_OF\_MESODERMAL\_CELL\_DIFFERENTIATION |  | 3 | -0.57 | -0.96 | 0.571 | 0.931 | 1.000 | 1424 | tags=67%, list=12%, signal=76% |
| 2221 | GOBP\_POSITIVE\_REGULATION\_OF\_SMOOTH\_MUSCLE\_CELL\_DIFFERENTIATION |  | 10 | -0.39 | -0.96 | 0.510 | 0.931 | 1.000 | 2144 | tags=30%, list=18%, signal=36% |
| 2222 | GOBP\_REGULATION\_OF\_PEPTIDYL\_CYSTEINE\_S\_NITROSYLATION |  | 8 | -0.42 | -0.96 | 0.510 | 0.932 | 1.000 | 1290 | tags=25%, list=11%, signal=28% |
| 2223 | GOBP\_POSITIVE\_REGULATION\_OF\_MONOCYTE\_DIFFERENTIATION |  | 9 | -0.40 | -0.96 | 0.515 | 0.932 | 1.000 | 2986 | tags=56%, list=25%, signal=74% |
| 2224 | GOBP\_T\_HELPER\_17\_CELL\_LINEAGE\_COMMITMENT |  | 7 | -0.44 | -0.96 | 0.515 | 0.932 | 1.000 | 2907 | tags=57%, list=24%, signal=75% |
| 2225 | GOBP\_REGULATION\_OF\_ANION\_CHANNEL\_ACTIVITY |  | 5 | -0.49 | -0.96 | 0.528 | 0.932 | 1.000 | 254 | tags=20%, list=2%, signal=20% |
| 2226 | GOCC\_MEDIATOR\_COMPLEX |  | 33 | -0.29 | -0.96 | 0.522 | 0.932 | 1.000 | 4706 | tags=52%, list=39%, signal=84% |
| 2227 | GOBP\_TETRAHYDROFOLATE\_INTERCONVERSION |  | 9 | -0.41 | -0.96 | 0.516 | 0.931 | 1.000 | 532 | tags=22%, list=4%, signal=23% |
| 2228 | GOMF\_TRANSFERRIN\_RECEPTOR\_BINDING |  | 8 | -0.42 | -0.96 | 0.517 | 0.931 | 1.000 | 3151 | tags=50%, list=26%, signal=68% |
| 2229 | GOBP\_MODULATION\_OF\_PROCESS\_OF\_OTHER\_ORGANISM\_INVOLVED\_IN\_SYMBIOTIC\_INTERACTION |  | 72 | -0.24 | -0.96 | 0.534 | 0.931 | 1.000 | 2541 | tags=29%, list=21%, signal=37% |
| 2230 | GOBP\_AXIS\_SPECIFICATION |  | 46 | -0.26 | -0.96 | 0.528 | 0.931 | 1.000 | 960 | tags=17%, list=8%, signal=19% |
| 2231 | GOBP\_REGULATION\_OF\_RNA\_POLYMERASE\_II\_REGULATORY\_REGION\_SEQUENCE\_SPECIFIC\_DNA\_BINDING |  | 12 | -0.38 | -0.96 | 0.505 | 0.931 | 1.000 | 720 | tags=17%, list=6%, signal=18% |
| 2232 | GOMF\_TITIN\_BINDING |  | 6 | -0.46 | -0.96 | 0.526 | 0.931 | 1.000 | 3195 | tags=67%, list=27%, signal=91% |
| 2233 | GOMF\_CIS\_TRANS\_ISOMERASE\_ACTIVITY |  | 31 | -0.29 | -0.96 | 0.519 | 0.930 | 1.000 | 1924 | tags=23%, list=16%, signal=27% |
| 2234 | GOMF\_PURINE\_RIBONUCLEOTIDE\_TRANSMEMBRANE\_TRANSPORTER\_ACTIVITY |  | 12 | -0.38 | -0.96 | 0.522 | 0.930 | 1.000 | 2911 | tags=50%, list=24%, signal=66% |
| 2235 | GOMF\_REPRESSING\_TRANSCRIPTION\_FACTOR\_BINDING |  | 57 | -0.25 | -0.96 | 0.523 | 0.931 | 1.000 | 2587 | tags=32%, list=22%, signal=40% |
| 2236 | GOBP\_STRIATED\_MUSCLE\_CELL\_APOPTOTIC\_PROCESS |  | 21 | -0.32 | -0.96 | 0.518 | 0.931 | 1.000 | 629 | tags=19%, list=5%, signal=20% |
| 2237 | GOMF\_SNRNA\_BINDING |  | 33 | -0.29 | -0.96 | 0.525 | 0.930 | 1.000 | 1841 | tags=24%, list=15%, signal=29% |
| 2238 | GOBP\_HEPARAN\_SULFATE\_PROTEOGLYCAN\_BIOSYNTHETIC\_PROCESS\_POLYSACCHARIDE\_CHAIN\_BIOSYNTHETIC\_PROCESS |  | 7 | -0.44 | -0.96 | 0.508 | 0.932 | 1.000 | 2594 | tags=43%, list=22%, signal=55% |
| 2239 | GOBP\_POSITIVE\_REGULATION\_OF\_OSTEOBLAST\_PROLIFERATION |  | 6 | -0.46 | -0.96 | 0.520 | 0.931 | 1.000 | 141 | tags=17%, list=1%, signal=17% |
| 2240 | GOMF\_CYTIDINE\_DEAMINASE\_ACTIVITY |  | 4 | -0.52 | -0.96 | 0.544 | 0.932 | 1.000 | 2022 | tags=50%, list=17%, signal=60% |
| 2241 | GOBP\_NEGATIVE\_REGULATION\_OF\_TRANSMEMBRANE\_RECEPTOR\_PROTEIN\_SERINE\_THREONINE\_KINASE\_SIGNALING\_PATHWAY |  | 87 | -0.23 | -0.96 | 0.552 | 0.932 | 1.000 | 2480 | tags=29%, list=21%, signal=36% |
| 2242 | GOBP\_POSITIVE\_REGULATION\_OF\_MEMBRANE\_PERMEABILITY |  | 45 | -0.27 | -0.96 | 0.525 | 0.932 | 1.000 | 1430 | tags=18%, list=12%, signal=20% |
| 2243 | GOBP\_POSITIVE\_REGULATION\_OF\_VASCULAR\_ENDOTHELIAL\_CELL\_PROLIFERATION |  | 12 | -0.37 | -0.96 | 0.521 | 0.932 | 1.000 | 2783 | tags=33%, list=23%, signal=43% |
| 2244 | GOBP\_CARTILAGE\_MORPHOGENESIS |  | 5 | -0.48 | -0.96 | 0.534 | 0.932 | 1.000 | 563 | tags=40%, list=5%, signal=42% |
| 2245 | GOBP\_SYNAPTIC\_VESICLE\_PRIMING |  | 15 | -0.35 | -0.96 | 0.515 | 0.932 | 1.000 | 1353 | tags=27%, list=11%, signal=30% |
| 2246 | GOBP\_LATE\_NUCLEOPHAGY |  | 3 | -0.56 | -0.96 | 0.562 | 0.932 | 1.000 | 4148 | tags=33%, list=35%, signal=51% |
| 2247 | GOBP\_RESPONSE\_TO\_ESTRADIOL |  | 88 | -0.23 | -0.96 | 0.548 | 0.932 | 1.000 | 2789 | tags=32%, list=23%, signal=41% |
| 2248 | GOBP\_GLUCOCORTICOID\_SECRETION |  | 5 | -0.48 | -0.96 | 0.533 | 0.933 | 1.000 | 12 | tags=20%, list=0%, signal=20% |
| 2249 | GOMF\_CALCIUM\_INDEPENDENT\_PHOSPHOLIPASE\_A2\_ACTIVITY |  | 7 | -0.44 | -0.96 | 0.525 | 0.933 | 1.000 | 808 | tags=43%, list=7%, signal=46% |
| 2250 | GOBP\_REGULATION\_OF\_OSTEOBLAST\_DIFFERENTIATION |  | 82 | -0.24 | -0.95 | 0.553 | 0.933 | 1.000 | 2558 | tags=24%, list=21%, signal=31% |
| 2251 | GOBP\_NEGATIVE\_REGULATION\_OF\_ENDOTHELIAL\_CELL\_CHEMOTAXIS |  | 3 | -0.56 | -0.95 | 0.578 | 0.934 | 1.000 | 751 | tags=33%, list=6%, signal=36% |
| 2252 | GOMF\_INTRAMOLECULAR\_OXIDOREDUCTASE\_ACTIVITY |  | 38 | -0.28 | -0.95 | 0.523 | 0.934 | 1.000 | 1831 | tags=29%, list=15%, signal=34% |
| 2253 | GOBP\_CEREBELLAR\_GRANULAR\_LAYER\_FORMATION |  | 5 | -0.48 | -0.95 | 0.537 | 0.935 | 1.000 | 527 | tags=20%, list=4%, signal=21% |
| 2254 | GOCC\_NUCLEAR\_CYCLIN\_DEPENDENT\_PROTEIN\_KINASE\_HOLOENZYME\_COMPLEX |  | 12 | -0.37 | -0.95 | 0.514 | 0.935 | 1.000 | 7574 | tags=100%, list=63%, signal=270% |
| 2255 | GOCC\_CELL\_BODY\_FIBER |  | 5 | -0.48 | -0.95 | 0.531 | 0.935 | 1.000 | 731 | tags=40%, list=6%, signal=43% |
| 2256 | GOBP\_PRESYNAPSE\_ORGANIZATION |  | 28 | -0.30 | -0.95 | 0.521 | 0.934 | 1.000 | 1711 | tags=21%, list=14%, signal=25% |
| 2257 | GOCC\_PHAGOCYTIC\_VESICLE |  | 101 | -0.23 | -0.95 | 0.553 | 0.934 | 1.000 | 2055 | tags=23%, list=17%, signal=27% |
| 2258 | GOCC\_OXOGLUTARATE\_DEHYDROGENASE\_COMPLEX |  | 6 | -0.46 | -0.95 | 0.526 | 0.935 | 1.000 | 3613 | tags=67%, list=30%, signal=95% |
| 2259 | GOMF\_BIOACTIVE\_LIPID\_RECEPTOR\_ACTIVITY |  | 11 | -0.38 | -0.95 | 0.531 | 0.935 | 1.000 | 3718 | tags=45%, list=31%, signal=66% |
| 2260 | GOBP\_NEGATIVE\_REGULATION\_OF\_KINASE\_ACTIVITY |  | 188 | -0.21 | -0.95 | 0.597 | 0.935 | 1.000 | 1802 | tags=16%, list=15%, signal=19% |
| 2261 | GOBP\_NEGATIVE\_REGULATION\_OF\_GLIAL\_CELL\_PROLIFERATION |  | 10 | -0.39 | -0.95 | 0.511 | 0.934 | 1.000 | 3624 | tags=50%, list=30%, signal=72% |
| 2262 | GOBP\_L\_METHIONINE\_SALVAGE\_FROM\_METHYLTHIOADENOSINE |  | 5 | -0.48 | -0.95 | 0.542 | 0.935 | 1.000 | 821 | tags=20%, list=7%, signal=21% |
| 2263 | GOBP\_NEPHRON\_DEVELOPMENT |  | 99 | -0.23 | -0.95 | 0.565 | 0.935 | 1.000 | 2042 | tags=21%, list=17%, signal=25% |
| 2264 | GOBP\_RETINA\_HOMEOSTASIS |  | 37 | -0.28 | -0.95 | 0.540 | 0.935 | 1.000 | 3222 | tags=46%, list=27%, signal=63% |
| 2265 | GOCC\_AMINOACYL\_TRNA\_SYNTHETASE\_MULTIENZYME\_COMPLEX |  | 3 | -0.57 | -0.95 | 0.577 | 0.936 | 1.000 | 5219 | tags=100%, list=43%, signal=177% |
| 2266 | GOBP\_REGULATION\_OF\_LONG\_TERM\_NEURONAL\_SYNAPTIC\_PLASTICITY |  | 15 | -0.35 | -0.95 | 0.529 | 0.935 | 1.000 | 441 | tags=13%, list=4%, signal=14% |
| 2267 | GOBP\_SYNAPTIC\_VESICLE\_EXOCYTOSIS |  | 73 | -0.24 | -0.95 | 0.549 | 0.936 | 1.000 | 2656 | tags=33%, list=22%, signal=42% |
| 2268 | GOBP\_POSITIVE\_REGULATION\_OF\_PROTEIN\_DEPOLYMERIZATION |  | 13 | -0.36 | -0.95 | 0.514 | 0.936 | 1.000 | 1920 | tags=38%, list=16%, signal=46% |
| 2269 | GOBP\_AXON\_DEVELOPMENT |  | 365 | -0.19 | -0.95 | 0.639 | 0.936 | 1.000 | 2172 | tags=21%, list=18%, signal=25% |
| 2270 | GOCC\_CYCLIN\_CDK\_POSITIVE\_TRANSCRIPTION\_ELONGATION\_FACTOR\_COMPLEX |  | 8 | -0.42 | -0.95 | 0.520 | 0.937 | 1.000 | 4630 | tags=50%, list=39%, signal=81% |
| 2271 | GOBP\_CELLULAR\_RESPONSE\_TO\_TESTOSTERONE\_STIMULUS |  | 7 | -0.43 | -0.95 | 0.522 | 0.938 | 1.000 | 2144 | tags=57%, list=18%, signal=70% |
| 2272 | GOBP\_LABYRINTHINE\_LAYER\_DEVELOPMENT |  | 31 | -0.29 | -0.95 | 0.531 | 0.938 | 1.000 | 1380 | tags=19%, list=11%, signal=22% |
| 2273 | GOCC\_PERINUCLEOLAR\_COMPARTMENT |  | 3 | -0.57 | -0.95 | 0.582 | 0.938 | 1.000 | 3317 | tags=33%, list=28%, signal=46% |
| 2274 | GOBP\_NEURAL\_CREST\_CELL\_DIFFERENTIATION |  | 68 | -0.24 | -0.95 | 0.562 | 0.938 | 1.000 | 715 | tags=13%, list=6%, signal=14% |
| 2275 | GOBP\_ENDOTHELIAL\_CELL\_ACTIVATION |  | 7 | -0.43 | -0.95 | 0.517 | 0.939 | 1.000 | 144 | tags=14%, list=1%, signal=14% |
| 2276 | GOBP\_CELL\_ACTIVATION\_INVOLVED\_IN\_IMMUNE\_RESPONSE |  | 460 | -0.19 | -0.95 | 0.685 | 0.939 | 1.000 | 2766 | tags=27%, list=23%, signal=33% |
| 2277 | GOMF\_LYSINE\_ACETYLATED\_HISTONE\_BINDING |  | 14 | -0.35 | -0.95 | 0.522 | 0.938 | 1.000 | 86 | tags=7%, list=1%, signal=7% |
| 2278 | GOBP\_MACROPHAGE\_PROLIFERATION |  | 7 | -0.43 | -0.95 | 0.530 | 0.938 | 1.000 | 964 | tags=29%, list=8%, signal=31% |
| 2279 | GOMF\_METHYL\_CPG\_BINDING |  | 15 | -0.34 | -0.95 | 0.514 | 0.939 | 1.000 | 3214 | tags=53%, list=27%, signal=73% |
| 2280 | GOBP\_CEREBRAL\_CORTEX\_TANGENTIAL\_MIGRATION |  | 5 | -0.48 | -0.95 | 0.537 | 0.939 | 1.000 | 197 | tags=20%, list=2%, signal=20% |
| 2281 | GOBP\_NEGATIVE\_REGULATION\_OF\_CALCIUM\_ION\_DEPENDENT\_EXOCYTOSIS |  | 8 | -0.42 | -0.95 | 0.526 | 0.939 | 1.000 | 424 | tags=13%, list=4%, signal=13% |
| 2282 | GOBP\_NEGATIVE\_REGULATION\_OF\_CYSTEINE\_TYPE\_ENDOPEPTIDASE\_ACTIVITY |  | 53 | -0.25 | -0.95 | 0.547 | 0.939 | 1.000 | 1173 | tags=19%, list=10%, signal=21% |
| 2283 | GOBP\_REGULATION\_OF\_LYMPHOCYTE\_MEDIATED\_IMMUNITY |  | 74 | -0.24 | -0.95 | 0.582 | 0.940 | 1.000 | 3209 | tags=39%, list=27%, signal=53% |
| 2284 | GOMF\_RETINOIC\_ACID\_BINDING |  | 9 | -0.40 | -0.95 | 0.523 | 0.940 | 1.000 | 105 | tags=22%, list=1%, signal=22% |
| 2285 | GOBP\_MODIFIED\_AMINO\_ACID\_TRANSPORT |  | 20 | -0.32 | -0.95 | 0.530 | 0.940 | 1.000 | 2274 | tags=30%, list=19%, signal=37% |
| 2286 | GOCC\_BASAL\_CORTEX |  | 5 | -0.48 | -0.95 | 0.545 | 0.940 | 1.000 | 4056 | tags=60%, list=34%, signal=91% |
| 2287 | GOBP\_REGULATION\_OF\_MICROTUBULE\_MOTOR\_ACTIVITY |  | 8 | -0.42 | -0.95 | 0.527 | 0.940 | 1.000 | 72 | tags=13%, list=1%, signal=13% |
| 2288 | GOBP\_CELLULAR\_RESPONSE\_TO\_PURINE\_CONTAINING\_COMPOUND |  | 10 | -0.39 | -0.95 | 0.523 | 0.940 | 1.000 | 2804 | tags=40%, list=23%, signal=52% |
| 2289 | GOBP\_TRANSCRIPTION\_INITIATION\_FROM\_RNA\_POLYMERASE\_III\_PROMOTER |  | 7 | -0.43 | -0.95 | 0.534 | 0.940 | 1.000 | 4584 | tags=71%, list=38%, signal=115% |
| 2290 | GOMF\_HYALURONONGLUCOSAMINIDASE\_ACTIVITY |  | 4 | -0.52 | -0.95 | 0.556 | 0.941 | 1.000 | 1699 | tags=50%, list=14%, signal=58% |
| 2291 | GOBP\_NEGATIVE\_REGULATION\_OF\_INTRACELLULAR\_SIGNAL\_TRANSDUCTION |  | 357 | -0.19 | -0.95 | 0.691 | 0.943 | 1.000 | 1921 | tags=18%, list=16%, signal=21% |
| 2292 | GOBP\_POSITIVE\_REGULATION\_OF\_VASCULAR\_ASSOCIATED\_SMOOTH\_MUSCLE\_CELL\_APOPTOTIC\_PROCESS |  | 7 | -0.43 | -0.95 | 0.538 | 0.943 | 1.000 | 173 | tags=14%, list=1%, signal=14% |
| 2293 | GOMF\_GROWTH\_FACTOR\_BINDING |  | 106 | -0.22 | -0.95 | 0.581 | 0.942 | 1.000 | 1498 | tags=14%, list=12%, signal=16% |
| 2294 | GOBP\_RECEPTOR\_INTERNALIZATION |  | 74 | -0.24 | -0.94 | 0.571 | 0.942 | 1.000 | 2129 | tags=24%, list=18%, signal=29% |
| 2295 | GOBP\_CELLULAR\_RESPONSE\_TO\_LEUCINE\_STARVATION |  | 8 | -0.42 | -0.94 | 0.522 | 0.942 | 1.000 | 2212 | tags=38%, list=18%, signal=46% |
| 2296 | GOBP\_REGULATION\_OF\_ATP\_METABOLIC\_PROCESS |  | 80 | -0.23 | -0.94 | 0.568 | 0.943 | 1.000 | 3122 | tags=36%, list=26%, signal=49% |
| 2297 | GOBP\_NUCLEOBASE\_CONTAINING\_SMALL\_MOLECULE\_INTERCONVERSION |  | 21 | -0.31 | -0.94 | 0.531 | 0.943 | 1.000 | 1117 | tags=19%, list=9%, signal=21% |
| 2298 | GOBP\_VACUOLE\_ORGANIZATION |  | 136 | -0.21 | -0.94 | 0.602 | 0.944 | 1.000 | 2846 | tags=27%, list=24%, signal=35% |
| 2299 | GOBP\_POSITIVE\_REGULATION\_OF\_GASTRULATION |  | 4 | -0.51 | -0.94 | 0.556 | 0.944 | 1.000 | 2810 | tags=50%, list=23%, signal=65% |
| 2300 | GOBP\_NEGATIVE\_REGULATION\_OF\_PROTEASOMAL\_PROTEIN\_CATABOLIC\_PROCESS |  | 36 | -0.28 | -0.94 | 0.540 | 0.944 | 1.000 | 2678 | tags=28%, list=22%, signal=36% |
| 2301 | GOCC\_M\_BAND |  | 18 | -0.33 | -0.94 | 0.542 | 0.944 | 1.000 | 626 | tags=17%, list=5%, signal=18% |
| 2302 | GOBP\_PROTEIN\_KINASE\_C\_SIGNALING |  | 16 | -0.34 | -0.94 | 0.541 | 0.944 | 1.000 | 1920 | tags=25%, list=16%, signal=30% |
| 2303 | GOBP\_REGULATION\_OF\_REACTIVE\_OXYGEN\_SPECIES\_BIOSYNTHETIC\_PROCESS |  | 59 | -0.25 | -0.94 | 0.562 | 0.944 | 1.000 | 1445 | tags=20%, list=12%, signal=23% |
| 2304 | GOBP\_ALDITOL\_BIOSYNTHETIC\_PROCESS |  | 6 | -0.45 | -0.94 | 0.536 | 0.944 | 1.000 | 44 | tags=17%, list=0%, signal=17% |
| 2305 | GOBP\_NEGATIVE\_REGULATION\_OF\_GENE\_EXPRESSION |  | 776 | -0.18 | -0.94 | 0.773 | 0.944 | 1.000 | 3161 | tags=30%, list=26%, signal=38% |
| 2306 | GOBP\_REGULATION\_OF\_STEM\_CELL\_POPULATION\_MAINTENANCE |  | 20 | -0.32 | -0.94 | 0.541 | 0.943 | 1.000 | 4515 | tags=60%, list=38%, signal=96% |
| 2307 | GOBP\_SEROTONIN\_UPTAKE |  | 9 | -0.40 | -0.94 | 0.520 | 0.944 | 1.000 | 1096 | tags=33%, list=9%, signal=37% |
| 2308 | GOCC\_HOPS\_COMPLEX |  | 11 | -0.38 | -0.94 | 0.543 | 0.944 | 1.000 | 2790 | tags=45%, list=23%, signal=59% |
| 2309 | GOBP\_BINDING\_OF\_SPERM\_TO\_ZONA\_PELLUCIDA |  | 18 | -0.33 | -0.94 | 0.535 | 0.944 | 1.000 | 3344 | tags=33%, list=28%, signal=46% |
| 2310 | GOBP\_SEGMENT\_SPECIFICATION |  | 10 | -0.38 | -0.94 | 0.534 | 0.945 | 1.000 | 677 | tags=30%, list=6%, signal=32% |
| 2311 | GOBP\_PHAGOSOME\_LYSOSOME\_FUSION |  | 8 | -0.41 | -0.94 | 0.539 | 0.944 | 1.000 | 786 | tags=25%, list=7%, signal=27% |
| 2312 | GOBP\_NEGATIVE\_REGULATION\_OF\_SMALL\_GTPASE\_MEDIATED\_SIGNAL\_TRANSDUCTION |  | 48 | -0.26 | -0.94 | 0.558 | 0.944 | 1.000 | 805 | tags=10%, list=7%, signal=11% |
| 2313 | GOBP\_POSITIVE\_REGULATION\_OF\_CELLULAR\_AMINE\_METABOLIC\_PROCESS |  | 5 | -0.48 | -0.94 | 0.551 | 0.945 | 1.000 | 2505 | tags=40%, list=21%, signal=51% |
| 2314 | GOBP\_PRIMARY\_AMINO\_COMPOUND\_BIOSYNTHETIC\_PROCESS |  | 4 | -0.51 | -0.94 | 0.566 | 0.945 | 1.000 | 193 | tags=25%, list=2%, signal=25% |
| 2315 | GOBP\_NEGATIVE\_REGULATION\_OF\_PLATELET\_DERIVED\_GROWTH\_FACTOR\_RECEPTOR\_SIGNALING\_PATHWAY |  | 11 | -0.38 | -0.94 | 0.533 | 0.945 | 1.000 | 771 | tags=18%, list=6%, signal=19% |
| 2316 | GOBP\_POSITIVE\_REGULATION\_OF\_CALCIUM\_ION\_DEPENDENT\_EXOCYTOSIS |  | 12 | -0.37 | -0.94 | 0.531 | 0.945 | 1.000 | 1353 | tags=33%, list=11%, signal=38% |
| 2317 | GOBP\_NEGATIVE\_REGULATION\_OF\_CHONDROCYTE\_PROLIFERATION |  | 3 | -0.56 | -0.94 | 0.579 | 0.945 | 1.000 | 1482 | tags=33%, list=12%, signal=38% |
| 2318 | GOMF\_RNA\_POLYMERASE\_II\_CTD\_HEPTAPEPTIDE\_REPEAT\_PHOSPHATASE\_ACTIVITY |  | 6 | -0.45 | -0.94 | 0.544 | 0.946 | 1.000 | 3175 | tags=50%, list=26%, signal=68% |
| 2319 | GOBP\_REGULATION\_OF\_SUPEROXIDE\_METABOLIC\_PROCESS |  | 20 | -0.31 | -0.94 | 0.529 | 0.946 | 1.000 | 798 | tags=25%, list=7%, signal=27% |
| 2320 | GOBP\_DERMATAN\_SULFATE\_METABOLIC\_PROCESS |  | 9 | -0.40 | -0.94 | 0.538 | 0.946 | 1.000 | 76 | tags=11%, list=1%, signal=11% |
| 2321 | GOCC\_STRIATED\_MUSCLE\_THIN\_FILAMENT |  | 12 | -0.36 | -0.94 | 0.528 | 0.946 | 1.000 | 1013 | tags=17%, list=8%, signal=18% |
| 2322 | GOBP\_NEGATIVE\_REGULATION\_OF\_B\_CELL\_ACTIVATION |  | 23 | -0.31 | -0.94 | 0.544 | 0.946 | 1.000 | 918 | tags=17%, list=8%, signal=19% |
| 2323 | GOBP\_REGULATION\_OF\_PROTEASOMAL\_UBIQUITIN\_DEPENDENT\_PROTEIN\_CATABOLIC\_PROCESS |  | 99 | -0.22 | -0.94 | 0.605 | 0.946 | 1.000 | 2783 | tags=24%, list=23%, signal=31% |
| 2324 | GOBP\_NEGATIVE\_REGULATION\_OF\_NEUROTRANSMITTER\_TRANSPORT |  | 11 | -0.38 | -0.94 | 0.533 | 0.946 | 1.000 | 2055 | tags=36%, list=17%, signal=44% |
| 2325 | GOBP\_IMMUNOGLOBULIN\_PRODUCTION\_INVOLVED\_IN\_IMMUNOGLOBULIN\_MEDIATED\_IMMUNE\_RESPONSE |  | 37 | -0.27 | -0.94 | 0.565 | 0.946 | 1.000 | 3209 | tags=46%, list=27%, signal=63% |
| 2326 | GOBP\_NEGATIVE\_REGULATION\_OF\_RNA\_POLYMERASE\_II\_REGULATORY\_REGION\_SEQUENCE\_SPECIFIC\_DNA\_BINDING |  | 6 | -0.45 | -0.94 | 0.540 | 0.947 | 1.000 | 720 | tags=33%, list=6%, signal=35% |
| 2327 | GOBP\_REGULATION\_OF\_ADIPONECTIN\_SECRETION |  | 4 | -0.50 | -0.94 | 0.555 | 0.947 | 1.000 | 2390 | tags=75%, list=20%, signal=94% |
| 2328 | GOBP\_SYNAPSE\_ORGANIZATION |  | 264 | -0.20 | -0.94 | 0.667 | 0.946 | 1.000 | 2575 | tags=24%, list=21%, signal=30% |
| 2329 | GOBP\_CELLULAR\_RESPONSE\_TO\_X\_RAY |  | 9 | -0.40 | -0.94 | 0.553 | 0.946 | 1.000 | 629 | tags=22%, list=5%, signal=23% |
| 2330 | GOMF\_LIPOPOLYSACCHARIDE\_IMMUNE\_RECEPTOR\_ACTIVITY |  | 4 | -0.51 | -0.94 | 0.560 | 0.946 | 1.000 | 4929 | tags=50%, list=41%, signal=85% |
| 2331 | GOBP\_REGULATION\_OF\_RAC\_PROTEIN\_SIGNAL\_TRANSDUCTION |  | 16 | -0.34 | -0.94 | 0.540 | 0.946 | 1.000 | 805 | tags=19%, list=7%, signal=20% |
| 2332 | GOBP\_RESPONSE\_TO\_ACETYLCHOLINE |  | 20 | -0.32 | -0.94 | 0.544 | 0.946 | 1.000 | 2530 | tags=30%, list=21%, signal=38% |
| 2333 | GOBP\_REGULATION\_OF\_DOPAMINE\_RECEPTOR\_SIGNALING\_PATHWAY |  | 7 | -0.43 | -0.94 | 0.542 | 0.946 | 1.000 | 2181 | tags=43%, list=18%, signal=52% |
| 2334 | GOBP\_BROWN\_FAT\_CELL\_DIFFERENTIATION |  | 33 | -0.28 | -0.94 | 0.558 | 0.946 | 1.000 | 3326 | tags=39%, list=28%, signal=54% |
| 2335 | GOBP\_POSITIVE\_REGULATION\_OF\_ISOTYPE\_SWITCHING\_TO\_IGG\_ISOTYPES |  | 7 | -0.43 | -0.94 | 0.548 | 0.945 | 1.000 | 3133 | tags=71%, list=26%, signal=97% |
| 2336 | GOBP\_CHAPERONE\_MEDIATED\_PROTEIN\_COMPLEX\_ASSEMBLY |  | 16 | -0.34 | -0.94 | 0.534 | 0.945 | 1.000 | 5172 | tags=75%, list=43%, signal=132% |
| 2337 | GOBP\_VITAMIN\_D3\_METABOLIC\_PROCESS |  | 3 | -0.55 | -0.94 | 0.595 | 0.945 | 1.000 | 2410 | tags=67%, list=20%, signal=83% |
| 2338 | GOBP\_BEHAVIORAL\_RESPONSE\_TO\_NICOTINE |  | 4 | -0.51 | -0.94 | 0.559 | 0.945 | 1.000 | 1245 | tags=50%, list=10%, signal=56% |
| 2339 | GOMF\_OXYGEN\_CARRIER\_ACTIVITY |  | 4 | -0.51 | -0.94 | 0.559 | 0.945 | 1.000 | 484 | tags=25%, list=4%, signal=26% |
| 2340 | GOBP\_REGULATION\_OF\_TRANSCRIPTION\_BY\_RNA\_POLYMERASE\_III |  | 17 | -0.33 | -0.94 | 0.536 | 0.945 | 1.000 | 3014 | tags=47%, list=25%, signal=63% |
| 2341 | GOBP\_MEMBRANE\_PROTEIN\_INTRACELLULAR\_DOMAIN\_PROTEOLYSIS |  | 18 | -0.33 | -0.94 | 0.536 | 0.944 | 1.000 | 3125 | tags=39%, list=26%, signal=52% |
| 2342 | GOBP\_POSITIVE\_REGULATION\_OF\_LEUKOCYTE\_DEGRANULATION |  | 13 | -0.36 | -0.94 | 0.541 | 0.944 | 1.000 | 4293 | tags=62%, list=36%, signal=96% |
| 2343 | GOBP\_PROTEIN\_K63\_LINKED\_UBIQUITINATION |  | 41 | -0.27 | -0.94 | 0.564 | 0.945 | 1.000 | 2739 | tags=24%, list=23%, signal=31% |
| 2344 | GOBP\_PROSTATE\_GLANDULAR\_ACINUS\_MORPHOGENESIS |  | 5 | -0.48 | -0.94 | 0.556 | 0.945 | 1.000 | 166 | tags=20%, list=1%, signal=20% |
| 2345 | GOMF\_OXIDOREDUCTASE\_ACTIVITY\_ACTING\_ON\_THE\_CH\_NH\_GROUP\_OF\_DONORS\_NAD\_OR\_NADP\_AS\_ACCEPTOR |  | 12 | -0.36 | -0.94 | 0.540 | 0.945 | 1.000 | 532 | tags=17%, list=4%, signal=17% |
| 2346 | GOBP\_NEURON\_APOPTOTIC\_PROCESS |  | 144 | -0.21 | -0.94 | 0.636 | 0.946 | 1.000 | 1430 | tags=15%, list=12%, signal=16% |
| 2347 | GOBP\_REGULATION\_OF\_LEUKOCYTE\_MEDIATED\_IMMUNITY |  | 106 | -0.22 | -0.94 | 0.605 | 0.946 | 1.000 | 3209 | tags=38%, list=27%, signal=51% |
| 2348 | GOBP\_REGULATION\_OF\_MITOCHONDRIAL\_GENE\_EXPRESSION |  | 23 | -0.31 | -0.94 | 0.551 | 0.946 | 1.000 | 2147 | tags=26%, list=18%, signal=32% |
| 2349 | GOBP\_DEVELOPMENT\_OF\_SECONDARY\_FEMALE\_SEXUAL\_CHARACTERISTICS |  | 3 | -0.55 | -0.94 | 0.598 | 0.946 | 1.000 | 2452 | tags=67%, list=20%, signal=84% |
| 2350 | GOCC\_NEUROMUSCULAR\_JUNCTION |  | 49 | -0.25 | -0.94 | 0.579 | 0.946 | 1.000 | 441 | tags=8%, list=4%, signal=8% |
| 2351 | GOBP\_COBALT\_ION\_TRANSPORT |  | 5 | -0.47 | -0.93 | 0.552 | 0.947 | 1.000 | 3884 | tags=40%, list=32%, signal=59% |
| 2352 | GOBP\_CELL\_CELL\_RECOGNITION |  | 30 | -0.29 | -0.93 | 0.561 | 0.946 | 1.000 | 3344 | tags=40%, list=28%, signal=55% |
| 2353 | GOBP\_REGULATION\_OF\_SYNAPTIC\_TRANSMISSION\_DOPAMINERGIC |  | 4 | -0.51 | -0.93 | 0.575 | 0.947 | 1.000 | 1644 | tags=50%, list=14%, signal=58% |
| 2354 | GOBP\_NEGATIVE\_REGULATION\_OF\_LAMELLIPODIUM\_ORGANIZATION |  | 3 | -0.56 | -0.93 | 0.597 | 0.947 | 1.000 | 860 | tags=33%, list=7%, signal=36% |
| 2355 | GOBP\_NCRNA\_EXPORT\_FROM\_NUCLEUS |  | 33 | -0.28 | -0.93 | 0.555 | 0.946 | 1.000 | 3998 | tags=42%, list=33%, signal=63% |
| 2356 | GOBP\_NEGATIVE\_REGULATION\_OF\_OXIDATIVE\_PHOSPHORYLATION |  | 5 | -0.48 | -0.93 | 0.557 | 0.947 | 1.000 | 3284 | tags=80%, list=27%, signal=110% |
| 2357 | GOBP\_DEFENSE\_RESPONSE\_TO\_GRAM\_NEGATIVE\_BACTERIUM |  | 25 | -0.30 | -0.93 | 0.554 | 0.947 | 1.000 | 3222 | tags=36%, list=27%, signal=49% |
| 2358 | GOBP\_SPERMATID\_NUCLEUS\_DIFFERENTIATION |  | 11 | -0.37 | -0.93 | 0.546 | 0.947 | 1.000 | 4062 | tags=73%, list=34%, signal=110% |
| 2359 | GOBP\_NEGATIVE\_REGULATION\_OF\_PEPTIDE\_HORMONE\_SECRETION |  | 24 | -0.30 | -0.93 | 0.548 | 0.946 | 1.000 | 662 | tags=17%, list=6%, signal=18% |
| 2360 | GOBP\_REGULATION\_OF\_CELL\_SIZE |  | 122 | -0.22 | -0.93 | 0.600 | 0.946 | 1.000 | 261 | tags=7%, list=2%, signal=7% |
| 2361 | GOBP\_STABILIZATION\_OF\_MEMBRANE\_POTENTIAL |  | 8 | -0.41 | -0.93 | 0.542 | 0.946 | 1.000 | 1578 | tags=50%, list=13%, signal=58% |
| 2362 | GOMF\_FATTY\_ACID\_BINDING |  | 22 | -0.31 | -0.93 | 0.553 | 0.946 | 1.000 | 1341 | tags=27%, list=11%, signal=31% |
| 2363 | GOMF\_NUCLEOSIDE\_KINASE\_ACTIVITY |  | 9 | -0.40 | -0.93 | 0.546 | 0.946 | 1.000 | 1302 | tags=33%, list=11%, signal=37% |
| 2364 | GOBP\_PROTEIN\_STABILIZATION |  | 136 | -0.21 | -0.93 | 0.623 | 0.945 | 1.000 | 3391 | tags=30%, list=28%, signal=42% |
| 2365 | GOMF\_METALLOENDOPEPTIDASE\_INHIBITOR\_ACTIVITY |  | 9 | -0.39 | -0.93 | 0.534 | 0.946 | 1.000 | 1202 | tags=22%, list=10%, signal=25% |
| 2366 | GOBP\_RECEPTOR\_CLUSTERING |  | 35 | -0.28 | -0.93 | 0.562 | 0.946 | 1.000 | 3477 | tags=49%, list=29%, signal=68% |
| 2367 | GOBP\_CELLULAR\_MODIFIED\_AMINO\_ACID\_CATABOLIC\_PROCESS |  | 19 | -0.32 | -0.93 | 0.550 | 0.946 | 1.000 | 1109 | tags=16%, list=9%, signal=17% |
| 2368 | GOBP\_COCHLEA\_MORPHOGENESIS |  | 9 | -0.40 | -0.93 | 0.546 | 0.946 | 1.000 | 677 | tags=11%, list=6%, signal=12% |
| 2369 | GOBP\_DENSE\_CORE\_GRANULE\_EXOCYTOSIS |  | 8 | -0.41 | -0.93 | 0.545 | 0.946 | 1.000 | 1353 | tags=38%, list=11%, signal=42% |
| 2370 | GOBP\_POSITIVE\_REGULATION\_OF\_OSTEOBLAST\_DIFFERENTIATION |  | 41 | -0.26 | -0.93 | 0.589 | 0.946 | 1.000 | 1051 | tags=15%, list=9%, signal=16% |
| 2371 | GOBP\_NEGATIVE\_REGULATION\_OF\_INSULIN\_SECRETION\_INVOLVED\_IN\_CELLULAR\_RESPONSE\_TO\_GLUCOSE\_STIMULUS |  | 5 | -0.47 | -0.93 | 0.560 | 0.948 | 1.000 | 88 | tags=20%, list=1%, signal=20% |
| 2372 | GOMF\_SIALIC\_ACID\_BINDING |  | 5 | -0.47 | -0.93 | 0.560 | 0.949 | 1.000 | 2870 | tags=60%, list=24%, signal=79% |
| 2373 | GOBP\_POSITIVE\_REGULATION\_OF\_NAD\_P\_H\_OXIDASE\_ACTIVITY |  | 5 | -0.48 | -0.93 | 0.557 | 0.949 | 1.000 | 3691 | tags=60%, list=31%, signal=87% |
| 2374 | GOMF\_AMP\_BINDING |  | 10 | -0.38 | -0.93 | 0.542 | 0.949 | 1.000 | 905 | tags=30%, list=8%, signal=32% |
| 2375 | GOBP\_NON\_CANONICAL\_WNT\_SIGNALING\_PATHWAY\_VIA\_MAPK\_CASCADE |  | 5 | -0.47 | -0.93 | 0.553 | 0.949 | 1.000 | 1391 | tags=40%, list=12%, signal=45% |
| 2376 | GOMF\_DIOXYGENASE\_ACTIVITY |  | 58 | -0.25 | -0.93 | 0.581 | 0.949 | 1.000 | 1616 | tags=24%, list=13%, signal=28% |
| 2377 | GOCC\_PHOTORECEPTOR\_RIBBON\_SYNAPSE |  | 4 | -0.50 | -0.93 | 0.576 | 0.950 | 1.000 | 3245 | tags=50%, list=27%, signal=68% |
| 2378 | GOCC\_MMXD\_COMPLEX |  | 3 | -0.55 | -0.93 | 0.594 | 0.949 | 1.000 | 5374 | tags=100%, list=45%, signal=181% |
| 2379 | GOBP\_HISTONE\_H3\_K27\_TRIMETHYLATION |  | 3 | -0.54 | -0.93 | 0.595 | 0.950 | 1.000 | 725 | tags=33%, list=6%, signal=35% |
| 2380 | GOBP\_POSITIVE\_REGULATION\_OF\_UBIQUITIN\_PROTEIN\_TRANSFERASE\_ACTIVITY |  | 26 | -0.29 | -0.93 | 0.563 | 0.950 | 1.000 | 3161 | tags=38%, list=26%, signal=52% |
| 2381 | GOBP\_SMOOTH\_MUSCLE\_CELL\_APOPTOTIC\_PROCESS |  | 17 | -0.33 | -0.93 | 0.545 | 0.951 | 1.000 | 3506 | tags=35%, list=29%, signal=50% |
| 2382 | GOMF\_TRANSMEMBRANE\_RECEPTOR\_PROTEIN\_TYROSINE\_KINASE\_ACTIVITY |  | 50 | -0.25 | -0.93 | 0.578 | 0.951 | 1.000 | 2667 | tags=28%, list=22%, signal=36% |
| 2383 | GOBP\_REGULATION\_OF\_HORMONE\_BIOSYNTHETIC\_PROCESS |  | 17 | -0.33 | -0.93 | 0.552 | 0.951 | 1.000 | 1418 | tags=24%, list=12%, signal=27% |
| 2384 | GOBP\_REGULATION\_OF\_NEURONAL\_SYNAPTIC\_PLASTICITY |  | 25 | -0.30 | -0.93 | 0.569 | 0.951 | 1.000 | 1259 | tags=16%, list=10%, signal=18% |
| 2385 | GOMF\_GLIAL\_CELL\_DERIVED\_NEUROTROPHIC\_FACTOR\_RECEPTOR\_ACTIVITY |  | 4 | -0.51 | -0.93 | 0.580 | 0.951 | 1.000 | 1158 | tags=25%, list=10%, signal=28% |
| 2386 | GOBP\_HEPARIN\_METABOLIC\_PROCESS |  | 11 | -0.37 | -0.93 | 0.552 | 0.951 | 1.000 | 3232 | tags=45%, list=27%, signal=62% |
| 2387 | GOMF\_NUCLEAR\_RECEPTOR\_COACTIVATOR\_ACTIVITY |  | 42 | -0.26 | -0.93 | 0.586 | 0.951 | 1.000 | 1412 | tags=19%, list=12%, signal=22% |
| 2388 | GOBP\_PRO\_B\_CELL\_DIFFERENTIATION |  | 11 | -0.37 | -0.93 | 0.542 | 0.951 | 1.000 | 3624 | tags=73%, list=30%, signal=104% |
| 2389 | GOBP\_HEMATOPOIETIC\_STEM\_CELL\_HOMEOSTASIS |  | 10 | -0.38 | -0.93 | 0.553 | 0.952 | 1.000 | 1903 | tags=40%, list=16%, signal=47% |
| 2390 | GOBP\_REGULATION\_OF\_SYNAPSE\_MATURATION |  | 10 | -0.38 | -0.93 | 0.555 | 0.952 | 1.000 | 2822 | tags=40%, list=23%, signal=52% |
| 2391 | GOMF\_NADPLUS\_BINDING |  | 12 | -0.36 | -0.93 | 0.559 | 0.952 | 1.000 | 2783 | tags=50%, list=23%, signal=65% |
| 2392 | GOBP\_GLUTAMINE\_FAMILY\_AMINO\_ACID\_BIOSYNTHETIC\_PROCESS |  | 8 | -0.40 | -0.93 | 0.560 | 0.952 | 1.000 | 1561 | tags=25%, list=13%, signal=29% |
| 2393 | GOMF\_G\_PROTEIN\_COUPLED\_GLUTAMATE\_RECEPTOR\_BINDING |  | 10 | -0.38 | -0.93 | 0.556 | 0.951 | 1.000 | 2938 | tags=30%, list=24%, signal=40% |
| 2394 | GOCC\_EXTRINSIC\_COMPONENT\_OF\_SYNAPTIC\_MEMBRANE |  | 9 | -0.39 | -0.93 | 0.556 | 0.951 | 1.000 | 3877 | tags=56%, list=32%, signal=82% |
| 2395 | GOBP\_BENZENE\_CONTAINING\_COMPOUND\_METABOLIC\_PROCESS |  | 11 | -0.37 | -0.93 | 0.539 | 0.952 | 1.000 | 968 | tags=18%, list=8%, signal=20% |
| 2396 | GOBP\_NEGATIVE\_REGULATION\_OF\_PEPTIDYL\_CYSTEINE\_S\_NITROSYLATION |  | 5 | -0.47 | -0.93 | 0.563 | 0.952 | 1.000 | 289 | tags=20%, list=2%, signal=20% |
| 2397 | GOBP\_POSITIVE\_REGULATION\_OF\_LEUKOCYTE\_CELL\_CELL\_ADHESION |  | 150 | -0.21 | -0.93 | 0.653 | 0.952 | 1.000 | 2760 | tags=32%, list=23%, signal=41% |
| 2398 | GOBP\_MYELOID\_LEUKOCYTE\_ACTIVATION |  | 427 | -0.18 | -0.93 | 0.783 | 0.952 | 1.000 | 2777 | tags=26%, list=23%, signal=33% |
| 2399 | GOBP\_IMMATURE\_T\_CELL\_PROLIFERATION\_IN\_THYMUS |  | 6 | -0.44 | -0.92 | 0.552 | 0.953 | 1.000 | 1656 | tags=33%, list=14%, signal=39% |
| 2400 | GOBP\_NEURON\_INTRINSIC\_APOPTOTIC\_SIGNALING\_PATHWAY\_IN\_RESPONSE\_TO\_OXIDATIVE\_STRESS |  | 6 | -0.44 | -0.92 | 0.559 | 0.954 | 1.000 | 4103 | tags=83%, list=34%, signal=127% |
| 2401 | GOBP\_REGULATION\_OF\_VASCULAR\_ASSOCIATED\_SMOOTH\_MUSCLE\_CELL\_DIFFERENTIATION |  | 13 | -0.35 | -0.92 | 0.564 | 0.955 | 1.000 | 133 | tags=8%, list=1%, signal=8% |
| 2402 | GOBP\_POSITIVE\_REGULATION\_OF\_CYCLIC\_NUCLEOTIDE\_PHOSPHODIESTERASE\_ACTIVITY |  | 3 | -0.55 | -0.92 | 0.613 | 0.955 | 1.000 | 458 | tags=33%, list=4%, signal=35% |
| 2403 | GOBP\_POSITIVE\_REGULATION\_OF\_TELOMERASE\_ACTIVITY |  | 27 | -0.29 | -0.92 | 0.571 | 0.955 | 1.000 | 1442 | tags=19%, list=12%, signal=21% |
| 2404 | GOBP\_HISTONE\_H3\_K36\_DEMETHYLATION |  | 6 | -0.44 | -0.92 | 0.564 | 0.955 | 1.000 | 1563 | tags=17%, list=13%, signal=19% |
| 2405 | GOBP\_REGULATION\_OF\_NON\_CANONICAL\_WNT\_SIGNALING\_PATHWAY |  | 19 | -0.32 | -0.92 | 0.563 | 0.955 | 1.000 | 2230 | tags=32%, list=19%, signal=39% |
| 2406 | GOBP\_POSITIVE\_REGULATION\_OF\_HEXOKINASE\_ACTIVITY |  | 3 | -0.55 | -0.92 | 0.614 | 0.955 | 1.000 | 5461 | tags=100%, list=45%, signal=183% |
| 2407 | GOBP\_REGULATION\_OF\_PHAGOCYTOSIS |  | 62 | -0.24 | -0.92 | 0.612 | 0.954 | 1.000 | 2055 | tags=26%, list=17%, signal=31% |
| 2408 | GOBP\_RESPONSE\_TO\_FOOD |  | 18 | -0.32 | -0.92 | 0.573 | 0.954 | 1.000 | 1052 | tags=22%, list=9%, signal=24% |
| 2409 | GOBP\_NEGATIVE\_REGULATION\_OF\_LEUKOCYTE\_MIGRATION |  | 27 | -0.29 | -0.92 | 0.576 | 0.954 | 1.000 | 2057 | tags=30%, list=17%, signal=36% |
| 2410 | GOCC\_INO80\_TYPE\_COMPLEX |  | 19 | -0.32 | -0.92 | 0.565 | 0.954 | 1.000 | 5494 | tags=63%, list=46%, signal=116% |
| 2411 | GOMF\_PROTEIN\_KINASE\_A\_REGULATORY\_SUBUNIT\_BINDING |  | 19 | -0.32 | -0.92 | 0.574 | 0.954 | 1.000 | 338 | tags=11%, list=3%, signal=11% |
| 2412 | GOBP\_ESTABLISHMENT\_OF\_PROTEIN\_LOCALIZATION\_TO\_VACUOLE |  | 41 | -0.26 | -0.92 | 0.598 | 0.954 | 1.000 | 3319 | tags=34%, list=28%, signal=47% |
| 2413 | GOBP\_NEGATIVE\_REGULATION\_OF\_NERVOUS\_SYSTEM\_PROCESS |  | 11 | -0.37 | -0.92 | 0.561 | 0.954 | 1.000 | 1650 | tags=36%, list=14%, signal=42% |
| 2414 | GOMF\_GTPASE\_ACTIVATOR\_ACTIVITY |  | 206 | -0.20 | -0.92 | 0.701 | 0.954 | 1.000 | 1221 | tags=12%, list=10%, signal=13% |
| 2415 | GOCC\_POSTSYNAPTIC\_DENSITY\_MEMBRANE |  | 39 | -0.26 | -0.92 | 0.580 | 0.953 | 1.000 | 2634 | tags=28%, list=22%, signal=36% |
| 2416 | GOCC\_SPERM\_MIDPIECE |  | 14 | -0.34 | -0.92 | 0.567 | 0.954 | 1.000 | 2561 | tags=36%, list=21%, signal=45% |
| 2417 | GOBP\_NEUROTRANSMITTER\_RECEPTOR\_TRANSPORT\_TO\_PLASMA\_MEMBRANE |  | 12 | -0.36 | -0.92 | 0.555 | 0.954 | 1.000 | 617 | tags=17%, list=5%, signal=18% |
| 2418 | GOMF\_RNA\_POLYMERASE\_III\_GENERAL\_TRANSCRIPTION\_INITIATION\_FACTOR\_ACTIVITY |  | 6 | -0.44 | -0.92 | 0.566 | 0.954 | 1.000 | 5221 | tags=83%, list=43%, signal=147% |
| 2419 | GOBP\_EXOCRINE\_SYSTEM\_DEVELOPMENT |  | 34 | -0.27 | -0.92 | 0.587 | 0.955 | 1.000 | 2040 | tags=18%, list=17%, signal=21% |
| 2420 | GOBP\_POSITIVE\_REGULATION\_OF\_NUCLEOTIDE\_BIOSYNTHETIC\_PROCESS |  | 11 | -0.37 | -0.92 | 0.550 | 0.954 | 1.000 | 1561 | tags=27%, list=13%, signal=31% |
| 2421 | GOMF\_PROTEIN\_SERINE\_THREONINE\_PHOSPHATASE\_ACTIVITY |  | 73 | -0.23 | -0.92 | 0.627 | 0.954 | 1.000 | 3532 | tags=30%, list=29%, signal=42% |
| 2422 | GOMF\_PHOSPHATIDYLINOSITOL\_N\_ACETYLGLUCOSAMINYLTRANSFERASE\_ACTIVITY |  | 5 | -0.47 | -0.92 | 0.571 | 0.954 | 1.000 | 718 | tags=40%, list=6%, signal=43% |
| 2423 | GOBP\_REGULATION\_OF\_TRANS\_SYNAPTIC\_SIGNALING |  | 259 | -0.19 | -0.92 | 0.734 | 0.954 | 1.000 | 2656 | tags=26%, list=22%, signal=33% |
| 2424 | GOBP\_TRABECULA\_FORMATION |  | 15 | -0.34 | -0.92 | 0.569 | 0.954 | 1.000 | 166 | tags=13%, list=1%, signal=14% |
| 2425 | GOCC\_CILIARY\_MEMBRANE |  | 42 | -0.26 | -0.92 | 0.600 | 0.954 | 1.000 | 1289 | tags=14%, list=11%, signal=16% |
| 2426 | GOBP\_NEGATIVE\_REGULATION\_OF\_MITOCHONDRIAL\_FUSION |  | 7 | -0.42 | -0.92 | 0.564 | 0.954 | 1.000 | 3000 | tags=57%, list=25%, signal=76% |
| 2427 | GOBP\_AMINO\_SUGAR\_METABOLIC\_PROCESS |  | 29 | -0.28 | -0.92 | 0.575 | 0.954 | 1.000 | 177 | tags=10%, list=1%, signal=10% |
| 2428 | GOBP\_REGULATION\_OF\_MYELOID\_LEUKOCYTE\_MEDIATED\_IMMUNITY |  | 31 | -0.28 | -0.92 | 0.570 | 0.955 | 1.000 | 1696 | tags=26%, list=14%, signal=30% |
| 2429 | GOBP\_POSITIVE\_REGULATION\_OF\_TRANSCRIPTION\_FROM\_RNA\_POLYMERASE\_II\_PROMOTER\_INVOLVED\_IN\_CELLULAR\_RESPONSE\_TO\_CHEMICAL\_STIMULUS |  | 17 | -0.32 | -0.92 | 0.573 | 0.955 | 1.000 | 1418 | tags=24%, list=12%, signal=27% |
| 2430 | GOBP\_ISOPRENOID\_CATABOLIC\_PROCESS |  | 6 | -0.44 | -0.92 | 0.561 | 0.954 | 1.000 | 42 | tags=17%, list=0%, signal=17% |
| 2431 | GOBP\_CALCINEURIN\_MEDIATED\_SIGNALING |  | 27 | -0.29 | -0.92 | 0.581 | 0.955 | 1.000 | 3347 | tags=37%, list=28%, signal=51% |
| 2432 | GOBP\_HEMATOPOIETIC\_STEM\_CELL\_PROLIFERATION |  | 18 | -0.32 | -0.92 | 0.576 | 0.955 | 1.000 | 1567 | tags=33%, list=13%, signal=38% |
| 2433 | GOMF\_PROTEIN\_KINASE\_A\_BINDING |  | 38 | -0.26 | -0.92 | 0.589 | 0.956 | 1.000 | 969 | tags=13%, list=8%, signal=14% |
| 2434 | GOBP\_C21\_STEROID\_HORMONE\_BIOSYNTHETIC\_PROCESS |  | 18 | -0.32 | -0.92 | 0.570 | 0.956 | 1.000 | 878 | tags=17%, list=7%, signal=18% |
| 2435 | GOBP\_COPULATION |  | 13 | -0.35 | -0.92 | 0.556 | 0.956 | 1.000 | 521 | tags=15%, list=4%, signal=16% |
| 2436 | GOBP\_LIPOSACCHARIDE\_METABOLIC\_PROCESS |  | 84 | -0.23 | -0.92 | 0.639 | 0.955 | 1.000 | 2870 | tags=35%, list=24%, signal=45% |
| 2437 | GOBP\_SYMPATHETIC\_GANGLION\_DEVELOPMENT |  | 8 | -0.40 | -0.92 | 0.564 | 0.955 | 1.000 | 640 | tags=25%, list=5%, signal=26% |
| 2438 | GOBP\_POSITIVE\_REGULATION\_OF\_BICELLULAR\_TIGHT\_JUNCTION\_ASSEMBLY |  | 6 | -0.44 | -0.92 | 0.566 | 0.955 | 1.000 | 3149 | tags=50%, list=26%, signal=68% |
| 2439 | GOBP\_CELLULAR\_MACROMOLECULE\_CATABOLIC\_PROCESS |  | 903 | -0.17 | -0.92 | 0.903 | 0.955 | 1.000 | 2713 | tags=23%, list=23%, signal=28% |
| 2440 | GOBP\_VASCULAR\_ENDOTHELIAL\_CELL\_PROLIFERATION |  | 15 | -0.34 | -0.92 | 0.576 | 0.956 | 1.000 | 3144 | tags=33%, list=26%, signal=45% |
| 2441 | GOBP\_RESPONSE\_TO\_ZINC\_ION |  | 26 | -0.29 | -0.92 | 0.583 | 0.957 | 1.000 | 1061 | tags=15%, list=9%, signal=17% |
| 2442 | GOBP\_NEGATIVE\_REGULATION\_OF\_CALCIUM\_ION\_TRANSPORT\_INTO\_CYTOSOL |  | 11 | -0.37 | -0.92 | 0.570 | 0.957 | 1.000 | 1815 | tags=27%, list=15%, signal=32% |
| 2443 | GOBP\_REGULATION\_OF\_EPITHELIAL\_CELL\_APOPTOTIC\_PROCESS |  | 55 | -0.25 | -0.92 | 0.623 | 0.957 | 1.000 | 1549 | tags=20%, list=13%, signal=23% |
| 2444 | GOCC\_BLOC\_COMPLEX |  | 18 | -0.32 | -0.92 | 0.582 | 0.957 | 1.000 | 3919 | tags=56%, list=33%, signal=82% |
| 2445 | GOBP\_PURINE\_NUCLEOSIDE\_DIPHOSPHATE\_BIOSYNTHETIC\_PROCESS |  | 5 | -0.46 | -0.92 | 0.568 | 0.957 | 1.000 | 2588 | tags=40%, list=22%, signal=51% |
| 2446 | GOMF\_CHANNEL\_INHIBITOR\_ACTIVITY |  | 27 | -0.28 | -0.92 | 0.591 | 0.957 | 1.000 | 458 | tags=11%, list=4%, signal=12% |
| 2447 | GOBP\_HISTONE\_H3\_K27\_METHYLATION |  | 11 | -0.37 | -0.92 | 0.565 | 0.957 | 1.000 | 3053 | tags=36%, list=25%, signal=49% |
| 2448 | GOBP\_NEURON\_DEATH |  | 215 | -0.19 | -0.92 | 0.725 | 0.957 | 1.000 | 2656 | tags=25%, list=22%, signal=32% |
| 2449 | GOBP\_REGULATION\_OF\_PROTEIN\_LOCALIZATION\_TO\_CELL\_SURFACE |  | 25 | -0.29 | -0.92 | 0.567 | 0.957 | 1.000 | 4 | tags=4%, list=0%, signal=4% |
| 2450 | GOBP\_MUSCLE\_CELL\_DEVELOPMENT |  | 98 | -0.22 | -0.92 | 0.647 | 0.957 | 1.000 | 2255 | tags=21%, list=19%, signal=26% |
| 2451 | GOBP\_REGULATION\_OF\_MONOOXYGENASE\_ACTIVITY |  | 39 | -0.26 | -0.91 | 0.603 | 0.958 | 1.000 | 1418 | tags=21%, list=12%, signal=23% |
| 2452 | GOBP\_REGULATION\_OF\_ATP\_BIOSYNTHETIC\_PROCESS |  | 11 | -0.36 | -0.91 | 0.563 | 0.958 | 1.000 | 2974 | tags=45%, list=25%, signal=60% |
| 2453 | GOCC\_SIDE\_OF\_MEMBRANE |  | 329 | -0.19 | -0.91 | 0.794 | 0.957 | 1.000 | 1989 | tags=21%, list=17%, signal=25% |
| 2454 | GOBP\_NEGATIVE\_REGULATION\_OF\_SYNAPTIC\_TRANSMISSION |  | 41 | -0.26 | -0.91 | 0.612 | 0.957 | 1.000 | 1691 | tags=27%, list=14%, signal=31% |
| 2455 | GOCC\_PLATELET\_DENSE\_GRANULE\_MEMBRANE |  | 4 | -0.50 | -0.91 | 0.594 | 0.957 | 1.000 | 1902 | tags=50%, list=16%, signal=59% |
| 2456 | GOBP\_PHOSPHATIDYLINOSITOL\_3\_KINASE\_SIGNALING |  | 108 | -0.21 | -0.91 | 0.652 | 0.957 | 1.000 | 2842 | tags=27%, list=24%, signal=35% |
| 2457 | GOBP\_PLASMA\_CELL\_DIFFERENTIATION |  | 4 | -0.50 | -0.91 | 0.602 | 0.957 | 1.000 | 5597 | tags=75%, list=47%, signal=140% |
| 2458 | GOBP\_BASE\_EXCISION\_REPAIR\_GAP\_FILLING |  | 5 | -0.46 | -0.91 | 0.572 | 0.957 | 1.000 | 2408 | tags=40%, list=20%, signal=50% |
| 2459 | GOBP\_DEFENSE\_RESPONSE\_TO\_FUNGUS |  | 8 | -0.40 | -0.91 | 0.568 | 0.957 | 1.000 | 2874 | tags=38%, list=24%, signal=49% |
| 2460 | GOBP\_MODULATION\_OF\_PROCESS\_OF\_OTHER\_ORGANISM |  | 77 | -0.23 | -0.91 | 0.642 | 0.957 | 1.000 | 2541 | tags=29%, list=21%, signal=36% |
| 2461 | GOBP\_SEMINIFEROUS\_TUBULE\_DEVELOPMENT |  | 9 | -0.39 | -0.91 | 0.569 | 0.958 | 1.000 | 2089 | tags=33%, list=17%, signal=40% |
| 2462 | GOBP\_POSITIVE\_REGULATION\_OF\_HEMATOPOIETIC\_PROGENITOR\_CELL\_DIFFERENTIATION |  | 4 | -0.49 | -0.91 | 0.577 | 0.958 | 1.000 | 974 | tags=25%, list=8%, signal=27% |
| 2463 | GOBP\_REVERSE\_CHOLESTEROL\_TRANSPORT |  | 8 | -0.40 | -0.91 | 0.579 | 0.958 | 1.000 | 2337 | tags=50%, list=19%, signal=62% |
| 2464 | GOBP\_DICARBOXYLIC\_ACID\_BIOSYNTHETIC\_PROCESS |  | 9 | -0.38 | -0.91 | 0.579 | 0.958 | 1.000 | 1561 | tags=22%, list=13%, signal=26% |
| 2465 | GOBP\_POSITIVE\_REGULATION\_OF\_TRANSCRIPTION\_REGULATORY\_REGION\_DNA\_BINDING |  | 17 | -0.32 | -0.91 | 0.571 | 0.960 | 1.000 | 2625 | tags=35%, list=22%, signal=45% |
| 2466 | GOBP\_TRANSCRIPTION\_BY\_RNA\_POLYMERASE\_III |  | 35 | -0.27 | -0.91 | 0.612 | 0.961 | 1.000 | 4584 | tags=54%, list=38%, signal=88% |
| 2467 | GOBP\_MEMBRANE\_DEPOLARIZATION\_DURING\_CARDIAC\_MUSCLE\_CELL\_ACTION\_POTENTIAL |  | 13 | -0.35 | -0.91 | 0.572 | 0.961 | 1.000 | 2333 | tags=38%, list=19%, signal=48% |
| 2468 | GOBP\_N\_ACETYLGLUCOSAMINE\_METABOLIC\_PROCESS |  | 14 | -0.34 | -0.91 | 0.572 | 0.961 | 1.000 | 951 | tags=21%, list=8%, signal=23% |
| 2469 | GOBP\_AMP\_METABOLIC\_PROCESS |  | 12 | -0.35 | -0.91 | 0.576 | 0.961 | 1.000 | 1117 | tags=25%, list=9%, signal=28% |
| 2470 | GOMF\_VOLTAGE\_GATED\_CALCIUM\_CHANNEL\_ACTIVITY\_INVOLVED\_IN\_REGULATION\_OF\_CYTOSOLIC\_CALCIUM\_LEVELS |  | 6 | -0.44 | -0.91 | 0.574 | 0.962 | 1.000 | 3323 | tags=67%, list=28%, signal=92% |
| 2471 | GOBP\_PROTEOGLYCAN\_BIOSYNTHETIC\_PROCESS |  | 46 | -0.25 | -0.91 | 0.631 | 0.962 | 1.000 | 2794 | tags=30%, list=23%, signal=40% |
| 2472 | GOMF\_RNA\_POLYMERASE\_I\_CORE\_BINDING |  | 3 | -0.54 | -0.91 | 0.624 | 0.961 | 1.000 | 5579 | tags=100%, list=46%, signal=187% |
| 2473 | GOBP\_RESPONSE\_TO\_PROTOZOAN |  | 9 | -0.39 | -0.91 | 0.587 | 0.961 | 1.000 | 2907 | tags=78%, list=24%, signal=103% |
| 2474 | GOMF\_HISTONE\_DEMETHYLASE\_ACTIVITY\_H3\_K36\_SPECIFIC |  | 6 | -0.44 | -0.91 | 0.578 | 0.961 | 1.000 | 1563 | tags=17%, list=13%, signal=19% |
| 2475 | GOBP\_POSITIVE\_REGULATION\_OF\_CELLULAR\_AMIDE\_METABOLIC\_PROCESS |  | 112 | -0.21 | -0.91 | 0.685 | 0.961 | 1.000 | 2555 | tags=25%, list=21%, signal=31% |
| 2476 | GOBP\_MYELOID\_LEUKOCYTE\_CYTOKINE\_PRODUCTION |  | 22 | -0.30 | -0.91 | 0.594 | 0.960 | 1.000 | 3381 | tags=41%, list=28%, signal=57% |
| 2477 | GOMF\_RECEPTOR\_SERINE\_THREONINE\_KINASE\_BINDING |  | 16 | -0.33 | -0.91 | 0.590 | 0.961 | 1.000 | 772 | tags=19%, list=6%, signal=20% |
| 2478 | GOBP\_MODULATION\_BY\_HOST\_OF\_SYMBIONT\_PROCESS |  | 45 | -0.25 | -0.91 | 0.629 | 0.963 | 1.000 | 2541 | tags=31%, list=21%, signal=39% |
| 2479 | GOMF\_BASAL\_TRANSCRIPTION\_MACHINERY\_BINDING |  | 42 | -0.26 | -0.91 | 0.614 | 0.963 | 1.000 | 4059 | tags=38%, list=34%, signal=57% |
| 2480 | GOCC\_RESPIRATORY\_CHAIN\_COMPLEX\_III |  | 9 | -0.38 | -0.91 | 0.578 | 0.963 | 1.000 | 4883 | tags=56%, list=41%, signal=94% |
| 2481 | GOBP\_MUSCLE\_CELL\_APOPTOTIC\_PROCESS |  | 41 | -0.26 | -0.91 | 0.600 | 0.964 | 1.000 | 629 | tags=12%, list=5%, signal=13% |
| 2482 | GOBP\_REGULATION\_OF\_PHOSPHOLIPASE\_C\_ACTIVITY |  | 33 | -0.27 | -0.91 | 0.599 | 0.964 | 1.000 | 846 | tags=12%, list=7%, signal=13% |
| 2483 | GOBP\_AUTOPHAGY\_OF\_PEROXISOME |  | 5 | -0.46 | -0.91 | 0.580 | 0.964 | 1.000 | 4023 | tags=60%, list=33%, signal=90% |
| 2484 | GOBP\_REGULATION\_OF\_ENDOTHELIAL\_CELL\_DIFFERENTIATION |  | 28 | -0.28 | -0.91 | 0.582 | 0.964 | 1.000 | 1409 | tags=21%, list=12%, signal=24% |
| 2485 | GOBP\_CYTIDINE\_TO\_URIDINE\_EDITING |  | 3 | -0.53 | -0.91 | 0.616 | 0.964 | 1.000 | 2022 | tags=67%, list=17%, signal=80% |
| 2486 | GOBP\_NEGATIVE\_REGULATION\_OF\_CALCIUM\_MEDIATED\_SIGNALING |  | 12 | -0.35 | -0.91 | 0.587 | 0.964 | 1.000 | 3045 | tags=50%, list=25%, signal=67% |
| 2487 | GOBP\_CALCIUM\_ION\_TRANSPORT\_INTO\_CYTOSOL |  | 95 | -0.22 | -0.91 | 0.673 | 0.965 | 1.000 | 1457 | tags=17%, list=12%, signal=19% |
| 2488 | GOBP\_REGULATION\_OF\_T\_CELL\_MEDIATED\_IMMUNE\_RESPONSE\_TO\_TUMOR\_CELL |  | 3 | -0.53 | -0.91 | 0.622 | 0.965 | 1.000 | 5595 | tags=100%, list=47%, signal=187% |
| 2489 | GOBP\_DIAPEDESIS |  | 6 | -0.43 | -0.91 | 0.578 | 0.966 | 1.000 | 2530 | tags=33%, list=21%, signal=42% |
| 2490 | GOBP\_DEFENSE\_RESPONSE\_TO\_BACTERIUM |  | 82 | -0.22 | -0.91 | 0.663 | 0.966 | 1.000 | 2209 | tags=27%, list=18%, signal=33% |
| 2491 | GOBP\_POSITIVE\_REGULATION\_OF\_ODONTOGENESIS |  | 5 | -0.46 | -0.91 | 0.587 | 0.966 | 1.000 | 156 | tags=20%, list=1%, signal=20% |
| 2492 | GOBP\_NEUROTROPHIN\_TRK\_RECEPTOR\_SIGNALING\_PATHWAY |  | 23 | -0.30 | -0.91 | 0.585 | 0.965 | 1.000 | 1334 | tags=17%, list=11%, signal=20% |
| 2493 | GOBP\_POSITIVE\_REGULATION\_OF\_INTRACELLULAR\_ESTROGEN\_RECEPTOR\_SIGNALING\_PATHWAY |  | 8 | -0.40 | -0.90 | 0.584 | 0.966 | 1.000 | 3561 | tags=75%, list=30%, signal=107% |
| 2494 | GOBP\_POSITIVE\_REGULATION\_OF\_MORPHOGENESIS\_OF\_AN\_EPITHELIUM |  | 19 | -0.31 | -0.90 | 0.605 | 0.966 | 1.000 | 2089 | tags=32%, list=17%, signal=38% |
| 2495 | GOMF\_SUMO\_LIGASE\_ACTIVITY |  | 7 | -0.41 | -0.90 | 0.582 | 0.967 | 1.000 | 1049 | tags=29%, list=9%, signal=31% |
| 2496 | GOBP\_VITAMIN\_D\_RECEPTOR\_SIGNALING\_PATHWAY |  | 8 | -0.39 | -0.90 | 0.581 | 0.967 | 1.000 | 79 | tags=13%, list=1%, signal=13% |
| 2497 | GOBP\_COVALENT\_CHROMATIN\_MODIFICATION |  | 332 | -0.18 | -0.90 | 0.816 | 0.967 | 1.000 | 3053 | tags=26%, list=25%, signal=33% |
| 2498 | GOBP\_CATECHOL\_CONTAINING\_COMPOUND\_CATABOLIC\_PROCESS |  | 5 | -0.46 | -0.90 | 0.585 | 0.966 | 1.000 | 2300 | tags=40%, list=19%, signal=49% |
| 2499 | GOBP\_POSITIVE\_REGULATION\_OF\_CHEMOKINE\_C\_C\_MOTIF\_LIGAND\_5\_PRODUCTION |  | 3 | -0.53 | -0.90 | 0.634 | 0.967 | 1.000 | 5612 | tags=100%, list=47%, signal=188% |
| 2500 | GOCC\_SPECIFIC\_GRANULE\_MEMBRANE |  | 59 | -0.24 | -0.90 | 0.660 | 0.967 | 1.000 | 777 | tags=12%, list=6%, signal=13% |
| 2501 | GOBP\_MONOSACCHARIDE\_BIOSYNTHETIC\_PROCESS |  | 70 | -0.23 | -0.90 | 0.651 | 0.967 | 1.000 | 2228 | tags=29%, list=19%, signal=35% |
| 2502 | GOBP\_AMINOGLYCAN\_CATABOLIC\_PROCESS |  | 47 | -0.25 | -0.90 | 0.642 | 0.968 | 1.000 | 2230 | tags=28%, list=19%, signal=34% |
| 2503 | GOBP\_PURINE\_NUCLEOSIDE\_DIPHOSPHATE\_CATABOLIC\_PROCESS |  | 3 | -0.54 | -0.90 | 0.631 | 0.968 | 1.000 | 5575 | tags=100%, list=46%, signal=187% |
| 2504 | GOBP\_VASCULAR\_ENDOTHELIAL\_GROWTH\_FACTOR\_RECEPTOR\_SIGNALING\_PATHWAY |  | 75 | -0.23 | -0.90 | 0.675 | 0.968 | 1.000 | 1557 | tags=19%, list=13%, signal=21% |
| 2505 | GOBP\_NEGATIVE\_REGULATION\_OF\_HISTONE\_MODIFICATION |  | 31 | -0.27 | -0.90 | 0.616 | 0.968 | 1.000 | 3147 | tags=42%, list=26%, signal=57% |
| 2506 | GOBP\_T\_CELL\_MEDIATED\_IMMUNE\_RESPONSE\_TO\_TUMOR\_CELL |  | 3 | -0.53 | -0.90 | 0.634 | 0.968 | 1.000 | 5595 | tags=100%, list=47%, signal=187% |
| 2507 | GOBP\_G\_PROTEIN\_COUPLED\_ACETYLCHOLINE\_RECEPTOR\_SIGNALING\_PATHWAY |  | 12 | -0.35 | -0.90 | 0.580 | 0.968 | 1.000 | 58 | tags=8%, list=0%, signal=8% |
| 2508 | GOBP\_REGULATION\_OF\_SMOOTHENED\_SIGNALING\_PATHWAY |  | 59 | -0.24 | -0.90 | 0.647 | 0.968 | 1.000 | 2558 | tags=22%, list=21%, signal=28% |
| 2509 | GOBP\_HISTONE\_DEUBIQUITINATION |  | 20 | -0.30 | -0.90 | 0.611 | 0.968 | 1.000 | 2992 | tags=40%, list=25%, signal=53% |
| 2510 | GOBP\_NERVE\_GROWTH\_FACTOR\_SIGNALING\_PATHWAY |  | 7 | -0.41 | -0.90 | 0.589 | 0.967 | 1.000 | 1615 | tags=14%, list=13%, signal=16% |
| 2511 | GOBP\_MUSCLE\_FIBER\_DEVELOPMENT |  | 27 | -0.28 | -0.90 | 0.613 | 0.967 | 1.000 | 2455 | tags=30%, list=20%, signal=37% |
| 2512 | GOBP\_POSITIVE\_REGULATION\_OF\_VESICLE\_FUSION |  | 6 | -0.43 | -0.90 | 0.600 | 0.967 | 1.000 | 1169 | tags=50%, list=10%, signal=55% |
| 2513 | GOMF\_SUMO\_TRANSFERASE\_ACTIVITY |  | 12 | -0.35 | -0.90 | 0.583 | 0.967 | 1.000 | 1049 | tags=25%, list=9%, signal=27% |
| 2514 | GOBP\_ANTIGEN\_RECEPTOR\_MEDIATED\_SIGNALING\_PATHWAY |  | 164 | -0.20 | -0.90 | 0.741 | 0.967 | 1.000 | 3464 | tags=36%, list=29%, signal=50% |
| 2515 | GOBP\_PHOSPHORYLATED\_CARBOHYDRATE\_DEPHOSPHORYLATION |  | 15 | -0.33 | -0.90 | 0.590 | 0.968 | 1.000 | 1084 | tags=13%, list=9%, signal=15% |
| 2516 | GOBP\_POSITIVE\_REGULATION\_OF\_RESPONSE\_TO\_ENDOPLASMIC\_RETICULUM\_STRESS |  | 25 | -0.29 | -0.90 | 0.596 | 0.968 | 1.000 | 720 | tags=16%, list=6%, signal=17% |
| 2517 | GOBP\_REGULATION\_OF\_CALCINEURIN\_MEDIATED\_SIGNALING |  | 19 | -0.31 | -0.90 | 0.600 | 0.968 | 1.000 | 3347 | tags=42%, list=28%, signal=58% |
| 2518 | GOBP\_REGULATION\_OF\_CALCIDIOL\_1\_MONOOXYGENASE\_ACTIVITY |  | 4 | -0.49 | -0.90 | 0.606 | 0.968 | 1.000 | 979 | tags=25%, list=8%, signal=27% |
| 2519 | GOBP\_CORTICOSTEROID\_HORMONE\_SECRETION |  | 10 | -0.37 | -0.90 | 0.585 | 0.969 | 1.000 | 12 | tags=10%, list=0%, signal=10% |
| 2520 | GOBP\_POSITIVE\_REGULATION\_OF\_MEMBRANE\_PROTEIN\_ECTODOMAIN\_PROTEOLYSIS |  | 11 | -0.36 | -0.90 | 0.585 | 0.969 | 1.000 | 3240 | tags=45%, list=27%, signal=62% |
| 2521 | GOBP\_SYNAPTIC\_MEMBRANE\_ADHESION |  | 15 | -0.33 | -0.90 | 0.601 | 0.969 | 1.000 | 3387 | tags=47%, list=28%, signal=65% |
| 2522 | GOBP\_RESPONSE\_TO\_WATER |  | 8 | -0.39 | -0.90 | 0.591 | 0.969 | 1.000 | 44 | tags=13%, list=0%, signal=13% |
| 2523 | GOMF\_METALLOENDOPEPTIDASE\_ACTIVITY |  | 69 | -0.23 | -0.90 | 0.666 | 0.969 | 1.000 | 3240 | tags=36%, list=27%, signal=49% |
| 2524 | GOBP\_INTRINSIC\_APOPTOTIC\_SIGNALING\_PATHWAY\_BY\_P53\_CLASS\_MEDIATOR |  | 48 | -0.25 | -0.90 | 0.642 | 0.969 | 1.000 | 2449 | tags=29%, list=20%, signal=36% |
| 2525 | GOMF\_G\_PROTEIN\_GAMMA\_SUBUNIT\_BINDING |  | 4 | -0.49 | -0.90 | 0.608 | 0.969 | 1.000 | 3104 | tags=75%, list=26%, signal=101% |
| 2526 | GOBP\_MUSCLE\_CELL\_DIFFERENTIATION |  | 217 | -0.19 | -0.90 | 0.796 | 0.969 | 1.000 | 2170 | tags=21%, list=18%, signal=25% |
| 2527 | GOCC\_EARLY\_PHAGOSOME |  | 11 | -0.36 | -0.90 | 0.595 | 0.970 | 1.000 | 2689 | tags=45%, list=22%, signal=59% |
| 2528 | GOBP\_CARDIOLIPIN\_BIOSYNTHETIC\_PROCESS |  | 7 | -0.41 | -0.90 | 0.584 | 0.970 | 1.000 | 1872 | tags=43%, list=16%, signal=51% |
| 2529 | GOBP\_NEGATIVE\_REGULATION\_OF\_TRANSCRIPTION\_INITIATION\_FROM\_RNA\_POLYMERASE\_II\_PROMOTER |  | 5 | -0.45 | -0.90 | 0.594 | 0.971 | 1.000 | 3844 | tags=60%, list=32%, signal=88% |
| 2530 | GOBP\_GASTRULATION\_WITH\_MOUTH\_FORMING\_SECOND |  | 17 | -0.31 | -0.90 | 0.612 | 0.971 | 1.000 | 998 | tags=18%, list=8%, signal=19% |
| 2531 | GOBP\_POSITIVE\_REGULATION\_OF\_SMOOTH\_MUSCLE\_CELL\_APOPTOTIC\_PROCESS |  | 10 | -0.37 | -0.90 | 0.582 | 0.971 | 1.000 | 173 | tags=10%, list=1%, signal=10% |
| 2532 | GOBP\_DNA\_MODIFICATION |  | 73 | -0.23 | -0.90 | 0.686 | 0.971 | 1.000 | 2934 | tags=36%, list=24%, signal=47% |
| 2533 | GOMF\_HISTONE\_METHYLTRANSFERASE\_ACTIVITY\_H3\_K27\_SPECIFIC |  | 3 | -0.53 | -0.90 | 0.632 | 0.972 | 1.000 | 2883 | tags=67%, list=24%, signal=88% |
| 2534 | GOBP\_THYROID\_HORMONE\_MEDIATED\_SIGNALING\_PATHWAY |  | 4 | -0.49 | -0.90 | 0.605 | 0.971 | 1.000 | 2452 | tags=50%, list=20%, signal=63% |
| 2535 | GOBP\_SOMATIC\_STEM\_CELL\_POPULATION\_MAINTENANCE |  | 46 | -0.25 | -0.90 | 0.639 | 0.971 | 1.000 | 1213 | tags=17%, list=10%, signal=19% |
| 2536 | GOBP\_NEGATIVE\_REGULATION\_OF\_CELL\_PROLIFERATION\_INVOLVED\_IN\_KIDNEY\_DEVELOPMENT |  | 4 | -0.48 | -0.90 | 0.610 | 0.971 | 1.000 | 350 | tags=25%, list=3%, signal=26% |
| 2537 | GOMF\_LIGAND\_GATED\_SODIUM\_CHANNEL\_ACTIVITY |  | 7 | -0.41 | -0.90 | 0.588 | 0.970 | 1.000 | 34 | tags=14%, list=0%, signal=14% |
| 2538 | GOBP\_PEPTIDYL\_LYSINE\_MODIFICATION |  | 294 | -0.18 | -0.90 | 0.815 | 0.970 | 1.000 | 2909 | tags=24%, list=24%, signal=31% |
| 2539 | GOMF\_FIBROBLAST\_GROWTH\_FACTOR\_BINDING |  | 18 | -0.31 | -0.90 | 0.609 | 0.970 | 1.000 | 1172 | tags=22%, list=10%, signal=25% |
| 2540 | GOMF\_INOSITOL\_HEXAKISPHOSPHATE\_KINASE\_ACTIVITY |  | 6 | -0.43 | -0.90 | 0.597 | 0.970 | 1.000 | 1015 | tags=33%, list=8%, signal=36% |
| 2541 | GOBP\_POST\_CHAPERONIN\_TUBULIN\_FOLDING\_PATHWAY |  | 5 | -0.46 | -0.90 | 0.597 | 0.969 | 1.000 | 4398 | tags=60%, list=37%, signal=95% |
| 2542 | GOMF\_3\_PHOSPHOADENOSINE\_5\_PHOSPHOSULFATE\_BINDING |  | 3 | -0.53 | -0.90 | 0.650 | 0.970 | 1.000 | 1824 | tags=67%, list=15%, signal=79% |
| 2543 | GOBP\_NEGATIVE\_REGULATION\_OF\_DEPHOSPHORYLATION |  | 71 | -0.23 | -0.89 | 0.682 | 0.971 | 1.000 | 3754 | tags=41%, list=31%, signal=59% |
| 2544 | GOBP\_REGULATION\_OF\_LYSOSOME\_ORGANIZATION |  | 4 | -0.49 | -0.89 | 0.601 | 0.973 | 1.000 | 1103 | tags=25%, list=9%, signal=28% |
| 2545 | GOBP\_CELLULAR\_IRON\_ION\_HOMEOSTASIS |  | 49 | -0.24 | -0.89 | 0.650 | 0.974 | 1.000 | 2316 | tags=31%, list=19%, signal=38% |
| 2546 | GOBP\_REGULATION\_OF\_MYOBLAST\_FUSION |  | 8 | -0.40 | -0.89 | 0.609 | 0.974 | 1.000 | 199 | tags=13%, list=2%, signal=13% |
| 2547 | GOBP\_PHOTORECEPTOR\_CELL\_MAINTENANCE |  | 22 | -0.29 | -0.89 | 0.617 | 0.974 | 1.000 | 930 | tags=18%, list=8%, signal=20% |
| 2548 | GOBP\_MULTI\_ORGANISM\_PROCESS |  | 578 | -0.17 | -0.89 | 0.920 | 0.974 | 1.000 | 3160 | tags=28%, list=26%, signal=36% |
| 2549 | GOBP\_INTERLEUKIN\_2\_PRODUCTION |  | 44 | -0.25 | -0.89 | 0.650 | 0.974 | 1.000 | 2533 | tags=36%, list=21%, signal=46% |
| 2550 | GOCC\_MRNA\_CLEAVAGE\_AND\_POLYADENYLATION\_SPECIFICITY\_FACTOR\_COMPLEX |  | 13 | -0.34 | -0.89 | 0.599 | 0.974 | 1.000 | 3103 | tags=31%, list=26%, signal=41% |
| 2551 | GOCC\_RNA\_POLYMERASE\_II\_CORE\_COMPLEX |  | 10 | -0.37 | -0.89 | 0.599 | 0.974 | 1.000 | 3230 | tags=40%, list=27%, signal=55% |
| 2552 | GOBP\_REGULATION\_OF\_REGULATED\_SECRETORY\_PATHWAY |  | 89 | -0.22 | -0.89 | 0.707 | 0.973 | 1.000 | 2617 | tags=31%, list=22%, signal=40% |
| 2553 | GOBP\_BRANCHING\_MORPHOGENESIS\_OF\_AN\_EPITHELIAL\_TUBE |  | 101 | -0.21 | -0.89 | 0.724 | 0.973 | 1.000 | 2230 | tags=23%, list=19%, signal=28% |
| 2554 | GOBP\_RESPONSE\_TO\_ARSENIC\_CONTAINING\_SUBSTANCE |  | 23 | -0.29 | -0.89 | 0.611 | 0.973 | 1.000 | 994 | tags=17%, list=8%, signal=19% |
| 2555 | GOBP\_B\_CELL\_MEDIATED\_IMMUNITY |  | 70 | -0.23 | -0.89 | 0.688 | 0.975 | 1.000 | 3209 | tags=47%, list=27%, signal=64% |
| 2556 | GOBP\_TRNA\_TRANSPORT |  | 32 | -0.27 | -0.89 | 0.647 | 0.975 | 1.000 | 3998 | tags=44%, list=33%, signal=65% |
| 2557 | GOBP\_POSITIVE\_T\_CELL\_SELECTION |  | 23 | -0.29 | -0.89 | 0.618 | 0.975 | 1.000 | 3199 | tags=52%, list=27%, signal=71% |
| 2558 | GOBP\_SOMATIC\_DIVERSIFICATION\_OF\_IMMUNOGLOBULINS |  | 46 | -0.25 | -0.89 | 0.671 | 0.975 | 1.000 | 3209 | tags=43%, list=27%, signal=59% |
| 2559 | GOMF\_UBIQUINOL\_CYTOCHROME\_C\_REDUCTASE\_ACTIVITY |  | 6 | -0.43 | -0.89 | 0.596 | 0.975 | 1.000 | 2206 | tags=33%, list=18%, signal=41% |
| 2560 | GOBP\_POSITIVE\_REGULATION\_OF\_B\_CELL\_PROLIFERATION |  | 29 | -0.27 | -0.89 | 0.638 | 0.975 | 1.000 | 3223 | tags=55%, list=27%, signal=75% |
| 2561 | GOBP\_PROTEIN\_NITROSYLATION |  | 11 | -0.35 | -0.89 | 0.613 | 0.975 | 1.000 | 1290 | tags=18%, list=11%, signal=20% |
| 2562 | GOBP\_DETECTION\_OF\_LIGHT\_STIMULUS\_INVOLVED\_IN\_SENSORY\_PERCEPTION |  | 8 | -0.39 | -0.89 | 0.601 | 0.975 | 1.000 | 1646 | tags=38%, list=14%, signal=43% |
| 2563 | GOBP\_NEURAL\_CREST\_FORMATION |  | 9 | -0.38 | -0.89 | 0.598 | 0.975 | 1.000 | 3066 | tags=44%, list=26%, signal=60% |
| 2564 | GOBP\_NEGATIVE\_REGULATION\_OF\_ACTION\_POTENTIAL |  | 5 | -0.46 | -0.89 | 0.608 | 0.975 | 1.000 | 1472 | tags=40%, list=12%, signal=46% |
| 2565 | GOBP\_MITOCHONDRIAL\_ELECTRON\_TRANSPORT\_UBIQUINOL\_TO\_CYTOCHROME\_C |  | 10 | -0.37 | -0.89 | 0.602 | 0.976 | 1.000 | 4883 | tags=50%, list=41%, signal=84% |
| 2566 | GOBP\_PYRIMIDINE\_NUCLEOBASE\_BIOSYNTHETIC\_PROCESS |  | 8 | -0.39 | -0.89 | 0.600 | 0.976 | 1.000 | 396 | tags=13%, list=3%, signal=13% |
| 2567 | GOBP\_POSITIVE\_REGULATION\_OF\_PROTEIN\_NEDDYLATION |  | 5 | -0.45 | -0.89 | 0.602 | 0.976 | 1.000 | 1950 | tags=40%, list=16%, signal=48% |
| 2568 | GOBP\_ANIMAL\_ORGAN\_REGENERATION |  | 55 | -0.24 | -0.89 | 0.680 | 0.976 | 1.000 | 669 | tags=11%, list=6%, signal=11% |
| 2569 | GOBP\_CATECHOLAMINE\_UPTAKE\_INVOLVED\_IN\_SYNAPTIC\_TRANSMISSION |  | 7 | -0.41 | -0.89 | 0.593 | 0.977 | 1.000 | 1289 | tags=43%, list=11%, signal=48% |
| 2570 | GOBP\_IRON\_ION\_HOMEOSTASIS |  | 63 | -0.23 | -0.89 | 0.692 | 0.977 | 1.000 | 1894 | tags=25%, list=16%, signal=30% |
| 2571 | GOBP\_STRIATED\_MUSCLE\_CELL\_PROLIFERATION |  | 43 | -0.25 | -0.89 | 0.659 | 0.976 | 1.000 | 3144 | tags=37%, list=26%, signal=50% |
| 2572 | GOBP\_GLYCEROL\_ETHER\_METABOLIC\_PROCESS |  | 16 | -0.32 | -0.89 | 0.617 | 0.977 | 1.000 | 2102 | tags=31%, list=18%, signal=38% |
| 2573 | GOMF\_NEDD8\_SPECIFIC\_PROTEASE\_ACTIVITY |  | 6 | -0.43 | -0.89 | 0.605 | 0.977 | 1.000 | 4565 | tags=83%, list=38%, signal=134% |
| 2574 | GOBP\_DOPAMINE\_RECEPTOR\_SIGNALING\_PATHWAY |  | 25 | -0.29 | -0.89 | 0.633 | 0.977 | 1.000 | 2774 | tags=36%, list=23%, signal=47% |
| 2575 | GOBP\_POSITIVE\_REGULATION\_OF\_CALCIUM\_ION\_TRANSMEMBRANE\_TRANSPORT |  | 47 | -0.24 | -0.89 | 0.674 | 0.977 | 1.000 | 1378 | tags=21%, list=11%, signal=24% |
| 2576 | GOBP\_NEGATIVE\_REGULATION\_OF\_AMYLOID\_FIBRIL\_FORMATION |  | 7 | -0.40 | -0.89 | 0.605 | 0.976 | 1.000 | 372 | tags=29%, list=3%, signal=29% |
| 2577 | GOBP\_COLUMNAR\_CUBOIDAL\_EPITHELIAL\_CELL\_MATURATION |  | 6 | -0.43 | -0.89 | 0.597 | 0.976 | 1.000 | 1418 | tags=33%, list=12%, signal=38% |
| 2578 | GOMF\_ADP\_BINDING |  | 28 | -0.27 | -0.89 | 0.641 | 0.976 | 1.000 | 966 | tags=18%, list=8%, signal=19% |
| 2579 | GOBP\_GERM\_CELL\_DEVELOPMENT |  | 129 | -0.20 | -0.89 | 0.740 | 0.977 | 1.000 | 3476 | tags=36%, list=29%, signal=51% |
| 2580 | GOBP\_REGULATION\_OF\_POSTSYNAPSE\_ORGANIZATION |  | 60 | -0.23 | -0.89 | 0.706 | 0.977 | 1.000 | 969 | tags=10%, list=8%, signal=11% |
| 2581 | GOCC\_DENDRITE\_CYTOPLASM |  | 22 | -0.29 | -0.89 | 0.636 | 0.978 | 1.000 | 1894 | tags=23%, list=16%, signal=27% |
| 2582 | GOBP\_NEGATIVE\_REGULATION\_OF\_MUSCLE\_TISSUE\_DEVELOPMENT |  | 8 | -0.39 | -0.89 | 0.602 | 0.978 | 1.000 | 4323 | tags=75%, list=36%, signal=117% |
| 2583 | GOMF\_GLUTAMATE\_BINDING |  | 3 | -0.52 | -0.88 | 0.637 | 0.979 | 1.000 | 267 | tags=33%, list=2%, signal=34% |
| 2584 | GOBP\_METANEPHRIC\_DISTAL\_TUBULE\_DEVELOPMENT |  | 3 | -0.52 | -0.88 | 0.639 | 0.979 | 1.000 | 5210 | tags=67%, list=43%, signal=118% |
| 2585 | GOBP\_HEART\_TRABECULA\_MORPHOGENESIS |  | 24 | -0.28 | -0.88 | 0.634 | 0.979 | 1.000 | 350 | tags=13%, list=3%, signal=13% |
| 2586 | GOBP\_PROTEIN\_PROCESSING\_INVOLVED\_IN\_PROTEIN\_TARGETING\_TO\_MITOCHONDRION |  | 7 | -0.41 | -0.88 | 0.606 | 0.979 | 1.000 | 2860 | tags=43%, list=24%, signal=56% |
| 2587 | GOBP\_RESPONSE\_TO\_INTERLEUKIN\_2 |  | 11 | -0.35 | -0.88 | 0.615 | 0.979 | 1.000 | 3221 | tags=45%, list=27%, signal=62% |
| 2588 | GOBP\_POSITIVE\_REGULATION\_OF\_PROTEIN\_ACETYLATION |  | 32 | -0.27 | -0.88 | 0.637 | 0.979 | 1.000 | 2541 | tags=31%, list=21%, signal=40% |
| 2589 | GOBP\_MITOCHONDRION\_ORGANIZATION |  | 394 | -0.18 | -0.88 | 0.913 | 0.979 | 1.000 | 3003 | tags=26%, list=25%, signal=34% |
| 2590 | GOBP\_POSITIVE\_REGULATION\_OF\_PRODUCTION\_OF\_MOLECULAR\_MEDIATOR\_OF\_IMMUNE\_RESPONSE |  | 56 | -0.23 | -0.88 | 0.689 | 0.980 | 1.000 | 3209 | tags=39%, list=27%, signal=53% |
| 2591 | GOMF\_L\_AMINO\_ACID\_TRANSMEMBRANE\_TRANSPORTER\_ACTIVITY |  | 33 | -0.26 | -0.88 | 0.655 | 0.980 | 1.000 | 1561 | tags=27%, list=13%, signal=31% |
| 2592 | GOBP\_NEGATIVE\_REGULATION\_OF\_CALCIUM\_ION\_TRANSMEMBRANE\_TRANSPORT |  | 24 | -0.29 | -0.88 | 0.633 | 0.980 | 1.000 | 1937 | tags=25%, list=16%, signal=30% |
| 2593 | GOBP\_REGULATION\_OF\_CELL\_ADHESION\_MEDIATED\_BY\_INTEGRIN |  | 36 | -0.26 | -0.88 | 0.643 | 0.980 | 1.000 | 1118 | tags=22%, list=9%, signal=24% |
| 2594 | GOBP\_POSITIVE\_REGULATION\_OF\_ENDOTHELIAL\_CELL\_DIFFERENTIATION |  | 8 | -0.39 | -0.88 | 0.621 | 0.980 | 1.000 | 1947 | tags=38%, list=16%, signal=45% |
| 2595 | GOBP\_G\_PROTEIN\_COUPLED\_PURINERGIC\_RECEPTOR\_SIGNALING\_PATHWAY |  | 7 | -0.40 | -0.88 | 0.606 | 0.980 | 1.000 | 1253 | tags=29%, list=10%, signal=32% |
| 2596 | GOBP\_NUCLEOSIDE\_TRIPHOSPHATE\_METABOLIC\_PROCESS |  | 62 | -0.23 | -0.88 | 0.692 | 0.980 | 1.000 | 1626 | tags=23%, list=14%, signal=26% |
| 2597 | GOBP\_DORSAL\_VENTRAL\_NEURAL\_TUBE\_PATTERNING |  | 16 | -0.32 | -0.88 | 0.626 | 0.980 | 1.000 | 2656 | tags=38%, list=22%, signal=48% |
| 2598 | GOBP\_SYNAPTIC\_GROWTH\_AT\_NEUROMUSCULAR\_JUNCTION |  | 6 | -0.43 | -0.88 | 0.613 | 0.980 | 1.000 | 3021 | tags=50%, list=25%, signal=67% |
| 2599 | GOBP\_POSITIVE\_REGULATION\_OF\_AXON\_EXTENSION |  | 32 | -0.26 | -0.88 | 0.659 | 0.980 | 1.000 | 2560 | tags=31%, list=21%, signal=40% |
| 2600 | GOBP\_ONE\_CARBON\_COMPOUND\_TRANSPORT |  | 8 | -0.39 | -0.88 | 0.599 | 0.980 | 1.000 | 1064 | tags=25%, list=9%, signal=27% |
| 2601 | GOBP\_ROSTROCAUDAL\_NEURAL\_TUBE\_PATTERNING |  | 5 | -0.44 | -0.88 | 0.622 | 0.981 | 1.000 | 3362 | tags=80%, list=28%, signal=111% |
| 2602 | GOMF\_EPHRIN\_RECEPTOR\_BINDING |  | 22 | -0.29 | -0.88 | 0.642 | 0.981 | 1.000 | 1429 | tags=14%, list=12%, signal=15% |
| 2603 | GOBP\_MATING |  | 25 | -0.28 | -0.88 | 0.639 | 0.981 | 1.000 | 1514 | tags=20%, list=13%, signal=23% |
| 2604 | GOMF\_CYCLOHYDROLASE\_ACTIVITY |  | 5 | -0.45 | -0.88 | 0.607 | 0.981 | 1.000 | 532 | tags=40%, list=4%, signal=42% |
| 2605 | GOMF\_GLUCOSE\_BINDING |  | 7 | -0.40 | -0.88 | 0.610 | 0.981 | 1.000 | 1596 | tags=43%, list=13%, signal=49% |
| 2606 | GOBP\_ZINC\_ION\_HOMEOSTASIS |  | 17 | -0.31 | -0.88 | 0.632 | 0.981 | 1.000 | 109 | tags=12%, list=1%, signal=12% |
| 2607 | GOMF\_UBIQUITIN\_LIKE\_PROTEIN\_CONJUGATING\_ENZYME\_BINDING |  | 27 | -0.28 | -0.88 | 0.654 | 0.982 | 1.000 | 2730 | tags=37%, list=23%, signal=48% |
| 2608 | GOBP\_AUTOPHAGY\_OF\_NUCLEUS |  | 11 | -0.35 | -0.88 | 0.624 | 0.982 | 1.000 | 1642 | tags=18%, list=14%, signal=21% |
| 2609 | GOBP\_METHIONINE\_METABOLIC\_PROCESS |  | 14 | -0.33 | -0.88 | 0.626 | 0.982 | 1.000 | 821 | tags=14%, list=7%, signal=15% |
| 2610 | GOMF\_TRANSMITTER\_GATED\_CHANNEL\_ACTIVITY |  | 14 | -0.33 | -0.88 | 0.618 | 0.982 | 1.000 | 1245 | tags=29%, list=10%, signal=32% |
| 2611 | GOBP\_REGULATION\_OF\_PROTEIN\_SERINE\_THREONINE\_KINASE\_ACTIVITY |  | 348 | -0.18 | -0.88 | 0.903 | 0.982 | 1.000 | 2796 | tags=22%, list=23%, signal=28% |
| 2612 | GOBP\_POSITIVE\_REGULATION\_OF\_CARDIOCYTE\_DIFFERENTIATION |  | 10 | -0.36 | -0.88 | 0.611 | 0.983 | 1.000 | 4196 | tags=60%, list=35%, signal=92% |
| 2613 | GOCC\_GLYCOSYLPHOSPHATIDYLINOSITOL\_N\_ACETYLGLUCOSAMINYLTRANSFERASE\_GPI\_GNT\_COMPLEX |  | 6 | -0.42 | -0.88 | 0.626 | 0.983 | 1.000 | 718 | tags=33%, list=6%, signal=35% |
| 2614 | GOBP\_NEGATIVE\_REGULATION\_OF\_CHROMATIN\_ORGANIZATION |  | 38 | -0.25 | -0.88 | 0.675 | 0.983 | 1.000 | 3147 | tags=37%, list=26%, signal=50% |
| 2615 | GOBP\_REGULATION\_OF\_GASTRIC\_ACID\_SECRETION |  | 7 | -0.40 | -0.88 | 0.615 | 0.983 | 1.000 | 536 | tags=29%, list=4%, signal=30% |
| 2616 | GOBP\_POSITIVE\_REGULATION\_OF\_STEROID\_BIOSYNTHETIC\_PROCESS |  | 11 | -0.35 | -0.88 | 0.604 | 0.984 | 1.000 | 1769 | tags=45%, list=15%, signal=53% |
| 2617 | GOCC\_ROUGH\_ENDOPLASMIC\_RETICULUM |  | 52 | -0.24 | -0.88 | 0.694 | 0.984 | 1.000 | 2333 | tags=31%, list=19%, signal=38% |
| 2618 | GOCC\_GOLGI\_LUMEN |  | 49 | -0.24 | -0.88 | 0.685 | 0.984 | 1.000 | 982 | tags=14%, list=8%, signal=15% |
| 2619 | GOMF\_FATTY\_ACID\_TRANSMEMBRANE\_TRANSPORTER\_ACTIVITY |  | 12 | -0.34 | -0.88 | 0.624 | 0.983 | 1.000 | 1561 | tags=33%, list=13%, signal=38% |
| 2620 | GOBP\_INOSITOL\_LIPID\_MEDIATED\_SIGNALING |  | 139 | -0.20 | -0.88 | 0.780 | 0.983 | 1.000 | 2842 | tags=26%, list=24%, signal=34% |
| 2621 | GOBP\_PEPTIDYL\_CYSTEINE\_MODIFICATION |  | 32 | -0.27 | -0.88 | 0.665 | 0.984 | 1.000 | 3635 | tags=44%, list=30%, signal=63% |
| 2622 | GOBP\_BIOLOGICAL\_PROCESS\_INVOLVED\_IN\_INTERACTION\_WITH\_SYMBIONT |  | 55 | -0.23 | -0.88 | 0.696 | 0.984 | 1.000 | 2541 | tags=27%, list=21%, signal=34% |
| 2623 | GOBP\_NEGATIVE\_REGULATION\_OF\_VOLTAGE\_GATED\_POTASSIUM\_CHANNEL\_ACTIVITY |  | 7 | -0.40 | -0.88 | 0.610 | 0.984 | 1.000 | 1002 | tags=29%, list=8%, signal=31% |
| 2624 | GOCC\_PROTON\_TRANSPORTING\_V\_TYPE\_ATPASE\_COMPLEX |  | 21 | -0.29 | -0.88 | 0.638 | 0.984 | 1.000 | 3769 | tags=43%, list=31%, signal=62% |
| 2625 | GOBP\_TORC2\_SIGNALING |  | 8 | -0.38 | -0.88 | 0.623 | 0.985 | 1.000 | 107 | tags=13%, list=1%, signal=13% |
| 2626 | GOBP\_POSITIVE\_REGULATION\_OF\_CALCIUM\_ION\_TRANSPORT\_INTO\_CYTOSOL |  | 36 | -0.25 | -0.88 | 0.667 | 0.985 | 1.000 | 1457 | tags=22%, list=12%, signal=25% |
| 2627 | GOBP\_PYRIMIDINE\_CONTAINING\_COMPOUND\_CATABOLIC\_PROCESS |  | 25 | -0.28 | -0.87 | 0.645 | 0.985 | 1.000 | 2048 | tags=36%, list=17%, signal=43% |
| 2628 | GOMF\_RIBONUCLEASE\_P\_RNA\_BINDING |  | 6 | -0.42 | -0.87 | 0.620 | 0.987 | 1.000 | 5686 | tags=83%, list=47%, signal=158% |
| 2629 | GOMF\_TRANSITION\_METAL\_ION\_BINDING |  | 654 | -0.17 | -0.87 | 0.970 | 0.987 | 1.000 | 2663 | tags=23%, list=22%, signal=28% |
| 2630 | GOBP\_NEGATIVE\_REGULATION\_OF\_PROTEIN\_MODIFICATION\_PROCESS |  | 378 | -0.18 | -0.87 | 0.919 | 0.987 | 1.000 | 2056 | tags=17%, list=17%, signal=19% |
| 2631 | GOBP\_ENZYME\_DIRECTED\_RRNA\_PSEUDOURIDINE\_SYNTHESIS |  | 4 | -0.47 | -0.87 | 0.640 | 0.987 | 1.000 | 621 | tags=25%, list=5%, signal=26% |
| 2632 | GOBP\_NUCLEOTIDE\_EXCISION\_REPAIR\_DNA\_DUPLEX\_UNWINDING |  | 19 | -0.30 | -0.87 | 0.644 | 0.987 | 1.000 | 2625 | tags=21%, list=22%, signal=27% |
| 2633 | GOBP\_NEGATIVE\_REGULATION\_OF\_PROTEIN\_ACETYLATION |  | 17 | -0.31 | -0.87 | 0.646 | 0.988 | 1.000 | 2909 | tags=41%, list=24%, signal=54% |
| 2634 | GOBP\_POSITIVE\_REGULATION\_OF\_ADENYLATE\_CYCLASE\_ACTIVITY |  | 7 | -0.40 | -0.87 | 0.618 | 0.988 | 1.000 | 902 | tags=29%, list=8%, signal=31% |
| 2635 | GOCC\_NEURON\_PROJECTION\_CYTOPLASM |  | 65 | -0.23 | -0.87 | 0.711 | 0.988 | 1.000 | 1894 | tags=20%, list=16%, signal=24% |
| 2636 | GOBP\_EPITHELIAL\_CELL\_PROLIFERATION\_INVOLVED\_IN\_PROSTATE\_GLAND\_DEVELOPMENT |  | 7 | -0.40 | -0.87 | 0.619 | 0.988 | 1.000 | 2089 | tags=43%, list=17%, signal=52% |
| 2637 | GOCC\_RNA\_POLYMERASE\_II\_TRANSCRIPTION\_REGULATOR\_COMPLEX |  | 132 | -0.20 | -0.87 | 0.802 | 0.988 | 1.000 | 2589 | tags=21%, list=22%, signal=27% |
| 2638 | GOMF\_SYNTAXIN\_1\_BINDING |  | 22 | -0.29 | -0.87 | 0.656 | 0.988 | 1.000 | 1353 | tags=23%, list=11%, signal=26% |
| 2639 | GOBP\_NEUTROPHIL\_DIFFERENTIATION |  | 5 | -0.44 | -0.87 | 0.640 | 0.988 | 1.000 | 2046 | tags=40%, list=17%, signal=48% |
| 2640 | GOBP\_METHYLATION |  | 245 | -0.18 | -0.87 | 0.876 | 0.988 | 1.000 | 2653 | tags=24%, list=22%, signal=30% |
| 2641 | GOBP\_CHEMICAL\_SYNAPTIC\_TRANSMISSION\_POSTSYNAPTIC |  | 51 | -0.24 | -0.87 | 0.714 | 0.989 | 1.000 | 1457 | tags=20%, list=12%, signal=22% |
| 2642 | GOBP\_POSITIVE\_REGULATION\_OF\_HETEROTYPIC\_CELL\_CELL\_ADHESION |  | 9 | -0.37 | -0.87 | 0.631 | 0.990 | 1.000 | 979 | tags=22%, list=8%, signal=24% |
| 2643 | GOBP\_POSITIVE\_REGULATION\_OF\_STRESS\_FIBER\_ASSEMBLY |  | 44 | -0.24 | -0.87 | 0.698 | 0.991 | 1.000 | 166 | tags=7%, list=1%, signal=7% |
| 2644 | GOBP\_POSITIVE\_REGULATION\_OF\_INOSITOL\_TRISPHOSPHATE\_BIOSYNTHETIC\_PROCESS |  | 3 | -0.51 | -0.87 | 0.671 | 0.991 | 1.000 | 1154 | tags=33%, list=10%, signal=37% |
| 2645 | GOMF\_RIBONUCLEASE\_P\_ACTIVITY |  | 6 | -0.42 | -0.87 | 0.621 | 0.991 | 1.000 | 5686 | tags=83%, list=47%, signal=158% |
| 2646 | GOCC\_TERMINAL\_BOUTON |  | 35 | -0.26 | -0.87 | 0.685 | 0.993 | 1.000 | 1257 | tags=23%, list=10%, signal=25% |
| 2647 | GOBP\_CATECHOL\_CONTAINING\_COMPOUND\_BIOSYNTHETIC\_PROCESS |  | 13 | -0.33 | -0.87 | 0.639 | 0.993 | 1.000 | 1044 | tags=31%, list=9%, signal=34% |
| 2648 | GOBP\_REGULATION\_OF\_BROWN\_FAT\_CELL\_DIFFERENTIATION |  | 16 | -0.31 | -0.87 | 0.636 | 0.993 | 1.000 | 408 | tags=13%, list=3%, signal=13% |
| 2649 | GOBP\_SHORT\_CHAIN\_FATTY\_ACID\_METABOLIC\_PROCESS |  | 13 | -0.33 | -0.87 | 0.642 | 0.992 | 1.000 | 2099 | tags=38%, list=17%, signal=47% |
| 2650 | GOBP\_COLLAGEN\_CATABOLIC\_PROCESS |  | 27 | -0.27 | -0.87 | 0.664 | 0.992 | 1.000 | 932 | tags=15%, list=8%, signal=16% |
| 2651 | GOCC\_MULTIMERIC\_RIBONUCLEASE\_P\_COMPLEX |  | 6 | -0.42 | -0.87 | 0.629 | 0.992 | 1.000 | 5686 | tags=83%, list=47%, signal=158% |
| 2652 | GOBP\_DORSAL\_VENTRAL\_AXIS\_SPECIFICATION |  | 4 | -0.47 | -0.87 | 0.632 | 0.992 | 1.000 | 166 | tags=25%, list=1%, signal=25% |
| 2653 | GOBP\_TRNA\_AMINOACYLATION\_FOR\_MITOCHONDRIAL\_PROTEIN\_TRANSLATION |  | 6 | -0.42 | -0.87 | 0.639 | 0.992 | 1.000 | 3250 | tags=67%, list=27%, signal=91% |
| 2654 | GOCC\_MYOFILAMENT |  | 13 | -0.33 | -0.87 | 0.638 | 0.992 | 1.000 | 1013 | tags=15%, list=8%, signal=17% |
| 2655 | GOBP\_HISTONE\_H2A\_K63\_LINKED\_UBIQUITINATION |  | 5 | -0.44 | -0.87 | 0.639 | 0.992 | 1.000 | 2718 | tags=40%, list=23%, signal=52% |
| 2656 | GOBP\_NEGATIVE\_REGULATION\_OF\_FAT\_CELL\_PROLIFERATION |  | 4 | -0.47 | -0.87 | 0.633 | 0.992 | 1.000 | 173 | tags=25%, list=1%, signal=25% |
| 2657 | GOMF\_PROTEIN\_PHOSPHATASE\_1\_BINDING |  | 16 | -0.31 | -0.87 | 0.634 | 0.992 | 1.000 | 13 | tags=6%, list=0%, signal=6% |
| 2658 | GOMF\_PHOSPHATIDYLINOSITOL\_3\_5\_BISPHOSPHATE\_3\_PHOSPHATASE\_ACTIVITY |  | 7 | -0.40 | -0.87 | 0.625 | 0.991 | 1.000 | 2294 | tags=29%, list=19%, signal=35% |
| 2659 | GOMF\_LIGASE\_ACTIVITY |  | 104 | -0.21 | -0.87 | 0.780 | 0.991 | 1.000 | 1916 | tags=21%, list=16%, signal=25% |
| 2660 | GOMF\_THIOREDOXIN\_DISULFIDE\_REDUCTASE\_ACTIVITY |  | 5 | -0.44 | -0.87 | 0.630 | 0.991 | 1.000 | 4382 | tags=60%, list=36%, signal=94% |
| 2661 | GOBP\_MONOCYTE\_DIFFERENTIATION |  | 26 | -0.27 | -0.87 | 0.667 | 0.992 | 1.000 | 2986 | tags=42%, list=25%, signal=56% |
| 2662 | GOBP\_DERMATAN\_SULFATE\_PROTEOGLYCAN\_METABOLIC\_PROCESS |  | 11 | -0.35 | -0.87 | 0.641 | 0.992 | 1.000 | 76 | tags=9%, list=1%, signal=9% |
| 2663 | GOBP\_NEGATIVE\_REGULATION\_OF\_STEM\_CELL\_POPULATION\_MAINTENANCE |  | 3 | -0.51 | -0.87 | 0.666 | 0.992 | 1.000 | 350 | tags=33%, list=3%, signal=34% |
| 2664 | GOCC\_JUNCTIONAL\_SARCOPLASMIC\_RETICULUM\_MEMBRANE |  | 5 | -0.44 | -0.87 | 0.640 | 0.992 | 1.000 | 338 | tags=20%, list=3%, signal=21% |
| 2665 | GOBP\_CHONDROITIN\_SULFATE\_CATABOLIC\_PROCESS |  | 10 | -0.36 | -0.87 | 0.637 | 0.992 | 1.000 | 3777 | tags=60%, list=31%, signal=87% |
| 2666 | GOBP\_SOMATIC\_RECOMBINATION\_OF\_IMMUNOGLOBULIN\_GENE\_SEGMENTS |  | 40 | -0.25 | -0.87 | 0.701 | 0.991 | 1.000 | 3209 | tags=43%, list=27%, signal=58% |
| 2667 | GOBP\_NEURON\_CELLULAR\_HOMEOSTASIS |  | 24 | -0.28 | -0.87 | 0.663 | 0.991 | 1.000 | 1620 | tags=29%, list=13%, signal=34% |
| 2668 | GOBP\_LEUCINE\_CATABOLIC\_PROCESS |  | 6 | -0.42 | -0.86 | 0.634 | 0.992 | 1.000 | 1381 | tags=33%, list=11%, signal=38% |
| 2669 | GOBP\_CANONICAL\_WNT\_SIGNALING\_PATHWAY |  | 237 | -0.18 | -0.86 | 0.880 | 0.991 | 1.000 | 2443 | tags=21%, list=20%, signal=26% |
| 2670 | GOCC\_CD40\_RECEPTOR\_COMPLEX |  | 9 | -0.37 | -0.86 | 0.631 | 0.991 | 1.000 | 3738 | tags=44%, list=31%, signal=64% |
| 2671 | GOCC\_NUCLEOLUS |  | 684 | -0.17 | -0.86 | 0.988 | 0.992 | 1.000 | 2665 | tags=21%, list=22%, signal=26% |
| 2672 | GOMF\_UNFOLDED\_PROTEIN\_BINDING |  | 83 | -0.21 | -0.86 | 0.758 | 0.992 | 1.000 | 3738 | tags=36%, list=31%, signal=52% |
| 2673 | GOBP\_BUNDLE\_OF\_HIS\_CELL\_TO\_PURKINJE\_MYOCYTE\_SIGNALING |  | 3 | -0.51 | -0.86 | 0.664 | 0.992 | 1.000 | 713 | tags=33%, list=6%, signal=35% |
| 2674 | GOCC\_PROTEASOME\_ACCESSORY\_COMPLEX |  | 20 | -0.29 | -0.86 | 0.667 | 0.992 | 1.000 | 8518 | tags=100%, list=71%, signal=343% |
| 2675 | GOMF\_ATP\_DEPENDENT\_MICROTUBULE\_MOTOR\_ACTIVITY |  | 19 | -0.30 | -0.86 | 0.651 | 0.992 | 1.000 | 1665 | tags=21%, list=14%, signal=24% |
| 2676 | GOCC\_MYOSIN\_COMPLEX |  | 33 | -0.26 | -0.86 | 0.692 | 0.991 | 1.000 | 1485 | tags=21%, list=12%, signal=24% |
| 2677 | GOBP\_PARTURITION |  | 8 | -0.38 | -0.86 | 0.635 | 0.991 | 1.000 | 3485 | tags=50%, list=29%, signal=70% |
| 2678 | GOBP\_NEGATIVE\_REGULATION\_OF\_CALCIUM\_ION\_TRANSPORT |  | 30 | -0.27 | -0.86 | 0.680 | 0.991 | 1.000 | 1937 | tags=23%, list=16%, signal=28% |
| 2679 | GOBP\_REGULATION\_OF\_SYNAPTIC\_VESICLE\_EXOCYTOSIS |  | 42 | -0.25 | -0.86 | 0.697 | 0.991 | 1.000 | 2617 | tags=36%, list=22%, signal=46% |
| 2680 | GOBP\_POSITIVE\_REGULATION\_OF\_EPITHELIAL\_CELL\_APOPTOTIC\_PROCESS |  | 21 | -0.29 | -0.86 | 0.663 | 0.991 | 1.000 | 1549 | tags=29%, list=13%, signal=33% |
| 2681 | GOBP\_VITAMIN\_TRANSPORT |  | 28 | -0.27 | -0.86 | 0.668 | 0.991 | 1.000 | 1476 | tags=21%, list=12%, signal=24% |
| 2682 | GOBP\_POSITIVE\_REGULATION\_OF\_GROWTH |  | 162 | -0.19 | -0.86 | 0.841 | 0.991 | 1.000 | 2560 | tags=25%, list=21%, signal=32% |
| 2683 | GOBP\_MITOCHONDRIAL\_PROTEIN\_PROCESSING |  | 13 | -0.33 | -0.86 | 0.653 | 0.991 | 1.000 | 3994 | tags=54%, list=33%, signal=81% |
| 2684 | GOBP\_MIDBRAIN\_DEVELOPMENT |  | 54 | -0.23 | -0.86 | 0.706 | 0.992 | 1.000 | 2206 | tags=20%, list=18%, signal=25% |
| 2685 | GOBP\_REGULATION\_OF\_CHROMATIN\_ORGANIZATION |  | 128 | -0.20 | -0.86 | 0.815 | 0.993 | 1.000 | 3053 | tags=27%, list=25%, signal=36% |
| 2686 | GOCC\_NON\_MOTILE\_CILIUM |  | 77 | -0.22 | -0.86 | 0.765 | 0.993 | 1.000 | 3318 | tags=30%, list=28%, signal=41% |
| 2687 | GOBP\_GLOMERULAR\_MESANGIUM\_DEVELOPMENT |  | 12 | -0.34 | -0.86 | 0.636 | 0.993 | 1.000 | 1051 | tags=17%, list=9%, signal=18% |
| 2688 | GOBP\_HISTONE\_H2A\_K119\_MONOUBIQUITINATION |  | 8 | -0.38 | -0.86 | 0.636 | 0.993 | 1.000 | 1379 | tags=25%, list=11%, signal=28% |
| 2689 | GOMF\_ALPHA\_N\_ACETYLGALACTOSAMINIDE\_ALPHA\_2\_6\_SIALYLTRANSFERASE\_ACTIVITY |  | 6 | -0.41 | -0.86 | 0.638 | 0.993 | 1.000 | 134 | tags=17%, list=1%, signal=17% |
| 2690 | GOBP\_LABYRINTHINE\_LAYER\_MORPHOGENESIS |  | 12 | -0.34 | -0.86 | 0.647 | 0.993 | 1.000 | 1380 | tags=25%, list=11%, signal=28% |
| 2691 | GOBP\_LUNG\_LOBE\_DEVELOPMENT |  | 5 | -0.44 | -0.86 | 0.635 | 0.994 | 1.000 | 1656 | tags=40%, list=14%, signal=46% |
| 2692 | GOCC\_CUL4B\_RING\_E3\_UBIQUITIN\_LIGASE\_COMPLEX |  | 5 | -0.43 | -0.86 | 0.637 | 0.993 | 1.000 | 2167 | tags=20%, list=18%, signal=24% |
| 2693 | GOBP\_COPPER\_ION\_IMPORT |  | 4 | -0.47 | -0.86 | 0.646 | 0.993 | 1.000 | 292 | tags=25%, list=2%, signal=26% |
| 2694 | GOCC\_PI\_BODY |  | 3 | -0.51 | -0.86 | 0.676 | 0.994 | 1.000 | 1152 | tags=33%, list=10%, signal=37% |
| 2695 | GOCC\_EXTRINSIC\_COMPONENT\_OF\_PRESYNAPTIC\_MEMBRANE |  | 5 | -0.44 | -0.86 | 0.637 | 0.993 | 1.000 | 726 | tags=20%, list=6%, signal=21% |
| 2696 | GOBP\_NEUROTRANSMITTER\_UPTAKE |  | 25 | -0.27 | -0.86 | 0.669 | 0.993 | 1.000 | 1289 | tags=24%, list=11%, signal=27% |
| 2697 | GOBP\_REGULATION\_OF\_COMPLEMENT\_ACTIVATION |  | 28 | -0.27 | -0.86 | 0.697 | 0.993 | 1.000 | 3035 | tags=46%, list=25%, signal=62% |
| 2698 | GOBP\_REGULATION\_OF\_MEMBRANE\_PERMEABILITY |  | 58 | -0.23 | -0.86 | 0.736 | 0.993 | 1.000 | 1596 | tags=17%, list=13%, signal=20% |
| 2699 | GOMF\_CILIARY\_NEUROTROPHIC\_FACTOR\_RECEPTOR\_BINDING |  | 8 | -0.38 | -0.86 | 0.635 | 0.993 | 1.000 | 61 | tags=13%, list=1%, signal=13% |
| 2700 | GOBP\_TRICARBOXYLIC\_ACID\_CYCLE |  | 31 | -0.26 | -0.86 | 0.703 | 0.993 | 1.000 | 4200 | tags=42%, list=35%, signal=64% |
| 2701 | GOCC\_TRANSCRIPTION\_FACTOR\_TFIIA\_COMPLEX |  | 4 | -0.46 | -0.86 | 0.650 | 0.993 | 1.000 | 4780 | tags=75%, list=40%, signal=125% |
| 2702 | GOBP\_TELENCEPHALON\_DEVELOPMENT |  | 154 | -0.19 | -0.86 | 0.846 | 0.992 | 1.000 | 2801 | tags=29%, list=23%, signal=38% |
| 2703 | GOMF\_GLYCOSPHINGOLIPID\_BINDING |  | 4 | -0.47 | -0.86 | 0.647 | 0.993 | 1.000 | 907 | tags=50%, list=8%, signal=54% |
| 2704 | GOMF\_MALATE\_DEHYDROGENASE\_ACTIVITY |  | 5 | -0.43 | -0.86 | 0.637 | 0.993 | 1.000 | 928 | tags=20%, list=8%, signal=22% |
| 2705 | GOBP\_POSITIVE\_REGULATION\_OF\_EXECUTION\_PHASE\_OF\_APOPTOSIS |  | 6 | -0.41 | -0.86 | 0.634 | 0.993 | 1.000 | 428 | tags=17%, list=4%, signal=17% |
| 2706 | GOBP\_REGULATION\_OF\_RECEPTOR\_LOCALIZATION\_TO\_SYNAPSE |  | 12 | -0.34 | -0.86 | 0.660 | 0.993 | 1.000 | 194 | tags=8%, list=2%, signal=8% |
| 2707 | GOCC\_SPANNING\_COMPONENT\_OF\_PLASMA\_MEMBRANE |  | 6 | -0.42 | -0.86 | 0.647 | 0.993 | 1.000 | 2338 | tags=50%, list=19%, signal=62% |
| 2708 | GOBP\_TRNA\_THIO\_MODIFICATION |  | 4 | -0.47 | -0.86 | 0.657 | 0.993 | 1.000 | 296 | tags=25%, list=2%, signal=26% |
| 2709 | GOBP\_PRESYNAPTIC\_MODULATION\_OF\_CHEMICAL\_SYNAPTIC\_TRANSMISSION |  | 8 | -0.38 | -0.86 | 0.645 | 0.993 | 1.000 | 2248 | tags=50%, list=19%, signal=61% |
| 2710 | GOCC\_NUCLEAR\_PORE\_CENTRAL\_TRANSPORT\_CHANNEL |  | 4 | -0.47 | -0.86 | 0.641 | 0.993 | 1.000 | 5150 | tags=75%, list=43%, signal=131% |
| 2711 | GOBP\_RESPONSE\_TO\_PEPTIDOGLYCAN |  | 6 | -0.41 | -0.86 | 0.641 | 0.993 | 1.000 | 1638 | tags=50%, list=14%, signal=58% |
| 2712 | GOBP\_REGULATION\_OF\_LOW\_DENSITY\_LIPOPROTEIN\_PARTICLE\_CLEARANCE |  | 7 | -0.39 | -0.86 | 0.635 | 0.993 | 1.000 | 935 | tags=14%, list=8%, signal=15% |
| 2713 | GOBP\_POSITIVE\_REGULATION\_OF\_PEROXISOME\_PROLIFERATOR\_ACTIVATED\_RECEPTOR\_SIGNALING\_PATHWAY |  | 4 | -0.46 | -0.86 | 0.650 | 0.993 | 1.000 | 217 | tags=25%, list=2%, signal=25% |
| 2714 | GOBP\_VITAMIN\_K\_METABOLIC\_PROCESS |  | 3 | -0.51 | -0.86 | 0.668 | 0.993 | 1.000 | 2761 | tags=67%, list=23%, signal=87% |
| 2715 | GOBP\_LEUKOCYTE\_TETHERING\_OR\_ROLLING |  | 19 | -0.29 | -0.86 | 0.667 | 0.994 | 1.000 | 1650 | tags=32%, list=14%, signal=37% |
| 2716 | GOBP\_POSITIVE\_REGULATION\_OF\_MIRNA\_METABOLIC\_PROCESS |  | 5 | -0.44 | -0.86 | 0.646 | 0.994 | 1.000 | 5247 | tags=60%, list=44%, signal=106% |
| 2717 | GOBP\_DIGESTIVE\_TRACT\_MORPHOGENESIS |  | 31 | -0.26 | -0.86 | 0.692 | 0.995 | 1.000 | 2021 | tags=26%, list=17%, signal=31% |
| 2718 | GOBP\_FACIAL\_NERVE\_MORPHOGENESIS |  | 5 | -0.43 | -0.86 | 0.647 | 0.994 | 1.000 | 640 | tags=20%, list=5%, signal=21% |
| 2719 | GOBP\_CYSTEINE\_CATABOLIC\_PROCESS |  | 3 | -0.51 | -0.86 | 0.676 | 0.994 | 1.000 | 69 | tags=33%, list=1%, signal=34% |
| 2720 | GOBP\_MODULATION\_BY\_HOST\_OF\_SYMBIONT\_MOLECULAR\_FUNCTION |  | 4 | -0.46 | -0.85 | 0.646 | 0.994 | 1.000 | 4672 | tags=75%, list=39%, signal=123% |
| 2721 | GOBP\_CELLULAR\_ANION\_HOMEOSTASIS |  | 8 | -0.38 | -0.85 | 0.642 | 0.993 | 1.000 | 1941 | tags=38%, list=16%, signal=45% |
| 2722 | GOMF\_NUCLEOTIDE\_SUGAR\_TRANSMEMBRANE\_TRANSPORTER\_ACTIVITY |  | 8 | -0.37 | -0.85 | 0.649 | 0.993 | 1.000 | 1961 | tags=38%, list=16%, signal=45% |
| 2723 | GOBP\_POSITIVE\_REGULATION\_OF\_MONOCYTE\_CHEMOTACTIC\_PROTEIN\_1\_PRODUCTION |  | 7 | -0.39 | -0.85 | 0.645 | 0.994 | 1.000 | 979 | tags=29%, list=8%, signal=31% |
| 2724 | GOBP\_EMBRYONIC\_PLACENTA\_MORPHOGENESIS |  | 14 | -0.32 | -0.85 | 0.663 | 0.994 | 1.000 | 1380 | tags=21%, list=11%, signal=24% |
| 2725 | GOBP\_POSITIVE\_REGULATION\_OF\_UBIQUITIN\_PROTEIN\_LIGASE\_ACTIVITY |  | 10 | -0.35 | -0.85 | 0.649 | 0.994 | 1.000 | 4805 | tags=70%, list=40%, signal=117% |
| 2726 | GOMF\_PROTEIN\_TYROSINE\_PHOSPHATASE\_ACTIVITY |  | 79 | -0.21 | -0.85 | 0.767 | 0.994 | 1.000 | 1479 | tags=16%, list=12%, signal=19% |
| 2727 | GOBP\_CARDIAC\_LEFT\_VENTRICLE\_MORPHOGENESIS |  | 11 | -0.34 | -0.85 | 0.649 | 0.994 | 1.000 | 2018 | tags=36%, list=17%, signal=44% |
| 2728 | GOBP\_MYOTUBE\_DIFFERENTIATION\_INVOLVED\_IN\_SKELETAL\_MUSCLE\_REGENERATION |  | 5 | -0.43 | -0.85 | 0.639 | 0.994 | 1.000 | 3277 | tags=60%, list=27%, signal=82% |
| 2729 | GOBP\_REGULATION\_OF\_BEHAVIOR |  | 33 | -0.25 | -0.85 | 0.711 | 0.995 | 1.000 | 1769 | tags=24%, list=15%, signal=28% |
| 2730 | GOBP\_REGULATION\_OF\_RESPONSE\_TO\_FOOD |  | 10 | -0.35 | -0.85 | 0.651 | 0.996 | 1.000 | 394 | tags=20%, list=3%, signal=21% |
| 2731 | GOCC\_ELONGATOR\_HOLOENZYME\_COMPLEX |  | 4 | -0.46 | -0.85 | 0.662 | 0.996 | 1.000 | 3039 | tags=75%, list=25%, signal=100% |
| 2732 | GOMF\_NEUROTRANSMITTER\_BINDING |  | 9 | -0.36 | -0.85 | 0.649 | 0.996 | 1.000 | 2156 | tags=44%, list=18%, signal=54% |
| 2733 | GOBP\_SEQUESTERING\_OF\_CALCIUM\_ION |  | 80 | -0.21 | -0.85 | 0.789 | 0.995 | 1.000 | 1378 | tags=15%, list=11%, signal=17% |
| 2734 | GOBP\_PENTOSE\_METABOLIC\_PROCESS |  | 10 | -0.35 | -0.85 | 0.645 | 0.995 | 1.000 | 1575 | tags=30%, list=13%, signal=34% |
| 2735 | GOBP\_POSITIVE\_REGULATION\_OF\_BLOOD\_CIRCULATION |  | 27 | -0.27 | -0.85 | 0.690 | 0.995 | 1.000 | 363 | tags=11%, list=3%, signal=11% |
| 2736 | GOBP\_NUCLEOTIDE\_SUGAR\_TRANSMEMBRANE\_TRANSPORT |  | 8 | -0.38 | -0.85 | 0.653 | 0.995 | 1.000 | 1220 | tags=25%, list=10%, signal=28% |
| 2737 | GOBP\_REGULATION\_OF\_SECONDARY\_METABOLIC\_PROCESS |  | 8 | -0.37 | -0.85 | 0.647 | 0.995 | 1.000 | 1806 | tags=25%, list=15%, signal=29% |
| 2738 | GOBP\_VACUOLAR\_ACIDIFICATION |  | 21 | -0.28 | -0.85 | 0.683 | 0.996 | 1.000 | 1852 | tags=24%, list=15%, signal=28% |
| 2739 | GOBP\_DEPHOSPHORYLATION\_OF\_RNA\_POLYMERASE\_II\_C\_TERMINAL\_DOMAIN |  | 5 | -0.43 | -0.85 | 0.647 | 0.996 | 1.000 | 620 | tags=20%, list=5%, signal=21% |
| 2740 | GOBP\_RAC\_PROTEIN\_SIGNAL\_TRANSDUCTION |  | 32 | -0.26 | -0.85 | 0.705 | 0.996 | 1.000 | 1300 | tags=19%, list=11%, signal=21% |
| 2741 | GOCC\_SAM\_COMPLEX |  | 10 | -0.35 | -0.85 | 0.656 | 0.996 | 1.000 | 4649 | tags=50%, list=39%, signal=82% |
| 2742 | GOMF\_MODIFICATION\_DEPENDENT\_PROTEIN\_BINDING |  | 105 | -0.20 | -0.85 | 0.819 | 0.996 | 1.000 | 3259 | tags=27%, list=27%, signal=36% |
| 2743 | GOBP\_DETECTION\_OF\_MECHANICAL\_STIMULUS |  | 25 | -0.27 | -0.85 | 0.689 | 0.996 | 1.000 | 2582 | tags=32%, list=21%, signal=41% |
| 2744 | GOBP\_PIRNA\_BIOSYNTHETIC\_PROCESS |  | 3 | -0.51 | -0.85 | 0.693 | 0.996 | 1.000 | 4989 | tags=67%, list=42%, signal=114% |
| 2745 | GOBP\_NEGATIVE\_REGULATION\_OF\_BLOOD\_VESSEL\_ENDOTHELIAL\_CELL\_PROLIFERATION\_INVOLVED\_IN\_SPROUTING\_ANGIOGENESIS |  | 4 | -0.46 | -0.85 | 0.660 | 0.996 | 1.000 | 156 | tags=25%, list=1%, signal=25% |
| 2746 | GOBP\_NEGATIVE\_REGULATION\_OF\_SECONDARY\_METABOLITE\_BIOSYNTHETIC\_PROCESS |  | 4 | -0.46 | -0.85 | 0.653 | 0.996 | 1.000 | 3311 | tags=50%, list=28%, signal=69% |
| 2747 | GOBP\_SNRNA\_TRANSCRIPTION |  | 66 | -0.22 | -0.85 | 0.768 | 0.996 | 1.000 | 3746 | tags=30%, list=31%, signal=44% |
| 2748 | GOBP\_POSITIVE\_REGULATION\_OF\_ENDOTHELIAL\_CELL\_APOPTOTIC\_PROCESS |  | 12 | -0.33 | -0.85 | 0.650 | 0.996 | 1.000 | 1549 | tags=33%, list=13%, signal=38% |
| 2749 | GOMF\_ACYL\_COA\_OXIDASE\_ACTIVITY |  | 6 | -0.41 | -0.85 | 0.652 | 0.996 | 1.000 | 4024 | tags=50%, list=34%, signal=75% |
| 2750 | GOBP\_REGULATION\_OF\_CYSTEINE\_TYPE\_ENDOPEPTIDASE\_ACTIVITY\_INVOLVED\_IN\_EXECUTION\_PHASE\_OF\_APOPTOSIS |  | 4 | -0.46 | -0.85 | 0.655 | 0.997 | 1.000 | 371 | tags=25%, list=3%, signal=26% |
| 2751 | GOBP\_PEPTIDE\_MODIFICATION |  | 3 | -0.50 | -0.85 | 0.692 | 0.996 | 1.000 | 2710 | tags=67%, list=23%, signal=86% |
[truncated: 173,610 more chars]
