# Supplementary material for: Time-series transcriptome analysis identified differentially expressed genes in broiler chicken infected with mixed Eimeria species
Source: Front Genet. 2022 Aug 8;13:886781. doi: 10.3389/fgene.2022.886781 (PMC9393255; doi:10.3389/fgene.2022.886781)
Supplement: Supplementary file 2 [file DataSheet1.ZIP › 4dpi_GO.Gsea.1625071243202/gsea_report_for_1_1625071243202.html]

Report for 1 1625071243202 [GSEA]

| GS  follow link to MSigDB | GS DETAILS | SIZE | ES | NES | NOM p-val | FDR q-val | FWER p-val | RANK AT MAX | LEADING EDGE || 1 | GOBP\_STEROL\_BIOSYNTHETIC\_PROCESS | Details ... | 58 | 0.65 | 2.32 | 0.000 | 0.000 | 0.000 | 1196 | tags=41%, list=10%, signal=46% |
| 2 | GOBP\_REGULATION\_OF\_CHOLESTEROL\_BIOSYNTHETIC\_PROCESS | Details ... | 37 | 0.70 | 2.31 | 0.000 | 0.000 | 0.000 | 1853 | tags=54%, list=15%, signal=64% |
| 3 | GOBP\_REGULATION\_OF\_CHOLESTEROL\_METABOLIC\_PROCESS | Details ... | 45 | 0.68 | 2.31 | 0.000 | 0.000 | 0.000 | 1853 | tags=51%, list=15%, signal=60% |
| 4 | GOBP\_CORNIFICATION | Details ... | 26 | 0.76 | 2.27 | 0.000 | 0.000 | 0.001 | 1910 | tags=73%, list=16%, signal=87% |
| 5 | GOBP\_KERATINIZATION | Details ... | 28 | 0.74 | 2.27 | 0.000 | 0.000 | 0.001 | 1910 | tags=68%, list=16%, signal=81% |
| 6 | GOBP\_LIPID\_HOMEOSTASIS | Details ... | 92 | 0.58 | 2.23 | 0.000 | 0.000 | 0.002 | 2060 | tags=45%, list=17%, signal=53% |
| 7 | GOBP\_DEFENSE\_RESPONSE\_TO\_VIRUS | Details ... | 135 | 0.54 | 2.22 | 0.000 | 0.000 | 0.003 | 1137 | tags=24%, list=9%, signal=26% |
| 8 | GOBP\_RESPONSE\_TO\_TYPE\_I\_INTERFERON | Details ... | 49 | 0.64 | 2.21 | 0.000 | 0.000 | 0.003 | 1101 | tags=37%, list=9%, signal=40% |
| 9 | GOBP\_REGULATION\_OF\_STEROID\_BIOSYNTHETIC\_PROCESS | Details ... | 67 | 0.60 | 2.20 | 0.000 | 0.000 | 0.003 | 1853 | tags=42%, list=15%, signal=49% |
| 10 | GOBP\_STEROL\_METABOLIC\_PROCESS | Details ... | 112 | 0.55 | 2.19 | 0.000 | 0.001 | 0.005 | 1777 | tags=39%, list=15%, signal=46% |
| 11 | GOBP\_RESPONSE\_TO\_GLUCAGON | Details ... | 27 | 0.72 | 2.18 | 0.000 | 0.001 | 0.007 | 1427 | tags=44%, list=12%, signal=50% |
| 12 | GOBP\_INTERFERON\_GAMMA\_MEDIATED\_SIGNALING\_PATHWAY | Details ... | 45 | 0.64 | 2.18 | 0.000 | 0.001 | 0.007 | 981 | tags=40%, list=8%, signal=43% |
| 13 | GOBP\_ACYLGLYCEROL\_HOMEOSTASIS | Details ... | 19 | 0.77 | 2.15 | 0.000 | 0.002 | 0.018 | 1369 | tags=63%, list=11%, signal=71% |
| 14 | GOBP\_STEROL\_HOMEOSTASIS | Details ... | 56 | 0.61 | 2.14 | 0.000 | 0.002 | 0.023 | 1967 | tags=48%, list=16%, signal=57% |
| 15 | GOBP\_REGULATION\_OF\_ALCOHOL\_BIOSYNTHETIC\_PROCESS | Details ... | 56 | 0.60 | 2.13 | 0.000 | 0.002 | 0.025 | 1853 | tags=43%, list=15%, signal=50% |
| 16 | GOBP\_REGULATION\_OF\_STEROID\_METABOLIC\_PROCESS | Details ... | 86 | 0.56 | 2.13 | 0.000 | 0.002 | 0.025 | 1853 | tags=38%, list=15%, signal=45% |
| 17 | GOBP\_KERATINOCYTE\_DIFFERENTIATION | Details ... | 75 | 0.57 | 2.13 | 0.000 | 0.002 | 0.026 | 1922 | tags=41%, list=16%, signal=49% |
| 18 | GOBP\_REGULATION\_OF\_LIPID\_BIOSYNTHETIC\_PROCESS | Details ... | 144 | 0.51 | 2.12 | 0.000 | 0.002 | 0.036 | 1917 | tags=36%, list=16%, signal=42% |
| 19 | GOBP\_DIGESTION | Details ... | 68 | 0.57 | 2.12 | 0.000 | 0.002 | 0.037 | 1853 | tags=50%, list=15%, signal=59% |
| 20 | GOCC\_CORNIFIED\_ENVELOPE | Details ... | 12 | 0.85 | 2.10 | 0.000 | 0.003 | 0.047 | 1583 | tags=92%, list=13%, signal=105% |
| 21 | GOBP\_INTESTINAL\_ABSORPTION |  | 27 | 0.69 | 2.10 | 0.000 | 0.002 | 0.048 | 1360 | tags=63%, list=11%, signal=71% |
| 22 | GOBP\_REGULATION\_OF\_RESPONSE\_TO\_INTERFERON\_GAMMA |  | 18 | 0.75 | 2.09 | 0.000 | 0.004 | 0.070 | 965 | tags=44%, list=8%, signal=48% |
| 23 | GOBP\_POSITIVE\_REGULATION\_OF\_RESPONSE\_TO\_CYTOKINE\_STIMULUS |  | 36 | 0.63 | 2.06 | 0.000 | 0.005 | 0.103 | 620 | tags=25%, list=5%, signal=26% |
| 24 | GOBP\_INTERFERON\_ALPHA\_PRODUCTION |  | 19 | 0.74 | 2.06 | 0.000 | 0.005 | 0.104 | 85 | tags=26%, list=1%, signal=26% |
| 25 | GOBP\_TRIGLYCERIDE\_METABOLIC\_PROCESS |  | 70 | 0.56 | 2.06 | 0.000 | 0.006 | 0.122 | 1172 | tags=40%, list=10%, signal=44% |
| 26 | GOBP\_RESPONSE\_TO\_VIRUS |  | 196 | 0.48 | 2.05 | 0.000 | 0.006 | 0.130 | 1137 | tags=21%, list=9%, signal=23% |
| 27 | GOBP\_RESPONSE\_TO\_INTERFERON\_GAMMA |  | 101 | 0.52 | 2.02 | 0.000 | 0.010 | 0.212 | 1535 | tags=34%, list=13%, signal=38% |
| 28 | GOBP\_LIPID\_STORAGE |  | 50 | 0.58 | 2.02 | 0.000 | 0.010 | 0.230 | 1636 | tags=44%, list=14%, signal=51% |
| 29 | GOBP\_DIGESTIVE\_SYSTEM\_PROCESS |  | 54 | 0.57 | 2.00 | 0.000 | 0.012 | 0.285 | 1853 | tags=48%, list=15%, signal=57% |
| 30 | GOBP\_CYTOPLASMIC\_PATTERN\_RECOGNITION\_RECEPTOR\_SIGNALING\_PATHWAY\_IN\_RESPONSE\_TO\_VIRUS |  | 20 | 0.70 | 1.99 | 0.000 | 0.017 | 0.381 | 1137 | tags=35%, list=9%, signal=39% |
| 31 | GOBP\_ISOPRENOID\_BIOSYNTHETIC\_PROCESS |  | 21 | 0.70 | 1.98 | 0.000 | 0.019 | 0.422 | 581 | tags=38%, list=5%, signal=40% |
| 32 | GOBP\_INTESTINAL\_LIPID\_ABSORPTION |  | 11 | 0.82 | 1.97 | 0.000 | 0.020 | 0.452 | 1326 | tags=82%, list=11%, signal=92% |
| 33 | GOBP\_NEGATIVE\_REGULATION\_OF\_VIRAL\_PROCESS |  | 38 | 0.60 | 1.97 | 0.000 | 0.019 | 0.452 | 1041 | tags=32%, list=9%, signal=34% |
| 34 | GOBP\_NEGATIVE\_REGULATION\_OF\_VIRAL\_GENOME\_REPLICATION |  | 22 | 0.69 | 1.96 | 0.001 | 0.022 | 0.509 | 438 | tags=32%, list=4%, signal=33% |
| 35 | GOBP\_ACTIVATION\_OF\_NF\_KAPPAB\_INDUCING\_KINASE\_ACTIVITY |  | 11 | 0.81 | 1.96 | 0.001 | 0.022 | 0.522 | 171 | tags=27%, list=1%, signal=28% |
| 36 | GOCC\_DESMOSOME |  | 15 | 0.74 | 1.96 | 0.000 | 0.022 | 0.530 | 1583 | tags=53%, list=13%, signal=61% |
| 37 | GOBP\_POSITIVE\_REGULATION\_OF\_LIPID\_STORAGE |  | 14 | 0.75 | 1.96 | 0.000 | 0.023 | 0.555 | 1326 | tags=50%, list=11%, signal=56% |
| 38 | GOBP\_POSITIVE\_REGULATION\_OF\_DEFENSE\_RESPONSE\_TO\_VIRUS\_BY\_HOST |  | 14 | 0.76 | 1.95 | 0.001 | 0.025 | 0.586 | 293 | tags=29%, list=2%, signal=29% |
| 39 | GOBP\_NEGATIVE\_REGULATION\_OF\_LIPID\_TRANSPORT |  | 19 | 0.70 | 1.95 | 0.001 | 0.025 | 0.598 | 876 | tags=32%, list=7%, signal=34% |
| 40 | GOMF\_STEROL\_TRANSPORTER\_ACTIVITY |  | 29 | 0.63 | 1.94 | 0.000 | 0.027 | 0.635 | 1686 | tags=45%, list=14%, signal=52% |
| 41 | GOBP\_POSITIVE\_REGULATION\_OF\_INTERFERON\_ALPHA\_PRODUCTION |  | 16 | 0.72 | 1.93 | 0.000 | 0.032 | 0.708 | 85 | tags=25%, list=1%, signal=25% |
| 42 | GOBP\_EPIDERMIS\_DEVELOPMENT |  | 183 | 0.45 | 1.93 | 0.000 | 0.034 | 0.736 | 1922 | tags=32%, list=16%, signal=38% |
| 43 | GOBP\_MDA\_5\_SIGNALING\_PATHWAY |  | 8 | 0.87 | 1.92 | 0.001 | 0.034 | 0.742 | 724 | tags=63%, list=6%, signal=66% |
| 44 | GOBP\_EPIDERMAL\_CELL\_DIFFERENTIATION |  | 115 | 0.48 | 1.92 | 0.000 | 0.034 | 0.752 | 2078 | tags=34%, list=17%, signal=41% |
| 45 | GOBP\_CELLULAR\_RESPONSE\_TO\_GLUCAGON\_STIMULUS |  | 19 | 0.69 | 1.92 | 0.002 | 0.034 | 0.759 | 1427 | tags=42%, list=12%, signal=48% |
| 46 | GOBP\_CELL\_DIFFERENTIATION\_INVOLVED\_IN\_EMBRYONIC\_PLACENTA\_DEVELOPMENT |  | 14 | 0.74 | 1.91 | 0.001 | 0.038 | 0.811 | 2138 | tags=57%, list=18%, signal=69% |
| 47 | GOBP\_LOW\_DENSITY\_LIPOPROTEIN\_PARTICLE\_CLEARANCE |  | 24 | 0.64 | 1.91 | 0.000 | 0.041 | 0.841 | 1326 | tags=33%, list=11%, signal=37% |
| 48 | GOBP\_REGULATION\_OF\_MYD88\_INDEPENDENT\_TOLL\_LIKE\_RECEPTOR\_SIGNALING\_PATHWAY |  | 6 | 0.93 | 1.90 | 0.000 | 0.043 | 0.858 | 85 | tags=33%, list=1%, signal=34% |
| 49 | GOBP\_INTERMEMBRANE\_LIPID\_TRANSFER |  | 34 | 0.59 | 1.90 | 0.001 | 0.043 | 0.860 | 1686 | tags=38%, list=14%, signal=44% |
| 50 | GOMF\_LIPID\_TRANSFER\_ACTIVITY |  | 34 | 0.59 | 1.90 | 0.000 | 0.043 | 0.864 | 1686 | tags=38%, list=14%, signal=44% |
| 51 | GOBP\_GLYCEROLIPID\_CATABOLIC\_PROCESS |  | 52 | 0.55 | 1.90 | 0.001 | 0.042 | 0.868 | 1752 | tags=46%, list=15%, signal=54% |
| 52 | GOBP\_POSITIVE\_REGULATION\_OF\_INTERFERON\_BETA\_PRODUCTION |  | 24 | 0.65 | 1.90 | 0.001 | 0.043 | 0.873 | 724 | tags=25%, list=6%, signal=27% |
| 53 | GOMF\_STEROL\_TRANSFER\_ACTIVITY |  | 17 | 0.69 | 1.89 | 0.002 | 0.045 | 0.892 | 733 | tags=41%, list=6%, signal=44% |
| 54 | GOBP\_DESMOSOME\_ORGANIZATION |  | 8 | 0.86 | 1.89 | 0.000 | 0.046 | 0.902 | 1276 | tags=75%, list=11%, signal=84% |
| 55 | GOBP\_ALCOHOL\_BIOSYNTHETIC\_PROCESS |  | 113 | 0.47 | 1.89 | 0.000 | 0.046 | 0.907 | 1424 | tags=29%, list=12%, signal=33% |
| 56 | GOBP\_NEUTRAL\_LIPID\_CATABOLIC\_PROCESS |  | 32 | 0.60 | 1.89 | 0.001 | 0.046 | 0.912 | 1105 | tags=44%, list=9%, signal=48% |
| 57 | GOBP\_POSITIVE\_REGULATION\_OF\_PEPTIDE\_SECRETION |  | 95 | 0.49 | 1.89 | 0.000 | 0.046 | 0.915 | 2049 | tags=37%, list=17%, signal=44% |
| 58 | GOBP\_POSITIVE\_REGULATION\_OF\_DOUBLE\_STRAND\_BREAK\_REPAIR\_VIA\_NONHOMOLOGOUS\_END\_JOINING |  | 11 | 0.78 | 1.88 | 0.001 | 0.046 | 0.919 | 526 | tags=36%, list=4%, signal=38% |
| 59 | GOBP\_TERPENOID\_BIOSYNTHETIC\_PROCESS |  | 11 | 0.78 | 1.88 | 0.001 | 0.049 | 0.933 | 581 | tags=55%, list=5%, signal=57% |
| 60 | GOBP\_LIPID\_DIGESTION |  | 9 | 0.81 | 1.88 | 0.001 | 0.052 | 0.943 | 1326 | tags=78%, list=11%, signal=87% |
| 61 | GOMF\_HORMONE\_ACTIVITY |  | 39 | 0.57 | 1.87 | 0.001 | 0.051 | 0.944 | 1049 | tags=31%, list=9%, signal=34% |
| 62 | GOBP\_NEUTRAL\_LIPID\_METABOLIC\_PROCESS |  | 91 | 0.49 | 1.87 | 0.000 | 0.054 | 0.955 | 1845 | tags=42%, list=15%, signal=49% |
| 63 | GOBP\_POSITIVE\_REGULATION\_OF\_PROTEIN\_LOCALIZATION\_TO\_CELL\_PERIPHERY |  | 46 | 0.55 | 1.86 | 0.001 | 0.059 | 0.970 | 3126 | tags=59%, list=26%, signal=79% |
| 64 | GOBP\_RESPONSE\_TO\_CORTICOSTERONE |  | 9 | 0.81 | 1.86 | 0.000 | 0.061 | 0.973 | 1216 | tags=44%, list=10%, signal=49% |
| 65 | GOBP\_PLASMA\_LIPOPROTEIN\_PARTICLE\_CLEARANCE |  | 31 | 0.60 | 1.86 | 0.001 | 0.060 | 0.973 | 1763 | tags=35%, list=15%, signal=41% |
| 66 | GOBP\_REGULATION\_OF\_RESPONSE\_TO\_CYTOKINE\_STIMULUS |  | 113 | 0.47 | 1.86 | 0.000 | 0.060 | 0.973 | 1317 | tags=25%, list=11%, signal=28% |
| 67 | GOBP\_MITOTIC\_SISTER\_CHROMATID\_SEGREGATION |  | 133 | 0.45 | 1.86 | 0.000 | 0.062 | 0.977 | 3978 | tags=57%, list=33%, signal=84% |
| 68 | GOBP\_CELLULAR\_RESPONSE\_TO\_LIPOPROTEIN\_PARTICLE\_STIMULUS |  | 24 | 0.63 | 1.85 | 0.001 | 0.064 | 0.981 | 1326 | tags=38%, list=11%, signal=42% |
| 69 | GOBP\_CELLULAR\_RESPONSE\_TO\_VIRUS |  | 39 | 0.56 | 1.85 | 0.002 | 0.066 | 0.985 | 724 | tags=26%, list=6%, signal=27% |
| 70 | GOBP\_VERY\_LOW\_DENSITY\_LIPOPROTEIN\_PARTICLE\_ASSEMBLY |  | 8 | 0.83 | 1.85 | 0.001 | 0.065 | 0.985 | 1853 | tags=88%, list=15%, signal=103% |
| 71 | GOMF\_EXTRACELLULAR\_MATRIX\_STRUCTURAL\_CONSTITUENT\_CONFERRING\_TENSILE\_STRENGTH |  | 27 | 0.61 | 1.85 | 0.003 | 0.064 | 0.985 | 3082 | tags=63%, list=26%, signal=85% |
| 72 | GOMF\_SINGLE\_STRANDED\_DNA\_HELICASE\_ACTIVITY |  | 17 | 0.68 | 1.85 | 0.002 | 0.063 | 0.986 | 2926 | tags=76%, list=24%, signal=101% |
| 73 | GOBP\_POSITIVE\_REGULATION\_OF\_PATTERN\_RECOGNITION\_RECEPTOR\_SIGNALING\_PATHWAY |  | 28 | 0.60 | 1.85 | 0.000 | 0.063 | 0.987 | 262 | tags=18%, list=2%, signal=18% |
| 74 | GOCC\_APICAL\_PART\_OF\_CELL |  | 277 | 0.41 | 1.84 | 0.000 | 0.067 | 0.990 | 1604 | tags=25%, list=13%, signal=28% |
| 75 | GOCC\_INTERMEDIATE\_FILAMENT |  | 49 | 0.53 | 1.84 | 0.000 | 0.070 | 0.992 | 1389 | tags=33%, list=12%, signal=37% |
| 76 | GOCC\_SITE\_OF\_DNA\_DAMAGE |  | 72 | 0.49 | 1.84 | 0.000 | 0.069 | 0.992 | 3641 | tags=51%, list=30%, signal=73% |
| 77 | GOBP\_STEROL\_TRANSPORT |  | 73 | 0.49 | 1.84 | 0.000 | 0.069 | 0.993 | 2129 | tags=40%, list=18%, signal=48% |
| 78 | GOBP\_POSITIVE\_REGULATION\_OF\_PEPTIDE\_HORMONE\_SECRETION |  | 59 | 0.51 | 1.84 | 0.001 | 0.071 | 0.994 | 1856 | tags=39%, list=15%, signal=46% |
| 79 | GOBP\_NEGATIVE\_REGULATION\_OF\_INNATE\_IMMUNE\_RESPONSE |  | 30 | 0.59 | 1.83 | 0.001 | 0.073 | 0.995 | 412 | tags=23%, list=3%, signal=24% |
| 80 | GOBP\_REGULATION\_OF\_MICROVILLUS\_ORGANIZATION |  | 11 | 0.76 | 1.83 | 0.003 | 0.073 | 0.995 | 1114 | tags=73%, list=9%, signal=80% |
| 81 | GOBP\_TRIGLYCERIDE\_CATABOLIC\_PROCESS |  | 25 | 0.61 | 1.83 | 0.003 | 0.073 | 0.995 | 1105 | tags=48%, list=9%, signal=53% |
| 82 | GOCC\_COPI\_COATED\_VESICLE\_MEMBRANE |  | 14 | 0.71 | 1.83 | 0.003 | 0.074 | 0.995 | 3372 | tags=93%, list=28%, signal=129% |
| 83 | GOCC\_APICAL\_PLASMA\_MEMBRANE |  | 228 | 0.42 | 1.83 | 0.000 | 0.073 | 0.995 | 1465 | tags=25%, list=12%, signal=28% |
| 84 | GOBP\_POSITIVE\_REGULATION\_OF\_TYPE\_I\_INTERFERON\_MEDIATED\_SIGNALING\_PATHWAY |  | 9 | 0.79 | 1.82 | 0.003 | 0.086 | 0.999 | 505 | tags=33%, list=4%, signal=35% |
| 85 | GOBP\_POSITIVE\_REGULATION\_OF\_TYPE\_I\_INTERFERON\_PRODUCTION |  | 57 | 0.51 | 1.81 | 0.000 | 0.093 | 0.999 | 942 | tags=19%, list=8%, signal=21% |
| 86 | GOBP\_CHROMOSOME\_ORGANIZATION\_INVOLVED\_IN\_MEIOTIC\_CELL\_CYCLE |  | 37 | 0.55 | 1.81 | 0.001 | 0.092 | 0.999 | 3684 | tags=59%, list=31%, signal=86% |
| 87 | GOBP\_POSITIVE\_REGULATION\_OF\_RIG\_I\_SIGNALING\_PATHWAY |  | 6 | 0.89 | 1.81 | 0.001 | 0.091 | 0.999 | 116 | tags=33%, list=1%, signal=34% |
| 88 | GOBP\_LIPID\_LOCALIZATION |  | 309 | 0.40 | 1.80 | 0.000 | 0.097 | 1.000 | 1967 | tags=31%, list=16%, signal=37% |
| 89 | GOBP\_MICROVILLUS\_ORGANIZATION |  | 18 | 0.66 | 1.80 | 0.003 | 0.096 | 1.000 | 2787 | tags=72%, list=23%, signal=94% |
| 90 | GOBP\_MESENCHYMAL\_STEM\_CELL\_DIFFERENTIATION |  | 6 | 0.89 | 1.80 | 0.002 | 0.096 | 1.000 | 477 | tags=33%, list=4%, signal=35% |
| 91 | GOBP\_REGULATION\_OF\_PEPTIDE\_SECRETION |  | 197 | 0.42 | 1.80 | 0.000 | 0.097 | 1.000 | 1856 | tags=29%, list=15%, signal=34% |
| 92 | GOBP\_SKIN\_DEVELOPMENT |  | 159 | 0.43 | 1.80 | 0.000 | 0.096 | 1.000 | 1922 | tags=31%, list=16%, signal=37% |
| 93 | GOBP\_DNA\_DOUBLE\_STRAND\_BREAK\_PROCESSING |  | 21 | 0.63 | 1.80 | 0.004 | 0.098 | 1.000 | 2597 | tags=48%, list=22%, signal=61% |
| 94 | GOBP\_STEROID\_BIOSYNTHETIC\_PROCESS |  | 132 | 0.44 | 1.79 | 0.000 | 0.103 | 1.000 | 1419 | tags=27%, list=12%, signal=30% |
| 95 | GOBP\_REGULATION\_OF\_VIRAL\_INDUCED\_CYTOPLASMIC\_PATTERN\_RECOGNITION\_RECEPTOR\_SIGNALING\_PATHWAY |  | 15 | 0.68 | 1.79 | 0.003 | 0.105 | 1.000 | 1137 | tags=33%, list=9%, signal=37% |
| 96 | GOBP\_RETROGRADE\_VESICLE\_MEDIATED\_TRANSPORT\_GOLGI\_TO\_ENDOPLASMIC\_RETICULUM |  | 75 | 0.48 | 1.79 | 0.000 | 0.106 | 1.000 | 3372 | tags=51%, list=28%, signal=70% |
| 97 | GOBP\_PEPTIDE\_HORMONE\_SECRETION |  | 165 | 0.42 | 1.79 | 0.000 | 0.105 | 1.000 | 1947 | tags=33%, list=16%, signal=39% |
| 98 | GOBP\_PHOSPHOLIPID\_TRANSPORT |  | 61 | 0.50 | 1.79 | 0.001 | 0.108 | 1.000 | 1319 | tags=30%, list=11%, signal=33% |
| 99 | GOBP\_REGULATION\_OF\_LIPID\_METABOLIC\_PROCESS |  | 288 | 0.40 | 1.79 | 0.000 | 0.108 | 1.000 | 1919 | tags=28%, list=16%, signal=33% |
| 100 | GOBP\_NEGATIVE\_REGULATION\_OF\_STEROL\_TRANSPORT |  | 7 | 0.85 | 1.79 | 0.002 | 0.107 | 1.000 | 333 | tags=43%, list=3%, signal=44% |
| 101 | GOBP\_ESTABLISHMENT\_OF\_ENDOTHELIAL\_INTESTINAL\_BARRIER |  | 10 | 0.76 | 1.79 | 0.005 | 0.108 | 1.000 | 1875 | tags=50%, list=16%, signal=59% |
| 102 | GOMF\_GLUTAMATE\_RECEPTOR\_ACTIVITY |  | 9 | 0.78 | 1.78 | 0.006 | 0.108 | 1.000 | 1546 | tags=56%, list=13%, signal=64% |
| 103 | GOBP\_PEPTIDE\_SECRETION |  | 262 | 0.40 | 1.78 | 0.000 | 0.108 | 1.000 | 2049 | tags=31%, list=17%, signal=37% |
| 104 | GOBP\_TRIGLYCERIDE\_BIOSYNTHETIC\_PROCESS |  | 30 | 0.58 | 1.78 | 0.004 | 0.107 | 1.000 | 1172 | tags=43%, list=10%, signal=48% |
| 105 | GOCC\_SITE\_OF\_DOUBLE\_STRAND\_BREAK |  | 56 | 0.50 | 1.78 | 0.001 | 0.107 | 1.000 | 3641 | tags=55%, list=30%, signal=79% |
| 106 | GOBP\_REGULATION\_OF\_LIPID\_STORAGE |  | 35 | 0.55 | 1.78 | 0.004 | 0.110 | 1.000 | 1636 | tags=40%, list=14%, signal=46% |
| 107 | GOBP\_FARNESYL\_DIPHOSPHATE\_METABOLIC\_PROCESS |  | 4 | 0.97 | 1.77 | 0.000 | 0.123 | 1.000 | 71 | tags=75%, list=1%, signal=75% |
| 108 | GOBP\_REGULATION\_OF\_CD8\_POSITIVE\_ALPHA\_BETA\_T\_CELL\_ACTIVATION |  | 12 | 0.71 | 1.77 | 0.006 | 0.124 | 1.000 | 822 | tags=42%, list=7%, signal=45% |
| 109 | GOBP\_REGULATION\_OF\_SEQUESTERING\_OF\_TRIGLYCERIDE |  | 9 | 0.77 | 1.77 | 0.005 | 0.123 | 1.000 | 1636 | tags=67%, list=14%, signal=77% |
| 110 | GOBP\_ANTIGEN\_PROCESSING\_AND\_PRESENTATION\_OF\_ENDOGENOUS\_PEPTIDE\_ANTIGEN |  | 6 | 0.86 | 1.77 | 0.002 | 0.123 | 1.000 | 1038 | tags=83%, list=9%, signal=91% |
| 111 | GOCC\_NEUROTRANSMITTER\_RECEPTOR\_COMPLEX |  | 16 | 0.66 | 1.77 | 0.006 | 0.123 | 1.000 | 1780 | tags=44%, list=15%, signal=51% |
| 112 | GOBP\_REGULATION\_OF\_RIG\_I\_SIGNALING\_PATHWAY |  | 12 | 0.71 | 1.77 | 0.007 | 0.122 | 1.000 | 482 | tags=25%, list=4%, signal=26% |
| 113 | GOBP\_INTERFERON\_BETA\_PRODUCTION |  | 36 | 0.54 | 1.77 | 0.002 | 0.124 | 1.000 | 116 | tags=17%, list=1%, signal=17% |
| 114 | GOMF\_QUATERNARY\_AMMONIUM\_GROUP\_TRANSMEMBRANE\_TRANSPORTER\_ACTIVITY |  | 6 | 0.86 | 1.76 | 0.002 | 0.131 | 1.000 | 616 | tags=67%, list=5%, signal=70% |
| 115 | GOBP\_POSITIVE\_REGULATION\_OF\_INSULIN\_SECRETION |  | 44 | 0.52 | 1.76 | 0.003 | 0.138 | 1.000 | 2049 | tags=43%, list=17%, signal=52% |
| 116 | GOBP\_STEROL\_IMPORT |  | 8 | 0.79 | 1.75 | 0.003 | 0.145 | 1.000 | 2049 | tags=75%, list=17%, signal=90% |
| 117 | GOBP\_REGULATION\_OF\_PROTEIN\_LOCALIZATION\_TO\_CELL\_PERIPHERY |  | 86 | 0.46 | 1.75 | 0.001 | 0.150 | 1.000 | 2622 | tags=37%, list=22%, signal=47% |
| 118 | GOMF\_OXIDOREDUCTASE\_ACTIVITY\_ACTING\_ON\_PAIRED\_DONORS\_WITH\_INCORPORATION\_OR\_REDUCTION\_OF\_MOLECULAR\_OXYGEN\_NAD\_P\_H\_AS\_ONE\_DONOR\_AND\_INCORPORATION\_OF\_ONE\_ATOM\_OF\_OXYGEN |  | 23 | 0.60 | 1.75 | 0.005 | 0.149 | 1.000 | 392 | tags=22%, list=3%, signal=22% |
| 119 | GOBP\_REGULATION\_OF\_PATTERN\_RECOGNITION\_RECEPTOR\_SIGNALING\_PATHWAY |  | 62 | 0.49 | 1.74 | 0.001 | 0.153 | 1.000 | 724 | tags=18%, list=6%, signal=19% |
| 120 | GOBP\_REGULATION\_OF\_DEFENSE\_RESPONSE\_TO\_VIRUS\_BY\_HOST |  | 18 | 0.64 | 1.74 | 0.007 | 0.154 | 1.000 | 293 | tags=22%, list=2%, signal=23% |
| 121 | GOBP\_TYPE\_I\_INTERFERON\_PRODUCTION |  | 88 | 0.46 | 1.74 | 0.001 | 0.155 | 1.000 | 981 | tags=19%, list=8%, signal=21% |
| 122 | GOBP\_RESPONSE\_TO\_FRUCTOSE |  | 8 | 0.78 | 1.74 | 0.005 | 0.155 | 1.000 | 1056 | tags=63%, list=9%, signal=68% |
| 123 | GOBP\_NEUTRAL\_LIPID\_BIOSYNTHETIC\_PROCESS |  | 34 | 0.54 | 1.74 | 0.003 | 0.155 | 1.000 | 1172 | tags=41%, list=10%, signal=45% |
| 124 | GOBP\_VENTRICULAR\_CARDIAC\_MUSCLE\_TISSUE\_DEVELOPMENT |  | 31 | 0.55 | 1.74 | 0.005 | 0.160 | 1.000 | 1800 | tags=35%, list=15%, signal=42% |
| 125 | GOBP\_REGULATION\_OF\_SUBSTRATE\_ADHESION\_DEPENDENT\_CELL\_SPREADING |  | 46 | 0.51 | 1.74 | 0.003 | 0.163 | 1.000 | 2440 | tags=37%, list=20%, signal=46% |
| 126 | GOBP\_REGULATION\_OF\_MICROVILLUS\_LENGTH |  | 6 | 0.84 | 1.73 | 0.003 | 0.169 | 1.000 | 1030 | tags=83%, list=9%, signal=91% |
| 127 | GOBP\_CELLULAR\_RESPONSE\_TO\_OXIDISED\_LOW\_DENSITY\_LIPOPROTEIN\_PARTICLE\_STIMULUS |  | 7 | 0.80 | 1.73 | 0.006 | 0.171 | 1.000 | 1326 | tags=43%, list=11%, signal=48% |
| 128 | GOMF\_NADPLUS\_ADP\_RIBOSYLTRANSFERASE\_ACTIVITY |  | 16 | 0.65 | 1.73 | 0.009 | 0.170 | 1.000 | 891 | tags=38%, list=7%, signal=40% |
| 129 | GOBP\_NEGATIVE\_REGULATION\_OF\_TYPE\_I\_INTERFERON\_PRODUCTION |  | 28 | 0.56 | 1.73 | 0.006 | 0.172 | 1.000 | 505 | tags=25%, list=4%, signal=26% |
| 130 | GOBP\_CHYLOMICRON\_ASSEMBLY |  | 7 | 0.81 | 1.73 | 0.005 | 0.174 | 1.000 | 2254 | tags=100%, list=19%, signal=123% |
| 131 | GOBP\_CD8\_POSITIVE\_ALPHA\_BETA\_T\_CELL\_PROLIFERATION |  | 5 | 0.90 | 1.73 | 0.001 | 0.174 | 1.000 | 405 | tags=40%, list=3%, signal=41% |
| 132 | GOBP\_PROTEIN\_ADP\_RIBOSYLATION |  | 23 | 0.59 | 1.72 | 0.007 | 0.175 | 1.000 | 974 | tags=35%, list=8%, signal=38% |
| 133 | GOBP\_HORMONE\_TRANSPORT |  | 203 | 0.40 | 1.72 | 0.000 | 0.175 | 1.000 | 1947 | tags=32%, list=16%, signal=37% |
| 134 | GOBP\_CHOLESTEROL\_EFFLUX |  | 33 | 0.54 | 1.72 | 0.005 | 0.177 | 1.000 | 2049 | tags=39%, list=17%, signal=47% |
| 135 | GOBP\_CELLULAR\_RESPONSE\_TO\_LOW\_DENSITY\_LIPOPROTEIN\_PARTICLE\_STIMULUS |  | 17 | 0.63 | 1.72 | 0.007 | 0.177 | 1.000 | 2169 | tags=53%, list=18%, signal=65% |
| 136 | GOMF\_CCR\_CHEMOKINE\_RECEPTOR\_BINDING |  | 13 | 0.68 | 1.72 | 0.004 | 0.178 | 1.000 | 2056 | tags=69%, list=17%, signal=83% |
| 137 | GOMF\_PROTEIN\_ADP\_RIBOSYLASE\_ACTIVITY |  | 12 | 0.69 | 1.72 | 0.010 | 0.178 | 1.000 | 797 | tags=42%, list=7%, signal=45% |
| 138 | GOBP\_REGULATION\_OF\_PEPTIDE\_HORMONE\_SECRETION |  | 136 | 0.42 | 1.72 | 0.001 | 0.180 | 1.000 | 1856 | tags=32%, list=15%, signal=37% |
| 139 | GOBP\_NEGATIVE\_REGULATION\_OF\_GLIAL\_CELL\_APOPTOTIC\_PROCESS |  | 7 | 0.81 | 1.72 | 0.007 | 0.181 | 1.000 | 1053 | tags=43%, list=9%, signal=47% |
| 140 | GOCC\_MHC\_CLASS\_I\_PEPTIDE\_LOADING\_COMPLEX |  | 6 | 0.85 | 1.72 | 0.003 | 0.180 | 1.000 | 1426 | tags=83%, list=12%, signal=95% |
| 141 | GOBP\_CELLULAR\_RESPONSE\_TO\_COLD |  | 7 | 0.81 | 1.71 | 0.005 | 0.185 | 1.000 | 2101 | tags=57%, list=17%, signal=69% |
| 142 | GOBP\_REGULATION\_OF\_CELL\_PROJECTION\_SIZE |  | 6 | 0.84 | 1.71 | 0.007 | 0.186 | 1.000 | 1030 | tags=83%, list=9%, signal=91% |
| 143 | GOBP\_REGULATION\_OF\_INTESTINAL\_ABSORPTION |  | 7 | 0.80 | 1.71 | 0.006 | 0.186 | 1.000 | 733 | tags=57%, list=6%, signal=61% |
| 144 | GOBP\_REGULATION\_OF\_PROTEIN\_LOCALIZATION\_TO\_PLASMA\_MEMBRANE |  | 70 | 0.47 | 1.71 | 0.002 | 0.185 | 1.000 | 1710 | tags=30%, list=14%, signal=35% |
| 145 | GOBP\_NEGATIVE\_REGULATION\_OF\_AMYLOID\_BETA\_CLEARANCE |  | 6 | 0.84 | 1.71 | 0.005 | 0.185 | 1.000 | 299 | tags=33%, list=2%, signal=34% |
| 146 | GOMF\_PRIMARY\_AMINE\_OXIDASE\_ACTIVITY |  | 5 | 0.89 | 1.71 | 0.003 | 0.187 | 1.000 | 69 | tags=20%, list=1%, signal=20% |
| 147 | GOBP\_TROPHOBLAST\_GIANT\_CELL\_DIFFERENTIATION |  | 8 | 0.77 | 1.71 | 0.004 | 0.186 | 1.000 | 1864 | tags=63%, list=16%, signal=74% |
| 148 | GOBP\_REGULATION\_OF\_LONG\_CHAIN\_FATTY\_ACID\_IMPORT\_INTO\_CELL |  | 6 | 0.83 | 1.71 | 0.007 | 0.185 | 1.000 | 1172 | tags=67%, list=10%, signal=74% |
| 149 | GOCC\_LATERAL\_ELEMENT |  | 7 | 0.79 | 1.71 | 0.005 | 0.186 | 1.000 | 1559 | tags=57%, list=13%, signal=66% |
| 150 | GOBP\_INTESTINAL\_HEXOSE\_ABSORPTION |  | 5 | 0.89 | 1.71 | 0.005 | 0.186 | 1.000 | 1360 | tags=100%, list=11%, signal=113% |
| 151 | GOBP\_POSITIVE\_REGULATION\_OF\_CHOLESTEROL\_STORAGE |  | 5 | 0.88 | 1.71 | 0.003 | 0.191 | 1.000 | 1326 | tags=80%, list=11%, signal=90% |
| 152 | GOBP\_REGULATION\_OF\_INNATE\_IMMUNE\_RESPONSE |  | 177 | 0.40 | 1.71 | 0.000 | 0.191 | 1.000 | 1104 | tags=16%, list=9%, signal=18% |
| 153 | GOBP\_UBIQUITIN\_DEPENDENT\_ENDOCYTOSIS |  | 4 | 0.93 | 1.70 | 0.001 | 0.195 | 1.000 | 79 | tags=25%, list=1%, signal=25% |
| 154 | GOBP\_NEGATIVE\_REGULATION\_OF\_LIPID\_LOCALIZATION |  | 31 | 0.54 | 1.70 | 0.007 | 0.195 | 1.000 | 1636 | tags=32%, list=14%, signal=37% |
| 155 | GOBP\_REGULATION\_OF\_VIRAL\_LIFE\_CYCLE |  | 80 | 0.45 | 1.70 | 0.001 | 0.198 | 1.000 | 1449 | tags=25%, list=12%, signal=28% |
| 156 | GOBP\_ORGANIC\_HYDROXY\_COMPOUND\_BIOSYNTHETIC\_PROCESS |  | 174 | 0.40 | 1.70 | 0.000 | 0.198 | 1.000 | 1424 | tags=25%, list=12%, signal=28% |
| 157 | GOBP\_BILE\_ACID\_SIGNALING\_PATHWAY |  | 9 | 0.74 | 1.70 | 0.011 | 0.199 | 1.000 | 1419 | tags=56%, list=12%, signal=63% |
| 158 | GOBP\_MITOTIC\_SPINDLE\_ASSEMBLY |  | 54 | 0.48 | 1.70 | 0.003 | 0.199 | 1.000 | 3691 | tags=54%, list=31%, signal=77% |
| 159 | GOBP\_STEROID\_METABOLIC\_PROCESS |  | 209 | 0.39 | 1.70 | 0.000 | 0.198 | 1.000 | 1777 | tags=28%, list=15%, signal=33% |
| 160 | GOBP\_REGULATION\_OF\_VIRAL\_GENOME\_REPLICATION |  | 48 | 0.49 | 1.70 | 0.002 | 0.202 | 1.000 | 438 | tags=17%, list=4%, signal=17% |
| 161 | GOBP\_REGULATION\_OF\_MITOTIC\_SISTER\_CHROMATID\_SEGREGATION |  | 39 | 0.51 | 1.70 | 0.006 | 0.201 | 1.000 | 3959 | tags=67%, list=33%, signal=99% |
| 162 | GOBP\_REGULATION\_OF\_INTESTINAL\_CHOLESTEROL\_ABSORPTION |  | 6 | 0.83 | 1.70 | 0.005 | 0.201 | 1.000 | 733 | tags=67%, list=6%, signal=71% |
| 163 | GOCC\_COPI\_VESICLE\_COAT |  | 10 | 0.72 | 1.70 | 0.013 | 0.200 | 1.000 | 3372 | tags=100%, list=28%, signal=139% |
| 164 | GOBP\_REGULATION\_OF\_PHOSPHOLIPID\_CATABOLIC\_PROCESS |  | 4 | 0.93 | 1.69 | 0.000 | 0.201 | 1.000 | 605 | tags=50%, list=5%, signal=53% |
| 165 | GOBP\_POSITIVE\_REGULATION\_OF\_CALCIUM\_ION\_IMPORT |  | 9 | 0.74 | 1.69 | 0.013 | 0.203 | 1.000 | 1710 | tags=44%, list=14%, signal=52% |
| 166 | GOBP\_ACTIN\_CROSSLINK\_FORMATION |  | 11 | 0.70 | 1.69 | 0.011 | 0.209 | 1.000 | 1672 | tags=64%, list=14%, signal=74% |
| 167 | GOMF\_DNA\_HELICASE\_ACTIVITY |  | 59 | 0.47 | 1.69 | 0.003 | 0.211 | 1.000 | 2926 | tags=44%, list=24%, signal=58% |
| 168 | GOBP\_HEMIDESMOSOME\_ASSEMBLY |  | 8 | 0.76 | 1.69 | 0.010 | 0.210 | 1.000 | 1603 | tags=75%, list=13%, signal=86% |
| 169 | GOBP\_RESPONSE\_TO\_INTERFERON\_BETA |  | 14 | 0.65 | 1.69 | 0.015 | 0.209 | 1.000 | 1101 | tags=36%, list=9%, signal=39% |
| 170 | GOBP\_PROTEIN\_LIPID\_COMPLEX\_ASSEMBLY |  | 17 | 0.62 | 1.69 | 0.010 | 0.208 | 1.000 | 2254 | tags=59%, list=19%, signal=72% |
| 171 | GOBP\_CELLULAR\_WATER\_HOMEOSTASIS |  | 5 | 0.88 | 1.69 | 0.001 | 0.208 | 1.000 | 132 | tags=40%, list=1%, signal=40% |
| 172 | GOBP\_POST\_EMBRYONIC\_ANIMAL\_ORGAN\_DEVELOPMENT |  | 10 | 0.71 | 1.69 | 0.010 | 0.207 | 1.000 | 2712 | tags=80%, list=23%, signal=103% |
| 173 | GOMF\_DOUBLE\_STRANDED\_RNA\_BINDING |  | 55 | 0.48 | 1.69 | 0.004 | 0.208 | 1.000 | 1107 | tags=18%, list=9%, signal=20% |
| 174 | GOBP\_PROTEIN\_MONO\_ADP\_RIBOSYLATION |  | 9 | 0.74 | 1.69 | 0.014 | 0.209 | 1.000 | 797 | tags=56%, list=7%, signal=59% |
| 175 | GOBP\_POSITIVE\_REGULATION\_OF\_INTERLEUKIN\_1\_PRODUCTION |  | 25 | 0.57 | 1.68 | 0.010 | 0.210 | 1.000 | 2376 | tags=52%, list=20%, signal=65% |
| 176 | GOBP\_CHYLOMICRON\_REMODELING |  | 4 | 0.92 | 1.68 | 0.001 | 0.216 | 1.000 | 954 | tags=100%, list=8%, signal=109% |
| 177 | GOBP\_PROTEIN\_LOCALIZATION\_TO\_PLASMA\_MEMBRANE |  | 208 | 0.39 | 1.68 | 0.000 | 0.215 | 1.000 | 2961 | tags=35%, list=25%, signal=45% |
| 178 | GOMF\_CALCIUM\_DEPENDENT\_PHOSPHOLIPID\_BINDING |  | 35 | 0.52 | 1.68 | 0.009 | 0.215 | 1.000 | 1858 | tags=43%, list=15%, signal=51% |
| 179 | GOMF\_LIGASE\_ACTIVITY\_FORMING\_CARBON\_SULFUR\_BONDS |  | 28 | 0.55 | 1.68 | 0.008 | 0.216 | 1.000 | 1433 | tags=36%, list=12%, signal=40% |
| 180 | GOBP\_INSULIN\_SECRETION |  | 140 | 0.41 | 1.68 | 0.000 | 0.215 | 1.000 | 1947 | tags=32%, list=16%, signal=38% |
| 181 | GOBP\_REGULATION\_OF\_MESENCHYMAL\_STEM\_CELL\_DIFFERENTIATION |  | 4 | 0.92 | 1.68 | 0.003 | 0.219 | 1.000 | 204 | tags=25%, list=2%, signal=25% |
| 182 | GOBP\_MITOTIC\_SPINDLE\_ORGANIZATION |  | 99 | 0.43 | 1.68 | 0.003 | 0.220 | 1.000 | 3920 | tags=55%, list=33%, signal=80% |
| 183 | GOBP\_REGULATION\_OF\_FEEDING\_BEHAVIOR |  | 12 | 0.68 | 1.68 | 0.010 | 0.222 | 1.000 | 2101 | tags=50%, list=17%, signal=61% |
| 184 | GOCC\_KERATIN\_FILAMENT |  | 9 | 0.73 | 1.68 | 0.014 | 0.222 | 1.000 | 820 | tags=56%, list=7%, signal=60% |
| 185 | GOBP\_ALCOHOL\_METABOLIC\_PROCESS |  | 258 | 0.38 | 1.67 | 0.000 | 0.222 | 1.000 | 1926 | tags=28%, list=16%, signal=33% |
| 186 | GOBP\_REGULATION\_OF\_PHOSPHATIDYLCHOLINE\_METABOLIC\_PROCESS |  | 8 | 0.75 | 1.67 | 0.014 | 0.221 | 1.000 | 1917 | tags=63%, list=16%, signal=74% |
| 187 | GOBP\_LEFT\_RIGHT\_PATTERN\_FORMATION |  | 13 | 0.67 | 1.67 | 0.013 | 0.227 | 1.000 | 63 | tags=15%, list=1%, signal=15% |
| 188 | GOBP\_REGULATION\_OF\_MDA\_5\_SIGNALING\_PATHWAY |  | 6 | 0.82 | 1.67 | 0.008 | 0.230 | 1.000 | 724 | tags=50%, list=6%, signal=53% |
| 189 | GOBP\_CELLULAR\_RESPONSE\_TO\_INTERFERON\_BETA |  | 11 | 0.69 | 1.67 | 0.013 | 0.231 | 1.000 | 114 | tags=27%, list=1%, signal=28% |
| 190 | GOBP\_PROTEIN\_LOCALIZATION\_TO\_CELL\_PERIPHERY |  | 242 | 0.38 | 1.67 | 0.000 | 0.236 | 1.000 | 2698 | tags=32%, list=22%, signal=41% |
| 191 | GOBP\_REGULATION\_OF\_PLASMA\_MEMBRANE\_ORGANIZATION |  | 12 | 0.67 | 1.67 | 0.010 | 0.236 | 1.000 | 957 | tags=50%, list=8%, signal=54% |
| 192 | GOMF\_INOSITOL\_TRISPHOSPHATE\_KINASE\_ACTIVITY |  | 4 | 0.92 | 1.67 | 0.002 | 0.235 | 1.000 | 717 | tags=75%, list=6%, signal=80% |
| 193 | GOCC\_CHYLOMICRON |  | 5 | 0.86 | 1.67 | 0.007 | 0.235 | 1.000 | 954 | tags=80%, list=8%, signal=87% |
| 194 | GOBP\_RECEPTOR\_SIGNALING\_PATHWAY\_VIA\_STAT |  | 93 | 0.43 | 1.67 | 0.002 | 0.234 | 1.000 | 1269 | tags=25%, list=11%, signal=27% |
| 195 | GOBP\_PROGRAMMED\_NECROTIC\_CELL\_DEATH |  | 32 | 0.53 | 1.67 | 0.010 | 0.233 | 1.000 | 769 | tags=25%, list=6%, signal=27% |
| 196 | GOBP\_CELLULAR\_RESPONSE\_TO\_CHOLESTEROL |  | 11 | 0.69 | 1.67 | 0.013 | 0.232 | 1.000 | 855 | tags=27%, list=7%, signal=29% |
| 197 | GOBP\_CARDIAC\_CELL\_FATE\_COMMITMENT |  | 6 | 0.81 | 1.66 | 0.012 | 0.237 | 1.000 | 1394 | tags=67%, list=12%, signal=75% |
| 198 | GOMF\_CYSTEINE\_TYPE\_ENDOPEPTIDASE\_ACTIVITY\_INVOLVED\_IN\_APOPTOTIC\_PROCESS |  | 9 | 0.73 | 1.66 | 0.016 | 0.244 | 1.000 | 2138 | tags=67%, list=18%, signal=81% |
| 199 | GOBP\_BONE\_MINERALIZATION |  | 72 | 0.44 | 1.66 | 0.004 | 0.246 | 1.000 | 2604 | tags=40%, list=22%, signal=51% |
| 200 | GOBP\_REGULATION\_OF\_PLASMA\_LIPOPROTEIN\_PARTICLE\_LEVELS |  | 52 | 0.47 | 1.66 | 0.007 | 0.246 | 1.000 | 1853 | tags=35%, list=15%, signal=41% |
| 201 | GOCC\_FIBRILLAR\_COLLAGEN\_TRIMER |  | 9 | 0.73 | 1.65 | 0.017 | 0.253 | 1.000 | 3082 | tags=78%, list=26%, signal=105% |
| 202 | GOCC\_ER\_TO\_GOLGI\_TRANSPORT\_VESICLE\_MEMBRANE |  | 37 | 0.51 | 1.65 | 0.010 | 0.256 | 1.000 | 1400 | tags=32%, list=12%, signal=37% |
| 203 | GOBP\_CELLULAR\_RESPONSE\_TO\_HYPEROXIA |  | 7 | 0.77 | 1.65 | 0.012 | 0.256 | 1.000 | 2221 | tags=57%, list=18%, signal=70% |
| 204 | GOBP\_HEPATOCYTE\_APOPTOTIC\_PROCESS |  | 12 | 0.66 | 1.65 | 0.020 | 0.259 | 1.000 | 1277 | tags=42%, list=11%, signal=47% |
| 205 | GOBP\_LONG\_CHAIN\_FATTY\_ACID\_TRANSPORT |  | 46 | 0.48 | 1.65 | 0.008 | 0.261 | 1.000 | 2389 | tags=48%, list=20%, signal=59% |
| 206 | GOCC\_MCM\_COMPLEX |  | 9 | 0.72 | 1.65 | 0.019 | 0.262 | 1.000 | 2631 | tags=78%, list=22%, signal=100% |
| 207 | GOBP\_INNATE\_IMMUNE\_RESPONSE |  | 435 | 0.35 | 1.65 | 0.000 | 0.262 | 1.000 | 1104 | tags=16%, list=9%, signal=17% |
| 208 | GOMF\_IONOTROPIC\_GLUTAMATE\_RECEPTOR\_ACTIVITY |  | 8 | 0.75 | 1.65 | 0.015 | 0.266 | 1.000 | 1546 | tags=50%, list=13%, signal=57% |
| 209 | GOBP\_CELL\_CYCLE\_DNA\_REPLICATION |  | 57 | 0.46 | 1.64 | 0.007 | 0.273 | 1.000 | 2926 | tags=47%, list=24%, signal=62% |
| 210 | GOMF\_NMDA\_GLUTAMATE\_RECEPTOR\_ACTIVITY |  | 5 | 0.85 | 1.64 | 0.010 | 0.274 | 1.000 | 1109 | tags=60%, list=9%, signal=66% |
| 211 | GOMF\_RNA\_POLYMERASE\_II\_ACTIVATING\_TRANSCRIPTION\_FACTOR\_BINDING |  | 35 | 0.51 | 1.64 | 0.010 | 0.273 | 1.000 | 1369 | tags=29%, list=11%, signal=32% |
| 212 | GOBP\_REGULATION\_OF\_HORMONE\_SECRETION |  | 166 | 0.39 | 1.64 | 0.001 | 0.274 | 1.000 | 1891 | tags=31%, list=16%, signal=36% |
| 213 | GOMF\_DNA\_REPLICATION\_ORIGIN\_BINDING |  | 19 | 0.59 | 1.64 | 0.013 | 0.273 | 1.000 | 2631 | tags=58%, list=22%, signal=74% |
| 214 | GOMF\_PEPTIDE\_HORMONE\_RECEPTOR\_BINDING |  | 12 | 0.66 | 1.64 | 0.018 | 0.273 | 1.000 | 2311 | tags=50%, list=19%, signal=62% |
| 215 | GOBP\_REGULATION\_OF\_DEFENSE\_RESPONSE\_TO\_VIRUS |  | 38 | 0.50 | 1.64 | 0.010 | 0.278 | 1.000 | 3156 | tags=39%, list=26%, signal=53% |
| 216 | GOBP\_RIG\_I\_SIGNALING\_PATHWAY |  | 15 | 0.63 | 1.64 | 0.018 | 0.277 | 1.000 | 482 | tags=20%, list=4%, signal=21% |
| 217 | GOBP\_EOSINOPHIL\_CHEMOTAXIS |  | 9 | 0.71 | 1.64 | 0.020 | 0.276 | 1.000 | 2056 | tags=78%, list=17%, signal=94% |
| 218 | GOMF\_NEUROPEPTIDE\_HORMONE\_ACTIVITY |  | 10 | 0.69 | 1.64 | 0.015 | 0.276 | 1.000 | 353 | tags=40%, list=3%, signal=41% |
| 219 | GOCC\_COPI\_COATED\_VESICLE |  | 23 | 0.55 | 1.64 | 0.016 | 0.275 | 1.000 | 3372 | tags=65%, list=28%, signal=90% |
| 220 | GOBP\_SISTER\_CHROMATID\_SEGREGATION |  | 159 | 0.39 | 1.64 | 0.001 | 0.280 | 1.000 | 3978 | tags=52%, list=33%, signal=77% |
| 221 | GOCC\_NMDA\_SELECTIVE\_GLUTAMATE\_RECEPTOR\_COMPLEX |  | 5 | 0.85 | 1.63 | 0.010 | 0.283 | 1.000 | 1109 | tags=60%, list=9%, signal=66% |
| 222 | GOBP\_NEGATIVE\_REGULATION\_OF\_TYROSINE\_PHOSPHORYLATION\_OF\_STAT\_PROTEIN |  | 11 | 0.67 | 1.63 | 0.018 | 0.284 | 1.000 | 2209 | tags=55%, list=18%, signal=67% |
| 223 | GOBP\_POSITIVE\_REGULATION\_OF\_INTERLEUKIN\_1\_ALPHA\_PRODUCTION |  | 4 | 0.90 | 1.63 | 0.005 | 0.282 | 1.000 | 613 | tags=75%, list=5%, signal=79% |
| 224 | GOBP\_REGULATION\_OF\_LIPID\_CATABOLIC\_PROCESS |  | 38 | 0.50 | 1.63 | 0.011 | 0.283 | 1.000 | 1873 | tags=42%, list=16%, signal=50% |
| 225 | GOBP\_PATTERN\_RECOGNITION\_RECEPTOR\_SIGNALING\_PATHWAY |  | 135 | 0.40 | 1.63 | 0.001 | 0.282 | 1.000 | 1326 | tags=17%, list=11%, signal=19% |
| 226 | GOBP\_MITOTIC\_CYTOKINESIS |  | 59 | 0.45 | 1.63 | 0.006 | 0.282 | 1.000 | 3045 | tags=46%, list=25%, signal=61% |
| 227 | GOMF\_CALMODULIN\_DEPENDENT\_PROTEIN\_KINASE\_ACTIVITY |  | 22 | 0.56 | 1.63 | 0.013 | 0.284 | 1.000 | 2166 | tags=41%, list=18%, signal=50% |
| 228 | GOBP\_HOMOLOGOUS\_CHROMOSOME\_SEGREGATION |  | 29 | 0.53 | 1.63 | 0.012 | 0.282 | 1.000 | 3684 | tags=59%, list=31%, signal=84% |
| 229 | GOBP\_DETECTION\_OF\_VIRUS |  | 4 | 0.89 | 1.63 | 0.008 | 0.282 | 1.000 | 40 | tags=50%, list=0%, signal=50% |
| 230 | GOBP\_CHOLESTEROL\_STORAGE |  | 14 | 0.64 | 1.63 | 0.020 | 0.280 | 1.000 | 1575 | tags=57%, list=13%, signal=66% |
| 231 | GOBP\_NEGATIVE\_REGULATION\_OF\_EPITHELIAL\_CELL\_DIFFERENTIATION |  | 27 | 0.54 | 1.63 | 0.017 | 0.280 | 1.000 | 1967 | tags=26%, list=16%, signal=31% |
| 232 | GOMF\_ACID\_THIOL\_LIGASE\_ACTIVITY |  | 20 | 0.58 | 1.63 | 0.018 | 0.280 | 1.000 | 1433 | tags=45%, list=12%, signal=51% |
| 233 | GOMF\_INTERFERON\_RECEPTOR\_ACTIVITY |  | 4 | 0.91 | 1.63 | 0.006 | 0.284 | 1.000 | 1101 | tags=75%, list=9%, signal=83% |
| 234 | GOBP\_REGULATION\_OF\_DOUBLE\_STRAND\_BREAK\_REPAIR\_VIA\_NONHOMOLOGOUS\_END\_JOINING |  | 19 | 0.59 | 1.63 | 0.020 | 0.283 | 1.000 | 526 | tags=21%, list=4%, signal=22% |
| 235 | GOBP\_REGULATION\_OF\_GOLGI\_ORGANIZATION |  | 13 | 0.64 | 1.63 | 0.024 | 0.282 | 1.000 | 3330 | tags=62%, list=28%, signal=85% |
| 236 | GOBP\_POSITIVE\_REGULATION\_OF\_HORMONE\_SECRETION |  | 76 | 0.44 | 1.63 | 0.003 | 0.287 | 1.000 | 1856 | tags=34%, list=15%, signal=40% |
| 237 | GOBP\_CENTRIOLE\_ASSEMBLY |  | 34 | 0.51 | 1.63 | 0.009 | 0.288 | 1.000 | 2271 | tags=44%, list=19%, signal=54% |
| 238 | GOCC\_ENDOPLASMIC\_RETICULUM\_GOLGI\_INTERMEDIATE\_COMPARTMENT |  | 101 | 0.41 | 1.62 | 0.002 | 0.292 | 1.000 | 3258 | tags=45%, list=27%, signal=61% |
| 239 | GOMF\_LIPID\_TRANSPORTER\_ACTIVITY |  | 99 | 0.42 | 1.62 | 0.003 | 0.294 | 1.000 | 1960 | tags=32%, list=16%, signal=38% |
| 240 | GOMF\_CGMP\_BINDING |  | 7 | 0.76 | 1.62 | 0.021 | 0.297 | 1.000 | 1155 | tags=29%, list=10%, signal=32% |
| 241 | GOCC\_INTERMEDIATE\_FILAMENT\_CYTOSKELETON |  | 81 | 0.43 | 1.62 | 0.005 | 0.300 | 1.000 | 1173 | tags=20%, list=10%, signal=22% |
| 242 | GOMF\_TAP1\_BINDING |  | 3 | 0.96 | 1.62 | 0.002 | 0.300 | 1.000 | 430 | tags=100%, list=4%, signal=104% |
| 243 | GOBP\_POSITIVE\_REGULATION\_OF\_SUBSTRATE\_ADHESION\_DEPENDENT\_CELL\_SPREADING |  | 33 | 0.51 | 1.62 | 0.013 | 0.299 | 1.000 | 1755 | tags=30%, list=15%, signal=35% |
| 244 | GOBP\_CD8\_POSITIVE\_ALPHA\_BETA\_T\_CELL\_ACTIVATION |  | 16 | 0.60 | 1.62 | 0.024 | 0.298 | 1.000 | 822 | tags=31%, list=7%, signal=34% |
| 245 | GOMF\_CHEMOKINE\_RECEPTOR\_BINDING |  | 19 | 0.58 | 1.62 | 0.020 | 0.298 | 1.000 | 2056 | tags=53%, list=17%, signal=63% |
| 246 | GOBP\_REGULATION\_OF\_CELL\_MIGRATION\_INVOLVED\_IN\_SPROUTING\_ANGIOGENESIS |  | 30 | 0.52 | 1.62 | 0.014 | 0.301 | 1.000 | 2918 | tags=47%, list=24%, signal=61% |
| 247 | GOBP\_NOTOCHORD\_DEVELOPMENT |  | 12 | 0.65 | 1.62 | 0.023 | 0.301 | 1.000 | 3037 | tags=50%, list=25%, signal=67% |
| 248 | GOBP\_HOMOTYPIC\_CELL\_CELL\_ADHESION |  | 58 | 0.45 | 1.62 | 0.008 | 0.306 | 1.000 | 1234 | tags=24%, list=10%, signal=27% |
| 249 | GOBP\_EOSINOPHIL\_MIGRATION |  | 11 | 0.67 | 1.62 | 0.021 | 0.306 | 1.000 | 2056 | tags=64%, list=17%, signal=77% |
| 250 | GOBP\_REGULATION\_OF\_TOLL\_LIKE\_RECEPTOR\_SIGNALING\_PATHWAY |  | 45 | 0.47 | 1.61 | 0.010 | 0.306 | 1.000 | 623 | tags=18%, list=5%, signal=19% |
| 251 | GOMF\_TAP\_BINDING |  | 3 | 0.96 | 1.61 | 0.001 | 0.307 | 1.000 | 430 | tags=100%, list=4%, signal=104% |
| 252 | GOCC\_CONDENSED\_NUCLEAR\_CHROMOSOME\_KINETOCHORE |  | 13 | 0.64 | 1.61 | 0.027 | 0.308 | 1.000 | 3700 | tags=85%, list=31%, signal=122% |
| 253 | GOBP\_NUCLEAR\_CHROMOSOME\_SEGREGATION |  | 200 | 0.37 | 1.61 | 0.000 | 0.310 | 1.000 | 3978 | tags=50%, list=33%, signal=73% |
| 254 | GOCC\_APICAL\_JUNCTION\_COMPLEX |  | 97 | 0.41 | 1.61 | 0.002 | 0.309 | 1.000 | 2375 | tags=32%, list=20%, signal=40% |
| 255 | GOBP\_POSITIVE\_REGULATION\_OF\_DOUBLE\_STRAND\_BREAK\_REPAIR |  | 29 | 0.52 | 1.61 | 0.017 | 0.310 | 1.000 | 3329 | tags=52%, list=28%, signal=71% |
| 256 | GOBP\_RESPONSE\_TO\_INTERFERON\_ALPHA |  | 11 | 0.67 | 1.61 | 0.025 | 0.309 | 1.000 | 1101 | tags=45%, list=9%, signal=50% |
| 257 | GOBP\_REGULATION\_OF\_STEROL\_TRANSPORT |  | 35 | 0.50 | 1.61 | 0.018 | 0.309 | 1.000 | 2049 | tags=31%, list=17%, signal=38% |
| 258 | GOMF\_DEAD\_H\_BOX\_RNA\_HELICASE\_BINDING |  | 7 | 0.75 | 1.61 | 0.021 | 0.309 | 1.000 | 959 | tags=43%, list=8%, signal=47% |
| 259 | GOBP\_CHROMATIN\_REMODELING\_AT\_CENTROMERE |  | 31 | 0.51 | 1.61 | 0.011 | 0.309 | 1.000 | 3970 | tags=68%, list=33%, signal=101% |
| 260 | GOCC\_NUCLEAR\_REPLICATION\_FORK |  | 27 | 0.53 | 1.61 | 0.019 | 0.308 | 1.000 | 1557 | tags=30%, list=13%, signal=34% |
| 261 | GOBP\_METAPHASE\_PLATE\_CONGRESSION |  | 53 | 0.46 | 1.61 | 0.010 | 0.309 | 1.000 | 3934 | tags=60%, list=33%, signal=89% |
| 262 | GOBP\_RESPONSE\_TO\_CISPLATIN |  | 6 | 0.79 | 1.61 | 0.015 | 0.314 | 1.000 | 2359 | tags=67%, list=20%, signal=83% |
| 263 | GOBP\_CHONDROCYTE\_DIFFERENTIATION |  | 73 | 0.43 | 1.61 | 0.006 | 0.316 | 1.000 | 2712 | tags=44%, list=23%, signal=56% |
| 264 | GOBP\_MITOTIC\_DNA\_REPLICATION |  | 14 | 0.62 | 1.60 | 0.026 | 0.324 | 1.000 | 2631 | tags=64%, list=22%, signal=82% |
| 265 | GOMF\_PROTEIN\_SERINE\_THREONINE\_KINASE\_INHIBITOR\_ACTIVITY |  | 22 | 0.55 | 1.60 | 0.024 | 0.331 | 1.000 | 2376 | tags=41%, list=20%, signal=51% |
| 266 | GOCC\_INTEGRAL\_COMPONENT\_OF\_PRESYNAPTIC\_ACTIVE\_ZONE\_MEMBRANE |  | 10 | 0.67 | 1.60 | 0.030 | 0.334 | 1.000 | 382 | tags=30%, list=3%, signal=31% |
| 267 | GOBP\_CELLULAR\_RESPONSE\_TO\_MINERALOCORTICOID\_STIMULUS |  | 5 | 0.82 | 1.60 | 0.018 | 0.334 | 1.000 | 485 | tags=40%, list=4%, signal=42% |
| 268 | GOMF\_EXTRACELLULAR\_MATRIX\_STRUCTURAL\_CONSTITUENT |  | 107 | 0.40 | 1.60 | 0.000 | 0.333 | 1.000 | 3750 | tags=55%, list=31%, signal=79% |
| 269 | GOMF\_LIPASE\_ACTIVITY |  | 80 | 0.42 | 1.60 | 0.006 | 0.333 | 1.000 | 1752 | tags=36%, list=15%, signal=42% |
| 270 | GOBP\_DNA\_REPLICATION |  | 232 | 0.36 | 1.60 | 0.000 | 0.335 | 1.000 | 2631 | tags=32%, list=22%, signal=41% |
| 271 | GOBP\_BLEB\_ASSEMBLY |  | 9 | 0.70 | 1.60 | 0.029 | 0.335 | 1.000 | 483 | tags=22%, list=4%, signal=23% |
| 272 | GOBP\_POSITIVE\_REGULATION\_OF\_LIPID\_CATABOLIC\_PROCESS |  | 16 | 0.59 | 1.60 | 0.023 | 0.335 | 1.000 | 1693 | tags=56%, list=14%, signal=65% |
| 273 | GOBP\_REGULATION\_OF\_RESPONSE\_TO\_BIOTIC\_STIMULUS |  | 237 | 0.36 | 1.60 | 0.001 | 0.334 | 1.000 | 1137 | tags=15%, list=9%, signal=16% |
| 274 | GOBP\_CHEMOREPULSION\_OF\_AXON |  | 4 | 0.87 | 1.60 | 0.016 | 0.335 | 1.000 | 307 | tags=50%, list=3%, signal=51% |
| 275 | GOMF\_GLUCOSE\_SODIUM\_SYMPORTER\_ACTIVITY |  | 4 | 0.88 | 1.60 | 0.010 | 0.334 | 1.000 | 1437 | tags=100%, list=12%, signal=114% |
| 276 | GOCC\_CELL\_CELL\_JUNCTION |  | 338 | 0.35 | 1.60 | 0.000 | 0.335 | 1.000 | 2614 | tags=31%, list=22%, signal=39% |
| 277 | GOBP\_POSITIVE\_REGULATION\_OF\_TRIGLYCERIDE\_METABOLIC\_PROCESS |  | 15 | 0.61 | 1.59 | 0.030 | 0.337 | 1.000 | 733 | tags=47%, list=6%, signal=50% |
| 278 | GOBP\_MICROTUBULE\_CYTOSKELETON\_ORGANIZATION\_INVOLVED\_IN\_MITOSIS |  | 121 | 0.39 | 1.59 | 0.002 | 0.337 | 1.000 | 3859 | tags=52%, list=32%, signal=76% |
| 279 | GOBP\_NEGATIVE\_REGULATION\_OF\_RECEPTOR\_SIGNALING\_PATHWAY\_VIA\_JAK\_STAT |  | 20 | 0.56 | 1.59 | 0.024 | 0.337 | 1.000 | 686 | tags=25%, list=6%, signal=26% |
| 280 | GOBP\_FAT\_PAD\_DEVELOPMENT |  | 5 | 0.83 | 1.59 | 0.016 | 0.337 | 1.000 | 1832 | tags=80%, list=15%, signal=94% |
| 281 | GOCC\_CLATHRIN\_COATED\_ENDOCYTIC\_VESICLE\_MEMBRANE |  | 17 | 0.58 | 1.59 | 0.023 | 0.336 | 1.000 | 1107 | tags=24%, list=9%, signal=26% |
| 282 | GOBP\_CHONDROCYTE\_DEVELOPMENT\_INVOLVED\_IN\_ENDOCHONDRAL\_BONE\_MORPHOGENESIS |  | 6 | 0.79 | 1.59 | 0.021 | 0.335 | 1.000 | 2420 | tags=67%, list=20%, signal=83% |
| 283 | GOBP\_FEMALE\_MEIOTIC\_NUCLEAR\_DIVISION |  | 16 | 0.59 | 1.59 | 0.023 | 0.336 | 1.000 | 3935 | tags=63%, list=33%, signal=93% |
| 284 | GOBP\_EMBRYONIC\_HINDLIMB\_MORPHOGENESIS |  | 15 | 0.61 | 1.59 | 0.028 | 0.335 | 1.000 | 5 | tags=13%, list=0%, signal=13% |
| 285 | GOBP\_DOUBLE\_STRAND\_BREAK\_REPAIR\_VIA\_BREAK\_INDUCED\_REPLICATION |  | 9 | 0.69 | 1.59 | 0.023 | 0.335 | 1.000 | 2631 | tags=89%, list=22%, signal=114% |
| 286 | GOBP\_PROTEIN\_KINASE\_A\_SIGNALING |  | 22 | 0.55 | 1.59 | 0.023 | 0.336 | 1.000 | 1049 | tags=32%, list=9%, signal=35% |
| 287 | GOMF\_NEUROPEPTIDE\_RECEPTOR\_BINDING |  | 16 | 0.60 | 1.59 | 0.025 | 0.339 | 1.000 | 353 | tags=31%, list=3%, signal=32% |
| 288 | GOBP\_REGULATION\_OF\_LONG\_CHAIN\_FATTY\_ACID\_IMPORT\_ACROSS\_PLASMA\_MEMBRANE |  | 5 | 0.82 | 1.59 | 0.018 | 0.338 | 1.000 | 1172 | tags=60%, list=10%, signal=66% |
| 289 | GOBP\_CENTROMERE\_COMPLEX\_ASSEMBLY |  | 39 | 0.48 | 1.59 | 0.017 | 0.339 | 1.000 | 3970 | tags=62%, list=33%, signal=92% |
| 290 | GOBP\_REGULATION\_OF\_DOUBLE\_STRAND\_BREAK\_REPAIR |  | 60 | 0.44 | 1.59 | 0.012 | 0.340 | 1.000 | 3537 | tags=45%, list=29%, signal=63% |
| 291 | GOBP\_NEGATIVE\_REGULATION\_OF\_METAPHASE\_ANAPHASE\_TRANSITION\_OF\_CELL\_CYCLE |  | 36 | 0.49 | 1.59 | 0.018 | 0.339 | 1.000 | 3959 | tags=64%, list=33%, signal=95% |
| 292 | GOBP\_ACTIN\_POLYMERIZATION\_OR\_DEPOLYMERIZATION |  | 150 | 0.39 | 1.59 | 0.003 | 0.341 | 1.000 | 2183 | tags=32%, list=18%, signal=39% |
| 293 | GOBP\_REGULATION\_OF\_SPINDLE\_ORGANIZATION |  | 32 | 0.50 | 1.59 | 0.015 | 0.342 | 1.000 | 3803 | tags=56%, list=32%, signal=82% |
| 294 | GOMF\_NEUREXIN\_FAMILY\_PROTEIN\_BINDING |  | 8 | 0.72 | 1.59 | 0.030 | 0.343 | 1.000 | 1206 | tags=50%, list=10%, signal=56% |
| 295 | GOBP\_NEGATIVE\_REGULATION\_OF\_ACTIVATED\_T\_CELL\_PROLIFERATION |  | 8 | 0.71 | 1.59 | 0.029 | 0.343 | 1.000 | 2457 | tags=75%, list=20%, signal=94% |
| 296 | GOCC\_ATPASE\_DEPENDENT\_TRANSMEMBRANE\_TRANSPORT\_COMPLEX |  | 10 | 0.67 | 1.58 | 0.031 | 0.348 | 1.000 | 725 | tags=30%, list=6%, signal=32% |
| 297 | GOBP\_LIPID\_DROPLET\_ORGANIZATION |  | 17 | 0.58 | 1.58 | 0.028 | 0.349 | 1.000 | 1730 | tags=35%, list=14%, signal=41% |
| 298 | GOBP\_LONG\_CHAIN\_FATTY\_ACYL\_COA\_METABOLIC\_PROCESS |  | 20 | 0.56 | 1.58 | 0.027 | 0.348 | 1.000 | 1433 | tags=40%, list=12%, signal=45% |
| 299 | GOMF\_CELL\_ADHESIVE\_PROTEIN\_BINDING\_INVOLVED\_IN\_BUNDLE\_OF\_HIS\_CELL\_PURKINJE\_MYOCYTE\_COMMUNICATION |  | 4 | 0.87 | 1.58 | 0.015 | 0.350 | 1.000 | 985 | tags=75%, list=8%, signal=82% |
| 300 | GOBP\_REGULATION\_OF\_POLYSACCHARIDE\_BIOSYNTHETIC\_PROCESS |  | 24 | 0.53 | 1.58 | 0.028 | 0.353 | 1.000 | 2440 | tags=54%, list=20%, signal=68% |
| 301 | GOBP\_REGULATION\_OF\_HEMOGLOBIN\_BIOSYNTHETIC\_PROCESS |  | 5 | 0.82 | 1.58 | 0.024 | 0.353 | 1.000 | 997 | tags=60%, list=8%, signal=65% |
| 302 | GOBP\_NEGATIVE\_REGULATION\_OF\_RESPONSE\_TO\_CYTOKINE\_STIMULUS |  | 38 | 0.48 | 1.58 | 0.016 | 0.356 | 1.000 | 1219 | tags=29%, list=10%, signal=32% |
| 303 | GOMF\_PLATELET\_DERIVED\_GROWTH\_FACTOR\_BINDING |  | 11 | 0.64 | 1.58 | 0.032 | 0.358 | 1.000 | 3082 | tags=55%, list=26%, signal=73% |
| 304 | GOBP\_LONG\_CHAIN\_FATTY\_ACYL\_COA\_BIOSYNTHETIC\_PROCESS |  | 16 | 0.59 | 1.58 | 0.031 | 0.358 | 1.000 | 2559 | tags=50%, list=21%, signal=63% |
| 305 | GOBP\_REGULATION\_OF\_LIPID\_LOCALIZATION |  | 111 | 0.40 | 1.58 | 0.004 | 0.358 | 1.000 | 2113 | tags=31%, list=18%, signal=37% |
| 306 | GOBP\_NEGATIVE\_REGULATION\_OF\_DIGESTIVE\_SYSTEM\_PROCESS |  | 8 | 0.72 | 1.58 | 0.024 | 0.358 | 1.000 | 132 | tags=38%, list=1%, signal=38% |
| 307 | GOMF\_PEPTIDE\_ANTIGEN\_BINDING |  | 6 | 0.77 | 1.58 | 0.023 | 0.358 | 1.000 | 357 | tags=50%, list=3%, signal=52% |
| 308 | GOBP\_CELLULAR\_RESPONSE\_TO\_EXOGENOUS\_DSRNA |  | 8 | 0.70 | 1.58 | 0.030 | 0.358 | 1.000 | 1083 | tags=38%, list=9%, signal=41% |
| 309 | GOCC\_ENDOPLASMIC\_RETICULUM\_PLASMA\_MEMBRANE\_CONTACT\_SITE |  | 8 | 0.71 | 1.58 | 0.036 | 0.357 | 1.000 | 1520 | tags=50%, list=13%, signal=57% |
| 310 | GOMF\_DOUBLE\_STRANDED\_TELOMERIC\_DNA\_BINDING |  | 8 | 0.71 | 1.58 | 0.031 | 0.356 | 1.000 | 983 | tags=50%, list=8%, signal=54% |
| 311 | GOBP\_REGULATION\_OF\_SMALL\_MOLECULE\_METABOLIC\_PROCESS |  | 309 | 0.35 | 1.58 | 0.000 | 0.355 | 1.000 | 1693 | tags=22%, list=14%, signal=25% |
| 312 | GOBP\_SEQUESTERING\_OF\_TRIGLYCERIDE |  | 10 | 0.67 | 1.58 | 0.023 | 0.355 | 1.000 | 1636 | tags=60%, list=14%, signal=69% |
| 313 | GOBP\_INTERLEUKIN\_35\_MEDIATED\_SIGNALING\_PATHWAY |  | 8 | 0.71 | 1.58 | 0.032 | 0.354 | 1.000 | 2301 | tags=63%, list=19%, signal=77% |
| 314 | GOBP\_ANTIGEN\_PROCESSING\_AND\_PRESENTATION\_OF\_ENDOGENOUS\_ANTIGEN |  | 10 | 0.66 | 1.58 | 0.029 | 0.354 | 1.000 | 1038 | tags=50%, list=9%, signal=55% |
| 315 | GOBP\_RESPONSE\_TO\_OLEIC\_ACID |  | 5 | 0.81 | 1.57 | 0.021 | 0.355 | 1.000 | 1172 | tags=60%, list=10%, signal=66% |
| 316 | GOMF\_CADHERIN\_BINDING\_INVOLVED\_IN\_CELL\_CELL\_ADHESION |  | 15 | 0.60 | 1.57 | 0.032 | 0.356 | 1.000 | 2578 | tags=60%, list=21%, signal=76% |
| 317 | GOMF\_CYCLIC\_NUCLEOTIDE\_BINDING |  | 22 | 0.54 | 1.57 | 0.024 | 0.365 | 1.000 | 1190 | tags=32%, list=10%, signal=35% |
| 318 | GOCC\_ACTIN\_FILAMENT\_BUNDLE |  | 56 | 0.44 | 1.57 | 0.011 | 0.364 | 1.000 | 2340 | tags=38%, list=19%, signal=46% |
| 319 | GOBP\_NECROPTOTIC\_PROCESS |  | 28 | 0.52 | 1.57 | 0.023 | 0.365 | 1.000 | 769 | tags=25%, list=6%, signal=27% |
| 320 | GOBP\_NEGATIVE\_REGULATION\_OF\_DEFENSE\_RESPONSE |  | 114 | 0.39 | 1.57 | 0.003 | 0.365 | 1.000 | 1223 | tags=20%, list=10%, signal=22% |
| 321 | GOBP\_DNA\_DEPENDENT\_DNA\_REPLICATION |  | 135 | 0.38 | 1.57 | 0.002 | 0.367 | 1.000 | 2413 | tags=34%, list=20%, signal=42% |
| 322 | GOBP\_ENDOPLASMIC\_RETICULUM\_LOCALIZATION |  | 4 | 0.86 | 1.57 | 0.019 | 0.368 | 1.000 | 1225 | tags=50%, list=10%, signal=56% |
| 323 | GOBP\_GLIAL\_CELL\_APOPTOTIC\_PROCESS |  | 12 | 0.64 | 1.57 | 0.031 | 0.374 | 1.000 | 1333 | tags=33%, list=11%, signal=37% |
| 324 | GOBP\_RESPONSE\_TO\_MINERALOCORTICOID |  | 18 | 0.58 | 1.57 | 0.029 | 0.373 | 1.000 | 1397 | tags=33%, list=12%, signal=38% |
| 325 | GOBP\_MAST\_CELL\_PROLIFERATION |  | 5 | 0.82 | 1.56 | 0.022 | 0.381 | 1.000 | 623 | tags=40%, list=5%, signal=42% |
| 326 | GOCC\_DENDRITIC\_SPINE\_HEAD |  | 5 | 0.81 | 1.56 | 0.023 | 0.383 | 1.000 | 1505 | tags=80%, list=13%, signal=91% |
| 327 | GOBP\_KERATINOCYTE\_DEVELOPMENT |  | 5 | 0.81 | 1.56 | 0.033 | 0.386 | 1.000 | 604 | tags=40%, list=5%, signal=42% |
| 328 | GOBP\_POSITIVE\_REGULATION\_OF\_CHOLESTEROL\_ESTERIFICATION |  | 4 | 0.87 | 1.56 | 0.017 | 0.386 | 1.000 | 733 | tags=75%, list=6%, signal=80% |
| 329 | GOBP\_DNA\_DEPENDENT\_DNA\_REPLICATION\_MAINTENANCE\_OF\_FIDELITY |  | 39 | 0.48 | 1.56 | 0.015 | 0.387 | 1.000 | 2359 | tags=41%, list=20%, signal=51% |
| 330 | GOMF\_TUMOR\_NECROSIS\_FACTOR\_RECEPTOR\_SUPERFAMILY\_BINDING |  | 32 | 0.49 | 1.56 | 0.025 | 0.388 | 1.000 | 769 | tags=19%, list=6%, signal=20% |
| 331 | GOBP\_CYTOPLASMIC\_PATTERN\_RECOGNITION\_RECEPTOR\_SIGNALING\_PATHWAY |  | 48 | 0.45 | 1.56 | 0.014 | 0.387 | 1.000 | 724 | tags=15%, list=6%, signal=15% |
| 332 | GOMF\_CYSTEINE\_TYPE\_ENDOPEPTIDASE\_ACTIVITY\_INVOLVED\_IN\_EXECUTION\_PHASE\_OF\_APOPTOSIS |  | 8 | 0.70 | 1.56 | 0.035 | 0.389 | 1.000 | 2138 | tags=63%, list=18%, signal=76% |
| 333 | GOCC\_CONDENSIN\_COMPLEX |  | 8 | 0.71 | 1.56 | 0.038 | 0.395 | 1.000 | 3251 | tags=88%, list=27%, signal=120% |
| 334 | GOBP\_HIGH\_DENSITY\_LIPOPROTEIN\_PARTICLE\_CLEARANCE |  | 6 | 0.76 | 1.56 | 0.035 | 0.395 | 1.000 | 1763 | tags=50%, list=15%, signal=59% |
| 335 | GOBP\_MAMMARY\_GLAND\_FORMATION |  | 6 | 0.77 | 1.55 | 0.027 | 0.401 | 1.000 | 2351 | tags=83%, list=20%, signal=104% |
| 336 | GOBP\_ACTIN\_FILAMENT\_REORGANIZATION |  | 7 | 0.73 | 1.55 | 0.043 | 0.403 | 1.000 | 957 | tags=57%, list=8%, signal=62% |
| 337 | GOBP\_MICROVILLUS\_ASSEMBLY |  | 12 | 0.63 | 1.55 | 0.037 | 0.404 | 1.000 | 2787 | tags=67%, list=23%, signal=87% |
| 338 | GOBP\_RESPONSE\_TO\_PURINE\_CONTAINING\_COMPOUND |  | 94 | 0.40 | 1.55 | 0.010 | 0.405 | 1.000 | 1419 | tags=27%, list=12%, signal=30% |
| 339 | GOBP\_ENDOPLASMIC\_RETICULUM\_PLASMA\_MEMBRANE\_TETHERING |  | 4 | 0.86 | 1.55 | 0.019 | 0.405 | 1.000 | 1225 | tags=50%, list=10%, signal=56% |
| 340 | GOCC\_ENDOCYTIC\_VESICLE\_LUMEN |  | 10 | 0.66 | 1.55 | 0.039 | 0.405 | 1.000 | 2576 | tags=60%, list=21%, signal=76% |
| 341 | GOBP\_REGULATION\_OF\_MITOTIC\_CENTROSOME\_SEPARATION |  | 9 | 0.67 | 1.55 | 0.043 | 0.405 | 1.000 | 2633 | tags=44%, list=22%, signal=57% |
| 342 | GOBP\_ORGANIC\_HYDROXY\_COMPOUND\_TRANSPORT |  | 168 | 0.37 | 1.55 | 0.002 | 0.407 | 1.000 | 2049 | tags=30%, list=17%, signal=36% |
| 343 | GOCC\_DENDRITE\_TERMINUS |  | 11 | 0.64 | 1.55 | 0.035 | 0.406 | 1.000 | 903 | tags=27%, list=8%, signal=29% |
| 344 | GOBP\_POSTSYNAPTIC\_NEUROTRANSMITTER\_RECEPTOR\_INTERNALIZATION |  | 10 | 0.66 | 1.55 | 0.034 | 0.406 | 1.000 | 2044 | tags=40%, list=17%, signal=48% |
| 345 | GOBP\_REGULATION\_OF\_CARDIAC\_MUSCLE\_CELL\_ACTION\_POTENTIAL |  | 18 | 0.57 | 1.55 | 0.035 | 0.407 | 1.000 | 987 | tags=33%, list=8%, signal=36% |
| 346 | GOBP\_PROTEIN\_LOCALIZATION\_TO\_EARLY\_ENDOSOME |  | 7 | 0.73 | 1.55 | 0.038 | 0.408 | 1.000 | 1922 | tags=57%, list=16%, signal=68% |
| 347 | GOCC\_LIPID\_DROPLET |  | 66 | 0.42 | 1.55 | 0.009 | 0.407 | 1.000 | 1519 | tags=32%, list=13%, signal=36% |
| 348 | GOBP\_BILE\_ACID\_SECRETION |  | 7 | 0.73 | 1.55 | 0.042 | 0.407 | 1.000 | 1419 | tags=57%, list=12%, signal=65% |
| 349 | GOBP\_METAPHASE\_ANAPHASE\_TRANSITION\_OF\_CELL\_CYCLE |  | 56 | 0.44 | 1.55 | 0.018 | 0.407 | 1.000 | 3959 | tags=57%, list=33%, signal=85% |
| 350 | GOCC\_AXON\_INITIAL\_SEGMENT |  | 10 | 0.66 | 1.55 | 0.043 | 0.406 | 1.000 | 3647 | tags=70%, list=30%, signal=100% |
| 351 | GOBP\_METANEPHRIC\_GLOMERULUS\_DEVELOPMENT |  | 11 | 0.64 | 1.55 | 0.035 | 0.409 | 1.000 | 2683 | tags=55%, list=22%, signal=70% |
| 352 | GOBP\_COMPLEMENT\_ACTIVATION\_LECTIN\_PATHWAY |  | 5 | 0.80 | 1.55 | 0.029 | 0.410 | 1.000 | 42 | tags=20%, list=0%, signal=20% |
| 353 | GOBP\_NEGATIVE\_REGULATION\_OF\_BEHAVIOR |  | 7 | 0.73 | 1.55 | 0.038 | 0.412 | 1.000 | 1771 | tags=57%, list=15%, signal=67% |
| 354 | GOBP\_CELLULAR\_RESPONSE\_TO\_CISPLATIN |  | 5 | 0.80 | 1.54 | 0.027 | 0.414 | 1.000 | 2359 | tags=80%, list=20%, signal=100% |
| 355 | GOMF\_RECEPTOR\_REGULATOR\_ACTIVITY |  | 206 | 0.36 | 1.54 | 0.001 | 0.417 | 1.000 | 2393 | tags=34%, list=20%, signal=42% |
| 356 | GOBP\_RESPONSE\_TO\_FATTY\_ACID |  | 40 | 0.47 | 1.54 | 0.020 | 0.416 | 1.000 | 1326 | tags=30%, list=11%, signal=34% |
| 357 | GOBP\_DEFENSE\_RESPONSE\_TO\_OTHER\_ORGANISM |  | 534 | 0.33 | 1.54 | 0.000 | 0.416 | 1.000 | 1137 | tags=15%, list=9%, signal=16% |
| 358 | GOBP\_GLYCOPROTEIN\_CATABOLIC\_PROCESS |  | 17 | 0.57 | 1.54 | 0.037 | 0.417 | 1.000 | 281 | tags=24%, list=2%, signal=24% |
| 359 | GOBP\_GONADOTROPIN\_SECRETION |  | 7 | 0.72 | 1.54 | 0.041 | 0.417 | 1.000 | 2275 | tags=71%, list=19%, signal=88% |
| 360 | GOBP\_FOLLICLE\_STIMULATING\_HORMONE\_SECRETION |  | 3 | 0.91 | 1.54 | 0.014 | 0.417 | 1.000 | 855 | tags=67%, list=7%, signal=72% |
| 361 | GOBP\_ANTERIOR\_POSTERIOR\_AXON\_GUIDANCE |  | 3 | 0.92 | 1.54 | 0.015 | 0.418 | 1.000 | 307 | tags=33%, list=3%, signal=34% |
| 362 | GOMF\_U2\_SNRNA\_BINDING |  | 4 | 0.85 | 1.54 | 0.024 | 0.418 | 1.000 | 1118 | tags=50%, list=9%, signal=55% |
| 363 | GOBP\_ISOPRENOID\_METABOLIC\_PROCESS |  | 84 | 0.40 | 1.54 | 0.010 | 0.419 | 1.000 | 795 | tags=23%, list=7%, signal=24% |
| 364 | GOMF\_HELICASE\_ACTIVITY |  | 118 | 0.38 | 1.54 | 0.006 | 0.419 | 1.000 | 2938 | tags=31%, list=24%, signal=41% |
| 365 | GOBP\_PARALLEL\_ACTIN\_FILAMENT\_BUNDLE\_ASSEMBLY |  | 4 | 0.84 | 1.54 | 0.028 | 0.422 | 1.000 | 872 | tags=50%, list=7%, signal=54% |
| 366 | GOMF\_RNA\_HELICASE\_ACTIVITY |  | 54 | 0.44 | 1.54 | 0.014 | 0.424 | 1.000 | 116 | tags=9%, list=1%, signal=9% |
| 367 | GOBP\_REGULATION\_OF\_POLYSACCHARIDE\_METABOLIC\_PROCESS |  | 29 | 0.50 | 1.54 | 0.031 | 0.425 | 1.000 | 2440 | tags=48%, list=20%, signal=60% |
| 368 | GOMF\_STRUCTURAL\_CONSTITUENT\_OF\_SYNAPSE |  | 8 | 0.69 | 1.54 | 0.042 | 0.424 | 1.000 | 1649 | tags=50%, list=14%, signal=58% |
| 369 | GOBP\_RESPONSE\_TO\_ACTIVITY |  | 48 | 0.44 | 1.54 | 0.019 | 0.424 | 1.000 | 1587 | tags=25%, list=13%, signal=29% |
| 370 | GOBP\_NEGATIVE\_REGULATION\_OF\_ACTIN\_FILAMENT\_DEPOLYMERIZATION |  | 33 | 0.48 | 1.54 | 0.031 | 0.423 | 1.000 | 2090 | tags=36%, list=17%, signal=44% |
| 371 | GOBP\_NEGATIVE\_REGULATION\_OF\_FEEDING\_BEHAVIOR |  | 5 | 0.80 | 1.54 | 0.034 | 0.424 | 1.000 | 1771 | tags=60%, list=15%, signal=70% |
| 372 | GOCC\_PRESYNAPTIC\_ACTIVE\_ZONE\_CYTOPLASMIC\_COMPONENT |  | 10 | 0.65 | 1.54 | 0.047 | 0.424 | 1.000 | 2339 | tags=60%, list=19%, signal=74% |
| 373 | GOBP\_NEGATIVE\_REGULATION\_OF\_IMMUNE\_SYSTEM\_PROCESS |  | 232 | 0.35 | 1.53 | 0.001 | 0.426 | 1.000 | 1140 | tags=18%, list=9%, signal=19% |
| 374 | GOMF\_PHOSPHOLIPASE\_ACTIVITY |  | 68 | 0.42 | 1.53 | 0.016 | 0.425 | 1.000 | 1752 | tags=35%, list=15%, signal=41% |
| 375 | GOBP\_MYD88\_INDEPENDENT\_TOLL\_LIKE\_RECEPTOR\_SIGNALING\_PATHWAY |  | 26 | 0.51 | 1.53 | 0.034 | 0.425 | 1.000 | 1693 | tags=27%, list=14%, signal=31% |
| 376 | GOMF\_CALCIUM\_DEPENDENT\_CYSTEINE\_TYPE\_ENDOPEPTIDASE\_ACTIVITY |  | 11 | 0.63 | 1.53 | 0.049 | 0.425 | 1.000 | 2231 | tags=64%, list=19%, signal=78% |
| 377 | GOBP\_LYMPHOCYTE\_CHEMOTAXIS |  | 27 | 0.51 | 1.53 | 0.032 | 0.424 | 1.000 | 2056 | tags=44%, list=17%, signal=54% |
| 378 | GOBP\_PROTON\_TRANSPORTING\_V\_TYPE\_ATPASE\_COMPLEX\_ASSEMBLY |  | 5 | 0.80 | 1.53 | 0.029 | 0.424 | 1.000 | 284 | tags=40%, list=2%, signal=41% |
| 379 | GOBP\_CELLULAR\_RESPONSE\_TO\_STEROL |  | 15 | 0.59 | 1.53 | 0.037 | 0.426 | 1.000 | 855 | tags=27%, list=7%, signal=29% |
| 380 | GOBP\_EXECUTION\_PHASE\_OF\_APOPTOSIS |  | 56 | 0.43 | 1.53 | 0.012 | 0.427 | 1.000 | 2246 | tags=32%, list=19%, signal=39% |
| 381 | GOBP\_INTRACELLULAR\_LIPID\_TRANSPORT |  | 33 | 0.49 | 1.53 | 0.028 | 0.426 | 1.000 | 1926 | tags=39%, list=16%, signal=47% |
| 382 | GOCC\_VESICLE\_COAT |  | 43 | 0.46 | 1.53 | 0.022 | 0.425 | 1.000 | 3737 | tags=56%, list=31%, signal=81% |
| 383 | GOMF\_GROWTH\_FACTOR\_RECEPTOR\_BINDING |  | 86 | 0.40 | 1.53 | 0.009 | 0.426 | 1.000 | 2759 | tags=33%, list=23%, signal=42% |
| 384 | GOCC\_U6\_SNRNP |  | 6 | 0.75 | 1.53 | 0.041 | 0.425 | 1.000 | 3035 | tags=100%, list=25%, signal=134% |
| 385 | GOBP\_PROLACTIN\_SECRETION |  | 6 | 0.75 | 1.53 | 0.038 | 0.424 | 1.000 | 1891 | tags=67%, list=16%, signal=79% |
| 386 | GOCC\_GOLGI\_CIS\_CISTERNA |  | 8 | 0.69 | 1.53 | 0.043 | 0.428 | 1.000 | 1028 | tags=25%, list=9%, signal=27% |
| 387 | GOBP\_POSITIVE\_REGULATION\_OF\_PROTEIN\_POLYUBIQUITINATION |  | 5 | 0.79 | 1.53 | 0.038 | 0.429 | 1.000 | 1659 | tags=60%, list=14%, signal=70% |
| 388 | GOMF\_CHEMOKINE\_ACTIVITY |  | 15 | 0.59 | 1.53 | 0.044 | 0.428 | 1.000 | 2056 | tags=60%, list=17%, signal=72% |
| 389 | GOBP\_ACID\_SECRETION |  | 22 | 0.53 | 1.53 | 0.037 | 0.428 | 1.000 | 792 | tags=36%, list=7%, signal=39% |
| 390 | GOMF\_SERINE\_TYPE\_ENDOPEPTIDASE\_INHIBITOR\_ACTIVITY |  | 32 | 0.49 | 1.53 | 0.029 | 0.427 | 1.000 | 3411 | tags=59%, list=28%, signal=83% |
| 391 | GOBP\_ACTIVATION\_OF\_PROTEIN\_KINASE\_A\_ACTIVITY |  | 12 | 0.63 | 1.53 | 0.051 | 0.426 | 1.000 | 1584 | tags=42%, list=13%, signal=48% |
| 392 | GOBP\_DTMP\_METABOLIC\_PROCESS |  | 3 | 0.91 | 1.53 | 0.015 | 0.425 | 1.000 | 904 | tags=67%, list=8%, signal=72% |
| 393 | GOCC\_COLLAGEN\_CONTAINING\_EXTRACELLULAR\_MATRIX |  | 253 | 0.35 | 1.53 | 0.001 | 0.426 | 1.000 | 2954 | tags=40%, list=25%, signal=51% |
| 394 | GOCC\_ACTOMYOSIN |  | 57 | 0.43 | 1.53 | 0.018 | 0.425 | 1.000 | 2340 | tags=37%, list=19%, signal=46% |
| 395 | GOMF\_5\_3\_DNA\_HELICASE\_ACTIVITY |  | 5 | 0.79 | 1.53 | 0.042 | 0.425 | 1.000 | 2321 | tags=60%, list=19%, signal=74% |
| 396 | GOBP\_NOSE\_DEVELOPMENT |  | 10 | 0.65 | 1.53 | 0.043 | 0.424 | 1.000 | 1528 | tags=50%, list=13%, signal=57% |
| 397 | GOMF\_PHOSPHATIDYLINOSITOL\_PHOSPHATE\_4\_PHOSPHATASE\_ACTIVITY |  | 5 | 0.78 | 1.53 | 0.038 | 0.427 | 1.000 | 1520 | tags=60%, list=13%, signal=69% |
| 398 | GOBP\_PHOSPHATIDYLSERINE\_EXPOSURE\_ON\_APOPTOTIC\_CELL\_SURFACE |  | 5 | 0.78 | 1.53 | 0.039 | 0.426 | 1.000 | 769 | tags=40%, list=6%, signal=43% |
| 399 | GOBP\_MEIOTIC\_CHROMOSOME\_SEGREGATION |  | 52 | 0.43 | 1.53 | 0.018 | 0.426 | 1.000 | 3684 | tags=52%, list=31%, signal=75% |
| 400 | GOCC\_LSM2\_8\_COMPLEX |  | 6 | 0.75 | 1.53 | 0.039 | 0.425 | 1.000 | 3035 | tags=100%, list=25%, signal=134% |
| 401 | GOBP\_PYRIMIDINE\_DEOXYRIBONUCLEOTIDE\_BIOSYNTHETIC\_PROCESS |  | 6 | 0.74 | 1.53 | 0.051 | 0.424 | 1.000 | 904 | tags=33%, list=8%, signal=36% |
| 402 | GOBP\_CELL\_SUBSTRATE\_JUNCTION\_ORGANIZATION |  | 88 | 0.40 | 1.53 | 0.012 | 0.424 | 1.000 | 2375 | tags=35%, list=20%, signal=44% |
| 403 | GOBP\_SPINDLE\_MIDZONE\_ASSEMBLY |  | 9 | 0.67 | 1.52 | 0.050 | 0.431 | 1.000 | 2607 | tags=78%, list=22%, signal=99% |
| 404 | GOBP\_INTERLEUKIN\_7\_MEDIATED\_SIGNALING\_PATHWAY |  | 13 | 0.61 | 1.52 | 0.046 | 0.432 | 1.000 | 755 | tags=31%, list=6%, signal=33% |
| 405 | GOBP\_AMINO\_SUGAR\_BIOSYNTHETIC\_PROCESS |  | 11 | 0.63 | 1.52 | 0.054 | 0.433 | 1.000 | 1618 | tags=45%, list=13%, signal=52% |
| 406 | GOBP\_CHROMOSOME\_CONDENSATION |  | 23 | 0.52 | 1.52 | 0.038 | 0.434 | 1.000 | 3251 | tags=61%, list=27%, signal=83% |
| 407 | GOMF\_TRANSFERASE\_ACTIVITY\_TRANSFERRING\_PENTOSYL\_GROUPS |  | 35 | 0.47 | 1.52 | 0.026 | 0.435 | 1.000 | 891 | tags=20%, list=7%, signal=22% |
| 408 | GOBP\_ACETYL\_COA\_METABOLIC\_PROCESS |  | 33 | 0.48 | 1.52 | 0.026 | 0.435 | 1.000 | 1777 | tags=27%, list=15%, signal=32% |
| 409 | GOBP\_POSTTRANSLATIONAL\_PROTEIN\_TARGETING\_TO\_ENDOPLASMIC\_RETICULUM\_MEMBRANE |  | 10 | 0.65 | 1.52 | 0.050 | 0.437 | 1.000 | 3573 | tags=80%, list=30%, signal=114% |
| 410 | GOCC\_MICROVILLUS\_MEMBRANE |  | 15 | 0.58 | 1.52 | 0.043 | 0.438 | 1.000 | 2185 | tags=47%, list=18%, signal=57% |
| 411 | GOBP\_NEGATIVE\_REGULATION\_OF\_RESPONSE\_TO\_BIOTIC\_STIMULUS |  | 51 | 0.44 | 1.52 | 0.019 | 0.437 | 1.000 | 1219 | tags=20%, list=10%, signal=22% |
| 412 | GOBP\_NEGATIVE\_REGULATION\_OF\_SPROUTING\_ANGIOGENESIS |  | 9 | 0.66 | 1.52 | 0.055 | 0.438 | 1.000 | 3693 | tags=78%, list=31%, signal=112% |
| 413 | GOBP\_CELLULAR\_RESPONSE\_TO\_INCREASED\_OXYGEN\_LEVELS |  | 13 | 0.60 | 1.52 | 0.051 | 0.438 | 1.000 | 240 | tags=23%, list=2%, signal=24% |
| 414 | GOBP\_ESTABLISHMENT\_OF\_CENTROSOME\_LOCALIZATION |  | 8 | 0.69 | 1.52 | 0.049 | 0.438 | 1.000 | 800 | tags=38%, list=7%, signal=40% |
| 415 | GOCC\_COLLAGEN\_TRIMER |  | 51 | 0.44 | 1.52 | 0.021 | 0.440 | 1.000 | 3916 | tags=59%, list=33%, signal=87% |
| 416 | GOMF\_TUMOR\_NECROSIS\_FACTOR\_RECEPTOR\_BINDING |  | 21 | 0.53 | 1.52 | 0.041 | 0.439 | 1.000 | 769 | tags=24%, list=6%, signal=25% |
| 417 | GOBP\_MHC\_PROTEIN\_COMPLEX\_ASSEMBLY |  | 3 | 0.90 | 1.52 | 0.020 | 0.440 | 1.000 | 357 | tags=67%, list=3%, signal=69% |
| 418 | GOBP\_REGULATION\_OF\_CELL\_MORPHOGENESIS\_INVOLVED\_IN\_DIFFERENTIATION |  | 75 | 0.41 | 1.52 | 0.010 | 0.440 | 1.000 | 2041 | tags=25%, list=17%, signal=30% |
| 419 | GOBP\_PROTEIN\_LOCALIZATION\_TO\_CELL\_CELL\_JUNCTION |  | 17 | 0.56 | 1.52 | 0.046 | 0.440 | 1.000 | 2955 | tags=53%, list=25%, signal=70% |
| 420 | GOMF\_CHEMOATTRACTANT\_ACTIVITY |  | 20 | 0.53 | 1.52 | 0.037 | 0.443 | 1.000 | 3323 | tags=55%, list=28%, signal=76% |
| 421 | GOBP\_TOLL\_SIGNALING\_PATHWAY |  | 4 | 0.84 | 1.51 | 0.029 | 0.445 | 1.000 | 1569 | tags=50%, list=13%, signal=57% |
| 422 | GOBP\_NEGATIVE\_REGULATION\_OF\_CELL\_MIGRATION\_INVOLVED\_IN\_SPROUTING\_ANGIOGENESIS |  | 14 | 0.58 | 1.51 | 0.049 | 0.445 | 1.000 | 1411 | tags=36%, list=12%, signal=40% |
| 423 | GOBP\_CYTOSKELETON\_DEPENDENT\_CYTOKINESIS |  | 72 | 0.41 | 1.51 | 0.012 | 0.444 | 1.000 | 3079 | tags=43%, list=26%, signal=58% |
| 424 | GOBP\_NEGATIVE\_REGULATION\_OF\_ASTROCYTE\_DIFFERENTIATION |  | 5 | 0.78 | 1.51 | 0.038 | 0.444 | 1.000 | 958 | tags=60%, list=8%, signal=65% |
| 425 | GOBP\_SMALL\_MOLECULE\_BIOSYNTHETIC\_PROCESS |  | 482 | 0.32 | 1.51 | 0.000 | 0.444 | 1.000 | 1424 | tags=19%, list=12%, signal=21% |
| 426 | GOCC\_ACTIN\_CYTOSKELETON |  | 352 | 0.33 | 1.51 | 0.000 | 0.444 | 1.000 | 2689 | tags=33%, list=22%, signal=41% |
| 427 | GOBP\_REGULATION\_OF\_PLATELET\_AGGREGATION |  | 14 | 0.59 | 1.51 | 0.046 | 0.444 | 1.000 | 1234 | tags=36%, list=10%, signal=40% |
| 428 | GOBP\_GLYCEROL\_3\_PHOSPHATE\_METABOLIC\_PROCESS |  | 7 | 0.72 | 1.51 | 0.046 | 0.445 | 1.000 | 909 | tags=43%, list=8%, signal=46% |
| 429 | GOBP\_SPINDLE\_ORGANIZATION |  | 144 | 0.37 | 1.51 | 0.003 | 0.446 | 1.000 | 3700 | tags=47%, list=31%, signal=67% |
| 430 | GOBP\_REGULATION\_OF\_SODIUM\_ION\_TRANSPORT |  | 56 | 0.43 | 1.51 | 0.020 | 0.447 | 1.000 | 2723 | tags=38%, list=23%, signal=48% |
| 431 | GOCC\_RUFFLE |  | 135 | 0.37 | 1.51 | 0.003 | 0.446 | 1.000 | 2316 | tags=31%, list=19%, signal=38% |
| 432 | GOBP\_CHROMOSOME\_LOCALIZATION |  | 64 | 0.42 | 1.51 | 0.015 | 0.445 | 1.000 | 3934 | tags=61%, list=33%, signal=90% |
| 433 | GOBP\_REGULATION\_OF\_GLIAL\_CELL\_APOPTOTIC\_PROCESS |  | 9 | 0.66 | 1.51 | 0.055 | 0.447 | 1.000 | 1053 | tags=33%, list=9%, signal=37% |
| 434 | GOMF\_DYSTROGLYCAN\_BINDING |  | 9 | 0.66 | 1.51 | 0.050 | 0.448 | 1.000 | 810 | tags=33%, list=7%, signal=36% |
| 435 | GOBP\_GROWTH\_PLATE\_CARTILAGE\_CHONDROCYTE\_DEVELOPMENT |  | 4 | 0.83 | 1.51 | 0.038 | 0.450 | 1.000 | 1738 | tags=50%, list=14%, signal=58% |
| 436 | GOBP\_ALDITOL\_PHOSPHATE\_METABOLIC\_PROCESS |  | 9 | 0.66 | 1.51 | 0.052 | 0.449 | 1.000 | 1089 | tags=44%, list=9%, signal=49% |
| 437 | GOBP\_NEGATIVE\_REGULATION\_OF\_RIBOSOME\_BIOGENESIS |  | 4 | 0.83 | 1.51 | 0.034 | 0.449 | 1.000 | 974 | tags=50%, list=8%, signal=54% |
| 438 | GOCC\_ATP\_BINDING\_CASSETTE\_ABC\_TRANSPORTER\_COMPLEX |  | 6 | 0.73 | 1.51 | 0.057 | 0.452 | 1.000 | 41 | tags=33%, list=0%, signal=33% |
| 439 | GOCC\_DNA\_REPLICATION\_PREINITIATION\_COMPLEX |  | 11 | 0.62 | 1.51 | 0.051 | 0.451 | 1.000 | 2631 | tags=73%, list=22%, signal=93% |
| 440 | GOBP\_EPITHELIAL\_CELL\_DIFFERENTIATION |  | 393 | 0.33 | 1.51 | 0.000 | 0.451 | 1.000 | 2706 | tags=31%, list=23%, signal=39% |
| 441 | GOBP\_POSITIVE\_REGULATION\_OF\_TRANSCRIPTION\_OF\_NOTCH\_RECEPTOR\_TARGET |  | 15 | 0.57 | 1.51 | 0.048 | 0.451 | 1.000 | 2120 | tags=33%, list=18%, signal=40% |
| 442 | GOBP\_PUTRESCINE\_METABOLIC\_PROCESS |  | 7 | 0.70 | 1.51 | 0.054 | 0.452 | 1.000 | 1014 | tags=29%, list=8%, signal=31% |
| 443 | GOCC\_TIGHT\_JUNCTION |  | 87 | 0.39 | 1.51 | 0.018 | 0.452 | 1.000 | 2316 | tags=30%, list=19%, signal=37% |
| 444 | GOBP\_MEIOTIC\_CHROMOSOME\_CONDENSATION |  | 6 | 0.73 | 1.51 | 0.053 | 0.452 | 1.000 | 2807 | tags=83%, list=23%, signal=109% |
| 445 | GOBP\_DIET\_INDUCED\_THERMOGENESIS |  | 6 | 0.73 | 1.51 | 0.052 | 0.451 | 1.000 | 132 | tags=33%, list=1%, signal=34% |
| 446 | GOBP\_INTERLEUKIN\_1\_PRODUCTION |  | 51 | 0.43 | 1.51 | 0.024 | 0.452 | 1.000 | 2138 | tags=39%, list=18%, signal=48% |
| 447 | GOBP\_VESICLE\_FUSION\_WITH\_ENDOPLASMIC\_RETICULUM\_GOLGI\_INTERMEDIATE\_COMPARTMENT\_ERGIC\_MEMBRANE |  | 4 | 0.83 | 1.50 | 0.044 | 0.459 | 1.000 | 357 | tags=50%, list=3%, signal=52% |
| 448 | GOMF\_QUATERNARY\_AMMONIUM\_GROUP\_BINDING |  | 20 | 0.53 | 1.50 | 0.045 | 0.461 | 1.000 | 1319 | tags=35%, list=11%, signal=39% |
| 449 | GOBP\_POSITIVE\_REGULATION\_OF\_RESPONSE\_TO\_INTERFERON\_GAMMA |  | 3 | 0.89 | 1.50 | 0.024 | 0.460 | 1.000 | 162 | tags=67%, list=1%, signal=68% |
| 450 | GOBP\_NEGATIVE\_REGULATION\_OF\_GLYCOGEN\_METABOLIC\_PROCESS |  | 4 | 0.82 | 1.50 | 0.035 | 0.461 | 1.000 | 2115 | tags=100%, list=18%, signal=121% |
| 451 | GOBP\_BILE\_ACID\_AND\_BILE\_SALT\_TRANSPORT |  | 16 | 0.57 | 1.50 | 0.051 | 0.460 | 1.000 | 1526 | tags=44%, list=13%, signal=50% |
| 452 | GOBP\_NEGATIVE\_REGULATION\_OF\_NUCLEAR\_DIVISION |  | 45 | 0.44 | 1.50 | 0.026 | 0.462 | 1.000 | 3959 | tags=62%, list=33%, signal=92% |
| 453 | GOMF\_PROTEIN\_LYSINE\_6\_OXIDASE\_ACTIVITY |  | 5 | 0.77 | 1.50 | 0.048 | 0.462 | 1.000 | 2504 | tags=80%, list=21%, signal=101% |
| 454 | GOCC\_ENDOLYSOSOME\_MEMBRANE |  | 11 | 0.62 | 1.50 | 0.054 | 0.461 | 1.000 | 1107 | tags=27%, list=9%, signal=30% |
| 455 | GOBP\_REGULATION\_OF\_SPROUTING\_ANGIOGENESIS |  | 23 | 0.51 | 1.50 | 0.042 | 0.462 | 1.000 | 3693 | tags=57%, list=31%, signal=81% |
| 456 | GOBP\_VESICLE\_CARGO\_LOADING |  | 19 | 0.54 | 1.50 | 0.052 | 0.462 | 1.000 | 1656 | tags=32%, list=14%, signal=37% |
| 457 | GOCC\_COPII\_COATED\_ER\_TO\_GOLGI\_TRANSPORT\_VESICLE |  | 58 | 0.42 | 1.50 | 0.025 | 0.463 | 1.000 | 2538 | tags=36%, list=21%, signal=46% |
| 458 | GOBP\_REGULATION\_OF\_ANION\_TRANSPORT |  | 577 | 0.32 | 1.50 | 0.000 | 0.462 | 1.000 | 2049 | tags=24%, list=17%, signal=27% |
| 459 | GOBP\_REGULATION\_OF\_PEPTIDE\_TRANSPORT |  | 403 | 0.32 | 1.50 | 0.000 | 0.462 | 1.000 | 2049 | tags=23%, list=17%, signal=27% |
| 460 | GOBP\_LIPID\_BIOSYNTHETIC\_PROCESS |  | 523 | 0.32 | 1.50 | 0.000 | 0.462 | 1.000 | 1998 | tags=25%, list=17%, signal=29% |
| 461 | GOBP\_QUATERNARY\_AMMONIUM\_GROUP\_TRANSPORT |  | 8 | 0.68 | 1.50 | 0.054 | 0.462 | 1.000 | 616 | tags=50%, list=5%, signal=53% |
| 462 | GOBP\_REGULATION\_OF\_PROTEIN\_LOCALIZATION |  | 618 | 0.31 | 1.50 | 0.000 | 0.461 | 1.000 | 2190 | tags=23%, list=18%, signal=27% |
| 463 | GOCC\_CELL\_CORTEX |  | 219 | 0.34 | 1.50 | 0.001 | 0.462 | 1.000 | 2397 | tags=32%, list=20%, signal=39% |
| 464 | GOBP\_NEGATIVE\_REGULATION\_OF\_SEQUESTERING\_OF\_TRIGLYCERIDE |  | 4 | 0.82 | 1.50 | 0.037 | 0.464 | 1.000 | 1636 | tags=75%, list=14%, signal=87% |
| 465 | GOBP\_REGULATION\_OF\_CHROMOSOME\_SEPARATION |  | 61 | 0.42 | 1.50 | 0.017 | 0.464 | 1.000 | 3959 | tags=56%, list=33%, signal=83% |
| 466 | GOCC\_DEATH\_INDUCING\_SIGNALING\_COMPLEX |  | 8 | 0.67 | 1.50 | 0.059 | 0.465 | 1.000 | 3470 | tags=88%, list=29%, signal=123% |
| 467 | GOMF\_NETRIN\_RECEPTOR\_ACTIVITY |  | 4 | 0.82 | 1.50 | 0.039 | 0.465 | 1.000 | 307 | tags=25%, list=3%, signal=26% |
| 468 | GOBP\_NEGATIVE\_REGULATION\_OF\_DOUBLE\_STRAND\_BREAK\_REPAIR\_VIA\_HOMOLOGOUS\_RECOMBINATION |  | 12 | 0.60 | 1.50 | 0.056 | 0.467 | 1.000 | 728 | tags=25%, list=6%, signal=27% |
| 469 | GOMF\_CYCLIN\_DEPENDENT\_PROTEIN\_SERINE\_THREONINE\_KINASE\_INHIBITOR\_ACTIVITY |  | 7 | 0.70 | 1.49 | 0.056 | 0.467 | 1.000 | 1333 | tags=57%, list=11%, signal=64% |
| 470 | GOMF\_ORGANIC\_CATION\_TRANSMEMBRANE\_TRANSPORTER\_ACTIVITY |  | 12 | 0.60 | 1.49 | 0.060 | 0.467 | 1.000 | 616 | tags=33%, list=5%, signal=35% |
| 471 | GOBP\_LUNG\_GROWTH |  | 5 | 0.77 | 1.49 | 0.057 | 0.466 | 1.000 | 2524 | tags=60%, list=21%, signal=76% |
| 472 | GOMF\_ORGANIC\_ACID\_SODIUM\_SYMPORTER\_ACTIVITY |  | 17 | 0.55 | 1.49 | 0.048 | 0.466 | 1.000 | 836 | tags=35%, list=7%, signal=38% |
| 473 | GOBP\_ATRIOVENTRICULAR\_CANAL\_DEVELOPMENT |  | 7 | 0.70 | 1.49 | 0.061 | 0.466 | 1.000 | 2440 | tags=71%, list=20%, signal=90% |
| 474 | GOBP\_TERPENOID\_METABOLIC\_PROCESS |  | 69 | 0.41 | 1.49 | 0.019 | 0.467 | 1.000 | 954 | tags=26%, list=8%, signal=28% |
| 475 | GOCC\_CHROMOSOME\_TELOMERIC\_REGION |  | 110 | 0.38 | 1.49 | 0.008 | 0.467 | 1.000 | 3149 | tags=35%, list=26%, signal=48% |
| 476 | GOBP\_POSITIVE\_REGULATION\_OF\_ANION\_TRANSPORT |  | 335 | 0.33 | 1.49 | 0.001 | 0.469 | 1.000 | 2049 | tags=25%, list=17%, signal=29% |
| 477 | GOBP\_REGULATION\_OF\_VESICLE\_SIZE |  | 10 | 0.63 | 1.49 | 0.056 | 0.470 | 1.000 | 3510 | tags=60%, list=29%, signal=85% |
| 478 | GOBP\_ACTIVATION\_OF\_PHOSPHOLIPASE\_D\_ACTIVITY |  | 5 | 0.77 | 1.49 | 0.052 | 0.470 | 1.000 | 732 | tags=40%, list=6%, signal=43% |
| 479 | GOBP\_DNA\_INTEGRITY\_CHECKPOINT |  | 122 | 0.37 | 1.49 | 0.009 | 0.470 | 1.000 | 3796 | tags=44%, list=32%, signal=64% |
| 480 | GOBP\_POSITIVE\_REGULATION\_OF\_BONE\_MINERALIZATION |  | 26 | 0.50 | 1.49 | 0.038 | 0.471 | 1.000 | 1918 | tags=35%, list=16%, signal=41% |
| 481 | GOBP\_TRIGEMINAL\_NERVE\_DEVELOPMENT |  | 3 | 0.89 | 1.49 | 0.027 | 0.470 | 1.000 | 613 | tags=33%, list=5%, signal=35% |
| 482 | GOBP\_MYOBLAST\_FATE\_COMMITMENT |  | 4 | 0.81 | 1.49 | 0.045 | 0.470 | 1.000 | 1549 | tags=75%, list=13%, signal=86% |
| 483 | GOBP\_PLASMA\_MEMBRANE\_ORGANIZATION |  | 78 | 0.40 | 1.49 | 0.018 | 0.469 | 1.000 | 2925 | tags=38%, list=24%, signal=51% |
| 484 | GOCC\_GERM\_CELL\_NUCLEUS |  | 10 | 0.63 | 1.49 | 0.064 | 0.471 | 1.000 | 1305 | tags=40%, list=11%, signal=45% |
| 485 | GOBP\_RESPONSE\_TO\_STAUROSPORINE |  | 3 | 0.89 | 1.49 | 0.031 | 0.471 | 1.000 | 1333 | tags=100%, list=11%, signal=112% |
| 486 | GOBP\_POSITIVE\_REGULATION\_OF\_LIPID\_METABOLIC\_PROCESS |  | 104 | 0.38 | 1.49 | 0.010 | 0.473 | 1.000 | 1517 | tags=28%, list=13%, signal=32% |
| 487 | GOBP\_SMAD\_PROTEIN\_SIGNAL\_TRANSDUCTION |  | 52 | 0.43 | 1.49 | 0.030 | 0.473 | 1.000 | 1379 | tags=33%, list=11%, signal=37% |
| 488 | GOBP\_POSITIVE\_REGULATION\_OF\_LIPID\_BIOSYNTHETIC\_PROCESS |  | 58 | 0.42 | 1.49 | 0.023 | 0.472 | 1.000 | 1419 | tags=34%, list=12%, signal=39% |
| 489 | GOBP\_MITOTIC\_RECOMBINATION |  | 18 | 0.54 | 1.49 | 0.047 | 0.472 | 1.000 | 1305 | tags=33%, list=11%, signal=37% |
| 490 | GOBP\_REGULATION\_OF\_BIOLOGICAL\_PROCESS\_INVOLVED\_IN\_SYMBIOTIC\_INTERACTION |  | 116 | 0.37 | 1.49 | 0.010 | 0.472 | 1.000 | 1449 | tags=18%, list=12%, signal=20% |
| 491 | GOBP\_CELLULAR\_RESPONSE\_TO\_DSRNA |  | 12 | 0.60 | 1.49 | 0.060 | 0.471 | 1.000 | 1083 | tags=33%, list=9%, signal=37% |
| 492 | GOBP\_ANATOMICAL\_STRUCTURE\_ARRANGEMENT |  | 9 | 0.64 | 1.49 | 0.062 | 0.470 | 1.000 | 2167 | tags=56%, list=18%, signal=68% |
| 493 | GOBP\_CELLULAR\_RESPONSE\_TO\_NICOTINE |  | 3 | 0.89 | 1.49 | 0.028 | 0.470 | 1.000 | 509 | tags=33%, list=4%, signal=35% |
| 494 | GOBP\_ALKANESULFONATE\_METABOLIC\_PROCESS |  | 5 | 0.77 | 1.49 | 0.052 | 0.470 | 1.000 | 661 | tags=40%, list=6%, signal=42% |
| 495 | GOCC\_SMAD\_PROTEIN\_COMPLEX |  | 7 | 0.69 | 1.49 | 0.054 | 0.472 | 1.000 | 1021 | tags=43%, list=9%, signal=47% |
| 496 | GOMF\_INSULIN\_RECEPTOR\_SUBSTRATE\_BINDING |  | 10 | 0.63 | 1.49 | 0.059 | 0.473 | 1.000 | 2311 | tags=60%, list=19%, signal=74% |
| 497 | GOBP\_REGULATION\_OF\_TYPE\_I\_INTERFERON\_MEDIATED\_SIGNALING\_PATHWAY |  | 27 | 0.49 | 1.49 | 0.043 | 0.472 | 1.000 | 1101 | tags=22%, list=9%, signal=24% |
| 498 | GOBP\_NEGATIVE\_REGULATION\_OF\_MEMBRANE\_PROTEIN\_ECTODOMAIN\_PROTEOLYSIS |  | 6 | 0.72 | 1.49 | 0.064 | 0.471 | 1.000 | 535 | tags=33%, list=4%, signal=35% |
| 499 | GOBP\_NEGATIVE\_REGULATION\_OF\_GLIAL\_CELL\_DIFFERENTIATION |  | 11 | 0.61 | 1.49 | 0.059 | 0.471 | 1.000 | 958 | tags=27%, list=8%, signal=30% |
| 500 | GOBP\_EXTRINSIC\_APOPTOTIC\_SIGNALING\_PATHWAY |  | 149 | 0.36 | 1.49 | 0.007 | 0.471 | 1.000 | 2138 | tags=26%, list=18%, signal=31% |
| 501 | GOBP\_MITOTIC\_METAPHASE\_PLATE\_CONGRESSION |  | 41 | 0.45 | 1.49 | 0.029 | 0.470 | 1.000 | 3934 | tags=61%, list=33%, signal=90% |
| 502 | GOBP\_REGULATION\_OF\_EPITHELIAL\_CELL\_PROLIFERATION\_INVOLVED\_IN\_LUNG\_MORPHOGENESIS |  | 6 | 0.73 | 1.48 | 0.060 | 0.473 | 1.000 | 1800 | tags=50%, list=15%, signal=59% |
| 503 | GOCC\_INTRINSIC\_COMPONENT\_OF\_PRESYNAPTIC\_ACTIVE\_ZONE\_MEMBRANE |  | 12 | 0.60 | 1.48 | 0.058 | 0.476 | 1.000 | 382 | tags=25%, list=3%, signal=26% |
| 504 | GOBP\_S\_ADENOSYLMETHIONINE\_CYCLE |  | 7 | 0.70 | 1.48 | 0.063 | 0.477 | 1.000 | 289 | tags=14%, list=2%, signal=15% |
| 505 | GOBP\_REGULATION\_OF\_BONE\_MINERALIZATION |  | 46 | 0.43 | 1.48 | 0.032 | 0.477 | 1.000 | 2604 | tags=37%, list=22%, signal=47% |
| 506 | GOBP\_RETROGRADE\_TRANS\_SYNAPTIC\_SIGNALING\_BY\_LIPID |  | 5 | 0.76 | 1.48 | 0.053 | 0.477 | 1.000 | 1368 | tags=60%, list=11%, signal=68% |
| 507 | GOBP\_PERISTALSIS |  | 5 | 0.77 | 1.48 | 0.047 | 0.477 | 1.000 | 1124 | tags=60%, list=9%, signal=66% |
| 508 | GOBP\_LUTEINIZING\_HORMONE\_SECRETION |  | 4 | 0.82 | 1.48 | 0.043 | 0.477 | 1.000 | 263 | tags=50%, list=2%, signal=51% |
| 509 | GOBP\_PYRIMIDINE\_DEOXYRIBONUCLEOSIDE\_TRIPHOSPHATE\_METABOLIC\_PROCESS |  | 5 | 0.76 | 1.48 | 0.064 | 0.478 | 1.000 | 61 | tags=20%, list=1%, signal=20% |
| 510 | GOBP\_T\_CELL\_EXTRAVASATION |  | 7 | 0.69 | 1.48 | 0.062 | 0.477 | 1.000 | 1157 | tags=43%, list=10%, signal=47% |
| 511 | GOBP\_PROTON\_TRANSPORTING\_TWO\_SECTOR\_ATPASE\_COMPLEX\_ASSEMBLY |  | 10 | 0.63 | 1.48 | 0.062 | 0.477 | 1.000 | 284 | tags=20%, list=2%, signal=20% |
| 512 | GOCC\_PHOTORECEPTOR\_CONNECTING\_CILIUM |  | 23 | 0.50 | 1.48 | 0.060 | 0.476 | 1.000 | 2249 | tags=39%, list=19%, signal=48% |
| 513 | GOBP\_MICROTUBULE\_NUCLEATION\_BY\_MICROTUBULE\_ORGANIZING\_CENTER |  | 5 | 0.76 | 1.48 | 0.065 | 0.477 | 1.000 | 2242 | tags=80%, list=19%, signal=98% |
| 514 | GOBP\_FILOPODIUM\_ASSEMBLY |  | 49 | 0.43 | 1.48 | 0.030 | 0.476 | 1.000 | 2200 | tags=43%, list=18%, signal=52% |
| 515 | GOBP\_ACYLGLYCEROL\_ACYL\_CHAIN\_REMODELING |  | 4 | 0.82 | 1.48 | 0.042 | 0.476 | 1.000 | 817 | tags=75%, list=7%, signal=80% |
| 516 | GOBP\_KINETOCHORE\_ORGANIZATION |  | 21 | 0.52 | 1.48 | 0.051 | 0.476 | 1.000 | 3832 | tags=67%, list=32%, signal=98% |
| 517 | GOMF\_FIBROBLAST\_GROWTH\_FACTOR\_RECEPTOR\_BINDING |  | 11 | 0.61 | 1.48 | 0.065 | 0.475 | 1.000 | 2585 | tags=64%, list=22%, signal=81% |
| 518 | GOBP\_RESPONSE\_TO\_VITAMIN\_A |  | 9 | 0.64 | 1.48 | 0.072 | 0.474 | 1.000 | 228 | tags=22%, list=2%, signal=23% |
| 519 | GOBP\_RESPONSE\_TO\_NUTRIENT |  | 121 | 0.37 | 1.48 | 0.012 | 0.476 | 1.000 | 1427 | tags=22%, list=12%, signal=25% |
| 520 | GOMF\_PHOSPHORIC\_DIESTER\_HYDROLASE\_ACTIVITY |  | 66 | 0.40 | 1.48 | 0.023 | 0.475 | 1.000 | 1700 | tags=30%, list=14%, signal=35% |
| 521 | GOBP\_REGULATION\_OF\_AMYLOID\_BETA\_CLEARANCE |  | 10 | 0.62 | 1.48 | 0.071 | 0.474 | 1.000 | 299 | tags=20%, list=2%, signal=20% |
| 522 | GOBP\_PRE\_REPLICATIVE\_COMPLEX\_ASSEMBLY\_INVOLVED\_IN\_CELL\_CYCLE\_DNA\_REPLICATION |  | 6 | 0.72 | 1.48 | 0.059 | 0.478 | 1.000 | 2631 | tags=83%, list=22%, signal=107% |
| 523 | GOMF\_CORTICOTROPIN\_RELEASING\_HORMONE\_RECEPTOR\_BINDING |  | 3 | 0.89 | 1.48 | 0.024 | 0.478 | 1.000 | 210 | tags=33%, list=2%, signal=34% |
| 524 | GOBP\_POSITIVE\_REGULATION\_OF\_PROTEIN\_LOCALIZATION\_TO\_MEMBRANE |  | 87 | 0.39 | 1.48 | 0.016 | 0.477 | 1.000 | 3126 | tags=41%, list=26%, signal=56% |
| 525 | GOBP\_DEOXYRIBONUCLEOSIDE\_DIPHOSPHATE\_METABOLIC\_PROCESS |  | 3 | 0.89 | 1.48 | 0.024 | 0.479 | 1.000 | 61 | tags=33%, list=1%, signal=33% |
| 526 | GOBP\_POSITIVE\_REGULATION\_OF\_ION\_TRANSPORT |  | 449 | 0.32 | 1.48 | 0.000 | 0.478 | 1.000 | 2056 | tags=24%, list=17%, signal=28% |
| 527 | GOBP\_REFLEX |  | 7 | 0.69 | 1.48 | 0.058 | 0.478 | 1.000 | 2510 | tags=71%, list=21%, signal=90% |
| 528 | GOBP\_INTERLEUKIN\_1\_BETA\_PRODUCTION |  | 42 | 0.44 | 1.48 | 0.037 | 0.480 | 1.000 | 2138 | tags=40%, list=18%, signal=49% |
| 529 | GOCC\_INTERCALATED\_DISC |  | 33 | 0.46 | 1.48 | 0.041 | 0.479 | 1.000 | 987 | tags=30%, list=8%, signal=33% |
| 530 | GOBP\_PEPTIDYL\_LYSINE\_OXIDATION |  | 5 | 0.77 | 1.48 | 0.052 | 0.478 | 1.000 | 2504 | tags=80%, list=21%, signal=101% |
| 531 | GOBP\_PROTEIN\_POLYMERIZATION |  | 200 | 0.34 | 1.48 | 0.004 | 0.478 | 1.000 | 2146 | tags=27%, list=18%, signal=32% |
| 532 | GOBP\_TRANS\_SYNAPTIC\_SIGNALING\_BY\_LIPID |  | 6 | 0.72 | 1.48 | 0.064 | 0.477 | 1.000 | 1368 | tags=50%, list=11%, signal=56% |
| 533 | GOBP\_RETROGRADE\_TRANS\_SYNAPTIC\_SIGNALING |  | 7 | 0.69 | 1.48 | 0.071 | 0.476 | 1.000 | 1368 | tags=43%, list=11%, signal=48% |
| 534 | GOBP\_REGULATION\_OF\_ERYTHROCYTE\_DIFFERENTIATION |  | 37 | 0.45 | 1.48 | 0.044 | 0.475 | 1.000 | 855 | tags=24%, list=7%, signal=26% |
| 535 | GOBP\_DOUBLE\_STRAND\_BREAK\_REPAIR |  | 184 | 0.35 | 1.48 | 0.005 | 0.475 | 1.000 | 3366 | tags=38%, list=28%, signal=52% |
| 536 | GOBP\_NEGATIVE\_REGULATION\_OF\_PEPTIDYL\_THREONINE\_PHOSPHORYLATION |  | 14 | 0.58 | 1.48 | 0.064 | 0.475 | 1.000 | 1977 | tags=29%, list=16%, signal=34% |
| 537 | GOBP\_THYMOCYTE\_MIGRATION |  | 5 | 0.77 | 1.47 | 0.051 | 0.476 | 1.000 | 512 | tags=40%, list=4%, signal=42% |
| 538 | GOCC\_ENDOPLASMIC\_RETICULUM\_EXIT\_SITE |  | 24 | 0.50 | 1.47 | 0.048 | 0.475 | 1.000 | 2538 | tags=42%, list=21%, signal=53% |
| 539 | GOMF\_ACTIN\_BINDING |  | 297 | 0.33 | 1.47 | 0.001 | 0.476 | 1.000 | 2340 | tags=31%, list=19%, signal=38% |
| 540 | GOBP\_FLOOR\_PLATE\_DEVELOPMENT |  | 4 | 0.81 | 1.47 | 0.048 | 0.477 | 1.000 | 2288 | tags=75%, list=19%, signal=93% |
| 541 | GOBP\_MUSCLE\_CELL\_FATE\_COMMITMENT |  | 6 | 0.73 | 1.47 | 0.056 | 0.477 | 1.000 | 2274 | tags=67%, list=19%, signal=82% |
| 542 | GOBP\_REGULATION\_OF\_HORMONE\_LEVELS |  | 318 | 0.33 | 1.47 | 0.002 | 0.477 | 1.000 | 1793 | tags=27%, list=15%, signal=31% |
| 543 | GOBP\_CELL\_CELL\_JUNCTION\_ORGANIZATION |  | 142 | 0.36 | 1.47 | 0.009 | 0.477 | 1.000 | 2738 | tags=32%, list=23%, signal=41% |
| 544 | GOBP\_POSITIVE\_REGULATION\_OF\_CHROMATIN\_BINDING |  | 10 | 0.62 | 1.47 | 0.077 | 0.476 | 1.000 | 2359 | tags=50%, list=20%, signal=62% |
| 545 | GOBP\_RESPONSE\_TO\_HYPEROXIA |  | 16 | 0.55 | 1.47 | 0.060 | 0.477 | 1.000 | 2221 | tags=38%, list=18%, signal=46% |
| 546 | GOCC\_LOW\_DENSITY\_LIPOPROTEIN\_PARTICLE |  | 6 | 0.72 | 1.47 | 0.063 | 0.476 | 1.000 | 589 | tags=50%, list=5%, signal=53% |
| 547 | GOBP\_DITERPENOID\_BIOSYNTHETIC\_PROCESS |  | 6 | 0.72 | 1.47 | 0.064 | 0.476 | 1.000 | 581 | tags=50%, list=5%, signal=53% |
| 548 | GOBP\_POSITIVE\_REGULATION\_OF\_CHONDROCYTE\_DIFFERENTIATION |  | 11 | 0.61 | 1.47 | 0.065 | 0.475 | 1.000 | 1595 | tags=36%, list=13%, signal=42% |
| 549 | GOBP\_CHROMOSOME\_SEPARATION |  | 80 | 0.39 | 1.47 | 0.020 | 0.475 | 1.000 | 3959 | tags=51%, list=33%, signal=76% |
| 550 | GOBP\_REGULATION\_OF\_LIPOPROTEIN\_LIPASE\_ACTIVITY |  | 12 | 0.60 | 1.47 | 0.068 | 0.476 | 1.000 | 1324 | tags=42%, list=11%, signal=47% |
| 551 | GOMF\_STEROL\_ESTERASE\_ACTIVITY |  | 3 | 0.89 | 1.47 | 0.035 | 0.475 | 1.000 | 458 | tags=67%, list=4%, signal=69% |
| 552 | GOBP\_CHROMOSOME\_SEGREGATION |  | 248 | 0.33 | 1.47 | 0.002 | 0.474 | 1.000 | 3978 | tags=47%, list=33%, signal=68% |
| 553 | GOBP\_INTERLEUKIN\_1\_ALPHA\_PRODUCTION |  | 6 | 0.73 | 1.47 | 0.074 | 0.476 | 1.000 | 613 | tags=50%, list=5%, signal=53% |
| 554 | GOMF\_METALLOCARBOXYPEPTIDASE\_ACTIVITY |  | 16 | 0.55 | 1.47 | 0.063 | 0.476 | 1.000 | 3029 | tags=69%, list=25%, signal=92% |
| 555 | GOBP\_PROTEIN\_LOCALIZATION\_TO\_CELL\_CORTEX |  | 10 | 0.63 | 1.47 | 0.067 | 0.475 | 1.000 | 3115 | tags=70%, list=26%, signal=94% |
| 556 | GOBP\_RESPONSE\_TO\_CARBOHYDRATE |  | 150 | 0.35 | 1.47 | 0.009 | 0.475 | 1.000 | 2115 | tags=29%, list=18%, signal=35% |
| 557 | GOBP\_REGULATION\_OF\_T\_CELL\_EXTRAVASATION |  | 4 | 0.81 | 1.47 | 0.054 | 0.475 | 1.000 | 512 | tags=50%, list=4%, signal=52% |
| 558 | GOBP\_NEGATIVE\_REGULATION\_OF\_CHROMOSOME\_ORGANIZATION |  | 71 | 0.40 | 1.47 | 0.022 | 0.478 | 1.000 | 3959 | tags=55%, list=33%, signal=81% |
| 559 | GOBP\_REGULATION\_OF\_PROTEIN\_LOCALIZATION\_TO\_MEMBRANE |  | 134 | 0.36 | 1.47 | 0.011 | 0.478 | 1.000 | 2190 | tags=28%, list=18%, signal=33% |
| 560 | GOBP\_REGULATION\_OF\_CHROMOSOME\_SEGREGATION |  | 74 | 0.40 | 1.47 | 0.020 | 0.478 | 1.000 | 3978 | tags=53%, list=33%, signal=78% |
| 561 | GOBP\_MUSCLE\_ORGAN\_MORPHOGENESIS |  | 39 | 0.44 | 1.47 | 0.039 | 0.478 | 1.000 | 1832 | tags=31%, list=15%, signal=36% |
| 562 | GOBP\_NUCLEOSIDE\_TRIPHOSPHATE\_CATABOLIC\_PROCESS |  | 10 | 0.62 | 1.47 | 0.068 | 0.480 | 1.000 | 1288 | tags=50%, list=11%, signal=56% |
| 563 | GOBP\_NEGATIVE\_REGULATION\_OF\_TUMOR\_NECROSIS\_FACTOR\_MEDIATED\_SIGNALING\_PATHWAY |  | 11 | 0.60 | 1.47 | 0.072 | 0.479 | 1.000 | 2701 | tags=55%, list=22%, signal=70% |
| 564 | GOBP\_POSITIVE\_REGULATION\_OF\_TOLERANCE\_INDUCTION |  | 4 | 0.81 | 1.47 | 0.049 | 0.479 | 1.000 | 682 | tags=25%, list=6%, signal=26% |
| 565 | GOCC\_CHROMOSOMAL\_REGION |  | 262 | 0.33 | 1.47 | 0.002 | 0.478 | 1.000 | 3978 | tags=46%, list=33%, signal=67% |
| 566 | GOBP\_GLUCOCORTICOID\_METABOLIC\_PROCESS |  | 12 | 0.60 | 1.47 | 0.069 | 0.478 | 1.000 | 3109 | tags=67%, list=26%, signal=90% |
| 567 | GOMF\_POLY\_G\_BINDING |  | 8 | 0.66 | 1.47 | 0.073 | 0.479 | 1.000 | 2880 | tags=50%, list=24%, signal=66% |
| 568 | GOBP\_SUBSTRATE\_ADHESION\_DEPENDENT\_CELL\_SPREADING |  | 87 | 0.38 | 1.47 | 0.021 | 0.479 | 1.000 | 2440 | tags=32%, list=20%, signal=40% |
| 569 | GOMF\_LIPASE\_INHIBITOR\_ACTIVITY |  | 8 | 0.66 | 1.47 | 0.065 | 0.479 | 1.000 | 1858 | tags=63%, list=15%, signal=74% |
| 570 | GOMF\_UDP\_GALACTOSYLTRANSFERASE\_ACTIVITY |  | 13 | 0.58 | 1.47 | 0.062 | 0.480 | 1.000 | 110 | tags=15%, list=1%, signal=16% |
| 571 | GOBP\_MEIOTIC\_CELL\_CYCLE\_PROCESS |  | 108 | 0.37 | 1.47 | 0.016 | 0.480 | 1.000 | 3935 | tags=48%, list=33%, signal=71% |
| 572 | GOBP\_MEMBRANE\_RAFT\_ORGANIZATION |  | 17 | 0.54 | 1.47 | 0.070 | 0.480 | 1.000 | 1967 | tags=35%, list=16%, signal=42% |
| 573 | GOBP\_CELL\_CYCLE\_CHECKPOINT |  | 166 | 0.35 | 1.46 | 0.006 | 0.481 | 1.000 | 4107 | tags=48%, list=34%, signal=71% |
| 574 | GOBP\_POSITIVE\_REGULATION\_OF\_GLYCOPROTEIN\_METABOLIC\_PROCESS |  | 21 | 0.51 | 1.46 | 0.052 | 0.481 | 1.000 | 1544 | tags=38%, list=13%, signal=44% |
| 575 | GOBP\_REGULATION\_OF\_HOMOTYPIC\_CELL\_CELL\_ADHESION |  | 20 | 0.52 | 1.46 | 0.060 | 0.481 | 1.000 | 1234 | tags=30%, list=10%, signal=33% |
| 576 | GOBP\_RESPONSE\_TO\_CELL\_CYCLE\_CHECKPOINT\_SIGNALING |  | 6 | 0.73 | 1.46 | 0.071 | 0.481 | 1.000 | 1611 | tags=50%, list=13%, signal=58% |
| 577 | GOBP\_CARDIAC\_MUSCLE\_TISSUE\_MORPHOGENESIS |  | 33 | 0.46 | 1.46 | 0.048 | 0.482 | 1.000 | 1800 | tags=33%, list=15%, signal=39% |
| 578 | GOMF\_CALCIUM\_CHANNEL\_INHIBITOR\_ACTIVITY |  | 8 | 0.66 | 1.46 | 0.078 | 0.482 | 1.000 | 2616 | tags=50%, list=22%, signal=64% |
| 579 | GOBP\_RESPONSE\_TO\_MONOSACCHARIDE |  | 130 | 0.36 | 1.46 | 0.013 | 0.481 | 1.000 | 2115 | tags=30%, list=18%, signal=36% |
| 580 | GOMF\_COLLAGEN\_BINDING |  | 54 | 0.42 | 1.46 | 0.034 | 0.481 | 1.000 | 4166 | tags=61%, list=35%, signal=93% |
| 581 | GOBP\_MONOCARBOXYLIC\_ACID\_BIOSYNTHETIC\_PROCESS |  | 145 | 0.35 | 1.46 | 0.011 | 0.481 | 1.000 | 1686 | tags=25%, list=14%, signal=29% |
| 582 | GOBP\_CARBOHYDRATE\_HOMEOSTASIS |  | 149 | 0.35 | 1.46 | 0.006 | 0.480 | 1.000 | 2049 | tags=28%, list=17%, signal=34% |
| 583 | GOBP\_RIBONUCLEOSIDE\_MONOPHOSPHATE\_CATABOLIC\_PROCESS |  | 8 | 0.66 | 1.46 | 0.071 | 0.480 | 1.000 | 140 | tags=25%, list=1%, signal=25% |
| 584 | GOMF\_SPECTRIN\_BINDING |  | 20 | 0.51 | 1.46 | 0.061 | 0.480 | 1.000 | 2625 | tags=40%, list=22%, signal=51% |
| 585 | GOBP\_COPI\_COATED\_VESICLE\_BUDDING |  | 5 | 0.75 | 1.46 | 0.064 | 0.480 | 1.000 | 1950 | tags=60%, list=16%, signal=72% |
| 586 | GOBP\_EPITHELIAL\_CELL\_PROLIFERATION\_INVOLVED\_IN\_LUNG\_MORPHOGENESIS |  | 8 | 0.66 | 1.46 | 0.076 | 0.479 | 1.000 | 2351 | tags=50%, list=20%, signal=62% |
| 587 | GOBP\_HEMOGLOBIN\_BIOSYNTHETIC\_PROCESS |  | 8 | 0.66 | 1.46 | 0.077 | 0.479 | 1.000 | 997 | tags=50%, list=8%, signal=54% |
| 588 | GOBP\_REGULATION\_OF\_LIPID\_TRANSPORT |  | 92 | 0.38 | 1.46 | 0.018 | 0.479 | 1.000 | 1223 | tags=21%, list=10%, signal=23% |
| 589 | GOCC\_ORGANELLE\_MEMBRANE\_CONTACT\_SITE |  | 31 | 0.47 | 1.46 | 0.048 | 0.479 | 1.000 | 2020 | tags=39%, list=17%, signal=46% |
| 590 | GOBP\_REGULATION\_OF\_ORGANIC\_ACID\_TRANSPORT |  | 37 | 0.45 | 1.46 | 0.039 | 0.479 | 1.000 | 1223 | tags=30%, list=10%, signal=33% |
| 591 | GOBP\_RECOMBINATIONAL\_REPAIR |  | 101 | 0.37 | 1.46 | 0.016 | 0.478 | 1.000 | 2631 | tags=32%, list=22%, signal=40% |
| 592 | GOBP\_POSITIVE\_REGULATION\_OF\_CELL\_PROLIFERATION\_INVOLVED\_IN\_HEART\_MORPHOGENESIS |  | 4 | 0.80 | 1.46 | 0.061 | 0.478 | 1.000 | 2351 | tags=75%, list=20%, signal=93% |
| 593 | GOBP\_REGULATION\_OF\_ASTROCYTE\_DIFFERENTIATION |  | 14 | 0.57 | 1.46 | 0.070 | 0.478 | 1.000 | 1379 | tags=29%, list=11%, signal=32% |
| 594 | GOBP\_GOLGI\_VESICLE\_TRANSPORT |  | 299 | 0.32 | 1.46 | 0.002 | 0.481 | 1.000 | 3011 | tags=35%, list=25%, signal=46% |
| 595 | GOBP\_NEGATIVE\_REGULATION\_OF\_VIRAL\_LIFE\_CYCLE |  | 9 | 0.64 | 1.46 | 0.074 | 0.480 | 1.000 | 957 | tags=33%, list=8%, signal=36% |
| 596 | GOBP\_CELLULAR\_RESPONSE\_TO\_CORTICOSTEROID\_STIMULUS |  | 43 | 0.44 | 1.46 | 0.039 | 0.481 | 1.000 | 1124 | tags=26%, list=9%, signal=28% |
| 597 | GOBP\_RESPONSE\_TO\_EXTRACELLULAR\_STIMULUS |  | 329 | 0.32 | 1.46 | 0.002 | 0.483 | 1.000 | 1427 | tags=19%, list=12%, signal=21% |
| 598 | GOBP\_POSITIVE\_REGULATION\_OF\_RESPONSE\_TO\_BIOTIC\_STIMULUS |  | 140 | 0.35 | 1.46 | 0.012 | 0.483 | 1.000 | 942 | tags=13%, list=8%, signal=14% |
| 599 | GOBP\_POSITIVE\_REGULATION\_OF\_FATTY\_ACID\_BETA\_OXIDATION |  | 7 | 0.69 | 1.46 | 0.076 | 0.483 | 1.000 | 2545 | tags=71%, list=21%, signal=91% |
| 600 | GOCC\_SPINDLE\_MICROTUBULE |  | 52 | 0.42 | 1.46 | 0.035 | 0.484 | 1.000 | 4279 | tags=58%, list=36%, signal=89% |
| 601 | GOBP\_DEADENYLATION\_DEPENDENT\_DECAPPING\_OF\_NUCLEAR\_TRANSCRIBED\_MRNA |  | 10 | 0.62 | 1.46 | 0.072 | 0.483 | 1.000 | 3796 | tags=40%, list=32%, signal=58% |
| 602 | GOBP\_ORGANIC\_HYDROXY\_COMPOUND\_METABOLIC\_PROCESS |  | 368 | 0.32 | 1.46 | 0.001 | 0.483 | 1.000 | 1777 | tags=24%, list=15%, signal=27% |
| 603 | GOBP\_REGULATION\_OF\_DNA\_DEPENDENT\_DNA\_REPLICATION |  | 43 | 0.43 | 1.46 | 0.038 | 0.482 | 1.000 | 2394 | tags=35%, list=20%, signal=43% |
| 604 | GOBP\_REGULATION\_OF\_EATING\_BEHAVIOR |  | 4 | 0.80 | 1.46 | 0.059 | 0.485 | 1.000 | 1771 | tags=75%, list=15%, signal=88% |
| 605 | GOBP\_BRUSH\_BORDER\_ASSEMBLY |  | 3 | 0.87 | 1.46 | 0.043 | 0.486 | 1.000 | 1030 | tags=67%, list=9%, signal=73% |
| 606 | GOBP\_CELL\_DIFFERENTIATION\_INVOLVED\_IN\_METANEPHROS\_DEVELOPMENT |  | 16 | 0.54 | 1.46 | 0.064 | 0.486 | 1.000 | 2680 | tags=50%, list=22%, signal=64% |
| 607 | GOBP\_SPINDLE\_ELONGATION |  | 7 | 0.68 | 1.46 | 0.078 | 0.486 | 1.000 | 3803 | tags=100%, list=32%, signal=146% |
| 608 | GOBP\_REGULATION\_OF\_FATTY\_ACID\_BIOSYNTHETIC\_PROCESS |  | 33 | 0.45 | 1.46 | 0.049 | 0.485 | 1.000 | 1559 | tags=30%, list=13%, signal=35% |
| 609 | GOBP\_DNA\_STRAND\_ELONGATION |  | 24 | 0.50 | 1.45 | 0.057 | 0.486 | 1.000 | 2225 | tags=46%, list=19%, signal=56% |
| 610 | GOBP\_CHONDROCYTE\_DEVELOPMENT |  | 17 | 0.54 | 1.45 | 0.065 | 0.485 | 1.000 | 2537 | tags=53%, list=21%, signal=67% |
| 611 | GOBP\_ACTIN\_FILAMENT\_BUNDLE\_ORGANIZATION |  | 121 | 0.36 | 1.45 | 0.011 | 0.486 | 1.000 | 2090 | tags=31%, list=17%, signal=38% |
| 612 | GOBP\_POSITIVE\_REGULATION\_OF\_ACTIN\_FILAMENT\_POLYMERIZATION |  | 64 | 0.40 | 1.45 | 0.032 | 0.486 | 1.000 | 2146 | tags=33%, list=18%, signal=40% |
| 613 | GOBP\_BLOOD\_COAGULATION\_INTRINSIC\_PATHWAY |  | 9 | 0.64 | 1.45 | 0.081 | 0.486 | 1.000 | 756 | tags=33%, list=6%, signal=36% |
| 614 | GOBP\_UDP\_N\_ACETYLGLUCOSAMINE\_METABOLIC\_PROCESS |  | 12 | 0.59 | 1.45 | 0.074 | 0.485 | 1.000 | 1621 | tags=42%, list=13%, signal=48% |
| 615 | GOCC\_TETRASPANIN\_ENRICHED\_MICRODOMAIN |  | 8 | 0.66 | 1.45 | 0.078 | 0.484 | 1.000 | 2539 | tags=63%, list=21%, signal=79% |
| 616 | GOCC\_COMPLEX\_OF\_COLLAGEN\_TRIMERS |  | 13 | 0.57 | 1.45 | 0.078 | 0.487 | 1.000 | 3082 | tags=69%, list=26%, signal=93% |
| 617 | GOCC\_HEMIDESMOSOME |  | 4 | 0.79 | 1.45 | 0.061 | 0.487 | 1.000 | 1603 | tags=75%, list=13%, signal=87% |
| 618 | GOBP\_REGULATION\_OF\_GLUCAN\_BIOSYNTHETIC\_PROCESS |  | 18 | 0.53 | 1.45 | 0.064 | 0.487 | 1.000 | 2368 | tags=56%, list=20%, signal=69% |
| 619 | GOBP\_REGULATION\_OF\_EXTRINSIC\_APOPTOTIC\_SIGNALING\_PATHWAY\_VIA\_DEATH\_DOMAIN\_RECEPTORS |  | 42 | 0.43 | 1.45 | 0.046 | 0.487 | 1.000 | 1710 | tags=29%, list=14%, signal=33% |
| 620 | GOBP\_BONE\_MATURATION |  | 14 | 0.56 | 1.45 | 0.074 | 0.487 | 1.000 | 2532 | tags=50%, list=21%, signal=63% |
| 621 | GOBP\_DNA\_DAMAGE\_INDUCED\_PROTEIN\_PHOSPHORYLATION |  | 8 | 0.66 | 1.45 | 0.067 | 0.487 | 1.000 | 2597 | tags=63%, list=22%, signal=80% |
| 622 | GOBP\_INTRACELLULAR\_STEROL\_TRANSPORT |  | 19 | 0.52 | 1.45 | 0.061 | 0.490 | 1.000 | 1926 | tags=42%, list=16%, signal=50% |
| 623 | GOBP\_NEGATIVE\_REGULATION\_OF\_LYASE\_ACTIVITY |  | 11 | 0.60 | 1.45 | 0.082 | 0.490 | 1.000 | 1386 | tags=36%, list=12%, signal=41% |
| 624 | GOBP\_COPII\_COATED\_VESICLE\_CARGO\_LOADING |  | 14 | 0.56 | 1.45 | 0.071 | 0.489 | 1.000 | 1088 | tags=29%, list=9%, signal=31% |
| 625 | GOBP\_ACTIN\_FILAMENT\_DEPOLYMERIZATION |  | 45 | 0.42 | 1.45 | 0.044 | 0.489 | 1.000 | 2183 | tags=36%, list=18%, signal=43% |
| 626 | GOBP\_REGULATION\_OF\_KERATINOCYTE\_MIGRATION |  | 13 | 0.57 | 1.45 | 0.084 | 0.491 | 1.000 | 2440 | tags=38%, list=20%, signal=48% |
| 627 | GOBP\_INTERLEUKIN\_23\_MEDIATED\_SIGNALING\_PATHWAY |  | 6 | 0.71 | 1.45 | 0.072 | 0.491 | 1.000 | 2978 | tags=83%, list=25%, signal=111% |
| 628 | GOBP\_REGULATION\_OF\_ACROSOME\_REACTION |  | 3 | 0.86 | 1.45 | 0.054 | 0.492 | 1.000 | 1420 | tags=67%, list=12%, signal=76% |
| 629 | GOCC\_POSTSYNAPTIC\_ENDOCYTIC\_ZONE |  | 5 | 0.75 | 1.45 | 0.072 | 0.494 | 1.000 | 2904 | tags=80%, list=24%, signal=105% |
| 630 | GOBP\_HEMOGLOBIN\_METABOLIC\_PROCESS |  | 10 | 0.62 | 1.45 | 0.073 | 0.497 | 1.000 | 997 | tags=40%, list=8%, signal=44% |
| 631 | GOCC\_CMG\_COMPLEX |  | 9 | 0.64 | 1.45 | 0.080 | 0.496 | 1.000 | 2631 | tags=78%, list=22%, signal=100% |
| 632 | GOBP\_ORGANOPHOSPHATE\_CATABOLIC\_PROCESS |  | 117 | 0.36 | 1.45 | 0.016 | 0.497 | 1.000 | 1752 | tags=25%, list=15%, signal=29% |
| 633 | GOBP\_RESPONSE\_TO\_ORGANOPHOSPHORUS |  | 82 | 0.38 | 1.45 | 0.027 | 0.496 | 1.000 | 1403 | tags=26%, list=12%, signal=29% |
| 634 | GOBP\_CELLULAR\_SENESCENCE |  | 54 | 0.41 | 1.45 | 0.034 | 0.496 | 1.000 | 1531 | tags=24%, list=13%, signal=27% |
| 635 | GOBP\_POSITIVE\_REGULATION\_OF\_PROTEIN\_LOCALIZATION\_TO\_EARLY\_ENDOSOME |  | 6 | 0.71 | 1.45 | 0.068 | 0.496 | 1.000 | 1922 | tags=50%, list=16%, signal=59% |
| 636 | GOBP\_ASTRAL\_MICROTUBULE\_ORGANIZATION |  | 10 | 0.61 | 1.45 | 0.082 | 0.496 | 1.000 | 913 | tags=40%, list=8%, signal=43% |
| 637 | GOBP\_EMBRYONIC\_HEART\_TUBE\_DEVELOPMENT |  | 49 | 0.42 | 1.45 | 0.038 | 0.497 | 1.000 | 934 | tags=16%, list=8%, signal=18% |
| 638 | GOBP\_NEGATIVE\_REGULATION\_OF\_TYPE\_B\_PANCREATIC\_CELL\_APOPTOTIC\_PROCESS |  | 5 | 0.75 | 1.45 | 0.067 | 0.497 | 1.000 | 607 | tags=40%, list=5%, signal=42% |
| 639 | GOBP\_REGULATION\_OF\_CELLULAR\_RESPONSE\_TO\_INSULIN\_STIMULUS |  | 48 | 0.42 | 1.45 | 0.042 | 0.496 | 1.000 | 2013 | tags=33%, list=17%, signal=40% |
| 640 | GOMF\_MANNOSYL\_OLIGOSACCHARIDE\_MANNOSIDASE\_ACTIVITY |  | 9 | 0.63 | 1.45 | 0.081 | 0.496 | 1.000 | 1253 | tags=33%, list=10%, signal=37% |
| 641 | GOBP\_POSITIVE\_REGULATION\_OF\_LYMPHOCYTE\_CHEMOTAXIS |  | 13 | 0.57 | 1.45 | 0.076 | 0.495 | 1.000 | 1945 | tags=54%, list=16%, signal=64% |
| 642 | GOMF\_PROFILIN\_BINDING |  | 8 | 0.65 | 1.44 | 0.082 | 0.495 | 1.000 | 3663 | tags=75%, list=30%, signal=108% |
| 643 | GOBP\_LONG\_CHAIN\_FATTY\_ACID\_IMPORT\_INTO\_CELL |  | 13 | 0.57 | 1.44 | 0.078 | 0.494 | 1.000 | 1396 | tags=46%, list=12%, signal=52% |
| 644 | GOCC\_CELL\_DIVISION\_SITE |  | 52 | 0.41 | 1.44 | 0.039 | 0.494 | 1.000 | 3111 | tags=44%, list=26%, signal=59% |
| 645 | GOBP\_REGULATION\_OF\_SECRETION |  | 388 | 0.31 | 1.44 | 0.001 | 0.493 | 1.000 | 1891 | tags=25%, list=16%, signal=28% |
| 646 | GOBP\_POSITIVE\_REGULATION\_OF\_BIOMINERALIZATION |  | 30 | 0.47 | 1.44 | 0.054 | 0.496 | 1.000 | 1918 | tags=30%, list=16%, signal=36% |
| 647 | GOBP\_REGULATION\_OF\_FOCAL\_ADHESION\_DISASSEMBLY |  | 6 | 0.71 | 1.44 | 0.087 | 0.496 | 1.000 | 1277 | tags=33%, list=11%, signal=37% |
| 648 | GOMF\_2\_ACYLGLYCEROL\_O\_ACYLTRANSFERASE\_ACTIVITY |  | 4 | 0.80 | 1.44 | 0.061 | 0.499 | 1.000 | 861 | tags=75%, list=7%, signal=81% |
| 649 | GOCC\_ENDOPLASMIC\_RETICULUM\_GOLGI\_INTERMEDIATE\_COMPARTMENT\_MEMBRANE |  | 55 | 0.41 | 1.44 | 0.035 | 0.500 | 1.000 | 2267 | tags=35%, list=19%, signal=42% |
| 650 | GOBP\_REGULATION\_OF\_NMDA\_RECEPTOR\_ACTIVITY |  | 17 | 0.53 | 1.44 | 0.072 | 0.499 | 1.000 | 1780 | tags=41%, list=15%, signal=48% |
| 651 | GOBP\_GOLGI\_LOCALIZATION |  | 13 | 0.57 | 1.44 | 0.074 | 0.500 | 1.000 | 2671 | tags=38%, list=22%, signal=49% |
| 652 | GOCC\_EGG\_COAT |  | 3 | 0.86 | 1.44 | 0.049 | 0.501 | 1.000 | 1420 | tags=67%, list=12%, signal=76% |
| 653 | GOCC\_PSEUDOPODIUM |  | 11 | 0.60 | 1.44 | 0.082 | 0.500 | 1.000 | 3764 | tags=64%, list=31%, signal=93% |
| 654 | GOBP\_VESICLE\_BUDDING\_FROM\_MEMBRANE |  | 87 | 0.38 | 1.44 | 0.023 | 0.502 | 1.000 | 3369 | tags=39%, list=28%, signal=54% |
| 655 | GOBP\_OTIC\_VESICLE\_FORMATION |  | 5 | 0.75 | 1.44 | 0.073 | 0.502 | 1.000 | 2274 | tags=60%, list=19%, signal=74% |
| 656 | GOBP\_MITOTIC\_CELL\_CYCLE\_CHECKPOINT |  | 127 | 0.35 | 1.44 | 0.014 | 0.503 | 1.000 | 4066 | tags=48%, list=34%, signal=72% |
| 657 | GOBP\_ATTACHMENT\_OF\_SPINDLE\_MICROTUBULES\_TO\_KINETOCHORE |  | 29 | 0.47 | 1.44 | 0.056 | 0.503 | 1.000 | 3884 | tags=66%, list=32%, signal=97% |
| 658 | GOMF\_FLAP\_ENDONUCLEASE\_ACTIVITY |  | 6 | 0.71 | 1.44 | 0.083 | 0.506 | 1.000 | 2781 | tags=67%, list=23%, signal=87% |
| 659 | GOBP\_HISTONE\_MRNA\_CATABOLIC\_PROCESS |  | 8 | 0.65 | 1.44 | 0.079 | 0.506 | 1.000 | 2829 | tags=38%, list=24%, signal=49% |
| 660 | GOBP\_PROTEIN\_LOCALIZATION\_TO\_ENDOSOME |  | 19 | 0.52 | 1.44 | 0.060 | 0.506 | 1.000 | 1922 | tags=37%, list=16%, signal=44% |
| 661 | GOMF\_LIPOPROTEIN\_LIPASE\_ACTIVITY |  | 3 | 0.85 | 1.44 | 0.051 | 0.507 | 1.000 | 954 | tags=67%, list=8%, signal=72% |
| 662 | GOBP\_MONOACYLGLYCEROL\_BIOSYNTHETIC\_PROCESS |  | 3 | 0.85 | 1.44 | 0.047 | 0.507 | 1.000 | 861 | tags=67%, list=7%, signal=72% |
| 663 | GOBP\_MITOTIC\_NUCLEAR\_DIVISION |  | 234 | 0.33 | 1.44 | 0.005 | 0.509 | 1.000 | 3884 | tags=47%, list=32%, signal=68% |
| 664 | GOBP\_IMPORT\_INTO\_CELL |  | 135 | 0.35 | 1.44 | 0.012 | 0.512 | 1.000 | 1436 | tags=25%, list=12%, signal=28% |
| 665 | GOBP\_CALCIUM\_ION\_IMPORT |  | 42 | 0.43 | 1.44 | 0.047 | 0.511 | 1.000 | 764 | tags=17%, list=6%, signal=18% |
| 666 | GOMF\_ACETYL\_COA\_HYDROLASE\_ACTIVITY |  | 5 | 0.74 | 1.44 | 0.077 | 0.510 | 1.000 | 572 | tags=40%, list=5%, signal=42% |
| 667 | GOBP\_NECROPTOTIC\_SIGNALING\_PATHWAY |  | 7 | 0.68 | 1.44 | 0.086 | 0.510 | 1.000 | 769 | tags=57%, list=6%, signal=61% |
| 668 | GOBP\_VESICLE\_MEDIATED\_TRANSPORT\_BETWEEN\_ENDOSOMAL\_COMPARTMENTS |  | 36 | 0.44 | 1.43 | 0.052 | 0.510 | 1.000 | 4749 | tags=67%, list=40%, signal=110% |
| 669 | GOMF\_CALCIUM\_DEPENDENT\_PROTEIN\_BINDING |  | 43 | 0.43 | 1.43 | 0.041 | 0.513 | 1.000 | 2538 | tags=35%, list=21%, signal=44% |
| 670 | GOCC\_CAVEOLA |  | 57 | 0.40 | 1.43 | 0.036 | 0.513 | 1.000 | 2261 | tags=32%, list=19%, signal=39% |
| 671 | GOBP\_CELL\_MIGRATION\_INVOLVED\_IN\_GASTRULATION |  | 9 | 0.63 | 1.43 | 0.091 | 0.514 | 1.000 | 1268 | tags=33%, list=11%, signal=37% |
| 672 | GOBP\_REGULATION\_OF\_PHOSPHATIDYLCHOLINE\_BIOSYNTHETIC\_PROCESS |  | 4 | 0.79 | 1.43 | 0.066 | 0.513 | 1.000 | 1917 | tags=75%, list=16%, signal=89% |
| 673 | GOBP\_PURINE\_CONTAINING\_COMPOUND\_CATABOLIC\_PROCESS |  | 37 | 0.44 | 1.43 | 0.050 | 0.513 | 1.000 | 1700 | tags=24%, list=14%, signal=28% |
| 674 | GOBP\_PHOSPHATIDYLCHOLINE\_METABOLIC\_PROCESS |  | 53 | 0.41 | 1.43 | 0.042 | 0.517 | 1.000 | 1917 | tags=38%, list=16%, signal=45% |
| 675 | GOBP\_PURINE\_DEOXYRIBONUCLEOTIDE\_CATABOLIC\_PROCESS |  | 5 | 0.74 | 1.43 | 0.084 | 0.518 | 1.000 | 1455 | tags=60%, list=12%, signal=68% |
| 676 | GOBP\_NEGATIVE\_REGULATION\_OF\_EXECUTION\_PHASE\_OF\_APOPTOSIS |  | 8 | 0.64 | 1.43 | 0.086 | 0.519 | 1.000 | 1181 | tags=38%, list=10%, signal=42% |
| 677 | GOBP\_CELLULAR\_RESPONSE\_TO\_FATTY\_ACID |  | 25 | 0.48 | 1.43 | 0.065 | 0.519 | 1.000 | 989 | tags=28%, list=8%, signal=30% |
| 678 | GOBP\_COLLAGEN\_FIBRIL\_ORGANIZATION |  | 40 | 0.43 | 1.43 | 0.052 | 0.519 | 1.000 | 3270 | tags=55%, list=27%, signal=75% |
| 679 | GOBP\_TOLL\_LIKE\_RECEPTOR\_SIGNALING\_PATHWAY |  | 104 | 0.36 | 1.43 | 0.022 | 0.521 | 1.000 | 623 | tags=13%, list=5%, signal=13% |
| 680 | GOBP\_CYTOKINE\_MEDIATED\_SIGNALING\_PATHWAY |  | 469 | 0.30 | 1.43 | 0.000 | 0.521 | 1.000 | 2169 | tags=24%, list=18%, signal=28% |
| 681 | GOCC\_KINETOCHORE |  | 116 | 0.36 | 1.43 | 0.021 | 0.521 | 1.000 | 3970 | tags=55%, list=33%, signal=82% |
| 682 | GOBP\_PANCREATIC\_JUICE\_SECRETION |  | 7 | 0.67 | 1.43 | 0.094 | 0.522 | 1.000 | 2349 | tags=57%, list=20%, signal=71% |
| 683 | GOBP\_REGULATION\_OF\_LYMPHOCYTE\_CHEMOTAXIS |  | 13 | 0.57 | 1.43 | 0.085 | 0.523 | 1.000 | 1945 | tags=54%, list=16%, signal=64% |
| 684 | GOMF\_PLATELET\_DERIVED\_GROWTH\_FACTOR\_RECEPTOR\_BINDING |  | 14 | 0.56 | 1.43 | 0.083 | 0.523 | 1.000 | 3323 | tags=36%, list=28%, signal=49% |
| 685 | GOBP\_MAINTENANCE\_OF\_ORGANELLE\_LOCATION |  | 7 | 0.67 | 1.43 | 0.087 | 0.522 | 1.000 | 3076 | tags=57%, list=26%, signal=77% |
| 686 | GOBP\_GANGLIOSIDE\_METABOLIC\_PROCESS |  | 18 | 0.52 | 1.43 | 0.085 | 0.521 | 1.000 | 153 | tags=17%, list=1%, signal=17% |
| 687 | GOBP\_DNA\_STRAND\_ELONGATION\_INVOLVED\_IN\_DNA\_REPLICATION |  | 17 | 0.52 | 1.43 | 0.085 | 0.521 | 1.000 | 2225 | tags=47%, list=19%, signal=58% |
| 688 | GOBP\_NEGATIVE\_REGULATION\_OF\_VIRAL\_ENTRY\_INTO\_HOST\_CELL |  | 8 | 0.64 | 1.43 | 0.093 | 0.521 | 1.000 | 957 | tags=38%, list=8%, signal=41% |
| 689 | GOBP\_NEGATIVE\_REGULATION\_OF\_ERK1\_AND\_ERK2\_CASCADE |  | 50 | 0.41 | 1.43 | 0.046 | 0.521 | 1.000 | 1249 | tags=22%, list=10%, signal=24% |
| 690 | GOMF\_MHC\_CLASS\_I\_PROTEIN\_BINDING |  | 10 | 0.61 | 1.43 | 0.090 | 0.521 | 1.000 | 430 | tags=30%, list=4%, signal=31% |
| 691 | GOCC\_BASEMENT\_MEMBRANE |  | 70 | 0.39 | 1.43 | 0.039 | 0.520 | 1.000 | 2936 | tags=37%, list=24%, signal=49% |
| 692 | GOBP\_NUCLEOSIDE\_PHOSPHATE\_CATABOLIC\_PROCESS |  | 62 | 0.39 | 1.43 | 0.038 | 0.520 | 1.000 | 1455 | tags=23%, list=12%, signal=26% |
| 693 | GOBP\_REGULATION\_OF\_FATTY\_ACID\_METABOLIC\_PROCESS |  | 66 | 0.39 | 1.43 | 0.040 | 0.520 | 1.000 | 1559 | tags=29%, list=13%, signal=33% |
| 694 | GOBP\_GOLGI\_VESICLE\_BUDDING |  | 9 | 0.62 | 1.43 | 0.097 | 0.522 | 1.000 | 2952 | tags=56%, list=25%, signal=74% |
| 695 | GOBP\_COAGULATION |  | 224 | 0.33 | 1.42 | 0.006 | 0.524 | 1.000 | 2956 | tags=36%, list=25%, signal=47% |
| 696 | GOBP\_MALE\_MEIOTIC\_NUCLEAR\_DIVISION |  | 25 | 0.48 | 1.42 | 0.068 | 0.524 | 1.000 | 3153 | tags=48%, list=26%, signal=65% |
| 697 | GOBP\_PURINE\_DEOXYRIBONUCLEOSIDE\_TRIPHOSPHATE\_CATABOLIC\_PROCESS |  | 4 | 0.78 | 1.42 | 0.082 | 0.523 | 1.000 | 585 | tags=50%, list=5%, signal=53% |
| 698 | GOBP\_ISOPENTENYL\_DIPHOSPHATE\_BIOSYNTHETIC\_PROCESS |  | 4 | 0.78 | 1.42 | 0.079 | 0.524 | 1.000 | 1777 | tags=50%, list=15%, signal=59% |
| 699 | GOBP\_RESPONSE\_TO\_WOUNDING |  | 431 | 0.31 | 1.42 | 0.002 | 0.524 | 1.000 | 2649 | tags=31%, list=22%, signal=38% |
| 700 | GOBP\_POSITIVE\_REGULATION\_OF\_SEQUESTERING\_OF\_TRIGLYCERIDE |  | 5 | 0.74 | 1.42 | 0.083 | 0.523 | 1.000 | 1299 | tags=60%, list=11%, signal=67% |
| 701 | GOBP\_MESENDODERM\_DEVELOPMENT |  | 4 | 0.78 | 1.42 | 0.073 | 0.523 | 1.000 | 1336 | tags=50%, list=11%, signal=56% |
| 702 | GOBP\_REGULATION\_OF\_TYPE\_B\_PANCREATIC\_CELL\_DEVELOPMENT |  | 5 | 0.74 | 1.42 | 0.088 | 0.522 | 1.000 | 2013 | tags=60%, list=17%, signal=72% |
| 703 | GOCC\_AXON\_HILLOCK |  | 6 | 0.71 | 1.42 | 0.091 | 0.523 | 1.000 | 3511 | tags=83%, list=29%, signal=118% |
| 704 | GOMF\_INTERMEDIATE\_FILAMENT\_BINDING |  | 8 | 0.65 | 1.42 | 0.096 | 0.522 | 1.000 | 1119 | tags=38%, list=9%, signal=41% |
| 705 | GOBP\_WOUND\_HEALING |  | 354 | 0.31 | 1.42 | 0.001 | 0.522 | 1.000 | 3082 | tags=35%, list=26%, signal=46% |
| 706 | GOBP\_REGULATION\_OF\_APOPTOTIC\_DNA\_FRAGMENTATION |  | 5 | 0.73 | 1.42 | 0.088 | 0.522 | 1.000 | 2246 | tags=80%, list=19%, signal=98% |
| 707 | GOBP\_URINARY\_TRACT\_SMOOTH\_MUSCLE\_CONTRACTION |  | 6 | 0.69 | 1.42 | 0.094 | 0.521 | 1.000 | 452 | tags=33%, list=4%, signal=35% |
| 708 | GOBP\_FATTY\_ACID\_BIOSYNTHETIC\_PROCESS |  | 105 | 0.36 | 1.42 | 0.018 | 0.522 | 1.000 | 1682 | tags=24%, list=14%, signal=27% |
| 709 | GOMF\_PHOSPHOLIPID\_BINDING |  | 327 | 0.31 | 1.42 | 0.003 | 0.522 | 1.000 | 1867 | tags=24%, list=16%, signal=27% |
| 710 | GOBP\_NEGATIVE\_REGULATION\_OF\_FATTY\_ACID\_BETA\_OXIDATION |  | 5 | 0.73 | 1.42 | 0.082 | 0.522 | 1.000 | 1873 | tags=60%, list=16%, signal=71% |
| 711 | GOMF\_PROTEIN\_SERINE\_THREONINE\_KINASE\_ACTIVATOR\_ACTIVITY |  | 30 | 0.46 | 1.42 | 0.066 | 0.521 | 1.000 | 2139 | tags=30%, list=18%, signal=36% |
| 712 | GOCC\_U2\_SNRNP |  | 18 | 0.51 | 1.42 | 0.084 | 0.521 | 1.000 | 3807 | tags=56%, list=32%, signal=81% |
| 713 | GOBP\_REGULATION\_OF\_TRIGLYCERIDE\_METABOLIC\_PROCESS |  | 23 | 0.49 | 1.42 | 0.077 | 0.521 | 1.000 | 733 | tags=30%, list=6%, signal=32% |
| 714 | GOBP\_MOTOR\_NEURON\_MIGRATION |  | 3 | 0.85 | 1.42 | 0.058 | 0.521 | 1.000 | 1794 | tags=100%, list=15%, signal=118% |
| 715 | GOBP\_THIOESTER\_METABOLIC\_PROCESS |  | 79 | 0.38 | 1.42 | 0.033 | 0.523 | 1.000 | 1433 | tags=23%, list=12%, signal=26% |
| 716 | GOBP\_BONE\_GROWTH |  | 17 | 0.52 | 1.42 | 0.083 | 0.523 | 1.000 | 2436 | tags=41%, list=20%, signal=52% |
| 717 | GOBP\_INTERMEDIATE\_FILAMENT\_BASED\_PROCESS |  | 29 | 0.46 | 1.42 | 0.060 | 0.523 | 1.000 | 3562 | tags=55%, list=30%, signal=78% |
| 718 | GOCC\_MICROFIBRIL |  | 7 | 0.67 | 1.42 | 0.095 | 0.524 | 1.000 | 2954 | tags=57%, list=25%, signal=76% |
| 719 | GOBP\_REGULATION\_OF\_ION\_TRANSPORT |  | 832 | 0.29 | 1.42 | 0.000 | 0.524 | 1.000 | 2056 | tags=23%, list=17%, signal=26% |
| 720 | GOMF\_CARNITINE\_O\_ACYLTRANSFERASE\_ACTIVITY |  | 4 | 0.79 | 1.42 | 0.079 | 0.525 | 1.000 | 1915 | tags=50%, list=16%, signal=59% |
| 721 | GOBP\_NEGATIVE\_REGULATION\_OF\_CELLULAR\_RESPONSE\_TO\_VASCULAR\_ENDOTHELIAL\_GROWTH\_FACTOR\_STIMULUS |  | 9 | 0.62 | 1.42 | 0.086 | 0.524 | 1.000 | 3792 | tags=67%, list=32%, signal=97% |
| 722 | GOCC\_MITOTIC\_SPINDLE\_POLE |  | 28 | 0.46 | 1.42 | 0.077 | 0.524 | 1.000 | 3959 | tags=54%, list=33%, signal=80% |
| 723 | GOCC\_ZONULA\_ADHERENS |  | 5 | 0.74 | 1.42 | 0.087 | 0.525 | 1.000 | 2131 | tags=80%, list=18%, signal=97% |
| 724 | GOMF\_DOLICHYL\_PHOSPHATE\_MANNOSE\_PROTEIN\_MANNOSYLTRANSFERASE\_ACTIVITY |  | 8 | 0.64 | 1.42 | 0.099 | 0.527 | 1.000 | 2370 | tags=50%, list=20%, signal=62% |
| 725 | GOBP\_POSITIVE\_REGULATION\_OF\_MEMBRANE\_POTENTIAL |  | 10 | 0.60 | 1.42 | 0.095 | 0.526 | 1.000 | 1648 | tags=20%, list=14%, signal=23% |
| 726 | GOBP\_RESPONSE\_TO\_CAMPTOTHECIN |  | 4 | 0.79 | 1.42 | 0.078 | 0.527 | 1.000 | 1419 | tags=50%, list=12%, signal=57% |
| 727 | GOBP\_CYTOKINE\_PRODUCTION |  | 444 | 0.30 | 1.42 | 0.001 | 0.526 | 1.000 | 1326 | tags=18%, list=11%, signal=19% |
| 728 | GOBP\_ENERGY\_HOMEOSTASIS |  | 27 | 0.47 | 1.42 | 0.062 | 0.527 | 1.000 | 1771 | tags=33%, list=15%, signal=39% |
| 729 | GOBP\_CARDIAC\_MUSCLE\_CELL\_FATE\_COMMITMENT |  | 3 | 0.85 | 1.42 | 0.061 | 0.527 | 1.000 | 1394 | tags=67%, list=12%, signal=75% |
| 730 | GOMF\_BUTYRATE\_COA\_LIGASE\_ACTIVITY |  | 3 | 0.85 | 1.42 | 0.063 | 0.527 | 1.000 | 1171 | tags=67%, list=10%, signal=74% |
| 731 | GOBP\_POST\_EMBRYONIC\_CAMERA\_TYPE\_EYE\_DEVELOPMENT |  | 4 | 0.78 | 1.42 | 0.076 | 0.528 | 1.000 | 1794 | tags=75%, list=15%, signal=88% |
| 732 | GOBP\_CRANIAL\_SUTURE\_MORPHOGENESIS |  | 5 | 0.73 | 1.42 | 0.097 | 0.529 | 1.000 | 2545 | tags=80%, list=21%, signal=101% |
| 733 | GOMF\_CARBOXYPEPTIDASE\_ACTIVITY |  | 25 | 0.47 | 1.42 | 0.074 | 0.528 | 1.000 | 2700 | tags=56%, list=22%, signal=72% |
| 734 | GOBP\_CELL\_CELL\_JUNCTION\_ASSEMBLY |  | 97 | 0.36 | 1.42 | 0.029 | 0.528 | 1.000 | 2609 | tags=31%, list=22%, signal=39% |
| 735 | GOMF\_ORGANIC\_HYDROXY\_COMPOUND\_TRANSMEMBRANE\_TRANSPORTER\_ACTIVITY |  | 26 | 0.47 | 1.42 | 0.072 | 0.529 | 1.000 | 1403 | tags=35%, list=12%, signal=39% |
| 736 | GOBP\_MOTILE\_CILIUM\_ASSEMBLY |  | 9 | 0.62 | 1.41 | 0.089 | 0.529 | 1.000 | 63 | tags=11%, list=1%, signal=11% |
| 737 | GOBP\_CELLULAR\_RESPONSE\_TO\_ALCOHOL |  | 63 | 0.39 | 1.41 | 0.035 | 0.530 | 1.000 | 1693 | tags=25%, list=14%, signal=29% |
| 738 | GOBP\_SPROUTING\_ANGIOGENESIS |  | 90 | 0.37 | 1.41 | 0.028 | 0.530 | 1.000 | 2649 | tags=34%, list=22%, signal=44% |
| 739 | GOBP\_ESTABLISHMENT\_OF\_VIRAL\_LATENCY |  | 9 | 0.62 | 1.41 | 0.100 | 0.529 | 1.000 | 1495 | tags=33%, list=12%, signal=38% |
| 740 | GOBP\_FATTY\_ACID\_TRANSPORT |  | 99 | 0.36 | 1.41 | 0.027 | 0.529 | 1.000 | 1915 | tags=32%, list=16%, signal=38% |
| 741 | GOBP\_CELL\_DIFFERENTIATION\_INVOLVED\_IN\_PHENOTYPIC\_SWITCHING |  | 3 | 0.84 | 1.41 | 0.063 | 0.529 | 1.000 | 575 | tags=33%, list=5%, signal=35% |
| 742 | GOBP\_ORGANIC\_ACID\_TRANSPORT |  | 206 | 0.33 | 1.41 | 0.007 | 0.529 | 1.000 | 1526 | tags=25%, list=13%, signal=28% |
| 743 | GOBP\_REGULATION\_OF\_LAMELLIPODIUM\_MORPHOGENESIS |  | 9 | 0.62 | 1.41 | 0.098 | 0.529 | 1.000 | 1722 | tags=44%, list=14%, signal=52% |
| 744 | GOCC\_ENDOLYSOSOME |  | 17 | 0.52 | 1.41 | 0.088 | 0.528 | 1.000 | 1107 | tags=24%, list=9%, signal=26% |
| 745 | GOMF\_O\_PALMITOYLTRANSFERASE\_ACTIVITY |  | 3 | 0.84 | 1.41 | 0.060 | 0.528 | 1.000 | 1915 | tags=100%, list=16%, signal=119% |
| 746 | GOBP\_POSITIVE\_REGULATION\_OF\_SODIUM\_ION\_TRANSMEMBRANE\_TRANSPORT |  | 15 | 0.53 | 1.41 | 0.094 | 0.531 | 1.000 | 2044 | tags=33%, list=17%, signal=40% |
| 747 | GOBP\_REGULATION\_OF\_GLYCOGEN\_METABOLIC\_PROCESS |  | 23 | 0.48 | 1.41 | 0.079 | 0.530 | 1.000 | 2368 | tags=48%, list=20%, signal=59% |
| 748 | GOBP\_REGULATION\_OF\_LUNG\_BLOOD\_PRESSURE |  | 3 | 0.84 | 1.41 | 0.067 | 0.532 | 1.000 | 934 | tags=67%, list=8%, signal=72% |
| 749 | GOBP\_N\_GLYCAN\_PROCESSING |  | 15 | 0.53 | 1.41 | 0.088 | 0.531 | 1.000 | 1253 | tags=27%, list=10%, signal=30% |
| 750 | GOBP\_INTERFERON\_GAMMA\_PRODUCTION |  | 57 | 0.40 | 1.41 | 0.049 | 0.532 | 1.000 | 1198 | tags=23%, list=10%, signal=25% |
| 751 | GOMF\_S100\_PROTEIN\_BINDING |  | 9 | 0.62 | 1.41 | 0.097 | 0.533 | 1.000 | 2287 | tags=56%, list=19%, signal=69% |
| 752 | GOBP\_DENTINOGENESIS |  | 3 | 0.84 | 1.41 | 0.076 | 0.532 | 1.000 | 626 | tags=33%, list=5%, signal=35% |
| 753 | GOBP\_RESPONSE\_TO\_LIGHT\_INTENSITY |  | 3 | 0.84 | 1.41 | 0.066 | 0.532 | 1.000 | 30 | tags=33%, list=0%, signal=33% |
| 754 | GOCC\_TRIGLYCERIDE\_RICH\_PLASMA\_LIPOPROTEIN\_PARTICLE |  | 9 | 0.62 | 1.41 | 0.091 | 0.533 | 1.000 | 954 | tags=44%, list=8%, signal=48% |
| 755 | GOBP\_NEGATIVE\_REGULATION\_OF\_PLATELET\_ACTIVATION |  | 12 | 0.56 | 1.41 | 0.100 | 0.533 | 1.000 | 4215 | tags=58%, list=35%, signal=90% |
| 756 | GOBP\_RESPONSE\_TO\_HYDROSTATIC\_PRESSURE |  | 5 | 0.72 | 1.41 | 0.085 | 0.532 | 1.000 | 820 | tags=40%, list=7%, signal=43% |
| 757 | GOBP\_PYRIMIDINE\_DEOXYRIBONUCLEOTIDE\_METABOLIC\_PROCESS |  | 17 | 0.51 | 1.41 | 0.088 | 0.532 | 1.000 | 904 | tags=18%, list=8%, signal=19% |
| 758 | GOMF\_PROTEIN\_SERINE\_KINASE\_ACTIVITY |  | 178 | 0.33 | 1.41 | 0.014 | 0.532 | 1.000 | 2652 | tags=32%, list=22%, signal=40% |
| 759 | GOBP\_MITOTIC\_CHROMOSOME\_CONDENSATION |  | 11 | 0.58 | 1.41 | 0.091 | 0.532 | 1.000 | 3251 | tags=73%, list=27%, signal=100% |
| 760 | GOBP\_REGULATION\_OF\_FATTY\_ACID\_OXIDATION |  | 28 | 0.46 | 1.41 | 0.066 | 0.532 | 1.000 | 1368 | tags=32%, list=11%, signal=36% |
| 761 | GOBP\_ACTIVATION\_OF\_CYSTEINE\_TYPE\_ENDOPEPTIDASE\_ACTIVITY\_INVOLVED\_IN\_APOPTOTIC\_PROCESS |  | 64 | 0.39 | 1.41 | 0.038 | 0.532 | 1.000 | 1604 | tags=28%, list=13%, signal=32% |
| 762 | GOCC\_GOLGI\_ASSOCIATED\_VESICLE\_MEMBRANE |  | 37 | 0.43 | 1.41 | 0.067 | 0.531 | 1.000 | 3853 | tags=65%, list=32%, signal=95% |
| 763 | GOBP\_HOMEOSTASIS\_OF\_NUMBER\_OF\_CELLS\_WITHIN\_A\_TISSUE |  | 19 | 0.50 | 1.41 | 0.081 | 0.534 | 1.000 | 2458 | tags=42%, list=20%, signal=53% |
| 764 | GOBP\_MESODERM\_MORPHOGENESIS |  | 43 | 0.42 | 1.41 | 0.059 | 0.534 | 1.000 | 1800 | tags=26%, list=15%, signal=30% |
| 765 | GOBP\_MULTIVESICULAR\_BODY\_SORTING\_PATHWAY |  | 30 | 0.45 | 1.41 | 0.072 | 0.535 | 1.000 | 3802 | tags=53%, list=32%, signal=78% |
| 766 | GOBP\_POSITIVE\_REGULATION\_OF\_INSULIN\_SECRETION\_INVOLVED\_IN\_CELLULAR\_RESPONSE\_TO\_GLUCOSE\_STIMULUS |  | 20 | 0.50 | 1.41 | 0.088 | 0.535 | 1.000 | 2049 | tags=45%, list=17%, signal=54% |
| 767 | GOMF\_ARACHIDONATE\_COA\_LIGASE\_ACTIVITY |  | 10 | 0.60 | 1.41 | 0.098 | 0.534 | 1.000 | 1433 | tags=60%, list=12%, signal=68% |
| 768 | GOBP\_RESPONSE\_TO\_OSMOTIC\_STRESS |  | 53 | 0.40 | 1.41 | 0.044 | 0.536 | 1.000 | 1537 | tags=25%, list=13%, signal=28% |
| 769 | GOBP\_REGULATION\_OF\_PROTEIN\_EXIT\_FROM\_ENDOPLASMIC\_RETICULUM |  | 22 | 0.49 | 1.41 | 0.088 | 0.538 | 1.000 | 892 | tags=18%, list=7%, signal=20% |
| 770 | GOBP\_INTERMEDIATE\_FILAMENT\_ORGANIZATION |  | 11 | 0.58 | 1.41 | 0.093 | 0.538 | 1.000 | 401 | tags=18%, list=3%, signal=19% |
| 771 | GOCC\_EXTERNAL\_ENCAPSULATING\_STRUCTURE |  | 329 | 0.31 | 1.41 | 0.004 | 0.539 | 1.000 | 2954 | tags=37%, list=25%, signal=47% |
| 772 | GOBP\_NEGATIVE\_REGULATION\_OF\_PLATELET\_AGGREGATION |  | 8 | 0.63 | 1.40 | 0.104 | 0.540 | 1.000 | 4215 | tags=75%, list=35%, signal=115% |
| 773 | GOMF\_PROTEIN\_SERINE\_THREONINE\_KINASE\_ACTIVITY |  | 315 | 0.31 | 1.40 | 0.005 | 0.539 | 1.000 | 2614 | tags=29%, list=22%, signal=36% |
| 774 | GOBP\_ASSEMBLY\_OF\_ACTOMYOSIN\_APPARATUS\_INVOLVED\_IN\_CYTOKINESIS |  | 5 | 0.72 | 1.40 | 0.100 | 0.540 | 1.000 | 3045 | tags=80%, list=25%, signal=107% |
| 775 | GOBP\_PHOSPHATIDYLCHOLINE\_CATABOLIC\_PROCESS |  | 10 | 0.60 | 1.40 | 0.095 | 0.540 | 1.000 | 448 | tags=30%, list=4%, signal=31% |
| 776 | GOCC\_CONDENSED\_CHROMOSOME\_OUTER\_KINETOCHORE |  | 11 | 0.58 | 1.40 | 0.100 | 0.541 | 1.000 | 3724 | tags=91%, list=31%, signal=132% |
| 777 | GOBP\_NEGATIVE\_REGULATION\_OF\_CELL\_MORPHOGENESIS\_INVOLVED\_IN\_DIFFERENTIATION |  | 12 | 0.56 | 1.40 | 0.101 | 0.540 | 1.000 | 784 | tags=25%, list=7%, signal=27% |
| 778 | GOBP\_NEGATIVE\_REGULATION\_OF\_FATTY\_ACID\_METABOLIC\_PROCESS |  | 17 | 0.52 | 1.40 | 0.093 | 0.540 | 1.000 | 1873 | tags=35%, list=16%, signal=42% |
| 779 | GOBP\_POSITIVE\_REGULATION\_OF\_SECRETION |  | 194 | 0.33 | 1.40 | 0.011 | 0.540 | 1.000 | 1891 | tags=25%, list=16%, signal=29% |
| 780 | GOCC\_PLASMA\_MEMBRANE\_REGION |  | 747 | 0.29 | 1.40 | 0.001 | 0.543 | 1.000 | 2050 | tags=24%, list=17%, signal=27% |
| 781 | GOBP\_POSITIVE\_REGULATION\_OF\_PROTEIN\_CATABOLIC\_PROCESS\_IN\_THE\_VACUOLE |  | 5 | 0.72 | 1.40 | 0.093 | 0.546 | 1.000 | 180 | tags=40%, list=1%, signal=41% |
| 782 | GOBP\_POSITIVE\_REGULATION\_OF\_CELL\_MIGRATION\_INVOLVED\_IN\_SPROUTING\_ANGIOGENESIS |  | 15 | 0.54 | 1.40 | 0.101 | 0.546 | 1.000 | 2764 | tags=47%, list=23%, signal=61% |
| 783 | GOBP\_POSITIVE\_REGULATION\_OF\_PROTEIN\_POLYMERIZATION |  | 88 | 0.37 | 1.40 | 0.035 | 0.546 | 1.000 | 2875 | tags=38%, list=24%, signal=49% |
| 784 | GOMF\_CYTOKINE\_RECEPTOR\_BINDING |  | 124 | 0.34 | 1.40 | 0.028 | 0.547 | 1.000 | 2146 | tags=26%, list=18%, signal=31% |
| 785 | GOBP\_ERBB\_SIGNALING\_PATHWAY |  | 114 | 0.35 | 1.40 | 0.027 | 0.548 | 1.000 | 2380 | tags=26%, list=20%, signal=33% |
| 786 | GOBP\_REGULATION\_OF\_FATTY\_ACID\_BETA\_OXIDATION |  | 16 | 0.53 | 1.40 | 0.094 | 0.548 | 1.000 | 2545 | tags=56%, list=21%, signal=71% |
| 787 | GOBP\_PRE\_B\_CELL\_DIFFERENTIATION |  | 4 | 0.78 | 1.40 | 0.095 | 0.549 | 1.000 | 2597 | tags=75%, list=22%, signal=96% |
| 788 | GOBP\_NEGATIVE\_REGULATION\_OF\_VASCULAR\_ASSOCIATED\_SMOOTH\_MUSCLE\_CELL\_PROLIFERATION |  | 15 | 0.53 | 1.40 | 0.094 | 0.549 | 1.000 | 3196 | tags=40%, list=27%, signal=54% |
| 789 | GOBP\_POSITIVE\_REGULATION\_OF\_DENDRITIC\_SPINE\_DEVELOPMENT |  | 33 | 0.44 | 1.40 | 0.073 | 0.548 | 1.000 | 3829 | tags=52%, list=32%, signal=75% |
| 790 | GOBP\_POSITIVE\_REGULATION\_OF\_TRANSPORT |  | 608 | 0.29 | 1.40 | 0.000 | 0.548 | 1.000 | 2171 | tags=24%, list=18%, signal=28% |
| 791 | GOBP\_HISTONE\_H4\_R3\_METHYLATION |  | 3 | 0.83 | 1.40 | 0.074 | 0.549 | 1.000 | 1309 | tags=67%, list=11%, signal=75% |
| 792 | GOBP\_PROTEIN\_LOCALIZATION\_TO\_MICROTUBULE\_ORGANIZING\_CENTER |  | 29 | 0.46 | 1.40 | 0.071 | 0.549 | 1.000 | 4415 | tags=55%, list=37%, signal=87% |
| 793 | GOBP\_CELLULAR\_RESPONSE\_TO\_FRUCTOSE\_STIMULUS |  | 5 | 0.72 | 1.40 | 0.108 | 0.549 | 1.000 | 1056 | tags=60%, list=9%, signal=66% |
| 794 | GOBP\_CENTROSOME\_DUPLICATION |  | 59 | 0.39 | 1.40 | 0.048 | 0.550 | 1.000 | 3045 | tags=44%, list=25%, signal=59% |
| 795 | GOBP\_RETINAL\_CONE\_CELL\_DIFFERENTIATION |  | 5 | 0.72 | 1.40 | 0.102 | 0.549 | 1.000 | 872 | tags=40%, list=7%, signal=43% |
| 796 | GOBP\_DEOXYRIBONUCLEOSIDE\_TRIPHOSPHATE\_CATABOLIC\_PROCESS |  | 6 | 0.68 | 1.40 | 0.101 | 0.549 | 1.000 | 3228 | tags=83%, list=27%, signal=114% |
| 797 | GOMF\_INOSITOL\_1\_4\_5\_TRISPHOSPHATE\_BINDING |  | 9 | 0.61 | 1.40 | 0.099 | 0.548 | 1.000 | 1663 | tags=44%, list=14%, signal=52% |
| 798 | GOCC\_CORTICAL\_CYTOSKELETON |  | 79 | 0.37 | 1.40 | 0.037 | 0.548 | 1.000 | 2236 | tags=29%, list=19%, signal=36% |
| 799 | GOBP\_NECROTIC\_CELL\_DEATH |  | 38 | 0.43 | 1.40 | 0.064 | 0.549 | 1.000 | 769 | tags=21%, list=6%, signal=22% |
| 800 | GOBP\_LEFT\_RIGHT\_AXIS\_SPECIFICATION |  | 10 | 0.59 | 1.40 | 0.097 | 0.548 | 1.000 | 55 | tags=10%, list=0%, signal=10% |
| 801 | GOBP\_SPINDLE\_LOCALIZATION |  | 42 | 0.42 | 1.40 | 0.059 | 0.549 | 1.000 | 3867 | tags=57%, list=32%, signal=84% |
| 802 | GOMF\_CYSTEINE\_TYPE\_ENDOPEPTIDASE\_ACTIVITY\_INVOLVED\_IN\_APOPTOTIC\_SIGNALING\_PATHWAY |  | 6 | 0.69 | 1.40 | 0.109 | 0.548 | 1.000 | 3466 | tags=83%, list=29%, signal=117% |
| 803 | GOBP\_POSITIVE\_REGULATION\_OF\_TOLL\_LIKE\_RECEPTOR\_SIGNALING\_PATHWAY |  | 19 | 0.50 | 1.40 | 0.087 | 0.547 | 1.000 | 262 | tags=16%, list=2%, signal=16% |
| 804 | GOBP\_APICAL\_JUNCTION\_ASSEMBLY |  | 56 | 0.39 | 1.40 | 0.047 | 0.548 | 1.000 | 3629 | tags=45%, list=30%, signal=64% |
| 805 | GOBP\_POSITIVE\_REGULATION\_OF\_PEPTIDYL\_TYROSINE\_PHOSPHORYLATION |  | 120 | 0.35 | 1.40 | 0.031 | 0.549 | 1.000 | 2017 | tags=28%, list=17%, signal=33% |
| 806 | GOBP\_MRNA\_CLEAVAGE |  | 15 | 0.53 | 1.40 | 0.095 | 0.550 | 1.000 | 27 | tags=7%, list=0%, signal=7% |
| 807 | GOCC\_PRECATALYTIC\_SPLICEOSOME |  | 42 | 0.42 | 1.39 | 0.068 | 0.550 | 1.000 | 3959 | tags=50%, list=33%, signal=74% |
| 808 | GOBP\_RESPONSE\_TO\_MUSCLE\_ACTIVITY |  | 18 | 0.50 | 1.39 | 0.092 | 0.551 | 1.000 | 308 | tags=17%, list=3%, signal=17% |
| 809 | GOBP\_NEGATIVE\_REGULATION\_OF\_CELLULAR\_RESPONSE\_TO\_INSULIN\_STIMULUS |  | 29 | 0.45 | 1.39 | 0.073 | 0.554 | 1.000 | 2013 | tags=34%, list=17%, signal=41% |
| 810 | GOBP\_CELL\_VOLUME\_HOMEOSTASIS |  | 21 | 0.48 | 1.39 | 0.090 | 0.555 | 1.000 | 1150 | tags=24%, list=10%, signal=26% |
| 811 | GOCC\_ELASTIC\_FIBER |  | 3 | 0.84 | 1.39 | 0.074 | 0.555 | 1.000 | 1718 | tags=67%, list=14%, signal=78% |
| 812 | GOBP\_CELLULAR\_RESPONSE\_TO\_CARBOHYDRATE\_STIMULUS |  | 89 | 0.36 | 1.39 | 0.037 | 0.555 | 1.000 | 2049 | tags=29%, list=17%, signal=35% |
| 813 | GOMF\_AZOLE\_TRANSMEMBRANE\_TRANSPORTER\_ACTIVITY |  | 7 | 0.65 | 1.39 | 0.113 | 0.555 | 1.000 | 388 | tags=29%, list=3%, signal=30% |
| 814 | GOBP\_NEGATIVE\_REGULATION\_OF\_ERAD\_PATHWAY |  | 5 | 0.71 | 1.39 | 0.109 | 0.555 | 1.000 | 2968 | tags=60%, list=25%, signal=80% |
| 815 | GOBP\_RESPONSE\_TO\_ATP |  | 22 | 0.48 | 1.39 | 0.076 | 0.554 | 1.000 | 1245 | tags=32%, list=10%, signal=35% |
| 816 | GOCC\_SARCOPLASMIC\_RETICULUM\_LUMEN |  | 4 | 0.77 | 1.39 | 0.097 | 0.556 | 1.000 | 1756 | tags=75%, list=15%, signal=88% |
| 817 | GOBP\_GLYCEROLIPID\_METABOLIC\_PROCESS |  | 316 | 0.31 | 1.39 | 0.005 | 0.556 | 1.000 | 1977 | tags=27%, list=16%, signal=32% |
| 818 | GOBP\_EXTRINSIC\_APOPTOTIC\_SIGNALING\_PATHWAY\_VIA\_DEATH\_DOMAIN\_RECEPTORS |  | 56 | 0.39 | 1.39 | 0.050 | 0.556 | 1.000 | 2138 | tags=27%, list=18%, signal=32% |
| 819 | GOBP\_DEOXYRIBONUCLEOSIDE\_TRIPHOSPHATE\_METABOLIC\_PROCESS |  | 12 | 0.56 | 1.39 | 0.104 | 0.556 | 1.000 | 585 | tags=25%, list=5%, signal=26% |
| 820 | GOBP\_REGULATION\_OF\_ASPARTIC\_TYPE\_PEPTIDASE\_ACTIVITY |  | 8 | 0.63 | 1.39 | 0.106 | 0.557 | 1.000 | 1922 | tags=38%, list=16%, signal=45% |
| 821 | GOBP\_TRYPTOPHAN\_CATABOLIC\_PROCESS\_TO\_KYNURENINE |  | 3 | 0.83 | 1.39 | 0.086 | 0.557 | 1.000 | 1295 | tags=67%, list=11%, signal=75% |
| 822 | GOBP\_BIOMINERALIZATION |  | 98 | 0.36 | 1.39 | 0.034 | 0.557 | 1.000 | 2604 | tags=33%, list=22%, signal=41% |
| 823 | GOBP\_ANIMAL\_ORGAN\_MATURATION |  | 17 | 0.51 | 1.39 | 0.094 | 0.557 | 1.000 | 2683 | tags=47%, list=22%, signal=61% |
| 824 | GOBP\_MONOACYLGLYCEROL\_METABOLIC\_PROCESS |  | 8 | 0.63 | 1.39 | 0.113 | 0.559 | 1.000 | 1752 | tags=50%, list=15%, signal=58% |
| 825 | GOBP\_NEGATIVE\_REGULATION\_OF\_PROTEIN\_CONTAINING\_COMPLEX\_DISASSEMBLY |  | 57 | 0.39 | 1.39 | 0.051 | 0.559 | 1.000 | 2090 | tags=26%, list=17%, signal=32% |
| 826 | GOCC\_FACIT\_COLLAGEN\_TRIMER |  | 5 | 0.72 | 1.39 | 0.104 | 0.558 | 1.000 | 242 | tags=20%, list=2%, signal=20% |
| 827 | GOCC\_COMMITMENT\_COMPLEX |  | 5 | 0.71 | 1.39 | 0.111 | 0.558 | 1.000 | 455 | tags=20%, list=4%, signal=21% |
| 828 | GOBP\_FEMALE\_MEIOSIS\_CHROMOSOME\_SEGREGATION |  | 5 | 0.73 | 1.39 | 0.110 | 0.559 | 1.000 | 3255 | tags=80%, list=27%, signal=110% |
| 829 | GOBP\_UBIQUITIN\_DEPENDENT\_GLYCOPROTEIN\_ERAD\_PATHWAY |  | 5 | 0.71 | 1.39 | 0.104 | 0.560 | 1.000 | 62 | tags=20%, list=1%, signal=20% |
| 830 | GOBP\_CELL\_FATE\_COMMITMENT |  | 132 | 0.34 | 1.39 | 0.022 | 0.559 | 1.000 | 1831 | tags=24%, list=15%, signal=28% |
| 831 | GOBP\_REGULATION\_OF\_TRANSLATIONAL\_INITIATION\_BY\_EIF2\_ALPHA\_PHOSPHORYLATION |  | 9 | 0.60 | 1.39 | 0.112 | 0.559 | 1.000 | 997 | tags=33%, list=8%, signal=36% |
| 832 | GOBP\_NEGATIVE\_REGULATION\_OF\_CYTOKINE\_PRODUCTION |  | 164 | 0.33 | 1.39 | 0.021 | 0.559 | 1.000 | 1313 | tags=18%, list=11%, signal=20% |
| 833 | GOBP\_POSITIVE\_REGULATION\_OF\_VIRAL\_LIFE\_CYCLE |  | 21 | 0.49 | 1.39 | 0.092 | 0.559 | 1.000 | 1449 | tags=33%, list=12%, signal=38% |
| 834 | GOMF\_CYTOSKELETAL\_PROTEIN\_BINDING |  | 693 | 0.29 | 1.39 | 0.001 | 0.559 | 1.000 | 3033 | tags=33%, list=25%, signal=42% |
| 835 | GOMF\_SODIUM\_CHANNEL\_REGULATOR\_ACTIVITY |  | 21 | 0.48 | 1.39 | 0.103 | 0.559 | 1.000 | 1515 | tags=29%, list=13%, signal=33% |
| 836 | GOBP\_POLYOL\_TRANSPORT |  | 8 | 0.63 | 1.39 | 0.117 | 0.558 | 1.000 | 2673 | tags=75%, list=22%, signal=96% |
| 837 | GOBP\_POSITIVE\_REGULATION\_OF\_SODIUM\_ION\_TRANSMEMBRANE\_TRANSPORTER\_ACTIVITY |  | 12 | 0.56 | 1.39 | 0.110 | 0.559 | 1.000 | 2044 | tags=42%, list=17%, signal=50% |
| 838 | GOCC\_X\_CHROMOSOME |  | 5 | 0.71 | 1.39 | 0.111 | 0.560 | 1.000 | 199 | tags=20%, list=2%, signal=20% |
| 839 | GOBP\_HISTONE\_ARGININE\_METHYLATION |  | 4 | 0.76 | 1.39 | 0.106 | 0.560 | 1.000 | 1309 | tags=50%, list=11%, signal=56% |
| 840 | GOBP\_REGULATION\_OF\_EXTRINSIC\_APOPTOTIC\_SIGNALING\_PATHWAY |  | 107 | 0.35 | 1.39 | 0.031 | 0.560 | 1.000 | 2351 | tags=28%, list=20%, signal=35% |
| 841 | GOMF\_N\_ACETYLGALACTOSAMINE\_4\_O\_SULFOTRANSFERASE\_ACTIVITY |  | 3 | 0.83 | 1.39 | 0.085 | 0.560 | 1.000 | 1039 | tags=33%, list=9%, signal=36% |
| 842 | GOBP\_NEGATIVE\_REGULATION\_OF\_EXTRINSIC\_APOPTOTIC\_SIGNALING\_PATHWAY |  | 74 | 0.37 | 1.39 | 0.044 | 0.559 | 1.000 | 2351 | tags=27%, list=20%, signal=33% |
| 843 | GOBP\_NEGATIVE\_REGULATION\_OF\_SIGNAL\_TRANSDUCTION\_IN\_ABSENCE\_OF\_LIGAND |  | 19 | 0.50 | 1.38 | 0.094 | 0.560 | 1.000 | 2274 | tags=37%, list=19%, signal=45% |
| 844 | GOBP\_REGULATION\_OF\_NEURON\_PROJECTION\_ARBORIZATION |  | 13 | 0.55 | 1.38 | 0.110 | 0.559 | 1.000 | 1096 | tags=31%, list=9%, signal=34% |
| 845 | GOBP\_POSITIVE\_REGULATION\_OF\_TRIGLYCERIDE\_BIOSYNTHETIC\_PROCESS |  | 10 | 0.59 | 1.38 | 0.107 | 0.561 | 1.000 | 691 | tags=40%, list=6%, signal=42% |
| 846 | GOMF\_FATTY\_ACID\_LIGASE\_ACTIVITY |  | 13 | 0.55 | 1.38 | 0.107 | 0.561 | 1.000 | 1433 | tags=46%, list=12%, signal=52% |
| 847 | GOBP\_DEOXYRIBONUCLEOTIDE\_METABOLIC\_PROCESS |  | 30 | 0.44 | 1.38 | 0.085 | 0.561 | 1.000 | 1455 | tags=23%, list=12%, signal=26% |
| 848 | GOMF\_NUCLEOSIDE\_TRIPHOSPHATE\_DIPHOSPHATASE\_ACTIVITY |  | 6 | 0.68 | 1.38 | 0.124 | 0.560 | 1.000 | 3228 | tags=83%, list=27%, signal=114% |
| 849 | GOBP\_NEGATIVE\_REGULATION\_OF\_RECEPTOR\_SIGNALING\_PATHWAY\_VIA\_STAT |  | 21 | 0.48 | 1.38 | 0.099 | 0.560 | 1.000 | 686 | tags=24%, list=6%, signal=25% |
| 850 | GOBP\_PLATELET\_ACTIVATION |  | 111 | 0.35 | 1.38 | 0.032 | 0.561 | 1.000 | 2649 | tags=31%, list=22%, signal=39% |
| 851 | GOBP\_FATTY\_ACID\_DERIVATIVE\_BIOSYNTHETIC\_PROCESS |  | 42 | 0.41 | 1.38 | 0.072 | 0.560 | 1.000 | 1433 | tags=29%, list=12%, signal=32% |
| 852 | GOBP\_FATTY\_ACYL\_COA\_METABOLIC\_PROCESS |  | 32 | 0.44 | 1.38 | 0.070 | 0.560 | 1.000 | 1433 | tags=31%, list=12%, signal=35% |
| 853 | GOBP\_RESPONSE\_TO\_BIOTIC\_STIMULUS |  | 778 | 0.29 | 1.38 | 0.000 | 0.560 | 1.000 | 1397 | tags=16%, list=12%, signal=17% |
| 854 | GOBP\_PHOSPHATIDYLCHOLINE\_BIOSYNTHETIC\_PROCESS |  | 29 | 0.45 | 1.38 | 0.092 | 0.560 | 1.000 | 1917 | tags=45%, list=16%, signal=53% |
| 855 | GOBP\_IONOTROPIC\_GLUTAMATE\_RECEPTOR\_SIGNALING\_PATHWAY |  | 13 | 0.55 | 1.38 | 0.105 | 0.560 | 1.000 | 1546 | tags=31%, list=13%, signal=35% |
| 856 | GOBP\_GLUCOCORTICOID\_BIOSYNTHETIC\_PROCESS |  | 8 | 0.62 | 1.38 | 0.112 | 0.560 | 1.000 | 3109 | tags=75%, list=26%, signal=101% |
| 857 | GOBP\_REGULATION\_OF\_ASPARTIC\_TYPE\_ENDOPEPTIDASE\_ACTIVITY\_INVOLVED\_IN\_AMYLOID\_PRECURSOR\_PROTEIN\_CATABOLIC\_PROCESS |  | 7 | 0.65 | 1.38 | 0.121 | 0.560 | 1.000 | 1922 | tags=43%, list=16%, signal=51% |
| 858 | GOMF\_STRUCTURAL\_CONSTITUENT\_OF\_PRESYNAPTIC\_ACTIVE\_ZONE |  | 3 | 0.82 | 1.38 | 0.092 | 0.560 | 1.000 | 1649 | tags=67%, list=14%, signal=77% |
| 859 | GOBP\_CELLULAR\_RESPONSE\_TO\_ACIDIC\_PH |  | 9 | 0.60 | 1.38 | 0.117 | 0.559 | 1.000 | 463 | tags=33%, list=4%, signal=35% |
| 860 | GOBP\_NEGATIVE\_REGULATION\_OF\_ADENYLATE\_CYCLASE\_ACTIVITY |  | 8 | 0.63 | 1.38 | 0.120 | 0.558 | 1.000 | 960 | tags=38%, list=8%, signal=41% |
| 861 | GOMF\_CYCLIC\_NUCLEOTIDE\_DEPENDENT\_PROTEIN\_KINASE\_ACTIVITY |  | 7 | 0.65 | 1.38 | 0.120 | 0.558 | 1.000 | 2262 | tags=43%, list=19%, signal=53% |
| 862 | GOBP\_POSITIVE\_REGULATION\_OF\_ESTABLISHMENT\_OF\_PROTEIN\_LOCALIZATION |  | 233 | 0.32 | 1.38 | 0.011 | 0.558 | 1.000 | 2049 | tags=23%, list=17%, signal=27% |
| 863 | GOBP\_REGULATION\_OF\_SODIUM\_ION\_TRANSMEMBRANE\_TRANSPORTER\_ACTIVITY |  | 33 | 0.43 | 1.38 | 0.074 | 0.558 | 1.000 | 2640 | tags=45%, list=22%, signal=58% |
| 864 | GOBP\_NEGATIVE\_REGULATION\_OF\_ANION\_TRANSPORT |  | 130 | 0.34 | 1.38 | 0.033 | 0.558 | 1.000 | 1451 | tags=18%, list=12%, signal=21% |
| 865 | GOBP\_MEMBRANE\_RAFT\_ASSEMBLY |  | 8 | 0.63 | 1.38 | 0.120 | 0.558 | 1.000 | 1967 | tags=38%, list=16%, signal=45% |
| 866 | GOBP\_PURINE\_NUCLEOSIDE\_TRIPHOSPHATE\_CATABOLIC\_PROCESS |  | 5 | 0.71 | 1.38 | 0.104 | 0.558 | 1.000 | 585 | tags=40%, list=5%, signal=42% |
| 867 | GOBP\_VIRAL\_LATENCY |  | 10 | 0.59 | 1.38 | 0.112 | 0.558 | 1.000 | 1495 | tags=30%, list=12%, signal=34% |
| 868 | GOBP\_PORE\_COMPLEX\_ASSEMBLY |  | 18 | 0.50 | 1.38 | 0.097 | 0.560 | 1.000 | 1918 | tags=39%, list=16%, signal=46% |
| 869 | GOBP\_REGULATION\_OF\_SODIUM\_ION\_TRANSMEMBRANE\_TRANSPORT |  | 41 | 0.42 | 1.38 | 0.071 | 0.560 | 1.000 | 2640 | tags=41%, list=22%, signal=53% |
| 870 | GOMF\_CHOLESTEROL\_BINDING |  | 38 | 0.42 | 1.38 | 0.068 | 0.559 | 1.000 | 2129 | tags=39%, list=18%, signal=48% |
| 871 | GOBP\_FUCOSE\_CATABOLIC\_PROCESS |  | 6 | 0.69 | 1.38 | 0.115 | 0.559 | 1.000 | 680 | tags=33%, list=6%, signal=35% |
| 872 | GOBP\_ARF\_PROTEIN\_SIGNAL\_TRANSDUCTION |  | 14 | 0.54 | 1.38 | 0.109 | 0.559 | 1.000 | 1950 | tags=43%, list=16%, signal=51% |
| 873 | GOBP\_NEGATIVE\_REGULATION\_OF\_ENDOTHELIAL\_CELL\_DIFFERENTIATION |  | 6 | 0.67 | 1.38 | 0.117 | 0.558 | 1.000 | 3072 | tags=67%, list=26%, signal=90% |
| 874 | GOBP\_POSITIVE\_REGULATION\_OF\_PROTEIN\_CONTAINING\_COMPLEX\_ASSEMBLY |  | 171 | 0.32 | 1.38 | 0.017 | 0.559 | 1.000 | 2875 | tags=32%, list=24%, signal=42% |
| 875 | GOBP\_DEOXYRIBONUCLEOSIDE\_MONOPHOSPHATE\_METABOLIC\_PROCESS |  | 13 | 0.54 | 1.38 | 0.107 | 0.559 | 1.000 | 1455 | tags=31%, list=12%, signal=35% |
| 876 | GOBP\_POSITIVE\_REGULATION\_OF\_IRE1\_MEDIATED\_UNFOLDED\_PROTEIN\_RESPONSE |  | 6 | 0.67 | 1.38 | 0.118 | 0.559 | 1.000 | 108 | tags=17%, list=1%, signal=17% |
| 877 | GOBP\_PYRIMIDINE\_DEOXYRIBONUCLEOSIDE\_MONOPHOSPHATE\_METABOLIC\_PROCESS |  | 7 | 0.65 | 1.38 | 0.118 | 0.559 | 1.000 | 904 | tags=29%, list=8%, signal=31% |
| 878 | GOBP\_REGULATION\_OF\_BIOMINERALIZATION |  | 56 | 0.39 | 1.38 | 0.057 | 0.559 | 1.000 | 2604 | tags=32%, list=22%, signal=41% |
| 879 | GOBP\_CYTOKINESIS |  | 125 | 0.34 | 1.38 | 0.025 | 0.559 | 1.000 | 2783 | tags=37%, list=23%, signal=47% |
| 880 | GOCC\_PLATELET\_DENSE\_GRANULE\_LUMEN |  | 6 | 0.67 | 1.38 | 0.114 | 0.559 | 1.000 | 1393 | tags=50%, list=12%, signal=57% |
| 881 | GOBP\_REGULATION\_OF\_ACTIN\_FILAMENT\_BASED\_PROCESS |  | 281 | 0.31 | 1.38 | 0.008 | 0.560 | 1.000 | 2146 | tags=27%, list=18%, signal=32% |
| 882 | GOMF\_LONG\_CHAIN\_FATTY\_ACID\_COA\_LIGASE\_ACTIVITY |  | 11 | 0.58 | 1.38 | 0.105 | 0.560 | 1.000 | 1433 | tags=55%, list=12%, signal=62% |
| 883 | GOMF\_PROTEIN\_MEMBRANE\_ADAPTOR\_ACTIVITY |  | 17 | 0.51 | 1.38 | 0.104 | 0.560 | 1.000 | 3549 | tags=65%, list=30%, signal=92% |
| 884 | GOBP\_MITOTIC\_CYTOKINETIC\_PROCESS |  | 20 | 0.49 | 1.38 | 0.093 | 0.560 | 1.000 | 3691 | tags=55%, list=31%, signal=79% |
| 885 | GOBP\_POSITIVE\_REGULATION\_OF\_ASPARTIC\_TYPE\_PEPTIDASE\_ACTIVITY |  | 5 | 0.71 | 1.38 | 0.118 | 0.560 | 1.000 | 1922 | tags=40%, list=16%, signal=48% |
| 886 | GOMF\_LYSOPHOSPHOLIPASE\_ACTIVITY |  | 19 | 0.49 | 1.38 | 0.100 | 0.559 | 1.000 | 1752 | tags=37%, list=15%, signal=43% |
| 887 | GOBP\_RESPONSE\_TO\_INSULIN |  | 203 | 0.32 | 1.38 | 0.016 | 0.559 | 1.000 | 2810 | tags=33%, list=23%, signal=42% |
| 888 | GOBP\_DNA\_REPLICATION\_INDEPENDENT\_NUCLEOSOME\_ORGANIZATION |  | 36 | 0.43 | 1.38 | 0.075 | 0.560 | 1.000 | 3970 | tags=61%, list=33%, signal=91% |
| 889 | GOBP\_DEFENSE\_RESPONSE |  | 843 | 0.28 | 1.38 | 0.000 | 0.559 | 1.000 | 1535 | tags=17%, list=13%, signal=18% |
| 890 | GOBP\_PHOSPHATIDYLETHANOLAMINE\_METABOLIC\_PROCESS |  | 19 | 0.49 | 1.38 | 0.100 | 0.560 | 1.000 | 2085 | tags=53%, list=17%, signal=64% |
| 891 | GOBP\_RESPONSE\_TO\_DRUG |  | 250 | 0.31 | 1.38 | 0.010 | 0.559 | 1.000 | 1469 | tags=20%, list=12%, signal=23% |
| 892 | GOCC\_CD95\_DEATH\_INDUCING\_SIGNALING\_COMPLEX |  | 5 | 0.71 | 1.38 | 0.119 | 0.559 | 1.000 | 3470 | tags=100%, list=29%, signal=141% |
| 893 | GOBP\_REGULATION\_OF\_PROTEIN\_CATABOLIC\_PROCESS\_IN\_THE\_VACUOLE |  | 9 | 0.60 | 1.38 | 0.117 | 0.559 | 1.000 | 2049 | tags=44%, list=17%, signal=54% |
| 894 | GOBP\_POSTTRANSLATIONAL\_PROTEIN\_TARGETING\_TO\_MEMBRANE\_TRANSLOCATION |  | 7 | 0.64 | 1.38 | 0.129 | 0.560 | 1.000 | 2308 | tags=57%, list=19%, signal=71% |
| 895 | GOBP\_EPHRIN\_RECEPTOR\_SIGNALING\_PATHWAY |  | 69 | 0.37 | 1.37 | 0.056 | 0.564 | 1.000 | 3387 | tags=51%, list=28%, signal=70% |
| 896 | GOMF\_COA\_HYDROLASE\_ACTIVITY |  | 16 | 0.52 | 1.37 | 0.104 | 0.564 | 1.000 | 911 | tags=31%, list=8%, signal=34% |
| 897 | GOBP\_PHOSPHOLIPID\_CATABOLIC\_PROCESS |  | 35 | 0.43 | 1.37 | 0.074 | 0.564 | 1.000 | 1752 | tags=37%, list=15%, signal=43% |
| 898 | GOMF\_ATPASE\_ACTIVITY |  | 336 | 0.30 | 1.37 | 0.006 | 0.563 | 1.000 | 2225 | tags=24%, list=19%, signal=28% |
| 899 | GOBP\_REGULATION\_OF\_SMALL\_GTPASE\_MEDIATED\_SIGNAL\_TRANSDUCTION |  | 251 | 0.31 | 1.37 | 0.011 | 0.563 | 1.000 | 2731 | tags=32%, list=23%, signal=41% |
| 900 | GOCC\_CELL\_CELL\_CONTACT\_ZONE |  | 47 | 0.40 | 1.37 | 0.068 | 0.564 | 1.000 | 1875 | tags=32%, list=16%, signal=38% |
| 901 | GOBP\_HOMOLOGOUS\_CHROMOSOME\_PAIRING\_AT\_MEIOSIS |  | 21 | 0.48 | 1.37 | 0.100 | 0.564 | 1.000 | 3684 | tags=52%, list=31%, signal=75% |
| 902 | GOBP\_RESPONSE\_TO\_INCREASED\_OXYGEN\_LEVELS |  | 22 | 0.47 | 1.37 | 0.101 | 0.565 | 1.000 | 2221 | tags=32%, list=18%, signal=39% |
| 903 | GOCC\_CLATHRIN\_COATED\_ENDOCYTIC\_VESICLE |  | 27 | 0.45 | 1.37 | 0.103 | 0.565 | 1.000 | 1880 | tags=26%, list=16%, signal=31% |
| 904 | GOMF\_PROTEIN\_BINDING\_INVOLVED\_IN\_HETEROTYPIC\_CELL\_CELL\_ADHESION |  | 7 | 0.64 | 1.37 | 0.119 | 0.566 | 1.000 | 985 | tags=43%, list=8%, signal=47% |
| 905 | GOBP\_NEGATIVE\_REGULATION\_OF\_BLOOD\_VESSEL\_ENDOTHELIAL\_CELL\_MIGRATION |  | 29 | 0.44 | 1.37 | 0.085 | 0.567 | 1.000 | 1553 | tags=28%, list=13%, signal=32% |
| 906 | GOBP\_REGULATION\_OF\_ACTIN\_FILAMENT\_BASED\_MOVEMENT |  | 23 | 0.47 | 1.37 | 0.101 | 0.567 | 1.000 | 1234 | tags=30%, list=10%, signal=34% |
| 907 | GOBP\_DNA\_REPLICATION\_CHECKPOINT |  | 15 | 0.52 | 1.37 | 0.114 | 0.568 | 1.000 | 4413 | tags=73%, list=37%, signal=116% |
| 908 | GOBP\_POSITIVE\_REGULATION\_OF\_LIPID\_LOCALIZATION |  | 72 | 0.37 | 1.37 | 0.054 | 0.568 | 1.000 | 1326 | tags=24%, list=11%, signal=26% |
| 909 | GOBP\_REGULATION\_OF\_EARLY\_ENDOSOME\_TO\_LATE\_ENDOSOME\_TRANSPORT |  | 15 | 0.52 | 1.37 | 0.109 | 0.569 | 1.000 | 4749 | tags=73%, list=40%, signal=121% |
| 910 | GOBP\_INOSITOL\_TRISPHOSPHATE\_METABOLIC\_PROCESS |  | 8 | 0.62 | 1.37 | 0.121 | 0.570 | 1.000 | 713 | tags=38%, list=6%, signal=40% |
| 911 | GOBP\_POSITIVE\_REGULATION\_OF\_T\_HELPER\_2\_CELL\_CYTOKINE\_PRODUCTION |  | 6 | 0.68 | 1.37 | 0.130 | 0.572 | 1.000 | 405 | tags=33%, list=3%, signal=34% |
| 912 | GOBP\_REGULATION\_OF\_RECEPTOR\_SIGNALING\_PATHWAY\_VIA\_STAT |  | 53 | 0.39 | 1.37 | 0.056 | 0.571 | 1.000 | 990 | tags=21%, list=8%, signal=23% |
| 913 | GOMF\_FILAMIN\_BINDING |  | 8 | 0.62 | 1.37 | 0.132 | 0.572 | 1.000 | 1383 | tags=38%, list=12%, signal=42% |
| 914 | GOBP\_MAMMARY\_GLAND\_MORPHOGENESIS |  | 30 | 0.44 | 1.37 | 0.092 | 0.573 | 1.000 | 2548 | tags=40%, list=21%, signal=51% |
| 915 | GOBP\_CARDIAC\_CONDUCTION\_SYSTEM\_DEVELOPMENT |  | 8 | 0.62 | 1.37 | 0.131 | 0.572 | 1.000 | 1336 | tags=50%, list=11%, signal=56% |
| 916 | GOBP\_REGULATION\_OF\_DEFENSE\_RESPONSE |  | 389 | 0.30 | 1.37 | 0.006 | 0.572 | 1.000 | 1519 | tags=17%, list=13%, signal=19% |
| 917 | GOMF\_ACYLGLYCEROL\_LIPASE\_ACTIVITY |  | 9 | 0.60 | 1.37 | 0.112 | 0.571 | 1.000 | 1752 | tags=56%, list=15%, signal=65% |
| 918 | GOBP\_INTERLEUKIN\_23\_PRODUCTION |  | 4 | 0.75 | 1.37 | 0.101 | 0.571 | 1.000 | 1737 | tags=50%, list=14%, signal=58% |
| 919 | GOBP\_ECTODERMAL\_PLACODE\_FORMATION |  | 7 | 0.64 | 1.37 | 0.126 | 0.571 | 1.000 | 3 | tags=14%, list=0%, signal=14% |
| 920 | GOBP\_REGULATION\_OF\_DNA\_DAMAGE\_CHECKPOINT |  | 12 | 0.55 | 1.37 | 0.119 | 0.570 | 1.000 | 4522 | tags=67%, list=38%, signal=107% |
| 921 | GOBP\_CELLULAR\_RESPONSE\_TO\_EXTERNAL\_STIMULUS |  | 230 | 0.31 | 1.37 | 0.017 | 0.570 | 1.000 | 1427 | tags=18%, list=12%, signal=20% |
| 922 | GOCC\_SEPTIN\_COMPLEX |  | 3 | 0.81 | 1.37 | 0.103 | 0.570 | 1.000 | 2123 | tags=67%, list=18%, signal=81% |
| 923 | GOMF\_RECEPTOR\_INHIBITOR\_ACTIVITY |  | 12 | 0.55 | 1.37 | 0.123 | 0.570 | 1.000 | 1607 | tags=42%, list=13%, signal=48% |
| 924 | GOBP\_NEGATIVE\_REGULATION\_OF\_VIRAL\_INDUCED\_CYTOPLASMIC\_PATTERN\_RECOGNITION\_RECEPTOR\_SIGNALING\_PATHWAY |  | 9 | 0.59 | 1.37 | 0.126 | 0.570 | 1.000 | 1137 | tags=33%, list=9%, signal=37% |
| 925 | GOMF\_PROTEIN\_KINASE\_ACTIVITY |  | 416 | 0.29 | 1.37 | 0.003 | 0.570 | 1.000 | 2854 | tags=30%, list=24%, signal=38% |
| 926 | GOBP\_EXOCYST\_LOCALIZATION |  | 5 | 0.70 | 1.37 | 0.124 | 0.571 | 1.000 | 2583 | tags=60%, list=22%, signal=76% |
| 927 | GOBP\_SIGNAL\_RELEASE |  | 312 | 0.30 | 1.37 | 0.010 | 0.573 | 1.000 | 2049 | tags=26%, list=17%, signal=31% |
| 928 | GOBP\_GLUCOSYLCERAMIDE\_METABOLIC\_PROCESS |  | 5 | 0.71 | 1.37 | 0.126 | 0.573 | 1.000 | 1419 | tags=60%, list=12%, signal=68% |
| 929 | GOBP\_MEIOTIC\_CELL\_CYCLE |  | 144 | 0.33 | 1.37 | 0.024 | 0.573 | 1.000 | 3960 | tags=45%, list=33%, signal=67% |
| 930 | GOBP\_NEGATIVE\_REGULATION\_OF\_PROTEIN\_LOCALIZATION\_TO\_CELL\_PERIPHERY |  | 19 | 0.49 | 1.37 | 0.109 | 0.573 | 1.000 | 2561 | tags=32%, list=21%, signal=40% |
| 931 | GOBP\_REGULATION\_OF\_PROTEIN\_CONTAINING\_COMPLEX\_ASSEMBLY |  | 309 | 0.30 | 1.37 | 0.008 | 0.572 | 1.000 | 2875 | tags=30%, list=24%, signal=38% |
| 932 | GOMF\_CARGO\_ADAPTOR\_ACTIVITY |  | 12 | 0.55 | 1.36 | 0.121 | 0.572 | 1.000 | 4558 | tags=58%, list=38%, signal=94% |
| 933 | GOBP\_POSITIVE\_REGULATION\_OF\_CELL\_SUBSTRATE\_ADHESION |  | 92 | 0.36 | 1.36 | 0.050 | 0.571 | 1.000 | 2843 | tags=36%, list=24%, signal=47% |
| 934 | GOBP\_CELLULAR\_RESPONSE\_TO\_HYDROGEN\_PEROXIDE |  | 68 | 0.37 | 1.36 | 0.057 | 0.572 | 1.000 | 1612 | tags=28%, list=13%, signal=32% |
| 935 | GOBP\_RIBONUCLEOTIDE\_CATABOLIC\_PROCESS |  | 29 | 0.44 | 1.36 | 0.093 | 0.572 | 1.000 | 1700 | tags=24%, list=14%, signal=28% |
| 936 | GOCC\_SEX\_CHROMOSOME |  | 18 | 0.49 | 1.36 | 0.112 | 0.571 | 1.000 | 2657 | tags=39%, list=22%, signal=50% |
| 937 | GOBP\_ACTIN\_NUCLEATION |  | 36 | 0.42 | 1.36 | 0.079 | 0.571 | 1.000 | 3900 | tags=50%, list=32%, signal=74% |
| 938 | GOBP\_MIDBODY\_ABSCISSION |  | 16 | 0.51 | 1.36 | 0.114 | 0.572 | 1.000 | 3691 | tags=63%, list=31%, signal=90% |
| 939 | GOCC\_CONDENSED\_CHROMOSOME\_CENTROMERIC\_REGION |  | 97 | 0.35 | 1.36 | 0.045 | 0.572 | 1.000 | 3970 | tags=54%, list=33%, signal=79% |
| 940 | GOBP\_POSITIVE\_REGULATION\_OF\_RESPONSE\_TO\_REACTIVE\_OXYGEN\_SPECIES |  | 3 | 0.81 | 1.36 | 0.097 | 0.572 | 1.000 | 1092 | tags=67%, list=9%, signal=73% |
| 941 | GOMF\_DNA\_BINDING\_TRANSCRIPTION\_REPRESSOR\_ACTIVITY |  | 154 | 0.33 | 1.36 | 0.026 | 0.571 | 1.000 | 1339 | tags=19%, list=11%, signal=21% |
| 942 | GOBP\_REGULATION\_OF\_APPETITE |  | 12 | 0.55 | 1.36 | 0.112 | 0.571 | 1.000 | 389 | tags=33%, list=3%, signal=34% |
| 943 | GOBP\_NEGATIVE\_REGULATION\_OF\_ION\_TRANSPORT |  | 192 | 0.32 | 1.36 | 0.023 | 0.570 | 1.000 | 2821 | tags=32%, list=23%, signal=42% |
| 944 | GOBP\_NEGATIVE\_REGULATION\_OF\_EPITHELIAL\_CELL\_MIGRATION |  | 58 | 0.38 | 1.36 | 0.064 | 0.570 | 1.000 | 1553 | tags=19%, list=13%, signal=22% |
| 945 | GOBP\_CELLULAR\_SODIUM\_ION\_HOMEOSTASIS |  | 10 | 0.58 | 1.36 | 0.115 | 0.570 | 1.000 | 739 | tags=30%, list=6%, signal=32% |
| 946 | GOBP\_POSITIVE\_REGULATION\_OF\_TUMOR\_NECROSIS\_FACTOR\_SUPERFAMILY\_CYTOKINE\_PRODUCTION |  | 50 | 0.39 | 1.36 | 0.079 | 0.570 | 1.000 | 3100 | tags=38%, list=26%, signal=51% |
| 947 | GOBP\_REGULATION\_OF\_INFLAMMATORY\_RESPONSE\_TO\_WOUNDING |  | 4 | 0.75 | 1.36 | 0.113 | 0.570 | 1.000 | 867 | tags=25%, list=7%, signal=27% |
| 948 | GOBP\_REGULATION\_OF\_PLATELET\_ACTIVATION |  | 23 | 0.47 | 1.36 | 0.105 | 0.570 | 1.000 | 1444 | tags=26%, list=12%, signal=30% |
| 949 | GOBP\_ACTIVATION\_INDUCED\_CELL\_DEATH\_OF\_T\_CELLS |  | 8 | 0.62 | 1.36 | 0.125 | 0.570 | 1.000 | 409 | tags=25%, list=3%, signal=26% |
| 950 | GOBP\_CELLULAR\_RESPONSE\_TO\_FLUID\_SHEAR\_STRESS |  | 15 | 0.52 | 1.36 | 0.120 | 0.569 | 1.000 | 713 | tags=27%, list=6%, signal=28% |
| 951 | GOCC\_APICAL\_DENDRITE |  | 15 | 0.52 | 1.36 | 0.116 | 0.570 | 1.000 | 2013 | tags=47%, list=17%, signal=56% |
| 952 | GOBP\_GLYCEROPHOSPHOLIPID\_CATABOLIC\_PROCESS |  | 22 | 0.47 | 1.36 | 0.108 | 0.570 | 1.000 | 1752 | tags=41%, list=15%, signal=48% |
| 953 | GOMF\_GALACTOSYLTRANSFERASE\_ACTIVITY |  | 15 | 0.52 | 1.36 | 0.113 | 0.569 | 1.000 | 110 | tags=13%, list=1%, signal=13% |
| 954 | GOCC\_EXOCYST |  | 15 | 0.52 | 1.36 | 0.120 | 0.570 | 1.000 | 3169 | tags=47%, list=26%, signal=63% |
| 955 | GOMF\_PEPTIDASE\_REGULATOR\_ACTIVITY |  | 104 | 0.35 | 1.36 | 0.042 | 0.570 | 1.000 | 1219 | tags=20%, list=10%, signal=22% |
| 956 | GOBP\_ENDOMEMBRANE\_SYSTEM\_ORGANIZATION |  | 344 | 0.30 | 1.36 | 0.006 | 0.570 | 1.000 | 3140 | tags=35%, list=26%, signal=45% |
| 957 | GOCC\_CONDENSED\_CHROMOSOME |  | 160 | 0.32 | 1.36 | 0.029 | 0.571 | 1.000 | 3978 | tags=47%, list=33%, signal=69% |
| 958 | GOBP\_NEGATIVE\_REGULATION\_OF\_SUPRAMOLECULAR\_FIBER\_ORGANIZATION |  | 104 | 0.35 | 1.36 | 0.042 | 0.572 | 1.000 | 2090 | tags=26%, list=17%, signal=31% |
| 959 | GOBP\_HEPATICOBILIARY\_SYSTEM\_DEVELOPMENT |  | 106 | 0.34 | 1.36 | 0.042 | 0.572 | 1.000 | 1864 | tags=23%, list=16%, signal=27% |
| 960 | GOCC\_CLEAVAGE\_FURROW |  | 41 | 0.41 | 1.36 | 0.075 | 0.572 | 1.000 | 3111 | tags=46%, list=26%, signal=62% |
| 961 | GOBP\_REGULATION\_OF\_MITOTIC\_CYTOKINESIS |  | 3 | 0.81 | 1.36 | 0.105 | 0.573 | 1.000 | 1532 | tags=67%, list=13%, signal=76% |
| 962 | GOBP\_PROTEIN\_LIPID\_COMPLEX\_SUBUNIT\_ORGANIZATION |  | 29 | 0.44 | 1.36 | 0.101 | 0.573 | 1.000 | 1396 | tags=31%, list=12%, signal=35% |
| 963 | GOBP\_ACTIN\_FILAMENT\_SEVERING |  | 13 | 0.54 | 1.36 | 0.122 | 0.574 | 1.000 | 1505 | tags=38%, list=13%, signal=44% |
| 964 | GOBP\_REGULATION\_OF\_WNT\_SIGNALING\_PATHWAY\_INVOLVED\_IN\_HEART\_DEVELOPMENT |  | 4 | 0.75 | 1.36 | 0.119 | 0.575 | 1.000 | 3037 | tags=100%, list=25%, signal=134% |
| 965 | GOCC\_DENDRITIC\_GROWTH\_CONE |  | 8 | 0.61 | 1.36 | 0.134 | 0.575 | 1.000 | 903 | tags=25%, list=8%, signal=27% |
| 966 | GOBP\_REGULATION\_OF\_ACTIN\_FILAMENT\_LENGTH |  | 130 | 0.33 | 1.36 | 0.035 | 0.575 | 1.000 | 2146 | tags=28%, list=18%, signal=33% |
| 967 | GOBP\_NEGATIVE\_REGULATION\_OF\_IMMUNE\_RESPONSE |  | 77 | 0.36 | 1.36 | 0.059 | 0.575 | 1.000 | 1060 | tags=17%, list=9%, signal=18% |
| 968 | GOBP\_EPITHELIAL\_CELL\_DIFFERENTIATION\_INVOLVED\_IN\_KIDNEY\_DEVELOPMENT |  | 34 | 0.42 | 1.36 | 0.093 | 0.578 | 1.000 | 2680 | tags=41%, list=22%, signal=53% |
| 969 | GOBP\_POSITIVE\_REGULATION\_OF\_RESPONSE\_TO\_TUMOR\_CELL |  | 5 | 0.71 | 1.36 | 0.130 | 0.578 | 1.000 | 2666 | tags=60%, list=22%, signal=77% |
| 970 | GOBP\_SECRETION |  | 948 | 0.28 | 1.36 | 0.000 | 0.578 | 1.000 | 2049 | tags=23%, list=17%, signal=25% |
| 971 | GOBP\_ENDOCRINE\_HORMONE\_SECRETION |  | 27 | 0.45 | 1.36 | 0.101 | 0.579 | 1.000 | 1274 | tags=30%, list=11%, signal=33% |
| 972 | GOBP\_ANTEROGRADE\_AXONAL\_TRANSPORT\_OF\_MITOCHONDRION |  | 5 | 0.70 | 1.36 | 0.127 | 0.579 | 1.000 | 2634 | tags=60%, list=22%, signal=77% |
| 973 | GOCC\_BRUSH\_BORDER |  | 72 | 0.37 | 1.35 | 0.062 | 0.582 | 1.000 | 1986 | tags=35%, list=17%, signal=41% |
| 974 | GOBP\_ESTABLISHMENT\_OR\_MAINTENANCE\_OF\_BIPOLAR\_CELL\_POLARITY |  | 33 | 0.43 | 1.35 | 0.091 | 0.581 | 1.000 | 3045 | tags=48%, list=25%, signal=65% |
| 975 | GOBP\_POSITIVE\_REGULATION\_OF\_RESPONSE\_TO\_DNA\_DAMAGE\_STIMULUS |  | 75 | 0.36 | 1.35 | 0.056 | 0.582 | 1.000 | 3609 | tags=43%, list=30%, signal=61% |
| 976 | GOMF\_ATPASE\_BINDING |  | 62 | 0.37 | 1.35 | 0.057 | 0.582 | 1.000 | 2903 | tags=34%, list=24%, signal=44% |
| 977 | GOMF\_BMP\_RECEPTOR\_ACTIVITY |  | 5 | 0.69 | 1.35 | 0.132 | 0.581 | 1.000 | 1909 | tags=60%, list=16%, signal=71% |
| 978 | GOBP\_CYTOKINETIC\_PROCESS |  | 34 | 0.43 | 1.35 | 0.095 | 0.582 | 1.000 | 3691 | tags=50%, list=31%, signal=72% |
| 979 | GOBP\_NEGATIVE\_REGULATION\_OF\_INTERFERON\_ALPHA\_PRODUCTION |  | 3 | 0.80 | 1.35 | 0.113 | 0.582 | 1.000 | 66 | tags=33%, list=1%, signal=34% |
| 980 | GOBP\_GANGLIOSIDE\_CATABOLIC\_PROCESS |  | 4 | 0.75 | 1.35 | 0.123 | 0.582 | 1.000 | 130 | tags=25%, list=1%, signal=25% |
| 981 | GOBP\_LIMBIC\_SYSTEM\_DEVELOPMENT |  | 58 | 0.38 | 1.35 | 0.079 | 0.581 | 1.000 | 2035 | tags=26%, list=17%, signal=31% |
| 982 | GOBP\_POSITIVE\_REGULATION\_OF\_LAMELLIPODIUM\_MORPHOGENESIS |  | 6 | 0.66 | 1.35 | 0.132 | 0.582 | 1.000 | 1722 | tags=50%, list=14%, signal=58% |
| 983 | GOMF\_SODIUM\_INDEPENDENT\_ORGANIC\_ANION\_TRANSMEMBRANE\_TRANSPORTER\_ACTIVITY |  | 5 | 0.69 | 1.35 | 0.132 | 0.581 | 1.000 | 3304 | tags=60%, list=28%, signal=83% |
| 984 | GOBP\_REGULATION\_OF\_MYOBLAST\_DIFFERENTIATION |  | 37 | 0.42 | 1.35 | 0.084 | 0.581 | 1.000 | 1395 | tags=22%, list=12%, signal=24% |
| 985 | GOBP\_INTERMEDIATE\_FILAMENT\_BUNDLE\_ASSEMBLY |  | 3 | 0.81 | 1.35 | 0.108 | 0.580 | 1.000 | 7 | tags=33%, list=0%, signal=33% |
| 986 | GOCC\_SYNAPTONEMAL\_STRUCTURE |  | 19 | 0.48 | 1.35 | 0.120 | 0.580 | 1.000 | 3507 | tags=47%, list=29%, signal=67% |
| 987 | GOBP\_REVERSIBLE\_DIFFERENTIATION |  | 5 | 0.70 | 1.35 | 0.133 | 0.580 | 1.000 | 575 | tags=20%, list=5%, signal=21% |
| 988 | GOBP\_CELLULAR\_PROTEIN\_CONTAINING\_COMPLEX\_LOCALIZATION |  | 17 | 0.50 | 1.35 | 0.118 | 0.580 | 1.000 | 1670 | tags=35%, list=14%, signal=41% |
| 989 | GOBP\_NEGATIVE\_REGULATION\_OF\_OSTEOCLAST\_DIFFERENTIATION |  | 14 | 0.52 | 1.35 | 0.116 | 0.580 | 1.000 | 2141 | tags=43%, list=18%, signal=52% |
| 990 | GOBP\_FORMATION\_OF\_EXTRACHROMOSOMAL\_CIRCULAR\_DNA |  | 12 | 0.54 | 1.35 | 0.134 | 0.580 | 1.000 | 983 | tags=33%, list=8%, signal=36% |
| 991 | GOMF\_1\_PHOSPHATIDYLINOSITOL\_BINDING |  | 14 | 0.52 | 1.35 | 0.120 | 0.580 | 1.000 | 654 | tags=21%, list=5%, signal=23% |
| 992 | GOBP\_POSITIVE\_REGULATION\_OF\_SODIUM\_ION\_TRANSPORT |  | 26 | 0.45 | 1.35 | 0.106 | 0.585 | 1.000 | 725 | tags=15%, list=6%, signal=16% |
| 993 | GOBP\_CARBOHYDRATE\_IMPORT\_ACROSS\_PLASMA\_MEMBRANE |  | 4 | 0.76 | 1.35 | 0.113 | 0.588 | 1.000 | 1360 | tags=75%, list=11%, signal=85% |
| 994 | GOBP\_CELLULAR\_RESPONSE\_TO\_FOLLICLE\_STIMULATING\_HORMONE\_STIMULUS |  | 5 | 0.70 | 1.35 | 0.135 | 0.588 | 1.000 | 855 | tags=40%, list=7%, signal=43% |
| 995 | GOCC\_CORTICAL\_ACTIN\_CYTOSKELETON |  | 58 | 0.38 | 1.35 | 0.067 | 0.588 | 1.000 | 2397 | tags=33%, list=20%, signal=41% |
| 996 | GOBP\_RIGHTING\_REFLEX |  | 4 | 0.74 | 1.35 | 0.126 | 0.587 | 1.000 | 831 | tags=50%, list=7%, signal=54% |
| 997 | GOMF\_WW\_DOMAIN\_BINDING |  | 16 | 0.50 | 1.35 | 0.125 | 0.588 | 1.000 | 3207 | tags=44%, list=27%, signal=60% |
| 998 | GOBP\_POSITIVE\_REGULATION\_OF\_DNA\_DEPENDENT\_DNA\_REPLICATION |  | 9 | 0.59 | 1.35 | 0.133 | 0.589 | 1.000 | 2394 | tags=44%, list=20%, signal=55% |
| 999 | GOBP\_REGULATION\_OF\_CELL\_CYCLE\_CHECKPOINT |  | 27 | 0.44 | 1.35 | 0.103 | 0.589 | 1.000 | 4038 | tags=56%, list=34%, signal=84% |
| 1000 | GOBP\_ESTABLISHMENT\_OR\_MAINTENANCE\_OF\_CELL\_POLARITY |  | 173 | 0.32 | 1.35 | 0.029 | 0.589 | 1.000 | 3045 | tags=39%, list=25%, signal=52% |
| 1001 | GOBP\_MEIOSIS\_I\_CELL\_CYCLE\_PROCESS |  | 70 | 0.37 | 1.35 | 0.059 | 0.591 | 1.000 | 3826 | tags=46%, list=32%, signal=67% |
| 1002 | GOBP\_REGULATION\_OF\_CALCIUM\_ION\_IMPORT |  | 17 | 0.50 | 1.35 | 0.131 | 0.591 | 1.000 | 1710 | tags=29%, list=14%, signal=34% |
| 1003 | GOBP\_HYPEROSMOTIC\_RESPONSE |  | 18 | 0.49 | 1.35 | 0.114 | 0.591 | 1.000 | 942 | tags=28%, list=8%, signal=30% |
| 1004 | GOBP\_ENDOPLASMIC\_RETICULUM\_TO\_GOLGI\_VESICLE\_MEDIATED\_TRANSPORT |  | 163 | 0.32 | 1.35 | 0.028 | 0.592 | 1.000 | 3458 | tags=39%, list=29%, signal=54% |
| 1005 | GOBP\_PROTEIN\_DE\_ADP\_RIBOSYLATION |  | 3 | 0.80 | 1.35 | 0.122 | 0.593 | 1.000 | 440 | tags=33%, list=4%, signal=35% |
| 1006 | GOBP\_NEGATIVE\_REGULATION\_OF\_CELL\_CYCLE\_CHECKPOINT |  | 5 | 0.70 | 1.35 | 0.148 | 0.592 | 1.000 | 2065 | tags=60%, list=17%, signal=72% |
| 1007 | GOBP\_NEGATIVE\_REGULATION\_OF\_CYTOSKELETON\_ORGANIZATION |  | 105 | 0.34 | 1.35 | 0.048 | 0.592 | 1.000 | 2105 | tags=26%, list=18%, signal=31% |
| 1008 | GOBP\_REGULATION\_OF\_PEPTIDYL\_TYROSINE\_PHOSPHORYLATION |  | 166 | 0.32 | 1.35 | 0.026 | 0.594 | 1.000 | 2311 | tags=28%, list=19%, signal=34% |
| 1009 | GOMF\_PHOSPHOLIPASE\_INHIBITOR\_ACTIVITY |  | 6 | 0.66 | 1.34 | 0.138 | 0.594 | 1.000 | 1858 | tags=67%, list=15%, signal=79% |
| 1010 | GOCC\_EXTRINSIC\_COMPONENT\_OF\_ENDOSOME\_MEMBRANE |  | 9 | 0.59 | 1.34 | 0.135 | 0.595 | 1.000 | 3069 | tags=56%, list=26%, signal=75% |
| 1011 | GOBP\_REGULATION\_OF\_SYNAPSE\_STRUCTURAL\_PLASTICITY |  | 6 | 0.66 | 1.34 | 0.137 | 0.595 | 1.000 | 2645 | tags=67%, list=22%, signal=85% |
| 1012 | GOBP\_PHOSPHOLIPID\_METABOLIC\_PROCESS |  | 312 | 0.30 | 1.34 | 0.013 | 0.594 | 1.000 | 1926 | tags=25%, list=16%, signal=29% |
| 1013 | GOBP\_POSITIVE\_REGULATION\_OF\_HISTONE\_H3\_K4\_METHYLATION |  | 15 | 0.51 | 1.34 | 0.130 | 0.595 | 1.000 | 228 | tags=13%, list=2%, signal=14% |
| 1014 | GOBP\_REGULATION\_OF\_LYMPHANGIOGENESIS |  | 4 | 0.74 | 1.34 | 0.134 | 0.596 | 1.000 | 2548 | tags=75%, list=21%, signal=95% |
| 1015 | GOBP\_TOLERANCE\_INDUCTION\_TO\_SELF\_ANTIGEN |  | 4 | 0.75 | 1.34 | 0.131 | 0.597 | 1.000 | 623 | tags=25%, list=5%, signal=26% |
| 1016 | GOBP\_RESPONSE\_TO\_STEROL |  | 23 | 0.46 | 1.34 | 0.115 | 0.596 | 1.000 | 855 | tags=17%, list=7%, signal=19% |
| 1017 | GOBP\_PLATELET\_AGGREGATION |  | 42 | 0.40 | 1.34 | 0.093 | 0.596 | 1.000 | 2649 | tags=33%, list=22%, signal=43% |
| 1018 | GOBP\_MANGANESE\_ION\_TRANSPORT |  | 14 | 0.52 | 1.34 | 0.132 | 0.596 | 1.000 | 1145 | tags=29%, list=10%, signal=32% |
| 1019 | GOBP\_RESPONSE\_TO\_CYTOKINE |  | 735 | 0.28 | 1.34 | 0.001 | 0.596 | 1.000 | 2513 | tags=26%, list=21%, signal=31% |
| 1020 | GOBP\_POSITIVE\_REGULATION\_OF\_CYTOPLASMIC\_TRANSPORT |  | 9 | 0.58 | 1.34 | 0.144 | 0.596 | 1.000 | 800 | tags=22%, list=7%, signal=24% |
| 1021 | GOBP\_LIPID\_METABOLIC\_PROCESS |  | 968 | 0.27 | 1.34 | 0.000 | 0.596 | 1.000 | 1980 | tags=23%, list=16%, signal=25% |
| 1022 | GOBP\_MITOTIC\_CELL\_CYCLE |  | 783 | 0.28 | 1.34 | 0.002 | 0.596 | 1.000 | 3708 | tags=38%, list=31%, signal=51% |
| 1023 | GOBP\_REGULATION\_OF\_RESPONSE\_TO\_OSMOTIC\_STRESS |  | 6 | 0.66 | 1.34 | 0.149 | 0.595 | 1.000 | 869 | tags=33%, list=7%, signal=36% |
| 1024 | GOBP\_PLATELET\_MORPHOGENESIS |  | 17 | 0.49 | 1.34 | 0.124 | 0.596 | 1.000 | 1378 | tags=29%, list=11%, signal=33% |
| 1025 | GOBP\_POSITIVE\_REGULATION\_OF\_T\_CELL\_CYTOKINE\_PRODUCTION |  | 15 | 0.51 | 1.34 | 0.136 | 0.596 | 1.000 | 1198 | tags=27%, list=10%, signal=30% |
| 1026 | GOBP\_CELLULAR\_RESPONSE\_TO\_EXTRACELLULAR\_STIMULUS |  | 178 | 0.32 | 1.34 | 0.025 | 0.595 | 1.000 | 1427 | tags=19%, list=12%, signal=21% |
| 1027 | GOCC\_FEMALE\_GERM\_CELL\_NUCLEUS |  | 3 | 0.80 | 1.34 | 0.118 | 0.595 | 1.000 | 1194 | tags=67%, list=10%, signal=74% |
| 1028 | GOBP\_PLASMA\_MEMBRANE\_TO\_ENDOSOME\_TRANSPORT |  | 8 | 0.60 | 1.34 | 0.146 | 0.595 | 1.000 | 3111 | tags=50%, list=26%, signal=67% |
| 1029 | GOBP\_REGULATION\_OF\_BODY\_FLUID\_LEVELS |  | 318 | 0.30 | 1.34 | 0.010 | 0.594 | 1.000 | 2846 | tags=34%, list=24%, signal=43% |
| 1030 | GOBP\_REGULATION\_OF\_VENTRICULAR\_CARDIAC\_MUSCLE\_CELL\_ACTION\_POTENTIAL |  | 10 | 0.57 | 1.34 | 0.145 | 0.595 | 1.000 | 985 | tags=40%, list=8%, signal=44% |
| 1031 | GOBP\_SPINDLE\_ASSEMBLY |  | 87 | 0.35 | 1.34 | 0.060 | 0.594 | 1.000 | 4415 | tags=57%, list=37%, signal=90% |
| 1032 | GOBP\_VITAMIN\_A\_METABOLIC\_PROCESS |  | 7 | 0.63 | 1.34 | 0.136 | 0.596 | 1.000 | 406 | tags=43%, list=3%, signal=44% |
| 1033 | GOBP\_INTRA\_S\_DNA\_DAMAGE\_CHECKPOINT |  | 12 | 0.54 | 1.34 | 0.141 | 0.595 | 1.000 | 3566 | tags=58%, list=30%, signal=83% |
| 1034 | GOBP\_NEPHRON\_TUBULE\_EPITHELIAL\_CELL\_DIFFERENTIATION |  | 11 | 0.56 | 1.34 | 0.142 | 0.596 | 1.000 | 78 | tags=18%, list=1%, signal=18% |
| 1035 | GOBP\_ORGANOPHOSPHATE\_ESTER\_TRANSPORT |  | 87 | 0.35 | 1.34 | 0.057 | 0.597 | 1.000 | 1960 | tags=28%, list=16%, signal=33% |
| 1036 | GOBP\_EYE\_PHOTORECEPTOR\_CELL\_DEVELOPMENT |  | 13 | 0.53 | 1.34 | 0.134 | 0.596 | 1.000 | 1870 | tags=46%, list=16%, signal=55% |
| 1037 | GOMF\_ACTIVATING\_TRANSCRIPTION\_FACTOR\_BINDING |  | 62 | 0.37 | 1.34 | 0.075 | 0.596 | 1.000 | 1369 | tags=23%, list=11%, signal=25% |
| 1038 | GOBP\_MUCUS\_SECRETION |  | 4 | 0.74 | 1.34 | 0.124 | 0.596 | 1.000 | 2821 | tags=75%, list=23%, signal=98% |
| 1039 | GOCC\_DNA\_REPLICATION\_FACTOR\_A\_COMPLEX |  | 11 | 0.55 | 1.34 | 0.134 | 0.595 | 1.000 | 637 | tags=18%, list=5%, signal=19% |
| 1040 | GOBP\_TRANSEPITHELIAL\_TRANSPORT |  | 23 | 0.46 | 1.34 | 0.116 | 0.596 | 1.000 | 1663 | tags=35%, list=14%, signal=40% |
| 1041 | GOBP\_POSITIVE\_REGULATION\_OF\_SMOOTH\_MUSCLE\_CELL\_MIGRATION |  | 32 | 0.43 | 1.34 | 0.098 | 0.596 | 1.000 | 2440 | tags=38%, list=20%, signal=47% |
| 1042 | GOBP\_METANEPHRIC\_NEPHRON\_TUBULE\_EPITHELIAL\_CELL\_DIFFERENTIATION |  | 4 | 0.74 | 1.34 | 0.130 | 0.596 | 1.000 | 1714 | tags=50%, list=14%, signal=58% |
| 1043 | GOBP\_REGULATION\_OF\_GLYCOGEN\_STARCH\_SYNTHASE\_ACTIVITY |  | 4 | 0.74 | 1.34 | 0.142 | 0.596 | 1.000 | 2680 | tags=75%, list=22%, signal=97% |
| 1044 | GOMF\_PHOSPHATIDYLINOSITOL\_3\_KINASE\_BINDING |  | 25 | 0.45 | 1.34 | 0.109 | 0.597 | 1.000 | 957 | tags=20%, list=8%, signal=22% |
| 1045 | GOCC\_LSM1\_7\_PAT1\_COMPLEX |  | 4 | 0.74 | 1.34 | 0.138 | 0.597 | 1.000 | 3035 | tags=75%, list=25%, signal=100% |
| 1046 | GOBP\_POSITIVE\_REGULATION\_OF\_GLUCONEOGENESIS |  | 11 | 0.55 | 1.34 | 0.143 | 0.597 | 1.000 | 1223 | tags=36%, list=10%, signal=40% |
| 1047 | GOBP\_FATTY\_ACID\_DERIVATIVE\_METABOLIC\_PROCESS |  | 50 | 0.38 | 1.34 | 0.080 | 0.596 | 1.000 | 1433 | tags=26%, list=12%, signal=29% |
| 1048 | GOCC\_CAMP\_DEPENDENT\_PROTEIN\_KINASE\_COMPLEX |  | 6 | 0.66 | 1.34 | 0.144 | 0.596 | 1.000 | 2262 | tags=50%, list=19%, signal=62% |
| 1049 | GOBP\_NEGATIVE\_REGULATION\_OF\_CELL\_ACTIVATION |  | 119 | 0.33 | 1.34 | 0.050 | 0.596 | 1.000 | 1368 | tags=18%, list=11%, signal=21% |
| 1050 | GOCC\_ANCHORING\_JUNCTION |  | 617 | 0.28 | 1.34 | 0.003 | 0.596 | 1.000 | 2614 | tags=29%, list=22%, signal=36% |
| 1051 | GOBP\_MARGINAL\_ZONE\_B\_CELL\_DIFFERENTIATION |  | 8 | 0.60 | 1.34 | 0.150 | 0.597 | 1.000 | 713 | tags=25%, list=6%, signal=27% |
| 1052 | GOBP\_REGULATION\_OF\_CELLULAR\_PROTEIN\_LOCALIZATION |  | 406 | 0.29 | 1.34 | 0.006 | 0.597 | 1.000 | 2190 | tags=22%, list=18%, signal=26% |
| 1053 | GOMF\_MANNOSYL\_OLIGOSACCHARIDE\_1\_2\_ALPHA\_MANNOSIDASE\_ACTIVITY |  | 7 | 0.63 | 1.34 | 0.144 | 0.597 | 1.000 | 1253 | tags=29%, list=10%, signal=32% |
| 1054 | GOBP\_REGULATION\_OF\_PROTEIN\_LOCALIZATION\_TO\_ENDOSOME |  | 10 | 0.56 | 1.34 | 0.144 | 0.597 | 1.000 | 1922 | tags=40%, list=16%, signal=48% |
| 1055 | GOBP\_TRIGLYCERIDE\_RICH\_LIPOPROTEIN\_PARTICLE\_REMODELING |  | 6 | 0.65 | 1.34 | 0.142 | 0.598 | 1.000 | 954 | tags=67%, list=8%, signal=72% |
| 1056 | GOBP\_NEGATIVE\_REGULATION\_OF\_ERYTHROCYTE\_DIFFERENTIATION |  | 8 | 0.61 | 1.33 | 0.148 | 0.601 | 1.000 | 264 | tags=25%, list=2%, signal=26% |
| 1057 | GOMF\_ENDOPEPTIDASE\_REGULATOR\_ACTIVITY |  | 79 | 0.35 | 1.33 | 0.060 | 0.601 | 1.000 | 1201 | tags=20%, list=10%, signal=22% |
| 1058 | GOBP\_REGULATION\_OF\_NON\_MEMBRANE\_SPANNING\_PROTEIN\_TYROSINE\_KINASE\_ACTIVITY |  | 5 | 0.69 | 1.33 | 0.144 | 0.600 | 1.000 | 273 | tags=20%, list=2%, signal=20% |
| 1059 | GOBP\_FATTY\_ACYL\_COA\_BIOSYNTHETIC\_PROCESS |  | 26 | 0.44 | 1.33 | 0.117 | 0.601 | 1.000 | 1433 | tags=31%, list=12%, signal=35% |
| 1060 | GOBP\_CHROMOSOME\_LOCALIZATION\_TO\_NUCLEAR\_ENVELOPE\_INVOLVED\_IN\_HOMOLOGOUS\_CHROMOSOME\_SEGREGATION |  | 4 | 0.74 | 1.33 | 0.137 | 0.600 | 1.000 | 2597 | tags=75%, list=22%, signal=96% |
| 1061 | GOBP\_SODIUM\_INDEPENDENT\_ORGANIC\_ANION\_TRANSPORT |  | 7 | 0.63 | 1.33 | 0.149 | 0.600 | 1.000 | 3304 | tags=43%, list=28%, signal=59% |
| 1062 | GOBP\_POSITIVE\_REGULATION\_OF\_SARCOMERE\_ORGANIZATION |  | 3 | 0.80 | 1.33 | 0.118 | 0.600 | 1.000 | 569 | tags=33%, list=5%, signal=35% |
| 1063 | GOBP\_ORGAN\_OR\_TISSUE\_SPECIFIC\_IMMUNE\_RESPONSE |  | 7 | 0.62 | 1.33 | 0.154 | 0.600 | 1.000 | 733 | tags=43%, list=6%, signal=46% |
| 1064 | GOBP\_MALE\_GENITALIA\_DEVELOPMENT |  | 13 | 0.53 | 1.33 | 0.140 | 0.601 | 1.000 | 68 | tags=15%, list=1%, signal=15% |
| 1065 | GOBP\_VASCULAR\_ENDOTHELIAL\_GROWTH\_FACTOR\_PRODUCTION |  | 26 | 0.44 | 1.33 | 0.115 | 0.601 | 1.000 | 2627 | tags=46%, list=22%, signal=59% |
| 1066 | GOBP\_NUCLEOSIDE\_MONOPHOSPHATE\_CATABOLIC\_PROCESS |  | 10 | 0.56 | 1.33 | 0.145 | 0.601 | 1.000 | 140 | tags=20%, list=1%, signal=20% |
| 1067 | GOBP\_RESPONSE\_TO\_DSRNA |  | 23 | 0.46 | 1.33 | 0.114 | 0.601 | 1.000 | 1967 | tags=30%, list=16%, signal=36% |
| 1068 | GOBP\_VIRAL\_GENOME\_REPLICATION |  | 82 | 0.35 | 1.33 | 0.060 | 0.601 | 1.000 | 647 | tags=11%, list=5%, signal=12% |
| 1069 | GOBP\_REGULATION\_OF\_CELLULAR\_RESPONSE\_TO\_OSMOTIC\_STRESS |  | 5 | 0.69 | 1.33 | 0.149 | 0.601 | 1.000 | 869 | tags=40%, list=7%, signal=43% |
| 1070 | GOBP\_NEGATIVE\_REGULATION\_OF\_EXTRINSIC\_APOPTOTIC\_SIGNALING\_PATHWAY\_VIA\_DEATH\_DOMAIN\_RECEPTORS |  | 29 | 0.43 | 1.33 | 0.116 | 0.600 | 1.000 | 1559 | tags=24%, list=13%, signal=28% |
| 1071 | GOBP\_MYELIN\_ASSEMBLY |  | 16 | 0.50 | 1.33 | 0.124 | 0.600 | 1.000 | 3317 | tags=50%, list=28%, signal=69% |
| 1072 | GOMF\_PHOSPHATIDYLINOSITOL\_BINDING |  | 187 | 0.31 | 1.33 | 0.028 | 0.600 | 1.000 | 1950 | tags=23%, list=16%, signal=27% |
| 1073 | GOBP\_PYRIMIDINE\_NUCLEOSIDE\_MONOPHOSPHATE\_CATABOLIC\_PROCESS |  | 4 | 0.73 | 1.33 | 0.145 | 0.601 | 1.000 | 6 | tags=25%, list=0%, signal=25% |
| 1074 | GOBP\_DNA\_DAMAGE\_RESPONSE\_SIGNAL\_TRANSDUCTION\_RESULTING\_IN\_TRANSCRIPTION |  | 12 | 0.54 | 1.33 | 0.135 | 0.601 | 1.000 | 2070 | tags=42%, list=17%, signal=50% |
| 1075 | GOCC\_MITOTIC\_SPINDLE\_MIDZONE |  | 12 | 0.54 | 1.33 | 0.138 | 0.600 | 1.000 | 2201 | tags=50%, list=18%, signal=61% |
| 1076 | GOBP\_REGULATION\_OF\_SARCOMERE\_ORGANIZATION |  | 4 | 0.74 | 1.33 | 0.140 | 0.601 | 1.000 | 569 | tags=25%, list=5%, signal=26% |
| 1077 | GOMF\_OXIDOREDUCTASE\_ACTIVITY\_ACTING\_ON\_THE\_CH\_NH2\_GROUP\_OF\_DONORS\_OXYGEN\_AS\_ACCEPTOR |  | 15 | 0.50 | 1.33 | 0.129 | 0.602 | 1.000 | 2504 | tags=40%, list=21%, signal=50% |
| 1078 | GOBP\_CELL\_DIVISION |  | 461 | 0.28 | 1.33 | 0.008 | 0.603 | 1.000 | 3867 | tags=44%, list=32%, signal=62% |
| 1079 | GOBP\_NEGATIVE\_REGULATION\_OF\_GLUCOCORTICOID\_RECEPTOR\_SIGNALING\_PATHWAY |  | 5 | 0.68 | 1.33 | 0.155 | 0.603 | 1.000 | 1561 | tags=60%, list=13%, signal=69% |
| 1080 | GOBP\_NON\_RECOMBINATIONAL\_REPAIR |  | 70 | 0.36 | 1.33 | 0.083 | 0.603 | 1.000 | 3849 | tags=49%, list=32%, signal=71% |
| 1081 | GOMF\_ACTIN\_FILAMENT\_BINDING |  | 151 | 0.32 | 1.33 | 0.037 | 0.602 | 1.000 | 2178 | tags=31%, list=18%, signal=38% |
| 1082 | GOMF\_FUCOSYLTRANSFERASE\_ACTIVITY |  | 8 | 0.61 | 1.33 | 0.149 | 0.603 | 1.000 | 680 | tags=25%, list=6%, signal=26% |
| 1083 | GOBP\_DOUBLE\_STRAND\_BREAK\_REPAIR\_VIA\_SINGLE\_STRAND\_ANNEALING |  | 4 | 0.73 | 1.33 | 0.146 | 0.602 | 1.000 | 1690 | tags=50%, list=14%, signal=58% |
| 1084 | GOBP\_REGULATION\_OF\_SODIUM\_PROTON\_ANTIPORTER\_ACTIVITY |  | 5 | 0.69 | 1.33 | 0.148 | 0.603 | 1.000 | 2044 | tags=40%, list=17%, signal=48% |
| 1085 | GOBP\_REGULATION\_OF\_CARBOHYDRATE\_BIOSYNTHETIC\_PROCESS |  | 64 | 0.36 | 1.33 | 0.073 | 0.604 | 1.000 | 1415 | tags=27%, list=12%, signal=30% |
| 1086 | GOBP\_NEGATIVE\_REGULATION\_OF\_SECRETION |  | 92 | 0.34 | 1.33 | 0.059 | 0.604 | 1.000 | 1891 | tags=26%, list=16%, signal=31% |
| 1087 | GOBP\_MITOTIC\_DNA\_INTEGRITY\_CHECKPOINT |  | 81 | 0.35 | 1.33 | 0.068 | 0.605 | 1.000 | 3700 | tags=41%, list=31%, signal=58% |
| 1088 | GOCC\_FILOPODIUM |  | 76 | 0.36 | 1.33 | 0.073 | 0.605 | 1.000 | 1939 | tags=28%, list=16%, signal=33% |
| 1089 | GOBP\_RESPONSE\_TO\_GLYCOPROTEIN |  | 6 | 0.65 | 1.33 | 0.160 | 0.605 | 1.000 | 62 | tags=17%, list=1%, signal=17% |
| 1090 | GOBP\_UMP\_CATABOLIC\_PROCESS |  | 4 | 0.73 | 1.33 | 0.140 | 0.605 | 1.000 | 6 | tags=25%, list=0%, signal=25% |
| 1091 | GOBP\_ACYLGLYCEROL\_TRANSPORT |  | 5 | 0.68 | 1.33 | 0.159 | 0.604 | 1.000 | 1853 | tags=60%, list=15%, signal=71% |
| 1092 | GOBP\_RESPONSE\_TO\_VITAMIN\_E |  | 9 | 0.58 | 1.33 | 0.158 | 0.605 | 1.000 | 30 | tags=11%, list=0%, signal=11% |
| 1093 | GOCC\_FICOLIN\_1\_RICH\_GRANULE\_MEMBRANE |  | 36 | 0.41 | 1.33 | 0.112 | 0.605 | 1.000 | 2375 | tags=36%, list=20%, signal=45% |
| 1094 | GOBP\_CELLULAR\_RESPONSE\_TO\_PEPTIDE\_HORMONE\_STIMULUS |  | 236 | 0.30 | 1.33 | 0.025 | 0.606 | 1.000 | 2722 | tags=31%, list=23%, signal=39% |
| 1095 | GOBP\_REGULATION\_OF\_T\_CELL\_CHEMOTAXIS |  | 10 | 0.57 | 1.33 | 0.155 | 0.606 | 1.000 | 1837 | tags=50%, list=15%, signal=59% |
| 1096 | GOBP\_CERAMIDE\_BIOSYNTHETIC\_PROCESS |  | 50 | 0.38 | 1.33 | 0.093 | 0.606 | 1.000 | 1698 | tags=28%, list=14%, signal=32% |
| 1097 | GOBP\_SODIUM\_ION\_TRANSPORT |  | 139 | 0.32 | 1.33 | 0.046 | 0.607 | 1.000 | 1743 | tags=25%, list=15%, signal=29% |
| 1098 | GOBP\_MONOCARBOXYLIC\_ACID\_TRANSPORT |  | 110 | 0.33 | 1.33 | 0.049 | 0.607 | 1.000 | 1711 | tags=24%, list=14%, signal=27% |
| 1099 | GOBP\_POSITIVE\_REGULATION\_OF\_CELL\_MATRIX\_ADHESION |  | 43 | 0.39 | 1.33 | 0.097 | 0.606 | 1.000 | 2843 | tags=42%, list=24%, signal=55% |
| 1100 | GOBP\_OSTEOBLAST\_PROLIFERATION |  | 20 | 0.47 | 1.33 | 0.125 | 0.606 | 1.000 | 1800 | tags=40%, list=15%, signal=47% |
| 1101 | GOBP\_ORGANELLE\_FISSION |  | 344 | 0.29 | 1.33 | 0.016 | 0.607 | 1.000 | 3935 | tags=45%, list=33%, signal=65% |
| 1102 | GOBP\_CELL\_AGING |  | 81 | 0.35 | 1.33 | 0.069 | 0.606 | 1.000 | 1894 | tags=26%, list=16%, signal=31% |
| 1103 | GOBP\_HEPATOCYTE\_DIFFERENTIATION |  | 14 | 0.51 | 1.33 | 0.146 | 0.606 | 1.000 | 1864 | tags=36%, list=16%, signal=42% |
| 1104 | GOMF\_ALCOHOL\_BINDING |  | 61 | 0.37 | 1.33 | 0.075 | 0.605 | 1.000 | 1967 | tags=38%, list=16%, signal=45% |
| 1105 | GOMF\_PRENYLTRANSFERASE\_ACTIVITY |  | 16 | 0.50 | 1.33 | 0.139 | 0.605 | 1.000 | 71 | tags=13%, list=1%, signal=13% |
| 1106 | GOMF\_SIGNALING\_RECEPTOR\_BINDING |  | 873 | 0.27 | 1.33 | 0.002 | 0.605 | 1.000 | 2355 | tags=23%, list=20%, signal=27% |
| 1107 | GOCC\_CTF18\_RFC\_LIKE\_COMPLEX |  | 8 | 0.60 | 1.32 | 0.159 | 0.608 | 1.000 | 3350 | tags=63%, list=28%, signal=87% |
| 1108 | GOMF\_NAD\_P\_H\_OXIDASE\_H2O2\_FORMING\_ACTIVITY |  | 4 | 0.73 | 1.32 | 0.147 | 0.608 | 1.000 | 1969 | tags=50%, list=16%, signal=60% |
| 1109 | GOBP\_NEGATIVE\_REGULATION\_OF\_TYPE\_I\_INTERFERON\_MEDIATED\_SIGNALING\_PATHWAY |  | 11 | 0.54 | 1.32 | 0.149 | 0.609 | 1.000 | 412 | tags=27%, list=3%, signal=28% |
| 1110 | GOCC\_NUCLEAR\_CHROMOSOME |  | 160 | 0.32 | 1.32 | 0.037 | 0.610 | 1.000 | 3700 | tags=38%, list=31%, signal=54% |
| 1111 | GOBP\_OTIC\_VESICLE\_DEVELOPMENT |  | 8 | 0.60 | 1.32 | 0.158 | 0.609 | 1.000 | 3399 | tags=50%, list=28%, signal=70% |
| 1112 | GOCC\_PROTEIN\_LIPID\_COMPLEX |  | 18 | 0.48 | 1.32 | 0.138 | 0.609 | 1.000 | 954 | tags=28%, list=8%, signal=30% |
| 1113 | GOBP\_EPIDERMAL\_GROWTH\_FACTOR\_RECEPTOR\_SIGNALING\_PATHWAY |  | 98 | 0.34 | 1.32 | 0.061 | 0.609 | 1.000 | 2380 | tags=26%, list=20%, signal=32% |
| 1114 | GOBP\_SUPRAMOLECULAR\_FIBER\_ORGANIZATION |  | 495 | 0.28 | 1.32 | 0.007 | 0.609 | 1.000 | 2885 | tags=32%, list=24%, signal=41% |
| 1115 | GOBP\_CAMP\_MEDIATED\_SIGNALING |  | 43 | 0.39 | 1.32 | 0.094 | 0.609 | 1.000 | 1245 | tags=21%, list=10%, signal=23% |
| 1116 | GOMF\_HISTONE\_METHYLTRANSFERASE\_BINDING |  | 7 | 0.62 | 1.32 | 0.150 | 0.609 | 1.000 | 1421 | tags=43%, list=12%, signal=49% |
| 1117 | GOBP\_NEGATIVE\_REGULATION\_OF\_MYOBLAST\_DIFFERENTIATION |  | 19 | 0.47 | 1.32 | 0.133 | 0.609 | 1.000 | 1395 | tags=26%, list=12%, signal=30% |
| 1118 | GOCC\_NUCLEAR\_OUTER\_MEMBRANE\_ENDOPLASMIC\_RETICULUM\_MEMBRANE\_NETWORK |  | 782 | 0.27 | 1.32 | 0.001 | 0.609 | 1.000 | 2226 | tags=24%, list=19%, signal=27% |
| 1119 | GOBP\_REGULATION\_OF\_POSTSYNAPTIC\_NEUROTRANSMITTER\_RECEPTOR\_INTERNALIZATION |  | 6 | 0.65 | 1.32 | 0.155 | 0.609 | 1.000 | 2044 | tags=33%, list=17%, signal=40% |
| 1120 | GOBP\_POSITIVE\_REGULATION\_OF\_CARTILAGE\_DEVELOPMENT |  | 19 | 0.47 | 1.32 | 0.134 | 0.608 | 1.000 | 1595 | tags=26%, list=13%, signal=30% |
| 1121 | GOBP\_REGULATION\_OF\_DOUBLE\_STRAND\_BREAK\_REPAIR\_VIA\_HOMOLOGOUS\_RECOMBINATION |  | 34 | 0.42 | 1.32 | 0.113 | 0.608 | 1.000 | 3537 | tags=44%, list=29%, signal=62% |
| 1122 | GOBP\_POSITIVE\_REGULATION\_OF\_CELL\_MORPHOGENESIS\_INVOLVED\_IN\_DIFFERENTIATION |  | 62 | 0.37 | 1.32 | 0.080 | 0.608 | 1.000 | 1986 | tags=23%, list=17%, signal=27% |
| 1123 | GOBP\_POSITIVE\_REGULATION\_OF\_EXTRACELLULAR\_MATRIX\_DISASSEMBLY |  | 6 | 0.65 | 1.32 | 0.171 | 0.608 | 1.000 | 1234 | tags=33%, list=10%, signal=37% |
| 1124 | GOBP\_POSITIVE\_REGULATION\_OF\_CYTOKINE\_PRODUCTION |  | 257 | 0.30 | 1.32 | 0.020 | 0.608 | 1.000 | 1375 | tags=17%, list=11%, signal=19% |
| 1125 | GOBP\_GOLGI\_TO\_PLASMA\_MEMBRANE\_TRANSPORT |  | 49 | 0.38 | 1.32 | 0.099 | 0.608 | 1.000 | 3501 | tags=49%, list=29%, signal=69% |
| 1126 | GOBP\_CHIASMA\_ASSEMBLY |  | 3 | 0.79 | 1.32 | 0.144 | 0.608 | 1.000 | 2560 | tags=100%, list=21%, signal=127% |
| 1127 | GOBP\_NEGATIVE\_REGULATION\_OF\_P38MAPK\_CASCADE |  | 6 | 0.65 | 1.32 | 0.157 | 0.613 | 1.000 | 800 | tags=33%, list=7%, signal=36% |
| 1128 | GOCC\_DNA\_PACKAGING\_COMPLEX |  | 18 | 0.48 | 1.32 | 0.140 | 0.613 | 1.000 | 3251 | tags=50%, list=27%, signal=68% |
| 1129 | GOCC\_INTRINSIC\_COMPONENT\_OF\_PRESYNAPTIC\_MEMBRANE |  | 39 | 0.40 | 1.32 | 0.110 | 0.612 | 1.000 | 1981 | tags=28%, list=16%, signal=34% |
| 1130 | GOBP\_CELLULAR\_RESPONSE\_TO\_INSULIN\_STIMULUS |  | 172 | 0.31 | 1.32 | 0.036 | 0.612 | 1.000 | 2810 | tags=33%, list=23%, signal=42% |
| 1131 | GOBP\_PROTEIN\_LOCALIZATION\_TO\_CYTOSKELETON |  | 47 | 0.39 | 1.32 | 0.096 | 0.612 | 1.000 | 1668 | tags=19%, list=14%, signal=22% |
| 1132 | GOBP\_FAS\_SIGNALING\_PATHWAY |  | 4 | 0.73 | 1.32 | 0.152 | 0.611 | 1.000 | 192 | tags=25%, list=2%, signal=25% |
| 1133 | GOMF\_HYDROLASE\_ACTIVITY\_ACTING\_ON\_ETHER\_BONDS |  | 8 | 0.60 | 1.32 | 0.159 | 0.612 | 1.000 | 2980 | tags=38%, list=25%, signal=50% |
| 1134 | GOBP\_REGULATION\_OF\_FERTILIZATION |  | 5 | 0.68 | 1.32 | 0.158 | 0.612 | 1.000 | 1082 | tags=40%, list=9%, signal=44% |
| 1135 | GOBP\_NEGATIVE\_REGULATION\_OF\_PROTEIN\_LOCALIZATION\_TO\_MEMBRANE |  | 23 | 0.45 | 1.32 | 0.127 | 0.612 | 1.000 | 2561 | tags=30%, list=21%, signal=39% |
| 1136 | GOBP\_PYRIMIDINE\_CONTAINING\_COMPOUND\_SALVAGE |  | 10 | 0.56 | 1.32 | 0.154 | 0.611 | 1.000 | 1179 | tags=30%, list=10%, signal=33% |
| 1137 | GOBP\_DENDRITIC\_CELL\_CYTOKINE\_PRODUCTION |  | 8 | 0.59 | 1.32 | 0.155 | 0.611 | 1.000 | 40 | tags=13%, list=0%, signal=13% |
| 1138 | GOMF\_JUN\_KINASE\_BINDING |  | 8 | 0.60 | 1.32 | 0.160 | 0.611 | 1.000 | 3329 | tags=38%, list=28%, signal=52% |
| 1139 | GOBP\_ACTIN\_FILAMENT\_ORGANIZATION |  | 309 | 0.29 | 1.32 | 0.018 | 0.613 | 1.000 | 2210 | tags=28%, list=18%, signal=34% |
| 1140 | GOBP\_CHEMOKINE\_C\_X\_C\_MOTIF\_LIGAND\_2\_PRODUCTION |  | 14 | 0.51 | 1.32 | 0.150 | 0.613 | 1.000 | 3000 | tags=50%, list=25%, signal=67% |
| 1141 | GOBP\_PHENOTYPIC\_SWITCHING |  | 6 | 0.64 | 1.32 | 0.159 | 0.613 | 1.000 | 575 | tags=17%, list=5%, signal=17% |
| 1142 | GOBP\_LACTATE\_TRANSMEMBRANE\_TRANSPORT |  | 6 | 0.64 | 1.32 | 0.164 | 0.613 | 1.000 | 0 | tags=17%, list=0%, signal=17% |
| 1143 | GOCC\_CYTOPLASMIC\_MICROTUBULE |  | 55 | 0.37 | 1.32 | 0.087 | 0.613 | 1.000 | 2201 | tags=29%, list=18%, signal=35% |
| 1144 | GOCC\_TRNA\_SPLICING\_LIGASE\_COMPLEX |  | 5 | 0.68 | 1.32 | 0.169 | 0.613 | 1.000 | 3798 | tags=100%, list=32%, signal=146% |
| 1145 | GOCC\_BASOLATERAL\_PLASMA\_MEMBRANE |  | 133 | 0.32 | 1.32 | 0.056 | 0.613 | 1.000 | 1320 | tags=20%, list=11%, signal=23% |
| 1146 | GOBP\_FLUID\_TRANSPORT |  | 22 | 0.45 | 1.32 | 0.142 | 0.613 | 1.000 | 2845 | tags=55%, list=24%, signal=71% |
| 1147 | GOBP\_ANOIKIS |  | 24 | 0.45 | 1.32 | 0.126 | 0.612 | 1.000 | 3030 | tags=50%, list=25%, signal=67% |
| 1148 | GOBP\_NEGATIVE\_REGULATION\_OF\_REGULATORY\_T\_CELL\_DIFFERENTIATION |  | 4 | 0.73 | 1.32 | 0.164 | 0.612 | 1.000 | 114 | tags=25%, list=1%, signal=25% |
| 1149 | GOCC\_MRE11\_COMPLEX |  | 6 | 0.65 | 1.32 | 0.159 | 0.612 | 1.000 | 816 | tags=33%, list=7%, signal=36% |
| 1150 | GOMF\_GLYCEROPHOSPHOLIPID\_FLIPPASE\_ACTIVITY |  | 11 | 0.54 | 1.32 | 0.155 | 0.613 | 1.000 | 786 | tags=27%, list=7%, signal=29% |
| 1151 | GOBP\_RESPONSE\_TO\_ALCOHOL |  | 153 | 0.32 | 1.32 | 0.040 | 0.613 | 1.000 | 2138 | tags=25%, list=18%, signal=31% |
| 1152 | GOBP\_AGING |  | 209 | 0.31 | 1.32 | 0.033 | 0.613 | 1.000 | 2160 | tags=27%, list=18%, signal=32% |
| 1153 | GOBP\_PATTERN\_SPECIFICATION\_INVOLVED\_IN\_KIDNEY\_DEVELOPMENT |  | 3 | 0.78 | 1.32 | 0.136 | 0.613 | 1.000 | 2604 | tags=100%, list=22%, signal=128% |
| 1154 | GOBP\_POSITIVE\_REGULATION\_OF\_CAMP\_MEDIATED\_SIGNALING |  | 6 | 0.65 | 1.32 | 0.173 | 0.613 | 1.000 | 132 | tags=33%, list=1%, signal=34% |
| 1155 | GOBP\_MORPHOGENESIS\_OF\_AN\_ENDOTHELIUM |  | 14 | 0.51 | 1.32 | 0.153 | 0.612 | 1.000 | 2414 | tags=50%, list=20%, signal=63% |
| 1156 | GOBP\_REGULATION\_OF\_CELL\_SUBSTRATE\_ADHESION |  | 159 | 0.31 | 1.32 | 0.040 | 0.612 | 1.000 | 2843 | tags=33%, list=24%, signal=42% |
| 1157 | GOCC\_CENTRIOLE |  | 98 | 0.34 | 1.32 | 0.069 | 0.612 | 1.000 | 4075 | tags=49%, list=34%, signal=74% |
| 1158 | GOBP\_IMPORT\_ACROSS\_PLASMA\_MEMBRANE |  | 93 | 0.34 | 1.31 | 0.072 | 0.612 | 1.000 | 1862 | tags=30%, list=16%, signal=35% |
| 1159 | GOBP\_RETINOL\_METABOLIC\_PROCESS |  | 28 | 0.43 | 1.31 | 0.125 | 0.612 | 1.000 | 795 | tags=32%, list=7%, signal=34% |
| 1160 | GOBP\_PHOSPHATIDYLETHANOLAMINE\_ACYL\_CHAIN\_REMODELING |  | 12 | 0.53 | 1.31 | 0.148 | 0.613 | 1.000 | 1853 | tags=50%, list=15%, signal=59% |
| 1161 | GOBP\_T\_CELL\_CHEMOTAXIS |  | 15 | 0.50 | 1.31 | 0.146 | 0.613 | 1.000 | 1837 | tags=40%, list=15%, signal=47% |
| 1162 | GOBP\_MEMBRANOUS\_SEPTUM\_MORPHOGENESIS |  | 5 | 0.68 | 1.31 | 0.165 | 0.612 | 1.000 | 3270 | tags=60%, list=27%, signal=82% |
| 1163 | GOCC\_ASTROCYTE\_PROJECTION |  | 10 | 0.56 | 1.31 | 0.158 | 0.612 | 1.000 | 800 | tags=20%, list=7%, signal=21% |
| 1164 | GOBP\_NEGATIVE\_REGULATION\_OF\_AXON\_EXTENSION\_INVOLVED\_IN\_AXON\_GUIDANCE |  | 19 | 0.47 | 1.31 | 0.136 | 0.612 | 1.000 | 3037 | tags=53%, list=25%, signal=70% |
| 1165 | GOBP\_DNA\_CONFORMATION\_CHANGE |  | 208 | 0.30 | 1.31 | 0.034 | 0.611 | 1.000 | 3580 | tags=40%, list=30%, signal=56% |
| 1166 | GOBP\_POSITIVE\_REGULATION\_OF\_CELL\_SUBSTRATE\_JUNCTION\_ORGANIZATION |  | 27 | 0.43 | 1.31 | 0.129 | 0.611 | 1.000 | 1794 | tags=30%, list=15%, signal=35% |
| 1167 | GOBP\_INTRINSIC\_APOPTOTIC\_SIGNALING\_PATHWAY\_IN\_RESPONSE\_TO\_OSMOTIC\_STRESS |  | 4 | 0.72 | 1.31 | 0.165 | 0.612 | 1.000 | 1333 | tags=50%, list=11%, signal=56% |
| 1168 | GOMF\_STEROL\_BINDING |  | 47 | 0.38 | 1.31 | 0.095 | 0.612 | 1.000 | 2129 | tags=36%, list=18%, signal=44% |
| 1169 | GOBP\_REGULATION\_OF\_CHROMATIN\_BINDING |  | 15 | 0.50 | 1.31 | 0.154 | 0.612 | 1.000 | 2359 | tags=40%, list=20%, signal=50% |
| 1170 | GOBP\_POSITIVE\_REGULATION\_OF\_MACROPHAGE\_DERIVED\_FOAM\_CELL\_DIFFERENTIATION |  | 10 | 0.56 | 1.31 | 0.155 | 0.611 | 1.000 | 2786 | tags=60%, list=23%, signal=78% |
| 1171 | GOBP\_PROTEIN\_LOCALIZATION\_TO\_ADHERENS\_JUNCTION |  | 5 | 0.67 | 1.31 | 0.167 | 0.612 | 1.000 | 2609 | tags=60%, list=22%, signal=77% |
| 1172 | GOBP\_TYROSINE\_PHOSPHORYLATION\_OF\_STAT\_PROTEIN |  | 42 | 0.39 | 1.31 | 0.101 | 0.616 | 1.000 | 2691 | tags=40%, list=22%, signal=52% |
| 1173 | GOBP\_PRESYNAPTIC\_ACTIVE\_ZONE\_ORGANIZATION |  | 5 | 0.68 | 1.31 | 0.169 | 0.616 | 1.000 | 1649 | tags=60%, list=14%, signal=70% |
| 1174 | GOBP\_POSITIVE\_REGULATION\_OF\_VASCULAR\_ENDOTHELIAL\_GROWTH\_FACTOR\_PRODUCTION |  | 21 | 0.46 | 1.31 | 0.134 | 0.616 | 1.000 | 2627 | tags=48%, list=22%, signal=61% |
| 1175 | GOBP\_POSITIVE\_REGULATION\_OF\_CELLULAR\_PROTEIN\_LOCALIZATION |  | 219 | 0.30 | 1.31 | 0.035 | 0.616 | 1.000 | 2171 | tags=22%, list=18%, signal=26% |
| 1176 | GOMF\_ACETYLGALACTOSAMINYLTRANSFERASE\_ACTIVITY |  | 32 | 0.41 | 1.31 | 0.124 | 0.616 | 1.000 | 3492 | tags=53%, list=29%, signal=75% |
| 1177 | GOBP\_HISTONE\_EXCHANGE |  | 38 | 0.40 | 1.31 | 0.113 | 0.616 | 1.000 | 3970 | tags=58%, list=33%, signal=86% |
| 1178 | GOBP\_REGULATION\_OF\_CELLULAR\_COMPONENT\_SIZE |  | 260 | 0.29 | 1.31 | 0.025 | 0.616 | 1.000 | 2722 | tags=32%, list=23%, signal=40% |
| 1179 | GOBP\_CENTRIOLE\_ELONGATION |  | 6 | 0.65 | 1.31 | 0.155 | 0.616 | 1.000 | 4185 | tags=83%, list=35%, signal=128% |
| 1180 | GOBP\_PYRIMIDINE\_NUCLEOSIDE\_BIOSYNTHETIC\_PROCESS |  | 14 | 0.51 | 1.31 | 0.151 | 0.617 | 1.000 | 1179 | tags=21%, list=10%, signal=24% |
| 1181 | GOBP\_CELLULAR\_RESPONSE\_TO\_RETINOIC\_ACID |  | 37 | 0.40 | 1.31 | 0.115 | 0.617 | 1.000 | 3082 | tags=41%, list=26%, signal=54% |
| 1182 | GOBP\_REGULATION\_OF\_ACTIN\_NUCLEATION |  | 21 | 0.46 | 1.31 | 0.133 | 0.617 | 1.000 | 1949 | tags=29%, list=16%, signal=34% |
| 1183 | GOBP\_PEPTIDYL\_ARGININE\_OMEGA\_N\_METHYLATION |  | 4 | 0.73 | 1.31 | 0.165 | 0.617 | 1.000 | 349 | tags=25%, list=3%, signal=26% |
| 1184 | GOBP\_LIPID\_TRANSLOCATION |  | 34 | 0.41 | 1.31 | 0.114 | 0.617 | 1.000 | 1191 | tags=26%, list=10%, signal=29% |
| 1185 | GOBP\_NEGATIVE\_REGULATION\_OF\_SIGNALING\_RECEPTOR\_ACTIVITY |  | 27 | 0.43 | 1.31 | 0.121 | 0.617 | 1.000 | 1533 | tags=22%, list=13%, signal=25% |
| 1186 | GOBP\_REGULATION\_OF\_SIGNALING\_RECEPTOR\_ACTIVITY |  | 86 | 0.34 | 1.31 | 0.071 | 0.617 | 1.000 | 1856 | tags=26%, list=15%, signal=30% |
| 1187 | GOMF\_LONG\_CHAIN\_FATTY\_ACYL\_COA\_BINDING |  | 6 | 0.65 | 1.31 | 0.167 | 0.617 | 1.000 | 1208 | tags=50%, list=10%, signal=56% |
| 1188 | GOBP\_PROTEIN\_K48\_LINKED\_DEUBIQUITINATION |  | 22 | 0.45 | 1.31 | 0.138 | 0.618 | 1.000 | 2575 | tags=36%, list=21%, signal=46% |
| 1189 | GOMF\_CAMP\_DEPENDENT\_PROTEIN\_KINASE\_INHIBITOR\_ACTIVITY |  | 8 | 0.59 | 1.31 | 0.165 | 0.617 | 1.000 | 925 | tags=25%, list=8%, signal=27% |
| 1190 | GOMF\_RNA\_STEM\_LOOP\_BINDING |  | 12 | 0.53 | 1.31 | 0.165 | 0.617 | 1.000 | 2457 | tags=42%, list=20%, signal=52% |
| 1191 | GOBP\_PROTEIN\_RETENTION\_IN\_ER\_LUMEN |  | 3 | 0.78 | 1.31 | 0.151 | 0.617 | 1.000 | 2622 | tags=100%, list=22%, signal=128% |
| 1192 | GOBP\_REGULATION\_OF\_ENDOSOME\_SIZE |  | 5 | 0.68 | 1.31 | 0.177 | 0.617 | 1.000 | 3111 | tags=80%, list=26%, signal=108% |
| 1193 | GOBP\_CELLULAR\_RESPONSE\_TO\_PEPTIDE |  | 280 | 0.29 | 1.31 | 0.023 | 0.617 | 1.000 | 2722 | tags=30%, list=23%, signal=38% |
| 1194 | GOBP\_ACTIN\_FILAMENT\_BASED\_PROCESS |  | 552 | 0.28 | 1.31 | 0.009 | 0.617 | 1.000 | 2323 | tags=27%, list=19%, signal=31% |
| 1195 | GOCC\_PROTEASOME\_CORE\_COMPLEX\_ALPHA\_SUBUNIT\_COMPLEX |  | 7 | 0.61 | 1.31 | 0.171 | 0.616 | 1.000 | 4284 | tags=86%, list=36%, signal=133% |
| 1196 | GOMF\_CARD\_DOMAIN\_BINDING |  | 10 | 0.55 | 1.31 | 0.170 | 0.617 | 1.000 | 1470 | tags=40%, list=12%, signal=46% |
| 1197 | GOCC\_HOST\_CELL\_PART |  | 3 | 0.78 | 1.31 | 0.150 | 0.618 | 1.000 | 620 | tags=33%, list=5%, signal=35% |
| 1198 | GOBP\_REGULATION\_OF\_PHENOTYPIC\_SWITCHING |  | 3 | 0.78 | 1.31 | 0.157 | 0.617 | 1.000 | 575 | tags=33%, list=5%, signal=35% |
| 1199 | GOBP\_POSITIVE\_REGULATION\_OF\_RESPONSE\_TO\_OXIDATIVE\_STRESS |  | 4 | 0.72 | 1.31 | 0.172 | 0.618 | 1.000 | 1092 | tags=50%, list=9%, signal=55% |
| 1200 | GOBP\_NEGATIVE\_REGULATION\_OF\_DNA\_RECOMBINATION |  | 24 | 0.44 | 1.31 | 0.129 | 0.618 | 1.000 | 4052 | tags=50%, list=34%, signal=75% |
| 1201 | GOCC\_APICOLATERAL\_PLASMA\_MEMBRANE |  | 12 | 0.53 | 1.31 | 0.154 | 0.619 | 1.000 | 832 | tags=25%, list=7%, signal=27% |
| 1202 | GOBP\_REGULATION\_OF\_CHOLESTEROL\_ESTERIFICATION |  | 7 | 0.61 | 1.31 | 0.177 | 0.618 | 1.000 | 733 | tags=43%, list=6%, signal=46% |
| 1203 | GOBP\_REGULATION\_OF\_VOLTAGE\_GATED\_SODIUM\_CHANNEL\_ACTIVITY |  | 3 | 0.78 | 1.31 | 0.154 | 0.618 | 1.000 | 2640 | tags=100%, list=22%, signal=128% |
| 1204 | GOBP\_NEGATIVE\_REGULATION\_OF\_CYTOSOLIC\_CALCIUM\_ION\_CONCENTRATION |  | 6 | 0.65 | 1.31 | 0.166 | 0.618 | 1.000 | 2049 | tags=50%, list=17%, signal=60% |
| 1205 | GOBP\_ENDOCRINE\_SYSTEM\_DEVELOPMENT |  | 76 | 0.35 | 1.31 | 0.083 | 0.618 | 1.000 | 1832 | tags=25%, list=15%, signal=29% |
| 1206 | GOBP\_MIDDLE\_EAR\_MORPHOGENESIS |  | 9 | 0.56 | 1.31 | 0.164 | 0.620 | 1.000 | 549 | tags=33%, list=5%, signal=35% |
| 1207 | GOBP\_NEGATIVE\_REGULATION\_OF\_MULTICELLULAR\_ORGANISMAL\_PROCESS |  | 616 | 0.27 | 1.30 | 0.006 | 0.620 | 1.000 | 2161 | tags=23%, list=18%, signal=26% |
| 1208 | GOBP\_BMP\_SIGNALING\_PATHWAY\_INVOLVED\_IN\_HEART\_DEVELOPMENT |  | 4 | 0.72 | 1.30 | 0.155 | 0.620 | 1.000 | 2351 | tags=75%, list=20%, signal=93% |
| 1209 | GOBP\_NOTOCHORD\_MORPHOGENESIS |  | 5 | 0.68 | 1.30 | 0.168 | 0.619 | 1.000 | 3037 | tags=60%, list=25%, signal=80% |
| 1210 | GOCC\_CATION\_TRANSPORTING\_ATPASE\_COMPLEX |  | 5 | 0.68 | 1.30 | 0.173 | 0.619 | 1.000 | 1854 | tags=60%, list=15%, signal=71% |
| 1211 | GOMF\_GROWTH\_FACTOR\_ACTIVITY |  | 72 | 0.35 | 1.30 | 0.088 | 0.619 | 1.000 | 1794 | tags=26%, list=15%, signal=31% |
| 1212 | GOBP\_DEOXYRIBOSE\_PHOSPHATE\_CATABOLIC\_PROCESS |  | 19 | 0.46 | 1.30 | 0.138 | 0.618 | 1.000 | 585 | tags=16%, list=5%, signal=17% |
| 1213 | GOBP\_DETECTION\_OF\_CELL\_DENSITY |  | 9 | 0.57 | 1.30 | 0.178 | 0.618 | 1.000 | 1030 | tags=33%, list=9%, signal=36% |
| 1214 | GOCC\_ADHERENS\_JUNCTION |  | 128 | 0.32 | 1.30 | 0.053 | 0.618 | 1.000 | 2665 | tags=30%, list=22%, signal=39% |
| 1215 | GOBP\_POSITIVE\_REGULATION\_OF\_STRESS\_ACTIVATED\_PROTEIN\_KINASE\_SIGNALING\_CASCADE |  | 80 | 0.34 | 1.30 | 0.082 | 0.617 | 1.000 | 1809 | tags=23%, list=15%, signal=26% |
| 1216 | GOBP\_CELLULAR\_RESPONSE\_TO\_ALKALOID |  | 19 | 0.47 | 1.30 | 0.139 | 0.617 | 1.000 | 1355 | tags=37%, list=11%, signal=41% |
| 1217 | GOMF\_CALCIUM\_ION\_BINDING |  | 390 | 0.28 | 1.30 | 0.015 | 0.617 | 1.000 | 2343 | tags=27%, list=20%, signal=33% |
| 1218 | GOCC\_GOLGI\_APPARATUS\_SUBCOMPARTMENT |  | 630 | 0.27 | 1.30 | 0.006 | 0.617 | 1.000 | 2622 | tags=27%, list=22%, signal=33% |
| 1219 | GOMF\_POLYPEPTIDE\_N\_ACETYLGALACTOSAMINYLTRANSFERASE\_ACTIVITY |  | 14 | 0.50 | 1.30 | 0.149 | 0.617 | 1.000 | 3492 | tags=64%, list=29%, signal=91% |
| 1220 | GOBP\_POSITIVE\_REGULATION\_OF\_RYANODINE\_SENSITIVE\_CALCIUM\_RELEASE\_CHANNEL\_ACTIVITY |  | 5 | 0.67 | 1.30 | 0.176 | 0.617 | 1.000 | 2462 | tags=40%, list=20%, signal=50% |
| 1221 | GOBP\_POSITIVE\_REGULATION\_OF\_SISTER\_CHROMATID\_COHESION |  | 8 | 0.60 | 1.30 | 0.165 | 0.617 | 1.000 | 4133 | tags=63%, list=34%, signal=95% |
| 1222 | GOBP\_NEUROTRANSMITTER\_RECEPTOR\_INTERNALIZATION |  | 11 | 0.54 | 1.30 | 0.165 | 0.617 | 1.000 | 2044 | tags=36%, list=17%, signal=44% |
| 1223 | GOBP\_REGULATION\_OF\_PROTEIN\_LOCALIZATION\_TO\_CELL\_CORTEX |  | 8 | 0.59 | 1.30 | 0.172 | 0.617 | 1.000 | 3115 | tags=63%, list=26%, signal=84% |
| 1224 | GOBP\_CELLULAR\_RESPONSE\_TO\_KETONE |  | 71 | 0.35 | 1.30 | 0.093 | 0.617 | 1.000 | 792 | tags=18%, list=7%, signal=19% |
| 1225 | GOBP\_T\_CELL\_CYTOKINE\_PRODUCTION |  | 21 | 0.46 | 1.30 | 0.137 | 0.618 | 1.000 | 1198 | tags=24%, list=10%, signal=26% |
| 1226 | GOBP\_FOAM\_CELL\_DIFFERENTIATION |  | 26 | 0.43 | 1.30 | 0.129 | 0.617 | 1.000 | 1326 | tags=27%, list=11%, signal=30% |
| 1227 | GOBP\_REGULATION\_OF\_ACTIN\_FILAMENT\_ORGANIZATION |  | 198 | 0.30 | 1.30 | 0.040 | 0.617 | 1.000 | 2146 | tags=27%, list=18%, signal=32% |
| 1228 | GOBP\_ADHERENS\_JUNCTION\_MAINTENANCE |  | 3 | 0.78 | 1.30 | 0.159 | 0.617 | 1.000 | 818 | tags=33%, list=7%, signal=36% |
| 1229 | GOBP\_REGULATION\_OF\_PROTEIN\_POLYMERIZATION |  | 155 | 0.31 | 1.30 | 0.052 | 0.616 | 1.000 | 2146 | tags=25%, list=18%, signal=30% |
| 1230 | GOBP\_WNT\_SIGNALING\_PATHWAY\_INVOLVED\_IN\_HEART\_DEVELOPMENT |  | 5 | 0.68 | 1.30 | 0.177 | 0.616 | 1.000 | 3857 | tags=100%, list=32%, signal=147% |
| 1231 | GOBP\_REGULATION\_OF\_RESPONSE\_TO\_TUMOR\_CELL |  | 6 | 0.63 | 1.30 | 0.175 | 0.616 | 1.000 | 822 | tags=33%, list=7%, signal=36% |
| 1232 | GOBP\_REGULATION\_OF\_PLATELET\_DERIVED\_GROWTH\_FACTOR\_RECEPTOR\_BETA\_SIGNALING\_PATHWAY |  | 8 | 0.58 | 1.30 | 0.164 | 0.616 | 1.000 | 2608 | tags=63%, list=22%, signal=80% |
| 1233 | GOBP\_NEGATIVE\_REGULATION\_OF\_ACTIN\_FILAMENT\_POLYMERIZATION |  | 44 | 0.38 | 1.30 | 0.108 | 0.616 | 1.000 | 2090 | tags=27%, list=17%, signal=33% |
| 1234 | GOMF\_PHOSPHATIDATE\_PHOSPHATASE\_ACTIVITY |  | 9 | 0.57 | 1.30 | 0.174 | 0.616 | 1.000 | 1889 | tags=44%, list=16%, signal=53% |
| 1235 | GOMF\_SOLUTE\_SODIUM\_SYMPORTER\_ACTIVITY |  | 42 | 0.39 | 1.30 | 0.124 | 0.616 | 1.000 | 1465 | tags=36%, list=12%, signal=41% |
| 1236 | GOBP\_POSITIVE\_REGULATION\_OF\_PROTEIN\_MATURATION |  | 14 | 0.51 | 1.30 | 0.158 | 0.616 | 1.000 | 1853 | tags=36%, list=15%, signal=42% |
| 1237 | GOBP\_HOMOLOGOUS\_RECOMBINATION |  | 39 | 0.39 | 1.30 | 0.116 | 0.616 | 1.000 | 2597 | tags=36%, list=22%, signal=46% |
| 1238 | GOMF\_TRANSFERASE\_ACTIVITY\_TRANSFERRING\_GLYCOSYL\_GROUPS |  | 185 | 0.30 | 1.30 | 0.042 | 0.616 | 1.000 | 2620 | tags=26%, list=22%, signal=33% |
| 1239 | GOBP\_RESPONSE\_TO\_IMMOBILIZATION\_STRESS |  | 17 | 0.48 | 1.30 | 0.154 | 0.616 | 1.000 | 1355 | tags=29%, list=11%, signal=33% |
| 1240 | GOBP\_POSITIVE\_REGULATION\_OF\_INTERFERON\_GAMMA\_PRODUCTION |  | 34 | 0.41 | 1.30 | 0.119 | 0.616 | 1.000 | 1198 | tags=21%, list=10%, signal=23% |
| 1241 | GOBP\_FUCOSYLATION |  | 11 | 0.53 | 1.30 | 0.166 | 0.616 | 1.000 | 680 | tags=18%, list=6%, signal=19% |
| 1242 | GOBP\_MULTIVESICULAR\_BODY\_ORGANIZATION |  | 29 | 0.42 | 1.30 | 0.126 | 0.616 | 1.000 | 4173 | tags=59%, list=35%, signal=90% |
| 1243 | GOMF\_OXIDOREDUCTASE\_ACTIVITY\_ACTING\_ON\_THE\_CH\_CH\_GROUP\_OF\_DONORS\_NAD\_OR\_NADP\_AS\_ACCEPTOR |  | 21 | 0.45 | 1.30 | 0.146 | 0.616 | 1.000 | 221 | tags=14%, list=2%, signal=15% |
| 1244 | GOMF\_ANKYRIN\_REPEAT\_BINDING |  | 4 | 0.72 | 1.30 | 0.165 | 0.616 | 1.000 | 983 | tags=25%, list=8%, signal=27% |
| 1245 | GOBP\_CARDIAC\_MUSCLE\_CELL\_CARDIAC\_MUSCLE\_CELL\_ADHESION |  | 5 | 0.68 | 1.30 | 0.183 | 0.616 | 1.000 | 985 | tags=60%, list=8%, signal=65% |
| 1246 | GOBP\_NEGATIVE\_REGULATION\_OF\_GLIOGENESIS |  | 20 | 0.46 | 1.30 | 0.144 | 0.615 | 1.000 | 958 | tags=15%, list=8%, signal=16% |
| 1247 | GOBP\_NUCLEOTIDE\_SALVAGE |  | 12 | 0.52 | 1.30 | 0.165 | 0.615 | 1.000 | 140 | tags=17%, list=1%, signal=17% |
| 1248 | GOBP\_REGULATION\_OF\_T\_HELPER\_2\_CELL\_CYTOKINE\_PRODUCTION |  | 7 | 0.61 | 1.30 | 0.172 | 0.615 | 1.000 | 405 | tags=29%, list=3%, signal=30% |
| 1249 | GOBP\_AZOLE\_TRANSMEMBRANE\_TRANSPORT |  | 8 | 0.59 | 1.30 | 0.172 | 0.616 | 1.000 | 388 | tags=25%, list=3%, signal=26% |
| 1250 | GOBP\_POSITIVE\_REGULATION\_OF\_FATTY\_ACID\_METABOLIC\_PROCESS |  | 27 | 0.43 | 1.30 | 0.138 | 0.616 | 1.000 | 1223 | tags=33%, list=10%, signal=37% |
| 1251 | GOBP\_CENTRIOLE\_CENTRIOLE\_COHESION |  | 13 | 0.51 | 1.30 | 0.160 | 0.615 | 1.000 | 1097 | tags=23%, list=9%, signal=25% |
| 1252 | GOBP\_ACTOMYOSIN\_CONTRACTILE\_RING\_ORGANIZATION |  | 6 | 0.64 | 1.30 | 0.168 | 0.615 | 1.000 | 3045 | tags=67%, list=25%, signal=89% |
| 1253 | GOBP\_LONG\_CHAIN\_FATTY\_ACID\_IMPORT\_ACROSS\_PLASMA\_MEMBRANE |  | 8 | 0.59 | 1.30 | 0.167 | 0.616 | 1.000 | 1326 | tags=50%, list=11%, signal=56% |
| 1254 | GOBP\_POSITIVE\_REGULATION\_OF\_DNA\_REPAIR |  | 52 | 0.37 | 1.30 | 0.102 | 0.616 | 1.000 | 3717 | tags=44%, list=31%, signal=64% |
| 1255 | GOBP\_POSITIVE\_REGULATION\_OF\_AMYLOID\_PRECURSOR\_PROTEIN\_BIOSYNTHETIC\_PROCESS |  | 4 | 0.71 | 1.30 | 0.176 | 0.619 | 1.000 | 2268 | tags=75%, list=19%, signal=92% |
| 1256 | GOBP\_NUCLEOSIDE\_DIPHOSPHATE\_CATABOLIC\_PROCESS |  | 10 | 0.56 | 1.30 | 0.166 | 0.621 | 1.000 | 2021 | tags=40%, list=17%, signal=48% |
| 1257 | GOBP\_CELL\_AGGREGATION |  | 9 | 0.56 | 1.30 | 0.177 | 0.622 | 1.000 | 204 | tags=11%, list=2%, signal=11% |
| 1258 | GOBP\_IMMUNE\_RESPONSE\_TO\_TUMOR\_CELL |  | 7 | 0.61 | 1.30 | 0.182 | 0.621 | 1.000 | 822 | tags=29%, list=7%, signal=31% |
| 1259 | GOBP\_FEMALE\_GENITALIA\_DEVELOPMENT |  | 10 | 0.55 | 1.30 | 0.168 | 0.622 | 1.000 | 620 | tags=20%, list=5%, signal=21% |
| 1260 | GOBP\_RESPONSE\_TO\_AXON\_INJURY |  | 53 | 0.37 | 1.30 | 0.104 | 0.622 | 1.000 | 2131 | tags=30%, list=18%, signal=37% |
| 1261 | GOBP\_CELLULAR\_RESPONSE\_TO\_HORMONE\_STIMULUS |  | 434 | 0.28 | 1.30 | 0.012 | 0.621 | 1.000 | 2013 | tags=22%, list=17%, signal=26% |
| 1262 | GOCC\_RUFFLE\_MEMBRANE |  | 68 | 0.35 | 1.30 | 0.094 | 0.621 | 1.000 | 1977 | tags=29%, list=16%, signal=35% |
| 1263 | GOBP\_AMYLOID\_FIBRIL\_FORMATION |  | 18 | 0.47 | 1.30 | 0.157 | 0.622 | 1.000 | 1326 | tags=33%, list=11%, signal=37% |
| 1264 | GOBP\_REGULATION\_OF\_CYTOSKELETON\_ORGANIZATION |  | 370 | 0.28 | 1.30 | 0.016 | 0.622 | 1.000 | 2722 | tags=29%, list=23%, signal=37% |
| 1265 | GOBP\_CHONDROCYTE\_DIFFERENTIATION\_INVOLVED\_IN\_ENDOCHONDRAL\_BONE\_MORPHOGENESIS |  | 11 | 0.53 | 1.30 | 0.168 | 0.622 | 1.000 | 2420 | tags=45%, list=20%, signal=57% |
| 1266 | GOBP\_REGULATION\_OF\_MEGAKARYOCYTE\_DIFFERENTIATION |  | 42 | 0.39 | 1.29 | 0.114 | 0.623 | 1.000 | 1666 | tags=19%, list=14%, signal=22% |
| 1267 | GOBP\_RESPONSE\_TO\_ORGANIC\_CYCLIC\_COMPOUND |  | 588 | 0.27 | 1.29 | 0.010 | 0.624 | 1.000 | 1421 | tags=18%, list=12%, signal=20% |
| 1268 | GOBP\_ESTABLISHMENT\_OF\_MEIOTIC\_SPINDLE\_LOCALIZATION |  | 3 | 0.77 | 1.29 | 0.170 | 0.624 | 1.000 | 704 | tags=33%, list=6%, signal=35% |
| 1269 | GOMF\_PHOSPHATIDYLINOSITOL\_4\_5\_BISPHOSPHATE\_BINDING |  | 51 | 0.37 | 1.29 | 0.103 | 0.624 | 1.000 | 1082 | tags=22%, list=9%, signal=24% |
| 1270 | GOBP\_CYTOSKELETON\_ORGANIZATION |  | 957 | 0.26 | 1.29 | 0.002 | 0.625 | 1.000 | 2722 | tags=28%, list=23%, signal=34% |
| 1271 | GOBP\_NEGATIVE\_REGULATION\_OF\_IRE1\_MEDIATED\_UNFOLDED\_PROTEIN\_RESPONSE |  | 5 | 0.67 | 1.29 | 0.180 | 0.625 | 1.000 | 2167 | tags=60%, list=18%, signal=73% |
| 1272 | GOBP\_NEGATIVE\_REGULATION\_OF\_LEUKOCYTE\_CELL\_CELL\_ADHESION |  | 73 | 0.35 | 1.29 | 0.094 | 0.625 | 1.000 | 2457 | tags=27%, list=20%, signal=34% |
| 1273 | GOBP\_ASYMMETRIC\_NEUROBLAST\_DIVISION |  | 5 | 0.67 | 1.29 | 0.182 | 0.625 | 1.000 | 3396 | tags=60%, list=28%, signal=84% |
| 1274 | GOMF\_NUCLEOSIDE\_MONOPHOSPHATE\_KINASE\_ACTIVITY |  | 14 | 0.51 | 1.29 | 0.162 | 0.626 | 1.000 | 629 | tags=21%, list=5%, signal=23% |
| 1275 | GOBP\_POSTSYNAPTIC\_MODULATION\_OF\_CHEMICAL\_SYNAPTIC\_TRANSMISSION |  | 8 | 0.58 | 1.29 | 0.181 | 0.625 | 1.000 | 1082 | tags=25%, list=9%, signal=27% |
| 1276 | GOMF\_LACTATE\_TRANSMEMBRANE\_TRANSPORTER\_ACTIVITY |  | 5 | 0.66 | 1.29 | 0.181 | 0.626 | 1.000 | 0 | tags=20%, list=0%, signal=20% |
| 1277 | GOMF\_MANNOSIDASE\_ACTIVITY |  | 13 | 0.51 | 1.29 | 0.161 | 0.625 | 1.000 | 1253 | tags=23%, list=10%, signal=26% |
| 1278 | GOBP\_REGULATION\_OF\_GLUCOCORTICOID\_METABOLIC\_PROCESS |  | 9 | 0.56 | 1.29 | 0.186 | 0.626 | 1.000 | 3109 | tags=67%, list=26%, signal=90% |
| 1279 | GOMF\_UDP\_GALACTOSE\_BETA\_N\_ACETYLGLUCOSAMINE\_BETA\_1\_3\_GALACTOSYLTRANSFERASE\_ACTIVITY |  | 3 | 0.77 | 1.29 | 0.175 | 0.626 | 1.000 | 110 | tags=33%, list=1%, signal=34% |
| 1280 | GOBP\_REGULATION\_OF\_PANCREATIC\_JUICE\_SECRETION |  | 3 | 0.77 | 1.29 | 0.178 | 0.630 | 1.000 | 1416 | tags=67%, list=12%, signal=76% |
| 1281 | GOBP\_POSITIVE\_REGULATION\_OF\_POTASSIUM\_ION\_TRANSMEMBRANE\_TRANSPORTER\_ACTIVITY |  | 12 | 0.52 | 1.29 | 0.168 | 0.630 | 1.000 | 725 | tags=25%, list=6%, signal=27% |
| 1282 | GOMF\_BILE\_ACID\_TRANSMEMBRANE\_TRANSPORTER\_ACTIVITY |  | 7 | 0.61 | 1.29 | 0.184 | 0.631 | 1.000 | 1257 | tags=43%, list=10%, signal=48% |
| 1283 | GOBP\_CORTISOL\_METABOLIC\_PROCESS |  | 7 | 0.61 | 1.29 | 0.185 | 0.631 | 1.000 | 3109 | tags=71%, list=26%, signal=96% |
| 1284 | GOBP\_POSITIVE\_REGULATION\_OF\_AMYLOID\_PRECURSOR\_PROTEIN\_CATABOLIC\_PROCESS |  | 16 | 0.48 | 1.29 | 0.159 | 0.631 | 1.000 | 2592 | tags=44%, list=22%, signal=56% |
| 1285 | GOMF\_LIPID\_BINDING |  | 510 | 0.27 | 1.29 | 0.013 | 0.632 | 1.000 | 1986 | tags=24%, list=17%, signal=28% |
| 1286 | GOBP\_REGULATION\_OF\_BLOOD\_BRAIN\_BARRIER\_PERMEABILITY |  | 6 | 0.63 | 1.29 | 0.190 | 0.633 | 1.000 | 1794 | tags=50%, list=15%, signal=59% |
| 1287 | GOBP\_POSITIVE\_REGULATION\_OF\_TRIGLYCERIDE\_LIPASE\_ACTIVITY |  | 4 | 0.72 | 1.29 | 0.181 | 0.633 | 1.000 | 733 | tags=50%, list=6%, signal=53% |
| 1288 | GOCC\_CHROMOSOME\_CENTROMERIC\_REGION |  | 163 | 0.31 | 1.29 | 0.058 | 0.633 | 1.000 | 3978 | tags=47%, list=33%, signal=70% |
| 1289 | GOBP\_POSITIVE\_REGULATION\_OF\_METAPHASE\_ANAPHASE\_TRANSITION\_OF\_CELL\_CYCLE |  | 14 | 0.50 | 1.29 | 0.162 | 0.633 | 1.000 | 2660 | tags=43%, list=22%, signal=55% |
| 1290 | GOBP\_SECONDARY\_PALATE\_DEVELOPMENT |  | 14 | 0.50 | 1.29 | 0.171 | 0.633 | 1.000 | 3541 | tags=57%, list=29%, signal=81% |
| 1291 | GOBP\_SMALL\_GTPASE\_MEDIATED\_SIGNAL\_TRANSDUCTION |  | 383 | 0.28 | 1.29 | 0.020 | 0.632 | 1.000 | 2274 | tags=26%, list=19%, signal=31% |
| 1292 | GOBP\_CELLULAR\_LIPID\_CATABOLIC\_PROCESS |  | 173 | 0.30 | 1.29 | 0.052 | 0.634 | 1.000 | 1915 | tags=27%, list=16%, signal=31% |
| 1293 | GOBP\_MYOBLAST\_DIFFERENTIATION |  | 63 | 0.36 | 1.29 | 0.103 | 0.634 | 1.000 | 2465 | tags=30%, list=21%, signal=38% |
| 1294 | GOBP\_KINETOCHORE\_ASSEMBLY |  | 16 | 0.48 | 1.29 | 0.162 | 0.634 | 1.000 | 3832 | tags=63%, list=32%, signal=92% |
| 1295 | GOBP\_NEGATIVE\_REGULATION\_OF\_SMALL\_MOLECULE\_METABOLIC\_PROCESS |  | 62 | 0.36 | 1.29 | 0.093 | 0.634 | 1.000 | 1559 | tags=26%, list=13%, signal=30% |
| 1296 | GOBP\_MAMMARY\_GLAND\_DUCT\_MORPHOGENESIS |  | 22 | 0.45 | 1.29 | 0.150 | 0.633 | 1.000 | 82 | tags=9%, list=1%, signal=9% |
| 1297 | GOBP\_REGULATION\_OF\_GLUCOCORTICOID\_BIOSYNTHETIC\_PROCESS |  | 7 | 0.60 | 1.29 | 0.179 | 0.633 | 1.000 | 3109 | tags=71%, list=26%, signal=96% |
| 1298 | GOMF\_STAT\_FAMILY\_PROTEIN\_BINDING |  | 5 | 0.66 | 1.29 | 0.187 | 0.633 | 1.000 | 143 | tags=40%, list=1%, signal=40% |
| 1299 | GOBP\_CELLULAR\_RESPONSE\_TO\_LITHIUM\_ION |  | 9 | 0.56 | 1.29 | 0.180 | 0.633 | 1.000 | 2013 | tags=56%, list=17%, signal=67% |
| 1300 | GOBP\_REGULATION\_OF\_LYSOSOMAL\_PROTEIN\_CATABOLIC\_PROCESS |  | 7 | 0.60 | 1.29 | 0.189 | 0.632 | 1.000 | 2049 | tags=43%, list=17%, signal=52% |
| 1301 | GOBP\_ANTIGEN\_PROCESSING\_AND\_PRESENTATION\_OF\_PEPTIDE\_OR\_POLYSACCHARIDE\_ANTIGEN\_VIA\_MHC\_CLASS\_II |  | 68 | 0.35 | 1.29 | 0.097 | 0.633 | 1.000 | 3868 | tags=46%, list=32%, signal=67% |
| 1302 | GOBP\_ENDODERM\_DEVELOPMENT |  | 49 | 0.37 | 1.29 | 0.120 | 0.636 | 1.000 | 2449 | tags=31%, list=20%, signal=38% |
| 1303 | GOBP\_VESICLE\_ORGANIZATION |  | 263 | 0.29 | 1.29 | 0.035 | 0.636 | 1.000 | 2737 | tags=29%, list=23%, signal=36% |
| 1304 | GOMF\_CYSTEINE\_TYPE\_ENDOPEPTIDASE\_ACTIVITY |  | 72 | 0.35 | 1.29 | 0.100 | 0.636 | 1.000 | 2750 | tags=39%, list=23%, signal=50% |
| 1305 | GOBP\_NEGATIVE\_REGULATION\_OF\_MITOTIC\_CELL\_CYCLE |  | 232 | 0.29 | 1.29 | 0.038 | 0.636 | 1.000 | 4084 | tags=45%, list=34%, signal=67% |
| 1306 | GOMF\_VITAMIN\_D\_BINDING |  | 4 | 0.70 | 1.28 | 0.191 | 0.636 | 1.000 | 2998 | tags=75%, list=25%, signal=100% |
| 1307 | GOBP\_POLYSACCHARIDE\_BIOSYNTHETIC\_PROCESS |  | 52 | 0.37 | 1.28 | 0.122 | 0.635 | 1.000 | 2729 | tags=37%, list=23%, signal=47% |
| 1308 | GOBP\_MINERALOCORTICOID\_SECRETION |  | 5 | 0.67 | 1.28 | 0.198 | 0.635 | 1.000 | 4000 | tags=100%, list=33%, signal=150% |
| 1309 | GOBP\_FEMALE\_SEX\_DIFFERENTIATION |  | 72 | 0.35 | 1.28 | 0.106 | 0.636 | 1.000 | 3133 | tags=36%, list=26%, signal=49% |
| 1310 | GOBP\_RESPONSE\_TO\_NITROGEN\_COMPOUND |  | 741 | 0.27 | 1.28 | 0.004 | 0.636 | 1.000 | 1650 | tags=19%, list=14%, signal=21% |
| 1311 | GOMF\_4\_GALACTOSYL\_N\_ACETYLGLUCOSAMINIDE\_3\_ALPHA\_L\_FUCOSYLTRANSFERASE\_ACTIVITY |  | 4 | 0.70 | 1.28 | 0.192 | 0.636 | 1.000 | 20 | tags=25%, list=0%, signal=25% |
| 1312 | GOMF\_STRUCTURAL\_CONSTITUENT\_OF\_POSTSYNAPSE |  | 4 | 0.71 | 1.28 | 0.186 | 0.636 | 1.000 | 1026 | tags=50%, list=9%, signal=55% |
| 1313 | GOBP\_CYCLIC\_NUCLEOTIDE\_METABOLIC\_PROCESS |  | 24 | 0.43 | 1.28 | 0.152 | 0.636 | 1.000 | 3608 | tags=50%, list=30%, signal=71% |
| 1314 | GOBP\_CONNECTIVE\_TISSUE\_DEVELOPMENT |  | 163 | 0.31 | 1.28 | 0.060 | 0.636 | 1.000 | 2838 | tags=35%, list=24%, signal=45% |
| 1315 | GOBP\_PROTEIN\_LOCALIZATION\_TO\_KINETOCHORE |  | 16 | 0.48 | 1.28 | 0.168 | 0.637 | 1.000 | 4242 | tags=69%, list=35%, signal=106% |
| 1316 | GOMF\_CALMODULIN\_BINDING |  | 133 | 0.31 | 1.28 | 0.061 | 0.637 | 1.000 | 2377 | tags=32%, list=20%, signal=40% |
| 1317 | GOMF\_HYDROLASE\_ACTIVITY\_ACTING\_ON\_ACID\_ANHYDRIDES |  | 636 | 0.27 | 1.28 | 0.009 | 0.637 | 1.000 | 2242 | tags=23%, list=19%, signal=27% |
| 1318 | GOCC\_G\_PROTEIN\_COUPLED\_RECEPTOR\_DIMERIC\_COMPLEX |  | 4 | 0.70 | 1.28 | 0.192 | 0.637 | 1.000 | 764 | tags=25%, list=6%, signal=27% |
| 1319 | GOBP\_SPLEEN\_DEVELOPMENT |  | 27 | 0.42 | 1.28 | 0.153 | 0.637 | 1.000 | 2457 | tags=33%, list=20%, signal=42% |
| 1320 | GOBP\_OLIGOSACCHARIDE\_CATABOLIC\_PROCESS |  | 7 | 0.60 | 1.28 | 0.196 | 0.637 | 1.000 | 130 | tags=14%, list=1%, signal=14% |
| 1321 | GOBP\_CELL\_PROLIFERATION\_INVOLVED\_IN\_HEART\_MORPHOGENESIS |  | 11 | 0.53 | 1.28 | 0.183 | 0.637 | 1.000 | 2351 | tags=45%, list=20%, signal=56% |
| 1322 | GOBP\_T\_HELPER\_2\_CELL\_CYTOKINE\_PRODUCTION |  | 8 | 0.58 | 1.28 | 0.189 | 0.637 | 1.000 | 405 | tags=25%, list=3%, signal=26% |
| 1323 | GOCC\_CLATHRIN\_COATED\_PIT |  | 55 | 0.36 | 1.28 | 0.116 | 0.637 | 1.000 | 2049 | tags=31%, list=17%, signal=37% |
| 1324 | GOMF\_CADHERIN\_BINDING |  | 258 | 0.29 | 1.28 | 0.032 | 0.638 | 1.000 | 2815 | tags=31%, list=23%, signal=40% |
| 1325 | GOBP\_CELLULAR\_RESPONSE\_TO\_LIPID |  | 349 | 0.28 | 1.28 | 0.027 | 0.637 | 1.000 | 1986 | tags=22%, list=17%, signal=26% |
| 1326 | GOBP\_REGULATION\_OF\_DNA\_REPAIR |  | 94 | 0.33 | 1.28 | 0.089 | 0.637 | 1.000 | 3609 | tags=38%, list=30%, signal=54% |
| 1327 | GOBP\_PYRIMIDINE\_NUCLEOSIDE\_DIPHOSPHATE\_METABOLIC\_PROCESS |  | 5 | 0.67 | 1.28 | 0.200 | 0.637 | 1.000 | 1288 | tags=40%, list=11%, signal=45% |
| 1328 | GOBP\_CORTICAL\_ACTIN\_CYTOSKELETON\_ORGANIZATION |  | 33 | 0.40 | 1.28 | 0.140 | 0.637 | 1.000 | 2549 | tags=36%, list=21%, signal=46% |
| 1329 | GOBP\_DNA\_GEOMETRIC\_CHANGE |  | 92 | 0.33 | 1.28 | 0.082 | 0.637 | 1.000 | 3580 | tags=41%, list=30%, signal=58% |
| 1330 | GOBP\_REGULATION\_OF\_MICROTUBULE\_CYTOSKELETON\_ORGANIZATION |  | 102 | 0.33 | 1.28 | 0.082 | 0.636 | 1.000 | 3691 | tags=41%, list=31%, signal=59% |
| 1331 | GOBP\_SRP\_DEPENDENT\_COTRANSLATIONAL\_PROTEIN\_TARGETING\_TO\_MEMBRANE\_SIGNAL\_SEQUENCE\_RECOGNITION |  | 3 | 0.76 | 1.28 | 0.192 | 0.636 | 1.000 | 179 | tags=33%, list=1%, signal=34% |
| 1332 | GOBP\_ENDOSOME\_TRANSPORT\_VIA\_MULTIVESICULAR\_BODY\_SORTING\_PATHWAY |  | 26 | 0.42 | 1.28 | 0.154 | 0.636 | 1.000 | 3802 | tags=54%, list=32%, signal=79% |
| 1333 | GOBP\_NEGATIVE\_REGULATION\_OF\_T\_CELL\_PROLIFERATION |  | 34 | 0.40 | 1.28 | 0.140 | 0.638 | 1.000 | 1333 | tags=24%, list=11%, signal=26% |
| 1334 | GOBP\_POSITIVE\_REGULATION\_OF\_ANION\_TRANSMEMBRANE\_TRANSPORT |  | 44 | 0.38 | 1.28 | 0.123 | 0.637 | 1.000 | 1636 | tags=32%, list=14%, signal=37% |
| 1335 | GOBP\_CELLULAR\_RESPONSE\_TO\_ORGANIC\_CYCLIC\_COMPOUND |  | 365 | 0.28 | 1.28 | 0.024 | 0.637 | 1.000 | 1561 | tags=19%, list=13%, signal=21% |
| 1336 | GOBP\_3\_UTR\_MEDIATED\_MRNA\_DESTABILIZATION |  | 14 | 0.49 | 1.28 | 0.168 | 0.637 | 1.000 | 2962 | tags=43%, list=25%, signal=57% |
| 1337 | GOBP\_REGULATION\_OF\_ENDOTHELIAL\_TUBE\_MORPHOGENESIS |  | 3 | 0.77 | 1.28 | 0.176 | 0.637 | 1.000 | 2414 | tags=67%, list=20%, signal=83% |
| 1338 | GOMF\_DNA\_BINDING\_TRANSCRIPTION\_FACTOR\_ACTIVITY |  | 541 | 0.27 | 1.28 | 0.011 | 0.637 | 1.000 | 1893 | tags=21%, list=16%, signal=23% |
| 1339 | GOMF\_DEATH\_RECEPTOR\_BINDING |  | 15 | 0.49 | 1.28 | 0.169 | 0.636 | 1.000 | 3205 | tags=40%, list=27%, signal=54% |
| 1340 | GOBP\_THIOESTER\_BIOSYNTHETIC\_PROCESS |  | 44 | 0.38 | 1.28 | 0.127 | 0.636 | 1.000 | 1433 | tags=25%, list=12%, signal=28% |
| 1341 | GOBP\_CELL\_JUNCTION\_ASSEMBLY |  | 279 | 0.29 | 1.28 | 0.033 | 0.636 | 1.000 | 2131 | tags=24%, list=18%, signal=28% |
| 1342 | GOBP\_POSITIVE\_REGULATION\_OF\_INTERLEUKIN\_12\_PRODUCTION |  | 24 | 0.44 | 1.28 | 0.154 | 0.635 | 1.000 | 1517 | tags=29%, list=13%, signal=33% |
| 1343 | GOBP\_REGULATION\_OF\_RIBONUCLEASE\_ACTIVITY |  | 5 | 0.65 | 1.28 | 0.213 | 0.636 | 1.000 | 52 | tags=20%, list=0%, signal=20% |
| 1344 | GOBP\_CELLULAR\_MONOVALENT\_INORGANIC\_ANION\_HOMEOSTASIS |  | 5 | 0.66 | 1.28 | 0.201 | 0.636 | 1.000 | 792 | tags=40%, list=7%, signal=43% |
| 1345 | GOCC\_ESCRT\_III\_COMPLEX |  | 9 | 0.56 | 1.28 | 0.180 | 0.636 | 1.000 | 3003 | tags=56%, list=25%, signal=74% |
| 1346 | GOMF\_CCR5\_CHEMOKINE\_RECEPTOR\_BINDING |  | 4 | 0.71 | 1.28 | 0.193 | 0.636 | 1.000 | 1945 | tags=50%, list=16%, signal=60% |
| 1347 | GOMF\_POLY\_PYRIMIDINE\_TRACT\_BINDING |  | 22 | 0.44 | 1.28 | 0.169 | 0.636 | 1.000 | 2406 | tags=32%, list=20%, signal=40% |
| 1348 | GOBP\_IRIS\_MORPHOGENESIS |  | 6 | 0.62 | 1.28 | 0.194 | 0.637 | 1.000 | 55 | tags=17%, list=0%, signal=17% |
| 1349 | GOCC\_COPII\_VESICLE\_COAT |  | 15 | 0.49 | 1.28 | 0.173 | 0.637 | 1.000 | 3458 | tags=53%, list=29%, signal=75% |
| 1350 | GOBP\_NEGATIVE\_REGULATION\_OF\_LEUKOCYTE\_PROLIFERATION |  | 45 | 0.38 | 1.28 | 0.134 | 0.637 | 1.000 | 1333 | tags=24%, list=11%, signal=27% |
| 1351 | GOMF\_LIGASE\_ACTIVITY\_FORMING\_PHOSPHORIC\_ESTER\_BONDS |  | 5 | 0.65 | 1.28 | 0.193 | 0.637 | 1.000 | 2899 | tags=60%, list=24%, signal=79% |
| 1352 | GOMF\_HIGH\_DENSITY\_LIPOPROTEIN\_PARTICLE\_BINDING |  | 7 | 0.60 | 1.28 | 0.184 | 0.638 | 1.000 | 1326 | tags=29%, list=11%, signal=32% |
| 1353 | GOMF\_GTP\_DEPENDENT\_PROTEIN\_BINDING |  | 18 | 0.46 | 1.28 | 0.173 | 0.638 | 1.000 | 2582 | tags=44%, list=21%, signal=57% |
| 1354 | GOMF\_GAMMA\_TUBULIN\_BINDING |  | 23 | 0.44 | 1.28 | 0.165 | 0.637 | 1.000 | 2942 | tags=39%, list=24%, signal=52% |
| 1355 | GOBP\_PITUITARY\_GLAND\_DEVELOPMENT |  | 24 | 0.43 | 1.28 | 0.163 | 0.637 | 1.000 | 1394 | tags=25%, list=12%, signal=28% |
| 1356 | GOBP\_SCHWANN\_CELL\_DEVELOPMENT |  | 22 | 0.44 | 1.28 | 0.160 | 0.638 | 1.000 | 3629 | tags=50%, list=30%, signal=72% |
| 1357 | GOBP\_ADRENAL\_GLAND\_DEVELOPMENT |  | 16 | 0.48 | 1.28 | 0.169 | 0.638 | 1.000 | 1832 | tags=38%, list=15%, signal=44% |
| 1358 | GOMF\_ACTIN\_MONOMER\_BINDING |  | 14 | 0.50 | 1.28 | 0.180 | 0.638 | 1.000 | 1693 | tags=36%, list=14%, signal=42% |
| 1359 | GOMF\_NADH\_PYROPHOSPHATASE\_ACTIVITY |  | 6 | 0.63 | 1.28 | 0.201 | 0.639 | 1.000 | 3152 | tags=67%, list=26%, signal=90% |
| 1360 | GOBP\_RESPONSE\_TO\_INDOLE\_3\_METHANOL |  | 5 | 0.66 | 1.28 | 0.205 | 0.640 | 1.000 | 4042 | tags=100%, list=34%, signal=151% |
| 1361 | GOMF\_NEUREGULIN\_BINDING |  | 5 | 0.66 | 1.28 | 0.196 | 0.640 | 1.000 | 1603 | tags=40%, list=13%, signal=46% |
| 1362 | GOMF\_STORE\_OPERATED\_CALCIUM\_CHANNEL\_ACTIVITY |  | 8 | 0.58 | 1.28 | 0.184 | 0.640 | 1.000 | 1145 | tags=38%, list=10%, signal=41% |
| 1363 | GOCC\_IMMUNOLOGICAL\_SYNAPSE |  | 28 | 0.42 | 1.28 | 0.156 | 0.640 | 1.000 | 1710 | tags=29%, list=14%, signal=33% |
| 1364 | GOBP\_REGULATION\_OF\_PEPTIDASE\_ACTIVITY |  | 251 | 0.29 | 1.28 | 0.045 | 0.639 | 1.000 | 1237 | tags=17%, list=10%, signal=19% |
| 1365 | GOBP\_BODY\_FLUID\_SECRETION |  | 49 | 0.37 | 1.28 | 0.115 | 0.639 | 1.000 | 2349 | tags=33%, list=20%, signal=40% |
| 1366 | GOMF\_ALPHA\_1\_3\_FUCOSYLTRANSFERASE\_ACTIVITY |  | 6 | 0.63 | 1.28 | 0.199 | 0.638 | 1.000 | 20 | tags=17%, list=0%, signal=17% |
| 1367 | GOMF\_GUANOSINE\_DIPHOSPHATASE\_ACTIVITY |  | 9 | 0.56 | 1.27 | 0.189 | 0.641 | 1.000 | 2021 | tags=44%, list=17%, signal=53% |
| 1368 | GOBP\_PUTRESCINE\_BIOSYNTHETIC\_PROCESS |  | 5 | 0.66 | 1.27 | 0.204 | 0.642 | 1.000 | 3763 | tags=60%, list=31%, signal=87% |
| 1369 | GOBP\_REGULATION\_OF\_MITOTIC\_SPINDLE\_ASSEMBLY |  | 14 | 0.49 | 1.27 | 0.184 | 0.643 | 1.000 | 3691 | tags=50%, list=31%, signal=72% |
| 1370 | GOBP\_ZYMOGEN\_INHIBITION |  | 3 | 0.76 | 1.27 | 0.196 | 0.642 | 1.000 | 1219 | tags=67%, list=10%, signal=74% |
| 1371 | GOMF\_PHOSPHATIDYL\_PHOSPHOLIPASE\_B\_ACTIVITY |  | 8 | 0.57 | 1.27 | 0.195 | 0.642 | 1.000 | 448 | tags=25%, list=4%, signal=26% |
| 1372 | GOBP\_VESICLE\_TETHERING\_TO\_GOLGI |  | 4 | 0.71 | 1.27 | 0.203 | 0.642 | 1.000 | 2438 | tags=75%, list=20%, signal=94% |
| 1373 | GOBP\_REGULATION\_OF\_CELL\_MORPHOGENESIS |  | 225 | 0.29 | 1.27 | 0.049 | 0.641 | 1.000 | 1808 | tags=20%, list=15%, signal=23% |
| 1374 | GOMF\_MICROTUBULE\_BINDING |  | 194 | 0.30 | 1.27 | 0.053 | 0.641 | 1.000 | 3972 | tags=51%, list=33%, signal=74% |
| 1375 | GOBP\_RECEPTOR\_MEDIATED\_ENDOCYTOSIS\_INVOLVED\_IN\_CHOLESTEROL\_TRANSPORT |  | 5 | 0.66 | 1.27 | 0.202 | 0.641 | 1.000 | 159 | tags=20%, list=1%, signal=20% |
| 1376 | GOCC\_SPLICEOSOMAL\_TRI\_SNRNP\_COMPLEX |  | 26 | 0.42 | 1.27 | 0.157 | 0.642 | 1.000 | 3035 | tags=42%, list=25%, signal=56% |
| 1377 | GOCC\_SUPRAMOLECULAR\_POLYMER |  | 581 | 0.27 | 1.27 | 0.014 | 0.642 | 1.000 | 3705 | tags=41%, list=31%, signal=56% |
| 1378 | GOMF\_PROTEIN\_CONTAINING\_COMPLEX\_BINDING |  | 854 | 0.26 | 1.27 | 0.007 | 0.642 | 1.000 | 2354 | tags=23%, list=20%, signal=27% |
| 1379 | GOBP\_MACROPHAGE\_COLONY\_STIMULATING\_FACTOR\_PRODUCTION |  | 3 | 0.76 | 1.27 | 0.180 | 0.641 | 1.000 | 2505 | tags=67%, list=21%, signal=84% |
| 1380 | GOBP\_MHC\_CLASS\_I\_BIOSYNTHETIC\_PROCESS |  | 4 | 0.70 | 1.27 | 0.203 | 0.642 | 1.000 | 2348 | tags=50%, list=20%, signal=62% |
| 1381 | GOBP\_REGULATION\_OF\_FATTY\_ACID\_TRANSPORT |  | 30 | 0.41 | 1.27 | 0.154 | 0.642 | 1.000 | 1223 | tags=27%, list=10%, signal=30% |
| 1382 | GOBP\_ADENOHYPOPHYSIS\_DEVELOPMENT |  | 5 | 0.67 | 1.27 | 0.197 | 0.642 | 1.000 | 1379 | tags=40%, list=11%, signal=45% |
| 1383 | GOBP\_MALE\_SEX\_DIFFERENTIATION |  | 102 | 0.32 | 1.27 | 0.091 | 0.643 | 1.000 | 902 | tags=14%, list=8%, signal=15% |
| 1384 | GOBP\_INSULIN\_SECRETION\_INVOLVED\_IN\_CELLULAR\_RESPONSE\_TO\_GLUCOSE\_STIMULUS |  | 42 | 0.38 | 1.27 | 0.140 | 0.644 | 1.000 | 2049 | tags=33%, list=17%, signal=40% |
| 1385 | GOBP\_GLUTAMATE\_RECEPTOR\_SIGNALING\_PATHWAY |  | 32 | 0.40 | 1.27 | 0.146 | 0.645 | 1.000 | 2778 | tags=38%, list=23%, signal=49% |
| 1386 | GOBP\_INTERSTRAND\_CROSS\_LINK\_REPAIR |  | 47 | 0.37 | 1.27 | 0.129 | 0.645 | 1.000 | 1948 | tags=28%, list=16%, signal=33% |
| 1387 | GOBP\_RESPONSE\_TO\_PEPTIDE\_HORMONE |  | 307 | 0.28 | 1.27 | 0.037 | 0.644 | 1.000 | 2013 | tags=23%, list=17%, signal=27% |
| 1388 | GOBP\_ISG15\_PROTEIN\_CONJUGATION |  | 3 | 0.75 | 1.27 | 0.194 | 0.644 | 1.000 | 104 | tags=33%, list=1%, signal=34% |
| 1389 | GOBP\_NEGATIVE\_REGULATION\_OF\_INTERFERON\_GAMMA\_PRODUCTION |  | 19 | 0.46 | 1.27 | 0.169 | 0.644 | 1.000 | 978 | tags=26%, list=8%, signal=29% |
| 1390 | GOBP\_REGULATION\_OF\_T\_CELL\_MEDIATED\_CYTOTOXICITY |  | 11 | 0.53 | 1.27 | 0.189 | 0.644 | 1.000 | 509 | tags=27%, list=4%, signal=28% |
| 1391 | GOMF\_DNA\_CLAMP\_LOADER\_ACTIVITY |  | 9 | 0.56 | 1.27 | 0.197 | 0.644 | 1.000 | 3350 | tags=56%, list=28%, signal=77% |
| 1392 | GOMF\_SINGLE\_STRANDED\_RNA\_BINDING |  | 66 | 0.35 | 1.27 | 0.117 | 0.645 | 1.000 | 1886 | tags=20%, list=16%, signal=23% |
| 1393 | GOBP\_REGULATION\_OF\_AMINO\_ACID\_TRANSPORT |  | 19 | 0.45 | 1.27 | 0.159 | 0.645 | 1.000 | 1055 | tags=26%, list=9%, signal=29% |
| 1394 | GOBP\_METANEPHRIC\_MESENCHYMAL\_CELL\_DIFFERENTIATION |  | 4 | 0.71 | 1.27 | 0.202 | 0.645 | 1.000 | 78 | tags=25%, list=1%, signal=25% |
| 1395 | GOBP\_CELLULAR\_LIPID\_METABOLIC\_PROCESS |  | 730 | 0.26 | 1.27 | 0.011 | 0.645 | 1.000 | 2004 | tags=23%, list=17%, signal=26% |
| 1396 | GOBP\_RESPONSE\_TO\_EPIDERMAL\_GROWTH\_FACTOR |  | 34 | 0.40 | 1.27 | 0.145 | 0.645 | 1.000 | 3247 | tags=44%, list=27%, signal=60% |
| 1397 | GOBP\_NIK\_NF\_KAPPAB\_SIGNALING |  | 123 | 0.31 | 1.27 | 0.080 | 0.645 | 1.000 | 711 | tags=10%, list=6%, signal=10% |
| 1398 | GOBP\_EOSINOPHIL\_ACTIVATION |  | 4 | 0.70 | 1.27 | 0.208 | 0.645 | 1.000 | 2701 | tags=75%, list=22%, signal=97% |
| 1399 | GOBP\_ACTIN\_CYTOSKELETON\_REORGANIZATION |  | 84 | 0.33 | 1.27 | 0.104 | 0.645 | 1.000 | 2375 | tags=33%, list=20%, signal=41% |
| 1400 | GOCC\_SUPRAMOLECULAR\_COMPLEX |  | 817 | 0.26 | 1.27 | 0.006 | 0.645 | 1.000 | 3705 | tags=39%, list=31%, signal=53% |
| 1401 | GOBP\_PROGRAMMED\_CELL\_DEATH\_IN\_RESPONSE\_TO\_REACTIVE\_OXYGEN\_SPECIES |  | 8 | 0.57 | 1.27 | 0.201 | 0.645 | 1.000 | 1612 | tags=50%, list=13%, signal=58% |
| 1402 | GOBP\_REGULATION\_OF\_INTERLEUKIN\_1\_MEDIATED\_SIGNALING\_PATHWAY |  | 4 | 0.70 | 1.27 | 0.206 | 0.645 | 1.000 | 1317 | tags=50%, list=11%, signal=56% |
| 1403 | GOBP\_SUCCINATE\_TRANSMEMBRANE\_TRANSPORT |  | 5 | 0.65 | 1.27 | 0.206 | 0.646 | 1.000 | 633 | tags=40%, list=5%, signal=42% |
| 1404 | GOBP\_LIPID\_IMPORT\_INTO\_CELL |  | 24 | 0.43 | 1.27 | 0.159 | 0.645 | 1.000 | 1862 | tags=38%, list=16%, signal=44% |
| 1405 | GOBP\_POSITIVE\_REGULATION\_OF\_CALCIUM\_ION\_TRANSPORT |  | 74 | 0.34 | 1.27 | 0.105 | 0.645 | 1.000 | 764 | tags=16%, list=6%, signal=17% |
| 1406 | GOBP\_REGULATION\_OF\_DIGESTIVE\_SYSTEM\_PROCESS |  | 21 | 0.44 | 1.27 | 0.171 | 0.645 | 1.000 | 1853 | tags=48%, list=15%, signal=56% |
| 1407 | GOBP\_POSITIVE\_REGULATION\_OF\_MHC\_CLASS\_I\_BIOSYNTHETIC\_PROCESS |  | 3 | 0.76 | 1.27 | 0.201 | 0.645 | 1.000 | 2348 | tags=67%, list=20%, signal=83% |
| 1408 | GOBP\_POSITIVE\_REGULATION\_OF\_TYROSINE\_PHOSPHORYLATION\_OF\_STAT\_PROTEIN |  | 30 | 0.41 | 1.27 | 0.159 | 0.645 | 1.000 | 1856 | tags=33%, list=15%, signal=39% |
| 1409 | GOCC\_CONDENSED\_CHROMOSOME\_INNER\_KINETOCHORE |  | 5 | 0.66 | 1.27 | 0.211 | 0.645 | 1.000 | 1180 | tags=40%, list=10%, signal=44% |
| 1410 | GOMF\_CYCLIC\_NUCLEOTIDE\_GATED\_ION\_CHANNEL\_ACTIVITY |  | 3 | 0.75 | 1.27 | 0.193 | 0.646 | 1.000 | 1190 | tags=33%, list=10%, signal=37% |
| 1411 | GOMF\_GDP\_BINDING |  | 60 | 0.35 | 1.27 | 0.122 | 0.646 | 1.000 | 1883 | tags=23%, list=16%, signal=28% |
| 1412 | GOBP\_REGULATION\_OF\_GLUCAGON\_SECRETION |  | 5 | 0.65 | 1.27 | 0.213 | 0.646 | 1.000 | 709 | tags=60%, list=6%, signal=64% |
| 1413 | GOMF\_MONOCARBOXYLIC\_ACID\_TRANSMEMBRANE\_TRANSPORTER\_ACTIVITY |  | 39 | 0.38 | 1.27 | 0.148 | 0.647 | 1.000 | 1862 | tags=31%, list=16%, signal=36% |
| 1414 | GOBP\_NEGATIVE\_REGULATION\_OF\_TRANSPORT |  | 267 | 0.28 | 1.27 | 0.048 | 0.647 | 1.000 | 2044 | tags=22%, list=17%, signal=26% |
| 1415 | GOBP\_CHEMICAL\_HOMEOSTASIS |  | 743 | 0.26 | 1.27 | 0.008 | 0.648 | 1.000 | 2083 | tags=22%, list=17%, signal=26% |
| 1416 | GOMF\_SUCCINATE\_TRANSMEMBRANE\_TRANSPORTER\_ACTIVITY |  | 5 | 0.65 | 1.26 | 0.201 | 0.649 | 1.000 | 633 | tags=40%, list=5%, signal=42% |
| 1417 | GOBP\_NEGATIVE\_REGULATION\_OF\_RESPONSE\_TO\_EXTERNAL\_STIMULUS |  | 210 | 0.29 | 1.26 | 0.052 | 0.649 | 1.000 | 1223 | tags=15%, list=10%, signal=16% |
| 1418 | GOBP\_NEGATIVE\_REGULATION\_OF\_CELL\_ADHESION |  | 186 | 0.30 | 1.26 | 0.063 | 0.649 | 1.000 | 2375 | tags=26%, list=20%, signal=32% |
| 1419 | GOBP\_NEGATIVE\_REGULATION\_OF\_COAGULATION |  | 31 | 0.41 | 1.26 | 0.156 | 0.650 | 1.000 | 3693 | tags=52%, list=31%, signal=74% |
| 1420 | GOBP\_RESPONSE\_TO\_HYDROXYUREA |  | 9 | 0.55 | 1.26 | 0.199 | 0.650 | 1.000 | 2359 | tags=44%, list=20%, signal=55% |
| 1421 | GOBP\_MICROTUBULE\_ORGANIZING\_CENTER\_LOCALIZATION |  | 27 | 0.42 | 1.26 | 0.166 | 0.650 | 1.000 | 2125 | tags=37%, list=18%, signal=45% |
| 1422 | GOCC\_STEREOCILIA\_COUPLING\_LINK |  | 3 | 0.75 | 1.26 | 0.197 | 0.650 | 1.000 | 1870 | tags=67%, list=16%, signal=79% |
| 1423 | GOBP\_POSITIVE\_REGULATION\_OF\_NEUROTRANSMITTER\_UPTAKE |  | 3 | 0.75 | 1.26 | 0.195 | 0.651 | 1.000 | 3030 | tags=100%, list=25%, signal=134% |
| 1424 | GOBP\_CELL\_ADHESION\_INVOLVED\_IN\_HEART\_MORPHOGENESIS |  | 5 | 0.65 | 1.26 | 0.199 | 0.651 | 1.000 | 3270 | tags=60%, list=27%, signal=82% |
| 1425 | GOMF\_PHOSPHATIDYLCHOLINE\_TRANSFER\_ACTIVITY |  | 5 | 0.65 | 1.26 | 0.210 | 0.651 | 1.000 | 943 | tags=40%, list=8%, signal=43% |
| 1426 | GOBP\_REGULATION\_OF\_RESPONSE\_TO\_STRESS |  | 890 | 0.26 | 1.26 | 0.006 | 0.652 | 1.000 | 2169 | tags=20%, list=18%, signal=23% |
| 1427 | GOMF\_CELL\_CELL\_ADHESION\_MEDIATOR\_ACTIVITY |  | 36 | 0.39 | 1.26 | 0.152 | 0.652 | 1.000 | 1060 | tags=22%, list=9%, signal=24% |
| 1428 | GOMF\_STRUCTURAL\_CONSTITUENT\_OF\_CYTOSKELETON |  | 60 | 0.35 | 1.26 | 0.121 | 0.653 | 1.000 | 3686 | tags=52%, list=31%, signal=74% |
| 1429 | GOBP\_REGULATION\_OF\_NATURAL\_KILLER\_CELL\_CHEMOTAXIS |  | 4 | 0.69 | 1.26 | 0.216 | 0.652 | 1.000 | 1945 | tags=75%, list=16%, signal=89% |
| 1430 | GOMF\_RNA\_POLYMERASE\_II\_TRANSCRIPTION\_FACTOR\_BINDING |  | 33 | 0.40 | 1.26 | 0.154 | 0.652 | 1.000 | 1169 | tags=15%, list=10%, signal=17% |
| 1431 | GOBP\_POSITIVE\_REGULATION\_OF\_LAMELLIPODIUM\_ORGANIZATION |  | 28 | 0.41 | 1.26 | 0.152 | 0.654 | 1.000 | 1737 | tags=32%, list=14%, signal=37% |
| 1432 | GOMF\_IGE\_BINDING |  | 3 | 0.76 | 1.26 | 0.199 | 0.655 | 1.000 | 2169 | tags=67%, list=18%, signal=81% |
| 1433 | GOBP\_NEGATIVE\_REGULATION\_OF\_LIPASE\_ACTIVITY |  | 11 | 0.52 | 1.26 | 0.202 | 0.655 | 1.000 | 1374 | tags=36%, list=11%, signal=41% |
| 1434 | GOBP\_POSITIVE\_REGULATION\_OF\_PROTEIN\_PHOSPHORYLATION |  | 554 | 0.27 | 1.26 | 0.018 | 0.655 | 1.000 | 2162 | tags=23%, list=18%, signal=27% |
| 1435 | GOBP\_NEGATIVE\_REGULATION\_OF\_PEPTIDE\_SECRETION |  | 42 | 0.38 | 1.26 | 0.139 | 0.655 | 1.000 | 1255 | tags=24%, list=10%, signal=26% |
| 1436 | GOBP\_BARBED\_END\_ACTIN\_FILAMENT\_CAPPING |  | 19 | 0.45 | 1.26 | 0.184 | 0.656 | 1.000 | 2509 | tags=37%, list=21%, signal=46% |
| 1437 | GOBP\_BONE\_MINERALIZATION\_INVOLVED\_IN\_BONE\_MATURATION |  | 7 | 0.60 | 1.26 | 0.201 | 0.655 | 1.000 | 1379 | tags=43%, list=11%, signal=48% |
| 1438 | GOBP\_NEGATIVE\_REGULATION\_OF\_CELL\_CYCLE\_G1\_S\_PHASE\_TRANSITION |  | 78 | 0.33 | 1.26 | 0.107 | 0.655 | 1.000 | 3466 | tags=44%, list=29%, signal=61% |
| 1439 | GOBP\_PROTEIN\_DNA\_COVALENT\_CROSS\_LINKING\_REPAIR |  | 5 | 0.66 | 1.26 | 0.221 | 0.656 | 1.000 | 4138 | tags=100%, list=34%, signal=152% |
| 1440 | GOBP\_PROTEIN\_AUTOPHOSPHORYLATION |  | 172 | 0.30 | 1.26 | 0.070 | 0.656 | 1.000 | 2912 | tags=32%, list=24%, signal=42% |
| 1441 | GOBP\_NUCLEOSIDE\_MONOPHOSPHATE\_PHOSPHORYLATION |  | 9 | 0.55 | 1.26 | 0.198 | 0.655 | 1.000 | 816 | tags=22%, list=7%, signal=24% |
| 1442 | GOBP\_POSITIVE\_REGULATION\_OF\_MULTICELLULAR\_ORGANISM\_GROWTH |  | 17 | 0.47 | 1.26 | 0.187 | 0.655 | 1.000 | 2959 | tags=53%, list=25%, signal=70% |
| 1443 | GOBP\_REGULATION\_OF\_FILOPODIUM\_ASSEMBLY |  | 36 | 0.39 | 1.26 | 0.162 | 0.655 | 1.000 | 2446 | tags=42%, list=20%, signal=52% |
| 1444 | GOCC\_MYELIN\_SHEATH\_ADAXONAL\_REGION |  | 3 | 0.76 | 1.26 | 0.205 | 0.655 | 1.000 | 778 | tags=33%, list=6%, signal=36% |
| 1445 | GOBP\_DNA\_RECOMBINATION |  | 198 | 0.29 | 1.26 | 0.063 | 0.654 | 1.000 | 2631 | tags=27%, list=22%, signal=34% |
| 1446 | GOBP\_CARNITINE\_SHUTTLE |  | 9 | 0.55 | 1.26 | 0.210 | 0.654 | 1.000 | 1915 | tags=44%, list=16%, signal=53% |
| 1447 | GOBP\_FIBROBLAST\_GROWTH\_FACTOR\_RECEPTOR\_SIGNALING\_PATHWAY |  | 74 | 0.34 | 1.26 | 0.116 | 0.654 | 1.000 | 3399 | tags=45%, list=28%, signal=62% |
| 1448 | GOBP\_REGULATION\_OF\_T\_HELPER\_17\_TYPE\_IMMUNE\_RESPONSE |  | 8 | 0.56 | 1.26 | 0.206 | 0.653 | 1.000 | 2978 | tags=50%, list=25%, signal=66% |
| 1449 | GOMF\_CYTOKINE\_ACTIVITY |  | 81 | 0.33 | 1.26 | 0.107 | 0.654 | 1.000 | 2691 | tags=41%, list=22%, signal=52% |
| 1450 | GOCC\_SPINDLE |  | 290 | 0.28 | 1.26 | 0.040 | 0.654 | 1.000 | 4110 | tags=46%, list=34%, signal=68% |
| 1451 | GOBP\_RETINAL\_METABOLIC\_PROCESS |  | 11 | 0.52 | 1.26 | 0.198 | 0.654 | 1.000 | 795 | tags=36%, list=7%, signal=39% |
| 1452 | GOBP\_PLACENTA\_BLOOD\_VESSEL\_DEVELOPMENT |  | 22 | 0.43 | 1.26 | 0.171 | 0.655 | 1.000 | 1531 | tags=23%, list=13%, signal=26% |
| 1453 | GOBP\_CHEMOSENSORY\_BEHAVIOR |  | 5 | 0.65 | 1.26 | 0.212 | 0.656 | 1.000 | 1485 | tags=40%, list=12%, signal=46% |
| 1454 | GOCC\_DENDRITIC\_SPINE\_NECK |  | 6 | 0.62 | 1.26 | 0.223 | 0.656 | 1.000 | 1681 | tags=50%, list=14%, signal=58% |
| 1455 | GOBP\_REGULATION\_OF\_CARDIAC\_MUSCLE\_CELL\_ACTION\_POTENTIAL\_INVOLVED\_IN\_REGULATION\_OF\_CONTRACTION |  | 5 | 0.65 | 1.26 | 0.215 | 0.655 | 1.000 | 1967 | tags=60%, list=16%, signal=72% |
| 1456 | GOBP\_NEGATIVE\_REGULATION\_OF\_CARDIAC\_MUSCLE\_ADAPTATION |  | 6 | 0.62 | 1.26 | 0.209 | 0.657 | 1.000 | 3464 | tags=67%, list=29%, signal=94% |
| 1457 | GOBP\_REGULATION\_OF\_HYDROGEN\_PEROXIDE\_MEDIATED\_PROGRAMMED\_CELL\_DEATH |  | 6 | 0.61 | 1.26 | 0.212 | 0.657 | 1.000 | 1612 | tags=50%, list=13%, signal=58% |
| 1458 | GOBP\_RESPONSE\_TO\_GLYCOSIDE |  | 3 | 0.75 | 1.26 | 0.210 | 0.657 | 1.000 | 3009 | tags=100%, list=25%, signal=133% |
| 1459 | GOBP\_REGULATION\_OF\_MICROVILLUS\_ASSEMBLY |  | 5 | 0.65 | 1.26 | 0.214 | 0.656 | 1.000 | 2185 | tags=80%, list=18%, signal=98% |
| 1460 | GOBP\_CELLULAR\_GLUCOSE\_HOMEOSTASIS |  | 95 | 0.32 | 1.26 | 0.103 | 0.657 | 1.000 | 2049 | tags=26%, list=17%, signal=31% |
| 1461 | GOBP\_ARACHIDONIC\_ACID\_METABOLIC\_PROCESS |  | 29 | 0.41 | 1.26 | 0.159 | 0.656 | 1.000 | 472 | tags=14%, list=4%, signal=14% |
| 1462 | GOBP\_POLYSACCHARIDE\_DIGESTION |  | 4 | 0.69 | 1.26 | 0.203 | 0.656 | 1.000 | 1279 | tags=50%, list=11%, signal=56% |
| 1463 | GOBP\_POSITIVE\_REGULATION\_OF\_SENSORY\_PERCEPTION\_OF\_PAIN |  | 4 | 0.69 | 1.26 | 0.212 | 0.656 | 1.000 | 1771 | tags=50%, list=15%, signal=59% |
| 1464 | GOBP\_LIPID\_CATABOLIC\_PROCESS |  | 229 | 0.29 | 1.26 | 0.057 | 0.656 | 1.000 | 1915 | tags=27%, list=16%, signal=32% |
| 1465 | GOMF\_SINGLE\_STRANDED\_DNA\_BINDING |  | 90 | 0.32 | 1.26 | 0.104 | 0.657 | 1.000 | 3378 | tags=36%, list=28%, signal=49% |
| 1466 | GOBP\_EGG\_ACTIVATION |  | 3 | 0.75 | 1.26 | 0.206 | 0.658 | 1.000 | 1082 | tags=33%, list=9%, signal=37% |
| 1467 | GOCC\_CALCIUM\_AND\_CALMODULIN\_DEPENDENT\_PROTEIN\_KINASE\_COMPLEX |  | 4 | 0.69 | 1.26 | 0.218 | 0.657 | 1.000 | 57 | tags=25%, list=0%, signal=25% |
| 1468 | GOMF\_ENDODEOXYRIBONUCLEASE\_ACTIVITY\_PRODUCING\_5\_PHOSPHOMONOESTERS |  | 10 | 0.53 | 1.26 | 0.202 | 0.657 | 1.000 | 3202 | tags=60%, list=27%, signal=82% |
| 1469 | GOBP\_POSITIVE\_REGULATION\_OF\_AUTOPHAGOSOME\_MATURATION |  | 5 | 0.65 | 1.26 | 0.226 | 0.657 | 1.000 | 992 | tags=40%, list=8%, signal=44% |
| 1470 | GOBP\_POSITIVE\_REGULATION\_OF\_NIK\_NF\_KAPPAB\_SIGNALING |  | 49 | 0.36 | 1.25 | 0.143 | 0.657 | 1.000 | 711 | tags=12%, list=6%, signal=13% |
| 1471 | GOBP\_PROTEIN\_CATABOLIC\_PROCESS\_IN\_THE\_VACUOLE |  | 14 | 0.49 | 1.25 | 0.194 | 0.657 | 1.000 | 240 | tags=21%, list=2%, signal=22% |
| 1472 | GOBP\_ACTIVATION\_OF\_JANUS\_KINASE\_ACTIVITY |  | 8 | 0.57 | 1.25 | 0.206 | 0.660 | 1.000 | 2475 | tags=63%, list=21%, signal=79% |
| 1473 | GOBP\_POSITIVE\_REGULATION\_OF\_CHEMOKINE\_PRODUCTION |  | 37 | 0.39 | 1.25 | 0.159 | 0.660 | 1.000 | 304 | tags=11%, list=3%, signal=11% |
| 1474 | GOBP\_REGULATION\_OF\_CARTILAGE\_DEVELOPMENT |  | 42 | 0.37 | 1.25 | 0.157 | 0.660 | 1.000 | 2712 | tags=38%, list=23%, signal=49% |
| 1475 | GOBP\_EPITHELIUM\_DEVELOPMENT |  | 740 | 0.26 | 1.25 | 0.013 | 0.661 | 1.000 | 1883 | tags=20%, list=16%, signal=22% |
| 1476 | GOBP\_NEGATIVE\_REGULATION\_OF\_CELL\_POPULATION\_PROLIFERATION |  | 437 | 0.27 | 1.25 | 0.030 | 0.663 | 1.000 | 1923 | tags=21%, list=16%, signal=24% |
| 1477 | GOCC\_INTEGRAL\_COMPONENT\_OF\_LYSOSOMAL\_MEMBRANE |  | 6 | 0.62 | 1.25 | 0.214 | 0.663 | 1.000 | 2619 | tags=67%, list=22%, signal=85% |
| 1478 | GOBP\_POSITIVE\_REGULATION\_OF\_RECEPTOR\_SIGNALING\_PATHWAY\_VIA\_STAT |  | 24 | 0.42 | 1.25 | 0.168 | 0.664 | 1.000 | 913 | tags=25%, list=8%, signal=27% |
| 1479 | GOBP\_REGULATION\_OF\_INSULIN\_RECEPTOR\_SIGNALING\_PATHWAY |  | 49 | 0.36 | 1.25 | 0.135 | 0.664 | 1.000 | 2013 | tags=29%, list=17%, signal=34% |
| 1480 | GOBP\_REPLICATION\_FORK\_PROCESSING |  | 32 | 0.40 | 1.25 | 0.161 | 0.663 | 1.000 | 2359 | tags=34%, list=20%, signal=43% |
| 1481 | GOBP\_NOTCH\_SIGNALING\_PATHWAY |  | 132 | 0.31 | 1.25 | 0.087 | 0.664 | 1.000 | 2274 | tags=24%, list=19%, signal=30% |
| 1482 | GOBP\_POSITIVE\_REGULATION\_OF\_NATURAL\_KILLER\_CELL\_CHEMOTAXIS |  | 4 | 0.69 | 1.25 | 0.222 | 0.665 | 1.000 | 1945 | tags=75%, list=16%, signal=89% |
| 1483 | GOMF\_VIRION\_BINDING |  | 6 | 0.62 | 1.25 | 0.218 | 0.664 | 1.000 | 125 | tags=17%, list=1%, signal=17% |
| 1484 | GOMF\_NF\_KAPPAB\_INDUCING\_KINASE\_ACTIVITY |  | 4 | 0.69 | 1.25 | 0.215 | 0.665 | 1.000 | 505 | tags=50%, list=4%, signal=52% |
| 1485 | GOBP\_NEGATIVE\_REGULATION\_OF\_WNT\_SIGNALING\_PATHWAY\_INVOLVED\_IN\_HEART\_DEVELOPMENT |  | 3 | 0.75 | 1.25 | 0.222 | 0.666 | 1.000 | 3037 | tags=100%, list=25%, signal=134% |
| 1486 | GOBP\_NEGATIVE\_REGULATION\_OF\_MUSCLE\_HYPERTROPHY |  | 22 | 0.43 | 1.25 | 0.175 | 0.666 | 1.000 | 2853 | tags=45%, list=24%, signal=60% |
| 1487 | GOCC\_POLYMERIC\_CYTOSKELETAL\_FIBER |  | 438 | 0.27 | 1.25 | 0.036 | 0.666 | 1.000 | 2875 | tags=30%, list=24%, signal=38% |
| 1488 | GOBP\_NEGATIVE\_REGULATION\_OF\_CELL\_CYCLE\_PHASE\_TRANSITION |  | 194 | 0.29 | 1.25 | 0.071 | 0.666 | 1.000 | 4084 | tags=44%, list=34%, signal=66% |
| 1489 | GOBP\_LINOLEIC\_ACID\_METABOLIC\_PROCESS |  | 9 | 0.54 | 1.25 | 0.216 | 0.665 | 1.000 | 2762 | tags=44%, list=23%, signal=58% |
| 1490 | GOCC\_CELL\_LEADING\_EDGE |  | 308 | 0.28 | 1.25 | 0.042 | 0.665 | 1.000 | 2201 | tags=25%, list=18%, signal=29% |
| 1491 | GOBP\_MEIOSIS\_II\_CELL\_CYCLE\_PROCESS |  | 6 | 0.61 | 1.25 | 0.224 | 0.666 | 1.000 | 3935 | tags=67%, list=33%, signal=99% |
| 1492 | GOCC\_MESSENGER\_RIBONUCLEOPROTEIN\_COMPLEX |  | 8 | 0.56 | 1.25 | 0.213 | 0.666 | 1.000 | 2205 | tags=38%, list=18%, signal=46% |
| 1493 | GOBP\_REGULATION\_OF\_DNA\_REPLICATION |  | 88 | 0.33 | 1.25 | 0.116 | 0.666 | 1.000 | 2394 | tags=27%, list=20%, signal=34% |
| 1494 | GOBP\_REGULATION\_OF\_EXTRINSIC\_APOPTOTIC\_SIGNALING\_PATHWAY\_IN\_ABSENCE\_OF\_LIGAND |  | 27 | 0.41 | 1.25 | 0.169 | 0.666 | 1.000 | 2274 | tags=30%, list=19%, signal=36% |
| 1495 | GOBP\_CHROMOSOME\_MOVEMENT\_TOWARDS\_SPINDLE\_POLE |  | 6 | 0.61 | 1.25 | 0.224 | 0.666 | 1.000 | 3483 | tags=67%, list=29%, signal=94% |
| 1496 | GOBP\_NEGATIVE\_REGULATION\_OF\_PROTEIN\_KINASE\_B\_SIGNALING |  | 34 | 0.39 | 1.25 | 0.158 | 0.666 | 1.000 | 2649 | tags=38%, list=22%, signal=49% |
| 1497 | GOBP\_EAR\_MORPHOGENESIS |  | 57 | 0.35 | 1.25 | 0.138 | 0.666 | 1.000 | 872 | tags=14%, list=7%, signal=15% |
| 1498 | GOBP\_PEPTIDE\_CROSS\_LINKING |  | 17 | 0.46 | 1.25 | 0.194 | 0.666 | 1.000 | 2360 | tags=59%, list=20%, signal=73% |
| 1499 | GOBP\_LUTEINIZATION |  | 7 | 0.58 | 1.25 | 0.223 | 0.665 | 1.000 | 1947 | tags=43%, list=16%, signal=51% |
| 1500 | GOMF\_KINASE\_ACTIVITY |  | 542 | 0.26 | 1.25 | 0.026 | 0.665 | 1.000 | 2715 | tags=27%, list=23%, signal=34% |
| 1501 | GOBP\_POSITIVE\_REGULATION\_OF\_CENTRIOLE\_REPLICATION |  | 6 | 0.62 | 1.25 | 0.220 | 0.666 | 1.000 | 4185 | tags=83%, list=35%, signal=128% |
| 1502 | GOBP\_NEGATIVE\_REGULATION\_OF\_ANTIGEN\_PROCESSING\_AND\_PRESENTATION |  | 3 | 0.74 | 1.25 | 0.209 | 0.666 | 1.000 | 278 | tags=33%, list=2%, signal=34% |
| 1503 | GOCC\_ASTER |  | 10 | 0.53 | 1.25 | 0.212 | 0.666 | 1.000 | 4415 | tags=80%, list=37%, signal=126% |
| 1504 | GOBP\_ESTABLISHMENT\_OR\_MAINTENANCE\_OF\_CYTOSKELETON\_POLARITY |  | 7 | 0.59 | 1.25 | 0.213 | 0.666 | 1.000 | 3920 | tags=57%, list=33%, signal=85% |
| 1505 | GOBP\_ESTABLISHMENT\_OR\_MAINTENANCE\_OF\_MONOPOLAR\_CELL\_POLARITY |  | 17 | 0.46 | 1.25 | 0.191 | 0.666 | 1.000 | 3037 | tags=53%, list=25%, signal=71% |
| 1506 | GOBP\_STEROID\_ESTERIFICATION |  | 10 | 0.53 | 1.25 | 0.214 | 0.666 | 1.000 | 733 | tags=40%, list=6%, signal=43% |
| 1507 | GOBP\_AMINO\_ACID\_NEUROTRANSMITTER\_REUPTAKE |  | 3 | 0.75 | 1.25 | 0.221 | 0.666 | 1.000 | 3030 | tags=100%, list=25%, signal=134% |
| 1508 | GOMF\_PALMITOYLTRANSFERASE\_ACTIVITY |  | 28 | 0.41 | 1.25 | 0.168 | 0.667 | 1.000 | 2018 | tags=32%, list=17%, signal=39% |
| 1509 | GOBP\_NEGATIVE\_REGULATION\_OF\_GLIAL\_CELL\_MIGRATION |  | 3 | 0.74 | 1.25 | 0.226 | 0.667 | 1.000 | 273 | tags=33%, list=2%, signal=34% |
| 1510 | GOBP\_CELLULAR\_RESPONSE\_TO\_OSMOTIC\_STRESS |  | 28 | 0.40 | 1.25 | 0.170 | 0.667 | 1.000 | 1416 | tags=29%, list=12%, signal=32% |
| 1511 | GOMF\_TRIGLYCERIDE\_LIPASE\_ACTIVITY |  | 13 | 0.49 | 1.25 | 0.209 | 0.668 | 1.000 | 954 | tags=38%, list=8%, signal=42% |
| 1512 | GOBP\_POSITIVE\_REGULATION\_OF\_HOMOTYPIC\_CELL\_CELL\_ADHESION |  | 6 | 0.61 | 1.25 | 0.220 | 0.667 | 1.000 | 1234 | tags=33%, list=10%, signal=37% |
| 1513 | GOBP\_NEGATIVE\_REGULATION\_OF\_CELL\_CYCLE\_PROCESS |  | 262 | 0.28 | 1.25 | 0.053 | 0.667 | 1.000 | 4084 | tags=44%, list=34%, signal=65% |
| 1514 | GOBP\_REGULATION\_OF\_ADENYLATE\_CYCLASE\_ACTIVITY |  | 17 | 0.46 | 1.25 | 0.189 | 0.667 | 1.000 | 960 | tags=24%, list=8%, signal=26% |
| 1515 | GOBP\_CARDIAC\_MUSCLE\_TISSUE\_REGENERATION |  | 4 | 0.69 | 1.25 | 0.225 | 0.666 | 1.000 | 908 | tags=50%, list=8%, signal=54% |
| 1516 | GOMF\_RNA\_POLYMERASE\_II\_CORE\_PROMOTER\_SEQUENCE\_SPECIFIC\_DNA\_BINDING |  | 11 | 0.52 | 1.24 | 0.208 | 0.668 | 1.000 | 1216 | tags=18%, list=10%, signal=20% |
| 1517 | GOBP\_POSITIVE\_REGULATION\_OF\_ORGANIC\_ACID\_TRANSPORT |  | 25 | 0.42 | 1.24 | 0.181 | 0.668 | 1.000 | 1172 | tags=24%, list=10%, signal=27% |
| 1518 | GOCC\_SPERM\_PLASMA\_MEMBRANE |  | 3 | 0.75 | 1.24 | 0.222 | 0.668 | 1.000 | 444 | tags=33%, list=4%, signal=35% |
| 1519 | GOBP\_REGULATION\_OF\_MEMBRANE\_LIPID\_DISTRIBUTION |  | 40 | 0.38 | 1.24 | 0.165 | 0.669 | 1.000 | 1960 | tags=30%, list=16%, signal=36% |
| 1520 | GOMF\_APOLIPOPROTEIN\_RECEPTOR\_BINDING |  | 3 | 0.74 | 1.24 | 0.225 | 0.669 | 1.000 | 589 | tags=33%, list=5%, signal=35% |
| 1521 | GOBP\_REGULATION\_OF\_MITOTIC\_RECOMBINATION |  | 4 | 0.68 | 1.24 | 0.236 | 0.669 | 1.000 | 1305 | tags=50%, list=11%, signal=56% |
| 1522 | GOBP\_REGULATION\_OF\_SUPRAMOLECULAR\_FIBER\_ORGANIZATION |  | 260 | 0.28 | 1.24 | 0.049 | 0.669 | 1.000 | 2090 | tags=24%, list=17%, signal=28% |
| 1523 | GOBP\_RESPONSE\_TO\_DISACCHARIDE |  | 4 | 0.69 | 1.24 | 0.231 | 0.668 | 1.000 | 3556 | tags=75%, list=30%, signal=107% |
| 1524 | GOBP\_NEGATIVE\_REGULATION\_OF\_GLUCOCORTICOID\_METABOLIC\_PROCESS |  | 4 | 0.68 | 1.24 | 0.226 | 0.668 | 1.000 | 3009 | tags=75%, list=25%, signal=100% |
| 1525 | GOBP\_COGNITION |  | 167 | 0.30 | 1.24 | 0.078 | 0.668 | 1.000 | 1485 | tags=21%, list=12%, signal=24% |
| 1526 | GOMF\_AP\_1\_ADAPTOR\_COMPLEX\_BINDING |  | 3 | 0.74 | 1.24 | 0.206 | 0.669 | 1.000 | 1678 | tags=33%, list=14%, signal=39% |
| 1527 | GOCC\_BASAL\_PART\_OF\_CELL |  | 164 | 0.29 | 1.24 | 0.082 | 0.669 | 1.000 | 1956 | tags=24%, list=16%, signal=28% |
| 1528 | GOBP\_SMOOTH\_MUSCLE\_CELL\_PROLIFERATION |  | 102 | 0.32 | 1.24 | 0.115 | 0.669 | 1.000 | 2221 | tags=26%, list=18%, signal=32% |
| 1529 | GOBP\_REGULATION\_OF\_DNA\_DIRECTED\_DNA\_POLYMERASE\_ACTIVITY |  | 9 | 0.54 | 1.24 | 0.209 | 0.669 | 1.000 | 3350 | tags=44%, list=28%, signal=62% |
| 1530 | GOBP\_REGULATION\_OF\_RESPONSE\_TO\_EXTERNAL\_STIMULUS |  | 612 | 0.26 | 1.24 | 0.020 | 0.670 | 1.000 | 2115 | tags=20%, list=18%, signal=23% |
| 1531 | GOBP\_NEGATIVE\_REGULATION\_OF\_TOLL\_LIKE\_RECEPTOR\_SIGNALING\_PATHWAY |  | 21 | 0.43 | 1.24 | 0.189 | 0.671 | 1.000 | 623 | tags=14%, list=5%, signal=15% |
| 1532 | GOBP\_VASCULAR\_ENDOTHELIAL\_GROWTH\_FACTOR\_RECEPTOR\_2\_SIGNALING\_PATHWAY |  | 4 | 0.68 | 1.24 | 0.233 | 0.670 | 1.000 | 3792 | tags=100%, list=32%, signal=146% |
| 1533 | GOBP\_CELLULAR\_RESPONSE\_TO\_GONADOTROPIN\_STIMULUS |  | 8 | 0.56 | 1.24 | 0.222 | 0.671 | 1.000 | 855 | tags=25%, list=7%, signal=27% |
| 1534 | GOBP\_DNA\_REPLICATION\_SYNTHESIS\_OF\_RNA\_PRIMER |  | 5 | 0.64 | 1.24 | 0.237 | 0.671 | 1.000 | 3695 | tags=80%, list=31%, signal=115% |
| 1535 | GOBP\_CYCLIC\_NUCLEOTIDE\_BIOSYNTHETIC\_PROCESS |  | 10 | 0.52 | 1.24 | 0.215 | 0.672 | 1.000 | 1413 | tags=30%, list=12%, signal=34% |
| 1536 | GOBP\_POSITIVE\_REGULATION\_OF\_CIRCADIAN\_RHYTHM |  | 10 | 0.53 | 1.24 | 0.217 | 0.674 | 1.000 | 2956 | tags=50%, list=25%, signal=66% |
| 1537 | GOBP\_RESPONSE\_TO\_LITHIUM\_ION |  | 15 | 0.47 | 1.24 | 0.199 | 0.673 | 1.000 | 2013 | tags=53%, list=17%, signal=64% |
| 1538 | GOBP\_RESPONSE\_TO\_CORTICOSTEROID |  | 97 | 0.32 | 1.24 | 0.120 | 0.673 | 1.000 | 1397 | tags=18%, list=12%, signal=20% |
| 1539 | GOBP\_PROTEIN\_LOCALIZATION\_TO\_SITE\_OF\_DOUBLE\_STRAND\_BREAK |  | 5 | 0.64 | 1.24 | 0.224 | 0.673 | 1.000 | 3849 | tags=80%, list=32%, signal=118% |
| 1540 | GOBP\_REGULATION\_OF\_IMMUNE\_EFFECTOR\_PROCESS |  | 222 | 0.28 | 1.24 | 0.067 | 0.673 | 1.000 | 1326 | tags=14%, list=11%, signal=16% |
| 1541 | GOCC\_IKAPPAB\_KINASE\_COMPLEX |  | 5 | 0.64 | 1.24 | 0.243 | 0.672 | 1.000 | 1315 | tags=40%, list=11%, signal=45% |
| 1542 | GOBP\_DOPAMINE\_BIOSYNTHETIC\_PROCESS |  | 8 | 0.56 | 1.24 | 0.217 | 0.674 | 1.000 | 1716 | tags=50%, list=14%, signal=58% |
| 1543 | GOBP\_FATTY\_ACID\_TRANSMEMBRANE\_TRANSPORT |  | 34 | 0.38 | 1.24 | 0.172 | 0.674 | 1.000 | 1915 | tags=35%, list=16%, signal=42% |
| 1544 | GOBP\_NEGATIVE\_REGULATION\_BY\_HOST\_OF\_VIRAL\_TRANSCRIPTION |  | 11 | 0.52 | 1.24 | 0.212 | 0.674 | 1.000 | 2717 | tags=45%, list=23%, signal=59% |
| 1545 | GOBP\_CELL\_CELL\_ADHESION\_INVOLVED\_IN\_GASTRULATION |  | 7 | 0.58 | 1.24 | 0.225 | 0.673 | 1.000 | 589 | tags=29%, list=5%, signal=30% |
| 1546 | GOMF\_PHOSPHATIDYLINOSITOL\_3\_KINASE\_REGULATOR\_ACTIVITY |  | 16 | 0.47 | 1.24 | 0.209 | 0.674 | 1.000 | 686 | tags=25%, list=6%, signal=26% |
| 1547 | GOBP\_ENDOSOME\_ORGANIZATION |  | 69 | 0.34 | 1.24 | 0.140 | 0.674 | 1.000 | 4173 | tags=51%, list=35%, signal=77% |
| 1548 | GOMF\_DIACYLGLYCEROL\_BINDING |  | 9 | 0.54 | 1.24 | 0.215 | 0.674 | 1.000 | 2339 | tags=56%, list=19%, signal=69% |
| 1549 | GOBP\_LEUKEMIA\_INHIBITORY\_FACTOR\_SIGNALING\_PATHWAY |  | 7 | 0.58 | 1.24 | 0.233 | 0.674 | 1.000 | 659 | tags=29%, list=5%, signal=30% |
| 1550 | GOBP\_SYNAPTIC\_VESICLE\_BUDDING\_FROM\_PRESYNAPTIC\_ENDOCYTIC\_ZONE\_MEMBRANE |  | 4 | 0.68 | 1.24 | 0.243 | 0.674 | 1.000 | 1128 | tags=50%, list=9%, signal=55% |
| 1551 | GOMF\_PROTEIN\_ARGININE\_OMEGA\_N\_MONOMETHYLTRANSFERASE\_ACTIVITY |  | 3 | 0.74 | 1.24 | 0.224 | 0.675 | 1.000 | 349 | tags=33%, list=3%, signal=34% |
| 1552 | GOBP\_MICROTUBULE\_CYTOSKELETON\_ORGANIZATION |  | 418 | 0.27 | 1.24 | 0.041 | 0.675 | 1.000 | 3886 | tags=44%, list=32%, signal=62% |
| 1553 | GOBP\_SPINAL\_CORD\_OLIGODENDROCYTE\_CELL\_DIFFERENTIATION |  | 4 | 0.69 | 1.24 | 0.231 | 0.674 | 1.000 | 347 | tags=50%, list=3%, signal=51% |
| 1554 | GOBP\_REGULATION\_OF\_GLUCOSE\_METABOLIC\_PROCESS |  | 80 | 0.33 | 1.24 | 0.139 | 0.674 | 1.000 | 1427 | tags=23%, list=12%, signal=25% |
| 1555 | GOBP\_POSITIVE\_REGULATION\_OF\_PROTEIN\_AUTOPHOSPHORYLATION |  | 20 | 0.44 | 1.24 | 0.190 | 0.674 | 1.000 | 2759 | tags=40%, list=23%, signal=52% |
| 1556 | GOBP\_PENILE\_ERECTION |  | 8 | 0.56 | 1.24 | 0.237 | 0.674 | 1.000 | 2845 | tags=75%, list=24%, signal=98% |
| 1557 | GOBP\_PHOTOPERIODISM |  | 20 | 0.44 | 1.24 | 0.186 | 0.674 | 1.000 | 2581 | tags=40%, list=21%, signal=51% |
| 1558 | GOBP\_ENDOTHELIAL\_CELL\_FATE\_COMMITMENT |  | 8 | 0.56 | 1.24 | 0.221 | 0.674 | 1.000 | 3075 | tags=50%, list=26%, signal=67% |
| 1559 | GOBP\_MRNA\_3\_SPLICE\_SITE\_RECOGNITION |  | 5 | 0.64 | 1.24 | 0.237 | 0.674 | 1.000 | 3639 | tags=80%, list=30%, signal=115% |
| 1560 | GOBP\_CELL\_COMMUNICATION\_BY\_ELECTRICAL\_COUPLING |  | 20 | 0.43 | 1.24 | 0.194 | 0.674 | 1.000 | 2434 | tags=30%, list=20%, signal=38% |
| 1561 | GOMF\_PHOSPHATIDYLINOSITOL\_BISPHOSPHATE\_BINDING |  | 68 | 0.34 | 1.24 | 0.138 | 0.674 | 1.000 | 1950 | tags=26%, list=16%, signal=31% |
| 1562 | GOBP\_PYRIMIDINE\_RIBONUCLEOTIDE\_CATABOLIC\_PROCESS |  | 6 | 0.61 | 1.24 | 0.236 | 0.673 | 1.000 | 1288 | tags=33%, list=11%, signal=37% |
| 1563 | GOCC\_EXTRINSIC\_COMPONENT\_OF\_MEMBRANE |  | 219 | 0.28 | 1.24 | 0.070 | 0.674 | 1.000 | 2649 | tags=28%, list=22%, signal=35% |
| 1564 | GOBP\_POSITIVE\_REGULATION\_OF\_TRIGLYCERIDE\_CATABOLIC\_PROCESS |  | 5 | 0.64 | 1.24 | 0.237 | 0.675 | 1.000 | 733 | tags=60%, list=6%, signal=64% |
| 1565 | GOBP\_POSITIVE\_REGULATION\_OF\_MONOCYTE\_CHEMOTAXIS |  | 9 | 0.54 | 1.24 | 0.232 | 0.675 | 1.000 | 1918 | tags=44%, list=16%, signal=53% |
| 1566 | GOBP\_ANTIGEN\_PROCESSING\_AND\_PRESENTATION |  | 152 | 0.30 | 1.24 | 0.097 | 0.674 | 1.000 | 2727 | tags=28%, list=23%, signal=36% |
| 1567 | GOBP\_RESPONSE\_TO\_CGMP |  | 5 | 0.64 | 1.24 | 0.237 | 0.675 | 1.000 | 4000 | tags=80%, list=33%, signal=120% |
| 1568 | GOCC\_REPLICATION\_FORK |  | 53 | 0.35 | 1.24 | 0.154 | 0.675 | 1.000 | 3791 | tags=43%, list=32%, signal=63% |
| 1569 | GOBP\_HETEROTYPIC\_CELL\_CELL\_ADHESION |  | 37 | 0.38 | 1.24 | 0.166 | 0.676 | 1.000 | 1187 | tags=22%, list=10%, signal=24% |
| 1570 | GOBP\_POSITIVE\_REGULATION\_OF\_HAIR\_FOLLICLE\_DEVELOPMENT |  | 7 | 0.58 | 1.24 | 0.230 | 0.676 | 1.000 | 3803 | tags=86%, list=32%, signal=125% |
| 1571 | GOMF\_CELL\_ADHESION\_MOLECULE\_BINDING |  | 398 | 0.27 | 1.24 | 0.040 | 0.675 | 1.000 | 2354 | tags=26%, list=20%, signal=31% |
| 1572 | GOBP\_REGULATION\_OF\_CELL\_SUBSTRATE\_JUNCTION\_ORGANIZATION |  | 61 | 0.35 | 1.24 | 0.138 | 0.675 | 1.000 | 3569 | tags=43%, list=30%, signal=60% |
| 1573 | GOBP\_REGULATION\_OF\_CELLULAR\_EXTRAVASATION |  | 23 | 0.42 | 1.24 | 0.194 | 0.675 | 1.000 | 1625 | tags=26%, list=14%, signal=30% |
| 1574 | GOBP\_REGULATION\_OF\_STEM\_CELL\_PROLIFERATION |  | 28 | 0.40 | 1.23 | 0.184 | 0.676 | 1.000 | 1268 | tags=14%, list=11%, signal=16% |
| 1575 | GOCC\_MITOTIC\_SPINDLE\_MICROTUBULE |  | 8 | 0.56 | 1.23 | 0.228 | 0.676 | 1.000 | 4855 | tags=75%, list=40%, signal=126% |
| 1576 | GOMF\_AROMATIC\_AMINO\_ACID\_TRANSMEMBRANE\_TRANSPORTER\_ACTIVITY |  | 7 | 0.58 | 1.23 | 0.232 | 0.676 | 1.000 | 422 | tags=29%, list=4%, signal=30% |
| 1577 | GOBP\_REGULATION\_OF\_T\_CELL\_MIGRATION |  | 29 | 0.40 | 1.23 | 0.185 | 0.676 | 1.000 | 3100 | tags=45%, list=26%, signal=60% |
| 1578 | GOMF\_ADENYLATE\_CYCLASE\_ACTIVATOR\_ACTIVITY |  | 4 | 0.68 | 1.23 | 0.238 | 0.676 | 1.000 | 3562 | tags=50%, list=30%, signal=71% |
| 1579 | GOBP\_REGULATION\_OF\_CHONDROCYTE\_DIFFERENTIATION |  | 33 | 0.39 | 1.23 | 0.174 | 0.677 | 1.000 | 2712 | tags=42%, list=23%, signal=55% |
| 1580 | GOBP\_REGULATION\_OF\_CELL\_MATRIX\_ADHESION |  | 93 | 0.32 | 1.23 | 0.124 | 0.677 | 1.000 | 3569 | tags=44%, list=30%, signal=62% |
| 1581 | GOMF\_ENZYME\_INHIBITOR\_ACTIVITY |  | 204 | 0.29 | 1.23 | 0.076 | 0.677 | 1.000 | 2420 | tags=25%, list=20%, signal=31% |
| 1582 | GOMF\_POLYOL\_TRANSMEMBRANE\_TRANSPORTER\_ACTIVITY |  | 7 | 0.57 | 1.23 | 0.232 | 0.678 | 1.000 | 2673 | tags=71%, list=22%, signal=92% |
| 1583 | GOBP\_MICROTUBULE\_BASED\_PROCESS |  | 577 | 0.26 | 1.23 | 0.029 | 0.678 | 1.000 | 3564 | tags=37%, list=30%, signal=51% |
| 1584 | GOBP\_APOPTOTIC\_CHROMOSOME\_CONDENSATION |  | 3 | 0.73 | 1.23 | 0.231 | 0.678 | 1.000 | 3248 | tags=100%, list=27%, signal=137% |
| 1585 | GOMF\_SIGNALING\_ADAPTOR\_ACTIVITY |  | 54 | 0.35 | 1.23 | 0.150 | 0.679 | 1.000 | 933 | tags=13%, list=8%, signal=14% |
| 1586 | GOBP\_PLASMA\_MEMBRANE\_RAFT\_ASSEMBLY |  | 4 | 0.68 | 1.23 | 0.241 | 0.679 | 1.000 | 1967 | tags=50%, list=16%, signal=60% |
| 1587 | GOBP\_POSITIVE\_REGULATION\_OF\_REPRODUCTIVE\_PROCESS |  | 42 | 0.37 | 1.23 | 0.157 | 0.679 | 1.000 | 3037 | tags=38%, list=25%, signal=51% |
| 1588 | GOBP\_NEGATIVE\_REGULATION\_OF\_CELL\_DIFFERENTIATION |  | 399 | 0.27 | 1.23 | 0.045 | 0.679 | 1.000 | 2988 | tags=30%, list=25%, signal=39% |
| 1589 | GOBP\_REGULATION\_OF\_VASCULAR\_ASSOCIATED\_SMOOTH\_MUSCLE\_CONTRACTION |  | 8 | 0.56 | 1.23 | 0.241 | 0.679 | 1.000 | 1161 | tags=50%, list=10%, signal=55% |
| 1590 | GOBP\_REGULATION\_OF\_IMMUNE\_RESPONSE |  | 502 | 0.26 | 1.23 | 0.032 | 0.679 | 1.000 | 1476 | tags=15%, list=12%, signal=17% |
| 1591 | GOMF\_CENTROMERIC\_DNA\_BINDING |  | 5 | 0.64 | 1.23 | 0.241 | 0.679 | 1.000 | 3430 | tags=60%, list=29%, signal=84% |
| 1592 | GOBP\_GOLGI\_ORGANIZATION |  | 104 | 0.31 | 1.23 | 0.122 | 0.679 | 1.000 | 3140 | tags=37%, list=26%, signal=49% |
| 1593 | GOMF\_CALCIUM\_ACTIVATED\_CATION\_CHANNEL\_ACTIVITY |  | 13 | 0.48 | 1.23 | 0.221 | 0.680 | 1.000 | 2186 | tags=38%, list=18%, signal=47% |
| 1594 | GOBP\_RESPONSE\_TO\_GRAVITY |  | 5 | 0.64 | 1.23 | 0.243 | 0.680 | 1.000 | 1216 | tags=40%, list=10%, signal=44% |
| 1595 | GOMF\_G\_QUADRUPLEX\_RNA\_BINDING |  | 5 | 0.64 | 1.23 | 0.248 | 0.679 | 1.000 | 666 | tags=40%, list=6%, signal=42% |
| 1596 | GOBP\_RESPONSE\_TO\_HEPATOCYTE\_GROWTH\_FACTOR |  | 16 | 0.46 | 1.23 | 0.214 | 0.679 | 1.000 | 178 | tags=13%, list=1%, signal=13% |
| 1597 | GOBP\_HIPPOCAMPUS\_DEVELOPMENT |  | 46 | 0.36 | 1.23 | 0.163 | 0.679 | 1.000 | 2035 | tags=26%, list=17%, signal=31% |
| 1598 | GOMF\_SEMAPHORIN\_RECEPTOR\_BINDING |  | 17 | 0.45 | 1.23 | 0.198 | 0.679 | 1.000 | 2986 | tags=47%, list=25%, signal=63% |
| 1599 | GOBP\_RESPONSE\_TO\_INTERLEUKIN\_7 |  | 22 | 0.42 | 1.23 | 0.192 | 0.679 | 1.000 | 1632 | tags=27%, list=14%, signal=32% |
| 1600 | GOBP\_VACUOLAR\_PROTON\_TRANSPORTING\_V\_TYPE\_ATPASE\_COMPLEX\_ASSEMBLY |  | 4 | 0.68 | 1.23 | 0.251 | 0.679 | 1.000 | 240 | tags=25%, list=2%, signal=26% |
| 1601 | GOBP\_POSITIVE\_REGULATION\_OF\_CYSTEINE\_TYPE\_ENDOPEPTIDASE\_ACTIVITY\_INVOLVED\_IN\_APOPTOTIC\_SIGNALING\_PATHWAY |  | 9 | 0.53 | 1.23 | 0.223 | 0.679 | 1.000 | 2138 | tags=56%, list=18%, signal=68% |
| 1602 | GOBP\_RESPONSE\_TO\_ANTIBIOTIC |  | 37 | 0.38 | 1.23 | 0.169 | 0.680 | 1.000 | 3097 | tags=46%, list=26%, signal=62% |
| 1603 | GOBP\_EQUILIBRIOCEPTION |  | 3 | 0.73 | 1.23 | 0.230 | 0.680 | 1.000 | 1870 | tags=67%, list=16%, signal=79% |
| 1604 | GOBP\_GLAND\_DEVELOPMENT |  | 293 | 0.27 | 1.23 | 0.063 | 0.682 | 1.000 | 1864 | tags=20%, list=16%, signal=24% |
| 1605 | GOBP\_REGULATION\_OF\_INTEGRIN\_ACTIVATION |  | 14 | 0.47 | 1.23 | 0.217 | 0.681 | 1.000 | 976 | tags=21%, list=8%, signal=23% |
| 1606 | GOCC\_SODIUM\_POTASSIUM\_EXCHANGING\_ATPASE\_COMPLEX |  | 4 | 0.68 | 1.23 | 0.247 | 0.681 | 1.000 | 3009 | tags=50%, list=25%, signal=67% |
| 1607 | GOBP\_CELL\_DIFFERENTIATION\_INVOLVED\_IN\_KIDNEY\_DEVELOPMENT |  | 43 | 0.37 | 1.23 | 0.171 | 0.681 | 1.000 | 2680 | tags=35%, list=22%, signal=45% |
| 1608 | GOBP\_REGULATION\_OF\_HYDROGEN\_PEROXIDE\_BIOSYNTHETIC\_PROCESS |  | 4 | 0.68 | 1.23 | 0.239 | 0.681 | 1.000 | 1127 | tags=25%, list=9%, signal=28% |
| 1609 | GOBP\_REGULATION\_OF\_MICROTUBULE\_DEPOLYMERIZATION |  | 19 | 0.44 | 1.23 | 0.191 | 0.681 | 1.000 | 4301 | tags=63%, list=36%, signal=98% |
| 1610 | GOBP\_FATTY\_ACID\_METABOLIC\_PROCESS |  | 258 | 0.28 | 1.23 | 0.068 | 0.681 | 1.000 | 1433 | tags=19%, list=12%, signal=21% |
| 1611 | GOBP\_PROGRAMMED\_CELL\_DEATH\_INVOLVED\_IN\_CELL\_DEVELOPMENT |  | 8 | 0.55 | 1.23 | 0.234 | 0.680 | 1.000 | 769 | tags=25%, list=6%, signal=27% |
| 1612 | GOBP\_REGULATION\_OF\_MESENCHYMAL\_CELL\_PROLIFERATION |  | 20 | 0.43 | 1.23 | 0.199 | 0.681 | 1.000 | 2351 | tags=40%, list=20%, signal=50% |
| 1613 | GOBP\_NEGATIVE\_REGULATION\_OF\_ALPHA\_BETA\_T\_CELL\_PROLIFERATION |  | 6 | 0.60 | 1.23 | 0.240 | 0.681 | 1.000 | 1059 | tags=50%, list=9%, signal=55% |
| 1614 | GOMF\_HISTONE\_KINASE\_ACTIVITY |  | 12 | 0.50 | 1.23 | 0.214 | 0.681 | 1.000 | 2652 | tags=50%, list=22%, signal=64% |
| 1615 | GOBP\_ORGANOPHOSPHATE\_METABOLIC\_PROCESS |  | 732 | 0.25 | 1.23 | 0.018 | 0.682 | 1.000 | 1788 | tags=19%, list=15%, signal=21% |
| 1616 | GOBP\_DEOXYRIBONUCLEOSIDE\_MONOPHOSPHATE\_CATABOLIC\_PROCESS |  | 6 | 0.60 | 1.23 | 0.244 | 0.682 | 1.000 | 6 | tags=17%, list=0%, signal=17% |
| 1617 | GOMF\_TRANSFERASE\_ACTIVITY\_TRANSFERRING\_ACYL\_GROUPS\_ACYL\_GROUPS\_CONVERTED\_INTO\_ALKYL\_ON\_TRANSFER |  | 4 | 0.67 | 1.23 | 0.242 | 0.682 | 1.000 | 30 | tags=25%, list=0%, signal=25% |
| 1618 | GOBP\_REGULATION\_OF\_TRANSLATIONAL\_INITIATION\_IN\_RESPONSE\_TO\_STRESS |  | 13 | 0.48 | 1.23 | 0.218 | 0.682 | 1.000 | 997 | tags=23%, list=8%, signal=25% |
| 1619 | GOBP\_RNA\_DECAPPING |  | 14 | 0.47 | 1.23 | 0.216 | 0.682 | 1.000 | 2829 | tags=29%, list=24%, signal=37% |
| 1620 | GOBP\_REGULATION\_OF\_PHOSPHOLIPID\_TRANSPORT |  | 10 | 0.52 | 1.23 | 0.220 | 0.682 | 1.000 | 769 | tags=30%, list=6%, signal=32% |
| 1621 | GOBP\_POSITIVE\_REGULATION\_OF\_SMALL\_MOLECULE\_METABOLIC\_PROCESS |  | 92 | 0.32 | 1.23 | 0.132 | 0.682 | 1.000 | 1419 | tags=24%, list=12%, signal=27% |
| 1622 | GOBP\_SYNAPTIC\_VESICLE\_DOCKING |  | 8 | 0.55 | 1.23 | 0.232 | 0.682 | 1.000 | 2339 | tags=63%, list=19%, signal=78% |
| 1623 | GOBP\_REGULATION\_OF\_NON\_MOTILE\_CILIUM\_ASSEMBLY |  | 6 | 0.60 | 1.23 | 0.232 | 0.682 | 1.000 | 2295 | tags=33%, list=19%, signal=41% |
| 1624 | GOBP\_AROMATIC\_AMINO\_ACID\_TRANSPORT |  | 9 | 0.54 | 1.23 | 0.233 | 0.682 | 1.000 | 1015 | tags=33%, list=8%, signal=36% |
| 1625 | GOBP\_POSITIVE\_REGULATION\_OF\_B\_CELL\_DIFFERENTIATION |  | 9 | 0.53 | 1.23 | 0.223 | 0.682 | 1.000 | 465 | tags=22%, list=4%, signal=23% |
| 1626 | GOBP\_NEGATIVE\_REGULATION\_OF\_SKELETAL\_MUSCLE\_CELL\_DIFFERENTIATION |  | 3 | 0.73 | 1.23 | 0.239 | 0.683 | 1.000 | 308 | tags=33%, list=3%, signal=34% |
| 1627 | GOBP\_ADENYLATE\_CYCLASE\_MODULATING\_G\_PROTEIN\_COUPLED\_RECEPTOR\_SIGNALING\_PATHWAY |  | 122 | 0.30 | 1.23 | 0.114 | 0.683 | 1.000 | 1619 | tags=22%, list=13%, signal=25% |
| 1628 | GOBP\_CELLULAR\_RESPONSE\_TO\_INTERFERON\_ALPHA |  | 6 | 0.60 | 1.22 | 0.246 | 0.684 | 1.000 | 640 | tags=33%, list=5%, signal=35% |
| 1629 | GOMF\_PHOSPHATIDYLSERINE\_FLIPPASE\_ACTIVITY |  | 5 | 0.63 | 1.22 | 0.250 | 0.684 | 1.000 | 786 | tags=40%, list=7%, signal=43% |
| 1630 | GOBP\_CYCLIC\_NUCLEOTIDE\_MEDIATED\_SIGNALING |  | 56 | 0.35 | 1.22 | 0.160 | 0.684 | 1.000 | 1386 | tags=21%, list=12%, signal=24% |
| 1631 | GOBP\_XENOBIOTIC\_TRANSPORT\_ACROSS\_BLOOD\_BRAIN\_BARRIER |  | 3 | 0.73 | 1.22 | 0.249 | 0.684 | 1.000 | 792 | tags=33%, list=7%, signal=36% |
| 1632 | GOBP\_REGULATION\_OF\_BILE\_ACID\_SECRETION |  | 3 | 0.74 | 1.22 | 0.252 | 0.684 | 1.000 | 3136 | tags=100%, list=26%, signal=135% |
| 1633 | GOBP\_DOUBLE\_STRAND\_BREAK\_REPAIR\_INVOLVED\_IN\_MEIOTIC\_RECOMBINATION |  | 3 | 0.73 | 1.22 | 0.263 | 0.684 | 1.000 | 2560 | tags=67%, list=21%, signal=85% |
| 1634 | GOBP\_POSITIVE\_REGULATION\_OF\_EPITHELIAL\_CELL\_PROLIFERATION\_INVOLVED\_IN\_LUNG\_MORPHOGENESIS |  | 4 | 0.68 | 1.22 | 0.242 | 0.684 | 1.000 | 1800 | tags=50%, list=15%, signal=59% |
| 1635 | GOCC\_SIGNAL\_RECOGNITION\_PARTICLE |  | 7 | 0.58 | 1.22 | 0.235 | 0.684 | 1.000 | 179 | tags=14%, list=1%, signal=14% |
| 1636 | GOBP\_REGULATION\_OF\_BLOOD\_VOLUME\_BY\_RENIN\_ANGIOTENSIN |  | 5 | 0.63 | 1.22 | 0.261 | 0.686 | 1.000 | 1015 | tags=40%, list=8%, signal=44% |
| 1637 | GOBP\_HEMATOPOIETIC\_STEM\_CELL\_MIGRATION |  | 7 | 0.58 | 1.22 | 0.248 | 0.685 | 1.000 | 604 | tags=29%, list=5%, signal=30% |
| 1638 | GOCC\_DENDRITIC\_SPINE\_MEMBRANE |  | 6 | 0.60 | 1.22 | 0.244 | 0.685 | 1.000 | 1355 | tags=33%, list=11%, signal=38% |
| 1639 | GOBP\_IMMUNE\_EFFECTOR\_PROCESS |  | 721 | 0.25 | 1.22 | 0.023 | 0.685 | 1.000 | 1341 | tags=13%, list=11%, signal=14% |
| 1640 | GOBP\_REGULATION\_OF\_RHO\_PROTEIN\_SIGNAL\_TRANSDUCTION |  | 62 | 0.34 | 1.22 | 0.154 | 0.684 | 1.000 | 2188 | tags=26%, list=18%, signal=31% |
| 1641 | GOBP\_DNA\_METHYLATION\_ON\_CYTOSINE |  | 3 | 0.73 | 1.22 | 0.240 | 0.685 | 1.000 | 228 | tags=33%, list=2%, signal=34% |
| 1642 | GOMF\_PHOSPHATIDYLINOSITOL\_BISPHOSPHATE\_KINASE\_ACTIVITY |  | 7 | 0.57 | 1.22 | 0.249 | 0.685 | 1.000 | 1564 | tags=43%, list=13%, signal=49% |
| 1643 | GOCC\_MPP7\_DLG1\_LIN7\_COMPLEX |  | 4 | 0.67 | 1.22 | 0.253 | 0.686 | 1.000 | 566 | tags=50%, list=5%, signal=52% |
| 1644 | GOBP\_TOLL\_LIKE\_RECEPTOR\_3\_SIGNALING\_PATHWAY |  | 16 | 0.45 | 1.22 | 0.217 | 0.685 | 1.000 | 3000 | tags=38%, list=25%, signal=50% |
| 1645 | GOBP\_REGULATION\_OF\_TYPE\_2\_IMMUNE\_RESPONSE |  | 19 | 0.44 | 1.22 | 0.215 | 0.685 | 1.000 | 1198 | tags=26%, list=10%, signal=29% |
| 1646 | GOBP\_NEGATIVE\_REGULATION\_OF\_FATTY\_ACID\_OXIDATION |  | 10 | 0.52 | 1.22 | 0.225 | 0.685 | 1.000 | 1873 | tags=40%, list=16%, signal=47% |
| 1647 | GOMF\_METALLOEXOPEPTIDASE\_ACTIVITY |  | 42 | 0.36 | 1.22 | 0.185 | 0.685 | 1.000 | 1547 | tags=26%, list=13%, signal=30% |
| 1648 | GOMF\_CAMP\_DEPENDENT\_PROTEIN\_KINASE\_REGULATOR\_ACTIVITY |  | 9 | 0.54 | 1.22 | 0.238 | 0.685 | 1.000 | 925 | tags=22%, list=8%, signal=24% |
| 1649 | GOBP\_MONOCYTE\_CHEMOTAXIS |  | 34 | 0.38 | 1.22 | 0.182 | 0.686 | 1.000 | 2113 | tags=41%, list=18%, signal=50% |
| 1650 | GOCC\_ENDOPLASMIC\_RETICULUM\_LUMEN |  | 214 | 0.28 | 1.22 | 0.091 | 0.686 | 1.000 | 3109 | tags=39%, list=26%, signal=51% |
| 1651 | GOMF\_ANGIOTENSIN\_RECEPTOR\_BINDING |  | 4 | 0.67 | 1.22 | 0.253 | 0.686 | 1.000 | 2845 | tags=75%, list=24%, signal=98% |
| 1652 | GOMF\_PHOSPHODIESTERASE\_I\_ACTIVITY |  | 3 | 0.72 | 1.22 | 0.241 | 0.686 | 1.000 | 495 | tags=33%, list=4%, signal=35% |
| 1653 | GOCC\_JUNCTIONAL\_MEMBRANE\_COMPLEX |  | 3 | 0.73 | 1.22 | 0.248 | 0.688 | 1.000 | 776 | tags=33%, list=6%, signal=36% |
| 1654 | GOBP\_REGULATION\_OF\_TRIGLYCERIDE\_CATABOLIC\_PROCESS |  | 8 | 0.55 | 1.22 | 0.244 | 0.688 | 1.000 | 733 | tags=38%, list=6%, signal=40% |
| 1655 | GOBP\_KETONE\_BODY\_BIOSYNTHETIC\_PROCESS |  | 6 | 0.59 | 1.22 | 0.254 | 0.688 | 1.000 | 1933 | tags=50%, list=16%, signal=60% |
| 1656 | GOBP\_REGULATION\_OF\_CELLULAR\_KETONE\_METABOLIC\_PROCESS |  | 132 | 0.30 | 1.22 | 0.112 | 0.688 | 1.000 | 1559 | tags=20%, list=13%, signal=23% |
| 1657 | GOBP\_IODIDE\_TRANSPORT |  | 3 | 0.73 | 1.22 | 0.250 | 0.688 | 1.000 | 393 | tags=33%, list=3%, signal=34% |
| 1658 | GOBP\_CELLULAR\_RESPONSE\_TO\_REACTIVE\_OXYGEN\_SPECIES |  | 112 | 0.31 | 1.22 | 0.124 | 0.688 | 1.000 | 1718 | tags=24%, list=14%, signal=28% |
| 1659 | GOBP\_MEMBRANE\_LIPID\_CATABOLIC\_PROCESS |  | 26 | 0.41 | 1.22 | 0.198 | 0.690 | 1.000 | 626 | tags=19%, list=5%, signal=20% |
| 1660 | GOBP\_ESTABLISHMENT\_OF\_SPINDLE\_ORIENTATION |  | 30 | 0.39 | 1.22 | 0.187 | 0.690 | 1.000 | 3030 | tags=47%, list=25%, signal=62% |
| 1661 | GOBP\_CELLULAR\_RESPONSE\_TO\_WATER\_STIMULUS |  | 3 | 0.71 | 1.22 | 0.251 | 0.690 | 1.000 | 515 | tags=33%, list=4%, signal=35% |
| 1662 | GOBP\_NEGATIVE\_REGULATION\_OF\_VASCULATURE\_DEVELOPMENT |  | 63 | 0.34 | 1.22 | 0.155 | 0.689 | 1.000 | 3917 | tags=49%, list=33%, signal=73% |
| 1663 | GOBP\_RESPONSE\_TO\_PEPTIDE |  | 366 | 0.27 | 1.22 | 0.060 | 0.689 | 1.000 | 2013 | tags=22%, list=17%, signal=25% |
| 1664 | GOCC\_MICROTUBULE\_MINUS\_END |  | 5 | 0.63 | 1.22 | 0.243 | 0.691 | 1.000 | 4225 | tags=80%, list=35%, signal=123% |
| 1665 | GOBP\_PURINE\_RIBONUCLEOSIDE\_MONOPHOSPHATE\_CATABOLIC\_PROCESS |  | 4 | 0.66 | 1.22 | 0.254 | 0.691 | 1.000 | 1455 | tags=50%, list=12%, signal=57% |
| 1666 | GOBP\_SIGNAL\_TRANSDUCTION\_INVOLVED\_IN\_CELL\_CYCLE\_CHECKPOINT |  | 57 | 0.34 | 1.22 | 0.172 | 0.691 | 1.000 | 3796 | tags=42%, list=32%, signal=61% |
| 1667 | GOBP\_CLUSTERING\_OF\_VOLTAGE\_GATED\_SODIUM\_CHANNELS |  | 4 | 0.67 | 1.22 | 0.263 | 0.691 | 1.000 | 3367 | tags=75%, list=28%, signal=104% |
| 1668 | GOBP\_FATTY\_ACID\_HOMEOSTASIS |  | 9 | 0.53 | 1.22 | 0.243 | 0.691 | 1.000 | 2506 | tags=44%, list=21%, signal=56% |
| 1669 | GOBP\_BONE\_MORPHOGENESIS |  | 58 | 0.34 | 1.22 | 0.163 | 0.691 | 1.000 | 2545 | tags=31%, list=21%, signal=39% |
| 1670 | GOBP\_NEGATIVE\_REGULATION\_OF\_RESPONSE\_TO\_INTERFERON\_GAMMA |  | 4 | 0.67 | 1.22 | 0.258 | 0.691 | 1.000 | 191 | tags=25%, list=2%, signal=25% |
| 1671 | GOBP\_HISTONE\_PHOSPHORYLATION |  | 29 | 0.39 | 1.22 | 0.207 | 0.692 | 1.000 | 2652 | tags=41%, list=22%, signal=53% |
| 1672 | GOBP\_T\_CELL\_MIGRATION |  | 39 | 0.37 | 1.22 | 0.187 | 0.692 | 1.000 | 1919 | tags=28%, list=16%, signal=33% |
| 1673 | GOBP\_REGULATION\_OF\_LENS\_FIBER\_CELL\_DIFFERENTIATION |  | 4 | 0.67 | 1.22 | 0.254 | 0.692 | 1.000 | 1013 | tags=25%, list=8%, signal=27% |
| 1674 | GOBP\_POSITIVE\_REGULATION\_OF\_CYTOSKELETON\_ORGANIZATION |  | 162 | 0.29 | 1.22 | 0.102 | 0.692 | 1.000 | 3335 | tags=39%, list=28%, signal=53% |
| 1675 | GOBP\_SENSORY\_SYSTEM\_DEVELOPMENT |  | 222 | 0.28 | 1.22 | 0.087 | 0.692 | 1.000 | 2843 | tags=31%, list=24%, signal=39% |
| 1676 | GOBP\_CARBOXYLIC\_ACID\_TRANSPORT |  | 174 | 0.29 | 1.22 | 0.094 | 0.692 | 1.000 | 1429 | tags=22%, list=12%, signal=24% |
| 1677 | GOBP\_PROTEIN\_LOCALIZATION\_TO\_CHROMOSOME\_CENTROMERIC\_REGION |  | 22 | 0.42 | 1.22 | 0.207 | 0.692 | 1.000 | 4242 | tags=64%, list=35%, signal=98% |
| 1678 | GOCC\_CHROMATIN |  | 666 | 0.25 | 1.22 | 0.026 | 0.691 | 1.000 | 1882 | tags=20%, list=16%, signal=22% |
| 1679 | GOBP\_REGULATION\_OF\_TOLL\_LIKE\_RECEPTOR\_7\_SIGNALING\_PATHWAY |  | 4 | 0.67 | 1.22 | 0.257 | 0.692 | 1.000 | 220 | tags=25%, list=2%, signal=25% |
| 1680 | GOCC\_SPINDLE\_MIDZONE |  | 30 | 0.39 | 1.22 | 0.203 | 0.692 | 1.000 | 2519 | tags=37%, list=21%, signal=46% |
| 1681 | GOBP\_REGULATION\_OF\_THE\_FORCE\_OF\_HEART\_CONTRACTION\_BY\_CHEMICAL\_SIGNAL |  | 3 | 0.73 | 1.22 | 0.252 | 0.693 | 1.000 | 2393 | tags=67%, list=20%, signal=83% |
| 1682 | GOBP\_REGULATION\_OF\_TRANSPOSITION |  | 7 | 0.57 | 1.21 | 0.245 | 0.693 | 1.000 | 1322 | tags=29%, list=11%, signal=32% |
| 1683 | GOBP\_T\_CELL\_LINEAGE\_COMMITMENT |  | 15 | 0.46 | 1.21 | 0.225 | 0.694 | 1.000 | 1632 | tags=33%, list=14%, signal=39% |
| 1684 | GOBP\_REGULATION\_OF\_MICROTUBULE\_BASED\_PROCESS |  | 158 | 0.29 | 1.21 | 0.109 | 0.694 | 1.000 | 3542 | tags=37%, list=29%, signal=52% |
| 1685 | GOBP\_ALPHA\_BETA\_T\_CELL\_PROLIFERATION |  | 24 | 0.41 | 1.21 | 0.213 | 0.695 | 1.000 | 1198 | tags=25%, list=10%, signal=28% |
| 1686 | GOBP\_PEPTIDYL\_ARGININE\_METHYLATION |  | 8 | 0.55 | 1.21 | 0.242 | 0.694 | 1.000 | 1309 | tags=25%, list=11%, signal=28% |
| 1687 | GOBP\_NEURAL\_NUCLEUS\_DEVELOPMENT |  | 39 | 0.37 | 1.21 | 0.183 | 0.695 | 1.000 | 2375 | tags=31%, list=20%, signal=38% |
| 1688 | GOBP\_BUNDLE\_OF\_HIS\_CELL\_TO\_PURKINJE\_MYOCYTE\_COMMUNICATION |  | 8 | 0.55 | 1.21 | 0.241 | 0.695 | 1.000 | 985 | tags=38%, list=8%, signal=41% |
| 1689 | GOCC\_SUMO\_LIGASE\_COMPLEX |  | 6 | 0.60 | 1.21 | 0.253 | 0.695 | 1.000 | 4510 | tags=67%, list=38%, signal=107% |
| 1690 | GOCC\_CONDENSED\_NUCLEAR\_CHROMOSOME |  | 54 | 0.34 | 1.21 | 0.179 | 0.695 | 1.000 | 3700 | tags=44%, list=31%, signal=64% |
| 1691 | GOBP\_MITOTIC\_CELL\_CYCLE\_ARREST |  | 13 | 0.48 | 1.21 | 0.227 | 0.696 | 1.000 | 2808 | tags=38%, list=23%, signal=50% |
| 1692 | GOBP\_REGULATION\_OF\_SPINDLE\_ASSEMBLY |  | 21 | 0.42 | 1.21 | 0.222 | 0.696 | 1.000 | 3803 | tags=48%, list=32%, signal=70% |
| 1693 | GOBP\_POSITIVE\_REGULATION\_OF\_CYSTEINE\_TYPE\_ENDOPEPTIDASE\_ACTIVITY |  | 100 | 0.31 | 1.21 | 0.140 | 0.696 | 1.000 | 1323 | tags=20%, list=11%, signal=22% |
| 1694 | GOMF\_PROCOLLAGEN\_PROLINE\_DIOXYGENASE\_ACTIVITY |  | 8 | 0.54 | 1.21 | 0.241 | 0.697 | 1.000 | 2003 | tags=63%, list=17%, signal=75% |
| 1695 | GOBP\_PROTEIN\_DEPOLYMERIZATION |  | 81 | 0.32 | 1.21 | 0.143 | 0.697 | 1.000 | 2201 | tags=27%, list=18%, signal=33% |
| 1696 | GOBP\_CARDIAC\_MUSCLE\_CELL\_CONTRACTION |  | 47 | 0.36 | 1.21 | 0.179 | 0.696 | 1.000 | 987 | tags=21%, list=8%, signal=23% |
| 1697 | GOBP\_NEGATIVE\_REGULATION\_OF\_FATTY\_ACID\_TRANSPORT |  | 7 | 0.57 | 1.21 | 0.252 | 0.696 | 1.000 | 755 | tags=29%, list=6%, signal=30% |
| 1698 | GOMF\_GLYCINE\_BINDING |  | 5 | 0.63 | 1.21 | 0.272 | 0.697 | 1.000 | 1138 | tags=40%, list=9%, signal=44% |
| 1699 | GOMF\_DEOXYRIBONUCLEOTIDE\_BINDING |  | 3 | 0.73 | 1.21 | 0.249 | 0.697 | 1.000 | 170 | tags=33%, list=1%, signal=34% |
| 1700 | GOCC\_SORTING\_ENDOSOME |  | 5 | 0.63 | 1.21 | 0.264 | 0.697 | 1.000 | 4467 | tags=100%, list=37%, signal=159% |
| 1701 | GOBP\_POSITIVE\_REGULATION\_OF\_FIBROBLAST\_MIGRATION |  | 14 | 0.47 | 1.21 | 0.230 | 0.697 | 1.000 | 3061 | tags=50%, list=25%, signal=67% |
| 1702 | GOBP\_POSITIVE\_REGULATION\_OF\_GLUCOSE\_METABOLIC\_PROCESS |  | 26 | 0.40 | 1.21 | 0.222 | 0.697 | 1.000 | 1415 | tags=31%, list=12%, signal=35% |
| 1703 | GOCC\_PLATELET\_ALPHA\_GRANULE\_LUMEN |  | 41 | 0.36 | 1.21 | 0.190 | 0.698 | 1.000 | 4260 | tags=56%, list=35%, signal=87% |
| 1704 | GOBP\_MODIFICATION\_OF\_POSTSYNAPTIC\_ACTIN\_CYTOSKELETON |  | 8 | 0.55 | 1.21 | 0.253 | 0.698 | 1.000 | 2722 | tags=50%, list=23%, signal=65% |
| 1705 | GOCC\_ZYMOGEN\_GRANULE\_MEMBRANE |  | 6 | 0.60 | 1.21 | 0.267 | 0.698 | 1.000 | 1237 | tags=33%, list=10%, signal=37% |
| 1706 | GOMF\_FLIPPASE\_ACTIVITY |  | 13 | 0.48 | 1.21 | 0.234 | 0.698 | 1.000 | 786 | tags=23%, list=7%, signal=25% |
| 1707 | GOCC\_CELL\_TRAILING\_EDGE |  | 8 | 0.54 | 1.21 | 0.241 | 0.698 | 1.000 | 2102 | tags=38%, list=18%, signal=45% |
| 1708 | GOBP\_TRANSPOSITION |  | 7 | 0.57 | 1.21 | 0.249 | 0.697 | 1.000 | 1322 | tags=29%, list=11%, signal=32% |
| 1709 | GOBP\_REGULATION\_OF\_AUTOPHAGOSOME\_MATURATION |  | 11 | 0.50 | 1.21 | 0.239 | 0.697 | 1.000 | 992 | tags=18%, list=8%, signal=20% |
| 1710 | GOCC\_CLUSTER\_OF\_ACTIN\_BASED\_CELL\_PROJECTIONS |  | 98 | 0.31 | 1.21 | 0.146 | 0.697 | 1.000 | 1986 | tags=30%, list=17%, signal=35% |
| 1711 | GOBP\_VIRAL\_BUDDING\_VIA\_HOST\_ESCRT\_COMPLEX |  | 19 | 0.44 | 1.21 | 0.228 | 0.697 | 1.000 | 3335 | tags=47%, list=28%, signal=65% |
| 1712 | GOMF\_PROTEIN\_MACROMOLECULE\_ADAPTOR\_ACTIVITY |  | 199 | 0.28 | 1.21 | 0.100 | 0.697 | 1.000 | 2733 | tags=27%, list=23%, signal=34% |
| 1713 | GOBP\_RESPONSE\_TO\_TUMOR\_NECROSIS\_FACTOR |  | 201 | 0.28 | 1.21 | 0.098 | 0.697 | 1.000 | 2727 | tags=28%, list=23%, signal=36% |
| 1714 | GOBP\_HARD\_PALATE\_DEVELOPMENT |  | 4 | 0.66 | 1.21 | 0.275 | 0.696 | 1.000 | 480 | tags=25%, list=4%, signal=26% |
| 1715 | GOBP\_REGULATION\_OF\_CELL\_PROJECTION\_ASSEMBLY |  | 134 | 0.30 | 1.21 | 0.124 | 0.697 | 1.000 | 2722 | tags=31%, list=23%, signal=39% |
| 1716 | GOBP\_REPLICATION\_FORK\_PROTECTION |  | 7 | 0.57 | 1.21 | 0.256 | 0.697 | 1.000 | 1404 | tags=43%, list=12%, signal=49% |
| 1717 | GOCC\_RECYCLING\_ENDOSOME\_MEMBRANE |  | 63 | 0.34 | 1.21 | 0.164 | 0.697 | 1.000 | 2023 | tags=33%, list=17%, signal=40% |
| 1718 | GOBP\_UMP\_BIOSYNTHETIC\_PROCESS |  | 10 | 0.52 | 1.21 | 0.249 | 0.697 | 1.000 | 6 | tags=10%, list=0%, signal=10% |
| 1719 | GOBP\_RESPONSE\_TO\_CAMP |  | 56 | 0.34 | 1.21 | 0.170 | 0.697 | 1.000 | 2167 | tags=32%, list=18%, signal=39% |
| 1720 | GOBP\_LAMELLIPODIUM\_ORGANIZATION |  | 68 | 0.33 | 1.21 | 0.161 | 0.696 | 1.000 | 2722 | tags=34%, list=23%, signal=43% |
| 1721 | GOBP\_POSITIVE\_REGULATION\_OF\_GLYCOGEN\_STARCH\_SYNTHASE\_ACTIVITY |  | 3 | 0.72 | 1.21 | 0.262 | 0.697 | 1.000 | 2680 | tags=67%, list=22%, signal=86% |
| 1722 | GOBP\_POSITIVE\_REGULATION\_OF\_STEM\_CELL\_PROLIFERATION |  | 15 | 0.46 | 1.21 | 0.229 | 0.696 | 1.000 | 3 | tags=7%, list=0%, signal=7% |
| 1723 | GOMF\_OXIDOREDUCTASE\_ACTIVITY\_ACTING\_ON\_THE\_CH\_NH2\_GROUP\_OF\_DONORS |  | 17 | 0.44 | 1.21 | 0.227 | 0.696 | 1.000 | 2504 | tags=35%, list=21%, signal=45% |
| 1724 | GOBP\_CELLULAR\_RESPONSE\_TO\_ANTIBIOTIC |  | 10 | 0.51 | 1.21 | 0.235 | 0.697 | 1.000 | 2264 | tags=50%, list=19%, signal=62% |
| 1725 | GOBP\_REGULATION\_OF\_NOTCH\_SIGNALING\_PATHWAY |  | 75 | 0.32 | 1.21 | 0.156 | 0.696 | 1.000 | 2274 | tags=27%, list=19%, signal=33% |
| 1726 | GOBP\_SIGNAL\_COMPLEX\_ASSEMBLY |  | 5 | 0.62 | 1.21 | 0.267 | 0.696 | 1.000 | 2340 | tags=40%, list=19%, signal=50% |
| 1727 | GOBP\_ENDOCARDIUM\_DEVELOPMENT |  | 9 | 0.53 | 1.21 | 0.244 | 0.696 | 1.000 | 1715 | tags=33%, list=14%, signal=39% |
| 1728 | GOBP\_REGULATION\_OF\_RRNA\_PROCESSING |  | 10 | 0.51 | 1.21 | 0.236 | 0.697 | 1.000 | 1322 | tags=30%, list=11%, signal=34% |
| 1729 | GOBP\_CORNEA\_DEVELOPMENT\_IN\_CAMERA\_TYPE\_EYE |  | 3 | 0.71 | 1.21 | 0.256 | 0.696 | 1.000 | 856 | tags=33%, list=7%, signal=36% |
| 1730 | GOMF\_PROTEIN\_KINASE\_C\_ACTIVITY |  | 11 | 0.50 | 1.21 | 0.240 | 0.696 | 1.000 | 1693 | tags=36%, list=14%, signal=42% |
| 1731 | GOBP\_REGULATION\_OF\_IMMUNE\_SYSTEM\_PROCESS |  | 860 | 0.25 | 1.21 | 0.023 | 0.696 | 1.000 | 1476 | tags=15%, list=12%, signal=15% |
| 1732 | GOBP\_ORGANIC\_ACID\_BIOSYNTHETIC\_PROCESS |  | 217 | 0.28 | 1.21 | 0.099 | 0.696 | 1.000 | 1686 | tags=21%, list=14%, signal=24% |
| 1733 | GOMF\_ACETYLGLUCOSAMINYLTRANSFERASE\_ACTIVITY |  | 39 | 0.37 | 1.21 | 0.197 | 0.696 | 1.000 | 474 | tags=13%, list=4%, signal=13% |
| 1734 | GOCC\_PRESYNAPTIC\_ACTIVE\_ZONE\_MEMBRANE |  | 18 | 0.44 | 1.21 | 0.224 | 0.696 | 1.000 | 382 | tags=17%, list=3%, signal=17% |
| 1735 | GOMF\_PHOSPHATIDIC\_ACID\_BINDING |  | 17 | 0.44 | 1.21 | 0.217 | 0.696 | 1.000 | 1579 | tags=18%, list=13%, signal=20% |
| 1736 | GOBP\_REGULATION\_BY\_VIRUS\_OF\_VIRAL\_PROTEIN\_LEVELS\_IN\_HOST\_CELL |  | 6 | 0.59 | 1.21 | 0.263 | 0.697 | 1.000 | 78 | tags=17%, list=1%, signal=17% |
| 1737 | GOBP\_NEGATIVE\_REGULATION\_OF\_POTASSIUM\_ION\_TRANSPORT |  | 20 | 0.43 | 1.21 | 0.222 | 0.697 | 1.000 | 1967 | tags=35%, list=16%, signal=42% |
| 1738 | GOMF\_RNA\_DNA\_HYBRID\_RIBONUCLEASE\_ACTIVITY |  | 3 | 0.72 | 1.21 | 0.250 | 0.697 | 1.000 | 2781 | tags=67%, list=23%, signal=87% |
| 1739 | GOBP\_PENTOSE\_CATABOLIC\_PROCESS |  | 5 | 0.62 | 1.21 | 0.270 | 0.697 | 1.000 | 106 | tags=20%, list=1%, signal=20% |
| 1740 | GOBP\_DENDRITIC\_CELL\_APOPTOTIC\_PROCESS |  | 8 | 0.55 | 1.21 | 0.247 | 0.697 | 1.000 | 1517 | tags=38%, list=13%, signal=43% |
| 1741 | GOBP\_RESPONSE\_TO\_INTERLEUKIN\_1 |  | 136 | 0.29 | 1.21 | 0.121 | 0.697 | 1.000 | 1569 | tags=18%, list=13%, signal=21% |
| 1742 | GOBP\_POSITIVE\_REGULATION\_OF\_ACTIN\_NUCLEATION |  | 12 | 0.49 | 1.21 | 0.244 | 0.696 | 1.000 | 4393 | tags=83%, list=37%, signal=131% |
| 1743 | GOMF\_MONOOXYGENASE\_ACTIVITY |  | 44 | 0.36 | 1.21 | 0.194 | 0.697 | 1.000 | 392 | tags=14%, list=3%, signal=14% |
| 1744 | GOBP\_REGULATION\_OF\_RESPONSE\_TO\_DNA\_DAMAGE\_STIMULUS |  | 160 | 0.29 | 1.21 | 0.115 | 0.696 | 1.000 | 3609 | tags=35%, list=30%, signal=49% |
| 1745 | GOBP\_LIPOPROTEIN\_LOCALIZATION |  | 12 | 0.49 | 1.21 | 0.239 | 0.696 | 1.000 | 2049 | tags=50%, list=17%, signal=60% |
| 1746 | GOMF\_MRNA\_METHYLTRANSFERASE\_ACTIVITY |  | 12 | 0.48 | 1.21 | 0.239 | 0.696 | 1.000 | 834 | tags=17%, list=7%, signal=18% |
| 1747 | GOMF\_O\_ACYLTRANSFERASE\_ACTIVITY |  | 37 | 0.37 | 1.21 | 0.187 | 0.696 | 1.000 | 1925 | tags=38%, list=16%, signal=45% |
| 1748 | GOBP\_DNA\_PACKAGING |  | 117 | 0.30 | 1.21 | 0.135 | 0.696 | 1.000 | 3463 | tags=40%, list=29%, signal=56% |
| 1749 | GOBP\_CARDIAC\_VENTRICLE\_DEVELOPMENT |  | 80 | 0.32 | 1.21 | 0.159 | 0.696 | 1.000 | 2013 | tags=20%, list=17%, signal=24% |
| 1750 | GOBP\_GROWTH\_PLATE\_CARTILAGE\_DEVELOPMENT |  | 9 | 0.53 | 1.21 | 0.255 | 0.696 | 1.000 | 1738 | tags=33%, list=14%, signal=39% |
| 1751 | GOBP\_RAP\_PROTEIN\_SIGNAL\_TRANSDUCTION |  | 13 | 0.48 | 1.21 | 0.234 | 0.695 | 1.000 | 2487 | tags=31%, list=21%, signal=39% |
| 1752 | GOBP\_10\_FORMYLTETRAHYDROFOLATE\_METABOLIC\_PROCESS |  | 4 | 0.66 | 1.21 | 0.275 | 0.695 | 1.000 | 2152 | tags=25%, list=18%, signal=30% |
| 1753 | GOBP\_NEGATIVE\_REGULATION\_OF\_MUSCLE\_ADAPTATION |  | 9 | 0.53 | 1.20 | 0.243 | 0.696 | 1.000 | 969 | tags=33%, list=8%, signal=36% |
| 1754 | GOBP\_SIGNAL\_TRANSDUCTION\_IN\_ABSENCE\_OF\_LIGAND |  | 44 | 0.36 | 1.20 | 0.184 | 0.696 | 1.000 | 2274 | tags=25%, list=19%, signal=31% |
| 1755 | GOBP\_GANGLIOSIDE\_BIOSYNTHETIC\_PROCESS |  | 11 | 0.50 | 1.20 | 0.248 | 0.697 | 1.000 | 153 | tags=18%, list=1%, signal=18% |
| 1756 | GOBP\_MAINTENANCE\_OF\_LOCATION |  | 208 | 0.28 | 1.20 | 0.103 | 0.696 | 1.000 | 2190 | tags=25%, list=18%, signal=29% |
| 1757 | GOBP\_FUCOSE\_METABOLIC\_PROCESS |  | 11 | 0.50 | 1.20 | 0.248 | 0.696 | 1.000 | 680 | tags=18%, list=6%, signal=19% |
| 1758 | GOBP\_REGULATION\_OF\_METAL\_ION\_TRANSPORT |  | 149 | 0.29 | 1.20 | 0.123 | 0.696 | 1.000 | 2536 | tags=29%, list=21%, signal=36% |
| 1759 | GOBP\_GLYCOSYLATION |  | 184 | 0.28 | 1.20 | 0.114 | 0.696 | 1.000 | 2835 | tags=27%, list=24%, signal=35% |
| 1760 | GOBP\_POSITIVE\_REGULATION\_OF\_PROTEIN\_MODIFICATION\_PROCESS |  | 727 | 0.25 | 1.20 | 0.032 | 0.698 | 1.000 | 2168 | tags=21%, list=18%, signal=24% |
| 1761 | GOBP\_CELL\_SUBSTRATE\_ADHESION |  | 259 | 0.27 | 1.20 | 0.094 | 0.698 | 1.000 | 2672 | tags=29%, list=22%, signal=36% |
| 1762 | GOBP\_RESPONSE\_TO\_LIPID |  | 545 | 0.26 | 1.20 | 0.045 | 0.698 | 1.000 | 1421 | tags=17%, list=12%, signal=18% |
| 1763 | GOBP\_REGULATION\_OF\_CARDIAC\_MUSCLE\_CONTRACTION |  | 47 | 0.35 | 1.20 | 0.188 | 0.698 | 1.000 | 1272 | tags=23%, list=11%, signal=26% |
| 1764 | GOBP\_APOPTOTIC\_SIGNALING\_PATHWAY |  | 400 | 0.26 | 1.20 | 0.063 | 0.699 | 1.000 | 2842 | tags=28%, list=24%, signal=35% |
| 1765 | GOBP\_REGULATION\_OF\_PROTEIN\_POLYUBIQUITINATION |  | 13 | 0.48 | 1.20 | 0.253 | 0.699 | 1.000 | 1659 | tags=23%, list=14%, signal=27% |
| 1766 | GOMF\_Y\_FORM\_DNA\_BINDING |  | 4 | 0.66 | 1.20 | 0.268 | 0.699 | 1.000 | 1690 | tags=25%, list=14%, signal=29% |
| 1767 | GOBP\_ADAPTIVE\_THERMOGENESIS |  | 108 | 0.30 | 1.20 | 0.141 | 0.699 | 1.000 | 1967 | tags=29%, list=16%, signal=34% |
| 1768 | GOBP\_MITOTIC\_G1\_S\_TRANSITION\_CHECKPOINT |  | 48 | 0.35 | 1.20 | 0.183 | 0.699 | 1.000 | 3466 | tags=38%, list=29%, signal=53% |
| 1769 | GOBP\_RESPONSE\_TO\_FLUID\_SHEAR\_STRESS |  | 26 | 0.40 | 1.20 | 0.216 | 0.699 | 1.000 | 1140 | tags=19%, list=9%, signal=21% |
| 1770 | GOBP\_POSITIVE\_REGULATION\_OF\_VIRAL\_RELEASE\_FROM\_HOST\_CELL |  | 13 | 0.48 | 1.20 | 0.244 | 0.699 | 1.000 | 1449 | tags=31%, list=12%, signal=35% |
| 1771 | GOBP\_REGULATION\_OF\_ANATOMICAL\_STRUCTURE\_SIZE |  | 348 | 0.26 | 1.20 | 0.076 | 0.699 | 1.000 | 2722 | tags=31%, list=23%, signal=39% |
| 1772 | GOBP\_MITOTIC\_SISTER\_CHROMATID\_COHESION |  | 19 | 0.43 | 1.20 | 0.225 | 0.698 | 1.000 | 1611 | tags=21%, list=13%, signal=24% |
| 1773 | GOBP\_PHOTORECEPTOR\_CELL\_DEVELOPMENT |  | 20 | 0.43 | 1.20 | 0.230 | 0.698 | 1.000 | 2125 | tags=40%, list=18%, signal=49% |
| 1774 | GOBP\_CELLULAR\_CARBOHYDRATE\_METABOLIC\_PROCESS |  | 208 | 0.28 | 1.20 | 0.102 | 0.699 | 1.000 | 1427 | tags=17%, list=12%, signal=19% |
| 1775 | GOBP\_POSITIVE\_REGULATION\_OF\_TISSUE\_REMODELING |  | 6 | 0.59 | 1.20 | 0.259 | 0.699 | 1.000 | 3380 | tags=67%, list=28%, signal=93% |
| 1776 | GOBP\_DEDIFFERENTIATION |  | 5 | 0.62 | 1.20 | 0.270 | 0.699 | 1.000 | 2813 | tags=60%, list=23%, signal=78% |
| 1777 | GOBP\_EMBRYONIC\_AXIS\_SPECIFICATION |  | 15 | 0.46 | 1.20 | 0.237 | 0.699 | 1.000 | 1200 | tags=27%, list=10%, signal=30% |
| 1778 | GOBP\_NEGATIVE\_REGULATION\_OF\_CELL\_CYCLE |  | 446 | 0.26 | 1.20 | 0.057 | 0.699 | 1.000 | 3959 | tags=40%, list=33%, signal=58% |
| 1779 | GOBP\_POSITIVE\_REGULATION\_OF\_MESENCHYMAL\_CELL\_PROLIFERATION |  | 16 | 0.45 | 1.20 | 0.234 | 0.699 | 1.000 | 2288 | tags=38%, list=19%, signal=46% |
| 1780 | GOBP\_EPINEPHRINE\_TRANSPORT |  | 6 | 0.58 | 1.20 | 0.269 | 0.699 | 1.000 | 1619 | tags=33%, list=13%, signal=39% |
| 1781 | GOBP\_REGULATION\_OF\_CYSTEINE\_TYPE\_ENDOPEPTIDASE\_ACTIVITY |  | 151 | 0.29 | 1.20 | 0.119 | 0.699 | 1.000 | 1219 | tags=17%, list=10%, signal=19% |
| 1782 | GOBP\_ACTIVATED\_T\_CELL\_PROLIFERATION |  | 24 | 0.41 | 1.20 | 0.221 | 0.698 | 1.000 | 2978 | tags=50%, list=25%, signal=66% |
| 1783 | GOBP\_RESPONSE\_TO\_STARVATION |  | 152 | 0.29 | 1.20 | 0.126 | 0.698 | 1.000 | 1223 | tags=16%, list=10%, signal=17% |
| 1784 | GOMF\_MOLECULAR\_ADAPTOR\_ACTIVITY |  | 247 | 0.27 | 1.20 | 0.098 | 0.699 | 1.000 | 2733 | tags=25%, list=23%, signal=32% |
| 1785 | GOBP\_ENDOPLASMIC\_RETICULUM\_MEMBRANE\_ORGANIZATION |  | 4 | 0.67 | 1.20 | 0.270 | 0.699 | 1.000 | 3330 | tags=75%, list=28%, signal=104% |
| 1786 | GOBP\_ENDOCYTIC\_RECYCLING |  | 35 | 0.37 | 1.20 | 0.208 | 0.699 | 1.000 | 1778 | tags=29%, list=15%, signal=33% |
| 1787 | GOBP\_CELLULAR\_RESPONSE\_TO\_DNA\_DAMAGE\_STIMULUS |  | 640 | 0.25 | 1.20 | 0.049 | 0.699 | 1.000 | 3199 | tags=28%, list=27%, signal=36% |
| 1788 | GOMF\_K48\_LINKED\_POLYUBIQUITIN\_MODIFICATION\_DEPENDENT\_PROTEIN\_BINDING |  | 3 | 0.71 | 1.20 | 0.271 | 0.700 | 1.000 | 505 | tags=33%, list=4%, signal=35% |
| 1789 | GOBP\_PROTEIN\_AUTO\_ADP\_RIBOSYLATION |  | 9 | 0.52 | 1.20 | 0.259 | 0.700 | 1.000 | 342 | tags=22%, list=3%, signal=23% |
| 1790 | GOBP\_POSITIVE\_REGULATION\_OF\_VASCULAR\_ASSOCIATED\_SMOOTH\_MUSCLE\_CELL\_MIGRATION |  | 12 | 0.49 | 1.20 | 0.240 | 0.699 | 1.000 | 2049 | tags=42%, list=17%, signal=50% |
| 1791 | GOMF\_PEPTIDE\_RECEPTOR\_ACTIVITY |  | 73 | 0.32 | 1.20 | 0.171 | 0.700 | 1.000 | 1488 | tags=26%, list=12%, signal=30% |
| 1792 | GOBP\_SINOATRIAL\_NODE\_DEVELOPMENT |  | 3 | 0.71 | 1.20 | 0.274 | 0.700 | 1.000 | 613 | tags=67%, list=5%, signal=70% |
| 1793 | GOBP\_MONOVALENT\_INORGANIC\_ANION\_HOMEOSTASIS |  | 9 | 0.52 | 1.20 | 0.253 | 0.699 | 1.000 | 1150 | tags=33%, list=10%, signal=37% |
| 1794 | GOBP\_RUFFLE\_ASSEMBLY |  | 28 | 0.39 | 1.20 | 0.217 | 0.701 | 1.000 | 1977 | tags=29%, list=16%, signal=34% |
| 1795 | GOBP\_REGULATION\_OF\_CELL\_CYCLE\_G2\_M\_PHASE\_TRANSITION |  | 171 | 0.28 | 1.20 | 0.126 | 0.701 | 1.000 | 4417 | tags=51%, list=37%, signal=79% |
| 1796 | GOMF\_MITOGEN\_ACTIVATED\_PROTEIN\_KINASE\_P38\_BINDING |  | 5 | 0.61 | 1.20 | 0.283 | 0.702 | 1.000 | 349 | tags=20%, list=3%, signal=21% |
| 1797 | GOMF\_ALANINE\_TRANSMEMBRANE\_TRANSPORTER\_ACTIVITY |  | 7 | 0.56 | 1.20 | 0.270 | 0.702 | 1.000 | 1743 | tags=57%, list=15%, signal=67% |
| 1798 | GOBP\_SEQUESTERING\_OF\_ACTIN\_MONOMERS |  | 6 | 0.59 | 1.20 | 0.272 | 0.701 | 1.000 | 957 | tags=33%, list=8%, signal=36% |
| 1799 | GOCC\_SPINDLE\_POLE |  | 132 | 0.29 | 1.20 | 0.140 | 0.701 | 1.000 | 4231 | tags=50%, list=35%, signal=76% |
| 1800 | GOBP\_REGULATION\_OF\_CELLULAR\_COMPONENT\_MOVEMENT |  | 701 | 0.25 | 1.20 | 0.035 | 0.702 | 1.000 | 2684 | tags=27%, list=22%, signal=33% |
| 1801 | GOBP\_REGULATION\_OF\_TRIGLYCERIDE\_BIOSYNTHETIC\_PROCESS |  | 14 | 0.46 | 1.20 | 0.247 | 0.702 | 1.000 | 691 | tags=29%, list=6%, signal=30% |
| 1802 | GOBP\_EMBRYONIC\_FORELIMB\_MORPHOGENESIS |  | 15 | 0.45 | 1.20 | 0.237 | 0.702 | 1.000 | 5 | tags=13%, list=0%, signal=13% |
| 1803 | GOBP\_NUCLEOBASE\_CONTAINING\_SMALL\_MOLECULE\_BIOSYNTHETIC\_PROCESS |  | 87 | 0.31 | 1.20 | 0.160 | 0.702 | 1.000 | 1288 | tags=17%, list=11%, signal=19% |
| 1804 | GOBP\_REGULATION\_OF\_NEUROTRANSMITTER\_RECEPTOR\_ACTIVITY |  | 31 | 0.38 | 1.20 | 0.209 | 0.703 | 1.000 | 2842 | tags=42%, list=24%, signal=55% |
| 1805 | GOBP\_POSITIVE\_REGULATION\_OF\_CELLULAR\_COMPONENT\_BIOGENESIS |  | 369 | 0.26 | 1.20 | 0.076 | 0.702 | 1.000 | 3380 | tags=36%, list=28%, signal=48% |
| 1806 | GOBP\_POSITIVE\_REGULATION\_OF\_CHROMOSOME\_SEGREGATION |  | 13 | 0.47 | 1.20 | 0.256 | 0.702 | 1.000 | 3978 | tags=69%, list=33%, signal=103% |
| 1807 | GOBP\_TYPE\_I\_PNEUMOCYTE\_DIFFERENTIATION |  | 4 | 0.66 | 1.20 | 0.283 | 0.702 | 1.000 | 3404 | tags=75%, list=28%, signal=105% |
| 1808 | GOMF\_ION\_CHANNEL\_BINDING |  | 93 | 0.31 | 1.20 | 0.163 | 0.702 | 1.000 | 2190 | tags=25%, list=18%, signal=30% |
| 1809 | GOBP\_CALCIUM\_ION\_EXPORT |  | 6 | 0.59 | 1.20 | 0.279 | 0.703 | 1.000 | 869 | tags=33%, list=7%, signal=36% |
| 1810 | GOCC\_UNIPLEX\_COMPLEX |  | 6 | 0.59 | 1.20 | 0.271 | 0.703 | 1.000 | 707 | tags=33%, list=6%, signal=35% |
| 1811 | GOCC\_GOLGI\_ASSOCIATED\_VESICLE |  | 66 | 0.33 | 1.20 | 0.173 | 0.702 | 1.000 | 3372 | tags=48%, list=28%, signal=67% |
| 1812 | GOBP\_NEGATIVE\_REGULATION\_OF\_CELLULAR\_PROTEIN\_LOCALIZATION |  | 88 | 0.31 | 1.20 | 0.165 | 0.702 | 1.000 | 2190 | tags=25%, list=18%, signal=30% |
| 1813 | GOBP\_POSITIVE\_REGULATION\_OF\_NON\_MOTILE\_CILIUM\_ASSEMBLY |  | 5 | 0.62 | 1.20 | 0.277 | 0.702 | 1.000 | 1097 | tags=20%, list=9%, signal=22% |
| 1814 | GOBP\_REGULATION\_OF\_RAS\_PROTEIN\_SIGNAL\_TRANSDUCTION |  | 146 | 0.29 | 1.19 | 0.132 | 0.703 | 1.000 | 2701 | tags=30%, list=22%, signal=38% |
| 1815 | GOBP\_CARBOHYDRATE\_TRANSPORT |  | 100 | 0.31 | 1.19 | 0.159 | 0.703 | 1.000 | 2130 | tags=34%, list=18%, signal=41% |
| 1816 | GOBP\_REGULATION\_OF\_PROTEIN\_DEPOLYMERIZATION |  | 61 | 0.33 | 1.19 | 0.175 | 0.703 | 1.000 | 2090 | tags=25%, list=17%, signal=30% |
| 1817 | GOBP\_RESPONSE\_TO\_MANGANESE\_ION |  | 14 | 0.46 | 1.19 | 0.248 | 0.703 | 1.000 | 2239 | tags=36%, list=19%, signal=44% |
| 1818 | GOBP\_NEGATIVE\_REGULATION\_OF\_DNA\_DEPENDENT\_DNA\_REPLICATION |  | 14 | 0.46 | 1.19 | 0.241 | 0.703 | 1.000 | 1766 | tags=29%, list=15%, signal=33% |
| 1819 | GOBP\_SINOATRIAL\_NODE\_CELL\_DEVELOPMENT |  | 3 | 0.71 | 1.19 | 0.278 | 0.704 | 1.000 | 613 | tags=67%, list=5%, signal=70% |
| 1820 | GOBP\_REPLICATION\_BORN\_DOUBLE\_STRAND\_BREAK\_REPAIR\_VIA\_SISTER\_CHROMATID\_EXCHANGE |  | 4 | 0.66 | 1.19 | 0.280 | 0.703 | 1.000 | 1046 | tags=25%, list=9%, signal=27% |
| 1821 | GOBP\_CELLULAR\_RESPONSE\_TO\_STARVATION |  | 124 | 0.29 | 1.19 | 0.144 | 0.703 | 1.000 | 1083 | tags=15%, list=9%, signal=16% |
| 1822 | GOBP\_CAMERA\_TYPE\_EYE\_DEVELOPMENT |  | 186 | 0.28 | 1.19 | 0.119 | 0.703 | 1.000 | 2843 | tags=31%, list=24%, signal=40% |
| 1823 | GOBP\_CELLULAR\_RESPONSE\_TO\_GAMMA\_RADIATION |  | 20 | 0.42 | 1.19 | 0.239 | 0.703 | 1.000 | 2597 | tags=45%, list=22%, signal=57% |
| 1824 | GOBP\_METANEPHRIC\_NEPHRON\_DEVELOPMENT |  | 25 | 0.40 | 1.19 | 0.226 | 0.703 | 1.000 | 2683 | tags=36%, list=22%, signal=46% |
| 1825 | GOBP\_POSITIVE\_REGULATION\_OF\_BLOOD\_VESSEL\_ENDOTHELIAL\_CELL\_PROLIFERATION\_INVOLVED\_IN\_SPROUTING\_ANGIOGENESIS |  | 7 | 0.56 | 1.19 | 0.270 | 0.703 | 1.000 | 1794 | tags=29%, list=15%, signal=34% |
| 1826 | GOBP\_MEMBRANE\_TO\_MEMBRANE\_DOCKING |  | 3 | 0.71 | 1.19 | 0.281 | 0.703 | 1.000 | 3380 | tags=67%, list=28%, signal=93% |
| 1827 | GOMF\_DNA\_TRANSLOCASE\_ACTIVITY |  | 4 | 0.66 | 1.19 | 0.281 | 0.703 | 1.000 | 768 | tags=25%, list=6%, signal=27% |
| 1828 | GOBP\_PYRIMIDINE\_NUCLEOSIDE\_MONOPHOSPHATE\_BIOSYNTHETIC\_PROCESS |  | 13 | 0.47 | 1.19 | 0.256 | 0.703 | 1.000 | 904 | tags=15%, list=8%, signal=17% |
| 1829 | GOBP\_PEPTIDYL\_TYROSINE\_MODIFICATION |  | 256 | 0.27 | 1.19 | 0.097 | 0.702 | 1.000 | 2764 | tags=28%, list=23%, signal=36% |
| 1830 | GOCC\_REPLISOME |  | 19 | 0.43 | 1.19 | 0.249 | 0.702 | 1.000 | 2174 | tags=26%, list=18%, signal=32% |
| 1831 | GOCC\_T\_TUBULE |  | 32 | 0.38 | 1.19 | 0.213 | 0.702 | 1.000 | 1140 | tags=22%, list=9%, signal=24% |
| 1832 | GOBP\_REGULATION\_OF\_CELLULAR\_COMPONENT\_BIOGENESIS |  | 682 | 0.25 | 1.19 | 0.044 | 0.702 | 1.000 | 2786 | tags=27%, list=23%, signal=33% |
| 1833 | GOBP\_PERICYTE\_CELL\_DIFFERENTIATION |  | 7 | 0.56 | 1.19 | 0.265 | 0.702 | 1.000 | 2604 | tags=57%, list=22%, signal=73% |
| 1834 | GOBP\_TRANSCYTOSIS |  | 17 | 0.44 | 1.19 | 0.246 | 0.703 | 1.000 | 2154 | tags=29%, list=18%, signal=36% |
| 1835 | GOBP\_INTRA\_GOLGI\_VESICLE\_MEDIATED\_TRANSPORT |  | 29 | 0.39 | 1.19 | 0.217 | 0.703 | 1.000 | 3011 | tags=38%, list=25%, signal=50% |
| 1836 | GOCC\_RECYCLING\_ENDOSOME |  | 134 | 0.29 | 1.19 | 0.142 | 0.703 | 1.000 | 2023 | tags=26%, list=17%, signal=31% |
| 1837 | GOBP\_SEX\_DIFFERENTIATION |  | 169 | 0.28 | 1.19 | 0.129 | 0.703 | 1.000 | 3133 | tags=34%, list=26%, signal=45% |
| 1838 | GOBP\_T\_CELL\_HOMEOSTASIS |  | 23 | 0.41 | 1.19 | 0.230 | 0.703 | 1.000 | 2797 | tags=43%, list=23%, signal=57% |
| 1839 | GOBP\_REGULATION\_OF\_SENSORY\_PERCEPTION |  | 16 | 0.45 | 1.19 | 0.248 | 0.703 | 1.000 | 1771 | tags=31%, list=15%, signal=37% |
| 1840 | GOBP\_REGULATION\_OF\_EOSINOPHIL\_MIGRATION |  | 3 | 0.71 | 1.19 | 0.283 | 0.703 | 1.000 | 599 | tags=33%, list=5%, signal=35% |
| 1841 | GOBP\_CYTOPLASMIC\_MICROTUBULE\_ORGANIZATION |  | 47 | 0.35 | 1.19 | 0.209 | 0.703 | 1.000 | 4378 | tags=60%, list=36%, signal=93% |
| 1842 | GOBP\_CELLULAR\_RESPONSE\_TO\_DEXAMETHASONE\_STIMULUS |  | 24 | 0.41 | 1.19 | 0.231 | 0.703 | 1.000 | 792 | tags=21%, list=7%, signal=22% |
| 1843 | GOBP\_POSITIVE\_REGULATION\_OF\_ENDOTHELIAL\_CELL\_CHEMOTAXIS\_BY\_VEGF\_ACTIVATED\_VASCULAR\_ENDOTHELIAL\_GROWTH\_FACTOR\_RECEPTOR\_SIGNALING\_PATHWAY |  | 4 | 0.66 | 1.19 | 0.283 | 0.703 | 1.000 | 2764 | tags=75%, list=23%, signal=97% |
| 1844 | GOMF\_PHOSPHOFRUCTOKINASE\_ACTIVITY |  | 5 | 0.62 | 1.19 | 0.275 | 0.703 | 1.000 | 793 | tags=40%, list=7%, signal=43% |
| 1845 | GOBP\_POSITIVE\_REGULATION\_OF\_EXTRINSIC\_APOPTOTIC\_SIGNALING\_PATHWAY |  | 33 | 0.37 | 1.19 | 0.216 | 0.703 | 1.000 | 2131 | tags=33%, list=18%, signal=40% |
| 1846 | GOBP\_CELL\_CELL\_ADHESION\_MEDIATED\_BY\_CADHERIN |  | 24 | 0.40 | 1.19 | 0.225 | 0.703 | 1.000 | 3388 | tags=46%, list=28%, signal=64% |
| 1847 | GOBP\_REGULATION\_OF\_CENTROSOME\_CYCLE |  | 39 | 0.36 | 1.19 | 0.209 | 0.703 | 1.000 | 3969 | tags=51%, list=33%, signal=76% |
| 1848 | GOBP\_CARDIAC\_PACEMAKER\_CELL\_DIFFERENTIATION |  | 4 | 0.66 | 1.19 | 0.269 | 0.703 | 1.000 | 613 | tags=50%, list=5%, signal=53% |
| 1849 | GOMF\_MOTOR\_ACTIVITY |  | 82 | 0.32 | 1.19 | 0.162 | 0.703 | 1.000 | 2993 | tags=50%, list=25%, signal=66% |
| 1850 | GOBP\_REGULATION\_OF\_NUCLEAR\_CELL\_CYCLE\_DNA\_REPLICATION |  | 13 | 0.47 | 1.19 | 0.253 | 0.703 | 1.000 | 2394 | tags=46%, list=20%, signal=58% |
| 1851 | GOBP\_POSITIVE\_REGULATION\_OF\_VOLTAGE\_GATED\_CALCIUM\_CHANNEL\_ACTIVITY |  | 8 | 0.54 | 1.19 | 0.272 | 0.704 | 1.000 | 1778 | tags=38%, list=15%, signal=44% |
| 1852 | GOBP\_ESTABLISHMENT\_OF\_CELL\_POLARITY |  | 114 | 0.30 | 1.19 | 0.151 | 0.704 | 1.000 | 3037 | tags=37%, list=25%, signal=49% |
| 1853 | GOCC\_GAP\_JUNCTION |  | 17 | 0.43 | 1.19 | 0.235 | 0.704 | 1.000 | 2738 | tags=41%, list=23%, signal=53% |
| 1854 | GOBP\_NEGATIVE\_REGULATION\_OF\_PEPTIDASE\_ACTIVITY |  | 122 | 0.30 | 1.19 | 0.150 | 0.704 | 1.000 | 1237 | tags=17%, list=10%, signal=19% |
| 1855 | GOCC\_ARYL\_HYDROCARBON\_RECEPTOR\_COMPLEX |  | 8 | 0.54 | 1.19 | 0.268 | 0.704 | 1.000 | 1270 | tags=38%, list=11%, signal=42% |
| 1856 | GOBP\_DNA\_REPAIR |  | 416 | 0.26 | 1.19 | 0.073 | 0.704 | 1.000 | 3378 | tags=31%, list=28%, signal=42% |
| 1857 | GOBP\_REGULATION\_OF\_MEIOTIC\_NUCLEAR\_DIVISION |  | 14 | 0.46 | 1.19 | 0.245 | 0.704 | 1.000 | 3910 | tags=57%, list=33%, signal=85% |
| 1858 | GOBP\_POSITIVE\_REGULATION\_OF\_PATHWAY\_RESTRICTED\_SMAD\_PROTEIN\_PHOSPHORYLATION |  | 32 | 0.38 | 1.19 | 0.222 | 0.704 | 1.000 | 1379 | tags=25%, list=11%, signal=28% |
| 1859 | GOBP\_COENZYME\_A\_METABOLIC\_PROCESS |  | 14 | 0.46 | 1.19 | 0.250 | 0.704 | 1.000 | 299 | tags=14%, list=2%, signal=15% |
| 1860 | GOBP\_PRIMARY\_ALCOHOL\_METABOLIC\_PROCESS |  | 54 | 0.34 | 1.19 | 0.200 | 0.704 | 1.000 | 1533 | tags=31%, list=13%, signal=36% |
| 1861 | GOBP\_POST\_EMBRYONIC\_ANIMAL\_ORGAN\_MORPHOGENESIS |  | 5 | 0.61 | 1.19 | 0.276 | 0.703 | 1.000 | 4631 | tags=100%, list=39%, signal=163% |
| 1862 | GOMF\_MICROTUBULE\_MOTOR\_ACTIVITY |  | 45 | 0.35 | 1.19 | 0.201 | 0.703 | 1.000 | 2633 | tags=49%, list=22%, signal=62% |
| 1863 | GOBP\_REGULATION\_OF\_POTASSIUM\_ION\_TRANSMEMBRANE\_TRANSPORTER\_ACTIVITY |  | 34 | 0.37 | 1.19 | 0.215 | 0.703 | 1.000 | 1967 | tags=32%, list=16%, signal=39% |
| 1864 | GOBP\_REGULATION\_OF\_STRESS\_ACTIVATED\_PROTEIN\_KINASE\_SIGNALING\_CASCADE |  | 125 | 0.29 | 1.19 | 0.154 | 0.703 | 1.000 | 1809 | tags=19%, list=15%, signal=22% |
| 1865 | GOBP\_CELLULAR\_RESPONSE\_TO\_OXYGEN\_CONTAINING\_COMPOUND |  | 779 | 0.24 | 1.19 | 0.038 | 0.703 | 1.000 | 3213 | tags=34%, list=27%, signal=43% |
| 1866 | GOCC\_DENDRITIC\_SHAFT |  | 24 | 0.40 | 1.19 | 0.225 | 0.703 | 1.000 | 831 | tags=21%, list=7%, signal=22% |
| 1867 | GOBP\_RESPONSE\_TO\_METHOTREXATE |  | 3 | 0.70 | 1.19 | 0.290 | 0.703 | 1.000 | 3568 | tags=100%, list=30%, signal=142% |
| 1868 | GOBP\_NEGATIVE\_REGULATION\_OF\_PROTEIN\_POLYMERIZATION |  | 55 | 0.34 | 1.19 | 0.195 | 0.703 | 1.000 | 2090 | tags=24%, list=17%, signal=28% |
| 1869 | GOBP\_G\_PROTEIN\_COUPLED\_RECEPTOR\_SIGNALING\_PATHWAY |  | 483 | 0.25 | 1.19 | 0.070 | 0.703 | 1.000 | 2083 | tags=24%, list=17%, signal=28% |
| 1870 | GOBP\_BIOLOGICAL\_ADHESION |  | 902 | 0.24 | 1.19 | 0.029 | 0.702 | 1.000 | 2691 | tags=27%, list=22%, signal=32% |
| 1871 | GOBP\_MRNA\_METHYLATION |  | 15 | 0.45 | 1.19 | 0.247 | 0.702 | 1.000 | 834 | tags=13%, list=7%, signal=14% |
| 1872 | GOMF\_AMINOPHOSPHOLIPID\_FLIPPASE\_ACTIVITY |  | 6 | 0.58 | 1.19 | 0.284 | 0.702 | 1.000 | 786 | tags=33%, list=7%, signal=36% |
| 1873 | GOBP\_FEEDING\_BEHAVIOR |  | 43 | 0.35 | 1.19 | 0.203 | 0.702 | 1.000 | 1771 | tags=26%, list=15%, signal=30% |
| 1874 | GOMF\_SEQUENCE\_SPECIFIC\_DNA\_BINDING |  | 728 | 0.25 | 1.19 | 0.043 | 0.702 | 1.000 | 1893 | tags=19%, list=16%, signal=21% |
| 1875 | GOBP\_POSITIVE\_REGULATION\_OF\_ORGANELLE\_ORGANIZATION |  | 425 | 0.26 | 1.19 | 0.075 | 0.703 | 1.000 | 3211 | tags=32%, list=27%, signal=43% |
| 1876 | GOBP\_ALKALOID\_METABOLIC\_PROCESS |  | 5 | 0.62 | 1.19 | 0.281 | 0.703 | 1.000 | 1072 | tags=40%, list=9%, signal=44% |
| 1877 | GOCC\_SMOOTH\_ENDOPLASMIC\_RETICULUM\_MEMBRANE |  | 5 | 0.62 | 1.19 | 0.300 | 0.702 | 1.000 | 1210 | tags=40%, list=10%, signal=44% |
| 1878 | GOBP\_REGULATION\_OF\_BILE\_ACID\_BIOSYNTHETIC\_PROCESS |  | 8 | 0.53 | 1.19 | 0.271 | 0.702 | 1.000 | 1264 | tags=38%, list=11%, signal=42% |
| 1879 | GOCC\_MITOTIC\_SPINDLE |  | 119 | 0.29 | 1.19 | 0.152 | 0.703 | 1.000 | 2865 | tags=30%, list=24%, signal=39% |
| 1880 | GOBP\_POSITIVE\_REGULATION\_OF\_T\_HELPER\_CELL\_DIFFERENTIATION |  | 15 | 0.45 | 1.19 | 0.251 | 0.703 | 1.000 | 1517 | tags=27%, list=13%, signal=30% |
| 1881 | GOBP\_MITOTIC\_G2\_DNA\_DAMAGE\_CHECKPOINT |  | 20 | 0.42 | 1.19 | 0.236 | 0.703 | 1.000 | 4242 | tags=60%, list=35%, signal=93% |
| 1882 | GOBP\_PLASMA\_MEMBRANE\_REPAIR |  | 9 | 0.52 | 1.19 | 0.268 | 0.703 | 1.000 | 704 | tags=33%, list=6%, signal=35% |
| 1883 | GOBP\_TRAIL\_ACTIVATED\_APOPTOTIC\_SIGNALING\_PATHWAY |  | 8 | 0.54 | 1.19 | 0.271 | 0.703 | 1.000 | 2797 | tags=63%, list=23%, signal=81% |
| 1884 | GOBP\_POSITIVE\_REGULATION\_OF\_PROTEIN\_KINASE\_A\_SIGNALING |  | 6 | 0.58 | 1.19 | 0.284 | 0.703 | 1.000 | 3061 | tags=67%, list=25%, signal=89% |
| 1885 | GOMF\_DTTP\_DIPHOSPHATASE\_ACTIVITY |  | 4 | 0.65 | 1.19 | 0.285 | 0.704 | 1.000 | 3152 | tags=75%, list=26%, signal=102% |
| 1886 | GOBP\_RHOMBOMERE\_DEVELOPMENT |  | 4 | 0.66 | 1.19 | 0.284 | 0.703 | 1.000 | 1816 | tags=75%, list=15%, signal=88% |
| 1887 | GOBP\_TRYPTOPHAN\_CATABOLIC\_PROCESS |  | 5 | 0.61 | 1.19 | 0.284 | 0.703 | 1.000 | 1295 | tags=40%, list=11%, signal=45% |
| 1888 | GOBP\_WHITE\_FAT\_CELL\_DIFFERENTIATION |  | 11 | 0.49 | 1.19 | 0.263 | 0.703 | 1.000 | 2274 | tags=45%, list=19%, signal=56% |
| 1889 | GOMF\_D\_GLUCOSE\_TRANSMEMBRANE\_TRANSPORTER\_ACTIVITY |  | 3 | 0.71 | 1.19 | 0.284 | 0.703 | 1.000 | 1360 | tags=67%, list=11%, signal=75% |
| 1890 | GOCC\_U2\_TYPE\_SPLICEOSOMAL\_COMPLEX |  | 75 | 0.32 | 1.19 | 0.182 | 0.703 | 1.000 | 4101 | tags=40%, list=34%, signal=60% |
| 1891 | GOBP\_RESPONSE\_TO\_ACIDIC\_PH |  | 18 | 0.43 | 1.19 | 0.246 | 0.703 | 1.000 | 463 | tags=22%, list=4%, signal=23% |
| 1892 | GOBP\_ORGANIC\_ACID\_TRANSMEMBRANE\_TRANSPORT |  | 104 | 0.30 | 1.19 | 0.162 | 0.703 | 1.000 | 1326 | tags=20%, list=11%, signal=23% |
| 1893 | GOBP\_NEGATIVE\_REGULATION\_OF\_CELL\_CELL\_ADHESION |  | 111 | 0.30 | 1.19 | 0.163 | 0.703 | 1.000 | 2457 | tags=26%, list=20%, signal=33% |
| 1894 | GOBP\_POSITIVE\_REGULATION\_OF\_PROTEIN\_GLYCOSYLATION |  | 8 | 0.54 | 1.19 | 0.271 | 0.703 | 1.000 | 2691 | tags=38%, list=22%, signal=48% |
| 1895 | GOBP\_SYNAPTIC\_VESICLE\_RECYCLING\_VIA\_ENDOSOME |  | 7 | 0.56 | 1.18 | 0.277 | 0.704 | 1.000 | 2582 | tags=57%, list=21%, signal=73% |
| 1896 | GOBP\_CELLULAR\_RESPONSE\_TO\_NITROGEN\_COMPOUND |  | 469 | 0.25 | 1.18 | 0.074 | 0.704 | 1.000 | 2885 | tags=30%, list=24%, signal=38% |
| 1897 | GOBP\_EMBRYONIC\_HEART\_TUBE\_MORPHOGENESIS |  | 44 | 0.35 | 1.18 | 0.205 | 0.703 | 1.000 | 1268 | tags=16%, list=11%, signal=18% |
| 1898 | GOBP\_REGULATION\_OF\_LEUKOCYTE\_MEDIATED\_CYTOTOXICITY |  | 27 | 0.39 | 1.18 | 0.236 | 0.703 | 1.000 | 822 | tags=19%, list=7%, signal=20% |
| 1899 | GOBP\_REGULATION\_OF\_POSTSYNAPTIC\_MEMBRANE\_NEUROTRANSMITTER\_RECEPTOR\_LEVELS |  | 35 | 0.36 | 1.18 | 0.218 | 0.705 | 1.000 | 2044 | tags=26%, list=17%, signal=31% |
| 1900 | GOBP\_NEGATIVE\_REGULATION\_OF\_BICELLULAR\_TIGHT\_JUNCTION\_ASSEMBLY |  | 3 | 0.71 | 1.18 | 0.291 | 0.705 | 1.000 | 3506 | tags=100%, list=29%, signal=141% |
| 1901 | GOBP\_PRIMITIVE\_HEMOPOIESIS |  | 7 | 0.56 | 1.18 | 0.283 | 0.705 | 1.000 | 1794 | tags=29%, list=15%, signal=34% |
| 1902 | GOBP\_MAINTENANCE\_OF\_CELL\_POLARITY |  | 14 | 0.45 | 1.18 | 0.246 | 0.705 | 1.000 | 3327 | tags=50%, list=28%, signal=69% |
| 1903 | GOMF\_ACTIVE\_TRANSMEMBRANE\_TRANSPORTER\_ACTIVITY |  | 192 | 0.27 | 1.18 | 0.126 | 0.705 | 1.000 | 1475 | tags=23%, list=12%, signal=26% |
| 1904 | GOCC\_INTRINSIC\_COMPONENT\_OF\_ENDOPLASMIC\_RETICULUM\_MEMBRANE |  | 110 | 0.30 | 1.18 | 0.163 | 0.705 | 1.000 | 1102 | tags=15%, list=9%, signal=16% |
| 1905 | GOBP\_UBIQUITIN\_INDEPENDENT\_PROTEIN\_CATABOLIC\_PROCESS\_VIA\_THE\_MULTIVESICULAR\_BODY\_SORTING\_PATHWAY |  | 5 | 0.61 | 1.18 | 0.290 | 0.705 | 1.000 | 3335 | tags=80%, list=28%, signal=111% |
| 1906 | GOBP\_SYNAPTIC\_VESICLE\_ENDOSOMAL\_PROCESSING |  | 9 | 0.52 | 1.18 | 0.273 | 0.705 | 1.000 | 2582 | tags=56%, list=21%, signal=71% |
| 1907 | GOBP\_RESPONSE\_TO\_PROSTAGLANDIN |  | 22 | 0.41 | 1.18 | 0.245 | 0.705 | 1.000 | 1693 | tags=32%, list=14%, signal=37% |
| 1908 | GOMF\_PHOSPHOLIPID\_TRANSPORTER\_ACTIVITY |  | 35 | 0.37 | 1.18 | 0.231 | 0.705 | 1.000 | 1918 | tags=26%, list=16%, signal=31% |
| 1909 | GOMF\_MHC\_PROTEIN\_COMPLEX\_BINDING |  | 11 | 0.49 | 1.18 | 0.266 | 0.705 | 1.000 | 753 | tags=27%, list=6%, signal=29% |
| 1910 | GOBP\_POSITIVE\_REGULATION\_OF\_REGULATORY\_T\_CELL\_DIFFERENTIATION |  | 4 | 0.65 | 1.18 | 0.290 | 0.705 | 1.000 | 672 | tags=25%, list=6%, signal=26% |
| 1911 | GOBP\_POSTSYNAPTIC\_ACTIN\_CYTOSKELETON\_ORGANIZATION |  | 6 | 0.58 | 1.18 | 0.288 | 0.704 | 1.000 | 5064 | tags=100%, list=42%, signal=173% |
| 1912 | GOBP\_REGULATION\_OF\_TRANSLATION\_INITIATION\_IN\_RESPONSE\_TO\_ENDOPLASMIC\_RETICULUM\_STRESS |  | 6 | 0.58 | 1.18 | 0.279 | 0.704 | 1.000 | 2101 | tags=33%, list=17%, signal=40% |
| 1913 | GOBP\_RESPONSE\_TO\_STILBENOID |  | 4 | 0.64 | 1.18 | 0.294 | 0.705 | 1.000 | 1326 | tags=75%, list=11%, signal=84% |
| 1914 | GOBP\_POSITIVE\_REGULATION\_OF\_TOLL\_LIKE\_RECEPTOR\_9\_SIGNALING\_PATHWAY |  | 5 | 0.61 | 1.18 | 0.296 | 0.705 | 1.000 | 220 | tags=20%, list=2%, signal=20% |
| 1915 | GOBP\_POSITIVE\_REGULATION\_OF\_RESPONSE\_TO\_EXTERNAL\_STIMULUS |  | 300 | 0.26 | 1.18 | 0.101 | 0.705 | 1.000 | 2113 | tags=21%, list=18%, signal=25% |
| 1916 | GOBP\_REGULATION\_OF\_HEART\_RATE\_BY\_CARDIAC\_CONDUCTION |  | 27 | 0.39 | 1.18 | 0.233 | 0.706 | 1.000 | 1459 | tags=30%, list=12%, signal=34% |
| 1917 | GOBP\_REGULATION\_OF\_CARDIAC\_MUSCLE\_CELL\_MEMBRANE\_POTENTIAL |  | 3 | 0.71 | 1.18 | 0.286 | 0.705 | 1.000 | 1778 | tags=67%, list=15%, signal=78% |
| 1918 | GOBP\_CELLULAR\_RESPONSE\_TO\_ATP |  | 10 | 0.51 | 1.18 | 0.256 | 0.705 | 1.000 | 2802 | tags=50%, list=23%, signal=65% |
| 1919 | GOBP\_ADENOSINE\_METABOLIC\_PROCESS |  | 6 | 0.58 | 1.18 | 0.290 | 0.705 | 1.000 | 45 | tags=17%, list=0%, signal=17% |
| 1920 | GOCC\_PLASMA\_MEMBRANE\_RAFT |  | 79 | 0.31 | 1.18 | 0.179 | 0.705 | 1.000 | 2261 | tags=27%, list=19%, signal=33% |
| 1921 | GOBP\_POSITIVE\_REGULATION\_OF\_PROTEIN\_SUMOYLATION |  | 11 | 0.49 | 1.18 | 0.268 | 0.706 | 1.000 | 1202 | tags=27%, list=10%, signal=30% |
| 1922 | GOCC\_MULTIVESICULAR\_BODY\_MEMBRANE |  | 10 | 0.50 | 1.18 | 0.281 | 0.707 | 1.000 | 1579 | tags=30%, list=13%, signal=35% |
| 1923 | GOCC\_CHROMOSOME\_PASSENGER\_COMPLEX |  | 3 | 0.71 | 1.18 | 0.289 | 0.706 | 1.000 | 3528 | tags=100%, list=29%, signal=142% |
| 1924 | GOBP\_POSITIVE\_REGULATION\_OF\_CD4\_POSITIVE\_ALPHA\_BETA\_T\_CELL\_ACTIVATION |  | 23 | 0.41 | 1.18 | 0.236 | 0.706 | 1.000 | 1968 | tags=30%, list=16%, signal=36% |
| 1925 | GOBP\_INNER\_EAR\_MORPHOGENESIS |  | 46 | 0.35 | 1.18 | 0.214 | 0.706 | 1.000 | 2441 | tags=28%, list=20%, signal=35% |
| 1926 | GOBP\_CELLULAR\_RESPONSE\_TO\_PROSTAGLANDIN\_STIMULUS |  | 16 | 0.44 | 1.18 | 0.244 | 0.705 | 1.000 | 2625 | tags=44%, list=22%, signal=56% |
| 1927 | GOBP\_CELL\_COMMUNICATION\_BY\_ELECTRICAL\_COUPLING\_INVOLVED\_IN\_CARDIAC\_CONDUCTION |  | 15 | 0.45 | 1.18 | 0.263 | 0.705 | 1.000 | 2434 | tags=33%, list=20%, signal=42% |
| 1928 | GOBP\_ENDODERMAL\_CELL\_DIFFERENTIATION |  | 31 | 0.38 | 1.18 | 0.221 | 0.705 | 1.000 | 2920 | tags=35%, list=24%, signal=47% |
| 1929 | GOBP\_CELL\_JUNCTION\_ORGANIZATION |  | 475 | 0.25 | 1.18 | 0.072 | 0.705 | 1.000 | 2446 | tags=25%, list=20%, signal=30% |
| 1930 | GOBP\_REGULATION\_OF\_ACTIVATION\_OF\_JANUS\_KINASE\_ACTIVITY |  | 3 | 0.70 | 1.18 | 0.296 | 0.705 | 1.000 | 672 | tags=67%, list=6%, signal=71% |
| 1931 | GOBP\_PEPTIDYL\_THREONINE\_MODIFICATION |  | 90 | 0.31 | 1.18 | 0.171 | 0.705 | 1.000 | 2652 | tags=26%, list=22%, signal=33% |
| 1932 | GOMF\_TRANSMEMBRANE\_RECEPTOR\_PROTEIN\_SERINE\_THREONINE\_KINASE\_ACTIVITY |  | 13 | 0.47 | 1.18 | 0.269 | 0.706 | 1.000 | 3734 | tags=46%, list=31%, signal=67% |
| 1933 | GOBP\_HISTAMINE\_METABOLIC\_PROCESS |  | 5 | 0.61 | 1.18 | 0.290 | 0.705 | 1.000 | 389 | tags=20%, list=3%, signal=21% |
| 1934 | GOBP\_NEGATIVE\_REGULATION\_OF\_DENDRITE\_DEVELOPMENT |  | 3 | 0.71 | 1.18 | 0.309 | 0.706 | 1.000 | 2013 | tags=67%, list=17%, signal=80% |
| 1935 | GOBP\_REGULATION\_OF\_PROTEIN\_LOCALIZATION\_TO\_NUCLEUS |  | 112 | 0.30 | 1.18 | 0.168 | 0.706 | 1.000 | 2131 | tags=21%, list=18%, signal=25% |
| 1936 | GOBP\_NEGATIVE\_REGULATION\_OF\_TELOMERE\_CAPPING |  | 6 | 0.57 | 1.18 | 0.274 | 0.707 | 1.000 | 816 | tags=33%, list=7%, signal=36% |
| 1937 | GOBP\_SRP\_DEPENDENT\_COTRANSLATIONAL\_PROTEIN\_TARGETING\_TO\_MEMBRANE\_TRANSLOCATION |  | 6 | 0.58 | 1.18 | 0.293 | 0.707 | 1.000 | 5068 | tags=100%, list=42%, signal=173% |
| 1938 | GOBP\_PROTEIN\_POLY\_ADP\_RIBOSYLATION |  | 5 | 0.60 | 1.18 | 0.300 | 0.707 | 1.000 | 191 | tags=20%, list=2%, signal=20% |
| 1939 | GOCC\_RIPOPTOSOME |  | 5 | 0.61 | 1.18 | 0.294 | 0.707 | 1.000 | 4121 | tags=80%, list=34%, signal=122% |
| 1940 | GOBP\_SUGAR\_MEDIATED\_SIGNALING\_PATHWAY |  | 4 | 0.65 | 1.18 | 0.298 | 0.707 | 1.000 | 1199 | tags=50%, list=10%, signal=56% |
| 1941 | GOBP\_REGULATION\_OF\_PROTEIN\_LOCALIZATION\_TO\_CENTROSOME |  | 6 | 0.57 | 1.18 | 0.284 | 0.706 | 1.000 | 3884 | tags=50%, list=32%, signal=74% |
| 1942 | GOBP\_AMINOPHOSPHOLIPID\_TRANSLOCATION |  | 6 | 0.58 | 1.18 | 0.297 | 0.707 | 1.000 | 786 | tags=33%, list=7%, signal=36% |
| 1943 | GOBP\_RECEPTOR\_GUANYLYL\_CYCLASE\_SIGNALING\_PATHWAY |  | 3 | 0.70 | 1.18 | 0.300 | 0.707 | 1.000 | 1413 | tags=67%, list=12%, signal=76% |
| 1944 | GOBP\_TRANSMEMBRANE\_RECEPTOR\_PROTEIN\_TYROSINE\_KINASE\_SIGNALING\_PATHWAY |  | 546 | 0.25 | 1.18 | 0.071 | 0.707 | 1.000 | 2722 | tags=28%, list=23%, signal=35% |
| 1945 | GOBP\_LIPID\_DROPLET\_FORMATION |  | 5 | 0.61 | 1.18 | 0.296 | 0.707 | 1.000 | 92 | tags=20%, list=1%, signal=20% |
| 1946 | GOBP\_REGULATION\_OF\_CELLULAR\_RESPONSE\_TO\_STRESS |  | 492 | 0.25 | 1.18 | 0.072 | 0.707 | 1.000 | 1967 | tags=18%, list=16%, signal=21% |
| 1947 | GOMF\_LYSINE\_N\_ACETYLTRANSFERASE\_ACTIVITY\_ACTING\_ON\_ACETYL\_PHOSPHATE\_AS\_DONOR |  | 5 | 0.61 | 1.18 | 0.300 | 0.707 | 1.000 | 974 | tags=40%, list=8%, signal=44% |
| 1948 | GOBP\_POSITIVE\_REGULATION\_OF\_PHOSPHORUS\_METABOLIC\_PROCESS |  | 662 | 0.25 | 1.18 | 0.058 | 0.707 | 1.000 | 2056 | tags=21%, list=17%, signal=24% |
| 1949 | GOBP\_REGULATION\_OF\_SMAD\_PROTEIN\_SIGNAL\_TRANSDUCTION |  | 16 | 0.44 | 1.18 | 0.258 | 0.708 | 1.000 | 2351 | tags=44%, list=20%, signal=54% |
| 1950 | GOBP\_REGULATION\_OF\_PEPTIDYL\_THREONINE\_PHOSPHORYLATION |  | 32 | 0.37 | 1.18 | 0.233 | 0.709 | 1.000 | 2013 | tags=25%, list=17%, signal=30% |
| 1951 | GOBP\_FIBRINOLYSIS |  | 12 | 0.48 | 1.18 | 0.272 | 0.709 | 1.000 | 4497 | tags=67%, list=37%, signal=106% |
| 1952 | GOBP\_KETONE\_BODY\_METABOLIC\_PROCESS |  | 7 | 0.55 | 1.18 | 0.279 | 0.710 | 1.000 | 1933 | tags=43%, list=16%, signal=51% |
| 1953 | GOBP\_SUBPALLIUM\_DEVELOPMENT |  | 15 | 0.44 | 1.18 | 0.262 | 0.710 | 1.000 | 1022 | tags=33%, list=9%, signal=36% |
| 1954 | GOBP\_REGULATION\_OF\_NUCLEAR\_DIVISION |  | 99 | 0.30 | 1.18 | 0.176 | 0.710 | 1.000 | 3959 | tags=49%, list=33%, signal=73% |
| 1955 | GOBP\_NEGATIVE\_REGULATION\_OF\_NEURON\_MIGRATION |  | 6 | 0.57 | 1.18 | 0.304 | 0.710 | 1.000 | 3133 | tags=83%, list=26%, signal=113% |
| 1956 | GOBP\_REGULATION\_OF\_ATTACHMENT\_OF\_SPINDLE\_MICROTUBULES\_TO\_KINETOCHORE |  | 13 | 0.46 | 1.18 | 0.270 | 0.710 | 1.000 | 3884 | tags=69%, list=32%, signal=102% |
| 1957 | GOBP\_REGULATION\_OF\_CELLULAR\_CARBOHYDRATE\_METABOLIC\_PROCESS |  | 96 | 0.30 | 1.18 | 0.180 | 0.710 | 1.000 | 1427 | tags=22%, list=12%, signal=25% |
| 1958 | GOBP\_CARTILAGE\_DEVELOPMENT |  | 118 | 0.29 | 1.18 | 0.167 | 0.710 | 1.000 | 2830 | tags=34%, list=24%, signal=44% |
| 1959 | GOBP\_REGULATION\_OF\_PROTEIN\_PHOSPHORYLATION |  | 805 | 0.24 | 1.18 | 0.048 | 0.710 | 1.000 | 2691 | tags=25%, list=22%, signal=31% |
| 1960 | GOCC\_EXTRINSIC\_COMPONENT\_OF\_PLASMA\_MEMBRANE |  | 120 | 0.29 | 1.18 | 0.172 | 0.709 | 1.000 | 2872 | tags=32%, list=24%, signal=41% |
| 1961 | GOBP\_MICROTUBULE\_ORGANIZING\_CENTER\_ORGANIZATION |  | 112 | 0.30 | 1.17 | 0.167 | 0.711 | 1.000 | 3045 | tags=34%, list=25%, signal=45% |
| 1962 | GOBP\_BONE\_DEVELOPMENT |  | 134 | 0.29 | 1.17 | 0.166 | 0.711 | 1.000 | 2545 | tags=28%, list=21%, signal=36% |
| 1963 | GOCC\_SYNAPTIC\_CLEFT |  | 8 | 0.53 | 1.17 | 0.282 | 0.711 | 1.000 | 875 | tags=38%, list=7%, signal=40% |
| 1964 | GOMF\_D\_LOOP\_DNA\_BINDING |  | 4 | 0.64 | 1.17 | 0.304 | 0.711 | 1.000 | 3149 | tags=50%, list=26%, signal=68% |
| 1965 | GOBP\_NAIL\_DEVELOPMENT |  | 4 | 0.65 | 1.17 | 0.304 | 0.711 | 1.000 | 3080 | tags=75%, list=26%, signal=101% |
| 1966 | GOBP\_POSITIVE\_REGULATION\_OF\_AMYLOID\_BETA\_FORMATION |  | 14 | 0.45 | 1.17 | 0.264 | 0.710 | 1.000 | 2592 | tags=43%, list=22%, signal=55% |
| 1967 | GOBP\_DEOXYRIBONUCLEOSIDE\_TRIPHOSPHATE\_BIOSYNTHETIC\_PROCESS |  | 5 | 0.61 | 1.17 | 0.302 | 0.710 | 1.000 | 61 | tags=20%, list=1%, signal=20% |
| 1968 | GOBP\_CIRCULATORY\_SYSTEM\_PROCESS |  | 386 | 0.25 | 1.17 | 0.091 | 0.710 | 1.000 | 2097 | tags=24%, list=17%, signal=29% |
| 1969 | GOMF\_3\_BETA\_HYDROXY\_DELTA5\_STEROID\_DEHYDROGENASE\_ACTIVITY |  | 3 | 0.70 | 1.17 | 0.297 | 0.711 | 1.000 | 369 | tags=33%, list=3%, signal=34% |
| 1970 | GOCC\_GOLGI\_STACK |  | 102 | 0.30 | 1.17 | 0.177 | 0.711 | 1.000 | 3444 | tags=36%, list=29%, signal=50% |
| 1971 | GOMF\_ESTROGEN\_16\_ALPHA\_HYDROXYLASE\_ACTIVITY |  | 3 | 0.70 | 1.17 | 0.299 | 0.711 | 1.000 | 146 | tags=33%, list=1%, signal=34% |
| 1972 | GOBP\_EMBRYONIC\_CAMERA\_TYPE\_EYE\_FORMATION |  | 5 | 0.60 | 1.17 | 0.299 | 0.711 | 1.000 | 3037 | tags=60%, list=25%, signal=80% |
| 1973 | GOBP\_PEPTIDYL\_ARGININE\_N\_METHYLATION |  | 6 | 0.57 | 1.17 | 0.304 | 0.711 | 1.000 | 349 | tags=17%, list=3%, signal=17% |
| 1974 | GOBP\_PYRIMIDINE\_NUCLEOTIDE\_SALVAGE |  | 5 | 0.61 | 1.17 | 0.303 | 0.711 | 1.000 | 6 | tags=20%, list=0%, signal=20% |
| 1975 | GOBP\_NUCLEOSIDE\_MONOPHOSPHATE\_METABOLIC\_PROCESS |  | 59 | 0.33 | 1.17 | 0.202 | 0.711 | 1.000 | 904 | tags=14%, list=8%, signal=15% |
| 1976 | GOBP\_MUSCLE\_HYPERTROPHY\_IN\_RESPONSE\_TO\_STRESS |  | 15 | 0.45 | 1.17 | 0.256 | 0.711 | 1.000 | 1308 | tags=33%, list=11%, signal=37% |
| 1977 | GOBP\_REGULATION\_OF\_ACTION\_POTENTIAL |  | 36 | 0.36 | 1.17 | 0.228 | 0.711 | 1.000 | 1368 | tags=28%, list=11%, signal=31% |
| 1978 | GOBP\_G2\_DNA\_DAMAGE\_CHECKPOINT |  | 29 | 0.38 | 1.17 | 0.243 | 0.711 | 1.000 | 4242 | tags=55%, list=35%, signal=85% |
| 1979 | GOBP\_CELL\_FATE\_SPECIFICATION |  | 47 | 0.34 | 1.17 | 0.209 | 0.711 | 1.000 | 1394 | tags=21%, list=12%, signal=24% |
| 1980 | GOMF\_MHC\_PROTEIN\_BINDING |  | 15 | 0.45 | 1.17 | 0.260 | 0.711 | 1.000 | 430 | tags=20%, list=4%, signal=21% |
| 1981 | GOBP\_DNA\_REPLICATION\_INITIATION |  | 35 | 0.36 | 1.17 | 0.228 | 0.711 | 1.000 | 3695 | tags=54%, list=31%, signal=78% |
| 1982 | GOBP\_MEMBRANE\_FISSION |  | 11 | 0.48 | 1.17 | 0.277 | 0.711 | 1.000 | 3113 | tags=64%, list=26%, signal=86% |
| 1983 | GOBP\_CELL\_CYCLE\_G2\_M\_PHASE\_TRANSITION |  | 213 | 0.27 | 1.17 | 0.141 | 0.711 | 1.000 | 4435 | tags=49%, list=37%, signal=77% |
| 1984 | GOBP\_REGULATION\_OF\_CELLULAR\_RESPONSE\_TO\_MACROPHAGE\_COLONY\_STIMULATING\_FACTOR\_STIMULUS |  | 4 | 0.65 | 1.17 | 0.309 | 0.711 | 1.000 | 2458 | tags=50%, list=20%, signal=63% |
| 1985 | GOBP\_HAIR\_CELL\_DIFFERENTIATION |  | 28 | 0.38 | 1.17 | 0.251 | 0.711 | 1.000 | 2077 | tags=25%, list=17%, signal=30% |
| 1986 | GOBP\_POSITIVE\_REGULATION\_OF\_AUTOPHAGOSOME\_ASSEMBLY |  | 9 | 0.51 | 1.17 | 0.276 | 0.711 | 1.000 | 2757 | tags=56%, list=23%, signal=72% |
| 1987 | GOBP\_DRUG\_TRANSMEMBRANE\_TRANSPORT |  | 8 | 0.52 | 1.17 | 0.281 | 0.712 | 1.000 | 792 | tags=38%, list=7%, signal=40% |
| 1988 | GOBP\_POSITIVE\_REGULATION\_OF\_LONG\_TERM\_NEURONAL\_SYNAPTIC\_PLASTICITY |  | 5 | 0.61 | 1.17 | 0.303 | 0.712 | 1.000 | 4062 | tags=60%, list=34%, signal=91% |
| 1989 | GOBP\_REGULATION\_OF\_CELL\_KILLING |  | 37 | 0.36 | 1.17 | 0.229 | 0.712 | 1.000 | 2978 | tags=38%, list=25%, signal=50% |
| 1990 | GOBP\_INTEGRIN\_ACTIVATION |  | 20 | 0.42 | 1.17 | 0.251 | 0.712 | 1.000 | 2354 | tags=30%, list=20%, signal=37% |
| 1991 | GOBP\_NEGATIVE\_REGULATION\_OF\_ANOIKIS |  | 13 | 0.47 | 1.17 | 0.269 | 0.711 | 1.000 | 2340 | tags=46%, list=19%, signal=57% |
| 1992 | GOBP\_REGULATION\_OF\_PROTEIN\_ADP\_RIBOSYLATION |  | 4 | 0.64 | 1.17 | 0.310 | 0.711 | 1.000 | 4237 | tags=75%, list=35%, signal=116% |
| 1993 | GOBP\_IRE1\_MEDIATED\_UNFOLDED\_PROTEIN\_RESPONSE |  | 54 | 0.33 | 1.17 | 0.216 | 0.711 | 1.000 | 2916 | tags=33%, list=24%, signal=44% |
| 1994 | GOBP\_TELOMERE\_MAINTENANCE\_VIA\_RECOMBINATION |  | 12 | 0.48 | 1.17 | 0.283 | 0.712 | 1.000 | 1046 | tags=25%, list=9%, signal=27% |
| 1995 | GOBP\_NEGATIVE\_REGULATION\_OF\_LYMPHOCYTE\_ACTIVATION |  | 87 | 0.30 | 1.17 | 0.188 | 0.712 | 1.000 | 1092 | tags=16%, list=9%, signal=18% |
| 1996 | GOBP\_REGULATION\_OF\_TRANSCRIPTION\_INVOLVED\_IN\_G1\_S\_TRANSITION\_OF\_MITOTIC\_CELL\_CYCLE |  | 28 | 0.38 | 1.17 | 0.249 | 0.713 | 1.000 | 3993 | tags=50%, list=33%, signal=75% |
| 1997 | GOBP\_HEPATOCYTE\_GROWTH\_FACTOR\_RECEPTOR\_SIGNALING\_PATHWAY |  | 14 | 0.46 | 1.17 | 0.273 | 0.713 | 1.000 | 2414 | tags=43%, list=20%, signal=54% |
| 1998 | GOCC\_U2\_TYPE\_CATALYTIC\_STEP\_2\_SPLICEOSOME |  | 25 | 0.39 | 1.17 | 0.254 | 0.713 | 1.000 | 3759 | tags=40%, list=31%, signal=58% |
| 1999 | GOBP\_ATTACHMENT\_OF\_MITOTIC\_SPINDLE\_MICROTUBULES\_TO\_KINETOCHORE |  | 13 | 0.47 | 1.17 | 0.266 | 0.712 | 1.000 | 3866 | tags=69%, list=32%, signal=102% |
| 2000 | GOBP\_POSITIVE\_REGULATION\_OF\_T\_CELL\_MIGRATION |  | 21 | 0.41 | 1.17 | 0.252 | 0.712 | 1.000 | 3100 | tags=48%, list=26%, signal=64% |
| 2001 | GOBP\_REGULATION\_OF\_WATER\_LOSS\_VIA\_SKIN |  | 11 | 0.48 | 1.17 | 0.278 | 0.712 | 1.000 | 1878 | tags=36%, list=16%, signal=43% |
| 2002 | GOBP\_POSITIVE\_REGULATION\_OF\_AMINO\_ACID\_TRANSPORT |  | 13 | 0.46 | 1.17 | 0.261 | 0.711 | 1.000 | 1015 | tags=23%, list=8%, signal=25% |
| 2003 | GOBP\_POSITIVE\_REGULATION\_OF\_VACUOLE\_ORGANIZATION |  | 10 | 0.50 | 1.17 | 0.286 | 0.711 | 1.000 | 2757 | tags=50%, list=23%, signal=65% |
| 2004 | GOMF\_PHOSPHORUS\_OXYGEN\_LYASE\_ACTIVITY |  | 13 | 0.46 | 1.17 | 0.264 | 0.711 | 1.000 | 2102 | tags=38%, list=18%, signal=47% |
| 2005 | GOBP\_SYNAPTIC\_VESICLE\_BUDDING |  | 7 | 0.55 | 1.17 | 0.282 | 0.711 | 1.000 | 1128 | tags=29%, list=9%, signal=32% |
| 2006 | GOBP\_NUCLEOSIDE\_DIPHOSPHATE\_BIOSYNTHETIC\_PROCESS |  | 9 | 0.51 | 1.17 | 0.275 | 0.711 | 1.000 | 61 | tags=11%, list=1%, signal=11% |
| 2007 | GOMF\_CYSTEINE\_TYPE\_ENDOPEPTIDASE\_REGULATOR\_ACTIVITY\_INVOLVED\_IN\_APOPTOTIC\_PROCESS |  | 22 | 0.41 | 1.17 | 0.255 | 0.711 | 1.000 | 1507 | tags=27%, list=13%, signal=31% |
| 2008 | GOBP\_CERAMIDE\_METABOLIC\_PROCESS |  | 74 | 0.31 | 1.17 | 0.208 | 0.711 | 1.000 | 1889 | tags=24%, list=16%, signal=29% |
| 2009 | GOBP\_ALDITOL\_CATABOLIC\_PROCESS |  | 6 | 0.57 | 1.17 | 0.299 | 0.712 | 1.000 | 3694 | tags=83%, list=31%, signal=120% |
| 2010 | GOBP\_REGULATION\_OF\_PROTEIN\_CONTAINING\_COMPLEX\_DISASSEMBLY |  | 86 | 0.31 | 1.17 | 0.193 | 0.712 | 1.000 | 1375 | tags=16%, list=11%, signal=18% |
| 2011 | GOBP\_POLY\_N\_ACETYLLACTOSAMINE\_METABOLIC\_PROCESS |  | 7 | 0.55 | 1.17 | 0.300 | 0.712 | 1.000 | 110 | tags=14%, list=1%, signal=14% |
| 2012 | GOBP\_TYPE\_2\_IMMUNE\_RESPONSE |  | 21 | 0.41 | 1.17 | 0.262 | 0.712 | 1.000 | 1198 | tags=24%, list=10%, signal=26% |
| 2013 | GOBP\_GLOMERULAR\_EPITHELIAL\_CELL\_DEVELOPMENT |  | 9 | 0.51 | 1.17 | 0.299 | 0.711 | 1.000 | 2680 | tags=56%, list=22%, signal=71% |
| 2014 | GOBP\_PHOSPHOLIPID\_BIOSYNTHETIC\_PROCESS |  | 213 | 0.27 | 1.17 | 0.141 | 0.711 | 1.000 | 2154 | tags=26%, list=18%, signal=31% |
| 2015 | GOBP\_PYROPTOSIS |  | 5 | 0.60 | 1.17 | 0.308 | 0.711 | 1.000 | 2552 | tags=60%, list=21%, signal=76% |
| 2016 | GOBP\_REGULATION\_OF\_PRESYNAPTIC\_CYTOSOLIC\_CALCIUM\_ION\_CONCENTRATION |  | 13 | 0.46 | 1.17 | 0.266 | 0.711 | 1.000 | 382 | tags=23%, list=3%, signal=24% |
| 2017 | GOBP\_DOUBLE\_STRAND\_BREAK\_REPAIR\_VIA\_SYNTHESIS\_DEPENDENT\_STRAND\_ANNEALING |  | 6 | 0.58 | 1.17 | 0.283 | 0.711 | 1.000 | 1046 | tags=33%, list=9%, signal=36% |
| 2018 | GOBP\_BASEMENT\_MEMBRANE\_ORGANIZATION |  | 26 | 0.39 | 1.17 | 0.252 | 0.711 | 1.000 | 2585 | tags=35%, list=22%, signal=44% |
| 2019 | GOMF\_PHOSPHATIDYLSERINE\_BINDING |  | 43 | 0.35 | 1.17 | 0.221 | 0.711 | 1.000 | 1045 | tags=19%, list=9%, signal=20% |
| 2020 | GOMF\_CATALYTIC\_ACTIVITY\_ACTING\_ON\_DNA |  | 146 | 0.28 | 1.17 | 0.163 | 0.712 | 1.000 | 3350 | tags=39%, list=28%, signal=53% |
| 2021 | GOBP\_REGULATION\_OF\_BRANCHING\_INVOLVED\_IN\_SALIVARY\_GLAND\_MORPHOGENESIS |  | 6 | 0.57 | 1.17 | 0.298 | 0.712 | 1.000 | 4375 | tags=67%, list=36%, signal=105% |
| 2022 | GOBP\_NEGATIVE\_REGULATION\_OF\_ESTABLISHMENT\_OF\_PROTEIN\_LOCALIZATION |  | 85 | 0.30 | 1.17 | 0.196 | 0.712 | 1.000 | 2311 | tags=26%, list=19%, signal=32% |
| 2023 | GOBP\_CHEMOKINE\_C\_X\_C\_MOTIF\_LIGAND\_1\_PRODUCTION |  | 3 | 0.69 | 1.17 | 0.305 | 0.712 | 1.000 | 304 | tags=33%, list=3%, signal=34% |
| 2024 | GOBP\_HISTONE\_MRNA\_METABOLIC\_PROCESS |  | 18 | 0.42 | 1.17 | 0.266 | 0.712 | 1.000 | 2829 | tags=33%, list=24%, signal=44% |
| 2025 | GOBP\_DNA\_ENDOREDUPLICATION |  | 5 | 0.61 | 1.17 | 0.314 | 0.712 | 1.000 | 1864 | tags=40%, list=16%, signal=47% |
| 2026 | GOMF\_METALLODIPEPTIDASE\_ACTIVITY |  | 5 | 0.61 | 1.17 | 0.304 | 0.712 | 1.000 | 1540 | tags=40%, list=13%, signal=46% |
| 2027 | GOBP\_EXTRACELLULAR\_VESICLE\_BIOGENESIS |  | 20 | 0.41 | 1.17 | 0.256 | 0.712 | 1.000 | 4173 | tags=60%, list=35%, signal=92% |
| 2028 | GOMF\_GTPASE\_BINDING |  | 172 | 0.27 | 1.17 | 0.160 | 0.712 | 1.000 | 2722 | tags=33%, list=23%, signal=42% |
| 2029 | GOBP\_LYMPHOCYTE\_MIGRATION |  | 62 | 0.32 | 1.17 | 0.210 | 0.712 | 1.000 | 3100 | tags=40%, list=26%, signal=54% |
| 2030 | GOBP\_GLUCOSE\_IMPORT\_ACROSS\_PLASMA\_MEMBRANE |  | 3 | 0.71 | 1.17 | 0.311 | 0.712 | 1.000 | 1360 | tags=67%, list=11%, signal=75% |
| 2031 | GOBP\_POSITIVE\_REGULATION\_OF\_BLOOD\_BRAIN\_BARRIER\_PERMEABILITY |  | 5 | 0.60 | 1.17 | 0.306 | 0.711 | 1.000 | 1445 | tags=40%, list=12%, signal=45% |
| 2032 | GOBP\_NEGATIVE\_REGULATION\_OF\_T\_CELL\_CYTOKINE\_PRODUCTION |  | 3 | 0.70 | 1.17 | 0.310 | 0.712 | 1.000 | 405 | tags=33%, list=3%, signal=34% |
| 2033 | GOBP\_LYMPH\_VESSEL\_MORPHOGENESIS |  | 16 | 0.44 | 1.17 | 0.274 | 0.712 | 1.000 | 2548 | tags=31%, list=21%, signal=40% |
| 2034 | GOMF\_CLATHRIN\_LIGHT\_CHAIN\_BINDING |  | 5 | 0.60 | 1.17 | 0.308 | 0.711 | 1.000 | 3572 | tags=80%, list=30%, signal=114% |
| 2035 | GOBP\_NEGATIVE\_REGULATION\_OF\_CYTOPLASMIC\_TRANSLATION |  | 7 | 0.55 | 1.17 | 0.297 | 0.711 | 1.000 | 2205 | tags=57%, list=18%, signal=70% |
| 2036 | GOBP\_REGULATION\_OF\_CELLULAR\_LOCALIZATION |  | 581 | 0.25 | 1.17 | 0.079 | 0.711 | 1.000 | 2190 | tags=20%, list=18%, signal=23% |
| 2037 | GOBP\_SIGNAL\_TRANSDUCTION\_INVOLVED\_IN\_REGULATION\_OF\_GENE\_EXPRESSION |  | 8 | 0.53 | 1.17 | 0.289 | 0.711 | 1.000 | 2802 | tags=63%, list=23%, signal=81% |
| 2038 | GOBP\_POSITIVE\_REGULATION\_OF\_RUFFLE\_ASSEMBLY |  | 8 | 0.53 | 1.17 | 0.295 | 0.711 | 1.000 | 3511 | tags=63%, list=29%, signal=88% |
| 2039 | GOBP\_VESICLE\_TARGETING |  | 74 | 0.31 | 1.17 | 0.203 | 0.710 | 1.000 | 3458 | tags=39%, list=29%, signal=55% |
| 2040 | GOMF\_ATP\_ACTIVATED\_INWARD\_RECTIFIER\_POTASSIUM\_CHANNEL\_ACTIVITY |  | 3 | 0.70 | 1.17 | 0.318 | 0.710 | 1.000 | 3637 | tags=100%, list=30%, signal=143% |
| 2041 | GOBP\_REGULATION\_OF\_MITOTIC\_CELL\_CYCLE |  | 444 | 0.25 | 1.17 | 0.096 | 0.711 | 1.000 | 4084 | tags=41%, list=34%, signal=60% |
| 2042 | GOCC\_INSULIN\_RESPONSIVE\_COMPARTMENT |  | 7 | 0.55 | 1.16 | 0.294 | 0.712 | 1.000 | 2578 | tags=43%, list=21%, signal=55% |
| 2043 | GOBP\_EPITHELIAL\_CILIUM\_MOVEMENT\_INVOLVED\_IN\_DETERMINATION\_OF\_LEFT\_RIGHT\_ASYMMETRY |  | 4 | 0.64 | 1.16 | 0.314 | 0.712 | 1.000 | 1985 | tags=50%, list=17%, signal=60% |
| 2044 | GOBP\_POSITIVE\_REGULATION\_OF\_TRANSCRIPTION\_FROM\_RNA\_POLYMERASE\_II\_PROMOTER\_INVOLVED\_IN\_HEART\_DEVELOPMENT |  | 5 | 0.60 | 1.16 | 0.313 | 0.711 | 1.000 | 182 | tags=20%, list=2%, signal=20% |
| 2045 | GOBP\_GLYCEROPHOSPHOLIPID\_METABOLIC\_PROCESS |  | 260 | 0.26 | 1.16 | 0.140 | 0.711 | 1.000 | 1925 | tags=24%, list=16%, signal=28% |
| 2046 | GOBP\_RESPONSE\_TO\_MUSCLE\_STRETCH |  | 16 | 0.44 | 1.16 | 0.277 | 0.711 | 1.000 | 1216 | tags=25%, list=10%, signal=28% |
| 2047 | GOBP\_CELL\_CYCLE\_ARREST |  | 176 | 0.28 | 1.16 | 0.150 | 0.711 | 1.000 | 3318 | tags=34%, list=28%, signal=46% |
| 2048 | GOBP\_REGULATION\_OF\_ORGANELLE\_ORGANIZATION |  | 850 | 0.24 | 1.16 | 0.056 | 0.712 | 1.000 | 3338 | tags=32%, list=28%, signal=41% |
| 2049 | GOMF\_PROTEIN\_ARGININE\_N\_METHYLTRANSFERASE\_ACTIVITY |  | 6 | 0.57 | 1.16 | 0.304 | 0.711 | 1.000 | 349 | tags=17%, list=3%, signal=17% |
| 2050 | GOBP\_OVULATION\_CYCLE\_PROCESS |  | 29 | 0.38 | 1.16 | 0.255 | 0.711 | 1.000 | 2500 | tags=34%, list=21%, signal=43% |
| 2051 | GOBP\_CELL\_CYCLE\_PHASE\_TRANSITION |  | 478 | 0.25 | 1.16 | 0.094 | 0.711 | 1.000 | 4084 | tags=41%, list=34%, signal=59% |
| 2052 | GOBP\_CELLULAR\_RESPONSE\_TO\_MANGANESE\_ION |  | 9 | 0.50 | 1.16 | 0.278 | 0.712 | 1.000 | 2167 | tags=44%, list=18%, signal=54% |
| 2053 | GOBP\_NEGATIVE\_REGULATION\_OF\_METALLOENDOPEPTIDASE\_ACTIVITY |  | 4 | 0.64 | 1.16 | 0.316 | 0.713 | 1.000 | 535 | tags=25%, list=4%, signal=26% |
| 2054 | GOBP\_NUCLEOTIDE\_EXCISION\_REPAIR\_DNA\_GAP\_FILLING |  | 19 | 0.41 | 1.16 | 0.250 | 0.712 | 1.000 | 1769 | tags=32%, list=15%, signal=37% |
| 2055 | GOBP\_DYNAMIN\_FAMILY\_PROTEIN\_POLYMERIZATION\_INVOLVED\_IN\_MEMBRANE\_FISSION |  | 5 | 0.60 | 1.16 | 0.314 | 0.712 | 1.000 | 1986 | tags=60%, list=17%, signal=72% |
| 2056 | GOBP\_POSITIVE\_REGULATION\_OF\_CATABOLIC\_PROCESS |  | 335 | 0.26 | 1.16 | 0.111 | 0.712 | 1.000 | 2859 | tags=28%, list=24%, signal=36% |
| 2057 | GOBP\_NEGATIVE\_REGULATION\_OF\_DEVELOPMENTAL\_PROCESS |  | 542 | 0.25 | 1.16 | 0.085 | 0.712 | 1.000 | 2476 | tags=25%, list=21%, signal=30% |
| 2058 | GOBP\_POSITIVE\_REGULATION\_OF\_CARBOHYDRATE\_METABOLIC\_PROCESS |  | 52 | 0.34 | 1.16 | 0.230 | 0.712 | 1.000 | 1419 | tags=25%, list=12%, signal=28% |
| 2059 | GOBP\_GROWTH\_PLATE\_CARTILAGE\_CHONDROCYTE\_DIFFERENTIATION |  | 6 | 0.57 | 1.16 | 0.305 | 0.711 | 1.000 | 1738 | tags=33%, list=14%, signal=39% |
| 2060 | GOBP\_INFLAMMATORY\_RESPONSE\_TO\_WOUNDING |  | 9 | 0.51 | 1.16 | 0.292 | 0.711 | 1.000 | 867 | tags=22%, list=7%, signal=24% |
| 2061 | GOBP\_NEGATIVE\_REGULATION\_OF\_ORGANELLE\_ORGANIZATION |  | 247 | 0.26 | 1.16 | 0.132 | 0.711 | 1.000 | 3984 | tags=43%, list=33%, signal=63% |
| 2062 | GOBP\_S\_ADENOSYLMETHIONINE\_METABOLIC\_PROCESS |  | 14 | 0.45 | 1.16 | 0.272 | 0.712 | 1.000 | 1074 | tags=21%, list=9%, signal=24% |
| 2063 | GOBP\_REGULATION\_OF\_VESICLE\_TRANSPORT\_ALONG\_MICROTUBULE |  | 3 | 0.70 | 1.16 | 0.324 | 0.711 | 1.000 | 1203 | tags=67%, list=10%, signal=74% |
| 2064 | GOBP\_NEUTRAL\_AMINO\_ACID\_TRANSPORT |  | 30 | 0.38 | 1.16 | 0.253 | 0.712 | 1.000 | 1743 | tags=33%, list=15%, signal=39% |
| 2065 | GOBP\_ESTABLISHMENT\_OF\_BLOOD\_BRAIN\_BARRIER |  | 6 | 0.57 | 1.16 | 0.306 | 0.711 | 1.000 | 373 | tags=17%, list=3%, signal=17% |
| 2066 | GOBP\_REGULATION\_OF\_LIPID\_TRANSPORTER\_ACTIVITY |  | 3 | 0.69 | 1.16 | 0.310 | 0.711 | 1.000 | 2680 | tags=67%, list=22%, signal=86% |
| 2067 | GOBP\_FORMATION\_OF\_PRIMARY\_GERM\_LAYER |  | 76 | 0.31 | 1.16 | 0.209 | 0.711 | 1.000 | 1603 | tags=20%, list=13%, signal=23% |
| 2068 | GOBP\_MIDGUT\_DEVELOPMENT |  | 10 | 0.49 | 1.16 | 0.289 | 0.711 | 1.000 | 48 | tags=10%, list=0%, signal=10% |
| 2069 | GOBP\_POSITIVE\_REGULATION\_OF\_NMDA\_GLUTAMATE\_RECEPTOR\_ACTIVITY |  | 3 | 0.70 | 1.16 | 0.308 | 0.711 | 1.000 | 512 | tags=33%, list=4%, signal=35% |
| 2070 | GOBP\_POSITIVE\_REGULATION\_OF\_PEPTIDASE\_ACTIVITY |  | 129 | 0.28 | 1.16 | 0.177 | 0.712 | 1.000 | 2842 | tags=33%, list=24%, signal=42% |
| 2071 | GOBP\_EMBRYONIC\_SKELETAL\_JOINT\_DEVELOPMENT |  | 11 | 0.48 | 1.16 | 0.283 | 0.713 | 1.000 | 655 | tags=18%, list=5%, signal=19% |
| 2072 | GOBP\_RESPIRATORY\_SYSTEM\_DEVELOPMENT |  | 131 | 0.29 | 1.16 | 0.175 | 0.714 | 1.000 | 2582 | tags=27%, list=21%, signal=35% |
| 2073 | GOBP\_MICROTUBULE\_ANCHORING |  | 18 | 0.42 | 1.16 | 0.264 | 0.714 | 1.000 | 4030 | tags=50%, list=34%, signal=75% |
| 2074 | GOBP\_REGULATION\_OF\_CIRCADIAN\_RHYTHM |  | 82 | 0.31 | 1.16 | 0.199 | 0.713 | 1.000 | 3213 | tags=35%, list=27%, signal=48% |
| 2075 | GOBP\_ORGANIC\_ANION\_TRANSPORT |  | 235 | 0.26 | 1.16 | 0.145 | 0.714 | 1.000 | 1865 | tags=24%, list=16%, signal=28% |
| 2076 | GOBP\_REGULATION\_OF\_NITRIC\_OXIDE\_MEDIATED\_SIGNAL\_TRANSDUCTION |  | 6 | 0.57 | 1.16 | 0.309 | 0.714 | 1.000 | 5137 | tags=100%, list=43%, signal=175% |
| 2077 | GOMF\_RECEPTOR\_ANTAGONIST\_ACTIVITY |  | 8 | 0.52 | 1.16 | 0.290 | 0.714 | 1.000 | 1607 | tags=50%, list=13%, signal=58% |
| 2078 | GOBP\_GOLGI\_RIBBON\_FORMATION |  | 10 | 0.49 | 1.16 | 0.298 | 0.715 | 1.000 | 2121 | tags=40%, list=18%, signal=49% |
| 2079 | GOMF\_SOMATOSTATIN\_RECEPTOR\_ACTIVITY |  | 3 | 0.69 | 1.16 | 0.320 | 0.715 | 1.000 | 3776 | tags=100%, list=31%, signal=146% |
| 2080 | GOCC\_GAMMA\_TUBULIN\_LARGE\_COMPLEX |  | 6 | 0.56 | 1.16 | 0.309 | 0.714 | 1.000 | 2491 | tags=50%, list=21%, signal=63% |
| 2081 | GOCC\_INTERSTITIAL\_MATRIX |  | 6 | 0.57 | 1.16 | 0.313 | 0.715 | 1.000 | 4760 | tags=83%, list=40%, signal=138% |
| 2082 | GOCC\_FILTRATION\_DIAPHRAGM |  | 4 | 0.64 | 1.16 | 0.323 | 0.715 | 1.000 | 2185 | tags=50%, list=18%, signal=61% |
| 2083 | GOBP\_IMMATURE\_B\_CELL\_DIFFERENTIATION |  | 8 | 0.53 | 1.16 | 0.306 | 0.715 | 1.000 | 1081 | tags=25%, list=9%, signal=27% |
| 2084 | GOBP\_POSITIVE\_REGULATION\_OF\_TROPHOBLAST\_CELL\_MIGRATION |  | 6 | 0.57 | 1.16 | 0.299 | 0.715 | 1.000 | 1794 | tags=33%, list=15%, signal=39% |
| 2085 | GOCC\_MICROTUBULE\_CYTOSKELETON |  | 906 | 0.24 | 1.16 | 0.054 | 0.715 | 1.000 | 3707 | tags=36%, list=31%, signal=48% |
| 2086 | GOBP\_ARACHIDONIC\_ACID\_SECRETION |  | 14 | 0.45 | 1.16 | 0.281 | 0.715 | 1.000 | 2044 | tags=43%, list=17%, signal=52% |
| 2087 | GOBP\_POSITIVE\_REGULATION\_OF\_DNA\_METHYLATION\_DEPENDENT\_HETEROCHROMATIN\_ASSEMBLY |  | 5 | 0.60 | 1.16 | 0.312 | 0.714 | 1.000 | 1354 | tags=20%, list=11%, signal=23% |
| 2088 | GOBP\_MESENCHYMAL\_TO\_EPITHELIAL\_TRANSITION\_INVOLVED\_IN\_METANEPHROS\_MORPHOGENESIS |  | 5 | 0.60 | 1.16 | 0.310 | 0.715 | 1.000 | 78 | tags=20%, list=1%, signal=20% |
| 2089 | GOBP\_POSITIVE\_REGULATION\_OF\_FATTY\_ACID\_BIOSYNTHETIC\_PROCESS |  | 15 | 0.44 | 1.16 | 0.279 | 0.715 | 1.000 | 1199 | tags=33%, list=10%, signal=37% |
| 2090 | GOBP\_REGULATION\_OF\_LIPASE\_ACTIVITY |  | 65 | 0.32 | 1.16 | 0.220 | 0.715 | 1.000 | 1374 | tags=20%, list=11%, signal=22% |
| 2091 | GOBP\_PYRIMIDINE\_CONTAINING\_COMPOUND\_BIOSYNTHETIC\_PROCESS |  | 31 | 0.37 | 1.16 | 0.249 | 0.715 | 1.000 | 1179 | tags=16%, list=10%, signal=18% |
| 2092 | GOBP\_MESENCHYMAL\_CELL\_DIFFERENTIATION\_INVOLVED\_IN\_KIDNEY\_DEVELOPMENT |  | 6 | 0.56 | 1.16 | 0.306 | 0.715 | 1.000 | 2604 | tags=50%, list=22%, signal=64% |
| 2093 | GOBP\_ENZYME\_LINKED\_RECEPTOR\_PROTEIN\_SIGNALING\_PATHWAY |  | 761 | 0.24 | 1.16 | 0.061 | 0.715 | 1.000 | 2722 | tags=28%, list=23%, signal=34% |
| 2094 | GOBP\_POSITIVE\_REGULATION\_OF\_OSSIFICATION |  | 31 | 0.37 | 1.16 | 0.249 | 0.715 | 1.000 | 2351 | tags=35%, list=20%, signal=44% |
| 2095 | GOBP\_PLATELET\_DERIVED\_GROWTH\_FACTOR\_RECEPTOR\_BETA\_SIGNALING\_PATHWAY |  | 14 | 0.45 | 1.16 | 0.283 | 0.715 | 1.000 | 5143 | tags=79%, list=43%, signal=137% |
| 2096 | GOBP\_DIACYLGLYCEROL\_METABOLIC\_PROCESS |  | 20 | 0.41 | 1.16 | 0.271 | 0.715 | 1.000 | 1765 | tags=40%, list=15%, signal=47% |
| 2097 | GOBP\_DE\_NOVO\_NAD\_BIOSYNTHETIC\_PROCESS |  | 4 | 0.64 | 1.16 | 0.319 | 0.715 | 1.000 | 1295 | tags=50%, list=11%, signal=56% |
| 2098 | GOMF\_KINASE\_ACTIVATOR\_ACTIVITY |  | 66 | 0.32 | 1.16 | 0.220 | 0.715 | 1.000 | 2214 | tags=30%, list=18%, signal=37% |
| 2099 | GOBP\_ECTOPIC\_GERM\_CELL\_PROGRAMMED\_CELL\_DEATH |  | 4 | 0.64 | 1.16 | 0.322 | 0.715 | 1.000 | 526 | tags=25%, list=4%, signal=26% |
| 2100 | GOBP\_INTERLEUKIN\_12\_PRODUCTION |  | 33 | 0.37 | 1.16 | 0.257 | 0.715 | 1.000 | 1517 | tags=21%, list=13%, signal=24% |
| 2101 | GOMF\_3\_5\_CYCLIC\_GMP\_PHOSPHODIESTERASE\_ACTIVITY |  | 8 | 0.52 | 1.16 | 0.295 | 0.715 | 1.000 | 4751 | tags=63%, list=40%, signal=103% |
| 2102 | GOBP\_REGULATION\_OF\_LAMELLIPODIUM\_ORGANIZATION |  | 39 | 0.35 | 1.16 | 0.251 | 0.715 | 1.000 | 1737 | tags=26%, list=14%, signal=30% |
| 2103 | GOBP\_REGULATION\_OF\_ERBB\_SIGNALING\_PATHWAY |  | 73 | 0.31 | 1.16 | 0.211 | 0.715 | 1.000 | 2608 | tags=25%, list=22%, signal=31% |
| 2104 | GOBP\_REGULATION\_OF\_INFLAMMATORY\_RESPONSE |  | 204 | 0.27 | 1.16 | 0.158 | 0.717 | 1.000 | 2115 | tags=24%, list=18%, signal=29% |
| 2105 | GOBP\_NEGATIVE\_REGULATION\_OF\_CELL\_ADHESION\_MOLECULE\_PRODUCTION |  | 3 | 0.69 | 1.16 | 0.330 | 0.717 | 1.000 | 589 | tags=33%, list=5%, signal=35% |
| 2106 | GOBP\_REGULATION\_OF\_PEPTIDYL\_SERINE\_PHOSPHORYLATION\_OF\_STAT\_PROTEIN |  | 4 | 0.64 | 1.16 | 0.321 | 0.717 | 1.000 | 3584 | tags=75%, list=30%, signal=107% |
| 2107 | GOBP\_HINDBRAIN\_RADIAL\_GLIA\_GUIDED\_CELL\_MIGRATION |  | 7 | 0.55 | 1.16 | 0.309 | 0.717 | 1.000 | 48 | tags=14%, list=0%, signal=14% |
| 2108 | GOBP\_INTEGRIN\_MEDIATED\_SIGNALING\_PATHWAY |  | 80 | 0.31 | 1.16 | 0.213 | 0.717 | 1.000 | 2354 | tags=29%, list=20%, signal=36% |
| 2109 | GOBP\_TOOTH\_ERUPTION |  | 6 | 0.57 | 1.16 | 0.316 | 0.717 | 1.000 | 87 | tags=17%, list=1%, signal=17% |
| 2110 | GOBP\_REGULATION\_OF\_DEVELOPMENT\_HETEROCHRONIC |  | 4 | 0.65 | 1.16 | 0.321 | 0.717 | 1.000 | 1394 | tags=50%, list=12%, signal=57% |
| 2111 | GOBP\_PROLINE\_BIOSYNTHETIC\_PROCESS |  | 3 | 0.69 | 1.15 | 0.323 | 0.718 | 1.000 | 3412 | tags=67%, list=28%, signal=93% |
| 2112 | GOBP\_GENITALIA\_DEVELOPMENT |  | 25 | 0.39 | 1.15 | 0.265 | 0.718 | 1.000 | 620 | tags=12%, list=5%, signal=13% |
| 2113 | GOCC\_ACTIN\_BASED\_CELL\_PROJECTION |  | 144 | 0.28 | 1.15 | 0.178 | 0.718 | 1.000 | 1956 | tags=24%, list=16%, signal=29% |
| 2114 | GOBP\_NEGATIVE\_REGULATION\_OF\_WOUND\_HEALING |  | 43 | 0.34 | 1.15 | 0.236 | 0.718 | 1.000 | 3693 | tags=42%, list=31%, signal=60% |
| 2115 | GOBP\_REGULATION\_OF\_SYSTEM\_PROCESS |  | 345 | 0.25 | 1.15 | 0.130 | 0.718 | 1.000 | 1274 | tags=16%, list=11%, signal=17% |
| 2116 | GOBP\_POSITIVE\_REGULATION\_OF\_MEIOTIC\_NUCLEAR\_DIVISION |  | 6 | 0.57 | 1.15 | 0.311 | 0.719 | 1.000 | 3037 | tags=67%, list=25%, signal=89% |
| 2117 | GOBP\_RESPONSE\_TO\_HYDROGEN\_PEROXIDE |  | 102 | 0.29 | 1.15 | 0.204 | 0.719 | 1.000 | 3082 | tags=36%, list=26%, signal=48% |
| 2118 | GOBP\_REGULATION\_OF\_GLUCONEOGENESIS |  | 33 | 0.36 | 1.15 | 0.252 | 0.718 | 1.000 | 1353 | tags=24%, list=11%, signal=27% |
| 2119 | GOBP\_ENDOTHELIUM\_DEVELOPMENT |  | 99 | 0.30 | 1.15 | 0.195 | 0.718 | 1.000 | 3890 | tags=47%, list=32%, signal=70% |
| 2120 | GOCC\_G\_PROTEIN\_COUPLED\_RECEPTOR\_COMPLEX |  | 5 | 0.59 | 1.15 | 0.327 | 0.719 | 1.000 | 764 | tags=20%, list=6%, signal=21% |
| 2121 | GOMF\_DEHYDROASCORBIC\_ACID\_TRANSMEMBRANE\_TRANSPORTER\_ACTIVITY |  | 6 | 0.56 | 1.15 | 0.304 | 0.719 | 1.000 | 836 | tags=33%, list=7%, signal=36% |
| 2122 | GOBP\_NLRP3\_INFLAMMASOME\_COMPLEX\_ASSEMBLY |  | 8 | 0.52 | 1.15 | 0.296 | 0.719 | 1.000 | 1326 | tags=25%, list=11%, signal=28% |
| 2123 | GOBP\_TRYPTOPHAN\_TRANSPORT |  | 3 | 0.69 | 1.15 | 0.322 | 0.720 | 1.000 | 1015 | tags=67%, list=8%, signal=73% |
| 2124 | GOBP\_LYMPHOCYTE\_APOPTOTIC\_PROCESS |  | 50 | 0.33 | 1.15 | 0.232 | 0.720 | 1.000 | 769 | tags=16%, list=6%, signal=17% |
| 2125 | GOMF\_THROMBIN\_ACTIVATED\_RECEPTOR\_ACTIVITY |  | 4 | 0.64 | 1.15 | 0.330 | 0.721 | 1.000 | 202 | tags=25%, list=2%, signal=25% |
| 2126 | GOBP\_REGULATION\_OF\_HYALURONAN\_BIOSYNTHETIC\_PROCESS |  | 6 | 0.57 | 1.15 | 0.318 | 0.721 | 1.000 | 2440 | tags=50%, list=20%, signal=63% |
| 2127 | GOBP\_VENTRICULAR\_SEPTUM\_DEVELOPMENT |  | 50 | 0.33 | 1.15 | 0.235 | 0.721 | 1.000 | 3445 | tags=36%, list=29%, signal=50% |
| 2128 | GOMF\_HEXOSE\_TRANSMEMBRANE\_TRANSPORTER\_ACTIVITY |  | 14 | 0.45 | 1.15 | 0.294 | 0.721 | 1.000 | 1437 | tags=50%, list=12%, signal=57% |
| 2129 | GOBP\_POSITIVE\_REGULATION\_OF\_NOTCH\_SIGNALING\_PATHWAY |  | 42 | 0.35 | 1.15 | 0.254 | 0.721 | 1.000 | 1632 | tags=24%, list=14%, signal=27% |
| 2130 | GOBP\_ZYMOGEN\_ACTIVATION |  | 27 | 0.37 | 1.15 | 0.266 | 0.721 | 1.000 | 1393 | tags=22%, list=12%, signal=25% |
| 2131 | GOBP\_POSITIVE\_REGULATION\_OF\_FC\_RECEPTOR\_MEDIATED\_STIMULATORY\_SIGNALING\_PATHWAY |  | 7 | 0.54 | 1.15 | 0.319 | 0.721 | 1.000 | 827 | tags=29%, list=7%, signal=31% |
| 2132 | GOBP\_ACTIN\_POLYMERIZATION\_DEPENDENT\_CELL\_MOTILITY |  | 7 | 0.54 | 1.15 | 0.306 | 0.722 | 1.000 | 3091 | tags=57%, list=26%, signal=77% |
| 2133 | GOBP\_CELLULAR\_RESPONSE\_TO\_INSULIN\_LIKE\_GROWTH\_FACTOR\_STIMULUS |  | 7 | 0.54 | 1.15 | 0.312 | 0.722 | 1.000 | 2776 | tags=43%, list=23%, signal=56% |
| 2134 | GOBP\_RESPONSE\_TO\_FOLLICLE\_STIMULATING\_HORMONE |  | 9 | 0.50 | 1.15 | 0.292 | 0.722 | 1.000 | 855 | tags=22%, list=7%, signal=24% |
| 2135 | GOBP\_SOMATOSTATIN\_RECEPTOR\_SIGNALING\_PATHWAY |  | 3 | 0.69 | 1.15 | 0.332 | 0.722 | 1.000 | 3776 | tags=100%, list=31%, signal=146% |
| 2136 | GOBP\_CRANIAL\_NERVE\_MORPHOGENESIS |  | 12 | 0.47 | 1.15 | 0.296 | 0.723 | 1.000 | 907 | tags=25%, list=8%, signal=27% |
| 2137 | GOMF\_CYTOSKELETAL\_ANCHOR\_ACTIVITY |  | 18 | 0.42 | 1.15 | 0.273 | 0.724 | 1.000 | 3987 | tags=56%, list=33%, signal=83% |
| 2138 | GOMF\_AMMONIA\_LYASE\_ACTIVITY |  | 4 | 0.63 | 1.15 | 0.334 | 0.724 | 1.000 | 1138 | tags=50%, list=9%, signal=55% |
| 2139 | GOBP\_POSITIVE\_REGULATION\_OF\_CLATHRIN\_DEPENDENT\_ENDOCYTOSIS |  | 4 | 0.64 | 1.15 | 0.336 | 0.724 | 1.000 | 4320 | tags=100%, list=36%, signal=156% |
| 2140 | GOBP\_RESPONSE\_TO\_DEXAMETHASONE |  | 30 | 0.37 | 1.15 | 0.265 | 0.723 | 1.000 | 792 | tags=17%, list=7%, signal=18% |
| 2141 | GOBP\_NEGATIVE\_REGULATION\_OF\_MYELOID\_LEUKOCYTE\_DIFFERENTIATION |  | 29 | 0.37 | 1.15 | 0.267 | 0.724 | 1.000 | 902 | tags=21%, list=8%, signal=22% |
| 2142 | GOBP\_ERROR\_PRONE\_TRANSLESION\_SYNTHESIS |  | 19 | 0.41 | 1.15 | 0.283 | 0.724 | 1.000 | 3366 | tags=47%, list=28%, signal=66% |
| 2143 | GOBP\_INFLAMMATORY\_CELL\_APOPTOTIC\_PROCESS |  | 12 | 0.46 | 1.15 | 0.295 | 0.724 | 1.000 | 1060 | tags=25%, list=9%, signal=27% |
| 2144 | GOBP\_REGULATION\_OF\_MUCUS\_SECRETION |  | 3 | 0.68 | 1.15 | 0.333 | 0.724 | 1.000 | 2821 | tags=67%, list=23%, signal=87% |
| 2145 | GOMF\_CAMP\_RESPONSE\_ELEMENT\_BINDING |  | 14 | 0.44 | 1.15 | 0.284 | 0.724 | 1.000 | 2326 | tags=36%, list=19%, signal=44% |
| 2146 | GOBP\_RESPONSE\_TO\_VITAMIN |  | 60 | 0.32 | 1.15 | 0.230 | 0.725 | 1.000 | 1408 | tags=22%, list=12%, signal=24% |
| 2147 | GOBP\_PHYSIOLOGICAL\_CARDIAC\_MUSCLE\_HYPERTROPHY |  | 17 | 0.43 | 1.15 | 0.279 | 0.725 | 1.000 | 2013 | tags=29%, list=17%, signal=35% |
| 2148 | GOBP\_POSITIVE\_REGULATION\_OF\_TYPE\_2\_IMMUNE\_RESPONSE |  | 11 | 0.48 | 1.15 | 0.298 | 0.725 | 1.000 | 1198 | tags=27%, list=10%, signal=30% |
| 2149 | GOBP\_RESPONSE\_TO\_ISOLATION\_STRESS |  | 4 | 0.63 | 1.15 | 0.343 | 0.724 | 1.000 | 1509 | tags=50%, list=13%, signal=57% |
| 2150 | GOBP\_RHO\_PROTEIN\_SIGNAL\_TRANSDUCTION |  | 98 | 0.29 | 1.15 | 0.218 | 0.724 | 1.000 | 3569 | tags=43%, list=30%, signal=60% |
| 2151 | GOCC\_CIS\_GOLGI\_NETWORK |  | 36 | 0.36 | 1.15 | 0.262 | 0.724 | 1.000 | 2228 | tags=36%, list=19%, signal=44% |
| 2152 | GOBP\_POSTREPLICATION\_REPAIR |  | 42 | 0.34 | 1.15 | 0.252 | 0.725 | 1.000 | 3479 | tags=40%, list=29%, signal=57% |
| 2153 | GOBP\_REGULATION\_OF\_CYTOKINESIS |  | 67 | 0.31 | 1.15 | 0.237 | 0.725 | 1.000 | 2757 | tags=39%, list=23%, signal=50% |
| 2154 | GOCC\_PRESYNAPTIC\_MEMBRANE |  | 85 | 0.30 | 1.15 | 0.213 | 0.725 | 1.000 | 2956 | tags=39%, list=25%, signal=51% |
| 2155 | GOBP\_POSITIVE\_REGULATION\_OF\_PROTEIN\_LOCALIZATION\_TO\_NUCLEUS |  | 75 | 0.31 | 1.15 | 0.231 | 0.725 | 1.000 | 1856 | tags=17%, list=15%, signal=20% |
| 2156 | GOBP\_REGULATION\_OF\_CARBOHYDRATE\_METABOLIC\_PROCESS |  | 148 | 0.28 | 1.15 | 0.194 | 0.725 | 1.000 | 1693 | tags=21%, list=14%, signal=24% |
| 2157 | GOBP\_MEMBRANE\_ORGANIZATION |  | 626 | 0.24 | 1.15 | 0.096 | 0.725 | 1.000 | 2757 | tags=25%, list=23%, signal=31% |
| 2158 | GOBP\_CAMP\_BIOSYNTHETIC\_PROCESS |  | 6 | 0.56 | 1.15 | 0.314 | 0.725 | 1.000 | 1147 | tags=33%, list=10%, signal=37% |
| 2159 | GOCC\_LAMININ\_COMPLEX |  | 11 | 0.48 | 1.15 | 0.301 | 0.726 | 1.000 | 875 | tags=27%, list=7%, signal=29% |
| 2160 | GOBP\_REGULATION\_OF\_PHOSPHOLIPID\_TRANSLOCATION |  | 5 | 0.60 | 1.15 | 0.321 | 0.726 | 1.000 | 769 | tags=40%, list=6%, signal=43% |
| 2161 | GOBP\_RECEPTOR\_MEDIATED\_ENDOCYTOSIS |  | 180 | 0.27 | 1.15 | 0.178 | 0.726 | 1.000 | 2236 | tags=26%, list=19%, signal=31% |
| 2162 | GOBP\_GABAERGIC\_NEURON\_DIFFERENTIATION |  | 6 | 0.56 | 1.15 | 0.323 | 0.726 | 1.000 | 1508 | tags=50%, list=13%, signal=57% |
| 2163 | GOBP\_P38MAPK\_CASCADE |  | 33 | 0.36 | 1.15 | 0.262 | 0.727 | 1.000 | 1809 | tags=27%, list=15%, signal=32% |
| 2164 | GOBP\_SPLICEOSOMAL\_COMPLEX\_ASSEMBLY |  | 41 | 0.35 | 1.15 | 0.258 | 0.726 | 1.000 | 4078 | tags=46%, list=34%, signal=70% |
| 2165 | GOBP\_WATER\_HOMEOSTASIS |  | 45 | 0.34 | 1.15 | 0.255 | 0.727 | 1.000 | 2718 | tags=40%, list=23%, signal=52% |
| 2166 | GOBP\_GAMMA\_AMINOBUTYRIC\_ACID\_SECRETION |  | 5 | 0.59 | 1.15 | 0.333 | 0.726 | 1.000 | 10 | tags=20%, list=0%, signal=20% |
| 2167 | GOBP\_MICROGLIAL\_CELL\_ACTIVATION |  | 23 | 0.39 | 1.15 | 0.269 | 0.726 | 1.000 | 273 | tags=13%, list=2%, signal=13% |
| 2168 | GOMF\_POLY\_PURINE\_TRACT\_BINDING |  | 24 | 0.39 | 1.15 | 0.278 | 0.726 | 1.000 | 3186 | tags=38%, list=27%, signal=51% |
| 2169 | GOBP\_MYD88\_DEPENDENT\_TOLL\_LIKE\_RECEPTOR\_SIGNALING\_PATHWAY |  | 23 | 0.39 | 1.15 | 0.281 | 0.726 | 1.000 | 304 | tags=13%, list=3%, signal=13% |
| 2170 | GOBP\_POSITIVE\_REGULATION\_OF\_GENE\_EXPRESSION |  | 651 | 0.24 | 1.15 | 0.095 | 0.726 | 1.000 | 1727 | tags=16%, list=14%, signal=18% |
| 2171 | GOMF\_KINASE\_BINDING |  | 551 | 0.24 | 1.15 | 0.103 | 0.726 | 1.000 | 2676 | tags=25%, list=22%, signal=30% |
| 2172 | GOMF\_NUCLEOSIDE\_DIPHOSPHATASE\_ACTIVITY |  | 12 | 0.46 | 1.15 | 0.292 | 0.726 | 1.000 | 2021 | tags=33%, list=17%, signal=40% |
| 2173 | GOBP\_NEGATIVE\_REGULATION\_OF\_RIG\_I\_SIGNALING\_PATHWAY |  | 6 | 0.56 | 1.15 | 0.333 | 0.726 | 1.000 | 116 | tags=17%, list=1%, signal=17% |
| 2174 | GOCC\_COATED\_VESICLE\_MEMBRANE |  | 122 | 0.29 | 1.14 | 0.206 | 0.726 | 1.000 | 2662 | tags=32%, list=22%, signal=41% |
| 2175 | GOBP\_CHEMOKINE\_PRODUCTION |  | 54 | 0.33 | 1.14 | 0.249 | 0.728 | 1.000 | 1276 | tags=17%, list=11%, signal=19% |
| 2176 | GOBP\_SMOOTH\_MUSCLE\_CELL\_MATRIX\_ADHESION |  | 4 | 0.63 | 1.14 | 0.343 | 0.728 | 1.000 | 3773 | tags=75%, list=31%, signal=109% |
| 2177 | GOMF\_L\_LYSINE\_TRANSMEMBRANE\_TRANSPORTER\_ACTIVITY |  | 4 | 0.63 | 1.14 | 0.346 | 0.728 | 1.000 | 3129 | tags=75%, list=26%, signal=101% |
| 2178 | GOBP\_COPII\_COATED\_VESICLE\_BUDDING |  | 60 | 0.32 | 1.14 | 0.238 | 0.729 | 1.000 | 3458 | tags=38%, list=29%, signal=54% |
| 2179 | GOMF\_LYSOPHOSPHOLIPID\_ACYLTRANSFERASE\_ACTIVITY |  | 16 | 0.43 | 1.14 | 0.286 | 0.729 | 1.000 | 1925 | tags=44%, list=16%, signal=52% |
| 2180 | GOBP\_SENSORY\_ORGAN\_MORPHOGENESIS |  | 139 | 0.28 | 1.14 | 0.199 | 0.729 | 1.000 | 2843 | tags=29%, list=24%, signal=37% |
| 2181 | GOBP\_ANTIGEN\_PROCESSING\_AND\_PRESENTATION\_OF\_PEPTIDE\_ANTIGEN |  | 126 | 0.28 | 1.14 | 0.205 | 0.729 | 1.000 | 3910 | tags=40%, list=33%, signal=58% |
| 2182 | GOBP\_REGULATION\_OF\_CELL\_SHAPE |  | 109 | 0.29 | 1.14 | 0.210 | 0.729 | 1.000 | 1794 | tags=22%, list=15%, signal=26% |
| 2183 | GOBP\_POSITIVE\_REGULATION\_OF\_CHROMOSOME\_SEPARATION |  | 16 | 0.43 | 1.14 | 0.292 | 0.729 | 1.000 | 2660 | tags=38%, list=22%, signal=48% |
| 2184 | GOBP\_ESTABLISHMENT\_OF\_LYMPHOCYTE\_POLARITY |  | 9 | 0.50 | 1.14 | 0.304 | 0.729 | 1.000 | 1517 | tags=44%, list=13%, signal=51% |
| 2185 | GOMF\_GUANYL\_NUCLEOTIDE\_BINDING |  | 271 | 0.26 | 1.14 | 0.162 | 0.729 | 1.000 | 1883 | tags=20%, list=16%, signal=23% |
| 2186 | GOMF\_ACTININ\_BINDING |  | 25 | 0.38 | 1.14 | 0.284 | 0.729 | 1.000 | 525 | tags=16%, list=4%, signal=17% |
| 2187 | GOBP\_NEGATIVE\_REGULATION\_OF\_STEROID\_METABOLIC\_PROCESS |  | 20 | 0.40 | 1.14 | 0.282 | 0.728 | 1.000 | 1533 | tags=30%, list=13%, signal=34% |
| 2188 | GOBP\_CARTILAGE\_DEVELOPMENT\_INVOLVED\_IN\_ENDOCHONDRAL\_BONE\_MORPHOGENESIS |  | 18 | 0.42 | 1.14 | 0.295 | 0.729 | 1.000 | 2436 | tags=39%, list=20%, signal=49% |
| 2189 | GOMF\_TRANSCRIPTION\_REGULATOR\_ACTIVITY |  | 890 | 0.23 | 1.14 | 0.078 | 0.729 | 1.000 | 1893 | tags=18%, list=16%, signal=20% |
| 2190 | GOMF\_LIPASE\_BINDING |  | 4 | 0.64 | 1.14 | 0.335 | 0.730 | 1.000 | 248 | tags=25%, list=2%, signal=26% |
| 2191 | GOBP\_NEGATIVE\_REGULATION\_OF\_T\_CELL\_RECEPTOR\_SIGNALING\_PATHWAY |  | 18 | 0.41 | 1.14 | 0.290 | 0.730 | 1.000 | 1710 | tags=28%, list=14%, signal=32% |
| 2192 | GOBP\_POSITIVE\_REGULATION\_OF\_CYTOKINE\_PRODUCTION\_INVOLVED\_IN\_INFLAMMATORY\_RESPONSE |  | 8 | 0.51 | 1.14 | 0.313 | 0.731 | 1.000 | 2161 | tags=38%, list=18%, signal=46% |
| 2193 | GOBP\_NEGATIVE\_REGULATION\_OF\_ENDOTHELIAL\_CELL\_PROLIFERATION |  | 29 | 0.37 | 1.14 | 0.275 | 0.731 | 1.000 | 1531 | tags=28%, list=13%, signal=32% |
| 2194 | GOMF\_CELL\_ADHESION\_MEDIATOR\_ACTIVITY |  | 41 | 0.34 | 1.14 | 0.254 | 0.731 | 1.000 | 1060 | tags=20%, list=9%, signal=21% |
| 2195 | GOCC\_XY\_BODY |  | 9 | 0.50 | 1.14 | 0.317 | 0.731 | 1.000 | 2248 | tags=33%, list=19%, signal=41% |
| 2196 | GOBP\_BASEMENT\_MEMBRANE\_ASSEMBLY |  | 14 | 0.44 | 1.14 | 0.292 | 0.732 | 1.000 | 1607 | tags=21%, list=13%, signal=25% |
| 2197 | GOBP\_RESPONSE\_TO\_INORGANIC\_SUBSTANCE |  | 358 | 0.25 | 1.14 | 0.142 | 0.732 | 1.000 | 2572 | tags=28%, list=21%, signal=35% |
| 2198 | GOBP\_REGULATION\_OF\_PENILE\_ERECTION |  | 6 | 0.56 | 1.14 | 0.328 | 0.731 | 1.000 | 2845 | tags=83%, list=24%, signal=109% |
| 2199 | GOBP\_MRNA\_3\_END\_PROCESSING\_BY\_STEM\_LOOP\_BINDING\_AND\_CLEAVAGE |  | 4 | 0.63 | 1.14 | 0.336 | 0.731 | 1.000 | 1441 | tags=25%, list=12%, signal=28% |
| 2200 | GOMF\_ACYL\_COA\_BINDING |  | 20 | 0.40 | 1.14 | 0.295 | 0.732 | 1.000 | 1208 | tags=25%, list=10%, signal=28% |
| 2201 | GOBP\_NEGATIVE\_REGULATION\_OF\_PROTEIN\_IMPORT |  | 11 | 0.47 | 1.14 | 0.307 | 0.733 | 1.000 | 1919 | tags=36%, list=16%, signal=43% |
| 2202 | GOMF\_TRANSFERASE\_ACTIVITY\_TRANSFERRING\_PHOSPHORUS\_CONTAINING\_GROUPS |  | 643 | 0.24 | 1.14 | 0.101 | 0.734 | 1.000 | 2715 | tags=26%, list=23%, signal=32% |
| 2203 | GOMF\_N\_ACETYLLACTOSAMINIDE\_BETA\_1\_3\_N\_ACETYLGLUCOSAMINYLTRANSFERASE\_ACTIVITY |  | 6 | 0.56 | 1.14 | 0.327 | 0.735 | 1.000 | 110 | tags=17%, list=1%, signal=17% |
| 2204 | GOBP\_GLYCEROLIPID\_BIOSYNTHETIC\_PROCESS |  | 203 | 0.26 | 1.14 | 0.182 | 0.735 | 1.000 | 1977 | tags=25%, list=16%, signal=30% |
| 2205 | GOMF\_CHROMO\_SHADOW\_DOMAIN\_BINDING |  | 3 | 0.68 | 1.14 | 0.353 | 0.734 | 1.000 | 2914 | tags=67%, list=24%, signal=88% |
| 2206 | GOBP\_NEGATIVE\_REGULATION\_OF\_PROTEIN\_LOCALIZATION\_TO\_NUCLEUS |  | 23 | 0.39 | 1.14 | 0.290 | 0.735 | 1.000 | 2131 | tags=30%, list=18%, signal=37% |
| 2207 | GOCC\_DNA\_REPLICATION\_FACTOR\_C\_COMPLEX |  | 5 | 0.59 | 1.14 | 0.336 | 0.735 | 1.000 | 2926 | tags=60%, list=24%, signal=79% |
| 2208 | GOMF\_PEPTIDE\_TRANSMEMBRANE\_TRANSPORTER\_ACTIVITY |  | 24 | 0.39 | 1.14 | 0.271 | 0.734 | 1.000 | 1831 | tags=29%, list=15%, signal=34% |
| 2209 | GOBP\_POSITIVE\_REGULATION\_OF\_LEUKOCYTE\_CHEMOTAXIS |  | 60 | 0.31 | 1.14 | 0.246 | 0.735 | 1.000 | 2458 | tags=33%, list=20%, signal=42% |
| 2210 | GOMF\_HISTONE\_SERINE\_KINASE\_ACTIVITY |  | 4 | 0.62 | 1.14 | 0.344 | 0.735 | 1.000 | 3528 | tags=75%, list=29%, signal=106% |
| 2211 | GOBP\_NEGATIVE\_REGULATION\_OF\_VESICLE\_FUSION |  | 4 | 0.63 | 1.14 | 0.349 | 0.734 | 1.000 | 3952 | tags=50%, list=33%, signal=74% |
| 2212 | GOMF\_PROTEIN\_C\_TERMINUS\_BINDING |  | 145 | 0.28 | 1.14 | 0.196 | 0.734 | 1.000 | 2419 | tags=27%, list=20%, signal=33% |
| 2213 | GOMF\_HYDROLASE\_ACTIVITY\_ACTING\_ON\_CARBON\_NITROGEN\_BUT\_NOT\_PEPTIDE\_BONDS\_IN\_CYCLIC\_AMIDINES |  | 20 | 0.40 | 1.14 | 0.296 | 0.734 | 1.000 | 1086 | tags=25%, list=9%, signal=27% |
| 2214 | GOMF\_TRANSMEMBRANE\_RECEPTOR\_PROTEIN\_TYROSINE\_KINASE\_ADAPTOR\_ACTIVITY |  | 9 | 0.50 | 1.14 | 0.328 | 0.734 | 1.000 | 711 | tags=22%, list=6%, signal=24% |
| 2215 | GOMF\_NON\_MEMBRANE\_SPANNING\_PROTEIN\_TYROSINE\_KINASE\_ACTIVITY |  | 39 | 0.34 | 1.14 | 0.263 | 0.734 | 1.000 | 2602 | tags=36%, list=22%, signal=46% |
| 2216 | GOBP\_NEGATIVE\_REGULATION\_OF\_METALLOPEPTIDASE\_ACTIVITY |  | 6 | 0.55 | 1.14 | 0.332 | 0.735 | 1.000 | 2049 | tags=33%, list=17%, signal=40% |
| 2217 | GOBP\_CARBOHYDRATE\_MEDIATED\_SIGNALING |  | 5 | 0.58 | 1.14 | 0.337 | 0.736 | 1.000 | 1199 | tags=40%, list=10%, signal=44% |
| 2218 | GOBP\_ENDOTHELIAL\_CELL\_MATRIX\_ADHESION |  | 3 | 0.68 | 1.14 | 0.349 | 0.735 | 1.000 | 694 | tags=33%, list=6%, signal=35% |
| 2219 | GOMF\_SUGAR\_TRANSMEMBRANE\_TRANSPORTER\_ACTIVITY |  | 17 | 0.42 | 1.14 | 0.294 | 0.735 | 1.000 | 1437 | tags=47%, list=12%, signal=53% |
| 2220 | GOCC\_HIGH\_DENSITY\_LIPOPROTEIN\_PARTICLE |  | 10 | 0.48 | 1.14 | 0.318 | 0.736 | 1.000 | 733 | tags=30%, list=6%, signal=32% |
| 2221 | GOBP\_REGULATION\_OF\_MULTI\_ORGANISM\_PROCESS |  | 28 | 0.37 | 1.14 | 0.279 | 0.736 | 1.000 | 2845 | tags=46%, list=24%, signal=61% |
| 2222 | GOBP\_CARDIAC\_MUSCLE\_CELL\_MYOBLAST\_DIFFERENTIATION |  | 10 | 0.48 | 1.14 | 0.314 | 0.735 | 1.000 | 2741 | tags=50%, list=23%, signal=65% |
| 2223 | GOBP\_REGULATION\_OF\_CELL\_AGING |  | 40 | 0.34 | 1.14 | 0.263 | 0.735 | 1.000 | 1789 | tags=28%, list=15%, signal=32% |
| 2224 | GOBP\_CELLULAR\_RESPONSE\_TO\_MECHANICAL\_STIMULUS |  | 58 | 0.32 | 1.14 | 0.253 | 0.737 | 1.000 | 1293 | tags=16%, list=11%, signal=17% |
| 2225 | GOBP\_REGULATION\_OF\_HETEROTYPIC\_CELL\_CELL\_ADHESION |  | 15 | 0.43 | 1.14 | 0.297 | 0.737 | 1.000 | 589 | tags=20%, list=5%, signal=21% |
| 2226 | GOCC\_EXTRACELLULAR\_MEMBRANE\_BOUNDED\_ORGANELLE |  | 3 | 0.68 | 1.14 | 0.351 | 0.737 | 1.000 | 3097 | tags=67%, list=26%, signal=90% |
| 2227 | GOMF\_TROPOMYOSIN\_BINDING |  | 10 | 0.48 | 1.14 | 0.310 | 0.737 | 1.000 | 3614 | tags=50%, list=30%, signal=71% |
| 2228 | GOBP\_DEHYDROASCORBIC\_ACID\_TRANSPORT |  | 6 | 0.56 | 1.14 | 0.335 | 0.736 | 1.000 | 836 | tags=33%, list=7%, signal=36% |
| 2229 | GOMF\_EPOXIDE\_HYDROLASE\_ACTIVITY |  | 6 | 0.55 | 1.14 | 0.329 | 0.736 | 1.000 | 2980 | tags=33%, list=25%, signal=44% |
| 2230 | GOBP\_REGULATION\_OF\_MACROPHAGE\_CHEMOTAXIS |  | 20 | 0.40 | 1.14 | 0.286 | 0.736 | 1.000 | 713 | tags=20%, list=6%, signal=21% |
| 2231 | GOMF\_DYNEIN\_COMPLEX\_BINDING |  | 17 | 0.42 | 1.14 | 0.288 | 0.736 | 1.000 | 2074 | tags=29%, list=17%, signal=36% |
| 2232 | GOMF\_ADENYLATE\_CYCLASE\_ACTIVITY |  | 6 | 0.56 | 1.14 | 0.335 | 0.736 | 1.000 | 1147 | tags=33%, list=10%, signal=37% |
| 2233 | GOBP\_CARDIAC\_MUSCLE\_MYOBLAST\_PROLIFERATION |  | 3 | 0.67 | 1.13 | 0.348 | 0.736 | 1.000 | 2326 | tags=67%, list=19%, signal=83% |
| 2234 | GOBP\_L\_LYSINE\_TRANSMEMBRANE\_TRANSPORT |  | 4 | 0.63 | 1.13 | 0.350 | 0.737 | 1.000 | 3129 | tags=75%, list=26%, signal=101% |
| 2235 | GOMF\_CYCLASE\_ACTIVITY |  | 12 | 0.46 | 1.13 | 0.303 | 0.738 | 1.000 | 2102 | tags=33%, list=18%, signal=40% |
| 2236 | GOBP\_REGULATION\_OF\_POTASSIUM\_ION\_IMPORT |  | 5 | 0.59 | 1.13 | 0.346 | 0.737 | 1.000 | 725 | tags=40%, list=6%, signal=43% |
| 2237 | GOBP\_REGULATION\_OF\_T\_CELL\_MEDIATED\_IMMUNITY |  | 33 | 0.36 | 1.13 | 0.274 | 0.737 | 1.000 | 512 | tags=15%, list=4%, signal=16% |
| 2238 | GOBP\_ROOF\_OF\_MOUTH\_DEVELOPMENT |  | 62 | 0.31 | 1.13 | 0.244 | 0.737 | 1.000 | 2604 | tags=27%, list=22%, signal=35% |
| 2239 | GOMF\_PROTEIN\_DIMERIZATION\_ACTIVITY |  | 650 | 0.24 | 1.13 | 0.114 | 0.737 | 1.000 | 2166 | tags=21%, list=18%, signal=24% |
| 2240 | GOBP\_TRICUSPID\_VALVE\_DEVELOPMENT |  | 5 | 0.58 | 1.13 | 0.343 | 0.737 | 1.000 | 1336 | tags=40%, list=11%, signal=45% |
| 2241 | GOBP\_NEURON\_PROJECTION\_REGENERATION |  | 46 | 0.33 | 1.13 | 0.266 | 0.737 | 1.000 | 2131 | tags=30%, list=18%, signal=37% |
| 2242 | GOBP\_PYRIMIDINE\_RIBONUCLEOSIDE\_METABOLIC\_PROCESS |  | 6 | 0.56 | 1.13 | 0.339 | 0.736 | 1.000 | 6 | tags=17%, list=0%, signal=17% |
| 2243 | GOCC\_MEMBRANE\_MICRODOMAIN |  | 230 | 0.26 | 1.13 | 0.182 | 0.737 | 1.000 | 2201 | tags=24%, list=18%, signal=29% |
| 2244 | GOBP\_TYPE\_B\_PANCREATIC\_CELL\_APOPTOTIC\_PROCESS |  | 8 | 0.51 | 1.13 | 0.322 | 0.737 | 1.000 | 607 | tags=25%, list=5%, signal=26% |
| 2245 | GOBP\_EXTERNAL\_ENCAPSULATING\_STRUCTURE\_ORGANIZATION |  | 271 | 0.25 | 1.13 | 0.164 | 0.737 | 1.000 | 2885 | tags=32%, list=24%, signal=41% |
| 2246 | GOMF\_SYMPORTER\_ACTIVITY |  | 90 | 0.29 | 1.13 | 0.233 | 0.737 | 1.000 | 1465 | tags=24%, list=12%, signal=28% |
| 2247 | GOBP\_NEGATIVE\_REGULATION\_OF\_AXON\_EXTENSION |  | 29 | 0.37 | 1.13 | 0.285 | 0.737 | 1.000 | 3037 | tags=45%, list=25%, signal=60% |
| 2248 | GOBP\_NEUTROPHIL\_MEDIATED\_KILLING\_OF\_SYMBIONT\_CELL |  | 4 | 0.63 | 1.13 | 0.353 | 0.737 | 1.000 | 4049 | tags=75%, list=34%, signal=113% |
| 2249 | GOBP\_REGULATION\_OF\_LYMPHOCYTE\_MIGRATION |  | 38 | 0.35 | 1.13 | 0.278 | 0.737 | 1.000 | 1945 | tags=29%, list=16%, signal=34% |
| 2250 | GOBP\_CELLULAR\_RESPONSE\_TO\_ABIOTIC\_STIMULUS |  | 223 | 0.26 | 1.13 | 0.182 | 0.736 | 1.000 | 1561 | tags=17%, list=13%, signal=20% |
| 2251 | GOBP\_LAMELLIPODIUM\_MORPHOGENESIS |  | 14 | 0.44 | 1.13 | 0.300 | 0.736 | 1.000 | 1949 | tags=36%, list=16%, signal=43% |
| 2252 | GOBP\_LYSOSOMAL\_LUMEN\_ACIDIFICATION |  | 8 | 0.51 | 1.13 | 0.325 | 0.737 | 1.000 | 284 | tags=25%, list=2%, signal=26% |
| 2253 | GOBP\_EMBRYONIC\_SKELETAL\_JOINT\_MORPHOGENESIS |  | 8 | 0.51 | 1.13 | 0.313 | 0.736 | 1.000 | 5 | tags=13%, list=0%, signal=12% |
| 2254 | GOBP\_POSITIVE\_REGULATION\_OF\_ORGAN\_GROWTH |  | 30 | 0.37 | 1.13 | 0.282 | 0.736 | 1.000 | 2241 | tags=23%, list=19%, signal=29% |
| 2255 | GOBP\_NEGATIVE\_REGULATION\_OF\_EXOCYTOSIS |  | 20 | 0.40 | 1.13 | 0.298 | 0.736 | 1.000 | 1060 | tags=20%, list=9%, signal=22% |
| 2256 | GOBP\_SIGNAL\_TRANSDUCTION\_IN\_RESPONSE\_TO\_DNA\_DAMAGE |  | 104 | 0.29 | 1.13 | 0.234 | 0.736 | 1.000 | 2597 | tags=26%, list=22%, signal=33% |
| 2257 | GOBP\_MACROPHAGE\_ACTIVATION\_INVOLVED\_IN\_IMMUNE\_RESPONSE |  | 12 | 0.46 | 1.13 | 0.306 | 0.736 | 1.000 | 262 | tags=17%, list=2%, signal=17% |
| 2258 | GOBP\_POSITIVE\_REGULATION\_OF\_PINOCYTOSIS |  | 6 | 0.56 | 1.13 | 0.329 | 0.738 | 1.000 | 894 | tags=33%, list=7%, signal=36% |
| 2259 | GOBP\_NEGATIVE\_REGULATION\_OF\_CELLULAR\_EXTRAVASATION |  | 4 | 0.63 | 1.13 | 0.356 | 0.739 | 1.000 | 1625 | tags=50%, list=14%, signal=58% |
| 2260 | GOBP\_REGULATION\_OF\_DNA\_CATABOLIC\_PROCESS |  | 7 | 0.54 | 1.13 | 0.326 | 0.741 | 1.000 | 2597 | tags=71%, list=22%, signal=91% |
| 2261 | GOMF\_ADENYLATE\_CYCLASE\_BINDING |  | 9 | 0.50 | 1.13 | 0.324 | 0.740 | 1.000 | 3608 | tags=56%, list=30%, signal=79% |
| 2262 | GOMF\_C4\_DICARBOXYLATE\_TRANSMEMBRANE\_TRANSPORTER\_ACTIVITY |  | 12 | 0.46 | 1.13 | 0.304 | 0.740 | 1.000 | 1862 | tags=42%, list=16%, signal=49% |
| 2263 | GOBP\_GLOMERULUS\_MORPHOGENESIS |  | 8 | 0.51 | 1.13 | 0.328 | 0.740 | 1.000 | 77 | tags=13%, list=1%, signal=13% |
| 2264 | GOBP\_POSITIVE\_REGULATION\_OF\_I\_KAPPAB\_KINASE\_NF\_KAPPAB\_SIGNALING |  | 127 | 0.28 | 1.13 | 0.230 | 0.740 | 1.000 | 2138 | tags=24%, list=18%, signal=28% |
| 2265 | GOBP\_CELL\_MIGRATION\_INVOLVED\_IN\_SPROUTING\_ANGIOGENESIS |  | 41 | 0.34 | 1.13 | 0.269 | 0.740 | 1.000 | 2918 | tags=37%, list=24%, signal=48% |
| 2266 | GOBP\_POSITIVE\_CHEMOTAXIS |  | 42 | 0.34 | 1.13 | 0.276 | 0.740 | 1.000 | 3037 | tags=48%, list=25%, signal=64% |
| 2267 | GOBP\_HISTONE\_H3\_K9\_ACETYLATION |  | 8 | 0.51 | 1.13 | 0.317 | 0.740 | 1.000 | 1559 | tags=38%, list=13%, signal=43% |
| 2268 | GOBP\_NEGATIVE\_REGULATION\_OF\_CARBOHYDRATE\_METABOLIC\_PROCESS |  | 32 | 0.36 | 1.13 | 0.284 | 0.740 | 1.000 | 2115 | tags=34%, list=18%, signal=42% |
| 2269 | GOCC\_MEMBRANE\_COAT |  | 75 | 0.30 | 1.13 | 0.244 | 0.741 | 1.000 | 3458 | tags=44%, list=29%, signal=61% |
| 2270 | GOBP\_PRESYNAPTIC\_DENSE\_CORE\_VESICLE\_EXOCYTOSIS |  | 5 | 0.59 | 1.13 | 0.345 | 0.741 | 1.000 | 2339 | tags=80%, list=19%, signal=99% |
| 2271 | GOBP\_POSITIVE\_REGULATION\_OF\_DEFENSE\_RESPONSE |  | 201 | 0.26 | 1.13 | 0.197 | 0.741 | 1.000 | 1561 | tags=16%, list=13%, signal=18% |
| 2272 | GOBP\_RESPONSE\_TO\_COLD |  | 29 | 0.37 | 1.13 | 0.288 | 0.741 | 1.000 | 2138 | tags=31%, list=18%, signal=38% |
| 2273 | GOBP\_NEGATIVE\_REGULATION\_OF\_SODIUM\_ION\_TRANSMEMBRANE\_TRANSPORT |  | 11 | 0.46 | 1.13 | 0.313 | 0.742 | 1.000 | 2604 | tags=45%, list=22%, signal=58% |
| 2274 | GOBP\_AMINO\_ACID\_TRANSPORT |  | 98 | 0.29 | 1.13 | 0.233 | 0.743 | 1.000 | 1429 | tags=20%, list=12%, signal=23% |
| 2275 | GOMF\_DNA\_BINDING\_TRANSCRIPTION\_ACTIVATOR\_ACTIVITY |  | 233 | 0.26 | 1.13 | 0.186 | 0.745 | 1.000 | 1751 | tags=21%, list=15%, signal=25% |
| 2276 | GOBP\_DETECTION\_OF\_BIOTIC\_STIMULUS |  | 19 | 0.40 | 1.13 | 0.308 | 0.745 | 1.000 | 1030 | tags=21%, list=9%, signal=23% |
| 2277 | GOCC\_BLEB |  | 9 | 0.49 | 1.13 | 0.314 | 0.745 | 1.000 | 483 | tags=22%, list=4%, signal=23% |
| 2278 | GOBP\_ENSHEATHMENT\_OF\_NEURONS |  | 92 | 0.29 | 1.13 | 0.235 | 0.745 | 1.000 | 2415 | tags=22%, list=20%, signal=27% |
| 2279 | GOBP\_KILLING\_OF\_CELLS\_IN\_OTHER\_ORGANISM\_INVOLVED\_IN\_SYMBIOTIC\_INTERACTION |  | 7 | 0.53 | 1.13 | 0.329 | 0.745 | 1.000 | 5346 | tags=86%, list=45%, signal=154% |
| 2280 | GOBP\_VASCULAR\_TRANSPORT |  | 63 | 0.31 | 1.13 | 0.252 | 0.745 | 1.000 | 2097 | tags=27%, list=17%, signal=33% |
| 2281 | GOMF\_PEPTIDE\_DISULFIDE\_OXIDOREDUCTASE\_ACTIVITY |  | 11 | 0.47 | 1.13 | 0.318 | 0.746 | 1.000 | 2919 | tags=55%, list=24%, signal=72% |
| 2282 | GOBP\_POSITIVE\_REGULATION\_OF\_PROTEIN\_EXIT\_FROM\_ENDOPLASMIC\_RETICULUM |  | 12 | 0.45 | 1.13 | 0.318 | 0.747 | 1.000 | 892 | tags=17%, list=7%, signal=18% |
| 2283 | GOBP\_TRICUSPID\_VALVE\_MORPHOGENESIS |  | 5 | 0.58 | 1.13 | 0.346 | 0.747 | 1.000 | 1336 | tags=40%, list=11%, signal=45% |
| 2284 | GOCC\_CELL\_CORTEX\_REGION |  | 33 | 0.36 | 1.13 | 0.285 | 0.747 | 1.000 | 3115 | tags=39%, list=26%, signal=53% |
| 2285 | GOBP\_REGULATION\_OF\_LEUKOCYTE\_MIGRATION |  | 133 | 0.28 | 1.13 | 0.224 | 0.747 | 1.000 | 2458 | tags=29%, list=20%, signal=36% |
| 2286 | GOBP\_REGULATION\_OF\_T\_HELPER\_1\_TYPE\_IMMUNE\_RESPONSE |  | 18 | 0.41 | 1.13 | 0.312 | 0.747 | 1.000 | 1517 | tags=28%, list=13%, signal=32% |
| 2287 | GOMF\_LIGAND\_ACTIVATED\_TRANSCRIPTION\_FACTOR\_ACTIVITY |  | 39 | 0.34 | 1.13 | 0.276 | 0.747 | 1.000 | 3404 | tags=44%, list=28%, signal=61% |
| 2288 | GOBP\_POSITIVE\_REGULATION\_OF\_TRANSMEMBRANE\_TRANSPORT |  | 135 | 0.27 | 1.13 | 0.221 | 0.747 | 1.000 | 2341 | tags=27%, list=19%, signal=34% |
| 2289 | GOBP\_RESPONSE\_TO\_KETONE |  | 135 | 0.27 | 1.13 | 0.220 | 0.747 | 1.000 | 1517 | tags=18%, list=13%, signal=20% |
| 2290 | GOBP\_REGULATION\_OF\_CYSTEINE\_TYPE\_ENDOPEPTIDASE\_ACTIVITY\_INVOLVED\_IN\_APOPTOTIC\_SIGNALING\_PATHWAY |  | 13 | 0.44 | 1.12 | 0.310 | 0.747 | 1.000 | 2138 | tags=46%, list=18%, signal=56% |
| 2291 | GOBP\_POSITIVE\_REGULATION\_OF\_INTERLEUKIN\_17\_PRODUCTION |  | 7 | 0.53 | 1.12 | 0.339 | 0.747 | 1.000 | 1232 | tags=43%, list=10%, signal=48% |
| 2292 | GOMF\_INTEGRIN\_BINDING |  | 102 | 0.29 | 1.12 | 0.239 | 0.748 | 1.000 | 2354 | tags=28%, list=20%, signal=35% |
| 2293 | GOBP\_LIPID\_TRANSPORT\_ACROSS\_BLOOD\_BRAIN\_BARRIER |  | 5 | 0.58 | 1.12 | 0.353 | 0.748 | 1.000 | 1326 | tags=60%, list=11%, signal=67% |
| 2294 | GOBP\_N\_ACETYLNEURAMINATE\_METABOLIC\_PROCESS |  | 9 | 0.49 | 1.12 | 0.317 | 0.748 | 1.000 | 952 | tags=33%, list=8%, signal=36% |
| 2295 | GOBP\_REGULATION\_OF\_TRAIL\_ACTIVATED\_APOPTOTIC\_SIGNALING\_PATHWAY |  | 4 | 0.62 | 1.12 | 0.354 | 0.748 | 1.000 | 1593 | tags=50%, list=13%, signal=58% |
| 2296 | GOBP\_SOMATIC\_DIVERSIFICATION\_OF\_T\_CELL\_RECEPTOR\_GENES |  | 5 | 0.58 | 1.12 | 0.359 | 0.748 | 1.000 | 1495 | tags=60%, list=12%, signal=69% |
| 2297 | GOMF\_CALCIUM\_ION\_TRANSMEMBRANE\_TRANSPORTER\_ACTIVITY |  | 78 | 0.30 | 1.12 | 0.252 | 0.748 | 1.000 | 1145 | tags=15%, list=10%, signal=17% |
| 2298 | GOBP\_NEGATIVE\_REGULATION\_OF\_CARDIAC\_MUSCLE\_CELL\_DIFFERENTIATION |  | 5 | 0.58 | 1.12 | 0.346 | 0.748 | 1.000 | 1379 | tags=40%, list=11%, signal=45% |
| 2299 | GOBP\_L\_ALANINE\_TRANSPORT |  | 5 | 0.58 | 1.12 | 0.352 | 0.748 | 1.000 | 422 | tags=40%, list=4%, signal=41% |
| 2300 | GOMF\_L\_ALANINE\_TRANSMEMBRANE\_TRANSPORTER\_ACTIVITY |  | 5 | 0.58 | 1.12 | 0.348 | 0.749 | 1.000 | 422 | tags=40%, list=4%, signal=41% |
| 2301 | GOCC\_SPECTRIN |  | 5 | 0.58 | 1.12 | 0.351 | 0.749 | 1.000 | 1375 | tags=60%, list=11%, signal=68% |
| 2302 | GOBP\_VESICLE\_TARGETING\_TO\_FROM\_OR\_WITHIN\_GOLGI |  | 60 | 0.31 | 1.12 | 0.264 | 0.749 | 1.000 | 3458 | tags=40%, list=29%, signal=56% |
| 2303 | GOBP\_NUCLEOSIDE\_SALVAGE |  | 15 | 0.43 | 1.12 | 0.308 | 0.749 | 1.000 | 1179 | tags=20%, list=10%, signal=22% |
| 2304 | GOBP\_MODULATION\_BY\_SYMBIONT\_OF\_ENTRY\_INTO\_HOST |  | 19 | 0.40 | 1.12 | 0.306 | 0.749 | 1.000 | 957 | tags=21%, list=8%, signal=23% |
| 2305 | GOBP\_CELLULAR\_RESPONSE\_TO\_BACTERIAL\_LIPOPROTEIN |  | 3 | 0.67 | 1.12 | 0.368 | 0.749 | 1.000 | 3000 | tags=67%, list=25%, signal=89% |
| 2306 | GOBP\_TRANSLESION\_SYNTHESIS |  | 32 | 0.35 | 1.12 | 0.284 | 0.749 | 1.000 | 848 | tags=16%, list=7%, signal=17% |
| 2307 | GOMF\_1\_PHOSPHATIDYLINOSITOL\_3\_KINASE\_REGULATOR\_ACTIVITY |  | 13 | 0.45 | 1.12 | 0.314 | 0.749 | 1.000 | 686 | tags=23%, list=6%, signal=24% |
| 2308 | GOBP\_POSITIVE\_REGULATION\_OF\_HYDROLASE\_ACTIVITY |  | 543 | 0.24 | 1.12 | 0.144 | 0.749 | 1.000 | 2843 | tags=28%, list=24%, signal=35% |
| 2309 | GOBP\_POSITIVE\_REGULATION\_OF\_NEUTROPHIL\_MIGRATION |  | 23 | 0.39 | 1.12 | 0.308 | 0.749 | 1.000 | 1986 | tags=30%, list=17%, signal=36% |
| 2310 | GOMF\_PHOSPHATIDYLCHOLINE\_TRANSPORTER\_ACTIVITY |  | 13 | 0.44 | 1.12 | 0.317 | 0.748 | 1.000 | 1319 | tags=31%, list=11%, signal=35% |
| 2311 | GOBP\_REGULATION\_OF\_CELL\_CYCLE |  | 867 | 0.23 | 1.12 | 0.108 | 0.748 | 1.000 | 3884 | tags=37%, list=32%, signal=51% |
| 2312 | GOBP\_REGULATION\_OF\_BILE\_ACID\_METABOLIC\_PROCESS |  | 11 | 0.47 | 1.12 | 0.310 | 0.748 | 1.000 | 1264 | tags=27%, list=11%, signal=30% |
| 2313 | GOBP\_REGULATION\_OF\_CELL\_CYCLE\_PHASE\_TRANSITION |  | 350 | 0.25 | 1.12 | 0.165 | 0.748 | 1.000 | 4084 | tags=41%, list=34%, signal=61% |
| 2314 | GOBP\_ORGANELLE\_INHERITANCE |  | 13 | 0.44 | 1.12 | 0.312 | 0.748 | 1.000 | 2652 | tags=38%, list=22%, signal=49% |
| 2315 | GOBP\_RESPONSE\_TO\_ENDOPLASMIC\_RETICULUM\_STRESS |  | 224 | 0.26 | 1.12 | 0.201 | 0.749 | 1.000 | 2919 | tags=29%, list=24%, signal=37% |
| 2316 | GOCC\_DENTATE\_GYRUS\_MOSSY\_FIBER |  | 5 | 0.58 | 1.12 | 0.361 | 0.748 | 1.000 | 1969 | tags=40%, list=16%, signal=48% |
| 2317 | GOMF\_PHOSPHATIDYLGLYCEROL\_BINDING |  | 10 | 0.47 | 1.12 | 0.326 | 0.749 | 1.000 | 943 | tags=30%, list=8%, signal=33% |
| 2318 | GOBP\_CELLULAR\_RESPONSE\_TO\_BIOTIC\_STIMULUS |  | 130 | 0.28 | 1.12 | 0.225 | 0.749 | 1.000 | 2167 | tags=23%, list=18%, signal=28% |
| 2319 | GOBP\_NEGATIVE\_REGULATION\_OF\_PROTEIN\_CONTAINING\_COMPLEX\_ASSEMBLY |  | 98 | 0.29 | 1.12 | 0.257 | 0.749 | 1.000 | 2234 | tags=22%, list=19%, signal=27% |
| 2320 | GOCC\_CENTROSOME |  | 461 | 0.24 | 1.12 | 0.144 | 0.749 | 1.000 | 3972 | tags=39%, list=33%, signal=55% |
| 2321 | GOBP\_POSITIVE\_REGULATION\_OF\_CARDIAC\_MUSCLE\_CELL\_PROLIFERATION |  | 15 | 0.43 | 1.12 | 0.318 | 0.749 | 1.000 | 4256 | tags=73%, list=35%, signal=113% |
| 2322 | GOBP\_VASCULAR\_ASSOCIATED\_SMOOTH\_MUSCLE\_CONTRACTION |  | 23 | 0.38 | 1.12 | 0.301 | 0.749 | 1.000 | 2845 | tags=48%, list=24%, signal=63% |
| 2323 | GOBP\_PROTEIN\_HOMOTETRAMERIZATION |  | 38 | 0.34 | 1.12 | 0.286 | 0.748 | 1.000 | 972 | tags=18%, list=8%, signal=20% |
| 2324 | GOMF\_MUTSALPHA\_COMPLEX\_BINDING |  | 6 | 0.55 | 1.12 | 0.342 | 0.750 | 1.000 | 1894 | tags=50%, list=16%, signal=59% |
| 2325 | GOBP\_MESODERM\_DEVELOPMENT |  | 71 | 0.30 | 1.12 | 0.262 | 0.750 | 1.000 | 1909 | tags=21%, list=16%, signal=25% |
| 2326 | GOBP\_ENDOCARDIUM\_MORPHOGENESIS |  | 5 | 0.58 | 1.12 | 0.350 | 0.751 | 1.000 | 1394 | tags=40%, list=12%, signal=45% |
| 2327 | GOBP\_HETEROCHROMATIN\_ORGANIZATION |  | 40 | 0.34 | 1.12 | 0.293 | 0.751 | 1.000 | 1770 | tags=20%, list=15%, signal=23% |
| 2328 | GOBP\_NUCLEOBASE\_CONTAINING\_SMALL\_MOLECULE\_METABOLIC\_PROCESS |  | 424 | 0.24 | 1.12 | 0.164 | 0.750 | 1.000 | 1700 | tags=17%, list=14%, signal=19% |
| 2329 | GOBP\_DNA\_SYNTHESIS\_INVOLVED\_IN\_DNA\_REPAIR |  | 37 | 0.34 | 1.12 | 0.286 | 0.751 | 1.000 | 3479 | tags=43%, list=29%, signal=61% |
| 2330 | GOBP\_CELL\_CYCLE\_G1\_S\_PHASE\_TRANSITION |  | 209 | 0.26 | 1.12 | 0.203 | 0.752 | 1.000 | 3466 | tags=34%, list=29%, signal=47% |
| 2331 | GOBP\_LONG\_CHAIN\_FATTY\_ACID\_METABOLIC\_PROCESS |  | 66 | 0.31 | 1.12 | 0.262 | 0.752 | 1.000 | 1433 | tags=20%, list=12%, signal=22% |
| 2332 | GOBP\_HINDLIMB\_MORPHOGENESIS |  | 20 | 0.39 | 1.12 | 0.307 | 0.752 | 1.000 | 5 | tags=10%, list=0%, signal=10% |
| 2333 | GOBP\_FOCAL\_ADHESION\_ASSEMBLY |  | 72 | 0.30 | 1.12 | 0.269 | 0.752 | 1.000 | 3900 | tags=50%, list=32%, signal=74% |
| 2334 | GOMF\_KINETOCHORE\_BINDING |  | 6 | 0.55 | 1.12 | 0.350 | 0.752 | 1.000 | 3866 | tags=83%, list=32%, signal=123% |
| 2335 | GOBP\_TYPE\_II\_PNEUMOCYTE\_DIFFERENTIATION |  | 4 | 0.61 | 1.12 | 0.370 | 0.754 | 1.000 | 2274 | tags=50%, list=19%, signal=62% |
| 2336 | GOBP\_MRNA\_5\_SPLICE\_SITE\_RECOGNITION |  | 5 | 0.58 | 1.12 | 0.353 | 0.754 | 1.000 | 4788 | tags=80%, list=40%, signal=133% |
| 2337 | GOCC\_NLRP3\_INFLAMMASOME\_COMPLEX |  | 5 | 0.58 | 1.12 | 0.350 | 0.754 | 1.000 | 1219 | tags=40%, list=10%, signal=44% |
| 2338 | GOBP\_POSITIVE\_REGULATION\_OF\_DENDRITIC\_SPINE\_MORPHOGENESIS |  | 15 | 0.43 | 1.12 | 0.318 | 0.754 | 1.000 | 4567 | tags=67%, list=38%, signal=107% |
| 2339 | GOBP\_AMYLOID\_BETA\_CLEARANCE |  | 26 | 0.37 | 1.12 | 0.299 | 0.753 | 1.000 | 1326 | tags=19%, list=11%, signal=22% |
| 2340 | GOMF\_CARBOXYLIC\_ESTER\_HYDROLASE\_ACTIVITY |  | 82 | 0.30 | 1.12 | 0.258 | 0.753 | 1.000 | 1857 | tags=27%, list=15%, signal=32% |
| 2341 | GOCC\_PERINUCLEAR\_REGION\_OF\_CYTOPLASM |  | 522 | 0.24 | 1.12 | 0.150 | 0.753 | 1.000 | 2058 | tags=21%, list=17%, signal=24% |
| 2342 | GOBP\_SOMITE\_SPECIFICATION |  | 3 | 0.67 | 1.12 | 0.380 | 0.753 | 1.000 | 2918 | tags=67%, list=24%, signal=88% |
| 2343 | GOMF\_SODIUM\_ION\_TRANSMEMBRANE\_TRANSPORTER\_ACTIVITY |  | 81 | 0.30 | 1.12 | 0.267 | 0.753 | 1.000 | 1546 | tags=26%, list=13%, signal=30% |
| 2344 | GOBP\_NEGATIVE\_REGULATION\_OF\_HEMOPOIESIS |  | 64 | 0.31 | 1.12 | 0.270 | 0.753 | 1.000 | 2457 | tags=27%, list=20%, signal=33% |
| 2345 | GOCC\_TRANSPORT\_VESICLE |  | 267 | 0.25 | 1.12 | 0.207 | 0.753 | 1.000 | 2490 | tags=29%, list=21%, signal=36% |
| 2346 | GOBP\_AXON\_ENSHEATHMENT\_IN\_CENTRAL\_NERVOUS\_SYSTEM |  | 11 | 0.46 | 1.12 | 0.319 | 0.753 | 1.000 | 2415 | tags=36%, list=20%, signal=45% |
| 2347 | GOCC\_ORIGIN\_RECOGNITION\_COMPLEX |  | 8 | 0.50 | 1.12 | 0.341 | 0.753 | 1.000 | 3102 | tags=50%, list=26%, signal=67% |
| 2348 | GOBP\_PROTEIN\_ACTIVATION\_CASCADE |  | 17 | 0.41 | 1.12 | 0.320 | 0.753 | 1.000 | 1033 | tags=35%, list=9%, signal=39% |
| 2349 | GOBP\_WALKING\_BEHAVIOR |  | 14 | 0.43 | 1.12 | 0.317 | 0.754 | 1.000 | 1035 | tags=14%, list=9%, signal=16% |
| 2350 | GOBP\_POSITIVE\_REGULATION\_OF\_TRANSLATION\_IN\_RESPONSE\_TO\_STRESS |  | 6 | 0.55 | 1.12 | 0.359 | 0.754 | 1.000 | 2863 | tags=33%, list=24%, signal=44% |
| 2351 | GOBP\_ESTABLISHMENT\_OF\_MITOTIC\_SPINDLE\_LOCALIZATION |  | 30 | 0.36 | 1.12 | 0.303 | 0.753 | 1.000 | 3803 | tags=57%, list=32%, signal=83% |
| 2352 | GOMF\_TUBULIN\_BINDING |  | 266 | 0.25 | 1.12 | 0.197 | 0.753 | 1.000 | 2295 | tags=25%, list=19%, signal=30% |
| 2353 | GOBP\_AMEBOIDAL\_TYPE\_CELL\_MIGRATION |  | 316 | 0.25 | 1.12 | 0.196 | 0.753 | 1.000 | 2548 | tags=25%, list=21%, signal=31% |
| 2354 | GOMF\_PROTEIN\_TYROSINE\_KINASE\_ACTIVATOR\_ACTIVITY |  | 13 | 0.44 | 1.12 | 0.331 | 0.753 | 1.000 | 1887 | tags=38%, list=16%, signal=46% |
| 2355 | GOBP\_REGULATION\_OF\_ANOIKIS |  | 16 | 0.42 | 1.12 | 0.317 | 0.753 | 1.000 | 2340 | tags=44%, list=19%, signal=54% |
| 2356 | GOBP\_REGULATION\_OF\_CGMP\_MEDIATED\_SIGNALING |  | 5 | 0.58 | 1.12 | 0.369 | 0.753 | 1.000 | 1386 | tags=40%, list=12%, signal=45% |
| 2357 | GOBP\_NEGATIVE\_REGULATION\_OF\_INTERLEUKIN\_1\_BETA\_PRODUCTION |  | 14 | 0.43 | 1.12 | 0.324 | 0.753 | 1.000 | 1219 | tags=29%, list=10%, signal=32% |
| 2358 | GOMF\_BETA\_1\_3\_GALACTOSYLTRANSFERASE\_ACTIVITY |  | 5 | 0.58 | 1.12 | 0.369 | 0.753 | 1.000 | 110 | tags=20%, list=1%, signal=20% |
| 2359 | GOBP\_POSITIVE\_REGULATION\_OF\_ACTIVIN\_RECEPTOR\_SIGNALING\_PATHWAY |  | 4 | 0.62 | 1.12 | 0.372 | 0.753 | 1.000 | 4337 | tags=50%, list=36%, signal=78% |
| 2360 | GOBP\_REGULATION\_OF\_INWARD\_RECTIFIER\_POTASSIUM\_CHANNEL\_ACTIVITY |  | 4 | 0.62 | 1.12 | 0.367 | 0.753 | 1.000 | 3030 | tags=75%, list=25%, signal=100% |
| 2361 | GOCC\_INFLAMMASOME\_COMPLEX |  | 5 | 0.58 | 1.12 | 0.366 | 0.753 | 1.000 | 1219 | tags=40%, list=10%, signal=44% |
| 2362 | GOBP\_NEGATIVE\_REGULATION\_OF\_LEUKOCYTE\_ADHESION\_TO\_VASCULAR\_ENDOTHELIAL\_CELL |  | 3 | 0.67 | 1.11 | 0.383 | 0.754 | 1.000 | 1625 | tags=67%, list=14%, signal=77% |
| 2363 | GOBP\_NEURON\_PROJECTION\_ARBORIZATION |  | 21 | 0.39 | 1.11 | 0.318 | 0.754 | 1.000 | 1096 | tags=19%, list=9%, signal=21% |
| 2364 | GOBP\_ERYTHROCYTE\_HOMEOSTASIS |  | 95 | 0.29 | 1.11 | 0.262 | 0.754 | 1.000 | 1909 | tags=22%, list=16%, signal=26% |
| 2365 | GOBP\_DEVELOPMENT\_OF\_PRIMARY\_FEMALE\_SEXUAL\_CHARACTERISTICS |  | 63 | 0.31 | 1.11 | 0.268 | 0.754 | 1.000 | 3133 | tags=35%, list=26%, signal=47% |
| 2366 | GOBP\_NEGATIVE\_REGULATION\_OF\_PRI\_MIRNA\_TRANSCRIPTION\_BY\_RNA\_POLYMERASE\_II |  | 10 | 0.47 | 1.11 | 0.342 | 0.755 | 1.000 | 2351 | tags=40%, list=20%, signal=50% |
| 2367 | GOBP\_CELLULAR\_RESPONSE\_TO\_CHEMICAL\_STRESS |  | 253 | 0.25 | 1.11 | 0.208 | 0.756 | 1.000 | 1612 | tags=20%, list=13%, signal=23% |
| 2368 | GOBP\_POSITIVE\_REGULATION\_OF\_P38MAPK\_CASCADE |  | 20 | 0.40 | 1.11 | 0.328 | 0.756 | 1.000 | 1809 | tags=35%, list=15%, signal=41% |
| 2369 | GOBP\_AMINO\_ACID\_BETAINE\_TRANSPORT |  | 5 | 0.58 | 1.11 | 0.368 | 0.756 | 1.000 | 616 | tags=40%, list=5%, signal=42% |
| 2370 | GOMF\_TUMOR\_NECROSIS\_FACTOR\_ACTIVATED\_RECEPTOR\_ACTIVITY |  | 10 | 0.47 | 1.11 | 0.336 | 0.756 | 1.000 | 2113 | tags=40%, list=18%, signal=48% |
| 2371 | GOBP\_MUSCLE\_CELL\_PROLIFERATION |  | 136 | 0.27 | 1.11 | 0.236 | 0.756 | 1.000 | 2241 | tags=24%, list=19%, signal=29% |
| 2372 | GOBP\_MITOCHONDRIAL\_DEPOLARIZATION |  | 19 | 0.40 | 1.11 | 0.318 | 0.757 | 1.000 | 231 | tags=16%, list=2%, signal=16% |
| 2373 | GOMF\_GLUCOCORTICOID\_RECEPTOR\_BINDING |  | 11 | 0.47 | 1.11 | 0.336 | 0.757 | 1.000 | 1067 | tags=27%, list=9%, signal=30% |
| 2374 | GOBP\_GLUTAMATE\_HOMEOSTASIS |  | 3 | 0.66 | 1.11 | 0.373 | 0.757 | 1.000 | 831 | tags=67%, list=7%, signal=72% |
| 2375 | GOBP\_AMINO\_ACID\_HOMEOSTASIS |  | 3 | 0.66 | 1.11 | 0.376 | 0.757 | 1.000 | 831 | tags=67%, list=7%, signal=72% |
| 2376 | GOBP\_REGULATION\_OF\_CELL\_ADHESION |  | 486 | 0.24 | 1.11 | 0.171 | 0.757 | 1.000 | 2691 | tags=26%, list=22%, signal=32% |
| 2377 | GOBP\_CARDIAC\_MUSCLE\_TISSUE\_DEVELOPMENT |  | 126 | 0.28 | 1.11 | 0.240 | 0.757 | 1.000 | 2478 | tags=25%, list=21%, signal=31% |
| 2378 | GOBP\_DNA\_CATABOLIC\_PROCESS\_ENDONUCLEOLYTIC |  | 18 | 0.40 | 1.11 | 0.319 | 0.756 | 1.000 | 3483 | tags=61%, list=29%, signal=86% |
| 2379 | GOBP\_REGULATION\_OF\_METALLOENDOPEPTIDASE\_ACTIVITY |  | 6 | 0.55 | 1.11 | 0.357 | 0.756 | 1.000 | 1632 | tags=33%, list=14%, signal=39% |
| 2380 | GOBP\_CARDIAC\_MUSCLE\_CELL\_DIFFERENTIATION |  | 66 | 0.30 | 1.11 | 0.280 | 0.756 | 1.000 | 2351 | tags=27%, list=20%, signal=34% |
| 2381 | GOBP\_POSITIVE\_REGULATION\_OF\_VASCULAR\_PERMEABILITY |  | 10 | 0.47 | 1.11 | 0.344 | 0.756 | 1.000 | 1891 | tags=40%, list=16%, signal=47% |
| 2382 | GOBP\_REGULATION\_OF\_VASCULAR\_ASSOCIATED\_SMOOTH\_MUSCLE\_CELL\_PROLIFERATION |  | 42 | 0.33 | 1.11 | 0.293 | 0.756 | 1.000 | 1336 | tags=19%, list=11%, signal=21% |
| 2383 | GOBP\_NEGATIVE\_REGULATION\_OF\_CAMP\_DEPENDENT\_PROTEIN\_KINASE\_ACTIVITY |  | 9 | 0.49 | 1.11 | 0.339 | 0.757 | 1.000 | 925 | tags=22%, list=8%, signal=24% |
| 2384 | GOBP\_POSITIVE\_REGULATION\_OF\_CD4\_POSITIVE\_ALPHA\_BETA\_T\_CELL\_PROLIFERATION |  | 4 | 0.61 | 1.11 | 0.375 | 0.757 | 1.000 | 1968 | tags=50%, list=16%, signal=60% |
| 2385 | GOBP\_HEAD\_DEVELOPMENT |  | 502 | 0.24 | 1.11 | 0.169 | 0.757 | 1.000 | 1839 | tags=19%, list=15%, signal=21% |
| 2386 | GOBP\_IMMUNOGLOBULIN\_V\_D\_J\_RECOMBINATION |  | 5 | 0.57 | 1.11 | 0.367 | 0.757 | 1.000 | 1495 | tags=40%, list=12%, signal=46% |
| 2387 | GOMF\_ALPHA\_CATENIN\_BINDING |  | 7 | 0.52 | 1.11 | 0.343 | 0.757 | 1.000 | 7 | tags=14%, list=0%, signal=14% |
| 2388 | GOBP\_MIRNA\_LOADING\_ONTO\_RISC\_INVOLVED\_IN\_GENE\_SILENCING\_BY\_MIRNA |  | 5 | 0.57 | 1.11 | 0.367 | 0.757 | 1.000 | 412 | tags=20%, list=3%, signal=21% |
| 2389 | GOCC\_LAMELLIPODIUM |  | 160 | 0.26 | 1.11 | 0.242 | 0.757 | 1.000 | 2185 | tags=25%, list=18%, signal=30% |
| 2390 | GOCC\_ARP2\_3\_PROTEIN\_COMPLEX |  | 9 | 0.48 | 1.11 | 0.335 | 0.757 | 1.000 | 3211 | tags=56%, list=27%, signal=76% |
| 2391 | GOCC\_MICROBODY\_MEMBRANE |  | 53 | 0.32 | 1.11 | 0.297 | 0.758 | 1.000 | 2081 | tags=25%, list=17%, signal=30% |
| 2392 | GOBP\_NUCLEOSIDE\_BISPHOSPHATE\_METABOLIC\_PROCESS |  | 100 | 0.28 | 1.11 | 0.265 | 0.758 | 1.000 | 1433 | tags=20%, list=12%, signal=23% |
| 2393 | GOBP\_MRNA\_SPLICE\_SITE\_SELECTION |  | 24 | 0.38 | 1.11 | 0.314 | 0.758 | 1.000 | 4078 | tags=58%, list=34%, signal=88% |
| 2394 | GOBP\_NEGATIVE\_REGULATION\_OF\_FAT\_CELL\_DIFFERENTIATION |  | 35 | 0.34 | 1.11 | 0.291 | 0.758 | 1.000 | 3399 | tags=46%, list=28%, signal=64% |
| 2395 | GOBP\_OUTFLOW\_TRACT\_MORPHOGENESIS |  | 53 | 0.32 | 1.11 | 0.290 | 0.759 | 1.000 | 2545 | tags=30%, list=21%, signal=38% |
| 2396 | GOBP\_CALCIUM\_ION\_EXPORT\_ACROSS\_PLASMA\_MEMBRANE |  | 4 | 0.61 | 1.11 | 0.382 | 0.759 | 1.000 | 126 | tags=25%, list=1%, signal=25% |
| 2397 | GOBP\_SKELETAL\_MUSCLE\_SATELLITE\_CELL\_DIFFERENTIATION |  | 5 | 0.58 | 1.11 | 0.373 | 0.759 | 1.000 | 507 | tags=40%, list=4%, signal=42% |
| 2398 | GOBP\_CELLULAR\_RESPONSE\_TO\_AMMONIUM\_ION |  | 3 | 0.66 | 1.11 | 0.391 | 0.759 | 1.000 | 831 | tags=33%, list=7%, signal=36% |
| 2399 | GOBP\_DICHOTOMOUS\_SUBDIVISION\_OF\_AN\_EPITHELIAL\_TERMINAL\_UNIT |  | 7 | 0.52 | 1.11 | 0.362 | 0.759 | 1.000 | 263 | tags=14%, list=2%, signal=15% |
| 2400 | GOBP\_METANEPHRIC\_EPITHELIUM\_DEVELOPMENT |  | 15 | 0.42 | 1.11 | 0.327 | 0.758 | 1.000 | 2680 | tags=40%, list=22%, signal=51% |
| 2401 | GOBP\_REGULATION\_OF\_AUTOPHAGY\_OF\_MITOCHONDRION |  | 26 | 0.37 | 1.11 | 0.315 | 0.758 | 1.000 | 2013 | tags=31%, list=17%, signal=37% |
| 2402 | GOCC\_ALPHA\_DNA\_POLYMERASE\_PRIMASE\_COMPLEX |  | 4 | 0.62 | 1.11 | 0.381 | 0.758 | 1.000 | 4619 | tags=100%, list=38%, signal=162% |
| 2403 | GOBP\_NEGATIVE\_REGULATION\_OF\_SMAD\_PROTEIN\_COMPLEX\_ASSEMBLY |  | 4 | 0.61 | 1.11 | 0.376 | 0.758 | 1.000 | 3445 | tags=75%, list=29%, signal=105% |
| 2404 | GOBP\_CELL\_MATRIX\_ADHESION |  | 165 | 0.27 | 1.11 | 0.240 | 0.758 | 1.000 | 3600 | tags=41%, list=30%, signal=57% |
| 2405 | GOMF\_HORMONE\_BINDING |  | 53 | 0.32 | 1.11 | 0.292 | 0.758 | 1.000 | 1427 | tags=23%, list=12%, signal=26% |
| 2406 | GOMF\_EPIDERMAL\_GROWTH\_FACTOR\_RECEPTOR\_BINDING |  | 26 | 0.37 | 1.11 | 0.311 | 0.758 | 1.000 | 2712 | tags=35%, list=23%, signal=45% |
| 2407 | GOBP\_REGULATION\_OF\_HYDROLASE\_ACTIVITY |  | 847 | 0.23 | 1.11 | 0.141 | 0.758 | 1.000 | 2843 | tags=27%, list=24%, signal=32% |
| 2408 | GOBP\_POSITIVE\_REGULATION\_OF\_PHOSPHOLIPID\_METABOLIC\_PROCESS |  | 10 | 0.47 | 1.11 | 0.340 | 0.758 | 1.000 | 1917 | tags=50%, list=16%, signal=59% |
| 2409 | GOBP\_VASCULAR\_PROCESS\_IN\_CIRCULATORY\_SYSTEM |  | 178 | 0.26 | 1.11 | 0.232 | 0.758 | 1.000 | 2097 | tags=25%, list=17%, signal=30% |
| 2410 | GOMF\_DEAMINASE\_ACTIVITY |  | 18 | 0.40 | 1.11 | 0.320 | 0.758 | 1.000 | 1086 | tags=22%, list=9%, signal=24% |
| 2411 | GOBP\_NEGATIVE\_REGULATION\_OF\_DENDRITE\_MORPHOGENESIS |  | 6 | 0.54 | 1.11 | 0.366 | 0.758 | 1.000 | 520 | tags=17%, list=4%, signal=17% |
| 2412 | GOBP\_BRANCHING\_INVOLVED\_IN\_MAMMARY\_GLAND\_DUCT\_MORPHOGENESIS |  | 16 | 0.42 | 1.11 | 0.330 | 0.758 | 1.000 | 3 | tags=6%, list=0%, signal=6% |
| 2413 | GOBP\_POST\_EMBRYONIC\_EYE\_MORPHOGENESIS |  | 3 | 0.66 | 1.11 | 0.373 | 0.759 | 1.000 | 4098 | tags=100%, list=34%, signal=152% |
| 2414 | GOBP\_AMYLOID\_BETA\_CLEARANCE\_BY\_TRANSCYTOSIS |  | 7 | 0.52 | 1.11 | 0.355 | 0.759 | 1.000 | 3111 | tags=57%, list=26%, signal=77% |
| 2415 | GOBP\_NEGATIVE\_REGULATION\_OF\_ISOTYPE\_SWITCHING |  | 4 | 0.61 | 1.11 | 0.378 | 0.759 | 1.000 | 4686 | tags=100%, list=39%, signal=164% |
| 2416 | GOBP\_DIGESTIVE\_SYSTEM\_DEVELOPMENT |  | 91 | 0.29 | 1.11 | 0.280 | 0.759 | 1.000 | 1604 | tags=16%, list=13%, signal=19% |
| 2417 | GOBP\_INTERLEUKIN\_6\_MEDIATED\_SIGNALING\_PATHWAY |  | 19 | 0.39 | 1.11 | 0.322 | 0.760 | 1.000 | 2602 | tags=32%, list=22%, signal=40% |
| 2418 | GOBP\_PEPTIDYL\_SERINE\_MODIFICATION |  | 231 | 0.25 | 1.11 | 0.224 | 0.759 | 1.000 | 2683 | tags=25%, list=22%, signal=32% |
| 2419 | GOMF\_KINASE\_INHIBITOR\_ACTIVITY |  | 53 | 0.32 | 1.11 | 0.287 | 0.760 | 1.000 | 2474 | tags=28%, list=21%, signal=35% |
| 2420 | GOBP\_NEURON\_PROJECTION\_EXTENSION\_INVOLVED\_IN\_NEURON\_PROJECTION\_GUIDANCE |  | 27 | 0.37 | 1.11 | 0.315 | 0.760 | 1.000 | 3037 | tags=44%, list=25%, signal=59% |
| 2421 | GOMF\_CIS\_REGULATORY\_REGION\_SEQUENCE\_SPECIFIC\_DNA\_BINDING |  | 507 | 0.23 | 1.11 | 0.172 | 0.761 | 1.000 | 1893 | tags=19%, list=16%, signal=22% |
| 2422 | GOBP\_CYTOKINE\_PRODUCTION\_INVOLVED\_IN\_IMMUNE\_RESPONSE |  | 56 | 0.31 | 1.11 | 0.286 | 0.761 | 1.000 | 1326 | tags=18%, list=11%, signal=20% |
| 2423 | GOMF\_G\_QUADRUPLEX\_DNA\_BINDING |  | 10 | 0.47 | 1.11 | 0.344 | 0.761 | 1.000 | 2359 | tags=40%, list=20%, signal=50% |
| 2424 | GOBP\_POSITIVE\_REGULATION\_OF\_CATALYTIC\_ACTIVITY |  | 996 | 0.22 | 1.11 | 0.127 | 0.761 | 1.000 | 2776 | tags=26%, list=23%, signal=31% |
| 2425 | GOBP\_ORGANOPHOSPHATE\_BIOSYNTHETIC\_PROCESS |  | 421 | 0.24 | 1.11 | 0.189 | 0.761 | 1.000 | 2154 | tags=22%, list=18%, signal=26% |
| 2426 | GOCC\_SCF\_UBIQUITIN\_LIGASE\_COMPLEX |  | 47 | 0.32 | 1.11 | 0.302 | 0.761 | 1.000 | 1877 | tags=19%, list=16%, signal=23% |
| 2427 | GOBP\_NEGATIVE\_REGULATION\_OF\_CGMP\_MEDIATED\_SIGNALING |  | 3 | 0.66 | 1.11 | 0.384 | 0.760 | 1.000 | 4047 | tags=100%, list=34%, signal=151% |
| 2428 | GOBP\_KERATINOCYTE\_MIGRATION |  | 15 | 0.42 | 1.11 | 0.330 | 0.760 | 1.000 | 2440 | tags=33%, list=20%, signal=42% |
| 2429 | GOBP\_RESPONSE\_TO\_INTERLEUKIN\_9 |  | 7 | 0.52 | 1.11 | 0.360 | 0.761 | 1.000 | 1942 | tags=43%, list=16%, signal=51% |
| 2430 | GOBP\_ORGANIC\_CATION\_TRANSPORT |  | 22 | 0.38 | 1.11 | 0.325 | 0.760 | 1.000 | 616 | tags=18%, list=5%, signal=19% |
| 2431 | GOBP\_POSITIVE\_REGULATION\_OF\_STEROID\_METABOLIC\_PROCESS |  | 15 | 0.42 | 1.10 | 0.328 | 0.761 | 1.000 | 1419 | tags=40%, list=12%, signal=45% |
| 2432 | GOBP\_NATURAL\_KILLER\_CELL\_CHEMOTAXIS |  | 6 | 0.54 | 1.10 | 0.369 | 0.761 | 1.000 | 438 | tags=33%, list=4%, signal=35% |
| 2433 | GOBP\_REGULATION\_OF\_CDC42\_PROTEIN\_SIGNAL\_TRANSDUCTION |  | 4 | 0.61 | 1.10 | 0.388 | 0.761 | 1.000 | 1755 | tags=50%, list=15%, signal=59% |
| 2434 | GOBP\_CHROMOSOME\_ATTACHMENT\_TO\_THE\_NUCLEAR\_ENVELOPE |  | 3 | 0.66 | 1.10 | 0.389 | 0.761 | 1.000 | 969 | tags=33%, list=8%, signal=36% |
| 2435 | GOBP\_RESPONSE\_TO\_MECHANICAL\_STIMULUS |  | 132 | 0.27 | 1.10 | 0.261 | 0.761 | 1.000 | 3700 | tags=41%, list=31%, signal=58% |
| 2436 | GOBP\_NEGATIVE\_REGULATION\_OF\_INTERLEUKIN\_2\_PRODUCTION |  | 19 | 0.40 | 1.10 | 0.333 | 0.761 | 1.000 | 800 | tags=16%, list=7%, signal=17% |
| 2437 | GOMF\_PRIMARY\_ACTIVE\_TRANSMEMBRANE\_TRANSPORTER\_ACTIVITY |  | 42 | 0.33 | 1.10 | 0.311 | 0.761 | 1.000 | 1578 | tags=21%, list=13%, signal=25% |
| 2438 | GOBP\_POSITIVE\_REGULATION\_OF\_DNA\_METABOLIC\_PROCESS |  | 142 | 0.27 | 1.10 | 0.256 | 0.761 | 1.000 | 1936 | tags=20%, list=16%, signal=23% |
| 2439 | GOBP\_CELL\_CELL\_ADHESION |  | 527 | 0.23 | 1.10 | 0.183 | 0.760 | 1.000 | 1794 | tags=18%, list=15%, signal=20% |
| 2440 | GOBP\_VESICLE\_TETHERING |  | 7 | 0.52 | 1.10 | 0.349 | 0.760 | 1.000 | 2438 | tags=57%, list=20%, signal=72% |
| 2441 | GOBP\_REGULATION\_OF\_POSTSYNAPTIC\_CYTOSOLIC\_CALCIUM\_ION\_CONCENTRATION |  | 6 | 0.54 | 1.10 | 0.361 | 0.760 | 1.000 | 4276 | tags=67%, list=36%, signal=103% |
| 2442 | GOBP\_REGULATION\_OF\_KIDNEY\_DEVELOPMENT |  | 19 | 0.40 | 1.10 | 0.324 | 0.760 | 1.000 | 2683 | tags=47%, list=22%, signal=61% |
| 2443 | GOMF\_PHOSPHOLIPASE\_C\_ACTIVITY |  | 22 | 0.38 | 1.10 | 0.323 | 0.760 | 1.000 | 1426 | tags=27%, list=12%, signal=31% |
| 2444 | GOBP\_NEUROTRANSMITTER\_RECEPTOR\_LOCALIZATION\_TO\_POSTSYNAPTIC\_SPECIALIZATION\_MEMBRANE |  | 10 | 0.47 | 1.10 | 0.346 | 0.760 | 1.000 | 1837 | tags=30%, list=15%, signal=35% |
| 2445 | GOMF\_L\_HISTIDINE\_TRANSMEMBRANE\_TRANSPORTER\_ACTIVITY |  | 4 | 0.61 | 1.10 | 0.387 | 0.760 | 1.000 | 169 | tags=25%, list=1%, signal=25% |
| 2446 | GOMF\_PHOSPHATIDYLINOSITOL\_PHOSPHATE\_BINDING |  | 119 | 0.27 | 1.10 | 0.262 | 0.760 | 1.000 | 2050 | tags=24%, list=17%, signal=28% |
| 2447 | GOBP\_LATE\_ENDOSOME\_TO\_VACUOLE\_TRANSPORT |  | 21 | 0.38 | 1.10 | 0.323 | 0.761 | 1.000 | 3802 | tags=52%, list=32%, signal=77% |
| 2448 | GOBP\_CARDIAC\_MUSCLE\_CONTRACTION |  | 80 | 0.29 | 1.10 | 0.283 | 0.761 | 1.000 | 1436 | tags=20%, list=12%, signal=23% |
| 2449 | GOCC\_BASAL\_DENDRITE |  | 5 | 0.57 | 1.10 | 0.377 | 0.763 | 1.000 | 644 | tags=20%, list=5%, signal=21% |
| 2450 | GOCC\_ENDOPLASMIC\_RETICULUM\_PROTEIN\_CONTAINING\_COMPLEX |  | 94 | 0.28 | 1.10 | 0.279 | 0.763 | 1.000 | 2873 | tags=32%, list=24%, signal=42% |
| 2451 | GOBP\_REGULATION\_OF\_MODIFICATION\_OF\_POSTSYNAPTIC\_STRUCTURE |  | 6 | 0.54 | 1.10 | 0.361 | 0.763 | 1.000 | 2722 | tags=50%, list=23%, signal=65% |
| 2452 | GOBP\_NEGATIVE\_REGULATION\_OF\_HOMOTYPIC\_CELL\_CELL\_ADHESION |  | 10 | 0.47 | 1.10 | 0.341 | 0.763 | 1.000 | 605 | tags=20%, list=5%, signal=21% |
| 2453 | GOMF\_GUANYL\_NUCLEOTIDE\_EXCHANGE\_FACTOR\_ACTIVITY |  | 153 | 0.26 | 1.10 | 0.252 | 0.762 | 1.000 | 2053 | tags=24%, list=17%, signal=28% |
| 2454 | GOBP\_MITOTIC\_G2\_M\_TRANSITION\_CHECKPOINT |  | 32 | 0.35 | 1.10 | 0.315 | 0.763 | 1.000 | 4413 | tags=50%, list=37%, signal=79% |
| 2455 | GOBP\_NEUTROPHIL\_MEDIATED\_KILLING\_OF\_GRAM\_NEGATIVE\_BACTERIUM |  | 3 | 0.66 | 1.10 | 0.388 | 0.762 | 1.000 | 4049 | tags=100%, list=34%, signal=151% |
| 2456 | GOBP\_CELLULAR\_METABOLIC\_COMPOUND\_SALVAGE |  | 28 | 0.36 | 1.10 | 0.317 | 0.763 | 1.000 | 556 | tags=11%, list=5%, signal=11% |
| 2457 | GOBP\_L\_ALPHA\_AMINO\_ACID\_TRANSMEMBRANE\_TRANSPORT |  | 36 | 0.34 | 1.10 | 0.312 | 0.763 | 1.000 | 1211 | tags=22%, list=10%, signal=25% |
| 2458 | GOBP\_UDP\_GLUCOSE\_METABOLIC\_PROCESS |  | 5 | 0.56 | 1.10 | 0.380 | 0.763 | 1.000 | 2373 | tags=40%, list=20%, signal=50% |
| 2459 | GOBP\_DNA\_DAMAGE\_RESPONSE\_SIGNAL\_TRANSDUCTION\_BY\_P53\_CLASS\_MEDIATOR |  | 84 | 0.29 | 1.10 | 0.284 | 0.763 | 1.000 | 2597 | tags=26%, list=22%, signal=33% |
| 2460 | GOBP\_RESPONSE\_TO\_AMMONIUM\_ION |  | 3 | 0.66 | 1.10 | 0.404 | 0.763 | 1.000 | 831 | tags=33%, list=7%, signal=36% |
| 2461 | GOBP\_PATHWAY\_RESTRICTED\_SMAD\_PROTEIN\_PHOSPHORYLATION |  | 45 | 0.33 | 1.10 | 0.300 | 0.763 | 1.000 | 1379 | tags=20%, list=11%, signal=23% |
| 2462 | GOBP\_OLIGOPEPTIDE\_TRANSPORT |  | 9 | 0.48 | 1.10 | 0.368 | 0.762 | 1.000 | 312 | tags=22%, list=3%, signal=23% |
| 2463 | GOBP\_ANION\_HOMEOSTASIS |  | 17 | 0.40 | 1.10 | 0.331 | 0.762 | 1.000 | 1150 | tags=29%, list=10%, signal=32% |
| 2464 | GOBP\_REGULATION\_OF\_MITOTIC\_NUCLEAR\_DIVISION |  | 88 | 0.29 | 1.10 | 0.277 | 0.762 | 1.000 | 3959 | tags=49%, list=33%, signal=72% |
| 2465 | GOBP\_POSITIVE\_REGULATION\_OF\_DNA\_LIGATION |  | 4 | 0.61 | 1.10 | 0.381 | 0.763 | 1.000 | 3154 | tags=75%, list=26%, signal=102% |
| 2466 | GOBP\_OVULATION\_CYCLE |  | 44 | 0.33 | 1.10 | 0.304 | 0.763 | 1.000 | 3270 | tags=39%, list=27%, signal=53% |
| 2467 | GOBP\_MULTICELLULAR\_ORGANISMAL\_HOMEOSTASIS |  | 334 | 0.24 | 1.10 | 0.213 | 0.763 | 1.000 | 2000 | tags=23%, list=17%, signal=27% |
| 2468 | GOBP\_REGULATION\_OF\_CARDIAC\_CONDUCTION |  | 28 | 0.36 | 1.10 | 0.324 | 0.763 | 1.000 | 2520 | tags=32%, list=21%, signal=41% |
| 2469 | GOMF\_LYSOPHOSPHATIDIC\_ACID\_BINDING |  | 8 | 0.50 | 1.10 | 0.358 | 0.765 | 1.000 | 1939 | tags=38%, list=16%, signal=45% |
| 2470 | GOBP\_TOLL\_LIKE\_RECEPTOR\_9\_SIGNALING\_PATHWAY |  | 19 | 0.40 | 1.10 | 0.335 | 0.766 | 1.000 | 304 | tags=11%, list=3%, signal=11% |
| 2471 | GOCC\_TRANSPORT\_VESICLE\_MEMBRANE |  | 126 | 0.27 | 1.10 | 0.273 | 0.766 | 1.000 | 2339 | tags=31%, list=19%, signal=38% |
| 2472 | GOMF\_PHOSPHOLIPASE\_A2\_ACTIVITY |  | 13 | 0.43 | 1.10 | 0.343 | 0.766 | 1.000 | 1054 | tags=38%, list=9%, signal=42% |
| 2473 | GOBP\_POSITIVE\_REGULATION\_OF\_FATTY\_ACID\_OXIDATION |  | 12 | 0.45 | 1.10 | 0.359 | 0.766 | 1.000 | 2545 | tags=50%, list=21%, signal=63% |
| 2474 | GOBP\_LACRIMAL\_GLAND\_DEVELOPMENT |  | 5 | 0.58 | 1.10 | 0.382 | 0.766 | 1.000 | 2274 | tags=40%, list=19%, signal=49% |
| 2475 | GOMF\_LONG\_CHAIN\_FATTY\_ACID\_TRANSPORTER\_ACTIVITY |  | 14 | 0.43 | 1.10 | 0.349 | 0.765 | 1.000 | 2389 | tags=50%, list=20%, signal=62% |
| 2476 | GOBP\_NEGATIVE\_REGULATION\_OF\_MYELOID\_CELL\_DIFFERENTIATION |  | 53 | 0.31 | 1.10 | 0.302 | 0.765 | 1.000 | 1373 | tags=21%, list=11%, signal=23% |
| 2477 | GOBP\_PROTEIN\_DEMANNOSYLATION |  | 16 | 0.41 | 1.10 | 0.340 | 0.765 | 1.000 | 2835 | tags=38%, list=24%, signal=49% |
| 2478 | GOMF\_STEROID\_BINDING |  | 68 | 0.30 | 1.10 | 0.287 | 0.766 | 1.000 | 1967 | tags=29%, list=16%, signal=35% |
| 2479 | GOCC\_INTRACILIARY\_TRANSPORT\_PARTICLE |  | 23 | 0.38 | 1.10 | 0.330 | 0.766 | 1.000 | 3223 | tags=48%, list=27%, signal=65% |
| 2480 | GOBP\_NUCLEOSOME\_ASSEMBLY |  | 53 | 0.31 | 1.10 | 0.297 | 0.766 | 1.000 | 3538 | tags=45%, list=29%, signal=64% |
| 2481 | GOBP\_RESPONSE\_TO\_HORMONE |  | 604 | 0.23 | 1.10 | 0.179 | 0.766 | 1.000 | 2013 | tags=20%, list=17%, signal=22% |
| 2482 | GOBP\_DEOXYRIBONUCLEOTIDE\_BIOSYNTHETIC\_PROCESS |  | 13 | 0.43 | 1.10 | 0.352 | 0.766 | 1.000 | 1152 | tags=23%, list=10%, signal=25% |
| 2483 | GOBP\_MINUS\_END\_DIRECTED\_ORGANELLE\_TRANSPORT\_ALONG\_MICROTUBULE |  | 5 | 0.57 | 1.10 | 0.380 | 0.766 | 1.000 | 4814 | tags=80%, list=40%, signal=133% |
| 2484 | GOBP\_NEGATIVE\_REGULATION\_OF\_INTERLEUKIN\_10\_PRODUCTION |  | 6 | 0.54 | 1.10 | 0.369 | 0.766 | 1.000 | 978 | tags=33%, list=8%, signal=36% |
| 2485 | GOMF\_PROTEIN\_LIPID\_COMPLEX\_BINDING |  | 20 | 0.39 | 1.10 | 0.333 | 0.766 | 1.000 | 2095 | tags=30%, list=17%, signal=36% |
| 2486 | GOCC\_HAUS\_COMPLEX |  | 4 | 0.61 | 1.10 | 0.391 | 0.766 | 1.000 | 4209 | tags=75%, list=35%, signal=115% |
| 2487 | GOBP\_SPHINGOMYELIN\_METABOLIC\_PROCESS |  | 14 | 0.42 | 1.10 | 0.345 | 0.766 | 1.000 | 2850 | tags=50%, list=24%, signal=65% |
| 2488 | GOMF\_CAMP\_BINDING |  | 14 | 0.43 | 1.10 | 0.356 | 0.766 | 1.000 | 1190 | tags=29%, list=10%, signal=32% |
| 2489 | GOBP\_EYELID\_DEVELOPMENT\_IN\_CAMERA\_TYPE\_EYE |  | 8 | 0.49 | 1.10 | 0.368 | 0.766 | 1.000 | 855 | tags=25%, list=7%, signal=27% |
| 2490 | GOBP\_OLIGODENDROCYTE\_DEVELOPMENT |  | 27 | 0.36 | 1.10 | 0.334 | 0.766 | 1.000 | 967 | tags=19%, list=8%, signal=20% |
| 2491 | GOBP\_REGULATION\_OF\_VASOCONSTRICTION |  | 45 | 0.32 | 1.10 | 0.315 | 0.766 | 1.000 | 1161 | tags=24%, list=10%, signal=27% |
| 2492 | GOBP\_REGULATION\_OF\_INTRACELLULAR\_LIPID\_TRANSPORT |  | 6 | 0.53 | 1.10 | 0.370 | 0.766 | 1.000 | 3963 | tags=67%, list=33%, signal=99% |
| 2493 | GOBP\_POSITIVE\_REGULATION\_OF\_CD4\_POSITIVE\_ALPHA\_BETA\_T\_CELL\_DIFFERENTIATION |  | 19 | 0.39 | 1.10 | 0.338 | 0.766 | 1.000 | 1517 | tags=26%, list=13%, signal=30% |
| 2494 | GOBP\_LEUKOCYTE\_APOPTOTIC\_PROCESS |  | 71 | 0.30 | 1.10 | 0.298 | 0.766 | 1.000 | 1824 | tags=21%, list=15%, signal=25% |
| 2495 | GOBP\_REGULATION\_OF\_MYELOID\_CELL\_DIFFERENTIATION |  | 158 | 0.26 | 1.10 | 0.270 | 0.766 | 1.000 | 1380 | tags=15%, list=11%, signal=17% |
| 2496 | GOBP\_T\_CELL\_MEDIATED\_CYTOTOXICITY |  | 17 | 0.40 | 1.10 | 0.341 | 0.766 | 1.000 | 1017 | tags=24%, list=8%, signal=26% |
| 2497 | GOBP\_POSITIVE\_REGULATION\_OF\_PROTEIN\_LOCALIZATION\_TO\_CENTROSOME |  | 4 | 0.60 | 1.10 | 0.390 | 0.767 | 1.000 | 3884 | tags=75%, list=32%, signal=111% |
| 2498 | GOBP\_POSITIVE\_REGULATION\_OF\_REACTIVE\_OXYGEN\_SPECIES\_BIOSYNTHETIC\_PROCESS |  | 33 | 0.35 | 1.10 | 0.327 | 0.767 | 1.000 | 1326 | tags=24%, list=11%, signal=27% |
| 2499 | GOBP\_CARDIAC\_SEPTUM\_DEVELOPMENT |  | 77 | 0.29 | 1.10 | 0.284 | 0.766 | 1.000 | 2478 | tags=23%, list=21%, signal=29% |
| 2500 | GOBP\_ADENYLATE\_CYCLASE\_ACTIVATING\_G\_PROTEIN\_COUPLED\_RECEPTOR\_SIGNALING\_PATHWAY |  | 80 | 0.29 | 1.10 | 0.296 | 0.767 | 1.000 | 2083 | tags=25%, list=17%, signal=30% |
| 2501 | GOBP\_NEGATIVE\_REGULATION\_OF\_GLUCOSE\_IMPORT |  | 6 | 0.53 | 1.10 | 0.374 | 0.767 | 1.000 | 3635 | tags=83%, list=30%, signal=119% |
| 2502 | GOMF\_PROTEASE\_BINDING |  | 81 | 0.29 | 1.10 | 0.293 | 0.767 | 1.000 | 1421 | tags=19%, list=12%, signal=21% |
| 2503 | GOBP\_T\_HELPER\_1\_CELL\_CYTOKINE\_PRODUCTION |  | 5 | 0.57 | 1.10 | 0.388 | 0.767 | 1.000 | 1198 | tags=40%, list=10%, signal=44% |
| 2504 | GOBP\_REGULATION\_OF\_EPITHELIAL\_CELL\_MIGRATION |  | 186 | 0.26 | 1.10 | 0.251 | 0.767 | 1.000 | 2548 | tags=24%, list=21%, signal=30% |
| 2505 | GOBP\_PHOSPHATIDIC\_ACID\_METABOLIC\_PROCESS |  | 35 | 0.34 | 1.10 | 0.321 | 0.767 | 1.000 | 1925 | tags=34%, list=16%, signal=41% |
| 2506 | GOMF\_LIPASE\_ACTIVATOR\_ACTIVITY |  | 8 | 0.49 | 1.10 | 0.364 | 0.767 | 1.000 | 1333 | tags=25%, list=11%, signal=28% |
| 2507 | GOBP\_PRONEPHROS\_DEVELOPMENT |  | 4 | 0.61 | 1.10 | 0.398 | 0.767 | 1.000 | 4480 | tags=75%, list=37%, signal=120% |
| 2508 | GOBP\_RUFFLE\_ORGANIZATION |  | 38 | 0.33 | 1.10 | 0.308 | 0.767 | 1.000 | 1977 | tags=26%, list=16%, signal=31% |
| 2509 | GOBP\_HETEROCHROMATIN\_ORGANIZATION\_INVOLVED\_IN\_CHROMATIN\_SILENCING |  | 5 | 0.57 | 1.09 | 0.382 | 0.766 | 1.000 | 2248 | tags=60%, list=19%, signal=74% |
| 2510 | GOBP\_RESPONSE\_TO\_FOLIC\_ACID |  | 6 | 0.54 | 1.09 | 0.375 | 0.766 | 1.000 | 1074 | tags=50%, list=9%, signal=55% |
| 2511 | GOBP\_RAS\_PROTEIN\_SIGNAL\_TRANSDUCTION |  | 256 | 0.25 | 1.09 | 0.242 | 0.767 | 1.000 | 2722 | tags=29%, list=23%, signal=37% |
| 2512 | GOBP\_PHASIC\_SMOOTH\_MUSCLE\_CONTRACTION |  | 14 | 0.43 | 1.09 | 0.353 | 0.767 | 1.000 | 2393 | tags=43%, list=20%, signal=53% |
| 2513 | GOBP\_ENDOCYTOSIS |  | 393 | 0.24 | 1.09 | 0.214 | 0.767 | 1.000 | 2701 | tags=25%, list=22%, signal=32% |
| 2514 | GOBP\_AMYLOID\_PRECURSOR\_PROTEIN\_METABOLIC\_PROCESS |  | 57 | 0.31 | 1.09 | 0.302 | 0.768 | 1.000 | 2592 | tags=28%, list=22%, signal=36% |
| 2515 | GOBP\_CD4\_POSITIVE\_ALPHA\_BETA\_T\_CELL\_CYTOKINE\_PRODUCTION |  | 13 | 0.43 | 1.09 | 0.350 | 0.768 | 1.000 | 1968 | tags=31%, list=16%, signal=37% |
| 2516 | GOBP\_REGULATION\_OF\_LONG\_TERM\_SYNAPTIC\_DEPRESSION |  | 7 | 0.51 | 1.09 | 0.374 | 0.768 | 1.000 | 2778 | tags=71%, list=23%, signal=93% |
| 2517 | GOCC\_CELL\_PROJECTION\_MEMBRANE |  | 212 | 0.25 | 1.09 | 0.254 | 0.769 | 1.000 | 2012 | tags=24%, list=17%, signal=28% |
| 2518 | GOBP\_ORGANELLE\_LOCALIZATION |  | 516 | 0.23 | 1.09 | 0.209 | 0.769 | 1.000 | 3128 | tags=31%, list=26%, signal=40% |
| 2519 | GOBP\_POSITIVE\_REGULATION\_OF\_MAINTENANCE\_OF\_SISTER\_CHROMATID\_COHESION |  | 4 | 0.60 | 1.09 | 0.399 | 0.768 | 1.000 | 4133 | tags=75%, list=34%, signal=114% |
| 2520 | GOBP\_REGULATION\_OF\_TRANSCRIPTION\_FROM\_RNA\_POLYMERASE\_II\_PROMOTER\_INVOLVED\_IN\_HEART\_DEVELOPMENT |  | 8 | 0.50 | 1.09 | 0.368 | 0.769 | 1.000 | 2241 | tags=38%, list=19%, signal=46% |
| 2521 | GOBP\_POSITIVE\_REGULATION\_OF\_FOCAL\_ADHESION\_ASSEMBLY |  | 22 | 0.38 | 1.09 | 0.338 | 0.769 | 1.000 | 3380 | tags=45%, list=28%, signal=63% |
| 2522 | GOBP\_METANEPHRIC\_RENAL\_VESICLE\_MORPHOGENESIS |  | 7 | 0.51 | 1.09 | 0.368 | 0.768 | 1.000 | 78 | tags=14%, list=1%, signal=14% |
| 2523 | GOBP\_LUNG\_ALVEOLUS\_DEVELOPMENT |  | 28 | 0.36 | 1.09 | 0.333 | 0.768 | 1.000 | 3114 | tags=43%, list=26%, signal=58% |
| 2524 | GOBP\_ENDODERMAL\_CELL\_FATE\_COMMITMENT |  | 8 | 0.49 | 1.09 | 0.369 | 0.769 | 1.000 | 4810 | tags=63%, list=40%, signal=104% |
| 2525 | GOCC\_ENDOCYTIC\_VESICLE |  | 210 | 0.25 | 1.09 | 0.255 | 0.769 | 1.000 | 2688 | tags=28%, list=22%, signal=36% |
| 2526 | GOBP\_REGULATION\_OF\_CELL\_DIVISION |  | 117 | 0.27 | 1.09 | 0.288 | 0.769 | 1.000 | 2759 | tags=33%, list=23%, signal=43% |
| 2527 | GOBP\_PYRIMIDINE\_RIBONUCLEOSIDE\_MONOPHOSPHATE\_METABOLIC\_PROCESS |  | 13 | 0.43 | 1.09 | 0.353 | 0.769 | 1.000 | 6 | tags=8%, list=0%, signal=8% |
| 2528 | GOBP\_POSITIVE\_REGULATION\_OF\_LOCOMOTION |  | 395 | 0.24 | 1.09 | 0.216 | 0.769 | 1.000 | 2764 | tags=29%, list=23%, signal=36% |
| 2529 | GOBP\_NEGATIVE\_REGULATION\_OF\_DNA\_METABOLIC\_PROCESS |  | 76 | 0.29 | 1.09 | 0.299 | 0.769 | 1.000 | 3469 | tags=36%, list=29%, signal=50% |
| 2530 | GOBP\_NEGATIVE\_REGULATION\_OF\_INTERLEUKIN\_1\_PRODUCTION |  | 17 | 0.40 | 1.09 | 0.347 | 0.768 | 1.000 | 1219 | tags=24%, list=10%, signal=26% |
| 2531 | GOBP\_PYRIMIDINE\_NUCLEOTIDE\_BIOSYNTHETIC\_PROCESS |  | 23 | 0.37 | 1.09 | 0.339 | 0.769 | 1.000 | 61 | tags=9%, list=1%, signal=9% |
| 2532 | GOMF\_LIPOPROTEIN\_PARTICLE\_RECEPTOR\_BINDING |  | 19 | 0.39 | 1.09 | 0.343 | 0.769 | 1.000 | 2763 | tags=37%, list=23%, signal=48% |
| 2533 | GOBP\_REGULATION\_OF\_SPHINGOLIPID\_BIOSYNTHETIC\_PROCESS |  | 14 | 0.42 | 1.09 | 0.347 | 0.769 | 1.000 | 2060 | tags=36%, list=17%, signal=43% |
| 2534 | GOMF\_ABC\_TYPE\_TRANSPORTER\_ACTIVITY |  | 18 | 0.40 | 1.09 | 0.338 | 0.769 | 1.000 | 1192 | tags=28%, list=10%, signal=31% |
| 2535 | GOBP\_REGULATION\_OF\_MODIFICATION\_OF\_POSTSYNAPTIC\_ACTIN\_CYTOSKELETON |  | 5 | 0.56 | 1.09 | 0.399 | 0.770 | 1.000 | 2722 | tags=60%, list=23%, signal=78% |
| 2536 | GOBP\_BLASTOCYST\_GROWTH |  | 18 | 0.39 | 1.09 | 0.345 | 0.770 | 1.000 | 735 | tags=17%, list=6%, signal=18% |
| 2537 | GOBP\_REGULATION\_OF\_STRIATED\_MUSCLE\_CONTRACTION |  | 50 | 0.32 | 1.09 | 0.322 | 0.770 | 1.000 | 1272 | tags=22%, list=11%, signal=25% |
| 2538 | GOBP\_ICOSANOID\_SECRETION |  | 24 | 0.37 | 1.09 | 0.334 | 0.770 | 1.000 | 2113 | tags=33%, list=18%, signal=40% |
| 2539 | GOBP\_NATURAL\_KILLER\_CELL\_PROLIFERATION |  | 3 | 0.65 | 1.09 | 0.402 | 0.770 | 1.000 | 4177 | tags=100%, list=35%, signal=153% |
| 2540 | GOBP\_REGULATION\_OF\_IRE1\_MEDIATED\_UNFOLDED\_PROTEIN\_RESPONSE |  | 13 | 0.43 | 1.09 | 0.353 | 0.770 | 1.000 | 2167 | tags=31%, list=18%, signal=38% |
| 2541 | GOBP\_REGULATION\_OF\_HEART\_RATE\_BY\_CHEMICAL\_SIGNAL |  | 6 | 0.54 | 1.09 | 0.387 | 0.770 | 1.000 | 3882 | tags=67%, list=32%, signal=98% |
| 2542 | GOBP\_INTRACELLULAR\_RECEPTOR\_SIGNALING\_PATHWAY |  | 187 | 0.26 | 1.09 | 0.263 | 0.770 | 1.000 | 2030 | tags=18%, list=17%, signal=22% |
| 2543 | GOBP\_VASCULAR\_ENDOTHELIAL\_GROWTH\_FACTOR\_SIGNALING\_PATHWAY |  | 33 | 0.34 | 1.09 | 0.330 | 0.770 | 1.000 | 3493 | tags=45%, list=29%, signal=64% |
| 2544 | GOBP\_SMOOTH\_MUSCLE\_CELL\_CHEMOTAXIS |  | 6 | 0.54 | 1.09 | 0.381 | 0.770 | 1.000 | 4456 | tags=50%, list=37%, signal=79% |
| 2545 | GOCC\_ESCRT\_COMPLEX |  | 24 | 0.37 | 1.09 | 0.336 | 0.771 | 1.000 | 3003 | tags=38%, list=25%, signal=50% |
| 2546 | GOCC\_TRANSPORTER\_COMPLEX |  | 165 | 0.26 | 1.09 | 0.272 | 0.771 | 1.000 | 1272 | tags=17%, list=11%, signal=19% |
| 2547 | GOMF\_RNA\_POLYMERASE\_II\_SPECIFIC\_DNA\_BINDING\_TRANSCRIPTION\_FACTOR\_BINDING |  | 214 | 0.25 | 1.09 | 0.259 | 0.772 | 1.000 | 1705 | tags=15%, list=14%, signal=17% |
| 2548 | GOBP\_REGULATION\_OF\_CELL\_CYCLE\_PROCESS |  | 574 | 0.23 | 1.09 | 0.200 | 0.772 | 1.000 | 3984 | tags=40%, list=33%, signal=57% |
| 2549 | GOBP\_CGMP\_METABOLIC\_PROCESS |  | 8 | 0.49 | 1.09 | 0.366 | 0.772 | 1.000 | 2627 | tags=38%, list=22%, signal=48% |
| 2550 | GOBP\_NEGATIVE\_REGULATION\_OF\_PEPTIDYL\_TYROSINE\_PHOSPHORYLATION |  | 38 | 0.33 | 1.09 | 0.315 | 0.772 | 1.000 | 842 | tags=16%, list=7%, signal=17% |
| 2551 | GOMF\_MYOSIN\_V\_BINDING |  | 12 | 0.44 | 1.09 | 0.365 | 0.772 | 1.000 | 3111 | tags=50%, list=26%, signal=67% |
| 2552 | GOBP\_PRI\_MIRNA\_TRANSCRIPTION\_BY\_RNA\_POLYMERASE\_II |  | 40 | 0.33 | 1.09 | 0.321 | 0.771 | 1.000 | 1651 | tags=28%, list=14%, signal=32% |
| 2553 | GOBP\_REGULATION\_OF\_HISTONE\_H3\_K4\_METHYLATION |  | 21 | 0.38 | 1.09 | 0.338 | 0.772 | 1.000 | 228 | tags=10%, list=2%, signal=10% |
| 2554 | GOBP\_NEGATIVE\_REGULATION\_OF\_MEIOTIC\_NUCLEAR\_DIVISION |  | 4 | 0.60 | 1.09 | 0.405 | 0.771 | 1.000 | 1205 | tags=25%, list=10%, signal=28% |
| 2555 | GOCC\_GARP\_COMPLEX |  | 3 | 0.65 | 1.09 | 0.405 | 0.773 | 1.000 | 2901 | tags=67%, list=24%, signal=88% |
| 2556 | GOMF\_BUBBLE\_DNA\_BINDING |  | 7 | 0.51 | 1.09 | 0.374 | 0.773 | 1.000 | 1136 | tags=14%, list=9%, signal=16% |
| 2557 | GOBP\_VESICLE\_FUSION\_WITH\_GOLGI\_APPARATUS |  | 8 | 0.49 | 1.09 | 0.376 | 0.773 | 1.000 | 4742 | tags=75%, list=39%, signal=124% |
| 2558 | GOBP\_REGULATION\_OF\_TOLL\_LIKE\_RECEPTOR\_9\_SIGNALING\_PATHWAY |  | 8 | 0.50 | 1.09 | 0.376 | 0.773 | 1.000 | 220 | tags=13%, list=2%, signal=13% |
| 2559 | GOMF\_PHOSPHOTYROSINE\_RESIDUE\_BINDING |  | 34 | 0.34 | 1.09 | 0.320 | 0.773 | 1.000 | 2311 | tags=24%, list=19%, signal=29% |
| 2560 | GOBP\_PROTEIN\_UFMYLATION |  | 6 | 0.54 | 1.09 | 0.391 | 0.773 | 1.000 | 3527 | tags=50%, list=29%, signal=71% |
| 2561 | GOBP\_POSITIVE\_REGULATION\_OF\_TRANSMEMBRANE\_RECEPTOR\_PROTEIN\_SERINE\_THREONINE\_KINASE\_SIGNALING\_PATHWAY |  | 73 | 0.29 | 1.09 | 0.316 | 0.774 | 1.000 | 1394 | tags=19%, list=12%, signal=22% |
| 2562 | GOMF\_DEOXYRIBONUCLEASE\_ACTIVITY |  | 35 | 0.34 | 1.09 | 0.339 | 0.773 | 1.000 | 3318 | tags=51%, list=28%, signal=71% |
| 2563 | GOMF\_GABA\_RECEPTOR\_BINDING |  | 9 | 0.48 | 1.09 | 0.371 | 0.773 | 1.000 | 2320 | tags=44%, list=19%, signal=55% |
| 2564 | GOCC\_NEURON\_SPINE |  | 120 | 0.27 | 1.09 | 0.288 | 0.773 | 1.000 | 1837 | tags=23%, list=15%, signal=27% |
| 2565 | GOMF\_GUANYLATE\_KINASE\_ACTIVITY |  | 7 | 0.51 | 1.09 | 0.371 | 0.773 | 1.000 | 629 | tags=29%, list=5%, signal=30% |
| 2566 | GOMF\_DRUG\_BINDING |  | 41 | 0.33 | 1.09 | 0.332 | 0.773 | 1.000 | 1403 | tags=17%, list=12%, signal=19% |
| 2567 | GOBP\_NEGATIVE\_REGULATION\_OF\_TUBULIN\_DEACETYLATION |  | 4 | 0.60 | 1.09 | 0.402 | 0.773 | 1.000 | 2242 | tags=50%, list=19%, signal=61% |
| 2568 | GOBP\_EMBRYONIC\_DIGIT\_MORPHOGENESIS |  | 37 | 0.33 | 1.09 | 0.330 | 0.773 | 1.000 | 5 | tags=5%, list=0%, signal=5% |
| 2569 | GOBP\_GLYCOPROTEIN\_BIOSYNTHETIC\_PROCESS |  | 238 | 0.25 | 1.09 | 0.264 | 0.773 | 1.000 | 1805 | tags=18%, list=15%, signal=20% |
| 2570 | GOCC\_VESICLE\_MEMBRANE |  | 524 | 0.23 | 1.09 | 0.215 | 0.773 | 1.000 | 2684 | tags=29%, list=22%, signal=35% |
| 2571 | GOBP\_CARDIAC\_VENTRICLE\_MORPHOGENESIS |  | 43 | 0.32 | 1.09 | 0.335 | 0.773 | 1.000 | 2013 | tags=23%, list=17%, signal=28% |
| 2572 | GOBP\_CALCIUM\_IMPORT\_INTO\_THE\_MITOCHONDRION |  | 11 | 0.45 | 1.09 | 0.366 | 0.773 | 1.000 | 3113 | tags=55%, list=26%, signal=74% |
| 2573 | GOBP\_REGULATION\_OF\_PINOCYTOSIS |  | 8 | 0.49 | 1.09 | 0.375 | 0.773 | 1.000 | 3532 | tags=63%, list=29%, signal=88% |
| 2574 | GOCC\_PODOSOME |  | 26 | 0.36 | 1.09 | 0.343 | 0.772 | 1.000 | 2609 | tags=31%, list=22%, signal=39% |
| 2575 | GOBP\_ALDITOL\_METABOLIC\_PROCESS |  | 19 | 0.39 | 1.09 | 0.348 | 0.774 | 1.000 | 909 | tags=26%, list=8%, signal=28% |
| 2576 | GOBP\_REGULATION\_OF\_PROTEIN\_KINASE\_C\_SIGNALING |  | 8 | 0.49 | 1.09 | 0.375 | 0.774 | 1.000 | 3061 | tags=63%, list=25%, signal=84% |
| 2577 | GOBP\_CELL\_MIGRATION\_IN\_HINDBRAIN |  | 8 | 0.49 | 1.09 | 0.378 | 0.774 | 1.000 | 48 | tags=13%, list=0%, signal=13% |
| 2578 | GOCC\_BLOOD\_MICROPARTICLE |  | 54 | 0.31 | 1.09 | 0.314 | 0.774 | 1.000 | 1951 | tags=28%, list=16%, signal=33% |
| 2579 | GOBP\_REGULATION\_OF\_OSTEOCLAST\_DIFFERENTIATION |  | 32 | 0.35 | 1.09 | 0.332 | 0.774 | 1.000 | 1351 | tags=19%, list=11%, signal=21% |
| 2580 | GOMF\_PROTEIN\_KINASE\_C\_BINDING |  | 38 | 0.33 | 1.08 | 0.328 | 0.774 | 1.000 | 1194 | tags=13%, list=10%, signal=15% |
| 2581 | GOBP\_COPPER\_ION\_TRANSMEMBRANE\_TRANSPORT |  | 3 | 0.64 | 1.08 | 0.407 | 0.774 | 1.000 | 3004 | tags=67%, list=25%, signal=89% |
| 2582 | GOCC\_CATALYTIC\_STEP\_1\_SPLICEOSOME |  | 10 | 0.46 | 1.08 | 0.374 | 0.775 | 1.000 | 6463 | tags=100%, list=54%, signal=216% |
| 2583 | GOBP\_PROLINE\_TRANSMEMBRANE\_TRANSPORT |  | 6 | 0.53 | 1.08 | 0.385 | 0.774 | 1.000 | 1015 | tags=50%, list=8%, signal=55% |
| 2584 | GOBP\_POSITIVE\_REGULATION\_OF\_OXIDATIVE\_STRESS\_INDUCED\_CELL\_DEATH |  | 10 | 0.46 | 1.08 | 0.365 | 0.775 | 1.000 | 2221 | tags=40%, list=18%, signal=49% |
| 2585 | GOBP\_POSITIVE\_REGULATION\_OF\_SMOOTH\_MUSCLE\_CELL\_PROLIFERATION |  | 67 | 0.29 | 1.08 | 0.321 | 0.776 | 1.000 | 2465 | tags=27%, list=21%, signal=34% |
| 2586 | GOMF\_PROTEIN\_DOMAIN\_SPECIFIC\_BINDING |  | 483 | 0.23 | 1.08 | 0.226 | 0.776 | 1.000 | 2465 | tags=24%, list=21%, signal=29% |
| 2587 | GOBP\_CRANIOFACIAL\_SUTURE\_MORPHOGENESIS |  | 10 | 0.46 | 1.08 | 0.368 | 0.777 | 1.000 | 2545 | tags=50%, list=21%, signal=63% |
| 2588 | GOMF\_PEPTIDYL\_PROLINE\_DIOXYGENASE\_ACTIVITY |  | 11 | 0.45 | 1.08 | 0.361 | 0.778 | 1.000 | 2003 | tags=45%, list=17%, signal=55% |
| 2589 | GOBP\_CITRATE\_METABOLIC\_PROCESS |  | 5 | 0.57 | 1.08 | 0.402 | 0.778 | 1.000 | 3252 | tags=60%, list=27%, signal=82% |
| 2590 | GOMF\_CARBOHYDRATE\_KINASE\_ACTIVITY |  | 18 | 0.39 | 1.08 | 0.356 | 0.778 | 1.000 | 793 | tags=22%, list=7%, signal=24% |
| 2591 | GOBP\_STRESS\_GRANULE\_DISASSEMBLY |  | 5 | 0.57 | 1.08 | 0.408 | 0.779 | 1.000 | 3306 | tags=60%, list=28%, signal=83% |
| 2592 | GOBP\_POSITIVE\_REGULATION\_OF\_DNA\_REPLICATION |  | 30 | 0.35 | 1.08 | 0.332 | 0.778 | 1.000 | 2394 | tags=30%, list=20%, signal=37% |
| 2593 | GOBP\_POSITIVE\_REGULATION\_OF\_MULTICELLULAR\_ORGANISMAL\_PROCESS |  | 864 | 0.22 | 1.08 | 0.193 | 0.778 | 1.000 | 1986 | tags=19%, list=17%, signal=21% |
| 2594 | GOBP\_REGULATION\_OF\_CYCLASE\_ACTIVITY |  | 24 | 0.37 | 1.08 | 0.356 | 0.779 | 1.000 | 1386 | tags=21%, list=12%, signal=24% |
| 2595 | GOBP\_LYMPHANGIOGENESIS |  | 13 | 0.43 | 1.08 | 0.365 | 0.778 | 1.000 | 4285 | tags=54%, list=36%, signal=84% |
| 2596 | GOBP\_NEGATIVE\_REGULATION\_OF\_PROTEIN\_EXIT\_FROM\_ENDOPLASMIC\_RETICULUM |  | 9 | 0.47 | 1.08 | 0.378 | 0.779 | 1.000 | 148 | tags=11%, list=1%, signal=11% |
| 2597 | GOCC\_MIDBODY |  | 145 | 0.26 | 1.08 | 0.293 | 0.779 | 1.000 | 2783 | tags=31%, list=23%, signal=40% |
| 2598 | GOBP\_REGULATION\_OF\_DNA\_METABOLIC\_PROCESS |  | 247 | 0.25 | 1.08 | 0.264 | 0.779 | 1.000 | 2597 | tags=23%, list=22%, signal=29% |
| 2599 | GOBP\_APOPTOTIC\_DNA\_FRAGMENTATION |  | 16 | 0.40 | 1.08 | 0.363 | 0.779 | 1.000 | 3483 | tags=63%, list=29%, signal=88% |
| 2600 | GOBP\_REGULATION\_OF\_AMINO\_ACID\_IMPORT\_ACROSS\_PLASMA\_MEMBRANE |  | 9 | 0.47 | 1.08 | 0.374 | 0.779 | 1.000 | 1055 | tags=33%, list=9%, signal=37% |
| 2601 | GOBP\_POSITIVE\_REGULATION\_OF\_MITOCHONDRIAL\_MEMBRANE\_POTENTIAL |  | 7 | 0.51 | 1.08 | 0.383 | 0.782 | 1.000 | 5330 | tags=71%, list=44%, signal=128% |
| 2602 | GOBP\_CELLULAR\_RESPONSE\_TO\_UV\_C |  | 3 | 0.65 | 1.08 | 0.420 | 0.782 | 1.000 | 2863 | tags=33%, list=24%, signal=44% |
| 2603 | GOCC\_EARP\_COMPLEX |  | 3 | 0.64 | 1.08 | 0.418 | 0.783 | 1.000 | 2901 | tags=67%, list=24%, signal=88% |
| 2604 | GOBP\_SYNAPTIC\_VESICLE\_LOCALIZATION |  | 38 | 0.33 | 1.08 | 0.342 | 0.782 | 1.000 | 1649 | tags=24%, list=14%, signal=27% |
| 2605 | GOBP\_ICOSANOID\_TRANSPORT |  | 31 | 0.35 | 1.08 | 0.344 | 0.782 | 1.000 | 2113 | tags=32%, list=18%, signal=39% |
| 2606 | GOBP\_MICROTUBULE\_DEPOLYMERIZATION |  | 29 | 0.35 | 1.08 | 0.351 | 0.783 | 1.000 | 4301 | tags=59%, list=36%, signal=91% |
| 2607 | GOBP\_GENITALIA\_MORPHOGENESIS |  | 5 | 0.55 | 1.08 | 0.403 | 0.782 | 1.000 | 68 | tags=20%, list=1%, signal=20% |
| 2608 | GOMF\_PHOSPHORIC\_ESTER\_HYDROLASE\_ACTIVITY |  | 278 | 0.24 | 1.08 | 0.268 | 0.783 | 1.000 | 1520 | tags=16%, list=13%, signal=18% |
| 2609 | GOBP\_FOREBRAIN\_NEURON\_FATE\_COMMITMENT |  | 4 | 0.59 | 1.08 | 0.416 | 0.783 | 1.000 | 604 | tags=50%, list=5%, signal=53% |
| 2610 | GOBP\_SIGNALING\_RECEPTOR\_LIGAND\_PRECURSOR\_PROCESSING |  | 19 | 0.39 | 1.08 | 0.353 | 0.783 | 1.000 | 1262 | tags=26%, list=11%, signal=29% |
| 2611 | GOMF\_TRANSFERASE\_ACTIVITY\_TRANSFERRING\_HEXOSYL\_GROUPS |  | 132 | 0.26 | 1.08 | 0.307 | 0.783 | 1.000 | 3546 | tags=36%, list=30%, signal=51% |
| 2612 | GOBP\_REGULATION\_OF\_ADENYLATE\_CYCLASE\_ACTIVATING\_G\_PROTEIN\_COUPLED\_RECEPTOR\_SIGNALING\_PATHWAY |  | 6 | 0.53 | 1.08 | 0.395 | 0.783 | 1.000 | 2013 | tags=50%, list=17%, signal=60% |
| 2613 | GOBP\_CD8\_POSITIVE\_ALPHA\_BETA\_T\_CELL\_DIFFERENTIATION |  | 10 | 0.46 | 1.08 | 0.377 | 0.783 | 1.000 | 672 | tags=20%, list=6%, signal=21% |
| 2614 | GOBP\_TUBE\_DEVELOPMENT |  | 700 | 0.22 | 1.08 | 0.213 | 0.783 | 1.000 | 2846 | tags=27%, list=24%, signal=33% |
| 2615 | GOBP\_NUCLEOBASE\_CONTAINING\_SMALL\_MOLECULE\_CATABOLIC\_PROCESS |  | 35 | 0.34 | 1.08 | 0.339 | 0.783 | 1.000 | 2021 | tags=23%, list=17%, signal=27% |
| 2616 | GOBP\_NEGATIVE\_REGULATION\_OF\_TELOMERASE\_ACTIVITY |  | 9 | 0.47 | 1.08 | 0.378 | 0.784 | 1.000 | 4500 | tags=78%, list=37%, signal=124% |
| 2617 | GOBP\_MEMORY |  | 72 | 0.29 | 1.08 | 0.316 | 0.785 | 1.000 | 1485 | tags=21%, list=12%, signal=24% |
| 2618 | GOBP\_PROLINE\_TRANSPORT |  | 6 | 0.53 | 1.08 | 0.386 | 0.786 | 1.000 | 1015 | tags=50%, list=8%, signal=55% |
| 2619 | GOCC\_MICROTUBULE\_ORGANIZING\_CENTER |  | 580 | 0.23 | 1.08 | 0.224 | 0.786 | 1.000 | 3170 | tags=28%, list=26%, signal=37% |
| 2620 | GOCC\_CALCITONIN\_FAMILY\_RECEPTOR\_COMPLEX |  | 4 | 0.59 | 1.08 | 0.426 | 0.786 | 1.000 | 764 | tags=25%, list=6%, signal=27% |
| 2621 | GOBP\_CORTICAL\_CYTOSKELETON\_ORGANIZATION |  | 49 | 0.31 | 1.08 | 0.334 | 0.786 | 1.000 | 2549 | tags=33%, list=21%, signal=41% |
| 2622 | GOMF\_NEUTRAL\_AMINO\_ACID\_TRANSMEMBRANE\_TRANSPORTER\_ACTIVITY |  | 23 | 0.37 | 1.08 | 0.352 | 0.786 | 1.000 | 1055 | tags=26%, list=9%, signal=29% |
| 2623 | GOBP\_ACTION\_POTENTIAL |  | 80 | 0.29 | 1.08 | 0.327 | 0.786 | 1.000 | 1436 | tags=23%, list=12%, signal=25% |
| 2624 | GOMF\_NEUROTRANSMITTER\_TRANSMEMBRANE\_TRANSPORTER\_ACTIVITY |  | 7 | 0.50 | 1.08 | 0.386 | 0.786 | 1.000 | 1403 | tags=43%, list=12%, signal=48% |
| 2625 | GOBP\_CARBOHYDRATE\_PHOSPHORYLATION |  | 21 | 0.38 | 1.08 | 0.356 | 0.786 | 1.000 | 793 | tags=19%, list=7%, signal=20% |
| 2626 | GOMF\_G\_PROTEIN\_COUPLED\_RECEPTOR\_BINDING |  | 139 | 0.26 | 1.08 | 0.300 | 0.786 | 1.000 | 2536 | tags=27%, list=21%, signal=33% |
| 2627 | GOBP\_REGULATION\_OF\_BLOOD\_CIRCULATION |  | 171 | 0.26 | 1.08 | 0.304 | 0.786 | 1.000 | 1549 | tags=20%, list=13%, signal=23% |
| 2628 | GOCC\_PROTEIN\_PHOSPHATASE\_TYPE\_1\_COMPLEX |  | 10 | 0.46 | 1.08 | 0.378 | 0.786 | 1.000 | 3172 | tags=50%, list=26%, signal=68% |
| 2629 | GOBP\_NEGATIVE\_REGULATION\_OF\_STEM\_CELL\_PROLIFERATION |  | 3 | 0.64 | 1.08 | 0.425 | 0.785 | 1.000 | 784 | tags=33%, list=7%, signal=36% |
| 2630 | GOBP\_INSULIN\_RECEPTOR\_SIGNALING\_PATHWAY |  | 111 | 0.27 | 1.08 | 0.312 | 0.785 | 1.000 | 2311 | tags=24%, list=19%, signal=30% |
| 2631 | GOBP\_POSITIVE\_REGULATION\_OF\_PROTEIN\_KINASE\_ACTIVITY |  | 383 | 0.23 | 1.08 | 0.259 | 0.785 | 1.000 | 2375 | tags=23%, list=20%, signal=27% |
| 2632 | GOCC\_U4\_SNRNP |  | 7 | 0.50 | 1.08 | 0.389 | 0.785 | 1.000 | 3759 | tags=71%, list=31%, signal=104% |
| 2633 | GOBP\_PROTEIN\_OXIDATION |  | 13 | 0.43 | 1.08 | 0.366 | 0.785 | 1.000 | 2504 | tags=38%, list=21%, signal=49% |
| 2634 | GOBP\_MONOSACCHARIDE\_METABOLIC\_PROCESS |  | 195 | 0.25 | 1.08 | 0.297 | 0.785 | 1.000 | 1427 | tags=17%, list=12%, signal=19% |
| 2635 | GOBP\_POSITIVE\_REGULATION\_OF\_INTERLEUKIN\_6\_PRODUCTION |  | 47 | 0.31 | 1.08 | 0.327 | 0.784 | 1.000 | 954 | tags=13%, list=8%, signal=14% |
| 2636 | GOBP\_PROTEIN\_EXIT\_FROM\_ENDOPLASMIC\_RETICULUM |  | 39 | 0.33 | 1.08 | 0.354 | 0.785 | 1.000 | 1167 | tags=15%, list=10%, signal=17% |
| 2637 | GOBP\_POSITIVE\_REGULATION\_OF\_DNA\_DAMAGE\_RESPONSE\_SIGNAL\_TRANSDUCTION\_BY\_P53\_CLASS\_MEDIATOR |  | 9 | 0.47 | 1.08 | 0.373 | 0.785 | 1.000 | 2597 | tags=44%, list=22%, signal=57% |
| 2638 | GOBP\_CARBOHYDRATE\_METABOLIC\_PROCESS |  | 440 | 0.23 | 1.08 | 0.250 | 0.785 | 1.000 | 1427 | tags=15%, list=12%, signal=17% |
| 2639 | GOBP\_TENDON\_DEVELOPMENT |  | 4 | 0.59 | 1.08 | 0.408 | 0.785 | 1.000 | 2838 | tags=75%, list=24%, signal=98% |
| 2640 | GOBP\_RESPONSE\_TO\_BACTERIUM |  | 313 | 0.24 | 1.08 | 0.275 | 0.785 | 1.000 | 2169 | tags=23%, list=18%, signal=27% |
| 2641 | GOMF\_COPPER\_ION\_TRANSMEMBRANE\_TRANSPORTER\_ACTIVITY |  | 3 | 0.64 | 1.07 | 0.430 | 0.785 | 1.000 | 3004 | tags=67%, list=25%, signal=89% |
| 2642 | GOBP\_VENTRICULAR\_COMPACT\_MYOCARDIUM\_MORPHOGENESIS |  | 6 | 0.53 | 1.07 | 0.400 | 0.785 | 1.000 | 1336 | tags=33%, list=11%, signal=37% |
| 2643 | GOBP\_TUMOR\_NECROSIS\_FACTOR\_SUPERFAMILY\_CYTOKINE\_PRODUCTION |  | 79 | 0.29 | 1.07 | 0.325 | 0.785 | 1.000 | 3100 | tags=34%, list=26%, signal=46% |
| 2644 | GOCC\_METHYLOSOME |  | 9 | 0.47 | 1.07 | 0.384 | 0.785 | 1.000 | 455 | tags=22%, list=4%, signal=23% |
| 2645 | GOMF\_NUCLEOSOMAL\_HISTONE\_BINDING |  | 4 | 0.59 | 1.07 | 0.417 | 0.785 | 1.000 | 3791 | tags=75%, list=32%, signal=110% |
| 2646 | GOBP\_SECRETION\_BY\_TISSUE |  | 16 | 0.40 | 1.07 | 0.361 | 0.785 | 1.000 | 2349 | tags=50%, list=20%, signal=62% |
| 2647 | GOBP\_FC\_RECEPTOR\_MEDIATED\_STIMULATORY\_SIGNALING\_PATHWAY |  | 70 | 0.29 | 1.07 | 0.329 | 0.785 | 1.000 | 2722 | tags=33%, list=23%, signal=42% |
| 2648 | GOCC\_MICROTUBULE |  | 309 | 0.24 | 1.07 | 0.271 | 0.785 | 1.000 | 3984 | tags=44%, list=33%, signal=65% |
| 2649 | GOBP\_HEART\_PROCESS |  | 169 | 0.25 | 1.07 | 0.307 | 0.785 | 1.000 | 2524 | tags=29%, list=21%, signal=36% |
| 2650 | GOBP\_REGULATION\_OF\_MAINTENANCE\_OF\_SISTER\_CHROMATID\_COHESION |  | 6 | 0.53 | 1.07 | 0.383 | 0.785 | 1.000 | 4133 | tags=50%, list=34%, signal=76% |
| 2651 | GOBP\_REGULATION\_OF\_CELL\_PROJECTION\_ORGANIZATION |  | 449 | 0.23 | 1.07 | 0.255 | 0.785 | 1.000 | 2375 | tags=24%, list=20%, signal=28% |
| 2652 | GOBP\_CELLULAR\_RESPONSE\_TO\_LAMINAR\_FLUID\_SHEAR\_STRESS |  | 6 | 0.53 | 1.07 | 0.389 | 0.785 | 1.000 | 571 | tags=33%, list=5%, signal=35% |
| 2653 | GOBP\_CELLULAR\_RESPONSE\_TO\_IONIZING\_RADIATION |  | 49 | 0.31 | 1.07 | 0.344 | 0.785 | 1.000 | 2597 | tags=35%, list=22%, signal=44% |
| 2654 | GOBP\_REGULATION\_OF\_TOLERANCE\_INDUCTION |  | 7 | 0.50 | 1.07 | 0.388 | 0.785 | 1.000 | 682 | tags=14%, list=6%, signal=15% |
| 2655 | GOBP\_TELOMERASE\_HOLOENZYME\_COMPLEX\_ASSEMBLY |  | 6 | 0.53 | 1.07 | 0.394 | 0.785 | 1.000 | 2597 | tags=50%, list=22%, signal=64% |
| 2656 | GOBP\_POSITIVE\_REGULATION\_OF\_EXTRINSIC\_APOPTOTIC\_SIGNALING\_PATHWAY\_VIA\_DEATH\_DOMAIN\_RECEPTORS |  | 10 | 0.46 | 1.07 | 0.389 | 0.785 | 1.000 | 1593 | tags=40%, list=13%, signal=46% |
| 2657 | GOBP\_EXTRACELLULAR\_MATRIX\_ASSEMBLY |  | 34 | 0.34 | 1.07 | 0.354 | 0.785 | 1.000 | 1718 | tags=18%, list=14%, signal=21% |
| 2658 | GOBP\_REGULATION\_OF\_RUFFLE\_ASSEMBLY |  | 18 | 0.39 | 1.07 | 0.372 | 0.785 | 1.000 | 3736 | tags=56%, list=31%, signal=81% |
| 2659 | GOBP\_RESPONSE\_TO\_STEROL\_DEPLETION |  | 14 | 0.42 | 1.07 | 0.369 | 0.785 | 1.000 | 691 | tags=29%, list=6%, signal=30% |
| 2660 | GOBP\_ALANINE\_CATABOLIC\_PROCESS |  | 3 | 0.64 | 1.07 | 0.427 | 0.785 | 1.000 | 1716 | tags=67%, list=14%, signal=78% |
| 2661 | GOMF\_CASPASE\_BINDING |  | 5 | 0.55 | 1.07 | 0.402 | 0.785 | 1.000 | 3148 | tags=80%, list=26%, signal=108% |
| 2662 | GOBP\_LAMELLIPODIUM\_ASSEMBLY |  | 54 | 0.30 | 1.07 | 0.336 | 0.785 | 1.000 | 2722 | tags=31%, list=23%, signal=41% |
| 2663 | GOBP\_MULTI\_CILIATED\_EPITHELIAL\_CELL\_DIFFERENTIATION |  | 3 | 0.64 | 1.07 | 0.431 | 0.785 | 1.000 | 2271 | tags=67%, list=19%, signal=82% |
| 2664 | GOBP\_CELLULAR\_RESPONSE\_TO\_STEROID\_HORMONE\_STIMULUS |  | 143 | 0.26 | 1.07 | 0.314 | 0.786 | 1.000 | 1301 | tags=17%, list=11%, signal=19% |
| 2665 | GOBP\_REGULATION\_OF\_ASTROCYTE\_ACTIVATION |  | 3 | 0.64 | 1.07 | 0.443 | 0.786 | 1.000 | 159 | tags=33%, list=1%, signal=34% |
| 2666 | GOBP\_APICAL\_PROTEIN\_LOCALIZATION |  | 13 | 0.42 | 1.07 | 0.382 | 0.786 | 1.000 | 1026 | tags=23%, list=9%, signal=25% |
| 2667 | GOBP\_REGULATION\_OF\_FOREBRAIN\_NEURON\_DIFFERENTIATION |  | 4 | 0.59 | 1.07 | 0.418 | 0.786 | 1.000 | 509 | tags=25%, list=4%, signal=26% |
| 2668 | GOBP\_DEOXYRIBOSE\_PHOSPHATE\_BIOSYNTHETIC\_PROCESS |  | 10 | 0.45 | 1.07 | 0.381 | 0.786 | 1.000 | 904 | tags=20%, list=8%, signal=22% |
| 2669 | GOMF\_INHIBIN\_BINDING |  | 4 | 0.59 | 1.07 | 0.409 | 0.786 | 1.000 | 1909 | tags=50%, list=16%, signal=59% |
| 2670 | GOBP\_NEGATIVE\_REGULATION\_OF\_MYELOID\_CELL\_APOPTOTIC\_PROCESS |  | 6 | 0.53 | 1.07 | 0.406 | 0.787 | 1.000 | 85 | tags=17%, list=1%, signal=17% |
| 2671 | GOBP\_AMINO\_ACID\_IMPORT |  | 34 | 0.34 | 1.07 | 0.347 | 0.786 | 1.000 | 1055 | tags=24%, list=9%, signal=26% |
| 2672 | GOBP\_EPOXIDE\_METABOLIC\_PROCESS |  | 4 | 0.59 | 1.07 | 0.424 | 0.787 | 1.000 | 2980 | tags=50%, list=25%, signal=66% |
| 2673 | GOBP\_REGULATION\_OF\_ALPHA\_BETA\_T\_CELL\_ACTIVATION |  | 62 | 0.30 | 1.07 | 0.346 | 0.787 | 1.000 | 1198 | tags=16%, list=10%, signal=18% |
| 2674 | GOBP\_RESPONSE\_TO\_INTERLEUKIN\_15 |  | 13 | 0.42 | 1.07 | 0.379 | 0.787 | 1.000 | 2691 | tags=54%, list=22%, signal=69% |
| 2675 | GOBP\_MACROPHAGE\_APOPTOTIC\_PROCESS |  | 7 | 0.51 | 1.07 | 0.388 | 0.787 | 1.000 | 85 | tags=14%, list=1%, signal=14% |
| 2676 | GOCC\_MEIOTIC\_COHESIN\_COMPLEX |  | 4 | 0.59 | 1.07 | 0.422 | 0.787 | 1.000 | 4903 | tags=75%, list=41%, signal=127% |
| 2677 | GOCC\_POLE\_PLASM |  | 10 | 0.45 | 1.07 | 0.388 | 0.787 | 1.000 | 977 | tags=20%, list=8%, signal=22% |
| 2678 | GOMF\_PROTEIN\_HOMODIMERIZATION\_ACTIVITY |  | 461 | 0.23 | 1.07 | 0.260 | 0.787 | 1.000 | 1839 | tags=19%, list=15%, signal=21% |
| 2679 | GOBP\_ERK1\_AND\_ERK2\_CASCADE |  | 190 | 0.25 | 1.07 | 0.302 | 0.787 | 1.000 | 1395 | tags=16%, list=12%, signal=18% |
| 2680 | GOBP\_POSITIVE\_REGULATION\_OF\_IMMUNE\_EFFECTOR\_PROCESS |  | 123 | 0.27 | 1.07 | 0.319 | 0.787 | 1.000 | 1326 | tags=15%, list=11%, signal=16% |
| 2681 | GOBP\_REGULATION\_OF\_NIK\_NF\_KAPPAB\_SIGNALING |  | 67 | 0.29 | 1.07 | 0.336 | 0.787 | 1.000 | 1198 | tags=13%, list=10%, signal=15% |
| 2682 | GOBP\_CARDIAC\_CHAMBER\_MORPHOGENESIS |  | 77 | 0.29 | 1.07 | 0.324 | 0.787 | 1.000 | 2048 | tags=21%, list=17%, signal=25% |
| 2683 | GOBP\_ENDOTHELIAL\_CELL\_MORPHOGENESIS |  | 8 | 0.48 | 1.07 | 0.389 | 0.787 | 1.000 | 4616 | tags=75%, list=38%, signal=122% |
| 2684 | GOBP\_DEOXYRIBONUCLEOSIDE\_MONOPHOSPHATE\_BIOSYNTHETIC\_PROCESS |  | 7 | 0.50 | 1.07 | 0.390 | 0.787 | 1.000 | 904 | tags=29%, list=8%, signal=31% |
| 2685 | GOBP\_NEGATIVE\_REGULATION\_OF\_DEFENSE\_RESPONSE\_TO\_VIRUS |  | 18 | 0.39 | 1.07 | 0.366 | 0.787 | 1.000 | 1137 | tags=17%, list=9%, signal=18% |
| 2686 | GOMF\_BETA\_2\_ADRENERGIC\_RECEPTOR\_BINDING |  | 4 | 0.59 | 1.07 | 0.432 | 0.788 | 1.000 | 4794 | tags=75%, list=40%, signal=125% |
| 2687 | GOBP\_CARDIAC\_CONDUCTION |  | 78 | 0.28 | 1.07 | 0.333 | 0.787 | 1.000 | 1778 | tags=23%, list=15%, signal=27% |
| 2688 | GOBP\_POSITIVE\_REGULATION\_OF\_PLASMA\_MEMBRANE\_BOUNDED\_CELL\_PROJECTION\_ASSEMBLY |  | 75 | 0.28 | 1.07 | 0.331 | 0.788 | 1.000 | 2200 | tags=25%, list=18%, signal=31% |
| 2689 | GOBP\_POSITIVE\_REGULATION\_OF\_NITRIC\_OXIDE\_SYNTHASE\_ACTIVITY |  | 18 | 0.39 | 1.07 | 0.381 | 0.788 | 1.000 | 1926 | tags=33%, list=16%, signal=40% |
| 2690 | GOBP\_RIBONUCLEOSIDE\_CATABOLIC\_PROCESS |  | 13 | 0.42 | 1.07 | 0.374 | 0.788 | 1.000 | 6 | tags=8%, list=0%, signal=8% |
| 2691 | GOBP\_SPHINGOMYELIN\_CATABOLIC\_PROCESS |  | 6 | 0.52 | 1.07 | 0.406 | 0.788 | 1.000 | 2850 | tags=67%, list=24%, signal=87% |
| 2692 | GOCC\_MAIN\_AXON |  | 40 | 0.32 | 1.07 | 0.355 | 0.788 | 1.000 | 3647 | tags=52%, list=30%, signal=75% |
| 2693 | GOMF\_GLUCOSIDASE\_ACTIVITY |  | 9 | 0.46 | 1.07 | 0.390 | 0.788 | 1.000 | 1279 | tags=33%, list=11%, signal=37% |
| 2694 | GOBP\_DNA\_CATABOLIC\_PROCESS |  | 24 | 0.36 | 1.07 | 0.371 | 0.789 | 1.000 | 3483 | tags=58%, list=29%, signal=82% |
| 2695 | GOBP\_CELL\_MIGRATION |  | 975 | 0.22 | 1.07 | 0.227 | 0.789 | 1.000 | 2611 | tags=25%, list=22%, signal=29% |
| 2696 | GOMF\_MANNOSYLTRANSFERASE\_ACTIVITY |  | 21 | 0.37 | 1.07 | 0.372 | 0.789 | 1.000 | 3062 | tags=33%, list=25%, signal=45% |
| 2697 | GOMF\_MECHANOSENSITIVE\_ION\_CHANNEL\_ACTIVITY |  | 11 | 0.44 | 1.07 | 0.379 | 0.789 | 1.000 | 3233 | tags=55%, list=27%, signal=75% |
| 2698 | GOMF\_SHORT\_CHAIN\_CARBOXYLESTERASE\_ACTIVITY |  | 3 | 0.63 | 1.07 | 0.441 | 0.790 | 1.000 | 1054 | tags=67%, list=9%, signal=73% |
| 2699 | GOBP\_REGULATION\_OF\_PROTEIN\_IMPORT |  | 51 | 0.31 | 1.07 | 0.355 | 0.790 | 1.000 | 2749 | tags=25%, list=23%, signal=33% |
| 2700 | GOBP\_CARBOHYDRATE\_BIOSYNTHETIC\_PROCESS |  | 145 | 0.26 | 1.07 | 0.323 | 0.790 | 1.000 | 1617 | tags=20%, list=13%, signal=23% |
| 2701 | GOBP\_POSITIVE\_REGULATION\_OF\_CELL\_CELL\_ADHESION\_MEDIATED\_BY\_INTEGRIN |  | 6 | 0.53 | 1.07 | 0.402 | 0.790 | 1.000 | 2185 | tags=50%, list=18%, signal=61% |
| 2702 | GOBP\_GAMMA\_AMINOBUTYRIC\_ACID\_TRANSPORT |  | 9 | 0.47 | 1.07 | 0.396 | 0.790 | 1.000 | 10 | tags=11%, list=0%, signal=11% |
| 2703 | GOBP\_CELLULAR\_RESPONSE\_TO\_MOLECULE\_OF\_BACTERIAL\_ORIGIN |  | 113 | 0.27 | 1.07 | 0.329 | 0.791 | 1.000 | 2115 | tags=22%, list=18%, signal=27% |
| 2704 | GOBP\_VERY\_LOW\_DENSITY\_LIPOPROTEIN\_PARTICLE\_CLEARANCE |  | 3 | 0.64 | 1.07 | 0.440 | 0.792 | 1.000 | 248 | tags=33%, list=2%, signal=34% |
| 2705 | GOBP\_POSITIVE\_REGULATION\_OF\_ENDOPLASMIC\_RETICULUM\_UNFOLDED\_PROTEIN\_RESPONSE |  | 11 | 0.44 | 1.07 | 0.389 | 0.791 | 1.000 | 108 | tags=9%, list=1%, signal=9% |
| 2706 | GOBP\_POSITIVE\_REGULATION\_OF\_RECEPTOR\_CATABOLIC\_PROCESS |  | 6 | 0.52 | 1.07 | 0.409 | 0.791 | 1.000 | 143 | tags=17%, list=1%, signal=17% |
| 2707 | GOBP\_CELL\_PROJECTION\_ASSEMBLY |  | 409 | 0.23 | 1.07 | 0.267 | 0.792 | 1.000 | 2609 | tags=25%, list=22%, signal=31% |
| 2708 | GOCC\_HIPPOCAMPAL\_MOSSY\_FIBER |  | 4 | 0.58 | 1.07 | 0.427 | 0.792 | 1.000 | 1969 | tags=50%, list=16%, signal=60% |
| 2709 | GOBP\_REGULATION\_OF\_METANEPHROS\_DEVELOPMENT |  | 3 | 0.64 | 1.07 | 0.452 | 0.792 | 1.000 | 2683 | tags=67%, list=22%, signal=86% |
| 2710 | GOBP\_REGULATION\_OF\_LEUKOCYTE\_CHEMOTAXIS |  | 78 | 0.28 | 1.07 | 0.337 | 0.792 | 1.000 | 2458 | tags=29%, list=20%, signal=37% |
| 2711 | GOBP\_NEGATIVE\_REGULATION\_OF\_T\_CELL\_MEDIATED\_IMMUNITY |  | 6 | 0.52 | 1.07 | 0.415 | 0.792 | 1.000 | 405 | tags=17%, list=3%, signal=17% |
| 2712 | GOBP\_REGULATION\_OF\_PROTEIN\_KINASE\_A\_SIGNALING |  | 15 | 0.41 | 1.07 | 0.381 | 0.792 | 1.000 | 3650 | tags=60%, list=30%, signal=86% |
| 2713 | GOCC\_PROXIMAL\_DENDRITE |  | 5 | 0.55 | 1.07 | 0.414 | 0.792 | 1.000 | 2013 | tags=60%, list=17%, signal=72% |
| 2714 | GOBP\_NEGATIVE\_REGULATION\_OF\_HORMONE\_SECRETION |  | 35 | 0.33 | 1.06 | 0.369 | 0.793 | 1.000 | 1255 | tags=26%, list=10%, signal=29% |
| 2715 | GOBP\_GLYCOPROTEIN\_METABOLIC\_PROCESS |  | 284 | 0.24 | 1.06 | 0.295 | 0.793 | 1.000 | 2588 | tags=24%, list=22%, signal=30% |
| 2716 | GOBP\_PROTEIN\_TETRAMERIZATION |  | 52 | 0.31 | 1.06 | 0.345 | 0.792 | 1.000 | 972 | tags=15%, list=8%, signal=17% |
| 2717 | GOBP\_SENSORY\_ORGAN\_DEVELOPMENT |  | 312 | 0.24 | 1.06 | 0.295 | 0.793 | 1.000 | 2843 | tags=29%, list=24%, signal=36% |
| 2718 | GOBP\_CANNABINOID\_SIGNALING\_PATHWAY |  | 6 | 0.52 | 1.06 | 0.406 | 0.793 | 1.000 | 2141 | tags=50%, list=18%, signal=61% |
| 2719 | GOMF\_MAP\_KINASE\_SCAFFOLD\_ACTIVITY |  | 9 | 0.47 | 1.06 | 0.387 | 0.793 | 1.000 | 933 | tags=11%, list=8%, signal=12% |
| 2720 | GOBP\_POSITIVE\_REGULATION\_OF\_SPROUTING\_ANGIOGENESIS |  | 11 | 0.44 | 1.06 | 0.385 | 0.794 | 1.000 | 1794 | tags=27%, list=15%, signal=32% |
| 2721 | GOBP\_POSITIVE\_REGULATION\_OF\_PROTEIN\_TYROSINE\_KINASE\_ACTIVITY |  | 42 | 0.32 | 1.06 | 0.369 | 0.795 | 1.000 | 1928 | tags=26%, list=16%, signal=31% |
| 2722 | GOMF\_14\_3\_3\_PROTEIN\_BINDING |  | 23 | 0.36 | 1.06 | 0.376 | 0.795 | 1.000 | 2171 | tags=30%, list=18%, signal=37% |
| 2723 | GOBP\_POLYSACCHARIDE\_METABOLIC\_PROCESS |  | 78 | 0.28 | 1.06 | 0.341 | 0.795 | 1.000 | 2897 | tags=29%, list=24%, signal=39% |
| 2724 | GOMF\_ACETYLESTERASE\_ACTIVITY |  | 3 | 0.63 | 1.06 | 0.433 | 0.795 | 1.000 | 1054 | tags=67%, list=9%, signal=73% |
| 2725 | GOCC\_COHESIN\_COMPLEX |  | 8 | 0.48 | 1.06 | 0.399 | 0.795 | 1.000 | 2851 | tags=38%, list=24%, signal=49% |
| 2726 | GOBP\_ORGANELLE\_MEMBRANE\_FUSION |  | 83 | 0.28 | 1.06 | 0.340 | 0.795 | 1.000 | 1783 | tags=22%, list=15%, signal=25% |
| 2727 | GOBP\_EPITHELIAL\_FLUID\_TRANSPORT |  | 9 | 0.46 | 1.06 | 0.393 | 0.795 | 1.000 | 2845 | tags=56%, list=24%, signal=73% |
| 2728 | GOBP\_POSITIVE\_REGULATION\_OF\_ERK1\_AND\_ERK2\_CASCADE |  | 116 | 0.27 | 1.06 | 0.344 | 0.795 | 1.000 | 2351 | tags=27%, list=20%, signal=33% |
| 2729 | GOMF\_NEUROPEPTIDE\_RECEPTOR\_ACTIVITY |  | 13 | 0.42 | 1.06 | 0.386 | 0.795 | 1.000 | 1124 | tags=31%, list=9%, signal=34% |
| 2730 | GOBP\_POTASSIUM\_ION\_IMPORT\_ACROSS\_PLASMA\_MEMBRANE |  | 24 | 0.36 | 1.06 | 0.371 | 0.796 | 1.000 | 1854 | tags=33%, list=15%, signal=39% |
| 2731 | GOBP\_RESPONSE\_TO\_FIBROBLAST\_GROWTH\_FACTOR |  | 96 | 0.27 | 1.06 | 0.343 | 0.796 | 1.000 | 3399 | tags=41%, list=28%, signal=56% |
| 2732 | GOCC\_BRUSH\_BORDER\_MEMBRANE |  | 41 | 0.32 | 1.06 | 0.359 | 0.796 | 1.000 | 1786 | tags=32%, list=15%, signal=37% |
| 2733 | GOBP\_VIRAL\_BUDDING |  | 24 | 0.35 | 1.06 | 0.375 | 0.796 | 1.000 | 4201 | tags=50%, list=35%, signal=77% |
| 2734 | GOBP\_REGULATION\_OF\_INTRACELLULAR\_PROTEIN\_TRANSPORT |  | 187 | 0.25 | 1.06 | 0.323 | 0.796 | 1.000 | 2778 | tags=26%, list=23%, signal=33% |
| 2735 | GOBP\_CARDIOCYTE\_DIFFERENTIATION |  | 88 | 0.28 | 1.06 | 0.349 | 0.796 | 1.000 | 2351 | tags=24%, list=20%, signal=29% |
| 2736 | GOBP\_REGULATION\_OF\_TRANSPORTER\_ACTIVITY |  | 162 | 0.25 | 1.06 | 0.331 | 0.796 | 1.000 | 2680 | tags=31%, list=22%, signal=39% |
| 2737 | GOBP\_ASTROCYTE\_CELL\_MIGRATION |  | 3 | 0.63 | 1.06 | 0.439 | 0.796 | 1.000 | 512 | tags=33%, list=4%, signal=35% |
| 2738 | GOBP\_REGULATION\_OF\_KERATINOCYTE\_PROLIFERATION |  | 23 | 0.36 | 1.06 | 0.370 | 0.796 | 1.000 | 2998 | tags=48%, list=25%, signal=64% |
| 2739 | GOBP\_NEGATIVE\_REGULATION\_OF\_CELL\_GROWTH\_INVOLVED\_IN\_CARDIAC\_MUSCLE\_CELL\_DEVELOPMENT |  | 7 | 0.50 | 1.06 | 0.399 | 0.796 | 1.000 | 2761 | tags=57%, list=23%, signal=74% |
| 2740 | GOBP\_ENTRAINMENT\_OF\_CIRCADIAN\_CLOCK |  | 22 | 0.37 | 1.06 | 0.381 | 0.796 | 1.000 | 2581 | tags=32%, list=21%, signal=40% |
| 2741 | GOBP\_REGULATION\_OF\_STEROID\_HORMONE\_BIOSYNTHETIC\_PROCESS |  | 9 | 0.46 | 1.06 | 0.400 | 0.796 | 1.000 | 3816 | tags=67%, list=32%, signal=98% |
| 2742 | GOBP\_POSITIVE\_REGULATION\_OF\_INTEGRIN\_ACTIVATION |  | 9 | 0.46 | 1.06 | 0.396 | 0.796 | 1.000 | 976 | tags=22%, list=8%, signal=24% |
| 2743 | GOBP\_SELENIUM\_COMPOUND\_METABOLIC\_PROCESS |  | 3 | 0.63 | 1.06 | 0.441 | 0.796 | 1.000 | 972 | tags=33%, list=8%, signal=36% |
| 2744 | GOMF\_MISMATCH\_REPAIR\_COMPLEX\_BINDING |  | 10 | 0.45 | 1.06 | 0.392 | 0.796 | 1.000 | 1894 | tags=30%, list=16%, signal=36% |
| 2745 | GOBP\_UDP\_N\_ACETYLGLUCOSAMINE\_BIOSYNTHETIC\_PROCESS |  | 9 | 0.46 | 1.06 | 0.396 | 0.796 | 1.000 | 1618 | tags=33%, list=13%, signal=38% |
| 2746 | GOBP\_POSITIVE\_REGULATION\_OF\_HISTONE\_METHYLATION |  | 27 | 0.35 | 1.06 | 0.372 | 0.798 | 1.000 | 228 | tags=7%, list=2%, signal=8% |
| 2747 | GOBP\_REGULATION\_OF\_AMYLOID\_PRECURSOR\_PROTEIN\_CATABOLIC\_PROCESS |  | 32 | 0.33 | 1.06 | 0.366 | 0.798 | 1.000 | 2013 | tags=25%, list=17%, signal=30% |
| 2748 | GOBP\_REGULATION\_OF\_EXOCYTOSIS |  | 135 | 0.26 | 1.06 | 0.329 | 0.798 | 1.000 | 1649 | tags=19%, list=14%, signal=22% |
| 2749 | GOBP\_PROTEIN\_TRANSPORT\_TO\_VACUOLE\_INVOLVED\_IN\_UBIQUITIN\_DEPENDENT\_PROTEIN\_CATABOLIC\_PROCESS\_VIA\_THE\_MULTIVESICULAR\_BODY\_SORTING\_PATHWAY |  | 4 | 0.58 | 1.06 | 0.442 | 0.798 | 1.000 | 4173 | tags=75%, list=35%, signal=115% |
| 2750 | GOBP\_FEMALE\_MEIOSIS\_I |  | 4 | 0.59 | 1.06 | 0.437 | 0.798 | 1.000 | 1205 | tags=25%, list=10%, signal=28% |
| 2751 | GOBP\_ESTABLISHMENT\_OF\_ORGANELLE\_LOCALIZATION |  | 338 | 0.23 | 1.06 | 0.305 | 0.798 | 1.000 | 3528 | tags=36%, list=29%, signal=50% |
| 2752 | GOBP\_REGULATION\_OF\_OSTEOBLAST\_PROLIFERATION |  | 17 | 0.39 | 1.06 | 0.383 | 0.798 | 1.000 | 1800 | tags=41%, list=15%, signal=48% |
| 2753 | GOMF\_CORE\_PROMOTER\_SEQUENCE\_SPECIFIC\_DNA\_BINDING |  | 26 | 0.35 | 1.06 | 0.383 | 0.799 | 1.000 | 1216 | tags=15%, list=10%, signal=17% |
| 2754 | GOBP\_MEMBRANE\_FUSION |  | 114 | 0.26 | 1.06 | 0.338 | 0.799 | 1.000 | 1986 | tags=21%, list=17%, signal=25% |
| 2755 | GOBP\_PYRIMIDINE\_NUCLEOSIDE\_MONOPHOSPHATE\_METABOLIC\_PROCESS |  | 16 | 0.40 | 1.06 | 0.385 | 0.799 | 1.000 | 904 | tags=13%, list=8%, signal=13% |
| 2756 | GOBP\_PROTEIN\_O\_LINKED\_GLYCOSYLATION |  | 63 | 0.29 | 1.06 | 0.362 | 0.799 | 1.000 | 2799 | tags=33%, list=23%, signal=43% |
| 2757 | GOBP\_NEGATIVE\_REGULATION\_OF\_LIPOPROTEIN\_LIPASE\_ACTIVITY |  | 3 | 0.63 | 1.06 | 0.449 | 0.799 | 1.000 | 1324 | tags=33%, list=11%, signal=37% |
| 2758 | GOMF\_MUTLALPHA\_COMPLEX\_BINDING |  | 5 | 0.54 | 1.06 | 0.416 | 0.799 | 1.000 | 5126 | tags=80%, list=43%, signal=140% |
| 2759 | GOBP\_RESPONSE\_TO\_RETINOIC\_ACID |  | 65 | 0.29 | 1.06 | 0.357 | 0.799 | 1.000 | 1403 | tags=17%, list=12%, signal=19% |
| 2760 | GOMF\_ACYLGLYCEROL\_O\_ACYLTRANSFERASE\_ACTIVITY |  | 18 | 0.39 | 1.06 | 0.392 | 0.799 | 1.000 | 1925 | tags=39%, list=16%, signal=46% |
| 2761 | GOBP\_CARDIAC\_CHAMBER\_DEVELOPMENT |  | 107 | 0.27 | 1.06 | 0.347 | 0.800 | 1.000 | 2478 | tags=23%, list=21%, signal=29% |
| 2762 | GOBP\_REGULATION\_OF\_CORE\_PROMOTER\_BINDING |  | 5 | 0.54 | 1.06 | 0.432 | 0.799 | 1.000 | 571 | tags=20%, list=5%, signal=21% |
| 2763 | GOBP\_ODONTOGENESIS |  | 83 | 0.28 | 1.06 | 0.356 | 0.800 | 1.000 | 1379 | tags=18%, list=11%, signal=20% |
| 2764 | GOBP\_IMMUNOLOGICAL\_MEMORY\_FORMATION\_PROCESS |  | 5 | 0.55 | 1.06 | 0.435 | 0.800 | 1.000 | 4333 | tags=80%, list=36%, signal=125% |
| 2765 | GOCC\_CHROMOCENTER |  | 10 | 0.45 | 1.06 | 0.396 | 0.800 | 1.000 | 3733 | tags=60%, list=31%, signal=87% |
| 2766 | GOBP\_PLUS\_END\_DIRECTED\_ORGANELLE\_TRANSPORT\_ALONG\_MICROTUBULE |  | 7 | 0.49 | 1.06 | 0.402 | 0.801 | 1.000 | 1656 | tags=43%, list=14%, signal=50% |
| 2767 | GOBP\_SYNAPTONEMAL\_COMPLEX\_ORGANIZATION |  | 11 | 0.44 | 1.06 | 0.399 | 0.801 | 1.000 | 3153 | tags=45%, list=26%, signal=62% |
| 2768 | GOBP\_ACTIN\_FILAMENT\_BASED\_MOVEMENT |  | 94 | 0.27 | 1.06 | 0.355 | 0.801 | 1.000 | 3106 | tags=39%, list=26%, signal=53% |
| 2769 | GOBP\_T\_CELL\_DIFFERENTIATION\_INVOLVED\_IN\_IMMUNE\_RESPONSE |  | 44 | 0.31 | 1.06 | 0.364 | 0.801 | 1.000 | 2627 | tags=30%, list=22%, signal=38% |
| 2770 | GOBP\_RESPONSE\_TO\_ACID\_CHEMICAL |  | 84 | 0.28 | 1.06 | 0.350 | 0.801 | 1.000 | 3113 | tags=33%, list=26%, signal=45% |
| 2771 | GOBP\_REGULATION\_OF\_CELLULAR\_RESPONSE\_TO\_VASCULAR\_ENDOTHELIAL\_GROWTH\_FACTOR\_STIMULUS |  | 17 | 0.39 | 1.06 | 0.394 | 0.801 | 1.000 | 3792 | tags=59%, list=32%, signal=86% |
| 2772 | GOBP\_RESPONSE\_TO\_X\_RAY |  | 24 | 0.36 | 1.06 | 0.382 | 0.801 | 1.000 | 1495 | tags=29%, list=12%, signal=33% |
| 2773 | GOBP\_NEGATIVE\_REGULATION\_OF\_CD4\_POSITIVE\_ALPHA\_BETA\_T\_CELL\_PROLIFERATION |  | 4 | 0.58 | 1.06 | 0.436 | 0.802 | 1.000 | 682 | tags=50%, list=6%, signal=53% |
| 2774 | GOBP\_ENDODERM\_FORMATION |  | 35 | 0.33 | 1.06 | 0.373 | 0.802 | 1.000 | 2920 | tags=31%, list=24%, signal=41% |
| 2775 | GOBP\_CELLULAR\_RESPONSE\_TO\_HEPARIN |  | 5 | 0.55 | 1.06 | 0.431 | 0.802 | 1.000 | 69 | tags=20%, list=1%, signal=20% |
| 2776 | GOBP\_REGULATION\_OF\_SPONTANEOUS\_SYNAPTIC\_TRANSMISSION |  | 4 | 0.58 | 1.06 | 0.445 | 0.802 | 1.000 | 3030 | tags=50%, list=25%, signal=67% |
| 2777 | GOCC\_KINESIN\_COMPLEX |  | 38 | 0.32 | 1.06 | 0.377 | 0.802 | 1.000 | 2633 | tags=42%, list=22%, signal=54% |
| 2778 | GOBP\_POSITIVE\_REGULATION\_OF\_COLD\_INDUCED\_THERMOGENESIS |  | 65 | 0.29 | 1.06 | 0.367 | 0.802 | 1.000 | 1967 | tags=31%, list=16%, signal=37% |
| 2779 | GOMF\_R\_SMAD\_BINDING |  | 16 | 0.40 | 1.06 | 0.391 | 0.802 | 1.000 | 1301 | tags=25%, list=11%, signal=28% |
| 2780 | GOCC\_MITOTIC\_COHESIN\_COMPLEX |  | 3 | 0.63 | 1.05 | 0.449 | 0.802 | 1.000 | 2359 | tags=33%, list=20%, signal=41% |
| 2781 | GOMF\_SIGNALING\_RECEPTOR\_COMPLEX\_ADAPTOR\_ACTIVITY |  | 33 | 0.33 | 1.05 | 0.373 | 0.802 | 1.000 | 711 | tags=9%, list=6%, signal=10% |
| 2782 | GOBP\_ACTIVATION\_OF\_PROTEIN\_KINASE\_ACTIVITY |  | 240 | 0.24 | 1.05 | 0.324 | 0.802 | 1.000 | 2351 | tags=23%, list=20%, signal=28% |
| 2783 | GOBP\_CELLULAR\_RESPONSE\_TO\_VASCULAR\_ENDOTHELIAL\_GROWTH\_FACTOR\_STIMULUS |  | 51 | 0.30 | 1.05 | 0.375 | 0.802 | 1.000 | 3493 | tags=37%, list=29%, signal=52% |
| 2784 | GOBP\_TUMOR\_NECROSIS\_FACTOR\_MEDIATED\_SIGNALING\_PATHWAY |  | 114 | 0.26 | 1.05 | 0.343 | 0.802 | 1.000 | 1366 | tags=14%, list=11%, signal=16% |
| 2785 | GOBP\_POSITIVE\_REGULATION\_OF\_RHO\_PROTEIN\_SIGNAL\_TRANSDUCTION |  | 26 | 0.35 | 1.05 | 0.370 | 0.802 | 1.000 | 2188 | tags=31%, list=18%, signal=38% |
| 2786 | GOBP\_REGULATION\_OF\_PHOSPHOLIPID\_METABOLIC\_PROCESS |  | 28 | 0.34 | 1.05 | 0.384 | 0.802 | 1.000 | 1396 | tags=21%, list=12%, signal=24% |
| 2787 | GOBP\_MACROPHAGE\_ACTIVATION |  | 60 | 0.29 | 1.05 | 0.359 | 0.802 | 1.000 | 651 | tags=10%, list=5%, signal=11% |
| 2788 | GOBP\_REGULATION\_OF\_VASCULAR\_PERMEABILITY |  | 30 | 0.34 | 1.05 | 0.383 | 0.802 | 1.000 | 2083 | tags=30%, list=17%, signal=36% |
| 2789 | GOBP\_NEGATIVE\_REGULATION\_OF\_ORGANIC\_ACID\_TRANSPORT |  | 11 | 0.43 | 1.05 | 0.400 | 0.802 | 1.000 | 1055 | tags=27%, list=9%, signal=30% |
| 2790 | GOBP\_ADENYLATE\_CYCLASE\_INHIBITING\_G\_PROTEIN\_COUPLED\_RECEPTOR\_SIGNALING\_PATHWAY |  | 42 | 0.32 | 1.05 | 0.376 | 0.802 | 1.000 | 1300 | tags=21%, list=11%, signal=24% |
| 2791 | GOBP\_GLUCOSE\_METABOLIC\_PROCESS |  | 148 | 0.25 | 1.05 | 0.337 | 0.802 | 1.000 | 1427 | tags=18%, list=12%, signal=20% |
| 2792 | GOBP\_GOLGI\_TO\_LYSOSOME\_TRANSPORT |  | 8 | 0.47 | 1.05 | 0.401 | 0.802 | 1.000 | 2290 | tags=50%, list=19%, signal=62% |
| 2793 | GOBP\_UNSATURATED\_FATTY\_ACID\_METABOLIC\_PROCESS |  | 64 | 0.29 | 1.05 | 0.360 | 0.802 | 1.000 | 1283 | tags=16%, list=11%, signal=17% |
| 2794 | GOBP\_NUCLEOSIDE\_PHOSPHATE\_BIOSYNTHETIC\_PROCESS |  | 170 | 0.25 | 1.05 | 0.335 | 0.802 | 1.000 | 1433 | tags=16%, list=12%, signal=18% |
| 2795 | GOMF\_DISORDERED\_DOMAIN\_SPECIFIC\_BINDING |  | 22 | 0.37 | 1.05 | 0.383 | 0.802 | 1.000 | 3248 | tags=45%, list=27%, signal=62% |
| 2796 | GOBP\_NEGATIVE\_REGULATION\_OF\_DNA\_REPAIR |  | 23 | 0.36 | 1.05 | 0.397 | 0.802 | 1.000 | 728 | tags=13%, list=6%, signal=14% |
| 2797 | GOBP\_REGULATION\_OF\_ADAPTIVE\_IMMUNE\_RESPONSE |  | 95 | 0.27 | 1.05 | 0.357 | 0.802 | 1.000 | 1305 | tags=15%, list=11%, signal=16% |
| 2798 | GOCC\_ZYMOGEN\_GRANULE |  | 7 | 0.49 | 1.05 | 0.423 | 0.802 | 1.000 | 1237 | tags=29%, list=10%, signal=32% |
| 2799 | GOCC\_MICROTUBULE\_END |  | 26 | 0.35 | 1.05 | 0.388 | 0.802 | 1.000 | 4415 | tags=69%, list=37%, signal=109% |
| 2800 | GOBP\_POSITIVE\_REGULATION\_OF\_ERYTHROCYTE\_DIFFERENTIATION |  | 23 | 0.36 | 1.05 | 0.384 | 0.802 | 1.000 | 1909 | tags=26%, list=16%, signal=31% |
| 2801 | GOBP\_CALCIUM\_ION\_TRANSPORT |  | 247 | 0.24 | 1.05 | 0.322 | 0.802 | 1.000 | 2196 | tags=21%, list=18%, signal=25% |
| 2802 | GOCC\_EXTRINSIC\_COMPONENT\_OF\_CYTOPLASMIC\_SIDE\_OF\_PLASMA\_MEMBRANE |  | 69 | 0.29 | 1.05 | 0.360 | 0.802 | 1.000 | 2798 | tags=33%, list=23%, signal=43% |
| 2803 | GOBP\_MEDIUM\_CHAIN\_FATTY\_ACID\_CATABOLIC\_PROCESS |  | 3 | 0.63 | 1.05 | 0.455 | 0.802 | 1.000 | 1054 | tags=67%, list=9%, signal=73% |
| 2804 | GOBP\_DNA\_LIGATION\_INVOLVED\_IN\_DNA\_REPAIR |  | 4 | 0.59 | 1.05 | 0.446 | 0.802 | 1.000 | 3591 | tags=50%, list=30%, signal=71% |
| 2805 | GOBP\_REGULATION\_OF\_TRANSLATION\_IN\_RESPONSE\_TO\_STRESS |  | 18 | 0.38 | 1.05 | 0.398 | 0.803 | 1.000 | 997 | tags=17%, list=8%, signal=18% |
| 2806 | GOBP\_CELLULAR\_RESPONSE\_TO\_HYDROPEROXIDE |  | 7 | 0.49 | 1.05 | 0.425 | 0.803 | 1.000 | 1326 | tags=43%, list=11%, signal=48% |
[truncated: 420,166 more chars]
