# Supplementary figures and images for: Time-series transcriptome analysis identified differentially expressed genes in broiler chicken infected with mixed Eimeria species (part 2 of 2)
Source: Front Genet. 2022 Aug 8;13:886781. doi: 10.3389/fgene.2022.886781 (PMC9393255; doi:10.3389/fgene.2022.886781)

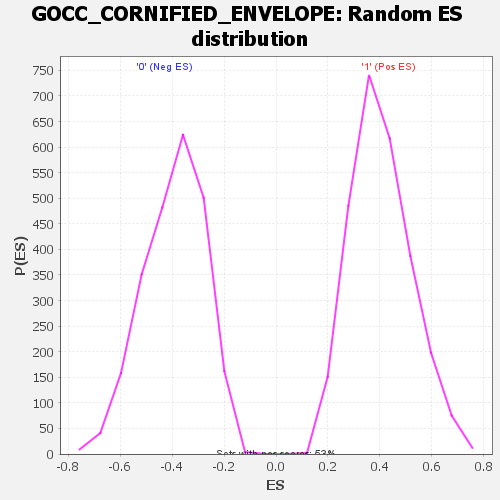

Supplement: Supplementary file 2 [file DataSheet1.ZIP › 4dpi_GO.Gsea.1625071243202/gset_rnd_es_dist_1823.png]

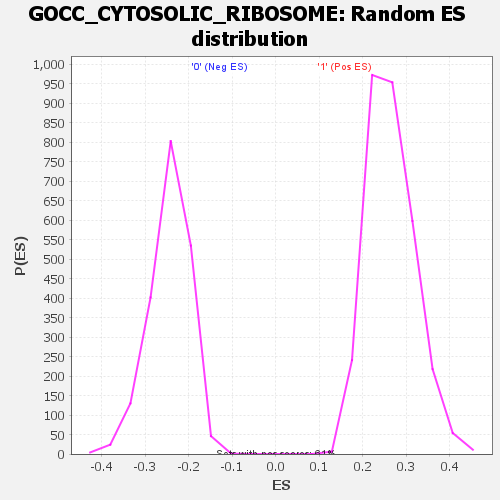

Supplement: Supplementary file 2 [file DataSheet1.ZIP › 4dpi_GO.Gsea.1625071243202/gset_rnd_es_dist_1826.png]

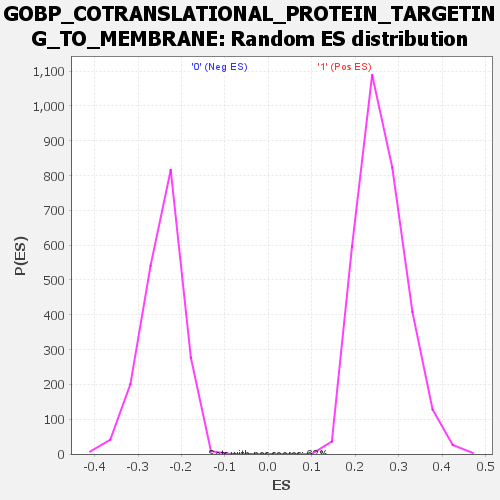

Supplement: Supplementary file 2 [file DataSheet1.ZIP › 4dpi_GO.Gsea.1625071243202/gset_rnd_es_dist_1829.png]

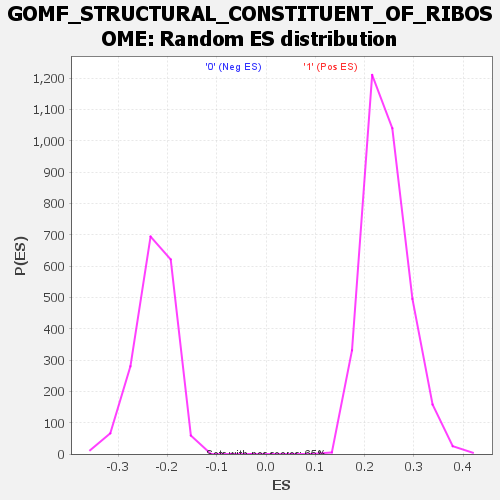

Supplement: Supplementary file 2 [file DataSheet1.ZIP › 4dpi_GO.Gsea.1625071243202/gset_rnd_es_dist_1832.png]

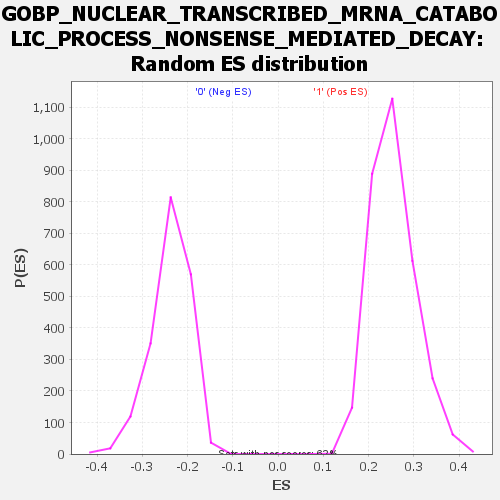

Supplement: Supplementary file 2 [file DataSheet1.ZIP › 4dpi_GO.Gsea.1625071243202/gset_rnd_es_dist_1835.png]

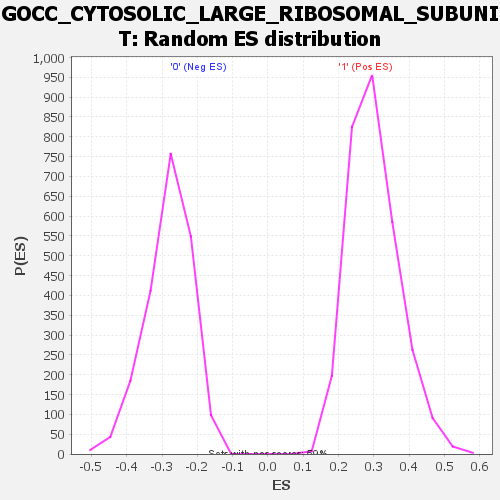

Supplement: Supplementary file 2 [file DataSheet1.ZIP › 4dpi_GO.Gsea.1625071243202/gset_rnd_es_dist_1838.png]

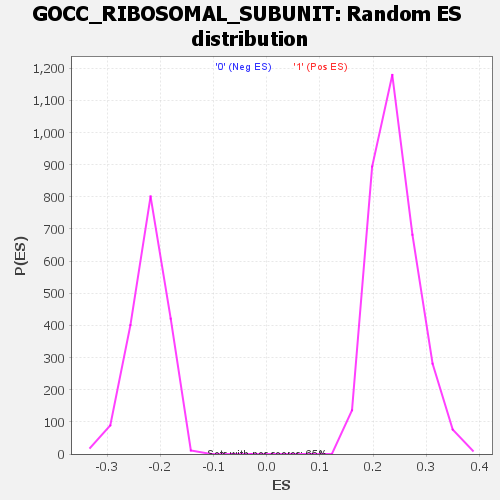

Supplement: Supplementary file 2 [file DataSheet1.ZIP › 4dpi_GO.Gsea.1625071243202/gset_rnd_es_dist_1841.png]

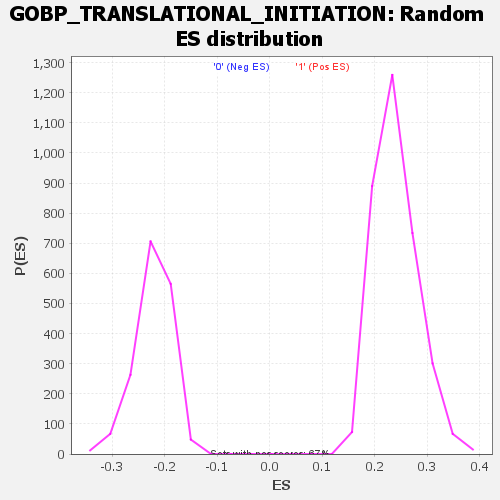

Supplement: Supplementary file 2 [file DataSheet1.ZIP › 4dpi_GO.Gsea.1625071243202/gset_rnd_es_dist_1844.png]

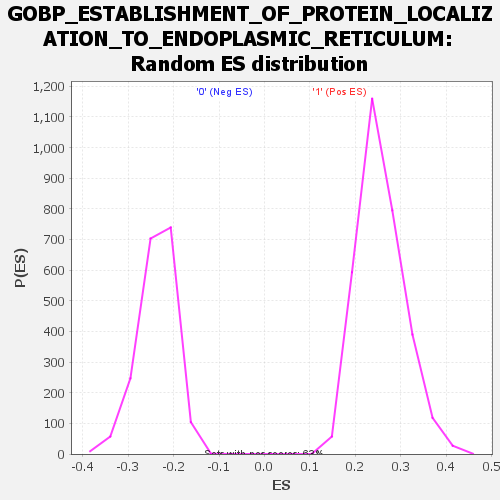

Supplement: Supplementary file 2 [file DataSheet1.ZIP › 4dpi_GO.Gsea.1625071243202/gset_rnd_es_dist_1847.png]

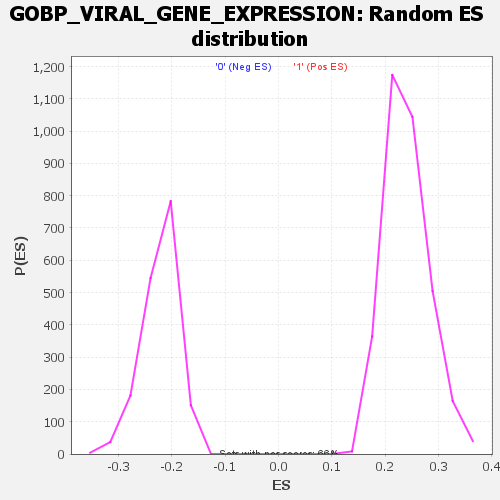

Supplement: Supplementary file 2 [file DataSheet1.ZIP › 4dpi_GO.Gsea.1625071243202/gset_rnd_es_dist_1850.png]

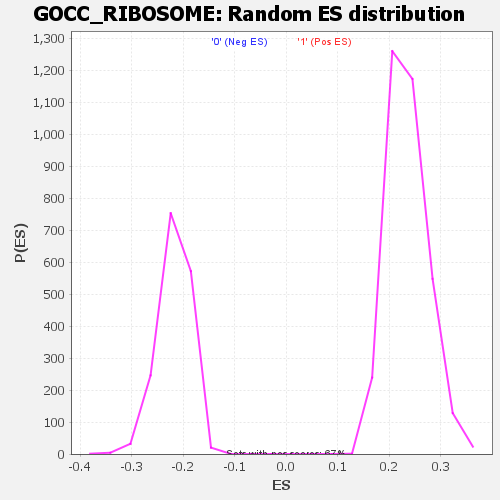

Supplement: Supplementary file 2 [file DataSheet1.ZIP › 4dpi_GO.Gsea.1625071243202/gset_rnd_es_dist_1853.png]

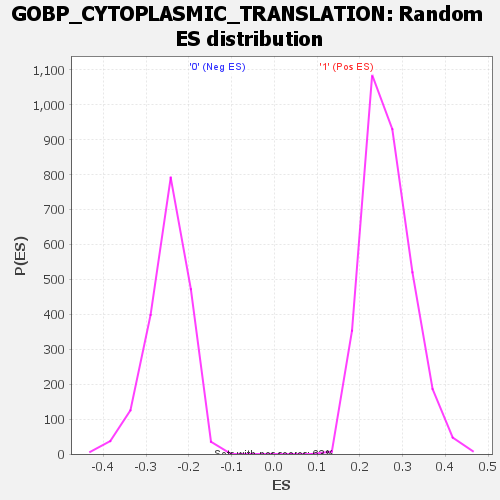

Supplement: Supplementary file 2 [file DataSheet1.ZIP › 4dpi_GO.Gsea.1625071243202/gset_rnd_es_dist_1856.png]

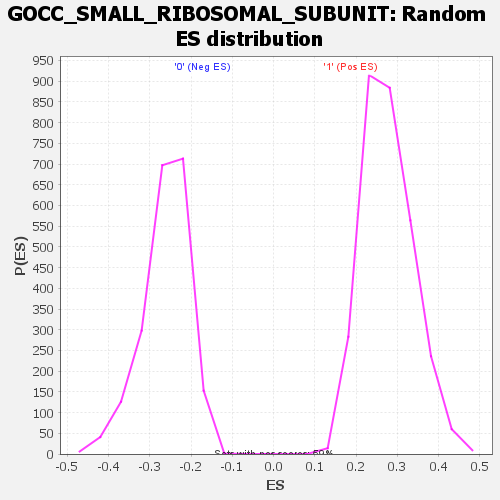

Supplement: Supplementary file 2 [file DataSheet1.ZIP › 4dpi_GO.Gsea.1625071243202/gset_rnd_es_dist_1859.png]

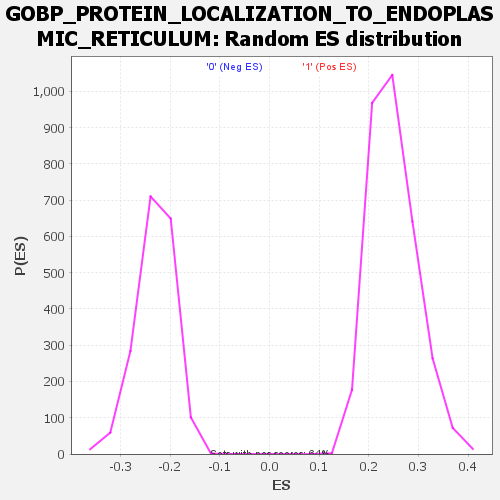

Supplement: Supplementary file 2 [file DataSheet1.ZIP › 4dpi_GO.Gsea.1625071243202/gset_rnd_es_dist_1862.png]
