# Supplementary figures and images for: Time-series transcriptome analysis identified differentially expressed genes in broiler chicken infected with mixed Eimeria species (part 1 of 2)
Source: Front Genet. 2022 Aug 8;13:886781. doi: 10.3389/fgene.2022.886781 (PMC9393255; doi:10.3389/fgene.2022.886781)

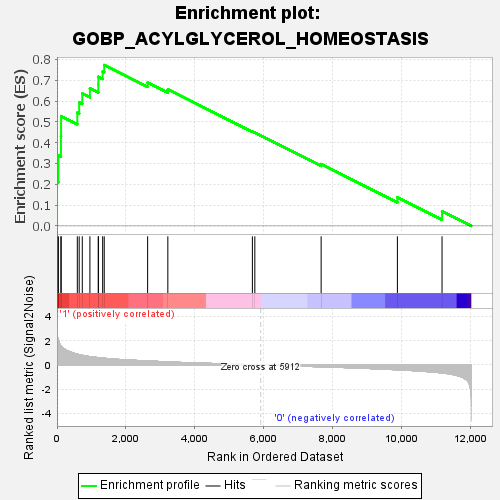

Supplement: Supplementary file 2 [file DataSheet1.ZIP › 4dpi_GO.Gsea.1625071243202/enplot_GOBP_ACYLGLYCEROL_HOMEOSTASIS_1800.png]

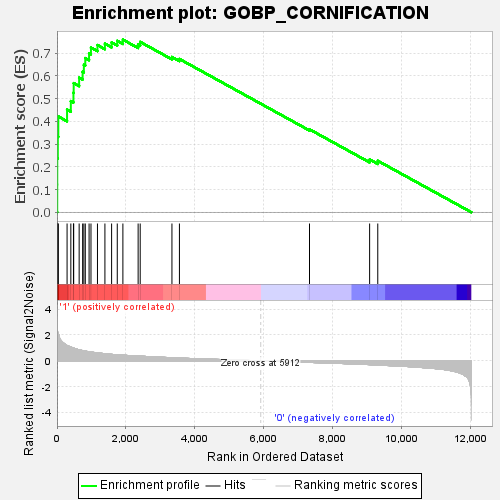

Supplement: Supplementary file 2 [file DataSheet1.ZIP › 4dpi_GO.Gsea.1625071243202/enplot_GOBP_CORNIFICATION_1773.png]

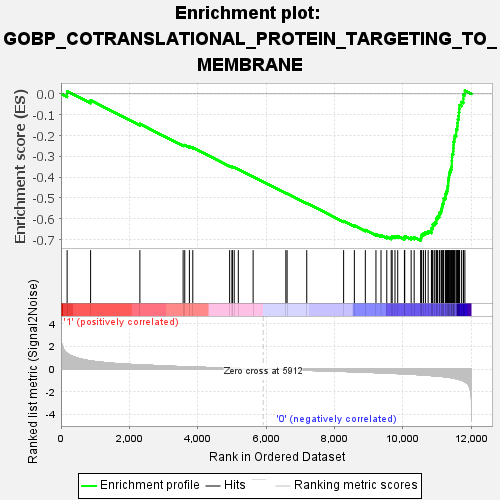

Supplement: Supplementary file 2 [file DataSheet1.ZIP › 4dpi_GO.Gsea.1625071243202/enplot_GOBP_COTRANSLATIONAL_PROTEIN_TARGETING_TO_MEMBRANE_1827.png]

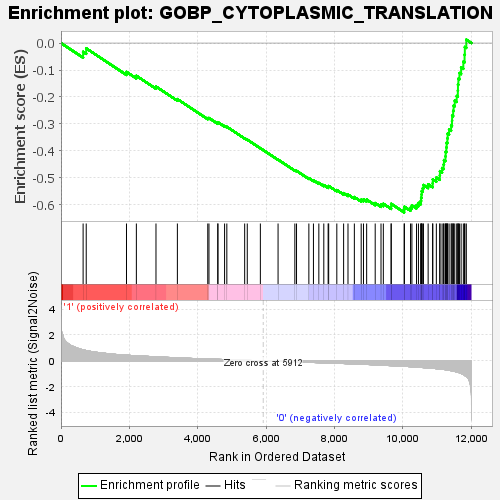

Supplement: Supplementary file 2 [file DataSheet1.ZIP › 4dpi_GO.Gsea.1625071243202/enplot_GOBP_CYTOPLASMIC_TRANSLATION_1854.png]

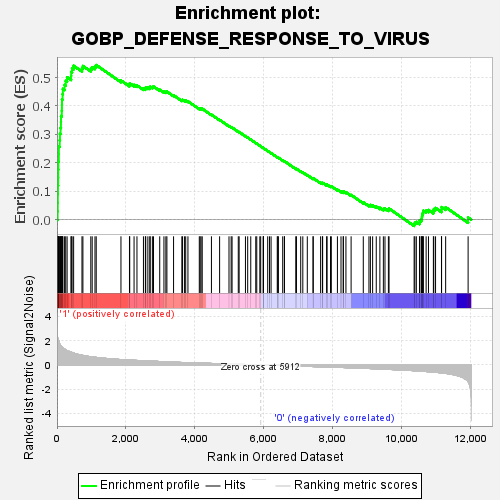

Supplement: Supplementary file 2 [file DataSheet1.ZIP › 4dpi_GO.Gsea.1625071243202/enplot_GOBP_DEFENSE_RESPONSE_TO_VIRUS_1782.png]

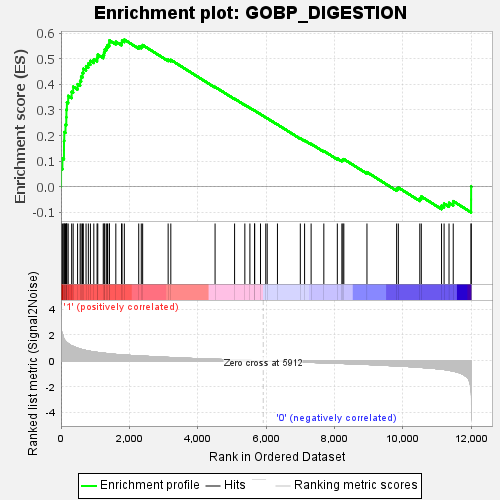

Supplement: Supplementary file 2 [file DataSheet1.ZIP › 4dpi_GO.Gsea.1625071243202/enplot_GOBP_DIGESTION_1818.png]

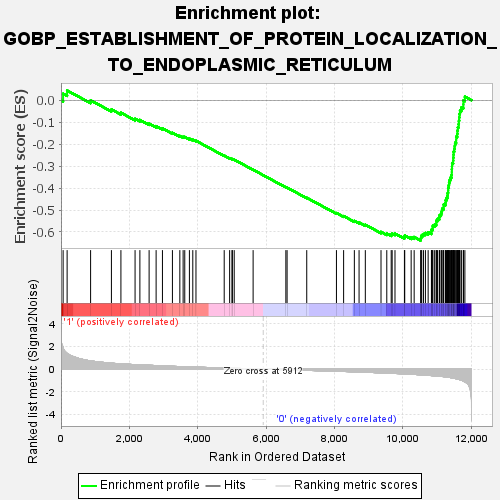

Supplement: Supplementary file 2 [file DataSheet1.ZIP › 4dpi_GO.Gsea.1625071243202/enplot_GOBP_ESTABLISHMENT_OF_PROTEIN_LOCALIZATION_TO_ENDOPLASMIC_RETICULUM_1845.png]

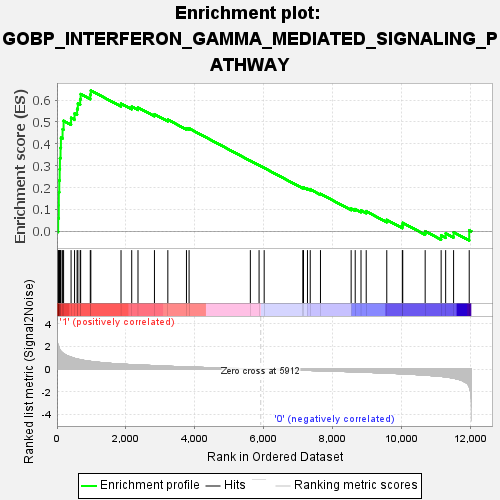

Supplement: Supplementary file 2 [file DataSheet1.ZIP › 4dpi_GO.Gsea.1625071243202/enplot_GOBP_INTERFERON_GAMMA_MEDIATED_SIGNALING_PATHWAY_1797.png]

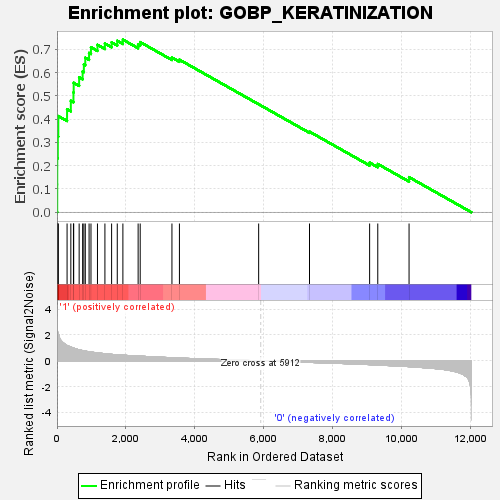

Supplement: Supplementary file 2 [file DataSheet1.ZIP › 4dpi_GO.Gsea.1625071243202/enplot_GOBP_KERATINIZATION_1776.png]

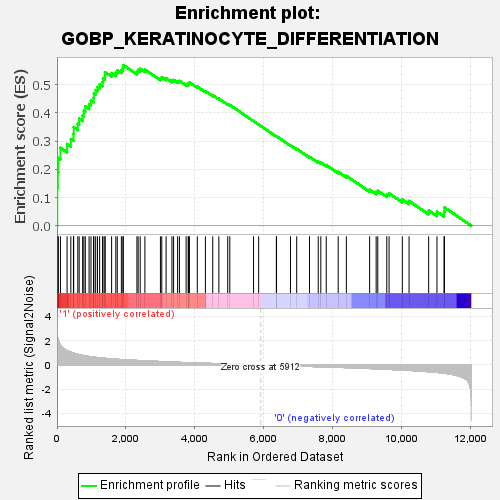

Supplement: Supplementary file 2 [file DataSheet1.ZIP › 4dpi_GO.Gsea.1625071243202/enplot_GOBP_KERATINOCYTE_DIFFERENTIATION_1812.png]

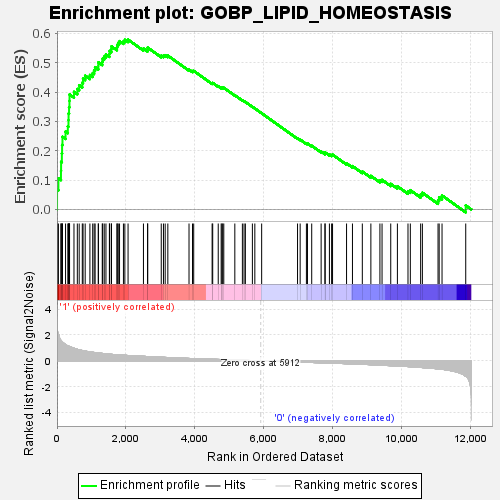

Supplement: Supplementary file 2 [file DataSheet1.ZIP › 4dpi_GO.Gsea.1625071243202/enplot_GOBP_LIPID_HOMEOSTASIS_1779.png]

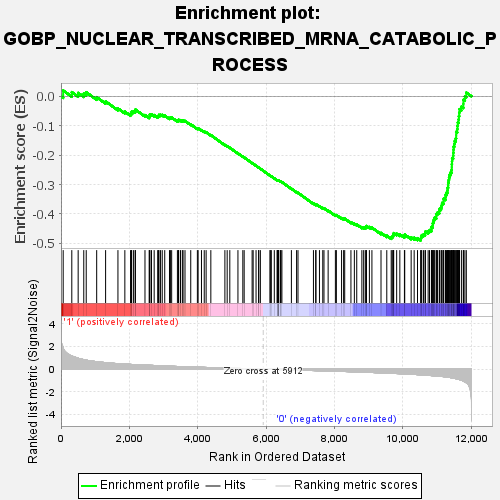

Supplement: Supplementary file 2 [file DataSheet1.ZIP › 4dpi_GO.Gsea.1625071243202/enplot_GOBP_NUCLEAR_TRANSCRIBED_MRNA_CATABOLIC_PROCESS_1881.png]

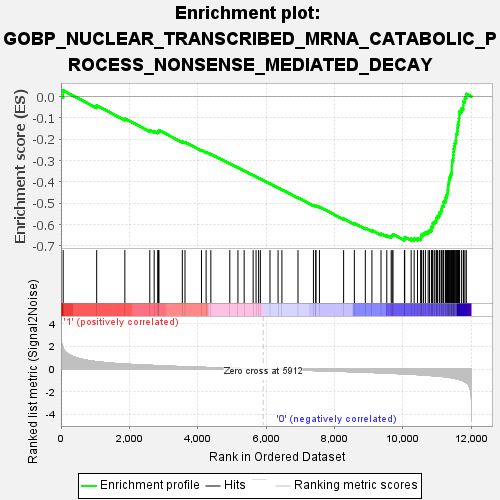

Supplement: Supplementary file 2 [file DataSheet1.ZIP › 4dpi_GO.Gsea.1625071243202/enplot_GOBP_NUCLEAR_TRANSCRIBED_MRNA_CATABOLIC_PROCESS_NONSENSE_MEDIATED_DECAY_1833.png]

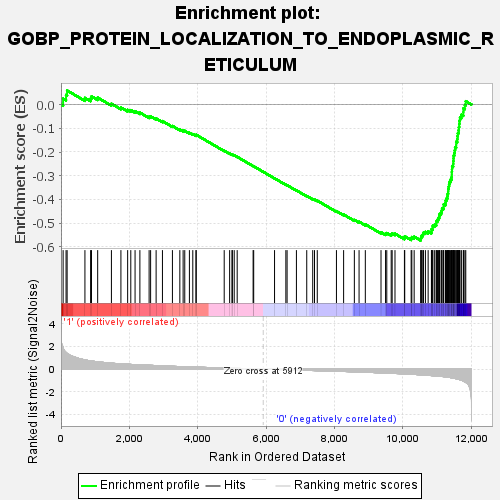

Supplement: Supplementary file 2 [file DataSheet1.ZIP › 4dpi_GO.Gsea.1625071243202/enplot_GOBP_PROTEIN_LOCALIZATION_TO_ENDOPLASMIC_RETICULUM_1860.png]

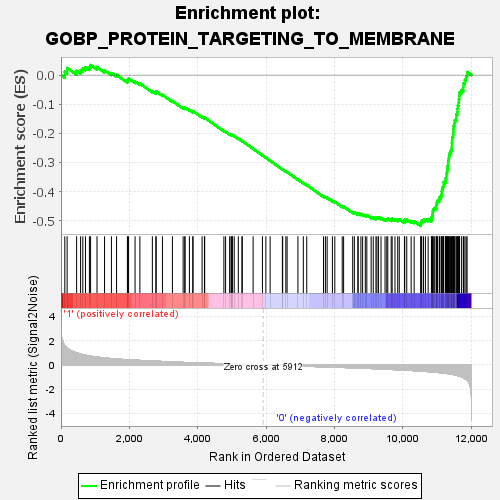

Supplement: Supplementary file 2 [file DataSheet1.ZIP › 4dpi_GO.Gsea.1625071243202/enplot_GOBP_PROTEIN_TARGETING_TO_MEMBRANE_1875.png]

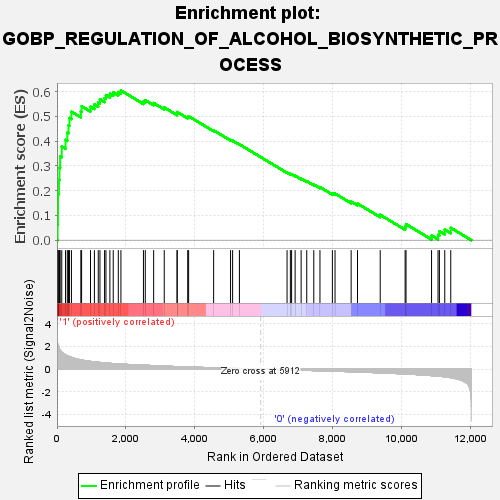

Supplement: Supplementary file 2 [file DataSheet1.ZIP › 4dpi_GO.Gsea.1625071243202/enplot_GOBP_REGULATION_OF_ALCOHOL_BIOSYNTHETIC_PROCESS_1806.png]

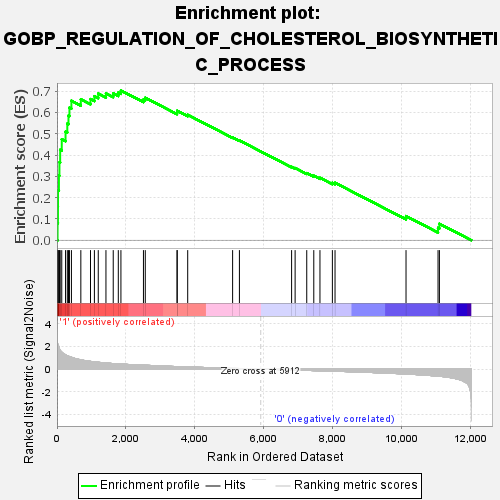

Supplement: Supplementary file 2 [file DataSheet1.ZIP › 4dpi_GO.Gsea.1625071243202/enplot_GOBP_REGULATION_OF_CHOLESTEROL_BIOSYNTHETIC_PROCESS_1767.png]

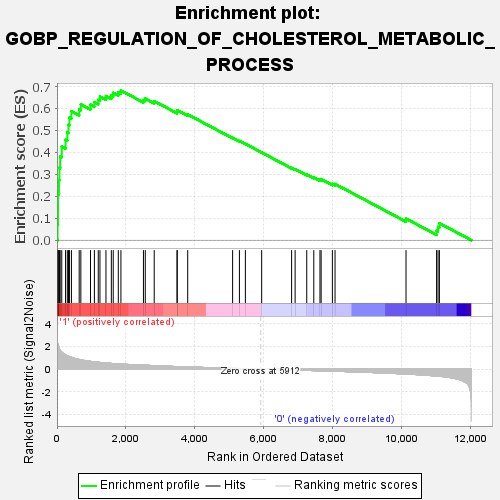

Supplement: Supplementary file 2 [file DataSheet1.ZIP › 4dpi_GO.Gsea.1625071243202/enplot_GOBP_REGULATION_OF_CHOLESTEROL_METABOLIC_PROCESS_1770.png]

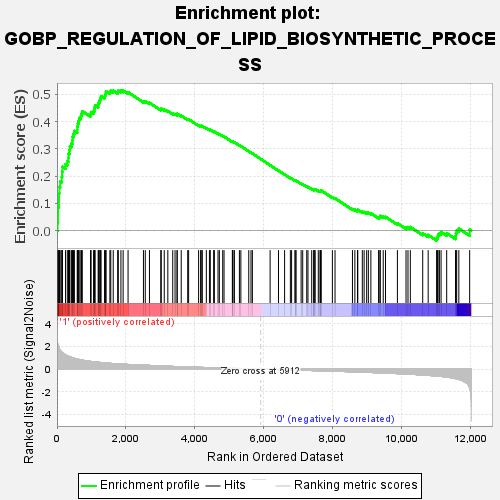

Supplement: Supplementary file 2 [file DataSheet1.ZIP › 4dpi_GO.Gsea.1625071243202/enplot_GOBP_REGULATION_OF_LIPID_BIOSYNTHETIC_PROCESS_1815.png]

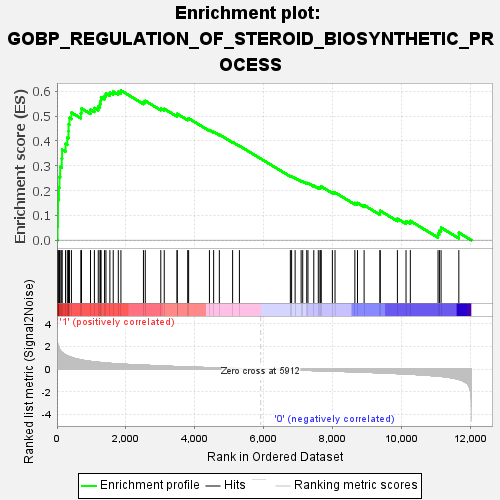

Supplement: Supplementary file 2 [file DataSheet1.ZIP › 4dpi_GO.Gsea.1625071243202/enplot_GOBP_REGULATION_OF_STEROID_BIOSYNTHETIC_PROCESS_1788.png]

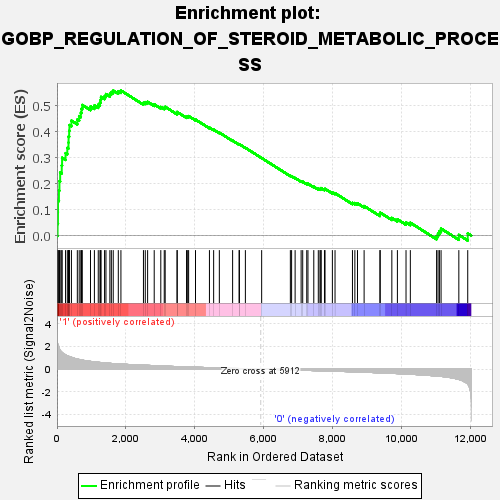

Supplement: Supplementary file 2 [file DataSheet1.ZIP › 4dpi_GO.Gsea.1625071243202/enplot_GOBP_REGULATION_OF_STEROID_METABOLIC_PROCESS_1809.png]

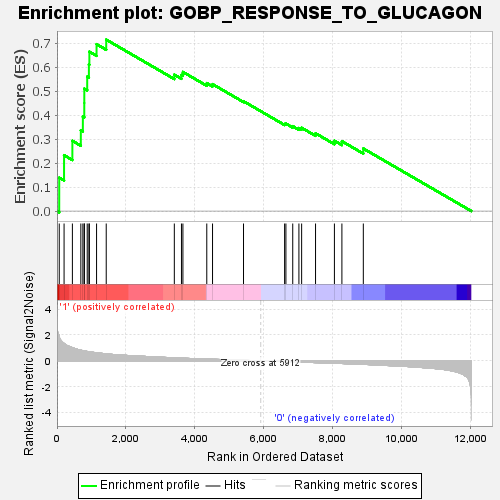

Supplement: Supplementary file 2 [file DataSheet1.ZIP › 4dpi_GO.Gsea.1625071243202/enplot_GOBP_RESPONSE_TO_GLUCAGON_1794.png]

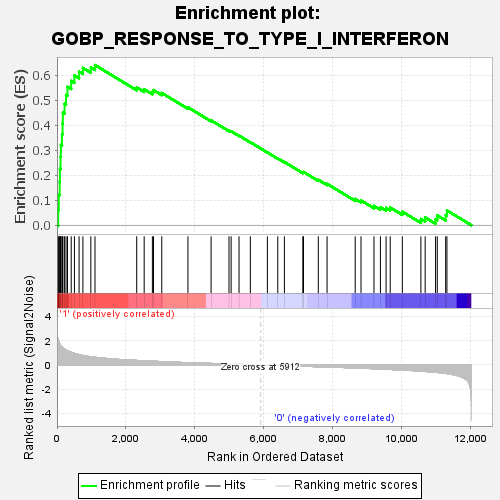

Supplement: Supplementary file 2 [file DataSheet1.ZIP › 4dpi_GO.Gsea.1625071243202/enplot_GOBP_RESPONSE_TO_TYPE_I_INTERFERON_1785.png]

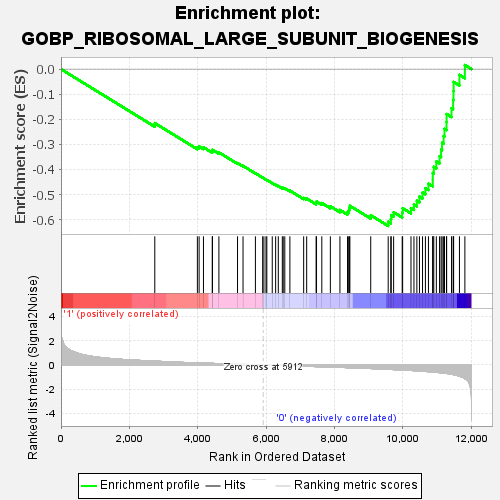

Supplement: Supplementary file 2 [file DataSheet1.ZIP › 4dpi_GO.Gsea.1625071243202/enplot_GOBP_RIBOSOMAL_LARGE_SUBUNIT_BIOGENESIS_1869.png]

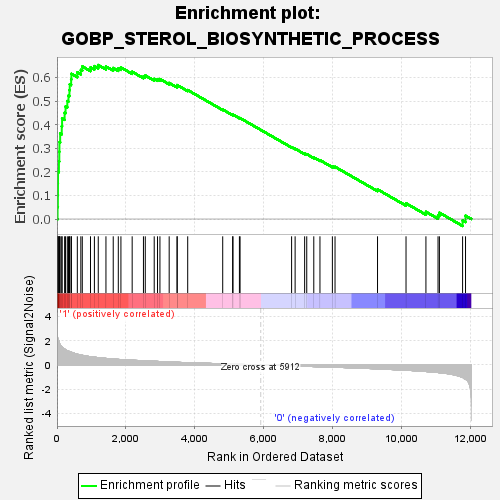

Supplement: Supplementary file 2 [file DataSheet1.ZIP › 4dpi_GO.Gsea.1625071243202/enplot_GOBP_STEROL_BIOSYNTHETIC_PROCESS_1764.png]

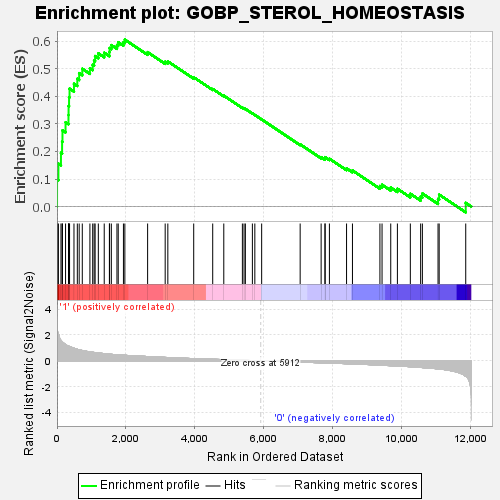

Supplement: Supplementary file 2 [file DataSheet1.ZIP › 4dpi_GO.Gsea.1625071243202/enplot_GOBP_STEROL_HOMEOSTASIS_1803.png]

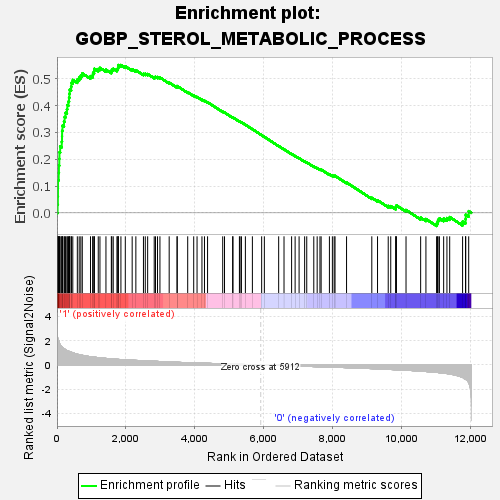

Supplement: Supplementary file 2 [file DataSheet1.ZIP › 4dpi_GO.Gsea.1625071243202/enplot_GOBP_STEROL_METABOLIC_PROCESS_1791.png]

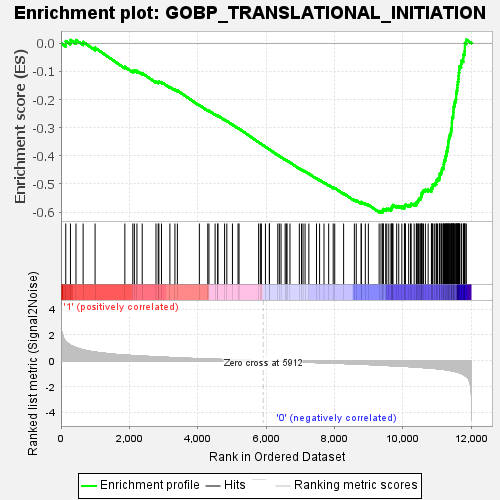

Supplement: Supplementary file 2 [file DataSheet1.ZIP › 4dpi_GO.Gsea.1625071243202/enplot_GOBP_TRANSLATIONAL_INITIATION_1842.png]

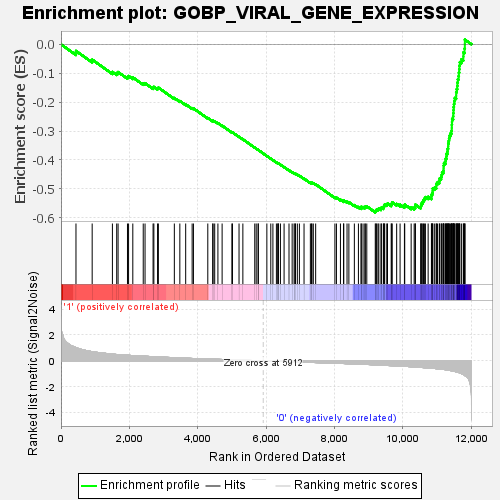

Supplement: Supplementary file 2 [file DataSheet1.ZIP › 4dpi_GO.Gsea.1625071243202/enplot_GOBP_VIRAL_GENE_EXPRESSION_1848.png]

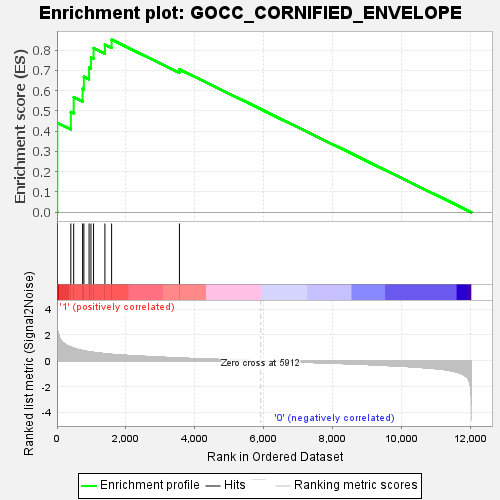

Supplement: Supplementary file 2 [file DataSheet1.ZIP › 4dpi_GO.Gsea.1625071243202/enplot_GOCC_CORNIFIED_ENVELOPE_1821.png]

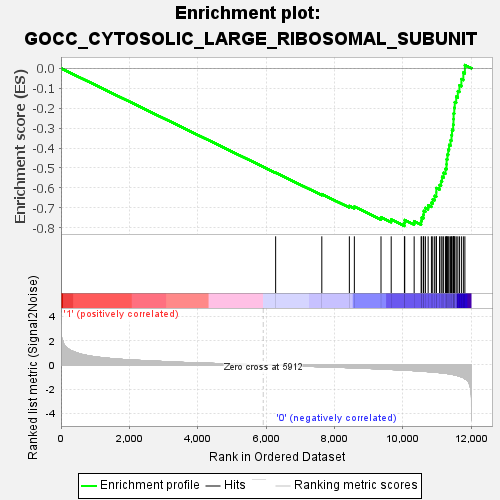

Supplement: Supplementary file 2 [file DataSheet1.ZIP › 4dpi_GO.Gsea.1625071243202/enplot_GOCC_CYTOSOLIC_LARGE_RIBOSOMAL_SUBUNIT_1836.png]

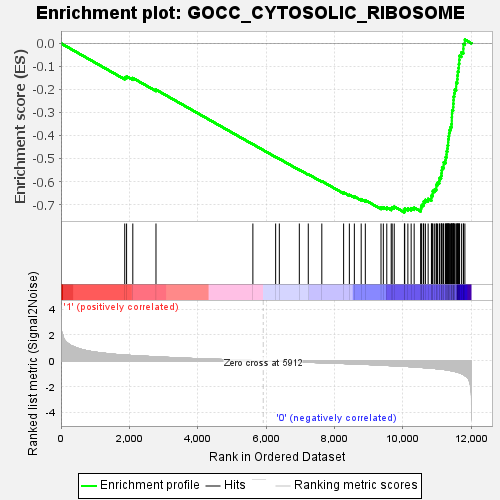

Supplement: Supplementary file 2 [file DataSheet1.ZIP › 4dpi_GO.Gsea.1625071243202/enplot_GOCC_CYTOSOLIC_RIBOSOME_1824.png]

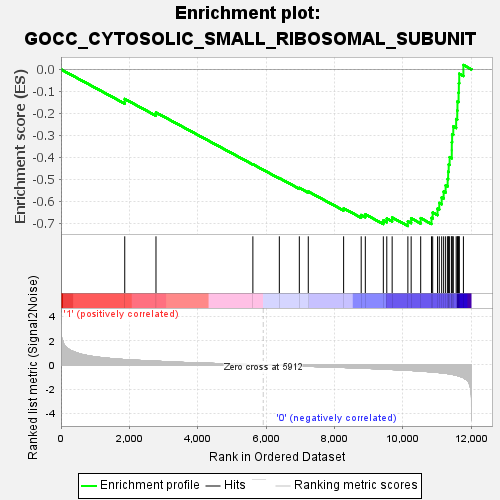

Supplement: Supplementary file 2 [file DataSheet1.ZIP › 4dpi_GO.Gsea.1625071243202/enplot_GOCC_CYTOSOLIC_SMALL_RIBOSOMAL_SUBUNIT_1863.png]

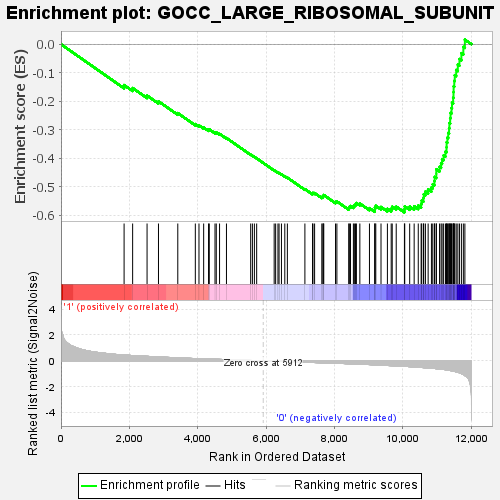

Supplement: Supplementary file 2 [file DataSheet1.ZIP › 4dpi_GO.Gsea.1625071243202/enplot_GOCC_LARGE_RIBOSOMAL_SUBUNIT_1866.png]

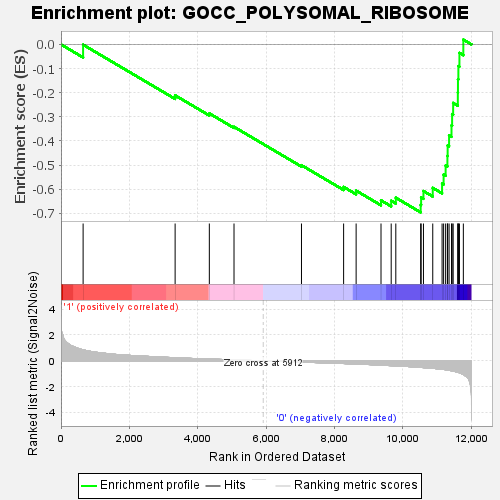

Supplement: Supplementary file 2 [file DataSheet1.ZIP › 4dpi_GO.Gsea.1625071243202/enplot_GOCC_POLYSOMAL_RIBOSOME_1878.png]

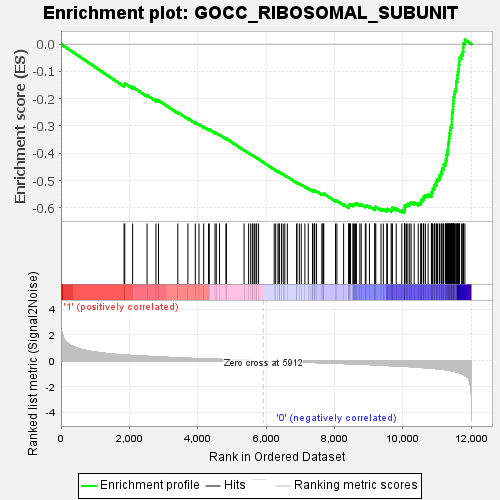

Supplement: Supplementary file 2 [file DataSheet1.ZIP › 4dpi_GO.Gsea.1625071243202/enplot_GOCC_RIBOSOMAL_SUBUNIT_1839.png]

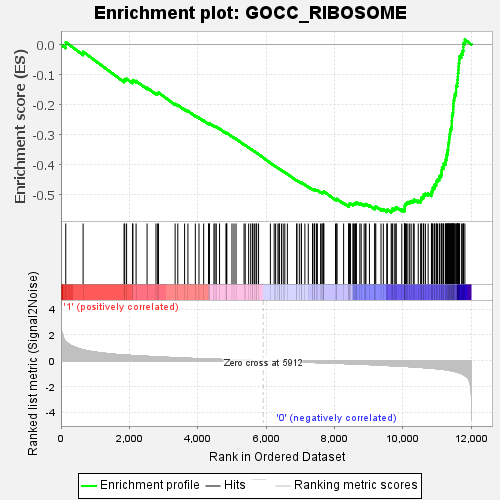

Supplement: Supplementary file 2 [file DataSheet1.ZIP › 4dpi_GO.Gsea.1625071243202/enplot_GOCC_RIBOSOME_1851.png]

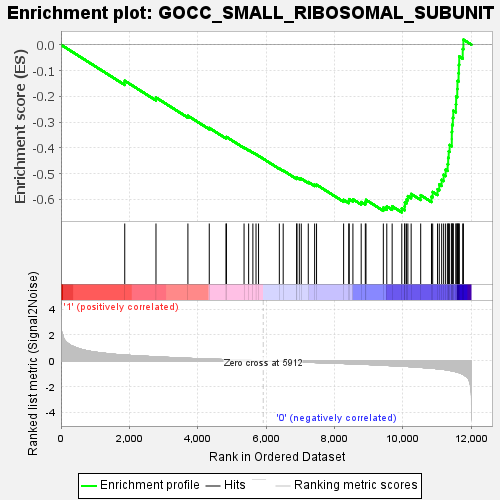

Supplement: Supplementary file 2 [file DataSheet1.ZIP › 4dpi_GO.Gsea.1625071243202/enplot_GOCC_SMALL_RIBOSOMAL_SUBUNIT_1857.png]

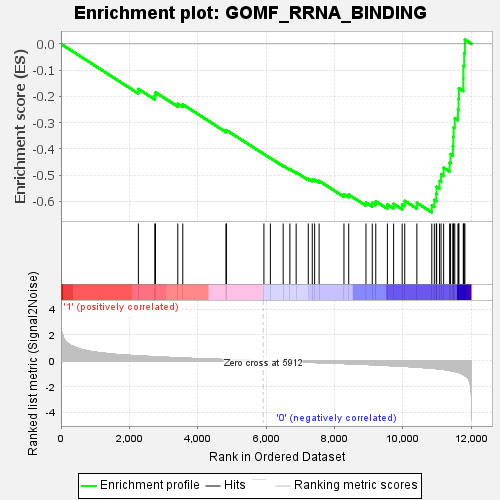

Supplement: Supplementary file 2 [file DataSheet1.ZIP › 4dpi_GO.Gsea.1625071243202/enplot_GOMF_RRNA_BINDING_1872.png]

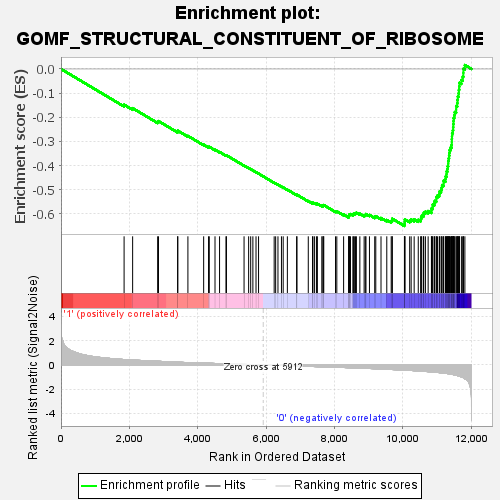

Supplement: Supplementary file 2 [file DataSheet1.ZIP › 4dpi_GO.Gsea.1625071243202/enplot_GOMF_STRUCTURAL_CONSTITUENT_OF_RIBOSOME_1830.png]

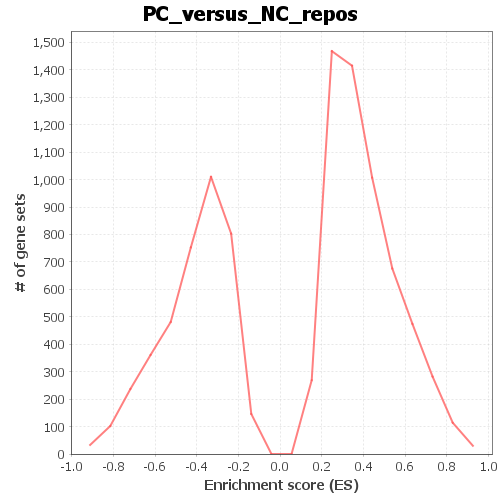

Supplement: Supplementary file 2 [file DataSheet1.ZIP › 4dpi_GO.Gsea.1625071243202/global_es_histogram.png]

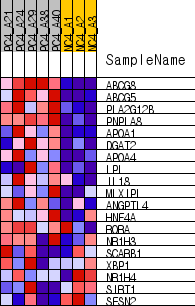

Supplement: Supplementary file 2 [file DataSheet1.ZIP › 4dpi_GO.Gsea.1625071243202/GOBP_ACYLGLYCEROL_HOMEOSTASIS_1801.png]

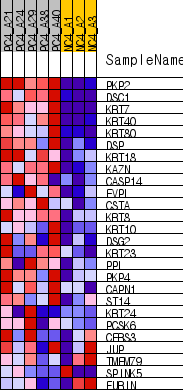

Supplement: Supplementary file 2 [file DataSheet1.ZIP › 4dpi_GO.Gsea.1625071243202/GOBP_CORNIFICATION_1774.png]

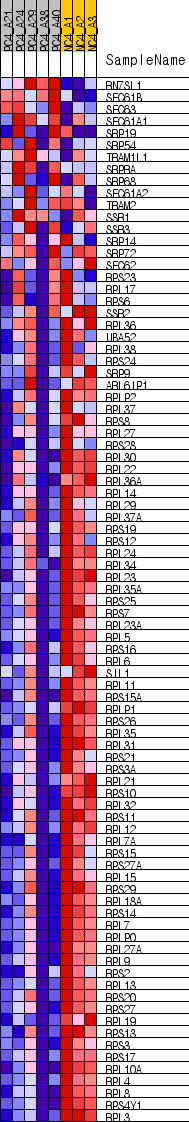

Supplement: Supplementary file 2 [file DataSheet1.ZIP › 4dpi_GO.Gsea.1625071243202/GOBP_COTRANSLATIONAL_PROTEIN_TARGETING_TO_MEMBRANE_1828.png]

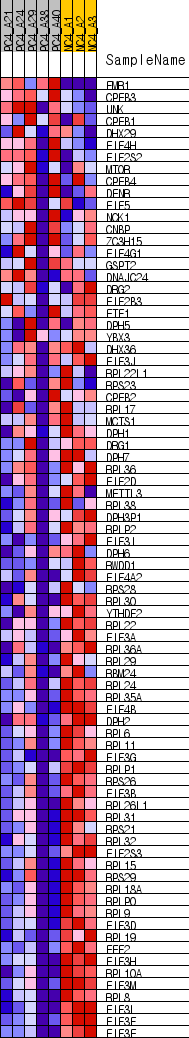

Supplement: Supplementary file 2 [file DataSheet1.ZIP › 4dpi_GO.Gsea.1625071243202/GOBP_CYTOPLASMIC_TRANSLATION_1855.png]

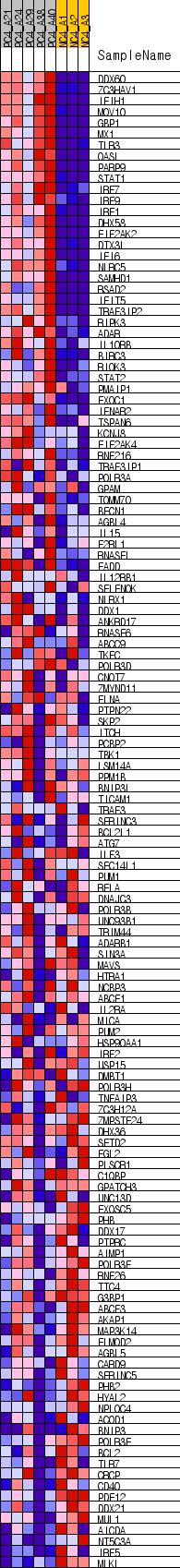

Supplement: Supplementary file 2 [file DataSheet1.ZIP › 4dpi_GO.Gsea.1625071243202/GOBP_DEFENSE_RESPONSE_TO_VIRUS_1783.png]

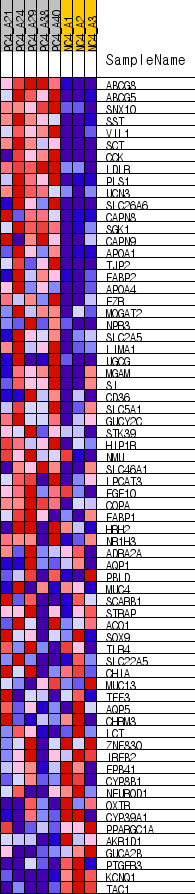

Supplement: Supplementary file 2 [file DataSheet1.ZIP › 4dpi_GO.Gsea.1625071243202/GOBP_DIGESTION_1819.png]

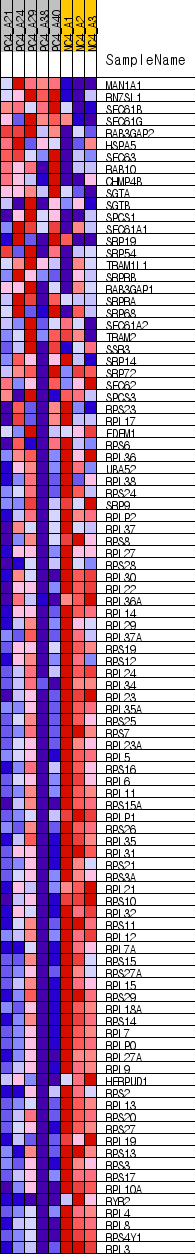

Supplement: Supplementary file 2 [file DataSheet1.ZIP › 4dpi_GO.Gsea.1625071243202/GOBP_ESTABLISHMENT_OF_PROTEIN_LOCALIZATION_TO_ENDOPLASMIC_RETICULUM_1846.png]

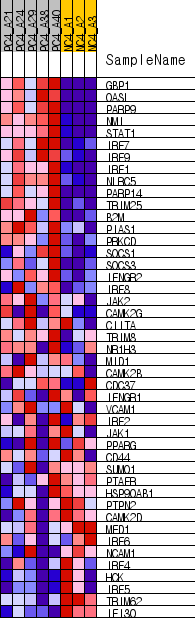

Supplement: Supplementary file 2 [file DataSheet1.ZIP › 4dpi_GO.Gsea.1625071243202/GOBP_INTERFERON_GAMMA_MEDIATED_SIGNALING_PATHWAY_1798.png]

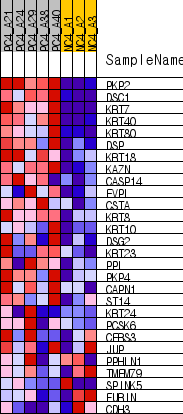

Supplement: Supplementary file 2 [file DataSheet1.ZIP › 4dpi_GO.Gsea.1625071243202/GOBP_KERATINIZATION_1777.png]

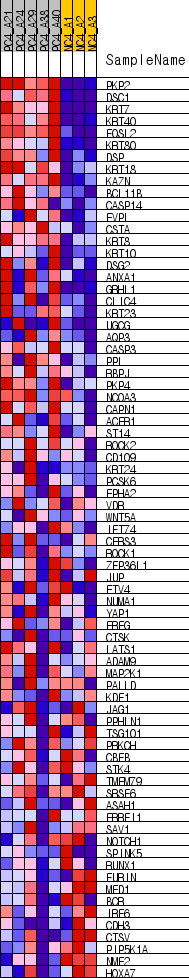

Supplement: Supplementary file 2 [file DataSheet1.ZIP › 4dpi_GO.Gsea.1625071243202/GOBP_KERATINOCYTE_DIFFERENTIATION_1813.png]

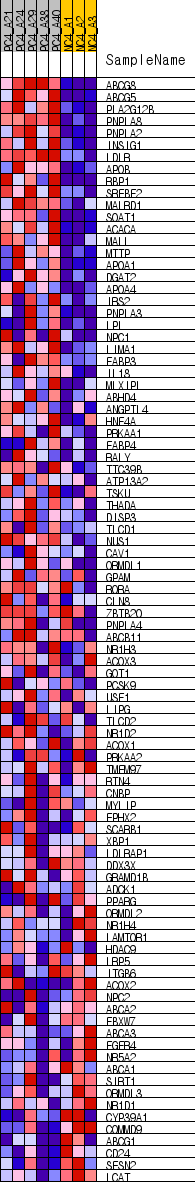

Supplement: Supplementary file 2 [file DataSheet1.ZIP › 4dpi_GO.Gsea.1625071243202/GOBP_LIPID_HOMEOSTASIS_1780.png]

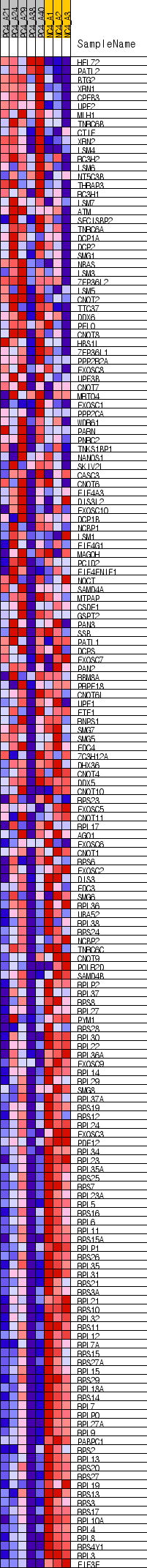

Supplement: Supplementary file 2 [file DataSheet1.ZIP › 4dpi_GO.Gsea.1625071243202/GOBP_NUCLEAR_TRANSCRIBED_MRNA_CATABOLIC_PROCESS_1882.png]

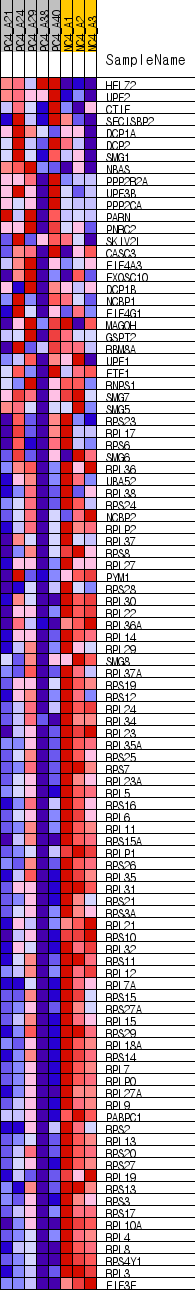

Supplement: Supplementary file 2 [file DataSheet1.ZIP › 4dpi_GO.Gsea.1625071243202/GOBP_NUCLEAR_TRANSCRIBED_MRNA_CATABOLIC_PROCESS_NONSENSE_MEDIATED_DECAY_1834.png]

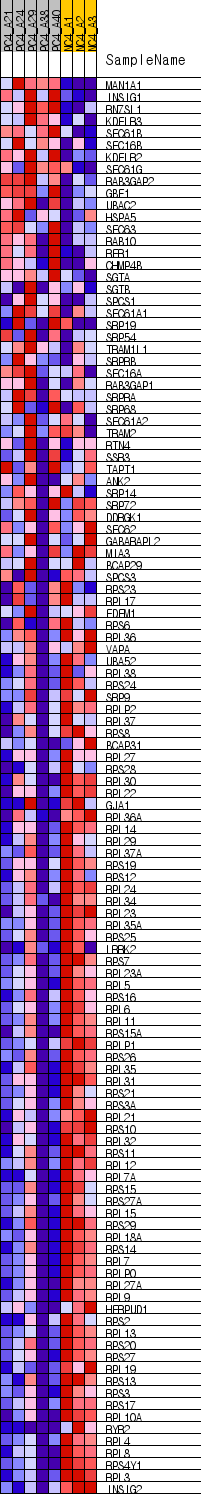

Supplement: Supplementary file 2 [file DataSheet1.ZIP › 4dpi_GO.Gsea.1625071243202/GOBP_PROTEIN_LOCALIZATION_TO_ENDOPLASMIC_RETICULUM_1861.png]

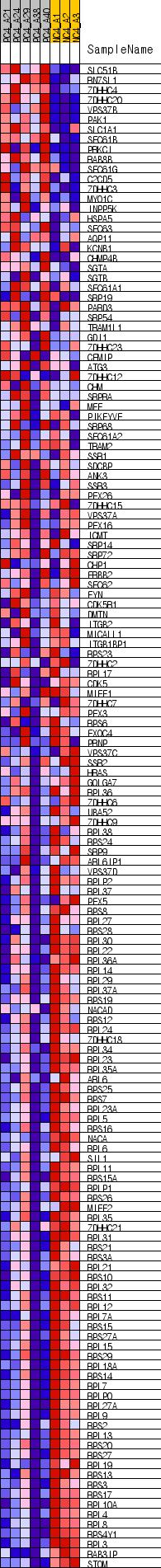

Supplement: Supplementary file 2 [file DataSheet1.ZIP › 4dpi_GO.Gsea.1625071243202/GOBP_PROTEIN_TARGETING_TO_MEMBRANE_1876.png]

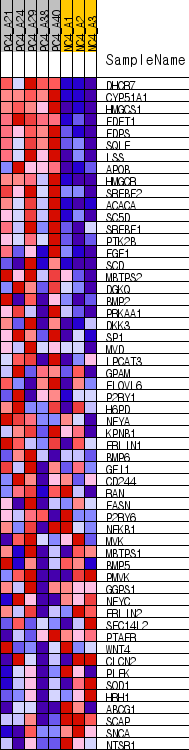

Supplement: Supplementary file 2 [file DataSheet1.ZIP › 4dpi_GO.Gsea.1625071243202/GOBP_REGULATION_OF_ALCOHOL_BIOSYNTHETIC_PROCESS_1807.png]

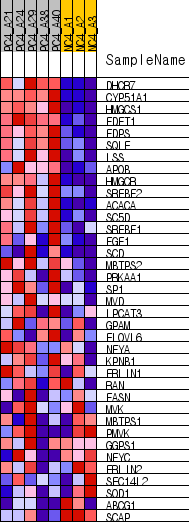

Supplement: Supplementary file 2 [file DataSheet1.ZIP › 4dpi_GO.Gsea.1625071243202/GOBP_REGULATION_OF_CHOLESTEROL_BIOSYNTHETIC_PROCESS_1768.png]

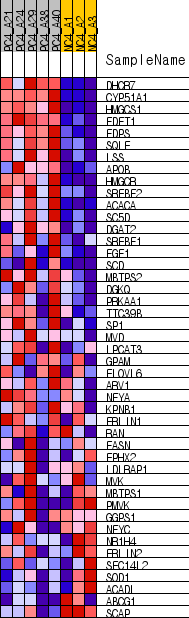

Supplement: Supplementary file 2 [file DataSheet1.ZIP › 4dpi_GO.Gsea.1625071243202/GOBP_REGULATION_OF_CHOLESTEROL_METABOLIC_PROCESS_1771.png]

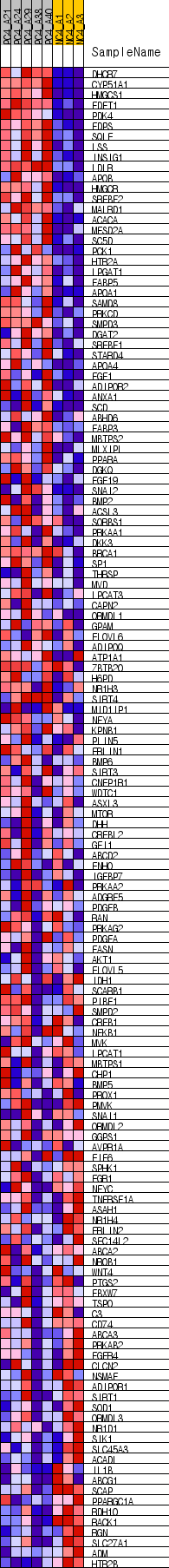

Supplement: Supplementary file 2 [file DataSheet1.ZIP › 4dpi_GO.Gsea.1625071243202/GOBP_REGULATION_OF_LIPID_BIOSYNTHETIC_PROCESS_1816.png]

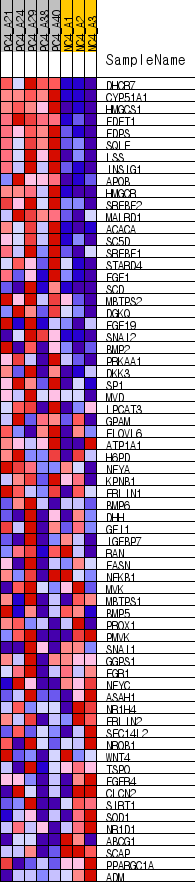

Supplement: Supplementary file 2 [file DataSheet1.ZIP › 4dpi_GO.Gsea.1625071243202/GOBP_REGULATION_OF_STEROID_BIOSYNTHETIC_PROCESS_1789.png]

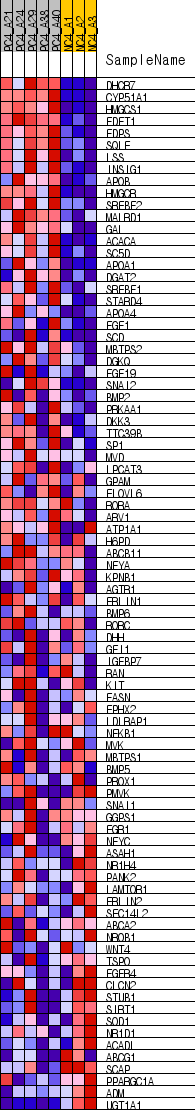

Supplement: Supplementary file 2 [file DataSheet1.ZIP › 4dpi_GO.Gsea.1625071243202/GOBP_REGULATION_OF_STEROID_METABOLIC_PROCESS_1810.png]

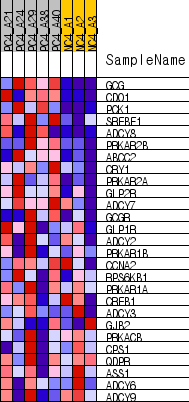

Supplement: Supplementary file 2 [file DataSheet1.ZIP › 4dpi_GO.Gsea.1625071243202/GOBP_RESPONSE_TO_GLUCAGON_1795.png]

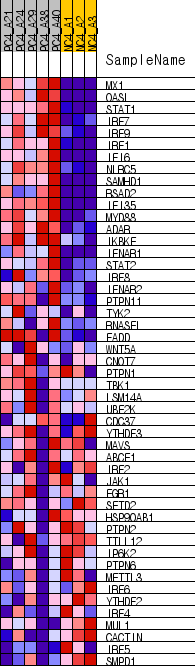

Supplement: Supplementary file 2 [file DataSheet1.ZIP › 4dpi_GO.Gsea.1625071243202/GOBP_RESPONSE_TO_TYPE_I_INTERFERON_1786.png]

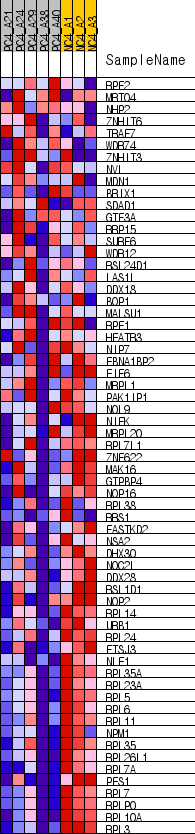

Supplement: Supplementary file 2 [file DataSheet1.ZIP › 4dpi_GO.Gsea.1625071243202/GOBP_RIBOSOMAL_LARGE_SUBUNIT_BIOGENESIS_1870.png]

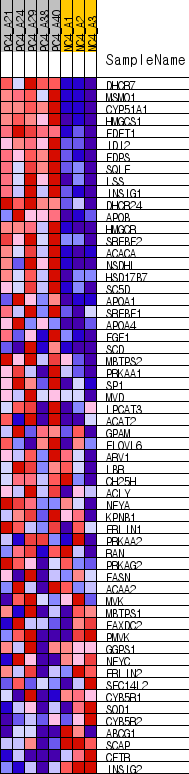

Supplement: Supplementary file 2 [file DataSheet1.ZIP › 4dpi_GO.Gsea.1625071243202/GOBP_STEROL_BIOSYNTHETIC_PROCESS_1765.png]

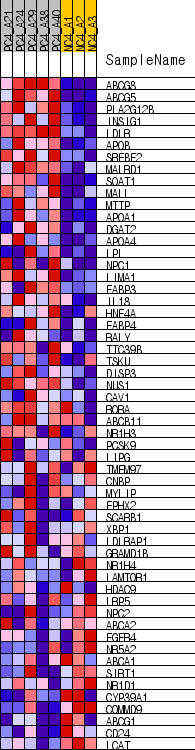

Supplement: Supplementary file 2 [file DataSheet1.ZIP › 4dpi_GO.Gsea.1625071243202/GOBP_STEROL_HOMEOSTASIS_1804.png]

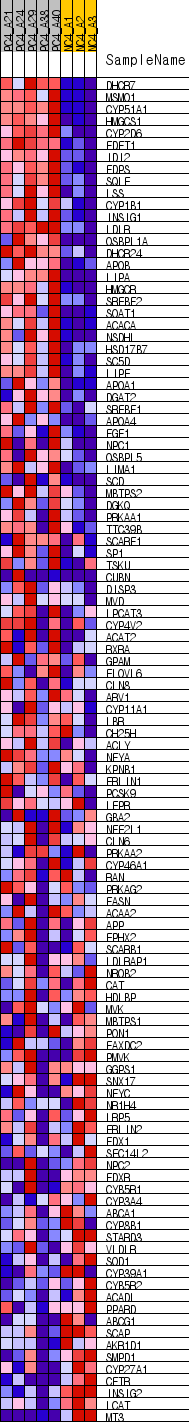

Supplement: Supplementary file 2 [file DataSheet1.ZIP › 4dpi_GO.Gsea.1625071243202/GOBP_STEROL_METABOLIC_PROCESS_1792.png]

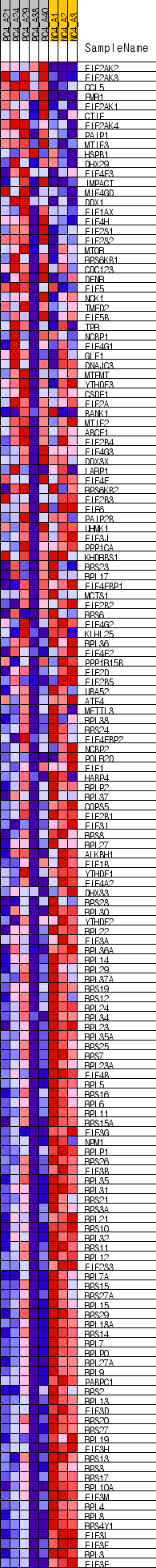

Supplement: Supplementary file 2 [file DataSheet1.ZIP › 4dpi_GO.Gsea.1625071243202/GOBP_TRANSLATIONAL_INITIATION_1843.png]

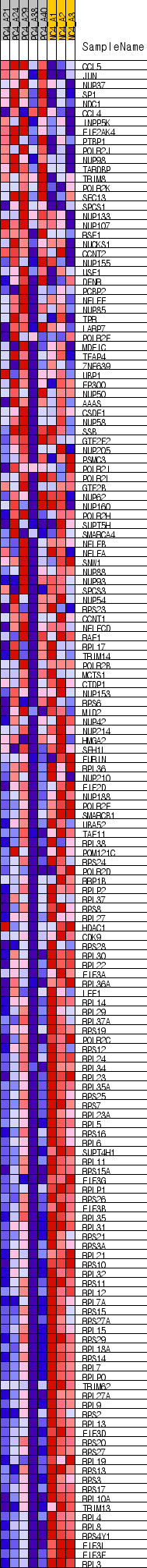

Supplement: Supplementary file 2 [file DataSheet1.ZIP › 4dpi_GO.Gsea.1625071243202/GOBP_VIRAL_GENE_EXPRESSION_1849.png]

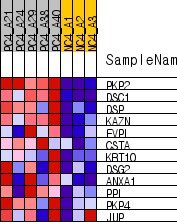

Supplement: Supplementary file 2 [file DataSheet1.ZIP › 4dpi_GO.Gsea.1625071243202/GOCC_CORNIFIED_ENVELOPE_1822.png]

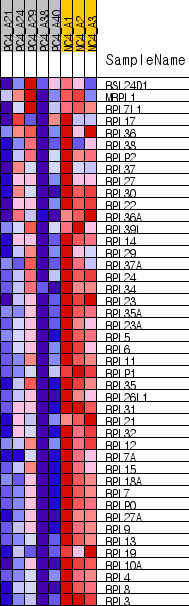

Supplement: Supplementary file 2 [file DataSheet1.ZIP › 4dpi_GO.Gsea.1625071243202/GOCC_CYTOSOLIC_LARGE_RIBOSOMAL_SUBUNIT_1837.png]

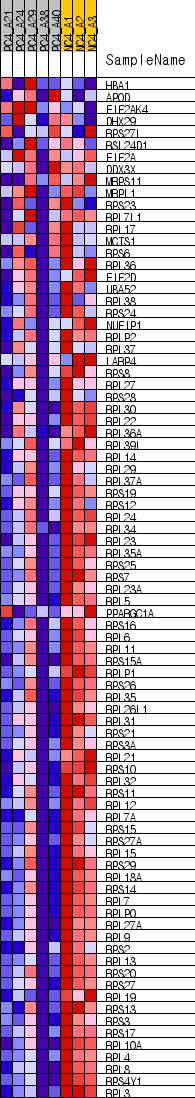

Supplement: Supplementary file 2 [file DataSheet1.ZIP › 4dpi_GO.Gsea.1625071243202/GOCC_CYTOSOLIC_RIBOSOME_1825.png]

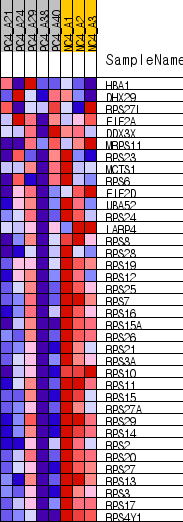

Supplement: Supplementary file 2 [file DataSheet1.ZIP › 4dpi_GO.Gsea.1625071243202/GOCC_CYTOSOLIC_SMALL_RIBOSOMAL_SUBUNIT_1864.png]

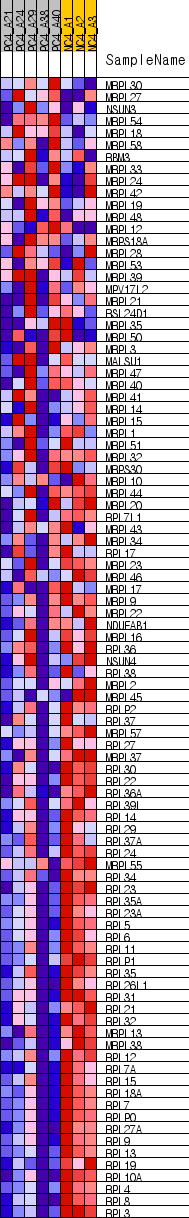

Supplement: Supplementary file 2 [file DataSheet1.ZIP › 4dpi_GO.Gsea.1625071243202/GOCC_LARGE_RIBOSOMAL_SUBUNIT_1867.png]

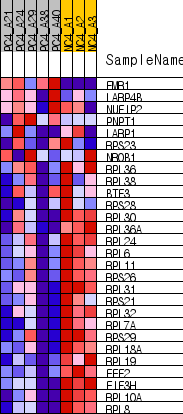

Supplement: Supplementary file 2 [file DataSheet1.ZIP › 4dpi_GO.Gsea.1625071243202/GOCC_POLYSOMAL_RIBOSOME_1879.png]

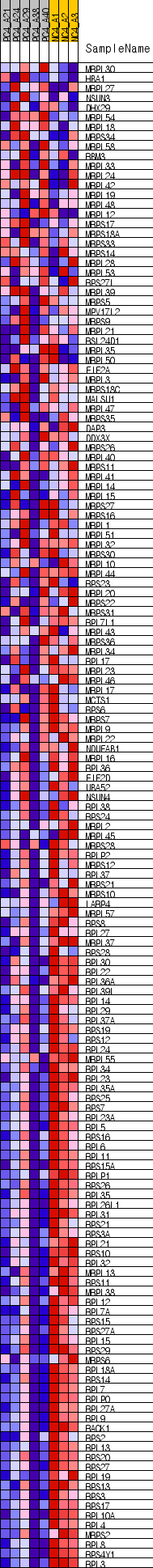

Supplement: Supplementary file 2 [file DataSheet1.ZIP › 4dpi_GO.Gsea.1625071243202/GOCC_RIBOSOMAL_SUBUNIT_1840.png]

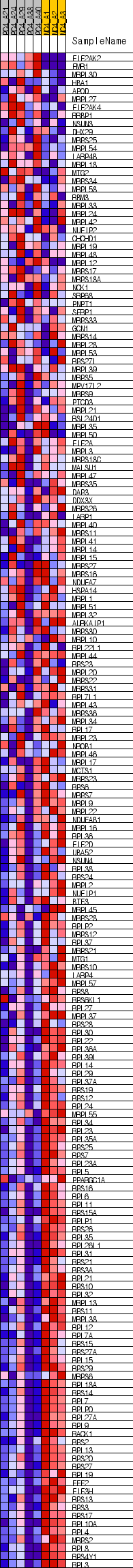

Supplement: Supplementary file 2 [file DataSheet1.ZIP › 4dpi_GO.Gsea.1625071243202/GOCC_RIBOSOME_1852.png]

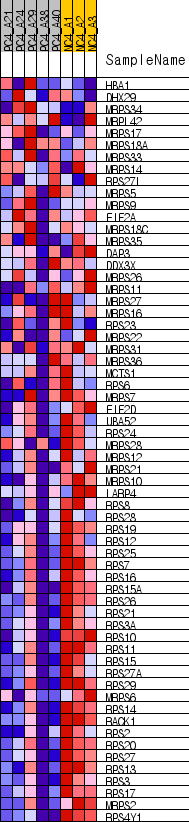

Supplement: Supplementary file 2 [file DataSheet1.ZIP › 4dpi_GO.Gsea.1625071243202/GOCC_SMALL_RIBOSOMAL_SUBUNIT_1858.png]

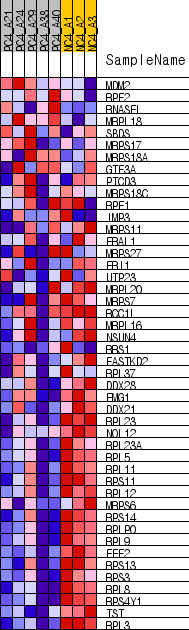

Supplement: Supplementary file 2 [file DataSheet1.ZIP › 4dpi_GO.Gsea.1625071243202/GOMF_RRNA_BINDING_1873.png]

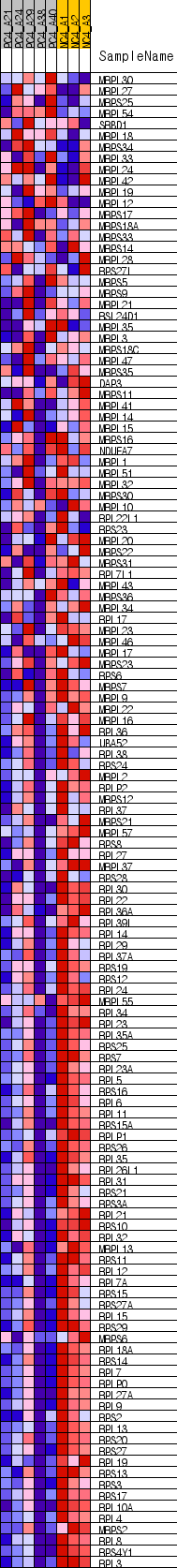

Supplement: Supplementary file 2 [file DataSheet1.ZIP › 4dpi_GO.Gsea.1625071243202/GOMF_STRUCTURAL_CONSTITUENT_OF_RIBOSOME_1831.png]

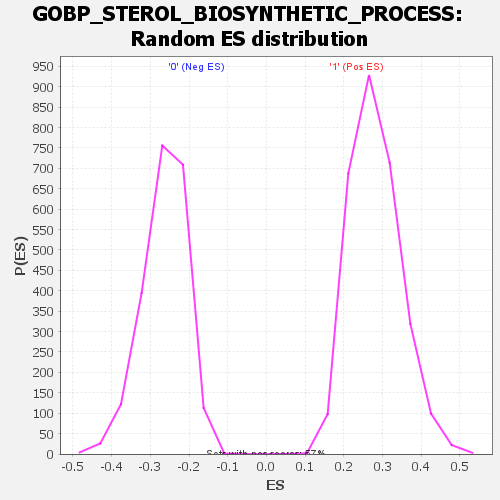

Supplement: Supplementary file 2 [file DataSheet1.ZIP › 4dpi_GO.Gsea.1625071243202/gset_rnd_es_dist_1766.png]

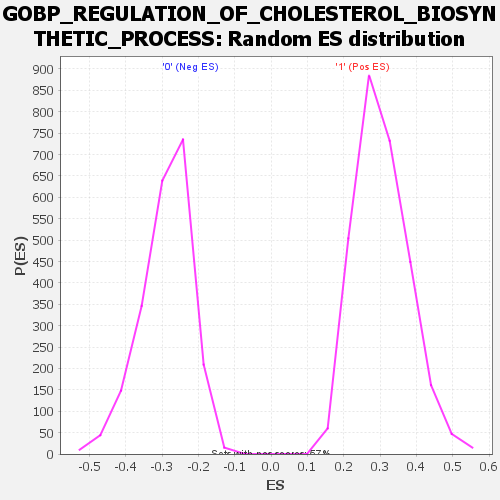

Supplement: Supplementary file 2 [file DataSheet1.ZIP › 4dpi_GO.Gsea.1625071243202/gset_rnd_es_dist_1769.png]

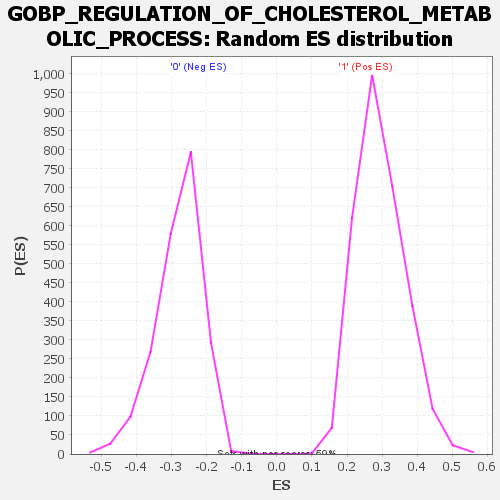

Supplement: Supplementary file 2 [file DataSheet1.ZIP › 4dpi_GO.Gsea.1625071243202/gset_rnd_es_dist_1772.png]

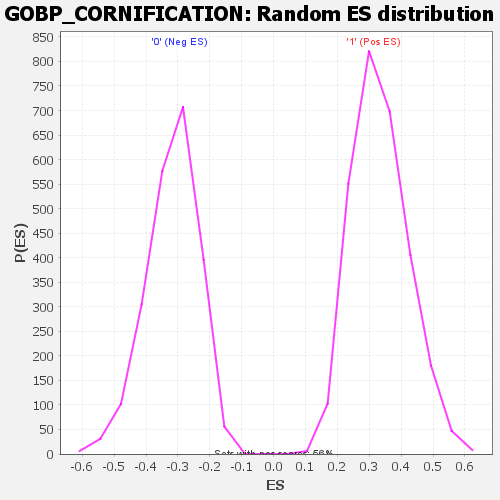

Supplement: Supplementary file 2 [file DataSheet1.ZIP › 4dpi_GO.Gsea.1625071243202/gset_rnd_es_dist_1775.png]

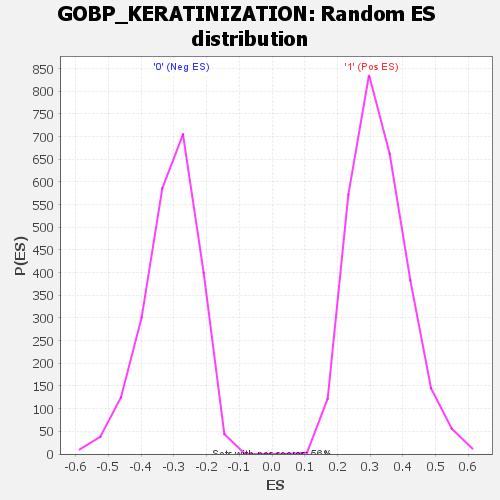

Supplement: Supplementary file 2 [file DataSheet1.ZIP › 4dpi_GO.Gsea.1625071243202/gset_rnd_es_dist_1778.png]

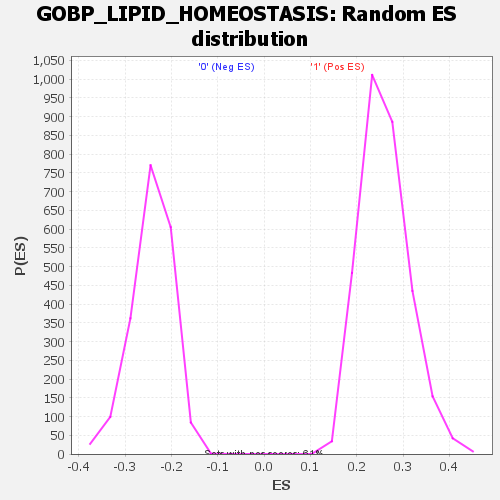

Supplement: Supplementary file 2 [file DataSheet1.ZIP › 4dpi_GO.Gsea.1625071243202/gset_rnd_es_dist_1781.png]

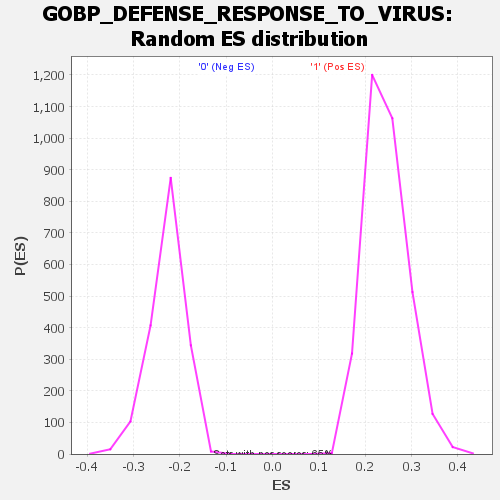

Supplement: Supplementary file 2 [file DataSheet1.ZIP › 4dpi_GO.Gsea.1625071243202/gset_rnd_es_dist_1784.png]

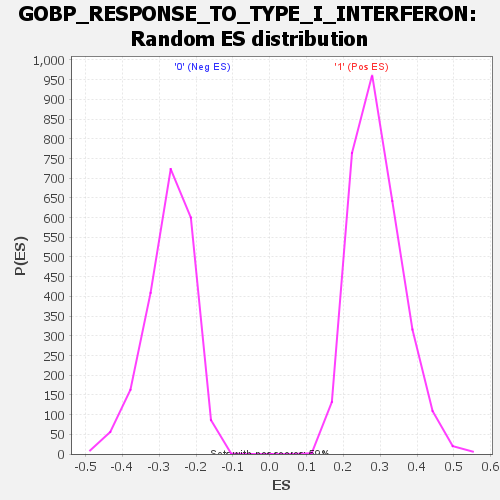

Supplement: Supplementary file 2 [file DataSheet1.ZIP › 4dpi_GO.Gsea.1625071243202/gset_rnd_es_dist_1787.png]

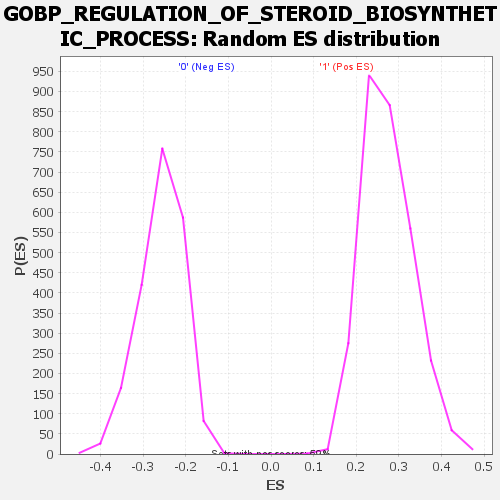

Supplement: Supplementary file 2 [file DataSheet1.ZIP › 4dpi_GO.Gsea.1625071243202/gset_rnd_es_dist_1790.png]

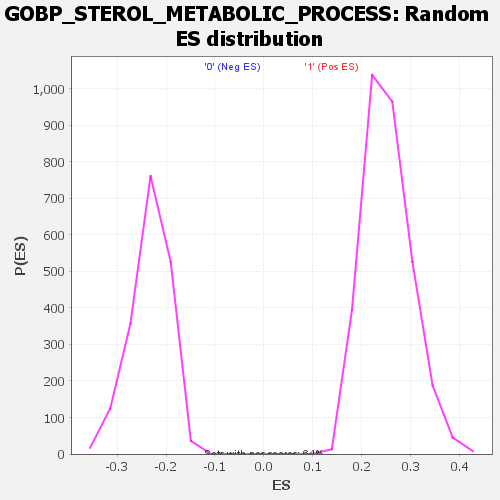

Supplement: Supplementary file 2 [file DataSheet1.ZIP › 4dpi_GO.Gsea.1625071243202/gset_rnd_es_dist_1793.png]

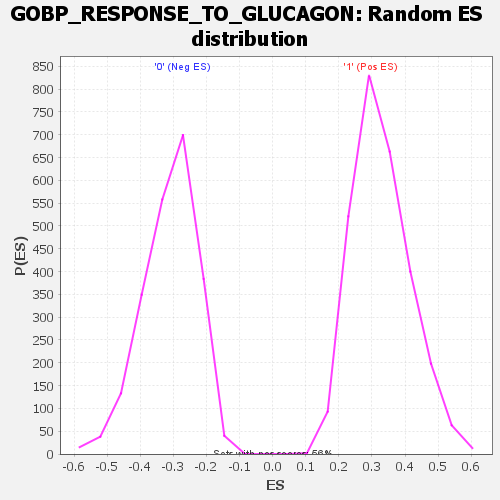

Supplement: Supplementary file 2 [file DataSheet1.ZIP › 4dpi_GO.Gsea.1625071243202/gset_rnd_es_dist_1796.png]

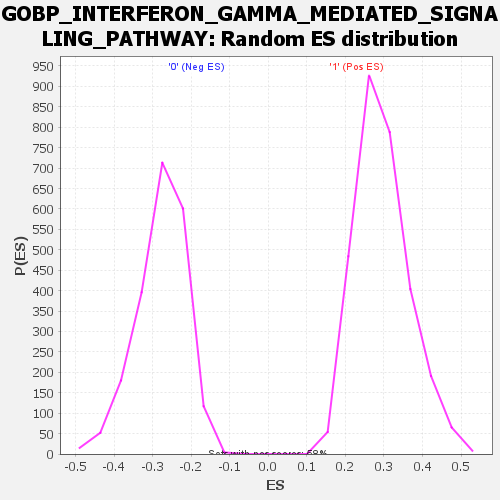

Supplement: Supplementary file 2 [file DataSheet1.ZIP › 4dpi_GO.Gsea.1625071243202/gset_rnd_es_dist_1799.png]

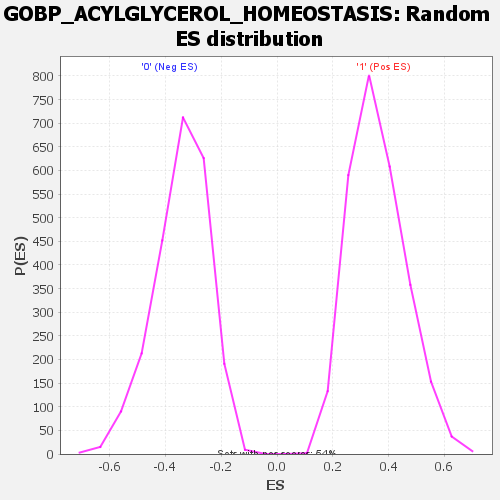

Supplement: Supplementary file 2 [file DataSheet1.ZIP › 4dpi_GO.Gsea.1625071243202/gset_rnd_es_dist_1802.png]

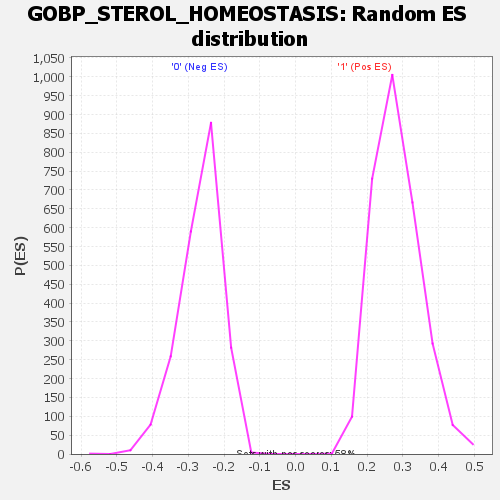

Supplement: Supplementary file 2 [file DataSheet1.ZIP › 4dpi_GO.Gsea.1625071243202/gset_rnd_es_dist_1805.png]

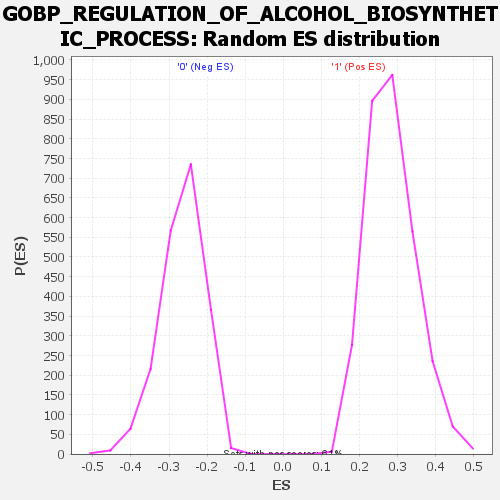

Supplement: Supplementary file 2 [file DataSheet1.ZIP › 4dpi_GO.Gsea.1625071243202/gset_rnd_es_dist_1808.png]

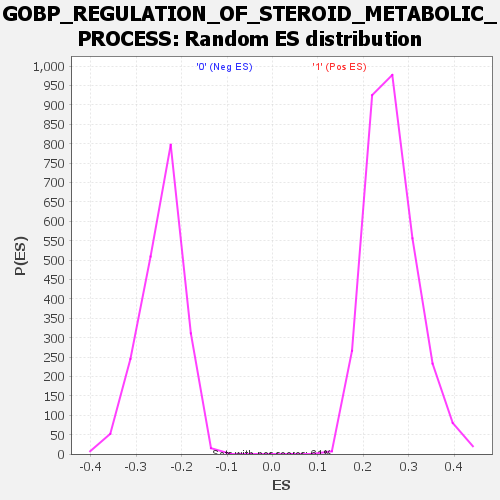

Supplement: Supplementary file 2 [file DataSheet1.ZIP › 4dpi_GO.Gsea.1625071243202/gset_rnd_es_dist_1811.png]

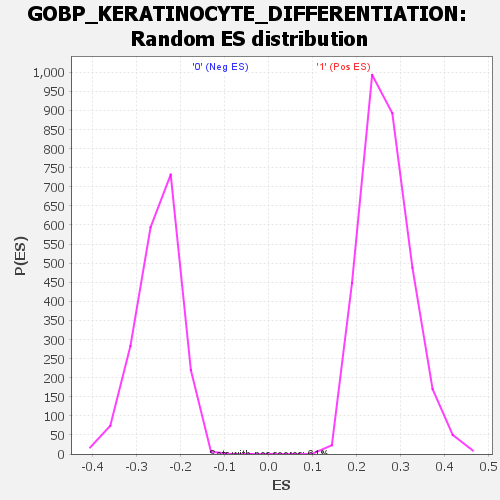

Supplement: Supplementary file 2 [file DataSheet1.ZIP › 4dpi_GO.Gsea.1625071243202/gset_rnd_es_dist_1814.png]

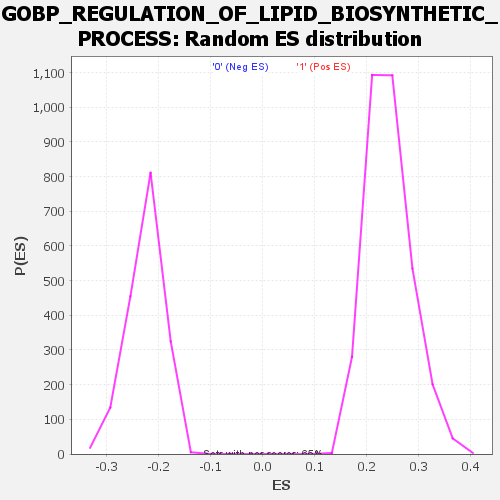

Supplement: Supplementary file 2 [file DataSheet1.ZIP › 4dpi_GO.Gsea.1625071243202/gset_rnd_es_dist_1817.png]

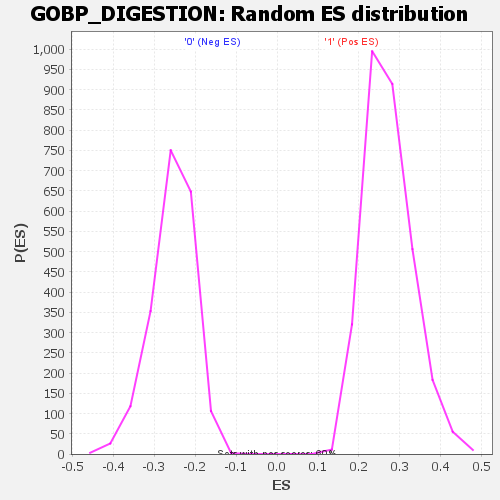

Supplement: Supplementary file 2 [file DataSheet1.ZIP › 4dpi_GO.Gsea.1625071243202/gset_rnd_es_dist_1820.png]
