# Supplementary material for: Time-series transcriptome analysis identified differentially expressed genes in broiler chicken infected with mixed Eimeria species
Source: Front Genet. 2022 Aug 8;13:886781. doi: 10.3389/fgene.2022.886781 (PMC9393255; doi:10.3389/fgene.2022.886781)
Supplement: Supplementary file 5 [file DataSheet2.docx]

Table S1. Primers used for qRT-PCR to validate DEGs identified in RNA-Seq

| Genes | Forward primer sequences (5’-3’) | Reverse primer sequences (5’-3’) |
| --- | --- | --- |
| *IFI6* | TCATGTCTGGTGAGGCAAAA | GGAGATCCCACTGCTGGTAA |
| *INSIG1* | TGGGTCTTTGGTGGACATTT | GGAAGCCAAGAACGGATGTA |
| *KRT40* | AAGCGCTGAACCAGGAAGTA | GCTCTGGCAGATCTGTCTCC |
| *IL13RA2* | CGTGGAGGTCCAGAGTGAAT | GCTCCAGACCCTCATACCAA |
| *IRF9* | GCCGTCTACAAGGGCAAATA | CTGGAAGTCCGTGCTTTTGT |
| *ART7B* | TGGAGTGGCGAAATCGATGG | CCCTGAATTGCTCGAAGGGT |
| *CCKAR* | TGCATGCCATTCACCCTCAT | AATGAGGCCATACGCAACCA |
| *XKR9* | ATTAGAAGCGCTCCCCAAGT | TTCCATCTCCTTGGATTTGC |
| *FABP2* | GGAGCCCACGATAATCTGAA | CGATGGTACGGAAGTTGCTT |
| *YWHAZ* | GAAGCATTGGGGATCAAGAA | CCCGTAGGTCATCTTGGAGA |
| *ACTB* | CTGTGCCCATCTATGAAGGCTA | ATTTCTCTCTCGGCTGTGGTG |

Table S2. Expression values of DEGs calculated by qRT-PCR

| Genes | log_2_FC_4dpi | log_2_FC_7dpi | log_2_FC_21dpi |
| --- | --- | --- | --- |
| *IFI6* | -1.959290456 | -1.359791437 | 0.825500981 |
| *INSIG1* | -0.30062413 | 0.452992668 | -0.499098881 |
| *KRT40* | 2.710436645 | 0.888694278 | -0.206981815 |
| *IL13RA2* | 1.195162134 | -0.85111914 | -0.433467142 |
| *IRF9* | -0.384328805 | -0.950396362 | -0.060387299 |
| *ART7B* | -0.878696423 | -1.176804306 | -0.42520043 |
| *CCKAR* | 2.673109077 | 1.243592378 | -0.545160171 |
| *XKR9* | 0.389295254 | -0.13765432 | -0.223244598 |
| *FABP2* | 2.070071862 | 1.079686711 | -0.750142462 |
| *YWHAZ* | -0.168864155 | 0.150558827 | -0.271219066 |
| *ACTB* | 0.361372915 | 0.009606012 | -0.256803317 |
